# Genetic architecture of gastric adenocarcinoma in West Asia

**DOI:** 10.1002/ctm2.70489

**Published:** 2025-09-25

**Authors:** Saeid Latifi‐Navid, Esmat Abdi, Tianpei Wang, Farhad Pourfarzi, Abbas Yazdanbod, Seyed Alireza Salami, Reza Safaralizadeh, Omolbanin Amjadi, Hamid Latifi‐Navid, Bahareh Safarnejad, Mahmoud Shokrabadi, Iradj Maleki, Vahid Hosseini, Seyed Mohammad Valizadeh, Mehdi Pourghasemian, Negin Abediasl, Arash Kazemi, Mohammad Eslami Jouybari, Zohreh Bari, Tarang Taghvaei, Caiwang Yan, Amir Taher Eftekhar Sadat, Seyed Yaghoub Moaddab, Ghasem Janbabaei, Mohammad Hossein Somi, Alireza Sadjadi, Ramin Shakeri, Farideh Siavoshi, Hafez Fakheri, Hossein Poustchi, Reza Malekzadeh, Guangfu Jin

**Affiliations:** ^1^ Department of Biology Faculty of Sciences University of Mohaghegh Ardabili Ardabil Iran; ^2^ Health Management Center The First Affiliated Hospital with Nanjing Medical University Nanjing Jiangsu China; ^3^ Department of Health Management School of Public Health Nanjing Medical University Nanjing Jiangsu China; ^4^ Digestive Disease Research Center Ardabil University of Medical Sciences Ardabil Iran; ^5^ Department of Biotechnology College of Agriculture and Natural Resources University of Tehran Karaj Iran; ^6^ Industrial and Medical Cannabis Research Institute (IMCRI) Tehran Iran; ^7^ Department of Biology Faculty of Natural Sciences University of Tabriz Tabriz Iran; ^8^ Gastrointestinal Cancer Research Center Non‐communicable Disease Institute Mazandaran University of Medical Sciences Sari Iran; ^9^ Department of Molecular Medicine National Institute of Genetic Engineering and Biotechnology Tehran Iran; ^10^ Ardabil University of Medical Sciences Ardabil Iran; ^11^ Farabi Medical Laboratory Ardabil Iran; ^12^ Gut and Liver Research Center Non‐communicable Diseases Research Institutes Mazandaran University of Medical Sciences Sari Iran; ^13^ Department of Oncology Ardabil University of Medical Sciences Ardabil Iran; ^14^ Department of Epidemiology Jiangsu Key Lab of Cancer Biomarkers Prevention and Treatment Collaborative Innovation Center for Cancer Medicine Center for Global Health School of Public Health Nanjing Medical University Nanjing China; ^15^ Public Health Institute of Gusu School The Affiliated Suzhou Hospital of Nanjing Medical University Suzhou China; ^16^ Jiangsu Key Laboratory of Molecular and Translational Cancer Research Jiangsu Cancer Hospital, Jiangsu Institute of Cancer Research The Affiliated Cancer Hospital of Nanjing Medical University Nanjing China; ^17^ Department of Pathology, Imam Reza Hospital Tabriz University of Medical Sciences Tabriz Iran; ^18^ Gastrointestinal and Liver Disease Research Center Tabriz University of Medical Sciences Tabriz Iran; ^19^ Department of Internal Medicine School of Medicine Hematologic Malignancies Research Center Tehran University of Medical Sciences Tehran Iran; ^20^ Digestive Disease Research Institute Tehran University of Medical Sciences Tehran Iran; ^21^ School of Biology, University College of Sciences University of Tehran Tehran Iran

1

Dear Editor,

Gastric cancer (GC) is a significant global health concern, with 968 000 new cases and 660 000 deaths in 2022, with male predominance.^1^ Despite a declining trend, the absolute number of GC cases is anticipated to rise, particularly in East and West Asia.^2^ Most genome‐wide association studies (GWASs) have focused on East Asian populations,^3^, ^4^ leaving a gap in understanding the genetic contributions to GC risk in West Asia, particularly in countries like Iran, where GC incidence is notably high. Most of Iran's northern and northwestern regions are located in the GC belt of West Asia. *Helicobacter pylori* infection, high salt intake, and smoking are major risk factors, along with gastroesophageal reflux disease, which contributes to higher cardia GC rates.^5^ This study presents a GWAS analysis of 4095 Iranian samples from high‐risk areas, with subsequent replication in a large Chinese dataset of 21 168 samples, aiming to delineate susceptibility loci associated with GC.

The analysis strategy is thoroughly outlined in Figure 1A (Supporting Information Methods). Of 2061 patients, 1531 (74.3%) were male; among 2034 controls, 1503 (73.9%) were male. The average age (mean ± SD) was 65.8 ± 11.0 and 67.8 ± 10.9 years for the patients and controls, respectively (Table S1). After filtering and quality control, 3686 subjects (1880 cases and 1806 controls) with 9 159 468 genetic variants were retained in the GWAS dataset (Figure 1A). A quantile‒quantile plot did not show substantial evidence of an inflation rate, with *λ* = 1.07 (Figure S1). Ethnicity and population structure were determined by the top two principal components for each study (Figure S2). Manhattan plots from the GWAS and multimarker analysis of the GenoMic Annotation (MAGMA) gene‐based analyses^6^ are shown in Figure 1B. Previous GWASs have identified a number of susceptibility loci (or common variants), including 1q22, 1p35.2, 2p11.2, 3q13.31, 5q14.3, 5p13.1, 6p22.1, 6p21.1, 8q24.3, 9q34.2, 10q23.33, 12q24.11–12, and 20q11.21.^3^, ^4^ In the present study, three loci reached genome‐wide significance (reported: 1q22 and 8q24.3; novel: 1p33; *p *< 5 × 10^−8^). Compared with previous results, consistent associations were observed for single nucleotide polymorphisms (SNPs) in *MUC1* at 1q22 (lead SNP: rs760077, OR = 1.39, 95% CI = 1.27–1.53; functional SNP: rs4072037, OR = 1.34, 95% CI = 1.22–1.47) and *PSCA* at 8q24.3 (lead SNP: rs2717562, OR = 1.39, 95% CI = 1.27–1.53). At 1p33, the lead (intergenic) SNP rs498352 near *FOXD2* was first found to be associated with GC risk (OR = 1.73, 95% CI = 1.43–2.10, *p *= 2.26 × 10^−8^; Table 1A; Figure 2A). FOXD2 binding reconfigures chromatin structure to suppress colorectal cancer by reprogramming enhancer interactions.^7^ In addition, four previously reported loci, 4q28.1 (*ANKRD50*), 5p13.1 (*PRKAA1*), 10q23.33 (*PLCE1, NOC3L*), and 12q24.11‐12 (*CUX2*), were also replicated in the present GWAS of West Asians (*p *< .05; Table S2).

**FIGURE 1:**
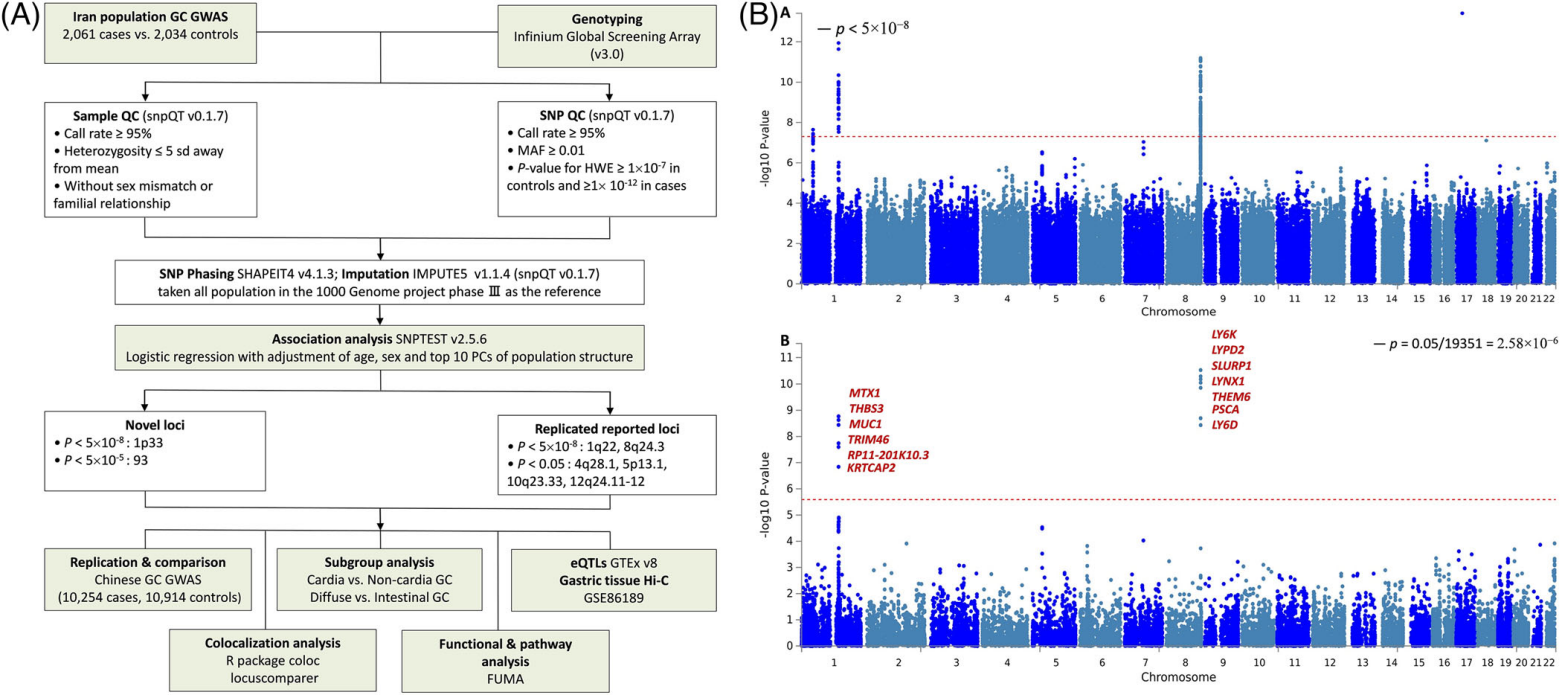
**(A) Study design and workflow. (B) Manhattan plots from GWAS and MAGMA gene‐based analyses**. (A) Manhattan plot of the GWAS analysis of gastric cancer, showing the negative log_10_‐transformed *p*‐value for each SNP. (B) Manhattan plot of the gene‐based test results computed by MAGMA. The input SNPs were mapped to 19351 protein‐coding genes. Genome‐wide significance (red dashed line in the plot) was defined at *p* = .05/19 351 = 2.58 × 10^−6^. A total of 13 genes were identified as significantly associated with GC risk. Among these were *MTX1*, *THBS3*, *MUC1*, *TRIM46*, *RP11‐201K10.3*, and *KRTCAP2*, which were located on chromosome 1. Additionally, *LY6K, LYPD2*, *SLURP1*, *LYNX1*, *THEM6*, *PSCA*, and *LY6D* were located on chromosome 8. On the *y*‐axis, the gene‐based test's negative log_10_‐transformed *p‐*value is displayed, and on the *x*‐axis, the starting position on the chromosome is shown.

**TABLE 1:** (A) The identified gastric cancer risk loci, including those reaching genome‐wide significance (*p* < 5 × 10^−8^) in this GWAS and those (potential novel loci) significantly replicated in a large Chinese (case–control) dataset of 21 168 samples. (B) The effect difference of reported and novel loci between cardia and noncardia gastric cancer patients, as well as between intestinal and diffuse gastric cancer patients, in the Ardabil population.

Locus	Associated gene	Variant^a^	Position (build 37)	Alleles (risk/other)	RAF^b^ (case, control)	Study	OR (95%CI)^c^		*p‐*value		*p*_heterogeneity_
(A) Risk loci at genome‐wide significance (*p* < 5 × 10^−8^)											
Known loci											
1q22	*MUC1*	Lead: rs760077	1:155178782	T/A	.55,.46	GWAS	1.39 (1.27–1.53)		1.15 × 10^−12^		
		Functional: rs4072037	1:155162067	T/C	.50,.42	GWAS	1.34 (1.22–1.47)		2.20 × 10^−10^		
8q24.3	*PSCA*	Lead: rs2717562	8:143776668	C/T	.46,.39	GWAS	1.39 (1.27–1.53)		6.34 × 10^−12^		
Novel loci											
1p33	*FOXD2*	Lead: rs498352	1:47952962	C/T	.08,.04	GWAS	1.73 (1.43–2.10)		2.26 × 10^−8^		
								
Replicated novel loci											
3p11.1	*ABCF2P1*	rs4859012	3:88377838	G/C	.35,.30	GWAS	1.25 (1.14–1.38)		5.35 × 10^−6^		
				C/G	.31,.30	Chinese	1.05 (1.00–1.10)		.040		
				C/G		meta	1.08 (1.04–1.13)		2.06 × 10^−4^		.016
3p22.1	*POMGNT2*	rs11720364	3:43186832	T/C	.94,.91	GWAS	1.60 (1.30–1.97)		8.20 × 10^−6^		
				T/C	.99,.99	Chinese	1.57 (1.08–2.29)		.018		
				T/C		meta	1.60 (1.33–1.92)		6.96 × 10^−7^		.265
10q25.2	*RBM20*	rs7899485	10:112567284	C/G	.60,.55	GWAS	1.24 (1.12–1.37)		2.07 × 10^−5^		
				G/C	.46,.46	Chinese	1.05 (1.01–1.10)		.023		
				G/C		meta	1.01 (.97–1.05)		.774		1.99 × 10^−4^
17q21.31	*MAPT‐AS1*	rs114469358	17:43962562	C/T	.11,.09	GWAS	1.41 (1.20–1.65)		1.99 × 10^−5^		
				T/C	.98,.98	Chinese	1.18 (1.01–1.37)		.043		
				C/T		meta	1.09 (.97–1.22)		.142		2.90 × 10^−4^
**(B)**							Cardia GC		Noncardia GC		
Novel loci							OR (95% CI)	*p‐*value	OR (95% CI)	*p‐*value	
2p13.1	*SLC4A5*	rs17009774	2:74480659	T/C	‐	GWAS	.76 (.59–.97)	.025	1.08 (.86–1.37)	.494	.036
8p23.2	*CSMD1*	rs10217067	8:3924335	A/G	‐	GWAS	.95 (.82–1.09)	.462	1.18 (1.02–1.36)	.022	.033
13q14.13	*LCP1*	rs1230471	13:46777563	T/G	‐	GWAS	1.16 (1.01–1.34)	.036	.94 (.81–1.09)	.399	.038
							Intestinal‐type GC		Diffuse‐type GC		
Known loci							OR (95% CI)	*p‐*value	OR (95% CI)	*p‐*value	
1q22	*MUC1*	rs760077	1:155178782	A/T	‐	GWAS	1.08 (.96–1.22)	.214	.86 (.73–1.02)	.083	.034
1q22	*MUC1*	rs2990223	1:155184975	A/G	‐	GWAS	1.08 (.96–1.23)	.203	.87 (.73–1.02)	.091	.035
1q22	*MUC1*	rs4072037	1:155162067	T/C	‐	GWAS	.96 (.85–1.08)	.480	1.19 (1.00–1.40)	.044	.042
5p13.1	*PTGER4*	rs10036575	5:40685795	T/C	‐	GWAS	1.01 (.88–1.17)	.843	.80 (.66–.97)	.021	.049

^a^ Lead variant is the most significant variant at the locus in this study; a functional variant was reported previously.

^b^ RAF, risk allele frequency, based on combined data of GWAS.

^c^ OR (95% CI), odds ratio and 95% confidence interval are estimated for the risk allele. All *p*‐values reported are based on fixed‐effects inverse variance–weighted meta‐analysis.

**FIGURE 2:**
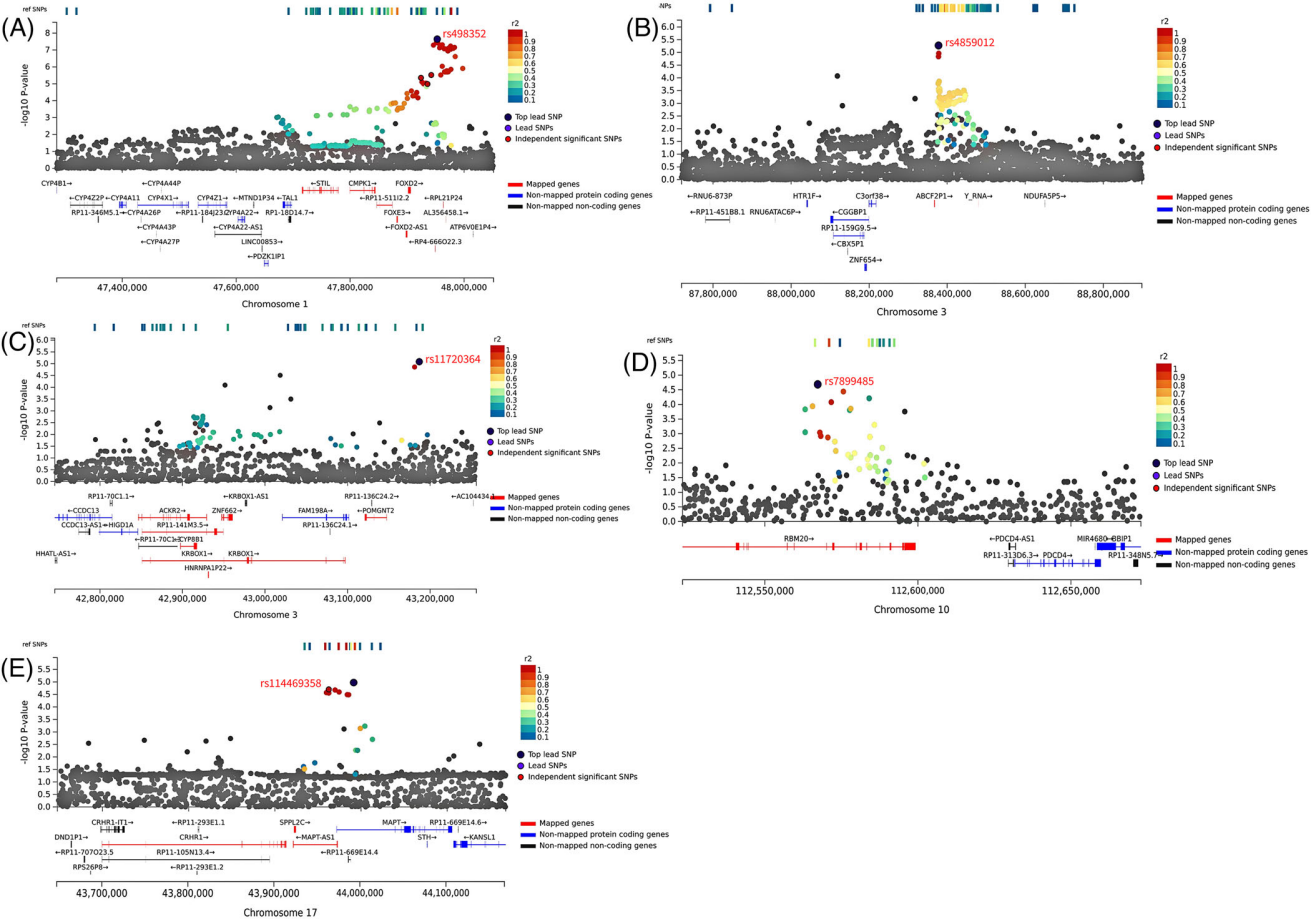
**Regional plots of five novel gastric cancer susceptibility loci on 1p33, 3p22.1, 3p11.1, 10q25.2, and 17q21.31**. Association *p*‐values from a trend test are displayed in –log_10_ (y axis) for each single‐nucleotide polymorphism according to their chromosomal positions (*x*‐axis) at (A) 1p33 (rs498352), (B) 3p11.1 (rs4859012), (C) 3p22.1 (rs11720364), (D) 10q25.2 (rs7899485), and (E) 17q21.31 (rs114469358). SNPs are coloured by their highest *r* ^2^ compared with one of the independent significant SNPs. Other SNPs are coloured in grey. The top lead SNPs in genomic risk loci, lead SNPs and independent significant SNPs are circled in black and coloured dark‐purple, purple and red, respectively. Pairwise *r* ^2^ values are from 1000G phase 3 data. SNPs were mapped to genes by physical distance from known genes encoding proteins in the human reference assembly (GRCh37/hg19).

The present study (discovery study) identified 1108 SNPs associated with GC risk with *p *< 5×10^−5^. We validated these SNPs in the largest Chinese GC GWAS dataset (replication study; 10 254 GC patients and 10 914 controls).^8^ Four novel loci were identified at 3p11.1, 3p22.1, 10q25.2, and 17q21.31 (Table 1A). There were consistent associations for SNPs rs4859012 near *ABCF2P1* at 3p11.1 (discovery: OR = 1.25, 95% CI = 1.14–1.38; replication: OR = 1.05, 95% CI = 1.00–1.10), rs11720364 near *POMGNT2* at 3p22.1 (discovery: OR = 1.60, 95% CI = 1.30–1.97; replication: OR = 1.57, 95% CI = 1.08–2.29), rs7899485 in *RBM20* at 10q25.2 (discovery: OR = 1.24, 95% CI = 1.12–1.37; replication: OR = 1.05, 95% CI = 1.01–1.10), and rs114469358 in *MAPT‐AS1* at 17q21.31 (discovery: OR = 1.41, 95% CI = 1.20–1.65; replication: OR = 1.18, 95% CI = 1.01–1.37; Figure 2B–E). We also developed a polygenic risk score (PRS) based on 93 potential novel SNPs and six replicated loci. There was a significant difference between the case and control distributions (Tables S3 and S4; Figure S3).

Among the 1880 GC patients (vs. 1806 controls), 833, 906, and 141 individuals were diagnosed with cardia, noncardia, and not otherwise specified (NOS) GC, respectively. We identified three novel loci that showed different effects between cardia and noncardia GCs (Table 1B). The intronic SNPs rs17009774 in *RP11‐287D1.3:SLC4A5* at 2p13.1 and rs1230471 in *LCP1* at 13q14.13 were associated only with cardia GCs; the ORs (95% CIs) were.76 (.59–.97) and 1.16 (1.01–1.34), respectively (Figure S4A,C). In contrast, the intronic SNP rs10217067 in *CSMD1* at 8p23.2 was associated with only noncardia GCs, with an OR (95% CI) of 1.18 (1.02–1.36) (Figure S4B).

SLC4A5 is biologically plausible as a cancer‐relevant transporter involved in pH regulation and trafficking; however, specific functional data in GC are limited or lacking. Although LCP1 may play a complex role in GC, gaps remain in understanding its differential expression and functional impact based on tumour location—specifically when comparing cardia and noncardia gastric cancers, which differ in aetiology, histology, and molecular characteristics. Mechanistically, infection with *H. pylori* induces GC cells to express LCP1 via the CagA‐activated ERK signalling pathway, which mediates the binding of SP1 to the LCP1 promoter. Furthermore, increased LCP1 expression facilitates the growth and metastasis of GC in vivo.^9^ Another protein, CSMD1, acts as a tumour suppressor gene, while microRNA‐10b drives GC cell invasion and metastasis by inhibiting CSMD1, activating the NF‐κB pathway, and upregulating c‐Myc, cyclin D1, and epithelial–mesenchymal transition (EMT) markers. Nevertheless, the expression levels and functional implications of CSMD1 concerning the tumour's location remain poorly understood.^10^

Among the 1880 GC patients, 1091, 483, and 306 individuals were diagnosed with intestinal, diffuse, and mixed‐type GC, respectively. Among previously reported loci, 1q22 and 5p13.1 were identified to play different roles in intestinal‐type and diffuse‐type GC (DGC; Table 1B). At 1q22, the exonic SNP rs4072037 in the *MUC1* gene showed a significant association with only DGC, with an OR (95% CI) of 1.19 (1.00–1.40). At this locus, the *p*‐values for the exonic SNPs rs760077 and rs2990223 were >.05. However, the effect of these SNPs on intestinal‐ and diffuse‐type GC differed (*p* for heterogeneity <.05). At 5p13.1, the intronic SNP rs10036575 in *PTGER4* (EP‐4) was associated with only DGC, with an OR (95% CI) of.80 (.66–.97; Figure S4D–E **)**.

MUC1‐C (the C‐terminal subunit) plays a crucial role in the loss of cell polarity, the induction of EMT, the activation of stem cell characteristics, and epigenetic reprogramming. It promotes cell growth by activating multiple signalling pathways, including RTKs, PI3K/AKT, and WNT/β‐catenin. Additionally, MUC1‐C regulates inflammation, protects against cell death, and facilitates immune evasion. Patients with positive MUC1 expression exhibit higher rates of aggressive pathological features, such as DGC, lymph node metastasis, and distant metastasis.^11^, ^12^ DGC is characterised by a scattered and infiltrative growth pattern, often associated with defects in CDH1/E‐cadherin. The EP4 signalling pathway promotes cellular migration and increases the population of stem‐like CD133⁺/CD44⁺ cells, which are associated with EMT and peritoneal dissemination—both key characteristics of DGC. EP4 activity also supports inflammatory and fibrotic cascades, including NLRP3 and phosphorylated p65 (p‐p65), which can prime the peritoneal environment. Notably, EP4 blockade reduces peritoneal fibrosis in preclinical models, a finding that is particularly relevant given the frequent intraperitoneal spread of DGC.^13^, ^14^

In the present GWAS, we identified 93 potential novel GC risk loci (*p *< 5 × 10^−5^). However, only four of these loci were replicated in the Chinese dataset (*p *< .05). This could be explained by the genetic diversity between West and East Asian populations. It was found that Iranians had a population structure between Europeans and East Asians, but were more closely related to Europeans. Even among the replicated ones, the risk alleles, allele frequencies, and effect sizes differed between populations. These findings underscore the importance of conducting GWASs in diverse populations to identify missed genetic variants that contribute to GC risk across different ancestry groups.

Functional analysis identified 89 genomic risk loci. Most of the 2732 annotated SNPs (93.1%) were in the intergenic, intronic, and ncRNA‐intronic regions. Forty‐seven genes were implicated by at least two mapping strategies and 14 by all three strategies; 17 had pLI scores of ≥.9, indicating that they were highly sensitive to mutations that lead to truncated or nonfunctional proteins. In the MAGMA gene analysis, 13 genes (all mapped by FUMA^15^) had aggregate associations based on all SNPs in a gene on chromosomes 1 and 8, with a stringent *p*‐value threshold of less than 2.58 × 10^−6^ (Figure 1B; Tables S5–S7; Figure S5A–M). Notably, *THBS3* and *TRIM46* exhibited high sensitivity to loss‐of‐function mutations, indicated by their pLI scores of 1.2 and.99, respectively. MAGMA gene set analysis identified the top 10 gene sets (*p *≤ .0007), but the associations did not reach significance after Bonferroni correction (*p*_bon_ < .05). GSEA identified 627 GO biological processes and 76 pathways from Biocarta, KEGG, and Reactome (FDR ≤ .05). However, 14 hallmark gene sets summarised well‐defined biological states or processes coherently (Tables S8–S11). Furthermore, the 1q22, 4q28.1, and 8q24.3 risk loci showed evidence of colocalization^16^ in stomach tissue (GTEx v8; PP4 = .84,.96, and.85, respectively), reflecting the fact that these loci contain shared GC causal variants in West Asia. Although novel loci, especially 11q14.1 and 13q14.11, did show some potential colocalization signals, PP4 did not reach the significance threshold (<.7; Table S12; Figure 3A–E). The study's strengths include a large sample size and subgroup analyses, although it acknowledged limitations such as the exclusion of lifestyle, dietary habits, and *H. pylori* infection. However, the latter may not be relevant because the prevalence of *H. pylori* infection is significantly high in northern/northwestern Iran, especially in Ardabil (89%), where more than 90% of adults aged 40 or older have *H. pylori*‐related chronic gastritis and GC is the most common malignancy (31%), with an ASR of 49.1/10^5^ for males and 25.4/10^5^ for females.^5^

**FIGURE 3:**
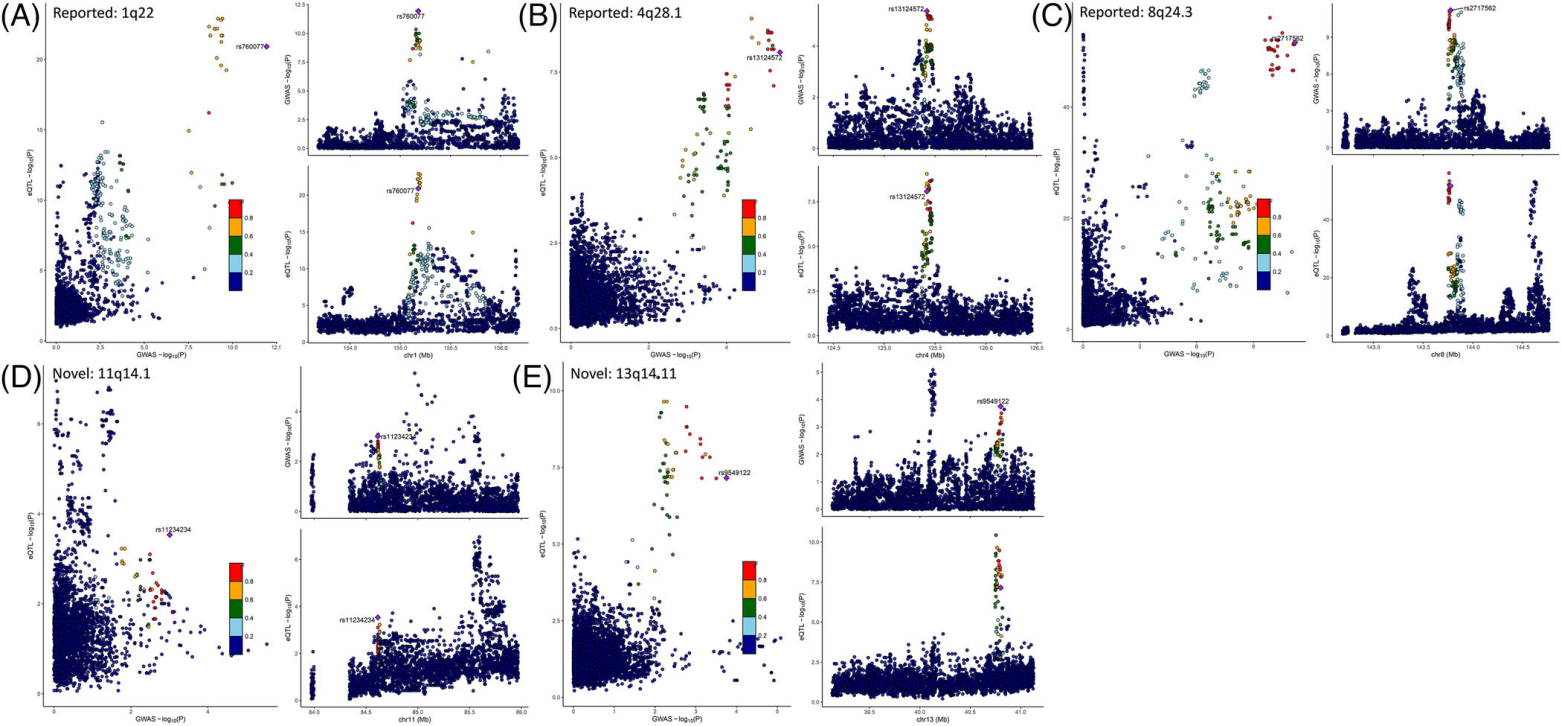
**Bayesian colocalization analysis of significant GWAS findings**. Colocalization analysis was carried out for the six reported (replicated) and 28 novel loci from GWASs in stomach tissue from GTEx v8. For each gene of a region, a posterior probability was obtained; the greater the posterior probability was (PP4 > .7), the stronger the evidence for colocalization. (A–C) Three loci, 1q22, 4q28.1, and 8q24.3, colocalised with eQTLs in stomach tissue (>.7). The regional lead SNPs were cis‐associated with gene expression (lower right) and simultaneously associated with facial variation in the GWAS (upper right). Therefore, these SNPs contribute to both cis‐eQTL signals and GWAS signals (left). (D, E) The novel loci, however, did show some potential colocalization signals (especially for 11q14.1 and 13q14.11), but the PP4 of colocalization was <.7.

In the present study, the developed PRS showed a significant difference between case and control distributions, which marks a pivotal advancement in personalised medicine. It quantifies an individual's genetic predisposition to diseases, which can enable risk stratification and potentially inform preventive care and treatment decisions. The implementation of these scores can help identify high‐risk individuals, allowing for tailored interventions that may improve overall patient outcomes. However, applying this PRS to large‐scale, long‐term Persian cohorts—especially those from high‐risk areas—will be essential to validate its predictive power and facilitate its eventual integration into clinical workflows.

In conclusion, we identified the GC risk‐related loci in West Asia, including those related to tumour site and pathology, which may contribute to future clinical risk assessments and genetic screening in West Asia.

## AUTHOR CONTRIBUTIONS

Saeid Latifi‐Navid, Abbas Yazdanbod, Farhad Pourfarzi, Guangfu Jin, and Reza Malekzadeh conceptualised the study. Saeid Latifi‐Navid, Tianpei Wang, and Caiwang Yan performed the statistical analyses. Abbas Yazdanbod, Farhad Pourfarzi, Esmat Abdi, Reza Safaralizadeh, Omolbanin Amjadi, Hamid Latifi‐Navid, Bahareh Safarnejad, Mahmoud Shokrabadi, Iradj Maleki, Vahid Hosseini, Seyed Mohammad Valizadeh, Mehdi Pourghasemian, Negin Abediasl, Arash Kazemi, Mohammad Eslami Jouybari, Zohreh Bari, Tarang Taghvaei, Amir Taher Eftekhar Sadat, Seyed Yaghoub Moaddab, Ghasem Janbabaei, Mohammad Hossein Somi, Alireza Sadjadi, Ramin Shakeri, Farideh Siavoshi, Hafez Fakheri, Hossein Poustchi, and Reza Malekzadeh contributed to evaluate and diagnose patients, sample and data collection or data interpretation. Saeid Latifi‐Navid, Esmat Abdi, and Seyed Alireza Salami conducted experiments. Saeid Latifi‐Navid, Tianpei Wang, and Guangfu Jin reviewed data and provided critical comments or suggestions. Saeid Latifi‐Navid wrote the manuscript. Guangfu Jin and Tianpei Wang revised the manuscript. Saeid Latifi‐Navid and Guangfu Jin supervised the study. All authors reviewed or revised the manuscript and approved the final draft for submission.

## CONFLICT OF INTEREST STATEMENT

The authors declare no conflict of interest.

## FUNDING INFORMATION

This study was funded by the National Institute for Medical Research Development (NIMAD) (grants 958117 and 962249), Tehran, Iran, and the National Natural Science Foundation of China (82125033 and 82230110).

## ETHICS STATEMENT

The research was performed on the basis of ethical principles of human research declared by the 1975 Declaration of Helsinki. All patients and/or their legal guardians signed written informed consent. The research was approved by the Ethics Committee of the National Institute for Medical Research Development (NIMAD)/IR.NIMAD.REC.1396.097.

## CONSENT FOR PUBLICATION

Not applicable

## Supporting information



Supporting information

## Data Availability

All data are available in the main text or the supplementary materials. Other raw data are available from the corresponding author upon reasonable request.

## References

[1] Bray F , Laversanne M , Sung H , et al. Global cancer statistics 2022: gLOBOCAN estimates of incidence and mortality worldwide for 36 cancers in 185 countries. CA: Cancer J Clin. 2024;74(3):229‐263. 38572751 10.3322/caac.21834

[2] Arnold M , Park JY , Camargo MC , Lunet N , Forman D , Soerjomataram I . Is gastric cancer becoming a rare disease? A global assessment of predicted incidence trends to 2035. Gut. 2020;69(5):823‐829. 32001553 10.1136/gutjnl-2019-320234 PMC8520492

[3] Tanikawa C , Kamatani Y , Toyoshima O , et al. Genome‐wide association study identifies gastric cancer susceptibility loci at 12q24.11‐12 and 20q11.21. Cancer Sci. 2018;109(12):4015‐4024. 30281874 10.1111/cas.13815 PMC6272082

[4] Yan C , Zhu M , Ding Y , et al. Meta‐analysis of genome‐wide association studies and functional assays decipher susceptibility genes for gastric cancer in Chinese populations. Gut. 2020;69(4):641‐651. 31383772 10.1136/gutjnl-2019-318760

[5] Malekzadeh R , Derakhshan MH , Malekzadeh Z . Gastric cancer in Iran: epidemiology and risk factors. Archives Iranian Med. 2009;12(6):576‐583. 19877751

[6] de Leeuw CA , Mooij JM , Heskes T , Posthuma D . MAGMA: generalized gene‐set analysis of GWAS data. PLoS Comput Biol. 2015;11(4):e1004219. 25885710 10.1371/journal.pcbi.1004219 PMC4401657

[7] Kim HM , Kang B , Park S , et al. Forkhead box protein D2 suppresses colorectal cancer by reprogramming enhancer interactions. Nucleic Acids Res. 2023;51(12):6143‐6155. 37158258 10.1093/nar/gkad361 PMC10325893

[8] Jin G , Lv J , Yang M , et al. Genetic risk, incident gastric cancer, and healthy lifestyle: a meta‐analysis of genome‐wide association studies and prospective cohort study. Lancet Oncol. 2020;21(10):1378‐1386. 33002439 10.1016/S1470-2045(20)30460-5

[9] Teng YS , Chen WY , Yan ZB , et al. L‐plastin promotes gastric cancer growth and metastasis in a *Helicobacter pylori* cagA‐ERK‐SP1‐dependent manner. Mol Can Res. 2021;19(6):968‐978. 10.1158/1541-7786.MCR-20-0936 33771880

[10] Chen XL , Hong LL , Wang KL , et al. Deregulation of CSMD1 targeted by microRNA‐10b drives gastric cancer progression through the NF‐kappaB pathway. Int J Biol Sci. 2019;15(10):2075‐2086. 31592231 10.7150/ijbs.23802 PMC6775299

[11] Kim YI , Pecha RL , Keihanian T , et al. MUC1 Expressions and its prognostic values in us gastric cancer patients. Cancers. 2023;15(4). 10.3390/cancers15040998 PMC9954699 36831343

[12] Rajabi H , Kufe D . MUC1‐C oncoprotein integrates a program of EMT, epigenetic reprogramming and immune evasion in human carcinomas. Biochimica et biophysica acta Rev Cancer. 2017;1868(1):117‐122. 10.1016/j.bbcan.2017.03.003 PMC5548633 28302417

[13] Echizen K , Hirose O , Maeda Y , Oshima M . Inflammation in gastric cancer: interplay of the COX‐2/prostaglandin E2 and Toll‐like receptor/MyD88 pathways. Cancer Sci. 2016;107(4):391‐397. 27079437 10.1111/cas.12901 PMC4832872

[14] Luo Q , Liu M , Tan Y , et al. Blockade of prostaglandin E2 receptor 4 ameliorates peritoneal dialysis‐associated peritoneal fibrosis. Frontiers in Pharmacol. 2022;13:1004619. 10.3389/fphar.2022.1004619 PMC9691893 36438844

[15] Watanabe K , Taskesen E , van Bochoven A , Posthuma D . Functional mapping and annotation of genetic associations with FUMA. Nature Commun. 2017;8(1):1826. 29184056 10.1038/s41467-017-01261-5 PMC5705698

[16] Giambartolomei C , Vukcevic D , Schadt EE , et al. Bayesian test for colocalisation between pairs of genetic association studies using summary statistics. PLoS Genetics. 2014;10(5):e1004383. 24830394 10.1371/journal.pgen.1004383 PMC4022491

