# Supplementary material for: Genetic architecture of gastric adenocarcinoma in West Asia
Source: Clin Transl Med. 2025 Sep 25;15(10):e70489. doi: 10.1002/ctm2.70489 (PMC12464345; doi:10.1002/ctm2.70489)
Supplement: Supplementary file 1 — Supporting information [file CTM2-15-e70489-s001.pdf]

Supplementary Materials for

Genetic Architecture of Gastric Adenocarcinoma in West Asia

Saeid Latifi-Navid *et al.*

*Corresponding author. Email: s_latifi@uma.ac.ir

This PDF file includes:

1. Supplementary methods
- **1.1** Gastric cancer GWAS
- **1.2** Genotyping, quality control, and imputation
- **1.3** Genetic association analysis
- **1.4** Functional analysis
- **1.4.1** Genomic risk loci characterization
- **1.4.2** Candidate SNPs annotation
- **1.4.3** Gene mapping
- **1.4.4** Multi-marker Analysis of GenoMic Annotation (MAGMA) gene, gene-set, and expression analysis
- **1.4.5** Gene-set enrichment pathway analysis
- **1.4.6** Differentially expressed genes (DEG) analysis
- **1.4.7** Colocalization analysis

2. Supplementary tables
- **Supplementary table S1.** Demographic characteristics of study participants
- **Supplementary table S2.** Summary of association results for the gastric cancer risk-related loci that were reported in previous studies.
- **Supplementary table S3.** Genomic risk loci
- **Supplementary table S4.** List of 93 novel SNPs
- **Supplementary table S5.** Candidate SNPs with chr, bp, *p*-value, CADD, RDB, nearest gene, genomic risk loci and lead SNPs
- **Supplementary table S6.** MAGMA gene-based test
- **Supplementary table S7.** Genes implicated by positional, eQTL, or chromatin interaction mapping of SNPs
- **Supplementary table S8.** MAGMA gene-set analysis
- **Supplementary table S9.** Gene set enrichment pathway analysis (only significant gene sets included)
- **Supplementary table S10.** MAGMA tissue expression analysis

Supplementary table S11. Enrichment test results of DEG sets
Supplementary table S12. Colocalization analysis of GWAS significant findings

3. Supplementary figures

Supplementary figure S1. Quantile-quantile plot and genomic inflation factor lambda for associations with gastric cancer risk in this GWAS
Supplementary figure S2. Ethnicity and population structure determined by top two principal components for each study
Supplementary figure S3. The distribution of polygenic risk score between gastric cancer cases and controls of this GWAS
Supplementary figure S4. Regional plots of three novel gastric cancer susceptibility loci on 2p13.1, 8p23.2, and 13q14.13 for the location of the tumour within the stomach (cardia or non-cardia) as well as two reported gastric cancer susceptibility loci on 1q22 and 5p13.1 for pathologic subtypes (northwestern Iran)
Supplementary figure S5. A-M, The Circos plots show which genes were linked to genomic risk loci (blue regions) in the GC GWAS on chromosomes 1, 4, 5, 6, 7, 8, 9, 10, 11, 12, 17, 20, and 22 based on eQTL mapping (dark green lines connecting eQTL SNPs to genes) and/or chromatin interactions (orange lines connecting two interacting regions).

4. References

1. Supplementary methods

1.1 Gastric cancer GWAS—We recruited 2061 cancer patients (918 cardia GC patients, 982 noncardia GC patients, and 161 not otherwise specified (NOS) GC patients) and 2034 cancer-free controls from three high-risk areas of Iran, West Asia. In total, 3004 samples were collected from Ardabil, 478 from Mazandaran, and 613 from Golestan provinces. Cases were selected from patients who had undergone endoscopy at Imam Khomeini Hospital in Ardabil and Sari, Shohada Hospital in Gonbad, and Sayyad Shirazi Hospital in Gorgan City. Cancer-free control subjects were selected from among subjects who received routine physical examinations at the same hospital. Controls were frequency-matched to cases by sex and 5-year age groups. Gastric adenocarcinoma was diagnosed based on endoscopic and histopathological results. We further classified the tumours by anatomic site according to the 10th Revision of the International Classification of Diseases (C16.0, cardia; and C16.1–C16.9, noncardia involving overlapping and unspecified subsites) [https://apps.who.int/iris/handle/10665/42980]. According to Lauren's classification [https://doi.org/10.1111/apm.1965.64.1.31], histological subtypes were evaluated as intestinal, diffuse, and mixed types. All participants signed an informed consent form. This study was approved by the Ethics Committee of the National Institute for Medical Research Development (NIMAD)/IR.NIMAD.REC.1396.097.

1.2 Genotyping, quality control, and imputation—DNA genotyping was performed by using the Infinium™ Global Screening Array-24 (v3.0). An Illumina iScan System was used to complete genotyping according to the manufacturer's protocols. We used snpQT (v0.1.7)[1], a scalable software pipeline implemented in NextFlow (v21.04.3)[2], Unix command line tools, and R for interactive quality control (QC), population stratification, phasing, and imputation analysis of genomic data. QC for individuals was performed to remove those with (1) per-individual call rate < 95%; (2) per-individual autosomal heterozygosity > 5 s.d. away from the mean; (3) sex discrepancies between the assigned sex and the genotype-based sex; and (4) cryptic relationships based on relationship-based pruning (king cut-off > 0.354 (geometric mean of 0.5 and 0.25)). Population structure was tested by principal component analysis (PCA) based on autosomal LD-pruned SNPs ($r^2<0.05$) using PLINK2 (v2.00a2.3)[3]. Population outliers, i.e., those with more than 6 s.d. from the mean on each of the top ten principle components (PCs), were recognised by smartpca from EIGENSOFT (v7.2.1)[4] and removed by five iterations.

We further excluded SNPs with (1) Hardy–Weinberg equilibrium (HWE) test $p < 10^{-12}$ in cases and $p < 10^{-7}$ in controls; (2) per SNP call rate < 95% in either cases or controls; (3) not mapped to autosomes; (4) minor allele frequency (MAF) < 1% in either cases or controls; and (5) significant differences in call rates between cases and controls ($p < 10^{-7}$). Filtered genotypes were phased for each chromosome using SHAPEIT4 (v4.1.3)[5]. Imputation was performed in nonoverlapping 5-Mb intervals using IMPUTE5 (v1.1.4)[6] based on haplotypes derived from the Phase III integrated variant set of the 1000 Genomes Project consisting of 2,504 samples. SNPs with an imputation quality score INFO < 0.5 or MAF < 0.01 were excluded from the analysis.

1.3 Genetic association analysis—To calculate per-allele odds ratios (ORs) and standard errors (SEs), we used logistic regression analysis with adjustments for age, sex, and the top 10 PCs of population structure with SNPTEST (v2.5.6)[7] based on a probabilistic dosage model. We performed replication analysis on the selected SNPs in a large published GC GWAS dataset including 10254 gastric cancer patients and 10914 controls from the Chinese population[8]. Then, the β estimates and SEs from the two studies were combined with an inverse variance-weighted fixed-effects method.

1.4 Functional analysis

1.4.1 Genomic risk loci characterisation—We used FUMA (v1.3.5) [9] to obtain functional information about the corresponding SNPs in these loci. FUMA prioritises the most likely causal SNPs and genes by applying multiple biological resources. First, independent significant SNPs (Ind.Sig. SNPs that had an association p value $< 1\times10^{-5}$ and were independent of each other at $r^2 < 0.6$ were identified. Based on Ind.Sig. SNPs were defined as independent lead SNPs that were in approximate linkage equilibrium with each other at $r^2 < 0.1$. To define independent genomic risk loci, we identified physical

regions > 250 kb apart (the maximum distance between LD blocks to merge into a locus) in LD with these lead SNPs. All known SNPs that had $r^2 \geq 0.6$ with one of the Ind.Sig. SNPs were also included. Each single independent genomic risk locus could thus contain all these candidate SNPs.

1.4.2 Candidate SNP annotation—ANNOVAR [10]—was performed to determine the functional implications of SNPs on genes using Ensembl genes (build 85). Combined annotation-dependent depletion (CADD) [11] and RegulomeDB [12] scores as well as 15-core chromatin states [13] were annotated to the SNPs in the 1000 Genomes Project phase 3 by matching chromosome locations, base-pair positions, alternative alleles, and references. CADD scores predict how deleterious a SNP's effect is to protein structure/function, as the higher the score, the more deleterious the effect of the SNP. The potential pathogenicity threshold was > 12.37. The RegulomeDB score is based on eQTLs (expression quantitative trait loci) and chromatin signatures, which range from 1a to 6. Lower scores indicate an elevated likelihood of regulatory function. The chromatin state indicates the reachability of the genomic regions (each 200 bp) with 15 categorical states of the 127 reference epigenomes in the Roadmap Epigenomics Project [14]. A lower state represents greater accessibility, and states 1-7 refer to open chromatin states.

1.4.3 Gene mapping—We used FUMA to map genome-wide significant loci to genes using three strategies:

(1) Positional mapping. SNPs were mapped to genes by physical distance (maximum distance: 30 kb) from known genes encoding proteins in the human reference assembly (GRCh37/hg19).

(2) eQTL mapping. SNPs were mapped to genes with which they exhibit significant eQTL associations (i.e., a gene whose expression level is associated with SNP allelic differences) using 324 normal stomach tissue samples from the GTEx v8 stomach data repository [15]. Based on the cis-eQTLs, we mapped SNPs to genes up to 1 Mb apart with a p value of < 0.05 for all significant SNP–gene pairs.

(3) Chromatin interaction mapping [16]. SNPs were mapped to genes by chromatin interaction. A SNP region interacts with another gene region in a three-dimensional manner without distance boundaries. Custom chromatin interaction matrices were built using gastric tissue Hi-C data from GSE86189 [17]. Chromatin interactions are defined at a resolution of 40 kb, allowing an interaction region to span multiple genes. If a SNP is placed in a region interacting with another region containing multiple genes, it is mapped to each of them. We used tissue-/cell type-specific predicted enhancers and promoters from the Epigenomics Roadmap project, including the GI Stomach, to prioritise candidate genes. FUMA identified significant chromatin interactions if one region overlapped with predicted enhancers and another overlapped with predicted promoters (250 bp upstream and 500 bp downstream of the TSS).

1.4.4 Multimarker analysis of the GenoMic Annotation (MAGMA) gene, gene set, and expression analysis—We used FUMA to compute gene-based and gene-set p values using MAGMA [18]. The gene-based p value was computed by mapping the input SNPs for 19351 protein-coding genes. Genome-wide significance was set at $p = 0.05/19351 = 2.58\times10^{-6}$. We used the gene-based p value for 10678 gene sets (Gene Ontology (GO) terms: 5917, curated gene sets: 4761) from MSigDB (v6.2) [19] to compute the gene set p value. A SNPwise model was used for the MAGMA gene-based analysis, while a competitive model was applied for gene-set analysis. As a reference panel, we used 1000G phase 3 to calculate LD across SNPs and genes. The Bonferroni significance threshold was set at $p = 0.05/10678 = 4.68\times10^{-6}$, correcting for the total number of gene sets tested. MAGMA gene property analysis was performed using gene-based p values, and average expression across all tissue types was conditionally tested on one side (greater). The GTEx v8 54 tissue type database [15] was used to obtain gene expression levels.

1.4.5 Gene-set enrichment pathway analysis—Hypergeometric tests in FUMA were used to enrich all GC risk-associated genes with MAGMA gene-based p values < 0.05 in the MSigDB gene sets, which consisted of KEGG, BioCarta, GO, and Reactome. A Benjamini–Hochberg adjusted p value (false discovery rate (FDR)) ≤ 0.05 was used. The number of genes overlapping with the gene set was > 1.

1.4.6 Differentially expressed gene (DEG) analysis—FUMA was used to construct a gene expression heatmap of prioritised genes from 54 tissues obtained from GTEx [15] (an average expression value per tissue per gene following $\log_2$ transformation with pseudocount 1 after winsorization at 50). Tissue specificity was tested by using DEGs defined for each of 54 tissue types in each expression dataset. The normalised expression ($\log_2$(RPKM+1) zero-mean) was applied. Two-sided *t* tests were performed per gene per tissue against all other genes. In addition, by considering the t score, we differentiated between genes whose expression was up-/downregulated in a specific tissue relative to that in other tissues. Genes with an absolute logarithmic fold change ≥ 0.58 and a Bonferroni-corrected p value < 0.05 were considered DEGs in a specific tissue relative to others.

1.4.7 Colocalization analysis—A Bayesian colocalization analysis was conducted between GWAS loci and GTEx v8 stomach tissue eQTL dataset using the R package coloc [20]. It allows the identification of shared causal variants responsible for both GWAS and gene expression in a genomic region of interest. The GWAS loci were defined as regions 1 Mb upstream and downstream of the regional lead SNPs. For each gene of a region, a posterior probability was obtained; the greater the posterior probability was (PP4 > 0.7), the stronger the evidence for colocalization. All plots were generated using locuscomparer [21].

2. Supplementary tables

Supplementary table S1. Demographic characteristics of study participants

	**Counts (%)**			**Percent male**			**Age in years, mean (SD)**			**Tumour location**			**Histological subtype**		
**Province**	**Cases**	**Controls**	**All**	**Cases**	**Controls**	**All**	**Cases**	**Controls**	**All**	**Cardia GC (%)**	**Non-cardia GC (%)**	**NOS[a] (%)**	**Intestinal-type GC (%)**	**Diffuse-type GC (%)**	**Mixed (%)**
**Ardabil**	1517 (50.5)	1487 (49.5)	3004	75.1%	74.9%	75.0%	65.9 (10.9)	69.4 (10.1)	67.6 (10.7)	689 (45.4)	699 (46.1)	129 (8.5)	878 (57.9)	394 (25.9)	245 (16.2)
**Mazandaran**	238 (49.8)	240 (50.2)	478	74.4%	74.6%	74.5%	67.3 (11.9)	66.8 (11.7)	67.1 (11.8)	66 (27.7)	161 (67.7)	11 (4.6)	103 (43.3)	99 (41.6)	36 (15.1)
**Golestan**	306 (49.9)	307 (50.1)	613	70.3%	68.4%	69.3%	63.9 (10.7)	60.6 (10.8)	62.2 (10.9)	163 (53.3)	122 (39.9)	21 (6.8)	203 (66.3)	56 (18.3)	47 (15.4)
**All**	2061 (50.3)	2034 (49.7)	4095	74.3%	73.9%	74.1%	65.8 (11.0)	67.8 (10.9)	66.8 (11.0)	918 (44.5)	982 (47.7)	161 (7.8)	1184 (57.5)	549 (26.6)	328 (15.9)

[a]Not otherwise specified

Supplementary table S2. Summary of association results for the gastric cancer risk-related loci that were reported in previous studies.

No.	Chr.	Variant	Associated genes	Alleles (Ref/Eff) [a]	EAF	OR (95%CI) [b]	*P* [b]	EAF in 1000 Genomes [c]				Most significant associations in previous reports [d]			
								AFR	AMR	ASN	EUR	OR	*P*	Eff allele	Study
1	1p35.2	rs112754928	*SPOCD1*	G/A	-	-	-	0	0	0.02	0	0.54 (0.44-0.67)	2.33E-08	A	[22]
2	1q22	rs760077	*MUC1*	T/A	0.46	0.72 (0.66-0.79)	1.15E-12	0.36	0.30	0.17	0.37	0.79 (0.73-0.85)	1.10E-09	A	[23]
		rs140081212	*MUC1*	G/A	0.46	0.72 (0.66-0.79)	2.31E-12	0.35	0.30	0.18	0.37	0.79 (0.73-0.85)	7.90E-10	A	[23]
		rs4072037	*MUC1*	A/G	0.58	0.75 (0.68-0.82)	2.20E-10	0.34	0.34	0.16	0.41	0.74 (0.69-0.79)	6.28E-17	G	[24-26]
		rs80142782	*ASH1L*	T/C	-	-	-	0	0	0.09	0	0.62 (0.56-0.69)	1.71E-19	C	[26]
3	2p11.2	chr2:86020821	intergenic	G/T	-	-	-	-	-	-	-	12.70 (5.03-32.08)	7.60E-08	T	[23]
4	3q11.2	rs7624041	*NSUN3*	G/A	0.90	1.03 (0.89-1.19)	7.17E-01	0.79	0.93	0.95	0.93	1.21 (1.13-1.28)	4.56E-09	G	[27]
5	3q13.31	rs9841504	*ZBTB20*	A/G	0.11	0.90 (0.78-1.05)	1.85E-01	0.34	0.17	0.16	0.08	0.58 (0.47-0.72)	6.10E-07	A	[28]
6	4q28.1	rs10029005	*ANKRD50*	A/G	0.71	0.82 (0.75-0.91)	9.09E-05	0.59	0.69	0.63	0.59	1.14 (1.10-1.19)	3.33E-11	A	[27]
7	5p13.1	rs10074991	*PRKAA1*	A/G	0.67	1.27 (1.15-1.41)	1.89E-06	0.67	0.7	0.46	0.71	1.25 (1.20-1.30)	4.83E-26	G	[25]
		rs10036575	*PRKAA1*	C/T	0.68	1.27 (1.15-1.41)	3.93E-06	0.71	0.74	0.46	0.77	1.23	4.80E-06	T	[23]
		rs13361707	*PRKAA1*	T/C	0.67	1.27 (1.15-1.41)	2.00E-06	0.67	0.7	0.46	0.71	1.32 (1.13-1.43)	9.70E-11	C	[26,28]
8	5q14.3	rs7712641	*lnc-POLR3G-4*	T/C	0.64	1.02 (0.93-1.13)	6.26E-01	0.32	0.59	0.58	0.65	0.84 (0.80-0.88)	1.21E-11	T	[26]
9	6p21.1	rs2294693	*UNC5CL, TSPO2*	T/C	0.21	1.01 (0.90-1.14)	8.26E-01	0.29	0.26	0.26	0.18	1.18 (1.12-1.26)	2.50E-08	C	[25]
10	6p22.1	rs1679709	*BTN3A2*	G/A	0.08	1.13 (0.97-1.33)	1.24E-01	0.23	0.12	0.14	0.12	0.80 (0.76-0.85)	1.04E-12	A	[22]
11	8q24.3	rs2294008	*PSCA*	C/T	0.38	1.37 (1.25-1.51)	5.03E-11	0.38	0.57	0.38	0.44	1.20 (1.15-1.28)	5.95E-11	T	[23,26,29]
12	9q34.2	rs7849280	*ABO*	A/G	0.10	0.97 (0.83-1.14)	7.18E-01	0.2	0.05	0.16	0.06	1.15	2.64E-13	G	[30]
13	10q23.33	rs2274223	*PLCE1, NOC3L*	A/G	0.40	1.10 (1.00-1.21)	4.32E-02	0.35	0.22	0.19	0.32	1.31 (1.19-1.43)	8.40E-09	G	[24]
		rs3765524	*PLCE1, NOC3L*	C/T	0.40	1.09 (0.99-1.20)	6.84E-02	0.42	0.22	0.19	0.29	1.31 (1.20–1.44)	5.32E-09	C	[24]
		rs3781264	*PLCE1, NOC3L*	T/C	0.35	1.12 (1.02-1.23)	2.02E-02	0.17	0.2	0.14	0.3	1.36 (1.23–1.50)	3.76E-09	C	[24]
		rs11187842	*PLCE1, NOC3L*	C/T	0.15	0.99 (0.87-1.13)	9.33E-01	0.03	0.07	0.13	0.09	1.34 (1.21–1.49)	2.53E-09	T	[24]
14	11q22.3	chr11:108137985	*ATM*	C/T	-	-	-	-	-	-	-	4.84 (2.67-8.77)	2.00E-07	T	[23]
		chr11:108124573	*ATM*	C/A	-	-	-	-	-	-	-	7.78 (3.38-17.91)	1.40E-06	A	[23]
15	11q22.3	chr11:102612948	intergenic	!A/A	-	-	-	-	-	-	-	4.90 (2.79-8.62)	3.40E-08	A	[23]
16	12q24.11-12	rs6490061	*CUX2*	C/T	0.75	0.90 (0.81-0.99)	4.00E-02	0.53	0.63	0.42	0.79	0.91	3.20E-08	T	[30]
17	20q11.21	rs2376549	*DEFB* families	T/C	0.44	0.98 (0.89-1.08)	6.90E-01	0.91	0.47	0.28	0.47	1.11	8.11E-10	C	[30]
18	20q12	rs55864139	*CHD6*	T/A	-	-	-	0	0.01	0	0.01	3.12 (1.90-5.11)	6.90E-06	A	[23]

[a] Ref, reference allele; Eff, effect allele; [b] derived from the GWAS results in Iran gastric cancer GWAS;

[c] EAF: effect allele frequency; AFR, African; AMR, Ad Mixed American; ASN: Asian; EUR: European;

[d] The associations were derived from published GWAS studies of gastric cancer, and the most significant associations were extracted.

Supplementary table S3. Genomic risk loci

Genomic Locus	uniqID	rsID	pos	*p*	start	end	nSNPs	nGWASSNPs	nIndSigSNPs	IndSigSNPs	LeadSNPs
1	1:4062730:C:T	1:4062730:A/G	4062730	3.89949e-05	4059365	4097079	14	13	1	1:4062730:A/G	1:4062730:A/G
2	1:4643432:A:C	1:4643432:A/C	4643432	7.13916e-06	4642126	4654329	7	7	1	1:4643432:A/C	1:4643432:A/C
3	1:47952962:C:T	1:47952962:A/G	47952962	2.26271e-08	47871356	47997658	71	68	4	1:47952962:A/G;1:47924371:A/G;1:47935863:A/T;1:47942422:A/G	1:47952962:A/G
4	1:155178782:A:T	1:155178782:A/T	155178782	1.14946e-12	155033308	156042915	64	58	8	1:155178782:A/T;1:155527235:A/C;1:155616555:A/T;1:155876613:A/G;1:155033308:A/G;1:155105882:A/C;1:155155731:A/G;1:156032887:A/G	1:155178782:A/T;1:156032887:A/G
5	1:231259851:A:G	1:231259851:A/G	231259851	4.31459e-05	231188455	231259997	12	11	1	1:231259851:A/G	1:231259851:A/G
6	2:29983090:C:T	2:29983090:A/G	29983090	4.88982e-05	29979985	29988753	17	17	1	2:29983090:A/G	2:29983090:A/G
7	2:38721171:C:G	2:38721171:C/G	38721171	6.23664e-06	38717105	38730814	15	13	1	2:38721171:C/G	2:38721171:C/G
8	2:74448672:A:C	2:74448672:A/C	74448672	6.32622e-06	74406874	74480659	25	24	2	2:74448672:A/C;2:74480659:A/G	2:74448672:A/C
9	2:80173676:A:G	2:80173676:A/G	80173676	3.50557e-05	80153660	80173676	14	14	1	2:80173676:A/G	2:80173676:A/G
10	2:180903712:C:T	2:180903712:A/G	180903712	1.33303e-05	180887549	180975968	43	35	1	2:180903712:A/G	2:180903712:A/G
11	2:224369405:C:T	2:224369405:A/G	224369405	7.31059e-06	224366956	224399923	28	28	1	2:224369405:A/G	2:224369405:A/G
12	2:238742059:C:T	2:238742059:A/G	238742059	2.87108e-05	238718254	238752088	3	3	1	2:238742059:A/G	2:238742059:A/G
13	2:240148856:C:G	2:240148856:C/G	240148856	9.5034e-06	240145208	240172182	13	12	1	2:240148856:C/G	2:240148856:C/G
14	3:28046439:A:G	3:28046439:A/G	28046439	3.87265e-05	28043123	28077836	6	6	1	3:28046439:A/G	3:28046439:A/G
15	3:43186832:C:T	3:43186832:A/G	43186832	8.20181e-06	43181148	43186832	2	2	1	3:43186832:A/G	3:43186832:A/G
16	3:45276160:C:T	3:45276160:A/G	45276160	1.10324e-05	45196727	45280066	18	18	1	3:45276160:A/G	3:45276160:A/G
17	3:88377838:C:G	3:88377838:C/G	88377838	5.35111e-06	88377746	88391859	4	3	1	3:88377838:C/G	3:88377838:C/G
18	3:154294388:C:T	3:154294388:A/G	154294388	1.63293e-05	154186078	154371064	322	287	2	3:154294388:A/G;3:154253687:A/G	3:154294388:A/G
19	4:24850083:C:T	4:24850083:A/G	24850083	3.36899e-05	24850083	24875910	2	2	1	4:24850083:A/G	4:24850083:A/G
20	4:69408461:A:C	4:69408461:A/C	69408461	2.33418e-06	69408461	69443377	2	2	1	4:69408461:A/C	4:69408461:A/C
21	4:90271485:C:T	4:90271485:A/G	90271485	1.87583e-05	90235778	90277638	32	24	1	4:90271485:A/G	4:90271485:A/G
22	4:100663948:A:G	4:100663948:A/G	100663948	1.70524e-06	100639988	100674186	38	37	2	4:100663948:A/G;4:100663968:A/G	4:100663948:A/G
23	4:125419635:A:C	4:125419635:A/C	125419635	4.2513e-06	125390164	125470241	134	118	2	4:125419635:A/C;4:125444404:A/G	4:125419635:A/C
24	4:130215421:A:G	4:130215421:A/G	130215421	2.08295e-05	130205964	130229271	71	63	1	4:130215421:A/G	4:130215421:A/G
25	4:183877690:G:T	4:183877690:A/C	183877690	1.01137e-05	183814104	183889586	61	54	2	4:183877690:A/C;4:183885485:A/G	4:183877690:A/C
26	5:15561046:A:G	5:15561046:A/G	15561046	9.19236e-06	15552869	15574631	6	5	1	5:15561046:A/G	5:15561046:A/G
27	5:40704074:A:G	5:40704074:A/G	40704074	2.95529e-07	40685795	40839206	63	53	2	5:40704074:A/G;5:40812231:A/G	5:40704074:A/G
28	5:106095212:A:G	5:106095212:A/G	106095212	1.14057e-05	106041131	106099371	32	28	1	5:106095212:A/G	5:106095212:A/G
29	5:141093702:A:G	5:141093702:A/G	141093702	3.9255e-06	141086579	141096921	8	8	2	5:141093702:A/G;5:141096265:A/C	5:141093702:A/G
30	5:177820543:C:T	5:177820543:A/G	177820543	6.3013e-07	177818373	177830769	7	6	1	5:177820543:A/G	5:177820543:A/G
31	6:12838853:C:G	6:12838853:C/G	12838853	1.25188e-06	12725378	12873791	6	6	1	6:12838853:C/G	6:12838853:C/G
32	6:140941310:C:T	6:140941310:A/G	140941310	1.32832e-05	140890348	141038357	29	25	1	6:140941310:A/G	6:140941310:A/G
33	6:148171512:C:T	6:148171512:A/G	148171512	2.92365e-05	148040366	148171512	3	3	1	6:148171512:A/G	6:148171512:A/G
34	6:151471421:C:T	6:151471421:A/G	151471421	2.18769e-05	151463492	151471492	6	5	1	6:151471421:A/G	6:151471421:A/G
35	7:31979673:A:G	7:31979673:A/G	31979673	3.98187e-05	31897146	32037357	22	18	1	7:31979673:A/G	7:31979673:A/G
36	7:35084601:C:T	7:35084601:A/G	35084601	4.58641e-05	35084601	35084602	2	2	1	7:35084601:A/G	7:35084601:A/G
37	7:79065472:C:T	7:79065472:A/G	79065472	2.30215e-05	79065472	79107057	11	10	1	7:79065472:A/G	7:79065472:A/G

38	7:79460117:A:G	7:79460117:A/G	79460117	9.20202e-08	79429672	79671968	6	6	1	7:79460117:A/G	7:79460117:A/G
39	7:122962994:A:T	7:122962994:A/T	122962994	8.93596e-06	122939382	122987393	34	28	2	7:122962994:A/T;7:122985615:A/C	7:122962994:A/T
40	7:149488065:C:T	7:149488065:A/G	149488065	6.16555e-06	149488065	149488065	1	1	1	7:149488065:A/G	7:149488065:A/G
41	7:154441305:C:T	7:154441305:A/G	154441305	2.1952e-05	154433651	154461715	23	21	2	7:154441305:A/G;7:154437099:A/G	7:154441305:A/G
42	8:3924335:A:G	8:3924335:A/G	3924335	2.72045e-05	3920204	3925024	10	10	1	8:3924335:A/G	8:3924335:A/G
43	8:90225474:C:G	8:90225474:C/G	90225474	4.91841e-05	90215184	90302714	77	68	1	8:90225474:C/G	8:90225474:C/G
44	8:133641587:C:T	8:133641587:A/G	133641587	1.28386e-05	133591952	133685650	18	17	1	8:133641587:A/G	8:133641587:A/G
45	8:143776668:C:T	8:143776668:A/G	143776668	6.33882e-12	143752235	144064882	395	361	17	8:143776668:A/G;8:143776719:A/C;8:143822208:A/G;8:143842101:A/G;8:143844650:A/G;8:143852964:C/G;8:143856967:C/G;8:143859081:A/G;8:143882420:A/G;8:143887054:A/G;8:143902968:A/G;8:143903181:A/G;8:143752235:A/G;8:143795038:A/G;8:143857208:A/G;8:143903612:A/G;8:144057069:C/G	8:143776668:A/G;8:143795038:A/G;8:143857208:A/G;8:143903612:A/G
46	9:74675922:A:G	9:74675922:A/G	74675922	2.06758e-05	74633850	74833123	26	25	1	9:74675922:A/G	9:74675922:A/G
47	9:104962227:A:G	9:104962227:A/G	104962227	3.36722e-05	104949003	104982511	41	40	1	9:104962227:A/G	9:104962227:A/G
48	9:112539953:C:T	9:112539953:A/G	112539953	5.51506e-06	112539953	112539953	1	1	1	9:112539953:A/G	9:112539953:A/G
49	10:45221505:A:G	10:45221505:A/G	45221505	3.41816e-05	45143639	45246273	38	34	1	10:45221505:A/G	10:45221505:A/G
50	10:96070375:A:G	10:96070375:A/G	96070375	0.0201937	96042893	96126999	52	42	3	10:96070375:A/G;10:96058298:A/G;10:96066341:A/G	10:96070375:A/G
51	10:102776360:C:T	10:102776360:A/G	102776360	8.18008e-06	102776360	102786536	10	9	1	10:102776360:A/G	10:102776360:A/G
52	10:112567284:C:G	10:112567284:C/G	112567284	2.065e-05	112565606	112578067	10	8	1	10:112567284:C/G	10:112567284:C/G
53	11:84969976:C:T	11:84969976:A/G	84969976	2.90707e-06	84932885	85513048	13	12	2	11:84969976:A/G;11:85033761:A/G	11:84969976:A/G
54	11:90696508:A:C	11:90696508:A/C	90696508	2.43125e-05	90646762	90784049	3	2	1	11:90696508:A/C	11:90696508:A/C
55	11:99047448:A:G	11:99047448:A/G	99047448	2.98744e-06	99047448	99346661	3	1	1	11:99047448:A/G	11:99047448:A/G
56	12:1485129:C:G	12:1485129:C/G	1485129	9.62982e-06	1179785	1579408	11	11	1	12:1485129:C/G	12:1485129:C/G
57	12:45932569:C:T	12:45932569:A/G	45932569	2.08015e-05	45857819	45948628	19	16	1	12:45932569:A/G	12:45932569:A/G
58	12:119288710:A:T	12:119288710:A/T	119288710	1.81082e-06	119278835	119303195	19	17	3	12:119285230:A/G;12:119285121:A/G;12:119288710:A/T	12:119285230:A/G;12:119288710:A/T
59	13:40129220:A:C	13:40129220:A/C	40129220	8.24609e-06	40095218	40149810	49	47	3	13:40129220:A/C;13:40136832:A/G;13:40108439:A/C	13:40129220:A/C
60	13:46777563:G:T	13:46777563:A/C	46777563	3.93174e-05	46755852	46786087	16	15	1	13:46777563:A/C	13:46777563:A/C
61	13:50282170:C:T	13:50282170:A/G	50282170	8.97777e-06	50282170	50282170	1	1	1	13:50282170:A/G	13:50282170:A/G
62	13:81442734:A:G	13:81442734:A/G	81442734	6.3056e-06	81292588	81617834	131	116	4	13:81442734:A/G;13:81561325:A/G;13:81390720:A/G;13:81441795:A/G	13:81442734:A/G
63	13:82064770:G:T	13:82064770:A/C	82064770	3.73569e-05	82042699	82085654	25	25	1	13:82064770:A/C	13:82064770:A/C
64	14:31343135:C:T	14:31343135:A/G	31343135	6.00526e-06	31325883	31343135	13	9	1	14:31343135:A/G	14:31343135:A/G
65	15:37611287:A:C	15:37611287:A/C	37611287	9.30247e-06	37584101	37630205	79	78	1	15:37611287:A/C	15:37611287:A/C
66	15:87449577:C:T	15:87449577:A/G	87449577	3.2341e-06	87439330	87458828	19	18	1	15:87449577:A/G	15:87449577:A/G
67	15:88368416:A:C	15:88368416:A/C	88368416	1.33672e-06	88368416	88368416	1	1	1	15:88368416:A/C	15:88368416:A/C
68	16:79400539:C:G	16:79400539:C/G	79400539	9.59564e-06	79331216	79452042	53	51	2	16:79400539:C/G;16:79415503:A/G	16:79400539:C/G
69	16:89514364:A:C	16:89514364:A/C	89514364	1.03529e-05	89511210	89612511	11	10	1	16:89514364:A/C	16:89514364:A/C
70	17:11655126:G:T	17:11655126:A/C	11655126	2.58207e-05	11579621	11665655	61	51	2	17:11655126:A/C;17:11642231:A/G	17:11655126:A/C
71	17:18967266:C:G	17:18967266:C/G	18967266	5.08941e-06	18967266	18967266	1	1	1	17:18967266:C/G	17:18967266:C/G
72	17:25541278:A:C	17:25541278:A/C	25541278	3.85254e-14	25271136	25541278	27	1	1	17:25541278:A/C	17:25541278:A/C

73	17:43991509:A:C	17:43991509:A/C	43991509	1.06225e-05	43934332	43999203	16	12	2	17:43991509:A/C;17:43962562:A/G	17:43991509:A/C
74	17:73893155:A:G	17:73893155:A/G	73893155	2.19773e-05	73863391	73893155	3	3	1	17:73893155:A/G	17:73893155:A/G
75	18:36922580:C:G	18:36922580:C/G	36922580	7.83492e-08	36096440	37799564	13	1	1	18:36922580:C/G	18:36922580:C/G
76	18:54484203:C:T	18:54484203:A/G	54484203	3.44005e-05	54484203	54532135	4	4	1	18:54484203:A/G	18:54484203:A/G
77	19:5812913:A:G	19:5812913:A/G	5812913	3.20454e-06	5812913	5853556	4	4	1	19:5812913:A/G	19:5812913:A/G
78	19:9224157:A:G	19:9224157:A/G	9224157	1.44264e-06	9205709	9224157	23	21	2	19:9224157:A/G;19:9224099:A/G	19:9224157:A/G
79	20:6693128:C:T	20:6693128:A/G	6693128	9.48773e-06	6690101	6706493	12	11	1	20:6693128:A/G	20:6693128:A/G
80	20:19934733:A:G	20:19934733:A/G	19934733	1.91218e-06	19934733	19946056	21	18	2	20:19934733:A/G;20:19935365:C/G	20:19934733:A/G
81	20:62486721:A:G	20:62486721:A/G	62486721	1.07164e-05	62486721	62486735	2	2	1	20:62486721:A/G	20:62486721:A/G
82	21:23023268:A:G	21:23023268:A/G	23023268	2.09546e-05	22920834	23072041	6	5	1	21:23023268:A/G	21:23023268:A/G
83	21:25199568:C:T	21:25199568:A/G	25199568	9.52755e-06	25199568	25238521	4	4	1	21:25199568:A/G	21:25199568:A/G
84	21:43948272:A:G	21:43948272:A/G	43948272	3.74244e-05	43940264	43973987	31	27	1	21:43948272:A/G	21:43948272:A/G
85	22:19577517:C:G	22:19577517:C/G	19577517	1.04826e-06	18716159	20323953	34	10	1	22:19577517:C/G	22:19577517:C/G
86	22:32849712:A:C	22:32849712:A/C	32849712	4.06314e-05	32844697	32855598	11	10	1	22:32849712:A/C	22:32849712:A/C
87	22:36132770:A:T	22:36132770:A/T	36132770	8.77288e-06	36132770	36132770	1	1	1	22:36132770:A/T	22:36132770:A/T
88	22:43181587:C:T	22:43181587:A/G	43181587	3.19575e-05	43165729	43206167	8	7	1	22:43181587:A/G	22:43181587:A/G
89	22:50974100:A:G	22:50974100:A/G	50974100	3.06206e-06	50945256	51004421	29	25	3	22:50974100:A/G;22:51004421:A/G;22:50945256:A/G	22:50974100:A/G

Supplementary table S4. List of 93 novel SNPs

SNP	CHR	BP	MarkerName	Effect_allele	Other_allele	BETA	SE	EAF	OR (95% CI)	*P*	Strand	N
rs78082845	1	4062730	1:4062730:A/G	C	T	0/984889	0/239437	0/00706313	2.68 (1.67-4.28)	3/90E-05	+	3686
rs12046016	1	47924371	1:47924371:A/G	A	G	-0/463093	0/100867	0/9571493	0.63 (0.52-0.77)	4/41E-06	+	3686
rs533894	1	47935863	1:47935863:A/T	A	T	-0/425801	0/0965298	0/9520744	0.65 (0.54-0.79)	1/03E-05	+	3686
rs545670	1	47942422	1:47942422:A/G	T	C	0/452972	0/0969975	0/0490401	1.57 (1.30-1.90)	3/01E-06	+	3686
rs498352	1	47952962	1:47952962:A/G	C	T	0/548826	0/0981689	0/0444057	1.73 (1.43-2.10)	2/26E-08	+	3686
rs374211215	1	171362554	1:171362554:A/G	C	T	1/00878	0/243286	0/01211	2.74 (1.70-4.42)	3/38E-05	+	3686
rs74143559	1	231259851	1:231259851:A/G	A	G	-0/438652	0/107251	0/9592569	0.64 (0.52-0.80)	4/31E-05	+	3686
rs2631943	2	29983090	2:29983090:A/G	T	C	-0/363802	0/0895886	0/123498	0.70 (0.58-0.83)	4/89E-05	+	3686
rs77347022	2	38721171	2:38721171:C/G	C	G	-0/536591	0/118762	0/9635671	0.58 (0.46-0.74)	6/24E-06	+	3686
rs1020485	2	74448672	2:74448672:A/C	C	A	0/367675	0/0814308	0/893411	1.44 (1.23-1.69)	6/33E-06	+	3686
rs17009774	2	74480659	2:74480659:A/G	C	T	0/369175	0/0843608	0/887966	1.45 (1.23-1.71)	1/21E-05	+	3686
rs10172408	2	80173676	2:80173676:A/G	A	G	0/403085	0/0974139	0/9271822	1.50 (1.24-1.81)	3/51E-05	+	3686
rs68035236	2	180903712	2:180903712:A/G	T	C	0/26598	0/0610802	0/795326	1.30 (1.16-1.47)	1/33E-05	+	3686
rs13391589	2	224369405	2:224369405:A/G	C	T	0/341197	0/0760847	0/870535	1.41 (1.21-1.63)	7/31E-06	+	3686
rs1198814	2	238742059	2:238742059:A/G	T	C	-0/320067	0/0765078	0/910299	0.73 (0.62-0.84)	2/87E-05	+	3686
rs3791618	2	240148856	2:240148856:C/G	G	C	-0/445582	0/100624	0/9409996	0.64 (0.53-0.78)	9/50E-06	+	3686
rs147670283	3	28046439	3:28046439:A/G	A	G	-0/510758	0/124123	0/0480434	0.60 (0.47-0.77)	3/87E-05	+	3686
rs11720364	3	43186832	3:43186832:A/G	C	T	-0/47184	0/105797	0/0852821	0.62 (0.51-0.77)	8/20E-06	+	3686
rs75858643	3	45276160	3:45276160:A/G	C	T	0/405481	0/0922411	0/917233	1.50 (1.25-1.80)	1/10E-05	+	3686
rs4859012	3	88377838	3:88377838:C/G	G	C	0/225909	0/0496445	0/303937	1.25 (1.14-1.38)	5/35E-06	-	3686
rs1159967	3	154253687	3:154253687:A/G	C	T	0/353165	0/0847032	0/9035265	1.42 (1.21-1.68)	3/05E-05	+	3686
rs7621176	3	154294388	3:154294388:A/G	C	T	0/362152	0/0840271	0/89816	1.44 (1.22-1.69)	1/63E-05	+	3686
rs147041430	4	24850083	4:24850083:A/G	T	C	-0/536803	0/129445	0/0435926	0.58 (0.45-0.75)	3/37E-05	+	3686
rs28475836	4	90271485	4:90271485:A/G	T	C	-0/205411	0/0480024	0/470465	0.81 (0.74-0.89)	1/88E-05	+	3686
rs56411289	4	100663948	4:100663948:A/G	A	G	0/542508	0/113364	0/9365467	1.72 (1.38-2.15)	1/71E-06	+	3686
rs71614614	4	100663968	4:100663968:A/G	A	G	0/527902	0/115808	0/9407758	1.70 (1.35-2.13)	5/15E-06	+	3686
rs12508680	4	130215421	4:130215421:A/G	G	A	-0/225422	0/0529684	0/768585	0.80 (0.72-0.89)	2/08E-05	+	3686
rs368996249	4	183877690	4:183877690:A/C	T	G	-0/478743	0/108442	0/9575106	0.62 (0.50-0.77)	1/01E-05	+	3686
rs2714509	4	183885485	4:183885485:A/G	G	A	-0/427083	0/0995268	0/9499515	0.65 (0.54-0.79)	1/78E-05	+	3686
rs4702085	5	15561046	5:15561046:A/G	A	G	-0/434299	0/0979178	0/9518063	0.65 (0.53-0.78)	9/19E-06	+	3686
rs79527399	5	106095212	5:106095212:A/G	G	A	0/508595	0/115889	0/9460438	1.66 (1.33-2.09)	1/14E-05	+	3686
rs182991	5	141093702	5:141093702:A/G	A	G	0/232086	0/0502862	0/565262	1.26 (1.14-1.39)	3/93E-06	+	3686
rs248623	5	141096265	5:141096265:A/C	T	G	0/204517	0/0492176	0/464169	1.23 (1.11-1.35)	3/25E-05	+	3686
rs115733757	6	12838853	6:12838853:C/G	C	G	-0/818466	0/168852	0/0289549	0.44 (0.32-0.61)	1/25E-06	+	3686
rs79377896	6	140941310	6:140941310:A/G	T	C	-0/576098	0/132272	0/0418051	0.56 (0.43-0.73)	1/33E-05	+	3686
rs12196571	6	148171512	6:148171512:A/G	T	C	0/595749	0/142546	0/026115	1.81 (1.37-2.40)	2/92E-05	+	3686
rs12179653	6	151471421	6:151471421:A/G	C	T	-0/484261	0/114083	0/9533669	0.62 (0.49-0.77)	2/19E-05	+	3686

rs34794532	7	31979673	7:31979673:A/G	A	G	0/221888	0/0540066	0/712507	1.25 (1.12-1.39)	3/98E-05	+	3686
rs370196636	7	35084601	7:35084601:A/G	T	C	-0/419981	0/103043	0/9062566	0.66 (0.54-0.80)	4/59E-05	+	3686
rs4730833	7	79065472	7:79065472:A/G	C	T	-0/635845	0/150198	0/9808111	0.53 (0.39-0.71)	2/30E-05	+	3686
rs146931367	7	79460117	7:79460117:A/G	G	A	-1/44542	0/270586	0/0175001	0.24 (0.14-0.40)	9/20E-08	+	3686
rs77543808	7	122985615	7:122985615:A/C	C	A	-0/236487	0/0576966	0/809606	0.79 (0.70-0.88)	4/15E-05	+	3686
rs7786902	7	154437099	7:154437099:A/G	A	G	0/227448	0/0551557	0/234163	1.26 (1.13-1.40)	3/73E-05	+	3686
rs12538959	7	154441305	7:154441305:A/G	T	C	0/217495	0/0512473	0/276447	1.24 (1.12-1.37)	2/20E-05	+	3686
rs10217067	8	3924335	8:3924335:A/G	G	A	0/205266	0/0489231	0/640365	1.23 (1.12-1.35)	2/72E-05	+	3686
rs11989379	8	90225474	8:90225474:C/G	G	C	-0/324352	0/0799001	0/9192466	0.72 (0.62-0.85)	4/92E-05	+	3686
rs7462984	8	133641587	8:133641587:A/G	C	T	0/209537	0/0480276	0/541372	1.23 (1.12-1.35)	1/28E-05	+	3686
rs36126387	9	74675922	9:74675922:A/G	G	A	0/78307	0/183929	0/0115454	2.19 (1.53-3.14)	2/07E-05	+	3686
rs16921359	9	104962227	9:104962227:A/G	A	G	0/317159	0/0764775	0/88289	1.37 (1.18-1.60)	3/37E-05	+	3686
rs10900099	10	45221505	10:45221505:A/G	G	A	0/785225	0/189501	0/9779958	2.19 (1.51-3.18)	3/42E-05	+	3686
rs113832687	10	102776360	10:102776360:A/G	T	C	-0/718765	0/161143	0/0350729	0.49 (0.36-0.67)	8/18E-06	+	3686
rs7899485	10	112567284	10:112567284:C/G	C	G	0/214815	0/0504528	0/553327	1.24 (1.12-1.37)	2/07E-05	+	3686
rs56397020	11	84969976	11:84969976:A/G	T	C	-0/420615	0/0899273	0/9393983	0.66 (0.55-0.78)	2/91E-06	+	3686
rs113451492	11	85033761	11:85033761:A/G	A	G	0/445606	0/104778	0/0438846	1.56 (1.27-1.92)	2/11E-05	+	3686
rs75603905	11	90696508	11:90696508:A/C	C	A	0/432864	0/102548	0/9329958	1.54 (1.26-1.88)	2/43E-05	+	3686
rs11613922	12	1485129	12:1485129:C/G	G	C	0/777404	0/175672	0/0132448	2.18 (1.54-3.07)	9/63E-06	+	3686
rs12296782	12	45932569	12:45932569:A/G	T	C	-0/349522	0/0821225	0/9219068	0.71 (0.60-0.83)	2/08E-05	+	3686
rs7979254	12	119285121	12:119285121:A/G	A	G	-0/252418	0/05826	0/30119	0.78 (0.69-0.87)	1/47E-05	+	3686
rs111362698	12	119285230	12:119285230:A/G	A	G	0/270107	0/0661087	0/815212	1.31 (1.15-1.49)	4/39E-05	+	3686
rs956026	12	119288710	12:119288710:A/T	A	T	0/311449	0/065246	0/828175	1.37 (1.20-1.55)	1/81E-06	+	3686
rs6563723	13	40108439	13:40108439:A/C	T	G	-0/266836	0/0609777	0/197398	0.77 (0.68-0.86)	1/21E-05	+	3686
rs72621302	13	40129220	13:40129220:A/C	C	A	0/35079	0/0786756	0/887579	1.42 (1.22-1.66)	8/25E-06	+	3686
rs80207369	13	40136832	13:40136832:A/G	C	T	0/39325	0/0928132	0/9146321	1.48 (1.24-1.78)	2/27E-05	+	3686
rs1230471	13	46777563	13:46777563:A/C	G	T	-0/204329	0/0496975	0/673663	0.82 (0.74-0.90)	3/93E-05	+	3686
rs9531113	13	81390720	13:81390720:A/G	C	T	-0/198633	0/0468428	0/556752	0.82 (0.75-0.90)	2/23E-05	+	3686
rs1342583	13	81441795	13:81441795:A/G	G	A	0/202736	0/0479312	0/387629	1.22 (1.11-1.35)	2/34E-05	+	3686
rs12583441	13	81442734	13:81442734:A/G	G	A	0/223191	0/0494235	0/5003	1.25 (1.13-1.38)	6/31E-06	+	3686
rs11149185	13	81561325	13:81561325:A/G	G	A	0/206319	0/0501592	0/309981	1.23 (1.11-1.36)	3/90E-05	+	3686
rs73516158	13	82064770	13:82064770:A/C	T	G	0/564605	0/136932	0/9603427	1.76 (1.34-2.30)	3/74E-05	+	3686
rs8014942	14	31343135	14:31343135:A/G	T	C	-0/229132	0/0506234	0/456453	0.80 (0.72-0.88)	6/01E-06	+	3686
rs28628097	15	87449577	15:87449577:A/G	C	T	-0/378681	0/0813432	0/9236619	0.68 (0.58-0.80)	3/23E-06	+	3686
rs115596622	16	79400539	16:79400539:C/G	C	G	-0/875453	0/197794	0/0231158	0.42 (0.28-0.61)	9/60E-06	+	3686
rs11859844	16	79415503	16:79415503:A/G	C	T	0/68056	0/167054	0/9713972	1.97 (1.42-2.74)	4/62E-05	+	3686
rs4889142	16	80409383	16:80409383:A/T	T	A	0/866584	0/203483	0/9791938	2.38 (1.60-3.54)	2/06E-05	+	3686
rs78150682	16	89514364	16:89514364:A/C	C	A	-0/383577	0/086985	0/9284341	0.68 (0.57-0.81)	1/04E-05	+	3686
rs61739481	17	11642231	17:11642231:A/G	A	G	0/716952	0/175747	0/9756368	2.05 (1.45-2.89)	4/51E-05	+	3686
rs12103853	17	11655126	17:11655126:A/C	G	T	-0/716527	0/170297	0/0275178	0.49 (0.35-0.68)	2/58E-05	+	3686

rs114469358	17	43962562	17:43962562:A/G	T	C	-0/341719	0/0801036	0/9136149	0.71 (0.61-0.83)	1/99E-05	+	3686
rs61421488	17	43991509	17:43991509:A/C	A	C	-0/355991	0/0808317	0/9124464	0.70 (0.60-0.82)	1/06E-05	+	3686
rs111468222	17	73893155	17:73893155:A/G	A	G	-1/12143	0/264251	0/0187169	0.33 (0.19-0.55)	2/20E-05	+	3686
rs118062399	18	54484203	18:54484203:A/G	C	T	-0/375702	0/0907013	0/9372185	0.69 (0.57-0.82)	3/44E-05	+	3686
rs10415534	19	9224099	19:9224099:A/G	G	A	-0/222	0/0521863	0/481588	0.80 (0.72-0.89)	2/10E-05	+	3686
rs6085661	20	6693128	20:6693128:A/G	C	T	0/227009	0/0512602	0/660354	1.25 (1.13-1.39)	9/49E-06	+	3686
rs199554	20	19934733	20:19934733:A/G	A	G	0/318699	0/0669188	0/799249	1.38 (1.21-1.57)	1/91E-06	+	3686
rs375395	20	19935365	20:19935365:C/G	C	G	0/253395	0/0582062	0/750341	1.29 (1.15-1.44)	1/34E-05	+	3686
rs13045775	20	62486721	20:62486721:A/G	G	A	-0/397293	0/0902494	0/9121969	0.67 (0.56-0.80)	1/07E-05	+	3686
rs117629130	21	23023268	21:23023268:A/G	A	G	-1/0224	0/240313	0/01677	0.36 (0.22-0.58)	2/10E-05	+	3686
rs2839542	21	43948272	21:43948272:A/G	A	G	-0/338362	0/0820707	0/9038978	0.71 (0.61-0.84)	3/74E-05	+	3686
rs4473487	22	19577517	22:19577517:C/G	C	G	1/09447	0/224169	0/978046	2.99 (1.93-4.64)	1/05E-06	+	3686
rs11703151	22	32849712	22:32849712:A/C	C	A	0/369106	0/0899412	0/9116982	1.45 (1.21-1.73)	4/06E-05	+	3686
rs7286822	22	43181587	22:43181587:A/G	C	T	0/420848	0/101188	0/9241442	1.52 (1.25-1.86)	3/20E-05	+	3686
rs4824129	22	50974100	22:50974100:A/G	A	G	-0/274355	0/0587909	0/213856	0.76 (0.68-0.85)	3/06E-06	+	3686
rs5770902	22	51004421	22:51004421:A/G	G	A	-0/245589	0/0602579	0/223967	0.78 (0.70-0.88)	4/59E-05	+	3686

Supplementary table S5. Candidate SNPs with chr, bp, *p*-value, CADD, RDB, nearest gene, genomic risk loci and lead SNPs.
IndSigSNPs = GWAS SNPs with significant p-values from which genes were mapped; CADD = Combined Annotation-Dependent depletion score (the higher the score, the more deleterious the effect of the SNP). RegulomeDB (RDB) score: 1a = eQTL + transcription factor binding + matched transcription factor motif + matched DNase footprint + DNase peak; 1b = eQTL + transcription factor binding + any motif + DNase footprint + DNase peak; 1c = eQTL + transcription factor binding + matched transcription factor motif + DNase peak; 1d = eQTL + transcription factor binding + any motif + DNase peak; 1e = eQTL + transcription factor binding + matched transcription factor motif; 1f = eQTL + transcription factor binding/DNase peak; 2a = transcription factor binding + matched transcription factor motif + matched DNase footprint + DNase peak; 2b = transcription factor binding + any motif + DNase footprint + DNase peak; 2c = transcription factor binding + matched transcription factor motif + DNase peak; 3a = transcription factor binding + any motif + DNase peak; 3b = transcription factor binding + matched transcription factor motif; 4 = transcription factor binding + DNase peak; 5 = transcription factor binding or DNase peak; 6 = other. The 15 core chromatin states (ChrState) as suggested by the Roadmap Epigenomics Project are as follows: 1 = active transcription start site (TSS); 2 = flanking active TSS; 3 = transcription at gene 5′ and 3′ ; 4 = strong transcription; 5 = weak transcription; 6 = genic enhancers; 7 = enhancers; 8 = zinc finger genes and repeats; 9 = heterochromatic; 10 = bivalent/poised TSS; 11 = flanking bivalent/poised TSS/enhancer; 12 = bivalent enhancer; 13 = repressed polycomb; 14 = weak repressed polycomb; 15 = quiescent/low.

uniqID	rsID	non_effect_allele	effect_allele	MAF	gwas*P*	beta	se	r2	IndSigSNP	Genomic Locus	nearestGene	func	CADD	RDB	minChrState	commonChrState
1:4059365:C:T	1:4059365:A/G	C	T	0.03479	3.20426e-05	0.880452	0.211726	1	1:4062730:A/G	1	RP13-614K11.1	intergenic	0.564	7	5	15
1:4059699:A:C	1:4059699:A/C	A	C	0.03877	0.00257511	-0.559238	0.185523	0.844089	1:4062730:A/G	1	RP13-614K11.1	intergenic	0.176	NA	5	15
1:4059700:A:G	1:4059700:A/G	G	A	0.03877	0.00257397	-0.559274	0.185526	0.844089	1:4062730:A/G	1	RP13-614K11.1	intergenic	0.598	5	5	15
1:4059877:A:G	1:4059877:A/G	G	A	0.04473	0.000638786	-0.495347	0.145068	0.836519	1:4062730:A/G	1	RP13-614K11.1	intergenic	0.022	5	5	15
1:4061572:C:T	1:4061572:A/G	C	T	0.03479	4.83058e-05	0.974742	0.239867	1	1:4062730:A/G	1	RP13-614K11.1	intergenic	0.033	5	8	15
1:4062139:C:G	1:4062139:C/G	G	C	0.03579	8.96226e-05	0.836546	0.213563	0.97183	1:4062730:A/G	1	RP13-614K11.1	intergenic	0.434	5	8	15
1:4062278:C:T	1:4062278:A/G	C	T	0.03678	0.0011087	0.643253	0.197232	0.945105	1:4062730:A/G	1	RP13-614K11.1	intergenic	5.715	5	8	15
1:4062279:C:T	1:4062279:A/G	C	T	0.03678	0.00111047	0.64317	0.197234	0.945105	1:4062730:A/G	1	RP13-614K11.1	intergenic	0.897	5	8	15
1:4062730:C:T	1:4062730:A/G	T	C	0.03479	3.89949e-05	0.984889	0.23944	1	1:4062730:A/G	1	RP13-614K11.1	intergenic	1.914	5	8	15
1:4062898:A:G	1:4062898:A/G	A	G	0.03678	0.000911693	0.65058	0.196166	0.945105	1:4062730:A/G	1	RP13-614K11.1	intergenic	0.141	5	8	15
1:4063368:A:G	1:4063368:A/G	A	G	0.03479	0.000108981	0.825911	0.21343	1	1:4062730:A/G	1	RP13-614K11.1	intergenic	2.246	7	8	15
1:4073989:A:G	1:4073989:A/G	G	A	0.02982	0.00695278	0.735825	0.272619	0.857286	1:4062730:A/G	1	RP13-614K11.1	intergenic	1.482	6	5	15
1:4096956:A:G	1:4096956:A/G	G	A	0.03181	0.0344642	0.416311	0.196876	0.799368	1:4062730:A/G	1	RP13-614K11.1	intergenic	1.02	7	5	15
1:4097079:A:AC	rs368823480	A	AC	0.03181	NA	NA	NA	0.799368	1:4062730:A/G	1	RP13-614K11.1	intergenic	0.542	NA	5	15
1:4642126:A:G	1:4642126:A/G	A	G	0.2316	5.25122e-05	0.249365	0.0616605	0.954461	1:4643432:A/C	2	RP1-37J18.2	ncRNA_intronic	1.088	5	9	14
1:4643432:A:C	1:4643432:A/C	C	A	0.2386	7.13916e-06	0.264142	0.0588354	1	1:4643432:A/C	2	RP1-37J18.2	ncRNA_exonic	1.161	NA	9	14
1:4653027:A:G	1:4653027:A/G	A	G	0.2217	0.000157036	0.229852	0.0608127	0.895866	1:4643432:A/C	2	RP1-37J18.2	intergenic	4.499	7	9	15
1:4653093:C:T	1:4653093:A/G	T	C	0.2217	0.000157495	0.229805	0.0608119	0.895866	1:4643432:A/C	2	RP1-37J18.2	intergenic	3.29	7	9	15
1:4653264:A:C	1:4653264:A/C	A	C	0.2286	5.58332e-05	0.236831	0.0587705	0.91952	1:4643432:A/C	2	RP1-37J18.2	intergenic	1.895	5	8	15
1:4654224:A:T	1:4654224:A/T	A	T	0.1998	7.02186e-05	0.240307	0.0604465	0.778973	1:4643432:A/C	2	RP1-37J18.2	intergenic	4.521	5	7	15
1:4654329:A:G	1:4654329:A/G	A	G	0.2087	0.000183417	0.224138	0.0599166	0.742655	1:4643432:A/C	2	RP1-37J18.2	intergenic	0.865	5	7	15
1:47871356:C:CT	rs74307606	CT	C	0.07157	NA	NA	NA	0.617866	1:47924371:A/G	3	RP11-511I2.2	ncRNA_intronic	9.966	NA	5	14
1:47871947:C:G	1:47871947:C/G	G	C	0.06958	0.000158243	-0.434205	0.114937	0.612484	1:47924371:A/G	3	RP11-511I2.2	ncRNA_intronic	7.905	NA	5	14
1:47875627:C:T	1:47875627:A/G	T	C	0.07157	0.000140582	-0.427118	0.112189	0.695868	1:47924371:A/G	3	RP11-511I2.2	intergenic	12.95	5	7	14
1:47879762:C:T	1:47879762:A/G	C	T	0.07256	0.000137	-0.425364	0.111541	0.685097	1:47924371:A/G	3	FOXE3	intergenic	0.843	5	5	14

1:47880903:T:TG	rs145528607	TG	T	0.07157	NA	NA	NA	0.695868	1:47924371:A/G	3	FOXE3	upstream	0.698	NA	5	13
1:47883019:C:T	1:47883019:A/G	C	T	0.07356	0.00035341	0.387284	0.108403	0.684945	1:47935863:A/T	3	FOXE3	UTR3	3.407	4	5	13
1:47884694:C:G	1:47884694:C/G	G	C	0.07157	0.000274657	-0.402039	0.110508	0.695868	1:47924371:A/G	3	FOXE3	downstream	1.71	4	5	13
1:47888256:A:G	1:47888256:A/G	A	G	0.07157	0.000273224	-0.401767	0.110393	0.695868	1:47924371:A/G	3	FOXE3	intergenic	0.286	7	5	14
1:47888999:A:C	1:47888999:A/C	A	C	0.07157	0.000277338	-0.401474	0.110429	0.695868	1:47924371:A/G	3	FOXE3	intergenic	0.43	6	5	14
1:47891487:C:T	1:47891487:A/G	C	T	0.07157	0.000262626	-0.402688	0.110337	0.695868	1:47924371:A/G	3	FOXD2-AS1	intergenic	2.89	5	5	14
1:47895070:A:G	1:47895070:A/G	A	G	0.07157	0.000144559	-0.426937	0.112345	0.695868	1:47924371:A/G	3	FOXD2-AS1	intergenic	4.005	5	4	14
1:47899897:C:T	1:47899897:A/G	C	T	0.07356	3.77043e-05	-0.444659	0.107898	0.760301	1:47924371:A/G	3	FOXD2-AS1	ncRNA_exonic	3.513	4	1	12
1:47900459:A:G	1:47900459:A/G	A	G	0.07455	7.34362e-05	-0.420236	0.10599	0.776448	1:47924371:A/G	3	FOXD2-AS1	upstream	3.436	4	1	12
1:47907604:G:T	1:47907604:A/C	T	G	0.06561	2.65168e-05	-0.454153	0.108093	0.890273	1:47924371:A/G	3	FOXD2	intergenic	3.349	5	1	13
1:47910113:C:T	1:47910113:A/G	C	T	0.06561	5.24107e-05	-0.429258	0.106131	0.890273	1:47924371:A/G	3	FOXD2	intergenic	12.72	4	1	12
1:47912628:C:T	1:47912628:A/G	C	T	0.06561	5.10852e-05	-0.429494	0.106032	0.890273	1:47924371:A/G	3	FOXD2	intergenic	5.526	NA	4	13
1:47913948:C:G	1:47913948:C/G	G	C	0.06561	4.92946e-05	-0.430069	0.105956	0.890273	1:47924371:A/G	3	FOXD2	intergenic	3.673	4	5	13
1:47916917:C:T	1:47916917:A/G	T	C	0.06163	3.27273e-05	-0.440364	0.10602	0.949646	1:47924371:A/G	3	FOXD2	intergenic	4.955	5	5	13
1:47916965:C:T	1:47916965:A/G	T	C	0.06064	3.28331e-05	-0.440054	0.105964	0.966316	1:47924371:A/G	3	FOXD2	intergenic	4.984	5	5	13
1:47919008:A:G	1:47919008:A/G	G	A	0.06163	6.7563e-05	-0.413895	0.103871	0.952399	1:47935863:A/T	3	FOXD2	intergenic	4.813	5	5	14
1:47924371:A:G	1:47924371:A/G	G	A	0.06262	4.40829e-06	-0.463093	0.100867	1	1:47924371:A/G	3	FOXD2	intergenic	8.289	5	5	14
1:47926022:A:G	1:47926022:A/G	G	A	0.06262	7.76284e-06	-0.441065	0.0986364	1	1:47924371:A/G	3	FOXD2	intergenic	0.231	5	5	13
1:47928997:C:G	1:47928997:C/G	G	C	0.06461	1.00909e-05	-0.433152	0.0981041	0.96833	1:47924371:A/G	3	RP4-666O22.3	intergenic	1.487	7	5	14
1:47933038:C:T	1:47933038:A/G	T	C	0.06362	9.09037e-06	-0.435558	0.0981484	0.984261	1:47935863:A/T	3	RP4-666O22.3	intergenic	7.238	6	5	14
1:47935863:A:T	1:47935863:A/T	T	A	0.06461	1.02854e-05	-0.425801	0.0965298	1	1:47935863:A/T	3	RP4-666O22.3	intergenic	1.284	4	5	14
1:47942422:C:T	1:47942422:A/G	C	T	0.08151	3.01359e-06	0.452972	0.0969975	1	1:47942422:A/G	3	RP4-666O22.3	intergenic	4.766	NA	5	14
1:47946305:C:G	1:47946305:C/G	G	C	0.08946	5.16281e-08	0.541858	0.0995039	1	1:47952962:A/G	3	RP4-666O22.3	intergenic	4.999	7	5	14
1:47951471:C:T	1:47951471:A/G	C	T	0.08946	3.72781e-08	0.543313	0.0987255	1	1:47952962:A/G	3	RP4-666O22.3	intergenic	3.026	NA	5	14
1:47952663:G:T	1:47952663:A/C	G	T	0.08946	3.55556e-08	0.542326	0.0983967	1	1:47952962:A/G	3	RP4-666O22.3	intergenic	0.857	NA	5	14
1:47952962:C:T	1:47952962:A/G	T	C	0.08946	2.26271e-08	0.548826	0.0981689	1	1:47952962:A/G	3	RP4-666O22.3	intergenic	0.702	5	5	14
1:47954232:A:C	1:47954232:A/C	A	C	0.08946	3.88578e-08	0.54596	0.0993386	1	1:47952962:A/G	3	RP4-666O22.3	intergenic	0.317	5	13	14
1:47957160:A:T	1:47957160:A/T	T	A	0.09046	7.45402e-08	0.531217	0.0987416	0.988732	1:47952962:A/G	3	RPL21P24	intergenic	15.43	3a	12	14
1:47959211:G:T	1:47959211:A/C	T	G	0.09742	3.00203e-06	0.437203	0.0936057	0.915512	1:47952962:A/G	3	RPL21P24	intergenic	9.477	NA	13	14
1:47961734:A:T	1:47961734:A/T	A	T	0.09642	9.21826e-07	0.45914	0.0935561	0.904167	1:47952962:A/G	3	RPL21P24	intergenic	1.635	7	5	14
1:47961744:C:T	1:47961744:A/G	C	T	0.09046	4.93439e-08	0.538331	0.0987097	0.966317	1:47952962:A/G	3	RPL21P24	intergenic	0.784	7	5	14
1:47965269:A:T	1:47965269:A/T	T	A	0.09145	8.49271e-08	0.529879	0.0989255	0.977677	1:47952962:A/G	3	RPL21P24	intergenic	8.987	6	5	14
1:47965813:C:T	1:47965813:A/G	T	C	0.09742	1.95355e-06	0.44629	0.0937945	0.915512	1:47952962:A/G	3	RPL21P24	intergenic	0.414	6	5	14
1:47965892:C:T	1:47965892:A/G	T	C	0.09145	8.37154e-08	0.530056	0.0989108	0.977677	1:47952962:A/G	3	RPL21P24	intergenic	1.791	7	5	14
1:47966000:C:T	1:47966000:A/G	C	T	0.09742	1.95505e-06	0.446269	0.0937932	0.915512	1:47952962:A/G	3	RPL21P24	intergenic	7.272	7	5	14
1:47966058:A:G	1:47966058:A/G	A	G	0.09742	1.95341e-06	0.446284	0.093793	0.915512	1:47952962:A/G	3	RPL21P24	intergenic	3.305	5	5	14
1:47966292:C:T	1:47966292:A/G	C	T	0.09742	1.9526e-06	0.446291	0.0937926	0.915512	1:47952962:A/G	3	AL356458.1	intergenic	1.106	7	5	14
1:47967172:G:T	1:47967172:A/C	G	T	0.09742	1.95113e-06	0.446302	0.093792	0.915512	1:47952962:A/G	3	AL356458.1	intergenic	1.176	6	5	14
1:47967242:A:G	1:47967242:A/G	A	G	0.09742	1.95112e-06	0.446303	0.093792	0.915512	1:47952962:A/G	3	AL356458.1	upstream	12.97	5	5	14
1:47967393:A:C	1:47967393:A/C	A	C	0.09742	1.95146e-06	0.446299	0.0937921	0.915512	1:47952962:A/G	3	AL356458.1	upstream	7.443	5	5	14
1:47967825:A:C	1:47967825:A/C	A	C	0.09742	1.95103e-06	0.446303	0.0937919	0.915512	1:47952962:A/G	3	AL356458.1	upstream	1.743	5	5	14
1:47968050:A:C	1:47968050:A/C	C	A	0.09742	1.95103e-06	0.446301	0.0937915	0.915512	1:47952962:A/G	3	AL356458.1	upstream	5.101	5	5	14
1:47968317:A:G	1:47968317:A/G	A	G	0.09742	1.94227e-06	0.446337	0.0937813	0.915512	1:47952962:A/G	3	AL356458.1	downstream	3.064	7	5	14

1:47968967:A:C	1:47968967:A/C	A	C	0.09742	1.9497e-06	0.446309	0.0937906	0.915512	1:47952962:A/G	3	AL356458.1	downstream	1.597	7	7	14
1:47969496:A:G	1:47969496:A/G	A	G	0.09742	1.71736e-06	0.447734	0.0935876	0.915512	1:47952962:A/G	3	AL356458.1	intergenic	3.297	5	5	14
1:47969853:A:T	1:47969853:A/T	T	A	0.09742	1.95027e-06	0.4463	0.0937898	0.915512	1:47952962:A/G	3	AL356458.1	intergenic	0.258	5	13	14
1:47969863:G:T	1:47969863:A/C	G	T	0.09742	1.95069e-06	0.446295	0.0937897	0.915512	1:47952962:A/G	3	AL356458.1	intergenic	1.701	5	13	14
1:47970081:A:G	1:47970081:A/G	A	G	0.09742	1.9506e-06	0.446296	0.0937898	0.915512	1:47952962:A/G	3	AL356458.1	intergenic	1.946	7	13	14
1:47970214:A:T	1:47970214:A/T	A	T	0.09742	1.71821e-06	0.447723	0.0935873	0.915512	1:47952962:A/G	3	AL356458.1	intergenic	2.525	6	13	14
1:47970621:G:T	1:47970621:A/C	G	T	0.09742	1.94936e-06	0.446307	0.0937895	0.915512	1:47952962:A/G	3	AL356458.1	intergenic	2.499	7	13	14
1:47970868:A:G	1:47970868:A/G	G	A	0.09742	1.94868e-06	0.446311	0.0937891	0.915512	1:47952962:A/G	3	AL356458.1	intergenic	1.12	5	13	14
1:47970912:A:C	1:47970912:A/C	C	A	0.09742	1.94896e-06	0.446308	0.0937891	0.915512	1:47952962:A/G	3	AL356458.1	intergenic	2.924	7	13	14
1:47971258:C:T	1:47971258:A/G	C	T	0.09742	1.94713e-06	0.446322	0.0937883	0.915512	1:47952962:A/G	3	AL356458.1	intergenic	4.058	7	9	14
1:47971283:C:T	1:47971283:A/G	C	T	0.09145	8.34002e-08	0.5301	0.0989066	0.977677	1:47952962:A/G	3	AL356458.1	intergenic	3.9	7	9	14
1:47971646:A:G	1:47971646:A/G	G	A	0.09145	1.14928e-07	0.525354	0.0990975	0.977677	1:47952962:A/G	3	AL356458.1	intergenic	3.632	5	9	13
1:47972149:A:G	1:47972149:A/G	A	G	0.09742	1.94721e-06	0.446319	0.0937876	0.915512	1:47952962:A/G	3	AL356458.1	intergenic	6.693	5	9	13
1:47972419:A:G	1:47972419:A/G	A	G	0.09742	1.93755e-06	0.44639	0.0937828	0.915512	1:47952962:A/G	3	AL356458.1	intergenic	11.55	5	7	13
1:47973296:A:T	1:47973296:A/T	A	T	0.09145	7.78939e-08	0.531163	0.0988776	0.977677	1:47952962:A/G	3	AL356458.1	intergenic	1.405	5	2	13
1:47974992:C:T	1:47974992:A/G	C	T	0.09145	8.86241e-08	0.529552	0.0990069	0.977677	1:47952962:A/G	3	AL356458.1	intergenic	9.525	NA	1	13
1:47975599:C:CAG	rs34620652	CAG	C	0.09145	NA	NA	NA	0.977677	1:47952962:A/G	3	AL356458.1	intergenic	11.4	NA	7	13
1:47976330:C:T	1:47976330:A/G	C	T	0.09145	9.09793e-08	0.528335	0.0988673	0.977677	1:47952962:A/G	3	AL356458.1	intergenic	16.49	4	5	13
1:47976383:A:C	1:47976383:A/C	A	C	0.09145	5.86938e-08	0.535666	0.0987819	0.977677	1:47952962:A/G	3	AL356458.1	intergenic	12.04	4	5	13
1:47976869:C:T	1:47976869:A/G	T	C	0.09742	1.40296e-06	0.451509	0.0935852	0.915512	1:47952962:A/G	3	AL356458.1	intergenic	6.689	NA	5	14
1:47980996:A:G	1:47980996:A/G	G	A	0.09145	8.05618e-08	0.529489	0.0986773	0.977677	1:47952962:A/G	3	AL356458.1	intergenic	19.4	NA	5	14
1:47983230:A:G	1:47983230:A/G	G	A	0.09245	7.04398e-08	0.531019	0.0985185	0.96683	1:47952962:A/G	3	AL356458.1	intergenic	0.043	NA	5	14
1:47984438:C:T	1:47984438:A/G	T	C	0.1034	2.44476e-07	0.507347	0.0982871	0.859696	1:47952962:A/G	3	AL356458.1	intergenic	10.46	NA	5	14
1:47997658:C:G	1:47997658:C/G	C	G	0.08748	1.23557e-06	0.497633	0.102607	0.822	1:47952962:A/G	3	ATP6V0E1P4	intergenic	1.152	7	5	14
1:155033308:A:G	1:155033308:A/G	A	G	0.1819	6.38828e-06	0.268846	0.0595697	1	1:155033308:A/G	4	ADAM15	exonic	19.89	5	3	5
1:155065627:A:G	1:155065627:A/G	A	G	0.1571	1.86488e-05	-0.258539	0.0604003	0.777928	1:155105882:A/C	4	EFNA3	intergenic	2.333	NA	1	5
1:155071167:A:T	1:155071167:A/T	T	A	0.1491	3.42212e-06	-0.284159	0.0611927	0.815265	1:155105882:A/C	4	EFNA3	intergenic	3.083	6	5	15
1:155087083:C:T	1:155087083:A/G	T	C	0.1481	1.60576e-06	-0.294284	0.0613394	0.807895	1:155105882:A/C	4	Y_RNA	intergenic	2.738	6	5	14
1:155095750:C:T	1:155095750:A/G	C	T	0.3579	2.10393e-08	-0.271535	0.0484605	0.622216	1:155178782:A/T	4	Y_RNA	intergenic	7.894	7	5	14
1:155105882:G:T	1:155105882:A/C	G	T	0.1461	1.39669e-06	-0.293332	0.0607877	1	1:155105882:A/C	4	EFNA1	intronic	8.866	7	3	5
1:155123837:C:G	1:155123837:C/G	C	G	0.3966	2.15423e-09	0.280231	0.0468166	0.877032	1:155178782:A/T	4	HMGN2P18	intergenic	1.722	7	7	15
1:155130798:C:T	1:155130798:A/G	T	C	0.1461	2.92046e-06	-0.282999	0.0605169	0.954618	1:155105882:A/C	4	HMGN2P18	intergenic	1.887	6	14	15
1:155143768:G:T	1:155143768:A/C	T	G	0.3658	0.000212244	-0.181386	0.0489706	0.646831	1:155155731:A/G	4	KRTCAP2:RP11-201K10.3	intronic	1.094	6	4	5
1:155144300:A:G	1:155144300:A/G	A	G	0.3668	0.00020537	-0.181582	0.0489134	0.650484	1:155155731:A/G	4	KRTCAP2:RP11-201K10.3	intronic	2.367	5	4	5
1:155148781:A:G	1:155148781:A/G	A	G	0.3529	0.00010479	-0.1896	0.0488759	0.709193	1:155155731:A/G	4	RP11-201K10.3:TRIM46	intronic	12.24	3a	1	5
1:155149718:A:G	1:155149718:A/G	A	G	0.3579	6.06766e-06	-0.223437	0.049389	0.747824	1:155155731:A/G	4	RP11-201K10.3:TRIM46	exonic	10.3	NA	2	7

1:155151493:C:T	1:155151493:A/G	T	C	0.4115	9.85863e-10	-0.288589	0.0472192	0.967358	1:155155731:A/G	4	RP11-201K10.3:TRIM46	intronic	3.037	4	2	4
1:155151754:A:T	1:155151754:A/T	T	A	0.4145	1.39872e-10	-0.302859	0.0472035	0.979536	1:155155731:A/G	4	RP11-201K10.3:TRIM46	intronic	4.637	3a	2	4
1:155155389:G:GTT	rs541049493	G	GTT	0.4056	NA	NA	NA	0.928243	1:155155731:A/G	4	RP11-201K10.3:TRIM46	intronic	2.738	NA	4	5
1:155155608:A:G	1:155155608:A/G	G	A	0.4354	2.00051e-09	-0.284513	0.0474365	0.9154	1:155155731:A/G	4	RP11-201K10.3:TRIM46	intronic	8.056	7	4	5
1:155155731:A:G	1:155155731:A/G	A	G	0.4135	4.50824e-11	-0.311717	0.047328	1	1:155155731:A/G	4	RP11-201K10.3:TRIM46	intronic	3.306	1f	4	5
1:155157635:A:G	1:155157635:A/G	A	G	0.4145	1.10313e-10	-0.30504	0.0472776	0.995932	1:155155731:A/G	4	RP11-201K10.3	intronic	7.628	4	2	5
1:155157715:A:G	1:155157715:A/G	G	A	0.4145	9.71437e-11	-0.305305	0.0471781	0.995932	1:155155731:A/G	4	RP11-201K10.3	intronic	6.073	NA	2	5
1:155161168:C:G	1:155161168:C/G	G	C	0.4523	2.84667e-10	-0.290931	0.046129	0.767767	1:155178782:A/T	4	MUC1	intronic	6.493	5	1	5
1:155162067:C:T	1:155162067:A/G	T	C	0.4523	2.20133e-10	-0.292228	0.0460449	0.767767	1:155178782:A/T	4	MUC1	exonic	5.819	NA	1	5
1:155164480:A:C	1:155164480:A/C	A	C	0.4523	4.41466e-10	0.287216	0.0460384	0.767767	1:155178782:A/T	4	MIR92B	upstream:downstream	8.379	4	1	1
1:155168849:C:T	1:155168849:A/G	T	C	0.4523	7.71825e-10	-0.283175	0.04604	0.767767	1:155178782:A/T	4	THBS3:RP11-263K19.4	ncRNA_intronic	0.578	6	4	5
1:155168930:A:G	1:155168930:A/G	A	G	0.4155	3.96412e-10	-0.295826	0.0472909	0.991925	1:155155731:A/G	4	THBS3:RP11-263K19.4	ncRNA_intronic	0.551	7	4	5
1:155169200:C:CA	rs11355526	C	CA	0.4364	NA	NA	NA	0.712128	1:155178782:A/T	4	THBS3:RP11-263K19.4	ncRNA_intronic	9.874	NA	4	5
1:155169355:G:T	1:155169355:A/C	T	G	0.4155	2.90067e-10	-0.298073	0.0472832	0.991925	1:155155731:A/G	4	THBS3:RP11-263K19.4	ncRNA_intronic	4.189	NA	4	5
1:155172379:A:G	1:155172379:A/G	A	G	0.4543	7.26608e-10	-0.282994	0.0459392	0.754098	1:155178782:A/T	4	THBS3:RP11-263K19.4	ncRNA_intronic	0.925	NA	3	5
1:155174106:C:T	1:155174106:A/G	T	C	0.4175	3.79879e-10	-0.295659	0.0472143	0.984091	1:155155731:A/G	4	THBS3:RP11-263K19.4	ncRNA_intronic	3.007	5	4	5
1:155175390:A:G	1:155175390:A/G	G	A	0.4553	1.02561e-09	-0.280503	0.0459437	0.759193	1:155178782:A/T	4	THBS3	intronic	0.31	NA	4	5
1:155175892:A:G	1:155175892:A/G	G	A	0.4553	1.03146e-09	-0.280465	0.0459443	0.759193	1:155178782:A/T	4	THBS3	intronic	3.532	1f	3	5
1:155178782:A:T	1:155178782:A/T	T	A	0.3946	1.14946e-12	-0.331028	0.0465495	1	1:155178782:A/T	4	THBS3:MTX1	exonic	9.315	4	1	1
1:155181843:G:T	1:155181843:A/C	T	G	0.4761	6.62851e-10	-0.28555	0.0462451	0.703249	1:155178782:A/T	4	MTX1:RP11-263K19.6	ncRNA_intronic	2.551	NA	1	5
1:155182164:C:T	1:155182164:A/G	T	C	0.4751	3.70291e-10	-0.289306	0.0461702	0.704905	1:155178782:A/T	4	MTX1:RP11-263K19.6	ncRNA_intronic	5.776	NA	1	5
1:155184057:A:G	1:155184057:A/G	A	G	0.3012	3.84555e-09	-0.318546	0.0540759	0.619501	1:155155731:A/G	4	GBAP1	ncRNA_exonic	6.242	5	4	5
1:155184975:A:G	1:155184975:A/G	G	A	0.3956	2.3149e-12	-0.326135	0.0464973	0.979151	1:155178782:A/T	4	GBAP1	ncRNA_exonic	1.623	5	2	5
1:155186729:C:T	1:155186729:A/G	C	T	0.4771	4.22213e-10	-0.287581	0.0460454	0.70162	1:155178782:A/T	4	GBAP1	ncRNA_exonic	11.61	7	4	5

1:155186742:A:G	1:155186742:A/G	A	G	0.3917	1.02237e-10	-0.30787	0.0476312	0.864371	1:155155731:A/G	4	GBAP1	ncRNA_exonic	9.692	NA	4	5
1:155190254:A:T	1:155190254:A/T	A	T	0.4771	4.21313e-10	-0.287634	0.0460514	0.70162	1:155178782:A/T	4	GBAP1	ncRNA_intronic	0.547	5	5	5
1:155193532:C:G	1:155193532:C/G	G	C	0.4771	4.21611e-10	-0.287594	0.0460459	0.70162	1:155178782:A/T	4	GBAP1	ncRNA_intronic	7.223	7	2	5
1:155194140:C:CAT	rs565825708	C	CAT	0.4771	NA	NA	NA	0.70162	1:155178782:A/T	4	GBAP1	ncRNA_intronic	0.409	NA	2	5
1:155194980:C:T	1:155194980:A/G	C	T	0.4771	3.69032e-10	-0.288455	0.0460306	0.70162	1:155178782:A/T	4	GBAP1	ncRNA_intronic	5.604	NA	2	5
1:155195071:A:ATTAT	rs374942809	ATTAT	A	0.4264	NA	NA	NA	0.873465	1:155178782:A/T	4	GBAP1	ncRNA_intronic	1.05	NA	1	5
1:155195215:C:G	1:155195215:C/G	G	C	0.4771	4.25825e-10	-0.287544	0.0460495	0.70162	1:155178782:A/T	4	GBAP1	ncRNA_intronic	2.647	7	1	5
1:155196717:A:G	1:155196717:A/G	G	A	0.4771	1.80128e-09	-0.278327	0.0462739	0.70162	1:155178782:A/T	4	GBAP1	ncRNA_intronic	8.192	NA	1	1
1:155197268:C:T	1:155197268:A/G	C	T	0.4771	4.41882e-10	-0.289407	0.0463906	0.694763	1:155178782:A/T	4	GBAP1	upstream	6.384	NA	1	1
1:155197462:C:T	1:155197462:A/G	T	C	0.4791	2.14771e-09	-0.27815	0.0464649	0.697161	1:155178782:A/T	4	GBAP1	upstream	6.669	1f	1	1
1:155197602:C:G	1:155197602:C/G	C	G	0.3728	6.80154e-09	-0.287722	0.0496438	0.662307	1:155155731:A/G	4	GBAP1	upstream	6.42	4	1	2
1:155358612:A:G	1:155358612:A/G	A	G	0.1342	0.00517074	0.258499	0.0924473	0.826854	1:155616555:A/T	4	ASH1L	intronic	9.769	7	4	5
1:155462867:C:T	1:155462867:A/G	T	C	0.1352	0.000614237	0.342069	0.0998669	0.819971	1:155616555:A/T	4	ASH1L	intronic	8.011	5	4	5
1:155521279:A:G	1:155521279:A/G	A	G	0.1252	0.00145998	0.321711	0.101087	0.73552	1:155616555:A/T	4	ASH1L	intronic	1.841	7	4	5
1:155527235:G:T	1:155527235:A/C	G	T	0.4742	4.2904e-09	0.299328	0.0509706	1	1:155527235:A/C	4	ASH1L	intronic	3.307	6	4	5
1:155616555:A:T	1:155616555:A/T	T	A	0.1531	1.61318e-08	0.479895	0.0849516	1	1:155616555:A/T	4	MSTO1:MSTO2P:RP11-29H23.5	ncRNA_intronic	6.331	5	2	15
1:155629116:A:G	1:155629116:A/G	A	G	0.1083	6.62724e-06	0.474491	0.105318	0.665989	1:155616555:A/T	4	MSTO1:MSTO2P:RP11-29H23.5	ncRNA_intronic	3.924	7	4	4
1:155722506:C:G	1:155722506:C/G	G	C	0.3728	2.97684e-08	0.296366	0.0534689	0.607624	1:155178782:A/T	4	GON4L	intronic	2.51	6	4	4
1:155811448:A:G	1:155811448:A/G	A	G	0.1521	0.000181911	0.33644	0.0898879	0.770235	1:155616555:A/T	4	GON4L	intronic	5.214	5	4	5
1:155824181:C:T	1:155824181:A/G	C	T	0.1521	0.000277298	0.327982	0.0902131	0.770235	1:155616555:A/T	4	GON4L	intronic	3.88	6	4	4
1:155846080:A:G	1:155846080:A/G	G	A	0.166	9.59949e-07	0.405161	0.082692	0.849639	1:155616555:A/T	4	SYT11	intronic	4.148	7	4	5
1:155872490:G:GA	rs140077956	GA	G	0.1431	NA	NA	NA	0.718427	1:155616555:A/T	4	RIT1	intronic	2.451	NA	4	4
1:155876613:A:G	1:155876613:A/G	A	G	0.4911	3.76591e-09	0.297074	0.0504015	1	1:155876613:A/G	4	RIT1	intronic	1.833	6	4	4
1:156022802:C:T	1:156022802:A/G	T	C	0.4264	0.000130157	0.187729	0.0490642	0.786528	1:156032887:A/G	4	UBQLN4	intronic	15.36	2b	1	1
1:156024373:A:G	1:156024373:A/G	G	A	0.4254	4.47117e-05	0.197319	0.0483426	0.790579	1:156032887:A/G	4	LAMTOR2	upstream	16.88	NA	1	1
1:156028534:C:CTG	rs150713468	CTG	C	0.4394	NA	NA	NA	0.949025	1:156032887:A/G	4	LAMTOR2	downstream	6.586	NA	4	4
1:156032887:A:G	1:156032887:A/G	A	G	0.4443	7.44244e-06	0.223447	0.0498694	1	1:156032887:A/G	4	RAB25	intronic	1.733	6	1	5
1:156042915:A:G	1:156042915:A/G	A	G	0.4453	2.50515e-05	0.208177	0.0493974	0.972469	1:156032887:A/G	4	MEX3A	UTR3	10.46	5	4	5
1:231188455:C:T	1:231188455:A/G	T	C	0.0169	0.00874111	0.372111	0.141918	0.668807	1:231259851:A/G	5	FAM89A	intergenic	1.794	6	5	14
1:231220183:C:T	1:231220183:A/G	T	C	0.02187	0.0110759	-0.326228	0.128421	0.874511	1:231259851:A/G	5	RP5-1097F14.1	intergenic	1.225	5	13	15
1:231246007:C:G	1:231246007:C/G	G	C	0.02485	9.91074e-05	-0.417713	0.107305	1	1:231259851:A/G	5	RP5-1097F14.1	intergenic	0.86	7	5	15
1:231248943:C:T	1:231248943:A/G	T	C	0.02485	6.35057e-05	-0.428384	0.107112	1	1:231259851:A/G	5	RP5-1097F14.1	intergenic	4.605	7	9	15
1:231250309:A:G	1:231250309:A/G	A	G	0.02485	5.65348e-05	-0.431193	0.10708	1	1:231259851:A/G	5	RP5-1097F14.1	intergenic	0.008	6	13	15
1:231251866:C:T	1:231251866:A/G	C	T	0.02485	5.14039e-05	-0.433575	0.107078	1	1:231259851:A/G	5	RP5-1097F14.1	intergenic	3.599	6	14	15
1:231255858:C:T	1:231255858:A/G	T	C	0.02485	4.38062e-05	-0.437126	0.10697	1	1:231259851:A/G	5	RP5-1097F14.1	intergenic	0.029	6	5	15
1:231257854:T:TAA	rs376665112	TAA	T	0.02485	NA	NA	NA	0.838719	1:231259851:A/G	5	RP5-1097F14.1	intergenic	0.301	NA	5	15
1:231257885:A:T	1:231257885:A/T	T	A	0.02485	4.34693e-05	-0.437923	0.107118	1	1:231259851:A/G	5	RP5-1097F14.1	intergenic	0.023	6	5	15
1:231258703:C:T	1:231258703:A/G	T	C	0.02485	4.34349e-05	-0.438126	0.107163	1	1:231259851:A/G	5	RP5-1097F14.1	intergenic	2.397	5	5	15
1:231259851:A:G	1:231259851:A/G	G	A	0.02485	4.31459e-05	-0.438652	0.107251	1	1:231259851:A/G	5	RP5-1097F14.1	intergenic	0.609	7	5	15
1:231259997:G:T	1:231259997:A/C	T	G	0.02584	0.000134077	-0.404814	0.106004	0.959527	1:231259851:A/G	5	RP5-1097F14.1	intergenic	1.925	NA	5	15
2:29979985:C:T	2:29979985:A/G	C	T	0.09344	6.20814e-05	-0.356733	0.0890769	0.988541	2:29983090:A/G	6	ALK	intronic	1.958	7	5	15

2:29980221:C:T	2:29980221:A/G	T	C	0.09443	7.7616e-05	-0.349662	0.0884852	0.977159	2:29983090:A/G	6	ALK	intronic	0.768	NA	5	15
2:29980408:G:T	2:29980408:A/C	G	T	0.1233	0.0143454	-0.170667	0.0697019	0.75498	2:29983090:A/G	6	ALK	intronic	2.204	NA	5	15
2:29981286:A:G	2:29981286:A/G	A	G	0.1233	0.0137693	-0.17127	0.0695309	0.75498	2:29983090:A/G	6	ALK	intronic	4.887	7	5	15
2:29981602:C:T	2:29981602:A/G	T	C	0.1233	0.0122395	-0.17493	0.069828	0.75498	2:29983090:A/G	6	ALK	intronic	0.462	6	5	15
2:29981696:C:G	2:29981696:C/G	C	G	0.1064	0.000909369	-0.262199	0.0790435	0.855779	2:29983090:A/G	6	ALK	intronic	3.296	7	5	15
2:29983090:C:T	2:29983090:A/G	C	T	0.09443	4.88982e-05	-0.363802	0.0895886	1	2:29983090:A/G	6	ALK	intronic	1.439	5	5	15
2:29984708:C:T	2:29984708:A/G	T	C	0.09443	6.10272e-05	-0.350866	0.0875238	1	2:29983090:A/G	6	ALK	intronic	10.85	7	5	15
2:29985721:A:G	2:29985721:A/G	A	G	0.1233	0.0177938	-0.164873	0.06957	0.75498	2:29983090:A/G	6	ALK	intronic	0.239	NA	5	15
2:29985820:A:G	2:29985820:A/G	G	A	0.1123	0.000773161	-0.257358	0.0765439	0.826433	2:29983090:A/G	6	ALK	intronic	2.121	NA	5	15
2:29986208:C:T	2:29986208:A/G	C	T	0.1113	0.00545262	-0.209954	0.07555	0.843727	2:29983090:A/G	6	ALK	intronic	3.131	7	5	15
2:29986534:C:T	2:29986534:A/G	T	C	0.1243	0.0144601	-0.168864	0.0690474	0.752444	2:29983090:A/G	6	ALK	intronic	0.106	7	5	15
2:29986555:C:T	2:29986555:A/G	T	C	0.1044	0.00122936	-0.253	0.0782802	0.813036	2:29983090:A/G	6	ALK	intronic	3.566	NA	5	15
2:29986574:A:G	2:29986574:A/G	A	G	0.1243	0.0138872	-0.171518	0.0697184	0.74826	2:29983090:A/G	6	ALK	intronic	1.101	NA	5	15
2:29986601:A:T	2:29986601:A/T	A	T	0.1233	0.0142993	-0.170966	0.0697919	0.75498	2:29983090:A/G	6	ALK	intronic	1.569	5	5	15
2:29987735:C:G	2:29987735:C/G	C	G	0.1233	0.0151696	-0.170435	0.0701859	0.75498	2:29983090:A/G	6	ALK	intronic	0.196	6	5	15
2:29988753:A:G	2:29988753:A/G	G	A	0.08648	4.94672e-05	-0.390949	0.0963373	0.887392	2:29983090:A/G	6	ALK	intronic	10.81	NA	5	15
2:38717105:G:T	2:38717105:A/C	G	T	0.03579	0.000175969	-0.444071	0.11838	0.793659	2:38721171:C/G	7	AC016995.3	ncRNA_intronic	4.061	5	5	15
2:38717638:C:G	2:38717638:C/G	G	C	0.03082	4.04701e-05	-0.457446	0.111442	0.943726	2:38721171:C/G	7	AC016995.3	ncRNA_intronic	1.772	6	5	15
2:38718614:C:T	2:38718614:A/G	T	C	0.02883	2.87312e-05	-0.466954	0.111623	1	2:38721171:C/G	7	AC016995.3	ncRNA_intronic	0.186	7	5	15
2:38718818:C:T	2:38718818:A/G	C	T	0.02883	3.61943e-05	-0.458338	0.110964	1	2:38721171:C/G	7	AC016995.3	ncRNA_intronic	5.405	5	5	15
2:38719394:G:T	2:38719394:A/C	G	T	0.02883	2.47308e-05	-0.471185	0.111728	1	2:38721171:C/G	7	AC016995.3	ncRNA_intronic	7.137	4	5	15
2:38719742:C:CT	rs200800703	CT	C	0.03082	NA	NA	NA	0.931537	2:38721171:C/G	7	AC016995.3	ncRNA_intronic	0.919	NA	5	15
2:38719750:C:CT	rs542913914	CT	C	0.02883	NA	NA	NA	1	2:38721171:C/G	7	AC016995.3	ncRNA_intronic	1.005	NA	5	15
2:38719824:C:T	2:38719824:A/G	T	C	0.02982	1.45681e-05	-0.484714	0.111811	0.895179	2:38721171:C/G	7	AC016995.3	ncRNA_intronic	1.736	6	5	15
2:38719839:C:T	2:38719839:A/G	T	C	0.02783	1.58591e-05	-0.483207	0.111947	0.963485	2:38721171:C/G	7	AC016995.3	ncRNA_intronic	1.84	7	5	15
2:38720951:A:G	2:38720951:A/G	A	G	0.02883	8.22062e-06	-0.53469	0.119903	1	2:38721171:C/G	7	AC016995.3	ncRNA_intronic	0.628	6	5	15
2:38721171:C:G	2:38721171:C/G	G	C	0.02883	6.23664e-06	-0.536591	0.118762	1	2:38721171:C/G	7	AC016995.3	ncRNA_intronic	2.812	7	2	15
2:38721279:G:T	2:38721279:A/C	G	T	0.02883	2.96746e-05	-0.466238	0.111649	1	2:38721171:C/G	7	AC016995.3	ncRNA_intronic	0.159	6	2	15
2:38726736:A:C	2:38726736:A/C	A	C	0.02883	6.17736e-05	-0.452936	0.113066	0.928154	2:38721171:C/G	7	AC016995.3	ncRNA_intronic	3.368	3a	2	15
2:38728628:C:G	2:38728628:C/G	C	G	0.02982	3.29682e-05	-0.46438	0.111847	0.964627	2:38721171:C/G	7	AC016995.3	ncRNA_intronic	1.864	6	5	15
2:38730814:C:T	2:38730814:A/G	T	C	0.02982	3.65558e-05	-0.467866	0.113333	0.964627	2:38721171:C/G	7	AC016995.3	ncRNA_intronic	1.881	6	5	15
2:74406874:G:T	2:74406874:A/C	G	T	0.0666	0.000697852	0.274484	0.0809587	0.662056	2:74448672:A/C	8	MOB1A	upstream	1.613	6	5	15
2:74406986:A:G	2:74406986:A/G	A	G	0.0666	0.000717762	0.275241	0.0813666	0.662056	2:74448672:A/C	8	MOB1A	upstream	1.944	7	5	15
2:74412382:A:T	2:74412382:A/T	A	T	0.06561	0.000429676	0.289032	0.0820848	0.668816	2:74448672:A/C	8	CATX-2	intergenic	1.168	5	5	15
2:74412502:C:T	2:74412502:A/G	T	C	0.06759	0.000218068	0.297885	0.0805721	0.677185	2:74448672:A/C	8	CATX-2	intergenic	0.885	6	2	15
2:74420561:C:CATTATT	rs151200817	CATTATT	C	0.06759	NA	NA	NA	0.677185	2:74448672:A/C	8	CATX-2	intergenic	0.908	NA	5	15
2:74425427:C:T	2:74425427:A/G	T	C	0.06958	0.000703751	0.280513	0.0827937	0.686328	2:74448672:A/C	8	MTHFD2	upstream	11.1	2b	1	2
2:74425447:G:T	2:74425447:A/C	T	G	0.07356	0.000605653	0.279117	0.0813972	0.657209	2:74448672:A/C	8	MTHFD2	upstream	7.474	4	1	2
2:74425932:A:G	2:74425932:A/G	A	G	0.06859	0.000466383	0.291496	0.0833003	0.692396	2:74448672:A/C	8	MTHFD2	intronic	6.255	NA	1	1
2:74426479:A:C	2:74426479:A/C	C	A	0.06859	0.000431812	0.293336	0.0833377	0.692396	2:74448672:A/C	8	MTHFD2	intronic	7.449	4	1	1
2:74430386:A:G	2:74430386:A/G	A	G	0.06859	0.000507383	0.29123	0.0837628	0.692396	2:74448672:A/C	8	MTHFD2	intronic	3.409	6	3	5
2:74432455:A:G	2:74432455:A/G	A	G	0.06759	0.00043814	0.297517	0.084618	0.70396	2:74448672:A/C	8	MTHFD2	intronic	2.118	5	2	5
2:74435332:A:G	2:74435332:A/G	G	A	0.07455	3.8939e-05	0.338834	0.0823675	0.817216	2:74448672:A/C	8	MTHFD2	intronic	2.722	7	4	4

2:74445035:A:G	2:74445035:A/G	G	A	0.07256	3.15955e-05	0.341156	0.0819759	0.841496	2:74448672:A/C	8	RP11-287D1.3:SLC4A5	UTR3	2.403	5	4	5
2:74448672:A:C	2:74448672:A/C	A	C	0.06561	6.32622e-06	0.367675	0.0814308	1	2:74448672:A/C	8	RP11-287D1.3:SLC4A5	intronic	1.176	NA	4	5
2:74448913:G:T	2:74448913:A/C	T	G	0.06561	2.68071e-05	0.348977	0.0831092	1	2:74448672:A/C	8	RP11-287D1.3:SLC4A5	intronic	3.316	4	4	5
2:74449302:A:G	2:74449302:A/G	A	G	0.06561	1.12522e-05	0.357743	0.0814607	1	2:74448672:A/C	8	RP11-287D1.3:SLC4A5	intronic	10.38	4	4	5
2:74450125:C:T	2:74450125:A/G	C	T	0.09443	0.000194062	0.267124	0.07168	0.66373	2:74448672:A/C	8	RP11-287D1.3:SLC4A5	intronic	8.374	5	4	5
2:74450426:A:G	2:74450426:A/G	A	G	0.0666	3.67172e-05	0.346576	0.0839733	0.95204	2:74448672:A/C	8	RP11-287D1.3:SLC4A5	intronic	0.494	3a	4	5
2:74452327:A:G	2:74452327:A/G	A	G	0.06561	1.30116e-05	0.362651	0.0831787	1	2:74448672:A/C	8	RP11-287D1.3:SLC4A5	intronic	6.616	5	5	5
2:74454448:C:G	2:74454448:C/G	C	G	0.06759	8.29731e-06	0.363566	0.0815654	0.968301	2:74448672:A/C	8	RP11-287D1.3:SLC4A5	intronic	2.52	5	2	15
2:74455626:A:G	2:74455626:A/G	G	A	0.09443	8.56094e-05	0.281671	0.0717062	0.66373	2:74448672:A/C	8	RP11-287D1.3:SLC4A5	intronic	0.529	6	5	15
2:74455795:A:C	2:74455795:A/C	A	C	0.09245	6.76274e-05	0.290014	0.0727859	0.679368	2:74448672:A/C	8	RP11-287D1.3:SLC4A5	intronic	0.01	6	5	15
2:74466805:C:T	2:74466805:A/G	T	C	0.05865	2.1016e-05	0.351091	0.0825355	0.9024	2:74480659:A/G	8	RP11-287D1.3:SLC4A5	intronic	3.3	5	5	15
2:74472942:A:G	2:74472942:A/G	A	G	0.06163	1.96178e-05	0.358406	0.0839519	0.918566	2:74480659:A/G	8	RP11-287D1.3:SLC4A5	intronic	0.419	6	5	15
2:74480659:C:T	2:74480659:A/G	T	C	0.06262	1.20832e-05	0.369175	0.0843608	1	2:74480659:A/G	8	RP11-287D1.3:SLC4A5	intronic	5.766	4	4	15
2:80153660:C:T	2:80153660:A/G	C	T	0.1083	0.0002153	0.364963	0.0986288	0.921055	2:80173676:A/G	9	CTNNA2	intronic	12.85	7	14	15
2:80155631:A:G	2:80155631:A/G	A	G	0.0994	0.000103348	0.385883	0.0993875	0.96851	2:80173676:A/G	9	CTNNA2	intronic	0.64	6	9	15
2:80155953:A:C	2:80155953:A/C	A	C	0.0994	5.92081e-05	0.398674	0.0992729	0.96851	2:80173676:A/G	9	CTNNA2	intronic	0.35	6	9	15
2:80157134:A:G	2:80157134:A/G	A	G	0.0994	9.63088e-05	0.387253	0.0993031	0.96851	2:80173676:A/G	9	CTNNA2	intronic	2.148	7	9	15
2:80159495:A:G	2:80159495:A/G	A	G	0.0994	7.04896e-05	0.392876	0.0988462	0.96851	2:80173676:A/G	9	CTNNA2	intronic	1.484	7	13	15
2:80161323:A:G	2:80161323:A/G	G	A	0.0994	3.82384e-05	0.401459	0.0974916	0.96851	2:80173676:A/G	9	CTNNA2	intronic	4.329	6	13	15
2:80162739:A:G	2:80162739:A/G	G	A	0.1014	4.40466e-05	0.39722	0.0972348	0.989534	2:80173676:A/G	9	CTNNA2	intronic	7.198	6	1	15
2:80163345:G:T	2:80163345:A/C	G	T	0.0994	5.19664e-05	0.395002	0.0976132	0.96851	2:80173676:A/G	9	CTNNA2	intronic	0.761	6	1	15
2:80164058:A:G	2:80164058:A/G	A	G	0.0994	4.46089e-05	0.399575	0.0978818	0.96851	2:80173676:A/G	9	CTNNA2	intronic	0.633	5	5	15
2:80165151:A:G	2:80165151:A/G	A	G	0.0994	3.56865e-05	0.404139	0.0977653	0.96851	2:80173676:A/G	9	CTNNA2	intronic	1.599	6	5	15
2:80166580:A:T	2:80166580:A/T	T	A	0.0994	4.24998e-05	0.400647	0.0978751	0.96851	2:80173676:A/G	9	CTNNA2	intronic	0.239	7	5	15
2:80166751:A:G	2:80166751:A/G	G	A	0.1004	5.02835e-05	0.395778	0.0976193	0.958753	2:80173676:A/G	9	CTNNA2	intronic	4.086	7	5	15
2:80173393:C:T	2:80173393:A/G	C	T	0.1004	3.6418e-05	0.402628	0.0975097	1	2:80173676:A/G	9	CTNNA2	intronic	7.999	6	7	15
2:80173676:A:G	2:80173676:A/G	G	A	0.1004	3.50557e-05	0.403085	0.0974139	1	2:80173676:A/G	9	CTNNA2	intronic	5.632	7	7	15
2:180887549:A:C	2:180887549:A/C	C	A	0.1978	0.00252601	0.166457	0.0551147	0.762672	2:180903712:A/G	10	CWC22	intergenic	4.725	6	1	15
2:180903712:C:T	2:180903712:A/G	C	T	0.162	1.33303e-05	0.26598	0.0610802	1	2:180903712:A/G	10	CWC22	intergenic	7.36	5	2	15
2:180916057:C:T	2:180916057:A/G	T	C	0.16	2.64673e-05	0.257142	0.0611965	0.969953	2:180903712:A/G	10	CWC22	intergenic	1.234	6	13	15

2:180916851:G:T	2:180916851:A/C	G	T	0.162	2.19849e-05	0.259819	0.0612244	0.969967	2:180903712:A/G	10	CWC22	intergenic	14.04	6	13	15
2:180919710:C:G	2:180919710:C/G	G	C	0.161	2.7437e-05	0.256669	0.0612028	0.977419	2:180903712:A/G	10	CWC22	intergenic	0.162	6	9	15
2:180921285:A:G	2:180921285:A/G	G	A	0.16	3.16486e-05	0.254891	0.0612532	0.984953	2:180903712:A/G	10	CWC22	intergenic	6.018	5	1	15
2:180921299:A:ATGTTTCTAGACTTGCTGGCTCCT	rs140865078	ATGTTTCTAGACTTGCTGGCTCCT	A	0.161	NA	NA	NA	0.977419	2:180903712:A/G	10	CWC22	intergenic	5.581	NA	1	15
2:180921948:A:G	2:180921948:A/G	A	G	0.2078	4.35092e-05	0.242049	0.0592096	0.700333	2:180903712:A/G	10	CWC22	intergenic	1.976	4	1	15
2:180922282:C:CA	rs199616551	CA	C	0.2078	NA	NA	NA	0.700333	2:180903712:A/G	10	CWC22	intergenic	1.102	NA	2	15
2:180922514:A:G	2:180922514:A/G	G	A	0.161	3.28166e-05	0.254286	0.0612297	0.977419	2:180903712:A/G	10	CWC22	intergenic	4.61	7	2	15
2:180923510:C:T	2:180923510:A/G	C	T	0.162	3.26942e-05	0.254217	0.0612005	0.969967	2:180903712:A/G	10	CWC22	intergenic	0.45	6	5	15
2:180924628:T:TTC	rs113165336	TTC	T	0.161	NA	NA	NA	0.977419	2:180903712:A/G	10	CWC22	intergenic	0.81	NA	5	15
2:180925068:A:G	2:180925068:A/G	A	G	0.162	3.81157e-05	0.252148	0.0612214	0.969967	2:180903712:A/G	10	CWC22	intergenic	4.669	7	5	15
2:180925173:G:GCT	rs148934177	GCT	G	0.162	NA	NA	NA	0.969967	2:180903712:A/G	10	CWC22	intergenic	3.9	NA	5	15
2:180925205:G:T	2:180925205:A/C	T	G	0.161	3.46869e-05	0.253635	0.06126	0.977419	2:180903712:A/G	10	CWC22	intergenic	1.126	7	5	15
2:180925353:C:T	2:180925353:A/G	C	T	0.2306	0.0241234	0.118706	0.0526373	0.627854	2:180903712:A/G	10	CWC22	intergenic	0.272	7	5	15
2:180927383:A:C	2:180927383:A/C	A	C	0.1571	3.78839e-05	0.252302	0.0612381	0.947801	2:180903712:A/G	10	CWC22	intergenic	5.8	7	5	15
2:180927718:C:G	2:180927718:C/G	C	G	0.1571	3.79854e-05	0.252263	0.0612375	0.947801	2:180903712:A/G	10	CWC22	intergenic	3.579	6	7	15
2:180927797:C:T	2:180927797:A/G	T	C	0.1938	0.00642324	0.15005	0.0550567	0.731749	2:180903712:A/G	10	CWC22	intergenic	6.595	6	7	15
2:180928071:C:T	2:180928071:A/G	T	C	0.1561	3.88958e-05	0.252062	0.0612701	0.955353	2:180903712:A/G	10	CWC22	intergenic	0.993	5	7	15
2:180928685:A:G	2:180928685:A/G	G	A	0.1571	3.80185e-05	0.252242	0.0612357	0.947801	2:180903712:A/G	10	CWC22	intergenic	0.349	6	7	15
2:180931158:A:G	2:180931158:A/G	A	G	0.1561	4.98681e-05	0.247859	0.0611056	0.940498	2:180903712:A/G	10	CWC22	intergenic	4.354	7	9	15
2:180932807:A:G	2:180932807:A/G	A	G	0.171	0.00356816	0.160664	0.0551351	0.709664	2:180903712:A/G	10	CWC22	intergenic	1.432	6	9	15
2:180933088:GA:GAA	rs559049874	GAA	GA	0.1103	NA	NA	NA	0.85348	2:180903712:A/G	10	CWC22	intergenic	NA	NA	9	15
2:180933088:G:GAA	rs550224562	GAA	G	0.1501	NA	NA	NA	0.85348	2:180903712:A/G	10	CWC22	intergenic	0.546	NA	9	15
2:180933378:A:AT	rs146806995	AT	A	0.171	NA	NA	NA	0.709664	2:180903712:A/G	10	CWC22	intergenic	0.555	NA	9	15
2:180933415:C:G	2:180933415:C/G	C	G	0.171	0.00443647	0.157141	0.0552277	0.709664	2:180903712:A/G	10	CWC22	intergenic	0.561	7	9	15
2:180934568:G:T	2:180934568:A/C	G	T	0.1461	6.92023e-05	0.244045	0.061333	0.869005	2:180903712:A/G	10	CWC22	intergenic	7.648	7	8	9
2:180934883:C:T	2:180934883:A/G	C	T	0.1451	6.91947e-05	0.244507	0.0614487	0.876681	2:180903712:A/G	10	CWC22	intergenic	7.691	3a	8	9
2:180934994:A:G	2:180934994:A/G	A	G	0.1581	0.000210433	0.226447	0.0611003	0.800004	2:180903712:A/G	10	CWC22	intergenic	2.505	7	8	9
2:180935579:G:T	2:180935579:A/C	G	T	0.1471	6.04717e-05	0.245852	0.061295	0.875955	2:180903712:A/G	10	CWC22	intergenic	2.123	7	8	15
2:180935621:A:G	2:180935621:A/G	G	A	0.1461	6.76985e-05	0.244447	0.0613535	0.869005	2:180903712:A/G	10	CWC22	intergenic	1.532	7	8	15
2:180935838:A:G	2:180935838:A/G	A	G	0.1451	7.49017e-05	0.243563	0.0615036	0.876681	2:180903712:A/G	10	CWC22	intergenic	2.174	7	5	15
2:180936223:C:T	2:180936223:A/G	T	C	0.1461	6.76794e-05	0.244453	0.0613541	0.869005	2:180903712:A/G	10	CWC22	intergenic	1.845	NA	7	15
2:180936487:A:AAG	rs142513284	AAG	A	0.1461	NA	NA	NA	0.869005	2:180903712:A/G	10	CWC22	intergenic	5.909	NA	2	15
2:180937300:C:G	2:180937300:C/G	C	G	0.1461	6.60124e-05	0.244847	0.0613619	0.869005	2:180903712:A/G	10	CWC22	intergenic	15.4	7	7	15
2:180937415:C:T	2:180937415:A/G	C	T	0.1461	6.75669e-05	0.244489	0.061357	0.869005	2:180903712:A/G	10	CWC22	intergenic	2.038	7	7	15
2:180937606:C:T	2:180937606:A/G	C	T	0.1481	4.07802e-05	-0.252189	0.0614643	0.853927	2:180903712:A/G	10	CWC22	intergenic	3.222	NA	7	15
2:180951352:A:G	2:180951352:A/G	A	G	0.1451	0.000122586	0.240818	0.0626981	0.876681	2:180903712:A/G	10	CWC22	intergenic	1.558	5	13	15
2:180955608:A:G	2:180955608:A/G	G	A	0.1571	0.00121561	0.196651	0.060785	0.806633	2:180903712:A/G	10	CWC22	intergenic	2.762	7	14	15
2:180957266:C:G	2:180957266:C/G	G	C	0.1581	0.00142453	0.195877	0.0614103	0.800004	2:180903712:A/G	10	CWC22	intergenic	1.64	4	14	15
2:180975234:C:T	2:180975234:A/G	C	T	0.1541	0.0024059	0.185317	0.0610613	0.798225	2:180903712:A/G	10	CWC22	intergenic	3.577	5	14	15
2:180975968:A:T	2:180975968:A/T	A	T	0.1541	0.00234994	0.185838	0.06109	0.798225	2:180903712:A/G	10	CWC22	intergenic	3.313	7	14	15
2:224366956:C:T	2:224366956:A/G	T	C	0.05765	8.19206e-06	0.338785	0.0759591	0.946123	2:224369405:A/G	11	AC013448.1	ncRNA_intronic	10.59	7	5	15

2:224369405:C:T	2:224369405:A/G	T	C	0.05467	7.31059e-06	0.341197	0.0760847	1	2:224369405:A/G	11	AC013448.1	upstream:downstream	1.232	5	5	15
2:224370681:G:T	2:224370681:A/C	T	G	0.05765	8.89772e-06	0.332665	0.0748849	0.946123	2:224369405:A/G	11	AC013448.2	ncRNA_intronic	1.702	7	5	15
2:224372147:C:T	2:224372147:A/G	C	T	0.05765	8.76678e-06	0.332979	0.0749017	0.946123	2:224369405:A/G	11	AC013448.2	ncRNA_intronic	7.589	7	14	15
2:224376688:A:G	2:224376688:A/G	G	A	0.05865	1.6358e-05	0.319343	0.0741012	0.859912	2:224369405:A/G	11	AC013448.2	intergenic	6.476	7	5	15
2:224377034:C:T	2:224377034:A/G	T	C	0.05865	1.15002e-05	0.322824	0.0735888	0.859912	2:224369405:A/G	11	AC013448.2	intergenic	0.274	7	7	15
2:224377587:A:T	2:224377587:A/T	A	T	0.07952	8.65305e-05	0.266268	0.0678295	0.624785	2:224369405:A/G	11	AC013448.2	intergenic	0.003	6	5	15
2:224378483:A:G	2:224378483:A/G	A	G	0.05964	5.93563e-05	0.2949	0.0734432	0.844697	2:224369405:A/G	11	AC013448.2	intergenic	0.908	7	5	15
2:224381091:A:G	2:224381091:A/G	A	G	0.05964	5.89908e-05	0.294885	0.0734127	0.844697	2:224369405:A/G	11	AC013448.2	intergenic	0.499	7	12	15
2:224382268:A:G	2:224382268:A/G	G	A	0.05964	5.99144e-05	0.294596	0.0734078	0.844697	2:224369405:A/G	11	AC013448.2	intergenic	0.131	NA	14	15
2:224382867:A:G	2:224382867:A/G	A	G	0.05964	5.88066e-05	0.294886	0.0733989	0.844697	2:224369405:A/G	11	AC013448.2	intergenic	0.702	7	7	15
2:224383305:A:C	2:224383305:A/C	A	C	0.07853	6.88975e-05	0.270282	0.067909	0.625296	2:224369405:A/G	11	AC013448.2	intergenic	0.683	7	14	15
2:224384118:C:T	2:224384118:A/G	C	T	0.06064	5.50938e-05	0.296017	0.0734008	0.82995	2:224369405:A/G	11	AC013448.2	intergenic	3.672	5	7	15
2:224385408:A:T	2:224385408:A/T	A	T	0.07853	7.45125e-05	0.268712	0.0678329	0.625296	2:224369405:A/G	11	AC013448.2	intergenic	0.073	6	5	15
2:224385409:A:T	2:224385409:A/T	T	A	0.07853	7.45054e-05	0.268714	0.0678328	0.625296	2:224369405:A/G	11	AC013448.2	intergenic	1.42	6	5	15
2:224385862:A:G	2:224385862:A/G	G	A	0.05964	5.83094e-05	0.294854	0.0733549	0.844697	2:224369405:A/G	11	AC013448.2	intergenic	0.645	6	5	15
2:224389483:G:T	2:224389483:A/C	T	G	0.05964	5.51658e-05	0.295687	0.0733244	0.844697	2:224369405:A/G	11	AC013448.2	intergenic	0.045	7	5	15
2:224390180:C:G	2:224390180:C/G	C	G	0.06064	5.11612e-05	0.297025	0.0733349	0.82995	2:224369405:A/G	11	AC013448.2	intergenic	3.722	4	5	15
2:224392062:G:T	2:224392062:A/C	T	G	0.05964	8.19807e-05	0.288623	0.0732819	0.844697	2:224369405:A/G	11	AC013448.2	intergenic	0.103	6	5	15
2:224393706:C:T	2:224393706:A/G	T	C	0.05964	5.09042e-05	0.29712	0.0733369	0.844697	2:224369405:A/G	11	AC013448.2	intergenic	0.71	NA	5	15
2:224394123:C:T	2:224394123:A/G	C	T	0.05964	4.94787e-05	0.297609	0.0733375	0.844697	2:224369405:A/G	11	AC013448.2	intergenic	0.137	7	5	15
2:224394506:C:T	2:224394506:A/G	T	C	0.05964	4.89171e-05	0.297571	0.0732801	0.844697	2:224369405:A/G	11	AC013448.2	intergenic	0.571	5	5	15
2:224394966:C:T	2:224394966:A/G	C	T	0.05964	4.91523e-05	0.297774	0.0733503	0.844697	2:224369405:A/G	11	AC013448.2	intergenic	3.299	7	5	15
2:224395891:A:G	2:224395891:A/G	A	G	0.05964	4.81847e-05	0.298294	0.0733945	0.844697	2:224369405:A/G	11	AC013448.2	intergenic	1.947	7	5	15
2:224396882:C:T	2:224396882:A/G	C	T	0.05964	4.51699e-05	0.301388	0.0738825	0.844697	2:224369405:A/G	11	AC013448.2	intergenic	20.8	NA	5	15
2:224397999:A:C	2:224397999:A/C	C	A	0.05964	4.36723e-05	0.301768	0.0738335	0.844697	2:224369405:A/G	11	AC013448.2	intergenic	10.34	6	5	15
2:224398740:A:C	2:224398740:A/C	C	A	0.05964	4.36284e-05	0.301856	0.0738509	0.844697	2:224369405:A/G	11	AC013448.2	intergenic	3.251	NA	5	15
2:224399923:A:T	2:224399923:A/T	A	T	0.05964	4.59579e-05	0.299539	0.0735012	0.844697	2:224369405:A/G	11	AC013448.2	intergenic	3.746	7	5	15
2:238718254:A:T	2:238718254:A/T	T	A	0.1064	0.006043	-0.262565	0.0956367	0.682855	2:238742059:A/G	12	LRRFIP1:RBM44	intronic	4.804	7	9	15
2:238742059:C:T	2:238742059:A/G	C	T	0.1421	2.87108e-05	-0.320067	0.0765078	1	2:238742059:A/G	12	RBM44	intronic	6.29	7	7	15
2:238752088:C:T	2:238752088:A/G	T	C	0.1362	3.0897e-05	-0.330932	0.0794214	0.938252	2:238742059:A/G	12	RBM44	downstream	0.209	7	14	15
2:240145208:A:T	2:240145208:A/T	T	A	0.1491	1.03058e-05	-0.443399	0.100529	1	2:240148856:C/G	13	HDAC4	intronic	4.198	4	2	7
2:240145638:C:T	2:240145638:A/G	C	T	0.1461	0.000132364	-0.410641	0.107441	0.976616	2:240148856:C/G	13	HDAC4	intronic	2.905	4	1	7
2:240145989:G:T	2:240145989:A/C	T	G	0.1461	2.10376e-05	-0.434843	0.102229	0.976616	2:240148856:C/G	13	HDAC4	intronic	1.266	4	2	7
2:240146026:C:T	2:240146026:A/G	T	C	0.1461	0.000132153	-0.410762	0.107461	0.976616	2:240148856:C/G	13	HDAC4	intronic	0.379	4	2	7
2:240147102:A:G	2:240147102:A/G	G	A	0.1461	1.9212e-05	-0.43697	0.102242	0.976616	2:240148856:C/G	13	HDAC4	intronic	2.071	5	2	7
2:240147459:G:T	2:240147459:A/C	G	T	0.1461	0.000126532	-0.41213	0.107518	0.976616	2:240148856:C/G	13	HDAC4	intronic	5.284	5	2	7
2:240148856:C:G	2:240148856:C/G	C	G	0.1491	9.5034e-06	-0.445582	0.100624	1	2:240148856:C/G	13	HDAC4	intronic	2.687	5	2	7
2:240149180:C:T	2:240149180:A/G	T	C	0.1461	3.51937e-05	-0.46489	0.112375	0.961073	2:240148856:C/G	13	HDAC4	intronic	4.886	5	2	7
2:240149285:C:G	2:240149285:C/G	G	C	0.1451	0.000143249	-0.410255	0.107891	0.968822	2:240148856:C/G	13	HDAC4	intronic	8.908	5	2	7
2:240151759:C:T	2:240151759:A/G	T	C	0.1461	1.82845e-05	-0.438616	0.102364	0.976616	2:240148856:C/G	13	HDAC4	intronic	1.623	4	2	5
2:240168808:C:T	2:240168808:A/G	C	T	0.1352	0.0008038	-0.407149	0.121483	0.862589	2:240148856:C/G	13	HDAC4	intronic	4.155	5	1	7
2:240169247:C:T	2:240169247:A/G	T	C	0.1322	0.000553248	-0.421675	0.122099	0.855653	2:240148856:C/G	13	HDAC4	intronic	0.217	4	1	5

2:240172182:C:CCTGCC ACTGGGG	rs577932425	C	CCTGCCACTG GGG	0.1302	NA	NA	NA	0.856943	2:240148856:C/G	13	HDAC4	intronic	0.975	NA	2	5
3:28043123:C:T	3:28043123:A/G	T	C	0.0338	4.59198e-05	-0.513113	0.125903	1	3:28046439:A/G	14	AC092415.1	ncRNA_intronic	3.102	6	9	15
3:28044301:G:T	3:28044301:A/C	T	G	0.0338	5.77876e-05	-0.476246	0.11842	1	3:28046439:A/G	14	AC092415.1	ncRNA_intronic	2.245	7	9	15
3:28046439:A:G	3:28046439:A/G	G	A	0.0338	3.87265e-05	-0.510758	0.124123	1	3:28046439:A/G	14	AC092415.1	ncRNA_intronic	1.564	7	9	15
3:28051430:A:G	3:28051430:A/G	G	A	0.0338	7.71995e-05	-0.482022	0.12194	1	3:28046439:A/G	14	AC092415.1	ncRNA_intronic	0.343	7	14	15
3:28053322:C:T	3:28053322:A/G	C	T	0.0338	5.168e-05	-0.505225	0.124812	1	3:28046439:A/G	14	AC092415.1	ncRNA_intronic	0.1	6	14	15
3:28077836:C:T	3:28077836:A/G	T	C	0.02783	0.00318456	-0.456584	0.154808	0.693317	3:28046439:A/G	14	AC092415.1	intergenic	5.381	5	7	15
3:43181148:C:T	3:43181148:A/G	T	C	0.165	1.37762e-05	-0.455692	0.104819	1	3:43186832:A/G	15	POMGNT2	intergenic	0.023	5	7	15
3:43186832:C:T	3:43186832:A/G	T	C	0.165	8.20181e-06	-0.47184	0.105797	1	3:43186832:A/G	15	POMGNT2	intergenic	0.137	2b	2	15
3:45196727:A:G	3:45196727:A/G	G	A	0.03181	0.0238722	-0.320299	0.141777	0.600291	3:45276160:A/G	16	RPS24P8	intergenic	2.4	3a	5	15
3:45220880:A:G	3:45220880:A/G	A	G	0.04771	0.0023629	0.270674	0.0890268	0.628405	3:45276160:A/G	16	RPS24P8	intergenic	1.485	3a	5	15
3:45221630:G:T	3:45221630:A/C	T	G	0.04771	0.00242203	0.269531	0.0888684	0.628405	3:45276160:A/G	16	RPS24P8	intergenic	2.732	5	5	15
3:45223868:C:G	3:45223868:C/G	G	C	0.04871	0.00334342	0.264242	0.090054	0.649789	3:45276160:A/G	16	RPS24P8	intergenic	1.458	6	5	15
3:45228202:C:G	3:45228202:C/G	C	G	0.05169	0.00103656	0.286803	0.0874291	0.677631	3:45276160:A/G	16	RPS24P8	intergenic	8.679	5	5	15
3:45232261:A:T	3:45232261:A/T	T	A	0.05169	0.00100433	0.286681	0.0871556	0.677631	3:45276160:A/G	16	RPS24P8	intergenic	3.222	6	5	15
3:45248903:A:G	3:45248903:A/G	A	G	0.04771	0.000453394	0.307763	0.0877599	0.742722	3:45276160:A/G	16	TMEM158	intergenic	3.896	5	2	15
3:45252284:G:T	3:45252284:A/C	T	G	0.04274	0.00470566	0.299727	0.106041	0.71322	3:45276160:A/G	16	TMEM158	intergenic	0.657	2b	2	7
3:45260774:C:G	3:45260774:C/G	G	C	0.04473	3.4917e-05	0.382651	0.0924552	0.886161	3:45276160:A/G	16	TMEM158	intergenic	3.634	2b	2	14
3:45273331:A:G	3:45273331:A/G	G	A	0.04374	1.44328e-05	0.398011	0.0917675	0.908678	3:45276160:A/G	16	TMEM158	intergenic	2.31	7	5	14
3:45273915:C:T	3:45273915:A/G	C	T	0.04473	1.72466e-05	0.394737	0.0918454	0.931359	3:45276160:A/G	16	TMEM158	intergenic	7.103	7	5	14
3:45274915:A:G	3:45274915:A/G	A	G	0.04473	1.12258e-05	0.405431	0.092309	0.931359	3:45276160:A/G	16	TMEM158	intergenic	5.752	6	7	14
3:45276160:C:T	3:45276160:A/G	T	C	0.04771	1.10324e-05	0.405481	0.0922411	1	3:45276160:A/G	16	TMEM158	intergenic	0.899	6	7	14
3:45276625:C:T	3:45276625:A/G	T	C	0.04771	1.10851e-05	0.405486	0.092264	1	3:45276160:A/G	16	TMEM158	intergenic	11.44	7	9	14
3:45278251:A:G	3:45278251:A/G	G	A	0.03082	0.000733382	-0.47187	0.139739	0.622572	3:45276160:A/G	16	TMEM158	intergenic	0.067	6	9	14
3:45279264:C:G	3:45279264:C/G	G	C	0.04771	1.1397e-05	0.405408	0.0923729	1	3:45276160:A/G	16	TMEM158	intergenic	1.334	7	5	14
3:45279271:C:T	3:45279271:A/G	T	C	0.04771	1.13906e-05	0.40542	0.0923732	1	3:45276160:A/G	16	TMEM158	intergenic	1.476	6	5	14
3:45280066:A:G	3:45280066:A/G	A	G	0.04771	1.15035e-05	0.405346	0.0924014	1	3:45276160:A/G	16	TMEM158	intergenic	3.123	7	5	14
3:88377746:C:T	3:88377746:A/G	C	T	0.337	1.45784e-05	0.2171	0.0500812	0.982063	3:88377838:C/G	17	ABCF2P1	intergenic	4.795	NA	14	15
3:88377838:C:G	3:88377838:C/G	C	G	0.341	5.35111e-06	0.225909	0.0496445	1	3:88377838:C/G	17	ABCF2P1	intergenic	0.19	7	14	15
3:88378348:A:G	3:88378348:A/G	G	A	0.338	1.10426e-05	0.219677	0.0499757	0.977489	3:88377838:C/G	17	ABCF2P1	intergenic	0.581	5	14	15
3:88391859:T:TAA	rs34419876	T	TAA	0.3648	NA	NA	NA	0.866375	3:88377838:C/G	17	ABCF2P1	intergenic	0.314	NA	14	15
3:154186078:C:T	3:154186078:A/G	T	C	0.06958	0.000441442	0.321278	0.0914284	0.609351	3:154253687:A/G	18	GPR149	intergenic	2.157	7	7	15
3:154191561:A:G	3:154191561:A/G	A	G	0.06958	0.000527972	0.315063	0.0908971	0.609351	3:154253687:A/G	18	RP11-656A15.1	intergenic	3.775	5	9	15
3:154194189:G:T	3:154194189:A/C	T	G	0.06958	0.000327416	0.325424	0.0905821	0.609351	3:154253687:A/G	18	RP11-656A15.1	intergenic	1.27	7	7	15
3:154194897:A:G	3:154194897:A/G	A	G	0.07157	0.000363325	0.321406	0.0901461	0.628208	3:154253687:A/G	18	RP11-656A15.1	intergenic	0.562	7	9	15
3:154198263:C:T	3:154198263:A/G	C	T	0.07157	0.000397736	0.318838	0.0900267	0.628208	3:154253687:A/G	18	RP11-656A15.1	intergenic	6.154	7	9	15
3:154198781:A:C	3:154198781:A/C	A	C	0.07157	0.000397165	0.318828	0.0900143	0.628208	3:154253687:A/G	18	RP11-656A15.1	intergenic	0.481	7	9	15
3:154199558:G:T	3:154199558:A/C	T	G	0.07157	0.000395634	0.31886	0.0899977	0.628208	3:154253687:A/G	18	RP11-656A15.1	intergenic	0.966	7	9	15
3:154199594:G:T	3:154199594:A/C	T	G	0.07157	0.000395478	0.318868	0.0899972	0.628208	3:154253687:A/G	18	RP11-656A15.1	intergenic	2.998	6	9	15
3:154200054:C:G	3:154200054:C/G	C	G	0.07157	0.000388801	0.318966	0.0899109	0.628208	3:154253687:A/G	18	RP11-656A15.1	intergenic	3.544	4	9	15
3:154201589:C:G	3:154201589:C/G	C	G	0.07157	0.00026331	0.328748	0.0900938	0.628208	3:154253687:A/G	18	RP11-656A15.1	intergenic	0.065	7	14	15
3:154202085:A:G	3:154202085:A/G	A	G	0.07256	0.000351572	0.320591	0.089701	0.637702	3:154253687:A/G	18	RP11-656A15.1	intergenic	5.925	6	14	15

3:154202689:A:G	3:154202689:A/G	A	G	0.07157	0.000313169	0.324492	0.0900326	0.628208	3:154253687:A/G	18	RP11-656A15.1	intergenic	8.378	6	14	15
3:154203270:C:G	3:154203270:C/G	G	C	0.07157	0.000337968	0.322476	0.0899686	0.628208	3:154253687:A/G	18	RP11-656A15.1	intergenic	1.618	7	7	15
3:154203735:A:G	3:154203735:A/G	G	A	0.07157	0.000316625	0.324159	0.0900114	0.628208	3:154253687:A/G	18	RP11-656A15.1	intergenic	7.587	6	9	15
3:154204344:A:G	3:154204344:A/G	G	A	0.07157	0.000335578	0.322643	0.0899687	0.628208	3:154253687:A/G	18	RP11-656A15.1	intergenic	0.467	5	9	15
3:154204842:A:C	3:154204842:A/C	A	C	0.07157	0.000335249	0.322655	0.0899658	0.628208	3:154253687:A/G	18	RP11-656A15.1	intergenic	0.037	5	7	15
3:154206643:C:T	3:154206643:A/G	C	T	0.07157	0.000332198	0.322873	0.0899667	0.628208	3:154253687:A/G	18	RP11-656A15.1	intergenic	9.196	6	9	15
3:154206684:C:T	3:154206684:A/G	C	T	0.07157	0.000256292	0.329384	0.0900969	0.628208	3:154253687:A/G	18	RP11-656A15.1	intergenic	2.355	7	9	15
3:154209130:A:C	3:154209130:A/C	A	C	0.07157	0.000328148	0.323163	0.0899675	0.628208	3:154253687:A/G	18	RP11-656A15.1	intergenic	2.014	7	14	15
3:154209327:C:T	3:154209327:A/G	T	C	0.07157	0.000327689	0.323196	0.0899676	0.628208	3:154253687:A/G	18	RP11-656A15.1	intergenic	2.016	6	14	15
3:154210035:C:T	3:154210035:A/G	T	C	0.07157	0.000251923	0.329784	0.0900977	0.628208	3:154253687:A/G	18	RP11-656A15.1	intergenic	0.12	6	14	15
3:154210381:A:G	3:154210381:A/G	A	G	0.07157	0.000337365	0.32263	0.0900001	0.628208	3:154253687:A/G	18	RP11-656A15.1	intergenic	3.416	6	14	15
3:154211111:C:T	3:154211111:A/G	C	T	0.07157	0.000332142	0.323015	0.0900051	0.628208	3:154253687:A/G	18	RP11-656A15.1	intergenic	0.463	7	9	15
3:154212394:A:AG	rs142925455	AG	A	0.07157	NA	NA	NA	0.628208	3:154253687:A/G	18	RP11-656A15.1	intergenic	4.717	NA	9	15
3:154213887:C:T	3:154213887:A/G	T	C	0.07157	0.000268105	0.328314	0.0900895	0.628208	3:154253687:A/G	18	RP11-656A15.1	intergenic	5.167	7	9	15
3:154214221:C:T	3:154214221:A/G	C	T	0.07157	0.000325162	0.323515	0.0900056	0.628208	3:154253687:A/G	18	RP11-656A15.1	intergenic	9.028	7	9	15
3:154214593:G:T	3:154214593:A/C	G	T	0.07157	0.000325002	0.323526	0.0900055	0.628208	3:154253687:A/G	18	RP11-656A15.1	intergenic	2.461	7	9	15
3:154217016:T:TCTC	rs72020210	TCTC	T	0.07157	NA	NA	NA	0.628208	3:154253687:A/G	18	RP11-656A15.1	intergenic	1.344	NA	9	15
3:154219106:A:T	3:154219106:A/T	T	A	0.07157	0.000265481	0.327561	0.0898204	0.628208	3:154253687:A/G	18	RP11-656A15.1	intergenic	4.479	7	9	15
3:154222712:A:G	3:154222712:A/G	A	G	0.07157	0.000941786	0.292963	0.088579	0.628208	3:154253687:A/G	18	RP11-656A15.1	intergenic	0.912	7	15	15
3:154222851:A:G	3:154222851:A/G	G	A	0.07157	0.000263577	0.327694	0.0898112	0.628208	3:154253687:A/G	18	RP11-656A15.1	intergenic	0.782	7	15	15
3:154224113:C:T	3:154224113:A/G	T	C	0.07157	0.000270856	0.327144	0.0898331	0.628208	3:154253687:A/G	18	RP11-656A15.1	intergenic	0.052	5	15	15
3:154224170:A:G	3:154224170:A/G	A	G	0.07157	0.000202746	0.334598	0.090053	0.628208	3:154253687:A/G	18	RP11-656A15.1	intergenic	1.462	5	15	15
3:154224310:A:C	3:154224310:A/C	C	A	0.07157	0.000266629	0.327495	0.0898296	0.628208	3:154253687:A/G	18	RP11-656A15.1	intergenic	1.526	7	15	15
3:154224335:C:T	3:154224335:A/G	T	C	0.07157	0.000266591	0.327498	0.0898295	0.628208	3:154253687:A/G	18	RP11-656A15.1	intergenic	1.863	6	15	15
3:154224522:C:T	3:154224522:A/G	C	T	0.07157	0.000266548	0.327489	0.0898261	0.628208	3:154253687:A/G	18	RP11-656A15.1	intergenic	5.347	6	15	15
3:154224645:A:AAAAC	rs370633247	AAAAC	A	0.06163	NA	NA	NA	0.628208	3:154253687:A/G	18	RP11-656A15.1	intergenic	9.093	NA	15	15
3:154224645:A:AAAACAAAC	rs143460413	AAAACAAAC	A	0.07157	NA	NA	NA	0.628208	3:154253687:A/G	18	RP11-656A15.1	NA	8.061	NA	15	15
3:154224645:AAAAC:AAAACAAAC	rs571966178	AAAAC	AAAACAAAC	0.06958	NA	NA	NA	0.628208	3:154253687:A/G	18	RP11-656A15.1	NA	NA	NA	15	15
3:154224988:C:T	3:154224988:A/G	C	T	0.07157	0.000264878	0.327656	0.0898321	0.628208	3:154253687:A/G	18	RP11-656A15.1	intergenic	1.733	7	15	15
3:154225336:C:CAA	rs34050922	CAA	C	0.07157	NA	NA	NA	0.628208	3:154253687:A/G	18	RP11-656A15.1	intergenic	2.284	NA	15	15
3:154225927:A:G	3:154225927:A/G	G	A	0.07157	0.000266072	0.327526	0.0898251	0.628208	3:154253687:A/G	18	RP11-656A15.1	intergenic	1.146	7	9	15
3:154226055:C:T	3:154226055:A/G	C	T	0.07157	0.00026603	0.32753	0.089825	0.628208	3:154253687:A/G	18	RP11-656A15.1	intergenic	1.969	7	9	15
3:154226273:A:G	3:154226273:A/G	G	A	0.07157	0.000264087	0.327722	0.0898312	0.628208	3:154253687:A/G	18	RP11-656A15.1	intergenic	0.139	6	9	15
3:154226372:C:T	3:154226372:A/G	T	C	0.07157	0.000264087	0.327722	0.0898312	0.628208	3:154253687:A/G	18	RP11-656A15.1	intergenic	1.895	7	9	15
3:154226911:G:T	3:154226911:A/C	G	T	0.07157	0.000265816	0.327546	0.0898243	0.628208	3:154253687:A/G	18	RP11-656A15.1	intergenic	5.286	6	9	15
3:154227113:A:C	3:154227113:A/C	C	A	0.07157	0.000265777	0.327549	0.0898242	0.628208	3:154253687:A/G	18	RP11-656A15.1	intergenic	1.075	7	9	15
3:154227114:A:G	3:154227114:A/G	A	G	0.07157	0.000265777	0.327549	0.0898242	0.628208	3:154253687:A/G	18	RP11-656A15.1	intergenic	1.394	7	9	15
3:154227203:A:G	3:154227203:A/G	G	A	0.07157	0.000265732	0.327553	0.0898241	0.628208	3:154253687:A/G	18	RP11-656A15.1	intergenic	1.528	6	9	15
3:154227572:C:T	3:154227572:A/G	C	T	0.07157	0.000335391	-0.321937	0.0897685	0.628208	3:154253687:A/G	18	RP11-656A15.1	intergenic	0.026	7	9	15
3:154228052:C:T	3:154228052:A/G	T	C	0.07157	0.000265563	0.327566	0.0898237	0.628208	3:154253687:A/G	18	RP11-656A15.1	intergenic	0.559	7	9	15
3:154228434:T:TA	rs144445536	T	TA	0.07256	NA	NA	NA	0.637702	3:154253687:A/G	18	RP11-656A15.1	downstream	2.098	NA	9	15

3:154229004:C:T	3:154229004:A/G	T	C	0.07157	0.000265395	0.327579	0.0898234	0.628208	3:154253687:A/G	18	RP11-656A15.1	downstream	0.091	5	9	15
3:154229165:A:AG	rs35213890	AG	A	0.07157	NA	NA	NA	0.628208	3:154253687:A/G	18	RP11-656A15.1	downstream	0.388	NA	9	15
3:154229905:A:C	3:154229905:A/C	C	A	0.07157	0.000354096	0.320232	0.0896475	0.628208	3:154253687:A/G	18	RP11-656A15.1	ncRNA_intronic	0.603	7	9	15
3:154230084:C:T	3:154230084:A/G	T	C	0.07256	0.000287691	0.324682	0.0895395	0.637702	3:154253687:A/G	18	RP11-656A15.1	ncRNA_intronic	1.529	6	9	15
3:154232436:C:T	3:154232436:A/G	C	T	0.07157	0.000262803	0.327843	0.0898336	0.628208	3:154253687:A/G	18	RP11-656A15.1	ncRNA_intronic	2.848	6	9	15
3:154232549:C:G	3:154232549:C/G	C	G	0.07157	0.000263161	0.327811	0.0898335	0.628208	3:154253687:A/G	18	RP11-656A15.1	ncRNA_intronic	1.02	5	9	15
3:154232603:A:G	3:154232603:A/G	A	G	0.07157	0.000265014	0.327607	0.089822	0.628208	3:154253687:A/G	18	RP11-656A15.1	ncRNA_intronic	6.672	5	9	15
3:154233967:A:G	3:154233967:A/G	G	A	0.07157	0.000264745	0.327629	0.0898216	0.628208	3:154253687:A/G	18	RP11-656A15.1	ncRNA_intronic	5.457	7	9	15
3:154234105:G:GTTTAT	rs140856495	GTTTAT	G	0.07157	NA	NA	NA	0.628208	3:154253687:A/G	18	RP11-656A15.1	ncRNA_intronic	10.82	NA	9	15
3:154234138:C:T	3:154234138:A/G	C	T	0.07157	0.000264709	0.327632	0.0898215	0.628208	3:154253687:A/G	18	RP11-656A15.1	ncRNA_intronic	1.175	7	9	15
3:154234266:A:G	3:154234266:A/G	G	A	0.07157	0.000264709	0.327632	0.0898215	0.628208	3:154253687:A/G	18	RP11-656A15.1	ncRNA_intronic	0.027	7	9	15
3:154234827:A:G	3:154234827:A/G	G	A	0.07157	0.000262459	0.32787	0.0898328	0.628208	3:154253687:A/G	18	RP11-656A15.1	ncRNA_intronic	10.05	6	9	15
3:154235124:C:T	3:154235124:A/G	C	T	0.07157	0.000264577	0.327643	0.0898213	0.628208	3:154253687:A/G	18	RP11-656A15.1	ncRNA_intronic	0.054	7	9	15
3:154235125:A:C	3:154235125:A/C	C	A	0.07157	0.000264577	0.327643	0.0898213	0.628208	3:154253687:A/G	18	RP11-656A15.1	ncRNA_intronic	6.382	7	9	15
3:154235459:G:T	3:154235459:A/C	G	T	0.07157	0.000264535	0.327646	0.0898212	0.628208	3:154253687:A/G	18	RP11-656A15.1	ncRNA_intronic	3.45	3a	9	15
3:154236292:C:CT	rs140354332	CT	C	0.07157	NA	NA	NA	0.628208	3:154253687:A/G	18	RP11-656A15.1	ncRNA_intronic	1.058	NA	9	15
3:154236498:A:G	3:154236498:A/G	G	A	0.07256	0.000186263	0.336186	0.0899627	0.637702	3:154253687:A/G	18	RP11-656A15.1	ncRNA_intronic	0.244	7	9	15
3:154237442:C:T	3:154237442:A/G	T	C	0.07256	0.000283496	0.324538	0.0894062	0.617075	3:154253687:A/G	18	RP11-656A15.1	ncRNA_intronic	1.8	NA	9	15
3:154237670:C:T	3:154237670:A/G	C	T	0.07157	0.000264278	0.327666	0.0898204	0.628208	3:154253687:A/G	18	RP11-656A15.1	ncRNA_intronic	1.432	7	9	15
3:154238443:A:G	3:154238443:A/G	G	A	0.07157	0.000264106	0.32768	0.0898201	0.628208	3:154253687:A/G	18	RP11-656A15.1	ncRNA_intronic	4.607	7	14	15
3:154238495:A:T	3:154238495:A/T	T	A	0.07157	0.00026389	0.327697	0.0898197	0.628208	3:154253687:A/G	18	RP11-656A15.1	ncRNA_intronic	1.801	6	14	15
3:154239762:A:AT	rs144671670	A	AT	0.07157	NA	NA	NA	0.628208	3:154253687:A/G	18	RP11-656A15.1	ncRNA_intronic	0.23	NA	14	15
3:154239763:A:T	3:154239763:A/T	T	A	0.07157	0.000256497	0.328239	0.0897887	0.628208	3:154253687:A/G	18	RP11-656A15.1	ncRNA_intronic	0.505	6	14	15
3:154240489:C:T	3:154240489:A/G	C	T	0.1213	0.000192391	0.299291	0.0802646	0.862157	3:154253687:A/G	18	RP11-656A15.1	ncRNA_intronic	2.402	7	14	15
3:154246071:C:T	3:154246071:A/G	T	C	0.1074	4.44364e-05	0.345338	0.0845771	0.977976	3:154253687:A/G	18	RP11-656A15.1	ncRNA_intronic	5.45	6	14	15
3:154246953:A:G	3:154246953:A/G	A	G	0.1083	5.0081e-05	0.342647	0.0844944	0.989154	3:154253687:A/G	18	RP11-656A15.1	ncRNA_intronic	1.895	7	14	15
3:154248317:G:T	3:154248317:A/C	G	T	0.1223	0.000159905	0.304579	0.0806796	0.85597	3:154253687:A/G	18	RP11-656A15.1	ncRNA_intronic	0.712	7	14	15
3:154249019:C:G	3:154249019:C/G	C	G	0.1074	5.76759e-05	0.338913	0.084262	1	3:154253687:A/G	18	RP11-656A15.1	ncRNA_intronic	0.054	7	14	15
3:154249759:A:AT	rs543709299	AT	A	0.1064	NA	NA	NA	0.988768	3:154253687:A/G	18	RP11-656A15.1	ncRNA_intronic	1.234	NA	14	15
3:154249773:C:T	3:154249773:A/G	C	T	0.1083	8.05052e-05	0.330658	0.0838615	0.989154	3:154253687:A/G	18	RP11-656A15.1	ncRNA_intronic	1.619	6	14	15
3:154249889:A:C	3:154249889:A/C	A	C	0.1074	4.52551e-05	0.34479	0.0845307	1	3:154253687:A/G	18	RP11-656A15.1	ncRNA_intronic	4.019	6	14	15
3:154250302:A:T	3:154250302:A/T	A	T	0.1213	0.000146171	0.308644	0.0812759	0.862157	3:154253687:A/G	18	RP11-656A15.1	ncRNA_intronic	2.225	6	14	15
3:154250305:G:T	3:154250305:A/C	T	G	0.1213	0.000126934	0.309662	0.0808022	0.862157	3:154253687:A/G	18	RP11-656A15.1	ncRNA_intronic	2.412	6	14	15
3:154252113:C:CT	rs113154552	CT	C	0.1074	NA	NA	NA	1	3:154253687:A/G	18	RP11-656A15.1	ncRNA_intronic	1.148	NA	14	15
3:154252271:C:T	3:154252271:A/G	T	C	0.1074	5.11993e-05	0.3416	0.0843439	1	3:154253687:A/G	18	RP11-656A15.1	ncRNA_intronic	0.243	7	14	15
3:154252288:G:T	3:154252288:A/C	T	G	0.1213	0.000390765	0.28384	0.0800395	0.862157	3:154253687:A/G	18	RP11-656A15.1	ncRNA_intronic	0.915	6	14	15
3:154252403:G:GT	rs138385717	G	GT	0.1093	NA	NA	NA	0.955872	3:154253687:A/G	18	RP11-656A15.1	NA	0.523	NA	14	15
3:154252403:G:T	rs562663153	G	T	0.000994	NA	NA	NA	0.955872	3:154253687:A/G	18	RP11-656A15.1	ncRNA_intronic	0.197	NA	14	15
3:154253163:C:G	3:154253163:C/G	G	C	0.1014	2.33083e-05	0.365805	0.0864667	0.933442	3:154253687:A/G	18	RP11-656A15.1	ncRNA_intronic	0.253	6	14	15
3:154253314:A:G	3:154253314:A/G	G	A	0.1213	0.000433792	0.281352	0.079961	0.862157	3:154253687:A/G	18	RP11-656A15.1	ncRNA_intronic	1.562	NA	14	15
3:154253687:C:T	3:154253687:A/G	T	C	0.1074	3.05356e-05	0.353165	0.0847032	1	3:154253687:A/G	18	RP11-656A15.1	ncRNA_intronic	2.348	NA	9	15
3:154257816:A:T	3:154257816:A/T	A	T	0.1064	3.47672e-05	0.350626	0.0846972	0.988768	3:154253687:A/G	18	RP11-656A15.1	upstream	9.068	7	5	15
3:154258918:G:T	3:154258918:A/C	T	G	0.1064	3.39913e-05	0.351102	0.0847062	0.988768	3:154253687:A/G	18	RP11-656A15.1	intergenic	3.195	NA	5	15

3:154263701:C:T	3:154263701:A/G	T	C	0.1213	0.000404241	0.283165	0.0800512	0.862157	3:154253687:A/G	18	RP11-656A15.1	intergenic	0.587	NA	9	15
3:154263757:G:GC	rs143580119	GC	G	0.07157	NA	NA	NA	0.628208	3:154253687:A/G	18	RP11-656A15.1	intergenic	0.268	NA	9	15
3:154265270:C:T	3:154265270:A/G	T	C	0.1064	3.31631e-05	0.351618	0.0847153	0.988768	3:154253687:A/G	18	RP11-656A15.1	intergenic	0.301	5	14	15
3:154267617:G:T	3:154267617:A/C	G	T	0.1193	0.000448706	0.280634	0.0799606	0.840101	3:154253687:A/G	18	RP11-656A15.1	intergenic	0.524	7	9	15
3:154269571:A:G	3:154269571:A/G	A	G	0.1213	0.000511564	0.277453	0.0798512	0.822887	3:154253687:A/G	18	RP11-656A15.1	intergenic	0.768	6	9	15
3:154271977:A:G	3:154271977:A/G	G	A	0.1044	2.06402e-05	0.359741	0.0844888	0.966473	3:154253687:A/G	18	RP11-656A15.1	intergenic	4.702	7	14	15
3:154272241:C:G	3:154272241:C/G	G	C	0.1223	0.00044502	0.280281	0.0798103	0.81448	3:154253687:A/G	18	RP11-656A15.1	intergenic	1.997	6	14	15
3:154272311:G:T	3:154272311:A/C	T	G	0.1223	0.000395896	0.28237	0.0797023	0.81448	3:154253687:A/G	18	RP11-656A15.1	intergenic	0.751	7	14	15
3:154272618:A:C	3:154272618:A/C	C	A	0.1223	0.000391436	0.282696	0.0797271	0.81448	3:154253687:A/G	18	RP11-656A15.1	intergenic	1.217	6	9	15
3:154273143:C:T	3:154273143:A/G	C	T	0.1074	2.87745e-05	0.34952	0.0835581	0.977876	3:154294388:A/G	18	RP11-656A15.1	intergenic	2.826	7	9	15
3:154273219:A:G	3:154273219:A/G	A	G	0.1074	2.70882e-05	0.350664	0.0835579	0.977876	3:154294388:A/G	18	RP11-656A15.1	intergenic	3.628	7	9	15
3:154273424:A:G	3:154273424:A/G	G	A	0.1074	2.68626e-05	0.350819	0.0835571	0.977876	3:154294388:A/G	18	RP11-656A15.1	intergenic	0.114	7	9	15
3:154273463:A:G	3:154273463:A/G	G	A	0.1074	2.67952e-05	0.350865	0.0835567	0.977876	3:154294388:A/G	18	RP11-656A15.1	intergenic	0.101	7	9	15
3:154273582:A:AC	rs139035450	AC	A	0.1064	NA	NA	NA	0.966896	3:154294388:A/G	18	RP11-656A15.1	intergenic	0.022	NA	9	15
3:154273611:A:AT	rs113895627	A	AT	0.1272	NA	NA	NA	0.770476	3:154294388:A/G	18	RP11-656A15.1	intergenic	1.228	NA	9	15
3:154273854:C:T	3:154273854:A/G	T	C	0.1074	2.62769e-05	0.351238	0.0835575	0.977876	3:154294388:A/G	18	RP11-656A15.1	intergenic	1.379	7	9	15
3:154273883:C:T	3:154273883:A/G	T	C	0.1074	2.62694e-05	0.351236	0.083556	0.977876	3:154294388:A/G	18	RP11-656A15.1	intergenic	1.457	6	9	15
3:154273970:C:T	3:154273970:A/G	C	T	0.1074	2.62091e-05	0.351322	0.083566	0.977876	3:154294388:A/G	18	RP11-656A15.1	intergenic	6.451	7	9	15
3:154273990:A:G	3:154273990:A/G	A	G	0.1074	2.6172e-05	0.35135	0.0835663	0.977876	3:154294388:A/G	18	RP11-656A15.1	intergenic	0.468	7	9	15
3:154273999:C:T	3:154273999:A/G	T	C	0.1074	2.61575e-05	0.351359	0.0835661	0.977876	3:154294388:A/G	18	RP11-656A15.1	intergenic	1.553	6	9	15
3:154274017:C:T	3:154274017:A/G	T	C	0.1074	2.61488e-05	0.351365	0.0835658	0.977876	3:154294388:A/G	18	RP11-656A15.1	intergenic	3.011	6	9	15
3:154274167:A:G	3:154274167:A/G	G	A	0.1074	2.59847e-05	0.35143	0.083553	0.977876	3:154294388:A/G	18	RP11-656A15.1	intergenic	2.849	6	9	15
3:154274394:G:T	3:154274394:A/C	T	G	0.1074	2.57311e-05	0.351606	0.0835507	0.977876	3:154294388:A/G	18	RP11-656A15.1	intergenic	3.076	6	9	15
3:154274625:A:T	3:154274625:A/T	A	T	0.1074	2.5426e-05	0.351825	0.0835494	0.977876	3:154294388:A/G	18	RP11-656A15.1	intergenic	2.673	5	9	15
3:154274944:G:T	3:154274944:A/C	T	G	0.1074	2.5069e-05	0.352079	0.0835463	0.977876	3:154294388:A/G	18	RP11-656A15.1	intergenic	0.384	6	9	15
3:154275099:A:G	3:154275099:A/G	A	G	0.1074	2.48755e-05	0.352214	0.0835438	0.977876	3:154294388:A/G	18	RP11-656A15.1	intergenic	1.166	6	9	15
3:154275379:C:T	3:154275379:A/G	C	T	0.1074	2.43352e-05	0.352635	0.0835455	0.977876	3:154294388:A/G	18	RP11-656A15.1	intergenic	6.952	6	9	15
3:154275492:C:T	3:154275492:A/G	C	T	0.1074	4.31697e-05	0.337785	0.0825914	0.977876	3:154294388:A/G	18	RP11-656A15.1	intergenic	0.614	5	7	15
3:154275664:C:T	3:154275664:A/G	C	T	0.1074	2.4273e-05	0.352649	0.0835373	0.977876	3:154294388:A/G	18	RP11-656A15.1	intergenic	2.224	5	7	15
3:154275767:C:T	3:154275767:A/G	T	C	0.1074	2.4153e-05	0.352736	0.0835357	0.977876	3:154294388:A/G	18	RP11-656A15.1	intergenic	2.659	7	7	15
3:154275967:G:T	3:154275967:A/C	T	G	0.1074	2.39435e-05	0.352889	0.0835332	0.977876	3:154294388:A/G	18	RP11-656A15.1	intergenic	6.211	7	7	15
3:154276080:A:G	3:154276080:A/G	A	G	0.1074	2.38168e-05	0.352981	0.0835314	0.977876	3:154294388:A/G	18	RP11-656A15.1	intergenic	0.221	7	7	15
3:154276249:C:T	3:154276249:A/G	C	T	0.1074	2.38089e-05	0.352991	0.0835323	0.977876	3:154294388:A/G	18	RP11-656A15.1	intergenic	0.146	7	7	15
3:154276285:C:T	3:154276285:A/G	T	C	0.1074	2.36507e-05	0.353106	0.0835298	0.977876	3:154294388:A/G	18	RP11-656A15.1	intergenic	5.636	6	7	15
3:154276293:A:G	3:154276293:A/G	A	G	0.1074	2.57961e-05	0.351446	0.083524	0.977876	3:154294388:A/G	18	RP11-656A15.1	intergenic	0.002	6	7	15
3:154276586:A:C	3:154276586:A/C	A	C	0.1074	2.33679e-05	0.353312	0.0835249	0.977876	3:154294388:A/G	18	RP11-656A15.1	intergenic	4.075	7	2	15
3:154276691:A:T	3:154276691:A/T	A	T	0.1074	2.32674e-05	0.353384	0.0835229	0.977876	3:154294388:A/G	18	RP11-656A15.1	intergenic	16.09	6	2	15
3:154276693:A:G	3:154276693:A/G	A	G	0.1074	2.32674e-05	0.353384	0.0835229	0.977876	3:154294388:A/G	18	RP11-656A15.1	intergenic	14.5	6	2	15
3:154276725:A:G	3:154276725:A/G	A	G	0.1074	2.32682e-05	0.353374	0.0835206	0.977876	3:154294388:A/G	18	RP11-656A15.1	intergenic	1.146	5	2	15
3:154276798:G:T	3:154276798:A/C	G	T	0.1074	2.31877e-05	0.353432	0.0835191	0.977876	3:154294388:A/G	18	RP11-656A15.1	intergenic	7.446	5	2	15
3:154276897:A:G	3:154276897:A/G	A	G	0.1223	0.000335237	0.283341	0.0790035	0.833204	3:154294388:A/G	18	RP11-656A15.1	intergenic	1.174	7	7	15
3:154276988:A:G	3:154276988:A/G	G	A	0.1074	2.30014e-05	0.353572	0.0835164	0.977876	3:154294388:A/G	18	RP11-656A15.1	intergenic	6.861	7	7	15
3:154277027:G:T	3:154277027:A/C	G	T	0.1074	2.29779e-05	0.353587	0.0835154	0.977876	3:154294388:A/G	18	RP11-656A15.1	intergenic	6.569	6	7	15

3:154277340:A:G	3:154277340:A/G	A	G	0.1074	2.26973e-05	0.353792	0.0835093	0.977876	3:154294388:A/G	18	RP11-656A15.1	intergenic	0.17	6	7	15
3:154277416:G:T	3:154277416:A/C	G	T	0.1223	0.000402195	0.279135	0.078882	0.833204	3:154294388:A/G	18	RP11-656A15.1	intergenic	2.501	6	7	15
3:154277650:C:T	3:154277650:A/G	T	C	0.1074	2.2389e-05	0.354024	0.0835036	0.977876	3:154294388:A/G	18	RP11-656A15.1	intergenic	3.346	5	7	15
3:154277730:A:G	3:154277730:A/G	G	A	0.1074	7.42365e-05	0.326835	0.0824864	0.977876	3:154294388:A/G	18	RP11-656A15.1	intergenic	5.704	6	7	15
3:154277740:A:C	3:154277740:A/C	A	C	0.1223	0.000397439	0.279349	0.0788723	0.833204	3:154294388:A/G	18	RP11-656A15.1	intergenic	2.923	7	7	15
3:154277837:C:T	3:154277837:A/G	C	T	0.1074	2.22475e-05	0.354126	0.0834995	0.977876	3:154294388:A/G	18	RP11-656A15.1	intergenic	12.16	7	7	15
3:154277908:G:T	3:154277908:A/C	G	T	0.1074	2.21668e-05	0.354186	0.0834976	0.977876	3:154294388:A/G	18	RP11-656A15.1	intergenic	5.42	7	7	15
3:154277932:G:T	3:154277932:A/C	T	G	0.1074	2.22003e-05	0.354149	0.0834957	0.977876	3:154294388:A/G	18	RP11-656A15.1	intergenic	3.783	7	7	15
3:154278147:G:T	3:154278147:A/C	G	T	0.1083	2.26496e-05	0.353828	0.0835085	0.966852	3:154294388:A/G	18	RP11-656A15.1	intergenic	0.248	6	7	15
3:154278250:C:T	3:154278250:A/G	C	T	0.1074	2.20283e-05	0.35431	0.0834991	0.977876	3:154294388:A/G	18	RP11-656A15.1	intergenic	4.914	7	7	15
3:154278396:C:T	3:154278396:A/G	T	C	0.1074	1.79985e-05	0.35812	0.0835094	0.977876	3:154294388:A/G	18	RP11-656A15.1	intergenic	1.613	7	7	15
3:154278496:A:T	3:154278496:A/T	T	A	0.1074	2.18106e-05	0.3545	0.0835002	0.977876	3:154294388:A/G	18	RP11-656A15.1	intergenic	0.559	6	9	15
3:154278503:G:T	3:154278503:A/C	G	T	0.1074	2.19562e-05	0.354369	0.0834985	0.977876	3:154294388:A/G	18	RP11-656A15.1	intergenic	0.248	6	9	15
3:154278643:A:G	3:154278643:A/G	G	A	0.1074	2.19125e-05	0.354407	0.0834988	0.977876	3:154294388:A/G	18	RP11-656A15.1	intergenic	1.369	7	9	15
3:154278756:A:T	3:154278756:A/T	T	A	0.1074	2.18733e-05	0.354442	0.0834991	0.977876	3:154294388:A/G	18	RP11-656A15.1	intergenic	6.571	7	9	15
3:154278840:C:T	3:154278840:A/G	T	C	0.1074	2.18424e-05	0.354469	0.0834993	0.977876	3:154294388:A/G	18	RP11-656A15.1	intergenic	5.277	6	9	15
3:154279121:C:T	3:154279121:A/G	C	T	0.1074	2.17551e-05	0.354546	0.0834999	0.977876	3:154294388:A/G	18	RP11-656A15.1	intergenic	2.584	7	9	15
3:154279234:C:T	3:154279234:A/G	C	T	0.07356	7.28001e-05	0.359549	0.0906364	0.614726	3:154294388:A/G	18	RP11-656A15.1	intergenic	2.407	7	14	15
3:154279275:C:T	3:154279275:A/G	C	T	0.1074	2.17012e-05	0.354593	0.0834999	0.977876	3:154294388:A/G	18	RP11-656A15.1	intergenic	3.195	7	14	15
3:154279469:A:G	3:154279469:A/G	G	A	0.1074	2.16372e-05	0.354649	0.0835001	0.977876	3:154294388:A/G	18	RP11-656A15.1	intergenic	3.573	6	14	15
3:154279509:A:T	3:154279509:A/T	T	A	0.1074	2.21019e-05	0.354323	0.0835171	0.977876	3:154294388:A/G	18	RP11-656A15.1	intergenic	1.876	7	14	15
3:154279609:C:T	3:154279609:A/G	C	T	0.1074	2.15818e-05	0.354698	0.0835004	0.977876	3:154294388:A/G	18	RP11-656A15.1	intergenic	4.232	7	14	15
3:154279750:C:T	3:154279750:A/G	C	T	0.1074	2.10912e-05	0.355132	0.0835013	0.977876	3:154294388:A/G	18	RP11-656A15.1	intergenic	4.596	7	14	15
3:154279947:C:T	3:154279947:A/G	T	C	0.1074	2.1083e-05	0.355139	0.0835013	0.977876	3:154294388:A/G	18	RP11-656A15.1	intergenic	4.113	5	14	15
3:154281430:A:G	3:154281430:A/G	A	G	0.1074	2.05305e-05	0.35565	0.0835047	0.977876	3:154294388:A/G	18	RP11-656A15.1	intergenic	1.352	7	9	15
3:154282025:A:G	3:154282025:A/G	A	G	0.1074	2.05739e-05	0.35559	0.0834999	0.977876	3:154294388:A/G	18	RP11-656A15.1	intergenic	1.576	6	9	15
3:154282832:A:G	3:154282832:A/G	A	G	0.1223	0.000374603	0.280257	0.0787824	0.833204	3:154294388:A/G	18	RP11-656A15.1	intergenic	0.976	6	9	15
3:154283960:C:G	3:154283960:C/G	C	G	0.1074	2.0519e-05	0.355639	0.0834996	0.977876	3:154294388:A/G	18	RP11-656A15.1	intergenic	0.116	6	9	15
3:154284222:C:T	3:154284222:A/G	C	T	0.1223	0.000374432	0.28026	0.0787805	0.833204	3:154294388:A/G	18	RP11-656A15.1	intergenic	3.725	6	9	15
3:154285372:G:T	3:154285372:A/C	T	G	0.1223	0.000396471	0.279397	0.0788718	0.833204	3:154294388:A/G	18	RP11-656A15.1	intergenic	2.443	6	7	15
3:154285786:C:T	3:154285786:A/G	C	T	0.1233	0.000215056	0.290646	0.0785394	0.824734	3:154294388:A/G	18	RP11-656A15.1	intergenic	2.881	7	9	15
3:154285931:C:T	3:154285931:A/G	T	C	0.1223	0.000382498	0.279817	0.07878	0.833204	3:154294388:A/G	18	RP11-656A15.1	intergenic	3.09	5	9	15
3:154286644:A:AT	rs11462698	A	AT	0.1223	NA	NA	NA	0.833204	3:154294388:A/G	18	RP11-656A15.1	intergenic	0.702	NA	9	15
3:154286676:A:T	3:154286676:A/T	A	T	0.1223	0.000373169	0.280323	0.0787785	0.833204	3:154294388:A/G	18	RP11-656A15.1	intergenic	2.785	7	9	15
3:154287190:A:G	3:154287190:A/G	A	G	0.1223	0.000419005	0.277885	0.0787696	0.833204	3:154294388:A/G	18	RP11-656A15.1	intergenic	0.184	6	9	15
3:154287263:G:T	3:154287263:A/C	T	G	0.1223	0.000372996	0.280328	0.0787773	0.833204	3:154294388:A/G	18	RP11-656A15.1	intergenic	1.545	6	9	15
3:154287271:A:G	3:154287271:A/G	G	A	0.1223	0.000373005	0.280328	0.0787773	0.833204	3:154294388:A/G	18	RP11-656A15.1	intergenic	0.45	6	9	15
3:154287333:A:C	3:154287333:A/C	C	A	0.1223	0.000373125	0.28032	0.078777	0.833204	3:154294388:A/G	18	RP11-656A15.1	intergenic	6.204	7	9	15
3:154287339:A:G	3:154287339:A/G	A	G	0.1074	2.02565e-05	0.355934	0.0835124	0.977876	3:154294388:A/G	18	RP11-656A15.1	intergenic	4.81	7	9	15
3:154287399:A:G	3:154287399:A/G	G	A	0.1223	0.000373123	0.28032	0.0787769	0.833204	3:154294388:A/G	18	RP11-656A15.1	intergenic	2.552	6	9	15
3:154287470:C:G	3:154287470:C/G	C	G	0.1223	0.000372372	0.280363	0.0787772	0.833204	3:154294388:A/G	18	RP11-656A15.1	intergenic	5.467	7	9	15
3:154287511:A:G	3:154287511:A/G	A	G	0.1074	1.84113e-05	0.357436	0.0834481	0.977876	3:154294388:A/G	18	RP11-656A15.1	intergenic	1.807	7	9	15
3:154287700:G:T	3:154287700:A/C	G	T	0.1223	0.000372845	0.28032	0.0787726	0.833204	3:154294388:A/G	18	RP11-656A15.1	intergenic	3.186	7	9	15

3:154287940:C:T	3:154287940:A/G	C	T	0.1223	0.000360853	0.280961	0.0787628	0.833204	3:154294388:A/G	18	RP11-656A15.1	intergenic	2.982	7	9	15
3:154288088:C:T	3:154288088:A/G	T	C	0.1223	0.000371309	0.280326	0.0787503	0.833204	3:154294388:A/G	18	RP11-656A15.1	intergenic	1.527	7	9	15
3:154288123:C:G	3:154288123:C/G	G	C	0.1074	8.52851e-05	0.323522	0.0823412	0.977876	3:154294388:A/G	18	RP11-656A15.1	intergenic	4.13	5	9	15
3:154288238:C:T	3:154288238:A/G	C	T	0.1074	8.65989e-05	0.323251	0.0823493	0.977876	3:154294388:A/G	18	RP11-656A15.1	intergenic	4.774	5	9	15
3:154288269:A:G	3:154288269:A/G	G	A	0.1074	8.63477e-05	0.323308	0.082349	0.977876	3:154294388:A/G	18	RP11-656A15.1	intergenic	1.503	6	9	15
3:154288315:A:T	3:154288315:A/T	T	A	0.1233	0.000402842	0.278297	0.0786545	0.824734	3:154294388:A/G	18	RP11-656A15.1	intergenic	5.108	6	9	15
3:154288411:C:G	3:154288411:C/G	G	C	0.1223	0.000103956	0.309318	0.0796972	0.833204	3:154294388:A/G	18	RP11-656A15.1	intergenic	3.221	7	9	15
3:154288772:A:G	3:154288772:A/G	A	G	0.1074	1.914e-05	0.356925	0.0834972	0.977876	3:154294388:A/G	18	RP11-656A15.1	intergenic	4.072	7	9	15
3:154288853:A:G	3:154288853:A/G	A	G	0.1074	2.3471e-05	0.352888	0.0834444	0.977876	3:154294388:A/G	18	RP11-656A15.1	intergenic	8.761	7	9	15
3:154289275:G:GTAATTT	rs10650069	G	GTAATTT	0.1074	NA	NA	NA	0.977876	3:154294388:A/G	18	RP11-656A15.1	intergenic	2.153	NA	9	15
3:154289512:A:G	3:154289512:A/G	A	G	0.1074	2.32641e-05	0.352962	0.0834226	0.977876	3:154294388:A/G	18	RP11-656A15.1	intergenic	4.967	5	7	15
3:154289618:C:T	3:154289618:A/G	C	T	0.1074	2.3019e-05	0.353208	0.0834338	0.977876	3:154294388:A/G	18	RP11-656A15.1	intergenic	2.048	5	7	15
3:154289734:C:T	3:154289734:A/G	T	C	0.1074	1.87417e-05	0.357274	0.0834872	0.977876	3:154294388:A/G	18	RP11-656A15.1	intergenic	10.79	5	7	15
3:154289811:C:T	3:154289811:A/G	C	T	0.1074	2.27854e-05	0.35337	0.0834269	0.977876	3:154294388:A/G	18	RP11-656A15.1	intergenic	4.114	5	7	15
3:154289986:C:T	3:154289986:A/G	C	T	0.1074	2.28015e-05	0.353362	0.0834279	0.977876	3:154294388:A/G	18	RP11-656A15.1	intergenic	2.71	5	7	15
3:154290003:A:G	3:154290003:A/G	A	G	0.1074	2.29813e-05	0.35316	0.0834152	0.977876	3:154294388:A/G	18	RP11-656A15.1	intergenic	4.384	5	7	15
3:154290118:A:G	3:154290118:A/G	G	A	0.1074	2.29346e-05	0.353193	0.0834139	0.977876	3:154294388:A/G	18	RP11-656A15.1	intergenic	0.984	6	7	15
3:154290308:C:T	3:154290308:A/G	T	C	0.1074	2.28497e-05	0.353253	0.0834117	0.977876	3:154294388:A/G	18	RP11-656A15.1	intergenic	2.245	6	7	15
3:154290336:G:GA	rs11314140	GA	G	0.1093	NA	NA	NA	0.956022	3:154294388:A/G	18	RP11-656A15.1	intergenic	1.458	NA	7	15
3:154290434:T:TA	rs566725727	T	TA	0.1074	NA	NA	NA	0.977876	3:154294388:A/G	18	RP11-656A15.1	intergenic	0.71	NA	7	15
3:154290439:A:G	3:154290439:A/G	A	G	0.1074	2.2796e-05	0.353291	0.0834101	0.977876	3:154294388:A/G	18	RP11-656A15.1	intergenic	1.411	6	7	15
3:154290474:C:T	3:154290474:A/G	T	C	0.1074	2.27835e-05	0.353299	0.0834098	0.977876	3:154294388:A/G	18	RP11-656A15.1	intergenic	3.443	7	7	15
3:154290631:A:G	3:154290631:A/G	G	A	0.1074	2.37475e-05	0.352534	0.0834126	0.977876	3:154294388:A/G	18	RP11-656A15.1	intergenic	2.5	7	15	15
3:154290766:C:T	3:154290766:A/G	T	C	0.1074	2.27016e-05	-0.353355	0.0834071	0.977876	3:154294388:A/G	18	RP11-656A15.1	intergenic	2.158	NA	15	15
3:154290828:A:G	3:154290828:A/G	A	G	0.1083	2.44108e-05	0.352047	0.0834198	0.966852	3:154294388:A/G	18	RP11-656A15.1	intergenic	3.421	7	15	15
3:154290854:A:G	3:154290854:A/G	G	A	0.1074	2.26864e-05	0.353366	0.0834066	0.977876	3:154294388:A/G	18	RP11-656A15.1	intergenic	1.16	7	15	15
3:154290959:C:T	3:154290959:A/G	T	C	0.1074	2.26789e-05	0.353371	0.0834064	0.977876	3:154294388:A/G	18	RP11-656A15.1	intergenic	3.16	7	15	15
3:154291009:A:G	3:154291009:A/G	G	A	0.1074	2.26751e-05	0.353374	0.0834063	0.977876	3:154294388:A/G	18	RP11-656A15.1	intergenic	1.589	7	9	15
3:154291048:A:G	3:154291048:A/G	A	G	0.1233	0.00035016	0.281159	0.0786446	0.824734	3:154294388:A/G	18	RP11-656A15.1	intergenic	0.824	6	9	15
3:154291363:C:T	3:154291363:A/G	C	T	0.1074	2.26524e-05	0.35339	0.0834056	0.977876	3:154294388:A/G	18	RP11-656A15.1	intergenic	9.561	6	9	15
3:154291456:C:G	3:154291456:C/G	C	G	0.1074	2.26298e-05	0.353405	0.0834049	0.977876	3:154294388:A/G	18	RP11-656A15.1	intergenic	6.039	7	9	15
3:154291478:A:G	3:154291478:A/G	A	G	0.1074	1.97336e-05	0.355124	0.0832084	0.977876	3:154294388:A/G	18	RP11-656A15.1	intergenic	6.598	7	9	15
3:154291627:A:AT	rs149204676	AT	A	0.1074	NA	NA	NA	0.977876	3:154294388:A/G	18	RP11-656A15.1	intergenic	0.612	NA	9	15
3:154291962:C:G	3:154291962:C/G	G	C	0.1074	2.15976e-05	0.356543	0.0839378	0.977876	3:154294388:A/G	18	RP11-656A15.1	intergenic	7.294	7	9	15
3:154291973:C:T	3:154291973:A/G	C	T	0.1074	2.67212e-05	0.352289	0.0838834	0.977876	3:154294388:A/G	18	RP11-656A15.1	intergenic	6.641	7	9	15
3:154292075:G:T	3:154292075:A/C	T	G	0.1083	2.48133e-05	0.353765	0.0839004	0.966852	3:154294388:A/G	18	RP11-656A15.1	intergenic	1.452	7	9	15
3:154292139:G:T	3:154292139:A/C	G	T	0.1074	2.68861e-05	0.352237	0.0838987	0.977876	3:154294388:A/G	18	RP11-656A15.1	intergenic	6.021	6	9	15
3:154292183:C:T	3:154292183:A/G	T	C	0.1074	2.69378e-05	0.352212	0.0839016	0.977876	3:154294388:A/G	18	RP11-656A15.1	intergenic	6.961	7	9	15
3:154292249:C:T	3:154292249:A/G	C	T	0.1074	2.69676e-05	0.352209	0.0839059	0.977876	3:154294388:A/G	18	RP11-656A15.1	intergenic	0.667	7	9	15
3:154292259:A:T	3:154292259:A/T	A	T	0.1074	2.69934e-05	0.352194	0.0839067	0.977876	3:154294388:A/G	18	RP11-656A15.1	intergenic	0.323	6	9	15
3:154292271:C:T	3:154292271:A/G	C	T	0.1074	3.08815e-05	0.349631	0.0839074	0.977876	3:154294388:A/G	18	RP11-656A15.1	intergenic	4.033	6	9	15
3:154292364:C:T	3:154292364:A/G	T	C	0.1074	2.70597e-05	0.352178	0.0839138	0.977876	3:154294388:A/G	18	RP11-656A15.1	intergenic	6.116	7	9	15

3:154292676:A:G	3:154292676:A/G	A	G	0.1074	2.74682e-05	0.352055	0.0839526	0.977876	3:154294388:A/G	18	RP11-656A15.1	intergenic	4.996	5	9	15
3:154292992:A:C	3:154292992:A/C	A	C	0.1074	2.8475e-05	0.351761	0.0840461	0.977876	3:154294388:A/G	18	RP11-656A15.1	intergenic	5.572	6	9	15
3:154293257:A:G	3:154293257:A/G	G	A	0.1074	2.89682e-05	0.35177	0.0841268	0.977876	3:154294388:A/G	18	RP11-656A15.1	intergenic	2.641	7	9	15
3:154293277:A:G	3:154293277:A/G	A	G	0.1074	2.95194e-05	0.35142	0.0841293	0.977876	3:154294388:A/G	18	RP11-656A15.1	intergenic	3.831	7	9	15
3:154293671:G:T	3:154293671:A/C	T	G	0.1233	0.000476345	0.276934	0.0792661	0.824734	3:154294388:A/G	18	RP11-656A15.1	intergenic	1.51	7	9	15
3:154294236:A:G	3:154294236:A/G	A	G	0.1074	3.31985e-05	0.350267	0.0843948	0.977876	3:154294388:A/G	18	RP11-656A15.1	intergenic	2.439	7	9	15
3:154294262:C:T	3:154294262:A/G	T	C	0.1074	3.33283e-05	0.350219	0.0844014	0.977876	3:154294388:A/G	18	RP11-656A15.1	intergenic	1.573	7	9	15
3:154294380:A:C	3:154294380:A/C	A	C	0.1233	0.00051862	0.275611	0.0794051	0.824734	3:154294388:A/G	18	RP11-656A15.1	intergenic	3.062	7	9	15
3:154294388:C:T	3:154294388:A/G	T	C	0.1093	1.63293e-05	0.362152	0.0840271	1	3:154294388:A/G	18	RP11-656A15.1	intergenic	0.281	7	9	15
3:154294697:C:CGTTAA	rs141871670	CGTTAA	C	0.1074	NA	NA	NA	0.977876	3:154294388:A/G	18	RP11-656A15.1	intergenic	3.032	NA	9	15
3:154294903:C:T	3:154294903:A/G	T	C	0.1074	3.43642e-05	0.349894	0.0844658	0.977876	3:154294388:A/G	18	RP11-656A15.1	intergenic	4.577	5	9	15
3:154295002:G:GAAAAAAAGAAAA	3:154295002:G:GAAAAAAAGAAAA	G	GAAAAAAAGAAAA	0.1074	NA	NA	NA	0.977876	3:154294388:A/G	18	RP11-656A15.1	intergenic	1.189	NA	9	15
3:154295002:G:GAAAAAGAAA	3:154295002:G:GAAAAAGAAA	G	GAAAAAGAAA	0.01491	NA	NA	NA	0.977876	3:154294388:A/G	18	RP11-656A15.1	NA	1.128	NA	9	15
3:154295303:A:G	3:154295303:A/G	A	G	0.1074	2.81806e-05	0.354093	0.0845558	0.977876	3:154294388:A/G	18	RP11-656A15.1	intergenic	3.343	6	9	15
3:154295410:C:T	3:154295410:A/G	C	T	0.1074	2.82645e-05	0.354063	0.0845623	0.977876	3:154294388:A/G	18	RP11-656A15.1	intergenic	7.086	7	9	15
3:154295433:G:T	3:154295433:A/C	G	T	0.1074	3.4923e-05	0.349712	0.0844975	0.977876	3:154294388:A/G	18	RP11-656A15.1	intergenic	8.054	6	9	15
3:154295581:A:G	3:154295581:A/G	A	G	0.1074	3.50608e-05	0.349672	0.0845062	0.977876	3:154294388:A/G	18	RP11-656A15.1	intergenic	5.026	7	9	15
3:154295650:A:G	3:154295650:A/G	A	G	0.1074	2.84738e-05	0.35398	0.0845763	0.977876	3:154294388:A/G	18	RP11-656A15.1	intergenic	0.177	7	9	15
3:154295718:C:T	3:154295718:A/G	T	C	0.1064	3.72332e-05	0.350063	0.0848842	0.966896	3:154294388:A/G	18	RP11-656A15.1	intergenic	1.663	6	9	15
3:154295736:C:T	3:154295736:A/G	C	T	0.1223	0.000672306	0.271099	0.0797201	0.813963	3:154294388:A/G	18	RP11-656A15.1	intergenic	0.266	7	9	15
3:154295856:C:T	3:154295856:A/G	T	C	0.1074	3.51724e-05	0.349706	0.0845294	0.977876	3:154294388:A/G	18	RP11-656A15.1	intergenic	3.366	6	9	15
3:154295977:A:T	3:154295977:A/T	A	T	0.1074	3.54474e-05	0.349559	0.0845305	0.977876	3:154294388:A/G	18	RP11-656A15.1	intergenic	3.423	7	9	15
3:154296990:C:CAA	rs60079932	C	CAA	0.1233	NA	NA	NA	0.824734	3:154294388:A/G	18	RP11-656A15.1	intergenic	1.968	NA	9	15
3:154297368:A:C	3:154297368:A/C	C	A	0.1083	3.43066e-05	0.350559	0.0846189	0.966852	3:154294388:A/G	18	RP11-656A15.1	intergenic	2.127	6	9	15
3:154297547:A:G	3:154297547:A/G	A	G	0.1074	3.71892e-05	0.349013	0.0846243	0.977876	3:154294388:A/G	18	RP11-656A15.1	intergenic	9.977	6	9	15
3:154297587:A:G	3:154297587:A/G	G	A	0.1074	3.71983e-05	0.349018	0.0846266	0.977876	3:154294388:A/G	18	RP11-656A15.1	intergenic	7.23	7	9	15
3:154298307:C:T	3:154298307:A/G	T	C	0.1243	0.000622807	0.272153	0.0795427	0.816393	3:154294388:A/G	18	RP11-656A15.1	intergenic	1.371	6	15	15
3:154298799:A:G	3:154298799:A/G	A	G	0.09543	0.00990006	0.246919	0.0957309	0.80718	3:154294388:A/G	18	RP11-656A15.1	intergenic	2.427	6	9	15
3:154299809:C:T	3:154299809:A/G	T	C	0.09443	0.011654	0.24175	0.0958391	0.817983	3:154294388:A/G	18	RP11-656A15.1	intergenic	1.512	7	9	15
3:154301325:C:T	3:154301325:A/G	C	T	0.09443	0.0143749	0.235247	0.0961071	0.817983	3:154294388:A/G	18	RP11-656A15.1	intergenic	1.057	5	9	15
3:154301888:C:CA	rs534815328	C	CA	0.1044	NA	NA	NA	0.718886	3:154294388:A/G	18	RP11-656A15.1	intergenic	2.056	NA	7	15
3:154302276:A:AT	3:154302276:A:AT	A	AT	0.1113	NA	NA	NA	0.659702	3:154294388:A/G	18	RP11-656A15.1	intergenic	0.654	NA	5	15
3:154304037:C:T	3:154304037:A/G	C	T	0.09443	0.0270839	0.214132	0.0968785	0.817983	3:154294388:A/G	18	RP11-656A15.1	intergenic	2.788	7	5	15
3:154305151:C:G	3:154305151:C/G	G	C	0.09443	0.0332683	0.205635	0.0965951	0.817983	3:154294388:A/G	18	RP11-656A15.1	intergenic	0.666	6	5	15
3:154309977:C:T	3:154309977:A/G	C	T	0.09443	0.0369826	0.202088	0.0968801	0.817983	3:154294388:A/G	18	RP11-656A15.1	intergenic	14.12	7	14	15
3:154311620:A:C	3:154311620:A/C	C	A	0.09443	0.037542	0.201477	0.0968722	0.817983	3:154294388:A/G	18	RP11-656A15.1	intergenic	1.939	7	14	15
3:154312961:C:T	3:154312961:A/G	T	C	0.09443	0.0378953	0.201027	0.0968344	0.817983	3:154294388:A/G	18	RP11-656A15.1	intergenic	0.212	7	14	15
3:154315837:C:T	3:154315837:A/G	C	T	0.09443	0.0390194	0.199903	0.0968531	0.817983	3:154294388:A/G	18	CTD-2501O3.2	intergenic	1.387	5	2	15
3:154316986:A:G	3:154316986:A/G	G	A	0.09443	0.0324341	0.20678	0.0966705	0.817983	3:154294388:A/G	18	CTD-2501O3.2	intergenic	1.834	6	9	15
3:154323832:C:G	3:154323832:C/G	C	G	0.09443	0.0310231	0.208716	0.0967721	0.817983	3:154294388:A/G	18	CTD-2501O3.2	intergenic	0.776	7	14	15

3:154324904:A:T	3:154324904:A/T	A	T	0.09443	0.0367937	0.202568	0.0970131	0.817983	3:154294388:A/G	18	CTD-2501O3.2	intergenic	1.035	7	14	15
3:154325046:C:T	3:154325046:A/G	C	T	0.09443	0.0308699	0.208921	0.0967792	0.817983	3:154294388:A/G	18	CTD-2501O3.2	intergenic	0.012	7	14	15
3:154326665:G:T	3:154326665:A/C	T	G	0.09543	0.0285233	0.210745	0.0962302	0.80718	3:154294388:A/G	18	CTD-2501O3.2	intergenic	0.525	5	14	15
3:154332053:C:T	3:154332053:A/G	C	T	0.09443	0.0358595	0.203629	0.0970348	0.817983	3:154294388:A/G	18	CTD-2501O3.2	intergenic	3.931	5	7	15
3:154332595:A:G	3:154332595:A/G	G	A	0.09443	0.0299979	0.210063	0.0967981	0.817983	3:154294388:A/G	18	CTD-2501O3.2	intergenic	0.155	3b	9	15
3:154334724:G:T	3:154334724:A/C	T	G	0.09443	0.0297535	0.210371	0.0967954	0.817983	3:154294388:A/G	18	CTD-2501O3.2	intergenic	1.059	6	9	15
3:154335034:C:T	3:154335034:A/G	C	T	0.09443	0.0297551	0.210368	0.0967948	0.817983	3:154294388:A/G	18	CTD-2501O3.2	intergenic	2.25	6	9	15
3:154335252:A:G	3:154335252:A/G	A	G	0.09443	0.0296798	0.210464	0.0967945	0.817983	3:154294388:A/G	18	CTD-2501O3.2	intergenic	0.176	7	9	15
3:154335534:C:T	3:154335534:A/G	T	C	0.09443	0.0390509	0.199872	0.0968538	0.817983	3:154294388:A/G	18	CTD-2501O3.2	intergenic	7.016	6	9	15
3:154335655:C:CA	rs36072235	CA	C	0.09443	NA	NA	NA	0.817983	3:154294388:A/G	18	CTD-2501O3.2	intergenic	10.52	NA	9	15
3:154337156:A:G	3:154337156:A/G	G	A	0.09443	0.0295221	0.210657	0.0967897	0.817983	3:154294388:A/G	18	CTD-2501O3.2	intergenic	0.68	7	9	15
3:154337189:C:G	3:154337189:C/G	G	C	0.09443	0.0295176	0.210665	0.0967904	0.817983	3:154294388:A/G	18	CTD-2501O3.2	intergenic	0.104	7	9	15
3:154337459:C:T	3:154337459:A/G	T	C	0.09443	0.0294575	0.210739	0.0967889	0.817983	3:154294388:A/G	18	CTD-2501O3.2	intergenic	0.526	5	9	15
3:154337797:A:C	3:154337797:A/C	A	C	0.09443	0.0294607	0.210733	0.0967879	0.817983	3:154294388:A/G	18	CTD-2501O3.2	intergenic	0.298	7	9	15
3:154337851:C:T	3:154337851:A/G	C	T	0.09443	0.0294247	0.210778	0.0967868	0.817983	3:154294388:A/G	18	CTD-2501O3.2	intergenic	0.767	7	9	15
3:154338149:C:T	3:154338149:A/G	T	C	0.09443	0.0294371	0.21076	0.0967862	0.817983	3:154294388:A/G	18	CTD-2501O3.2	intergenic	0.681	6	9	15
3:154338289:C:T	3:154338289:A/G	T	C	0.09443	0.0294177	0.210784	0.0967858	0.817983	3:154294388:A/G	18	CTD-2501O3.2	intergenic	0.659	7	9	15
3:154338299:A:G	3:154338299:A/G	A	G	0.09443	0.0293861	0.210824	0.0967853	0.817983	3:154294388:A/G	18	CTD-2501O3.2	intergenic	0.591	7	9	15
3:154338463:C:T	3:154338463:A/G	T	C	0.09443	0.0278334	0.212467	0.0965922	0.817983	3:154294388:A/G	18	CTD-2501O3.2	intergenic	0.559	7	9	15
3:154338565:C:T	3:154338565:A/G	T	C	0.09443	0.0293542	0.210864	0.0967842	0.817983	3:154294388:A/G	18	CTD-2501O3.2	intergenic	0.788	6	9	15
3:154338659:A:G	3:154338659:A/G	A	G	0.09443	0.0326213	0.207092	0.0969209	0.817983	3:154294388:A/G	18	CTD-2501O3.2	intergenic	0.428	7	9	15
3:154339009:A:G	3:154339009:A/G	G	A	0.09443	0.0293431	0.210875	0.0967827	0.817983	3:154294388:A/G	18	CTD-2501O3.2	intergenic	0.573	7	14	15
3:154339719:C:G	3:154339719:C/G	C	G	0.1203	0.0149302	0.208853	0.085804	0.610634	3:154294388:A/G	18	CTD-2501O3.2	intergenic	0.201	6	14	15
3:154339843:A:G	3:154339843:A/G	G	A	0.09443	0.0292589	0.210976	0.0967787	0.817983	3:154294388:A/G	18	CTD-2501O3.2	intergenic	0.901	6	14	15
3:154339861:C:G	3:154339861:C/G	C	G	0.09443	0.02923	0.211012	0.0967781	0.817983	3:154294388:A/G	18	CTD-2501O3.2	intergenic	0.347	7	14	15
3:154340509:C:T	3:154340509:A/G	T	C	0.09543	0.031616	0.207382	0.0964916	0.80718	3:154294388:A/G	18	CTD-2501O3.2	intergenic	0.465	7	14	15
3:154341000:C:T	3:154341000:A/G	T	C	0.09443	0.0291602	0.21109	0.0967721	0.817983	3:154294388:A/G	18	CTD-2501O3.2	intergenic	0.262	6	14	15
3:154341963:A:G	3:154341963:A/G	G	A	0.09443	0.0290364	0.211239	0.0967656	0.817983	3:154294388:A/G	18	CTD-2501O3.2	intergenic	0.601	7	14	15
3:154344110:C:T	3:154344110:A/G	C	T	0.09443	0.0289217	0.211354	0.0967493	0.817983	3:154294388:A/G	18	CTD-2501O3.2	intergenic	4.06	7	9	15
3:154344256:C:G	3:154344256:C/G	G	C	0.09443	0.0289173	0.211358	0.0967484	0.817983	3:154294388:A/G	18	CTD-2501O3.2	intergenic	1.185	7	9	15
3:154344309:C:T	3:154344309:A/G	T	C	0.09443	0.0289306	0.21134	0.0967483	0.817983	3:154294388:A/G	18	CTD-2501O3.2	intergenic	2.397	7	9	15
3:154345917:G:GA	rs569014933	G	GA	0.09443	NA	NA	NA	0.817983	3:154294388:A/G	18	CTD-2501O3.2	intergenic	7.561	NA	9	15
3:154345944:A:AC	rs113119506	A	AC	0.09443	NA	NA	NA	0.817983	3:154294388:A/G	18	CTD-2501O3.2	intergenic	1.046	NA	9	15
3:154346137:C:CAT	rs113811665	CAT	C	0.09443	NA	NA	NA	0.817983	3:154294388:A/G	18	CTD-2501O3.2	intergenic	1.225	NA	9	15
3:154346234:C:T	3:154346234:A/G	C	T	0.09443	0.0287624	0.211524	0.0967308	0.817983	3:154294388:A/G	18	CTD-2501O3.2	intergenic	2.446	7	9	15
3:154346326:C:T	3:154346326:A/G	T	C	0.09543	0.0275488	0.21288	0.0966033	0.80718	3:154294388:A/G	18	CTD-2501O3.2	intergenic	0.215	6	9	15
3:154348546:T:TTTA	rs140927183	T	TTTA	0.09543	NA	NA	NA	0.80718	3:154294388:A/G	18	CTD-2501O3.2	intergenic	5.377	NA	14	15
3:154348693:C:T	3:154348693:A/G	C	T	0.09543	0.0272959	0.213165	0.0965743	0.80718	3:154294388:A/G	18	CTD-2501O3.2	intergenic	1.749	6	14	15
3:154348746:A:G	3:154348746:A/G	A	G	0.09543	0.0273158	0.213136	0.0965738	0.80718	3:154294388:A/G	18	CTD-2501O3.2	intergenic	0.974	6	14	15
3:154351032:C:T	3:154351032:A/G	T	C	0.09543	0.0317517	0.206916	0.0963509	0.80718	3:154294388:A/G	18	CTD-2501O3.2	intergenic	4.668	6	9	15
3:154351759:G:T	3:154351759:A/C	G	T	0.09543	0.0263987	0.212696	0.0957961	0.80718	3:154294388:A/G	18	CTD-2501O3.2	intergenic	3.871	6	9	15
3:154352631:A:G	3:154352631:A/G	G	A	0.09543	0.0306499	0.206317	0.0954471	0.80718	3:154294388:A/G	18	CTD-2501O3.2	intergenic	8.03	7	14	15
3:154352754:A:G	3:154352754:A/G	G	A	0.09543	0.0259389	0.211812	0.0951054	0.80718	3:154294388:A/G	18	CTD-2501O3.2	intergenic	2.415	7	14	15

3:154353017:A:G	3:154353017:A/G	A	G	0.09543	0.0348025	0.199031	0.0942986	0.80718	3:154294388:A/G	18	CTD-2501O3.2	intergenic	8.828	7	14	15
3:154353330:C:T	3:154353330:A/G	C	T	0.09543	0.025905	0.211355	0.0948784	0.80718	3:154294388:A/G	18	CTD-2501O3.2	intergenic	4.017	7	14	15
3:154353985:C:G	3:154353985:C/G	G	C	0.09543	0.0258541	0.211462	0.0948941	0.80718	3:154294388:A/G	18	CTD-2501O3.2	intergenic	0.065	6	7	15
3:154354109:A:T	3:154354109:A/T	A	T	0.09543	0.0258593	0.211462	0.0948972	0.80718	3:154294388:A/G	18	CTD-2501O3.2	intergenic	4.726	7	7	15
3:154354690:A:G	3:154354690:A/G	A	G	0.09543	0.0258134	0.211556	0.0949103	0.80718	3:154294388:A/G	18	CTD-2501O3.2	intergenic	13.23	7	1	15
3:154354998:G:T	3:154354998:A/C	T	G	0.09543	0.0257964	0.211595	0.0949173	0.80718	3:154294388:A/G	18	CTD-2501O3.2	intergenic	0.087	7	7	15
3:154355658:C:T	3:154355658:A/G	T	C	0.09543	0.0257335	0.211723	0.094934	0.80718	3:154294388:A/G	18	CTD-2501O3.2	intergenic	1.159	7	9	15
3:154355814:A:G	3:154355814:A/G	A	G	0.09543	0.0257553	0.2117	0.0949375	0.80718	3:154294388:A/G	18	CTD-2501O3.2	intergenic	1.763	7	9	15
3:154356840:A:G	3:154356840:A/G	A	G	0.09543	0.0301601	0.206397	0.0952022	0.80718	3:154294388:A/G	18	CTD-2501O3.2	intergenic	6.325	6	9	15
3:154359954:A:ATCT	rs148944836	ATCT	A	0.09543	NA	NA	NA	0.80718	3:154294388:A/G	18	CTD-2501O3.2	intergenic	4.307	NA	7	15
3:154361091:A:C	3:154361091:A/C	A	C	0.09443	0.0263185	0.211474	0.0951951	0.817983	3:154294388:A/G	18	CTD-2501O3.2	intergenic	0.083	2b	7	15
3:154361137:C:T	3:154361137:A/G	T	C	0.09443	0.0263135	0.211484	0.0951964	0.817983	3:154294388:A/G	18	CTD-2501O3.2	intergenic	1.587	4	7	15
3:154362973:C:T	3:154362973:A/G	C	T	0.09443	0.0304237	0.206716	0.0955018	0.817983	3:154294388:A/G	18	CTD-2501O3.2	intergenic	10.79	6	9	15
3:154364784:C:T	3:154364784:A/G	C	T	0.09443	0.0320699	0.2048	0.095543	0.817983	3:154294388:A/G	18	CTD-2501O3.2	intergenic	3.849	5	14	15
3:154368058:A:G	3:154368058:A/G	G	A	0.09443	0.0299864	0.207569	0.0956419	0.817983	3:154294388:A/G	18	CTD-2501O3.2	intergenic	3.398	7	9	15
3:154371064:C:T	3:154371064:A/G	T	C	0.09443	0.0308103	0.207636	0.0961495	0.817983	3:154294388:A/G	18	CTD-2501O3.2	upstream	3.792	7	14	15
4:24850083:C:T	4:24850083:A/G	C	T	0.02087	3.36899e-05	-0.536803	0.129445	1	4:24850083:A/G	19	CCDC149	intronic	12.05	6	4	15
4:24875910:A:G	4:24875910:A/G	A	G	0.02087	4.11351e-05	-0.530039	0.129246	1	4:24850083:A/G	19	CCDC149	intronic	5.916	6	4	5
4:69408461:A:C	4:69408461:A/C	A	C	0.02783	2.33418e-06	-1.18388	0.250709	1	4:69408461:A/C	20	UGT2B17	intronic	2.504	6	4	15
4:69443377:G:T	4:69443377:A/C	T	G	0.01789	0.000220251	-1.02887	0.27848	0.609406	4:69408461:A/C	20	UGT2B17	intergenic	3.325	5	5	15
4:90235778:C:T	4:90235778:A/G	C	T	0.4453	0.000584246	-0.174152	0.050642	0.657068	4:90271485:A/G	21	GPRIN3	intergenic	2.697	5	5	15
4:90255150:A:G	4:90255150:A/G	G	A	0.4006	3.99921e-05	-0.194845	0.0474359	0.965565	4:90271485:A/G	21	GPRIN3	intergenic	8.425	7	5	15
4:90255167:A:G	4:90255167:A/G	A	G	0.2952	0.00110854	-0.159571	0.0489267	0.613363	4:90271485:A/G	21	GPRIN3	intergenic	4.641	7	5	15
4:90256050:A:AGTGCCAG	rs62958160	A	AGTGCCAG	0.2952	NA	NA	NA	0.613363	4:90271485:A/G	21	GPRIN3	intergenic	2.364	NA	5	15
4:90256211:C:T	4:90256211:A/G	T	C	0.4016	4.68279e-05	-0.191888	0.0471363	0.961303	4:90271485:A/G	21	GPRIN3	intergenic	0.164	7	5	15
4:90256463:G:T	4:90256463:A/C	T	G	0.2962	0.00137911	-0.156206	0.0488298	0.61538	4:90271485:A/G	21	GPRIN3	intergenic	2.144	6	5	15
4:90257775:CA:CAA	rs559262968	CAA	CA	0.2982	NA	NA	NA	0.605146	4:90271485:A/G	21	GPRIN3	intergenic	NA	NA	5	15
4:90258588:G:T	4:90258588:A/C	G	T	0.4016	2.61493e-05	-0.198631	0.0472407	0.978411	4:90271485:A/G	21	GPRIN3	intergenic	2.761	7	5	15
4:90259940:T:TG	rs5860166	TG	T	0.2962	NA	NA	NA	0.621313	4:90271485:A/G	21	GPRIN3	intergenic	2.381	NA	5	15
4:90262643:A:T	4:90262643:A/T	A	T	0.2962	0.00086779	-0.162824	0.0488929	0.621313	4:90271485:A/G	21	GPRIN3	intergenic	0.388	7	14	15
4:90262855:G:GTTAATTAATTAA	rs143095890	GTTAATTAATTAA	G	0.2982	NA	NA	NA	0.612285	4:90271485:A/G	21	GPRIN3	intergenic	6.756	NA	14	15
4:90262909:A:G	4:90262909:A/G	G	A	0.2962	0.000868812	-0.162814	0.0488949	0.621313	4:90271485:A/G	21	GPRIN3	intergenic	0.456	7	14	15
4:90263021:C:T	4:90263021:A/G	T	C	0.337	0.000122616	-0.184914	0.0481444	0.747596	4:90271485:A/G	21	GPRIN3	intergenic	1.602	7	1	15
4:90263893:A:G	4:90263893:A/G	G	A	0.2972	0.00152615	-0.154939	0.0488818	0.63065	4:90271485:A/G	21	GPRIN3	intergenic	1.423	7	5	15
4:90264361:C:T	4:90264361:A/G	T	C	0.2952	0.00400045	-0.141858	0.0492885	0.625294	4:90271485:A/G	21	GPRIN3	intergenic	1.324	NA	5	15
4:90264609:G:T	4:90264609:A/C	T	G	0.2972	0.0017958	-0.152645	0.0488921	0.63065	4:90271485:A/G	21	GPRIN3	intergenic	2.184	6	5	15
4:90264912:C:CGTT	rs35688314	C	CGTT	0.4414	NA	NA	NA	0.792807	4:90271485:A/G	21	GPRIN3	intergenic	6.893	NA	5	15
4:90266958:C:T	4:90266958:A/G	T	C	0.4831	8.81005e-05	-0.195645	0.0498942	0.723452	4:90271485:A/G	21	GPRIN3	intergenic	3.976	7	7	15
4:90267231:A:G	4:90267231:A/G	G	A	0.3986	1.89848e-05	-0.203813	0.0476587	0.99132	4:90271485:A/G	21	GPRIN3	intergenic	11.14	7	5	15
4:90267380:A:G	4:90267380:A/G	A	G	0.2942	0.000788585	-0.165824	0.0494002	0.646426	4:90271485:A/G	21	GPRIN3	intergenic	3.011	7	5	15
4:90267733:AT:ATT	rs573081103	ATT	AT	0.4891	NA	NA	NA	0.692429	4:90271485:A/G	21	GPRIN3	intergenic	NA	NA	5	15

4:90267733:A:AT	rs67523886	AT	A	0.06561	NA	NA	NA	0.692429	4:90271485:A/G	21	GPRIN3	intergenic	2.085	NA	5	15
4:90268041:G:T	4:90268041:A/C	G	T	0.2942	0.000788307	-0.165859	0.0494089	0.646426	4:90271485:A/G	21	GPRIN3	intergenic	0.909	6	5	15
4:90269335:A:G	4:90269335:A/G	G	A	0.2942	0.00101989	-0.16236	0.049425	0.646426	4:90271485:A/G	21	GPRIN3	intergenic	6.111	5	7	9
4:90270991:C:T	4:90270991:A/G	C	T	0.2922	0.000724214	-0.16726	0.0494814	0.64114	4:90271485:A/G	21	GPRIN3	intergenic	0.126	6	9	15
4:90271485:C:T	4:90271485:A/G	C	T	0.3966	1.87583e-05	-0.205411	0.0480024	1	4:90271485:A/G	21	GPRIN3	intergenic	1.34	7	9	15
4:90272120:A:G	4:90272120:A/G	A	G	0.2932	0.00100316	-0.162867	0.0495087	0.650615	4:90271485:A/G	21	GPRIN3	intergenic	0.529	NA	9	15
4:90273016:C:T	4:90273016:A/G	T	C	0.2932	0.000759604	-0.16666	0.0494962	0.637015	4:90271485:A/G	21	GPRIN3	intergenic	5.189	7	14	15
4:90273098:A:C	4:90273098:A/C	C	A	0.4831	4.95615e-05	-0.203821	0.050231	0.723452	4:90271485:A/G	21	GPRIN3	intergenic	2.041	6	14	15
4:90275862:A:G	4:90275862:A/G	A	G	0.3897	4.70842e-05	-0.197324	0.048487	0.952669	4:90271485:A/G	21	GPRIN3	intergenic	0.418	7	14	15
4:90276397:C:CACACACAT	rs111616094	CACACACAT	C	0.3757	NA	NA	NA	0.871847	4:90271485:A/G	21	GPRIN3	intergenic	0.873	NA	14	15
4:90277638:A:G	4:90277638:A/G	A	G	0.3966	6.3031e-05	-0.195109	0.0487636	0.914037	4:90271485:A/G	21	GPRIN3	intergenic	8.651	6	5	15
4:100639988:A:C	4:100639988:A/C	C	A	0.02982	0.000345772	0.374735	0.104723	0.798811	4:100663948:A/G	22	RP11-766F14.2	intergenic	4.943	6	5	15
4:100641031:C:T	4:100641031:A/G	C	T	0.0338	0.00161659	0.28866	0.0915537	0.695874	4:100663948:A/G	22	RP11-766F14.2	intergenic	3.854	5	2	15
4:100641968:C:T	4:100641968:A/G	C	T	0.02982	0.000363941	0.37301	0.104633	0.798811	4:100663948:A/G	22	RP11-766F14.2	intergenic	2.217	6	5	15
4:100642359:A:G	4:100642359:A/G	G	A	0.02982	0.000419024	0.369228	0.104662	0.798811	4:100663948:A/G	22	RP11-766F14.2	intergenic	4.197	7	5	15
4:100651570:C:T	4:100651570:A/G	C	T	0.02982	0.000344465	0.375938	0.10503	0.798811	4:100663948:A/G	22	RP11-766F14.2	intergenic	8.579	6	9	15
4:100652178:A:G	4:100652178:A/G	A	G	0.02982	0.000366379	0.374265	0.105037	0.798811	4:100663948:A/G	22	RP11-766F14.2	intergenic	0.019	NA	9	15
4:100652334:C:T	4:100652334:A/G	T	C	0.02982	0.000366328	0.374295	0.105044	0.798811	4:100663948:A/G	22	RP11-766F14.2	intergenic	1.652	7	9	15
4:100654685:A:G	4:100654685:A/G	A	G	0.02982	8.83013e-05	0.428011	0.109168	0.798811	4:100663948:A/G	22	RP11-766F14.2	intergenic	10.48	5	7	15
4:100654835:C:T	4:100654835:A/G	T	C	0.02982	0.00033944	0.377832	0.105446	0.798811	4:100663948:A/G	22	RP11-766F14.2	intergenic	5.923	NA	7	15
4:100655610:C:T	4:100655610:A/G	C	T	0.02982	0.000226283	0.386709	0.104865	0.798811	4:100663948:A/G	22	RP11-766F14.2	intergenic	2.393	6	9	15
4:100657300:A:G	4:100657300:A/G	G	A	0.03082	0.000199008	0.39006	0.104847	0.770586	4:100663948:A/G	22	DAPP1	intergenic	3.919	5	7	15
4:100657530:A:G	4:100657530:A/G	G	A	0.02982	0.000257905	0.38581	0.105578	0.798811	4:100663948:A/G	22	DAPP1	intergenic	4.54	NA	7	15
4:100658135:A:G	4:100658135:A/G	A	G	0.0338	0.00127958	0.32627	0.10131	0.695874	4:100663948:A/G	22	DAPP1	intergenic	0.297	7	7	15
4:100658145:A:G	4:100658145:A/G	G	A	0.02982	0.000251482	0.386649	0.10562	0.798811	4:100663948:A/G	22	DAPP1	intergenic	2.836	7	7	15
4:100658372:A:C	4:100658372:A/C	A	C	0.02982	0.00021862	0.389076	0.105256	0.798811	4:100663948:A/G	22	DAPP1	intergenic	0.632	6	14	15
4:100658553:C:T	4:100658553:A/G	C	T	0.02982	0.000185827	0.392197	0.104934	0.798811	4:100663948:A/G	22	DAPP1	intergenic	2.775	NA	14	15
4:100659509:C:T	4:100659509:A/G	T	C	0.02982	0.000278319	0.383433	0.105493	0.798811	4:100663948:A/G	22	DAPP1	intergenic	0.287	6	14	15
4:100661092:C:T	4:100661092:A/G	C	T	0.02982	0.000329457	0.379727	0.105745	0.798811	4:100663948:A/G	22	DAPP1	intergenic	0.453	6	9	15
4:100661309:A:G	4:100661309:A/G	A	G	0.02982	0.000295266	0.383782	0.106034	0.798811	4:100663948:A/G	22	DAPP1	intergenic	1.087	7	9	15
4:100662154:A:C	4:100662154:A/C	A	C	0.03181	0.000704856	0.37189	0.109778	0.855684	4:100663948:A/G	22	DAPP1	intergenic	1.221	6	9	15
4:100663822:A:G	4:100663822:A/G	A	G	0.0328	1.46306e-05	0.505706	0.116679	0.884301	4:100663948:A/G	22	DAPP1	intergenic	5.599	7	9	15
4:100663948:A:G	4:100663948:A/G	G	A	0.03678	1.70524e-06	0.542508	0.113364	1	4:100663948:A/G	22	DAPP1	intergenic	3.335	7	9	15
4:100663968:A:G	4:100663968:A/G	G	A	0.03678	5.15387e-06	0.527902	0.115808	1	4:100663948:A/G	22	DAPP1	intergenic	5.375	6	9	15
4:100663984:C:T	4:100663984:A/G	T	C	0.03678	5.15772e-06	0.527886	0.115809	1	4:100663948:A/G	22	DAPP1	intergenic	1.342	7	9	15
4:100664582:C:G	4:100664582:C/G	G	C	0.03678	2.43626e-06	0.532664	0.113011	1	4:100663948:A/G	22	DAPP1	intergenic	2.496	6	9	15
4:100665907:C:T	4:100665907:A/G	C	T	0.03976	0.00093095	0.406329	0.122736	0.669004	4:100663948:A/G	22	DAPP1	intergenic	2.376	7	14	15
4:100666364:C:T	4:100666364:A/G	T	C	0.03479	0.00503741	0.350254	0.124884	0.625575	4:100663948:A/G	22	DAPP1	intergenic	2.773	7	14	15
4:100667215:C:T	4:100667215:A/G	T	C	0.03479	0.00313759	0.370161	0.125311	0.625575	4:100663948:A/G	22	DAPP1	intergenic	0.826	7	14	15
4:100667705:C:T	4:100667705:A/G	C	T	0.03479	0.00314955	0.370105	0.125342	0.625575	4:100663948:A/G	22	DAPP1	intergenic	4.041	NA	14	15
4:100667816:A:G	4:100667816:A/G	G	A	0.03479	0.00315965	0.370035	0.125361	0.625575	4:100663948:A/G	22	DAPP1	intergenic	0.109	5	14	15
4:100669217:C:CTT	rs34579737	CTT	C	0.03579	NA	NA	NA	0.606111	4:100663948:A/G	22	DAPP1	intergenic	1.945	NA	5	15

4:100669401:A:G	4:100669401:A/G	A	G	0.03479	0.00392671	0.365643	0.126784	0.625575	4:100663948:A/G	22	DAPP1	intergenic	0.908	7	5	15
4:100670709:C:G	4:100670709:C/G	C	G	0.03479	0.00395958	0.365474	0.12684	0.625575	4:100663948:A/G	22	DAPP1	intergenic	2.171	5	5	15
4:100671474:C:T	4:100671474:A/G	C	T	0.03479	0.00404715	0.365074	0.127005	0.625575	4:100663948:A/G	22	DAPP1	intergenic	2.678	NA	5	15
4:100672429:A:G	4:100672429:A/G	A	G	0.03479	0.00406999	0.365377	0.12719	0.625575	4:100663948:A/G	22	DAPP1	intergenic	0.853	5	5	15
4:100673407:A:G	4:100673407:A/G	G	A	0.03479	0.00466648	0.360701	0.127492	0.625575	4:100663948:A/G	22	DAPP1	intergenic	1.965	7	5	15
4:100673897:C:G	4:100673897:C/G	C	G	0.03479	0.00466621	0.361384	0.127733	0.625575	4:100663948:A/G	22	DAPP1	intergenic	1.676	7	5	15
4:100674186:C:T	4:100674186:A/G	T	C	0.03479	0.00468601	0.361649	0.127888	0.625575	4:100663948:A/G	22	DAPP1	intergenic	10.68	NA	5	15
4:125390164:G:T	4:125390164:A/C	G	T	0.4354	0.000626902	0.174753	0.0511015	0.659628	4:125419635:A/C	23	TECRP2	intergenic	1.691	6	5	15
4:125391249:A:G	4:125391249:A/G	G	A	0.4354	0.000625534	0.174784	0.0511017	0.659628	4:125419635:A/C	23	TECRP2	intergenic	0.701	5	5	15
4:125394958:A:T	4:125394958:A/T	T	A	0.4344	0.000692839	0.173784	0.051227	0.65582	4:125419635:A/C	23	TECRP2	intergenic	3.269	7	5	15
4:125395250:A:G	4:125395250:A/G	A	G	0.4344	0.000693125	0.173776	0.0512267	0.65582	4:125419635:A/C	23	TECRP2	intergenic	3.979	7	5	15
4:125396586:C:T	4:125396586:A/G	T	C	0.4364	0.000632841	0.174633	0.0511049	0.655955	4:125419635:A/C	23	TECRP2	intergenic	9.844	7	5	15
4:125396636:C:T	4:125396636:A/G	T	C	0.4374	0.000640849	0.1745	0.0511172	0.653506	4:125419635:A/C	23	TECRP2	intergenic	7.389	6	5	15
4:125396945:A:G	4:125396945:A/G	A	G	0.4324	0.000415051	0.182173	0.0516019	0.651889	4:125419635:A/C	23	TECRP2	intergenic	2.992	6	5	15
4:125397924:C:T	4:125397924:A/G	T	C	0.493	0.000116017	0.189064	0.049051	0.653233	4:125419635:A/C	23	TECRP2	intergenic	1.487	5	5	15
4:125397988:C:T	4:125397988:A/G	C	T	0.4324	0.00068589	0.174287	0.0513333	0.655727	4:125419635:A/C	23	TECRP2	intergenic	0.755	5	5	15
4:125398008:C:T	4:125398008:A/G	C	T	0.4334	0.000668577	0.17435	0.0512462	0.658329	4:125419635:A/C	23	TECRP2	intergenic	2.75	7	5	15
4:125399775:C:T	4:125399775:A/G	C	T	0.4344	0.000569998	0.175919	0.051057	0.66213	4:125419635:A/C	23	TECRP2	intergenic	4.211	4	5	15
4:125404239:C:T	4:125404239:A/G	C	T	0.2913	0.000422417	-0.20109	0.0570357	0.675689	4:125444404:A/G	23	RP11-93I21.3	intergenic	9.498	5	2	15
4:125404621:G:T	4:125404621:A/C	G	T	0.4354	0.00112274	0.166803	0.0512007	0.66594	4:125419635:A/C	23	RP11-93I21.3	intergenic	1.496	2b	2	15
4:125404706:A:G	4:125404706:A/G	A	G	0.4354	0.00135011	0.164598	0.051355	0.66594	4:125419635:A/C	23	RP11-93I21.3	intergenic	0.626	3a	2	15
4:125406335:A:G	4:125406335:A/G	G	A	0.4334	0.00134188	0.165138	0.0514952	0.664666	4:125419635:A/C	23	RP11-93I21.3	intergenic	1.043	4	5	15
4:125406642:A:T	4:125406642:A/T	A	T	0.4334	0.00153491	-0.16309	0.0514807	0.664666	4:125419635:A/C	23	RP11-93I21.3	intergenic	1.754	6	5	15
4:125410414:A:G	4:125410414:A/G	A	G	0.2813	0.000169192	-0.217708	0.0578849	0.834944	4:125444404:A/G	23	RP11-93I21.3	intergenic	0.278	7	5	15
4:125411237:C:T	4:125411237:A/G	T	C	0.3419	6.04596e-05	0.214387	0.0534496	0.842405	4:125444404:A/G	23	RP11-93I21.3	intergenic	0.66	7	5	15
4:125414429:C:CA	4:125414429:C:CA	CA	C	0.4473	NA	NA	NA	0.980797	4:125419635:A/C	23	RP11-93I21.3	intergenic	0.996	NA	5	15
4:125414429:C:CAA	4:125414429:C:CAA	C	CAA	0.000994	NA	NA	NA	0.980797	4:125419635:A/C	23	RP11-93I21.3	NA	0.97	NA	5	15
4:125414644:C:T	4:125414644:A/G	T	C	0.4543	8.80955e-05	0.186434	0.0475449	0.666752	4:125419635:A/C	23	RP11-93I21.3	intergenic	0.3	6	5	15
4:125414757:C:G	4:125414757:C/G	G	C	0.3072	0.000404738	-0.194012	0.0548525	0.733164	4:125444404:A/G	23	RP11-93I21.3	intergenic	2.52	7	5	15
4:125415149:A:G	4:125415149:A/G	A	G	0.2813	7.95434e-05	-0.225372	0.0571173	0.843228	4:125444404:A/G	23	RP11-93I21.3	intergenic	1.001	5	5	15
4:125415534:C:T	4:125415534:A/G	T	C	0.3419	1.49796e-05	0.227088	0.0524576	0.842405	4:125444404:A/G	23	RP11-93I21.3	intergenic	2.012	5	5	15
4:125415922:A:C	4:125415922:A/C	A	C	0.2813	9.41446e-05	-0.224558	0.0575023	0.834583	4:125444404:A/G	23	RP11-93I21.3	intergenic	2.363	6	5	15
4:125418343:G:T	4:125418343:A/C	T	G	0.2813	0.000100008	-0.223246	0.0573814	0.843228	4:125444404:A/G	23	RP11-93I21.3	intergenic	0.418	7	5	15
4:125418536:A:T	4:125418536:A/T	T	A	0.3419	2.22394e-05	0.223916	0.0527964	0.842405	4:125444404:A/G	23	RP11-93I21.3	intergenic	11.69	6	5	15
4:125418773:T:TA	rs56061866	T	TA	0.3817	NA	NA	NA	0.783583	4:125419635:A/C	23	RP11-93I21.3	intergenic	1.013	NA	5	15
4:125419177:C:G	4:125419177:C/G	G	C	0.2813	0.000104635	-0.222693	0.0574013	0.843228	4:125444404:A/G	23	RP11-93I21.3	intergenic	0.832	5	5	15
4:125419243:C:T	4:125419243:A/G	C	T	0.2813	0.000105017	-0.222647	0.0574024	0.843228	4:125444404:A/G	23	RP11-93I21.3	intergenic	4.459	5	5	15
4:125419479:G:T	4:125419479:A/C	G	T	0.338	2.28704e-05	0.223876	0.0528651	0.855722	4:125444404:A/G	23	RP11-93I21.3	intergenic	2.45	5	5	15
4:125419635:A:C	4:125419635:A/C	C	A	0.4423	4.2513e-06	0.225877	0.0491174	1	4:125419635:A/C	23	RP11-93I21.3	intergenic	7.473	5	5	15
4:125419666:C:T	4:125419666:A/G	C	T	0.4364	0.000261112	0.175965	0.048195	0.610863	4:125419635:A/C	23	RP11-93I21.3	intergenic	5.29	5	5	15
4:125422463:A:G	4:125422463:A/G	A	G	0.2823	0.000106642	-0.22243	0.0574019	0.846793	4:125444404:A/G	23	RP11-93I21.3	ncRNA_intronic	0.652	5	5	15

4:125423945:C:T	4:125423945:A/G	C	T	0.4463	6.17913e-06	0.223077	0.0493514	0.977099	4:125419635:A/C	23	RP11-93I21.3	ncRNA_intronic	0.787	7	5	15
4:125425570:G:T	4:125425570:A/C	G	T	0.2853	9.34012e-05	-0.222995	0.0570738	0.866578	4:125444404:A/G	23	RP11-93I21.3	ncRNA_intronic	1.954	6	5	15
4:125425698:C:T	4:125425698:A/G	T	C	0.2853	9.15886e-05	-0.22324	0.0570674	0.866578	4:125444404:A/G	23	RP11-93I21.3	ncRNA_intronic	5.665	5	5	15
4:125425867:C:T	4:125425867:A/G	T	C	0.2853	9.17571e-05	-0.223213	0.0570669	0.866578	4:125444404:A/G	23	RP11-93I21.3	ncRNA_intronic	1.944	5	5	15
4:125426113:C:T	4:125426113:A/G	C	T	0.2853	9.19079e-05	-0.223187	0.0570661	0.866578	4:125444404:A/G	23	RP11-93I21.3	ncRNA_intronic	1.635	6	5	15
4:125426468:A:G	4:125426468:A/G	G	A	0.2853	0.000129714	-0.218638	0.0571304	0.866578	4:125444404:A/G	23	RP11-93I21.3	ncRNA_intronic	0.422	5	5	15
4:125428678:C:G	4:125428678:C/G	C	G	0.4463	6.10978e-06	0.222095	0.0491082	0.977099	4:125419635:A/C	23	RP11-93I21.3	ncRNA_intronic	2.706	3a	5	15
4:125428768:A:G	4:125428768:A/G	G	A	0.2853	0.00016531	-0.215759	0.0572781	0.866578	4:125444404:A/G	23	RP11-93I21.3	ncRNA_intronic	4.287	5	5	15
4:125429497:A:G	4:125429497:A/G	A	G	0.2873	9.64268e-05	-0.222626	0.0570921	0.841092	4:125444404:A/G	23	RP11-93I21.3	ncRNA_intronic	0.213	5	2	15
4:125430262:A:T	4:125430262:A/T	A	T	0.2853	0.000132171	-0.218349	0.0571239	0.866578	4:125444404:A/G	23	RP11-93I21.3	ncRNA_intronic	2.045	6	5	15
4:125431455:A:G	4:125431455:A/G	A	G	0.4463	7.61547e-06	0.22095	0.0493663	0.977099	4:125419635:A/C	23	RP11-93I21.3	ncRNA_intronic	0.225	NA	5	15
4:125431543:A:C	4:125431543:A/C	C	A	0.2853	0.000136028	-0.217891	0.0571102	0.866578	4:125444404:A/G	23	RP11-93I21.3	ncRNA_intronic	4.044	7	5	15
4:125432031:C:T	4:125432031:A/G	T	C	0.2853	0.000136308	-0.217856	0.0571086	0.866578	4:125444404:A/G	23	RP11-93I21.3	ncRNA_intronic	0.067	6	5	15
4:125434572:T:TTTG	rs200066005	TTTG	T	0.02684	NA	NA	NA	0.921828	4:125419635:A/C	23	RP11-93I21.3	ncRNA_intronic	1.376	NA	5	15
4:125434572:TTTG:TTTGTTG	rs563558902	TTTGTTG	TTTG	0.4612	NA	NA	NA	0.921828	4:125419635:A/C	23	RP11-93I21.3	NA	NA	NA	5	15
4:125435196:TA:TAA	rs577847440	TAA	TA	0.4364	NA	NA	NA	0.915065	4:125419635:A/C	23	RP11-93I21.3	NA	NA	NA	5	15
4:125435196:T:TAA	rs566231881	TAA	T	0.005964	NA	NA	NA	0.915065	4:125419635:A/C	23	RP11-93I21.3	ncRNA_intronic	0.303	NA	5	15
4:125435781:C:T	4:125435781:A/G	T	C	0.4423	8.21494e-06	0.219097	0.0491304	0.961514	4:125419635:A/C	23	RP11-93I21.3	ncRNA_intronic	4.968	7	5	15
4:125436914:G:T	4:125436914:A/C	G	T	0.4463	6.98261e-06	0.220748	0.0491182	0.977099	4:125419635:A/C	23	RP11-93I21.3	ncRNA_intronic	2.485	NA	5	15
4:125438407:A:T	4:125438407:A/T	T	A	0.4453	6.89344e-06	0.220876	0.0491167	0.973146	4:125419635:A/C	23	RP11-93I21.3	ncRNA_intronic	4.471	7	5	15
4:125440118:C:T	4:125440118:A/G	T	C	0.2853	0.000145017	-0.216814	0.0570647	0.866578	4:125444404:A/G	23	RP11-93I21.3	ncRNA_intronic	3.18	7	7	15
4:125440131:A:G	4:125440131:A/G	A	G	0.2853	0.000145068	-0.216807	0.0570642	0.866578	4:125444404:A/G	23	RP11-93I21.3	ncRNA_intronic	0.716	6	7	15
4:125440622:T:TA	rs56379340	TA	T	0.2893	NA	NA	NA	0.848747	4:125444404:A/G	23	RP11-93I21.3	ncRNA_intronic	0.409	NA	7	15
4:125440801:C:T	4:125440801:A/G	T	C	0.2853	0.000152073	-0.216075	0.0570471	0.866578	4:125444404:A/G	23	RP11-93I21.3	ncRNA_intronic	15.6	5	5	15
4:125442616:G:T	4:125442616:A/C	G	T	0.4463	8.7622e-06	0.218448	0.0491373	0.977099	4:125419635:A/C	23	RP11-93I21.3	ncRNA_intronic	5.134	NA	14	15
4:125443757:C:T	4:125443757:A/G	T	C	0.2863	0.000160232	-0.215393	0.0570631	0.870231	4:125444404:A/G	23	RP11-93I21.3	ncRNA_intronic	1.894	7	14	15
4:125444404:A:G	4:125444404:A/G	G	A	0.3161	6.5751e-06	0.24643	0.0546773	1	4:125444404:A/G	23	RP11-93I21.3	ncRNA_intronic	1.65	NA	7	15
4:125446214:C:T	4:125446214:A/G	T	C	0.4463	8.78354e-06	0.218862	0.0492364	0.969328	4:125419635:A/C	23	RP11-93I21.3	ncRNA_intronic	0.524	6	1	15
4:125446780:A:G	4:125446780:A/G	A	G	0.4463	8.484e-06	0.218956	0.0491749	0.969328	4:125419635:A/C	23	RP11-93I21.3	ncRNA_intronic	2.813	NA	5	15
4:125448257:A:G	4:125448257:A/G	G	A	0.2893	9.64636e-05	0.220324	0.0565032	0.848327	4:125444404:A/G	23	RP11-93I21.3	ncRNA_intronic	0.426	4	7	15
4:125448288:C:T	4:125448288:A/G	T	C	0.4453	7.87204e-06	0.219544	0.04913	0.965376	4:125419635:A/C	23	RP11-93I21.3	ncRNA_intronic	6.345	5	7	15
4:125449003:A:G	4:125449003:A/G	A	G	0.4095	9.33019e-05	-0.195688	0.0500816	0.838064	4:125419635:A/C	23	RP11-93I21.3	ncRNA_intronic	0.71	7	5	15
4:125449107:A:ATATATAATTATAATT	rs151058873	ATATATAATTATAATT	A	0.3787	NA	NA	NA	0.732168	4:125419635:A/C	23	RP11-93I21.3	ncRNA_intronic	1.426	NA	5	15
4:125449506:A:C	4:125449506:A/C	A	C	0.4095	0.000106702	-0.193869	0.050033	0.838064	4:125419635:A/C	23	RP11-93I21.3	ncRNA_intronic	0.678	6	5	15
4:125449988:C:T	4:125449988:A/G	T	C	0.4145	9.97063e-05	-0.193977	0.049849	0.854669	4:125419635:A/C	23	RP11-93I21.3	ncRNA_intronic	1.49	7	5	15
4:125450369:A:T	4:125450369:A/T	A	T	0.4145	9.79486e-05	-0.194166	0.0498423	0.854669	4:125419635:A/C	23	RP11-93I21.3	ncRNA_intronic	3.68	5	5	15
4:125450960:C:T	4:125450960:A/G	C	T	0.4095	0.000101416	-0.194522	0.050042	0.830328	4:125419635:A/C	23	RP11-93I21.3	ncRNA_intronic	1.134	6	5	15
4:125451032:C:T	4:125451032:A/G	T	C	0.4145	9.27161e-05	-0.194723	0.0498153	0.854669	4:125419635:A/C	23	RP11-93I21.3	ncRNA_intronic	4.271	7	5	15
4:125451364:A:G	4:125451364:A/G	A	G	0.4095	9.08645e-05	-0.195387	0.049923	0.838064	4:125419635:A/C	23	RP11-93I21.3	ncRNA_intronic	1.492	5	2	15
4:125451962:A:G	4:125451962:A/G	A	G	0.2833	0.000141257	-0.216755	0.0569516	0.859033	4:125444404:A/G	23	RP11-93I21.3	ncRNA_intronic	4.502	4	2	15
4:125452066:C:G	4:125452066:C/G	G	C	0.4155	8.94433e-05	-0.195278	0.0498466	0.850482	4:125419635:A/C	23	RP11-93I21.3	ncRNA_intronic	2.381	4	2	15

4:125452368:C:T	4:125452368:A/G	C	T	0.4453	7.53719e-06	-0.220398	0.0492187	0.965376	4:125419635:A/C	23	RP11-93I21.3	ncRNA_intronic	1.98	NA	5	15
4:125452372:C:T	4:125452372:A/G	C	T	0.493	2.37467e-05	0.201427	0.0476593	0.757738	4:125419635:A/C	23	RP11-93I21.3	ncRNA_intronic	6.834	5	5	15
4:125453276:A:C	4:125453276:A/C	C	A	0.4105	9.81845e-05	-0.194669	0.0499788	0.833819	4:125419635:A/C	23	RP11-93I21.3	ncRNA_intronic	1.163	5	5	15
4:125453701:C:T	4:125453701:A/G	T	C	0.4165	8.38389e-05	-0.195967	0.0498241	0.846311	4:125419635:A/C	23	RP11-93I21.3	ncRNA_intronic	1.62	6	5	15
4:125454139:C:T	4:125454139:A/G	T	C	0.4105	9.86404e-05	-0.194636	0.0499845	0.833819	4:125419635:A/C	23	RP11-93I21.3	ncRNA_intronic	7.095	5	5	15
4:125454820:A:G	4:125454820:A/G	A	G	0.3091	0.000387715	-0.192142	0.0541502	0.74889	4:125444404:A/G	23	RP11-93I21.3	ncRNA_intronic	0.837	6	5	15
4:125455249:C:T	4:125455249:A/G	T	C	0.3141	0.00037851	-0.19194	0.0539971	0.738397	4:125444404:A/G	23	RP11-93I21.3	ncRNA_intronic	3.901	5	5	15
4:125455457:A:G	4:125455457:A/G	A	G	0.3091	0.000383943	-0.192427	0.0541913	0.74889	4:125444404:A/G	23	RP11-93I21.3	ncRNA_intronic	10.74	7	5	15
4:125456105:T:TC	rs143126739	TC	T	0.3091	NA	NA	NA	0.74889	4:125444404:A/G	23	RP11-93I21.3	ncRNA_intronic	10.48	NA	5	15
4:125456107:A:T	4:125456107:A/T	A	T	0.3091	0.000384363	-0.192409	0.0541906	0.74889	4:125444404:A/G	23	RP11-93I21.3	ncRNA_intronic	11.71	7	5	15
4:125456502:C:T	4:125456502:A/G	C	T	0.3141	0.000379708	-0.191969	0.0540179	0.738397	4:125444404:A/G	23	RP11-93I21.3	ncRNA_intronic	4.22	6	5	15
4:125457073:C:T	4:125457073:A/G	T	C	0.3141	0.000379723	-0.191971	0.0540188	0.738397	4:125444404:A/G	23	RP11-93I21.3	ncRNA_intronic	4.437	6	5	15
4:125457123:A:G	4:125457123:A/G	G	A	0.4453	6.89551e-06	-0.221266	0.0492044	0.965376	4:125419635:A/C	23	RP11-93I21.3	ncRNA_intronic	0.908	7	5	15
4:125457244:A:G	4:125457244:A/G	A	G	0.2833	8.60215e-05	-0.222425	0.0566402	0.859033	4:125444404:A/G	23	RP11-93I21.3	ncRNA_intronic	8.577	6	5	15
4:125457329:A:C	4:125457329:A/C	C	A	0.3141	0.000379812	-0.191972	0.0540199	0.738397	4:125444404:A/G	23	RP11-93I21.3	ncRNA_intronic	1.237	6	5	15
4:125457377:G:GA	rs57523386	GA	G	0.3151	NA	NA	NA	0.734472	4:125444404:A/G	23	RP11-93I21.3	ncRNA_intronic	2.858	NA	5	15
4:125457662:C:T	4:125457662:A/G	C	T	0.3151	0.000381055	-0.191928	0.0540205	0.74204	4:125444404:A/G	23	RP11-93I21.3	ncRNA_intronic	1.524	6	5	15
4:125457781:C:T	4:125457781:A/G	C	T	0.3141	0.000380721	-0.191943	0.0540212	0.738397	4:125444404:A/G	23	RP11-93I21.3	ncRNA_intronic	0.928	6	5	15
4:125458226:A:G	4:125458226:A/G	A	G	0.3141	0.000380915	-0.191943	0.0540234	0.738397	4:125444404:A/G	23	RP11-93I21.3	ncRNA_intronic	6.247	6	5	15
4:125458268:A:C	4:125458268:A/C	C	A	0.3141	0.000380969	-0.19194	0.0540232	0.738397	4:125444404:A/G	23	RP11-93I21.3	ncRNA_intronic	0.227	6	5	15
4:125458401:A:G	4:125458401:A/G	G	A	0.2833	0.000139997	-0.217189	0.0570325	0.859033	4:125444404:A/G	23	RP11-93I21.3	ncRNA_intronic	1.598	7	5	15
4:125459169:GA:GAA	rs558942133	GAA	GA	0.00497	NA	NA	NA	0.74889	4:125444404:A/G	23	RP11-93I21.3	ncRNA_intronic	NA	NA	7	15
4:125459169:G:GAA	rs144045444	GAA	G	0.3091	NA	NA	NA	0.74889	4:125444404:A/G	23	RP11-93I21.3	ncRNA_intronic	2.349	NA	7	15
4:125459632:A:G	4:125459632:A/G	A	G	0.3141	0.000383308	-0.191922	0.0540425	0.738397	4:125444404:A/G	23	RP11-93I21.3	ncRNA_intronic	5.572	7	7	15
4:125459674:A:G	4:125459674:A/G	G	A	0.3141	0.00037484	-0.192293	0.0540575	0.738397	4:125444404:A/G	23	RP11-93I21.3	ncRNA_intronic	3.06	6	7	15
4:125459813:A:AT	rs59610773	AT	A	0.3111	NA	NA	NA	0.748496	4:125444404:A/G	23	RP11-93I21.3	ncRNA_intronic	0.354	NA	7	15
4:125460069:A:C	4:125460069:A/C	A	C	0.3091	0.000381431	-0.192673	0.0542342	0.74889	4:125444404:A/G	23	RP11-93I21.3	ncRNA_intronic	5.431	6	15	15
4:125460187:G:T	4:125460187:A/C	G	T	0.3141	0.000384235	-0.191911	0.0540491	0.738397	4:125444404:A/G	23	RP11-93I21.3	ncRNA_intronic	3.285	6	15	15
4:125460344:C:T	4:125460344:A/G	T	C	0.3141	0.000384895	-0.191889	0.0540496	0.738397	4:125444404:A/G	23	RP11-93I21.3	ncRNA_intronic	10.29	7	15	15
4:125461014:A:C	4:125461014:A/C	A	C	0.3151	0.000385792	-0.191862	0.0540515	0.74204	4:125444404:A/G	23	RP11-93I21.3	ncRNA_intronic	1.896	6	15	15
4:125461021:C:T	4:125461021:A/G	C	T	0.2843	8.76405e-05	-0.222666	0.0567665	0.862652	4:125444404:A/G	23	RP11-93I21.3	ncRNA_intronic	2.719	6	15	15
4:125461314:A:G	4:125461314:A/G	G	A	0.3141	0.000385537	-0.191875	0.0540524	0.738397	4:125444404:A/G	23	RP11-93I21.3	ncRNA_intronic	4.643	5	9	15
4:125462125:A:C	4:125462125:A/C	A	C	0.3141	0.000385882	-0.191869	0.0540545	0.738397	4:125444404:A/G	23	RP11-93I21.3	ncRNA_intronic	1.097	5	15	15
4:125462137:A:G	4:125462137:A/G	G	A	0.3141	0.000385904	-0.191869	0.0540545	0.738397	4:125444404:A/G	23	RP11-93I21.3	ncRNA_intronic	0.963	5	15	15
4:125462188:C:G	4:125462188:C/G	C	G	0.2833	0.000139676	-0.217391	0.057077	0.859033	4:125444404:A/G	23	RP11-93I21.3	ncRNA_intronic	4.263	7	15	15
4:125462941:A:G	4:125462941:A/G	A	G	0.3091	0.000381235	-0.192682	0.0542346	0.74889	4:125444404:A/G	23	RP11-93I21.3	ncRNA_intronic	0.169	5	14	15
4:125463025:A:G	4:125463025:A/G	G	A	0.3141	0.000477975	-0.188984	0.0541066	0.738397	4:125444404:A/G	23	RP11-93I21.3	ncRNA_intronic	4.482	4	14	15
4:125463491:G:T	4:125463491:A/C	G	T	0.2833	8.75164e-05	-0.222733	0.0567788	0.859033	4:125444404:A/G	23	RP11-93I21.3	ncRNA_intronic	12.14	5	14	15
4:125463533:A:G	4:125463533:A/G	A	G	0.3141	0.000388096	-0.191801	0.0540582	0.738397	4:125444404:A/G	23	RP11-93I21.3	ncRNA_intronic	2.414	7	14	15
4:125463652:A:G	4:125463652:A/G	A	G	0.2833	8.75323e-05	-0.222733	0.0567796	0.859033	4:125444404:A/G	23	RP11-93I21.3	ncRNA_intronic	2.27	5	14	15
4:125464140:C:T	4:125464140:A/G	T	C	0.3082	0.000375781	-0.193149	0.0543082	0.759821	4:125444404:A/G	23	RP11-93I21.3	ncRNA_intronic	6.918	5	14	15
4:125465119:C:T	4:125465119:A/G	C	T	0.3141	0.000389834	-0.19158	0.0540138	0.738397	4:125444404:A/G	23	RP11-93I21.3	ncRNA_intronic	2.81	5	1	15
4:125465163:A:G	4:125465163:A/G	A	G	0.3141	0.000392258	-0.191478	0.0540099	0.738397	4:125444404:A/G	23	RP11-93I21.3	ncRNA_intronic	3.169	5	1	15

4:125465452:A:G	4:125465452:A/G	A	G	0.3141	0.000390683	-0.191549	0.0540139	0.738397	4:125444404:A/G	23	RP11-93I21.3	ncRNA_intronic	1.972	5	14	15
4:125466142:C:T	4:125466142:A/G	C	T	0.3151	0.000490346	-0.188343	0.0540293	0.74204	4:125444404:A/G	23	RP11-93I21.3	ncRNA_intronic	4.165	6	14	15
4:125466363:C:T	4:125466363:A/G	T	C	0.2843	9.59204e-05	-0.221749	0.056849	0.862652	4:125444404:A/G	23	RP11-93I21.3	ncRNA_intronic	3.083	7	14	15
4:125466433:A:G	4:125466433:A/G	G	A	0.2843	0.000139842	-0.21759	0.0571338	0.862652	4:125444404:A/G	23	RP11-93I21.3	ncRNA_intronic	1.142	6	15	15
4:125466534:A:G	4:125466534:A/G	G	A	0.2843	9.58917e-05	-0.221759	0.0568504	0.862652	4:125444404:A/G	23	RP11-93I21.3	ncRNA_intronic	7.65	6	15	15
4:125466711:C:T	4:125466711:A/G	C	T	0.3151	0.000479165	-0.188688	0.0540322	0.74204	4:125444404:A/G	23	RP11-93I21.3	ncRNA_intronic	0.813	7	15	15
4:125467120:C:T	4:125467120:A/G	T	C	0.3141	0.000479753	-0.188691	0.0540381	0.738397	4:125444404:A/G	23	RP11-93I21.3	ncRNA_intronic	0.094	7	15	15
4:125467439:A:G	4:125467439:A/G	A	G	0.3141	0.000479069	-0.188722	0.0540413	0.738397	4:125444404:A/G	23	RP11-93I21.3	ncRNA_intronic	1.213	7	15	15
4:125469037:A:T	4:125469037:A/T	A	T	0.3141	0.00035539	-0.19305	0.0540582	0.738397	4:125444404:A/G	23	RP11-93I21.3	ncRNA_intronic	1.446	6	15	15
4:125469272:C:T	4:125469272:A/G	C	T	0.4443	8.71422e-06	-0.219641	0.0493925	0.961579	4:125419635:A/C	23	RP11-93I21.3	ncRNA_intronic	3.366	5	7	15
4:125469529:A:ATGTGATC	rs113390135	A	ATGTGATC	0.3171	NA	NA	NA	0.727681	4:125444404:A/G	23	RP11-93I21.3	ncRNA_intronic	0.888	NA	7	15
4:125469723:A:G	4:125469723:A/G	G	A	0.3181	0.000540126	-0.187278	0.0541261	0.724859	4:125444404:A/G	23	RP11-93I21.3	ncRNA_intronic	0.978	7	7	15
4:125469871:G:T	4:125469871:A/C	G	T	0.2883	0.000113609	-0.219766	0.0569412	0.836502	4:125444404:A/G	23	RP11-93I21.3	ncRNA_intronic	0.079	6	15	15
4:125469932:A:C	4:125469932:A/C	A	C	0.2883	0.000113322	-0.219835	0.0569501	0.836502	4:125444404:A/G	23	RP11-93I21.3	ncRNA_intronic	2.708	7	15	15
4:125470187:C:T	4:125470187:A/G	C	T	0.3161	0.000412989	-0.191342	0.054179	0.724071	4:125444404:A/G	23	RP11-93I21.3	ncRNA_intronic	1.404	6	15	15
4:125470241:C:CT	rs11294878	CT	C	0.3161	NA	NA	NA	0.724071	4:125444404:A/G	23	RP11-93I21.3	ncRNA_intronic	0.457	NA	15	15
4:130205964:C:T	4:130205964:A/G	T	C	0.2306	0.000170364	-0.192366	0.0511702	0.892137	4:130215421:A/G	24	RP11-419L4.1	intergenic	2.679	7	5	15
4:130206071:G:T	4:130206071:A/C	G	T	0.2247	8.04959e-05	-0.211927	0.0537487	0.93684	4:130215421:A/G	24	RP11-419L4.1	intergenic	3.105	6	14	15
4:130206201:G:T	4:130206201:A/C	G	T	0.2237	7.81301e-05	-0.212237	0.0537301	0.94283	4:130215421:A/G	24	RP11-419L4.1	intergenic	4.648	7	14	15
4:130206511:G:T	4:130206511:A/C	T	G	0.2326	9.53717e-05	-0.199525	0.0511332	0.892094	4:130215421:A/G	24	RP11-419L4.1	intergenic	0.801	6	14	15
4:130206567:A:G	4:130206567:A/G	A	G	0.2247	7.19018e-05	-0.212393	0.0535009	0.948412	4:130215421:A/G	24	RP11-419L4.1	intergenic	0.69	7	14	15
4:130206602:A:T	4:130206602:A/T	A	T	0.2247	5.91997e-05	-0.214689	0.0534589	0.948412	4:130215421:A/G	24	RP11-419L4.1	intergenic	0.193	6	14	15
4:130206687:T:TTA	rs10645798	T	TTA	0.2247	NA	NA	NA	0.948412	4:130215421:A/G	24	RP11-419L4.1	intergenic	7.985	NA	14	15
4:130207400:C:G	4:130207400:C/G	C	G	0.2247	5.34663e-05	-0.216143	0.0535017	0.948412	4:130215421:A/G	24	RP11-419L4.1	intergenic	4.424	6	14	15
4:130207490:A:AATGT	rs113456066	AATGT	A	0.2247	NA	NA	NA	0.948412	4:130215421:A/G	24	RP11-419L4.1	intergenic	3.099	NA	14	15
4:130207829:C:T	4:130207829:A/G	C	T	0.2336	7.65195e-05	0.201429	0.0509298	0.887264	4:130215421:A/G	24	RP11-419L4.1	intergenic	1.243	7	14	15
4:130208223:A:G	4:130208223:A/G	A	G	0.2247	5.3099e-05	-0.216035	0.0534536	0.948412	4:130215421:A/G	24	RP11-419L4.1	intergenic	2.37	6	14	15
4:130208319:A:G	4:130208319:A/G	A	G	0.2247	5.30495e-05	-0.216043	0.0534528	0.948412	4:130215421:A/G	24	RP11-419L4.1	intergenic	1.027	6	14	15
4:130208685:C:T	4:130208685:A/G	T	C	0.2247	5.30289e-05	-0.216038	0.0534505	0.948412	4:130215421:A/G	24	RP11-419L4.1	intergenic	1.55	7	14	15
4:130208949:A:G	4:130208949:A/G	A	G	0.2247	5.24887e-05	-0.216154	0.0534474	0.948412	4:130215421:A/G	24	RP11-419L4.1	intergenic	8.772	7	9	15
4:130209180:A:G	4:130209180:A/G	G	A	0.2247	5.25155e-05	-0.216142	0.0534458	0.948412	4:130215421:A/G	24	RP11-419L4.1	intergenic	3.603	6	9	15
4:130209218:C:T	4:130209218:A/G	T	C	0.2247	5.25165e-05	-0.216139	0.0534453	0.948412	4:130215421:A/G	24	RP11-419L4.1	intergenic	4.435	6	9	15
4:130209321:A:T	4:130209321:A/T	A	T	0.2326	9.83258e-05	-0.198058	0.0508534	0.892094	4:130215421:A/G	24	RP11-419L4.1	intergenic	4.287	7	9	15
4:130209327:A:G	4:130209327:A/G	G	A	0.2247	5.25262e-05	-0.216135	0.0534447	0.948412	4:130215421:A/G	24	RP11-419L4.1	intergenic	5.8	7	9	15
4:130209470:C:T	4:130209470:A/G	C	T	0.2247	5.2513e-05	-0.216134	0.0534437	0.948412	4:130215421:A/G	24	RP11-419L4.1	intergenic	2.648	7	9	15
4:130209601:A:G	4:130209601:A/G	A	G	0.2247	5.24823e-05	-0.216138	0.0534427	0.948412	4:130215421:A/G	24	RP11-419L4.1	intergenic	5.542	6	9	15
4:130209909:C:G	4:130209909:C/G	G	C	0.2256	6.82661e-05	-0.212661	0.0534023	0.942574	4:130215421:A/G	24	RP11-419L4.1	intergenic	7.115	7	9	15
4:130209933:C:T	4:130209933:A/G	C	T	0.2256	6.82536e-05	-0.212663	0.0534022	0.942574	4:130215421:A/G	24	RP11-419L4.1	intergenic	7.509	7	9	15
4:130210210:C:G	4:130210210:C/G	C	G	0.2256	6.88448e-05	-0.21255	0.0534013	0.942574	4:130215421:A/G	24	RP11-419L4.1	intergenic	1.355	6	9	15
4:130210438:G:T	4:130210438:A/C	T	G	0.2256	6.81016e-05	-0.212675	0.0533982	0.942574	4:130215421:A/G	24	RP11-419L4.1	intergenic	5.414	7	14	15
4:130210547:A:T	4:130210547:A/T	T	A	0.2256	6.81803e-05	-0.212658	0.0533974	0.942574	4:130215421:A/G	24	RP11-419L4.1	intergenic	7.22	6	14	15
4:130210764:A:G	4:130210764:A/G	G	A	0.2256	6.81708e-05	-0.212653	0.0533959	0.942574	4:130215421:A/G	24	RP11-419L4.1	intergenic	5.448	7	9	15

4:130210926:A:G	4:130210926:A/G	G	A	0.2256	6.825e-05	-0.212634	0.0533948	0.942574	4:130215421:A/G	24	RP11-419L4.1	intergenic	6.832	6	14	15
4:130211105:C:T	4:130211105:A/G	C	T	0.2256	6.8133e-05	-0.212649	0.0533931	0.942574	4:130215421:A/G	24	RP11-419L4.1	intergenic	0.236	6	14	15
4:130211356:G:T	4:130211356:A/C	G	T	0.2256	6.65223e-05	-0.21298	0.0534001	0.942574	4:130215421:A/G	24	RP11-419L4.1	intergenic	5.794	6	14	15
4:130212054:G:GC	rs36171802	G	GC	0.2256	NA	NA	NA	0.942574	4:130215421:A/G	24	RP11-419L4.1	intergenic	8.018	NA	14	15
4:130212285:G:T	4:130212285:A/C	G	T	0.2256	6.80086e-05	-0.212636	0.0533838	0.942574	4:130215421:A/G	24	RP11-419L4.1	intergenic	9.381	5	14	15
4:130213000:T:TC	rs140758181	T	TC	0.2237	NA	NA	NA	0.94283	4:130215421:A/G	24	RP11-419L4.1	intergenic	8.487	NA	14	15
4:130213002:A:C	4:130213002:A/C	C	A	0.2256	6.79852e-05	-0.212616	0.0533777	0.942574	4:130215421:A/G	24	RP11-419L4.1	intergenic	9.993	6	9	15
4:130213168:A:G	4:130213168:A/G	A	G	0.2256	6.80204e-05	-0.212605	0.0533767	0.942574	4:130215421:A/G	24	RP11-419L4.1	intergenic	4.938	7	9	15
4:130213226:C:T	4:130213226:A/G	T	C	0.2256	6.79652e-05	-0.212613	0.0533763	0.942574	4:130215421:A/G	24	RP11-419L4.1	intergenic	1.252	7	9	15
4:130213278:G:T	4:130213278:A/C	G	T	0.2256	6.79641e-05	-0.212611	0.0533756	0.942574	4:130215421:A/G	24	RP11-419L4.1	intergenic	2.673	6	9	15
4:130213437:A:G	4:130213437:A/G	G	A	0.2256	6.8468e-05	-0.212515	0.0533749	0.942574	4:130215421:A/G	24	RP11-419L4.1	intergenic	6.19	5	9	15
4:130213485:A:G	4:130213485:A/G	A	G	0.2256	6.79398e-05	-0.212609	0.0533739	0.942574	4:130215421:A/G	24	RP11-419L4.1	intergenic	1.381	3b	9	15
4:130213622:A:G	4:130213622:A/G	A	G	0.2018	0.00017853	-0.205705	0.0548897	0.793642	4:130215421:A/G	24	RP11-419L4.1	intergenic	0.465	4	9	15
4:130214024:A:G	4:130214024:A/G	A	G	0.2237	5.46167e-05	-0.215802	0.0534834	0.94283	4:130215421:A/G	24	RP11-419L4.1	intergenic	3.943	6	14	15
4:130214217:G:T	4:130214217:A/C	G	T	0.2256	7.56886e-05	-0.211246	0.0533767	0.942574	4:130215421:A/G	24	RP11-419L4.1	intergenic	1.524	6	14	15
4:130214260:C:T	4:130214260:A/G	T	C	0.2256	7.59696e-05	-0.211193	0.0533751	0.942574	4:130215421:A/G	24	RP11-419L4.1	intergenic	4.763	6	14	15
4:130214334:C:G	4:130214334:C/G	G	C	0.2256	7.15083e-05	-0.212011	0.0533869	0.942574	4:130215421:A/G	24	RP11-419L4.1	intergenic	1.887	7	14	15
4:130214616:A:G	4:130214616:A/G	A	G	0.2256	7.14675e-05	-0.211996	0.0533815	0.942574	4:130215421:A/G	24	RP11-419L4.1	intergenic	5.401	7	14	15
4:130214619:C:T	4:130214619:A/G	T	C	0.2018	0.000188328	-0.204994	0.0548966	0.793642	4:130215421:A/G	24	RP11-419L4.1	intergenic	3.397	7	14	15
4:130214692:A:G	4:130214692:A/G	A	G	0.2256	7.13946e-05	-0.212001	0.0533795	0.942574	4:130215421:A/G	24	RP11-419L4.1	intergenic	1.425	6	14	15
4:130215318:C:G	4:130215318:C/G	G	C	0.2256	6.27865e-05	-0.213513	0.0533503	0.942574	4:130215421:A/G	24	RP11-419L4.1	intergenic	1.942	6	14	15
4:130215393:C:G	4:130215393:C/G	G	C	0.2256	5.8331e-05	-0.214492	0.0533636	0.942574	4:130215421:A/G	24	RP11-419L4.1	intergenic	0.828	7	14	15
4:130215421:A:G	4:130215421:A/G	A	G	0.2336	2.08295e-05	-0.225422	0.0529684	1	4:130215421:A/G	24	RP11-419L4.1	intergenic	1.16	7	9	15
4:130215427:A:C	4:130215427:A/C	C	A	0.2336	2.0826e-05	-0.225424	0.0529683	1	4:130215421:A/G	24	RP11-419L4.1	intergenic	1.688	7	9	15
4:130215732:G:GTTTATTTTTAA	4:130215732:G:GTTTATTTTTAA	G	GTTTATTTTTAA	0.00994	NA	NA	NA	0.888578	4:130215421:A/G	24	RP11-419L4.1	intergenic	2.605	NA	9	15
4:130215732:G:GTTTTATTTTTAA	4:130215732:G:GTTTTATTTTTAA	G	GTTTTATTTTTAA	0.2137	NA	NA	NA	0.888578	4:130215421:A/G	24	RP11-419L4.1	NA	2.547	NA	9	15
4:130221251:G:T	4:130221251:A/C	T	G	0.2247	3.67341e-05	-0.219066	0.0530798	0.948412	4:130215421:A/G	24	RP11-419L4.1	ncRNA_intronic	0.62	7	14	15
4:130221441:C:T	4:130221441:A/G	T	C	0.2048	0.000255033	-0.212033	0.057978	0.842652	4:130215421:A/G	24	RP11-419L4.1	ncRNA_intronic	1.196	6	14	15
4:130222138:C:T	4:130222138:A/G	T	C	0.2048	0.000271887	-0.211295	0.0580367	0.842652	4:130215421:A/G	24	RP11-419L4.1	ncRNA_intronic	2.919	6	14	15
4:130222730:C:CTGTT	rs141009392	C	CTGTT	0.2247	NA	NA	NA	0.948412	4:130215421:A/G	24	RP11-419L4.1	ncRNA_intronic	1.63	NA	14	15
4:130222900:C:T	4:130222900:A/G	T	C	0.2078	0.00031531	-0.209135	0.0580547	0.835451	4:130215421:A/G	24	RP11-419L4.1	ncRNA_intronic	5.348	6	14	15
4:130222976:C:T	4:130222976:A/G	C	T	0.2247	5.27639e-05	-0.2154	0.0532768	0.948412	4:130215421:A/G	24	RP11-419L4.1	ncRNA_intronic	0.959	7	14	15
4:130224111:A:G	4:130224111:A/G	A	G	0.2247	5.60111e-05	-0.214795	0.0533122	0.948412	4:130215421:A/G	24	RP11-419L4.1	ncRNA_intronic	1.93	7	9	15
4:130224323:C:T	4:130224323:A/G	C	T	0.2326	7.489e-05	-0.200755	0.0506932	0.892094	4:130215421:A/G	24	RP11-419L4.1	ncRNA_intronic	1.748	7	9	15
4:130224598:A:G	4:130224598:A/G	A	G	0.2247	5.78606e-05	-0.214456	0.0533292	0.948412	4:130215421:A/G	24	RP11-419L4.1	ncRNA_intronic	0.069	5	9	15
4:130225327:C:T	4:130225327:A/G	T	C	0.2326	8.46659e-05	-0.199434	0.0507366	0.892094	4:130215421:A/G	24	RP11-419L4.1	ncRNA_intronic	1.241	6	9	15
4:130225515:G:T	4:130225515:A/C	T	G	0.2247	6.24874e-05	-0.213624	0.053363	0.948412	4:130215421:A/G	24	RP11-419L4.1	ncRNA_intronic	7.048	7	9	15
4:130226032:C:T	4:130226032:A/G	C	T	0.2247	6.27102e-05	-0.213717	0.0533977	0.948412	4:130215421:A/G	24	RP11-419L4.1	ncRNA_intronic	2.809	7	9	15
4:130226065:A:C	4:130226065:A/C	C	A	0.2247	6.28673e-05	-0.21369	0.0533987	0.948412	4:130215421:A/G	24	RP11-419L4.1	ncRNA_exonic	2.001	7	9	15
4:130226484:A:G	4:130226484:A/G	A	G	0.2247	6.81454e-05	-0.212635	0.0533901	0.948412	4:130215421:A/G	24	RP11-419L4.1	upstream	2.779	7	9	15
4:130227612:C:G	4:130227612:C/G	C	G	0.2147	0.000160072	-0.213681	0.056606	0.893843	4:130215421:A/G	24	RP11-419L4.1	intergenic	0.863	5	9	15

4:130227810:A:G	4:130227810:A/G	G	A	0.2147	0.000152419	-0.214283	0.0565824	0.893843	4:130215421:A/G	24	RP11-419L4.1	intergenic	5.452	6	9	15
4:130228555:A:C	4:130228555:A/C	A	C	0.2147	0.000175466	-0.212441	0.0566215	0.893843	4:130215421:A/G	24	RP11-419L4.1	intergenic	3.443	7	9	15
4:130228809:G:GA	rs60820346	G	GA	0.2147	NA	NA	NA	0.893843	4:130215421:A/G	24	RP11-419L4.1	intergenic	0.243	NA	9	15
4:130229271:C:G	4:130229271:C/G	G	C	0.2147	0.000185802	-0.211523	0.0565934	0.893843	4:130215421:A/G	24	RP11-419L4.1	intergenic	0.526	6	9	15
4:183814104:A:AAGG	rs72032204	AAGG	A	0.06362	NA	NA	NA	0.608698	4:183885485:A/G	25	DCTD	intronic	5.771	NA	4	4
4:183820458:C:T	4:183820458:A/G	C	T	0.06362	0.0194149	-0.208373	0.0891447	0.635386	4:183885485:A/G	25	DCTD	intronic	9.269	7	4	5
4:183825036:C:T	4:183825036:A/G	C	T	0.06163	0.00684386	-0.242388	0.0896287	0.715342	4:183885485:A/G	25	DCTD	intronic	6.352	6	4	4
4:183826037:A:G	4:183826037:A/G	A	G	0.06163	0.00530186	-0.251059	0.0900464	0.715342	4:183885485:A/G	25	DCTD	intronic	1.214	4	2	5
4:183826268:C:T	4:183826268:A/G	T	C	0.06262	0.00385989	-0.2576	0.0891536	0.702725	4:183885485:A/G	25	DCTD	intronic	3.715	5	4	5
4:183826318:A:T	4:183826318:A/T	T	A	0.06262	0.00385112	-0.257673	0.0891567	0.702725	4:183885485:A/G	25	DCTD	intronic	5.756	5	4	5
4:183826577:G:T	4:183826577:A/C	G	T	0.06262	0.00413098	-0.255711	0.0891607	0.702725	4:183885485:A/G	25	DCTD	intronic	3.893	7	4	5
4:183826602:A:C	4:183826602:A/C	A	C	0.06262	0.00381694	-0.257966	0.0891719	0.702725	4:183885485:A/G	25	DCTD	intronic	2.457	6	4	5
4:183826788:C:T	4:183826788:A/G	T	C	0.06262	0.0037921	-0.258172	0.0891797	0.702725	4:183885485:A/G	25	DCTD	intronic	0.286	5	4	5
4:183826927:C:T	4:183826927:A/G	C	T	0.06262	0.00477327	-0.253818	0.0899441	0.702725	4:183885485:A/G	25	DCTD	intronic	1.056	5	4	5
4:183827126:C:T	4:183827126:A/G	T	C	0.06362	0.0130572	-0.219323	0.0883583	0.690479	4:183885485:A/G	25	DCTD	intronic	0.709	5	4	5
4:183827394:A:G	4:183827394:A/G	A	G	0.06262	0.00469035	-0.254384	0.0899659	0.702725	4:183885485:A/G	25	DCTD	intronic	1.435	5	4	5
4:183827522:C:T	4:183827522:A/G	C	T	0.06262	0.00466313	-0.254565	0.0899707	0.702725	4:183885485:A/G	25	DCTD	intronic	6.232	5	4	5
4:183827524:A:G	4:183827524:A/G	G	A	0.06262	0.00466191	-0.254573	0.0899709	0.702725	4:183885485:A/G	25	DCTD	intronic	6.824	5	4	5
4:183827650:A:G	4:183827650:A/G	A	G	0.06163	0.00497255	-0.253158	0.0901301	0.715342	4:183885485:A/G	25	DCTD	intronic	3.98	5	4	5
4:183827898:A:C	4:183827898:A/C	A	C	0.06163	0.00492731	-0.253456	0.090142	0.715342	4:183885485:A/G	25	DCTD	intronic	16.52	5	4	5
4:183828053:A:G	4:183828053:A/G	A	G	0.06262	0.00458835	-0.25511	0.0899986	0.702725	4:183885485:A/G	25	DCTD	intronic	0.336	4	4	5
4:183828776:G:GT	rs35418900	G	GT	0.06262	NA	NA	NA	0.702725	4:183885485:A/G	25	DCTD	intronic	5.762	NA	4	4
4:183829027:A:G	4:183829027:A/G	A	G	0.06561	0.00332536	-0.261198	0.0889657	0.722757	4:183885485:A/G	25	DCTD	intronic	0.789	7	4	5
4:183829645:A:G	4:183829645:A/G	A	G	0.06262	0.00412921	-0.258455	0.090113	0.702725	4:183885485:A/G	25	DCTD	intronic	2.588	7	4	5
4:183829751:G:T	4:183829751:A/C	T	G	0.06163	0.00438899	-0.257135	0.0902615	0.715342	4:183885485:A/G	25	DCTD	intronic	1.369	7	4	5
4:183830277:C:T	4:183830277:A/G	T	C	0.06262	0.00415393	-0.258467	0.0901763	0.702725	4:183885485:A/G	25	DCTD	intronic	2.076	7	4	5
4:183830485:A:G	4:183830485:A/G	A	G	0.06262	0.00411732	-0.258749	0.0901869	0.702725	4:183885485:A/G	25	DCTD	intronic	3.372	5	4	5
4:183831646:A:T	4:183831646:A/T	A	T	0.06262	0.0041091	-0.25883	0.0901951	0.702725	4:183885485:A/G	25	DCTD	intronic	7.131	5	4	5
4:183832334:G:T	4:183832334:A/C	T	G	0.06262	0.00371311	-0.26194	0.0902753	0.702725	4:183885485:A/G	25	DCTD	intronic	2.763	4	4	4
4:183832468:A:G	4:183832468:A/G	G	A	0.06561	0.00280263	-0.266529	0.0891822	0.722757	4:183885485:A/G	25	DCTD	intronic	1.004	5	4	4
4:183832937:C:T	4:183832937:A/G	C	T	0.06262	0.00361298	-0.262783	0.0902994	0.702725	4:183885485:A/G	25	DCTD	intronic	0.677	5	4	4
4:183833296:C:CGAAA	rs200263531	CGAAA	C	0.06262	NA	NA	NA	0.702725	4:183885485:A/G	25	DCTD	intronic	8.152	NA	4	4
4:183833502:T:TTTTAGAAACCA	rs72369981	TTTTAGAAACCA	T	0.06362	NA	NA	NA	0.718884	4:183885485:A/G	25	DCTD	intronic	14.6	NA	4	4
4:183834195:A:C	4:183834195:A/C	A	C	0.06461	0.00536271	-0.26213	0.0941429	0.711641	4:183885485:A/G	25	DCTD	intronic	0.474	7	3	4
4:183834468:A:G	4:183834468:A/G	G	A	0.0666	0.00222846	-0.274085	0.0896293	0.743899	4:183885485:A/G	25	DCTD	intronic	2.541	5	3	4
4:183834597:A:G	4:183834597:A/G	A	G	0.0666	0.00184004	-0.27877	0.0894951	0.743899	4:183885485:A/G	25	DCTD	intronic	3.628	7	3	4
4:183834728:A:G	4:183834728:A/G	G	A	0.0666	0.00186532	-0.278243	0.0894415	0.743899	4:183885485:A/G	25	DCTD	intronic	13.22	5	3	4
4:183834789:A:G	4:183834789:A/G	G	A	0.06759	0.00185945	-0.278239	0.0894131	0.732068	4:183885485:A/G	25	DCTD	intronic	12.18	5	3	4
4:183835024:C:T	4:183835024:A/G	C	T	0.0666	0.00194087	-0.277156	0.0894298	0.743899	4:183885485:A/G	25	DCTD	intronic	6.685	5	4	4
4:183835131:C:CT	rs375988489	C	CT	0.06561	NA	NA	NA	0.75606	4:183885485:A/G	25	DCTD	intronic	2.116	NA	4	4

4:183835131:C:CTTTGTTTTTGTTTTGTTTTTTGTTTG	rs141909108	CTTTGTTTTTGTTTTGTTTTTTGTTTG	C	0.04871	NA	NA	NA	0.75606	4:183885485:A/G	25	DCTD	NA	1.009	NA	4	4
4:183835135:C:G	4:183835135:C/G	C	G	0.06561	0.00196525	-0.277237	0.0895625	0.75606	4:183885485:A/G	25	DCTD	intronic	0.152	6	4	4
4:183835285:C:T	4:183835285:A/G	T	C	0.0666	0.00182517	-0.278832	0.0894464	0.743899	4:183885485:A/G	25	DCTD	intronic	1.88	7	4	4
4:183848500:A:G	4:183848500:A/G	A	G	0.05865	0.000121826	-0.393224	0.102337	0.882047	4:183885485:A/G	25	DCTD	intergenic	2.932	6	5	15
4:183849605:G:T	4:183849605:A/C	T	G	0.05865	0.000118473	-0.393651	0.102266	0.882047	4:183885485:A/G	25	DCTD	intergenic	4.767	NA	5	15
4:183852625:C:G	4:183852625:C/G	G	C	0.05865	7.93599e-05	-0.40214	0.101902	0.882047	4:183885485:A/G	25	DCTD	intergenic	0.226	7	5	15
4:183852769:C:T	4:183852769:A/G	C	T	0.05865	7.92124e-05	-0.402188	0.101903	0.882047	4:183885485:A/G	25	DCTD	intergenic	4.345	6	5	15
4:183852808:C:G	4:183852808:C/G	C	G	0.05865	7.90433e-05	-0.402239	0.101903	0.882047	4:183885485:A/G	25	DCTD	intergenic	1.706	7	5	15
4:183856420:C:T	4:183856420:A/G	C	T	0.05865	6.65696e-05	-0.407262	0.102116	0.882047	4:183885485:A/G	25	DCTD	intergenic	3.187	5	5	15
4:183857829:A:G	4:183857829:A/G	A	G	0.05865	7.23897e-05	-0.408122	0.102846	0.882047	4:183885485:A/G	25	DCTD	intergenic	1.256	6	5	15
4:183861729:C:T	4:183861729:A/G	T	C	0.05865	0.000105158	-0.397839	0.102579	0.882047	4:183885485:A/G	25	DCTD	intergenic	0.786	7	14	15
4:183862791:A:G	4:183862791:A/G	A	G	0.05865	0.000102404	-0.398426	0.10256	0.882047	4:183885485:A/G	25	DCTD	intergenic	0.65	6	13	14
4:183864856:A:C	4:183864856:A/C	A	C	0.05964	6.33362e-05	-0.409556	0.102388	0.866213	4:183885485:A/G	25	DCTD	intergenic	1.302	6	13	14
4:183870059:C:T	4:183870059:A/G	T	C	0.05865	5.30387e-05	-0.412368	0.102025	0.882047	4:183885485:A/G	25	DCTD	intergenic	7.176	7	14	15
4:183871573:C:T	4:183871573:A/G	T	C	0.05865	5.46158e-05	-0.412686	0.102278	0.882047	4:183885485:A/G	25	DCTD	intergenic	0.212	7	14	15
4:183871708:C:G	4:183871708:C/G	G	C	0.06362	5.41877e-05	-0.4049	0.100302	0.807535	4:183885485:A/G	25	DCTD	intergenic	1.615	7	14	15
4:183877690:G:T	4:183877690:A/C	G	T	0.05368	1.01137e-05	-0.478743	0.108442	1	4:183877690:A/C	25	DCTD	intergenic	1.34	6	5	15
4:183878967:C:T	4:183878967:A/G	C	T	0.05865	3.45956e-05	-0.41346	0.099848	0.948937	4:183885485:A/G	25	DCTD	intergenic	5.765	6	5	15
4:183879199:C:G	4:183879199:C/G	C	G	0.05865	3.45096e-05	-0.413469	0.0998364	0.948937	4:183885485:A/G	25	DCTD	intergenic	1.629	7	5	15
4:183880488:A:G	4:183880488:A/G	A	G	0.05865	3.42283e-05	-0.413385	0.0997709	0.948937	4:183885485:A/G	25	DCTD	intergenic	2.1	6	5	15
4:183881143:C:T	4:183881143:A/G	T	C	0.05865	3.42563e-05	-0.413364	0.0997704	0.948937	4:183885485:A/G	25	DCTD	intergenic	7.018	6	5	15
4:183881614:A:T	4:183881614:A/T	A	T	0.05964	3.96322e-05	-0.408182	0.0993235	0.897802	4:183885485:A/G	25	DCTD	intergenic	2.124	5	7	15
4:183885485:A:G	4:183885485:A/G	A	G	0.06163	1.77761e-05	-0.427083	0.0995268	1	4:183885485:A/G	25	DCTD	intergenic	7.655	NA	5	15
4:183889333:C:CA	rs34259668	CA	C	0.05865	NA	NA	NA	0.948937	4:183885485:A/G	25	DCTD	intergenic	8.755	NA	5	15
4:183889586:C:T	4:183889586:A/G	T	C	0.06064	2.04859e-05	-0.423243	0.0993638	0.982903	4:183885485:A/G	25	DCTD	intergenic	0.444	5	5	15
5:15552869:A:G	5:15552869:A/G	A	G	0.02783	0.000183668	-0.415062	0.110965	0.788211	5:15561046:A/G	26	FBXL7	intronic	1.373	7	4	15
5:15557212:A:G	5:15557212:A/G	G	A	0.0328	1.03877e-05	-0.433612	0.0983484	0.938845	5:15561046:A/G	26	FBXL7	intronic	3.002	6	5	15
5:15561046:A:G	5:15561046:A/G	G	A	0.03479	9.19236e-06	-0.434299	0.0979178	1	5:15561046:A/G	26	FBXL7	intronic	3.206	6	5	15
5:15561208:C:T	5:15561208:A/G	C	T	0.03479	2.54991e-05	-0.409147	0.0971768	1	5:15561046:A/G	26	FBXL7	intronic	10.36	NA	5	15
5:15564014:C:CAAAAAAAAT	rs536610537	C	CAAAAAAAAT	0.03976	NA	NA	NA	0.722275	5:15561046:A/G	26	FBXL7	intronic	NA	NA	5	15
5:15574631:A:G	5:15574631:A/G	G	A	0.03976	1.14551e-05	-0.418521	0.095385	0.722275	5:15561046:A/G	26	FBXL7	intronic	8.005	6	5	15
5:40685795:C:T	5:40685795:A/G	C	T	0.2247	3.9266e-06	0.239798	0.051958	0.656807	5:40812231:A/G	27	PTGER4	intronic	0.755	4	1	5
5:40687463:C:T	5:40687463:A/G	T	C	0.2247	2.99789e-06	0.242168	0.0518455	0.656807	5:40812231:A/G	27	PTGER4	intronic	10.04	5	1	5
5:40688059:A:G	5:40688059:A/G	G	A	0.2237	2.87863e-06	0.242776	0.051883	0.661941	5:40812231:A/G	27	PTGER4	intronic	6.979	5	1	7
5:40692940:A:G	5:40692940:A/G	A	G	0.1372	0.00567888	0.189988	0.0686926	0.68943	5:40704074:A/G	27	PTGER4	UTR3	13.56	6	3	15
5:40694154:C:T	5:40694154:A/G	C	T	0.2565	3.3162e-06	0.237912	0.0511616	0.649809	5:40812231:A/G	27	PTGER4	downstream	0.05	5	4	5
5:40694966:A:C	5:40694966:A/C	C	A	0.2555	3.07619e-06	0.238831	0.051189	0.645899	5:40812231:A/G	27	PTGER4	intergenic	4.564	5	4	15
5:40695196:A:AGAT	rs10672218	A	AGAT	0.2555	NA	NA	NA	0.645899	5:40812231:A/G	27	PTGER4	intergenic	1.937	NA	4	15
5:40699186:A:C	5:40699186:A/C	C	A	0.2555	3.07924e-06	0.238818	0.0511885	0.645899	5:40812231:A/G	27	PTGER4	intergenic	6.055	6	4	15
5:40699684:A:T	5:40699684:A/T	T	A	0.2555	3.07883e-06	0.238819	0.0511885	0.645899	5:40812231:A/G	27	PTGER4	intergenic	6.76	6	4	15

5:40701169:A:ATCCACG	rs146005788	A	ATCCACG	0.2495	NA	NA	NA	0.626379	5:40704074:A/G	27	PTGER4	intergenic	11.64	NA	4	15
5:40704074:A:G	5:40704074:A/G	G	A	0.1839	2.95529e-07	0.277975	0.0542256	1	5:40704074:A/G	27	PTGER4	intergenic	4.502	7	5	15
5:40704090:C:T	5:40704090:A/G	T	C	0.2565	3.31874e-06	0.237899	0.0511607	0.649809	5:40812231:A/G	27	PTGER4	intergenic	8.603	7	5	15
5:40705419:A:T	5:40705419:A/T	T	A	0.2555	3.08333e-06	0.238802	0.051188	0.645899	5:40812231:A/G	27	TTC33	intergenic	11.64	7	5	15
5:40711361:A:G	5:40711361:A/G	G	A	0.2565	3.32664e-06	0.237874	0.0511606	0.649809	5:40812231:A/G	27	TTC33	intergenic	6.474	6	5	15
5:40712797:A:C	5:40712797:A/C	C	A	0.2565	3.32738e-06	0.237868	0.0511598	0.649809	5:40812231:A/G	27	TTC33	intergenic	5.543	NA	4	15
5:40713949:C:CTA	rs144876108	CTA	C	0.1372	NA	NA	NA	0.68943	5:40704074:A/G	27	TTC33	downstream	2.524	NA	5	5
5:40717105:C:T	5:40717105:A/G	T	C	0.2565	3.3187e-06	0.237896	0.0511599	0.649809	5:40812231:A/G	27	TTC33	intronic	3.449	6	4	5
5:40718788:C:T	5:40718788:A/G	T	C	0.2565	3.33359e-06	0.237845	0.0511591	0.649809	5:40812231:A/G	27	TTC33	intronic	0.148	7	4	5
5:40719543:C:T	5:40719543:A/G	C	T	0.2565	3.32386e-06	0.237879	0.0511598	0.649809	5:40812231:A/G	27	TTC33	intronic	0.749	6	4	5
5:40720128:A:C	5:40720128:A/C	C	A	0.2555	3.09444e-06	0.238756	0.0511863	0.645899	5:40812231:A/G	27	TTC33	intronic	0.682	5	4	5
5:40722451:A:G	5:40722451:A/G	A	G	0.1392	0.00760373	0.181704	0.068075	0.699625	5:40704074:A/G	27	TTC33	intronic	1.046	7	4	5
5:40722822:A:G	5:40722822:A/G	A	G	0.2575	2.94785e-06	0.239177	0.0511673	0.653744	5:40812231:A/G	27	TTC33	intronic	1.333	5	4	5
5:40726138:C:T	5:40726138:A/G	T	C	0.1849	3.02461e-07	0.277838	0.0542452	0.993492	5:40704074:A/G	27	TTC33	intronic	6.059	NA	4	5
5:40729974:C:T	5:40729974:A/G	C	T	0.2565	3.23875e-06	0.238155	0.0511605	0.649809	5:40812231:A/G	27	TTC33	intronic	7.233	6	4	5
5:40739261:C:T	5:40739261:A/G	T	C	0.2575	3.7257e-06	0.236498	0.0511223	0.646992	5:40812231:A/G	27	TTC33	intronic	0.934	6	4	5
5:40743061:A:G	5:40743061:A/G	A	G	0.2575	3.64981e-06	0.236707	0.0511204	0.646992	5:40812231:A/G	27	TTC33	intronic	2.063	7	4	5
5:40744038:C:T	5:40744038:A/G	T	C	0.1849	3.53072e-07	0.275846	0.0541654	0.993587	5:40704074:A/G	27	TTC33	intronic	0.425	5	4	5
5:40745400:A:ATATGT	rs72008631	ATATGT	A	0.1402	NA	NA	NA	0.694042	5:40704074:A/G	27	TTC33	intronic	2.165	NA	4	5
5:40746885:A:T	5:40746885:A/T	T	A	0.2565	3.46835e-06	0.237359	0.0511445	0.643902	5:40704074:A/G	27	TTC33	intronic	6.676	3a	4	5
5:40748268:A:G	5:40748268:A/G	A	G	0.2575	3.64656e-06	0.236703	0.0511175	0.646992	5:40812231:A/G	27	TTC33	intronic	4.576	6	5	5
5:40748968:A:G	5:40748968:A/G	A	G	0.2575	3.759e-06	0.236374	0.0511158	0.646992	5:40812231:A/G	27	TTC33	intronic	3.898	7	5	5
5:40752536:A:G	5:40752536:A/G	G	A	0.2575	3.66912e-06	0.236636	0.051117	0.646992	5:40812231:A/G	27	TTC33	intronic	0.765	7	5	5
5:40755568:C:T	5:40755568:A/G	T	C	0.2575	3.32384e-06	0.237775	0.0511374	0.646992	5:40812231:A/G	27	TTC33	intronic	8.618	NA	1	1
5:40756681:A:G	5:40756681:A/G	A	G	0.2575	2.70701e-06	0.239841	0.0511183	0.646992	5:40812231:A/G	27	TTC33	upstream	3.936	5	2	5
5:40767397:A:G	5:40767397:A/G	G	A	0.2863	2.20252e-06	0.241487	0.0510123	0.795979	5:40812231:A/G	27	PRKAA1	intronic	1.943	NA	4	4
5:40767458:C:T	5:40767458:A/G	T	C	0.2863	2.202e-06	0.241488	0.0510121	0.795979	5:40812231:A/G	27	PRKAA1	intronic	14.94	NA	4	4
5:40777531:T:TTA	rs144157386	T	TTA	0.2883	NA	NA	NA	0.788531	5:40812231:A/G	27	PRKAA1	intronic	9.954	NA	4	5
5:40780569:C:T	5:40780569:A/G	C	T	0.2903	2.01573e-06	0.242103	0.0509493	0.790197	5:40812231:A/G	27	PRKAA1	intronic	3.284	3a	4	5
5:40780786:A:G	5:40780786:A/G	G	A	0.2903	2.01565e-06	0.242104	0.0509493	0.790197	5:40812231:A/G	27	PRKAA1	intronic	1.069	6	5	5
5:40781187:C:T	5:40781187:A/G	C	T	0.2903	2.01565e-06	0.242104	0.0509493	0.790197	5:40812231:A/G	27	PRKAA1	intronic	2.71	3a	5	5
5:40786356:C:CA	rs35972942	CA	C	0.2942	NA	NA	NA	0.696573	5:40812231:A/G	27	PRKAA1	intronic	3.304	NA	5	5
5:40787523:C:T	5:40787523:A/G	T	C	0.2873	2.03853e-06	0.241992	0.0509503	0.800713	5:40812231:A/G	27	PRKAA1	intronic	1.195	6	4	5
5:40787569:A:AAATAAT	rs150503090	AAATAAT	A	0.2873	NA	NA	NA	0.800713	5:40812231:A/G	27	PRKAA1	intronic	1.552	NA	4	5
5:40790551:A:G	5:40790551:A/G	A	G	0.2873	1.89201e-06	0.242789	0.0509565	0.800713	5:40812231:A/G	27	PRKAA1	intronic	5.902	7	4	5
5:40791501:C:T	5:40791501:A/G	T	C	0.2873	2.03843e-06	0.241993	0.0509503	0.800713	5:40812231:A/G	27	PRKAA1	intronic	2.482	2b	2	5
5:40791884:C:T	5:40791884:A/G	T	C	0.2873	2.00182e-06	0.242274	0.0509703	0.800713	5:40812231:A/G	27	PRKAA1	intronic	6.594	4	2	5
5:40796033:C:T	5:40796033:A/G	T	C	0.2883	1.55835e-06	0.244695	0.05094	0.796385	5:40812231:A/G	27	PRKAA1	intronic	9.631	NA	1	5
5:40798974:C:T	5:40798974:A/G	T	C	0.2863	1.92234e-06	0.243414	0.0511222	0.794848	5:40812231:A/G	27	PRKAA1	upstream	13.5	2b	1	1
5:40807927:C:T	5:40807927:A/G	T	C	0.2704	1.50421e-06	0.245287	0.0509883	0.99483	5:40812231:A/G	27	PRKAA1	intergenic	0.891	NA	5	15
5:40810426:A:C	5:40810426:A/C	A	C	0.2714	1.54537e-06	0.244835	0.0509514	1	5:40812231:A/G	27	PRKAA1	intergenic	7.539	NA	5	15
5:40812231:A:G	5:40812231:A/G	A	G	0.2714	1.40528e-06	0.245386	0.0508651	1	5:40812231:A/G	27	RPL37	intergenic	8.867	5	5	15
5:40812377:A:G	5:40812377:A/G	A	G	0.2714	1.40616e-06	0.245365	0.050862	1	5:40812231:A/G	27	RPL37	intergenic	3.76	4	5	15

5:40812873:A:AT	5:40812873:A:AT	A	AT	0.2614	NA	NA	NA	0.949905	5:40812231:A/G	27	RPL37	intergenic	2.304	NA	5	15
5:40812873:A:ATT	5:40812873:A:ATT	A	ATT	0.00994	NA	NA	NA	0.949905	5:40812231:A/G	27	RPL37	NA	2.25	NA	5	15
5:40819088:C:T	5:40819088:A/G	T	C	0.2942	2.26556e-05	0.212255	0.0500961	0.890294	5:40812231:A/G	27	RPL37	intergenic	5.631	6	5	15
5:40819547:A:G	5:40819547:A/G	A	G	0.2863	1.65249e-05	0.217159	0.0504164	0.926862	5:40812231:A/G	27	RPL37	intergenic	0.518	7	5	15
5:40819753:C:T	5:40819753:A/G	T	C	0.2942	2.59059e-05	0.21069	0.0500837	0.890294	5:40812231:A/G	27	RPL37	intergenic	4.351	7	5	15
5:40829311:C:T	5:40829311:A/G	T	C	0.2942	2.60101e-05	0.210526	0.0500554	0.890294	5:40812231:A/G	27	RPL37	UTR3	2.48	4	3	4
5:40829915:C:T	5:40829915:A/G	T	C	0.2942	2.25532e-05	0.211623	0.0499348	0.890294	5:40812231:A/G	27	RPL37	UTR3	0.899	5	4	4
5:40830602:A:AT	rs201454585	A	AT	0.2972	NA	NA	NA	0.806298	5:40812231:A/G	27	RPL37	UTR3	0.898	NA	3	4
5:40835627:C:T	5:40835627:A/G	T	C	0.2942	2.01912e-05	0.213115	0.0499946	0.890294	5:40812231:A/G	27	RPL37	upstream	4.265	4	1	1
5:40838022:A:C	5:40838022:A/C	C	A	0.2942	1.8431e-05	0.214167	0.0500027	0.890294	5:40812231:A/G	27	RPL37	intergenic	3.265	NA	5	5
5:40839206:C:G	5:40839206:C/G	G	C	0.2962	1.89229e-05	0.213982	0.0500281	0.871766	5:40812231:A/G	27	CARD6	intergenic	0.453	6	5	5
5:106041131:G:GGTTTA	rs141087430	GGTTTA	G	0.03579	NA	NA	NA	0.706979	5:106095212:A/G	28	CTC-254B4.1	intergenic	0.42	NA	14	15
5:106042332:C:T	5:106042332:A/G	C	T	0.03579	0.00271837	0.330106	0.110112	0.706979	5:106095212:A/G	28	CTC-254B4.1	intergenic	1.662	7	14	15
5:106043534:A:G	5:106043534:A/G	A	G	0.03082	0.000190016	0.432391	0.115862	0.897521	5:106095212:A/G	28	CTC-254B4.1	intergenic	0.717	7	14	15
5:106044549:G:T	5:106044549:A/C	G	T	0.03082	2.7235e-05	-0.491816	0.117227	0.897521	5:106095212:A/G	28	CTC-254B4.1	intergenic	0.562	NA	15	15
5:106045322:C:G	5:106045322:C/G	C	G	0.02982	2.25244e-05	0.496306	0.117101	0.929404	5:106095212:A/G	28	CTC-254B4.1	intergenic	1.599	7	9	15
5:106045323:A:T	5:106045323:A/T	A	T	0.02982	2.25244e-05	0.496306	0.117101	0.929404	5:106095212:A/G	28	CTC-254B4.1	intergenic	0.332	7	9	15
5:106049736:C:T	5:106049736:A/G	T	C	0.03181	2.94568e-05	0.460994	0.110349	0.867632	5:106095212:A/G	28	CTC-254B4.1	intergenic	1.058	6	15	15
5:106052217:A:G	5:106052217:A/G	A	G	0.02982	0.000506574	0.445597	0.128146	0.929404	5:106095212:A/G	28	CTC-254B4.1	intergenic	1.419	7	15	15
5:106052831:C:T	5:106052831:A/G	T	C	0.02883	5.93191e-05	0.510633	0.127166	0.891838	5:106095212:A/G	28	CTC-254B4.1	intergenic	0.499	7	15	15
5:106053561:A:G	5:106053561:A/G	G	A	0.02982	9.70775e-05	0.446712	0.114607	0.929404	5:106095212:A/G	28	CTC-254B4.1	intergenic	0.609	7	15	15
5:106053867:A:C	5:106053867:A/C	A	C	0.02982	2.31667e-05	0.490442	0.11589	0.929404	5:106095212:A/G	28	CTC-254B4.1	intergenic	0.218	7	15	15
5:106054718:A:G	5:106054718:A/G	G	A	0.02982	9.79048e-05	0.445667	0.114399	0.929404	5:106095212:A/G	28	CTC-254B4.1	intergenic	0.235	6	15	15
5:106054849:G:T	5:106054849:A/C	T	G	0.02982	8.98422e-05	0.448487	0.114512	0.929404	5:106095212:A/G	28	CTC-254B4.1	intergenic	0.079	6	15	15
5:106061172:A:G	5:106061172:A/G	A	G	0.02883	3.52573e-05	0.491761	0.118882	0.891838	5:106095212:A/G	28	CTC-254B4.1	intergenic	0.927	7	15	15
5:106075773:C:T	5:106075773:A/G	C	T	0.02982	0.000114227	0.438065	0.113541	0.929404	5:106095212:A/G	28	CTC-254B4.1	intergenic	1.843	6	15	15
5:106081481:C:T	5:106081481:A/G	T	C	0.02982	4.51361e-05	0.468672	0.114885	0.929404	5:106095212:A/G	28	CTC-254B4.1	intergenic	1.291	7	15	15
5:106085565:A:G	5:106085565:A/G	G	A	0.0328	0.000365212	0.396491	0.111248	0.839553	5:106095212:A/G	28	CTC-254B4.1	intergenic	0.257	5	9	15
5:106087685:A:T	5:106087685:A/T	A	T	0.02982	4.0141e-05	0.468327	0.114041	0.929404	5:106095212:A/G	28	CTC-254B4.1	intergenic	0.255	6	15	15
5:106087948:C:G	5:106087948:C/G	C	G	0.02982	0.000155533	0.425904	0.112612	0.929404	5:106095212:A/G	28	CTC-254B4.1	intergenic	0.684	7	15	15
5:106088659:C:CTGA	rs139175084	CTGA	C	0.03082	NA	NA	NA	0.897521	5:106095212:A/G	28	CTC-254B4.1	intergenic	2.899	NA	15	15
5:106093230:A:C	5:106093230:A/C	A	C	0.02982	3.76521e-05	0.470805	0.114233	0.929404	5:106095212:A/G	28	CTC-254B4.1	intergenic	0.404	6	5	15
5:106093232:C:T	5:106093232:A/G	C	T	0.02982	3.76437e-05	0.470811	0.114233	0.929404	5:106095212:A/G	28	CTC-254B4.1	intergenic	1.787	6	5	15
5:106094166:A:T	5:106094166:A/T	T	A	0.02982	3.70165e-05	0.471886	0.114387	0.929404	5:106095212:A/G	28	CTC-254B4.1	intergenic	0.707	6	15	15
5:106095212:A:G	5:106095212:A/G	A	G	0.02783	1.14057e-05	0.508595	0.115889	1	5:106095212:A/G	28	CTC-254B4.1	intergenic	0.035	7	15	15
5:106095239:AT:ATT	rs35001053	AT	ATT	0.3171	NA	NA	NA	0.813127	5:106095212:A/G	28	CTC-254B4.1	intergenic	NA	NA	15	15
5:106095239:A:AT	rs112926073	AT	A	0.0338	NA	NA	NA	0.813127	5:106095212:A/G	28	CTC-254B4.1	intergenic	1.792	NA	15	15
5:106095472:A:G	5:106095472:A/G	A	G	0.02982	3.45521e-05	0.473962	0.114451	0.929404	5:106095212:A/G	28	CTC-254B4.1	intergenic	0.737	5	15	15
5:106095937:A:T	5:106095937:A/T	T	A	0.02982	3.60504e-05	0.473063	0.114503	0.929404	5:106095212:A/G	28	CTC-254B4.1	intergenic	5.565	7	15	15
5:106095968:C:G	5:106095968:C/G	C	G	0.02982	3.56697e-05	0.473224	0.114474	0.929404	5:106095212:A/G	28	CTC-254B4.1	intergenic	0.982	6	15	15
5:106099051:A:T	5:106099051:A/T	T	A	0.02982	3.12475e-05	0.478729	0.114964	0.929404	5:106095212:A/G	28	CTC-254B4.1	intergenic	0.307	6	15	15
5:106099359:C:T	5:106099359:A/G	C	T	0.02982	2.03323e-05	0.489104	0.114781	0.929404	5:106095212:A/G	28	CTC-254B4.1	intergenic	1.286	7	15	15

5:106099371:A:G	5:106099371:A/G	G	A	0.02982	2.03507e-05	0.489089	0.114782	0.929404	5:106095212:A/G	28	CTC-254B4.1	intergenic	0.792	6	15	15
5:141086579:A:C	5:141086579:A/C	C	A	0.499	4.68756e-05	0.20046	0.049245	0.833706	5:141096265:A/C	29	ARAP3	intergenic	2.685	7	5	14
5:141092705:G:T	5:141092705:A/C	G	T	0.3479	5.2301e-06	0.228201	0.0500955	0.910379	5:141093702:A/G	29	ARAP3	intergenic	6.043	5	2	14
5:141093702:A:G	5:141093702:A/G	G	A	0.3419	3.9255e-06	0.232086	0.0502862	1	5:141093702:A/G	29	ARAP3	intergenic	5.044	2b	2	14
5:141095027:A:T	5:141095027:A/T	A	T	0.3598	6.47355e-05	0.198425	0.0496704	0.917849	5:141093702:A/G	29	ARAP3	intergenic	17.02	5	2	14
5:141095249:G:T	5:141095249:A/C	T	G	0.3598	6.49742e-05	0.198419	0.04968	0.917849	5:141093702:A/G	29	ARAP3	intergenic	17.05	5	2	14
5:141095753:C:T	5:141095753:A/G	C	T	0.3608	6.09531e-05	0.199456	0.0497513	0.91389	5:141093702:A/G	29	ARAP3	intergenic	1.478	NA	2	14
5:141096265:G:T	5:141096265:A/C	G	T	0.4583	3.24767e-05	0.204517	0.0492176	1	5:141096265:A/C	29	ARAP3	intergenic	3.208	NA	2	14
5:141096921:C:T	5:141096921:A/G	C	T	0.3598	7.32976e-05	0.197262	0.0497464	0.917849	5:141093702:A/G	29	ARAP3	intergenic	1.894	NA	5	14
5:177818373:C:T	5:177818373:A/G	T	C	0.1879	0.000185177	0.219746	0.0587806	0.810633	5:177820543:A/G	30	COL23A1	intronic	0.178	5	5	15
5:177819837:A:G	5:177819837:A/G	A	G	0.1869	0.000139766	0.222997	0.0585513	0.804763	5:177820543:A/G	30	COL23A1	intronic	1.098	5	5	15
5:177820243:G:T	5:177820243:A/C	G	T	0.2465	1.53913e-05	0.220091	0.0509114	0.853352	5:177820543:A/G	30	COL23A1	intronic	0.699	4	2	15
5:177820543:C:T	5:177820543:A/G	C	T	0.2197	6.3013e-07	0.263629	0.0529192	1	5:177820543:A/G	30	COL23A1	intronic	2.66	6	2	15
5:177820653:C:CT	rs36102461	C	CT	0.2018	NA	NA	NA	0.8453	5:177820543:A/G	30	COL23A1	intronic	0.576	NA	5	15
5:177826929:C:G	5:177826929:C/G	C	G	0.2475	3.85713e-06	0.232281	0.0502888	0.845821	5:177820543:A/G	30	COL23A1	intronic	0.39	5	5	15
5:177830769:A:G	5:177830769:A/G	G	A	0.2485	1.66225e-06	0.237899	0.0496588	0.801005	5:177820543:A/G	30	COL23A1	intronic	11.29	6	5	15
6:12725378:A:G	6:12725378:A/G	G	A	0.01889	0.00212711	-0.559727	0.18221	0.666164	6:12838853:C/G	31	PHACTR1	intronic	4.177	7	5	15
6:12737540:C:T	6:12737540:A/G	T	C	0.02087	0.00350007	-0.519137	0.177785	0.666954	6:12838853:C/G	31	PHACTR1	intronic	8.014	6	14	15
6:12763284:A:G	6:12763284:A/G	A	G	0.02087	0.000127123	-1.05204	0.274542	0.815457	6:12838853:C/G	31	PHACTR1	intronic	4.289	5	5	15
6:12770194:C:T	6:12770194:A/G	T	C	0.02485	7.67337e-06	-0.731837	0.163572	0.962563	6:12838853:C/G	31	PHACTR1	intronic	3.25	5	5	15
6:12838853:C:G	6:12838853:C/G	G	C	0.02584	1.25188e-06	-0.818466	0.168852	1	6:12838853:C/G	31	PHACTR1	intronic	0.607	6	5	15
6:12873791:C:T	6:12873791:A/G	C	T	0.02386	0.000271773	-0.567522	0.155878	0.925282	6:12838853:C/G	31	PHACTR1	intronic	0.924	7	5	15
6:140890348:C:T	6:140890348:A/G	C	T	0.02883	3.76853e-05	-0.530863	0.128812	0.890913	6:140941310:A/G	32	AL356137.1	intergenic	3.244	6	5	15
6:140890737:C:T	6:140890737:A/G	T	C	0.02883	5.43494e-05	-0.523899	0.129804	0.890913	6:140941310:A/G	32	AL356137.1	intergenic	1.532	6	5	15
6:140907693:C:T	6:140907693:A/G	T	C	0.02584	2.65844e-05	-0.562521	0.133905	1	6:140941310:A/G	32	AL356137.1	intergenic	13	4	2	15
6:140928794:C:T	6:140928794:A/G	T	C	0.02783	3.58877e-05	-0.540279	0.13074	0.924678	6:140941310:A/G	32	AL356137.1	intergenic	4.994	6	5	15
6:140931566:T:TCACA	6:140931566:T:TCACA	T	TCACA	0.07753	NA	NA	NA	0.959527	6:140941310:A/G	32	AL356137.1	intergenic	3.269	NA	5	15
6:140931566:T:TCACACACA	6:140931566:T:TCACACACA	T	TCACACACA	0.02485	NA	NA	NA	0.959527	6:140941310:A/G	32	AL356137.1	NA	3.016	NA	5	15
6:140931566:T:TCACACA	6:140931566:T:TCACACA	T	TCACACA	0.494	NA	NA	NA	0.959527	6:140941310:A/G	32	AL356137.1	NA	3.141	NA	5	15
6:140937311:A:T	6:140937311:A/T	T	A	0.02584	1.46902e-05	-0.575266	0.132755	1	6:140941310:A/G	32	AL356137.1	intergenic	5.992	6	5	15
6:140938402:C:G	6:140938402:C/G	C	G	0.02584	1.42892e-05	-0.575544	0.132633	1	6:140941310:A/G	32	AL356137.1	intergenic	0.06	7	5	15
6:140941305:C:T	6:140941305:A/G	T	C	0.0159	0.00857599	-0.501361	0.190739	0.602748	6:140941310:A/G	32	AL356137.1	intergenic	10.19	6	5	15
6:140941310:C:T	6:140941310:A/G	C	T	0.02584	1.32832e-05	-0.576098	0.132272	1	6:140941310:A/G	32	AL356137.1	intergenic	9.028	6	5	15
6:140957369:A:G	6:140957369:A/G	G	A	0.02584	2.79464e-05	-0.559515	0.133549	0.920529	6:140941310:A/G	32	AL356137.1	intergenic	6.135	7	1	15
6:140964282:C:CCTGA	rs202235240	C	CCTGA	0.02584	NA	NA	NA	0.920529	6:140941310:A/G	32	AL356137.1	intergenic	0.206	NA	5	15
6:140968139:A:G	6:140968139:A/G	A	G	0.02584	2.79573e-05	-0.559528	0.133555	0.920529	6:140941310:A/G	32	AL356137.1	intergenic	5.352	7	5	9
6:140972068:A:T	6:140972068:A/T	A	T	0.02584	2.79988e-05	-0.559487	0.133556	0.920529	6:140941310:A/G	32	AL356137.1	intergenic	0.457	6	5	15
6:140972715:A:G	6:140972715:A/G	A	G	0.02584	2.79993e-05	-0.559486	0.133556	0.920529	6:140941310:A/G	32	AL356137.1	intergenic	0.245	6	5	15
6:140973759:A:G	6:140973759:A/G	A	G	0.02485	2.30499e-05	-0.569786	0.134603	0.880288	6:140941310:A/G	32	AL356137.1	intergenic	3.623	6	5	15
6:140975669:C:T	6:140975669:A/G	T	C	0.02584	2.80042e-05	-0.559513	0.133563	0.920529	6:140941310:A/G	32	AL356137.1	intergenic	1.554	6	5	15

6:140978723:A:G	6:140978723:A/G	A	G	0.02584	2.80111e-05	-0.559508	0.133564	0.920529	6:140941310:A/G	32	AL356137.1	intergenic	5.405	7	5	15
6:140990854:A:G	6:140990854:A/G	G	A	0.02584	2.95843e-05	-0.559135	0.133872	0.920529	6:140941310:A/G	32	AL356137.1	intergenic	0.063	3a	2	15
6:140992721:A:T	6:140992721:A/T	T	A	0.02584	2.95829e-05	-0.55915	0.133875	0.920529	6:140941310:A/G	32	AL356137.1	intergenic	3.528	6	4	15
6:140998200:A:G	6:140998200:A/G	G	A	0.02584	3.60206e-05	-0.555536	0.134459	0.920529	6:140941310:A/G	32	MIR4465	intergenic	0.224	7	5	15
6:141003074:A:T	6:141003074:A/T	T	A	0.01988	0.000462527	-0.595197	0.16998	0.681679	6:140941310:A/G	32	MIR4465	intergenic	8.818	NA	5	15
6:141006355:C:G	6:141006355:C/G	G	C	0.02584	5.07677e-05	-0.547193	0.135041	0.920529	6:140941310:A/G	32	MIR4465	intergenic	0.324	3a	5	15
6:141009692:A:C	6:141009692:A/C	A	C	0.02584	3.76388e-05	-0.554427	0.13452	0.920529	6:140941310:A/G	32	MIR4465	intergenic	0.753	7	5	15
6:141010233:A:T	6:141010233:A/T	A	T	0.02584	3.76322e-05	-0.554436	0.134521	0.920529	6:140941310:A/G	32	MIR4465	intergenic	0.88	6	5	15
6:141022287:A:G	6:141022287:A/G	G	A	0.02584	3.77634e-05	-0.554343	0.134525	0.920529	6:140941310:A/G	32	MIR4465	intergenic	0.222	7	9	15
6:141038243:G:T	6:141038243:A/C	T	G	0.02584	5.83754e-05	-0.540489	0.134474	0.920529	6:140941310:A/G	32	MIR4465	intergenic	0.983	5	9	15
6:141038357:C:T	6:141038357:A/G	T	C	0.02584	5.83754e-05	-0.540489	0.134474	0.920529	6:140941310:A/G	32	MIR4465	intergenic	5.057	5	9	15
6:148040366:C:G	6:148040366:C/G	G	C	0.03479	0.00184545	0.413626	0.132826	0.76824	6:148171512:A/G	33	SAMD5:RP11-307P5.1	ncRNA_intronic	11.15	NA	5	15
6:148103793:A:G	6:148103793:A/G	A	G	0.03678	3.49847e-05	0.559328	0.135158	0.871056	6:148171512:A/G	33	RP11-307P5.1	ncRNA_intronic	17.2	2b	1	15
6:148171512:C:T	6:148171512:A/G	C	T	0.03181	2.92365e-05	0.595749	0.142546	1	6:148171512:A/G	33	RP11-307P5.1	ncRNA_intronic	11.36	6	5	15
6:151463492:A:C	6:151463492:A/C	A	C	0.09046	9.48949e-05	-0.379014	0.0971013	0.702453	6:151471421:A/G	34	RP1-292B18.4	ncRNA_intronic	0.075	6	5	15
6:151466391:C:CCTCA	rs67692303	CCTCA	C	0.09046	NA	NA	NA	0.695326	6:151471421:A/G	34	RP1-292B18.4	ncRNA_intronic	1.2	NA	5	15
6:151469255:A:G	6:151469255:A/G	A	G	0.07555	0.000220771	-0.374339	0.101337	0.823938	6:151471421:A/G	34	RP1-292B18.4	ncRNA_intronic	1.234	5	1	15
6:151470874:A:G	6:151470874:A/G	A	G	0.06759	0.000197554	-0.406021	0.109083	0.902877	6:151471421:A/G	34	RP1-292B18.4	ncRNA_intronic	0.562	5	5	15
6:151471421:C:T	6:151471421:A/G	T	C	0.06561	2.18769e-05	-0.484261	0.114083	1	6:151471421:A/G	34	RP1-292B18.4	ncRNA_intronic	0.412	6	5	15
6:151471492:C:T	6:151471492:A/G	T	C	0.06859	4.51598e-05	-0.463912	0.113722	0.888103	6:151471421:A/G	34	RP1-292B18.4	ncRNA_intronic	0.231	7	5	15
7:31897146:A:G	7:31897146:A/G	G	A	0.2992	0.000653167	0.169341	0.049682	0.745929	7:31979673:A/G	35	PDE1C	intronic	8.375	2b	2	15
7:31898733:C:CTA	rs71559205	C	CTA	0.2962	NA	NA	NA	0.748823	7:31979673:A/G	35	PDE1C	intronic	0.249	NA	5	15
7:31898751:C:T	7:31898751:A/G	C	T	0.2962	0.000739125	0.167614	0.0496686	0.748823	7:31979673:A/G	35	PDE1C	intronic	0.761	6	5	15
7:31898797:C:T	7:31898797:A/G	C	T	0.2962	0.000739346	0.167602	0.0496662	0.748823	7:31979673:A/G	35	PDE1C	intronic	2.371	6	5	15
7:31899061:T:TAATAATATA	rs150371494	TAATAATATA	T	0.2962	NA	NA	NA	0.748823	7:31979673:A/G	35	PDE1C	intronic	0.688	NA	5	15
7:31902703:C:T	7:31902703:A/G	C	T	0.2972	0.000618747	0.1693	0.0494558	0.746652	7:31979673:A/G	35	PDE1C	intronic	0.64	7	4	15
7:31909841:A:T	7:31909841:A/T	A	T	0.2922	0.000215853	0.187401	0.0506531	0.724302	7:31979673:A/G	35	PDE1C	intronic	3.038	6	4	15
7:31909850:A:G	7:31909850:A/G	G	A	0.2922	0.000215829	0.187403	0.0506532	0.724302	7:31979673:A/G	35	PDE1C	intronic	0.405	6	4	15
7:31910092:A:G	7:31910092:A/G	A	G	0.2932	0.00058343	0.171154	0.0497655	0.748769	7:31979673:A/G	35	PDE1C	intronic	7.432	3a	4	15
7:31914487:C:G	7:31914487:C/G	C	G	0.2744	0.000330612	0.186983	0.0520838	0.83417	7:31979673:A/G	35	PDE1C	intronic	2.261	5	4	15
7:31932142:C:G	7:31932142:C/G	C	G	0.2753	0.000169352	0.197068	0.0524002	0.838907	7:31979673:A/G	35	PDE1C	intronic	4.648	4	5	15
7:31941013:G:GTA	rs71559207	G	GTA	0.2614	NA	NA	NA	0.979569	7:31979673:A/G	35	PDE1C	intronic	0.584	NA	5	15
7:31960767:A:C	7:31960767:A/C	C	A	0.2634	5.8173e-05	0.217902	0.0542031	0.98979	7:31979673:A/G	35	PDE1C	intronic	3.703	5	5	15
7:31961123:A:C	7:31961123:A/C	C	A	0.2634	4.06186e-05	0.222023	0.0541	0.98979	7:31979673:A/G	35	PDE1C	intronic	2.383	3a	5	15
7:31966432:G:GT	rs149037437	G	GT	0.2614	NA	NA	NA	0.979569	7:31979673:A/G	35	PDE1C	intronic	0.953	NA	4	15
7:31969870:C:T	7:31969870:A/G	T	C	0.2584	4.76533e-05	0.219977	0.0540903	0.974673	7:31979673:A/G	35	PDE1C	intronic	9.728	6	5	15
7:31979673:A:G	7:31979673:A/G	G	A	0.2634	3.98187e-05	0.221888	0.0540066	1	7:31979673:A/G	35	PDE1C	intronic	3.018	6	5	15
7:32008871:A:G	7:32008871:A/G	G	A	0.2644	0.00043266	0.195071	0.0554289	0.800207	7:31979673:A/G	35	PDE1C	intronic	2.821	7	5	15
7:32017412:A:G	7:32017412:A/G	G	A	0.2575	0.000611162	0.194308	0.0567057	0.766704	7:31979673:A/G	35	PDE1C	intronic	6.786	6	4	15
7:32017493:C:T	7:32017493:A/G	C	T	0.2823	0.00074853	0.1855	0.0550257	0.726779	7:31979673:A/G	35	PDE1C	intronic	7.3	5	4	15
7:32034404:A:G	7:32034404:A/G	G	A	0.2644	0.00073089	0.187986	0.0556545	0.791029	7:31979673:A/G	35	PDE1C	intronic	6.548	6	5	15

7:32037357:G:T	7:32037357:A/C	G	T	0.3012	0.00374834	0.150626	0.0519651	0.663251	7:31979673:A/G	35	PDE1C	intronic	4.854	5	5	15
7:35084601:C:T	7:35084601:A/G	C	T	0.09642	4.58641e-05	-0.419981	0.103043	1	7:35084601:A/G	36	DPY19L1	intergenic	9.55	7	5	15
7:35084602:C:T	7:35084602:A/G	C	T	0.09642	4.58711e-05	-0.419978	0.103043	1	7:35084601:A/G	36	DPY19L1	intergenic	8.31	NA	5	15
7:79065472:C:T	7:79065472:A/G	T	C	0.005964	2.30215e-05	-0.635845	0.150198	1	7:79065472:A/G	37	MAGI2	intronic	0.302	7	5	15
7:79065987:A:G	7:79065987:A/G	A	G	0.005964	2.32615e-05	-0.635733	0.150254	1	7:79065472:A/G	37	MAGI2	intronic	9.068	5	5	15
7:79068753:G:T	7:79068753:A/C	T	G	0.005964	4.55613e-05	-0.610893	0.149828	1	7:79065472:A/G	37	MAGI2	intronic	2.26	7	5	15
7:79070967:A:G	7:79070967:A/G	G	A	0.005964	4.95905e-05	-0.607978	0.149839	1	7:79065472:A/G	37	MAGI2	intronic	3.347	7	5	15
7:79074462:A:C	7:79074462:A/C	A	C	0.005964	5.59271e-05	-0.604017	0.149904	1	7:79065472:A/G	37	MAGI2	intronic	0.365	6	5	15
7:79076267:C:CA	rs201762140	C	CA	0.007952	NA	NA	NA	0.746982	7:79065472:A/G	37	MAGI2	intronic	0.584	NA	5	15
7:79083061:G:T	7:79083061:A/C	T	G	0.005964	9.1993e-05	-0.591338	0.151207	1	7:79065472:A/G	37	MAGI2-AS3	ncRNA_intronic	14.89	2b	1	1
7:79086438:A:G	7:79086438:A/G	A	G	0.005964	0.000160398	-0.568802	0.1507	1	7:79065472:A/G	37	MAGI2-AS3	ncRNA_intronic	5.249	7	4	15
7:79086439:A:C	7:79086439:A/C	C	A	0.005964	0.000160398	-0.568802	0.1507	1	7:79065472:A/G	37	MAGI2-AS3	ncRNA_intronic	5.316	7	4	15
7:79106173:A:C	7:79106173:A/C	A	C	0.005964	0.000173618	-0.574316	0.152963	1	7:79065472:A/G	37	MAGI2-AS3	intergenic	1.473	7	5	15
7:79107057:C:T	7:79107057:A/G	T	C	0.005964	0.000173645	-0.574307	0.152962	1	7:79065472:A/G	37	MAGI2-AS3	intergenic	6.253	6	5	15
7:79429672:C:T	7:79429672:A/G	C	T	0.03877	3.77181e-07	-1.40648	0.276859	0.868375	7:79460117:A/G	38	RNU6-849P	intergenic	3.386	6	13	15
7:79439672:A:G	7:79439672:A/G	A	G	0.03877	3.76117e-07	-1.40538	0.276614	0.868375	7:79460117:A/G	38	RNU6-849P	intergenic	3.399	6	5	15
7:79449277:C:T	7:79449277:A/G	C	T	0.04473	1.85099e-07	-1.41532	0.271462	0.957606	7:79460117:A/G	38	RNU6-849P	intergenic	1.003	6	5	15
7:79460117:A:G	7:79460117:A/G	A	G	0.04274	9.20202e-08	-1.44542	0.270586	1	7:79460117:A/G	38	RNU6-849P	intergenic	6.55	6	14	15
7:79584385:C:T	7:79584385:A/G	C	T	0.04473	1.849e-07	-1.42568	0.273438	0.874653	7:79460117:A/G	38	RNU6-849P	intergenic	4.887	7	14	15
7:79671968:G:T	7:79671968:A/C	G	T	0.0507	0.00246338	0.712561	0.235339	0.816782	7:79460117:A/G	38	AC004159.1	intergenic	1.839	6	14	15
7:122939382:C:T	7:122939382:A/G	T	C	0.2565	7.24343e-05	-0.224381	0.0565456	0.984991	7:122985615:A/C	39	LYPLA1P1	intergenic	5.026	7	15	15
7:122939424:C:T	7:122939424:A/G	T	C	0.2575	7.29513e-05	-0.224241	0.0565345	0.98003	7:122985615:A/C	39	LYPLA1P1	intergenic	8.047	7	15	15
7:122940247:A:T	7:122940247:A/T	A	T	0.2575	7.92913e-05	-0.223157	0.0565451	0.98003	7:122985615:A/C	39	LYPLA1P1	intergenic	4.939	6	9	15
7:122941859:A:G	7:122941859:A/G	A	G	0.2584	5.35221e-05	-0.232134	0.0574633	0.975295	7:122985615:A/C	39	LYPLA1P1	intergenic	2.44	7	9	15
7:122944950:C:T	7:122944950:A/G	C	T	0.2594	5.98523e-05	-0.226129	0.0563435	0.970419	7:122985615:A/C	39	LYPLA1P1	intergenic	1.073	5	7	15
7:122944966:C:T	7:122944966:A/G	C	T	0.2575	6.16078e-05	-0.225683	0.0563282	0.98003	7:122985615:A/C	39	LYPLA1P1	intergenic	1.34	5	7	15
7:122946635:C:T	7:122946635:A/G	C	T	0.2575	7.04754e-05	-0.224266	0.0564238	0.98003	7:122985615:A/C	39	LYPLA1P1	intergenic	6.162	7	5	15
7:122946841:A:C	7:122946841:A/C	A	C	0.2575	7.21498e-05	-0.223945	0.0564225	0.98003	7:122985615:A/C	39	LYPLA1P1	intergenic	0.084	6	5	15
7:122953307:A:G	7:122953307:A/G	A	G	0.2584	0.000105101	-0.221447	0.057096	0.975295	7:122985615:A/C	39	LYPLA1P1	intergenic	1.234	6	14	15
7:122955347:A:G	7:122955347:A/G	G	A	0.2565	0.000102741	-0.218408	0.0562324	0.984991	7:122985615:A/C	39	LYPLA1P1	intergenic	0.884	7	14	15
7:122956897:A:G	7:122956897:A/G	A	G	0.2565	0.000160778	-0.211827	0.0561308	0.984991	7:122985615:A/C	39	LYPLA1P1	intergenic	0.211	7	14	15
7:122959862:G:GTATA	7:122959862:G:GTATA	G	GTATA	0.1918	NA	NA	NA	0.677439	7:122985615:A/C	39	LYPLA1P1	intergenic	0.701	NA	9	15
7:122959862:G:GTGTATA	7:122959862:G:GTGTATA	G	GTGTATA	0.2883	NA	NA	NA	0.677439	7:122985615:A/C	39	LYPLA1P1	NA	0.666	NA	9	15
7:122962992:A:T	7:122962992:A/T	T	A	0.3529	0.000308063	-0.196422	0.0544341	0.604149	7:122985615:A/C	39	LYPLA1P1	intergenic	0.053	7	9	15
7:122962994:A:T	7:122962994:A/T	T	A	0.4712	8.93596e-06	-0.246542	0.0555095	1	7:122962994:A/T	39	LYPLA1P1	intergenic	0.052	7	9	15
7:122964017:A:G	7:122964017:A/G	A	G	0.2565	6.74264e-05	-0.227928	0.0571939	0.984991	7:122985615:A/C	39	LYPLA1P1	intergenic	0.989	5	5	15
7:122967237:A:G	7:122967237:A/G	A	G	0.2565	5.62594e-05	-0.230834	0.0573078	0.984991	7:122985615:A/C	39	LYPLA1P1	intergenic	1.407	7	5	15
7:122968098:C:T	7:122968098:A/G	T	C	0.2565	5.61729e-05	-0.23086	0.0573089	0.984991	7:122985615:A/C	39	LYPLA1P1	intergenic	0.327	6	5	15
7:122968443:G:T	7:122968443:A/C	T	G	0.2565	5.60949e-05	-0.230881	0.0573095	0.984991	7:122985615:A/C	39	LYPLA1P1	intergenic	2.147	6	7	15
7:122968560:A:G	7:122968560:A/G	G	A	0.1779	0.000823548	-0.276501	0.0826673	0.627215	7:122985615:A/C	39	LYPLA1P1	intergenic	12.52	5	7	15
7:122970488:A:G	7:122970488:A/G	G	A	0.2575	6.18506e-05	-0.229541	0.0573045	0.98003	7:122985615:A/C	39	LYPLA1P1	intergenic	3.967	7	7	15

7:122970584:A:G	7:122970584:A/G	A	G	0.1779	0.000819073	-0.276643	0.0826725	0.627215	7:122985615:A/C	39	LYPLA1P1	intergenic	0.276	7	7	15
7:122971829:C:CAA	7:122971829:C:CAA	C	CAA	0.2704	NA	NA	NA	0.862679	7:122985615:A/C	39	LYPLA1P1	intergenic	0.829	NA	9	15
7:122971829:C:CA	7:122971829:C:CA	CA	C	0.4622	NA	NA	NA	0.862679	7:122985615:A/C	39	LYPLA1P1	NA	0.851	NA	9	15
7:122975189:A:T	7:122975189:A/T	A	T	0.2565	0.000105248	-0.222894	0.0574741	0.994958	7:122985615:A/C	39	LYPLA1P1	intergenic	3.29	6	9	15
7:122977764:T:TC	rs149423887	TC	T	0.2565	NA	NA	NA	0.994958	7:122985615:A/C	39	LYPLA1P1	intergenic	1.208	NA	15	15
7:122978456:G:T	7:122978456:A/C	G	T	0.2575	0.000111105	-0.222085	0.0574612	0.989947	7:122985615:A/C	39	LYPLA1P1	intergenic	18.05	6	14	15
7:122978568:C:CA	rs141170933	C	CA	0.2555	NA	NA	NA	0.989909	7:122985615:A/C	39	LYPLA1P1	intergenic	0.237	NA	14	15
7:122978667:G:T	7:122978667:A/C	T	G	0.2565	0.000123135	-0.220657	0.0574657	0.994958	7:122985615:A/C	39	LYPLA1P1	intergenic	6.655	6	7	15
7:122982459:A:T	7:122982459:A/T	A	T	0.2555	4.6739e-05	-0.234148	0.0575108	1	7:122985615:A/C	39	IQUB	intergenic	10.07	6	7	15
7:122985615:A:C	7:122985615:A/C	A	C	0.2555	4.15295e-05	-0.236487	0.0576966	1	7:122985615:A/C	39	IQUB	intergenic	0.225	7	5	15
7:122986304:A:G	7:122986304:A/G	A	G	0.2555	4.88658e-05	-0.233721	0.0575527	1	7:122985615:A/C	39	IQUB	intergenic	0.846	7	5	15
7:122986331:C:T	7:122986331:A/G	T	C	0.2555	4.17643e-05	-0.236468	0.0577103	1	7:122985615:A/C	39	IQUB	intergenic	0.035	6	5	15
7:122987393:A:G	7:122987393:A/G	G	A	0.2565	4.68707e-05	-0.235003	0.0577301	0.994958	7:122985615:A/C	39	IQUB	intergenic	13.64	6	5	15
7:149488065:C:T	7:149488065:A/G	C	T	0.007952	6.16555e-06	0.899792	0.19904	1	7:149488065:A/G	40	SSPO	ncRNA_intronic	3.313	5	5	13
7:154433651:A:G	7:154433651:A/G	G	A	0.3569	0.000740728	0.171572	0.0508504	0.61056	7:154437099:A/G	41	DPP6	intronic	4.662	NA	5	15
7:154434131:C:T	7:154434131:A/G	C	T	0.3579	0.00110393	0.164125	0.0503049	0.607507	7:154437099:A/G	41	DPP6	intronic	3.76	6	5	15
7:154434216:C:T	7:154434216:A/G	C	T	0.2694	4.42802e-05	0.227241	0.055643	0.964201	7:154437099:A/G	41	DPP6	intronic	0.779	7	5	15
7:154434906:A:G	7:154434906:A/G	G	A	0.2783	5.3197e-05	0.219174	0.0542362	0.929352	7:154437099:A/G	41	DPP6	intronic	5.825	5	5	15
7:154435369:A:G	7:154435369:A/G	A	G	0.3678	0.00104806	0.162577	0.0496069	0.601136	7:154437099:A/G	41	DPP6	intronic	1.479	7	5	15
7:154435481:A:G	7:154435481:A/G	G	A	0.2793	5.83095e-05	0.218674	0.0544027	0.934347	7:154437099:A/G	41	DPP6	intronic	1.168	5	5	15
7:154436468:A:G	7:154436468:A/G	G	A	0.3638	0.000732045	0.169281	0.0501238	0.640409	7:154437099:A/G	41	DPP6	intronic	3.996	NA	5	15
7:154436563:C:T	7:154436563:A/G	C	T	0.2753	3.98457e-05	0.226458	0.0551212	0.99482	7:154437099:A/G	41	DPP6	intronic	0.512	NA	5	15
7:154437099:A:G	7:154437099:A/G	G	A	0.2763	3.72752e-05	0.227448	0.0551557	1	7:154437099:A/G	41	DPP6	intronic	3.264	6	5	15
7:154441270:C:T	7:154441270:A/G	C	T	0.325	3.04021e-05	0.213189	0.0511196	0.976408	7:154441305:A/G	41	DPP6	intronic	1.216	5	8	15
7:154441305:C:T	7:154441305:A/G	C	T	0.3221	2.1952e-05	0.217495	0.0512473	1	7:154441305:A/G	41	DPP6	intronic	7.098	5	8	15
7:154443285:A:G	7:154443285:A/G	A	G	0.2932	5.80584e-05	0.208919	0.0519626	0.901133	7:154437099:A/G	41	DPP6	intronic	4.296	NA	9	15
7:154443525:C:T	7:154443525:A/G	T	C	0.4145	0.000672294	0.161774	0.0475718	0.650375	7:154441305:A/G	41	DPP6	intronic	5	5	9	15
7:154443755:A:G	7:154443755:A/G	A	G	0.2922	4.33556e-05	0.212969	0.0520856	0.896127	7:154437099:A/G	41	DPP6	intronic	2.384	NA	9	15
7:154444461:C:T	7:154444461:A/G	C	T	0.4105	0.000590919	0.163836	0.0476857	0.660606	7:154441305:A/G	41	DPP6	intronic	1.14	5	9	15
7:154445684:GA:GGAA	rs148540765	GA	GGAA	0.2773	NA	NA	NA	0.840929	7:154437099:A/G	41	DPP6	intronic	NA	NA	9	15
7:154445684:G:GGAA	rs377714874	GGAA	G	0.1561	NA	NA	NA	0.840929	7:154437099:A/G	41	DPP6	intronic	0.414	NA	9	15
7:154445693:A:G	7:154445693:A/G	G	A	0.2922	3.61448e-05	0.215369	0.0521368	0.904906	7:154437099:A/G	41	DPP6	intronic	1.445	6	9	15
7:154451972:A:G	7:154451972:A/G	A	G	0.4155	0.000931998	0.157501	0.0475794	0.639865	7:154441305:A/G	41	DPP6	intronic	0.133	7	9	9
7:154452324:A:G	7:154452324:A/G	G	A	0.4195	0.000660438	0.162252	0.0476445	0.642305	7:154441305:A/G	41	DPP6	intronic	3.509	7	5	15
7:154458446:A:G	7:154458446:A/G	A	G	0.3698	0.00175895	0.150739	0.0481875	0.614728	7:154437099:A/G	41	DPP6	intronic	1.414	7	5	15
7:154459674:G:T	7:154459674:A/C	T	G	0.2903	6.79162e-05	0.207623	0.0521212	0.90373	7:154437099:A/G	41	DPP6	intronic	0.371	7	5	15
7:154461715:A:G	7:154461715:A/G	A	G	0.2853	0.000136616	-0.202906	0.0531972	0.895412	7:154437099:A/G	41	DPP6	intronic	0.19	7	5	15
8:3920204:C:G	8:3920204:C/G	G	C	0.327	0.000192369	0.195242	0.0523598	0.605461	8:3924335:A/G	42	CSMD1	intronic	1.077	7	5	15
8:3920296:C:T	8:3920296:A/G	T	C	0.326	0.000175393	0.195919	0.0522167	0.61003	8:3924335:A/G	42	CSMD1	intronic	5.12	7	5	15
8:3920413:C:T	8:3920413:A/G	T	C	0.326	0.000191571	0.194448	0.0521328	0.608808	8:3924335:A/G	42	CSMD1	intronic	0.025	7	5	15
8:3920571:A:T	8:3920571:A/T	A	T	0.327	0.000200198	-0.193879	0.0521355	0.605461	8:3924335:A/G	42	CSMD1	intronic	4.407	NA	5	15

8:3920652:C:G	8:3920652:C/G	C	G	0.327	0.000189405	0.194553	0.0521203	0.612442	8:3924335:A/G	42	CSMD1	intronic	0.695	6	5	15
8:3923401:A:G	8:3923401:A/G	G	A	0.498	0.00134796	0.153874	0.0480018	0.611792	8:3924335:A/G	42	CSMD1	intronic	1.423	5	2	15
8:3923626:C:G	8:3923626:C/G	G	C	0.499	0.00151487	0.152858	0.0481925	0.618022	8:3924335:A/G	42	CSMD1	intronic	1.393	NA	5	15
8:3924335:A:G	8:3924335:A/G	A	G	0.3907	2.72045e-05	0.205266	0.0489231	1	8:3924335:A/G	42	CSMD1	intronic	1.904	6	5	15
8:3924652:C:G	8:3924652:C/G	G	C	0.4861	0.00487093	-0.13921	0.0494451	0.600584	8:3924335:A/G	42	CSMD1	intronic	1.746	7	5	15
8:3925024:A:C	8:3925024:A/C	C	A	0.3191	3.70192e-05	0.225258	0.0546041	0.691741	8:3924335:A/G	42	CSMD1	intronic	1.633	7	5	15
8:90215184:A:G	8:90215184:A/G	A	G	0.07058	0.00026696	0.287268	0.0788021	0.754412	8:90225474:C/G	43	RP11-255L13.1	intergenic	0.612	NA	5	15
8:90220200:C:T	8:90220200:A/G	T	C	0.05268	4.96046e-05	-0.324249	0.0799137	0.983101	8:90225474:C/G	43	RP11-255L13.1	intergenic	0.333	7	5	15
8:90222307:C:G	8:90222307:C/G	C	G	0.04573	0.0014754	-0.271575	0.0854148	0.86476	8:90225474:C/G	43	RP11-255L13.1	intergenic	0.433	6	5	15
8:90222590:C:T	8:90222590:A/G	C	T	0.04573	0.00147513	-0.271575	0.0854134	0.86476	8:90225474:C/G	43	RP11-255L13.1	intergenic	11.51	7	5	15
8:90223015:A:C	8:90223015:A/C	C	A	0.04573	0.00115818	-0.277243	0.0853323	0.86476	8:90225474:C/G	43	RP11-255L13.1	intergenic	4.365	6	5	15
8:90225474:C:G	8:90225474:C/G	C	G	0.05368	4.91841e-05	-0.324352	0.0799001	1	8:90225474:C/G	43	RP11-255L13.1	intergenic	0.273	7	5	15
8:90227845:C:G	8:90227845:C/G	C	G	0.04871	0.00312748	-0.248968	0.0842554	0.813894	8:90225474:C/G	43	RP11-255L13.1	intergenic	0.342	5	5	15
8:90231065:A:G	8:90231065:A/G	A	G	0.0507	0.00256447	-0.253114	0.083934	0.782864	8:90225474:C/G	43	RP11-255L13.1	intergenic	0.924	6	9	15
8:90231890:A:G	8:90231890:A/G	G	A	0.0507	0.00297245	-0.249484	0.0839854	0.782864	8:90225474:C/G	43	RP11-255L13.1	intergenic	0.607	6	9	15
8:90233871:C:G	8:90233871:C/G	C	G	0.05964	0.00017441	-0.292807	0.0780099	0.905776	8:90225474:C/G	43	RP11-255L13.1	intergenic	0.145	7	15	15
8:90236608:A:AAAAAG	rs76278752	A	AAAAAG	0.0507	NA	NA	NA	0.748729	8:90225474:C/G	43	RP11-255L13.1	intergenic	3.151	NA	15	15
8:90238179:A:G	8:90238179:A/G	A	G	0.05865	0.000152227	-0.294504	0.0777589	0.886155	8:90225474:C/G	43	RP11-255L13.1	intergenic	3.291	5	9	15
8:90238321:C:T	8:90238321:A/G	C	T	0.05865	0.000158312	-0.293681	0.0777415	0.886155	8:90225474:C/G	43	RP11-255L13.1	intergenic	3.08	4	7	15
8:90239506:A:T	8:90239506:A/T	T	A	0.05169	0.000670615	-0.273007	0.0802649	0.768125	8:90225474:C/G	43	RP11-255L13.1	intergenic	8.561	5	7	15
8:90239851:C:T	8:90239851:A/G	C	T	0.05169	0.00112529	-0.261818	0.0803822	0.768125	8:90225474:C/G	43	RP11-255L13.1	intergenic	5.342	7	7	15
8:90240835:C:G	8:90240835:C/G	C	G	0.05865	0.000160654	-0.290192	0.0768925	0.886155	8:90225474:C/G	43	RP11-255L13.1	intergenic	2.546	7	14	15
8:90241306:A:AG	rs11359733	AG	A	0.0507	NA	NA	NA	0.748729	8:90225474:C/G	43	RP11-255L13.1	intergenic	0.199	NA	14	15
8:90241517:C:G	8:90241517:C/G	C	G	0.05865	0.000160877	-0.290139	0.0768856	0.886155	8:90225474:C/G	43	RP11-255L13.1	intergenic	1.091	6	14	15
8:90243302:A:G	8:90243302:A/G	G	A	0.0507	0.00062435	-0.275126	0.0804271	0.748729	8:90225474:C/G	43	RP11-255L13.1	intergenic	2.383	6	14	15
8:90243460:T:TAGG	rs10584168	TAGG	T	0.0507	NA	NA	NA	0.748729	8:90225474:C/G	43	RP11-255L13.1	intergenic	1.567	NA	14	15
8:90243722:A:G	8:90243722:A/G	G	A	0.05865	0.000161109	-0.290044	0.0768679	0.886155	8:90225474:C/G	43	RP11-255L13.1	intergenic	0.083	NA	9	15
8:90244014:C:T	8:90244014:A/G	C	T	0.0507	0.000626434	-0.275273	0.0804915	0.748729	8:90225474:C/G	43	RP11-255L13.1	intergenic	3.723	6	9	15
8:90244025:G:T	8:90244025:A/C	G	T	0.0507	0.000624269	-0.275105	0.0804203	0.748729	8:90225474:C/G	43	RP11-255L13.1	intergenic	0.701	6	9	15
8:90244238:C:T	8:90244238:A/G	T	C	0.0497	0.00316276	-0.243298	0.082433	0.763895	8:90225474:C/G	43	RP11-255L13.1	intergenic	12.25	5	9	15
8:90244354:A:G	8:90244354:A/G	A	G	0.05169	0.00236885	-0.249044	0.081933	0.734087	8:90225474:C/G	43	RP11-255L13.1	intergenic	0.02	7	9	15
8:90244958:A:G	8:90244958:A/G	G	A	0.05169	0.00232839	-0.249674	0.082	0.768125	8:90225474:C/G	43	RP11-255L13.1	intergenic	6.076	6	13	15
8:90246015:A:C	8:90246015:A/C	C	A	0.05169	0.000694959	-0.271632	0.0800907	0.768125	8:90225474:C/G	43	RP11-255L13.1	intergenic	0.407	5	5	15
8:90246044:G:T	8:90246044:A/C	T	G	0.0507	0.00231825	-0.249737	0.0819855	0.748729	8:90225474:C/G	43	RP11-255L13.1	intergenic	6.256	7	5	15
8:90246200:A:C	8:90246200:A/C	C	A	0.05169	0.000654242	-0.272918	0.0800801	0.768125	8:90225474:C/G	43	RP11-255L13.1	intergenic	2.392	6	5	15
8:90247596:C:T	8:90247596:A/G	C	T	0.05964	0.00021908	-0.285935	0.0773648	0.905776	8:90225474:C/G	43	RP11-255L13.1	intergenic	0.136	6	5	15
8:90247761:C:G	8:90247761:C/G	G	C	0.05964	0.000202332	-0.287692	0.0774179	0.905776	8:90225474:C/G	43	RP11-255L13.1	intergenic	11.44	7	5	15
8:90247929:C:T	8:90247929:A/G	C	T	0.05964	0.000216851	-0.286197	0.0773812	0.905776	8:90225474:C/G	43	RP11-255L13.1	intergenic	2.316	5	5	15
8:90248270:A:G	8:90248270:A/G	G	A	0.05964	0.000258272	-0.282774	0.0773891	0.905776	8:90225474:C/G	43	RP11-255L13.1	intergenic	0.209	4	5	15
8:90248505:C:G	8:90248505:C/G	G	C	0.0507	0.00277761	-0.246494	0.0824026	0.782864	8:90225474:C/G	43	RP11-255L13.1	intergenic	1.972	6	5	15
8:90249440:C:T	8:90249440:A/G	T	C	0.05964	0.000166017	-0.289015	0.0767474	0.905776	8:90225474:C/G	43	RP11-255L13.1	intergenic	3.373	5	5	15
8:90250006:T:TCCTCTCTC	rs375022079	T	TCCTCTCTC	0.05865	NA	NA	NA	0.920339	8:90225474:C/G	43	RP11-255L13.1	intergenic	8.577	NA	7	15

8:90251221:A:G	8:90251221:A/G	G	A	0.06064	0.000177822	-0.287367	0.0766596	0.891625	8:90225474:C/G	43	RP11-255L13.1	intergenic	2.798	6	5	15
8:90251859:T:TTCTC	rs144060065	T	TTCTC	0.05964	NA	NA	NA	0.905776	8:90225474:C/G	43	RP11-255L13.1	intergenic	8.441	NA	7	15
8:90252064:A:G	8:90252064:A/G	G	A	0.05169	0.000744397	-0.271651	0.0805446	0.768125	8:90225474:C/G	43	RP11-255L13.1	intergenic	4.895	5	7	15
8:90252368:A:C	8:90252368:A/C	C	A	0.05964	0.00016711	-0.288996	0.0767756	0.905776	8:90225474:C/G	43	RP11-255L13.1	intergenic	0.045	NA	7	15
8:90252801:G:T	8:90252801:A/C	G	T	0.05964	0.000158935	-0.289485	0.0766507	0.905776	8:90225474:C/G	43	RP11-255L13.1	intergenic	0.698	7	5	15
8:90254337:A:AT	rs199874306	AT	A	0.05765	NA	NA	NA	0.689093	8:90225474:C/G	43	RP11-255L13.1	intergenic	0.15	NA	5	15
8:90254517:A:G	8:90254517:A/G	G	A	0.05865	0.000170908	-0.288114	0.0766557	0.886155	8:90225474:C/G	43	RP11-255L13.1	intergenic	0.452	NA	2	15
8:90255693:A:G	8:90255693:A/G	G	A	0.05865	0.000170135	-0.288206	0.0766571	0.886155	8:90225474:C/G	43	RP11-255L13.1	ncRNA_exonic	3.149	5	2	15
8:90255886:C:CT	rs34069790	CT	C	0.05865	NA	NA	NA	0.886155	8:90225474:C/G	43	RP11-255L13.1	ncRNA_exonic	7.747	NA	2	15
8:90257657:C:T	8:90257657:A/G	C	T	0.0507	0.00266596	-0.247891	0.0825247	0.782864	8:90225474:C/G	43	RP11-255L13.1	intergenic	3.161	7	5	15
8:90257952:A:G	8:90257952:A/G	A	G	0.0497	0.00267008	-0.247873	0.0825315	0.763895	8:90225474:C/G	43	RP11-255L13.1	intergenic	0.014	7	5	15
8:90258032:A:C	8:90258032:A/C	A	C	0.0507	0.00307387	-0.244117	0.082465	0.748729	8:90225474:C/G	43	RP11-255L13.1	intergenic	1.985	6	5	15
8:90258085:C:G	8:90258085:C/G	G	C	0.05865	0.000157257	-0.290262	0.0768028	0.886155	8:90225474:C/G	43	RP11-255L13.1	intergenic	0.274	7	5	15
8:90258190:C:CCCTT	rs147054216	CCCTT	C	0.0507	NA	NA	NA	0.748729	8:90225474:C/G	43	RP11-255L13.1	intergenic	0.704	NA	5	15
8:90258323:A:G	8:90258323:A/G	G	A	0.0507	0.000841272	-0.269416	0.0806913	0.748729	8:90225474:C/G	43	RP11-255L13.1	intergenic	2.986	7	5	15
8:90258767:C:T	8:90258767:A/G	C	T	0.0507	0.000838838	-0.269482	0.0806915	0.748729	8:90225474:C/G	43	RP11-255L13.1	intergenic	8.77	6	5	15
8:90258928:G:T	8:90258928:A/C	G	T	0.0507	0.00263391	-0.248075	0.0824852	0.782864	8:90225474:C/G	43	RP11-255L13.1	intergenic	2.012	6	5	15
8:90262496:A:G	8:90262496:A/G	G	A	0.05964	0.000156818	-0.289577	0.0766074	0.905776	8:90225474:C/G	43	RP11-255L13.1	intergenic	3.529	6	4	15
8:90263457:G:T	8:90263457:A/C	T	G	0.0497	0.00240188	-0.250506	0.0825272	0.763895	8:90225474:C/G	43	RP11-255L13.1	intergenic	7.536	7	5	15
8:90266673:G:GTCTGCA	rs146441477	G	GTCTGCA	0.0497	NA	NA	NA	0.763895	8:90225474:C/G	43	RP11-255L13.1	intergenic	1.363	NA	5	15
8:90267622:A:G	8:90267622:A/G	A	G	0.05865	0.000150426	-0.293566	0.0774505	0.886155	8:90225474:C/G	43	RP11-255L13.1	intergenic	0.034	6	5	15
8:90267673:A:C	8:90267673:A/C	C	A	0.05865	0.000150405	-0.293568	0.0774504	0.886155	8:90225474:C/G	43	RP11-255L13.1	intergenic	2.318	7	5	15
8:90267875:C:T	8:90267875:A/G	T	C	0.0507	0.00276246	-0.246805	0.0824607	0.748729	8:90225474:C/G	43	RP11-255L13.1	intergenic	3.026	5	5	15
8:90267924:C:G	8:90267924:C/G	C	G	0.05865	0.000158895	-0.289645	0.0766917	0.886155	8:90225474:C/G	43	RP11-255L13.1	intergenic	1.448	5	5	15
8:90268042:A:C	8:90268042:A/C	C	A	0.05865	0.000153781	-0.290258	0.0766887	0.886155	8:90225474:C/G	43	RP11-255L13.1	intergenic	0.549	5	5	15
8:90268622:C:T	8:90268622:A/G	T	C	0.05865	0.00014354	-0.291808	0.0767514	0.886155	8:90225474:C/G	43	RP11-255L13.1	intergenic	0.905	5	4	15
8:90268625:G:T	8:90268625:A/C	T	G	0.05865	0.000175312	-0.290719	0.0774803	0.886155	8:90225474:C/G	43	RP11-255L13.1	intergenic	0.759	5	4	15
8:90268935:A:T	8:90268935:A/T	T	A	0.05865	0.000172508	-0.291022	0.0774775	0.886155	8:90225474:C/G	43	RP11-255L13.1	intergenic	2.807	6	4	15
8:90268943:A:T	8:90268943:A/T	A	T	0.05169	0.00272059	-0.246854	0.0823489	0.768125	8:90225474:C/G	43	RP11-255L13.1	intergenic	0.019	7	4	15
8:90269066:A:G	8:90269066:A/G	A	G	0.05964	0.000156444	-0.289623	0.0766074	0.905776	8:90225474:C/G	43	RP11-255L13.1	intergenic	1.283	6	4	15
8:90270016:A:G	8:90270016:A/G	A	G	0.0507	0.000648421	-0.27474	0.0805568	0.748729	8:90225474:C/G	43	RP11-255L13.1	intergenic	3.03	7	5	15
8:90270072:A:G	8:90270072:A/G	G	A	0.05865	0.000212904	-0.28619	0.077282	0.886155	8:90225474:C/G	43	RP11-255L13.1	intergenic	1.745	6	5	15
8:90270196:G:T	8:90270196:A/C	T	G	0.05865	0.000156956	-0.289578	0.076612	0.886155	8:90225474:C/G	43	RP11-255L13.1	intergenic	6.224	7	5	15
8:90270609:C:T	8:90270609:A/G	C	T	0.05169	0.000646104	-0.2748	0.0805513	0.768125	8:90225474:C/G	43	RP11-255L13.1	intergenic	3.252	7	5	15
8:90270903:C:T	8:90270903:A/G	C	T	0.05169	0.00271675	-0.246889	0.0823486	0.768125	8:90225474:C/G	43	RP11-255L13.1	intergenic	1.832	4	5	15
8:90270941:A:G	8:90270941:A/G	A	G	0.0507	0.000650921	-0.274654	0.0805565	0.748729	8:90225474:C/G	43	RP11-255L13.1	intergenic	1.067	4	5	15
8:90271710:A:C	8:90271710:A/C	C	A	0.0507	0.00243135	-0.250098	0.0824925	0.782864	8:90225474:C/G	43	RP11-255L13.1	intergenic	7.321	NA	5	15
8:90272076:C:T	8:90272076:A/G	C	T	0.05964	0.000160538	-0.289155	0.076614	0.905776	8:90225474:C/G	43	RP11-255L13.1	intergenic	12.97	4	5	15
8:90275769:A:G	8:90275769:A/G	A	G	0.04573	6.09901e-05	-0.321726	0.080252	0.636007	8:90225474:C/G	43	RP11-255L13.1	intergenic	0.916	7	5	15
8:90275942:C:T	8:90275942:A/G	T	C	0.04573	8.78492e-05	-0.318202	0.0811345	0.636007	8:90225474:C/G	43	RP11-255L13.1	intergenic	3.562	6	5	15
8:90302714:C:T	8:90302714:A/G	C	T	0.04871	0.019636	-0.243043	0.104166	0.749758	8:90225474:C/G	43	RP11-255L13.1	intergenic	9.987	7	1	15
8:133591952:A:G	8:133591952:A/G	A	G	0.3569	2.43291e-05	0.204615	0.0484758	0.848232	8:133641587:A/G	44	LRRC6	intronic	3.253	NA	5	15
8:133596889:A:G	8:133596889:A/G	G	A	0.3787	5.61941e-05	0.194199	0.048209	0.968427	8:133641587:A/G	44	LRRC6	intronic	0.702	NA	5	15

8:133597036:C:G	8:133597036:C/G	C	G	0.3787	4.80545e-05	0.196102	0.0482429	0.968427	8:133641587:A/G	44	LRRC6	intronic	0.137	1f	5	15
8:133614258:C:T	8:133614258:A/G	C	T	0.4751	0.0245496	0.114307	0.0508391	0.64324	8:133641587:A/G	44	LRRC6	intronic	4.428	1f	5	15
8:133618929:A:T	8:133618929:A/T	T	A	0.3767	1.68054e-05	0.209121	0.0485924	0.960664	8:133641587:A/G	44	LRRC6	intronic	0.081	5	5	15
8:133627603:A:G	8:133627603:A/G	G	A	0.3777	2.06512e-05	0.205629	0.0482956	0.964608	8:133641587:A/G	44	LRRC6	intronic	18.4	1f	5	15
8:133627999:C:T	8:133627999:A/G	C	T	0.3777	2.54689e-05	0.202967	0.0482039	0.964608	8:133641587:A/G	44	LRRC6	intronic	4.134	5	5	15
8:133629614:C:T	8:133629614:A/G	C	T	0.4732	0.019871	0.118086	0.0507083	0.652295	8:133641587:A/G	44	LRRC6	intronic	5.409	5	5	15
8:133631705:T:TA	rs60661885	T	TA	0.3429	NA	NA	NA	0.837675	8:133641587:A/G	44	LRRC6	intronic	1.245	NA	5	15
8:133633669:A:G	8:133633669:A/G	A	G	0.3767	3.3848e-05	0.199965	0.0482319	0.968651	8:133641587:A/G	44	LRRC6	intronic	1.218	5	4	15
8:133634814:C:T	8:133634814:A/G	T	C	0.3767	1.96583e-05	0.206521	0.0483799	0.968651	8:133641587:A/G	44	LRRC6	intronic	0.078	5	4	15
8:133637659:A:G	8:133637659:A/G	A	G	0.3777	3.05522e-05	0.201033	0.0482171	0.972461	8:133641587:A/G	44	LRRC6	exonic	0.001	5	5	15
8:133641417:A:G	8:133641417:A/G	A	G	0.3817	1.30006e-05	0.209278	0.0479985	0.996022	8:133641587:A/G	44	LRRC6	intronic	3.936	5	5	15
8:133641587:C:T	8:133641587:A/G	T	C	0.3827	1.28386e-05	0.209537	0.0480276	1	8:133641587:A/G	44	LRRC6	intronic	2.988	5	5	15
8:133654978:C:T	8:133654978:A/G	C	T	0.3827	3.58582e-05	0.198977	0.0481474	0.992026	8:133641587:A/G	44	LRRC6	intronic	1.008	5	4	15
8:133655147:A:C	8:133655147:A/C	C	A	0.3827	3.80897e-05	0.198197	0.0481205	0.992026	8:133641587:A/G	44	LRRC6	intronic	2.467	5	4	15
8:133667805:A:T	8:133667805:A/T	A	T	0.3807	1.92924e-05	0.206368	0.048297	0.992079	8:133641587:A/G	44	LRRC6	intronic	4.137	5	5	15
8:133685650:C:T	8:133685650:A/G	C	T	0.3986	2.00994e-05	0.207104	0.048573	0.923504	8:133641587:A/G	44	LRRC6	intronic	6.759	5	1	15
8:143752235:C:T	8:143752235:A/G	C	T	0.4314	2.41789e-08	0.28149	0.0504543	1	8:143752235:A/G	45	JRK:PSCA	ncRNA_intronic	1.05	5	1	15
8:143752994:C:T	8:143752994:A/G	C	T	0.4473	1.03155e-10	-0.319673	0.0494676	0.983771	8:143776668:A/G	45	JRK:PSCA	ncRNA_intronic	2.731	5	1	15
8:143753289:C:T	8:143753289:A/G	T	C	0.4742	7.18511e-08	0.264433	0.0490919	0.737788	8:143752235:A/G	45	JRK:PSCA	ncRNA_intronic	3.975	NA	1	15
8:143753546:A:G	8:143753546:A/G	G	A	0.4125	1.81865e-07	0.27058	0.051865	0.906721	8:143752235:A/G	45	JRK:PSCA	ncRNA_intronic	0.108	5	2	15
8:143753693:T:TCCC	rs111617116	T	TCCC	0.3857	NA	NA	NA	0.742664	8:143776668:A/G	45	JRK:PSCA	ncRNA_intronic	2.848	NA	2	15
8:143754728:A:G	8:143754728:A/G	A	G	0.4463	9.78496e-11	-0.318474	0.0492213	0.979741	8:143776668:A/G	45	JRK:PSCA	ncRNA_intronic	1.387	NA	3	15
8:143755426:A:C	8:143755426:A/C	C	A	0.4473	7.61265e-11	-0.318874	0.0489966	0.983771	8:143776668:A/G	45	JRK:PSCA	ncRNA_intronic	2.41	2b	5	15
8:143755720:A:C	8:143755720:A/C	A	C	0.4195	2.93912e-10	-0.319351	0.0506751	0.871911	8:143776668:A/G	45	JRK:PSCA	ncRNA_intronic	0.621	4	2	15
8:143756218:C:G	8:143756218:C/G	C	G	0.4473	1.10628e-10	-0.313634	0.048613	0.983771	8:143776668:A/G	45	JRK:PSCA	ncRNA_intronic	1.771	4	2	15
8:143756530:C:T	8:143756530:A/G	C	T	0.4473	1.05932e-10	-0.313487	0.0485406	0.983771	8:143776668:A/G	45	JRK:PSCA	ncRNA_intronic	2.617	4	1	15
8:143756890:A:G	8:143756890:A/G	G	A	0.4414	1.15216e-10	-0.315685	0.0489775	0.959839	8:143776668:A/G	45	JRK:PSCA	ncRNA_intronic	4.534	4	2	7
8:143756892:A:G	8:143756892:A/G	A	G	0.4473	1.63183e-10	-0.31136	0.0487069	0.983771	8:143776668:A/G	45	JRK:PSCA	ncRNA_intronic	3.256	4	2	7
8:143756895:C:T	8:143756895:A/G	T	C	0.4473	1.63164e-10	-0.31136	0.0487069	0.983771	8:143776668:A/G	45	JRK:PSCA	ncRNA_intronic	4.028	4	2	7
8:143756919:A:G	8:143756919:A/G	G	A	0.4473	1.06463e-10	-0.313368	0.0485279	0.983771	8:143776668:A/G	45	JRK:PSCA	ncRNA_intronic	0.055	NA	2	7
8:143757037:C:T	8:143757037:A/G	C	T	0.4473	1.06107e-10	-0.313344	0.0485204	0.983771	8:143776668:A/G	45	JRK:PSCA	ncRNA_intronic	4.985	NA	2	7
8:143757286:A:C	8:143757286:A/C	A	C	0.4473	1.11998e-10	-0.312668	0.0484772	0.983771	8:143776668:A/G	45	JRK:PSCA	ncRNA_intronic	1.644	4	2	7
8:143757409:A:G	8:143757409:A/G	A	G	0.4463	1.27473e-10	-0.312227	0.0485567	0.979741	8:143776668:A/G	45	JRK:PSCA	ncRNA_exonic	3.543	4	2	7
8:143757708:G:T	8:143757708:A/C	G	T	0.4473	1.11818e-10	-0.312594	0.0484637	0.983771	8:143776668:A/G	45	JRK:PSCA	ncRNA_intronic	6.059	4	2	7
8:143757763:C:T	8:143757763:A/G	C	T	0.4473	2.40659e-10	-0.307447	0.0485478	0.983771	8:143776668:A/G	45	JRK:PSCA	ncRNA_intronic	4.804	NA	2	7
8:143759137:C:T	8:143759137:A/G	T	C	0.4473	9.40666e-11	-0.313703	0.0484395	0.983771	8:143776668:A/G	45	JRK:PSCA	ncRNA_intronic	9.56	3a	2	15
8:143759364:A:G	8:143759364:A/G	A	G	0.4334	1.50273e-10	-0.318691	0.0497561	0.922323	8:143776668:A/G	45	JRK:PSCA	ncRNA_intronic	4.72	5	2	15
8:143760141:C:G	8:143760141:C/G	G	C	0.4046	1.2003e-09	-0.315986	0.0519697	0.823403	8:143776668:A/G	45	JRK:PSCA	ncRNA_intronic	1.783	5	3	15
8:143760147:C:T	8:143760147:A/G	C	T	0.4046	1.1997e-09	-0.31599	0.0519697	0.823403	8:143776668:A/G	45	JRK:PSCA	ncRNA_intronic	0.131	5	3	15
8:143760166:C:T	8:143760166:A/G	C	T	0.4085	1.3005e-09	-0.314511	0.051837	0.835154	8:143776668:A/G	45	JRK:PSCA	ncRNA_intronic	0.269	5	3	15
8:143760168:A:C	8:143760168:A/C	A	C	0.4085	1.30059e-09	-0.31451	0.0518369	0.835154	8:143776668:A/G	45	JRK:PSCA	ncRNA_intronic	0.044	5	3	15
8:143760179:C:T	8:143760179:A/G	C	T	0.4254	1.20812e-10	-0.323938	0.0503143	0.89463	8:143776668:A/G	45	JRK:PSCA	ncRNA_intronic	0.206	5	3	15
8:143760256:A:G	8:143760256:A/G	A	G	0.4354	1.34936e-10	-0.318813	0.0496477	0.921941	8:143776668:A/G	45	JRK:PSCA	ncRNA_intronic	3.465	5	3	15

8:143760366:A:ACCAC	rs143484304	ACCAC	A	0.4483	NA	NA	NA	0.987818	8:143776668:A/G	45	JRK:PSCA	ncRNA_intronic	1.326	NA	3	15
8:143760421:C:T	8:143760421:A/G	C	T	0.4483	1.2391e-10	-0.311672	0.048438	0.987818	8:143776668:A/G	45	JRK:PSCA	ncRNA_intronic	8.137	5	2	15
8:143760444:C:T	8:143760444:A/G	T	C	0.4334	1.40984e-10	-0.319209	0.0497613	0.922323	8:143776668:A/G	45	JRK:PSCA	ncRNA_intronic	5.291	NA	2	15
8:143760588:A:C	8:143760588:A/C	C	A	0.0328	0.00040148	0.342408	0.0967492	0.890676	8:143795038:A/G	45	JRK:PSCA	ncRNA_intronic	0.145	5	2	15
8:143760836:C:CA	rs11413827	C	CA	0.4483	NA	NA	NA	0.987818	8:143776668:A/G	45	JRK:PSCA	ncRNA_intronic	10.55	NA	3	15
8:143761144:C:G	8:143761144:C/G	C	G	0.4334	1.68342e-10	-0.317741	0.0497423	0.922323	8:143776668:A/G	45	JRK:PSCA	ncRNA_intronic	2.409	5	3	15
8:143761931:C:T	8:143761931:A/G	T	C	0.4473	5.02934e-11	-0.315808	0.0480677	0.991907	8:143776668:A/G	45	JRK:PSCA	ncRNA_exonic	13.71	5	1	15
8:143762135:A:C	8:143762135:A/C	C	A	0.4473	4.89958e-11	-0.317777	0.0483387	0.991907	8:143776668:A/G	45	JRK:PSCA	ncRNA_exonic	3.242	NA	1	15
8:143762430:A:G	8:143762430:A/G	G	A	0.4473	5.83998e-11	-0.316121	0.0482791	0.991907	8:143776668:A/G	45	JRK:PSCA	ncRNA_exonic	0.3	5	1	15
8:143762724:A:C	8:143762724:A/C	A	C	0.4722	8.84568e-09	0.274772	0.047774	0.724598	8:143752235:A/G	45	JRK:PSCA	ncRNA_intronic	3.882	2b	1	15
8:143762932:A:G	8:143762932:A/G	A	G	0.4463	5.32079e-11	-0.317196	0.0483408	0.987877	8:143776668:A/G	45	JRK:PSCA	ncRNA_intronic	3.587	4	1	15
8:143763043:A:G	8:143763043:A/G	G	A	0.4473	5.46438e-11	-0.317037	0.0483458	0.991907	8:143776668:A/G	45	JRK:PSCA	ncRNA_intronic	0.431	NA	1	15
8:143763083:A:G	8:143763083:A/G	G	A	0.4473	5.58655e-11	-0.316846	0.0483411	0.991907	8:143776668:A/G	45	JRK:PSCA	ncRNA_intronic	0.079	NA	1	15
8:143763109:A:G	8:143763109:A/G	G	A	0.4473	5.46468e-11	-0.317039	0.0483463	0.991907	8:143776668:A/G	45	JRK:PSCA	ncRNA_intronic	4.63	NA	1	15
8:143763259:T:TG	rs71514093	T	TG	0.4473	NA	NA	NA	0.991907	8:143776668:A/G	45	JRK:PSCA	ncRNA_exonic	3.136	NA	1	15
8:143763490:C:T	8:143763490:A/G	C	T	0.4473	5.3105e-11	-0.317188	0.0483374	0.991907	8:143776668:A/G	45	PSCA	exonic	3.569	4	1	15
8:143763547:A:C	8:143763547:A/C	A	C	0.4473	5.44993e-11	-0.317079	0.0483493	0.991907	8:143776668:A/G	45	PSCA	exonic	8.581	4	1	15
8:143763618:C:G	8:143763618:C/G	G	C	0.4483	5.13689e-11	-0.31767	0.0483744	0.987864	8:143776668:A/G	45	PSCA	UTR3	3.37	4	1	15
8:143763622:C:T	8:143763622:A/G	T	C	0.4473	5.74014e-11	-0.316831	0.0483686	0.991907	8:143776668:A/G	45	PSCA	UTR3	1.192	4	1	15
8:143763690:A:G	8:143763690:A/G	A	G	0.4473	5.32705e-11	-0.317208	0.0483439	0.991907	8:143776668:A/G	45	PSCA	UTR3	0.273	5	1	15
8:143763750:A:G	8:143763750:A/G	A	G	0.4473	5.44127e-11	-0.317115	0.0483529	0.991907	8:143776668:A/G	45	PSCA	UTR3	8.531	5	1	15
8:143763757:G:T	8:143763757:A/C	G	T	0.4473	5.44058e-11	-0.317116	0.0483529	0.991907	8:143776668:A/G	45	PSCA	UTR3	5.845	5	1	15
8:143763958:A:G	8:143763958:A/G	A	G	0.4473	5.50949e-11	-0.317062	0.0483587	0.991907	8:143776668:A/G	45	PSCA	UTR3	0.214	1f	1	15
8:143764001:A:G	8:143764001:A/G	A	G	0.4473	5.48788e-11	-0.31706	0.0483541	0.991907	8:143776668:A/G	45	PSCA	UTR3	3.124	1f	2	15
8:143764101:C:G	8:143764101:C/G	G	C	0.4473	4.89256e-11	-0.317841	0.0483471	0.991907	8:143776668:A/G	45	PSCA	UTR3	3.21	4	2	15
8:143764613:G:T	8:143764613:A/C	T	G	0.4314	8.31457e-11	-0.323214	0.0497651	0.922768	8:143776668:A/G	45	PSCA	downstream	2.155	5	2	15
8:143764879:C:G	8:143764879:C/G	C	G	0.4473	4.53529e-11	-0.3188	0.0484098	0.991907	8:143776668:A/G	45	PSCA	downstream	0.09	2b	2	15
8:143764937:A:G	8:143764937:A/G	G	A	0.4483	3.00457e-11	-0.32174	0.0484084	0.987818	8:143776668:A/G	45	PSCA	downstream	3.741	NA	2	15
8:143765326:C:G	8:143765326:C/G	G	C	0.4473	5.32541e-11	-0.317332	0.0483624	0.991907	8:143776668:A/G	45	PSCA	intergenic	0.917	5	2	15
8:143765414:C:G	8:143765414:C/G	G	C	0.4493	8.78386e-12	-0.330055	0.0483586	0.991864	8:143776668:A/G	45	PSCA	intergenic	0.206	5	2	15
8:143765699:A:G	8:143765699:A/G	G	A	0.4483	7.66863e-12	-0.330174	0.0482386	0.99594	8:143776668:A/G	45	PSCA	intergenic	2.809	NA	2	15
8:143765842:A:G	8:143765842:A/G	G	A	0.4702	1.74969e-09	0.287342	0.0477351	0.729199	8:143752235:A/G	45	PSCA	intergenic	0.437	3a	2	15
8:143766059:C:G	8:143766059:C/G	G	C	0.4334	1.61914e-11	-0.333808	0.04955	0.930149	8:143776668:A/G	45	PSCA	intergenic	7.642	2b	2	15
8:143769252:A:T	8:143769252:A/T	T	A	0.4553	1.13335e-08	-0.29177	0.0511031	0.706632	8:143776668:A/G	45	PSCA	intergenic	0.997	6	5	15
8:143769560:C:CT	rs587689684	C	CT	0.3897	NA	NA	NA	0.600336	8:143752235:A/G	45	PSCA	intergenic	1.471	NA	5	15
8:143770135:C:G	8:143770135:C/G	G	C	0.4483	7.98939e-12	-0.330066	0.0482642	0.99594	8:143776668:A/G	45	PSCA	intergenic	0.033	7	5	15
8:143771120:G:GCA	rs149382903	GCA	G	0.4553	NA	NA	NA	0.862756	8:143776668:A/G	45	PSCA	intergenic	4.707	NA	5	15
8:143771712:A:C	8:143771712:A/C	C	A	0.4473	4.9281e-11	-0.318286	0.0484226	0.991907	8:143776668:A/G	45	PSCA	intergenic	1.177	NA	5	15
8:143771714:A:C	8:143771714:A/C	A	C	0.4473	4.92703e-11	-0.318288	0.0484226	0.991907	8:143776668:A/G	45	PSCA	intergenic	0.845	NA	5	15
8:143773354:T:TG	rs5895729	T	TG	0.4483	NA	NA	NA	0.99594	8:143776668:A/G	45	LY6K	intergenic	8.538	NA	5	15
8:143774193:A:G	8:143774193:A/G	A	G	0.4493	6.80818e-12	-0.331245	0.0482748	1	8:143776668:A/G	45	LY6K	intergenic	8.453	NA	5	15
8:143774592:A:G	8:143774592:A/G	A	G	0.4692	1.57237e-09	0.288219	0.047744	0.732769	8:143752235:A/G	45	LY6K	intergenic	1.512	NA	5	15
8:143776668:C:T	8:143776668:A/G	C	T	0.4493	6.33882e-12	-0.331911	0.0483003	1	8:143776668:A/G	45	LY6K	intergenic	0.193	NA	5	15

8:143776715:C:CAA	rs67757444	CAA	C	0.4294	NA	NA	NA	0.91487	8:143776668:A/G	45	LY6K	intergenic	2.514	NA	5	15
8:143776717:A:C	8:143776717:A/C	C	A	0.3777	5.74959e-10	-0.330732	0.0533686	0.748491	8:143776668:A/G	45	LY6K	intergenic	3.813	NA	5	15
8:143776719:A:C	8:143776719:A/C	C	A	0.2793	4.97409e-09	-0.36452	0.0623319	1	8:143776719:A/C	45	LY6K	intergenic	3.158	5	5	15
8:143778381:A:T	8:143778381:A/T	T	A	0.4245	1.73177e-11	-0.337362	0.0501502	0.896385	8:143776668:A/G	45	LY6K	intergenic	8.333	6	5	15
8:143779622:C:CTT	rs35464379	CTT	C	0.4354	NA	NA	NA	0.937881	8:143776668:A/G	45	LY6K	intergenic	10.78	NA	5	15
8:143780261:T:TAA	rs10674898	T	TAA	0.4016	NA	NA	NA	0.8258	8:143776668:A/G	45	LY6K	intergenic	14.6	NA	2	15
8:143780775:C:T	8:143780775:A/G	T	C	0.4692	1.70759e-09	0.28788	0.0477934	0.724386	8:143752235:A/G	45	LY6K	upstream	3.947	NA	2	14
8:143781058:C:T	8:143781058:A/G	T	C	0.2674	5.69151e-06	-0.252236	0.0555887	0.622131	8:143822208:A/G	45	LY6K	upstream	0.338	2b	1	14
8:143781289:C:G	8:143781289:C/G	C	G	0.4732	2.10504e-08	0.271392	0.0484356	0.670857	8:143752235:A/G	45	LY6K	upstream	3.361	4	1	14
8:143781858:A:G	8:143781858:A/G	A	G	0.4682	1.61968e-08	0.273688	0.0484543	0.644688	8:143752235:A/G	45	LY6K	exonic	6.171	4	1	14
8:143782044:A:G	8:143782044:A/G	G	A	0.4722	1.48667e-08	0.27482	0.0485284	0.63373	8:143752235:A/G	45	LY6K	exonic	6.307	NA	1	14
8:143782274:C:G	8:143782274:C/G	C	G	0.4563	8.17612e-09	0.275684	0.0478221	0.679327	8:143752235:A/G	45	LY6K	intronic	4.416	4	1	14
8:143782614:C:T	8:143782614:A/G	C	T	0.4592	1.15581e-08	0.273521	0.0479347	0.676619	8:143752235:A/G	45	LY6K	intronic	4.77	3a	1	14
8:143783485:C:T	8:143783485:A/G	T	C	0.4553	2.19022e-09	0.285361	0.047695	0.683019	8:143752235:A/G	45	LY6K	UTR3	2.644	NA	1	14
8:143784500:G:T	8:143784500:A/C	G	T	0.4553	2.21808e-09	0.285075	0.0476638	0.683019	8:143752235:A/G	45	LY6K:CTD-2292P10.4	ncRNA_exonic	7.948	NA	4	14
8:143784885:A:C	8:143784885:A/C	A	C	0.4692	6.95814e-09	0.278943	0.0481607	0.642671	8:143752235:A/G	45	LY6K:CTD-2292P10.4	ncRNA_exonic	4.828	NA	4	14
8:143785345:A:G	8:143785345:A/G	A	G	0.4682	4.60143e-09	0.281965	0.048109	0.639488	8:143752235:A/G	45	LY6K:CTD-2292P10.4	ncRNA_exonic	2.595	NA	4	14
8:143785549:A:G	8:143785549:A/G	G	A	0.4682	6.07752e-09	0.280063	0.0481653	0.637977	8:143752235:A/G	45	LY6K:CTD-2292P10.4	ncRNA_exonic	0.225	5	5	14
8:143785659:C:T	8:143785659:A/G	T	C	0.4692	6.97419e-09	0.278862	0.04815	0.642671	8:143752235:A/G	45	LY6K:CTD-2292P10.4	ncRNA_exonic	2.446	NA	5	14
8:143786214:A:T	8:143786214:A/T	T	A	0.4692	6.96849e-09	0.278854	0.0481475	0.642671	8:143752235:A/G	45	LY6K:CTD-2292P10.4	ncRNA_exonic	3.011	4	5	14
8:143786461:A:G	8:143786461:A/G	G	A	0.4553	2.09312e-09	0.285583	0.0476736	0.683019	8:143752235:A/G	45	LY6K:CTD-2292P10.4	ncRNA_exonic	1.287	NA	5	14
8:143787808:A:T	8:143787808:A/T	A	T	0.4692	6.83811e-09	0.279004	0.0481469	0.642671	8:143752235:A/G	45	CTD-2292P10.4	ncRNA_exonic	4.672	5	5	15
8:143787999:A:G	8:143787999:A/G	A	G	0.4692	6.82594e-09	0.279022	0.0481475	0.642671	8:143752235:A/G	45	CTD-2292P10.4	ncRNA_exonic	1.985	NA	5	15
8:143788831:A:T	8:143788831:A/T	T	A	0.4553	2.3726e-09	0.284541	0.047662	0.683019	8:143752235:A/G	45	CTD-2292P10.4	ncRNA_exonic	8.559	6	5	14
8:143789171:C:T	8:143789171:A/G	T	C	0.4553	2.25014e-09	0.284924	0.0476571	0.683019	8:143752235:A/G	45	CTD-2292P10.4	ncRNA_intronic	9.631	NA	5	14
8:143789531:T:TTCTA	rs587685947	TTCTA	T	0.4563	NA	NA	NA	0.679327	8:143752235:A/G	45	CTD-2292P10.4	ncRNA_intronic	1.213	NA	5	14
8:143789775:C:T	8:143789775:A/G	C	T	0.4553	2.2416e-09	0.284967	0.0476592	0.683019	8:143752235:A/G	45	CTD-2292P10.4	ncRNA_exonic	3.198	NA	5	14
8:143789966:A:G	8:143789966:A/G	G	A	0.4692	6.81719e-09	0.279059	0.0481522	0.642671	8:143752235:A/G	45	CTD-2292P10.4	ncRNA_intronic	3.294	7	5	14
8:143790574:A:G	8:143790574:A/G	A	G	0.4692	6.77186e-09	0.279108	0.0481514	0.642671	8:143752235:A/G	45	CTD-2292P10.4	ncRNA_intronic	0.814	NA	5	14
8:143790934:A:G	8:143790934:A/G	A	G	0.4553	2.14417e-09	0.285394	0.047673	0.683019	8:143752235:A/G	45	CTD-2292P10.4	ncRNA_intronic	3.04	6	8	9
8:143791300:C:T	8:143791300:A/G	C	T	0.4682	7.74082e-09	0.27888	0.0482993	0.637977	8:143752235:A/G	45	CTD-2292P10.4	ncRNA_intronic	2.32	6	5	9
8:143791305:G:T	8:143791305:A/C	G	T	0.4543	2.48355e-09	-0.285224	0.0478361	0.67842	8:143752235:A/G	45	CTD-2292P10.4	ncRNA_intronic	0.029	NA	5	9
8:143791387:A:G	8:143791387:A/G	G	A	0.4692	6.73634e-09	0.279163	0.0481536	0.642671	8:143752235:A/G	45	CTD-2292P10.4	ncRNA_intronic	0.151	6	5	9
8:143791406:A:G	8:143791406:A/G	G	A	0.4553	2.21749e-09	0.285079	0.0476641	0.683019	8:143752235:A/G	45	CTD-2292P10.4	ncRNA_intronic	0.215	7	5	14
8:143792193:A:C	8:143792193:A/C	C	A	0.4692	6.67517e-09	0.279249	0.0481556	0.642671	8:143752235:A/G	45	CTD-2292P10.4	ncRNA_intronic	6.039	5	5	14
8:143792357:C:G	8:143792357:C/G	C	G	0.332	1.35413e-08	-0.303293	0.0534053	0.878643	8:143822208:A/G	45	CTD-2292P10.4	ncRNA_intronic	8.732	5	5	14

8:143792669:C:T	8:143792669:A/G	T	C	0.4692	6.5933e-09	0.279376	0.0481603	0.642671	8:143752235:A/G	45	CTD-2292P10.4	ncRNA_intronic	8.427	NA	5	15
8:143793320:C:T	8:143793320:A/G	C	T	0.4692	6.45796e-09	0.279582	0.0481671	0.642671	8:143752235:A/G	45	CTD-2292P10.4	ncRNA_intronic	4.466	NA	5	15
8:143794037:T:TA	rs139436842	T	TA	0.4533	NA	NA	NA	0.673833	8:143752235:A/G	45	CTD-2292P10.4	ncRNA_intronic	0.822	NA	5	14
8:143795038:C:T	8:143795038:A/G	C	T	0.03678	5.93946e-07	0.413903	0.0828937	1	8:143795038:A/G	45	CTD-2292P10.4	ncRNA_intronic	11.05	6	5	15
8:143796193:C:G	8:143796193:C/G	G	C	0.4553	2.02244e-09	0.286048	0.0477065	0.683019	8:143752235:A/G	45	CTD-2292P10.4	ncRNA_intronic	3.852	7	5	14
8:143797104:C:T	8:143797104:A/G	T	C	0.4543	2.03883e-09	0.286069	0.0477204	0.679795	8:143752235:A/G	45	CTD-2292P10.4	ncRNA_intronic	0.28	6	5	15
8:143798269:A:G	8:143798269:A/G	A	G	0.4553	1.99622e-09	0.286189	0.0477131	0.683019	8:143752235:A/G	45	CTD-2292P10.4	ncRNA_intronic	0.363	6	5	14
8:143798641:G:T	8:143798641:A/C	T	G	0.332	1.23157e-08	-0.303642	0.0533146	0.878643	8:143822208:A/G	45	CTD-2292P10.4	ncRNA_intronic	0.295	NA	5	15
8:143799457:A:G	8:143799457:A/G	A	G	0.4692	7.00344e-09	0.279262	0.048225	0.642671	8:143752235:A/G	45	CTD-2292P10.4	ncRNA_intronic	1.346	NA	1	7
8:143799496:A:G	8:143799496:A/G	A	G	0.4692	7.09483e-09	0.279154	0.0482244	0.642671	8:143752235:A/G	45	CTD-2292P10.4	ncRNA_intronic	0.099	NA	1	7
8:143799666:A:AT	rs144081504	A	AT	0.4553	NA	NA	NA	0.683019	8:143752235:A/G	45	CTD-2292P10.4	ncRNA_intronic	3.395	NA	1	15
8:143800269:A:T	8:143800269:A/T	T	A	0.4692	7.04386e-09	0.279219	0.0482255	0.642671	8:143752235:A/G	45	CTD-2292P10.4	ncRNA_intronic	2.428	6	5	15
8:143801015:G:T	8:143801015:A/C	G	T	0.4553	1.9699e-09	0.286316	0.0477174	0.683019	8:143752235:A/G	45	CTD-2292P10.4	ncRNA_intronic	13.94	NA	5	15
8:143801938:A:G	8:143801938:A/G	A	G	0.4553	1.95936e-09	0.286368	0.047719	0.683019	8:143752235:A/G	45	CTD-2292P10.4	ncRNA_intronic	2.214	7	5	15
8:143802456:A:C	8:143802456:A/C	A	C	0.332	8.30723e-09	-0.30753	0.0533714	0.878643	8:143822208:A/G	45	CTD-2292P10.4	ncRNA_intronic	2.395	5	5	15
8:143802636:A:G	8:143802636:A/G	G	A	0.4553	1.95086e-09	0.286409	0.0477203	0.683019	8:143752235:A/G	45	CTD-2292P10.4	ncRNA_intronic	12.6	6	5	15
8:143802858:A:C	8:143802858:A/C	A	C	0.02982	1.96282e-06	0.395501	0.0831371	0.809892	8:143795038:A/G	45	CTD-2292P10.4	ncRNA_intronic	3.312	7	5	15
8:143803193:C:T	8:143803193:A/G	C	T	0.4692	6.92836e-09	0.279458	0.0482436	0.642671	8:143752235:A/G	45	CTD-2292P10.4	ncRNA_intronic	4.503	NA	5	15
8:143804134:C:T	8:143804134:A/G	T	C	0.332	1.13762e-08	-0.304696	0.0533729	0.878643	8:143822208:A/G	45	CTD-2292P10.4	ncRNA_intronic	1.996	7	5	15
8:143805683:A:G	8:143805683:A/G	A	G	0.4563	1.90392e-09	0.286772	0.0477493	0.687631	8:143752235:A/G	45	CTD-2292P10.4	ncRNA_intronic	0.684	NA	5	15
8:143806069:T:TAG	rs150051936	T	TAG	0.4692	NA	NA	NA	0.642671	8:143752235:A/G	45	CTD-2292P10.4	ncRNA_intronic	0.269	NA	5	15
8:143806343:A:G	8:143806343:A/G	A	G	0.333	9.09814e-09	-0.306417	0.0533202	0.882408	8:143822208:A/G	45	CTD-2292P10.4	ncRNA_intronic	8.642	NA	5	15
8:143806464:C:G	8:143806464:C/G	G	C	0.4692	6.71584e-09	0.279857	0.0482689	0.642671	8:143752235:A/G	45	CTD-2292P10.4	ncRNA_intronic	0.567	6	5	15
8:143806896:A:G	8:143806896:A/G	G	A	0.4692	6.75602e-09	0.279808	0.0482689	0.642671	8:143752235:A/G	45	CTD-2292P10.4	ncRNA_intronic	0.063	NA	2	15
8:143807651:A:G	8:143807651:A/G	A	G	0.2664	0.007582	-0.174252	0.0652595	0.663017	8:143822208:A/G	45	CTD-2292P10.4	ncRNA_intronic	5.078	5	1	2
8:143807924:C:T	8:143807924:A/G	C	T	0.4712	7.20232e-09	0.279716	0.0483426	0.638725	8:143752235:A/G	45	CTD-2292P10.4	ncRNA_intronic	5.92	NA	1	2
8:143808175:C:G	8:143808175:C/G	G	C	0.4523	1.70047e-09	0.287941	0.0477981	0.676197	8:143752235:A/G	45	CTD-2292P10.4	ncRNA_exonic	8.316	4	1	1
8:143808667:C:T	8:143808667:A/G	C	T	0.4722	6.10431e-09	0.280837	0.0483047	0.636795	8:143752235:A/G	45	THEM6	UTR5	10.27	NA	1	1
8:143808951:C:T	8:143808951:A/G	T	C	0.4722	5.82239e-09	0.281261	0.0483118	0.636795	8:143752235:A/G	45	THEM6:CTD-2292P10.2	exonic	19.79	NA	1	1
8:143809193:C:T	8:143809193:A/G	T	C	0.4722	5.80137e-09	0.281346	0.0483215	0.636795	8:143752235:A/G	45	THEM6	exonic	21.5	NA	1	1
8:143812141:A:G	8:143812141:A/G	G	A	0.4563	1.48896e-09	0.289292	0.0478521	0.660124	8:143752235:A/G	45	THEM6	intronic	9.629	NA	5	5
8:143813513:G:T	8:143813513:A/C	T	G	0.3032	3.36108e-07	-0.285825	0.0560223	0.805111	8:143822208:A/G	45	THEM6	intronic	2.297	3a	1	5
8:143813988:A:G	8:143813988:A/G	G	A	0.4513	6.37128e-10	-0.295957	0.0478821	0.635078	8:143752235:A/G	45	THEM6	intronic	3.774	NA	1	5
8:143814193:A:G	8:143814193:A/G	G	A	0.4553	9.49475e-10	-0.292887	0.0478755	0.650232	8:143752235:A/G	45	THEM6	intronic	8.313	NA	1	5
8:143814554:A:G	8:143814554:A/G	A	G	0.3499	1.83558e-09	-0.307136	0.0510895	0.995579	8:143822208:A/G	45	THEM6	intronic	0.093	5	2	5
8:143814849:A:G	8:143814849:A/G	G	A	0.3499	1.82972e-09	-0.307151	0.0510875	0.995579	8:143822208:A/G	45	THEM6	intronic	2.298	6	4	5
8:143815697:C:CTG	rs10624245	CTG	C	0.4553	NA	NA	NA	0.650232	8:143752235:A/G	45	THEM6	intronic	4.02	NA	1	5
8:143817503:C:T	8:143817503:A/G	C	T	0.3052	6.72074e-08	-0.292574	0.0541957	0.81226	8:143822208:A/G	45	THEM6	UTR3	2.663	NA	4	5
8:143817789:A:G	8:143817789:A/G	A	G	0.3469	3.11532e-09	-0.301668	0.050911	0.982397	8:143822208:A/G	45	THEM6	UTR3	0.23	4	4	5
8:143818750:A:C	8:143818750:A/C	A	C	0.3052	6.74559e-08	-0.292458	0.0541809	0.81226	8:143822208:A/G	45	THEM6	downstream	0.088	5	4	5
8:143819383:A:G	8:143819383:A/G	G	A	0.3499	2.50312e-09	-0.303384	0.0508927	0.995579	8:143822208:A/G	45	THEM6	intergenic	0.547	4	1	5
8:143819429:T:TTGTG	rs36009328	TTGTG	T	0.3728	NA	NA	NA	0.902359	8:143822208:A/G	45	THEM6	intergenic	3.177	NA	1	5

8:143819429:TTGTG:TTGTGTG	rs587695331	TTGTG	TTGTGTG	0.003976	NA	NA	NA	0.902359	8:143822208:A/G	45	THEM6	intergenic	NA	NA	1	5
8:143819450:C:T	8:143819450:A/G	C	T	0.3082	5.27477e-08	-0.293595	0.0539519	0.830955	8:143822208:A/G	45	THEM6	intergenic	0.876	2b	1	5
8:143820410:C:G	8:143820410:C/G	C	G	0.3509	2.61886e-09	-0.302996	0.0508907	1	8:143822208:A/G	45	SLURP1	intergenic	9.02	2b	1	1
8:143822028:C:T	8:143822028:A/G	T	C	0.3062	8.47009e-08	-0.288721	0.0538979	0.815524	8:143822208:A/G	45	SLURP1	downstream	4.081	4	2	15
8:143822194:C:T	8:143822194:A/G	T	C	0.3062	8.46915e-08	-0.288711	0.0538958	0.815524	8:143822208:A/G	45	SLURP1	downstream	5.464	4	2	15
8:143822208:C:T	8:143822208:A/G	C	T	0.3509	1.82852e-09	-0.30723	0.0510998	1	8:143822208:A/G	45	SLURP1	downstream	2.829	4	2	15
8:143823863:C:G	8:143823863:C/G	C	G	0.3509	2.7116e-09	-0.302301	0.0508224	1	8:143822208:A/G	45	SLURP1	upstream	5.298	3a	5	15
8:143823876:A:G	8:143823876:A/G	A	G	0.3062	9.06093e-08	-0.287861	0.05386	0.815524	8:143822208:A/G	45	SLURP1	upstream	0.027	3a	5	15
8:143824373:A:G	8:143824373:A/G	G	A	0.3052	4.2445e-08	-0.296596	0.0541197	0.81226	8:143822208:A/G	45	SLURP1	upstream	0.762	5	5	15
8:143824483:A:G	8:143824483:A/G	G	A	0.3509	2.73774e-09	-0.302125	0.0508062	1	8:143822208:A/G	45	SLURP1	upstream	5.052	5	5	15
8:143825951:G:T	8:143825951:A/C	G	T	0.4553	1.13517e-09	-0.29067	0.0477359	0.650232	8:143752235:A/G	45	SLURP1	intergenic	2.115	5	5	15
8:143825981:G:GGATA	rs141156042	GGATA	G	0.3509	NA	NA	NA	1	8:143822208:A/G	45	SLURP1	intergenic	0.434	NA	5	15
8:143827387:A:C	8:143827387:A/C	C	A	0.3062	7.23403e-08	-0.289268	0.0537148	0.815524	8:143822208:A/G	45	SLURP1	intergenic	2.744	5	5	15
8:143829269:A:G	8:143829269:A/G	A	G	0.3042	7.87041e-08	-0.289317	0.053876	0.808342	8:143822208:A/G	45	LYPD2	intergenic	0.793	5	5	15
8:143829499:G:T	8:143829499:A/C	G	T	0.3489	3.27254e-09	-0.299755	0.0506573	0.99116	8:143822208:A/G	45	LYPD2	intergenic	1.294	5	5	15
8:143830359:C:T	8:143830359:A/G	T	C	0.3072	6.19945e-08	-0.289414	0.053467	0.827016	8:143822208:A/G	45	LYPD2	intergenic	0.283	3a	5	15
8:143830462:A:G	8:143830462:A/G	A	G	0.3489	3.37995e-09	-0.299244	0.0506165	0.99116	8:143822208:A/G	45	LYPD2	intergenic	1.06	5	5	15
8:143830599:C:T	8:143830599:A/G	T	C	0.3111	8.02982e-08	-0.288063	0.0536784	0.8083	8:143822208:A/G	45	LYPD2	downstream	2.779	NA	5	15
8:143836118:A:G	8:143836118:A/G	A	G	0.3757	2.14936e-07	-0.247158	0.0476595	0.853372	8:143844650:A/G	45	LYPD2	intergenic	1.493	NA	5	15
8:143836215:G:T	8:143836215:A/C	T	G	0.3479	1.04143e-08	-0.294076	0.0513775	0.926233	8:143822208:A/G	45	LYPD2	intergenic	5.022	NA	5	15
8:143836248:A:G	8:143836248:A/G	G	A	0.336	1.01538e-08	-0.298307	0.0520775	0.878859	8:143822208:A/G	45	LYPD2	intergenic	2.183	NA	5	15
8:143836255:C:T	8:143836255:A/G	C	T	0.336	1.01568e-08	-0.298304	0.0520775	0.878859	8:143822208:A/G	45	LYPD2	intergenic	1.742	4	5	15
8:143837219:A:G	8:143837219:A/G	G	A	0.3757	1.50891e-07	-0.251063	0.0478083	0.853372	8:143844650:A/G	45	LYPD2	intergenic	2.113	NA	5	15
8:143837749:A:G	8:143837749:A/G	A	G	0.3738	2.19596e-07	0.247087	0.0476825	0.853939	8:143844650:A/G	45	LYPD2	intergenic	0.048	5	5	15
8:143838062:A:G	8:143838062:A/G	A	G	0.3748	2.31736e-07	0.246657	0.0476918	0.857459	8:143844650:A/G	45	LYPD2	intergenic	0.108	5	2	15
8:143838519:C:T	8:143838519:A/G	C	T	0.3748	2.4686e-07	0.246117	0.0476966	0.857459	8:143844650:A/G	45	LYPD2	intergenic	0.031	4	2	15
8:143838669:A:G	8:143838669:A/G	A	G	0.3757	2.36143e-07	0.246872	0.0477658	0.853372	8:143844650:A/G	45	LYPD2	intergenic	5.039	3a	2	15
8:143839158:C:CGTGTACTTCCAGAAGGGAT	rs587743867	C	CGTGTACTTCCAGAAGGGAT	0.339	NA	NA	NA	0.743112	8:143844650:A/G	45	LYPD2	intergenic	0.193	NA	2	15
8:143839166:C:T	8:143839166:A/G	C	T	0.3748	2.25351e-07	0.246996	0.0477092	0.857459	8:143844650:A/G	45	LYPD2	intergenic	13.35	5	2	15
8:143839324:C:CTG	rs35489125	CTG	C	0.3757	NA	NA	NA	0.860606	8:143844650:A/G	45	RP11-706C16.7	intergenic	2.364	NA	2	15
8:143841249:C:T	8:143841249:A/G	T	C	0.3757	1.45584e-07	0.250379	0.0476181	0.860606	8:143844650:A/G	45	RP11-706C16.7	intergenic	1.97	7	5	15
8:143841594:C:T	8:143841594:A/G	C	T	0.4662	9.62891e-07	-0.244437	0.0498944	0.603157	8:143844650:A/G	45	RP11-706C16.7	intergenic	1.05	7	5	15
8:143842101:A:G	8:143842101:A/G	A	G	0.1531	5.06379e-07	-0.421572	0.0839136	1	8:143842101:A/G	45	RP11-706C16.7	intergenic	2.959	NA	5	15
8:143842689:A:G	8:143842689:A/G	G	A	0.3757	1.70525e-07	0.249593	0.0477334	0.860606	8:143844650:A/G	45	RP11-706C16.7	intergenic	3.207	5	5	15
8:143842901:C:T	8:143842901:A/G	C	T	0.3757	1.45953e-07	0.250358	0.0476183	0.860606	8:143844650:A/G	45	RP11-706C16.7	intergenic	4.41	NA	5	15
8:143843302:G:T	8:143843302:A/C	G	T	0.3767	1.63523e-07	-0.24927	0.0476011	0.864151	8:143844650:A/G	45	RP11-706C16.7	intergenic	0.42	3a	5	15
8:143843834:C:T	8:143843834:A/G	T	C	0.3738	1.45122e-07	0.249844	0.0475111	0.85434	8:143844650:A/G	45	RP11-706C16.7	upstream	0.904	7	5	15
8:143844051:C:T	8:143844051:A/G	T	C	0.3708	2.00374e-07	0.249235	0.0479392	0.845154	8:143844650:A/G	45	RP11-706C16.7	upstream	3.688	3a	5	15
8:143844210:C:T	8:143844210:A/G	C	T	0.3748	1.95318e-07	0.248981	0.0478467	0.835689	8:143844650:A/G	45	RP11-706C16.7	upstream	0.483	4	2	15
8:143844458:A:G	8:143844458:A/G	A	G	0.4006	1.46548e-07	0.250661	0.0476828	0.917577	8:143844650:A/G	45	RP11-706C16.7	upstream	1.603	2a	1	15

8:143844650:A:G	8:143844650:A/G	G	A	0.4135	5.79987e-08	0.258427	0.0476375	1	8:143844650:A/G	45	RP11-706C16.7	ncRNA_exonic	1.72	1b	2	15
8:143844661:C:T	8:143844661:A/G	T	C	0.3976	9.08497e-08	0.254718	0.0476632	0.937896	8:143844650:A/G	45	RP11-706C16.7	ncRNA_exonic	1.088	3a	2	15
8:143844819:C:T	8:143844819:A/G	T	C	0.3986	6.44488e-08	0.25777	0.0476825	0.94157	8:143844650:A/G	45	RP11-706C16.7	ncRNA_exonic	0.028	4	1	15
8:143845023:C:T	8:143845023:A/G	T	C	0.3966	8.26327e-08	0.255507	0.0476581	0.934256	8:143844650:A/G	45	RP11-706C16.7	ncRNA_intronic	0.613	4	1	15
8:143845269:A:G	8:143845269:A/G	A	G	0.3976	1.48025e-07	0.250958	0.0477562	0.937896	8:143844650:A/G	45	RP11-706C16.7	ncRNA_intronic	3.708	5	1	15
8:143845449:C:T	8:143845449:A/G	C	T	0.4394	5.43237e-06	0.216784	0.0476727	0.778129	8:143844650:A/G	45	RP11-706C16.7	ncRNA_intronic	0.073	5	2	15
8:143845459:A:G	8:143845459:A/G	A	G	0.4374	3.61393e-06	0.220344	0.0475654	0.784324	8:143844650:A/G	45	RP11-706C16.7	ncRNA_intronic	0.547	5	2	15
8:143845714:C:T	8:143845714:A/G	C	T	0.3817	1.62884e-07	0.250053	0.0477442	0.881341	8:143844650:A/G	45	RP11-706C16.7	ncRNA_intronic	2.423	5	2	15
8:143846593:C:T	8:143846593:A/G	C	T	0.3668	2.31957e-07	-0.260089	0.0502906	0.81036	8:143822208:A/G	45	RP11-706C16.7:LYNX1	ncRNA_intronic	5.34	2b	2	15
8:143846892:G:T	8:143846892:A/C	T	G	0.3231	4.87521e-08	-0.293603	0.0538149	0.723972	8:143856967:C/G	45	RP11-706C16.7:LYNX1	ncRNA_intronic	0.405	5	5	15
8:143846895:G:T	8:143846895:A/C	T	G	0.3231	6.97429e-08	-0.289765	0.0537415	0.723972	8:143856967:C/G	45	RP11-706C16.7:LYNX1	ncRNA_intronic	0.369	5	5	15
8:143846898:A:G	8:143846898:A/G	G	A	0.3221	1.17003e-07	-0.283703	0.0535479	0.72115	8:143856967:C/G	45	RP11-706C16.7:LYNX1	ncRNA_intronic	0.156	5	5	15
8:143846969:C:T	8:143846969:A/G	C	T	0.3658	1.54643e-06	-0.256781	0.0534387	0.850673	8:143856967:C/G	45	RP11-706C16.7:LYNX1	ncRNA_intronic	2.186	5	5	15
8:143847003:A:G	8:143847003:A/G	A	G	0.3231	5.92291e-08	-0.291475	0.0537665	0.723972	8:143856967:C/G	45	RP11-706C16.7:LYNX1	ncRNA_intronic	0.458	5	5	14
8:143847387:A:AAAAAC	rs200191799	AAAAAC	A	0.3231	NA	NA	NA	0.723972	8:143856967:C/G	45	RP11-706C16.7:LYNX1	ncRNA_intronic	0.55	NA	5	14
8:143847558:A:G	8:143847558:A/G	A	G	0.3022	6.26886e-08	-0.294506	0.0544276	0.711116	8:143822208:A/G	45	RP11-706C16.7:LYNX1	ncRNA_exonic	0.281	5	5	14
8:143848210:A:G	8:143848210:A/G	G	A	0.3201	2.9753e-08	-0.298909	0.0539267	0.730739	8:143856967:C/G	45	LYNX1	intronic	0.467	NA	5	15
8:143848302:C:T	8:143848302:A/G	T	C	0.3201	3.89641e-08	-0.297793	0.0541886	0.730739	8:143856967:C/G	45	LYNX1	intronic	1.191	NA	5	15
8:143849203:C:T	8:143849203:A/G	T	C	0.3877	2.9976e-07	0.241929	0.0472186	0.832399	8:143844650:A/G	45	LYNX1	intronic	3.504	5	1	15
8:143850340:A:G	8:143850340:A/G	G	A	0.3211	2.53094e-08	-0.304019	0.0545706	0.725933	8:143856967:C/G	45	LYNX1	intronic	2.513	5	1	15
8:143850854:A:G	8:143850854:A/G	G	A	0.329	4.68713e-09	-0.319379	0.0545208	0.657746	8:143856967:C/G	45	LYNX1	intronic	5.783	NA	2	15
8:143850858:C:G	8:143850858:C/G	C	G	0.3867	2.65316e-07	0.247651	0.0481199	0.740011	8:143844650:A/G	45	LYNX1	intronic	0.187	5	2	15
8:143851375:C:T	8:143851375:A/G	T	C	0.327	4.74182e-08	-0.300752	0.0550754	0.742022	8:143852964:C/G	45	LYNX1	intronic	4.75	4	2	15
8:143851422:C:T	8:143851422:A/G	T	C	0.4225	4.31059e-07	0.242291	0.0479336	0.674767	8:143844650:A/G	45	LYNX1	intronic	0.652	NA	5	15
8:143851755:C:G	8:143851755:C/G	G	C	0.326	3.98921e-08	-0.302913	0.0551619	0.745421	8:143852964:C/G	45	LYNX1	intronic	6.276	4	5	15
8:143852588:A:G	8:143852588:A/G	A	G	0.4175	8.22804e-06	0.213057	0.0477792	0.679576	8:143844650:A/G	45	LYNX1	intronic	0.121	1f	5	15
8:143852964:C:G	8:143852964:C/G	G	C	0.2664	1.57609e-11	-0.42854	0.0635749	1	8:143852964:C/G	45	LYNX1	UTR3	1.958	5	2	15
8:143856967:C:G	8:143856967:C/G	G	C	0.3777	9.2612e-07	-0.276569	0.0563652	1	8:143856967:C/G	45	LYNX1	intronic	6.153	5	4	15
8:143857208:A:G	8:143857208:A/G	A	G	0.1889	6.34457e-06	-0.377383	0.0835927	1	8:143857208:A/G	45	LYNX1	intronic	1.245	NA	1	15
8:143858846:G:T	8:143858846:A/C	T	G	0.3917	1.72052e-07	0.252109	0.0482296	0.757744	8:143859081:A/G	45	LYNX1	intronic	10.63	2a	1	1
8:143859081:A:G	8:143859081:A/G	A	G	0.331	5.54768e-06	0.239043	0.0526181	1	8:143859081:A/G	45	LYNX1	intronic	3.264	2b	1	1
8:143859494:A:C	8:143859494:A/C	A	C	0.4016	4.05614e-07	0.24623	0.0486012	0.740906	8:143903181:A/G	45	LYNX1	UTR5	5.359	2b	1	1
8:143861450:G:T	8:143861450:A/C	G	T	0.4016	2.32717e-07	0.24876	0.0481057	0.740787	8:143859081:A/G	45	LYNX1	intergenic	5.434	5	5	14
8:143865065:A:C	8:143865065:A/C	A	C	0.34	1.68909e-08	-0.308911	0.0547603	0.623087	8:143882420:A/G	45	LY6D	intergenic	1.214	5	5	15
8:143865768:C:T	8:143865768:A/G	T	C	0.34	1.65523e-08	-0.309081	0.0547566	0.623087	8:143882420:A/G	45	LY6D	downstream	0.3	5	2	15

8:143867157:C:T	8:143867157:A/G	T	C	0.3976	2.31279e-07	0.249171	0.0481741	0.747219	8:143903181:A/G	45	LY6D:RP11-706C16.8	ncRNA_intronic	4.432	2b	1	15
8:143867905:C:T	8:143867905:A/G	T	C	0.3996	5.02178e-07	0.241517	0.0480583	0.740617	8:143903181:A/G	45	LY6D:RP11-706C16.8	exonic	0.792	NA	1	15
8:143868977:C:T	8:143868977:A/G	C	T	0.3996	7.51755e-07	0.237971	0.0480994	0.740617	8:143903181:A/G	45	RP11-706C16.8	ncRNA_intronic	2.025	5	1	15
8:143869114:C:T	8:143869114:A/G	T	C	0.3996	5.96595e-07	0.240224	0.0481188	0.740617	8:143903181:A/G	45	RP11-706C16.8	ncRNA_intronic	7.219	NA	1	15
8:143870183:A:T	8:143870183:A/T	T	A	0.4006	4.89615e-07	0.241464	0.0480015	0.738277	8:143903181:A/G	45	RP11-706C16.8	ncRNA_intronic	0.954	5	2	15
8:143870981:A:G	8:143870981:A/G	G	A	0.4563	0.000355801	0.169544	0.0474792	0.60255	8:143902968:A/G	45	RP11-706C16.8	ncRNA_intronic	7.463	5	2	15
8:143871652:A:G	8:143871652:A/G	A	G	0.3996	6.65614e-07	0.238881	0.0480534	0.740617	8:143903181:A/G	45	RP11-706C16.8	ncRNA_intronic	7.673	NA	5	15
8:143872904:C:T	8:143872904:A/G	C	T	0.3996	6.63451e-07	0.238866	0.0480444	0.740617	8:143903181:A/G	45	RP11-706C16.8	ncRNA_intronic	6.728	NA	5	15
8:143873333:C:T	8:143873333:A/G	C	T	0.4433	1.87285e-06	0.228592	0.0479562	0.673939	8:143887054:A/G	45	RP11-706C16.8	ncRNA_intronic	4.602	NA	5	15
8:143874097:C:T	8:143874097:A/G	T	C	0.4443	2.04297e-06	0.227595	0.0479237	0.676883	8:143887054:A/G	45	RP11-706C16.8	ncRNA_intronic	0.552	NA	5	15
8:143874231:C:T	8:143874231:A/G	C	T	0.4433	1.90381e-06	0.228428	0.047955	0.673939	8:143887054:A/G	45	RP11-706C16.8	ncRNA_intronic	0.446	NA	5	15
8:143875204:A:G	8:143875204:A/G	A	G	0.4016	7.61374e-07	0.237588	0.0480458	0.740906	8:143903181:A/G	45	RP11-706C16.8	ncRNA_intronic	5.606	NA	5	15
8:143875721:A:C	8:143875721:A/C	A	C	0.3996	4.73459e-07	0.24193	0.0480326	0.740617	8:143903181:A/G	45	RP11-706C16.8	ncRNA_intronic	1.703	NA	5	15
8:143875817:A:G	8:143875817:A/G	A	G	0.3996	6.46313e-07	0.239215	0.0480655	0.740617	8:143903181:A/G	45	RP11-706C16.8	ncRNA_intronic	0.379	NA	7	15
8:143875832:C:T	8:143875832:A/G	T	C	0.3996	6.4668e-07	0.239206	0.0480649	0.740617	8:143903181:A/G	45	RP11-706C16.8	ncRNA_intronic	3.307	NA	7	15
8:143876196:A:C	8:143876196:A/C	A	C	0.4443	3.36066e-06	0.223719	0.0481381	0.676883	8:143887054:A/G	45	RP11-706C16.8	ncRNA_intronic	3.626	NA	2	15
8:143876236:A:T	8:143876236:A/T	A	T	0.4433	2.10726e-06	0.227272	0.0479189	0.673939	8:143887054:A/G	45	RP11-706C16.8	ncRNA_intronic	2.211	5	2	15
8:143877091:C:G	8:143877091:C/G	G	C	0.4463	2.8991e-06	0.223539	0.0477867	0.680402	8:143887054:A/G	45	RP11-706C16.8	ncRNA_intronic	0.608	4	2	15
8:143877275:A:G	8:143877275:A/G	A	G	0.4433	1.66342e-06	0.229247	0.0478543	0.673939	8:143887054:A/G	45	RP11-706C16.8	ncRNA_intronic	0.618	NA	6	15
8:143878733:A:G	8:143878733:A/G	G	A	0.4433	1.7802e-06	-0.228112	0.0477532	0.687273	8:143887054:A/G	45	RP11-706C16.8	ncRNA_intronic	3.831	5	5	15
8:143879290:C:CA	rs11292196	C	CA	0.3986	NA	NA	NA	0.750769	8:143903181:A/G	45	RP11-706C16.8	ncRNA_intronic	0.708	NA	5	15
8:143879795:A:G	8:143879795:A/G	A	G	0.3986	3.36548e-07	-0.242818	0.0475952	0.750769	8:143903181:A/G	45	RP11-706C16.8	ncRNA_intronic	0.921	NA	5	15
8:143879927:A:G	8:143879927:A/G	A	G	0.4652	5.56045e-06	-0.215053	0.0473429	0.847023	8:143902968:A/G	45	RP11-706C16.8	ncRNA_intronic	0.114	NA	5	15
8:143881380:C:T	8:143881380:A/G	T	C	0.4185	3.15399e-05	-0.20314	0.0488076	0.798704	8:143887054:A/G	45	RP11-706C16.8	ncRNA_intronic	1.272	5	5	15
8:143881670:A:G	8:143881670:A/G	A	G	0.3996	6.2982e-07	-0.236865	0.0475456	0.747468	8:143903181:A/G	45	RP11-706C16.8	ncRNA_intronic	2.619	NA	5	15
8:143881683:A:C	8:143881683:A/C	C	A	0.3986	5.17967e-07	-0.238473	0.0475091	0.750769	8:143903181:A/G	45	RP11-706C16.8	ncRNA_intronic	0.755	5	5	15
8:143881976:C:T	8:143881976:A/G	C	T	0.4135	1.42997e-05	-0.215542	0.0496733	0.779723	8:143887054:A/G	45	RP11-706C16.8	ncRNA_intronic	9.255	5	5	15
8:143882420:A:G	8:143882420:A/G	G	A	0.4394	9.39524e-12	-0.338567	0.0496762	1	8:143882420:A/G	45	RP11-706C16.8	ncRNA_intronic	0.535	NA	5	15
8:143884869:A:G	8:143884869:A/G	G	A	0.3986	2.56014e-07	-0.244267	0.0474004	0.750769	8:143903181:A/G	45	RP11-706C16.8	ncRNA_intronic	3.281	4	5	9
8:143884938:C:T	8:143884938:A/G	T	C	0.3986	3.24259e-07	-0.242324	0.0474329	0.750769	8:143903181:A/G	45	RP11-706C16.8	ncRNA_intronic	0.13	4	5	9
8:143884985:A:G	8:143884985:A/G	A	G	0.3429	7.87049e-09	-0.314298	0.0544596	0.646643	8:143882420:A/G	45	RP11-706C16.8	ncRNA_intronic	7.612	4	5	9
8:143885501:A:G	8:143885501:A/G	A	G	0.4205	1.98073e-05	-0.207532	0.0486359	0.81273	8:143887054:A/G	45	RP11-706C16.8	ncRNA_intronic	3.105	4	5	9
8:143885639:A:G	8:143885639:A/G	G	A	0.3986	2.90627e-07	-0.243191	0.0474109	0.750769	8:143903181:A/G	45	RP11-706C16.8	ncRNA_intronic	1.447	2b	5	9
8:143886601:A:G	8:143886601:A/G	A	G	0.4016	3.71817e-07	-0.241672	0.0475466	0.741829	8:143903181:A/G	45	RP11-706C16.8	ncRNA_intronic	2.686	1f	5	15
8:143886613:C:T	8:143886613:A/G	C	T	0.4006	3.34641e-07	-0.242645	0.0475512	0.738277	8:143903181:A/G	45	RP11-706C16.8	ncRNA_intronic	2.177	5	5	15
8:143887054:A:G	8:143887054:A/G	G	A	0.4722	4.809e-08	-0.259624	0.0475656	1	8:143887054:A/G	45	RP11-706C16.8	ncRNA_intronic	14.55	5	5	15
8:143887568:G:T	8:143887568:A/C	T	G	0.3996	2.96558e-07	-0.242935	0.0473962	0.755241	8:143903181:A/G	45	RP11-706C16.8	ncRNA_intronic	0.224	5	5	15
8:143887957:C:T	8:143887957:A/G	T	C	0.4006	2.7326e-07	-0.243428	0.0473501	0.751951	8:143903181:A/G	45	RP11-706C16.8	ncRNA_intronic	1.946	5	5	15
8:143889053:A:T	8:143889053:A/T	A	T	0.4215	5.24607e-05	-0.196619	0.0486151	0.808832	8:143887054:A/G	45	RP11-706C16.8	ncRNA_intronic	3.565	5	7	15
8:143890452:T:TGA	rs142528663	TGA	T	0.4016	NA	NA	NA	0.741829	8:143903181:A/G	45	RP11-706C16.8	ncRNA_intronic	1.664	NA	1	15
8:143895957:A:T	8:143895957:A/T	A	T	0.4245	6.53582e-05	-0.194138	0.0486246	0.797456	8:143887054:A/G	45	RP11-706C16.8	intergenic	5.204	5	5	15

8:143897882:A:G	8:143897882:A/G	G	A	0.3996	1.28325e-06	-0.229608	0.0474169	0.769054	8:143903181:A/G	45	RP11-706C16.8	intergenic	3.281	4	1	15
8:143898707:G:GAC	rs10546750	GAC	G	0.4235	NA	NA	NA	0.801196	8:143887054:A/G	45	RP11-706C16.8	intergenic	1.499	NA	1	15
8:143900135:A:G	8:143900135:A/G	G	A	0.4404	3.48654e-06	-0.220572	0.0475385	0.695425	8:143887054:A/G	45	RP11-706C16.8	intergenic	0.955	5	5	15
8:143900712:A:G	8:143900712:A/G	G	A	0.3976	1.214e-06	-0.231288	0.0476556	0.775701	8:143903181:A/G	45	RP11-706C16.8	intergenic	3.286	5	2	15
8:143902819:C:T	8:143902819:A/G	T	C	0.4354	5.49291e-06	-0.233025	0.0512703	0.939913	8:143902968:A/G	45	RP11-706C16.8	intergenic	0.659	5	5	15
8:143902901:A:T	8:143902901:A/T	A	T	0.4483	1.1746e-06	0.246014	0.0506213	0.973005	8:143902968:A/G	45	RP11-706C16.8	intergenic	1.737	5	5	15
8:143902968:C:T	8:143902968:A/G	T	C	0.4433	9.14894e-08	-0.274396	0.0513576	1	8:143902968:A/G	45	RP11-706C16.8	intergenic	0.149	5	5	15
8:143903172:A:G	8:143903172:A/G	A	G	0.3767	6.91917e-09	0.299909	0.0517721	0.965303	8:143903181:A/G	45	RP11-706C16.8	intergenic	2.286	5	5	15
8:143903181:A:G	8:143903181:A/G	A	G	0.3698	3.36128e-09	0.304835	0.0515539	1	8:143903181:A/G	45	RP11-706C16.8	intergenic	0.197	5	5	15
8:143903291:A:G	8:143903291:A/G	A	G	0.4573	1.34487e-06	0.257734	0.0533282	0.781451	8:143902968:A/G	45	RP11-706C16.8	intergenic	0.972	5	5	15
8:143903612:C:T	8:143903612:A/G	T	C	0.4036	1.83432e-07	-0.281247	0.0539263	1	8:143903612:A/G	45	RP11-706C16.8	intergenic	0.272	5	5	15
8:143924052:A:G	8:143924052:A/G	A	G	0.4891	0.000395234	0.173335	0.0489198	0.66277	8:144057069:C/G	45	GML	intronic	1.644	7	2	14
8:143925609:C:T	8:143925609:A/G	T	C	0.4841	0.000520898	0.168161	0.0484644	0.695275	8:144057069:C/G	45	GML	intronic	3.969	5	5	14
8:143926927:G:T	8:143926927:A/C	T	G	0.4841	0.000511231	0.168752	0.0485646	0.695275	8:144057069:C/G	45	GML	intronic	0.75	5	2	15
8:143928233:G:T	8:143928233:A/C	T	G	0.4841	0.000522643	0.168046	0.048444	0.695275	8:144057069:C/G	45	GML	UTR3	0.966	5	5	15
8:143931725:A:G	8:143931725:A/G	A	G	0.4821	0.000508303	0.168301	0.0484134	0.688225	8:144057069:C/G	45	GML	intronic	1.481	6	8	9
8:143935079:A:G	8:143935079:A/G	A	G	0.4821	0.000509625	0.168239	0.0484052	0.688225	8:144057069:C/G	45	GML	intronic	1.125	6	5	15
8:143938107:G:T	8:143938107:A/C	G	T	0.4821	0.000501342	0.168697	0.0484755	0.688225	8:144057069:C/G	45	GML	intronic	1.153	3a	5	14
8:143938263:C:T	8:143938263:A/G	T	C	0.4821	0.000508289	0.168243	0.0483966	0.688225	8:144057069:C/G	45	GML	intronic	4.005	5	5	14
8:143939220:G:GTGT	rs569193670	GTGT	G	0.4901	NA	NA	NA	0.615019	8:144057069:C/G	45	GML	intronic	NA	NA	5	14
8:143939224:G:GTGT	rs536677230	GTGT	G	0.4901	NA	NA	NA	0.615019	8:144057069:C/G	45	GML	intronic	1.312	NA	5	14
8:143942602:A:G	8:143942602:A/G	G	A	0.4821	0.000504493	0.168304	0.0483861	0.688225	8:144057069:C/G	45	GML	intronic	11.33	7	9	14
8:143944138:A:G	8:143944138:A/G	A	G	0.4821	0.000503358	0.168312	0.0483798	0.688225	8:144057069:C/G	45	GML	intronic	0.284	5	9	14
8:143951849:A:C	8:143951849:A/C	A	C	0.4821	0.000536536	0.167223	0.0483049	0.688225	8:144057069:C/G	45	GML	intronic	0.371	5	5	14
8:143955273:C:T	8:143955273:A/G	C	T	0.4821	0.000692971	0.163542	0.0482092	0.688225	8:144057069:C/G	45	GML:CYP11B1	UTR3	1.217	NA	1	14
8:143985583:C:T	8:143985583:A/G	T	C	0.4821	0.00636225	0.129951	0.0476263	0.642447	8:144057069:C/G	45	GML	intronic	11.43	5	5	14
8:143986436:A:T	8:143986436:A/T	A	T	0.4841	0.00451365	0.133806	0.0471174	0.646545	8:144057069:C/G	45	GML	intronic	10.77	5	5	14
8:143986555:C:T	8:143986555:A/G	C	T	0.4841	0.00431745	0.134467	0.0471155	0.646545	8:144057069:C/G	45	GML	intronic	8.266	5	5	14
8:143986701:A:G	8:143986701:A/G	G	A	0.4811	0.000618323	0.165544	0.0483561	0.690381	8:144057069:C/G	45	GML	intronic	5.415	7	5	14
8:143987191:A:G	8:143987191:A/G	A	G	0.4811	0.000631018	0.165175	0.0483262	0.690381	8:144057069:C/G	45	GML	intronic	4.563	6	5	14
8:143987619:G:T	8:143987619:A/C	T	G	0.493	0.00242761	0.1443	0.0475888	0.703869	8:144057069:C/G	45	GML	intronic	0.859	6	5	14
8:143988986:C:G	8:143988986:C/G	G	C	0.4821	0.000813841	0.156745	0.0468166	0.648244	8:144057069:C/G	45	GML	intronic	0.323	6	5	14
8:143992661:C:T	8:143992661:A/G	T	C	0.4821	0.000828976	0.155957	0.0466529	0.643284	8:144057069:C/G	45	GML:CYP11B2	UTR3	3.591	5	5	14
8:143994944:G:T	8:143994944:A/C	G	T	0.4851	0.000263451	-0.174976	0.0479542	0.705829	8:144057069:C/G	45	GML:CYP11B2	intronic	4.298	5	5	14
8:143995994:C:T	8:143995994:A/G	T	C	0.493	0.000418402	-0.167661	0.0475204	0.691172	8:144057069:C/G	45	GML:CYP11B2	intronic	0.813	5	5	14
8:143996539:C:T	8:143996539:A/G	C	T	0.4841	0.000962081	0.154887	0.0469156	0.634144	8:144057069:C/G	45	GML:CYP11B2	exonic	1.141	NA	5	14
8:143996553:A:G	8:143996553:A/G	A	G	0.4841	0.000962212	0.154886	0.0469159	0.634144	8:144057069:C/G	45	GML:CYP11B2	exonic	9.476	NA	5	14
8:143996852:A:G	8:143996852:A/G	G	A	0.492	0.000218274	0.174215	0.047125	0.641019	8:144057069:C/G	45	GML:CYP11B2	intronic	2.57	5	5	14
8:143996876:C:T	8:143996876:A/G	C	T	0.4881	0.000256522	0.172651	0.0472282	0.630895	8:144057069:C/G	45	GML:CYP11B2	intronic	2.483	5	5	14
8:143996877:G:T	8:143996877:A/C	T	G	0.4781	0.000955983	0.155188	0.0469812	0.633552	8:144057069:C/G	45	GML:CYP11B2	intronic	0.02	5	5	14
8:143996935:A:C	8:143996935:A/C	C	A	0.498	0.000326711	-0.17467	0.0486119	0.644655	8:144057069:C/G	45	GML:CYP11B2	intronic	0.154	5	5	14
8:143997415:C:T	8:143997415:A/G	C	T	0.4841	0.000997552	0.154403	0.0469138	0.634144	8:144057069:C/G	45	GML:CYP11B2	intronic	2.589	NA	5	14
8:143997472:C:T	8:143997472:A/G	C	T	0.4841	0.000999189	0.15438	0.0469133	0.634144	8:144057069:C/G	45	GML:CYP11B2	intronic	0.056	3a	5	14

8:143997509:C:CAT	rs34144193	C	CAT	0.4841	NA	NA	NA	0.634144	8:144057069:C/G	45	GML:CYP11B2	intronic	0.398	NA	5	14
8:143997877:C:G	8:143997877:C/G	G	C	0.4811	0.000728419	0.158243	0.0468361	0.646616	8:144057069:C/G	45	GML:CYP11B2	exonic	0.099	5	5	14
8:143997935:C:T	8:143997935:A/G	C	T	0.4821	0.00101362	0.155081	0.0471843	0.625209	8:144057069:C/G	45	CYP11B2	intronic	2.965	5	5	14
8:143997957:C:T	8:143997957:A/G	T	C	0.4821	0.00112951	0.153661	0.047192	0.625209	8:144057069:C/G	45	CYP11B2	intronic	0.002	5	5	14
8:143998063:C:T	8:143998063:A/G	C	T	0.4841	0.000903416	-0.155793	0.0469397	0.634144	8:144057069:C/G	45	CYP11B2	intronic	1.126	5	5	14
8:143998088:C:T	8:143998088:A/G	T	C	0.4841	0.000991327	0.154509	0.0469207	0.634144	8:144057069:C/G	45	CYP11B2	intronic	0.086	5	5	14
8:143998159:C:G	8:143998159:C/G	G	C	0.4811	0.00126343	0.151947	0.0471272	0.626772	8:144057069:C/G	45	CYP11B2	intronic	0.396	5	5	14
8:143999600:A:G	8:143999600:A/G	G	A	0.4861	0.000766746	0.157605	0.0468432	0.626753	8:144057069:C/G	45	CYP11B2	upstream	11.32	NA	5	14
8:143999726:A:G	8:143999726:A/G	A	G	0.4831	0.000792855	0.156943	0.0467751	0.639132	8:144057069:C/G	45	CYP11B2	upstream	0.5	5	5	14
8:143999919:A:T	8:143999919:A/T	A	T	0.4841	0.00104898	0.153569	0.046862	0.634144	8:144057069:C/G	45	CYP11B2	upstream	0.145	5	5	14
8:144000727:C:T	8:144000727:A/G	T	C	0.4841	0.00103549	0.153791	0.0468775	0.634144	8:144057069:C/G	45	CYP11B2	intergenic	0.459	7	5	14
8:144000768:A:G	8:144000768:A/G	A	G	0.4841	0.00112335	0.152737	0.0468857	0.634144	8:144057069:C/G	45	CYP11B2	intergenic	0.454	6	5	14
8:144000906:A:G	8:144000906:A/G	G	A	0.4831	0.000121128	-0.184825	0.0480834	0.691408	8:144057069:C/G	45	CYP11B2	intergenic	0.727	7	5	14
8:144000922:C:G	8:144000922:C/G	G	C	0.4831	0.000119441	-0.183955	0.0478143	0.691408	8:144057069:C/G	45	CYP11B2	intergenic	6.547	6	5	14
8:144002283:G:T	8:144002283:A/C	T	G	0.4841	0.000996749	0.154418	0.046915	0.634144	8:144057069:C/G	45	CYP11B2	intergenic	0.454	5	5	14
8:144003138:C:G	8:144003138:C/G	C	G	0.4851	0.000849501	0.1567	0.0469705	0.630012	8:144057069:C/G	45	CYP11B2	intergenic	1.722	2b	5	13
8:144003207:A:G	8:144003207:A/G	A	G	0.4911	0.000189748	0.175887	0.0471257	0.644237	8:144057069:C/G	45	CYP11B2	intergenic	0.231	4	5	14
8:144003290:A:G	8:144003290:A/G	G	A	0.494	0.00025831	0.172518	0.0472152	0.632073	8:144057069:C/G	45	CYP11B2	intergenic	0.834	4	5	14
8:144003917:T:TC	rs10717492	TC	T	0.494	NA	NA	NA	0.632073	8:144057069:C/G	45	CYP11B2	intergenic	2.89	NA	5	14
8:144005260:A:C	8:144005260:A/C	C	A	0.4751	0.000466832	0.16479	0.047095	0.674094	8:144057069:C/G	45	CYP11B2	intergenic	0.02	4	5	14
8:144006073:A:G	8:144006073:A/G	G	A	0.498	0.00010302	-0.184819	0.0475925	0.709892	8:144057069:C/G	45	CYP11B2	intergenic	0.959	5	5	14
8:144009349:A:T	8:144009349:A/T	T	A	0.4771	0.000272154	0.172172	0.0472939	0.659596	8:144057069:C/G	45	CYP11B2	intergenic	9.956	5	5	14
8:144009765:C:T	8:144009765:A/G	T	C	0.4791	0.00161711	0.149858	0.0475318	0.651105	8:144057069:C/G	45	CYP11B2	intergenic	0.927	4	5	14
8:144014045:A:AGAAAAGTTT	rs200655985	A	AGAAAAGTTT	0.499	NA	NA	NA	0.712749	8:144057069:C/G	45	CYP11B2	intergenic	11.53	NA	5	14
8:144015486:C:T	8:144015486:A/G	T	C	0.5	0.000169192	-0.179583	0.0477479	0.721888	8:144057069:C/G	45	CYP11B2	intergenic	1.898	7	7	15
8:144015662:C:T	8:144015662:A/G	C	T	0.5	0.000169299	-0.179584	0.0477501	0.721888	8:144057069:C/G	45	CYP11B2	intergenic	0.056	5	7	15
8:144017045:C:CTT	rs373569443	CTT	C	0.4891	NA	NA	NA	0.717944	8:144057069:C/G	45	CYP11B2	intergenic	4.622	NA	5	9
8:144018250:G:GTGTC	rs370135383	GTGTC	G	0.4712	NA	NA	NA	0.684704	8:144057069:C/G	45	CYP11B2	intergenic	2.04	NA	7	15
8:144018254:C:G	8:144018254:C/G	G	C	0.4513	0.000278354	0.172888	0.0475667	0.608811	8:144057069:C/G	45	CYP11B2	intergenic	2.626	NA	7	15
8:144020271:C:T	8:144020271:A/G	T	C	0.4732	0.000262054	0.171942	0.047105	0.683398	8:144057069:C/G	45	CYP11B2	intergenic	5.243	5	5	15
8:144020500:C:T	8:144020500:A/G	C	T	0.494	6.16705e-05	0.189095	0.047199	0.740383	8:144057069:C/G	45	CYP11B2	intergenic	5.978	5	5	15
8:144023756:A:G	8:144023756:A/G	G	A	0.499	7.69203e-05	-0.187493	0.0474208	0.776437	8:144057069:C/G	45	CYP11B2	intergenic	4.218	5	5	15
8:144031637:A:G	8:144031637:A/G	G	A	0.499	3.12336e-05	-0.197189	0.0473524	0.789318	8:144057069:C/G	45	RP11-273G15.2	intergenic	6.477	5	9	15
8:144034815:A:G	8:144034815:A/G	A	G	0.496	4.9005e-05	-0.193158	0.0475723	0.788715	8:144057069:C/G	45	RP11-273G15.2	intergenic	1.18	4	5	15
8:144035121:G:T	8:144035121:A/C	G	T	0.5	2.75037e-05	-0.197535	0.0471084	0.785182	8:144057069:C/G	45	RP11-273G15.2	intergenic	0.645	2b	5	15
8:144035499:A:G	8:144035499:A/G	A	G	0.4682	0.000178617	0.176008	0.0469669	0.720463	8:144057069:C/G	45	RP11-273G15.2	intergenic	2.811	5	5	15
8:144036416:C:G	8:144036416:C/G	C	G	0.4682	0.000146774	0.178441	0.0470017	0.720463	8:144057069:C/G	45	RP11-273G15.2	intergenic	2.328	5	5	15
8:144037500:A:G	8:144037500:A/G	G	A	0.497	3.26292e-05	-0.195826	0.0471382	0.786336	8:144057069:C/G	45	RP11-273G15.2	intergenic	2.701	NA	5	15
8:144037815:A:G	8:144037815:A/G	A	G	0.4692	0.000159105	0.177406	0.0469773	0.722735	8:144057069:C/G	45	RP11-273G15.2	intergenic	0.493	6	5	15
8:144038623:A:G	8:144038623:A/G	G	A	0.499	2.09497e-05	-0.200687	0.0471704	0.796061	8:144057069:C/G	45	RP11-273G15.2	intergenic	2.194	NA	5	15
8:144038731:A:G	8:144038731:A/G	A	G	0.497	3.27145e-05	-0.195854	0.0471517	0.786336	8:144057069:C/G	45	RP11-273G15.2	intergenic	1.362	NA	5	15
8:144042624:A:T	8:144042624:A/T	T	A	0.4702	0.000157587	0.177608	0.0470011	0.72502	8:144057069:C/G	45	RP11-273G15.2	intergenic	0.969	5	5	15

8:144049868:A:C	8:144049868:A/C	A	C	0.4682	0.000176335	0.176648	0.0470971	0.73388	8:144057069:C/G	45	RP11-273G15.2	intergenic	0.535	6	7	15
8:144051099:A:ATTT	rs200401958	A	ATTT	0.4791	NA	NA	NA	0.603192	8:144057069:C/G	45	RP11-273G15.2	intergenic	1.477	NA	7	15
8:144051193:A:G	8:144051193:A/G	A	G	0.4672	0.000180749	0.176399	0.0471088	0.73162	8:144057069:C/G	45	RP11-273G15.2	intergenic	2.611	6	7	15
8:144051836:A:C	8:144051836:A/C	A	C	0.4662	0.000195416	0.175433	0.0470978	0.742795	8:144057069:C/G	45	RP11-273G15.2	intergenic	0.641	5	7	15
8:144052791:A:G	8:144052791:A/G	A	G	0.4662	0.000200234	0.175195	0.0471118	0.742795	8:144057069:C/G	45	RP11-273G15.2	intergenic	6.144	NA	8	15
8:144054291:C:T	8:144054291:A/G	T	C	0.4662	0.000231567	0.173297	0.0470682	0.742795	8:144057069:C/G	45	RP11-273G15.2	intergenic	1.365	NA	9	9
8:144054517:A:G	8:144054517:A/G	G	A	0.4662	0.000198124	0.175149	0.0470655	0.742795	8:144057069:C/G	45	RP11-273G15.2	intergenic	8.901	2b	9	9
8:144055871:A:G	8:144055871:A/G	A	G	0.499	5.2191e-05	-0.193177	0.04775	0.822545	8:144057069:C/G	45	RP11-273G15.2	intergenic	6.434	NA	9	9
8:144056123:A:G	8:144056123:A/G	G	A	0.498	4.97671e-05	-0.193545	0.0477098	0.820187	8:144057069:C/G	45	RP11-273G15.2	intergenic	2.656	NA	9	9
8:144056229:A:C	8:144056229:A/C	A	C	0.496	5.71731e-05	-0.192026	0.047718	0.80882	8:144057069:C/G	45	RP11-273G15.2	intergenic	3.257	NA	9	9
8:144056237:C:T	8:144056237:A/G	T	C	0.496	5.71695e-05	-0.192026	0.0477179	0.80882	8:144057069:C/G	45	RP11-273G15.2	intergenic	4.372	NA	9	9
8:144056247:C:T	8:144056247:A/G	T	C	0.496	5.71316e-05	-0.192034	0.047718	0.80882	8:144057069:C/G	45	RP11-273G15.2	intergenic	5.443	NA	9	9
8:144056424:A:G	8:144056424:A/G	A	G	0.499	5.04445e-05	-0.193538	0.0477453	0.822545	8:144057069:C/G	45	RP11-273G15.2	intergenic	8.265	3a	9	15
8:144056432:C:T	8:144056432:A/G	T	C	0.498	4.84434e-05	-0.194688	0.0479175	0.81015	8:144057069:C/G	45	RP11-273G15.2	intergenic	7.564	4	9	15
8:144056585:G:T	8:144056585:A/C	T	G	0.4642	6.44602e-05	-0.200912	0.0502802	0.710351	8:144057069:C/G	45	RP11-273G15.2	intergenic	6.11	4	9	15
8:144056604:C:T	8:144056604:A/G	T	C	0.492	4.79634e-05	-0.195279	0.0480352	0.804689	8:144057069:C/G	45	RP11-273G15.2	intergenic	8.508	4	9	15
8:144056634:C:T	8:144056634:A/G	T	C	0.4463	0.000375611	-0.181251	0.0509611	0.683954	8:144057069:C/G	45	RP11-273G15.2	intergenic	8.854	4	9	15
8:144056742:G:T	8:144056742:A/C	T	G	0.3946	0.000293593	-0.200681	0.0554231	0.771602	8:144057069:C/G	45	RP11-273G15.2	intergenic	0.072	4	9	15
8:144056869:A:T	8:144056869:A/T	A	T	0.4583	3.11827e-05	-0.209443	0.0502907	0.912255	8:144057069:C/G	45	RP11-273G15.2	intergenic	6.226	NA	9	15
8:144056979:A:G	8:144056979:A/G	A	G	0.4443	1.47447e-05	-0.2213	0.0510797	0.976545	8:144057069:C/G	45	RP11-273G15.2	intergenic	6.167	2b	9	15
8:144057010:A:G	8:144057010:A/G	A	G	0.4463	8.27037e-06	-0.227462	0.0510225	0.983308	8:144057069:C/G	45	RP11-273G15.2	intergenic	1.024	2b	9	15
8:144057040:C:G	8:144057040:C/G	G	C	0.4463	9.3766e-06	-0.226468	0.0511093	0.983308	8:144057069:C/G	45	RP11-273G15.2	intergenic	5.111	5	9	15
8:144057069:C:G	8:144057069:C/G	G	C	0.4414	7.15668e-06	-0.230926	0.0514427	1	8:144057069:C/G	45	RP11-273G15.2	intergenic	0.202	5	9	15
8:144057098:A:G	8:144057098:A/G	G	A	0.5	5.66945e-05	-0.192273	0.0477557	0.825147	8:144057069:C/G	45	RP11-273G15.2	intergenic	7.643	5	9	15
8:144057287:C:T	8:144057287:A/G	T	C	0.499	5.36146e-05	-0.192744	0.0477174	0.822545	8:144057069:C/G	45	RP11-273G15.2	intergenic	6.623	5	9	15
8:144057376:C:T	8:144057376:A/G	C	T	0.499	5.46328e-05	-0.192506	0.0477106	0.822545	8:144057069:C/G	45	RP11-273G15.2	intergenic	8.986	7	9	15
8:144057443:C:G	8:144057443:C/G	G	C	0.4245	0.00208471	-0.160189	0.0520449	0.609113	8:144057069:C/G	45	RP11-273G15.2	intergenic	4.387	5	9	15
8:144057865:C:T	8:144057865:A/G	T	C	0.4245	0.00210768	-0.160014	0.0520435	0.609113	8:144057069:C/G	45	RP11-273G15.2	intergenic	6.733	5	7	15
8:144057899:C:T	8:144057899:A/G	C	T	0.4245	0.00210837	-0.160009	0.0520435	0.609113	8:144057069:C/G	45	RP11-273G15.2	intergenic	6.515	5	7	15
8:144057920:C:CTGAG	rs146554497	CTGAG	C	0.4662	NA	NA	NA	0.742795	8:144057069:C/G	45	RP11-273G15.2	intergenic	1.798	NA	7	15
8:144058126:A:C	8:144058126:A/C	C	A	0.4245	0.00212094	-0.159915	0.0520427	0.609113	8:144057069:C/G	45	RP11-273G15.2	intergenic	6.569	5	9	15
8:144058347:A:G	8:144058347:A/G	A	G	0.4702	0.000149548	0.180677	0.0476491	0.719404	8:144057069:C/G	45	RP11-273G15.2	intergenic	0.078	NA	9	15
8:144058355:A:G	8:144058355:A/G	A	G	0.4254	0.00257228	-0.15704	0.0520913	0.606702	8:144057069:C/G	45	RP11-273G15.2	intergenic	1.957	5	9	15
8:144058386:C:G	8:144058386:C/G	C	G	0.4662	0.000217691	0.174191	0.0471097	0.742795	8:144057069:C/G	45	RP11-273G15.2	intergenic	2.491	5	9	15
8:144058531:A:G	8:144058531:A/G	G	A	0.4245	0.00224978	-0.158992	0.0520414	0.609113	8:144057069:C/G	45	RP11-273G15.2	intergenic	4.989	NA	9	15
8:144058542:C:T	8:144058542:A/G	T	C	0.4245	0.00225066	-0.158986	0.0520415	0.609113	8:144057069:C/G	45	RP11-273G15.2	intergenic	3.657	NA	9	15
8:144058625:A:G	8:144058625:A/G	A	G	0.4245	0.00225583	-0.15895	0.052041	0.609113	8:144057069:C/G	45	RP11-273G15.2	intergenic	2.158	NA	7	15
8:144058649:C:G	8:144058649:C/G	C	G	0.499	5.66274e-05	-0.192128	0.0477166	0.822545	8:144057069:C/G	45	RP11-273G15.2	intergenic	2.401	5	7	15
8:144058656:G:T	8:144058656:A/C	T	G	0.4245	0.00225659	-0.158943	0.0520408	0.609113	8:144057069:C/G	45	RP11-273G15.2	intergenic	0.921	NA	7	15
8:144059526:C:T	8:144059526:A/G	C	T	0.498	7.08373e-05	-0.190562	0.0479589	0.813696	8:144057069:C/G	45	RP11-273G15.2	intergenic	0.001	NA	5	15
8:144059545:C:T	8:144059545:A/G	T	C	0.497	6.71329e-05	-0.19125	0.0479778	0.811115	8:144057069:C/G	45	RP11-273G15.2	intergenic	1.747	NA	5	15
8:144059693:C:T	8:144059693:A/G	C	T	0.4682	0.000325005	0.169635	0.0471929	0.746806	8:144057069:C/G	45	RP11-273G15.2	intergenic	0.136	6	5	15
8:144059809:A:ACT	rs111941540	ACT	A	0.499	NA	NA	NA	0.822545	8:144057069:C/G	45	RP11-273G15.2	intergenic	2.117	NA	5	15

8:144059912:A:G	8:144059912:A/G	A	G	0.4662	0.000226092	0.173733	0.0471086	0.742795	8:144057069:C/G	45	RP11-273G15.2	intergenic	0.537	5	5	15
8:144064275:C:T	8:144064275:A/G	T	C	0.4662	0.000478038	0.165458	0.0473716	0.742795	8:144057069:C/G	45	RP11-273G15.2	ncRNA_intronic	5.065	6	5	15
8:144064882:C:T	8:144064882:A/G	T	C	0.4314	0.00563401	0.132757	0.0479549	0.657091	8:144057069:C/G	45	RP11-273G15.2	ncRNA_intronic	1.512	5	2	15
9:74633850:C:G	9:74633850:C/G	C	G	0.02584	0.00579898	0.723494	0.262237	0.728841	9:74675922:A/G	46	HSPB1P1	intergenic	0.046	7	5	15
9:74675922:A:G	9:74675922:A/G	A	G	0.03479	2.06758e-05	0.78307	0.183929	1	9:74675922:A/G	46	C9orf57	intronic	4.455	6	14	15
9:74678088:A:G	9:74678088:A/G	A	G	0.02883	0.000592075	0.901692	0.262484	0.818083	9:74675922:A/G	46	C9orf57	intronic	1.667	6	14	15
9:74683283:A:G	9:74683283:A/G	A	G	0.03181	0.000159121	0.723934	0.1917	0.848462	9:74675922:A/G	46	C9orf57	intronic	2.139	6	9	15
9:74688612:C:T	9:74688612:A/G	C	T	0.03181	0.00019397	0.714179	0.191636	0.848462	9:74675922:A/G	46	C9orf57	upstream	0.579	6	9	15
9:74690582:C:G	9:74690582:C/G	G	C	0.03479	3.44099e-05	0.753335	0.181871	0.939527	9:74675922:A/G	46	C9orf57	intergenic	0.46	7	9	15
9:74691888:A:G	9:74691888:A/G	G	A	0.03479	3.74472e-05	0.749778	0.181866	0.939527	9:74675922:A/G	46	C9orf57	intergenic	0.975	7	9	15
9:74692072:A:C	9:74692072:A/C	C	A	0.03479	0.000639971	0.643153	0.188382	0.939527	9:74675922:A/G	46	C9orf57	intergenic	0.512	7	9	15
9:74692163:A:C	9:74692163:A/C	A	C	0.03479	3.39518e-05	-0.754051	0.181909	0.939527	9:74675922:A/G	46	C9orf57	intergenic	0.471	7	9	15
9:74692939:A:G	9:74692939:A/G	A	G	0.04076	0.000418242	-0.62365	0.176757	0.791101	9:74675922:A/G	46	C9orf57	intergenic	5.956	7	9	15
9:74701425:A:G	9:74701425:A/G	A	G	0.03678	0.000841423	0.537125	0.160874	0.884703	9:74675922:A/G	46	C9orf57	intergenic	5.101	7	14	15
9:74738162:C:G	9:74738162:C/G	C	G	0.03678	0.00105239	0.521394	0.159149	0.884703	9:74675922:A/G	46	GDA	intronic	1.06	6	5	15
9:74769627:A:G	9:74769627:A/G	A	G	0.03579	0.00123669	0.505303	0.156427	0.799283	9:74675922:A/G	46	GDA	intronic	4.763	4	1	15
9:74784162:C:T	9:74784162:A/G	C	T	0.03479	0.00138065	0.501223	0.156698	0.769422	9:74675922:A/G	46	GDA	intronic	2.831	7	4	15
9:74795354:A:G	9:74795354:A/G	A	G	0.03479	0.00138986	0.500759	0.156646	0.769422	9:74675922:A/G	46	GDA	intronic	2.088	6	4	15
9:74795603:A:G	9:74795603:A/G	G	A	0.03479	0.00182865	0.498661	0.159994	0.769422	9:74675922:A/G	46	GDA	intronic	0.612	7	4	15
9:74807723:A:G	9:74807723:A/G	G	A	0.03479	0.00214394	0.457692	0.149108	0.769422	9:74675922:A/G	46	GDA	intronic	1.101	5	5	15
9:74813492:A:G	9:74813492:A/G	A	G	0.03479	0.00245343	0.446109	0.147278	0.769422	9:74675922:A/G	46	GDA	intronic	0.235	7	4	15
9:74813577:C:CAT	rs34517745	CAT	C	0.0338	NA	NA	NA	0.739714	9:74675922:A/G	46	GDA	intronic	0.512	NA	4	15
9:74815070:C:T	9:74815070:A/G	C	T	0.03181	0.0170675	0.376058	0.157659	0.680759	9:74675922:A/G	46	GDA	intronic	3.328	7	4	15
9:74815128:A:G	9:74815128:A/G	G	A	0.02982	0.0349753	0.440735	0.209013	0.675577	9:74675922:A/G	46	GDA	intronic	1.645	7	4	15
9:74816398:A:G	9:74816398:A/G	G	A	0.03479	0.0125891	0.351358	0.140815	0.769422	9:74675922:A/G	46	GDA	intronic	1.723	7	4	15
9:74819270:A:G	9:74819270:A/G	G	A	0.03579	0.0144013	0.337711	0.138005	0.746004	9:74675922:A/G	46	GDA	intronic	0.42	5	4	15
9:74828114:G:T	9:74828114:A/C	T	G	0.02982	0.0462878	0.412613	0.207057	0.675577	9:74675922:A/G	46	GDA	intronic	2.79	7	4	15
9:74828339:A:G	9:74828339:A/G	G	A	0.03777	0.0329188	0.278908	0.130754	0.702864	9:74675922:A/G	46	GDA	intronic	13.55	6	4	15
9:74833123:A:C	9:74833123:A/C	C	A	0.04274	0.048895	0.228829	0.116186	0.612574	9:74675922:A/G	46	GDA	intronic	0.11	6	4	15
9:104949003:A:G	9:104949003:A/G	G	A	0.04672	0.0181663	0.309748	0.131126	0.639408	9:104962227:A/G	47	RNU6-329P	intergenic	3.784	7	5	15
9:104950328:A:T	9:104950328:A/T	T	A	0.04771	0.0099141	0.334441	0.129688	0.654341	9:104962227:A/G	47	RNU6-329P	intergenic	7.153	7	7	15
9:104950965:G:T	9:104950965:A/C	G	T	0.04771	0.00976757	0.334973	0.129636	0.654341	9:104962227:A/G	47	RNU6-329P	intergenic	4.46	7	9	15
9:104951348:C:T	9:104951348:A/G	C	T	0.06759	3.58285e-05	0.320049	0.07744	0.96738	9:104962227:A/G	47	RNU6-329P	intergenic	0.628	7	9	15
9:104952086:A:G	9:104952086:A/G	A	G	0.04672	0.0113181	0.328697	0.12978	0.639408	9:104962227:A/G	47	RNU6-329P	intergenic	2.319	7	9	15
9:104952088:A:T	9:104952088:A/T	T	A	0.04771	0.00953267	0.335825	0.129546	0.654341	9:104962227:A/G	47	RNU6-329P	intergenic	1.408	7	9	15
9:104952502:A:G	9:104952502:A/G	G	A	0.04771	0.00961346	0.335352	0.129508	0.654341	9:104962227:A/G	47	RNU6-329P	intergenic	0.171	7	9	15
9:104955832:A:G	9:104955832:A/G	A	G	0.04573	0.0121957	0.32489	0.129623	0.655545	9:104962227:A/G	47	RNU6-329P	intergenic	0.605	6	9	15
9:104955959:A:C	9:104955959:A/C	A	C	0.04573	0.0121798	0.324937	0.129617	0.655545	9:104962227:A/G	47	RNU6-329P	intergenic	0.896	7	9	15
9:104958748:C:T	9:104958748:A/G	T	C	0.04672	0.0154232	0.315762	0.130357	0.639408	9:104962227:A/G	47	RNU6-329P	intergenic	0.038	6	9	15
9:104959379:C:T	9:104959379:A/G	T	C	0.04672	0.0155538	0.315345	0.13035	0.639408	9:104962227:A/G	47	RNU6-329P	intergenic	1.38	7	9	15
9:104961694:C:T	9:104961694:A/G	T	C	0.04672	0.0147377	0.317204	0.130068	0.670484	9:104962227:A/G	47	RNU6-329P	intergenic	0.266	7	9	15
9:104961743:A:T	9:104961743:A/T	A	T	0.04672	0.0102271	0.331416	0.129053	0.670484	9:104962227:A/G	47	RNU6-329P	intergenic	6.292	6	9	15
9:104962227:A:G	9:104962227:A/G	G	A	0.06759	3.36722e-05	0.317159	0.0764775	1	9:104962227:A/G	47	RNU6-329P	intergenic	2.954	7	9	15

9:104962230:A:C	9:104962230:A/C	C	A	0.04573	0.0122456	0.323733	0.129235	0.655545	9:104962227:A/G	47	RNU6-329P	intergenic	2.555	7	9	15
9:104962942:C:T	9:104962942:A/G	C	T	0.04573	0.0209346	0.301754	0.130677	0.655545	9:104962227:A/G	47	RNU6-329P	intergenic	1.02	6	9	15
9:104962943:C:T	9:104962943:A/G	C	T	0.04573	0.0209511	0.301721	0.13068	0.655545	9:104962227:A/G	47	RNU6-329P	intergenic	0.245	6	9	15
9:104963004:C:T	9:104963004:A/G	C	T	0.04573	0.0213315	0.30104	0.13077	0.655545	9:104962227:A/G	47	RNU6-329P	intergenic	2.434	7	9	15
9:104967678:A:G	9:104967678:A/G	G	A	0.04573	0.0486686	0.249628	0.12662	0.655545	9:104962227:A/G	47	RNU6-329P	intergenic	0.365	5	9	15
9:104969387:A:G	9:104969387:A/G	G	A	0.04672	0.0294145	0.28639	0.131499	0.639408	9:104962227:A/G	47	RNU6-329P	intergenic	0.425	6	9	15
9:104969611:C:G	9:104969611:C/G	G	C	0.04672	0.0294047	0.286404	0.131498	0.639408	9:104962227:A/G	47	RNU6-329P	intergenic	6.74	6	9	15
9:104969679:A:G	9:104969679:A/G	G	A	0.04672	0.0294002	0.286411	0.131497	0.639408	9:104962227:A/G	47	RNU6-329P	intergenic	2.8	6	9	15
9:104971264:A:G	9:104971264:A/G	A	G	0.04672	0.0292541	0.286631	0.131479	0.639408	9:104962227:A/G	47	RNU6-329P	intergenic	0.097	6	9	15
9:104971265:C:G	9:104971265:C/G	G	C	0.04672	0.0292481	0.286642	0.13148	0.639408	9:104962227:A/G	47	RNU6-329P	intergenic	0.02	6	9	15
9:104971423:C:T	9:104971423:A/G	C	T	0.04672	0.0292466	0.286623	0.13147	0.639408	9:104962227:A/G	47	RNU6-329P	intergenic	0.219	5	1	15
9:104972141:A:G	9:104972141:A/G	G	A	0.04672	0.029204	0.286689	0.131465	0.639408	9:104962227:A/G	47	RNU6-329P	intergenic	1.576	6	9	15
9:104973434:A:G	9:104973434:A/G	A	G	0.04672	0.0291783	0.286748	0.131471	0.639408	9:104962227:A/G	47	RNU6-329P	intergenic	0.344	5	9	15
9:104973626:A:G	9:104973626:A/G	A	G	0.04672	0.0312646	0.283114	0.131455	0.639408	9:104962227:A/G	47	RNU6-329P	intergenic	0.007	NA	9	15
9:104973771:A:G	9:104973771:A/G	A	G	0.04672	0.0351317	0.276418	0.1312	0.639408	9:104962227:A/G	47	RNU6-329P	intergenic	1.002	NA	9	15
9:104974084:A:T	9:104974084:A/T	T	A	0.04672	0.028957	0.287178	0.131488	0.639408	9:104962227:A/G	47	RNU6-329P	intergenic	0.724	7	9	15
9:104974087:A:G	9:104974087:A/G	G	A	0.04672	0.028957	0.287178	0.131488	0.639408	9:104962227:A/G	47	RNU6-329P	intergenic	6.348	7	9	15
9:104974205:G:T	9:104974205:A/C	G	T	0.04672	0.0289039	0.287303	0.131501	0.639408	9:104962227:A/G	47	RNU6-329P	intergenic	0.087	6	9	15
9:104974790:A:G	9:104974790:A/G	G	A	0.04672	0.0286423	0.287901	0.131559	0.639408	9:104962227:A/G	47	RNU6-329P	intergenic	0.733	6	9	15
9:104975065:A:G	9:104975065:A/G	A	G	0.04672	0.0285162	0.288185	0.131585	0.639408	9:104962227:A/G	47	RNU6-329P	intergenic	0.451	7	9	15
9:104975447:A:T	9:104975447:A/T	A	T	0.04672	0.0283432	0.288576	0.131619	0.639408	9:104962227:A/G	47	RNU6-329P	intergenic	0.083	7	9	15
9:104976635:G:T	9:104976635:A/C	T	G	0.04672	0.0278338	0.28972	0.131714	0.639408	9:104962227:A/G	47	RNU6-329P	intergenic	0.54	6	9	15
9:104979091:A:G	9:104979091:A/G	A	G	0.04672	0.0292338	0.288037	0.132107	0.639408	9:104962227:A/G	47	RNU6-329P	intergenic	1.251	6	9	15
9:104979615:C:T	9:104979615:A/G	C	T	0.04672	0.0297675	0.2873	0.132203	0.639408	9:104962227:A/G	47	RNU6-329P	intergenic	1.358	7	9	15
9:104980347:A:C	9:104980347:A/C	A	C	0.04672	0.0305116	0.28649	0.132427	0.639408	9:104962227:A/G	47	RNU6-329P	intergenic	0.285	6	15	15
9:104980376:G:GT	rs573755354	G	GT	0.04573	NA	NA	NA	0.624541	9:104962227:A/G	47	RNU6-329P	intergenic	0.507	NA	15	15
9:104982511:G:T	9:104982511:A/C	T	G	0.04672	0.0317505	0.284913	0.132669	0.639408	9:104962227:A/G	47	RNU6-329P	intergenic	0.957	7	15	15
9:112539953:C:T	9:112539953:A/G	T	C	0.3926	5.51506e-06	-0.21807	0.047989	1	9:112539953:A/G	48	PALM2	intronic	6.803	5	5	14
10:45143639:A:G	10:45143639:A/G	G	A	0.001988	0.00103432	0.713829	0.217563	0.665336	10:45221505:A/G	49	RP11-733D4.1	intergenic	12.99	NA	5	15
10:45147047:C:T	10:45147047:A/G	T	C	0.001988	0.00150494	0.691685	0.217941	0.665336	10:45221505:A/G	49	RP11-733D4.1	intergenic	0.249	NA	2	15
10:45162852:A:G	10:45162852:A/G	A	G	0.001988	0.000751992	0.72911	0.21636	0.665336	10:45221505:A/G	49	RP11-733D4.1	intergenic	5.418	5	5	15
10:45165599:AAAA:CAAA	rs539790580	CAAA	AAAA	0.000994	NA	NA	NA	0.665336	10:45221505:A/G	49	RP11-733D4.1	intergenic	NA	NA	5	15
10:45165599:C:CAAA	rs61262799	CAAA	C	0.001988	NA	NA	NA	0.665336	10:45221505:A/G	49	RP11-733D4.1	intergenic	12.88	NA	5	15
10:45165642:A:G	10:45165642:A/G	G	A	0.001988	0.000734729	0.729971	0.216206	0.665336	10:45221505:A/G	49	RP11-733D4.1	intergenic	13.96	6	5	15
10:45171582:C:T	10:45171582:A/G	T	C	0.001988	0.000918074	0.721075	0.217552	0.665336	10:45221505:A/G	49	RP11-733D4.1	intergenic	1.093	7	5	15
10:45183876:C:T	10:45183876:A/G	C	T	0.001988	0.00114541	0.704286	0.216561	0.665336	10:45221505:A/G	49	RP11-733D4.1	intergenic	0.273	6	5	15
10:45187031:A:G	10:45187031:A/G	A	G	0.001988	0.00122384	0.701314	0.216905	0.665336	10:45221505:A/G	49	RP11-733D4.1	intergenic	0.349	5	14	15
10:45193060:A:G	10:45193060:A/G	A	G	0.002982	4.33773e-05	0.797859	0.195137	1	10:45221505:A/G	49	RP11-733D4.1	intergenic	0.101	3a	5	15
10:45194727:C:T	10:45194727:A/G	C	T	0.002982	4.3248e-05	0.797278	0.194962	1	10:45221505:A/G	49	RP11-733D4.1	intergenic	6.928	7	5	15
10:45194916:A:G	10:45194916:A/G	A	G	0.002982	4.32486e-05	0.798009	0.195141	1	10:45221505:A/G	49	RP11-733D4.1	intergenic	0.464	6	5	15
10:45195541:C:T	10:45195541:A/G	T	C	0.002982	4.31085e-05	0.798182	0.195147	1	10:45221505:A/G	49	RP11-733D4.1	intergenic	2.638	7	8	15
10:45195716:A:T	10:45195716:A/T	A	T	0.002982	4.31539e-05	0.798122	0.195144	1	10:45221505:A/G	49	RP11-733D4.1	intergenic	0.528	6	5	15

10:45196611:C:T	10:45196611:A/G	C	T	0.002982	4.29691e-05	0.797671	0.194986	1	10:45221505:A/G	49	RP11-733D4.1	intergenic	1.086	7	5	15
10:45197293:A:G	10:45197293:A/G	G	A	0.002982	4.2885e-05	0.797769	0.194989	1	10:45221505:A/G	49	RP11-733D4.1	intergenic	4.78	5	5	15
10:45199031:C:T	10:45199031:A/G	T	C	0.002982	4.44417e-05	0.79809	0.195462	1	10:45221505:A/G	49	RP11-733D4.1	intergenic	4.73	3a	2	15
10:45200119:C:G	10:45200119:C/G	G	C	0.002982	3.77879e-05	-0.802471	0.194746	1	10:45221505:A/G	49	EIF2AP4	intergenic	0.109	7	5	15
10:45202831:A:G	10:45202831:A/G	A	G	0.002982	3.75995e-05	0.802516	0.194702	1	10:45221505:A/G	49	EIF2AP4	intergenic	0.477	NA	2	15
10:45204500:A:ATTTGTTTTCAACCTCAG	rs11272320	ATTTGTTTTCAACCTCAG	A	0.002982	NA	NA	NA	1	10:45221505:A/G	49	EIF2AP4	intergenic	3.614	NA	5	15
10:45205038:T:TA	rs59814141	T	TA	0.002982	NA	NA	NA	1	10:45221505:A/G	49	EIF2AP4	intergenic	1.283	NA	5	15
10:45208025:A:G	10:45208025:A/G	A	G	0.002982	4.50003e-05	0.790739	0.1938	1	10:45221505:A/G	49	EIF2AP4	intergenic	2.9	7	5	15
10:45213993:A:G	10:45213993:A/G	A	G	0.002982	4.00833e-05	0.794164	0.193368	1	10:45221505:A/G	49	EIF2AP4	intergenic	0.965	5	5	15
10:45214296:C:T	10:45214296:A/G	C	T	0.002982	4.01391e-05	0.793969	0.193336	1	10:45221505:A/G	49	EIF2AP4	intergenic	5.393	7	5	15
10:45214374:C:T	10:45214374:A/G	C	T	0.002982	4.01761e-05	0.793881	0.193325	1	10:45221505:A/G	49	EIF2AP4	intergenic	5.356	7	5	15
10:45215950:C:T	10:45215950:A/G	C	T	0.002982	4.56335e-05	0.784007	0.192303	1	10:45221505:A/G	49	EIF2AP4	intergenic	1.942	7	5	15
10:45221505:A:G	10:45221505:A/G	A	G	0.002982	3.41816e-05	0.785225	0.189501	1	10:45221505:A/G	49	EIF2AP4	intergenic	1.422	7	5	15
10:45222548:C:T	10:45222548:A/G	C	T	0.002982	4.73435e-05	0.774406	0.190348	1	10:45221505:A/G	49	EIF2AP4	intergenic	0.807	6	5	15
10:45224270:A:T	10:45224270:A/T	A	T	0.002982	4.78505e-05	0.774218	0.190418	1	10:45221505:A/G	49	EIF2AP4	intergenic	6.523	7	5	15
10:45227648:A:T	10:45227648:A/T	A	T	0.002982	3.83566e-05	0.78322	0.190233	1	10:45221505:A/G	49	EIF2AP4	intergenic	1.267	7	5	15
10:45230828:G:T	10:45230828:A/C	T	G	0.002982	3.93628e-05	0.782664	0.190374	1	10:45221505:A/G	49	EIF2AP4	intergenic	0.794	7	5	15
10:45231382:C:T	10:45231382:A/G	C	T	0.002982	3.9255e-05	0.782886	0.190399	1	10:45221505:A/G	49	EIF2AP4	intergenic	0.103	7	5	15
10:45237043:A:G	10:45237043:A/G	A	G	0.002982	5.17009e-05	0.772649	0.190881	1	10:45221505:A/G	49	EIF2AP4	intergenic	0.615	5	5	15
10:45237483:G:T	10:45237483:A/C	T	G	0.003976	4.05472e-05	0.78254	0.190662	0.7485	10:45221505:A/G	49	EIF2AP4	intergenic	9.06	6	5	15
10:45242523:C:T	10:45242523:A/G	T	C	0.002982	0.00014458	0.738193	0.194251	1	10:45221505:A/G	49	EIF2AP4	intergenic	3.866	6	5	15
10:45243798:A:G	10:45243798:A/G	A	G	0.002982	0.000168543	0.731032	0.194318	1	10:45221505:A/G	49	EIF2AP4	intergenic	0.043	7	5	15
10:45245297:C:T	10:45245297:A/G	C	T	0.002982	0.000171651	0.730618	0.194444	1	10:45221505:A/G	49	EIF2AP4	intergenic	1.839	6	5	15
10:45246273:A:T	10:45246273:A/T	T	A	0.002982	0.000170211	0.731295	0.194516	1	10:45221505:A/G	49	EIF2AP4	intergenic	0.04	6	5	15
10:96042893:A:G	10:96042893:A/G	G	A	0.2107	0.0223166	-0.127141	0.0556433	0.602306	10:96070375:A/G	50	PLCE1:PLCE1-AS1	ncRNA_intronic	4.716	7	4	5
10:96043732:A:G	10:96043732:A/G	G	A	0.338	0.0358396	-0.100183	0.0477348	1	10:96066341:A/G	50	PLCE1:PLCE1-AS1	ncRNA_intronic	8.467	7	4	15
10:96058298:C:T	10:96058298:A/G	T	C	0.3091	0.0684369	-0.0871817	0.0478463	1	10:96058298:A/G	50	PLCE1	exonic	19.2	7	4	15
10:96060610:C:G	10:96060610:C/G	G	C	0.338	0.0415127	-0.0973532	0.0477603	1	10:96066341:A/G	50	PLCE1	intronic	1.443	6	4	15
10:96060711:A:G	10:96060711:A/G	G	A	0.2117	0.0236562	-0.125564	0.055494	0.606036	10:96070375:A/G	50	PLCE1	intronic	2.28	7	4	15
10:96060847:A:T	10:96060847:A/T	A	T	0.338	0.0415664	-0.0973263	0.0477596	1	10:96066341:A/G	50	PLCE1	intronic	2.932	6	4	15
10:96060875:A:G	10:96060875:A/G	G	A	0.338	0.0415726	-0.0973233	0.0477596	1	10:96066341:A/G	50	PLCE1	intronic	2.875	6	4	15
10:96061123:A:T	10:96061123:A/T	A	T	0.2386	0.0253523	-0.116988	0.0523205	0.607797	10:96066341:A/G	50	PLCE1	intronic	0.618	6	4	15
10:96061663:C:CTT	rs5787111	C	CTT	0.2386	NA	NA	NA	0.607797	10:96066341:A/G	50	PLCE1	intronic	3.534	NA	4	15
10:96061793:A:G	10:96061793:A/G	A	G	0.2117	0.0236261	-0.12546	0.055436	0.606036	10:96070375:A/G	50	PLCE1	intronic	2.047	7	4	15
10:96062399:G:T	10:96062399:A/C	G	T	0.2127	0.0219954	0.127105	0.0554935	0.609795	10:96070375:A/G	50	PLCE1	intronic	1.135	6	4	15
10:96063279:A:T	10:96063279:A/T	A	T	0.338	0.0371373	-0.0995184	0.0477478	1	10:96066341:A/G	50	PLCE1	intronic	2.765	6	4	15
10:96065694:C:T	10:96065694:A/G	C	T	0.338	0.0379598	-0.0990467	0.0477268	1	10:96066341:A/G	50	PLCE1	intronic	3.901	7	4	15
10:96066341:A:G	10:96066341:A/G	G	A	0.338	0.0432354	-0.0964304	0.0477041	1	10:96066341:A/G	50	PLCE1	exonic	17.48	3a	4	15
10:96067947:A:G	10:96067947:A/G	G	A	0.338	0.0471093	-0.0949196	0.0478107	1	10:96066341:A/G	50	PLCE1	intronic	0.441	6	4	15
10:96068480:A:G	10:96068480:A/G	G	A	0.335	0.0331149	-0.102296	0.0480109	0.987008	10:96066341:A/G	50	PLCE1	intronic	9.986	7	4	15

10:96069054:G:T	10:96069054:A/C	G	T	0.3082	0.0359713	-0.102824	0.0490282	0.97697	10:96070375:A/G	50	PLCE1	intronic	2.519	5	4	15
10:96069208:C:T	10:96069208:A/G	T	C	0.3082	0.0361125	-0.10273	0.0490205	0.97697	10:96070375:A/G	50	PLCE1	intronic	4.711	7	4	15
10:96069405:G:T	10:96069405:A/C	G	T	0.338	0.0413177	-0.0973832	0.0477292	1	10:96066341:A/G	50	PLCE1	intronic	0.925	7	4	15
10:96069875:A:G	10:96069875:A/G	A	G	0.3042	0.0284766	-0.10752	0.0490812	0.995292	10:96070375:A/G	50	PLCE1	intronic	0.254	7	4	15
10:96070375:A:G	10:96070375:A/G	G	A	0.3032	0.0201937	-0.113987	0.0490748	1	10:96070375:A/G	50	PLCE1	intronic	5.965	NA	2	15
10:96073325:C:T	10:96073325:A/G	T	C	0.333	0.0294503	-0.104147	0.0478304	0.978307	10:96066341:A/G	50	PLCE1	intronic	1.295	6	4	15
10:96073563:C:T	10:96073563:A/G	C	T	0.333	0.0295966	-0.104067	0.0478367	0.978307	10:96066341:A/G	50	PLCE1	intronic	3.996	7	4	15
10:96074157:C:T	10:96074157:A/G	C	T	0.333	0.030368	-0.103643	0.0478663	0.978307	10:96066341:A/G	50	PLCE1	intronic	2.564	6	4	15
10:96074939:G:T	10:96074939:A/C	G	T	0.3012	0.0481671	-0.0953205	0.0482417	0.964399	10:96058298:A/G	50	PLCE1:RP11-76P2.4	ncRNA_exonic	0.346	2b	2	15
10:96079609:C:CTCAAAGG	rs113406892	C	CTCAAAGG	0.3002	NA	NA	NA	0.985837	10:96070375:A/G	50	PLCE1:NOC3L	intronic	10.41	NA	4	5
10:96081137:C:T	10:96081137:A/G	T	C	0.2982	0.0257277	-0.107909	0.0483831	0.942235	10:96058298:A/G	50	PLCE1:NOC3L	intronic	2.184	6	4	5
10:96083452:A:G	10:96083452:A/G	G	A	0.3052	0.0454729	-0.0963255	0.0481567	0.972963	10:96058298:A/G	50	PLCE1:NOC3L	intronic	3.701	6	4	4
10:96083920:A:G	10:96083920:A/G	A	G	0.2753	0.0347367	-0.104793	0.0496317	0.866304	10:96070375:A/G	50	PLCE1:NOC3L	intronic	3.023	6	4	4
10:96084372:A:AT	rs3831084	A	AT	0.2753	NA	NA	NA	0.866304	10:96070375:A/G	50	PLCE1:NOC3L	intronic	6.885	NA	4	4
10:96086078:G:T	10:96086078:A/C	G	T	0.3052	0.0493269	-0.0947281	0.0481889	0.972963	10:96058298:A/G	50	PLCE1:NOC3L	intronic	3.173	6	4	5
10:96087497:C:T	10:96087497:A/G	T	C	0.2744	0.0349729	-0.104654	0.0496305	0.861899	10:96070375:A/G	50	PLCE1:NOC3L	intronic	0.675	5	4	5
10:96089393:A:G	10:96089393:A/G	G	A	0.3052	0.049416	-0.094695	0.0481912	0.972963	10:96058298:A/G	50	PLCE1:NOC3L	UTR3	1.168	7	4	5
10:96089707:A:T	10:96089707:A/T	A	T	0.3052	0.0493956	-0.0947038	0.048191	0.972963	10:96058298:A/G	50	PLCE1:NOC3L	UTR3	2.06	5	4	5
10:96089919:G:GCTTT	rs111898995	G	GCTTT	0.3052	NA	NA	NA	0.972963	10:96058298:A/G	50	PLCE1:NOC3L	UTR3	2.197	NA	4	5
10:96090139:C:T	10:96090139:A/G	T	C	0.3052	0.049436	-0.0946872	0.0481915	0.972963	10:96058298:A/G	50	PLCE1:NOC3L	UTR3	0.572	6	4	5
10:96090672:A:G	10:96090672:A/G	G	A	0.3052	0.0494637	-0.0946769	0.0481921	0.972963	10:96058298:A/G	50	PLCE1:NOC3L	UTR3	1.312	7	4	4
10:96093375:C:T	10:96093375:A/G	T	C	0.2753	0.0351476	-0.104578	0.0496418	0.866304	10:96070375:A/G	50	NOC3L	UTR3	4.397	6	4	4
10:96097114:C:CAAAAAAA	rs551061011	CAAAAAAA	C	0.2753	NA	NA	NA	0.866304	10:96070375:A/G	50	NOC3L	intronic	2.284	NA	4	4
10:96099111:G:GAGTT	rs139024109	G	GAGTT	0.3052	NA	NA	NA	0.972963	10:96058298:A/G	50	NOC3L	intronic	1.398	NA	4	4
10:96100752:A:AG	rs36027832	A	AG	0.3062	NA	NA	NA	0.789806	10:96058298:A/G	50	NOC3L	intronic	0.094	NA	4	5
10:96105979:C:G	10:96105979:C/G	G	C	0.2704	0.0445353	-0.100084	0.0498173	0.83701	10:96070375:A/G	50	NOC3L	intronic	3.494	6	4	5
10:96106025:C:T	10:96106025:A/G	T	C	0.2704	0.0445303	-0.100087	0.0498174	0.83701	10:96070375:A/G	50	NOC3L	intronic	1.676	6	4	5
10:96107867:A:T	10:96107867:A/T	T	A	0.2704	0.0463364	-0.0992789	0.04983	0.83701	10:96070375:A/G	50	NOC3L	intronic	1.507	6	4	5
10:96108102:C:G	10:96108102:C/G	C	G	0.2704	0.0463153	-0.0992887	0.0498307	0.83701	10:96070375:A/G	50	NOC3L	intronic	1.211	7	4	5
10:96108364:G:T	10:96108364:A/C	G	T	0.2704	0.0438386	-0.100416	0.0498186	0.845598	10:96070375:A/G	50	NOC3L	intronic	2.232	6	4	5
10:96108800:C:T	10:96108800:A/G	C	T	0.2704	0.0464871	-0.099204	0.0498274	0.83701	10:96070375:A/G	50	NOC3L	intronic	3.058	7	4	5
10:96109478:G:GGA	10:96109478:G:GGA	G	GGA	0.000994	NA	NA	NA	0.928287	10:96058298:A/G	50	NOC3L	intronic	0.218	NA	4	4
10:96109478:G:GA	10:96109478:G:GA	GA	G	0.3032	NA	NA	NA	0.928287	10:96058298:A/G	50	NOC3L	NA	0.224	NA	4	4
10:96121022:A:G	10:96121022:A/G	G	A	0.2664	0.0426447	-0.101614	0.050126	0.821676	10:96070375:A/G	50	NOC3L	intronic	12.28	4	2	7
10:96122826:C:T	10:96122826:A/G	C	T	0.2664	0.0444684	0.100883	0.0501997	0.821676	10:96070375:A/G	50	NOC3L	upstream	7.965	NA	1	1
10:96126999:T:TTG	rs144179839	TTG	T	0.3419	NA	NA	NA	0.60473	10:96058298:A/G	50	NOC3L	intergenic	0.21	NA	5	15
10:102776360:C:T	10:102776360:A/G	C	T	0.00497	8.18008e-06	-0.718765	0.161143	1	10:102776360:A/G	51	PDZD7	intronic	1.678	5	4	14

10:102776841:C:G	10:102776841:C/G	C	G	0.00497	0.000584906	0.354458	0.103085	1	10:102776360:A/G	51	PDZD7	intronic	6.274	5	4	13
10:102777309:C:T	10:102777309:A/G	C	T	0.00497	1.28925e-05	-0.676895	0.155183	1	10:102776360:A/G	51	PDZD7	UTR3	0.45	5	4	13
10:102778892:A:G	10:102778892:A/G	A	G	0.00497	0.000398169	0.361827	0.102174	1	10:102776360:A/G	51	PDZD7	exonic	12.58	NA	1	7
10:102781830:G:T	10:102781830:A/C	T	G	0.00497	0.000776838	0.335122	0.0997115	1	10:102776360:A/G	51	PDZD7	UTR3	0.05	5	4	14
10:102782599:A:G	10:102782599:A/G	A	G	0.00497	0.0007919	0.334015	0.0995391	1	10:102776360:A/G	51	PDZD7	intronic	0.369	6	4	14
10:102783421:C:T	10:102783421:A/G	C	T	0.00497	0.0010286	0.315162	0.0960104	1	10:102776360:A/G	51	PDZD7	UTR3	5.109	5	4	14
10:102784102:C:G	10:102784102:C/G	C	G	0.00497	0.000807878	0.333316	0.0994949	1	10:102776360:A/G	51	PDZD7	intronic	0.978	7	4	14
10:102785808:C:T	10:102785808:A/G	T	C	0.00497	0.000699733	0.338273	0.0997951	1	10:102776360:A/G	51	PDZD7	intronic	0.344	5	5	14
10:102786536:A:AG	rs142859761	AG	A	0.00497	NA	NA	NA	1	10:102776360:A/G	51	PDZD7	intronic	12.14	NA	5	14
10:112565606:C:T	10:112565606:A/G	T	C	0.4016	0.000115015	0.198874	0.0515691	0.608987	10:112567284:C/G	52	RBM20	intronic	0.544	6	5	15
10:112567284:C:G	10:112567284:C/G	G	C	0.4463	2.065e-05	0.214815	0.0504528	1	10:112567284:C/G	52	RBM20	intronic	4.677	7	4	15
10:112567991:C:G	10:112567991:C/G	C	G	0.4781	0.000913831	0.164392	0.0495786	0.858925	10:112567284:C/G	52	RBM20	intronic	1.336	7	5	15
10:112568281:A:G	10:112568281:A/G	A	G	0.4751	0.00120463	0.159795	0.0493532	0.862428	10:112567284:C/G	52	RBM20	intronic	0.042	7	5	15
10:112570575:A:G	10:112570575:A/G	A	G	0.4742	0.00134424	0.157808	0.0492176	0.802711	10:112567284:C/G	52	RBM20	intronic	4.123	6	4	15
10:112570604:C:CATGT	10:112570604:C:CATGT	C	CATGT	0.02087	NA	NA	NA	0.792091	10:112567284:C/G	52	RBM20	intronic	1.675	NA	4	15
10:112570604:C:CGT	10:112570604:C:CGT	C	CGT	0.4592	NA	NA	NA	0.792091	10:112567284:C/G	52	RBM20	NA	1.384	NA	4	15
10:112571694:C:G	10:112571694:C/G	C	G	0.4533	8.3539e-05	0.195378	0.0496638	0.927367	10:112567284:C/G	52	RBM20	intronic	5.223	5	5	15
10:112575657:A:G	10:112575657:A/G	G	A	0.4473	3.62563e-05	0.201603	0.0488127	0.793252	10:112567284:C/G	52	RBM20	intronic	3.962	2b	5	15
10:112578067:A:C	10:112578067:A/C	A	C	0.4513	0.000139702	0.183597	0.0482048	0.604719	10:112567284:C/G	52	RBM20	intronic	0.772	6	5	15
11:84932885:A:C	11:84932885:A/C	C	A	0.03181	0.0307894	0.374356	0.173331	0.620586	11:85033761:A/G	53	DLG2	intronic	1.926	7	5	15
11:84969976:C:T	11:84969976:A/G	C	T	0.07753	2.90707e-06	-0.420615	0.0899273	1	11:84969976:A/G	53	DLG2	intronic	3.488	6	5	15
11:85004122:A:G	11:85004122:A/G	A	G	0.0507	8.84659e-06	0.467062	0.105109	0.960038	11:85033761:A/G	53	DLG2	intronic	4.579	6	5	15
11:85033761:A:G	11:85033761:A/G	G	A	0.04871	2.11042e-05	0.445606	0.104778	1	11:85033761:A/G	53	DLG2	intronic	2.888	5	9	15
11:85035536:C:G	11:85035536:C/G	G	C	0.04871	2.2092e-05	0.453508	0.106893	1	11:85033761:A/G	53	DLG2	intronic	11.46	6	7	15

11:85091127:C:T	11:85091127:A/G	T	C	0.05368	5.07527e-05	-0.412507	0.1018	0.867738	11:85033761:A/G	53	DLG2	intronic	8.886	7	9	15
11:85137327:C:T	11:85137327:A/G	T	C	0.0497	4.21822e-05	0.438096	0.106978	0.979649	11:85033761:A/G	53	DLG2	intronic	1.538	7	7	15
11:85165754:C:T	11:85165754:A/G	C	T	0.04771	2.43264e-05	0.44685	0.105864	0.97931	11:85033761:A/G	53	DLG2	intronic	0.048	7	5	15
11:85344481:C:T	11:85344481:A/G	T	C	0.04274	0.0016004	0.35803	0.11345	0.755828	11:85033761:A/G	53	TMEM126B	intronic	8.752	7	4	5
11:85439123:C:T	11:85439123:A/G	T	C	0.03579	0.00102386	0.362921	0.110515	0.656646	11:85033761:A/G	53	SYTL2	intronic	2.679	5	4	15
11:85461883:G:GA	rs201107221	G	GA	0.03579	NA	NA	NA	0.61802	11:85033761:A/G	53	SYTL2	intronic	15.81	NA	2	15
11:85491920:C:T	11:85491920:A/G	C	T	0.03877	0.000277409	0.40247	0.110705	0.606715	11:85033761:A/G	53	SYTL2	intronic	3.593	5	5	15
11:85513048:A:G	11:85513048:A/G	G	A	0.03777	0.000158027	0.416637	0.110277	0.623383	11:85033761:A/G	53	SYTL2	intronic	8.474	5	5	15
11:90646762:A:ATATTT	rs202177501	ATATTT	A	0.04374	NA	NA	NA	0.831738	11:90696508:A/C	54	DISC1FP1	ncRNA_intronic	16.72	NA	15	15
11:90696508:A:C	11:90696508:A/C	A	C	0.04274	2.43125e-05	0.432864	0.102548	1	11:90696508:A/C	54	DISC1FP1	intergenic	1.328	6	9	15
11:90784049:A:G	11:90784049:A/G	G	A	0.04374	3.45189e-05	0.424337	0.102462	0.975148	11:90696508:A/C	54	OSBPL9P2	intergenic	0.058	6	15	15
11:99047448:A:G	11:99047448:A/G	A	G	0.002982	2.98744e-06	1.01205	0.216636	1	11:99047448:A/G	55	CNTN5	intronic	2.327	NA	9	15
11:99147050:C:T	rs73536805	C	T	0.001988	NA	NA	NA	0.665336	11:99047448:A/G	55	CNTN5	intronic	1.551	7	5	15
11:99346661:C:T	rs186150910	T	C	0.001988	NA	NA	NA	0.665336	11:99047448:A/G	55	CNTN5	intronic	3.921	6	9	15
12:1179785:C:G	12:1179785:C/G	C	G	0.0328	0.00124272	0.531004	0.164454	0.660491	12:1485129:C/G	56	ERC1	intronic	1.45	7	5	15
12:1232059:C:G	12:1232059:C/G	C	G	0.03777	0.00317036	0.45731	0.154983	0.602789	12:1485129:C/G	56	ERC1	intronic	3.843	4	2	5
12:1274631:A:C	12:1274631:A/C	C	A	0.02883	0.00341838	0.480519	0.164147	0.808728	12:1485129:C/G	56	ERC1	intronic	0.743	7	5	5
12:1282114:C:T	12:1282114:A/G	C	T	0.02883	0.00321197	0.484404	0.164389	0.808728	12:1485129:C/G	56	ERC1	intronic	0.213	7	5	5
12:1353915:C:T	12:1353915:A/G	C	T	0.02783	0.000956408	0.556137	0.168371	0.839783	12:1485129:C/G	56	ERC1	intronic	0.615	3a	4	5
12:1452134:G:T	12:1452134:A/C	G	T	0.03976	0.000121635	0.458351	0.119275	0.711317	12:1485129:C/G	56	ERC1	intronic	12.38	6	5	15
12:1453210:A:T	12:1453210:A/T	T	A	0.02982	1.49876e-05	0.76433	0.176566	0.968907	12:1485129:C/G	56	ERC1	intronic	1.519	7	5	15
12:1485129:C:G	12:1485129:C/G	C	G	0.03082	9.62982e-06	0.777404	0.175672	1	12:1485129:C/G	56	ERC1	intronic	1.279	6	2	5
12:1526897:A:G	12:1526897:A/G	A	G	0.02883	0.00658529	0.526547	0.193789	0.87161	12:1485129:C/G	56	ERC1	intronic	2.027	6	4	5
12:1571319:A:G	12:1571319:A/G	G	A	0.02883	0.0287436	0.410175	0.187553	0.87161	12:1485129:C/G	56	ERC1	intronic	0.32	5	5	5
12:1579408:C:T	12:1579408:A/G	C	T	0.02883	0.0373999	0.404143	0.194171	0.87161	12:1485129:C/G	56	ERC1	intronic	1.63	4	5	5
12:45857819:A:G	12:45857819:A/G	A	G	0.05865	0.0149996	-0.213009	0.0875731	0.629486	12:45932569:A/G	57	RP11-352M15.1	intergenic	11.74	7	14	15
12:45861856:A:G	12:45861856:A/G	A	G	0.06064	0.00854308	-0.223428	0.0849595	0.660115	12:45932569:A/G	57	RP11-352M15.1	intergenic	6.811	5	7	15
12:45871926:A:G	12:45871926:A/G	A	G	0.05567	0.0177501	-0.199332	0.0840784	0.697897	12:45932569:A/G	57	RP11-352M15.1	intergenic	0.102	7	5	15
12:45873319:C:T	12:45873319:A/G	C	T	0.05567	0.0169365	-0.198	0.0829117	0.697897	12:45932569:A/G	57	RP11-352M15.1	intergenic	1.701	7	5	15
12:45895806:A:G	12:45895806:A/G	G	A	0.05467	0.0113311	-0.230536	0.0910381	0.682427	12:45932569:A/G	57	RP11-352M15.1	ncRNA_intronic	7.925	5	5	15
12:45922354:G:GA	rs71437729	G	GA	0.06262	NA	NA	NA	1	12:45932569:A/G	57	RP11-352M15.1	ncRNA_intronic	4.265	NA	5	15
12:45926484:G:GT	rs66561027	GT	G	0.06262	NA	NA	NA	0.837547	12:45932569:A/G	57	RP11-352M15.1	ncRNA_intronic	0.817	NA	5	15
12:45926494:G:T	12:45926494:A/C	G	T	0.05964	2.41944e-05	-0.354029	0.0838496	0.952005	12:45932569:A/G	57	RP11-352M15.1	ncRNA_intronic	1.231	6	5	15
12:45926678:T:TC	rs71301236	T	TC	0.06759	NA	NA	NA	0.923714	12:45932569:A/G	57	RP11-352M15.1	ncRNA_intronic	0.528	NA	5	15
12:45931931:A:C	12:45931931:A/C	C	A	0.06262	2.62157e-05	-0.345136	0.0820957	1	12:45932569:A/G	57	RP11-352M15.1	ncRNA_intronic	4.414	6	5	15
12:45932386:A:G	12:45932386:A/G	A	G	0.03777	0.00134567	-0.309482	0.09653	0.611561	12:45932569:A/G	57	RP11-352M15.1	ncRNA_intronic	0.638	7	5	15
12:45932569:C:T	12:45932569:A/G	C	T	0.06262	2.08015e-05	-0.349522	0.0821225	1	12:45932569:A/G	57	RP11-352M15.1	ncRNA_intronic	1.397	6	5	15
12:45940761:C:T	12:45940761:A/G	C	T	0.0497	0.00671902	0.238485	0.0879871	0.7933	12:45932569:A/G	57	RP11-352M15.1	ncRNA_intronic	2.208	5	5	15
12:45941662:A:G	12:45941662:A/G	A	G	0.05865	0.000182164	-0.293912	0.0785324	0.902944	12:45932569:A/G	57	RP11-352M15.1	ncRNA_intronic	8.448	4	5	15
12:45941969:A:G	12:45941969:A/G	A	G	0.06362	0.000842716	-0.252158	0.0755333	0.832132	12:45932569:A/G	57	RP11-352M15.1	ncRNA_intronic	2.595	4	5	15
12:45946105:A:G	12:45946105:A/G	A	G	0.06262	0.00111565	-0.246978	0.0757689	0.816336	12:45932569:A/G	57	RP11-352M15.1	ncRNA_intronic	6.458	7	5	15
12:45946125:C:T	12:45946125:A/G	C	T	0.05865	0.000627694	-0.265868	0.0777538	0.871325	12:45932569:A/G	57	RP11-352M15.1	ncRNA_intronic	6.338	6	5	15
12:45947611:A:C	12:45947611:A/C	A	C	0.06262	0.000812061	-0.251513	0.0751088	0.816336	12:45932569:A/G	57	RP11-352M15.1	ncRNA_intronic	1.986	7	5	15

12:45948628:C:T	12:45948628:A/G	T	C	0.05567	0.000185624	-0.291856	0.0780817	0.855246	12:45932569:A/G	57	RP11-352M15.1	ncRNA_intronic	1.223	5	5	15
12:119278835:C:T	12:119278835:A/G	T	C	0.16	0.000142117	0.235854	0.0619943	0.669702	12:119285230:A/G	58	RP11-357K6.2	intergenic	3.579	5	9	15
12:119280136:G:T	12:119280136:A/C	G	T	0.1223	9.79858e-05	0.238618	0.0612544	1	12:119285230:A/G	58	RP11-357K6.3	intergenic	4.902	7	5	15
12:119280490:G:T	12:119280490:A/C	G	T	0.1223	6.50296e-05	0.247652	0.0620097	1	12:119285230:A/G	58	RP11-357K6.3	intergenic	7.015	6	5	15
12:119284639:C:T	12:119284639:A/G	C	T	0.1223	5.12129e-05	0.267852	0.0661356	1	12:119285230:A/G	58	RP11-357K6.3	intergenic	3.568	NA	9	15
12:119284872:A:G	12:119284872:A/G	G	A	0.1223	4.48935e-05	0.268935	0.065903	1	12:119285230:A/G	58	RP11-357K6.3	intergenic	2.548	6	9	15
12:119285121:A:G	12:119285121:A/G	G	A	0.3429	1.47374e-05	-0.252418	0.05826	1	12:119285121:A/G	58	RP11-357K6.3	intergenic	3.828	7	9	15
12:119285230:A:G	12:119285230:A/G	G	A	0.1223	4.39189e-05	0.270107	0.0661087	1	12:119285230:A/G	58	RP11-357K6.3	intergenic	2.865	7	9	15
12:119285542:A:G	12:119285542:A/G	A	G	0.1223	8.66658e-05	0.264551	0.0673984	1	12:119285230:A/G	58	RP11-357K6.3	intergenic	3.807	6	9	15
12:119286138:A:C	12:119286138:A/C	A	C	0.3429	2.31326e-05	-0.247069	0.0583769	0.991184	12:119285121:A/G	58	RP11-357K6.3	intergenic	1.717	NA	9	15
12:119287041:A:G	12:119287041:A/G	G	A	0.3042	0.000899643	-0.211768	0.0637817	0.773731	12:119285121:A/G	58	RP11-357K6.3	downstream	3.752	5	9	15
12:119288710:A:T	12:119288710:A/T	T	A	0.1531	1.81082e-06	0.311449	0.065246	1	12:119288710:A/T	58	RP11-357K6.3	ncRNA_intronic	2.337	6	9	15
12:119294183:C:CT	rs36070611	CT	C	0.166	NA	NA	NA	0.883005	12:119288710:A/T	58	RP11-357K6.3	ncRNA_intronic	3.496	NA	9	15
12:119295697:A:G	12:119295697:A/G	A	G	0.1531	2.70046e-06	0.304612	0.0649162	0.983243	12:119288710:A/T	58	RP11-357K6.3	ncRNA_intronic	2.689	7	9	15
12:119298782:T:TCTTC	rs148898795	T	TCTTC	0.1531	NA	NA	NA	0.983243	12:119288710:A/T	58	RP11-357K6.3	ncRNA_intronic	1.535	NA	9	15
12:119299636:A:G	12:119299636:A/G	G	A	0.1531	5.81295e-06	0.292984	0.0646323	0.983243	12:119288710:A/T	58	RP11-357K6.3	ncRNA_intronic	2.773	6	9	15
12:119301029:C:T	12:119301029:A/G	T	C	0.16	8.50651e-06	0.286541	0.0643619	0.928208	12:119288710:A/T	58	RP11-357K6.3	ncRNA_intronic	0.095	7	9	15
12:119301245:C:T	12:119301245:A/G	T	C	0.16	9.91711e-06	0.283542	0.0641648	0.928208	12:119288710:A/T	58	RP11-357K6.3	ncRNA_intronic	2.276	7	9	15
12:119303044:C:G	12:119303044:C/G	G	C	0.1799	5.83771e-05	0.284541	0.0707942	0.746311	12:119288710:A/T	58	RP11-357K6.3	ncRNA_intronic	0.792	7	9	15
12:119303195:C:T	12:119303195:A/G	C	T	0.2038	6.05613e-06	0.304806	0.0673688	0.614522	12:119288710:A/T	58	RP11-357K6.3	ncRNA_intronic	3.69	7	9	15
13:40095218:A:G	13:40095218:A/G	G	A	0.2296	2.09725e-05	-0.250591	0.0589033	0.63231	13:40108439:A/C	59	LHFP	intronic	0.337	6	4	15
13:40096844:C:T	13:40096844:A/G	C	T	0.0994	4.52279e-05	0.344238	0.0843923	0.966877	13:40129220:A/C	59	LHFP	intronic	6.071	6	4	15
13:40108439:G:T	13:40108439:A/C	G	T	0.1581	1.20902e-05	-0.266836	0.0609777	1	13:40108439:A/C	59	LHFP	intronic	4.637	6	4	15
13:40109099:G:T	13:40109099:A/C	T	G	0.1004	5.38565e-05	0.337633	0.083609	0.977864	13:40129220:A/C	59	LHFP	intronic	5.758	5	5	15

13:40109402:A:T	13:40109402:A/T	A	T	0.1014	2.7734e-05	0.349093	0.0832894	0.967246	13:40129220:A/C	59	LHFP	intronic	1.612	7	5	15
13:40109927:C:T	13:40109927:A/G	C	T	0.1004	5.28624e-05	0.337945	0.0835962	0.977864	13:40129220:A/C	59	LHFP	intronic	7.955	6	5	15
13:40111043:C:G	13:40111043:C/G	G	C	0.1004	5.31433e-05	0.337842	0.0835963	0.977864	13:40129220:A/C	59	LHFP	intronic	10.69	6	4	15
13:40112189:A:G	13:40112189:A/G	A	G	0.17	1.39693e-05	-0.258109	0.0594127	0.918735	13:40108439:A/C	59	LHFP	intronic	0.062	7	5	15
13:40112190:G:T	13:40112190:A/C	T	G	0.17	1.39272e-05	-0.258148	0.0594126	0.918735	13:40108439:A/C	59	LHFP	intronic	0.013	7	5	15
13:40112259:C:T	13:40112259:A/G	C	T	0.1004	5.35056e-05	0.337709	0.0835963	0.977864	13:40129220:A/C	59	LHFP	intronic	0.549	6	5	15
13:40115442:C:T	13:40115442:A/G	C	T	0.1004	5.65304e-05	0.336552	0.083577	0.977864	13:40129220:A/C	59	LHFP	intronic	1.711	6	5	15
13:40116233:T:TATAATA	rs5802996	TATAATA	T	0.1412	NA	NA	NA	0.881696	13:40108439:A/C	59	LHFP	intronic	1.538	NA	5	15
13:40117465:C:T	13:40117465:A/G	T	C	0.1004	5.69745e-05	0.336377	0.0835719	0.977864	13:40129220:A/C	59	LHFP	intronic	0.74	7	5	15
13:40117614:A:G	13:40117614:A/G	A	G	0.1004	5.71032e-05	0.336328	0.0835706	0.977864	13:40129220:A/C	59	LHFP	intronic	0.496	6	5	15
13:40117691:C:T	13:40117691:A/G	C	T	0.1004	5.68338e-05	0.336585	0.0836114	0.977864	13:40129220:A/C	59	LHFP	intronic	4.439	7	5	15
13:40117774:C:CCTCTT	rs5802997	CCTCTT	C	0.1421	NA	NA	NA	0.888471	13:40108439:A/C	59	LHFP	intronic	1.052	NA	5	15
13:40118408:C:T	13:40118408:A/G	C	T	0.1004	5.73349e-05	0.33623	0.0835662	0.977864	13:40129220:A/C	59	LHFP	intronic	5.213	5	4	15
13:40118421:C:T	13:40118421:A/G	C	T	0.1004	5.73367e-05	0.33623	0.0835662	0.977864	13:40129220:A/C	59	LHFP	intronic	3.868	5	4	15
13:40118575:A:G	13:40118575:A/G	G	A	0.1004	5.72944e-05	0.336246	0.0835667	0.977864	13:40129220:A/C	59	LHFP	intronic	1.773	7	4	15
13:40121460:C:G	13:40121460:C/G	G	C	0.1282	0.000360532	0.249061	0.0698157	0.752955	13:40129220:A/C	59	LHFP	intronic	1.317	7	5	15
13:40122096:A:G	13:40122096:A/G	G	A	0.1004	4.97824e-05	0.339353	0.0836537	0.977864	13:40129220:A/C	59	LHFP	intronic	5.162	6	5	15
13:40122589:A:T	13:40122589:A/T	T	A	0.1183	0.00026114	0.266768	0.0730654	0.824215	13:40129220:A/C	59	LHFP	intronic	2.1	7	4	15
13:40124883:C:T	13:40124883:A/G	T	C	0.1203	0.000154869	0.264165	0.069827	0.845421	13:40129220:A/C	59	LHFP	intronic	4.984	6	4	15
13:40125010:A:C	13:40125010:A/C	C	A	0.1004	3.6709e-05	0.345952	0.083821	0.977864	13:40129220:A/C	59	LHFP	intronic	3.602	7	4	15
13:40128873:A:C	13:40128873:A/C	A	C	0.1004	2.65132e-05	0.352366	0.0838664	0.977864	13:40129220:A/C	59	LHFP	intronic	0.509	5	5	15
13:40129220:A:C	13:40129220:A/C	A	C	0.1024	8.24609e-06	0.35079	0.0786756	1	13:40129220:A/C	59	LHFP	intronic	0.537	7	5	15
13:40131251:A:C	13:40131251:A/C	A	C	0.1004	1.12069e-05	0.367444	0.0836531	0.977864	13:40129220:A/C	59	LHFP	intronic	5.198	5	4	15
13:40132045:A:C	13:40132045:A/C	A	C	0.1004	6.46632e-05	0.369917	0.0925921	0.977864	13:40129220:A/C	59	LHFP	intronic	1.821	5	4	15
13:40132055:C:G	13:40132055:C/G	C	G	0.1014	5.34154e-05	0.37337	0.0924145	0.966835	13:40129220:A/C	59	LHFP	intronic	2.633	5	4	15
13:40132432:C:T	13:40132432:A/G	T	C	0.1004	9.28378e-05	0.362077	0.0926362	0.977864	13:40129220:A/C	59	LHFP	intronic	1.554	5	4	15
13:40132582:C:T	13:40132582:A/G	C	T	0.1312	6.06166e-05	0.26921	0.0671279	0.767144	13:40129220:A/C	59	LHFP	intronic	6.337	6	4	15
13:40133785:C:T	13:40133785:A/G	T	C	0.1193	0.000221417	0.289416	0.0783639	0.816628	13:40129220:A/C	59	LHFP	intronic	4.717	5	4	15
13:40134616:A:G	13:40134616:A/G	G	A	0.1014	7.09432e-05	0.368618	0.0927786	0.966835	13:40129220:A/C	59	LHFP	intronic	0.575	5	5	15
13:40134835:C:T	13:40134835:A/G	C	T	0.1014	8.12349e-05	0.364557	0.0925103	0.966835	13:40129220:A/C	59	LHFP	intronic	2.516	5	4	15
13:40136040:C:T	13:40136040:A/G	C	T	0.1004	2.45518e-05	0.389455	0.0923122	0.977864	13:40136832:A/G	59	LHFP	intronic	2.176	5	5	15
13:40136832:C:T	13:40136832:A/G	T	C	0.1024	2.26533e-05	0.39325	0.0928132	1	13:40136832:A/G	59	LHFP	intronic	4.679	5	5	15
13:40138554:G:T	13:40138554:A/C	T	G	0.1004	6.111e-05	0.372273	0.0928712	0.977864	13:40136832:A/G	59	LHFP	intronic	4.833	7	2	15
13:40139773:C:T	13:40139773:A/G	C	T	0.1004	2.28761e-05	0.39081	0.092285	0.977864	13:40136832:A/G	59	LHFP	intronic	11.1	4	1	15
13:40140615:C:G	13:40140615:C/G	G	C	0.1183	0.000248653	0.288186	0.0786612	0.820939	13:40136832:A/G	59	LHFP	intronic	0.609	2b	5	15
13:40143281:C:T	13:40143281:A/G	C	T	0.0994	4.7864e-05	0.394598	0.0970526	0.966853	13:40136832:A/G	59	LHFP	intronic	5.954	6	4	15
13:40144707:A:G	13:40144707:A/G	G	A	0.1004	2.36658e-05	0.391933	0.0927176	0.977864	13:40136832:A/G	59	LHFP	intronic	0.666	6	5	15
13:40145137:C:T	13:40145137:A/G	C	T	0.1004	2.35171e-05	0.392182	0.0927455	0.977864	13:40136832:A/G	59	LHFP	intronic	1.011	6	5	15
13:40145690:C:T	13:40145690:A/G	T	C	0.1004	2.33392e-05	0.392489	0.0927805	0.977864	13:40136832:A/G	59	LHFP	intronic	2.794	7	4	15
13:40145808:C:T	13:40145808:A/G	T	C	0.1004	6.63447e-05	0.371962	0.0932459	0.977864	13:40136832:A/G	59	LHFP	intronic	1.885	6	2	15
13:40146427:C:G	13:40146427:C/G	C	G	0.1133	2.73279e-05	0.399733	0.0952953	0.848608	13:40136832:A/G	59	LHFP	intronic	1.429	5	5	15
13:40147090:C:T	13:40147090:A/G	C	T	0.1024	1.18611e-05	0.353382	0.0806784	0.977879	13:40129220:A/C	59	LHFP	intronic	3.744	5	5	15

13:40147349:A:C	13:40147349:A/C	C	A	0.1004	3.00355e-05	0.391834	0.0938928	0.977864	13:40136832:A/G	59	LHFP	intronic	1.483	7	5	15
13:40148708:C:T	13:40148708:A/G	T	C	0.09543	0.000146296	0.370449	0.0975569	0.815787	13:40136832:A/G	59	LHFP	intronic	3.242	6	4	15
13:40149810:A:G	13:40149810:A/G	A	G	0.0994	0.000122096	0.376535	0.0980074	0.754155	13:40136832:A/G	59	LHFP	intronic	1.056	2b	2	15
13:46755852:A:G	13:46755852:A/G	G	A	0.3141	0.00518741	0.156799	0.0560965	0.664161	13:46777563:A/C	60	LCP1	intronic	7.185	5	1	15
13:46757299:A:G	13:46757299:A/G	G	A	0.4215	0.0097639	-0.12636	0.0488989	0.601819	13:46777563:A/C	60	LCP1	intronic	4.888	4	1	15
13:46766821:C:T	13:46766821:A/G	T	C	0.4254	0.0116829	-0.1225	0.0485804	0.627796	13:46777563:A/C	60	LCP1	intronic	3.929	NA	5	15
13:46767862:C:G	13:46767862:C/G	C	G	0.4264	0.00976289	-0.12514	0.0484269	0.626565	13:46777563:A/C	60	LCP1	intronic	0.245	6	5	15
13:46774761:A:G	13:46774761:A/G	A	G	0.3529	0.000509596	-0.169266	0.0487002	0.923027	13:46777563:A/C	60	LCP1	intronic	6.266	4	2	15
13:46775519:C:T	13:46775519:A/G	C	T	0.3529	0.00100456	-0.16081	0.0488898	0.931874	13:46777563:A/C	60	LCP1	intronic	0.118	NA	5	15
13:46777501:C:T	13:46777501:A/G	T	C	0.339	4.21544e-05	-0.203497	0.0496897	1	13:46777563:A/C	60	LCP1	intronic	7.778	7	5	15
13:46777563:G:T	13:46777563:A/C	T	G	0.339	3.93174e-05	-0.204329	0.0496975	1	13:46777563:A/C	60	LCP1	intronic	0.646	NA	5	15
13:46777873:A:G	13:46777873:A/G	A	G	0.3539	0.000790703	-0.163793	0.0488058	0.936363	13:46777563:A/C	60	LCP1	intronic	2.517	NA	5	15
13:46777936:C:T	13:46777936:A/G	C	T	0.3539	0.000754562	-0.164454	0.0488146	0.936363	13:46777563:A/C	60	LCP1	intronic	3.867	NA	5	15
13:46778298:C:G	13:46778298:C/G	C	G	0.336	9.9184e-05	-0.194846	0.0500557	0.986874	13:46777563:A/C	60	LCP1	intronic	4.799	6	5	15
13:46779078:A:T	13:46779078:A/T	T	A	0.3549	0.000704918	-0.166214	0.0490648	0.932549	13:46777563:A/C	60	LCP1	intronic	0.778	5	5	15
13:46779952:C:T	13:46779952:A/G	T	C	0.3559	0.000506422	-0.170429	0.0490111	0.928492	13:46777563:A/C	60	LCP1	intronic	9.255	NA	5	15
13:46781666:A:T	13:46781666:A/T	A	T	0.3111	0.00133686	-0.165151	0.0514815	0.885097	13:46777563:A/C	60	LCP1	intronic	2.764	7	5	15
13:46783599:C:CTGG	rs78541696	C	CTGG	0.3549	NA	NA	NA	0.924013	13:46777563:A/C	60	LCP1	intronic	1.053	NA	5	15
13:46786087:C:T	13:46786087:A/G	T	C	0.3121	0.000811308	-0.17511	0.0522884	0.888811	13:46777563:A/C	60	LRRC63	UTR5	7.964	3a	1	1
13:50282170:C:T	13:50282170:A/G	C	T	0.06064	8.97777e-06	0.583415	0.131387	1	13:50282170:A/G	61	KPNA3	intronic	2.54	7	4	5
13:81292588:A:G	13:81292588:A/G	A	G	0.2753	0.00138905	0.172039	0.0538142	0.66165	13:81561325:A/G	62	PWWP2AP1	intergenic	3.157	7	5	15
13:81301440:C:T	13:81301440:A/G	T	C	0.2883	0.00243302	0.157005	0.0517906	0.601857	13:81441795:A/G	62	PWWP2AP1	intergenic	4.462	6	7	15
13:81307836:C:CTTAAA	rs149129645	CTTAAA	C	0.3579	NA	NA	NA	0.646493	13:81390720:A/G	62	PWWP2AP1	intergenic	1.9	NA	15	15
13:81318410:G:GA	rs71112814	GA	G	0.3579	NA	NA	NA	0.646493	13:81390720:A/G	62	PWWP2AP1	intergenic	1.02	NA	9	15
13:81323013:G:GTT	rs35485242	GTT	G	0.3648	NA	NA	NA	0.617306	13:81390720:A/G	62	PWWP2AP1	intergenic	0.753	NA	5	15
13:81337408:A:G	13:81337408:A/G	G	A	0.3827	0.00744164	0.130339	0.0486995	0.624161	13:81390720:A/G	62	PWWP2AP1	intergenic	4.416	7	5	15
13:81338756:G:GA	rs67266443	GA	G	0.3847	NA	NA	NA	0.615267	13:81390720:A/G	62	PWWP2AP1	intergenic	2.606	NA	5	15
13:81355623:A:G	13:81355623:A/G	A	G	0.2982	0.00207987	-0.169807	0.0551575	0.709735	13:81561325:A/G	62	ARF4P4	intergenic	2.873	5	1	15
13:81357846:A:G	13:81357846:A/G	A	G	0.2982	0.00202854	-0.170212	0.0551557	0.709735	13:81561325:A/G	62	ARF4P4	intergenic	3.977	7	1	15
13:81365872:C:G	13:81365872:C/G	C	G	0.4622	2.71401e-05	-0.197234	0.0470027	0.992341	13:81390720:A/G	62	ARF4P4	intergenic	1.206	7	9	15
13:81368422:C:G	13:81368422:C/G	C	G	0.4652	8.01668e-05	-0.185087	0.0469299	0.980943	13:81390720:A/G	62	ARF4P4	intergenic	6.759	6	7	15
13:81370189:C:T	13:81370189:A/G	T	C	0.4622	2.76699e-05	-0.19697	0.0469889	0.992341	13:81390720:A/G	62	ARF4P4	intergenic	12.73	7	9	15
13:81370617:G:T	13:81370617:A/C	G	T	0.4622	2.76938e-05	-0.196956	0.0469878	0.992341	13:81390720:A/G	62	ARF4P4	intergenic	1.508	7	7	15
13:81376473:A:C	13:81376473:A/C	C	A	0.3042	0.00158294	-0.173699	0.0549848	0.729141	13:81561325:A/G	62	ARF4P4	intergenic	15.12	7	9	15
13:81376583:A:G	13:81376583:A/G	G	A	0.4622	2.74933e-05	-0.19704	0.0469893	0.992341	13:81390720:A/G	62	ARF4P4	intergenic	4.782	7	9	15
13:81377156:C:T	13:81377156:A/G	T	C	0.4622	2.79441e-05	-0.196835	0.0469816	0.992341	13:81390720:A/G	62	ARF4P4	intergenic	3.552	5	7	15
13:81378886:A:C	13:81378886:A/C	A	C	0.2992	0.0015459	-0.17461	0.0551528	0.71291	13:81561325:A/G	62	ARF4P4	intergenic	3.205	7	9	15
13:81381145:A:G	13:81381145:A/G	G	A	0.4036	4.03983e-05	-0.197204	0.0480377	0.786854	13:81441795:A/G	62	ARF4P4	intergenic	5.386	6	9	15
13:81384853:A:G	13:81384853:A/G	A	G	0.4642	6.33996e-05	-0.188343	0.0470883	0.984732	13:81390720:A/G	62	ARF4P4	intergenic	6.636	6	9	15
13:81385474:C:T	13:81385474:A/G	T	C	0.4612	2.52578e-05	-0.198575	0.0471398	0.996178	13:81390720:A/G	62	ARF4P4	intergenic	2.716	7	7	15
13:81386520:A:G	13:81386520:A/G	G	A	0.4006	9.96665e-05	-0.185098	0.047566	0.781123	13:81441795:A/G	62	ARF4P4	intergenic	1.296	5	5	15
13:81389340:A:G	13:81389340:A/G	G	A	0.4642	7.5767e-05	-0.185397	0.0468482	0.984732	13:81390720:A/G	62	ARF4P4	intergenic	3.387	6	5	15
13:81390720:C:T	13:81390720:A/G	T	C	0.4622	2.23106e-05	-0.198633	0.0468428	1	13:81390720:A/G	62	ARF4P4	intergenic	1.794	7	8	15

13:81394324:A:G	13:81394324:A/G	A	G	0.2992	0.00122978	-0.177245	0.0548424	0.71291	13:81561325:A/G	62	ARF4P4	intergenic	8.455	7	5	15
13:81402108:A:G	13:81402108:A/G	G	A	0.4652	6.55542e-05	-0.186546	0.0467316	0.988601	13:81390720:A/G	62	ARF4P4	intergenic	3.166	6	5	15
13:81406067:C:G	13:81406067:C/G	C	G	0.3002	0.00117781	-0.177673	0.0547661	0.716109	13:81561325:A/G	62	ARF4P4	intergenic	2.768	3a	5	15
13:81411946:C:T	13:81411946:A/G	C	T	0.3658	3.32983e-05	-0.199033	0.0479637	0.855853	13:81441795:A/G	62	ARF4P4	intergenic	3.826	5	5	15
13:81412836:C:T	13:81412836:A/G	T	C	0.3002	0.00124495	-0.17707	0.0548478	0.716109	13:81561325:A/G	62	ARF4P4	intergenic	3.607	3a	5	15
13:81419379:A:G	13:81419379:A/G	A	G	0.3002	0.00129052	-0.176441	0.0548282	0.716109	13:81561325:A/G	62	ARF4P4	intergenic	1.738	5	5	15
13:81421742:A:G	13:81421742:A/G	A	G	0.3012	0.00128834	0.176491	0.0548354	0.719331	13:81561325:A/G	62	ARF4P4	intergenic	0.701	7	5	15
13:81424200:C:G	13:81424200:C/G	C	G	0.4563	8.13758e-05	0.183878	0.0466657	0.954932	13:81390720:A/G	62	ARF4P4	intergenic	0.845	7	5	15
13:81429073:C:CTG	rs34973788	CTG	C	0.4563	NA	NA	NA	0.954932	13:81390720:A/G	62	ARF4P4	intergenic	7.761	NA	5	15
13:81429951:A:C	13:81429951:A/C	C	A	0.4563	5.81784e-05	0.187888	0.0467374	0.954932	13:81390720:A/G	62	ARF4P4	intergenic	4.02	7	5	15
13:81430774:A:G	13:81430774:A/G	G	A	0.3002	0.000970946	0.18165	0.0550651	0.716109	13:81561325:A/G	62	ARF4P4	intergenic	1.707	7	5	15
13:81433543:C:T	13:81433543:A/G	C	T	0.3012	0.000952445	0.181979	0.055075	0.719331	13:81561325:A/G	62	ARF4P4	intergenic	1.854	7	5	15
13:81436124:C:T	13:81436124:A/G	T	C	0.3062	0.000585999	0.189477	0.0551124	0.735803	13:81561325:A/G	62	ARF4P4	intergenic	4.871	5	5	15
13:81437573:C:CA	rs60270962	CA	C	0.493	NA	NA	NA	0.729787	13:81390720:A/G	62	ARF4P4	intergenic	1.606	NA	5	15
13:81437698:C:CT	rs11306747	C	CT	0.4652	NA	NA	NA	0.805902	13:81390720:A/G	62	ARF4P4	intergenic	1.248	NA	5	15
13:81440532:A:C	13:81440532:A/C	A	C	0.4573	5.5142e-05	0.188736	0.0468015	0.958521	13:81390720:A/G	62	ARF4P4	intergenic	19.17	6	5	15
13:81441795:A:G	13:81441795:A/G	A	G	0.4056	2.33956e-05	0.202736	0.0479312	1	13:81441795:A/G	62	ARF4P4	intergenic	0.727	7	5	15
13:81442689:A:AAT	rs10550215	A	AAT	0.3141	NA	NA	NA	0.750589	13:81561325:A/G	62	ARF4P4	intergenic	0.43	NA	5	15
13:81442734:A:G	13:81442734:A/G	A	G	0.4771	6.3056e-06	0.223191	0.0494235	1	13:81442734:A/G	62	ARF4P4	intergenic	0.64	6	5	15
13:81442736:A:G	13:81442736:A/G	A	G	0.4414	2.30673e-05	0.200061	0.0472631	0.839063	13:81390720:A/G	62	ARF4P4	intergenic	1.029	6	5	15
13:81444075:C:T	13:81444075:A/G	T	C	0.4791	9.29023e-05	0.183175	0.0468669	0.894722	13:81390720:A/G	62	ARF4P4	intergenic	4.677	7	5	15
13:81445499:A:G	13:81445499:A/G	G	A	0.329	0.00132572	0.175322	0.0546114	0.733326	13:81441795:A/G	62	ARF4P4	intergenic	2.538	6	5	15
13:81446298:A:G	13:81446298:A/G	G	A	0.4781	7.26087e-05	0.186248	0.0469427	0.890652	13:81390720:A/G	62	ARF4P4	intergenic	0.666	5	5	15
13:81453591:A:G	13:81453591:A/G	A	G	0.325	0.00233897	0.169515	0.0556984	0.72399	13:81441795:A/G	62	ARF4P4	intergenic	1.912	7	5	15
13:81453956:G:GGAACA	rs199837349	G	GGAACA	0.4801	NA	NA	NA	0.876359	13:81390720:A/G	62	ARF4P4	intergenic	0.677	NA	5	15
13:81464425:C:CT	rs57005716	CT	C	0.4354	NA	NA	NA	0.777187	13:81390720:A/G	62	ARF4P4	intergenic	0.485	NA	5	15
13:81464524:G:GT	rs34591462	G	GT	0.3062	NA	NA	NA	0.710306	13:81561325:A/G	62	ARF4P4	intergenic	1.442	NA	5	15
13:81473270:G:T	13:81473270:A/C	T	G	0.3598	0.00012583	0.190929	0.0497926	0.823006	13:81441795:A/G	62	ARF4P4	intergenic	1.59	7	5	15
13:81491062:A:C	13:81491062:A/C	A	C	0.3231	0.000204096	0.197482	0.0531739	0.850435	13:81561325:A/G	62	ARF4P4	intergenic	0.546	6	5	15
13:81491065:C:T	13:81491065:A/G	C	T	0.3231	0.000204137	0.19748	0.053174	0.850435	13:81561325:A/G	62	ARF4P4	intergenic	1.281	6	5	15
13:81492790:A:G	13:81492790:A/G	G	A	0.336	6.8516e-05	0.200304	0.0503101	0.92068	13:81561325:A/G	62	ARF4P4	intergenic	5.756	6	5	15
13:81493047:A:G	13:81493047:A/G	A	G	0.336	6.62643e-05	0.200571	0.0502771	0.92068	13:81561325:A/G	62	ARF4P4	intergenic	2.439	6	5	15
13:81493080:G:T	13:81493080:A/C	T	G	0.335	6.66856e-05	0.200499	0.0502782	0.916027	13:81561325:A/G	62	ARF4P4	intergenic	1.234	6	5	15
13:81493325:A:G	13:81493325:A/G	G	A	0.3072	0.00028716	0.194106	0.0535228	0.80998	13:81561325:A/G	62	ARF4P4	intergenic	1.53	6	5	15
13:81493664:A:T	13:81493664:A/T	T	A	0.3072	0.000282295	0.194282	0.0535059	0.80998	13:81561325:A/G	62	ARF4P4	intergenic	2.194	7	5	15
13:81493761:A:C	13:81493761:A/C	C	A	0.336	6.32057e-05	0.20091	0.0502212	0.92068	13:81561325:A/G	62	ARF4P4	intergenic	4.332	6	5	15
13:81493801:C:T	13:81493801:A/G	T	C	0.335	6.60923e-05	0.200591	0.0502742	0.916409	13:81561325:A/G	62	ARF4P4	intergenic	1.963	6	5	15
13:81493805:G:T	13:81493805:A/C	G	T	0.335	6.60887e-05	0.200592	0.0502742	0.916409	13:81561325:A/G	62	ARF4P4	intergenic	1.337	6	5	15
13:81493965:A:C	13:81493965:A/C	A	C	0.336	5.10156e-05	0.203457	0.0502247	0.92068	13:81561325:A/G	62	ARF4P4	intergenic	0.049	6	5	15
13:81494098:A:G	13:81494098:A/G	G	A	0.336	5.72201e-05	0.202166	0.0502401	0.92068	13:81561325:A/G	62	ARF4P4	intergenic	0.016	7	5	15
13:81494311:C:T	13:81494311:A/G	C	T	0.3072	0.000281519	0.194308	0.0535029	0.80998	13:81561325:A/G	62	ARF4P4	intergenic	2.652	7	5	15
13:81495247:A:T	13:81495247:A/T	T	A	0.3052	0.000389876	0.189967	0.0535594	0.802091	13:81561325:A/G	62	ARF4P4	intergenic	0.725	6	5	15
13:81495532:A:C	13:81495532:A/C	A	C	0.336	6.7878e-05	0.200045	0.0502171	0.92068	13:81561325:A/G	62	ARF4P4	intergenic	7.339	6	5	15

13:81496061:A:G	13:81496061:A/G	G	A	0.336	7.46253e-05	0.198806	0.0501905	0.92068	13:81561325:A/G	62	ARF4P4	intergenic	1.361	3a	5	15
13:81496913:A:C	13:81496913:A/C	A	C	0.3082	0.000303335	0.193152	0.0534686	0.813612	13:81561325:A/G	62	ARF4P4	intergenic	1.065	7	5	15
13:81497018:C:T	13:81497018:A/G	C	T	0.337	7.46455e-05	0.1988	0.0501897	0.924985	13:81561325:A/G	62	ARF4P4	intergenic	2.23	7	5	15
13:81498940:A:G	13:81498940:A/G	A	G	0.338	6.53498e-05	0.200368	0.0501846	0.929325	13:81561325:A/G	62	ARF4P4	intergenic	2.865	2b	2	15
13:81499507:A:T	13:81499507:A/T	T	A	0.338	6.47645e-05	0.200453	0.0501791	0.929325	13:81561325:A/G	62	ARF4P4	intergenic	2.945	6	5	15
13:81499895:A:G	13:81499895:A/G	G	A	0.338	6.47586e-05	0.200475	0.0501845	0.929325	13:81561325:A/G	62	ARF4P4	intergenic	0.455	5	5	15
13:81501670:A:G	13:81501670:A/G	A	G	0.338	6.31301e-05	0.200727	0.0501717	0.929325	13:81561325:A/G	62	ARF4P4	intergenic	2.901	6	5	15
13:81501842:C:T	13:81501842:A/G	C	T	0.338	6.2997e-05	0.20074	0.0501689	0.929325	13:81561325:A/G	62	ARF4P4	intergenic	3.744	6	5	15
13:81502144:A:G	13:81502144:A/G	A	G	0.338	6.27563e-05	0.200765	0.0501637	0.929325	13:81561325:A/G	62	ARF4P4	intergenic	1.978	6	5	15
13:81504410:A:C	13:81504410:A/C	C	A	0.338	6.18546e-05	0.200826	0.0501361	0.929325	13:81561325:A/G	62	ARF4P4	intergenic	3.35	6	5	15
13:81504507:A:C	13:81504507:A/C	A	C	0.338	6.9577e-05	0.199349	0.0501163	0.929325	13:81561325:A/G	62	ARF4P4	intergenic	0.121	7	5	15
13:81506503:C:T	13:81506503:A/G	T	C	0.338	6.09897e-05	0.201401	0.0502378	0.929325	13:81561325:A/G	62	ARF4P4	intergenic	0.593	7	5	15
13:81508230:A:G	13:81508230:A/G	G	A	0.3479	5.00267e-05	0.202999	0.0500551	0.880648	13:81561325:A/G	62	ARF4P4	intergenic	0.518	7	5	15
13:81508293:C:G	13:81508293:C/G	G	C	0.3082	0.000279508	0.194489	0.0535253	0.813612	13:81561325:A/G	62	ARF4P4	intergenic	0.694	7	5	15
13:81509694:A:G	13:81509694:A/G	G	A	0.3082	0.000279408	0.194514	0.0535309	0.813612	13:81561325:A/G	62	ARF4P4	intergenic	0.41	7	14	15
13:81509823:A:G	13:81509823:A/G	G	A	0.338	6.16783e-05	0.201465	0.0502872	0.929325	13:81561325:A/G	62	ARF4P4	intergenic	3.958	7	14	15
13:81510960:A:T	13:81510960:A/T	T	A	0.3091	0.000284273	0.194715	0.053652	0.808965	13:81561325:A/G	62	ARF4P4	intergenic	9.885	6	14	15
13:81510961:A:G	13:81510961:A/G	G	A	0.3091	0.000284285	0.194715	0.0536521	0.808965	13:81561325:A/G	62	ARF4P4	intergenic	10.31	6	14	15
13:81512530:C:CT	rs71112830	CT	C	0.3101	NA	NA	NA	0.81263	13:81561325:A/G	62	ARF4P4	intergenic	4.068	NA	9	15
13:81517643:A:G	13:81517643:A/G	A	G	0.3091	0.000270469	0.195107	0.0535706	0.808965	13:81561325:A/G	62	ARF4P4	intergenic	1.904	7	9	15
13:81520664:C:T	13:81520664:A/G	T	C	0.3479	5.60049e-05	0.201884	0.0501072	0.880648	13:81561325:A/G	62	ARF4P4	intergenic	0.171	5	5	15
13:81521084:G:T	13:81521084:A/C	T	G	0.3181	0.000241549	0.195387	0.0532236	0.7637	13:81561325:A/G	62	ARF4P4	intergenic	1.827	7	5	15
13:81525383:C:T	13:81525383:A/G	C	T	0.4165	0.000319714	0.172741	0.0479998	0.621745	13:81390720:A/G	62	ARF4P4	intergenic	3.82	7	5	15
13:81525967:A:T	13:81525967:A/T	A	T	0.3559	6.92788e-05	0.202982	0.0510165	0.661955	13:81561325:A/G	62	ARF4P4	intergenic	0.031	6	5	15
13:81529384:C:G	13:81529384:C/G	G	C	0.3111	0.000299744	0.191524	0.0529728	0.834272	13:81561325:A/G	62	ARF4P4	intergenic	0.234	7	7	15
13:81529694:A:G	13:81529694:A/G	G	A	0.4175	0.000317093	0.172329	0.0478569	0.62382	13:81390720:A/G	62	ARF4P4	intergenic	3.794	3a	5	15
13:81529823:A:G	13:81529823:A/G	A	G	0.3111	0.000302087	0.191401	0.0529683	0.834272	13:81561325:A/G	62	ARF4P4	intergenic	3.312	2b	5	15
13:81530176:A:C	13:81530176:A/C	C	A	0.3111	0.000307827	0.191146	0.0529693	0.834272	13:81561325:A/G	62	ARF4P4	intergenic	2.768	7	5	15
13:81530818:A:G	13:81530818:A/G	G	A	0.4175	0.000307281	0.172999	0.0479342	0.62382	13:81390720:A/G	62	ARF4P4	intergenic	4.148	6	7	15
13:81531621:G:T	13:81531621:A/C	T	G	0.3111	0.000334188	0.189988	0.052962	0.834272	13:81561325:A/G	62	ARF4P4	intergenic	19.82	4	5	15
13:81531839:A:G	13:81531839:A/G	A	G	0.3628	5.86107e-05	0.201508	0.0501472	0.644552	13:81561325:A/G	62	ARF4P4	intergenic	4.559	5	5	15
13:81533945:C:T	13:81533945:A/G	T	C	0.4473	8.16997e-05	0.185587	0.047111	0.701529	13:81390720:A/G	62	ARF4P4	intergenic	1.716	7	5	15
13:81534544:A:C	13:81534544:A/C	A	C	0.3668	0.00180835	0.154747	0.0495979	0.617127	13:81561325:A/G	62	ARF4P4	intergenic	0.527	7	5	15
13:81535119:A:G	13:81535119:A/G	A	G	0.3111	0.000220115	0.197539	0.0534652	0.834272	13:81561325:A/G	62	ARF4P4	intergenic	1.653	7	5	15
13:81545761:A:G	13:81545761:A/G	A	G	0.4175	0.000705894	0.163579	0.0482924	0.616483	13:81390720:A/G	62	RP11-521J24.1	intergenic	0.012	6	9	15
13:81547556:G:GTT	rs34566903	GTT	G	0.3857	NA	NA	NA	0.606318	13:81561325:A/G	62	RP11-521J24.1	intergenic	1.146	NA	7	15
13:81549575:A:G	13:81549575:A/G	A	G	0.3091	0.000613652	0.180802	0.0527811	0.851987	13:81561325:A/G	62	RP11-521J24.1	intergenic	0.885	6	5	15
13:81550449:C:T	13:81550449:A/G	C	T	0.3062	0.000168764	0.199503	0.0530351	0.864799	13:81561325:A/G	62	RP11-521J24.1	intergenic	0.566	6	5	15
13:81552417:A:T	13:81552417:A/T	T	A	0.3091	0.000307474	0.191478	0.0530569	0.859923	13:81561325:A/G	62	RP11-521J24.1	intergenic	0.445	6	5	15
13:81554857:C:CAA	rs140346350	C	CAA	0.3211	NA	NA	NA	0.798833	13:81561325:A/G	62	RP11-521J24.1	intergenic	10.32	NA	5	15
13:81555225:A:C	13:81555225:A/C	A	C	0.341	0.000307964	0.183235	0.0507785	0.753691	13:81561325:A/G	62	RP11-521J24.1	intergenic	6.899	7	5	15
13:81558684:C:T	13:81558684:A/G	T	C	0.3101	0.000123612	0.203064	0.0528971	0.855202	13:81561325:A/G	62	RP11-521J24.1	intergenic	0.496	7	5	15
13:81561325:A:G	13:81561325:A/G	A	G	0.334	3.90064e-05	0.206319	0.0501592	1	13:81561325:A/G	62	RP11-521J24.1	intergenic	0.596	7	5	15

13:81565903:C:T	13:81565903:A/G	C	T	0.334	4.22307e-05	0.205542	0.0501944	1	13:81561325:A/G	62	RP11-521J24.1	intergenic	3.462	5	7	15
13:81566241:A:C	13:81566241:A/C	C	A	0.3062	0.000113295	0.204991	0.0531038	0.873805	13:81561325:A/G	62	RP11-521J24.1	intergenic	1.737	6	5	15
13:81566407:C:T	13:81566407:A/G	T	C	0.3091	0.000135703	0.202149	0.0529758	0.851441	13:81561325:A/G	62	RP11-521J24.1	intergenic	0.031	6	5	15
13:81570600:C:T	13:81570600:A/G	T	C	0.3091	0.00013613	0.202098	0.0529732	0.851441	13:81561325:A/G	62	RP11-521J24.1	intergenic	1.171	7	5	15
13:81573546:A:G	13:81573546:A/G	G	A	0.3062	0.000112077	0.205085	0.0530919	0.873805	13:81561325:A/G	62	RP11-521J24.1	intergenic	0.074	5	5	15
13:81577275:G:T	13:81577275:A/C	G	T	0.3022	0.000163519	0.201406	0.0534292	0.858023	13:81561325:A/G	62	RP11-521J24.1	intergenic	1.106	6	9	15
13:81578239:C:G	13:81578239:C/G	C	G	0.3101	8.13891e-05	0.210695	0.0534726	0.8216	13:81561325:A/G	62	RP11-521J24.1	intergenic	0.556	7	5	15
13:81585318:A:G	13:81585318:A/G	A	G	0.337	9.06925e-05	0.203627	0.0520221	0.677266	13:81561325:A/G	62	RP11-521J24.1	intergenic	0.605	7	5	15
13:81585973:A:C	13:81585973:A/C	C	A	0.333	8.26981e-05	0.205053	0.0520908	0.691632	13:81561325:A/G	62	RP11-521J24.1	intergenic	3.759	5	4	15
13:81586155:C:T	13:81586155:A/G	C	T	0.333	8.27219e-05	0.205033	0.0520867	0.691632	13:81561325:A/G	62	RP11-521J24.1	intergenic	5.626	4	4	15
13:81586560:C:T	13:81586560:A/G	C	T	0.333	8.31368e-05	0.204933	0.0520774	0.691632	13:81561325:A/G	62	RP11-521J24.1	intergenic	2.087	5	4	15
13:81595758:A:G	13:81595758:A/G	G	A	0.3429	0.000229021	0.190263	0.0516367	0.657141	13:81561325:A/G	62	RP11-521J24.1	intergenic	2.317	NA	5	15
13:81600547:C:T	13:81600547:A/G	T	C	0.3439	0.000401941	0.181806	0.0513747	0.63754	13:81561325:A/G	62	RP11-521J24.1	intergenic	0.708	7	5	15
13:81605917:C:G	13:81605917:C/G	G	C	0.4165	0.012268	0.121074	0.0483463	0.638936	13:81561325:A/G	62	RP11-521J24.1	intergenic	6.451	7	5	15
13:81606745:C:T	13:81606745:A/G	C	T	0.3429	0.00155698	0.163444	0.0516602	0.641267	13:81561325:A/G	62	RP11-521J24.1	intergenic	0.863	NA	5	15
13:81608496:A:G	13:81608496:A/G	G	A	0.4175	0.00168278	0.152304	0.0484871	0.629337	13:81561325:A/G	62	RP11-521J24.1	intergenic	0.192	7	14	15
13:81610387:G:T	13:81610387:A/C	G	T	0.4195	0.0142817	0.118549	0.048386	0.632174	13:81561325:A/G	62	RP11-521J24.1	intergenic	3.377	5	9	15
13:81611325:A:G	13:81611325:A/G	G	A	0.3449	0.0020388	0.159924	0.0518468	0.633829	13:81561325:A/G	62	RP11-521J24.1	intergenic	1.302	6	9	15
13:81614581:A:T	13:81614581:A/T	A	T	0.3757	0.000617141	0.169158	0.0494039	0.753773	13:81561325:A/G	62	RP11-521J24.1	intergenic	0.037	6	9	15
13:81615536:A:G	13:81615536:A/G	A	G	0.3469	0.00165783	0.163154	0.0518684	0.650902	13:81561325:A/G	62	RP11-521J24.1	intergenic	1.618	6	5	15
13:81615671:C:T	13:81615671:A/G	T	C	0.3509	0.00186949	0.161073	0.0517886	0.63775	13:81561325:A/G	62	RP11-521J24.1	intergenic	0.159	6	5	15
13:81617834:A:AT	rs36001066	A	AT	0.4235	NA	NA	NA	0.622779	13:81561325:A/G	62	RP11-521J24.1	ncRNA_exonic	0.178	NA	5	15
13:82042699:A:C	13:82042699:A/C	A	C	0.02087	0.00129892	-0.552315	0.171727	0.617824	13:82064770:A/C	63	RP11-452B18.2	intergenic	0.384	7	9	15
13:82042986:G:T	13:82042986:A/C	T	G	0.02087	0.00134919	-0.550474	0.171738	0.617824	13:82064770:A/C	63	RP11-452B18.2	intergenic	1.224	6	9	15
13:82043785:A:G	13:82043785:A/G	A	G	0.02187	0.00180311	-0.526543	0.168716	0.673582	13:82064770:A/C	63	RP11-452B18.2	intergenic	1.318	7	9	15
13:82044733:A:G	13:82044733:A/G	A	G	0.02087	0.00214482	-0.514479	0.167614	0.707317	13:82064770:A/C	63	RP11-452B18.2	intergenic	1.535	6	15	15
13:82046664:C:T	13:82046664:A/G	T	C	0.0169	0.000772499	-0.606251	0.1803	0.881944	13:82064770:A/C	63	RP11-452B18.2	intergenic	0.441	7	15	15
13:82047655:A:G	13:82047655:A/G	G	A	0.0169	0.000763744	-0.606121	0.180095	0.881944	13:82064770:A/C	63	RP11-452B18.2	intergenic	2.539	5	7	15
13:82049607:A:G	13:82049607:A/G	G	A	0.0169	0.000747753	-0.605772	0.179677	0.881944	13:82064770:A/C	63	RP11-452B18.2	intergenic	1.091	6	15	15
13:82050815:C:T	13:82050815:A/G	C	T	0.0169	0.000737849	-0.605436	0.179382	0.881944	13:82064770:A/C	63	RP11-452B18.2	intergenic	1.779	7	15	15
13:82051241:A:G	13:82051241:A/G	G	A	0.0169	0.000736369	-0.605215	0.179287	0.881944	13:82064770:A/C	63	RP11-452B18.2	intergenic	0.026	6	9	15
13:82051306:C:T	13:82051306:A/G	C	T	0.0169	0.000736584	-0.605149	0.179272	0.881944	13:82064770:A/C	63	RP11-452B18.2	intergenic	1.028	7	9	15
13:82051352:C:G	13:82051352:C/G	C	G	0.0169	0.000734827	-0.605216	0.179257	0.881944	13:82064770:A/C	63	RP11-452B18.2	intergenic	0.152	7	9	15
13:82051392:C:T	13:82051392:A/G	C	T	0.0169	0.000735605	-0.605143	0.179251	0.881944	13:82064770:A/C	63	RP11-452B18.2	intergenic	0.083	7	9	15
13:82052105:A:G	13:82052105:A/G	A	G	0.0169	0.000731782	-0.604906	0.179107	0.881944	13:82064770:A/C	63	RP11-452B18.2	intergenic	1.673	6	15	15
13:82056908:A:C	13:82056908:A/C	C	A	0.0169	0.000694785	-0.604966	0.17837	0.881944	13:82064770:A/C	63	RP11-452B18.2	intergenic	0.244	7	9	15
13:82058535:C:G	13:82058535:C/G	C	G	0.0169	0.00069097	-0.605076	0.178325	0.881944	13:82064770:A/C	63	RP11-452B18.2	intergenic	0.555	7	15	15
13:82059510:A:G	13:82059510:A/G	G	A	0.0169	0.00068932	-0.605123	0.178303	0.881944	13:82064770:A/C	63	RP11-452B18.2	intergenic	0.936	6	14	15
13:82064365:C:T	13:82064365:A/G	T	C	0.0169	4.04956e-05	0.561773	0.136863	1	13:82064770:A/C	63	RP11-452B18.2	intergenic	1.067	7	5	15
13:82064583:C:T	13:82064583:A/G	C	T	0.0169	3.78427e-05	0.564045	0.136895	1	13:82064770:A/C	63	RP11-452B18.2	intergenic	0.776	7	5	15
13:82064586:A:G	13:82064586:A/G	G	A	0.0169	3.78417e-05	0.564046	0.136895	1	13:82064770:A/C	63	RP11-452B18.2	intergenic	2.903	7	5	15
13:82064661:A:G	13:82064661:A/G	A	G	0.0169	0.000222879	-0.592514	0.160505	1	13:82064770:A/C	63	RP11-452B18.2	intergenic	2.981	6	5	15
13:82064770:G:T	13:82064770:A/C	G	T	0.0169	3.73569e-05	0.564605	0.136932	1	13:82064770:A/C	63	RP11-452B18.2	intergenic	0.14	6	5	15

13:82065009:A:T	13:82065009:A/T	T	A	0.0169	0.000526478	-0.613557	0.176975	1	13:82064770:A/C	63	RP11-452B18.2	intergenic	2.591	NA	5	15
13:82065010:G:T	13:82065010:A/C	G	T	0.0169	0.000526483	-0.613557	0.176975	1	13:82064770:A/C	63	RP11-452B18.2	intergenic	0.671	NA	5	15
13:82065172:C:T	13:82065172:A/G	T	C	0.0169	4.47293e-05	0.55896	0.136947	1	13:82064770:A/C	63	RP11-452B18.2	intergenic	2.531	7	5	15
13:82085654:A:G	13:82085654:A/G	A	G	0.01292	0.00197853	-0.714113	0.230847	0.758463	13:82064770:A/C	63	PTMAP5	intergenic	0.962	7	15	15
14:31325883:C:T	14:31325883:A/G	C	T	0.4563	2.5891e-05	-0.197003	0.0468287	0.896105	14:31343135:A/G	64	COCH	intergenic	2.762	NA	5	15
14:31325917:T:TTTTG	14:31325917:T:T TTTG	T	TTTTG	0.4543	NA	NA	NA	0.89652	14:31343135:A/G	64	COCH	intergenic	5.245	NA	5	15
14:31326658:C:T	14:31326658:A/G	C	T	0.4553	2.35783e-05	-0.197554	0.0467251	0.900306	14:31343135:A/G	64	COCH	intergenic	8.012	7	5	15
14:31328308:A:C	14:31328308:A/C	A	C	0.4553	2.25004e-05	-0.198684	0.0468757	0.900306	14:31343135:A/G	64	COCH	intergenic	0.216	NA	5	15
14:31329154:A:G	14:31329154:A/G	G	A	0.4533	3.85672e-05	-0.193991	0.0471318	0.901142	14:31343135:A/G	64	COCH	intergenic	5.528	5	5	15
14:31332032:C:CA	rs534269021	C	CA	0.3926	NA	NA	NA	0.668938	14:31343135:A/G	64	COCH	intergenic	1.011	NA	5	14
14:31333514:C:T	14:31333514:A/G	T	C	0.4543	2.6959e-05	-0.197863	0.0471357	0.904522	14:31343135:A/G	64	COCH	intergenic	2.464	NA	5	15
14:31333660:A:G	14:31333660:A/G	G	A	0.4553	2.27721e-05	-0.200267	0.0472795	0.908307	14:31343135:A/G	64	COCH	intergenic	13.44	NA	5	14
14:31334942:C:T	14:31334942:A/G	T	C	0.4543	2.77408e-05	-0.197792	0.0471911	0.904522	14:31343135:A/G	64	COCH	intergenic	2.241	NA	5	14
14:31337866:C:T	14:31337866:A/G	T	C	0.4543	3.14813e-05	-0.198075	0.0475859	0.904522	14:31343135:A/G	64	COCH	intergenic	0.24	7	5	14
14:31338415:C:CA	rs373177340	CA	C	0.4742	NA	NA	NA	0.777837	14:31343135:A/G	64	COCH	intergenic	0.545	NA	5	14
14:31342700:G:GGA	rs35918712	G	GGA	0.4702	NA	NA	NA	0.996011	14:31343135:A/G	64	COCH	intergenic	7.734	NA	1	13
14:31343135:C:T	14:31343135:A/G	C	T	0.4712	6.00526e-06	-0.229132	0.0506234	1	14:31343135:A/G	64	COCH	upstream	6.618	1f	1	13
15:37584101:G:T	15:37584101:A/C	G	T	0.3857	0.000318133	0.169563	0.0471	0.767755	15:37611287:A/C	65	RP11-597G23.1	intergenic	3.413	6	7	15
15:37595916:C:G	15:37595916:C/G	C	G	0.3757	0.000113405	0.181145	0.0469289	0.793945	15:37611287:A/C	65	RP11-597G23.1	intergenic	2.321	6	15	15
15:37597668:A:G	15:37597668:A/G	A	G	0.3757	0.000104139	0.182162	0.0469398	0.793945	15:37611287:A/C	65	RP11-597G23.1	intergenic	2.021	6	15	15
15:37597709:A:C	15:37597709:A/C	A	C	0.3748	0.000102088	0.182326	0.0469237	0.796158	15:37611287:A/C	65	RP11-597G23.1	intergenic	1.681	7	15	15
15:37597916:A:G	15:37597916:A/G	G	A	0.3748	0.000101949	0.182334	0.0469218	0.796158	15:37611287:A/C	65	RP11-597G23.1	intergenic	2.651	6	15	15
15:37598392:A:G	15:37598392:A/G	A	G	0.3728	4.97014e-05	0.191209	0.04713	0.787668	15:37611287:A/C	65	RP11-597G23.1	intergenic	1.277	7	15	15
15:37598736:A:G	15:37598736:A/G	G	A	0.3748	0.000118774	0.180774	0.0469709	0.796158	15:37611287:A/C	65	RP11-597G23.1	intergenic	2.289	7	15	15
15:37599451:C:T	15:37599451:A/G	C	T	0.3748	0.00010167	0.18232	0.0469104	0.796158	15:37611287:A/C	65	RP11-597G23.1	intergenic	3.138	6	15	15
15:37599455:C:T	15:37599455:A/G	C	T	0.3748	0.000101674	0.18232	0.0469103	0.796158	15:37611287:A/C	65	RP11-597G23.1	intergenic	3.083	6	15	15
15:37599556:C:G	15:37599556:C/G	G	C	0.3748	0.000105692	0.181884	0.046912	0.796158	15:37611287:A/C	65	RP11-597G23.1	intergenic	1.481	7	15	15
15:37600039:A:G	15:37600039:A/G	A	G	0.3748	0.000101541	0.182318	0.046906	0.796158	15:37611287:A/C	65	RP11-597G23.1	intergenic	2.376	6	9	15
15:37600064:A:G	15:37600064:A/G	G	A	0.3748	0.000101577	0.182327	0.0469093	0.796158	15:37611287:A/C	65	RP11-597G23.1	intergenic	3.442	6	9	15
15:37600123:C:T	15:37600123:A/G	T	C	0.3738	9.932e-05	0.182649	0.0469262	0.790813	15:37611287:A/C	65	RP11-597G23.1	intergenic	2.071	7	9	15
15:37600774:C:T	15:37600774:A/G	C	T	0.3748	0.000110105	0.181291	0.0468794	0.796158	15:37611287:A/C	65	RP11-597G23.1	intergenic	4.566	6	9	15
15:37600921:A:G	15:37600921:A/G	G	A	0.3748	0.000122049	0.180203	0.0469037	0.796158	15:37611287:A/C	65	RP11-597G23.1	intergenic	4.482	6	9	15
15:37600960:A:G	15:37600960:A/G	A	G	0.3738	0.00020855	0.17433	0.0470089	0.790813	15:37611287:A/C	65	RP11-597G23.1	intergenic	2.636	7	9	15
15:37601059:G:T	15:37601059:A/C	T	G	0.3748	0.000131887	0.179399	0.0469273	0.796158	15:37611287:A/C	65	RP11-597G23.1	intergenic	1.674	7	9	15
15:37601641:C:T	15:37601641:A/G	T	C	0.3748	0.000154956	-0.177661	0.0469632	0.796158	15:37611287:A/C	65	RP11-597G23.1	intergenic	2.843	6	9	15
15:37602166:A:C	15:37602166:A/C	A	C	0.3748	0.000109816	0.181277	0.0468682	0.796158	15:37611287:A/C	65	RP11-597G23.1	intergenic	0.551	6	9	15
15:37602260:G:T	15:37602260:A/C	G	T	0.3748	0.000109812	0.181275	0.0468673	0.796158	15:37611287:A/C	65	RP11-720L8.1	intergenic	0.802	7	9	15
15:37603113:A:G	15:37603113:A/G	A	G	0.3748	0.000109929	0.181226	0.0468581	0.796158	15:37611287:A/C	65	RP11-720L8.1	intergenic	5.475	6	9	15
15:37603803:G:GA	rs5811975	G	GA	0.3738	NA	NA	NA	0.790813	15:37611287:A/C	65	RP11-720L8.1	intergenic	2.647	NA	9	15
15:37604088:C:G	15:37604088:C/G	G	C	0.3748	0.000110337	0.18114	0.0468463	0.796158	15:37611287:A/C	65	RP11-720L8.1	intergenic	0.122	5	9	15
15:37604602:A:G	15:37604602:A/G	G	A	0.4394	5.57534e-05	0.192182	0.0476865	0.600282	15:37611287:A/C	65	RP11-720L8.1	intergenic	5.613	7	15	15
15:37604998:C:T	15:37604998:A/G	C	T	0.3748	0.000101401	0.18215	0.0468586	0.796158	15:37611287:A/C	65	RP11-720L8.1	intergenic	2.109	7	15	15

15:37605067:A:G	15:37605067:A/G	G	A	0.3738	0.000115979	0.180862	0.0469224	0.791896	15:37611287:A/C	65	RP11-720L8.1	intergenic	2.19	7	15	15
15:37605148:A:G	15:37605148:A/G	A	G	0.3718	0.000137997	0.179307	0.0470409	0.790994	15:37611287:A/C	65	RP11-720L8.1	intergenic	4.217	7	15	15
15:37605454:A:G	15:37605454:A/G	G	A	0.3519	0.000200385	0.181907	0.0489193	0.755987	15:37611287:A/C	65	RP11-720L8.1	intergenic	1.069	6	15	15
15:37605501:A:T	15:37605501:A/T	A	T	0.3062	0.0171548	0.129601	0.0543767	0.609695	15:37611287:A/C	65	RP11-720L8.1	intergenic	1.16	6	15	15
15:37605901:A:G	15:37605901:A/G	G	A	0.3151	0.00213719	0.159631	0.0519883	0.61282	15:37611287:A/C	65	RP11-720L8.1	intergenic	1.32	7	15	15
15:37605909:A:G	15:37605909:A/G	G	A	0.3151	0.00214438	0.159578	0.051988	0.61282	15:37611287:A/C	65	RP11-720L8.1	intergenic	1.379	7	15	15
15:37605922:A:T	15:37605922:A/T	A	T	0.3151	0.00211247	0.159806	0.0519867	0.61282	15:37611287:A/C	65	RP11-720L8.1	intergenic	1.247	7	15	15
15:37606172:C:T	15:37606172:A/G	T	C	0.3738	0.000123754	0.180008	0.0468946	0.791896	15:37611287:A/C	65	RP11-720L8.1	intergenic	0.466	7	15	15
15:37606728:C:T	15:37606728:A/G	T	C	0.3608	0.00014418	0.181482	0.047747	0.76003	15:37611287:A/C	65	RP11-720L8.1	intergenic	0.809	6	15	15
15:37606729:A:G	15:37606729:A/G	G	A	0.3608	0.00014418	0.181482	0.047747	0.76003	15:37611287:A/C	65	RP11-720L8.1	intergenic	1.375	6	15	15
15:37606733:A:G	15:37606733:A/G	G	A	0.3608	0.000144198	0.18148	0.0477469	0.76003	15:37611287:A/C	65	RP11-720L8.1	intergenic	1.391	6	15	15
15:37606857:C:T	15:37606857:A/G	C	T	0.3748	0.000100975	0.182117	0.0468379	0.796158	15:37611287:A/C	65	RP11-720L8.1	intergenic	1.569	7	15	15
15:37606925:A:G	15:37606925:A/G	A	G	0.3539	0.000163594	0.183134	0.0485833	0.768856	15:37611287:A/C	65	RP11-720L8.1	intergenic	0.928	6	15	15
15:37607555:A:G	15:37607555:A/G	A	G	0.3728	0.000118043	0.180242	0.0468141	0.786566	15:37611287:A/C	65	RP11-720L8.1	intergenic	0.841	NA	9	15
15:37608034:G:T	15:37608034:A/C	G	T	0.3728	0.000126056	0.179423	0.0467975	0.786566	15:37611287:A/C	65	RP11-720L8.1	intergenic	1.174	6	9	15
15:37608441:A:G	15:37608441:A/G	A	G	0.3728	0.000118133	0.180215	0.0468096	0.786566	15:37611287:A/C	65	RP11-720L8.1	intergenic	0.747	7	9	15
15:37608932:A:G	15:37608932:A/G	G	A	0.3728	0.00011835	0.180196	0.0468099	0.786566	15:37611287:A/C	65	RP11-720L8.1	intergenic	1.581	6	9	15
15:37609819:A:C	15:37609819:A/C	A	C	0.3728	0.000128688	0.179335	0.0468365	0.786566	15:37611287:A/C	65	RP11-720L8.1	intergenic	0.549	7	9	15
15:37609865:G:T	15:37609865:A/C	G	T	0.3728	0.000128724	0.179333	0.0468368	0.786566	15:37611287:A/C	65	RP11-720L8.1	intergenic	1.314	7	9	15
15:37609895:C:T	15:37609895:A/G	T	C	0.3728	0.000128716	0.179333	0.0468366	0.786566	15:37611287:A/C	65	RP11-720L8.1	intergenic	0.65	6	9	15
15:37610421:A:G	15:37610421:A/G	A	G	0.3728	0.000118568	0.180176	0.0468103	0.786566	15:37611287:A/C	65	RP11-720L8.1	intergenic	0.905	7	9	15
15:37610687:A:G	15:37610687:A/G	G	A	0.3728	0.000119062	0.180133	0.0468114	0.786566	15:37611287:A/C	65	RP11-720L8.1	intergenic	1.138	6	9	15
15:37610903:C:T	15:37610903:A/G	C	T	0.3728	0.000132441	0.178857	0.0467982	0.786566	15:37611287:A/C	65	RP11-720L8.1	intergenic	1.332	6	9	15
15:37611063:A:C	15:37611063:A/C	C	A	0.3728	0.000132359	0.178874	0.0468007	0.786566	15:37611287:A/C	65	RP11-720L8.1	intergenic	1.017	6	9	15
15:37611287:A:C	15:37611287:A/C	C	A	0.325	9.30247e-06	0.22203	0.0500883	1	15:37611287:A/C	65	RP11-720L8.1	intergenic	0.807	6	9	15
15:37611453:A:T	15:37611453:A/T	T	A	0.3728	0.000132707	0.178836	0.0467988	0.786566	15:37611287:A/C	65	RP11-720L8.1	intergenic	1.202	6	9	15
15:37612179:C:G	15:37612179:C/G	G	C	0.3728	0.000119757	-0.180077	0.0468142	0.786566	15:37611287:A/C	65	RP11-720L8.1	intergenic	0.892	NA	9	15
15:37612179:A:G	15:37612179:A/G	G	A	0.03479	0.00415503	0.527898	0.184184	0.786566	15:37611287:A/C	65	RP11-720L8.1	NA	1.309	6	9	15
15:37612292:A:T	15:37612292:A/T	T	A	0.3738	0.000156501	0.177271	0.0468907	0.783248	15:37611287:A/C	65	RP11-720L8.1	intergenic	2.025	7	9	15
15:37612303:C:T	15:37612303:A/G	T	C	0.3738	0.000156515	0.17727	0.0468908	0.783248	15:37611287:A/C	65	RP11-720L8.1	intergenic	1.237	7	9	15
15:37612305:C:T	15:37612305:A/G	C	T	0.3738	0.000156515	0.17727	0.0468908	0.783248	15:37611287:A/C	65	RP11-720L8.1	intergenic	2.024	7	9	15
15:37612452:C:T	15:37612452:A/G	T	C	0.3728	0.000132692	0.178837	0.0467988	0.786566	15:37611287:A/C	65	RP11-720L8.1	intergenic	1.392	6	9	15
15:37612692:C:T	15:37612692:A/G	T	C	0.3728	0.00013278	0.17883	0.0467989	0.786566	15:37611287:A/C	65	RP11-720L8.1	intergenic	1.075	7	9	15
15:37612899:C:T	15:37612899:A/G	C	T	0.3728	0.000102115	0.182526	0.0469761	0.786566	15:37611287:A/C	65	RP11-720L8.1	intergenic	1.66	7	9	15
15:37613583:A:G	15:37613583:A/G	A	G	0.3728	0.000133177	0.178799	0.0467998	0.786566	15:37611287:A/C	65	RP11-720L8.1	intergenic	0.604	7	9	15
15:37614668:G:T	15:37614668:A/C	G	T	0.3738	0.000107017	0.181303	0.0467987	0.784356	15:37611287:A/C	65	RP11-720L8.1	intergenic	1.073	7	9	15
15:37615354:G:T	15:37615354:A/C	G	T	0.3728	0.00012056	0.179999	0.046814	0.786566	15:37611287:A/C	65	RP11-720L8.1	intergenic	1.078	6	9	15
15:37615735:G:T	15:37615735:A/C	T	G	0.3728	0.000123499	0.17977	0.0468264	0.786566	15:37611287:A/C	65	RP11-720L8.1	intergenic	0.494	7	9	15
15:37615907:A:G	15:37615907:A/G	G	A	0.3728	0.000120775	0.17998	0.0468144	0.786566	15:37611287:A/C	65	RP11-720L8.1	intergenic	1.188	7	9	15
15:37616491:G:T	15:37616491:A/C	G	T	0.3728	0.00012101	0.17996	0.0468149	0.786566	15:37611287:A/C	65	RP11-720L8.1	intergenic	0.776	6	9	15
15:37616778:C:T	15:37616778:A/G	T	C	0.3728	0.000120312	0.180028	0.0468153	0.786566	15:37611287:A/C	65	RP11-720L8.1	intergenic	0.599	6	9	15
15:37617600:A:C	15:37617600:A/C	C	A	0.3728	0.000121446	0.179922	0.0468157	0.786566	15:37611287:A/C	65	RP11-720L8.1	intergenic	0.845	7	9	15
15:37618534:G:T	15:37618534:A/C	G	T	0.3817	0.000199111	0.174158	0.0468148	0.76316	15:37611287:A/C	65	RP11-720L8.1	intergenic	9.711	7	9	15

15:37618817:A:G	15:37618817:A/G	A	G	0.3728	0.000121882	0.179891	0.0468184	0.786566	15:37611287:A/C	65	RP11-720L8.1	intergenic	2.095	7	9	15
15:37618963:C:T	15:37618963:A/G	T	C	0.3728	0.000121692	0.179918	0.0468209	0.786566	15:37611287:A/C	65	RP11-720L8.1	intergenic	0.034	7	9	15
15:37619102:G:T	15:37619102:A/C	G	T	0.3728	0.000122047	0.179879	0.0468194	0.786566	15:37611287:A/C	65	RP11-720L8.1	intergenic	0.267	6	9	15
15:37619275:C:T	15:37619275:A/G	C	T	0.3728	0.000122312	0.179856	0.0468199	0.786566	15:37611287:A/C	65	RP11-720L8.1	intergenic	1.684	6	9	15
15:37619728:A:G	15:37619728:A/G	A	G	0.3728	0.000122662	0.17983	0.0468216	0.786566	15:37611287:A/C	65	RP11-720L8.1	intergenic	0.248	NA	9	15
15:37620053:C:T	15:37620053:A/G	C	T	0.3728	0.00012511	0.179663	0.0468374	0.786566	15:37611287:A/C	65	RP11-720L8.1	intergenic	0.877	7	9	15
15:37620104:C:T	15:37620104:A/G	T	C	0.3728	0.000125627	0.179629	0.0468409	0.786566	15:37611287:A/C	65	RP11-720L8.1	intergenic	0.87	6	9	15
15:37620118:A:C	15:37620118:A/C	A	C	0.3728	0.000125258	0.179644	0.0468359	0.786566	15:37611287:A/C	65	RP11-720L8.1	intergenic	0.767	6	9	15
15:37620648:G:T	15:37620648:A/C	T	G	0.3728	0.000128621	0.179367	0.0468432	0.786566	15:37611287:A/C	65	RP11-720L8.1	intergenic	0.454	7	9	15
15:37628787:C:T	15:37628787:A/G	T	C	0.3459	9.00721e-05	0.184575	0.0471347	0.686516	15:37611287:A/C	65	RP11-720L8.1	intergenic	0.8	6	9	15
15:37630205:A:G	15:37630205:A/G	G	A	0.3469	8.37059e-05	0.185516	0.0471627	0.682945	15:37611287:A/C	65	RP11-720L8.1	intergenic	1.115	7	9	15
15:87439330:A:G	15:87439330:A/G	G	A	0.1024	0.000233747	-0.330296	0.0897677	0.715871	15:87449577:A/G	66	AGBL1	intronic	0.379	7	9	15
15:87439332:G:T	15:87439332:A/C	T	G	0.1044	0.000558451	-0.312617	0.0905863	0.702201	15:87449577:A/G	66	AGBL1	intronic	0.105	7	9	15
15:87439628:T:TAA	rs79782840	TAA	T	0.0994	NA	NA	NA	0.678395	15:87449577:A/G	66	AGBL1	intronic	0.279	NA	9	15
15:87440294:C:T	15:87440294:A/G	C	T	0.1143	0.000110928	-0.305603	0.0790622	0.887916	15:87449577:A/G	66	AGBL1	intronic	0.61	6	9	15
15:87440334:G:T	15:87440334:A/C	T	G	0.1123	0.000120511	-0.304499	0.0791918	0.905213	15:87449577:A/G	66	AGBL1	intronic	0.946	6	9	15
15:87441471:G:T	15:87441471:A/C	T	G	0.1064	3.95898e-06	-0.379476	0.0822531	1	15:87449577:A/G	66	AGBL1	intronic	1.664	7	9	15
15:87441514:A:G	15:87441514:A/G	A	G	0.1143	4.56686e-05	-0.316015	0.0775164	0.925088	15:87449577:A/G	66	AGBL1	intronic	1.972	7	9	15
15:87441734:A:T	15:87441734:A/T	A	T	0.0994	7.75014e-05	-0.345027	0.0873042	0.776042	15:87449577:A/G	66	AGBL1	intronic	2.383	7	9	15
15:87442894:C:T	15:87442894:A/G	T	C	0.09046	7.13159e-06	-0.416032	0.0926627	0.845459	15:87449577:A/G	66	AGBL1	intronic	3.718	6	9	15
15:87443538:C:T	15:87443538:A/G	T	C	0.09245	7.85481e-06	-0.413624	0.0925516	0.824609	15:87449577:A/G	66	AGBL1	intronic	4.328	7	9	15
15:87444337:G:T	15:87444337:A/C	G	T	0.164	0.000302686	-0.26102	0.0722448	0.61118	15:87449577:A/G	66	AGBL1	intronic	5.03	5	9	15
15:87444509:A:G	15:87444509:A/G	G	A	0.1143	0.000129104	-0.338164	0.0883358	0.63689	15:87449577:A/G	66	AGBL1	intronic	3.418	6	9	15
15:87444682:C:T	15:87444682:A/G	C	T	0.164	0.000288617	-0.260932	0.0719753	0.61118	15:87449577:A/G	66	AGBL1	intronic	0.236	7	9	15
15:87448688:C:T	15:87448688:A/G	C	T	0.1083	0.000546692	-0.303828	0.0878933	0.688439	15:87449577:A/G	66	AGBL1	intronic	0.089	7	5	15
15:87449130:A:G	15:87449130:A/G	G	A	0.1143	7.09495e-06	-0.362418	0.080702	0.907749	15:87449577:A/G	66	AGBL1	intronic	4.292	7	5	15
15:87449577:C:T	15:87449577:A/G	T	C	0.1064	3.2341e-06	-0.378681	0.0813432	1	15:87449577:A/G	66	AGBL1	intronic	0.649	5	5	15
15:87458272:G:T	15:87458272:A/C	T	G	0.1233	7.23564e-05	-0.333866	0.0841308	0.773798	15:87449577:A/G	66	AGBL1	intronic	0.293	7	5	15
15:87458777:A:T	15:87458777:A/T	A	T	0.1074	0.000461153	-0.363445	0.103771	0.614662	15:87449577:A/G	66	AGBL1	intronic	6.782	5	5	15
15:87458828:A:G	15:87458828:A/G	A	G	0.1074	0.000260967	-0.379523	0.103943	0.614662	15:87449577:A/G	66	AGBL1	intronic	1.306	NA	2	15
15:88368416:A:C	15:88368416:A/C	A	C	0.07157	1.33672e-06	-0.581371	0.120261	1	15:88368416:A/C	67	NTRK3	intergenic	1.203	6	5	15
16:79331216:C:T	16:79331216:A/G	C	T	0.01193	0.0474254	-0.507657	0.256071	0.605755	16:79415503:A/G	68	RNA5SP431	intergenic	0.055	6	5	15
16:79331712:A:C	16:79331712:A/C	C	A	0.01193	0.0384512	-0.548783	0.265111	0.605755	16:79415503:A/G	68	RNA5SP431	intergenic	3.134	7	5	15
16:79369755:C:T	16:79369755:A/G	T	C	0.01193	0.00105726	0.520354	0.158895	0.752345	16:79415503:A/G	68	RNA5SP431	intergenic	2.464	5	5	15
16:79369930:A:G	16:79369930:A/G	A	G	0.01392	0.000704799	0.514904	0.151992	0.641701	16:79415503:A/G	68	RNA5SP431	intergenic	4.451	6	5	15
16:79371686:C:T	16:79371686:A/G	C	T	0.01193	0.000757491	0.521469	0.154836	0.752345	16:79415503:A/G	68	RNA5SP431	intergenic	0.007	5	5	15
16:79371993:A:G	16:79371993:A/G	A	G	0.01193	0.000781948	0.519551	0.154669	0.752345	16:79415503:A/G	68	RNA5SP431	intergenic	0.22	5	5	15
16:79372899:G:T	16:79372899:A/C	T	G	0.007952	0.000247102	-0.912508	0.248962	0.722865	16:79415503:A/G	68	RNA5SP431	intergenic	2.645	7	5	15
16:79377362:A:G	16:79377362:A/G	G	A	0.01491	0.000692616	0.521652	0.153767	0.863129	16:79400539:C/G	68	RNA5SP431	intergenic	0.928	7	5	15
16:79385123:C:G	16:79385123:C/G	G	C	0.01392	0.000953593	0.492347	0.149021	0.785831	16:79400539:C/G	68	RNA5SP431	intergenic	0.183	7	5	15
16:79388278:G:T	16:79388278:A/C	G	T	0.01193	0.00178653	0.466854	0.14946	0.752345	16:79415503:A/G	68	RNA5SP431	intergenic	4.245	NA	5	15
16:79388303:C:T	16:79388303:A/G	T	C	0.01292	0.00119296	0.454606	0.140286	0.692767	16:79415503:A/G	68	RNA5SP431	intergenic	0.227	5	5	15
16:79391184:A:AC	rs144959615	AC	A	0.01292	NA	NA	NA	0.842714	16:79415503:A/G	68	RNA5SP431	intergenic	1.253	NA	5	15

16:79391219:C:T	16:79391219:A/G	T	C	0.01193	0.000399256	0.528638	0.149308	0.752345	16:79415503:A/G	68	RNA5SP431	intergenic	0.64	7	5	15
16:79391350:A:C	16:79391350:A/C	C	A	0.00994	1.90027e-05	-0.858208	0.200689	0.907247	16:79415503:A/G	68	RNA5SP431	intergenic	0.52	6	5	15
16:79391438:G:T	16:79391438:A/C	T	G	0.01193	0.00026803	0.563572	0.154641	0.752345	16:79415503:A/G	68	RNA5SP431	intergenic	0.498	7	5	15
16:79391848:C:G	16:79391848:C/G	C	G	0.01193	0.000306099	0.551984	0.152901	0.752345	16:79415503:A/G	68	RNA5SP431	intergenic	1.866	7	5	15
16:79392306:C:T	16:79392306:A/G	T	C	0.01093	1.39296e-05	-0.870467	0.200339	0.842714	16:79400539:C/G	68	RNA5SP431	intergenic	8.908	6	5	15
16:79392954:A:G	16:79392954:A/G	A	G	0.01193	0.000412447	0.539721	0.152809	0.752345	16:79415503:A/G	68	RNA5SP431	intergenic	4.957	6	5	15
16:79394082:C:T	16:79394082:A/G	C	T	0.01392	0.000231594	0.564763	0.153393	0.785831	16:79400539:C/G	68	RNA5SP431	intergenic	0.037	7	5	15
16:79394646:C:G	16:79394646:C/G	G	C	0.00994	0.000396705	0.552697	0.156029	0.907247	16:79415503:A/G	68	RNA5SP431	intergenic	1.366	7	5	15
16:79395948:A:G	16:79395948:A/G	G	A	0.01193	1.32851e-05	-0.872239	0.200268	0.921197	16:79400539:C/G	68	RNA5SP431	intergenic	1.65	5	2	15
16:79396484:C:T	16:79396484:A/G	T	C	0.01093	0.000353798	0.553931	0.155061	0.842714	16:79400539:C/G	68	RNA5SP431	intergenic	0.03	5	1	15
16:79397909:C:T	16:79397909:A/G	T	C	0.01093	0.000254384	0.580909	0.158814	0.842714	16:79400539:C/G	68	RNA5SP431	intergenic	0.282	NA	5	15
16:79397910:C:G	16:79397910:C/G	C	G	0.01093	0.000254381	0.58091	0.158814	0.842714	16:79400539:C/G	68	RNA5SP431	intergenic	0.023	NA	5	15
16:79397913:A:G	16:79397913:A/G	G	A	0.01093	0.000254318	0.580917	0.158813	0.842714	16:79400539:C/G	68	RNA5SP431	intergenic	1.827	NA	5	15
16:79397914:A:C	16:79397914:A/C	C	A	0.01093	0.000254318	0.580917	0.158813	0.842714	16:79400539:C/G	68	RNA5SP431	intergenic	1.583	NA	5	15
16:79397915:A:G	16:79397915:A/G	A	G	0.01093	0.000254318	0.580917	0.158813	0.842714	16:79400539:C/G	68	RNA5SP431	intergenic	1.226	NA	5	15
16:79398454:C:G	16:79398454:C/G	G	C	0.01292	0.000378573	0.539581	0.151799	0.848308	16:79400539:C/G	68	RNA5SP431	intergenic	5.002	6	5	15
16:79398656:C:T	16:79398656:A/G	C	T	0.01292	0.000423104	0.532289	0.150994	0.848308	16:79400539:C/G	68	RNA5SP431	intergenic	2.001	7	5	15
16:79398961:C:T	16:79398961:A/G	C	T	0.01392	0.000408626	0.533519	0.150948	0.926676	16:79400539:C/G	68	RNA5SP431	intergenic	1.104	5	5	15
16:79399237:C:T	16:79399237:A/G	C	T	0.01292	0.000452283	0.526932	0.150229	0.848308	16:79400539:C/G	68	RNA5SP431	intergenic	6.017	6	5	15
16:79399383:A:G	16:79399383:A/G	A	G	0.01193	0.000355203	0.540931	0.151466	0.921197	16:79400539:C/G	68	RNA5SP431	intergenic	4.315	7	5	15
16:79399653:A:AGGG	rs140454269	A	AGGG	0.01193	NA	NA	NA	0.921197	16:79400539:C/G	68	RNA5SP431	intergenic	2.157	NA	5	15
16:79400539:C:G	16:79400539:C/G	G	C	0.01292	9.59564e-06	-0.875453	0.197794	1	16:79400539:C/G	68	RNA5SP431	intergenic	1.823	7	5	15
16:79403297:G:T	16:79403297:A/C	G	T	0.01491	0.000383785	0.529614	0.149146	0.731685	16:79400539:C/G	68	RNA5SP431	intergenic	0.308	6	7	15
16:79407767:A:C	16:79407767:A/C	C	A	0.01093	9.15294e-06	-0.874592	0.197146	1	16:79415503:A/G	68	RNA5SP431	intergenic	0.194	7	5	15
16:79410146:A:T	16:79410146:A/T	T	A	0.01093	4.70911e-05	0.679324	0.166926	1	16:79415503:A/G	68	RNA5SP431	intergenic	1.175	7	5	15
16:79413261:C:G	16:79413261:C/G	G	C	0.01093	0.000446149	0.558841	0.159161	1	16:79415503:A/G	68	RNA5SP431	intergenic	1.178	4	5	15
16:79415503:C:T	16:79415503:A/G	T	C	0.01093	4.62339e-05	0.68056	0.167054	1	16:79415503:A/G	68	RNA5SP431	intergenic	3.25	5	1	15
16:79417709:A:C	16:79417709:A/C	A	C	0.01292	4.99429e-05	0.668602	0.164847	1	16:79400539:C/G	68	RNA5SP431	intergenic	1.226	7	5	15
16:79432837:C:T	16:79432837:A/G	T	C	0.01193	0.000390602	-0.572502	0.161434	0.632949	16:79400539:C/G	68	RP11-467I17.1	intergenic	3.559	5	5	15
16:79433043:A:G	16:79433043:A/G	A	G	0.01193	0.000411207	-0.569983	0.161341	0.632949	16:79400539:C/G	68	RP11-467I17.1	intergenic	0.214	5	5	15
16:79433141:A:G	16:79433141:A/G	G	A	0.01193	0.000374426	-0.573906	0.161323	0.632949	16:79400539:C/G	68	RP11-467I17.1	intergenic	0.733	3a	5	15
16:79433474:A:C	16:79433474:A/C	A	C	0.01193	0.000357568	-0.575356	0.161183	0.632949	16:79400539:C/G	68	RP11-467I17.1	intergenic	1.583	5	5	15
16:79436479:A:G	16:79436479:A/G	G	A	0.01193	0.000680073	-0.582279	0.171385	0.632949	16:79400539:C/G	68	RP11-467I17.1	intergenic	11.36	4	5	15
16:79437472:C:T	16:79437472:A/G	T	C	0.01193	0.00124447	-0.497637	0.154139	0.632949	16:79400539:C/G	68	RP11-467I17.1	intergenic	1.913	5	5	15
16:79437704:G:T	16:79437704:A/C	G	T	0.01193	0.00129103	-0.495592	0.154008	0.632949	16:79400539:C/G	68	RP11-467I17.1	intergenic	1.62	2b	5	15
16:79437744:A:G	16:79437744:A/G	A	G	0.01193	0.000882562	-0.53802	0.161786	0.632949	16:79400539:C/G	68	RP11-467I17.1	intergenic	2.697	4	5	15
16:79437782:G:T	16:79437782:A/C	G	T	0.01193	0.00127172	-0.49612	0.153965	0.632949	16:79400539:C/G	68	RP11-467I17.1	intergenic	0.155	4	5	15
16:79444393:A:T	16:79444393:A/T	A	T	0.00994	0.00160875	-0.522355	0.1656	0.61564	16:79400539:C/G	68	RP11-467I17.1	intergenic	0.909	6	5	15
16:79446445:A:G	16:79446445:A/G	A	G	0.008946	0.00103928	-0.547615	0.166973	0.686702	16:79400539:C/G	68	RP11-467I17.1	intergenic	5.325	7	2	15
16:79449539:A:G	16:79449539:A/G	G	A	0.00994	0.00223251	-0.500688	0.163762	0.61564	16:79400539:C/G	68	RP11-467I17.1	intergenic	0.237	5	5	15
16:79452042:C:T	16:79452042:A/G	T	C	0.008946	0.00645834	-0.441645	0.162157	0.686702	16:79400539:C/G	68	RP11-467I17.1	intergenic	0.251	5	2	15
16:89511210:T:TA	rs139086360	T	TA	0.05964	NA	NA	NA	0.966046	16:89514364:A/C	69	ANKRD11	intronic	4.716	NA	2	7
16:89512498:A:T	16:89512498:A/T	A	T	0.04672	1.378e-05	-0.386891	0.088995	0.750692	16:89514364:A/C	69	ANKRD11	intronic	2.76	5	5	7

16:89512810:A:G	16:89512810:A/G	A	G	0.04672	1.39335e-05	-0.386636	0.0889861	0.750692	16:89514364:A/C	69	ANKRD11	intronic	0.5	5	2	7
16:89514364:A:C	16:89514364:A/C	A	C	0.06163	1.03529e-05	-0.383577	0.086985	1	16:89514364:A/C	69	ANKRD11	intronic	7.291	7	5	5
16:89517502:C:G	16:89517502:C/G	C	G	0.04473	1.26667e-05	-0.394636	0.0903931	0.718647	16:89514364:A/C	69	ANKRD11	intronic	2.817	5	5	5
16:89534004:A:T	16:89534004:A/T	A	T	0.05865	1.21348e-05	-0.388711	0.0888452	0.914668	16:89514364:A/C	69	ANKRD11	intronic	4.738	5	4	5
16:89543154:A:C	16:89543154:A/C	A	C	0.06064	2.36723e-05	-0.378552	0.0895536	0.949001	16:89514364:A/C	69	ANKRD11	intronic	4.662	4	2	7
16:89545575:G:T	16:89545575:A/C	T	G	0.04573	1.19223e-05	-0.399896	0.0913211	0.70255	16:89514364:A/C	69	ANKRD11	intronic	3.691	4	2	7
16:89561123:C:T	16:89561123:A/G	T	C	0.06362	0.000138073	-0.329183	0.0863634	0.83823	16:89514364:A/C	69	SPG7:RP11-104N10.1	ncRNA_intronic	0.425	5	2	5
16:89582928:C:T	16:89582928:A/G	T	C	0.06461	0.000464015	-0.316366	0.090372	0.735127	16:89514364:A/C	69	SPG7	intronic	6.954	7	4	4
16:89612511:G:T	16:89612511:A/C	G	T	0.07356	0.00301869	-0.248984	0.0839509	0.67377	16:89514364:A/C	69	SPG7	intronic	0.227	2b	2	4
17:11579621:C:T	rs537510979	C	T	0.00994	NA	NA	NA	0.668616	17:11642231:A/G	70	DNAH9	intronic	0.071	NA	5	15
17:11581321:C:CTA	rs10544168	C	CTA	0.01093	NA	NA	NA	0.752345	17:11642231:A/G	70	DNAH9	intronic	1.252	NA	5	15
17:11582854:A:G	17:11582854:A/G	G	A	0.01193	0.000784959	-0.591964	0.176282	0.713575	17:11655126:A/C	70	DNAH9	intronic	0.097	6	5	15
17:11583089:C:T	17:11583089:A/G	C	T	0.01093	0.00175197	-0.558439	0.178453	0.752345	17:11642231:A/G	70	DNAH9	exonic	0.35	7	5	15
17:11588360:G:T	17:11588360:A/C	T	G	0.01093	0.00092058	-0.567748	0.171331	0.752345	17:11642231:A/G	70	DNAH9	intronic	5.47	6	5	15
17:11589152:C:T	17:11589152:A/G	T	C	0.01093	0.00112771	0.592109	0.18182	0.752345	17:11642231:A/G	70	DNAH9	intronic	1.421	7	5	15
17:11591418:A:G	17:11591418:A/G	A	G	0.01093	0.00170474	-0.561947	0.179114	0.752345	17:11642231:A/G	70	DNAH9	intronic	0.49	6	5	15
17:11595385:C:CT	rs5819332	CT	C	0.01093	NA	NA	NA	0.752345	17:11642231:A/G	70	DNAH9	intronic	0.637	NA	5	15
17:11596648:A:C	17:11596648:A/C	A	C	0.01093	0.000351434	-0.615989	0.172348	0.752345	17:11642231:A/G	70	DNAH9	intronic	0.653	7	5	15
17:11596796:A:G	17:11596796:A/G	G	A	0.01093	0.000349725	-0.616423	0.172408	0.752345	17:11642231:A/G	70	DNAH9	intronic	0.487	7	5	15
17:11603300:G:T	17:11603300:A/C	T	G	0.01491	0.0036474	-0.386386	0.132909	0.677319	17:11655126:A/C	70	DNAH9	intronic	1.283	NA	5	15
17:11604598:A:T	17:11604598:A/T	A	T	0.01093	0.000185338	-0.631393	0.168903	0.752345	17:11642231:A/G	70	DNAH9	intronic	0.355	7	5	15
17:11605565:A:T	17:11605565:A/T	A	T	0.01093	0.000182491	-0.632189	0.16894	0.752345	17:11642231:A/G	70	DNAH9	intronic	1.169	7	5	15
17:11606440:A:G	rs56382756	A	G	0.00994	NA	NA	NA	0.668616	17:11642231:A/G	70	DNAH9	intronic	0.2	7	5	15
17:11606483:C:T	17:11606483:A/G	T	C	0.01093	0.000182354	-0.632386	0.168984	0.752345	17:11642231:A/G	70	DNAH9	intronic	1.58	7	5	15
17:11609610:G:GT	rs5819333	G	GT	0.01093	NA	NA	NA	0.752345	17:11642231:A/G	70	DNAH9	intronic	0.455	NA	5	15
17:11609637:C:T	rs117522326	T	C	0.00994	NA	NA	NA	0.668616	17:11642231:A/G	70	DNAH9	intronic	0.61	7	5	15
17:11612969:C:T	17:11612969:A/G	C	T	0.01093	0.000167729	-0.6324	0.168047	0.752345	17:11642231:A/G	70	DNAH9	intronic	6.305	NA	5	15
17:11613470:C:T	17:11613470:A/G	C	T	0.01392	0.00379835	-0.383796	0.132597	0.727699	17:11655126:A/C	70	DNAH9	intronic	0.547	NA	5	15
17:11613656:A:G	17:11613656:A/G	G	A	0.01093	0.000187857	-0.627399	0.167987	0.752345	17:11642231:A/G	70	DNAH9	intronic	0.19	NA	5	15
17:11613987:A:T	17:11613987:A/T	T	A	0.01392	0.00277852	-0.397761	0.132976	0.727699	17:11655126:A/C	70	DNAH9	intronic	0.576	7	5	15
17:11614385:C:G	17:11614385:C/G	G	C	0.01292	0.000117864	-0.606542	0.157522	0.785831	17:11655126:A/C	70	DNAH9	intronic	1.32	5	5	15
17:11614568:C:T	17:11614568:A/G	C	T	0.01292	0.000117774	-0.606593	0.157527	0.785831	17:11655126:A/C	70	DNAH9	intronic	11.97	3a	1	15
17:11615003:A:G	17:11615003:A/G	G	A	0.01292	0.00011817	-0.606661	0.157579	0.785831	17:11655126:A/C	70	DNAH9	intronic	4.123	7	5	15
17:11615611:A:C	17:11615611:A/C	C	A	0.01093	0.00018468	-0.628296	0.168034	0.752345	17:11642231:A/G	70	DNAH9	intronic	1.998	6	7	15
17:11615787:A:G	17:11615787:A/G	G	A	0.01392	0.000909599	-0.445317	0.134249	0.727699	17:11655126:A/C	70	DNAH9	intronic	4.215	6	7	15
17:11616900:A:G	17:11616900:A/G	G	A	0.01392	0.000907737	-0.445464	0.13427	0.727699	17:11655126:A/C	70	DNAH9	intronic	3.607	6	9	15
17:11617016:A:G	17:11617016:A/G	A	G	0.01193	0.000971152	-0.477018	0.144606	0.687672	17:11642231:A/G	70	DNAH9	intronic	5.116	6	9	15
17:11617585:A:G	17:11617585:A/G	G	A	0.01093	0.000187198	-0.627871	0.168073	0.752345	17:11642231:A/G	70	DNAH9	intronic	2.068	7	9	15
17:11617763:A:G	17:11617763:A/G	A	G	0.01392	0.000862106	-0.447712	0.134366	0.727699	17:11655126:A/C	70	DNAH9	intronic	6.205	6	9	15
17:11618498:G:T	17:11618498:A/C	T	G	0.01193	0.00139724	-0.453702	0.141994	0.687672	17:11642231:A/G	70	DNAH9	intronic	12.7	6	9	15
17:11619568:A:T	17:11619568:A/T	A	T	0.01392	0.000711008	-0.454429	0.134236	0.727699	17:11655126:A/C	70	DNAH9	intronic	3.193	6	9	15
17:11621461:C:T	17:11621461:A/G	C	T	0.01093	0.000184286	-0.629681	0.16838	0.752345	17:11642231:A/G	70	DNAH9	intronic	0.466	6	9	15

17:11622270:A:G	17:11622270:A/G	G	A	0.01392	0.000864505	-0.449212	0.134847	0.727699	17:11655126:A/C	70	DNAH9	intronic	0.332	5	9	15
17:11622460:C:T	17:11622460:A/G	T	C	0.01292	0.0001108	-0.612063	0.158334	0.785831	17:11655126:A/C	70	DNAH9	intronic	1.665	5	9	15
17:11622494:A:G	17:11622494:A/G	A	G	0.01392	0.000840273	0.450123	0.134801	0.727699	17:11655126:A/C	70	DNAH9	intronic	2.634	4	9	15
17:11622953:A:G	17:11622953:A/G	A	G	0.01093	0.000191115	-0.632324	0.169502	0.752345	17:11642231:A/G	70	DNAH9	intronic	0.494	7	7	15
17:11625676:A:G	17:11625676:A/G	G	A	0.01193	4.93709e-05	0.704554	0.173596	1	17:11642231:A/G	70	DNAH9	intronic	2.055	7	5	15
17:11626982:A:AT	rs111749738	AT	A	0.01789	NA	NA	NA	0.65852	17:11642231:A/G	70	DNAH9	intronic	0.545	NA	5	15
17:11627571:C:G	17:11627571:C/G	G	C	0.01193	4.95015e-05	0.701519	0.172874	1	17:11642231:A/G	70	DNAH9	intronic	1.127	6	5	15
17:11629171:A:G	17:11629171:A/G	G	A	0.01193	4.71816e-05	0.686931	0.168813	1	17:11642231:A/G	70	DNAH9	intronic	4.588	6	5	15
17:11629183:C:CA	rs201362147	C	CA	0.01193	NA	NA	NA	1	17:11642231:A/G	70	DNAH9	intronic	5.403	NA	5	15
17:11629570:C:T	17:11629570:A/G	T	C	0.01193	0.0014235	-0.513415	0.160953	1	17:11642231:A/G	70	DNAH9	intronic	1.935	6	5	15
17:11629624:C:T	17:11629624:A/G	C	T	0.01193	0.00142157	-0.513445	0.160942	1	17:11642231:A/G	70	DNAH9	intronic	1.073	7	5	15
17:11629684:C:T	17:11629684:A/G	C	T	0.01193	0.00142245	-0.513384	0.160932	1	17:11642231:A/G	70	DNAH9	intronic	6.106	6	5	15
17:11631855:A:AAC	rs140653405	A	AAC	0.01193	NA	NA	NA	1	17:11642231:A/G	70	DNAH9	intronic	0.982	NA	5	15
17:11631857:A:C	17:11631857:A/C	A	C	0.01193	0.00122771	-0.499091	0.154404	1	17:11642231:A/G	70	DNAH9	intronic	0.874	6	5	15
17:11631908:A:T	17:11631908:A/T	T	A	0.01193	0.00167411	-0.502358	0.15985	1	17:11642231:A/G	70	DNAH9	intronic	6.355	7	5	15
17:11640265:C:T	17:11640265:A/G	C	T	0.01193	7.69986e-05	0.676526	0.171119	1	17:11642231:A/G	70	DNAH9	intronic	6.641	4	1	15
17:11642175:C:T	17:11642175:A/G	T	C	0.01193	8.3505e-05	0.67223	0.170872	1	17:11642231:A/G	70	DNAH9	intronic	0.242	5	9	15
17:11642231:A:G	17:11642231:A/G	G	A	0.01193	4.51421e-05	0.716952	0.175747	1	17:11642231:A/G	70	DNAH9	exonic	22	5	9	15
17:11648823:C:T	17:11648823:A/G	C	T	0.01193	0.00629948	-0.790975	0.289546	1	17:11642231:A/G	70	DNAH9	intronic	0.818	6	9	15
17:11651377:C:T	17:11651377:A/G	C	T	0.01193	6.68246e-05	0.683169	0.171335	1	17:11642231:A/G	70	DNAH9	intronic	4.765	7	9	15
17:11652480:C:G	17:11652480:C/G	G	C	0.01193	6.16821e-05	0.687041	0.171491	1	17:11642231:A/G	70	DNAH9	intronic	0.83	6	9	15
17:11655126:G:T	17:11655126:A/C	T	G	0.01392	2.58207e-05	-0.716527	0.170297	1	17:11655126:A/C	70	DNAH9	intronic	2.415	6	9	15
17:11655858:C:T	17:11655858:A/G	C	T	0.01491	3.79262e-05	-0.704471	0.170997	0.931425	17:11655126:A/C	70	DNAH9	intronic	2.677	7	9	15
17:11660722:G:T	17:11660722:A/C	T	G	0.01491	8.60772e-05	-0.677612	0.17256	0.931425	17:11655126:A/C	70	DNAH9	intronic	2.15	6	5	15
17:11662413:T:TTTC	rs201864129	T	TTTC	0.01491	NA	NA	NA	0.931425	17:11655126:A/C	70	DNAH9	intronic	0.474	NA	5	15
17:11663394:A:G	17:11663394:A/G	G	A	0.01491	7.42287e-05	-0.685019	0.172884	0.931425	17:11655126:A/C	70	DNAH9	intronic	0.182	7	5	15
17:11664658:A:G	17:11664658:A/G	A	G	0.01491	6.27431e-05	-0.690392	0.172501	0.931425	17:11655126:A/C	70	DNAH9	intronic	0.045	7	5	15
17:11665655:A:G	17:11665655:A/G	G	A	0.01491	9.66786e-05	-0.677793	0.173847	0.931425	17:11655126:A/C	70	DNAH9	intronic	0.093	7	5	15
17:18967266:C:G	17:18967266:C/G	G	C	0.07753	5.08941e-06	0.596429	0.130766	1	17:18967266:C/G	71	SNORD3B-2	ncRNA_exonic	1.732	NA	1	15
17:25271136:A:ACT	rs142515842	A	ACT	0.3449	NA	NA	NA	0.910298	17:25541278:A/C	72	RP11-260A9.1	intergenic	1.838	NA	5	9
17:25278529:C:CTT	rs141329779	CTT	C	0.3748	NA	NA	NA	0.773879	17:25541278:A/C	72	RP11-260A9.1	intergenic	0.737	NA	1	9
17:25289229:C:CTCTA	rs138697975	CTCTA	C	0.2644	NA	NA	NA	0.619108	17:25541278:A/C	72	RP11-260A9.1	intergenic	6.742	NA	8	9
17:25290222:G:GC	rs146890704	G	GC	0.3449	NA	NA	NA	0.910298	17:25541278:A/C	72	RP11-260A9.1	intergenic	7.454	NA	8	9
17:25302994:G:GTC	rs201983419	G	GTC	0.336	NA	NA	NA	0.825687	17:25541278:A/C	72	RP11-260A9.1	intergenic	0.998	NA	8	15
17:25328463:C:CT	rs35303382	CT	C	0.3449	NA	NA	NA	0.910298	17:25541278:A/C	72	PDLIM1P3	intergenic	0.444	NA	9	15
17:25398171:G:GT	rs34564798	G	GT	0.328	NA	NA	NA	0.983368	17:25541278:A/C	72	TUFMP1	intergenic	1.014	NA	4	15
17:25399144:T:TA	rs35743485	TA	T	0.328	NA	NA	NA	0.983368	17:25541278:A/C	72	TUFMP1	intergenic	0.799	NA	4	15
17:25404731:G:GA	rs60312733	G	GA	0.3221	NA	NA	NA	0.916792	17:25541278:A/C	72	TUFMP1	intergenic	0.515	NA	4	15
17:25407207:C:CT	rs11444267	C	CT	0.2674	NA	NA	NA	0.737743	17:25541278:A/C	72	TUFMP1	intergenic	3.906	NA	4	15
17:25412844:A:AC	rs35585092	A	AC	0.2654	NA	NA	NA	0.747561	17:25541278:A/C	72	TUFMP1	intergenic	0.59	NA	2	15
17:25447979:A:AACACACACAC	rs111667803	AACACACACAC	A	0.2753	NA	NA	NA	0.783178	17:25541278:A/C	72	TUFMP1	intergenic	1.857	NA	5	15
17:25452226:G:T	rs374445179	G	T	0.000994	NA	NA	NA	0.99584	17:25541278:A/C	72	TUFMP1	NA	1.554	NA	5	15

17:25452226:G:GTC	rs146140481	G	GTC	0.327	NA	NA	NA	0.99584	17:25541278:A/C	72	TUFMP1	intergenic	1.712	NA	5	15
17:25453507:A:AAAG	rs58901700	AAAG	A	0.2664	NA	NA	NA	0.750958	17:25541278:A/C	72	TUFMP1	intergenic	2.621	NA	5	15
17:25461575:G:GA	rs34425468	GA	G	0.327	NA	NA	NA	0.99584	17:25541278:A/C	72	TUFMP1	intergenic	0.6	NA	5	15
17:25463772:G:GAA	rs57258604	GAA	G	0.2664	NA	NA	NA	0.750958	17:25541278:A/C	72	TUFMP1	intergenic	0.569	NA	5	15
17:25463944:A:ATGC	rs34008519	A	ATGC	0.327	NA	NA	NA	0.99584	17:25541278:A/C	72	TUFMP1	intergenic	0.798	NA	5	15
17:25473879:G:GACAA	rs200439087	GACAA	G	0.2664	NA	NA	NA	0.750958	17:25541278:A/C	72	TUFMP1	intergenic	0.388	NA	5	15
17:25475744:T:TAAA	rs149985739	T	TAAA	0.327	NA	NA	NA	0.99584	17:25541278:A/C	72	TUFMP1	intergenic	1.762	NA	5	15
17:25477048:A:AG	rs71369080	AG	A	0.2664	NA	NA	NA	0.750958	17:25541278:A/C	72	TUFMP1	intergenic	2.023	NA	5	15
17:25480105:G:GACAC	rs147610256	G	GACAC	0.2376	NA	NA	NA	0.668244	17:25541278:A/C	72	TUFMP1	intergenic	1.38	NA	5	15
17:25484993:G:GA	rs35425176	G	GA	0.2555	NA	NA	NA	0.716691	17:25541278:A/C	72	TUFMP1	intergenic	0.71	NA	8	15
17:25492776:A:ATTCTTTTTTTTACTGTAATATAT	rs528766573	ATTCTTTTTTTTACTGTAATATAT	A	0.341	NA	NA	NA	0.825725	17:25541278:A/C	72	RP11-663N22.1	intergenic	1.422	NA	9	15
17:25494522:C:CT	rs71359209	C	CT	0.327	NA	NA	NA	0.99584	17:25541278:A/C	72	RP11-663N22.1	intergenic	4.762	NA	9	15
17:25523208:C:CT	rs57211242	C	CT	0.327	NA	NA	NA	0.99584	17:25541278:A/C	72	RP11-663N22.1	intergenic	2.038	NA	9	15
17:25541278:A:C	17:25541278:A/C	C	A	0.328	3.85254e-14	0.615635	0.08137	1	17:25541278:A/C	72	RP11-663N22.1	intergenic	2.236	5	9	15
17:43934332:C:T	17:43934332:A/G	T	C	0.1402	0.0305433	-0.178633	0.0825869	0.67784	17:43962562:A/G	73	MAPT-AS1	ncRNA_intronic	5.805	7	5	14
17:43934387:A:G	17:43934387:A/G	A	G	0.1402	0.0299527	-0.179143	0.0825261	0.67784	17:43962562:A/G	73	MAPT-AS1	ncRNA_intronic	7.231	6	5	14
17:43957121:T:TA	rs138468364	TA	T	0.1233	NA	NA	NA	1	17:43962562:A/G	73	MAPT-AS1	ncRNA_intronic	0.04	NA	14	14
17:43959825:C:T	17:43959825:A/G	T	C	0.1223	2.67822e-05	-0.339357	0.080814	0.990885	17:43962562:A/G	73	MAPT-AS1	ncRNA_intronic	9.543	NA	9	14
17:43962562:C:T	17:43962562:A/G	C	T	0.1233	1.99062e-05	-0.341719	0.0801036	1	17:43962562:A/G	73	MAPT-AS1	ncRNA_intronic	2.697	7	9	14
17:43962575:C:T	17:43962575:A/G	T	C	0.1223	2.7736e-05	-0.342082	0.0816168	0.990885	17:43962562:A/G	73	MAPT-AS1	ncRNA_intronic	3.7	7	9	14
17:43969897:A:C	17:43969897:A/C	C	A	0.1243	2.10289e-05	-0.335074	0.0787727	0.990927	17:43962562:A/G	73	MAPT-AS1	ncRNA_intronic	4.339	7	5	14
17:43972781:A:AG	rs558423866	AG	A	0.1243	NA	NA	NA	0.990927	17:43962562:A/G	73	MAPT-AS1:MAPT	ncRNA_exonic	15.89	NA	1	11
17:43974594:A:G	17:43974594:A/G	A	G	0.1243	2.53333e-05	-0.329286	0.0781816	0.990927	17:43962562:A/G	73	MAPT	intronic	9.6	4	1	13
17:43981686:T:TAA	rs544804290	TAA	T	0.1233	NA	NA	NA	1	17:43962562:A/G	73	MAPT	intronic	0.971	NA	5	14
17:43984081:A:G	17:43984081:A/G	A	G	0.1233	3.2293e-05	-0.332512	0.079995	1	17:43962562:A/G	73	MAPT	intronic	1.558	5	2	14
17:43985723:A:G	17:43985723:A/G	A	G	0.1223	3.24747e-05	-0.336876	0.0810699	0.990885	17:43962562:A/G	73	MAPT:RP11-669E14.4	ncRNA_exonic	7.16	4	2	14
17:43991509:A:C	17:43991509:A/C	C	A	0.1213	1.06225e-05	-0.355991	0.0808317	1	17:43991509:A/C	73	MAPT	intronic	2.933	5	4	14
17:43991571:G:GAGGGTGTGGGCAGCC	rs141801658	G	GAGGGTGTGGGCAGCC	0.1402	NA	NA	NA	0.81264	17:43991509:A/C	73	MAPT	intronic	5.633	NA	4	14
17:43991643:C:G	17:43991643:C/G	C	G	0.1213	1.15252e-05	-0.352356	0.0803296	0.96319	17:43991509:A/C	73	MAPT	intronic	1.534	5	2	14
17:43999203:A:G	17:43999203:A/G	G	A	0.1123	0.000723319	0.337037	0.0996977	0.637053	17:43991509:A/C	73	MAPT	intronic	6.069	5	4	14
17:73863391:C:G	17:73863391:C/G	G	C	0.03082	4.42256e-05	-0.943508	0.231013	0.872959	17:73893155:A/G	74	TRIM47	intergenic	7.045	6	5	5
17:73871866:C:G	17:73871866:C/G	C	G	0.0328	4.21897e-05	-0.952447	0.23258	0.879203	17:73893155:A/G	74	TRIM47	intronic	9.206	4	2	7
17:73893155:A:G	17:73893155:A/G	G	A	0.02883	2.19773e-05	-1.12143	0.264251	1	17:73893155:A/G	74	RP11-552F3.10	ncRNA_exonic	7.697	4	1	1
18:36096440:A:G	rs187315033	A	G	0.000994	NA	NA	NA	1	18:36922580:C/G	75	RP11-687D19.1	intergenic	3.06	7	5	15
18:36674085:A:G	rs572437609	A	G	0.000994	NA	NA	NA	1	18:36922580:C/G	75	RNU6-706P	intergenic	3.835	NA	5	15
18:36878243:C:G	rs548487954	C	G	0.000994	NA	NA	NA	1	18:36922580:C/G	75	LINC00669	ncRNA_intronic	0.856	NA	5	15
18:36887380:C:T	rs191731320	T	C	0.000994	NA	NA	NA	1	18:36922580:C/G	75	LINC00669	ncRNA_intronic	3.847	5	5	15
18:36922580:C:G	18:36922580:C/G	G	C	0.04076	7.83492e-08	-0.74386	0.138498	1	18:36922580:C/G	75	LINC00669	ncRNA_intronic	13.68	6	5	15

18:37045316:A:G	rs551826130	A	G	0.000994	NA	NA	NA	1	18:36922580:C/G	75	LINC00669	ncRNA_intronic	2.127	NA	5	15
18:37074083:C:T	rs186655142	T	C	0.000994	NA	NA	NA	1	18:36922580:C/G	75	LINC00669	ncRNA_intronic	0.035	6	5	15
18:37234725:C:T	rs577997863	T	C	0.000994	NA	NA	NA	1	18:36922580:C/G	75	LINC00669	ncRNA_intronic	4.684	NA	5	15
18:37325780:A:G	rs185165909	G	A	0.000994	NA	NA	NA	1	18:36922580:C/G	75	LINC00669	ncRNA_intronic	0.379	7	5	15
18:37335992:C:CA	rs573030459	C	CA	0.000994	NA	NA	NA	1	18:36922580:C/G	75	LINC00669	ncRNA_intronic	3.328	NA	5	15
18:37381585:G:T	rs185884705	T	G	0.000994	NA	NA	NA	1	18:36922580:C/G	75	LINC00669	intergenic	7.314	4	7	9
18:37637865:A:G	rs568966403	A	G	0.000994	NA	NA	NA	1	18:36922580:C/G	75	RP11-653G8.2	intergenic	3.539	NA	14	15
18:37799564:C:T	rs563699171	T	C	0.000994	NA	NA	NA	1	18:36922580:C/G	75	RPL17P45	intergenic	4.904	NA	5	15
18:54484203:C:T	18:54484203:A/G	T	C	0.06163	3.44005e-05	-0.375702	0.0907013	1	18:54484203:A/G	76	WDR7	intronic	0.844	7	4	15
18:54524708:G:T	18:54524708:A/C	T	G	0.06163	4.66494e-05	-0.363197	0.0891981	0.968184	18:54484203:A/G	76	WDR7	intronic	0.195	7	5	15
18:54532128:A:T	18:54532128:A/T	T	A	0.05865	4.87457e-05	-0.38268	0.0942197	0.920592	18:54484203:A/G	76	WDR7	intronic	5.166	NA	5	15
18:54532135:C:T	18:54532135:A/G	T	C	0.06064	4.91252e-05	-0.368318	0.0907244	0.95225	18:54484203:A/G	76	WDR7	intronic	1.5	NA	5	15
19:5812913:A:G	19:5812913:A/G	G	A	0.007952	3.20454e-06	-0.765272	0.164318	1	19:5812913:A/G	77	AC011499.1	intergenic	2.449	NA	4	15
19:5838660:G:T	19:5838660:A/C	G	T	0.01292	5.50204e-05	0.509685	0.126372	0.609169	19:5812913:A/G	77	FUT6	intronic	0.342	2b	1	15
19:5853552:A:T	19:5853552:A/T	T	A	0.01093	0.00161238	0.498101	0.157944	0.722865	19:5812913:A/G	77	AC024592.9	ncRNA_intronic	1.655	7	5	15
19:5853556:A:G	19:5853556:A/G	G	A	0.01193	0.00161835	0.497973	0.157957	0.661279	19:5812913:A/G	77	AC024592.9	ncRNA_intronic	1.053	6	5	15
19:9205709:A:G	19:9205709:A/G	G	A	0.3509	0.00138474	-0.155906	0.0487539	0.603139	19:9224099:A/G	78	OR1M1	downstream	0.53	6	5	15
19:9206434:A:G	19:9206434:A/G	A	G	0.3509	0.0020637	-0.149934	0.0486662	0.603139	19:9224099:A/G	78	OR1M1	intergenic	1.43	7	5	15
19:9206949:C:T	19:9206949:A/G	T	C	0.3509	0.00147316	-0.154819	0.0486869	0.603139	19:9224099:A/G	78	OR1M1	intergenic	0.785	5	5	15
19:9206963:A:G	19:9206963:A/G	A	G	0.3509	0.00228465	-0.148362	0.0486358	0.603139	19:9224099:A/G	78	OR1M1	intergenic	0.836	5	5	15
19:9207417:A:C	19:9207417:A/C	A	C	0.3509	0.00207226	-0.149776	0.0486337	0.603139	19:9224099:A/G	78	OR1M1	intergenic	0.845	6	9	15
19:9207826:A:AT	rs79894237	AT	A	0.3509	NA	NA	NA	0.603139	19:9224099:A/G	78	OR1M1	intergenic	3.735	NA	9	15
19:9207943:A:G	19:9207943:A/G	A	G	0.3509	0.00204571	-0.149841	0.0485946	0.603139	19:9224099:A/G	78	OR1M1	intergenic	1.598	6	9	15
19:9208186:C:T	19:9208186:A/G	T	C	0.3509	0.00202166	-0.149957	0.0485765	0.603139	19:9224099:A/G	78	OR1M1	intergenic	1.121	5	9	15
19:9208427:C:CG	rs35856746	C	CG	0.3509	NA	NA	NA	0.603139	19:9224099:A/G	78	OR1M1	intergenic	3.022	NA	9	15
19:9208452:C:T	19:9208452:A/G	T	C	0.3509	0.00200657	-0.150019	0.0485617	0.603139	19:9224099:A/G	78	OR1M1	intergenic	1.282	7	9	15
19:9209561:A:T	19:9209561:A/T	A	T	0.3509	0.00262171	-0.145845	0.0484708	0.603139	19:9224099:A/G	78	OR7G2	intergenic	0.933	7	9	15
19:9210433:A:G	19:9210433:A/G	A	G	0.3519	0.00279232	-0.144615	0.0483707	0.604631	19:9224099:A/G	78	OR7G2	intergenic	0.027	6	9	15
19:9212864:A:C	19:9212864:A/C	C	A	0.4732	0.000112505	-0.187566	0.0485679	0.821926	19:9224099:A/G	78	OR7G2	downstream	0.26	7	9	15
19:9217951:A:T	19:9217951:A/T	T	A	0.4732	0.000118803	-0.188656	0.0490198	0.815131	19:9224099:A/G	78	OR7G2	intergenic	1.537	6	9	15
19:9220791:C:T	19:9220791:A/G	T	C	0.3519	0.00312745	-0.145198	0.0491377	0.604631	19:9224099:A/G	78	OR7G1	intergenic	0.95	5	7	15
19:9220910:C:G	19:9220910:C/G	C	G	0.3519	0.00325513	-0.144546	0.049122	0.604631	19:9224099:A/G	78	OR7G1	intergenic	0.725	4	7	15
19:9222876:C:G	19:9222876:C/G	G	C	0.3519	0.00431246	-0.141995	0.0497465	0.604631	19:9224099:A/G	78	OR7G1	intergenic	0.727	7	9	15
19:9223007:A:G	19:9223007:A/G	A	G	0.3708	0.004782	-0.141748	0.0502404	0.665872	19:9224099:A/G	78	OR7G1	intergenic	1.012	6	9	15
19:9223257:C:T	19:9223257:A/G	C	T	0.3907	0.00603494	-0.138578	0.0504672	0.603226	19:9224099:A/G	78	OR7G1	intergenic	0.51	6	9	15
19:9223491:C:G	19:9223491:C/G	G	C	0.4761	0.000181896	-0.192925	0.0515441	0.967767	19:9224099:A/G	78	OR7G1	intergenic	0.782	6	9	15
19:9223787:C:T	19:9223787:A/G	C	T	0.4751	2.75728e-05	-0.216612	0.0516648	0.992838	19:9224099:A/G	78	OR7G1	intergenic	1.012	6	9	15
19:9224099:A:G	19:9224099:A/G	A	G	0.4732	2.09963e-05	-0.222	0.0521863	1	19:9224099:A/G	78	OR7G1	intergenic	0.903	7	9	15
19:9224157:A:G	19:9224157:A/G	G	A	0.3042	1.44264e-06	-0.303596	0.0629992	1	19:9224157:A/G	78	OR7G1	intergenic	1.219	6	9	15
20:6690101:C:T	20:6690101:A/G	T	C	0.325	0.000108669	0.197171	0.0509438	0.834362	20:6693128:A/G	79	RP5-859D4.3	intergenic	1.826	4	1	15
20:6690400:T:TG	rs5840154	T	TG	0.3469	NA	NA	NA	0.930669	20:6693128:A/G	79	RP5-859D4.3	intergenic	3.152	NA	1	15
20:6690502:A:T	20:6690502:A/T	A	T	0.4463	0.00137031	0.153137	0.0478426	0.610099	20:6693128:A/G	79	RP5-859D4.3	intergenic	7.28	4	1	15
20:6692529:A:G	20:6692529:A/G	A	G	0.3519	1.40203e-05	0.222793	0.0512927	0.986771	20:6693128:A/G	79	RP5-859D4.3	intergenic	0.523	NA	5	15

20:6693128:C:T	20:6693128:A/G	T	C	0.3529	9.48773e-06	0.227009	0.0512602	1	20:6693128:A/G	79	RP5-859D4.3	intergenic	9.96	4	5	15
20:6693522:C:G	20:6693522:C/G	G	C	0.3529	1.70963e-05	0.220641	0.0513142	1	20:6693128:A/G	79	RP5-859D4.3	intergenic	10.67	7	5	15
20:6693578:G:T	20:6693578:A/C	G	T	0.4225	6.78887e-05	0.192527	0.0483304	0.744029	20:6693128:A/G	79	RP5-859D4.3	intergenic	2.517	NA	5	15
20:6693590:A:G	20:6693590:A/G	A	G	0.3529	1.11394e-05	0.226848	0.0516294	0.991245	20:6693128:A/G	79	RP5-859D4.3	intergenic	1.376	NA	5	15
20:6693879:C:T	20:6693879:A/G	C	T	0.4235	6.5802e-05	0.19318	0.0484041	0.742161	20:6693128:A/G	79	RP5-859D4.3	intergenic	2.906	NA	5	15
20:6698372:C:G	20:6698372:C/G	C	G	0.3201	0.00012671	0.203733	0.0531557	0.744271	20:6693128:A/G	79	RP5-859D4.3	intergenic	0.514	5	5	15
20:6699595:G:T	20:6699595:A/C	G	T	0.3181	0.000150126	0.201578	0.0531746	0.73875	20:6693128:A/G	79	RP5-859D4.3	intergenic	14.82	3a	2	15
20:6706493:C:T	20:6706493:A/G	T	C	0.3111	0.000214346	0.196981	0.0532167	0.699674	20:6693128:A/G	79	RP5-859D4.3	intergenic	0.961	6	9	15
20:19934733:A:G	20:19934733:A/G	G	A	0.2107	1.91218e-06	0.318699	0.0669188	1	20:19934733:A/G	80	RIN2	intronic	4.142	7	4	5
20:19935337:GCCCCTGTGTCCCTCAC:GCCCTGTGCCCCTCACCCCTGTGTCCCTCAC	rs534003762	GCCCTGTGCCCCTCACCCCTGTGTCCCTCAC	GCCCCTGTGTCCCTCAC	0.2594	NA	NA	NA	1	20:19935365:C/G	80	RIN2	intronic	NA	NA	4	5
20:19935337:G:GCCCTGTGCCCCTCACCCCTGTGTCCCTCAC	rs556718941	GCCCTGTGCCCCTCACCCCTGTGTCCCTCAC	G	0.002982	NA	NA	NA	1	20:19935365:C/G	80	RIN2	intronic	0.889	NA	4	5
20:19935355:C:CT	rs530665384	CT	C	0.2594	NA	NA	NA	1	20:19935365:C/G	80	RIN2	intronic	NA	NA	4	5
20:19935365:C:G	20:19935365:C/G	G	C	0.2594	1.34032e-05	0.253395	0.0582062	1	20:19935365:C/G	80	RIN2	intronic	0.653	6	4	5
20:19935920:C:T	20:19935920:A/G	T	C	0.2058	4.25322e-05	0.249558	0.0609677	0.804195	20:19934733:A/G	80	RIN2	intronic	0.023	7	4	5
20:19936395:A:G	20:19936395:A/G	A	G	0.2078	2.9678e-05	0.247193	0.0591949	0.813882	20:19934733:A/G	80	RIN2	intronic	0.227	6	4	5
20:19938324:A:C	20:19938324:A/C	A	C	0.2575	0.000139067	0.218729	0.057412	0.927222	20:19935365:C/G	80	RIN2	intronic	0.057	6	4	4
20:19938351:A:T	20:19938351:A/T	T	A	0.2575	8.38711e-05	0.224167	0.0569957	0.927222	20:19935365:C/G	80	RIN2	intronic	3.964	7	4	4
20:19938390:C:G	20:19938390:C/G	C	G	0.2058	0.000271831	0.220507	0.0605655	0.779962	20:19934733:A/G	80	RIN2	intronic	1.703	6	4	4
20:19939888:C:T	20:19939888:A/G	T	C	0.1968	0.00168724	0.195821	0.0623557	0.741757	20:19934733:A/G	80	RIN2	intronic	0.767	5	1	4
20:19940022:C:T	20:19940022:A/G	C	T	0.1998	0.00196778	0.191805	0.061971	0.747636	20:19934733:A/G	80	RIN2	intronic	1.531	5	2	4
20:19940220:C:T	20:19940220:A/G	T	C	0.2535	0.000643077	0.196516	0.0575823	0.896704	20:19935365:C/G	80	RIN2	intronic	0.206	6	4	4
20:19940301:A:T	20:19940301:A/T	T	A	0.1909	0.00295369	0.184439	0.062048	0.68859	20:19934733:A/G	80	RIN2	intronic	6.118	6	4	4
20:19941031:A:G	20:19941031:A/G	A	G	0.2525	0.000874241	0.189001	0.0567898	0.79342	20:19935365:C/G	80	RIN2	intronic	0.311	7	4	4
20:19941538:A:T	20:19941538:A/T	T	A	0.2604	0.000733133	0.188816	0.0559141	0.767512	20:19935365:C/G	80	RIN2	intronic	1.844	5	4	4
20:19942200:C:T	20:19942200:A/G	T	C	0.2575	0.00141876	0.177756	0.0557087	0.771202	20:19935365:C/G	80	RIN2	intronic	0.691	5	4	4
20:19944137:C:T	20:19944137:A/G	T	C	0.2227	0.00565662	0.168078	0.060743	0.615371	20:19935365:C/G	80	RIN2	intronic	9.274	6	4	5
20:19944441:C:T	20:19944441:A/G	C	T	0.2296	0.00284222	0.175443	0.058788	0.618507	20:19935365:C/G	80	RIN2	intronic	3.54	5	4	5
20:19946053:A:C	20:19946053:A/C	C	A	0.2316	0.000693499	0.197479	0.0582154	0.626289	20:19935365:C/G	80	RIN2	intronic	4.457	5	4	15
20:19946056:C:T	20:19946056:A/G	C	T	0.2306	0.000680823	0.197809	0.058226	0.622386	20:19935365:C/G	80	RIN2	intronic	0.341	5	4	15
20:62486721:A:G	20:62486721:A/G	A	G	0.04274	1.07164e-05	-0.397293	0.0902494	1	20:62486721:A/G	81	ABHD16B	intergenic	0.283	4	1	7
20:62486735:A:G	20:62486735:A/G	G	A	0.04274	1.07132e-05	-0.397282	0.0902451	1	20:62486721:A/G	81	ABHD16B	intergenic	3.565	2b	1	7
21:22920834:C:T	21:22920834:A/G	T	C	0.01988	0.00147285	-0.831019	0.261328	0.751578	21:23023268:A/G	82	NCAM2	intergenic	1.787	6	9	15
21:22930744:C:T	rs1460086	C	T	0.02187	NA	NA	NA	0.766405	21:23023268:A/G	82	AF241725.4	intergenic	1.19	NA	9	15
21:22980288:A:G	21:22980288:A/G	G	A	0.01889	4.29175e-05	-1.00729	0.24621	1	21:23023268:A/G	82	AF241725.4	ncRNA_intronic	0.155	6	5	15
21:23002388:A:C	21:23002388:A/C	C	A	0.01889	2.89378e-05	-1.02324	0.244696	1	21:23023268:A/G	82	AF241725.1	intergenic	0.375	6	9	15
21:23023268:A:G	21:23023268:A/G	G	A	0.01889	2.09546e-05	-1.0224	0.240313	1	21:23023268:A/G	82	AF241725.6	intergenic	1.327	7	9	15
21:23072041:C:T	21:23072041:A/G	T	C	0.01988	0.000672289	-0.698087	0.205281	0.948037	21:23023268:A/G	82	AF241725.6	intergenic	0.07	6	9	15

21:25199568:C:T	21:25199568:A/G	T	C	0.00497	9.52755e-06	-1.41336	0.319214	1	21:25199568:A/G	83	AP000474.1	intergenic	1.64	7	9	15
21:25224202:A:G	21:25224202:A/G	G	A	0.005964	0.000121353	-1.31868	0.343105	0.83166	21:25199568:A/G	83	AP000474.1	intergenic	1.319	NA	9	15
21:25231862:A:G	21:25231862:A/G	A	G	0.005964	0.000137615	-1.31541	0.345034	0.83166	21:25199568:A/G	83	AP000474.1	intergenic	1.826	6	5	15
21:25238521:C:G	21:25238521:C/G	G	C	0.005964	0.00013763	-1.32045	0.346358	0.83166	21:25199568:A/G	83	AP000474.1	intergenic	0.855	7	5	15
21:43940264:A:G	21:43940264:A/G	A	G	0.07555	0.0171121	-0.249434	0.104615	0.601815	21:43948272:A/G	84	SLC37A1	intronic	3.183	5	4	5
21:43942696:A:T	21:43942696:A/T	A	T	0.07853	0.016239	-0.251411	0.104602	0.626141	21:43948272:A/G	84	SLC37A1	intronic	2.222	5	4	4
21:43943120:A:G	21:43943120:A/G	A	G	0.07952	0.00860666	-0.268362	0.102144	0.616328	21:43948272:A/G	84	SLC37A1	intronic	0.076	5	2	5
21:43944210:C:T	21:43944210:A/G	T	C	0.07952	0.00839515	-0.269167	0.102122	0.616328	21:43948272:A/G	84	SLC37A1	intronic	2.885	4	2	7
21:43945200:C:T	21:43945200:A/G	T	C	0.07952	0.00609879	-0.280147	0.102154	0.616328	21:43948272:A/G	84	SLC37A1	intronic	0.263	4	1	2
21:43947627:A:G	21:43947627:A/G	A	G	0.1173	4.10723e-05	-0.337795	0.0823619	1	21:43948272:A/G	84	SLC37A1	intronic	3.365	2b	1	5
21:43948162:A:G	21:43948162:A/G	A	G	0.1183	5.44963e-05	-0.329952	0.0817634	0.990664	21:43948272:A/G	84	SLC37A1	intronic	3.259	2b	1	5
21:43948210:G:T	21:43948210:A/C	T	G	0.1173	7.48032e-05	-0.322757	0.0814948	1	21:43948272:A/G	84	SLC37A1	intronic	5.787	4	1	5
21:43948272:A:G	21:43948272:A/G	G	A	0.1173	3.74244e-05	-0.338362	0.0820707	1	21:43948272:A/G	84	SLC37A1	intronic	3.677	4	1	5
21:43948864:A:G	21:43948864:A/G	A	G	0.09145	0.000224043	-0.338833	0.0918181	0.732026	21:43948272:A/G	84	SLC37A1	intronic	0.02	7	4	5
21:43950035:C:T	21:43950035:A/G	T	C	0.09344	0.000564487	-0.315264	0.0914304	0.713061	21:43948272:A/G	84	SLC37A1	intronic	0.956	4	2	5
21:43951834:G:GCATTG TCT	rs113952001	G	GCATTGTCT	0.08847	NA	NA	NA	0.724674	21:43948272:A/G	84	SLC37A1	intronic	0.588	NA	2	5
21:43952365:C:T	21:43952365:A/G	T	C	0.08847	0.000204053	-0.343365	0.092452	0.724674	21:43948272:A/G	84	SLC37A1	intronic	0.454	5	2	5
21:43953225:G:T	21:43953225:A/C	T	G	0.08847	0.000313034	-0.330893	0.0918055	0.724674	21:43948272:A/G	84	SLC37A1	intronic	2.868	2a	2	5
21:43958015:C:G	21:43958015:C/G	G	C	0.08847	0.000183801	-0.345296	0.0923178	0.724674	21:43948272:A/G	84	SLC37A1	intronic	0.042	5	4	5
21:43964037:C:T	21:43964037:A/G	T	C	0.08847	0.000174488	-0.346549	0.0923309	0.724674	21:43948272:A/G	84	SLC37A1	intronic	7.731	7	4	4
21:43965738:A:G	21:43965738:A/G	A	G	0.08946	0.000187193	-0.344195	0.0921367	0.732952	21:43948272:A/G	84	SLC37A1	intronic	1.047	3a	4	4
21:43967084:C:T	21:43967084:A/G	C	T	0.08946	0.00016848	-0.347174	0.0922814	0.732952	21:43948272:A/G	84	SLC37A1	intronic	1.008	4	2	4
21:43968330:A:G	21:43968330:A/G	A	G	0.08946	0.000167159	-0.347338	0.0922771	0.732952	21:43948272:A/G	84	SLC37A1	intronic	0.674	5	4	4
21:43969226:A:G	21:43969226:A/G	A	G	0.09245	0.000303477	-0.32955	0.0912295	0.703696	21:43948272:A/G	84	SLC37A1	intronic	1.421	5	4	4
21:43969284:C:T	21:43969284:A/G	T	C	0.08946	0.000157486	-0.348576	0.0922413	0.732952	21:43948272:A/G	84	SLC37A1	intronic	0.311	5	4	4
21:43970731:A:AAAG	rs144314112	A	AAAG	0.09245	NA	NA	NA	0.703696	21:43948272:A/G	84	SLC37A1	intronic	0.821	NA	4	5
21:43970855:C:T	21:43970855:A/G	C	T	0.09245	0.000187496	-0.339117	0.0907869	0.703696	21:43948272:A/G	84	SLC37A1	intronic	4.053	5	4	5
21:43971002:T:TCAA	rs139004490	TCAA	T	0.09245	NA	NA	NA	0.703696	21:43948272:A/G	84	SLC37A1	intronic	2.541	NA	4	5
21:43971310:C:T	21:43971310:A/G	C	T	0.09245	0.000180753	-0.339928	0.0907798	0.703696	21:43948272:A/G	84	SLC37A1	intronic	10.3	2b	4	5
21:43971913:T:TTTTTGT TTTGTTTTG	rs374568400	TTTTTGTTTT GTTTTG	T	0.08946	NA	NA	NA	0.732952	21:43948272:A/G	84	SLC37A1	intronic	0.737	NA	4	5
21:43972926:A:G	21:43972926:A/G	A	G	0.08946	0.000136883	-0.351368	0.0921324	0.732952	21:43948272:A/G	84	SLC37A1	intronic	1.598	6	4	5
21:43973445:C:T	21:43973445:A/G	C	T	0.09245	0.000112807	-0.35072	0.09083	0.703696	21:43948272:A/G	84	SLC37A1	intronic	4.807	1f	4	5
21:43973721:A:G	21:43973721:A/G	G	A	0.09543	0.000345907	-0.330823	0.0924537	0.693474	21:43948272:A/G	84	SLC37A1	intronic	0.711	4	4	5
21:43973839:A:T	21:43973839:A/T	A	T	0.08946	9.17116e-05	-0.360642	0.0921998	0.714717	21:43948272:A/G	84	SLC37A1	intronic	5.205	4	4	5
21:43973987:C:T	21:43973987:A/G	T	C	0.09344	0.000232776	-0.334061	0.0907648	0.676849	21:43948272:A/G	84	SLC37A1	intronic	6.427	7	4	5
22:18716159:G:T	rs540680916	T	G	0.000994	NA	NA	NA	1	22:19577517:C/G	85	AC008132.1	intergenic	4.175	NA	15	15
22:18984669:C:T	rs144183227	C	T	0.000994	NA	NA	NA	1	22:19577517:C/G	85	DGCR5:AC007326.9	ncRNA_exonic	1.297	2b	5	14
22:19018570:A:G	rs572695740	G	A	0.000994	NA	NA	NA	1	22:19577517:C/G	85	DGCR5	ncRNA_exonic	3.607	NA	5	13
22:19020569:A:G	rs187542467	G	A	0.000994	NA	NA	NA	1	22:19577517:C/G	85	CA15P1	ncRNA_exonic	3.973	4	4	14
22:19053914:C:G	rs139296413	G	C	0.000994	NA	NA	NA	1	22:19577517:C/G	85	DGCR2	intronic	2.853	6	4	4

22:19117163:C:G	22:19117163:C:G	C	G	0.000994	NA	NA	NA	1	22:19577517:C/G	85	DGCR14	downstream	1.139	NA	5	5
22:19131568:A:T	rs146554670	A	T	0.000994	NA	NA	NA	1	22:19577517:C/G	85	DGCR14	intronic	9.984	4	1	2
22:19133721:C:T	rs182065390	C	T	0.000994	NA	NA	NA	1	22:19577517:C/G	85	DGCR14	intergenic	7.691	3a	5	14
22:19144769:C:T	rs182287460	C	T	0.000994	NA	NA	NA	1	22:19577517:C/G	85	GSC2	intergenic	0.882	5	5	14
22:19152553:C:G	rs528077361	G	C	0.000994	NA	NA	NA	1	22:19577517:C/G	85	AC004463.6	intergenic	2.437	NA	5	14
22:19172242:A:G	rs552194897	G	A	0.000994	NA	NA	NA	1	22:19577517:C/G	85	CLTCL1	intronic	0.714	NA	2	5
22:19185601:C:T	rs180686054	C	T	0.000994	NA	NA	NA	1	22:19577517:C/G	85	CLTCL1	intronic	2.278	4	4	5
22:19188245:C:G	rs191855402	G	C	0.000994	NA	NA	NA	1	22:19577517:C/G	85	CLTCL1	intronic	0.044	6	4	5
22:19373534:A:G	rs148476306	G	A	0.000994	NA	NA	NA	1	22:19577517:C/G	85	HIRA:C22orf39	intronic	5.087	4	4	4
22:19532615:C:T	rs528702620	C	T	0.000994	NA	NA	NA	1	22:19577517:C/G	85	CLDN5	intergenic	0.533	NA	5	15
22:19572199:C:T	22:19572199:A/G	T	C	0.000994	3.18975e-06	1.06803	0.229278	1	22:19577517:C/G	85	AC000077.2	intergenic	0.16	6	5	15
22:19573811:A:AG	rs145105869	AG	A	0.000994	NA	NA	NA	1	22:19577517:C/G	85	AC000077.2	intergenic	0.205	NA	4	15
22:19573972:A:G	22:19573972:A/G	A	G	0.000994	1.559e-06	1.08604	0.226093	1	22:19577517:C/G	85	AC000077.2	intergenic	2.455	5	4	15
22:19574076:A:G	22:19574076:A/G	A	G	0.000994	1.56348e-06	1.08569	0.226046	1	22:19577517:C/G	85	AC000077.2	intergenic	2.389	7	4	15
22:19575375:C:T	rs146650338	C	T	0.000994	NA	NA	NA	1	22:19577517:C/G	85	AC000077.2	intergenic	0.878	5	4	15
22:19575868:C:T	22:19575868:A/G	C	T	0.000994	1.71422e-06	1.07813	0.225338	1	22:19577517:C/G	85	AC000077.2	intergenic	3.258	5	5	15
22:19576376:A:G	22:19576376:A/G	A	G	0.000994	1.06512e-06	1.10426	0.22632	1	22:19577517:C/G	85	AC000077.2	intergenic	0.386	7	5	15
22:19576380:C:T	22:19576380:A/G	C	T	0.000994	1.0651e-06	1.10426	0.22632	1	22:19577517:C/G	85	AC000077.2	intergenic	0.623	7	5	15
22:19576382:C:T	22:19576382:A/G	C	T	0.000994	1.06435e-06	1.10429	0.226318	1	22:19577517:C/G	85	AC000077.2	intergenic	0.058	7	5	15
22:19576387:C:T	22:19576387:A/G	T	C	0.000994	1.06364e-06	1.10432	0.226319	1	22:19577517:C/G	85	AC000077.2	intergenic	0.011	NA	5	15
22:19576388:C:T	22:19576388:A/G	C	T	0.000994	1.06431e-06	1.10428	0.226318	1	22:19577517:C/G	85	AC000077.2	intergenic	0.021	7	5	15
22:19577069:A:T	rs562001941	A	T	0.000994	NA	NA	NA	1	22:19577517:C/G	85	AC000077.2	intergenic	7.06	NA	5	15
22:19577517:C:G	22:19577517:C/G	G	C	0.000994	1.04826e-06	1.09447	0.224169	1	22:19577517:C/G	85	AC000077.2	intergenic	0.094	6	5	15
22:19643338:A:G	rs149531482	G	A	0.000994	NA	NA	NA	1	22:19577517:C/G	85	AC000067.1	intergenic	10.99	5	5	15
22:19862277:C:T	rs372625142	C	T	0.000994	NA	NA	NA	1	22:19577517:C/G	85	TXNRD2	downstream	11.29	4	2	7
22:19888388:A:G	rs528444983	G	A	0.000994	NA	NA	NA	1	22:19577517:C/G	85	TXNRD2	intronic	7.763	NA	4	5
22:19954825:A:G	rs559908437	G	A	0.000994	NA	NA	NA	1	22:19577517:C/G	85	COMT	exonic	0.997	NA	4	4
22:20264219:A:G	rs372085049	G	A	0.000994	NA	NA	NA	1	22:19577517:C/G	85	RTN4R	intergenic	1.784	5	5	15
22:20323953:T:TA	rs544240222	TA	T	0.000994	NA	NA	NA	1	22:19577517:C/G	85	XXbac-B33L19.3	intergenic	1.032	NA	5	15
22:32844697:A:C	22:32844697:A/C	A	C	0.1879	7.53966e-05	0.358986	0.0906856	0.993992	22:32849712:A/C	86	BPIFC	intronic	0.006	6	13	14
22:32846594:A:T	22:32846594:A/T	A	T	0.1869	7.29368e-05	0.360449	0.0908736	1	22:32849712:A/C	86	BPIFC	intronic	2.187	6	14	14
22:32849054:A:G	22:32849054:A/G	A	G	0.1869	7.27124e-05	0.360012	0.0907463	1	22:32849712:A/C	86	BPIFC	intronic	0.124	7	9	14
22:32849712:A:C	22:32849712:A/C	A	C	0.1869	4.06314e-05	0.369106	0.0899412	1	22:32849712:A/C	86	BPIFC	intronic	4.097	5	9	14
22:32849914:A:C	22:32849914:A/C	A	C	0.1869	4.06428e-05	0.369104	0.0899421	1	22:32849712:A/C	86	BPIFC	intronic	0.726	6	9	14
22:32849931:A:AC	rs35118395	A	AC	0.1879	NA	NA	NA	0.993992	22:32849712:A/C	86	BPIFC	intronic	0.498	NA	9	14
22:32850349:A:G	22:32850349:A/G	A	G	0.1869	5.30101e-05	0.366519	0.0906788	1	22:32849712:A/C	86	BPIFC	intronic	6.033	7	13	14
22:32851633:A:G	22:32851633:A/G	A	G	0.1879	7.51035e-05	0.359548	0.0908063	0.993992	22:32849712:A/C	86	BPIFC	intronic	2.537	5	13	14
22:32854391:A:C	22:32854391:A/C	C	A	0.1859	4.78174e-05	0.366681	0.0901813	0.993983	22:32849712:A/C	86	BPIFC	intronic	2.439	7	13	14
22:32855486:C:T	22:32855486:A/G	C	T	0.1869	4.45602e-05	0.368042	0.090152	1	22:32849712:A/C	86	BPIFC	intronic	5.892	6	13	14
22:32855598:C:T	22:32855598:A/G	T	C	0.1869	5.39793e-05	0.366281	0.0907153	1	22:32849712:A/C	86	BPIFC	intronic	0.307	7	13	14
22:36132770:A:T	22:36132770:A/T	T	A	0.04672	8.77288e-06	0.432148	0.0972124	1	22:36132770:A/T	87	RBFOX2	intergenic	6.197	5	4	5
22:43165729:A:G	22:43165729:A/G	G	A	0.07654	0.000123225	-0.390674	0.101748	0.879715	22:43181587:A/G	88	RPL5P34	intergenic	1.349	4	1	15
22:43169578:C:T	22:43169578:A/G	C	T	0.07654	6.1921e-05	-0.405273	0.101182	0.905874	22:43181587:A/G	88	RPL5P34	intergenic	2.947	5	2	15

22:43176847:C:T	22:43176847:A/G	C	T	0.07555	2.19872e-05	-0.423754	0.0998549	0.91905	22:43181587:A/G	88	RPL5P34	intergenic	1.642	7	5	15
22:43177959:G:GTC	rs142991510	G	GTC	0.07555	NA	NA	NA	0.91905	22:43181587:A/G	88	GOLGA2P4	intergenic	1.173	NA	5	15
22:43179166:G:T	22:43179166:A/C	T	G	0.07555	4.5491e-05	-0.41005	0.10056	0.91905	22:43181587:A/G	88	GOLGA2P4	intergenic	2.83	7	5	15
22:43179681:A:G	22:43179681:A/G	G	A	0.07455	7.70915e-05	-0.429237	0.108578	0.905745	22:43181587:A/G	88	GOLGA2P4	upstream	4.703	7	5	15
22:43181587:C:T	22:43181587:A/G	T	C	0.07952	3.19575e-05	0.420848	0.101188	1	22:43181587:A/G	88	GOLGA2P4	ncRNA_intronic	4.768	5	5	15
22:43206167:C:G	22:43206167:C/G	G	C	0.05567	0.000210888	-0.412667	0.111363	0.661543	22:43181587:A/G	88	ARFGAP3	intronic	2.664	7	4	5
22:50945256:A:G	22:50945256:A/G	G	A	0.4185	4.60607e-06	0.256936	0.0560752	1	22:50945256:A/G	89	LMF2	exonic	6.326	NA	1	2
22:50950076:A:AT	rs131821	A	AT	0.3429	NA	NA	NA	0.666954	22:50945256:A/G	89	NCAPH2	intronic	0.819	NA	4	5
22:50957462:C:T	22:50957462:A/G	C	T	0.2505	5.79245e-05	0.236343	0.0587755	0.825311	22:50974100:A/G	89	NCAPH2	intronic	3.925	NA	4	4
22:50957520:A:G	22:50957520:A/G	A	G	0.2366	2.51943e-05	-0.251919	0.0597951	0.891643	22:50974100:A/G	89	NCAPH2	intronic	0.143	NA	4	4
22:50961169:A:G	22:50961169:A/G	G	A	0.2276	8.09635e-06	-0.266499	0.0597183	0.938232	22:50974100:A/G	89	NCAPH2	intronic	7.007	NA	3	4
22:50964153:C:T	22:50964153:A/G	C	T	0.2247	2.59691e-05	-0.252225	0.059965	0.943215	22:50974100:A/G	89	SCO2	intronic	7.737	NA	1	1
22:50971306:C:CA	rs131799	CA	C	0.2256	NA	NA	NA	0.960163	22:50974100:A/G	89	ODF3B	upstream	10.01	NA	1	13
22:50971509:G:T	22:50971509:A/C	G	T	0.2207	6.93362e-06	-0.261225	0.0581054	0.988568	22:50974100:A/G	89	ODF3B	upstream	8.195	NA	2	13
22:50971631:T:TAAAAA	rs131797	TAAAAA	T	0.2406	NA	NA	NA	0.885696	22:50974100:A/G	89	ODF3B	upstream	1.435	NA	2	13
22:50971639:G:GA	rs131796	GA	G	0.2406	NA	NA	NA	0.885696	22:50974100:A/G	89	ODF3B	upstream	1.615	NA	2	13
22:50971706:A:T	22:50971706:A/T	T	A	0.2207	6.71032e-06	-0.261573	0.0580927	0.988568	22:50974100:A/G	89	ODF3B	upstream	0.462	2b	2	13
22:50971752:A:C	22:50971752:A/C	C	A	0.2207	6.42773e-06	-0.262108	0.0580939	0.988568	22:50974100:A/G	89	ODF3B	upstream	3.048	NA	2	13
22:50971795:A:G	22:50971795:A/G	A	G	0.2207	6.44504e-06	-0.262142	0.0581087	0.988568	22:50974100:A/G	89	ODF3B	upstream	3.153	NA	2	13
22:50974100:A:G	22:50974100:A/G	G	A	0.2227	3.06206e-06	-0.274355	0.0587909	1	22:50974100:A/G	89	ODF3B	intergenic	1.911	7	5	14
22:50977738:C:T	22:50977738:A/G	C	T	0.3042	0.000320269	-0.188494	0.0523841	0.60098	22:50974100:A/G	89	CTA-384D8.35	intergenic	5.239	NA	1	2
22:50978024:A:C	22:50978024:A/C	A	C	0.2962	0.000146228	-0.205432	0.0540982	0.625932	22:50974100:A/G	89	CTA-384D8.35	intergenic	5.699	NA	1	2
22:50980071:A:G	22:50980071:A/G	A	G	0.2932	0.000119032	-0.210105	0.0545994	0.610895	22:50974100:A/G	89	CTA-384D8.35	downstream	5.792	NA	1	14
22:50985703:A:G	22:50985703:A/G	G	A	0.2714	0.0021617	-0.171662	0.0559696	0.630859	22:51004421:A/G	89	CTA-384D8.31	ncRNA_intronic	6.952	4	2	13
22:50992232:A:G	22:50992232:A/G	G	A	0.2763	0.00112034	-0.180517	0.0554004	0.713537	22:51004421:A/G	89	SYCE3	intronic	4.197	7	5	14
22:50993420:A:G	22:50993420:A/G	A	G	0.2753	0.000785296	-0.186244	0.0554644	0.715616	22:51004421:A/G	89	SYCE3	intronic	0.797	7	5	14
22:50993807:C:T	22:50993807:A/G	C	T	0.2763	0.000911157	-0.184087	0.0555047	0.713537	22:51004421:A/G	89	SYCE3	intronic	9.335	NA	2	15
22:50996681:A:C	22:50996681:A/C	C	A	0.2753	0.000921878	-0.184041	0.0555454	0.715616	22:51004421:A/G	89	SYCE3	intronic	2.843	NA	5	15
22:50999182:C:T	22:50999182:A/G	T	C	0.3062	0.004034	-0.154023	0.053564	0.643067	22:51004421:A/G	89	SYCE3	intronic	5.357	NA	5	15
22:51001394:G:T	22:51001394:A/C	G	T	0.2316	8.13804e-05	-0.23804	0.0604119	1	22:51004421:A/G	89	SYCE3	upstream	13.69	NA	1	1
22:51001838:C:T	22:51001838:A/G	T	C	0.2316	5.27995e-05	-0.243331	0.0601877	1	22:51004421:A/G	89	SYCE3	upstream	8.426	NA	1	2
22:51003836:A:G	22:51003836:A/G	G	A	0.2316	4.82887e-05	-0.244786	0.0602366	1	22:51004421:A/G	89	SYCE3	intergenic	3.949	NA	5	5
22:51004053:C:T	22:51004053:A/G	C	T	0.2316	4.91183e-05	-0.244503	0.0602257	1	22:51004421:A/G	89	SYCE3	intergenic	4.51	NA	5	5
22:51004348:C:T	22:51004348:A/G	C	T	0.2316	4.69031e-05	-0.245219	0.0602424	1	22:51004421:A/G	89	CPT1B	intergenic	2.724	NA	5	5
22:51004421:A:G	22:51004421:A/G	A	G	0.2316	4.58887e-05	-0.245589	0.0602579	1	22:51004421:A/G	89	CPT1B	intergenic	2.064	7	5	5

Supplementary table S6. MAGMA gene-based test (The gene-based *p*-value was computed by mapping input SNPs for 19,351 protein-coding genes. Genome-wide significance was set at $p = 0.05/19{,}351 = 2.58\times10^{-6}$ and those meeting this criterion are shown below.)

GENE	CHR	START	STOP	NSNPS	NPARAM	N	ZSTAT	*P*	SYMBOL
ENSG00000160886	8	143751529	143816545	237	16	3686	6.5482	2.9118e-11	LY6K
ENSG00000197353	8	143801568	143863952	220	25	3686	6.4659	5.0365e-11	LYPD2
ENSG00000126233	8	143792362	143853829	204	21	3686	6.4253	6.5799e-11	SLURP1
ENSG00000180155	8	143815752	143889640	324	22	3686	6.3787	8.9297e-11	LYNX1
ENSG00000130193	8	143778621	143848345	241	21	3686	6.3106	1.3897e-10	THEM6
ENSG00000173171	1	155148490	155213615	108	16	3686	5.9107	1.7033e-09	MTX1
ENSG00000167653	8	143721726	143794142	353	19	3686	5.8829	2.0159e-09	PSCA
ENSG00000169231	1	155135379	155208842	125	18	3686	5.8568	2.3598e-09	THBS3
ENSG00000185499	1	155128300	155192707	115	18	3686	5.7852	3.6214e-09	MUC1
ENSG00000167656	8	143836296	143898008	319	22	3686	5.782	3.6898e-09	LY6D
ENSG00000163462	1	155115873	155187447	135	18	3686	5.5074	1.8212e-08	TRIM46
ENSG00000273088	1	155111885	155189748	144	19	3686	5.4492	2.5297e-08	RP11-201K10.3
ENSG00000163463	1	155111884	155175951	121	16	3686	5.1303	1.4462e-07	KRTCAP2

Supplementary table S7. Genes implicated by positional, eQTL, or chromatin interaction mapping of SNPs.
pLI = probability of loss of function mutation intolerance (the higher the score, the greater the probability); posMapSNPs = SNPs mapped to this gene by positional mapping; eqtlMap = SNPs mapped to this gene by expression quantitative traic locus mapping; eqtlMapminP = minimum p-value of eQTL association for the mapped gene; eqtlMapminQ = minimum false discovery rate Q value of eQTL association for the mapped gene; eqtlDirection = the direction in which eQTL SNPs are associated with expression levels (upregulation or downregulation); ciMap = chromatin interaction mapping; minGwasP = minimum GWAS *p*-value of SNP(s) implicating the gene; IndSigSNPs = GWAS SNPs with significant *p*-values from which genes were mapped.

ensg	symbol	start	end	strand	type	entrezID	HUGO	pLI	ncRVIS	posMapSNPs	posMapMaxCAD	eqtlMapSNPs	eqtlMapminP	eqtlMapminQ	eqtlMapts	eqtlDirection	ciMap	ciMapts	minGwasP	IndSigSNPs	GenomicLocus
ENSG00000070759	TESK2	45809555	45956872	-1	protein_coding	10420	TESK2	8.65426854901905e-06	0.629949731	0	0	0	NA	NA	NA	NA	Yes	GSE86189_GA.po.all.FUMA	5.24107e-05	1:47924371:A/G;1:47935863:A/T;1:47942422:A/G	3
ENSG00000236624	CCDC163P	45959538	45965751	-1	protein_coding	126661	CCDC163P	0.143398568804261	NA	0	0	0	NA	NA	NA	NA	Yes	GSE86189_GA.po.all.FUMA	5.24107e-05	1:47924371:A/G;1:47935863:A/T;1:47942422:A/G	3
ENSG00000132763	MMACHC	45965725	45976739	1	protein_coding	25974	MMACHC	5.25633027461793e-14	0.553694665	0	0	0	NA	NA	NA	NA	Yes	GSE86189_GA.po.all.FUMA	5.24107e-05	1:47924371:A/G;1:47935863:A/T;1:47942422:A/G	3
ENSG00000117450	PRDX1	45976708	45988719	-1	protein_coding	5052	PRDX1	1.43818422373501e-07	-0.573941146	0	0	0	NA	NA	NA	NA	Yes	GSE86189_GA.po.all.FUMA	5.24107e-05	1:47924371:A/G;1:47935863:A/T;1:47942422:A/G	3
ENSG00000234379	HMGB1P48	45996599	45997811	1	pseudogene	100128639	HMGB1P48	NA	NA	0	0	0	NA	NA	NA	NA	Yes	GSE86189_GA.po.all.FUMA	5.24107e-05	1:47924371:A/G;1:47935863:A/T;1:47942422:A/G	3
ENSG00000117448	AKR1A1	46016215	46035721	1	protein_coding	10327	AKR1A1	0.000107486413477255	-0.636567263	0	0	0	NA	NA	NA	NA	Yes	GSE86189_GA.po.all.FUMA	5.24107e-05	1:47924371:A/G;1:47935863:A/T;1:47942422:A/G	3
ENSG00000132780	NASP	46049518	46084566	1	protein_coding	4678	NASP	0.470216299614197	-0.593389587	0	0	0	NA	NA	NA	NA	Yes	GSE86189_GA.po.all.FUMA	5.24107e-05	1:47924371:A/G;1:47935863:A/T;1:47942422:A/G	3
ENSG00000159588	CCDC17	46085716	46089729	-1	protein_coding	149483	CCDC17	3.58916148308353e-13	0.307685475	0	0	0	NA	NA	NA	NA	Yes	GSE86189_GA.po.all.FUMA	5.24107e-05	1:47924371:A/G;1:47935863:A/T;1:47942422:A/G	3
ENSG00000159592	GPBP1L1	46092976	46153785	-1	protein_coding	60313	GPBP1L1	0.435324050435863	-0.428470523	0	0	0	NA	NA	NA	NA	Yes	GSE86189_GA.po.all.FUMA	5.24107e-05	1:47924371:A/G;1:47935863:A/T;1:47942422:A/G	3
ENSG00000159596	TMEM69	46152886	46160115	1	protein_coding	51249	TMEM69	0.000261682873521805	-1.029265497	0	0	0	NA	NA	NA	NA	Yes	GSE86189_GA.po.all.FUMA	5.24107e-05	1:47924371:A/G;1:47935863:A/T;1:47942422:A/G	3
ENSG00000197429	IPP	46159996	46216322	-1	protein_coding	3652	IPP	0.00253170996710155	-0.243672916	0	0	0	NA	NA	NA	NA	Yes	GSE86189_GA.po.all.FUMA	5.24107e-05	1:47924371:A/G;1:47935863:A/T;1:47942422:A/G	3
ENSG00000086015	MAST2	46252659	46501796	1	protein_coding	23139	MAST2	5.44402882251562e-11	NA	0	0	0	NA	NA	NA	NA	Yes	GSE86189_GA.po.all.FUMA	5.24107e-05	1:47924371:A/G;1:47935863:A/T;1:47942422:A/G	3
ENSG00000117461	PIK3R3	46505812	46642160	-1	protein_coding	8503	PIK3R3	1.40585793273596e-06	-1.620545781	0	0	0	NA	NA	NA	NA	Yes	GSE86189_GA.po.all.FUMA	5.24107e-05	1:47924371:A/G;1:47935863:A/T;1:47942422:A/G	3
ENSG00000117472	TSPAN1	46640745	46651630	1	protein_coding	10103	TSPAN1	0.000714382124602965	-0.51072909	0	0	0	NA	NA	NA	NA	Yes	GSE86189_GA.po.all.FUMA	5.24107e-05	1:47924371:A/G;1:47935863:A/T;1:47942422:A/G	3

ENSG00000250719	RP11-322N21.2	46641158	46642150	-1	antisense	110117498	LOC110117498	NA	NA	0	0	0	NA	NA	NA	NA	Yes	GSE86189_GA.po.all.FUMA	5.24107e-05	1:47924371:A/G;1:47935863:A/T;1:47942422:A/G	3
ENSG00000085998	POMGNT1	46654354	46685977	-1	protein_coding	55624	POMGNT1	1.68023697303463e-09	NA	0	0	0	NA	NA	NA	NA	Yes	GSE86189_GA.po.all.FUMA	5.24107e-05	1:47924371:A/G;1:47935863:A/T;1:47942422:A/G	3
ENSG00000171357	LURAP1	46669006	46686933	1	protein_coding	541468	LURAP1	0.220934118593464	-0.892110208	0	0	0	NA	NA	NA	NA	Yes	GSE86189_GA.po.all.FUMA	5.24107e-05	1:47924371:A/G;1:47935863:A/T;1:47942422:A/G	3
ENSG00000085999	RAD54L	46713360	46744145	1	protein_coding	8438	RAD54L	2.73718135268854e-13	-0.60342968	0	0	0	NA	NA	NA	NA	Yes	GSE86189_GA.po.all.FUMA	5.24107e-05	1:47924371:A/G;1:47935863:A/T;1:47942422:A/G	3
ENSG00000132128	LRRC41	46726868	46769280	-1	protein_coding	10489	LRRC41	0.999804640576002	-0.769306483	0	0	0	NA	NA	NA	NA	Yes	GSE86189_GA.po.all.FUMA	5.24107e-05	1:47924371:A/G;1:47935863:A/T;1:47942422:A/G	3
ENSG00000173660	UQCRH	46769303	46782448	1	protein_coding	7388	UQCRH	0.218440721378243	-0.452702747	0	0	0	NA	NA	NA	NA	Yes	GSE86189_GA.po.all.FUMA	5.24107e-05	1:47924371:A/G;1:47935863:A/T;1:47942422:A/G	3
ENSG00000117481	NSUN4	46805849	46830824	1	protein_coding	387338	NSUN4	1.38867585570257e-06	0.106940615	0	0	0	NA	NA	NA	NA	Yes	GSE86189_GA.po.all.FUMA	5.24107e-05	1:47924371:A/G;1:47935863:A/T;1:47942422:A/G	3
ENSG00000117480	FAAH	46859937	46879520	1	protein_coding	2166	FAAH	5.9295002678292e-07	NA	0	0	0	NA	NA	NA	NA	Yes	GSE86189_GA.po.all.FUMA	5.24107e-05	1:47924371:A/G;1:47935863:A/T;1:47942422:A/G	3
ENSG00000197587	DMBX1	46972668	46979898	1	protein_coding	127343	DMBX1	0.915995646935682	0.936273869	0	0	0	NA	NA	NA	NA	Yes	GSE86189_GA.po.all.FUMA	5.24107e-05	1:47924371:A/G;1:47935863:A/T;1:47942422:A/G	3
ENSG00000162456	KNCN	47011316	47017199	-1	protein_coding	148930	KNCN	0.0295306711881479	0.225446274	0	0	0	NA	NA	NA	NA	Yes	GSE86189_GA.po.all.FUMA	5.24107e-05	1:47924371:A/G;1:47935863:A/T;1:47942422:A/G	3
ENSG00000079277	MKNK1	47023090	47082515	-1	protein_coding	8569	MKNK1	0.000222399042685413	-0.663848179	0	0	0	NA	NA	NA	NA	Yes	GSE86189_GA.po.all.FUMA	5.24107e-05	1:47924371:A/G;1:47935863:A/T;1:47942422:A/G	3
ENSG00000142961	MOB3C	47073387	47082563	-1	protein_coding	148932	MOB3C	0.0340049542449835	-0.404012168	0	0	0	NA	NA	NA	NA	Yes	GSE86189_GA.po.all.FUMA	5.24107e-05	1:47924371:A/G;1:47935863:A/T;1:47942422:A/G	3
ENSG00000123472	ATPAF1	47098409	47139539	-1	protein_coding	64756	ATPAF1	0.236382638181407	0.513623415	0	0	0	NA	NA	NA	NA	Yes	GSE86189_GA.po.all.FUMA	5.24107e-05	1:47924371:A/G;1:47935863:A/T;1:47942422:A/G	3
ENSG00000159658	EFCAB14	47124366	47184824	-1	protein_coding	9813	EFCAB14	0.000132862974740103	-0.514808887	0	0	0	NA	NA	NA	NA	Yes	GSE86189_GA.po.all.FUMA	5.24107e-05	1:47924371:A/G;1:47935863:A/T;1:47942422:A/G	3
ENSG00000186118	TEX38	47134527	47139266	1	protein_coding	374973	TEX38	NA	0.173803607	0	0	0	NA	NA	NA	NA	Yes	GSE86189_GA.po.all.FUMA	5.24107e-05	1:47924371:A/G;1:47935863:A/T;1:47942422:A/G	3
ENSG00000228237	EFCAB14-AS1	47139708	47157770	1	antisense	100130197	EFCAB14-AS1	NA	NA	0	0	0	NA	NA	NA	NA	Yes	GSE86189_GA.po.all.FUMA	5.24107e-05	1:47924371:A/G;1:47935863:A/T;1:47942422:A/G	3
ENSG00000142973	CYP4B1	47223510	47285085	1	protein_coding	1580	CYP4B1	5.37386386780508e-10	1.046576656	0	0	0	NA	NA	NA	NA	Yes	GSE86189_GA.po.all.FUMA	5.24107e-05	1:47924371:A/G;1:47935863:A/T;1:47942422:A/G	3
ENSG00000187048	CYP4A11	47394849	47407156	-1	protein_coding	1579	CYP4A11	1.57399320908396e-15	0.786483004	0	0	0	NA	NA	NA	NA	Yes	GSE86189_GA.po.all.FUMA	5.24107e-05	1:47924371:A/G;1:47935863:A/T;1:47942422:A/G	3
ENSG00000186377	CYP4X1	47427036	47516423	1	protein_coding	260293	CYP4X1	1.72089900913095e-06	-0.254750815	0	0	0	NA	NA	NA	NA	Yes	GSE86189_GA.po.all.FUMA	5.24107e-05	1:47924371:A/G;1:47935863:A/T;1:47942422:A/G	3
ENSG00000259832	CYP4A26P	47433351	47433546	-1	pseudogene	107080641	CYP4A26P	NA	NA	0	0	0	NA	NA	NA	NA	Yes	GSE86189_GA.po.all.FUMA	5.24107e-05	1:47924371:A/G;1:47935863:A/T;1:47942422:A/G	3
ENSG00000186160	CYP4Z1	47533160	47583991	1	protein_coding	199974	CYP4Z1	5.28062048296512e-06	-0.342554663	0	0	0	NA	NA	NA	NA	Yes	GSE86189_GA.po.all.FUMA	5.24107e-05	1:47924371:A/G;1:47935863:A/T;1:47942422:A/G	3
ENSG00000162365	CYP4A22	47603107	47615413	1	protein_coding	284541	CYP4A22	1.4221825850504e-08	2.250673923	0	0	0	NA	NA	NA	NA	Yes	GSE86189_GA.po.all.FUMA	5.24107e-05	1:47924371:A/G;1:47935863:A/T;1:47942422:A/G	3

ENSG00000162366	PDZK1IP1	47649265	47656716	-1	protein_coding	10158	PDZK1IP1	0.000484064755406402	-0.343318344	0	0	0	NA	NA	NA	NA	Yes	GSE86189_GA.po.all.FUMA	5.24107e-05	1:47924371:A/G;1:47935863:A/T;1:47942422:A/G	3
ENSG00000162367	TAL1	47681962	47697892	-1	protein_coding	6886	TAL1	0.824692214968038	-0.797257236	0	0	0	NA	NA	NA	NA	Yes	GSE86189_GA.po.all.FUMA	5.24107e-05	1:47924371:A/G;1:47935863:A/T;1:47942422:A/G	3
ENSG00000123473	STIL	47715811	47779819	-1	protein_coding	6491	STIL	0.202687840102417	-0.129749688	0	0	0	NA	NA	NA	NA	Yes	GSE86189_GA.po.all.FUMA	5.24107e-05	1:47924371:A/G;1:47935863:A/T;1:47942422:A/G	3
ENSG00000162368	CMPK1	47799469	47844511	1	protein_coding	51727	CMPK1	0.319273509909851	-0.042960407	0	0	0	NA	NA	NA	NA	Yes	GSE86189_GA.po.all.FUMA	5.24107e-05	1:47924371:A/G;1:47935863:A/T;1:47942422:A/G	3
ENSG00000225762	RP11-511I2.2	47846600	47874149	-1	antisense	NA	NA	NA	NA	0	0	0	NA	NA	NA	NA	Yes	GSE86189_GA.po.all.FUMA	5.24107e-05	1:47924371:A/G;1:47935863:A/T;1:47942422:A/G	3
ENSG00000186790	FOXE3	47881744	47883723	1	protein_coding	2301	FOXE3	NA	-0.345111816	1	12.702		NA	NA	NA	NA	Yes	GSE86189_GA.po.all.FUMA	5.24107e-05	1:47924371:A/G;1:47935863:A/T;1:47942422:A/G	3
ENSG00000237424	FOXD2-AS1	47897805	47900313	-1	antisense	84793	FOXD2-AS1	NA	NA	1	12.702		NA	NA	NA	NA	Yes	GSE86189_GA.po.all.FUMA	5.24107e-05	1:47924371:A/G;1:47935863:A/T;1:47942422:A/G	3
ENSG00000186564	FOXD2	47901689	47906363	1	protein_coding	2306	FOXD2	NA	-0.586901745	1	12.702		NA	NA	NA	NA	Yes	GSE86189_GA.po.all.FUMA	5.24107e-05	1:47924371:A/G;1:47935863:A/T;1:47942422:A/G	3
ENSG00000269113	TRABD2B	48226200	48462567	-1	protein_coding	388630	TRABD2B	0.370916244398265	NA	0	0	0	NA	NA	NA	NA	Yes	GSE86189_GA.po.all.FUMA	5.24107e-05	1:47924371:A/G;1:47935863:A/T;1:47942422:A/G	3
ENSG00000223814	RP11-543D5.2	48226804	48231219	-1	lincRNA	NA	NA	NA	NA	0	0	0	NA	NA	NA	NA	Yes	GSE86189_GA.po.all.FUMA	5.24107e-05	1:47924371:A/G;1:47935863:A/T;1:47942422:A/G	3
ENSG00000273395	FLJ00388	48226979	48227290	-1	protein_coding	NA	NA	NA	NA	0	0	0	NA	NA	NA	NA	Yes	GSE86189_GA.po.all.FUMA	5.24107e-05	1:47924371:A/G;1:47935863:A/T;1:47942422:A/G	3
ENSG00000237180	CYP46A4P	48555040	48555306	1	pseudogene	100874494	CYP46A4P	NA	NA	0	0	0	NA	NA	NA	NA	Yes	GSE86189_GA.po.all.FUMA	5.24107e-05	1:47924371:A/G;1:47935863:A/T;1:47942422:A/G	3
ENSG00000269709	AL109659.1	48569306	48569878	1	protein_coding	NA	NA	NA	NA	0	0	0	NA	NA	NA	NA	Yes	GSE86189_GA.po.all.FUMA	5.24107e-05	1:47924371:A/G;1:47935863:A/T;1:47942422:A/G	3
ENSG00000117834	SLC5A9	48688357	48714316	1	protein_coding	200010	SLC5A9	6.41959687057975e-13	-0.222478075	0	0	0	NA	NA	NA	NA	Yes	GSE86189_GA.po.all.FUMA	5.24107e-05	1:47924371:A/G;1:47935863:A/T;1:47942422:A/G	3
ENSG00000132122	SPATA6	48761044	48937845	-1	protein_coding	54558	SPATA6	2.02900598310508e-11	-0.304001755	0	0	0	NA	NA	NA	NA	Yes	GSE86189_GA.po.all.FUMA	5.24107e-05	1:47924371:A/G;1:47935863:A/T;1:47942422:A/G	3
ENSG00000162373	BEND5	49193195	49242641	-1	protein_coding	79656	BEND5	0.114990689141033	NA	0	0	0	NA	NA	NA	NA	Yes	GSE86189_GA.po.all.FUMA	5.24107e-05	1:47924371:A/G;1:47935863:A/T;1:47942422:A/G	3
ENSG00000241794	SPRR2A	153028589	153030013	-1	protein_coding	6700	SPRR2A	0.160255855292028	-0.203742013	0	0	0	NA	NA	NA	NA	Yes	GSE86189_GA.po.all.FUMA	6.38828e-06	1:155033308:A/G	4
ENSG00000196805	SPRR2B	153042700	153113927	-1	protein_coding	6701	SPRR2B	0.157442680643288	0.273940428	0	0	0	NA	NA	NA	NA	Yes	GSE86189_GA.po.all.FUMA	6.38828e-06	1:155033308:A/G	4
ENSG00000203785	SPRR2E	153065611	153078660	-1	protein_coding	6704	SPRR2E	0.15458733678847	0.04799456	0	0	0	NA	NA	NA	NA	Yes	GSE86189_GA.po.all.FUMA	6.38828e-06	1:155033308:A/G	4
ENSG00000244094	SPRR2F	153084590	153085991	-1	protein_coding	6705	SPRR2F	0.00362203147645598	-0.186943673	0	0	0	NA	NA	NA	NA	Yes	GSE86189_GA.po.all.FUMA	6.38828e-06	1:155033308:A/G	4
ENSG00000229035	SPRR2C	153112967	153113185	-1	pseudogene	6702	SPRR2C	NA	NA	0	0	0	NA	NA	NA	NA	Yes	GSE86189_GA.po.all.FUMA	6.38828e-06	1:155033308:A/G	4
ENSG00000159516	SPRR2G	153122058	153123345	-1	protein_coding	6706	SPRR2G	0.0266080186616705	0.092655652	0	0	0	NA	NA	NA	NA	Yes	GSE86189_GA.po.all.FUMA	6.38828e-06	1:155033308:A/G	4

ENSG00000203784	LELP1	153175919	153177596	1	protein_coding	149018	LELP1	0.000473718090816196	0.161596053	0	0	0	NA	NA	NA	NA	Yes	GSE86189_GA.po.all.FUMA	6.38828e-06	1:155033308:A/G	4
ENSG00000203783	PRR9	153190060	153191793	1	protein_coding	574414	PRR9	NA	-0.032711126	0	0	0	NA	NA	NA	NA	Yes	GSE86189_GA.po.all.FUMA	6.38828e-06	1:155033308:A/G	4
ENSG00000230779	RP1-140J1.4	153197556	153198482	1	pseudogene	NA	NA	NA	NA	0	0	0	NA	NA	NA	NA	Yes	GSE86189_GA.po.all.FUMA	6.38828e-06	1:155033308:A/G	4
ENSG00000203782	LOR	153232176	153234598	1	protein_coding	4014	LOR	0.179479999341018	-0.139897696	0	0	0	NA	NA	NA	NA	Yes	GSE86189_GA.po.all.FUMA	6.38828e-06	1:155033308:A/G	4
ENSG00000159527	PGLYRP3	153270338	153283194	-1	protein_coding	114771	PGLYRP3	9.00246757206822e-05	0.627415477	0	0	0	NA	NA	NA	NA	Yes	GSE86189_GA.po.all.FUMA	6.38828e-06	1:155033308:A/G	4
ENSG00000163218	PGLYRP4	153302596	153321316	-1	protein_coding	57115	PGLYRP4	2.63028984617915e-08	0.529329994	0	0	0	NA	NA	NA	NA	Yes	GSE86189_GA.po.all.FUMA	6.38828e-06	1:155033308:A/G	4
ENSG00000163220	S100A9	153330330	153333503	1	protein_coding	6280	S100A9	0.667440117616727	0.10100566	0	0	0	NA	NA	NA	NA	Yes	GSE86189_GA.po.all.FUMA	6.38828e-06	1:155033308:A/G	4
ENSG00000163221	S100A12	153346184	153348125	-1	protein_coding	6283	S100A12	0.0335297458461187	-0.048060867	0	0	0	NA	NA	NA	NA	Yes	GSE86189_GA.po.all.FUMA	6.38828e-06	1:155033308:A/G	4
ENSG00000143546	S100A8	153362508	153363664	-1	protein_coding	6279	S100A8	0.186216605301329	-0.286220998	0	0	0	NA	NA	NA	NA	Yes	GSE86189_GA.po.all.FUMA	6.38828e-06	1:155033308:A/G	4
ENSG00000224784	AL591704.7	153369067	153369247	-1	pseudogene	NA	NA	NA	NA	0	0	0	NA	NA	NA	NA	Yes	GSE86189_GA.po.all.FUMA	6.38828e-06	1:155033308:A/G	4
ENSG00000184330	S100A7A	153389000	153395701	1	protein_coding	338324	S100A7A	0.0315992767749722	0.979371556	0	0	0	NA	NA	NA	NA	Yes	GSE86189_GA.po.all.FUMA	6.38828e-06	1:155033308:A/G	4
ENSG00000203781	AL591704.9	153399496	153400877	-1	pseudogene	NA	NA	NA	NA	0	0	0	NA	NA	NA	NA	Yes	GSE86189_GA.po.all.FUMA	6.38828e-06	1:155033308:A/G	4
ENSG00000197364	S100A7L2	153409534	153412425	-1	protein_coding	645922	S100A7L2	0.558124073655943	NA	0	0	0	NA	NA	NA	NA	Yes	GSE86189_GA.po.all.FUMA	6.38828e-06	1:155033308:A/G	4
ENSG00000143556	S100A7	153430220	153433177	-1	protein_coding	6278	S100A7	0.547478872262447	-0.238780496	0	0	0	NA	NA	NA	NA	Yes	GSE86189_GA.po.all.FUMA	6.38828e-06	1:155033308:A/G	4
ENSG00000238279	BX470102.3	153506079	153507591	1	antisense	NA	NA	NA	NA	0	0	0	NA	NA	NA	NA	Yes	GSE86189_GA.po.all.FUMA	6.38828e-06	1:155033308:A/G	4
ENSG00000197956	S100A6	153507075	153508720	-1	protein_coding	6277	S100A6	0.178917966295076	0.53720034	0	0	0	NA	NA	NA	NA	Yes	GSE86189_GA.po.all.FUMA	6.38828e-06	1:155033308:A/G	4
ENSG00000196420	S100A5	153509623	153514241	-1	protein_coding	6276	S100A5	4.81203056762956e-05	0.864406767	0	0	0	NA	NA	NA	NA	Yes	GSE86189_GA.po.all.FUMA	6.38828e-06	1:155033308:A/G	4
ENSG00000196154	S100A4	153516089	153522612	-1	protein_coding	6275	S100A4	0.00465145853748136	0.0479362	0	0	0	NA	NA	NA	NA	Yes	GSE86189_GA.po.all.FUMA	6.38828e-06	1:155033308:A/G	4
ENSG00000188015	S100A3	153519805	153521848	-1	protein_coding	6274	S100A3	0.000978354164779786	0.503645217	0	0	0	NA	NA	NA	NA	Yes	GSE86189_GA.po.all.FUMA	6.38828e-06	1:155033308:A/G	4
ENSG00000196754	S100A2	153533584	153540366	-1	protein_coding	6273	S100A2	0.000135122428502476	0.051427591	0	0	0	NA	NA	NA	NA	Yes	GSE86189_GA.po.all.FUMA	6.38828e-06	1:155033308:A/G	4
ENSG00000188643	S100A16	153579362	153585621	-1	protein_coding	140576	S100A16	0.00563845182351615	-0.327655231	0	0	0	NA	NA	NA	NA	Yes	GSE86189_GA.po.all.FUMA	6.38828e-06	1:155033308:A/G	4
ENSG00000189334	S100A14	153586731	153589462	-1	protein_coding	57402	S100A14	0.0570469536230336	0.20033313	0	0	0	NA	NA	NA	NA	Yes	GSE86189_GA.po.all.FUMA	6.38828e-06	1:155033308:A/G	4

ENSG00000189171	S100A13	153591263	153606568	-1	protein_coding	6284	S100A13	0.0351531421862098	-0.436440334	0	0	0	NA	NA	NA	NA	Yes	GSE86189_GA.po.all.FUMA	6.38828e-06	1:155033308:A/G	4
ENSG00000271853	RP1-178F15.5	153598808	153606816	-1	processed_transcript	NA	NA	NA	NA	0	0	0	NA	NA	NA	NA	Yes	GSE86189_GA.po.all.FUMA	6.38828e-06	1:155033308:A/G	4
ENSG00000160678	S100A1	153600402	153604513	1	protein_coding	6271	S100A1	0.00465602079501881	-0.05346962	0	0	0	NA	NA	NA	NA	Yes	GSE86189_GA.po.all.FUMA	6.38828e-06	1:155033308:A/G	4
ENSG00000272030	RP1-178F15.4	153603914	153606873	-1	antisense	NA	NA	NA	NA	0	0	0	NA	NA	NA	NA	Yes	GSE86189_GA.po.all.FUMA	6.38828e-06	1:155033308:A/G	4
ENSG00000160679	CHTOP	153606525	153618782	1	protein_coding	26097	CHTOP	0.998109469154056	0.088373592	0	0	0	NA	NA	NA	NA	Yes	GSE86189_GA.po.all.FUMA	6.38828e-06	1:155033308:A/G	4
ENSG00000143553	SNAPIN	153631130	153634306	1	protein_coding	23557	SNAPIN	3.04880662881494e-05	0.367183084	0	0	0	NA	NA	NA	NA	Yes	GSE86189_GA.po.all.FUMA	6.38828e-06	1:155033308:A/G	4
ENSG00000143621	ILF2	153634512	153643524	-1	protein_coding	3608	ILF2	0.997052017177466	-0.374027549	0	0	0	NA	NA	NA	NA	Yes	GSE86189_GA.po.all.FUMA	6.38828e-06	1:155033308:A/G	4
ENSG00000169418	NPR1	153651113	153666468	1	protein_coding	4881	NPR1	0.000172508543840191	-1.095207972	0	0	0	NA	NA	NA	NA	Yes	GSE86189_GA.po.all.FUMA	6.38828e-06	1:155033308:A/G	4
ENSG00000235015	RP11-216N14.1	153689779	153690823	-1	pseudogene	NA	NA	NA	NA	0	0	0	NA	NA	NA	NA	Yes	GSE86189_GA.po.all.FUMA	6.38828e-06	1:155033308:A/G	4
ENSG00000199565	Y_RNA	153698728	153698829	-1	misc_RNA	NA	NA	NA	NA	0	0	0	NA	NA	NA	NA	Yes	GSE86189_GA.po.all.FUMA	6.38828e-06	1:155033308:A/G	4
ENSG00000143624	INTS3	153700543	153746555	1	protein_coding	65123	INTS3	0.999997023490929	-0.57894986	0	0	0	NA	NA	NA	NA	Yes	GSE86189_GA.po.all.FUMA	6.38828e-06	1:155033308:A/G	4
ENSG00000143554	SLC27A3	153746830	153752633	1	protein_coding	11000	SLC27A3	7.8014773851824e-11	-0.248954346	0	0	0	NA	NA	NA	NA	Yes	GSE86189_GA.po.all.FUMA	6.38828e-06	1:155033308:A/G	4
ENSG00000143614	GATAD2B	153777201	153895451	-1	protein_coding	57459	GATAD2B	0.998594716203308	-0.756320233	0	0	0	NA	NA	NA	NA	Yes	GSE86189_GA.po.all.FUMA	6.38828e-06	1:155033308:A/G	4
ENSG00000198837	DENND4B	153901977	153919172	-1	protein_coding	9909	DENND4B	0.999987069079939	NA	0	0	0	NA	NA	NA	NA	Yes	GSE86189_GA.po.all.FUMA	6.38828e-06	1:155033308:A/G	4
ENSG00000160741	CRTC2	153920145	153931101	-1	protein_coding	200186	CRTC2	0.996361791046642	-0.166194581	0	0	0	NA	NA	NA	NA	Yes	GSE86189_GA.po.all.FUMA	6.38828e-06	1:155033308:A/G	4
ENSG00000143570	SLC39A1	153931575	153940188	-1	protein_coding	27173	SLC39A1	0.167076829803909	-0.194108872	0	0	0	NA	NA	NA	NA	Yes	GSE86189_GA.po.all.FUMA	6.38828e-06	1:155033308:A/G	4
ENSG00000273026	RP11-422P24.10	153938992	153939406	1	antisense	NA	NA	NA	NA	0	0	0	NA	NA	NA	NA	Yes	GSE86189_GA.po.all.FUMA	6.38828e-06	1:155033308:A/G	4
ENSG00000143578	CREB3L4	153940010	153946839	1	protein_coding	148327	CREB3L4	8.06202744664506e-05	-0.607235894	0	0	0	NA	NA	NA	NA	Yes	GSE86189_GA.po.all.FUMA	6.38828e-06	1:155033308:A/G	4
ENSG00000143543	JTB	153946745	153950164	-1	protein_coding	10899	JTB	0.645223905316483	-1.138138424	0	0	0	NA	NA	NA	NA	Yes	GSE86189_GA.po.all.FUMA	6.38828e-06	1:155033308:A/G	4
ENSG00000272654	RP11-422P24.11	153950219	153951636	1	lincRNA	NA	NA	NA	NA	0	0	0	NA	NA	NA	NA	Yes	GSE86189_GA.po.all.FUMA	6.38828e-06	1:155033308:A/G	4
ENSG00000143545	RAB13	153954127	153958834	-1	protein_coding	5872	RAB13	0.57012570509712	-0.127798629	0	0	0	NA	NA	NA	NA	Yes	GSE86189_GA.po.all.FUMA	6.38828e-06	1:155033308:A/G	4

ENSG00000177954	RPS27	153963235	153964626	1	protein_coding	6232	RPS27	0.697777961357601	-0.293706852	0	0	0	NA	NA	NA	NA	Yes	GSE86189_GA.po.all.FUMA	6.38828e-06	1:155033308:A/G	4
ENSG00000143552	NUP210L	153965161	154127592	-1	protein_coding	91181	NUP210L	0.144640619732186	0.363113447	0	0	0	NA	NA	NA	NA	Yes	GSE86189_GA.po.all.FUMA:GSE86189_GA.pp.all.FUMA	6.38828e-06	1:155033308:A/G:1:156032887:A/G	4
ENSG00000231416	RP11-422P24.9	153968108	153968436	1	pseudogene	NA	NA	NA	NA	0	0	0	NA	NA	NA	NA	Yes	GSE86189_GA.po.all.FUMA	6.38828e-06	1:155033308:A/G	4
ENSG00000252669	U3	153970517	153970627	-1	snoRNA	NA	NA	NA	NA	0	0	0	NA	NA	NA	NA	Yes	GSE86189_GA.po.all.FUMA	6.38828e-06	1:155033308:A/G	4
ENSG00000231837	RPS7P2	154051342	154052439	1	pseudogene	100270868	RPS7P2	NA	NA	0	0	0	NA	NA	NA	NA	Yes	GSE86189_GA.po.all.FUMA	0.000130157	1:156032887:A/G	4
ENSG00000263987	MIR5698	154076997	154077068	-1	miRNA	100847024	MIR5698	NA	NA	0	0	0	NA	NA	NA	NA	Yes	GSE86189_GA.po.all.FUMA	0.000130157	1:156032887:A/G	4
ENSG00000143549	TPM3	154127784	154167124	-1	protein_coding	7170	TPM3	0.191983984043152	-0.527201952	0	0	1	0.0171261	NA	GTEx/v8/Stomach	+	Yes	GSE86189_GA.po.all.FUMA:GSE86189_GA.pp.all.FUMA	6.38828e-06	1:155033308:A/G;1:155033308:A/G:1:156032887:A/G	4
ENSG00000264384	RN7SL431P	154138723	154139025	-1	misc_RNA	106866913	RN7SL431P	NA	NA	0	0	0	NA	NA	NA	NA	Yes	GSE86189_GA.po.all.FUMA	0.000130157	1:156032887:A/G	4
ENSG00000215938	MIR190B	154166141	154166219	-1	miRNA	100126346	MIR190B	NA	NA	0	0	0	NA	NA	NA	NA	Yes	GSE86189_GA.po.all.FUMA:GSE86189_GA.pp.all.FUMA	6.38828e-06	1:155033308:A/G:1:156032887:A/G	4
ENSG00000163263	C1orf189	154171848	154178809	-1	protein_coding	388701	C1orf189	0.000114764896185549	-0.162537364	0	0	0	NA	NA	NA	NA	Yes	GSE86189_GA.po.all.FUMA:GSE86189_GA.pp.all.FUMA	6.38828e-06	1:155033308:A/G:1:156032887:A/G	4
ENSG00000143612	C1orf43	154179182	154193104	-1	protein_coding	25912	C1orf43	0.173985957447014	-0.684900977	0	0	0	NA	NA	NA	NA	Yes	GSE86189_GA.po.all.FUMA:GSE86189_GA.pp.all.FUMA	6.38828e-06	1:155033308:A/G:1:156032887:A/G	4
ENSG00000143569	UBAP2L	154192655	154243986	1	protein_coding	9898	UBAP2L	0.999999873126418	0.35646256	0	0	0	NA	NA	NA	NA	Yes	GSE86189_GA.po.all.FUMA:GSE86189_GA.pp.all.FUMA	6.38828e-06	1:155033308:A/G:1:156032887:A/G	4
ENSG00000252817	AL590431.1	154226921	154226997	1	miRNA	NA	NA	NA	NA	0	0	0	NA	NA	NA	NA	Yes	GSE86189_GA.po.all.FUMA	0.000130157	1:156032887:A/G	4
ENSG00000201129	SNORA58	154232203	154232338	1	snoRNA	109617023	SNORA58B	NA	NA	0	0	0	NA	NA	NA	NA	Yes	GSE86189_GA.po.all.FUMA	0.000130157	1:156032887:A/G	4
ENSG00000143575	HAX1	154244987	154248351	1	protein_coding	10456	HAX1	1.10582340278236e-05	-0.2412109	0	0	0	NA	NA	NA	NA	Yes	GSE86189_GA.po.all.FUMA:G	6.38828e-06	1:155033308:A/G:1:156032887:A/G	4

																		SE86189_GA.p p.all.FUMA			
ENSG00000252682	SNORD59	154260936	154261003	1	snoRNA	NA	NA	NA	NA	0	0	0	NA	NA	NA	NA	Yes	GSE86189_GA.po.all.FUMA	0.000130157	1:156032887:A/G	4
ENSG00000212292	RNU6-239P	154267979	154268077	1	snRNA	106480563	RNU6-239P	NA	NA	0	0	0	NA	NA	NA	NA	Yes	GSE86189_GA.po.all.FUMA	0.000130157	1:156032887:A/G	4
ENSG00000222457	RNU6-121P	154270126	154270224	1	snRNA	106481215	RNU6-121P	NA	NA	0	0	0	NA	NA	NA	NA	Yes	GSE86189_GA.po.all.FUMA	0.000130157	1:156032887:A/G	4
ENSG00000143595	AQP10	154293566	154297801	1	protein_coding	89872	AQP10	0.0152782932027765	-0.980404431	0	0	0	NA	NA	NA	NA	Yes	GSE86189_GA.po.all.FUMA:GSE86189_GA.pp.all.FUMA	6.38828e-06	1:155033308:A/G:1:156032887:A/G	4
ENSG00000143515	ATP8B2	154298029	154323783	1	protein_coding	57198	ATP8B2	0.999564591733884	-0.493030255	0	0	0	NA	NA	NA	NA	Yes	GSE86189_GA.po.all.FUMA:GSE86189_GA.pp.all.FUMA	6.38828e-06	1:155033308:A/G:1:156032887:A/G	4
ENSG00000238365	RNU7-57P	154311219	154311278	1	snRNA	100151652	RNU7-57P	NA	NA	0	0	0	NA	NA	NA	NA	Yes	GSE86189_GA.po.all.FUMA	0.000130157	1:156032887:A/G	4
ENSG00000237920	RP11-350G8.4	154349442	154351749	-1	pseudogene	NA	NA	NA	NA	0	0	0	NA	NA	NA	NA	Yes	GSE86189_GA.po.all.FUMA	0.000130157	1:156032887:A/G	4
ENSG00000226855	RP11-350G8.3	154350683	154351565	-1	pseudogene	NA	NA	NA	NA	0	0	0	NA	NA	NA	NA	Yes	GSE86189_GA.po.all.FUMA	0.000130157	1:156032887:A/G	4
ENSG00000228013	RP11-350G8.5	154374804	154379040	-1	antisense	101928101	IL6R-AS1	NA	NA	0	0	0	NA	NA	NA	NA	Yes	GSE86189_GA.po.all.FUMA:GSE86189_GA.pp.all.FUMA	6.38828e-06	1:155033308:A/G:1:156032887:A/G	4
ENSG00000160712	IL6R	154377669	154441926	1	protein_coding	3570	IL6R	0.0478690367242407	-0.79334249	0	0	0	NA	NA	NA	NA	Yes	GSE86189_GA.po.all.FUMA:GSE86189_GA.pp.all.FUMA	6.38828e-06	1:155033308:A/G:1:156032887:A/G	4
ENSG00000228264	RP11-350G8.7	154386845	154387613	-1	pseudogene	NA	NA	NA	NA	0	0	0	NA	NA	NA	NA	Yes	GSE86189_GA.po.all.FUMA	0.000130157	1:156032887:A/G	4
ENSG00000169291	SHE	154442248	154474589	-1	protein_coding	126669	SHE	0.721620118578427	-1.32995799	0	0	0	NA	NA	NA	NA	Yes	GSE86189_GA.po.all.FUMA:GSE86189_GA.pp.all.FUMA	6.38828e-06	1:155033308:A/G:1:156032887:A/G	4
ENSG00000273110	RP11-350G8.9	154452488	154453977	1	antisense	NA	NA	NA	NA	0	0	0	NA	NA	NA	NA	Yes	GSE86189_GA.po.all.FUMA	0.000130157	1:156032887:A/G	4
ENSG00000163239	TDRD10	154474695	154520623	1	protein_coding	126668	TDRD10	2.45421655192587e-06	-0.428923944	0	0	0	NA	NA	NA	NA	Yes	GSE86189_GA.po.all.FUMA:GSE86189_GA.pp.all.FUMA	6.38828e-06	1:155033308:A/G:1:156032887:A/G	4
ENSG00000160714	UBE2Q1	154521053	154531504	-1	protein_coding	55585	UBE2Q1	0.969363486984384	-0.5126963	0	0	0	NA	NA	NA	NA	Yes	GSE86189_GA.po.all.FUMA:G	6.38828e-06	1:155033308:A/G:1:156032887:A/G	4

																		SE86189_GA.pp.all.FUMA			
ENSG00000229780	UBE2Q1-AS1	154526085	154527493	1	antisense	100874097	UBE2Q1-AS1	NA	NA	0	0	0	NA	NA	NA	NA	Yes	GSE86189_GA.po.all.FUMA	0.000130157	1:156032887:A/G	4
ENSG00000160716	CHRNB2	154540257	154552502	1	protein_coding	1141	CHRNB2	0.720254345017317	-0.35158637	0	0	0	NA	NA	NA	NA	Yes	GSE86189_GA.po.all.FUMA:GSE86189_GA.pp.all.FUMA	6.38828e-06	1:155033308:A/G:1:156032887:A/G	4
ENSG00000233875	RP11-61L14.6	154551541	154552139	-1	antisense	NA	NA	NA	NA	0	0	0	NA	NA	NA	NA	Yes	GSE86189_GA.po.all.FUMA	0.000130157	1:156032887:A/G	4
ENSG00000160710	ADAR	154554538	154600475	-1	protein_coding	103	ADAR	0.907595373477161	-0.566599759	0	0	0	NA	NA	NA	NA	Yes	GSE86189_GA.po.all.FUMA:GSE86189_GA.pp.all.FUMA	6.38828e-06	1:155033308:A/G:1:156032887:A/G	4
ENSG00000268141	AL606500.1	154585067	154585190	1	protein_coding	NA	NA	NA	NA	0	0	0	NA	NA	NA	NA	Yes	GSE86189_GA.po.all.FUMA:GSE86189_GA.pp.all.FUMA	6.38828e-06	1:155033308:A/G:1:156032887:A/G	4
ENSG00000143603	KCNN3	154669931	154842756	-1	protein_coding	3782	KCNN3	0.864940881345047	-0.629504502	0	0	0	NA	NA	NA	NA	Yes	GSE86189_GA.po.all.FUMA:GSE86189_GA.pp.all.FUMA	6.38828e-06	1:155033308:A/G:1:156032887:A/G	4
ENSG00000163344	PMVK	154897210	154909467	-1	protein_coding	10654	PMVK	0.000305454915643329	-0.527689982	0	0	0	NA	NA	NA	NA	Yes	GSE86189_GA.po.all.FUMA:GSE86189_GA.pp.all.FUMA	6.38828e-06	1:155033308:A/G:1:156032887:A/G	4
ENSG00000270361	RP11-307C12.13	154909846	154910535	1	lincRNA	NA	NA	NA	NA	0	0	0	NA	NA	NA	NA	Yes	GSE86189_GA.po.all.FUMA:GSE86189_GA.pp.all.FUMA	6.38828e-06	1:155033308:A/G:1:156032887:A/G	4
ENSG00000163346	PBXIP1	154916552	154928599	-1	protein_coding	57326	PBXIP1	5.79843921369484e-07	0.443722875	0	0	0	NA	NA	NA	NA	Yes	GSE86189_GA.po.all.FUMA:GSE86189_GA.pp.all.FUMA	6.38828e-06	1:155033308:A/G:1:156032887:A/G	4
ENSG00000163348	PYGO2	154929502	154936329	-1	protein_coding	90780	PYGO2	0.923104365059159	-0.525257716	0	0	0	NA	NA	NA	NA	Yes	GSE86189_GA.po.all.FUMA:GSE86189_GA.pp.all.FUMA	6.38828e-06	1:155033308:A/G:1:156032887:A/G	4
ENSG00000271380	RP11-307C12.12	154934301	154935099	1	antisense	NA	NA	NA	NA	0	0	0	NA	NA	NA	NA	Yes	GSE86189_GA.po.all.FUMA:GSE86189_GA.pp.all.FUMA	6.38828e-06	1:156032887:A/G:1:155033308:A/G	4
ENSG00000160691	SHC1	154934774	154946871	-1	protein_coding	6464	SHC1	0.341634863000658	-1.175729097	0	0	0	NA	NA	NA	NA	Yes	GSE86189_GA.po.all.FUMA:G	6.38828e-06	1:155033308:A/G:1:156032887:A/G	4

																		SE86189_GA.pp.all.FUMA			
ENSG00000173207	CKS1B	154947129	154951725	1	protein_coding	1163	CKS1B	0.723280936456517	-0.667051695	0	0	0	NA	NA	NA	NA	Yes	GSE86189_GA.po.all.FUMA:GSE86189_GA.pp.all.FUMA	6.38828e-06	1:155033308:A/G:1:156032887:A/G	4
ENSG00000264349	MIR4258	154948169	154948259	1	miRNA	100423020	MIR4258	NA	NA	0	0	0	NA	NA	NA	NA	Yes	GSE86189_GA.po.all.FUMA:GSE86189_GA.pp.all.FUMA	6.38828e-06	1:155033308:A/G:1:156032887:A/G	4
ENSG00000160688	FLAD1	154955814	154965587	1	protein_coding	80308	FLAD1	0.0917935111458052	-0.342100174	0	0	0	NA	NA	NA	NA	Yes	GSE86189_GA.po.all.FUMA:GSE86189_GA.pp.all.FUMA	6.38828e-06	1:155033308:A/G:1:156032887:A/G	4
ENSG00000163352	LENEP	154966062	154966791	1	protein_coding	55891	LENEP	0.0322826229786669	-0.345882836	0	0	0	NA	NA	NA	NA	Yes	GSE86189_GA.po.all.FUMA:GSE86189_GA.pp.all.FUMA	6.38828e-06	1:155033308:A/G:1:156032887:A/G	4
ENSG00000160685	ZBTB7B	154975127	154990998	1	protein_coding	51043	ZBTB7B	0.837393370882557	NA	0	0	0	NA	NA	NA	NA	Yes	GSE86189_GA.po.all.FUMA:GSE86189_GA.pp.all.FUMA	6.38828e-06	1:155033308:A/G:1:156032887:A/G	4
ENSG00000163354	DCST2	154990996	155006257	-1	protein_coding	127579	DCST2	1.45881629274481e-09	0.250200946	1	19.809		NA	NA	NA	NA	Yes	GSE86189_GA.po.all.FUMA:GSE86189_GA.pp.all.FUMA	6.38828e-06	1:155033308:A/G;1:155033308:A/G:1:156032887:A/G	4
ENSG00000163357	DCST1	155006300	155023406	1	protein_coding	149095	DCST1	3.88695922784292e-15	NA	1	19.809		NA	NA	NA	NA	Yes	GSE86189_GA.po.all.FUMA:GSE86189_GA.pp.all.FUMA	6.38828e-06	1:155033308:A/G;1:155033308:A/G:1:156032887:A/G	4
ENSG00000232093	RP11-307C12.11	155017667	155018594	-1	antisense	NA	NA	NA	NA	1	19.809		NA	NA	NA	NA	Yes	GSE86189_GA.po.all.FUMA	6.38828e-06	1:155033308:A/G;1:156032887:A/G	4
ENSG00000143537	ADAM15	155023042	155035252	1	protein_coding	8751	ADAM15	1.07719204651477e-11	NA	1	19.809		NA	NA	NA	NA	Yes	GSE86189_GA.po.all.FUMA:GSE86189_GA.pp.all.FUMA	6.38828e-06	1:155033308:A/G;1:155033308:A/G:1:156032887:A/G	4
ENSG00000243364	EFNA4	155036207	155042029	1	protein_coding	1945	EFNA4	0.000252891317706202	-0.595252367	1	19.809		NA	NA	NA	NA	Yes	GSE86189_GA.po.all.FUMA:GSE86189_GA.pp.all.FUMA	6.38828e-06	1:155033308:A/G;1:155033308:A/G:1:156032887:A/G	4
ENSG00000143590	EFNA3	155036224	155060014	1	protein_coding	1944	EFNA3	0.18056410470307	-0.355848667	1	19.809		NA	NA	NA	NA	Yes	GSE86189_GA.po.all.FUMA:GSE86189_GA.pp.all.FUMA	6.38828e-06	1:155033308:A/G;1:155033308:A/G:1:156032887:A/G	4

ENSG00000251246	EFNA3	155036224	155059283	1	protein_coding	1944	EFNA3	NA	-0.355848667	1	19.809		NA	NA	NA	NA	Yes	GSE86189_GA.po.all.FUMA:GSE86189_GA.pp.all.FUMA	6.38828e-06	1:155033308:A/G;1:155033308:A/G:1:156032887:A/G	4
ENSG00000202027	Y_RNA	155092966	155093074	1	misc_RNA	NA	NA	NA	NA	0	0	0	NA	NA	NA	NA	Yes	GSE86189_GA.po.all.FUMA:GSE86189_GA.pp.all.FUMA	6.38828e-06	1:155033308:A/G:1:156032887:A/G	4
ENSG00000169242	EFNA1	155099936	155107333	1	protein_coding	1942	EFNA1	0.872063427950098	0.409868429	0	0	0	NA	NA	NA	NA	Yes	GSE86189_GA.po.all.FUMA:GSE86189_GA.pp.all.FUMA	6.38828e-06	1:155033308:A/G:1:156032887:A/G	4
ENSG00000169241	SLC50A1	155107820	155111329	1	protein_coding	55974	SLC50A1	0.130229792296048	-0.609412945	0	0	0	NA	NA	NA	NA	Yes	GSE86189_GA.po.all.FUMA:GSE86189_GA.pp.all.FUMA	6.38828e-06	1:155033308:A/G:1:156032887:A/G	4
ENSG00000179085	DPM3	155112367	155113071	-1	protein_coding	54344	DPM3	0.233470170067034	0.032111124	0	0	0	NA	NA	NA	NA	Yes	GSE86189_GA.po.all.FUMA:GSE86189_GA.pp.all.FUMA	6.38828e-06	1:155033308:A/G:1:156032887:A/G	4
ENSG00000251780	RNU7-150P	155115747	155115808	1	snRNA	NA	RNU7-150P	NA	NA	0	0	0	NA	NA	NA	NA	Yes	GSE86189_GA.po.all.FUMA:GSE86189_GA.pp.all.FUMA	6.38828e-06	1:155033308:A/G:1:156032887:A/G	4
ENSG00000223452	HMGN2P18	155121020	155121295	1	pseudogene	648822	HMGN2P18	NA	NA	0	0	0	NA	NA	NA	NA	Yes	GSE86189_GA.po.all.FUMA:GSE86189_GA.pp.all.FUMA	6.38828e-06	1:155033308:A/G:1:156032887:A/G	4
ENSG00000163463	KRTCAP2	155141884	155145951	-1	protein_coding	200185	KRTCAP2	0.0299704903065574	-0.290243823	0	0	0	NA	NA	NA	NA	Yes	GSE86189_GA.po.all.FUMA:GSE86189_GA.pp.all.FUMA	6.38828e-06	1:155033308:A/G:1:156032887:A/G	4
ENSG00000273088	RP11-201K10.3	155141885	155159748	-1	protein_coding	NA	NA	NA	NA	0	0	0	NA	NA	NA	NA	Yes	GSE86189_GA.po.all.FUMA:GSE86189_GA.pp.all.FUMA	6.38828e-06	1:155033308:A/G:1:156032887:A/G	4
ENSG00000163462	TRIM46	155145873	155157447	1	protein_coding	80128	TRIM46	0.998652125444883	-0.656689926	0	0	2	0.0328311	NA	GTEx/v8/Stomach	+	Yes	GSE86189_GA.po.all.FUMA:GSE86189_GA.pp.all.FUMA	6.38828e-06	1:156032887:A/G;1:155033308:A/G:1:156032887:A/G	4
ENSG00000185499	MUC1	155158300	155162707	-1	protein_coding	4582	MUC1	0.730964975196719	NA	0	0	0	NA	NA	NA	NA	Yes	GSE86189_GA.po.all.FUMA:G	6.38828e-06	1:155033308:A/G:1:156032887:A/G	4

																		SE86189_GA.pp.all.FUMA			
ENSG00000271748	MIR92B	155164968	155165063	1	miRNA	693235	MIR92B	NA	NA	0	0	0	NA	NA	NA	NA	Yes	GSE86189_GA.po.all.FUMA:GSE86189_GA.pp.all.FUMA	6.38828e-06	1:155033308:A/G:1:156032887:A/G	4
ENSG00000169231	THBS3	155165379	155178842	-1	protein_coding	7059	THBS3	1.20600565280442e-08	-0.314766046	0	0	1	1.36919e-05	2.97001e-16	GTEx/v8/Stomach	+	Yes	GSE86189_GA.po.all.FUMA:GSE86189_GA.pp.all.FUMA	6.38828e-06	1:155033308:A/G;1:155033308:A/G:1:156032887:A/G	4
ENSG00000231064	RP11-263K19.4	155166659	155175286	1	antisense	NA	NA	NA	NA	0	0	0	NA	NA	NA	NA	Yes	GSE86189_GA.po.all.FUMA:GSE86189_GA.pp.all.FUMA	6.38828e-06	1:155033308:A/G:1:156032887:A/G	4
ENSG00000173171	MTX1	155178490	155183615	1	protein_coding	4580	MTX1	0.198221290051836	-0.480958216	0	0	0	NA	NA	NA	NA	Yes	GSE86189_GA.po.all.FUMA:GSE86189_GA.pp.all.FUMA	6.38828e-06	1:155033308:A/G:1:156032887:A/G	4
ENSG00000236263	RP11-263K19.6	155180942	155183610	-1	antisense	NA	NA	NA	NA	0	0	1	0.00049245	NA	GTEx/v8/Stomach	+	Yes	GSE86189_GA.po.all.FUMA	6.38828e-06	1:155033308:A/G;1:156032887:A/G	4
ENSG00000160766	GBAP1	155183616	155197214	-1	pseudogene	2630	GBAP1	NA	NA	0	0	3	0.000399532	NA	GTEx/v8/Stomach	-	Yes	GSE86189_GA.po.all.FUMA	6.38828e-06	1:155033308:A/G;1:156032887:A/G	4
ENSG00000236675	MTX1P1	155200766	155204116	1	pseudogene	4581	MTX1P1	NA	NA	0	0	0	NA	NA	NA	NA	Yes	GSE86189_GA.po.all.FUMA	0.000130157	1:156032887:A/G	4
ENSG00000177628	GBA	155204243	155214490	-1	protein_coding	2629	GBA	0.0340405400496017	-0.78504846	0	0	0	NA	NA	NA	NA	Yes	GSE86189_GA.po.all.FUMA:GSE86189_GA.pp.all.FUMA	6.38828e-06	1:155033308:A/G:1:156032887:A/G	4
ENSG00000216109	AL713999.1	155206540	155206628	-1	miRNA	NA	NA	NA	NA	0	0	0	NA	NA	NA	NA	Yes	GSE86189_GA.po.all.FUMA:GSE86189_GA.pp.all.FUMA	6.38828e-06	1:155033308:A/G:1:156032887:A/G	4
ENSG00000160767	FAM189B	155216996	155225274	-1	protein_coding	10712	FAM189B	0.000482815313135408	-0.121568494	0	0	0	NA	NA	NA	NA	Yes	GSE86189_GA.po.all.FUMA:GSE86189_GA.pp.all.FUMA	6.38828e-06	1:155033308:A/G:1:156032887:A/G	4

ENSG00000116521	SCAMP3	155225770	155232221	-1	protein_coding	10067	SCAMP3	0.0128944102681846	-0.24976638	0	0	0	NA	NA	NA	NA	Yes	GSE86189_GA.po.all.FUMA:GSE86189_GA.pp.all.FUMA	6.38828e-06	1:155033308:A/G:1:156032887:A/G	4
ENSG00000176444	CLK2	155232659	155248282	-1	protein_coding	1196	CLK2	0.999952949082341	NA	0	0	0	NA	NA	NA	NA	Yes	GSE86189_GA.po.all.FUMA:GSE86189_GA.pp.all.FUMA	6.38828e-06	1:155033308:A/G:1:156032887:A/G	4
ENSG00000143630	HCN3	155247374	155259639	1	protein_coding	57657	HCN3	0.0150933826344395	0.374943277	0	0	0	NA	NA	NA	NA	Yes	GSE86189_GA.po.all.FUMA:GSE86189_GA.pp.all.FUMA	6.38828e-06	1:155033308:A/G:1:156032887:A/G	4
ENSG00000143627	PKLR	155259630	155271225	-1	protein_coding	5313	PKLR	1.00020289233933e-05	0.059195648	0	0	0	NA	NA	NA	NA	Yes	GSE86189_GA.po.all.FUMA:GSE86189_GA.pp.all.FUMA	6.38828e-06	1:155033308:A/G:1:156032887:A/G	4
ENSG00000160752	FDPS	155278539	155290457	1	protein_coding	2224	FDPS	0.00175127849063184	-0.55139783	0	0	0	NA	NA	NA	NA	Yes	GSE86189_GA.po.all.FUMA:GSE86189_GA.pp.all.FUMA	6.38828e-06	1:155033308:A/G:1:156032887:A/G	4
ENSG00000225855	RUSC1-AS1	155286654	155293967	-1	antisense	284618	RUSC1-AS1	NA	NA	0	0	0	NA	NA	NA	NA	Yes	GSE86189_GA.po.all.FUMA:GSE86189_GA.pp.all.FUMA	6.38828e-06	1:155033308:A/G:1:156032887:A/G	4
ENSG00000160753	RUSC1	155290687	155300905	1	protein_coding	23623	RUSC1	0.949429117518656	-0.575598472	0	0	0	NA	NA	NA	NA	Yes	GSE86189_GA.po.all.FUMA:GSE86189_GA.pp.all.FUMA	6.38828e-06	1:155033308:A/G:1:156032887:A/G	4
ENSG00000116539	ASH1L	155305059	155532598	-1	protein_coding	55870	ASH1L	0.999999999999834	-0.515030375	0	0	0	NA	NA	NA	NA	Yes	GSE86189_GA.po.all.FUMA:GSE86189_GA.pp.all.FUMA	6.38828e-06	1:155033308:A/G:1:156032887:A/G	4
ENSG00000207720	MIR555	155316141	155316236	-1	miRNA	693140	MIR555	NA	NA	0	0	0	NA	NA	NA	NA	Yes	GSE86189_GA.po.all.FUMA	0.000130157	1:156032887:A/G	4
ENSG00000207134	RNU6-106P	155328503	155328609	-1	snRNA	106479613	RNU6-106P	NA	NA	0	0	0	NA	NA	NA	NA	Yes	GSE86189_GA.po.all.FUMA	0.000130157	1:156032887:A/G	4
ENSG00000227773	ASH1L-IT1	155365801	155366769	-1	sense_intronic	106478976	ASH1L-IT1	NA	NA	0	0	0	NA	NA	NA	NA	Yes	GSE86189_GA.po.all.FUMA	0.000130157	1:156032887:A/G	4
ENSG00000238805	snoU13	155385349	155385453	-1	snoRNA	NA	NA	NA	NA	0	0	0	NA	NA	NA	NA	Yes	GSE86189_GA.po.all.FUMA	0.000130157	1:156032887:A/G	4
ENSG00000207144	RNU6-1297P	155389188	155389291	1	snRNA	106480657	RNU6-1297P	NA	NA	0	0	0	NA	NA	NA	NA	Yes	GSE86189_GA.po.all.FUMA	0.000130157	1:156032887:A/G	4
ENSG00000237872	POU5F1P4	155402969	155404053	1	pseudogene	645682	POU5F1P4	NA	NA	0	0	0	NA	NA	NA	NA	Yes	GSE86189_GA.po.all.FUMA	0.000130157	1:156032887:A/G	4

ENSG00000235919	ASH1L-AS1	155531821	155533775	1	processed_transcript	645676	ASH1L-AS1	NA	NA	0	0	0	NA	NA	NA	NA	Yes	GSE86189_GA.po.all.FUMA:GSE86189_GA.pp.all.FUMA	6.38828e-06	1:155033308:A/G:1:156032887:A/G	4
ENSG00000271267	RP11-29H23.7	155535841	155536097	-1	pseudogene	NA	NA	NA	NA	0	0	0	NA	NA	NA	NA	Yes	GSE86189_GA.po.all.FUMA:GSE86189_GA.pp.all.FUMA	6.38828e-06	1:155033308:A/G:1:156032887:A/G	4
ENSG00000225082	DAP3P1	155556435	155571988	1	pseudogene	359804	DAP3P1	NA	NA	0	0	0	NA	NA	NA	NA	Yes	GSE86189_GA.po.all.FUMA	0.000130157	1:156032887:A/G	4
ENSG00000223503	RP11-29H23.6	155560392	155561053	-1	pseudogene	NA	NA	NA	NA	0	0	0	NA	NA	NA	NA	Yes	GSE86189_GA.po.all.FUMA	0.000130157	1:156032887:A/G	4
ENSG00000232519	RP11-29H23.4	155579567	155580171	-1	antisense	NA	NA	NA	NA	0	0	0	NA	NA	NA	NA	Yes	GSE86189_GA.po.all.FUMA:GSE86189_GA.pp.all.FUMA	6.38828e-06	1:155033308:A/G:1:156032887:A/G	4
ENSG00000125459	MSTO1	155579979	155718153	1	protein_coding	55154	MSTO1	0.0418044849918912	-0.606608289	0	0	0	NA	NA	NA	NA	Yes	GSE86189_GA.po.all.FUMA:GSE86189_GA.pp.all.FUMA	6.38828e-06	1:155033308:A/G:1:156032887:A/G	4
ENSG00000203761	MSTO2P	155581011	155720105	1	pseudogene	100129405	MSTO2P	NA	NA	0	0	0	NA	NA	NA	NA	Yes	GSE86189_GA.po.all.FUMA:GSE86189_GA.pp.all.FUMA	6.38828e-06	1:155033308:A/G:1:156032887:A/G	4
ENSG00000246203	RP11-29H23.5	155584517	155630036	-1	pseudogene	NA	NA	NA	NA	0	0	0	NA	NA	NA	NA	Yes	GSE86189_GA.po.all.FUMA	0.000130157	1:156032887:A/G	4
ENSG00000163374	YY1AP1	155629237	155658791	-1	protein_coding	55249	YY1AP1	0.000145221258212536	0.040412345	0	0	0	NA	NA	NA	NA	Yes	GSE86189_GA.po.all.FUMA:GSE86189_GA.pp.all.FUMA	6.38828e-06	1:155033308:A/G:1:156032887:A/G	4
ENSG00000132676	DAP3	155657751	155708801	1	protein_coding	7818	DAP3	1.9076333934558e-05	0.001567485	0	0	0	NA	NA	NA	NA	Yes	GSE86189_GA.po.all.FUMA:GSE86189_GA.pp.all.FUMA	6.38828e-06	1:155033308:A/G:1:156032887:A/G	4
ENSG00000227673	RP11-243J18.2	155679889	155680354	-1	antisense	NA	NA	NA	NA	0	0	0	NA	NA	NA	NA	Yes	GSE86189_GA.po.all.FUMA	0.000130157	1:156032887:A/G	4
ENSG00000116580	GON4L	155719508	155829191	-1	protein_coding	54856	GON4L	0.999998891638014	-0.637167859	0	0	0	NA	NA	NA	NA	Yes	GSE86189_GA.po.all.FUMA:GSE86189_GA.pp.all.FUMA	6.38828e-06	1:155033308:A/G:1:156032887:A/G	4
ENSG00000234937	RP11-101O6.2	155815158	155816415	1	pseudogene	NA	NA	NA	NA	0	0	0	NA	NA	NA	NA	Yes	GSE86189_GA.po.all.FUMA	0.000130157	1:156032887:A/G	4
ENSG00000132718	SYT11	155829300	155854990	1	protein_coding	23208	SYT11	0.801740134718319	-1.924430366	0	0	0	NA	NA	NA	NA	Yes	GSE86189_GA.po.all.FUMA:G	6.38828e-06	1:155033308:A/G:1:156032887:A/G	4

																		SE86189_GA.pp.all.FUMA			
ENSG00000201614	RNU4-19P	155864072	155864177	1	snRNA	NA	RNU4-19P	NA	NA	0	0	0	NA	NA	NA	NA	Yes	GSE86189_GA.po.all.FUMA	0.000130157	1:156032887:A/G	4
ENSG00000143622	RIT1	155867599	155881195	-1	protein_coding	6016	RIT1	0.666809792340233	-0.183927618	0	0	2	0.0247293	NA	GTEx/v8/Stomach	-	Yes	GSE86189_GA.po.all.FUMA:GSE86189_GA.pp.all.FUMA	6.38828e-06	1:156032887:A/G;1:155033308:A/G:1:156032887:A/G	4
ENSG00000132680	KIAA0907	155882834	155904191	-1	protein_coding	22889	KIAA0907	0.99711361843545	-0.510403212	0	0	0	NA	NA	NA	NA	Yes	GSE86189_GA.po.all.FUMA:GSE86189_GA.pp.all.FUMA	6.38828e-06	1:155033308:A/G:1:156032887:A/G	4
ENSG00000207475	SNORA42	155889700	155889836	-1	snoRNA	677823	SNORA42	NA	NA	0	0	0	NA	NA	NA	NA	Yes	GSE86189_GA.po.all.FUMA	0.000130157	1:156032887:A/G	4
ENSG00000252808	SCARNA4	155895749	155895877	-1	snoRNA	677771	SCARNA4	NA	NA	0	0	0	NA	NA	NA	NA	Yes	GSE86189_GA.po.all.FUMA	0.000130157	1:156032887:A/G	4
ENSG00000173080	RXFP4	155911480	155912625	1	protein_coding	339403	RXFP4	0.00549581832537321	-0.164025255	0	0	0	NA	NA	NA	NA	Yes	GSE86189_GA.po.all.FUMA:GSE86189_GA.pp.all.FUMA	6.38828e-06	1:155033308:A/G:1:156032887:A/G	4
ENSG00000116584	ARHGEF2	155916630	155976861	-1	protein_coding	9181	ARHGEF2	0.99999323549835	-0.492253581	0	0	2	0.0291653	NA	GTEx/v8/Stomach	-	Yes	GSE86189_GA.po.all.FUMA:GSE86189_GA.pp.all.FUMA	6.38828e-06	1:156032887:A/G;1:155033308:A/G:1:156032887:A/G	4
ENSG00000273002	RP11-336K24.12	155948590	155952777	1	antisense	NA	NA	NA	NA	0	0	0	NA	NA	NA	NA	Yes	GSE86189_GA.po.all.FUMA	0.000130157	1:156032887:A/G	4
ENSG00000224276	RP11-336K24.5	155961181	155971578	1	antisense	107985210	LOC107985210	NA	NA	0	0	0	NA	NA	NA	NA	Yes	GSE86189_GA.po.all.FUMA:GSE86189_GA.pp.all.FUMA	6.38828e-06	1:155033308:A/G:1:156032887:A/G	4
ENSG00000163479	SSR2	155978839	155990750	-1	protein_coding	6746	SSR2	0.822108921079836	-0.380458352	0	0	0	NA	NA	NA	NA	Yes	GSE86189_GA.po.all.FUMA:GSE86189_GA.pp.all.FUMA	6.38828e-06	1:155033308:A/G:1:156032887:A/G	4
ENSG00000160803	UBQLN4	156005092	156023585	-1	protein_coding	56893	UBQLN4	0.997522419654586	-0.671923208	1	15.326		0.000279085	NA	GTEx/v8/Stomach	-	Yes	GSE86189_GA.po.all.FUMA:GSE86189_GA.pp.all.FUMA	6.38828e-06	1:156032887:A/G;1:155033308:A/G:1:156032887:A/G	4
ENSG00000116586	LAMTOR2	156024543	156028301	1	protein_coding	28956	LAMTOR2	0.481086311682732	-0.350910656	1	15.316		0.0439126	NA	GTEx/v8/St	-	Yes	GSE86189_GA.po.all.FUMA:G	6.38828e-06	1:156032887:A/G;1:155033308:A/G:1:156032887:A/G	4

															om ach			SE86189_GA.pp.all.FUMA			
ENSG00000132698	RAB25	156030951	156040295	1	protein_coding	57111	RAB25	9.31079417686485e-06	-0.280558233	1	15.306		NA	NA	NA	NA	Yes	GSE86189_GA.po.all.FUMA:GSE86189_GA.pp.all.FUMA	6.38828e-06	1:156032887:A/G;1:155033308:A/G:1:156032887:A/G	4
ENSG00000254726	MEX3A	156041804	156051789	-1	protein_coding	92312	MEX3A	0.881462711195155	-0.805061324	1	15.306		NA	NA	NA	NA	Yes	GSE86189_GA.po.all.FUMA:GSE86189_GA.pp.all.FUMA	6.38828e-06	1:156032887:A/G;1:155033308:A/G:1:156032887:A/G	4
ENSG00000222611	AL355388.1	156047164	156047245	1	miRNA	NA	NA	NA	NA	1	15.306		NA	NA	NA	NA	Yes	GSE86189_GA.po.all.FUMA:GSE86189_GA.pp.all.FUMA	6.38828e-06	1:156032887:A/G;1:155033308:A/G:1:156032887:A/G	4
ENSG00000160789	LMNA	156052364	156109880	1	protein_coding	4000	LMNA	0.993449023376761	-0.151530173	1	15.306		NA	NA	NA	NA	Yes	GSE86189_GA.po.all.FUMA:GSE86189_GA.pp.all.FUMA	6.38828e-06	1:156032887:A/G;1:155033308:A/G:1:156032887:A/G	4
ENSG00000196189	SEMA4A	156117157	156147543	1	protein_coding	64218	SEMA4A	0.615371620982326	-0.205497495	0	0	0	NA	NA	NA	NA	Yes	GSE86189_GA.po.all.FUMA:GSE86189_GA.pp.all.FUMA	6.38828e-06	1:155033308:A/G:1:156032887:A/G	4
ENSG00000252236	SNORA26	156161854	156161994	-1	snoRNA	677810	SNORA26	NA	NA	0	0	0	NA	NA	NA	NA	Yes	GSE86189_GA.po.all.FUMA:GSE86189_GA.pp.all.FUMA	6.38828e-06	1:155033308:A/G:1:156032887:A/G	4
ENSG00000160785	SLC25A44	156163880	156182587	1	protein_coding	9673	SLC25A44	0.247810862226725	0.031341681	0	0	0	NA	NA	NA	NA	Yes	GSE86189_GA.po.all.FUMA:GSE86189_GA.pp.all.FUMA	6.38828e-06	1:155033308:A/G:1:156032887:A/G	4
ENSG00000260238	PMF1-BGLAP	156182773	156213123	1	protein_coding	100527963	PMF1-BGLAP	1.71367442696473e-05	-0.193896901	0	0	0	NA	NA	NA	NA	Yes	GSE86189_GA.po.all.FUMA:GSE86189_GA.pp.all.FUMA	6.38828e-06	1:155033308:A/G:1:156032887:A/G	4
ENSG00000160783	PMF1	156182784	156212874	1	protein_coding	11243	PMF1	1.71367442696473e-05	-0.116474655	0	0	0	NA	NA	NA	NA	Yes	GSE86189_GA.po.all.FUMA:GSE86189_GA.pp.all.FUMA	6.38828e-06	1:155033308:A/G:1:156032887:A/G	4
ENSG00000242252	BGLAP	156211753	156213112	1	protein_coding	632	BGLAP	1.75722490280257e-05	-0.06864469	0	0	0	NA	NA	NA	NA	Yes	GSE86189_GA.po.all.FUMA:GSE86189_GA.pp.all.FUMA	6.38828e-06	1:155033308:A/G:1:156032887:A/G	4
ENSG00000160781	PAQR6	156213206	156217881	-1	protein_coding	79957	PAQR6	0.000721604365184446	-0.290453061	0	0	0	NA	NA	NA	NA	Yes	GSE86189_GA.po.all.FUMA:G	6.38828e-06	1:156032887:A/G:1:155033308:A/G	4

																		SE86189_GA.pp.all.FUMA			
ENSG00000198952	SMG5	156219015	156252620	-1	protein_coding	23381	SMG5	0.998179752831908	-0.601125197	0	0	0	NA	NA	NA	NA	Yes	GSE86189_GA.po.all.FUMA:GSE86189_GA.pp.all.FUMA	6.38828e-06	1:155033308:A/G:1:156032887:A/G	4
ENSG00000163472	TMEM79	156252726	156262976	1	protein_coding	84283	TMEM79	3.49884123421551e-07	-0.533253936	0	0	0	NA	NA	NA	NA	Yes	GSE86189_GA.po.all.FUMA:GSE86189_GA.pp.all.FUMA	6.38828e-06	1:155033308:A/G:1:156032887:A/G	4
ENSG00000198715	C1orf85	156259880	156265463	-1	protein_coding	112770	C1orf85	4.68709760252752e-08	0.200003517	0	0	0	NA	NA	NA	NA	Yes	GSE86189_GA.po.all.FUMA:GSE86189_GA.pp.all.FUMA	6.38828e-06	1:155033308:A/G:1:156032887:A/G	4
ENSG00000189030	VHLL	156268415	156269428	-1	protein_coding	391104	VHLL	0.00290057346415176	NA	0	0	0	NA	NA	NA	NA	Yes	GSE86189_GA.po.all.FUMA:GSE86189_GA.pp.all.FUMA	6.38828e-06	1:155033308:A/G:1:156032887:A/G	4
ENSG00000163468	CCT3	156278759	156337664	-1	protein_coding	7203	CCT3	0.998745412971341	-0.354171565	0	0	0	NA	NA	NA	NA	Yes	GSE86189_GA.po.all.FUMA:GSE86189_GA.pp.all.FUMA	6.38828e-06	1:155033308:A/G:1:156032887:A/G	4
ENSG00000228155	AL589685.1	156293302	156293487	1	pseudogene	NA	NA	NA	NA	0	0	0	NA	NA	NA	NA	Yes	GSE86189_GA.po.all.FUMA	0.000130157	1:156032887:A/G	4
ENSG00000163467	TSACC	156307105	156316786	1	protein_coding	128229	TSACC	0.0697917263737303	-0.097382218	0	0	0	NA	NA	NA	NA	Yes	GSE86189_GA.po.all.FUMA:GSE86189_GA.pp.all.FUMA	6.38828e-06	1:155033308:A/G:1:156032887:A/G	4
ENSG00000132677	RHBG	156339003	156355011	1	protein_coding	57127	RHBG	3.72564357556274e-10	NA	0	0	0	NA	NA	NA	NA	Yes	GSE86189_GA.po.all.FUMA:GSE86189_GA.pp.all.FUMA	6.38828e-06	1:155033308:A/G:1:156032887:A/G	4
ENSG00000237390	RP11-139I14.2	156358018	156365401	1	lincRNA	NA	NA	NA	NA	0	0	0	NA	NA	NA	NA	Yes	GSE86189_GA.po.all.FUMA	0.000130157	1:156032887:A/G	4
ENSG00000125462	C1orf61	156374042	156400493	-1	protein_coding	10485	C1orf61	0.104422489726842	-0.188055329	0	0	0	NA	NA	NA	NA	Yes	GSE86189_GA.po.all.FUMA:GSE86189_GA.pp.all.FUMA	6.38828e-06	1:155033308:A/G:1:156032887:A/G	4
ENSG00000207933	MIR9-1	156390123	156390230	1	miRNA	407046	MIR9-1	NA	NA	0	0	0	NA	NA	NA	NA	Yes	GSE86189_GA.po.all.FUMA:GSE86189_GA.pp.all.FUMA	6.38828e-06	1:155033308:A/G:1:156032887:A/G	4
ENSG00000236947	RP11-98G7.1	156416204	156426369	-1	lincRNA	NA	NA	NA	NA	0	0	0	NA	NA	NA	NA	Yes	GSE86189_GA.po.all.FUMA	0.000130157	1:156032887:A/G	4

ENSG00000116604	MEF2D	156433519	156470620	-1	protein_coding	4209	MEF2D	0.998547093289291	-1.450366264	0	0	0	NA	NA	NA	NA	Yes	GSE86189_GA.po.all.FUMA:GSE86189_GA.pp.all.FUMA	6.38828e-06	1:155033308:A/G:1:156032887:A/G	4
ENSG00000206651	Y_RNA	156453890	156454002	-1	misc_RNA	NA	NA	NA	NA	0	0	0	NA	NA	NA	NA	Yes	GSE86189_GA.po.all.FUMA	0.000130157	1:156032887:A/G	4
ENSG00000260460	RP11-284F21.8	156479646	156481473	1	lincRNA	NA	NA	NA	NA	0	0	0	NA	NA	NA	NA	Yes	GSE86189_GA.po.all.FUMA:GSE86189_GA.pp.all.FUMA	6.38828e-06	1:155033308:A/G:1:156032887:A/G	4
ENSG00000183856	IQGAP3	156495197	156542396	-1	protein_coding	128239	IQGAP3	6.47051186060707e-16	1.011463836	0	0	0	NA	NA	NA	NA	Yes	GSE86189_GA.po.all.FUMA:GSE86189_GA.pp.all.FUMA	6.38828e-06	1:155033308:A/G:1:156032887:A/G	4
ENSG00000238843	snoU13	156499122	156499221	1	snoRNA	NA	NA	NA	NA	0	0	0	NA	NA	NA	NA	Yes	GSE86189_GA.po.all.FUMA	0.000130157	1:156032887:A/G	4
ENSG00000187862	TTC24	156549519	156556562	1	protein_coding	164118	TTC24	6.58039647151808e-05	0.114085676	0	0	0	NA	NA	NA	NA	Yes	GSE86189_GA.po.all.FUMA:GSE86189_GA.pp.all.FUMA	6.38828e-06	1:155033308:A/G:1:156032887:A/G	4
ENSG00000265960	AL365181.1	156557648	156557747	-1	miRNA	NA	NA	NA	NA	0	0	0	NA	NA	NA	NA	Yes	GSE86189_GA.po.all.FUMA:GSE86189_GA.pp.all.FUMA	6.38828e-06	1:155033308:A/G:1:156032887:A/G	4
ENSG00000163382	APOA1BP	156561554	156564091	1	protein_coding	128240	APOA1BP	1.72708938017144e-06	0.413152862	0	0	0	NA	NA	NA	NA	Yes	GSE86189_GA.po.all.FUMA:GSE86189_GA.pp.all.FUMA	6.38828e-06	1:155033308:A/G:1:156032887:A/G	4
ENSG00000160818	GPATCH4	156564279	156571288	-1	protein_coding	54865	GPATCH4	1.68775621140413e-07	0.151806796	0	0	0	NA	NA	NA	NA	Yes	GSE86189_GA.po.all.FUMA:GSE86189_GA.pp.all.FUMA	6.38828e-06	1:155033308:A/G:1:156032887:A/G	4
ENSG00000272971	RP11-284F21.11	156584534	156585080	-1	lincRNA	NA	NA	NA	NA	0	0	0	NA	NA	NA	NA	Yes	GSE86189_GA.po.all.FUMA:GSE86189_GA.pp.all.FUMA	6.38828e-06	1:155033308:A/G:1:156032887:A/G	4
ENSG00000132702	HAPLN2	156589086	156595517	1	protein_coding	60484	HAPLN2	1.05244127720139e-05	-0.469464157	0	0	0	NA	NA	NA	NA	Yes	GSE86189_GA.po.all.FUMA:GSE86189_GA.pp.all.FUMA	6.38828e-06	1:155033308:A/G:1:156032887:A/G	4
ENSG00000272068	RP11-284F21.9	156607575	156610796	-1	lincRNA	NA	NA	NA	NA	0	0	0	NA	NA	NA	NA	Yes	GSE86189_GA.po.all.FUMA:GSE86189_GA.pp.all.FUMA	6.38828e-06	1:155033308:A/G:1:156032887:A/G	4

ENSG00000132692	BCAN	156611182	156629324	1	protein_coding	63827	BCAN	0.00361212462845155	NA	0	0	0	NA	NA	NA	NA	Yes	GSE86189_GA.po.all.FUMA:GSE86189_GA.pp.all.FUMA	6.38828e-06	1:155033308:A/G:1:156032887:A/G	4
ENSG00000272405	RP11-284F21.10	156611458	156614679	-1	antisense	NA	NA	NA	NA	0	0	0	NA	NA	NA	NA	Yes	GSE86189_GA.po.all.FUMA:GSE86189_GA.pp.all.FUMA	6.38828e-06	1:155033308:A/G:1:156032887:A/G	4
ENSG00000229953	RP11-284F21.7	156616299	156631216	-1	antisense	NA	NA	NA	NA	0	0	0	NA	NA	NA	NA	Yes	GSE86189_GA.po.all.FUMA:GSE86189_GA.pp.all.FUMA	6.38828e-06	1:155033308:A/G:1:156032887:A/G	4
ENSG00000132688	NES	156638555	156647189	-1	protein_coding	10763	NES	2.06955630735523e-05	-0.507845132	0	0	0	NA	NA	NA	NA	Yes	GSE86189_GA.po.all.FUMA:GSE86189_GA.pp.all.FUMA	6.38828e-06	1:155033308:A/G:1:156032887:A/G	4
ENSG00000237588	RP11-66D17.3	156657487	156661789	-1	lincRNA	NA	NA	NA	NA	0	0	0	NA	NA	NA	NA	Yes	GSE86189_GA.po.all.FUMA	0.000130157	1:156032887:A/G	4
ENSG00000143320	CRABP2	156669398	156675608	-1	protein_coding	1382	CRABP2	0.00816686942900527	-0.463429953	0	0	0	NA	NA	NA	NA	Yes	GSE86189_GA.po.all.FUMA:GSE86189_GA.pp.all.FUMA	6.38828e-06	1:155033308:A/G:1:156032887:A/G	4
ENSG00000223356	RP11-66D17.5	156682004	156682966	-1	antisense	NA	NA	NA	NA	0	0	0	NA	NA	NA	NA	Yes	GSE86189_GA.po.all.FUMA:GSE86189_GA.pp.all.FUMA	6.38828e-06	1:155033308:A/G:1:156032887:A/G	4
ENSG00000143319	ISG20L2	156691683	156698591	-1	protein_coding	81875	ISG20L2	0.821397244338996	-0.720864737	0	0	0	NA	NA	NA	NA	Yes	GSE86189_GA.po.all.FUMA:GSE86189_GA.pp.all.FUMA	6.38828e-06	1:155033308:A/G:1:156032887:A/G	4
ENSG00000143303	RRNAD1	156698234	156706752	1	protein_coding	51093	RRNAD1	2.11535175142641e-08	-0.437664169	0	0	0	NA	NA	NA	NA	Yes	GSE86189_GA.po.all.FUMA:GSE86189_GA.pp.all.FUMA	6.38828e-06	1:155033308:A/G:1:156032887:A/G	4
ENSG00000143314	MRPL24	156707095	156711382	-1	protein_coding	79590	MRPL24	0.00149122661138067	-0.055703152	0	0	0	NA	NA	NA	NA	Yes	GSE86189_GA.po.all.FUMA:GSE86189_GA.pp.all.FUMA	6.38828e-06	1:156032887:A/G:1:155033308:A/G	4
ENSG00000143321	HDGF	156711899	156736717	-1	protein_coding	3068	HDGF	0.269699273955011	0.146888659	0	0	0	NA	NA	NA	NA	Yes	GSE86189_GA.po.all.FUMA:GSE86189_GA.pp.all.FUMA	6.38828e-06	1:155033308:A/G:1:156032887:A/G	4
ENSG00000143294	PRCC	156720402	156770607	1	protein_coding	5546	PRCC	0.921293121521907	-0.644689348	0	0	0	NA	NA	NA	NA	Yes	GSE86189_GA.po.all.FUMA:G	6.38828e-06	1:155033308:A/G:1:156032887:A/G	4

																		SE86189_GA.pp.all.FUMA			
ENSG00000027869	SH2D2A	156776035	156786654	-1	protein_coding	9047	SH2D2A	8.43213730306944e-05	0.607906002	0	0	0	NA	NA	NA	NA	Yes	GSE86189_GA.po.all.FUMA:GSE86189_GA.pp.all.FUMA	6.38828e-06	1:155033308:A/G:1:156032887:A/G	4
ENSG00000198400	NTRK1	156785432	156851642	1	protein_coding	4914	NTRK1	0.000424899217244118	NA	0	0	2	0.0114783	NA	GTEx/v8/Stomach	+	Yes	GSE86189_GA.po.all.FUMA:GSE86189_GA.pp.all.FUMA	6.38828e-06	1:156032887:A/G;1:155033308:A/G:1:156032887:A/G	4
ENSG00000027644	INSRR	156809855	156828810	-1	protein_coding	3645	INSRR	4.91565760465697e-07	-0.13632802	0	0	0	NA	NA	NA	NA	Yes	GSE86189_GA.po.all.FUMA:GSE86189_GA.pp.all.FUMA	6.38828e-06	1:155033308:A/G:1:156032887:A/G	4
ENSG00000187800	PEAR1	156863490	156886226	1	protein_coding	375033	PEAR1	1.18896498529681e-14	0.034106255	0	0	0	NA	NA	NA	NA	Yes	GSE86189_GA.po.all.FUMA:GSE86189_GA.pp.all.FUMA	6.38828e-06	1:155033308:A/G:1:156032887:A/G	4
ENSG00000160838	LRRC71	156890442	156902886	1	protein_coding	149499	LRRC71	0.00904921773201473	-0.642421626	0	0	0	NA	NA	NA	NA	Yes	GSE86189_GA.po.all.FUMA:GSE86189_GA.pp.all.FUMA	6.38828e-06	1:155033308:A/G:1:156032887:A/G	4
ENSG00000132694	ARHGEF11	156904632	157015162	-1	protein_coding	9826	ARHGEF11	0.999945339206174	0.702353149	0	0	0	NA	NA	NA	NA	Yes	GSE86189_GA.po.all.FUMA:GSE86189_GA.pp.all.FUMA	6.38828e-06	1:155033308:A/G:1:156032887:A/G	4
ENSG00000211581	MIR765	156905923	156906036	-1	miRNA	768220	MIR765	NA	NA	0	0	0	NA	NA	NA	NA	Yes	GSE86189_GA.po.all.FUMA:GSE86189_GA.pp.all.FUMA	6.38828e-06	1:155033308:A/G:1:156032887:A/G	4
ENSG00000266160	RN7SL612P	156955549	156955841	-1	misc_RNA	106479445	RN7SL612P	NA	NA	0	0	0	NA	NA	NA	NA	Yes	GSE86189_GA.po.all.FUMA	0.000130157	1:156032887:A/G	4
ENSG00000237842	RP11-110J1.2	157029024	157030554	-1	pseudogene	NA	NA	NA	NA	0	0	0	NA	NA	NA	NA	Yes	GSE86189_GA.po.all.FUMA:GSE86189_GA.pp.all.FUMA	6.38828e-06	1:156032887:A/G:1:155033308:A/G	4
ENSG00000224520	KRT8P45	157043049	157044495	1	pseudogene	149501	KRT8P45	NA	NA	0	0	0	NA	NA	NA	NA	Yes	GSE86189_GA.po.all.FUMA	0.000130157	1:156032887:A/G	4
ENSG00000253831	ETV3L	157061836	157069600	-1	protein_coding	440695	ETV3L	1.20827466358355e-05	1.282266072	0	0	0	NA	NA	NA	NA	Yes	GSE86189_GA.pp.all.FUMA	0.000130157	1:156032887:A/G	4
ENSG00000117036	ETV3	157090983	157108266	-1	protein_coding	2117	ETV3	0.783799556047856	-1.58224079	0	0	0	NA	NA	NA	NA	Yes	GSE86189_GA.pp.all.FUMA	0.000130157	1:156032887:A/G	4

ENSG00000235700	CYCSP52	157098154	157098463	1	pseudogene	360155	CYCSP52	NA	NA	0	0	0	NA	NA	NA	NA	Yes	GSE86189_GA.po.all.FUMA	0.000130157	1:156032887:A/G	4
ENSG00000229961	RP11-71G12.1	157155178	157157195	1	lincRNA	NA	NA	NA	NA	0	0	0	NA	NA	NA	NA	Yes	GSE86189_GA.po.all.FUMA	0.000130157	1:156032887:A/G	4
ENSG00000228239	RP11-85G21.1	157202021	157206926	-1	lincRNA	NA	NA	NA	NA	0	0	0	NA	NA	NA	NA	Yes	GSE86189_GA.po.all.FUMA	0.000130157	1:156032887:A/G	4
ENSG00000271736	RP11-85G21.3	157250506	157252858	1	lincRNA	NA	NA	NA	NA	0	0	0	NA	NA	NA	NA	Yes	GSE86189_GA.po.all.FUMA	0.000130157	1:156032887:A/G	4
ENSG00000237189	RP11-85G21.2	157257493	157257843	-1	lincRNA	NA	NA	NA	NA	0	0	0	NA	NA	NA	NA	Yes	GSE86189_GA.po.all.FUMA	0.000130157	1:156032887:A/G	4
ENSG00000143297	FCRL5	157483167	157522310	-1	protein_coding	83416	FCRL5	7.49244691962338e-14	3.931017951	0	0	0	NA	NA	NA	NA	Yes	GSE86189_GA.pp.all.FUMA	0.000130157	1:156032887:A/G	4
ENSG00000163518	FCRL4	157543539	157567870	-1	protein_coding	83417	FCRL4	1.59511072407105e-07	0.664405006	0	0	0	NA	NA	NA	NA	Yes	GSE86189_GA.pp.all.FUMA	0.000130157	1:156032887:A/G	4
ENSG00000236731	RP4-801G22.2	157599729	157600518	-1	pseudogene	NA	NA	NA	NA	0	0	0	NA	NA	NA	NA	Yes	GSE86189_GA.po.all.FUMA	0.000130157	1:156032887:A/G	4
ENSG00000233712	RP4-801G22.3	157606090	157610091	-1	pseudogene	NA	NA	NA	NA	0	0	0	NA	NA	NA	NA	Yes	GSE86189_GA.po.all.FUMA	0.000130157	1:156032887:A/G	4
ENSG00000160856	FCRL3	157644111	157670647	-1	protein_coding	115352	FCRL3	4.5604025041299e-18	1.538707152	0	0	0	NA	NA	NA	NA	Yes	GSE86189_GA.pp.all.FUMA	0.000130157	1:156032887:A/G	4
ENSG00000227217	RP11-367J7.3	157661552	157666249	1	antisense	NA	NA	NA	NA	0	0	0	NA	NA	NA	NA	Yes	GSE86189_GA.po.all.FUMA	0.000130157	1:156032887:A/G	4
ENSG00000231700	RP11-367J7.4	157678876	157679433	1	pseudogene	NA	NA	NA	NA	0	0	0	NA	NA	NA	NA	Yes	GSE86189_GA.po.all.FUMA	0.000130157	1:156032887:A/G	4
ENSG00000232366	VDAC1P9	157693970	157694810	-1	pseudogene	391106	VDAC1P9	NA	NA	0	0	0	NA	NA	NA	NA	Yes	GSE86189_GA.po.all.FUMA	0.000130157	1:156032887:A/G	4
ENSG00000132704	FCRL2	157715523	157746922	-1	protein_coding	79368	FCRL2	0.000160040383245631	1.436639631	0	0	0	NA	NA	NA	NA	Yes	GSE86189_GA.pp.all.FUMA	0.000130157	1:156032887:A/G	4
ENSG00000163534	FCRL1	157764193	157789895	-1	protein_coding	115350	FCRL1	0.00159526703104064	0.138953155	0	0	0	NA	NA	NA	NA	Yes	GSE86189_GA.pp.all.FUMA	0.000130157	1:156032887:A/G	4
ENSG00000073754	CD5L	157800704	157868046	-1	protein_coding	922	CD5L	7.09602208888946e-07	0.036599563	0	0	0	NA	NA	NA	NA	Yes	GSE86189_GA.pp.all.FUMA	0.000130157	1:156032887:A/G	4
ENSG00000227425	MRPS21P2	157830987	157831250	1	pseudogene	359766	MRPS21P2	NA	NA	0	0	0	NA	NA	NA	NA	Yes	GSE86189_GA.po.all.FUMA	0.000130157	1:156032887:A/G	4
ENSG00000236957	RP11-451O13.1	157894855	157918861	1	pseudogene	105371458	LOC105371458	NA	NA	0	0	0	NA	NA	NA	NA	Yes	GSE86189_GA.po.all.FUMA	0.000130157	1:156032887:A/G	4
ENSG00000183853	KIRREL	157963063	158070052	1	protein_coding	55243	KIRREL	0.95946008263139	NA	0	0	0	NA	NA	NA	NA	Yes	GSE86189_GA.pp.all.FUMA	0.000130157	1:156032887:A/G	4
ENSG00000226520	KIRREL-IT1	157995340	158000956	1	sense_intronic	100505785	KIRREL-IT1	NA	NA	0	0	0	NA	NA	NA	NA	Yes	GSE86189_GA.po.all.FUMA	0.000130157	1:156032887:A/G	4
ENSG00000177822	AC108142.1	182795591	183066402	-1	antisense	NA	NA	NA	NA	0	0	0	NA	NA	NA	NA	Yes	GSE86189_GA.po.all.FUMA	0.00186532	4:183877690:A/C;4:183885485:A/G	25
ENSG00000218336	TENM3	183065140	183724177	1	protein_coding	55714	TENM3	0.999937943986087	0.558316153	0	0	0	NA	NA	NA	NA	Yes	GSE86189_GA.po.all.FUMA	0.00186532	4:183877690:A/C;4:183885485:A/G	25

ENSG00000248266	RP11-402C9.1	183066005	183111535	1	sense_intronic	NA	NA	NA	NA	0	0	0	NA	NA	NA	NA	Yes	GSE86189_GA.po.all.FUMA	0.00186532	4:183877690:A/C;4:183885485:A/G	25
ENSG00000272646	RP11-188P17.2	183802930	183803356	-1	lincRNA	NA	NA	NA	NA	1	16.502		NA	NA	NA	NA	No	NA	0.00492731	4:183877690:A/C;4:183885485:A/G	25
ENSG00000129187	DCTD	183811213	183839089	-1	protein_coding	1635	DCTD	1.63560551829744e-05	0.821915515	2	16.532		0.00271944	NA	GTEx/v8/Stomach	-	Yes	GSE86189_GA.po.all.FUMA	0.00186532	4:183877690:A/C;4:183885485:A/G	25
ENSG00000251359	WWC2-AS2	184018170	184020352	-1	lincRNA	152641	WWC2-AS2	NA	NA	0	0	0	NA	NA	NA	NA	Yes	GSE86189_GA.po.all.FUMA	0.00186532	4:183877690:A/C;4:183885485:A/G	25
ENSG00000151718	WWC2	184020446	184241930	1	protein_coding	80014	WWC2	2.48112813993575e-05	-1.369884799	0	0	0	NA	NA	NA	NA	Yes	GSE86189_GA.po.all.FUMA	0.00186532	4:183877690:A/C;4:183885485:A/G	25
ENSG00000177300	CLDN22	184239220	184241927	-1	protein_coding	53842	CLDN22	0.000437370884697617	-0.866283131	0	0	0	NA	NA	NA	NA	Yes	GSE86189_GA.po.all.FUMA	0.00186532	4:183877690:A/C;4:183885485:A/G	25
ENSG00000185758	CLDN24	184242917	184243579	-1	protein_coding	100132463	CLDN24	0.000259988316035725	-0.152545374	0	0	0	NA	NA	NA	NA	Yes	GSE86189_GA.po.all.FUMA	0.00186532	4:183877690:A/C;4:183885485:A/G	25
ENSG00000168564	CDKN2AIP	184365744	184369351	1	protein_coding	55602	CDKN2AIP	0.987251415538184	0.013970214	0	0	0	NA	NA	NA	NA	Yes	GSE86189_GA.po.all.FUMA	0.00186532	4:183877690:A/C;4:183885485:A/G	25
ENSG00000232648	RP11-367N14.2	184416091	184425647	-1	antisense	NA	NA	NA	NA	0	0	0	NA	NA	NA	NA	Yes	GSE86189_GA.po.all.FUMA	0.00186532	4:183877690:A/C;4:183885485:A/G	25
ENSG00000168556	ING2	184426147	184432249	1	protein_coding	3622	ING2	0.283379994488456	NA	0	0	0	NA	NA	NA	NA	Yes	GSE86189_GA.po.all.FUMA	0.00186532	4:183877690:A/C;4:183885485:A/G	25
ENSG00000182552	RWDD4	184560788	184580378	-1	protein_coding	201965	RWDD4	0.482983522176144	-0.574116963	0	0	0	NA	NA	NA	NA	Yes	GSE86189_GA.po.all.FUMA	0.00186532	4:183877690:A/C;4:183885485:A/G	25
ENSG00000168538	TRAPPC11	184580420	184634745	1	protein_coding	60684	TRAPPC11	0.000208772147312059	-0.321007447	0	0	0	NA	NA	NA	NA	Yes	GSE86189_GA.po.all.FUMA	0.00186532	4:183877690:A/C;4:183885485:A/G	25
ENSG00000173320	STOX2	184774584	184944679	1	protein_coding	56977	STOX2	0.96321644241095	1.870838676	0	0	0	NA	NA	NA	NA	Yes	GSE86189_GA.po.all.FUMA	0.00186532	4:183877690:A/C;4:183885485:A/G	25
ENSG00000164303	ENPP6	185009859	185142383	-1	protein_coding	133121	ENPP6	6.11082610084163e-07	-0.543460526	0	0	0	NA	NA	NA	NA	Yes	GSE86189_GA.po.all.FUMA	0.00186532	4:183877690:A/C;4:183885485:A/G	25
ENSG00000168310	IRF2	185308867	185395734	-1	protein_coding	3660	IRF2	0.382047181106152	-0.790691891	0	0	0	NA	NA	NA	NA	Yes	GSE86189_GA.po.all.FUMA	0.00186532	4:183877690:A/C;4:183885485:A/G	25
ENSG00000270426	RP11-326I11.5	185393078	185393763	-1	sense_intronic	NA	NA	NA	NA	0	0	0	NA	NA	NA	NA	Yes	GSE86189_GA.po.all.FUMA	0.00186532	4:183877690:A/C;4:183885485:A/G	25
ENSG00000271646	RP11-326I11.3	185395956	185398458	1	lincRNA	NA	NA	NA	NA	0	0	0	NA	NA	NA	NA	Yes	GSE86189_GA.po.all.FUMA	0.00186532	4:183877690:A/C;4:183885485:A/G	25
ENSG00000164305	CASP3	185548850	185570663	-1	protein_coding	836	CASP3	0.184808404916883	0.110986029	0	0	0	NA	NA	NA	NA	Yes	GSE86189_GA.po.all.FUMA	0.00186532	4:183877690:A/C;4:183885485:A/G	25
ENSG00000164306	PRIMPOL	185570767	185616117	1	protein_coding	201973	PRIMPOL	2.78745619908396e-13	NA	0	0	0	NA	NA	NA	NA	Yes	GSE86189_GA.po.all.FUMA	0.00186532	4:183877690:A/C;4:183885485:A/G	25
ENSG00000151726	ACSL1	185676749	185747972	-1	protein_coding	2180	ACSL1	0.0918373313295172	-0.017894833	0	0	0	NA	NA	NA	NA	Yes	GSE86189_GA.po.all.FUMA	0.00186532	4:183877690:A/C;4:183885485:A/G	25

ENSG00000251139	RP11-701P16.2	185734773	185742443	1	protein_coding	NA	NA	NA	NA	0	0	0	NA	NA	NA	NA	Yes	GSE86189_GA.po.all.FUMA	0.00186532	4:183877690:A/C;4:183885485:A/G	25
ENSG00000249740	CTD-2127H9.1	38736157	38845931	-1	lincRNA	101926904	LINC01265	NA	NA	0	0	0	NA	NA	NA	NA	Yes	GSE86189_GA.po.all.FUMA:GSE86189_GA.pp.all.FUMA	1.92234e-06	5:40704074:A/G:5:40812231:A/G	27
ENSG00000145623	OSMR	38845960	38945698	1	protein_coding	9180	OSMR	3.10306262078619e-11	-0.176500312	0	0	0	NA	NA	NA	NA	Yes	GSE86189_GA.po.all.FUMA:GSE86189_GA.pp.all.FUMA	1.92234e-06	5:40704074:A/G:5:40812231:A/G	27
ENSG00000164327	RICTOR	38938021	39074510	-1	protein_coding	253260	RICTOR	0.999999999869293	-0.167173978	0	0	0	NA	NA	NA	NA	Yes	GSE86189_GA.po.all.FUMA:GSE86189_GA.pp.all.FUMA	1.92234e-06	5:40704074:A/G:5:40812231:A/G	27
ENSG00000249089	AIG1P1	39022513	39022660	1	pseudogene	106479028	AIG1P1	NA	NA	0	0	0	NA	NA	NA	NA	Yes	GSE86189_GA.po.all.FUMA	1.92234e-06	5:40812231:A/G	27
ENSG00000082074	FYB	39105338	39274630	-1	protein_coding	2533	FYB	0.792005858585418	0.193923315	0	0	0	NA	NA	NA	NA	Yes	GSE86189_GA.po.all.FUMA:GSE86189_GA.pp.all.FUMA	1.92234e-06	5:40704074:A/G:5:40812231:A/G	27
ENSG00000269215	AC008964.1	39105358	39107350	1	protein_coding	NA	NA	NA	NA	0	0	0	NA	NA	NA	NA	Yes	GSE86189_GA.po.all.FUMA:GSE86189_GA.pp.all.FUMA	1.92234e-06	5:40704074:A/G:5:40812231:A/G	27
ENSG00000251215	GOLGA5P1	39169312	39170437	-1	pseudogene	100418736	GOLGA5P1	NA	NA	0	0	0	NA	NA	NA	NA	Yes	GSE86189_GA.po.all.FUMA	1.92234e-06	5:40812231:A/G	27
ENSG00000113600	C9	39284364	39424970	-1	protein_coding	735	C9	6.82499306595342e-17	-0.141308955	0	0	0	NA	NA	NA	NA	Yes	GSE86189_GA.po.all.FUMA:GSE86189_GA.pp.all.FUMA	1.92234e-06	5:40704074:A/G:5:40812231:A/G	27
ENSG00000153071	DAB2	39371780	39462402	-1	protein_coding	1601	DAB2	0.980417113899471	0.197521272	0	0	0	NA	NA	NA	NA	Yes	GSE86189_GA.po.all.FUMA:GSE86189_GA.pp.all.FUMA	1.92234e-06	5:40704074:A/G:5:40812231:A/G	27
ENSG00000271334	CTD-2078B5.2	39520533	39524810	1	lincRNA	101926940	LINC02104	NA	NA	0	0	0	NA	NA	NA	NA	Yes	GSE86189_GA.po.all.FUMA	1.92234e-06	5:40812231:A/G	27
ENSG00000251515	CCDC11P1	39571580	39572741	-1	pseudogene	100129040	CCDC11P1	NA	NA	0	0	0	NA	NA	NA	NA	Yes	GSE86189_GA.po.all.FUMA	1.92234e-06	5:40812231:A/G	27
ENSG00000250492	INTS6P1	39719086	39721615	-1	pseudogene	285634	INTS6P1	NA	NA	0	0	0	NA	NA	NA	NA	Yes	GSE86189_GA.po.all.FUMA	1.92234e-06	5:40812231:A/G	27
ENSG00000248651	GCSHP1	39888678	39889161	-1	pseudogene	106480441	GCSHP1	NA	NA	0	0	0	NA	NA	NA	NA	Yes	GSE86189_GA.po.all.FUMA	1.92234e-06	5:40812231:A/G	27
ENSG00000250048	LINC00603	40052393	40053426	1	lincRNA	102467077	LINC00603	NA	NA	0	0	0	NA	NA	NA	NA	Yes	GSE86189_GA.po.all.FUMA	1.92234e-06	5:40812231:A/G	27

ENSG00000249668	KRT18P56	40066753	40067302	1	pseudogene	106479027	KRT18P56	NA	NA	0	0	0	NA	NA	NA	NA	Yes	GSE86189_GA.po.all.FUMA	1.92234e-06	5:40812231:A/G	27
ENSG00000250585	LINC00604	40260759	40267759	1	lincRNA	106144578	LINC00604	NA	NA	0	0	0	NA	NA	NA	NA	Yes	GSE86189_GA.po.all.FUMA	1.92234e-06	5:40812231:A/G	27
ENSG00000199361	RNU1-150P	40269660	40269821	1	snRNA	106480413	RNU1-150P	NA	NA	0	0	0	NA	NA	NA	NA	Yes	GSE86189_GA.po.all.FUMA	1.92234e-06	5:40812231:A/G	27
ENSG00000265615	AC108105.1	40320420	40320517	1	miRNA	NA	NA	NA	NA	0	0	0	NA	NA	NA	NA	Yes	GSE86189_GA.po.all.FUMA	1.92234e-06	5:40812231:A/G	27
ENSG00000199552	SNORA63	40655065	40655196	1	snoRNA	6043	SNORA63	NA	NA	0	0	0	NA	NA	NA	NA	Yes	GSE86189_GA.po.all.FUMA	1.92234e-06	5:40812231:A/G	27
ENSG00000171522	PTGER4	40679600	40693837	1	protein_coding	5734	PTGER4	0.896912956538018	-1.342868943	1	13.536		0.000121293	NA	GTEx/v8/Stomach	+	Yes	GSE86189_GA.po.all.FUMA:GSE86189_GA.pp.all.FUMA	1.92234e-06	5:40704074:A/G;5:40812231:A/G;5:40704074:A/G:5:40812231:A/G	27
ENSG00000113638	TTC33	40714577	40756077	-1	protein_coding	23548	TTC33	0.0226185448110003	-0.592575381	1	13.506		NA	NA	NA	NA	Yes	GSE86189_GA.po.all.FUMA:GSE86189_GA.pp.all.FUMA	1.92234e-06	5:40704074:A/G;5:40704074:A/G:5:40812231:A/G	27
ENSG00000132356	PRKAA1	40759481	40798476	-1	protein_coding	5562	PRKAA1	0.0351010343853684	NA	1	13.53		0.000161646	NA	GTEx/v8/Stomach	-	Yes	GSE86189_GA.po.all.FUMA:GSE86189_GA.pp.all.FUMA	1.92234e-06	5:40812231:A/G;5:40704074:A/G;5:40704074:A/G:5:40812231:A/G	27
ENSG00000212567	SNORA57	40790179	40790306	-1	snoRNA	692158	SNORA57	NA	NA	1	13.50		NA	NA	NA	NA	Yes	GSE86189_GA.po.all.FUMA	1.92234e-06	5:40812231:A/G	27
ENSG00000145592	RPL37	40825364	40835437	-1	protein_coding	6167	RPL37	0.577671288406045	-0.429460046	1	13.50		NA	NA	NA	NA	Yes	GSE86189_GA.po.all.FUMA:GSE86189_GA.pp.all.FUMA	1.92234e-06	5:40812231:A/G;5:40704074:A/G:5:40812231:A/G	27
ENSG00000212296	SNORD72	40832758	40832837	-1	snoRNA	619564	SNORD72	NA	NA	0	0	0	NA	NA	NA	NA	Yes	GSE86189_GA.po.all.FUMA	1.92234e-06	5:40812231:A/G	27
ENSG00000132357	CARD6	40841286	40860275	1	protein_coding	84674	CARD6	1.45631048490053e-16	0.23529618	0	0	0	NA	NA	NA	NA	Yes	GSE86189_GA.po.all.FUMA:GSE86189_GA.pp.all.FUMA	1.92234e-06	5:40704074:A/G:5:40812231:A/G	27
ENSG00000112936	C7	40909354	40983041	1	protein_coding	730	C7	1.26145650921317e-11	1.602410483	0	0	0	NA	NA	NA	NA	Yes	GSE86189_GA.po.all.FUMA:GSE86189_GA.pp.all.FUMA	1.92234e-06	5:40704074:A/G:5:40812231:A/G	27
ENSG00000253098	RNU7-161P	40911169	40911230	-1	snRNA	106480452	RNU7-161P	NA	NA	0	0	0	NA	NA	NA	NA	Yes	GSE86189_GA.po.all.FUMA:GSE86189_GA.pp.all.FUMA	1.92234e-06	5:40704074:A/G:5:40812231:A/G	27

ENSG00000248120	RP11-301A5.2	40967607	40967865	-1	pseudogene	NA	NA	NA	NA	0	0	0	NA	NA	NA	NA	Yes	GSE86189_GA.po.all.FUMA	1.92234e-06	5:40812231:A/G	27
ENSG00000171495	MROH2B	40998119	41071444	-1	protein_coding	133558	MROH2B	1.28868466121395e-26	-0.161162542	0	0	0	NA	NA	NA	NA	Yes	GSE86189_GA.po.all.FUMA:GSE86189_GA.pp.all.FUMA	1.92234e-06	5:40704074:A/G:5:40812231:A/G	27
ENSG00000039537	C6	41142336	41261540	-1	protein_coding	729	C6	1.2016606841251e-14	0.654854493	0	0	0	NA	NA	NA	NA	Yes	GSE86189_GA.po.all.FUMA:GSE86189_GA.pp.all.FUMA	1.92234e-06	5:40704074:A/G:5:40812231:A/G	27
ENSG00000182836	PLCXD3	41307056	41510730	-1	protein_coding	345557	PLCXD3	0.0484887392356897	-0.319158709	0	0	0	NA	NA	NA	NA	Yes	GSE86189_GA.po.all.FUMA:GSE86189_GA.pp.all.FUMA	1.92234e-06	5:40704074:A/G:5:40812231:A/G	27
ENSG00000251478	CTD-2122P11.1	41585984	41587889	-1	pseudogene	NA	NA	NA	NA	0	0	0	NA	NA	NA	NA	Yes	GSE86189_GA.po.all.FUMA	1.92234e-06	5:40812231:A/G	27
ENSG00000083720	OXCT1	41730167	41870621	-1	protein_coding	5019	OXCT1	0.267382736691876	-1.206699839	0	0	0	NA	NA	NA	NA	Yes	GSE86189_GA.po.all.FUMA:GSE86189_GA.pp.all.FUMA	1.92234e-06	5:40704074:A/G:5:40812231:A/G	27
ENSG00000248668	OXCT1-AS1	41870132	41871059	1	processed_transcript	100874002	OXCT1-AS1	NA	NA	0	0	0	NA	NA	NA	NA	Yes	GSE86189_GA.po.all.FUMA:GSE86189_GA.pp.all.FUMA	1.92234e-06	5:40704074:A/G:5:40812231:A/G	27
ENSG00000240281	CTD-2062O1.1	41895170	41895701	1	pseudogene	NA	NA	NA	NA	0	0	0	NA	NA	NA	NA	Yes	GSE86189_GA.po.all.FUMA	1.92234e-06	5:40812231:A/G	27
ENSG00000205765	C5orf51	41904290	41921738	1	protein_coding	285636	C5orf51	0.00388889950519938	-0.967440208	0	0	0	NA	NA	NA	NA	Yes	GSE86189_GA.po.all.FUMA:GSE86189_GA.pp.all.FUMA	1.92234e-06	5:40704074:A/G:5:40812231:A/G	27
ENSG00000151876	FBXO4	41925356	41941845	1	protein_coding	26272	FBXO4	7.10839893911415e-05	-0.664218696	0	0	0	NA	NA	NA	NA	Yes	GSE86189_GA.po.all.FUMA:GSE86189_GA.pp.all.FUMA	1.92234e-06	5:40704074:A/G:5:40812231:A/G	27
ENSG00000239694	CTD-2156G10.1	41951415	41952295	-1	pseudogene	NA	NA	NA	NA	0	0	0	NA	NA	NA	NA	Yes	GSE86189_GA.po.all.FUMA	1.92234e-06	5:40812231:A/G	27
ENSG00000199487	Y_RNA	41963970	41964071	-1	misc_RNA	NA	NA	NA	NA	0	0	0	NA	NA	NA	NA	Yes	GSE86189_GA.po.all.FUMA	1.92234e-06	5:40812231:A/G	27
ENSG00000249266	MTHFD2P6	41967241	41968468	-1	pseudogene	100130042	MTHFD2P6	NA	NA	0	0	0	NA	NA	NA	NA	Yes	GSE86189_GA.po.all.FUMA	1.92234e-06	5:40812231:A/G	27
ENSG00000260786	RP11-112L7.1	42188368	42191625	1	lincRNA	NA	NA	NA	NA	0	0	0	NA	NA	NA	NA	Yes	GSE86189_GA.po.all.FUMA	1.92234e-06	5:40812231:A/G	27
ENSG00000112964	GHR	42423879	42721979	1	protein_coding	2690	GHR	0.000232177140704853	-0.246807514	0	0	0	NA	NA	NA	NA	Yes	GSE86189_GA.po.all.FUMA:G	1.92234e-06	5:40704074:A/G:5:40812231:A/G	27

																	SE86189_GA.pp.all.FUMA			
ENSG00000248873	SERBP1P6	42465502	42468970	-1	pseudogene	100420450	SERBP1P6	NA	NA	0	0	0	NA	NA	NA	NA	Yes	GSE86189_GA.po.all.FUMA	1.92234e-06	5:40812231:A/G	27
ENSG00000250860	CTD-2265D6.2	42466995	42468402	-1	pseudogene	NA	NA	NA	NA	0	0	0	NA	NA	NA	NA	Yes	GSE86189_GA.po.all.FUMA	1.92234e-06	5:40812231:A/G	27
ENSG00000270513	RP11-116B13.1	42729240	42729621	1	pseudogene	NA	NA	NA	NA	0	0	0	NA	NA	NA	NA	Yes	GSE86189_GA.po.all.FUMA	1.92234e-06	5:40812231:A/G	27
ENSG00000198865	CCDC152	42756903	42802462	1	protein_coding	100129792	CCDC152	NA	0.021341096	0	0	0	NA	NA	NA	NA	Yes	GSE86189_GA.pp.all.FUMA	1.92234e-06	5:40812231:A/G	27
ENSG00000118496	FBXO30	146114638	146135889	-1	protein_coding	84085	FBXO30	0.987340441468601	0.325150484	0	0	0	NA	NA	NA	NA	Yes	GSE86189_GA.po.all.FUMA	3.49847e-05	6:148171512:A/G	33
ENSG00000235652	RP11-545I5.3	146136048	146207721	1	antisense	100507557	LOC100507557	NA	NA	0	0	0	NA	NA	NA	NA	Yes	GSE86189_GA.po.all.FUMA	3.49847e-05	6:148171512:A/G	33
ENSG00000146414	SHPRH	146185381	146285559	-1	protein_coding	257218	SHPRH	0.491308215820232	-0.613553002	0	0	0	NA	NA	NA	NA	Yes	GSE86189_GA.po.all.FUMA	3.49847e-05	6:148171512:A/G	33
ENSG00000152822	GRM1	146348782	146758734	1	protein_coding	2911	GRM1	0.989964591707789	-0.267244582	0	0	0	NA	NA	NA	NA	Yes	GSE86189_GA.po.all.FUMA	3.49847e-05	6:148171512:A/G	33
ENSG00000118508	RAB32	146864829	146876101	1	protein_coding	10981	RAB32	0.00877092275554058	NA	0	0	0	NA	NA	NA	NA	Yes	GSE86189_GA.po.all.FUMA	3.49847e-05	6:148171512:A/G	33
ENSG00000237468	RP11-715G15.1	146915652	146920067	-1	lincRNA	101928661	LOC101928661	NA	NA	0	0	0	NA	NA	NA	NA	Yes	GSE86189_GA.po.all.FUMA	3.49847e-05	6:148171512:A/G	33
ENSG00000118492	ADGB	146920101	147136598	1	protein_coding	79747	ADGB	NA	NA	0	0	0	NA	NA	NA	NA	Yes	GSE86189_GA.po.all.FUMA	3.49847e-05	6:148171512:A/G	33
ENSG00000233452	STXBP5-AS1	147163037	147525750	-1	antisense	729178	STXBP5-AS1	NA	NA	0	0	0	NA	NA	NA	NA	Yes	GSE86189_GA.po.all.FUMA	3.49847e-05	6:148171512:A/G	33
ENSG00000164506	STXBP5	147525561	147706866	1	protein_coding	134957	STXBP5	0.999991599632041	NA	0	0	0	NA	NA	NA	NA	Yes	GSE86189_GA.po.all.FUMA	3.49847e-05	6:148171512:A/G	33
ENSG00000203727	SAMD5	147830063	148058683	1	protein_coding	389432	SAMD5	0.000898003812903688	1.680995061	0	0	0	NA	NA	NA	NA	Yes	GSE86189_GA.po.all.FUMA	3.49847e-05	6:148171512:A/G	33
ENSG00000227681	RP11-307P5.1	147981839	148275073	1	lincRNA	NA	NA	NA	NA	1	17.20		NA	NA	NA	NA	No	NA	3.49847e-05	6:148171512:A/G	33
ENSG00000111961	SASH1	148593440	148873186	1	protein_coding	23328	SASH1	0.00325444461467519	-1.839894093	0	0	0	NA	NA	NA	NA	Yes	GSE86189_GA.po.all.FUMA	3.49847e-05	6:148171512:A/G	33
ENSG00000111962	UST	149068464	149398126	1	protein_coding	10090	UST	0.984203294606023	-0.711878088	0	0	0	NA	NA	NA	NA	Yes	GSE86189_GA.po.all.FUMA	3.49847e-05	6:148171512:A/G	33
ENSG00000228408	RP1-111D6.3	149539062	149565208	1	lincRNA	23118	TAB2	NA	NA	0	0	0	NA	NA	NA	NA	Yes	GSE86189_GA.po.all.FUMA	3.49847e-05	6:148171512:A/G	33
ENSG00000055208	TAB2	149539777	149732749	1	protein_coding	23118	TAB2	0.99975796201866	-0.094395118	0	0	0	NA	NA	NA	NA	Yes	GSE86189_GA.po.all.FUMA	3.49847e-05	6:148171512:A/G	33
ENSG00000177688	SUMO4	149721495	149722177	1	protein_coding	387082	SUMO4	0.217538234779752	0.214056968	0	0	0	NA	NA	NA	NA	Yes	GSE86189_GA.po.all.FUMA	3.49847e-05	6:148171512:A/G	33
ENSG00000178199	ZC3H12D	149768794	149806197	-1	protein_coding	340152	ZC3H12D	0.727798125573567	NA	0	0	0	NA	NA	NA	NA	Yes	GSE86189_GA.po.all.FUMA	3.49847e-05	6:148171512:A/G	33

ENSG00000131013	PPIL4	149825869	149867174	-1	protein_coding	85313	PPIL4	1.68093009893407e-05	-0.526398148	0	0	0	NA	NA	NA	NA	Yes	GSE86189_GA.po.all.FUMA	3.49847e-05	6:148171512:A/G	33
ENSG00000055211	GINM1	149887430	149912884	1	protein_coding	116254	GINM1	7.70683279294107e-05	-0.261942356	0	0	0	NA	NA	NA	NA	Yes	GSE86189_GA.po.all.FUMA	3.49847e-05	6:148171512:A/G	33
ENSG00000186625	KATNA1	149916009	149970108	-1	protein_coding	11104	KATNA1	0.00192587515145671	-0.381084529	0	0	0	NA	NA	NA	NA	Yes	GSE86189_GA.po.all.FUMA	3.49847e-05	6:148171512:A/G	33
ENSG00000131023	LATS1	149979289	150039392	-1	protein_coding	9113	LATS1	0.999926526245065	0.77212935	0	0	0	NA	NA	NA	NA	Yes	GSE86189_GA.po.all.FUMA	3.49847e-05	6:148171512:A/G	33
ENSG00000120253	NUP43	150045451	150070801	-1	protein_coding	348995	NUP43	3.54205607988959e-06	0.080187546	0	0	0	NA	NA	NA	NA	Yes	GSE86189_GA.po.all.FUMA	3.49847e-05	6:148171512:A/G	33
ENSG00000120265	PCMT1	150070579	150132556	1	protein_coding	5110	PCMT1	0.446383565895946	-0.308497206	0	0	0	NA	NA	NA	NA	Yes	GSE86189_GA.po.all.FUMA	3.49847e-05	6:148171512:A/G	33
ENSG00000237620	GCNT1P5	77090775	77091456	1	pseudogene	100421598	GCNT1P5	NA	NA	0	0	0	NA	NA	NA	NA	Yes	GSE86189_GA.po.all.FUMA	9.1993e-05	7:79065472:A/G	37
ENSG00000127947	PTPN12	77166592	77269388	1	protein_coding	5782	PTPN12	0.999459858785815	NA	0	0	0	NA	NA	NA	NA	Yes	GSE86189_GA.pp.all.FUMA	9.1993e-05	7:79065472:A/G	37
ENSG00000214293	RSBN1L-AS1	77286977	77325582	-1	lincRNA	100505854	RSBN1L-AS1	NA	NA	0	0	0	NA	NA	NA	NA	Yes	GSE86189_GA.pp.all.FUMA	9.1993e-05	7:79065472:A/G	37
ENSG00000187257	RSBN1L	77325760	77412339	1	protein_coding	222194	RSBN1L	0.500030091124699	-0.401014262	0	0	0	NA	NA	NA	NA	Yes	GSE86189_GA.pp.all.FUMA	9.1993e-05	7:79065472:A/G	37
ENSG00000135211	TMEM60	77423045	77427897	-1	protein_coding	85025	TMEM60	0.661431575510107	0.010916165	0	0	0	NA	NA	NA	NA	Yes	GSE86189_GA.pp.all.FUMA	9.1993e-05	7:79065472:A/G	37
ENSG00000006576	PHTF2	77428122	77586818	1	protein_coding	57157	PHTF2	0.0249464272569149	0.430860463	0	0	0	NA	NA	NA	NA	Yes	GSE86189_GA.pp.all.FUMA	9.1993e-05	7:79065472:A/G	37
ENSG00000222432	Y_RNA	77525197	77525309	1	misc_RNA	NA	NA	NA	NA	0	0	0	NA	NA	NA	NA	Yes	GSE86189_GA.po.all.FUMA	9.1993e-05	7:79065472:A/G	37
ENSG00000232756	RP5-1185I7.1	77619701	77624488	1	antisense	NA	NA	NA	NA	0	0	0	NA	NA	NA	NA	Yes	GSE86189_GA.po.all.FUMA	9.1993e-05	7:79065472:A/G	37
ENSG00000187391	MAGI2	77646393	79082890	-1	protein_coding	9863	MAGI2	0.883174386756098	-1.160246053	1	14.809	NA	NA	NA	NA	NA	Yes	GSE86189_GA.pp.all.FUMA	9.1993e-05	7:79065472:A/G	37
ENSG00000231322	RPL13AP17	77976459	77988775	1	pseudogene	399670	RPL13AP17	NA	NA	0	0	0	NA	NA	NA	NA	Yes	GSE86189_GA.po.all.FUMA	9.1993e-05	7:79065472:A/G	37
ENSG00000226230	AC007237.2	78115847	78116345	1	pseudogene	NA	NA	NA	NA	0	0	0	NA	NA	NA	NA	Yes	GSE86189_GA.po.all.FUMA	9.1993e-05	7:79065472:A/G	37
ENSG00000235751	MAGI2-IT1	78530591	78629614	-1	sense_intronic	NA	MAGI2-IT1	NA	NA	0	0	0	NA	NA	NA	NA	Yes	GSE86189_GA.po.all.FUMA	9.1993e-05	7:79065472:A/G	37
ENSG00000251276	MAGI2-AS1	78569166	78570211	1	antisense	100874020	MAGI2-AS1	NA	NA	0	0	0	NA	NA	NA	NA	Yes	GSE86189_GA.po.all.FUMA	9.1993e-05	7:79065472:A/G	37
ENSG00000226978	MAGI2-AS2	78638304	78641593	1	antisense	100874021	MAGI2-AS2	NA	NA	0	0	0	NA	NA	NA	NA	Yes	GSE86189_GA.po.all.FUMA	9.1993e-05	7:79065472:A/G	37
ENSG00000212545	RNU6-337P	78659556	78659661	1	snRNA	106479716	RNU6-337P	NA	NA	0	0	0	NA	NA	NA	NA	Yes	GSE86189_GA.po.all.FUMA	9.1993e-05	7:79065472:A/G	37
ENSG00000229110	AC006355.3	78754055	78755066	1	pseudogene	NA	NA	NA	NA	0	0	0	NA	NA	NA	NA	Yes	GSE86189_GA.po.all.FUMA	9.1993e-05	7:79065472:A/G	37

ENSG00000212482	RNU6-530P	78972582	78972688	-1	snRNA	106479801	RNU6-530P	NA	NA	0	0	0	NA	NA	NA	NA	Yes	GSE86189_GA.po.all.FUMA	9.1993e-05	7:79065472:A/G	37
ENSG00000222024	AC004945.1	78982726	78983708	1	pseudogene	NA	NA	NA	NA	0	0	0	NA	NA	NA	NA	Yes	GSE86189_GA.po.all.FUMA	9.1993e-05	7:79065472:A/G	37
ENSG00000234456	MAGI2-AS3	79082198	79100524	1	processed_transcript	100505881	MAGI2-AS3	NA	NA	1	14.809		NA	NA	NA	NA	Yes	GSE86189_GA.pp.all.FUMA	9.1993e-05	7:79065472:A/G	37
ENSG00000226285	AC091813.2	79173227	79174169	-1	pseudogene	NA	NA	NA	NA	0	0	0	NA	NA	NA	NA	Yes	GSE86189_GA.po.all.FUMA	9.1993e-05	7:79065472:A/G	37
ENSG00000200786	RNA5SP234	79283425	79283543	1	rRNA	100873492	RNA5SP234	NA	NA	0	0	0	NA	NA	NA	NA	Yes	GSE86189_GA.po.all.FUMA	9.1993e-05	7:79065472:A/G	37
ENSG00000206818	RNU6-849P	79541420	79541524	-1	snRNA	106481449	RNU6-849P	NA	NA	0	0	0	NA	NA	NA	NA	Yes	GSE86189_GA.po.all.FUMA	9.1993e-05	7:79065472:A/G	37
ENSG00000230853	AC004159.1	79725916	79726531	-1	pseudogene	NA	NA	NA	NA	0	0	0	NA	NA	NA	NA	Yes	GSE86189_GA.po.all.FUMA	9.1993e-05	7:79065472:A/G	37
ENSG00000127955	GNAI1	79763271	79848718	1	protein_coding	2770	GNAI1	0.917528243665787	0.288400901	0	0	0	NA	NA	NA	NA	Yes	GSE86189_GA.pp.all.FUMA	9.1993e-05	7:79065472:A/G	37
ENSG00000244392	RN7SL869P	79875242	79875538	1	misc_RNA	106481864	RN7SL869P	NA	NA	0	0	0	NA	NA	NA	NA	Yes	GSE86189_GA.po.all.FUMA	9.1993e-05	7:79065472:A/G	37
ENSG00000234223	AC003988.1	79875725	79941772	-1	lincRNA	NA	NA	NA	NA	0	0	0	NA	NA	NA	NA	Yes	GSE86189_GA.po.all.FUMA	9.1993e-05	7:79065472:A/G	37
ENSG00000240347	RN7SL35P	79947569	79947857	1	misc_RNA	106480931	RN7SL35P	NA	NA	0	0	0	NA	NA	NA	NA	Yes	GSE86189_GA.po.all.FUMA	9.1993e-05	7:79065472:A/G	37
ENSG00000232667	AC004862.6	79959508	80014295	-1	antisense	NA	NA	NA	NA	0	0	0	NA	NA	NA	NA	Yes	GSE86189_GA.po.all.FUMA	9.1993e-05	7:79065472:A/G	37
ENSG00000135218	CD36	79998891	80308593	1	protein_coding	948	CD36	8.31103233660849e-46	-0.1216654	0	0	0	NA	NA	NA	NA	Yes	GSE86189_GA.pp.all.FUMA	9.1993e-05	7:79065472:A/G	37
ENSG00000223550	SNRPBP1	80006870	80007151	-1	pseudogene	100499258	SNRPBP1	NA	NA	0	0	0	NA	NA	NA	NA	Yes	GSE86189_GA.po.all.FUMA	9.1993e-05	7:79065472:A/G	37
ENSG00000214415	GNAT3	80087987	80141336	-1	protein_coding	346562	GNAT3	0.000297548170729583	0.04372583	0	0	0	NA	NA	NA	NA	Yes	GSE86189_GA.pp.all.FUMA	9.1993e-05	7:79065472:A/G	37
ENSG00000229436	AC073850.6	80291647	80291901	-1	pseudogene	NA	NA	NA	NA	0	0	0	NA	NA	NA	NA	Yes	GSE86189_GA.po.all.FUMA	9.1993e-05	7:79065472:A/G	37
ENSG00000075223	SEMA3C	80371854	80551675	-1	protein_coding	10512	SEMA3C	0.296856253558956	-0.134328866	0	0	0	NA	NA	NA	NA	Yes	GSE86189_GA.pp.all.FUMA	9.1993e-05	7:79065472:A/G	37
ENSG00000237896	AC005008.2	80804824	80828431	1	protein_coding	NA	NA	NA	NA	0	0	0	NA	NA	NA	NA	Yes	GSE86189_GA.pp.all.FUMA	9.1993e-05	7:79065472:A/G	37
ENSG00000226671	AC005008.3	80820714	80821325	1	pseudogene	NA	NA	NA	NA	0	0	0	NA	NA	NA	NA	Yes	GSE86189_GA.po.all.FUMA	9.1993e-05	7:79065472:A/G	37
ENSG00000223514	AC004866.1	80964422	80964499	1	pseudogene	NA	NA	NA	NA	0	0	0	NA	NA	NA	NA	Yes	GSE86189_GA.po.all.FUMA	9.1993e-05	7:79065472:A/G	37
ENSG00000224134	AC004866.3	81061047	81062112	1	pseudogene	NA	NA	NA	NA	0	0	0	NA	NA	NA	NA	Yes	GSE86189_GA.po.all.FUMA	9.1993e-05	7:79065472:A/G	37

ENSG00000155897	ADCY8	131792547	132054672	-1	protein_coding	114	ADCY8	0.0029856615213929	-1.076586681	0	0	0	NA	NA	NA	NA	Yes	GSE86189_GA.po.all.FUMA	2.06512e-05	8:133641587:A/G	44
ENSG00000132294	EFR3A	132916335	133025889	1	protein_coding	23167	EFR3A	0.00926619083230622	0.053806681	0	0	0	NA	NA	NA	NA	Yes	GSE86189_GA.po.all.FUMA	2.06512e-05	8:133641587:A/G	44
ENSG00000258417	OC90	133036467	133097902	-1	protein_coding	729330	OC90	NA	0.300827774	0	0	0	NA	NA	NA	NA	Yes	GSE86189_GA.po.all.FUMA	2.06512e-05	8:133641587:A/G	44
ENSG00000253117	OC90	133036467	133071627	-1	protein_coding	729330	OC90	8.16819622863985e-09	0.300827774	0	0	0	NA	NA	NA	NA	Yes	GSE86189_GA.po.all.FUMA	2.06512e-05	8:133641587:A/G	44
ENSG00000132297	HHLA1	133073733	133123406	-1	protein_coding	10086	HHLA1	NA	NA	0	0	0	NA	NA	NA	NA	Yes	GSE86189_GA.po.all.FUMA	2.06512e-05	8:133641587:A/G	44
ENSG00000184156	KCNQ3	133133108	133493200	-1	protein_coding	3786	KCNQ3	0.985604293672352	4.015419742	0	0	0	NA	NA	NA	NA	Yes	GSE86189_GA.po.all.FUMA	2.06512e-05	8:133641587:A/G	44
ENSG00000129295	LRRC6	133584320	133687838	-1	protein_coding	23639	LRRC6	8.29727962409699e-08	-0.489983494	1	18.40		NA	NA	NA	NA	Yes	GSE86189_GA.po.all.FUMA	2.06512e-05	8:133641587:A/G	44
ENSG00000165071	TMEM71	133697253	133772958	-1	protein_coding	137835	TMEM71	4.16584061056541e-10	0.169537614	0	0	0	NA	NA	NA	NA	Yes	GSE86189_GA.po.all.FUMA	2.06512e-05	8:133641587:A/G	44
ENSG00000129292	PHF20L1	133787618	133861052	1	protein_coding	51105	PHF20L1	0.99991198433104	-0.146737214	0	0	0	NA	NA	NA	NA	Yes	GSE86189_GA.po.all.FUMA	2.06512e-05	8:133641587:A/G	44
ENSG00000042832	TG	133879203	134147147	1	protein_coding	7038	TG	2.62956477021395e-29	-0.23993182	0	0	0	NA	NA	NA	NA	Yes	GSE86189_GA.po.all.FUMA	2.06512e-05	8:133641587:A/G	44
ENSG00000155926	SLA	134048973	134115298	-1	protein_coding	6503	SLA	0.722953169634171	-0.370064375	0	0	0	NA	NA	NA	NA	Yes	GSE86189_GA.po.all.FUMA	2.06512e-05	8:133641587:A/G	44
ENSG00000104415	WISP1	134203282	134242587	1	protein_coding	8840	WISP1	0.00332828025066983	0.45333974	0	0	0	NA	NA	NA	NA	Yes	GSE86189_GA.po.all.FUMA	2.06512e-05	8:133641587:A/G	44
ENSG00000104419	NDRG1	134249414	134314265	-1	protein_coding	10397	NDRG1	0.0538232412513876	-0.014490555	0	0	0	NA	NA	NA	NA	Yes	GSE86189_GA.po.all.FUMA	2.06512e-05	8:133641587:A/G	44
ENSG00000008513	ST3GAL1	134467091	134584183	-1	protein_coding	6482	ST3GAL1	0.59355924559558	0.818609209	0	0	0	NA	NA	NA	NA	Yes	GSE86189_GA.po.all.FUMA	2.06512e-05	8:133641587:A/G	44
ENSG00000261220	RP11-629O1.2	134585426	134586104	1	lincRNA	NA	NA	NA	NA	0	0	0	NA	NA	NA	NA	Yes	GSE86189_GA.po.all.FUMA	2.06512e-05	8:133641587:A/G	44
ENSG00000169398	PTK2	141667999	142012315	-1	protein_coding	5747	PTK2	0.999992957786805	-0.489090912	0	0	0	NA	NA	NA	NA	Yes	GSE86189_GA.po.all.FUMA:GSE86189_GA.pp.all.FUMA	5.02934e-11	8:143776668:A/G:8:143776668:A/G;8:143752235:A/G:8:143844650:A/G	45
ENSG00000252864	RNA5SP278	141950661	141950762	1	rRNA	100873531	RNA5SP278	NA	NA	0	0	0	NA	NA	NA	NA	Yes	GSE86189_GA.po.all.FUMA	1.95086e-09	8:143776668:A/G;8:143752235:A/G:8:143844650:A/G	45
ENSG00000253266	RP11-128L5.1	142065389	142070695	1	lincRNA	NA	NA	NA	NA	0	0	0	NA	NA	NA	NA	Yes	GSE86189_GA.po.all.FUMA	1.95086e-09	8:143776668:A/G;8:143752235:A/G:8:143844650:A/G	45
ENSG00000105339	DENND3	142127377	142205907	1	protein_coding	22898	DENND3	0.0171730327867355	0.99937938	0	0	0	NA	NA	NA	NA	Yes	GSE86189_GA.po.all.FUMA:GSE86189_GA.pp.all.FUMA	5.02934e-11	8:143776668:A/G:8:143776668:A/G;8:143752235:A/G:8:143844650:A/G	45

ENSG00000253210	RP11-809O17.1	142136143	142140060	-1	antisense	NA	NA	NA	NA	0	0	0	NA	NA	NA	NA	Yes	GSE86189_GA.po.all.FUMA	1.95086e-09	8:143776668:A/G;8:1437 4552235:A/G:8:143844650:A/G
ENSG00000022567	SLC45A4	142217265	142318404	-1	protein_coding	57210	SLC45A4	0.00458960961270734	-0.006523435	0	0	0	NA	NA	NA	NA	Yes	GSE86189_GA.po.all.FUMA:GSE86189_GA.pp.all.FUMA	5.02934e-11	8:143776668:A/G;8:1437 4576668:A/G;8:143752235:A/G:8:143844650:A/G
ENSG00000253307	RP11-10J21.4	142262385	142263391	-1	antisense	NA	NA	NA	NA	0	0	0	NA	NA	NA	NA	Yes	GSE86189_GA.po.all.FUMA:GSE86189_GA.pp.all.FUMA	5.02934e-11	8:143776668:A/G;8:1437 4576668:A/G;8:143752235:A/G:8:143844650:A/G
ENSG00000254019	RP11-10J21.3	142264664	142266916	1	protein_coding	NA	NA	NA	NA	0	0	0	NA	NA	NA	NA	Yes	GSE86189_GA.po.all.FUMA:GSE86189_GA.pp.all.FUMA	5.02934e-11	8:143776668:A/G;8:1437 4576668:A/G;8:143752235:A/G:8:143844650:A/G
ENSG00000254197	RP11-10J21.5	142288327	142302961	1	lincRNA	NA	NA	NA	NA	0	0	0	NA	NA	NA	NA	Yes	GSE86189_GA.po.all.FUMA	1.95086e-09	8:143776668:A/G;8:1437 4552235:A/G:8:143844650:A/G
ENSG00000254291	RP11-10J21.6	142336229	142337236	1	antisense	NA	NA	NA	NA	0	0	0	NA	NA	NA	NA	Yes	GSE86189_GA.po.all.FUMA	1.95086e-09	8:143776668:A/G;8:1437 4552235:A/G:8:143844650:A/G
ENSG00000253595	CTD-3064M3.1	142350648	142354720	1	antisense	731779	LINC01300	NA	NA	0	0	0	NA	NA	NA	NA	Yes	GSE86189_GA.po.all.FUMA:GSE86189_GA.pp.all.FUMA	5.02934e-11	8:143776668:A/G;8:1437 4576668:A/G;8:143752235:A/G:8:143844650:A/G
ENSG00000261655	CTD-3064M3.3	142363503	142365465	-1	sense_overlapping	NA	NA	NA	NA	0	0	0	NA	NA	NA	NA	Yes	GSE86189_GA.po.all.FUMA:GSE86189_GA.pp.all.FUMA	5.02934e-11	8:143776668:A/G;8:1437 4576668:A/G;8:143752235:A/G:8:143844650:A/G
ENSG00000204882	GPR20	142366600	142377367	-1	protein_coding	2843	GPR20	0.315068599677581	-0.3845718	0	0	0	NA	NA	NA	NA	Yes	GSE86189_GA.po.all.FUMA:GSE86189_GA.pp.all.FUMA	5.02934e-11	8:143776668:A/G;8:1437 4576668:A/G;8:143752235:A/G:8:143844650:A/G
ENSG00000244998	CTD-3064M3.4	142400039	142402674	-1	antisense	NA	NA	NA	NA	0	0	0	NA	NA	NA	NA	Yes	GSE86189_GA.po.all.FUMA:GSE86189_GA.pp.all.FUMA	5.02934e-11	8:143776668:A/G;8:1437 4576668:A/G;8:143752235:A/G:8:143844650:A/G
ENSG00000184489	PTP4A3	142402093	142441620	1	protein_coding	11156	PTP4A3	0.127669884218514	-0.840871046	0	0	0	NA	NA	NA	NA	Yes	GSE86189_GA.po.all.FUMA:GSE86189_GA.pp.all.FUMA	5.02934e-11	8:143776668:A/G;8:1437 4576668:A/G;8:143752235:A/G:8:143844650:A/G
ENSG00000226807	MROH5	142443929	142517330	-1	polymorphic_pseudogene	389690	MROH5	NA	0.06511661	0	0	0	NA	NA	NA	NA	Yes	GSE86189_GA.po.all.FUMA	1.95086e-09	8:143776668:A/G;8:1437 4552235:A/G:8:143844650:A/G

ENSG00000271959	CTD-3064M3.7	142444645	142448054	1	antisense	NA	NA	NA	NA	0	0	0	NA	NA	NA	NA	Yes	GSE86189_GA.po.all.FUMA	1.95086e-09	8:143776668:A/G;8:1437 45 52235:A/G:8:143844650:A/G
ENSG00000238854	SNORD5	142457575	142457649	1	snoRNA	692072	SNORD5	NA	NA	0	0	0	NA	NA	NA	NA	Yes	GSE86189_GA.po.all.FUMA	1.95086e-09	8:143776668:A/G;8:1437 45 52235:A/G:8:143844650:A/G
ENSG00000253605	HNRNPA1P38	142469175	142470131	1	pseudogene	100421384	HNRNPA1P38	NA	NA	0	0	0	NA	NA	NA	NA	Yes	GSE86189_GA.po.all.FUMA	1.95086e-09	8:143776668:A/G;8:1437 45 52235:A/G:8:143844650:A/G
ENSG00000226490	AC138647.1	142524738	142528837	1	protein_coding	NA	NA	NA	NA	0	0	0	NA	NA	NA	NA	Yes	GSE86189_GA.po.all.FUMA:GSE86189_GA.pp.all.FUMA	5.02934e-11	8:143776668:A/G:8:1437 45 76668:A/G;8:143752235:A/G:8:143844650:A/G
ENSG00000221123	AC104417.1	143026860	143026986	1	miRNA	NA	NA	NA	NA	0	0	0	NA	NA	NA	NA	Yes	GSE86189_GA.po.all.FUMA	1.95086e-09	8:143776668:A/G;8:1437 45 52235:A/G:8:143844650:A/G
ENSG00000254183	RP11-953B20.2	143049452	143050202	-1	pseudogene	NA	NA	NA	NA	0	0	0	NA	NA	NA	NA	Yes	GSE86189_GA.po.all.FUMA	1.95086e-09	8:143776668:A/G;8:1437 45 52235:A/G:8:143844650:A/G
ENSG00000261710	RP11-953B20.1	143171188	143172980	1	lincRNA	NA	NA	NA	NA	0	0	0	NA	NA	NA	NA	Yes	GSE86189_GA.po.all.FUMA	1.95086e-09	8:143776668:A/G;8:1437 45 52235:A/G:8:143844650:A/G
ENSG00000265247	MIR4472-1	143257700	143257779	1	miRNA	100616268	MIR4472-1	NA	NA	0	0	0	NA	NA	NA	NA	Yes	GSE86189_GA.po.all.FUMA	1.95086e-09	8:143776668:A/G;8:1437 45 52235:A/G:8:143844650:A/G
ENSG00000254008	LINC00051	143279717	143290364	1	antisense	619434	LINC00051	NA	NA	0	0	0	NA	NA	NA	NA	Yes	GSE86189_GA.po.all.FUMA	1.95086e-09	8:143776668:A/G;8:1437 45 52235:A/G:8:143844650:A/G
ENSG00000171045	TSNARE1	143293441	143484601	-1	protein_coding	203062	TSNARE1	3.60235223112282e-07	NA	0	0	1	0.0297013	NA	GTEx/v8/Stomach	+	Yes	GSE86189_GA.po.all.FUMA:GSE86189_GA.pp.all.FUMA	5.02934e-11	8:143776668:A/G;8:1437 45 76668:A/G:8:143776668:A/G;8:143752235:A/G:8:143844650:A/G
ENSG00000253602	CTD-3135A9.3	143328948	143329227	-1	misc_RNA	NA	NA	NA	NA	0	0	0	NA	NA	NA	NA	Yes	GSE86189_GA.po.all.FUMA	1.95086e-09	8:143776668:A/G;8:1437 45 52235:A/G:8:143844650:A/G
ENSG00000261693	RP13-467H17.1	143485013	143488389	1	antisense	NA	NA	NA	NA	0	0	0	NA	NA	NA	NA	Yes	GSE86189_GA.po.all.FUMA:GSE86189_GA.pp.all.FUMA	5.02934e-11	8:143776668:A/G:8:1437 45 76668:A/G;8:143752235:A/G:8:143844650:A/G
ENSG00000181790	BAI1	143530791	143626370	1	protein_coding	575	BAI1	0.999875311419222	NA	0	0	4	0.0255042	NA	GTEx/v8/St	+	Yes	GSE86189_GA.po.all.FUMA:GSE86189_GA.pp.all.FUMA	5.02934e-11	8:143776668:A/G;8:1437 45 52235:A/G;8:143776668:A/G:8:143776668:A/G;8:1

															omach					43752235:A/G:8:143844650:A/G
ENSG00000232722	MROH4P	143648621	143653366	-1	pseudogene	101154686	MROH4P	NA	NA	0	0	0	NA	NA	NA	NA	Yes	GSE86189_GA.po.all.FUMA	1.95086e-09	8:143776668:A/G;8:1437 4552235:A/G:8:143844650:A/G
ENSG00000198576	ARC	143692405	143696833	-1	protein_coding	23237	ARC	0.920765733774356	-0.809951034	0	0	0	NA	NA	NA	NA	Yes	GSE86189_GA.po.all.FUMA:GSE86189_GA.pp.all.FUMA	5.02934e-11	8:143776668:A/G:8:1437 4576668:A/G;8:143752235:A/G:8:143844650:A/G
ENSG00000261044	AP006547.3	143701734	143702425	1	antisense	NA	NA	NA	NA	0	0	0	NA	NA	NA	NA	Yes	GSE86189_GA.po.all.FUMA:GSE86189_GA.pp.all.FUMA	5.02934e-11	8:143776668:A/G:8:1437 4576668:A/G;8:143752235:A/G:8:143844650:A/G
ENSG00000253753	AC145123.2	143719949	143722027	1	antisense	NA	NA	NA	NA	0	0	0	NA	NA	NA	NA	Yes	GSE86189_GA.po.all.FUMA	1.95086e-09	8:143776668:A/G;8:1437 4552235:A/G:8:143844650:A/G
ENSG00000234616	JRK	143738874	143763386	-1	processed_transcript	8629	JRK	NA	NA	1	13.711		0.0213638	NA	GTEx/v8/Stomach	-	Yes	GSE86189_GA.po.all.FUMA	5.02934e-11	8:143776668:A/G;8:1437 4552235:A/G:8:143844650:A/G
ENSG00000167653	PSCA	143751726	143764142	1	protein_coding	8000	PSCA	0.00894842686480651	1.448535139	1	13.761		8.87721e-53	1.4886e-47	GTEx/v8/Stomach	+	Yes	GSE86189_GA.po.all.FUMA:GSE86189_GA.pp.all.FUMA	5.02934e-11	8:143776668:A/G;8:1437 4552235:A/G;8:143844650:A/G;8:143776668:A/G:8:143776668:A/G;8:143752235:A/G:8:143844650:A/G
ENSG00000160886	LY6K	143781529	143786545	1	protein_coding	54742	LY6K	0.000583632193494971	0.620706009	2	13.761		8.60331e-09	4.79215e-05	GTEx/v8/Stomach	+	Yes	GSE86189_GA.po.all.FUMA:GSE86189_GA.pp.all.FUMA	5.02934e-11	8:143776668:A/G;8:1437 4552235:A/G;8:143844650:A/G;8:143776668:A/G:8:143776668:A/G;8:143752235:A/G:8:143844650:A/G
ENSG00000253741	CTD-2292P10.4	143783670	143808391	-1	antisense	100288181	LOC100288181	NA	NA	2	13.761		3.23573e-11	2.92856e-07	GTEx/v8/Stomach	+	Yes	GSE86189_GA.po.all.FUMA:GSE86189_GA.pp.all.FUMA	5.02934e-11	8:143776668:A/G;8:1437 4552235:A/G;8:143844650:A/G;8:143776668:A/G:8:143776668:A/G;8:143752235:A/G:8:143844650:A/G
ENSG00000130193	THEM6	143808621	143818345	1	protein_coding	51337	THEM6	0.0242798295662762	-0.051935087	2	13.365		1.37641e-16	9.37308e-12	GTEx/v8/Stomach	+	Yes	GSE86189_GA.po.all.FUMA:GSE86189_GA.pp.all.FUMA	5.02934e-11	8:143776668:A/G;8:1437 4552235:A/G;8:143844650:A/G;8:143776668:A/G:8:143776668:A/G;8:143752235:A/G:8:143844650:A/G
ENSG00000253806	CTD-2292P10.2	143808701	143809108	-1	antisense	NA	NA	NA	NA	1	12.60		NA	NA	NA	NA	Yes	GSE86189_GA.po.all.FUMA:G	5.02934e-11	8:143776668:A/G;8:1437 4552235:A/G;8:143776668:A/G:8:143776668:A/G;8:1

																		SE86189_GA.pp.all.FUMA		43752235:A/G:8:143844650:A/G	
ENSG00000126233	SLURP1	143822362	143823829	-1	protein_coding	57152	SLURP1	0.328314103428116	0.273296193	2	13.305		NA	NA	NA	NA	Yes	GSE86189_GA.po.all.FUMA:GSE86189_GA.pp.all.FUMA	5.02934e-11	8:143776668:A/G;8:143752235:A/G;8:143844650:A/G;8:143752235:A/G:8:143844650:A/G:8:143776668:A/G	45
ENSG00000197353	LYPD2	143831568	143833952	-1	protein_coding	137797	LYPD2	0.0633022046112556	-0.383771532	2	13.305		NA	NA	NA	NA	Yes	GSE86189_GA.po.all.FUMA:GSE86189_GA.pp.all.FUMA	5.02934e-11	8:143776668:A/G;8:143752235:A/G;8:143844650:A/G;8:143776668:A/G:8:143776668:A/G;8:143752235:A/G:8:143844650:A/G	45
ENSG00000253196	RP11-706C16.7	143844534	143847845	1	antisense	NA	NA	NA	NA	1	13.355		0.00216471	NA	GTEx/v8/Stomach	-	Yes	GSE86189_GA.po.all.FUMA	1.95086e-09	8:143844650:A/G;8:143776668:A/G;8:143752235:A/G;8:143752235:A/G:8:143844650:A/G	45
ENSG00000180155	LYNX1	143845752	143859640	-1	protein_coding	66004	LYNX1	0.110678246204466	0.158244904	1	13.365		5.44343e-23	1.08354e-38	GTEx/v8/Stomach	-	Yes	GSE86189_GA.po.all.FUMA:GSE86189_GA.pp.all.FUMA	5.02934e-11	8:143844650:A/G;8:143776668:A/G;8:143752235:A/G;8:143776668:A/G:8:143776668:A/G;8:143752235:A/G:8:143844650:A/G	45
ENSG00000167656	LY6D	143866296	143868008	-1	protein_coding	8581	LY6D	0.000620135417616238	NA	1	13.305		NA	NA	NA	NA	Yes	GSE86189_GA.po.all.FUMA:GSE86189_GA.pp.all.FUMA	5.02934e-11	8:143844650:A/G;8:143776668:A/G:8:143776668:A/G;8:143752235:A/G:8:143844650:A/G	45
ENSG00000253715	RP11-706C16.8	143866790	143893536	1	antisense	NA	NA	NA	NA	1	13.305		NA	NA	NA	NA	Yes	GSE86189_GA.po.all.FUMA:GSE86189_GA.pp.all.FUMA	5.02934e-11	8:143844650:A/G;8:143776668:A/G:8:143776668:A/G;8:143752235:A/G:8:143844650:A/G	45
ENSG00000253728	RP11-706C16.5	143915554	143916356	-1	antisense	NA	NA	NA	NA	0	0	0	NA	NA	NA	NA	Yes	GSE86189_GA.po.all.FUMA:GSE86189_GA.pp.all.FUMA	5.02934e-11	8:143776668:A/G:8:143776668:A/G;8:143752235:A/G:8:143844650:A/G	45
ENSG00000104499	GML	143915663	143997922	1	protein_coding	2765	GML	0.439134841904715	0.402881037	0	0	0	NA	NA	NA	NA	Yes	GSE86189_GA.po.all.FUMA:GSE86189_GA.pp.all.FUMA	5.02934e-11	8:143776668:A/G:8:143776668:A/G;8:143752235:A/G:8:143844650:A/G	45
ENSG00000253421	ZNHIT1P1	143939770	143940213	1	pseudogene	100288248	ZNHIT1P1	NA	NA	0	0	0	NA	NA	NA	NA	Yes	GSE86189_GA.po.all.FUMA	1.95086e-09	8:143776668:A/G;8:143752235:A/G:8:143844650:A/G	45
ENSG00000160882	CYP11B1	143953772	143961262	-1	protein_coding	1584	CYP11B1	0.00143393188162045	1.413855756	0	0	0	NA	NA	NA	NA	Yes	GSE86189_GA.po.all.FUMA	1.95086e-09	8:143776668:A/G;8:143752235:A/G:8:143844650:A/G	45

ENSG00000179142	CYP11B2	143991975	143999259	-1	protein_coding	1585	CYP11B2	3.92718052728051e-13	0.351824995	0	0	0	NA	NA	NA	NA	Yes	GSE86189_GA.po.all.FUMA:GSE86189_GA.pp.all.FUMA	5.02934e-11	8:143776668:A/G;8:1437 4576668:A/G;8:143752235:A/G:8:143844650:A/G
ENSG00000247317	RP11-273G15.2	144063155	144099854	-1	lincRNA	NA	NA	NA	NA	0	0	6	0.0263723	NA	GTEx/v8/Stomach	+	Yes	GSE86189_GA.po.all.FUMA:GSE86189_GA.pp.all.FUMA	5.02934e-11	8:143776668:A/G;8:1437 4552235:A/G;8:143844650:A/G;8:143776668:A/G:8:143776668:A/G;8:143752235:A/G:8:143844650:A/G
ENSG00000253971	CDC42P3	144077267	144077899	1	pseudogene	100128627	CDC42P3	NA	NA	0	0	0	NA	NA	NA	NA	Yes	GSE86189_GA.po.all.FUMA	1.95086e-09	8:143776668:A/G;8:1437 4552235:A/G:8:143844650:A/G
ENSG00000160932	LY6E	144099399	144105249	1	protein_coding	4061	LY6E	0.733420181505572	NA	0	0	0	NA	NA	NA	NA	Yes	GSE86189_GA.po.all.FUMA:GSE86189_GA.pp.all.FUMA	5.02934e-11	8:143776668:A/G;8:1437 4576668:A/G;8:143752235:A/G:8:143844650:A/G
ENSG00000177335	C8orf31	144120626	144141359	1	protein_coding	286122	C8orf31	1.08582352031841e-05	1.902115733	0	0	0	NA	NA	NA	NA	Yes	GSE86189_GA.po.all.FUMA:GSE86189_GA.pp.all.FUMA	5.02934e-11	8:143776668:A/G;8:1437 4576668:A/G;8:143752235:A/G:8:143844650:A/G
ENSG00000250115	AK3P2	144138477	144139233	-1	pseudogene	100419074	AK3P2	NA	NA	0	0	0	NA	NA	NA	NA	Yes	GSE86189_GA.po.all.FUMA	1.95086e-09	8:143776668:A/G;8:1437 4552235:A/G:8:143844650:A/G
ENSG00000243328	RP11-520P18.1	144147332	144148004	-1	pseudogene	NA	NA	NA	NA	0	0	0	NA	NA	NA	NA	Yes	GSE86189_GA.po.all.FUMA	1.95086e-09	8:143776668:A/G;8:1437 4552235:A/G:8:143844650:A/G
ENSG00000261667	RP11-520P18.5	144161874	144164418	1	protein_coding	101928108	LY6L	NA	NA	0	0	0	NA	NA	NA	NA	Yes	GSE86189_GA.po.all.FUMA:GSE86189_GA.pp.all.FUMA	5.02934e-11	8:143776668:A/G;8:1437 4576668:A/G;8:143752235:A/G:8:143844650:A/G
ENSG00000176956	LY6H	144239331	144242128	-1	protein_coding	4062	LY6H	0.46060556878133	NA	0	0	0	NA	NA	NA	NA	Yes	GSE86189_GA.po.all.FUMA:GSE86189_GA.pp.all.FUMA	5.02934e-11	8:143776668:A/G;8:1437 4576668:A/G;8:143752235:A/G:8:143844650:A/G
ENSG00000182851	GPIHBP1	144295068	144299044	1	protein_coding	338328	GPIHBP1	0.023780962945537	-0.412250838	0	0	0	NA	NA	NA	NA	Yes	GSE86189_GA.po.all.FUMA:GSE86189_GA.pp.all.FUMA	5.02934e-11	8:143776668:A/G;8:1437 4576668:A/G;8:143752235:A/G:8:143844650:A/G
ENSG00000181638	ZFP41	144328991	144344875	1	protein_coding	286128	ZFP41	0.000671957504039482	1.344096711	0	0	0	NA	NA	NA	NA	Yes	GSE86189_GA.po.all.FUMA:GSE86189_GA.pp.all.FUMA	5.02934e-11	8:143776668:A/G;8:1437 4576668:A/G;8:143752235:A/G:8:143844650:A/G

ENSG00000264668	ZFP41	144329280	144358573	1	protein_coding	286128	ZFP41	NA	1.344096711	0	0	0	NA	NA	NA	NA	Yes	GSE86189_GA.po.all.FUMA:GSE86189_GA.pp.all.FUMA	5.02934e-11	8:143776668:A/G:8:1437 4576668:A/G;8:143752235:A/G:8:143844650:A/G
ENSG00000250571	GLI4	144349603	144359101	1	protein_coding	2738	GLI4	0.00102128735288296	NA	0	0	0	NA	NA	NA	NA	Yes	GSE86189_GA.po.all.FUMA:GSE86189_GA.pp.all.FUMA	5.02934e-11	8:143776668:A/G:8:1437 4576668:A/G;8:143752235:A/G:8:143844650:A/G
ENSG00000253716	RP13-582O9.5	144362331	144363860	-1	antisense	100507316	MINCR	NA	NA	0	0	0	NA	NA	NA	NA	Yes	GSE86189_GA.po.all.FUMA:GSE86189_GA.pp.all.FUMA	5.02934e-11	8:143776668:A/G:8:1437 4576668:A/G;8:143752235:A/G:8:143844650:A/G
ENSG00000185730	ZNF696	144371846	144380231	1	protein_coding	79943	ZNF696	0.00597972241429247	NA	0	0	0	NA	NA	NA	NA	Yes	GSE86189_GA.po.all.FUMA:GSE86189_GA.pp.all.FUMA	5.02934e-11	8:143776668:A/G:8:1437 4576668:A/G;8:143752235:A/G:8:143844650:A/G
ENSG00000272172	RP13-582O9.7	144372569	144372791	-1	antisense	NA	NA	NA	NA	0	0	1	0.0165036	NA	GTEx/v8/Stomach	-	Yes	GSE86189_GA.po.all.FUMA:GSE86189_GA.pp.all.FUMA	5.02934e-11	8:143776668:A/G;8:1437 4576668:A/G:8:143776668:A/G;8:143752235:A/G:8:143844650:A/G
ENSG00000184428	TOP1MT	144386554	144442149	-1	protein_coding	116447	TOP1MT	1.63072339882279e-19	NA	0	0	0	NA	NA	NA	NA	Yes	GSE86189_GA.po.all.FUMA:GSE86189_GA.pp.all.FUMA	5.02934e-11	8:143776668:A/G:8:1437 4576668:A/G;8:143752235:A/G:8:143844650:A/G
ENSG00000263660	AC087793.1	144397091	144397176	1	miRNA	NA	NA	NA	NA	0	0	0	NA	NA	NA	NA	Yes	GSE86189_GA.po.all.FUMA	1.95086e-09	8:143776668:A/G;8:1437 4552235:A/G:8:143844650:A/G
ENSG00000212221	RNU6-220P	144432342	144432448	1	snRNA	106481247	RNU6-220P	NA	NA	0	0	0	NA	NA	NA	NA	Yes	GSE86189_GA.po.all.FUMA	1.95086e-09	8:143776668:A/G;8:1437 4552235:A/G:8:143844650:A/G
ENSG00000254389	RHPN1-AS1	144448801	144450718	-1	antisense	78998	RHPN1-AS1	NA	NA	0	0	0	NA	NA	NA	NA	Yes	GSE86189_GA.po.all.FUMA:GSE86189_GA.pp.all.FUMA	5.02934e-11	8:143776668:A/G:8:1437 4576668:A/G;8:143752235:A/G:8:143844650:A/G
ENSG00000158106	RHPN1	144451057	144466390	1	protein_coding	114822	RHPN1	1.30045250011161e-06	3.262650123	0	0	0	NA	NA	NA	NA	Yes	GSE86189_GA.po.all.FUMA:GSE86189_GA.pp.all.FUMA	5.02934e-11	8:143776668:A/G:8:1437 4576668:A/G;8:143752235:A/G:8:143844650:A/G
ENSG00000253931	RP11-909N17.2	144494919	144499224	1	antisense	NA	NA	NA	NA	0	0	0	NA	NA	NA	NA	Yes	GSE86189_GA.po.all.FUMA:GSE86189_GA.pp.all.FUMA	5.02934e-11	8:143776668:A/G:8:1437 4576668:A/G;8:143752235:A/G:8:143844650:A/G

ENSG00000254338	RP11-909N17.3	144499849	144501320	1	antisense	104326051	MAFA-AS1	NA	NA	0	0	0	NA	NA	NA	NA	Yes	GSE86189_GA.po.all.FUMA:GSE86189_GA.pp.all.FUMA	5.02934e-11	8:143776668:A/G:8:1437 45 76668:A/G;8:143752235:A/G:8:143844650:A/G
ENSG00000182759	MAFA	144501352	144512576	-1	protein_coding	389692	MAFA	NA	NA	0	0	1	0.0238549	NA	GTEx/v8/Stomach	+	Yes	GSE86189_GA.po.all.FUMA:GSE86189_GA.pp.all.FUMA	5.02934e-11	8:143844650:A/G;8:1437 45 76668:A/G:8:143776668:A/G;8:143752235:A/G:8:143844650:A/G
ENSG00000014164	ZC3H3	144519825	144623623	-1	protein_coding	23144	ZC3H3	0.96453876461509	0.541836659	0	0	0	NA	NA	NA	NA	Yes	GSE86189_GA.po.all.FUMA:GSE86189_GA.pp.all.FUMA	5.02934e-11	8:143776668:A/G:8:1437 45 76668:A/G;8:143752235:A/G:8:143844650:A/G
ENSG00000254859	RP11-661A12.5	144624143	144631899	-1	antisense	NA	NA	NA	NA	0	0	0	NA	NA	NA	NA	Yes	GSE86189_GA.po.all.FUMA:GSE86189_GA.pp.all.FUMA	5.02934e-11	8:143776668:A/G:8:1437 45 76668:A/G;8:143752235:A/G:8:143844650:A/G
ENSG00000254144	7SK	144624280	144624570	1	antisense	125050	RN7SK	NA	NA	0	0	0	NA	NA	NA	NA	Yes	GSE86189_GA.po.all.FUMA:GSE86189_GA.pp.all.FUMA	5.02934e-11	8:143776668:A/G:8:1437 45 76668:A/G;8:143752235:A/G:8:143844650:A/G
ENSG00000104518	GSDMD	144635377	144645232	1	protein_coding	79792	GSDMD	4.80694673198233e-07	NA	0	0	0	NA	NA	NA	NA	Yes	GSE86189_GA.po.all.FUMA:GSE86189_GA.pp.all.FUMA	5.02934e-11	8:143776668:A/G:8:1437 45 76668:A/G;8:143752235:A/G:8:143844650:A/G
ENSG00000204839	MROH6	144648357	144655141	-1	protein_coding	642475	MROH6	1.27469283128293e-10	0.994308428	0	0	0	NA	NA	NA	NA	Yes	GSE86189_GA.po.all.FUMA:GSE86189_GA.pp.all.FUMA	5.02934e-11	8:143776668:A/G:8:1437 45 76668:A/G;8:143752235:A/G:8:143844650:A/G
ENSG00000255050	RP11-661A12.9	144655660	144659567	1	antisense	NA	NA	NA	NA	0	0	0	NA	NA	NA	NA	Yes	GSE86189_GA.po.all.FUMA:GSE86189_GA.pp.all.FUMA	5.02934e-11	8:143776668:A/G:8:1437 45 76668:A/G;8:143752235:A/G:8:143844650:A/G
ENSG00000147813	NAPRT1	144656955	144660819	-1	protein_coding	93100	NAPRT1	9.84204377802944e-13	-0.020365279	0	0	0	NA	NA	NA	NA	Yes	GSE86189_GA.po.all.FUMA:GSE86189_GA.pp.all.FUMA	5.02934e-11	8:143776668:A/G:8:1437 45 76668:A/G;8:143752235:A/G:8:143844650:A/G
ENSG00000254741	RP11-661A12.7	144661806	144662840	1	antisense	NA	NA	NA	NA	0	0	0	NA	NA	NA	NA	Yes	GSE86189_GA.po.all.FUMA:GSE86189_GA.pp.all.FUMA	5.02934e-11	8:143776668:A/G:8:1437 45 76668:A/G;8:143752235:A/G:8:143844650:A/G
ENSG00000104529	EEF1D	144661867	144681711	-1	protein_coding	1936	EEF1D	0.394239744215719	NA	0	0	0	NA	NA	NA	NA	Yes	GSE86189_GA.po.all.FUMA:G	5.02934e-11	8:143776668:A/G:8:1437 45 76668:A/G;8:143752235:A/G:8:143844650:A/G

																		SE86189_GA.pp.all.FUMA		
ENSG00000179886	TIGD5	144680074	144682485	1	protein_coding	84948	TIGD5	0.000172428541029168	NA	0	0	0	NA	NA	NA	NA	Yes	GSE86189_GA.po.all.FUMA:GSE86189_GA.pp.all.FUMA	5.02934e-11	8:143776668:A/G:8:1437 45 76668:A/G;8:143752235:A/G:8:143844650:A/G
ENSG00000271912	RP11-661A12.14	144683416	144685394	1	lincRNA	NA	NA	NA	NA	0	0	0	NA	NA	NA	NA	Yes	GSE86189_GA.po.all.FUMA:GSE86189_GA.pp.all.FUMA	5.02934e-11	8:143776668:A/G:8:1437 45 76668:A/G;8:143752235:A/G:8:143844650:A/G
ENSG00000104524	PYCRL	144686083	144691943	-1	protein_coding	65263	PYCRL	0.0171284135571899	-0.571118117	0	0	1	0.0228026	NA	GTEx/v8/Stomach	-	Yes	GSE86189_GA.po.all.FUMA:GSE86189_GA.pp.all.FUMA	5.02934e-11	8:143844650:A/G;8:1437 45 76668:A/G:8:143776668:A/G;8:143752235:A/G:8:143844650:A/G
ENSG00000104522	TSTA3	144694788	144700218	-1	protein_coding	7264	TSTA3	0.0049919240345017	NA	0	0	0	NA	NA	NA	NA	Yes	GSE86189_GA.po.all.FUMA:GSE86189_GA.pp.all.FUMA	5.02934e-11	8:143776668:A/G:8:1437 45 76668:A/G;8:143752235:A/G:8:143844650:A/G
ENSG00000254812	RP11-661A12.12	144714241	144715926	-1	antisense	NA	NA	NA	NA	0	0	0	NA	NA	NA	NA	Yes	GSE86189_GA.po.all.FUMA:GSE86189_GA.pp.all.FUMA	5.02934e-11	8:143776668:A/G:8:1437 45 76668:A/G;8:143752235:A/G:8:143844650:A/G
ENSG00000183309	ZNF623	144718183	144735900	1	protein_coding	9831	ZNF623	0.0941970020128073	0.83729999	0	0	0	NA	NA	NA	NA	Yes	GSE86189_GA.po.all.FUMA:GSE86189_GA.pp.all.FUMA	5.02934e-11	8:143776668:A/G:8:1437 45 76668:A/G;8:143752235:A/G:8:143844650:A/G
ENSG00000252075	RNU7-109P	144728080	144728139	-1	snRNA	NA	RNU7-109P	NA	NA	0	0	0	NA	NA	NA	NA	Yes	GSE86189_GA.po.all.FUMA	1.95086e-09	8:143776668:A/G;8:1437 45 52235:A/G:8:143844650:A/G
ENSG00000181135	ZNF707	144766622	144796068	1	protein_coding	286075	ZNF707	0.0127700852104931	1.683314744	0	0	0	NA	NA	NA	NA	Yes	GSE86189_GA.po.all.FUMA:GSE86189_GA.pp.all.FUMA	5.02934e-11	8:143776668:A/G:8:1437 45 76668:A/G;8:143752235:A/G:8:143844650:A/G
ENSG00000181097	RP11-429J17.2	144778324	144780583	1	antisense	NA	NA	NA	NA	0	0	1	0.0112853	NA	GTEx/v8/Stomach	-	Yes	GSE86189_GA.po.all.FUMA:GSE86189_GA.pp.all.FUMA	5.02934e-11	8:143844650:A/G;8:1437 45 76668:A/G:8:143776668:A/G;8:143752235:A/G:8:143844650:A/G
ENSG00000255181	CCDC166	144788864	144790279	-1	protein_coding	100130274	CCDC166	NA	-0.040246329	0	0	0	NA	NA	NA	NA	Yes	GSE86189_GA.po.all.FUMA:GSE86189_GA.pp.all.FUMA	5.02934e-11	8:143776668:A/G:8:1437 45 76668:A/G;8:143752235:A/G:8:143844650:A/G

ENSG00000254574	RP11-429J17.4	144791177	144795754	-1	antisense	NA	NA	NA	NA	0	0	0	NA	NA	NA	NA	Yes	GSE86189_GA.po.all.FUMA:GSE86189_GA.pp.all.FUMA	5.02934e-11	8:143776668:A/G:8:1437 4576668:A/G;8:143752235:A/G:8:143844650:A/G
ENSG00000181085	MAPK15	144798429	144804628	1	protein_coding	225689	MAPK15	1.28603703609388e-16	NA	0	0	0	NA	NA	NA	NA	Yes	GSE86189_GA.po.all.FUMA:GSE86189_GA.pp.all.FUMA	5.02934e-11	8:143776668:A/G:8:1437 4576668:A/G;8:143752235:A/G:8:143844650:A/G
ENSG00000254548	RP11-429J17.5	144800416	144801061	-1	antisense	NA	NA	NA	NA	0	0	0	NA	NA	NA	NA	Yes	GSE86189_GA.po.all.FUMA	1.95086e-09	8:143776668:A/G;8:1437 4552235:A/G:8:143844650:A/G
ENSG00000180921	FAM83H	144806103	144815971	-1	protein_coding	286077	FAM83H	0.884824710028448	NA	0	0	0	NA	NA	NA	NA	Yes	GSE86189_GA.po.all.FUMA:GSE86189_GA.pp.all.FUMA	5.02934e-11	8:143776668:A/G:8:1437 4576668:A/G;8:143752235:A/G:8:143844650:A/G
ENSG00000265660	MIR4664	144815253	144815323	-1	miRNA	100616318	MIR4664	NA	NA	0	0	0	NA	NA	NA	NA	Yes	GSE86189_GA.po.all.FUMA:GSE86189_GA.pp.all.FUMA	5.02934e-11	8:143776668:A/G:8:1437 4576668:A/G;8:143752235:A/G:8:143844650:A/G
ENSG00000203499	FAM83H-AS1	144816310	144828507	1	lincRNA	100128338	FAM83H-AS1	NA	NA	0	0	0	NA	NA	NA	NA	Yes	GSE86189_GA.po.all.FUMA:GSE86189_GA.pp.all.FUMA	5.02934e-11	8:143776668:A/G:8:1437 4576668:A/G;8:143752235:A/G:8:143844650:A/G
ENSG00000254973	RP11-429J17.7	144840323	144853992	-1	lincRNA	105375800	LOC105375800	NA	NA	0	0	0	NA	NA	NA	NA	Yes	GSE86189_GA.po.all.FUMA	1.95086e-09	8:143776668:A/G;8:1437 4552235:A/G:8:143844650:A/G
ENSG00000214733	RP11-429J17.8	144871090	144872629	1	antisense	NA	NA	NA	NA	0	0	0	NA	NA	NA	NA	Yes	GSE86189_GA.po.all.FUMA:GSE86189_GA.pp.all.FUMA	5.02934e-11	8:143776668:A/G:8:1437 4576668:A/G;8:143752235:A/G:8:143844650:A/G
ENSG00000180900	SCRIB	144873090	144897549	-1	protein_coding	23513	SCRIB	0.91327318061127	NA	0	0	0	NA	NA	NA	NA	Yes	GSE86189_GA.po.all.FUMA:GSE86189_GA.pp.all.FUMA	5.02934e-11	8:143776668:A/G:8:1437 4576668:A/G;8:143752235:A/G:8:143844650:A/G
ENSG00000216090	MIR937	144895127	144895212	-1	miRNA	100126338	MIR937	NA	NA	0	0	0	NA	NA	NA	NA	Yes	GSE86189_GA.po.all.FUMA:GSE86189_GA.pp.all.FUMA	5.02934e-11	8:143776668:A/G:8:1437 4576668:A/G;8:143752235:A/G:8:143844650:A/G
ENSG00000179950	PUF60	144898514	144912029	-1	protein_coding	22827	PUF60	0.849324854511446	-0.807405155	0	0	0	NA	NA	NA	NA	Yes	GSE86189_GA.po.all.FUMA	1.95086e-09	8:143776668:A/G;8:1437 4552235:A/G:8:143844650:A/G
ENSG00000255343	RP11-299M14.2	144915440	144916233	-1	3prime_overlapping_ncrna	NA	NA	NA	NA	0	0	0	NA	NA	NA	NA	Yes	GSE86189_GA.po.all.FUMA	1.95086e-09	8:143776668:A/G;8:1437 4552235:A/G:8:143844650:A/G

ENSG00000185189	NRBP2	144915764	144924200	-1	protein_coding	340371	NRBP2	0.00241294545324528	0.015131378	0	0	0	NA	NA	NA	NA	Yes	GSE86189_GA.po.all.FUMA:GSE86189_GA.pp.all.FUMA	5.02934e-11	8:143776668:A/G:8:1437 4576668:A/G;8:143752235:A/G:8:143844650:A/G
ENSG00000264144	AC105049.1	144926240	144926325	1	miRNA	NA	NA	NA	NA	0	0	0	NA	NA	NA	NA	Yes	GSE86189_GA.po.all.FUMA:GSE86189_GA.pp.all.FUMA	5.02934e-11	8:143776668:A/G:8:1437 4576668:A/G;8:143752235:A/G:8:143844650:A/G
ENSG00000227184	EPPK1	144939497	144952632	-1	protein_coding	83481	EPPK1	4.95287341529358e-21	NA	0	0	0	NA	NA	NA	NA	Yes	GSE86189_GA.po.all.FUMA:GSE86189_GA.pp.all.FUMA	5.02934e-11	8:143776668:A/G:8:1437 4576668:A/G;8:143752235:A/G:8:143844650:A/G
ENSG00000178209	PLEC	144989321	145050902	-1	protein_coding	5339	PLEC	0.024936270552365	-0.44044438	0	0	0	NA	NA	NA	NA	Yes	GSE86189_GA.po.all.FUMA:GSE86189_GA.pp.all.FUMA	5.02934e-11	8:143776668:A/G:8:1437 4576668:A/G;8:143752235:A/G:8:143844650:A/G
ENSG00000207574	MIR661	145019359	145019447	-1	miRNA	724031	MIR661	NA	NA	0	0	0	NA	NA	NA	NA	Yes	GSE86189_GA.po.all.FUMA	1.95086e-09	8:143776668:A/G;8:1437 4552235:A/G:8:143844650:A/G
ENSG00000178685	PARP10	145051321	145086940	-1	protein_coding	84875	PARP10	0.00291131564961967	-0.428650279	0	0	0	NA	NA	NA	NA	Yes	GSE86189_GA.po.all.FUMA:GSE86189_GA.pp.all.FUMA	5.02934e-11	8:143776668:A/G:8:1437 4576668:A/G;8:143752235:A/G:8:143844650:A/G
ENSG00000178719	GRINA	145064226	145067583	1	protein_coding	2907	GRINA	0.757342473317658	NA	0	0	0	NA	NA	NA	NA	Yes	GSE86189_GA.po.all.FUMA:GSE86189_GA.pp.all.FUMA	5.02934e-11	8:143776668:A/G:8:1437 4576668:A/G;8:143752235:A/G:8:143844650:A/G
ENSG00000186583	SPATC1	145086582	145101933	1	protein_coding	375686	SPATC1	3.5903133043143e-08	0.129497291	0	0	0	NA	NA	NA	NA	Yes	GSE86189_GA.po.all.FUMA:GSE86189_GA.pp.all.FUMA	5.02934e-11	8:143776668:A/G:8:1437 4576668:A/G;8:143752235:A/G:8:143844650:A/G
ENSG00000204791	CTD-3065J16.6	145104030	145106423	1	antisense	NA	NA	NA	NA	0	0	0	NA	NA	NA	NA	Yes	GSE86189_GA.po.all.FUMA:GSE86189_GA.pp.all.FUMA	5.02934e-11	8:143776668:A/G:8:1437 4576668:A/G;8:143752235:A/G:8:143844650:A/G
ENSG00000178814	OPLAH	145106167	145118735	-1	protein_coding	26873	OPLAH	2.99003379437065e-11	NA	0	0	0	NA	NA	NA	NA	Yes	GSE86189_GA.po.all.FUMA:GSE86189_GA.pp.all.FUMA	5.02934e-11	8:143776668:A/G:8:1437 4576668:A/G;8:143752235:A/G:8:143844650:A/G
ENSG00000255224	CTD-3065J16.9	145132905	145134168	-1	antisense	NA	NA	NA	NA	0	0	0	NA	NA	NA	NA	Yes	GSE86189_GA.po.all.FUMA:GSE86189_GA.pp.all.FUMA	5.02934e-11	8:143776668:A/G:8:1437 4576668:A/G;8:143752235:A/G:8:143844650:A/G

ENSG00000178896	EXOSC4	145133529	145135550	1	protein_coding	54512	EXOSC4	0.0994501752994272	-0.382152458	0	0	0	NA	NA	NA	NA	Yes	GSE86189_GA.po.all.FUMA:GSE86189_GA.pp.all.FUMA	5.02934e-11	8:143776668:A/G:8:1437 45 76668:A/G;8:143752235:A/G:8:143844650:A/G
ENSG00000197858	GPAA1	145137493	145141119	1	protein_coding	8733	GPAA1	2.89309136314948e-07	NA	0	0	0	NA	NA	NA	NA	Yes	GSE86189_GA.po.all.FUMA:GSE86189_GA.pp.all.FUMA	5.02934e-11	8:143776668:A/G:8:1437 45 76668:A/G;8:143752235:A/G:8:143844650:A/G
ENSG00000179091	CYC1	145149930	145152428	1	protein_coding	1537	CYC1	0.979014367766699	NA	0	0	0	NA	NA	NA	NA	Yes	GSE86189_GA.po.all.FUMA:GSE86189_GA.pp.all.FUMA	5.02934e-11	8:143776668:A/G:8:1437 45 76668:A/G;8:143752235:A/G:8:143844650:A/G
ENSG00000179526	SHARPIN	145153536	145163027	-1	protein_coding	81858	SHARPIN	0.0198873358156404	NA	0	0	0	NA	NA	NA	NA	Yes	GSE86189_GA.po.all.FUMA:GSE86189_GA.pp.all.FUMA	5.02934e-11	8:143776668:A/G:8:1437 45 76668:A/G;8:143752235:A/G:8:143844650:A/G
ENSG00000179632	MAF1	145159402	145162514	1	protein_coding	84232	MAF1	0.794024579551437	NA	0	0	0	NA	NA	NA	NA	Yes	GSE86189_GA.po.all.FUMA:GSE86189_GA.pp.all.FUMA	5.02934e-11	8:143776668:A/G:8:1437 45 76668:A/G;8:143752235:A/G:8:143844650:A/G
ENSG00000179698	KIAA1875	145162629	145173218	1	protein_coding	340390	KIAA1875	NA	NA	0	0	0	NA	NA	NA	NA	Yes	GSE86189_GA.po.all.FUMA:GSE86189_GA.pp.all.FUMA	5.02934e-11	8:143776668:A/G:8:1437 45 76668:A/G;8:143752235:A/G:8:143844650:A/G
ENSG00000235173	FAM203A	145192672	145195746	1	protein_coding	51236	FAM203A	NA	NA	0	0	0	NA	NA	NA	NA	Yes	GSE86189_GA.po.all.FUMA:GSE86189_GA.pp.all.FUMA	5.02934e-11	8:143776668:A/G:8:1437 45 76668:A/G;8:143752235:A/G:8:143844650:A/G
ENSG00000227473	TSSK5P1	145196117	145198567	-1	pseudogene	648630	TSSK5P1	NA	NA	0	0	0	NA	NA	NA	NA	Yes	GSE86189_GA.po.all.FUMA:GSE86189_GA.pp.all.FUMA	5.02934e-11	8:143776668:A/G:8:1437 45 76668:A/G;8:143752235:A/G:8:143844650:A/G
ENSG00000179832	MROH1	145202919	145316843	1	protein_coding	727957	MROH1	0.587142254682183	NA	0	0	0	NA	NA	NA	NA	Yes	GSE86189_GA.po.all.FUMA:GSE86189_GA.pp.all.FUMA	5.02934e-11	8:143776668:A/G:8:1437 45 76668:A/G;8:143752235:A/G:8:143844650:A/G
ENSG00000264721	AC104592.1	145210801	145210892	-1	miRNA	NA	NA	NA	NA	0	0	0	NA	NA	NA	NA	Yes	GSE86189_GA.po.all.FUMA	1.95086e-09	8:143776668:A/G;8:1437 45 52235:A/G:8:143844650:A/G
ENSG00000204775	KM-PA-2	145317003	145331153	-1	protein_coding	NA	NA	NA	NA	0	0	0	NA	NA	NA	NA	Yes	GSE86189_GA.po.all.FUMA:GSE86189_GA.pp.all.FUMA	5.02934e-11	8:143776668:A/G:8:1437 45 76668:A/G;8:143752235:A/G:8:143844650:A/G

ENSG00000187786	SCXB	145321517	145323045	1	protein_coding	642658	SCXB	NA	NA	0	0	0	NA	NA	NA	NA	Yes	GSE86189_GA.po.all.FUMA:GSE86189_GA.pp.all.FUMA	5.02934e-11	8:143776668:A/G:8:1437 4576668:A/G;8:143752235:A/G:8:143844650:A/G
ENSG00000230567	FAM203B	145437880	145440835	1	protein_coding	51236	FAM203B	NA	NA	0	0	0	NA	NA	NA	NA	Yes	GSE86189_GA.po.all.FUMA	1.95086e-09	8:143776668:A/G;8:1437 4552235:A/G:8:143844650:A/G
ENSG00000264987	AC110280.1	145456008	145456099	-1	miRNA	NA	NA	NA	NA	0	0	0	NA	NA	NA	NA	Yes	GSE86189_GA.po.all.FUMA	1.95086e-09	8:143776668:A/G;8:1437 4552235:A/G:8:143844650:A/G
ENSG00000204771	CTD-3232M19.2	145462709	145485885	1	pseudogene	NA	NA	NA	NA	0	0	0	NA	NA	NA	NA	Yes	GSE86189_GA.po.all.FUMA	1.95086e-09	8:143776668:A/G;8:1437 4552235:A/G:8:143844650:A/G
ENSG00000170727	BOP1	145486055	145515082	-1	protein_coding	23246	BOP1	0.526839488528947	NA	0	0	0	NA	NA	NA	NA	Yes	GSE86189_GA.po.all.FUMA:GSE86189_GA.pp.all.FUMA	5.02934e-11	8:143776668:A/G:8:1437 4576668:A/G;8:143752235:A/G:8:143844650:A/G
ENSG00000188686	SCXA	145490549	145492470	1	protein_coding	642658	SCXA	NA	NA	0	0	0	NA	NA	NA	NA	Yes	GSE86189_GA.po.all.FUMA:GSE86189_GA.pp.all.FUMA	5.02934e-11	8:143776668:A/G:8:1437 4576668:A/G;8:143752235:A/G:8:143844650:A/G
ENSG00000185122	HSF1	145515280	145538385	1	protein_coding	3297	HSF1	0.586374294516982	NA	0	0	0	NA	NA	NA	NA	Yes	GSE86189_GA.po.all.FUMA:GSE86189_GA.pp.all.FUMA	5.02934e-11	8:143776668:A/G:8:1437 4576668:A/G;8:143752235:A/G:8:143844650:A/G
ENSG00000254690	GS1-393G12.12	145538253	145538801	-1	antisense	NA	NA	NA	NA	0	0	0	NA	NA	NA	NA	Yes	GSE86189_GA.po.all.FUMA:GSE86189_GA.pp.all.FUMA	5.02934e-11	8:143776668:A/G:8:1437 4576668:A/G;8:143752235:A/G:8:143844650:A/G
ENSG00000185000	DGAT1	145539954	145550573	-1	protein_coding	8694	DGAT1	2.90054760621977e-10	NA	0	0	0	NA	NA	NA	NA	Yes	GSE86189_GA.po.all.FUMA:GSE86189_GA.pp.all.FUMA	5.02934e-11	8:143776668:A/G:8:1437 4576668:A/G;8:143752235:A/G:8:143844650:A/G
ENSG00000170616	SCRT1	145554228	145559943	-1	protein_coding	83482	SCRT1	0.294251146505632	NA	0	0	0	NA	NA	NA	NA	Yes	GSE86189_GA.po.all.FUMA:GSE86189_GA.pp.all.FUMA	5.02934e-11	8:143776668:A/G:8:1437 4576668:A/G;8:143752235:A/G:8:143844650:A/G
ENSG00000214597	TMEM249	145575878	145578574	-1	protein_coding	340393	TMEM249	0.341068399494477	-0.291697706	0	0	0	NA	NA	NA	NA	Yes	GSE86189_GA.po.all.FUMA:GSE86189_GA.pp.all.FUMA	5.02934e-11	8:143776668:A/G:8:1437 4576668:A/G;8:143752235:A/G:8:143844650:A/G
ENSG00000271698	GS1-393G12.13	145576888	145579269	-1	protein_coding	NA	NA	NA	NA	0	0	0	NA	NA	NA	NA	Yes	GSE86189_GA.po.all.FUMA:G	5.02934e-11	8:143776668:A/G:8:1437 4576668:A/G;8:143752235:A/G:8:143844650:A/G

																		SE86189_GA.pp.all.FUMA		
ENSG00000185803	SLC52A2	145577795	145584932	1	protein_coding	79581	SLC52A2	0.0119600051418378	NA	0	0	0	NA	NA	NA	NA	Yes	GSE86189_GA.po.all.FUMA:GSE86189_GA.pp.all.FUMA	5.02934e-11	8:143776668:A/G:8:1437 4576668:A/G;8:143752235:A/G:8:143844650:A/G
ENSG00000182325	FBXL6	145579091	145583036	-1	protein_coding	26233	FBXL6	3.69520806815842e-06	NA	0	0	0	NA	NA	NA	NA	Yes	GSE86189_GA.po.all.FUMA:GSE86189_GA.pp.all.FUMA	5.02934e-11	8:143776668:A/G:8:1437 4576668:A/G;8:143752235:A/G:8:143844650:A/G
ENSG00000173137	ADCK5	145596790	145618457	1	protein_coding	203054	ADCK5	0.908275225235627	NA	0	0	0	NA	NA	NA	NA	Yes	GSE86189_GA.po.all.FUMA:GSE86189_GA.pp.all.FUMA	5.02934e-11	8:143776668:A/G:8:1437 4576668:A/G;8:143752235:A/G:8:143844650:A/G
ENSG00000071894	CPSF1	145618444	145634753	-1	protein_coding	29894	CPSF1	0.0011044541536769	NA	0	0	0	NA	NA	NA	NA	Yes	GSE86189_GA.po.all.FUMA:GSE86189_GA.pp.all.FUMA	5.02934e-11	8:143776668:A/G:8:1437 4576668:A/G;8:143752235:A/G:8:143844650:A/G
ENSG00000216133	MIR939	145619364	145619445	-1	miRNA	100126351	MIR939	NA	NA	0	0	0	NA	NA	NA	NA	Yes	GSE86189_GA.po.all.FUMA	1.95086e-09	8:143776668:A/G;8:1437 4552235:A/G:8:143844650:A/G
ENSG00000221802	MIR1234	145625476	145625559	-1	miRNA	100302196	MIR1234	NA	NA	0	0	0	NA	NA	NA	NA	Yes	GSE86189_GA.po.all.FUMA	1.95086e-09	8:143776668:A/G;8:1437 4552235:A/G:8:143844650:A/G
ENSG00000272115	GS1-393G12.14	145634876	145635360	1	antisense	NA	NA	NA	NA	0	0	0	NA	NA	NA	NA	Yes	GSE86189_GA.po.all.FUMA:GSE86189_GA.pp.all.FUMA	5.02934e-11	8:143776668:A/G:8:1437 4576668:A/G;8:143752235:A/G:8:143844650:A/G
ENSG00000147804	SLC39A4	145635126	145642279	-1	protein_coding	55630	SLC39A4	0.00573736084471825	-0.104491152	0	0	0	NA	NA	NA	NA	Yes	GSE86189_GA.po.all.FUMA:GSE86189_GA.pp.all.FUMA	5.02934e-11	8:143776668:A/G:8:1437 4576668:A/G;8:143752235:A/G:8:143844650:A/G
ENSG00000160948	VPS28	145649000	145653931	-1	protein_coding	51160	VPS28	0.115974874224198	NA	0	0	0	NA	NA	NA	NA	Yes	GSE86189_GA.po.all.FUMA:GSE86189_GA.pp.all.FUMA	5.02934e-11	8:143776668:A/G:8:1437 4576668:A/G;8:143752235:A/G:8:143844650:A/G
ENSG00000160949	TONSL	145654165	145669827	-1	protein_coding	4796	TONSL	6.06324987077416e-05	NA	0	0	0	NA	NA	NA	NA	Yes	GSE86189_GA.po.all.FUMA:GSE86189_GA.pp.all.FUMA	5.02934e-11	8:143776668:A/G:8:1437 4576668:A/G;8:143752235:A/G:8:143844650:A/G
ENSG00000232600	AC084125.4	145660602	145665354	1	antisense	NA	NA	NA	NA	0	0	0	NA	NA	NA	NA	Yes	GSE86189_GA.po.all.FUMA:GSE86189_GA.pp.all.FUMA	5.02934e-11	8:143776668:A/G:8:1437 4576668:A/G;8:143752235:A/G:8:143844650:A/G

ENSG00000187954	CYHR1	145674965	145691060	-1	protein_coding	50626	CYHR1	0.0538307875149217	0.478412825	0	0	0	NA	NA	NA	NA	Yes	GSE86189_GA.po.all.FUMA:GSE86189_GA.pp.all.FUMA	5.02934e-11	8:143776668:A/G:8:1437 4576668:A/G;8:143752235:A/G:8:143844650:A/G
ENSG00000254578	CTD-2517M22.16	145689200	145690484	1	antisense	NA	NA	NA	NA	0	0	0	NA	NA	NA	NA	Yes	GSE86189_GA.po.all.FUMA:GSE86189_GA.pp.all.FUMA	5.02934e-11	8:143776668:A/G:8:1437 4576668:A/G;8:143752235:A/G:8:143844650:A/G
ENSG00000167702	KIFC2	145691426	145699585	1	protein_coding	90990	KIFC2	0.00134057888635577	NA	0	0	0	NA	NA	NA	NA	Yes	GSE86189_GA.po.all.FUMA:GSE86189_GA.pp.all.FUMA	5.02934e-11	8:143776668:A/G:8:1437 4576668:A/G;8:143752235:A/G:8:143844650:A/G
ENSG00000160973	FOXH1	145698795	145701718	-1	protein_coding	8928	FOXH1	0.0132911961348389	-0.725512722	0	0	0	NA	NA	NA	NA	Yes	GSE86189_GA.po.all.FUMA:GSE86189_GA.pp.all.FUMA	5.02934e-11	8:143776668:A/G:8:1437 4576668:A/G;8:143752235:A/G:8:143844650:A/G
ENSG00000160972	PPP1R16A	145703352	145727504	1	protein_coding	84988	PPP1R16A	0.0476703373113261	-0.308870257	0	0	0	NA	NA	NA	NA	Yes	GSE86189_GA.po.all.FUMA:GSE86189_GA.pp.all.FUMA	5.02934e-11	8:143776668:A/G:8:1437 4576668:A/G;8:143752235:A/G:8:143844650:A/G
ENSG00000255182	CTD-2517M22.14	145721001	145730827	-1	processed_transcript	NA	NA	NA	NA	0	0	0	NA	NA	NA	NA	Yes	GSE86189_GA.po.all.FUMA:GSE86189_GA.pp.all.FUMA	5.02934e-11	8:143776668:A/G:8:1437 4576668:A/G;8:143752235:A/G:8:143844650:A/G
ENSG00000261139	CTD-2517M14.5	145721763	145725663	-1	processed_transcript	NA	NA	NA	NA	0	0	0	NA	NA	NA	NA	Yes	GSE86189_GA.po.all.FUMA:GSE86189_GA.pp.all.FUMA	5.02934e-11	8:143776668:A/G:8:1437 4576668:A/G;8:143752235:A/G:8:143844650:A/G
ENSG00000167701	GPT	145728356	145732557	1	protein_coding	2875	GPT	2.84324204580575e-15	-0.01188789	0	0	0	NA	NA	NA	NA	Yes	GSE86189_GA.po.all.FUMA:GSE86189_GA.pp.all.FUMA	5.02934e-11	8:143776668:A/G:8:1437 4576668:A/G;8:143752235:A/G:8:143844650:A/G
ENSG00000167700	MFSD3	145734457	145736596	1	protein_coding	113655	MFSD3	2.01615229921387e-08	NA	0	0	0	NA	NA	NA	NA	Yes	GSE86189_GA.po.all.FUMA:GSE86189_GA.pp.all.FUMA	5.02934e-11	8:143776668:A/G:8:1437 4576668:A/G;8:143752235:A/G:8:143844650:A/G
ENSG00000160957	RECQL4	145736667	145743229	-1	protein_coding	9401	RECQL4	NA	NA	0	0	0	NA	NA	NA	NA	Yes	GSE86189_GA.po.all.FUMA:GSE86189_GA.pp.all.FUMA	5.02934e-11	8:143776668:A/G:8:1437 4576668:A/G;8:143752235:A/G:8:143844650:A/G
ENSG00000265393	CTD-2517M22.17	145737950	145739056	1	antisense	NA	NA	NA	NA	0	0	0	NA	NA	NA	NA	Yes	GSE86189_GA.po.all.FUMA:GSE86189_GA.pp.all.FUMA	5.02934e-11	8:143776668:A/G:8:1437 4576668:A/G;8:143752235:A/G:8:143844650:A/G

ENSG00000160959	LRRC14	145743376	145750557	1	protein_coding	9684	LRRC14	0.00665273421562502	NA	0	0	0	NA	NA	NA	NA	Yes	GSE86189_GA.po.all.FUMA:GSE86189_GA.pp.all.FUMA	5.02934e-11	8:143776668:A/G:8:143776668:A/G;8:143752235:A/G:8:143844650:A/G	45
ENSG00000254402	LRRC24	145747761	145752416	-1	protein_coding	441381	LRRC24	0.775315502193512	NA	0	0	0	NA	NA	NA	NA	Yes	GSE86189_GA.po.all.FUMA:GSE86189_GA.pp.all.FUMA	5.02934e-11	8:143776668:A/G:8:143776668:A/G;8:143752235:A/G:8:143844650:A/G	45
ENSG00000213563	C8orf82	145751117	145754516	-1	protein_coding	414919	C8orf82	0.00666763298298849	NA	0	0	0	NA	NA	NA	NA	Yes	GSE86189_GA.po.all.FUMA:GSE86189_GA.pp.all.FUMA	5.02934e-11	8:143776668:A/G:8:143776668:A/G;8:143752235:A/G:8:143844650:A/G	45
ENSG00000255681	AC084125.1	145774891	145775057	1	pseudogene	NA	NA	NA	NA	0	0	0	NA	NA	NA	NA	Yes	GSE86189_GA.po.all.FUMA	1.95086e-09	8:143776668:A/G;8:143752235:A/G:8:143844650:A/G	45
ENSG00000256822	AC084125.2	145784429	145784599	-1	pseudogene	NA	NA	NA	NA	0	0	0	NA	NA	NA	NA	Yes	GSE86189_GA.po.all.FUMA	1.95086e-09	8:143776668:A/G;8:143752235:A/G:8:143844650:A/G	45
ENSG00000265295	AC084125.3	145792691	145792776	1	miRNA	NA	NA	NA	NA	0	0	0	NA	NA	NA	NA	Yes	GSE86189_GA.po.all.FUMA	1.95086e-09	8:143776668:A/G;8:143752235:A/G:8:143844650:A/G	45
ENSG00000255456	CTD-2517M22.9	145809424	145811872	1	antisense	NA	NA	NA	NA	0	0	0	NA	NA	NA	NA	Yes	GSE86189_GA.po.all.FUMA	2.25351e-07	8:143844650:A/G	45
ENSG00000268364	SMC5-AS1	72808913	72873782	-1	antisense	100507299	SMC5-AS1	NA	NA	0	0	0	NA	NA	NA	NA	Yes	GSE86189_GA.po.all.FUMA	0.0329188	9:74675922:A/G	46
ENSG00000198887	SMC5	72873937	72969804	1	protein_coding	23137	SMC5	0.980364885210913	-0.777329193	0	0	0	NA	NA	NA	NA	Yes	GSE86189_GA.po.all.FUMA	0.0329188	9:74675922:A/G	46
ENSG00000119138	KLF9	72999503	73029540	-1	protein_coding	687	KLF9	0.882452241010758	-1.932843421	0	0	0	NA	NA	NA	NA	Yes	GSE86189_GA.po.all.FUMA	0.0329188	9:74675922:A/G	46
ENSG00000083067	TRPM3	73143979	74061820	-1	protein_coding	80036	TRPM3	7.73754359412344e-06	0.320867397	0	0	0	NA	NA	NA	NA	Yes	GSE86189_GA.po.all.FUMA	0.0329188	9:74675922:A/G	46
ENSG00000135048	TMEM2	74298282	74431606	-1	protein_coding	23670	TMEM2	1.28387737688986e-09	1.442747733	0	0	0	NA	NA	NA	NA	Yes	GSE86189_GA.po.all.FUMA	0.0329188	9:74675922:A/G	46
ENSG00000107362	ABHD17B	74477368	74525847	-1	protein_coding	51104	ABHD17B	0.961448199081918	NA	0	0	0	NA	NA	NA	NA	Yes	GSE86189_GA.po.all.FUMA	0.0329188	9:74675922:A/G	46
ENSG00000155621	C9orf85	74526426	74600970	1	protein_coding	138241	C9orf85	0.00692105108649798	0.417999146	0	0	0	NA	NA	NA	NA	Yes	GSE86189_GA.po.all.FUMA	0.0329188	9:74675922:A/G	46
ENSG00000204669	C9orf57	74666292	74687733	-1	protein_coding	138240	C9orf57	NA	-0.10623724	0	0	0	NA	NA	NA	NA	Yes	GSE86189_GA.po.all.FUMA	0.0329188	9:74675922:A/G	46
ENSG00000119125	GDA	74729511	74872109	1	protein_coding	9615	GDA	0.998845432686572	-0.283357759	1	13.505	0	NA	NA	NA	NA	Yes	GSE86189_GA.po.all.FUMA	0.0329188	9:74675922:A/G	46
ENSG00000107372	ZFAND5	74966341	74980163	-1	protein_coding	7763	ZFAND5	0.91258506652532	0.130844409	0	0	0	NA	NA	NA	NA	Yes	GSE86189_GA.po.all.FUMA	0.0329188	9:74675922:A/G	46

ENSG00000165091	TMC1	75136717	75451267	1	protein_coding	117531	TMC1	3.80770445106551e-07	0.709993682	0	0	0	NA	NA	NA	NA	Yes	GSE86189_GA.po.all.FUMA	0.0329188	9:74675922:A/G	46
ENSG00000238402	snoU13	75142152	75142255	1	snoRNA	NA	NA	NA	NA	0	0	0	NA	NA	NA	NA	Yes	GSE86189_GA.po.all.FUMA	0.0329188	9:74675922:A/G	46
ENSG00000165092	ALDH1A1	75515578	75695358	-1	protein_coding	216	ALDH1A1	0.993850728751138	-0.080946535	0	0	0	NA	NA	NA	NA	Yes	GSE86189_GA.po.all.FUMA	0.0329188	9:74675922:A/G	46
ENSG00000135046	ANXA1	75766673	75785309	1	protein_coding	301	ANXA1	0.000442370109951712	0.218836669	0	0	0	NA	NA	NA	NA	Yes	GSE86189_GA.po.all.FUMA	0.0329188	9:74675922:A/G	46
ENSG00000119912	IDE	94211441	94333833	-1	protein_coding	3416	IDE	0.426771018104833	-0.602024969	0	0	0	NA	NA	NA	NA	Yes	GSE86189_GA.po.all.FUMA	0.0432354	10:96058298:A/G:10:96058298:A/G;10:96066341:A/G;10:96070375:A/G	50
ENSG00000138160	KIF11	94353043	94415150	1	protein_coding	3832	KIF11	0.999996042973183	0.14526449	0	0	0	NA	NA	NA	NA	Yes	GSE86189_GA.po.all.FUMA	0.0432354	10:96058298:A/G:10:96058298:A/G;10:96066341:A/G;10:96070375:A/G	50
ENSG00000152804	HHEX	94447945	94455403	1	protein_coding	3087	HHEX	0.216268139022434	-1.588878356	0	0	0	NA	NA	NA	NA	Yes	GSE86189_GA.po.all.FUMA	0.0432354	10:96058298:A/G:10:96058298:A/G;10:96066341:A/G;10:96070375:A/G	50
ENSG00000138190	EXOC6	94590935	94819250	1	protein_coding	54536	EXOC6	0.0281606351001072	0.013822901	0	0	0	NA	NA	NA	NA	Yes	GSE86189_GA.po.all.FUMA	0.0432354	10:96058298:A/G:10:96058298:A/G;10:96066341:A/G;10:96070375:A/G	50
ENSG00000226425	RP11-348J12.2	94819420	94820183	-1	antisense	NA	NA	NA	NA	0	0	0	NA	NA	NA	NA	Yes	GSE86189_GA.po.all.FUMA	0.0432354	10:96058298:A/G:10:96058298:A/G;10:96066341:A/G;10:96070375:A/G	50
ENSG00000187553	CYP26C1	94821021	94828454	1	protein_coding	340665	CYP26C1	0.0229549008545053	-0.091096245	0	0	0	NA	NA	NA	NA	Yes	GSE86189_GA.po.all.FUMA	0.0432354	10:96058298:A/G:10:96058298:A/G;10:96066341:A/G;10:96070375:A/G	50
ENSG00000095596	CYP26A1	94833232	94837647	1	protein_coding	1592	CYP26A1	6.64311184026187e-05	-0.097008268	0	0	0	NA	NA	NA	NA	Yes	GSE86189_GA.po.all.FUMA	0.0432354	10:96058298:A/G:10:96058298:A/G;10:96066341:A/G;10:96070375:A/G	50
ENSG00000138119	MYOF	95066186	95242074	-1	protein_coding	26509	MYOF	2.08401244116976e-36	-0.140295777	0	0	0	NA	NA	NA	NA	Yes	GSE86189_GA.po.all.FUMA	0.0432354	10:96058298:A/G:10:96058298:A/G;10:96066341:A/G;10:96070375:A/G	50
ENSG00000138180	CEP55	95256389	95288849	1	protein_coding	55165	CEP55	1.30065957563854e-07	0.140097025	0	0	0	NA	NA	NA	NA	Yes	GSE86189_GA.po.all.FUMA	0.0432354	10:96058298:A/G:10:96058298:A/G;10:96066341:A/G;10:96070375:A/G	50
ENSG00000186188	FFAR4	95326422	95364237	1	protein_coding	338557	FFAR4	0.0055724932654211	2.243003875	0	0	0	NA	NA	NA	NA	Yes	GSE86189_GA.po.all.FUMA	0.0432354	10:96058298:A/G:10:96058298:A/G;10:96066341:A/G;10:96070375:A/G	50
ENSG00000138207	RBP4	95351444	95361501	-1	protein_coding	5950	RBP4	0.57861895575558	-0.611735994	0	0	1	0.00915895	NA	GTEx/v8/Stomach	+	Yes	GSE86189_GA.po.all.FUMA	0.0432354	10:96058298:A/G;10:96058298:A/G:10:96058298:A/G;10:96066341:A/G;10:96070375:A/G	50

ENSG00000221352	AL356214.1	95368343	95368456	1	miRNA	NA	NA	NA	NA	0	0	0	NA	NA	NA	NA	Yes	GSE86189_GA.po.all.FUMA	0.0432354	10:96058298:A/G:10:96058298:A/G;10:96066341:A/G;10:96070375:A/G	50
ENSG00000095464	PDE6C	95372345	95425767	1	protein_coding	5146	PDE6C	7.4249966167879e-08	0.073360413	0	0	0	NA	NA	NA	NA	Yes	GSE86189_GA.po.all.FUMA	0.0432354	10:96058298:A/G:10:96058298:A/G;10:96066341:A/G;10:96070375:A/G	50
ENSG00000148690	FRA10AC1	95427640	95462329	-1	protein_coding	118924	FRA10AC1	9.71537508229325e-10	-0.338090023	0	0	0	NA	NA	NA	NA	Yes	GSE86189_GA.po.all.FUMA	0.0432354	10:96058298:A/G:10:96058298:A/G;10:96066341:A/G;10:96070375:A/G	50
ENSG00000108231	LGI1	95517566	95557916	1	protein_coding	9211	LGI1	0.997175814162011	-0.476433397	0	0	1	0.0341501	NA	GTEx/v8/Stomach	+	Yes	GSE86189_GA.po.all.FUMA	0.0432354	10:96058298:A/G;10:96058298:A/G:10:96058298:A/G;10:96066341:A/G;10:96070375:A/G	50
ENSG00000176273	SLC35G1	95653730	95715819	1	protein_coding	159371	SLC35G1	0.917135938028142	-0.68831192	0	0	0	NA	NA	NA	NA	Yes	GSE86189_GA.po.all.FUMA	0.0432354	10:96058298:A/G:10:96058298:A/G;10:96066341:A/G;10:96070375:A/G	50
ENSG00000138193	PLCE1	95753746	96092580	1	protein_coding	51196	PLCE1	0.204555596267865	NA	2	19.20		NA	NA	NA	NA	Yes	GSE86189_GA.po.all.FUMA	0.0432354	10:96058298:A/G;10:96066341:A/G;10:96070375:A/G;10:96058298:A/G:10:96058298:A/G	50
ENSG00000268894	PLCE1-AS1	96039034	96046828	-1	antisense	100128054	PLCE1-AS1	NA	NA	2	19.20		NA	NA	NA	NA	No	NA	0.0432354	10:96058298:A/G;10:96066341:A/G;10:96070375:A/G	50
ENSG00000273450	RP11-76P2.4	96074664	96075084	-1	antisense	NA	NA	NA	NA	2	19.20		NA	NA	NA	NA	No	NA	0.0432354	10:96058298:A/G;10:96066341:A/G;10:96070375:A/G	50
ENSG00000173145	NOC3L	96075004	96122716	-1	protein_coding	64318	NOC3L	1.14013419227493e-06	1.122626106	2	19.22		9.11868e-07	5.3296 1e-07	GTEx/v8/Stomach	+	Yes	GSE86189_GA.po.all.FUMA	0.0432354	10:96058298:A/G;10:96066341:A/G;10:96070375:A/G;10:96058298:A/G:10:96058298:A/G	50
ENSG00000108239	TBC1D12	96162261	96295687	1	protein_coding	23232	TBC1D12	0.00264365020875562	0.292901819	0	0	0	NA	NA	NA	NA	Yes	GSE86189_GA.po.all.FUMA	0.0432354	10:96058298:A/G:10:96058298:A/G;10:96066341:A/G;10:96070375:A/G	50
ENSG00000119969	HELLS	96305547	96373662	1	protein_coding	3070	HELLS	0.999418431126719	0.065854322	0	0	0	NA	NA	NA	NA	Yes	GSE86189_GA.po.all.FUMA	0.0432354	10:96058298:A/G:10:96058298:A/G;10:96066341:A/G;10:96070375:A/G	50
ENSG00000108242	CYP2C18	96443251	96495947	1	protein_coding	1562	CYP2C18	5.267291853526e-08	0.613728973	0	0	0	NA	NA	NA	NA	Yes	GSE86189_GA.po.all.FUMA	0.0432354	10:96058298:A/G:10:96058298:A/G;10:96066341:A/G;10:96070375:A/G	50
ENSG00000165841	CYP2C19	96447911	96613017	1	protein_coding	1557	CYP2C19	2.53768601148421e-10	-0.174574379	0	0	0	NA	NA	NA	NA	Yes	GSE86189_GA.po.all.FUMA	0.0432354	10:96058298:A/G:10:96058298:A/G;10:96066341:A/G;10:96070375:A/G	50

ENSG00000138109	CYP2C9	96698415	96749147	1	protein_coding	1559	CYP2C9	2.53599347417585e-08	0.077353466	0	0	1	0.0343056	NA	GTEx/v8/Stomach	+	Yes	GSE86189_GA.po.all.FUMA	0.0432354	10:96058298:A/G;10:96066341:A/G;10:96070375:A/G;10:96058298:A/G:10:96058298:A/G	50
ENSG00000233377	MTND4P20	96700010	96700878	-1	pseudogene	100873352	MTND4P20	NA	NA	0	0	0	NA	NA	NA	NA	Yes	GSE86189_GA.po.all.FUMA	0.0432354	10:96058298:A/G:10:96058298:A/G;10:96066341:A/G;10:96070375:A/G	50
ENSG00000138115	CYP2C8	96796530	96829254	-1	protein_coding	1558	CYP2C8	5.35039546572966e-12	0.073211053	0	0	2	0.00456084	NA	GTEx/v8/Stomach	+	Yes	GSE86189_GA.po.all.FUMA	0.0432354	10:96058298:A/G;10:96066341:A/G;10:96070375:A/G;10:96058298:A/G:10:96058298:A/G	50
ENSG00000173124	C10orf129	96953957	96988685	1	protein_coding	142827	C10orf129	0.0112101925841759	-0.050977718	0	0	0	NA	NA	NA	NA	Yes	GSE86189_GA.po.all.FUMA	0.0432354	10:96058298:A/G:10:96058298:A/G;10:96066341:A/G;10:96070375:A/G	50
ENSG00000107438	PDLIM1	96997329	97050781	-1	protein_coding	9124	PDLIM1	1.38008290255861e-05	NA	0	0	0	NA	NA	NA	NA	Yes	GSE86189_GA.po.all.FUMA	0.0432354	10:96058298:A/G:10:96058298:A/G;10:96066341:A/G;10:96070375:A/G	50
ENSG00000095637	SORBS1	97071528	97321171	-1	protein_coding	10580	SORBS1	2.14384718511001e-08	1.928613752	0	0	0	NA	NA	NA	NA	Yes	GSE86189_GA.po.all.FUMA	0.0432354	10:96058298:A/G:10:96058298:A/G;10:96066341:A/G;10:96070375:A/G	50
ENSG00000059573	ALDH18A1	97365696	97416463	-1	protein_coding	5832	ALDH18A1	0.855933081745869	-0.050105021	0	0	0	NA	NA	NA	NA	Yes	GSE86189_GA.po.all.FUMA	0.0432354	10:96058298:A/G:10:96058298:A/G;10:96066341:A/G;10:96070375:A/G	50
ENSG00000119977	TCTN3	97423158	97453900	-1	protein_coding	26123	TCTN3	6.43975615999618e-08	-0.133500764	0	0	0	NA	NA	NA	NA	Yes	GSE86189_GA.po.all.FUMA	0.0432354	10:96058298:A/G:10:96058298:A/G;10:96066341:A/G;10:96070375:A/G	50
ENSG00000138185	ENTPD1	97471536	97637023	1	protein_coding	953	ENTPD1	0.519393999413089	-1.085439194	0	0	0	NA	NA	NA	NA	Yes	GSE86189_GA.po.all.FUMA	0.0432354	10:96058298:A/G:10:96058298:A/G;10:96066341:A/G;10:96070375:A/G	50
ENSG00000270099	RP11-248J23.7	97620305	97684601	1	protein_coding	NA	NA	NA	NA	0	0	0	NA	NA	NA	NA	Yes	GSE86189_GA.po.all.FUMA	0.0432354	10:96058298:A/G:10:96058298:A/G;10:96066341:A/G;10:96070375:A/G	50
ENSG00000173088	C10orf131	97667360	97698480	1	protein_coding	387707	C10orf131	NA	1.318571629	0	0	0	NA	NA	NA	NA	Yes	GSE86189_GA.po.all.FUMA	0.0432354	10:96058298:A/G:10:96058298:A/G;10:96066341:A/G;10:96070375:A/G	50
ENSG00000269948	RP11-248J23.6	97709725	97744785	1	protein_coding	NA	NA	NA	NA	0	0	0	NA	NA	NA	NA	Yes	GSE86189_GA.po.all.FUMA	0.0432354	10:96058298:A/G:10:96058298:A/G;10:96066341:A/G;10:96070375:A/G	50
ENSG00000188649	CC2D2B	97733786	97792441	1	protein_coding	387707	CC2D2B	2.5176722476543e-05	-0.041520194	0	0	0	NA	NA	NA	NA	Yes	GSE86189_GA.po.all.FUMA	0.0432354	10:96058298:A/G:10:96058298:A/G;10:96066341:A/G;10:96070375:A/G	50

ENSG00000269920	RP11-690P14.4	97733786	97792432	1	protein_coding	NA	NA	NA	NA	0	0	0	NA	NA	NA	NA	Yes	GSE86189_GA.po.all.FUMA	0.0432354	10:96058298:A/G:10:96058298:A/G;10:96066341:A/G;10:96070375:A/G	50
ENSG00000107443	CCNJ	97803151	97820627	1	protein_coding	54619	CCNJ	0.983940395518084	-0.222999335	0	0	0	NA	NA	NA	NA	Yes	GSE86189_GA.po.all.FUMA	0.0432354	10:96058298:A/G:10:96058298:A/G;10:96066341:A/G;10:96070375:A/G	50
ENSG00000095585	BLNK	97951458	98031344	-1	protein_coding	29760	BLNK	0.990011101848006	0.063424178	0	0	0	NA	NA	NA	NA	Yes	GSE86189_GA.po.all.FUMA	0.0432354	10:96058298:A/G:10:96058298:A/G;10:96066341:A/G;10:96070375:A/G	50
ENSG00000229418	RP11-35J23.1	98052442	98066452	-1	antisense	NA	NA	NA	NA	0	0	0	NA	NA	NA	NA	Yes	GSE86189_GA.po.all.FUMA	0.0432354	10:96058298:A/G;10:96066341:A/G;10:96070375:A/G	50
ENSG00000107447	DNTT	98064085	98098321	1	protein_coding	1791	DNTT	8.53802251177926e-05	0.258479961	0	0	0	NA	NA	NA	NA	Yes	GSE86189_GA.po.all.FUMA	0.0432354	10:96058298:A/G;10:96066341:A/G;10:96070375:A/G	50
ENSG00000171204	TMEM126B	85339629	85347580	1	protein_coding	55863	TMEM126B	0.000531075640903117	0.407304023	0	0	1	0.0429655	NA	GTEx/v8/Stomach	NA	No	NA	NA	11:85033761:A/G	53
ENSG00000120645	IQSEC3	175931	287626	1	protein_coding	440073	IQSEC3	0.925104239220891	1.476521509	0	0	0	NA	NA	NA	NA	Yes	GSE86189_GA.po.all.FUMA	0.000121635	12:1485129:C/G	56
ENSG00000111181	SLC6A12	299243	323736	-1	protein_coding	6539	SLC6A12	7.90050068276314e-07	0.848532671	0	0	0	NA	NA	NA	NA	Yes	GSE86189_GA.po.all.FUMA	0.000121635	12:1485129:C/G	56
ENSG00000256577	RP11-283I3.2	312808	314189	1	antisense	101929384	LOC101929384	NA	NA	0	0	0	NA	NA	NA	NA	Yes	GSE86189_GA.po.all.FUMA	0.000121635	12:1485129:C/G	56
ENSG00000010379	SLC6A13	329789	372039	-1	protein_coding	6540	SLC6A13	1.85726644531532e-08	-0.222156827	0	0	0	NA	NA	NA	NA	Yes	GSE86189_GA.po.all.FUMA	0.000121635	12:1485129:C/G	56
ENSG00000255746	RP11-283I3.4	362608	366465	-1	sense_intronic	102723544	LOC102723544	NA	NA	0	0	0	NA	NA	NA	NA	Yes	GSE86189_GA.po.all.FUMA	0.000121635	12:1485129:C/G	56
ENSG00000073614	KDM5A	389295	498620	-1	protein_coding	5927	KDM5A	0.999994827471308	-1.610267207	0	0	0	NA	NA	NA	NA	Yes	GSE86189_GA.po.all.FUMA	0.000121635	12:1485129:C/G	56
ENSG00000120647	CCDC77	498439	551811	1	protein_coding	84318	CCDC77	9.40308683942061e-15	0.089994362	0	0	0	NA	NA	NA	NA	Yes	GSE86189_GA.po.all.FUMA	0.000121635	12:1485129:C/G	56
ENSG00000139044	B4GALNT3	569530	672675	1	protein_coding	283358	B4GALNT3	1.06179854846531e-07	1.746140946	0	0	0	NA	NA	NA	NA	Yes	GSE86189_GA.po.all.FUMA	0.000121635	12:1485129:C/G	56
ENSG00000171840	NINJ2	673462	772945	-1	protein_coding	4815	NINJ2	0.0201388621613476	0.878754913	0	0	0	NA	NA	NA	NA	Yes	GSE86189_GA.po.all.FUMA	0.000121635	12:1485129:C/G	56
ENSG00000060237	WNK1	861759	1020618	1	protein_coding	65125	WNK1	0.999999999364508	-1.061984083	0	0	0	NA	NA	NA	NA	Yes	GSE86189_GA.po.all.FUMA	0.000121635	12:1485129:C/G	56
ENSG00000002016	RAD52	1021243	1099219	-1	protein_coding	5893	RAD52	2.38441629175774e-07	1.602620466	0	0	0	NA	NA	NA	NA	Yes	GSE86189_GA.po.all.FUMA	0.000121635	12:1485129:C/G	56
ENSG00000250132	RP11-359B12.2	1083299	1100356	-1	antisense	NA	NA	NA	NA	0	0	0	NA	NA	NA	NA	Yes	GSE86189_GA.po.all.FUMA	0.000121635	12:1485129:C/G	56

ENSG00000082805	ERC1	1099675	1605099	1	protein_coding	23085	ERC1	0.00412388322590132	1.00578732	1	12.318		0.0490853	NA	GTEx/v8/Stomach	+	Yes	GSE86189_GA.po.all.FUMA	0.000121635	12:1485129:C/G	56
ENSG00000249628	LINC00942	1609691	1616484	-1	lincRNA	100292680	LINC00942	NA	NA	0	0	1	0.0155409	NA	GTEx/v8/Stomach	+	No	NA	0.000121635	12:1485129:C/G	56
ENSG00000111186	WNT5B	1639057	1756409	1	protein_coding	81029	WNT5B	0.835340308837053	-0.105698207	0	0	0	NA	NA	NA	NA	Yes	GSE86189_GA.po.all.FUMA	0.000121635	12:1485129:C/G	56
ENSG00000171823	FBXL14	1675159	1703331	-1	protein_coding	144699	FBXL14	0.40527008579802	NA	0	0	1	0.0177894	NA	GTEx/v8/Stomach	-	Yes	GSE86189_GA.po.all.FUMA	0.000121635	12:1485129:C/G	56
ENSG00000006831	ADIPOR2	1797740	1897844	1	protein_coding	79602	ADIPOR2	0.810597984007902	0.190570438	0	0	0	NA	NA	NA	NA	Yes	GSE86189_GA.po.all.FUMA	0.000121635	12:1485129:C/G	56
ENSG00000243663	RPS4XP14	1866397	1867177	-1	pseudogene	729097	RPS4XP14	NA	NA	0	0	1	0.0473	NA	GTEx/v8/Stomach	-	No	NA	0.000121635	12:1485129:C/G	56
ENSG00000151062	CACNA2D4	1901123	2028002	-1	protein_coding	93589	CACNA2D4	4.57168482146793e-22	0.980801755	0	0	0	NA	NA	NA	NA	Yes	GSE86189_GA.po.all.FUMA	0.000121635	12:1485129:C/G	56
ENSG00000166159	LRTM2	1929433	1945918	1	protein_coding	654429	LRTM2	0.739704570731645	0.216122806	0	0	0	NA	NA	NA	NA	Yes	GSE86189_GA.po.all.FUMA	0.000121635	12:1485129:C/G	56
ENSG00000256706	RP5-1096D14.3	2027117	2032033	1	antisense	NA	NA	NA	NA	0	0	0	NA	NA	NA	NA	Yes	GSE86189_GA.po.all.FUMA	0.000121635	12:1485129:C/G	56
ENSG00000151065	DCP1B	2055220	2113701	-1	protein_coding	196513	DCP1B	5.3210820854203e-07	0.164044189	0	0	0	NA	NA	NA	NA	Yes	GSE86189_GA.po.all.FUMA	0.000121635	12:1485129:C/G	56
ENSG00000151067	CACNA1C	2079952	2802108	1	protein_coding	775	CACNA1C	0.99999843310185	-0.35401516	0	0	0	NA	NA	NA	NA	Yes	GSE86189_GA.po.all.FUMA	0.000121635	12:1485129:C/G	56
ENSG00000203593	RP5-1096D14.6	2113832	2120558	1	antisense	NA	NA	NA	NA	0	0	0	NA	NA	NA	NA	Yes	GSE86189_GA.po.all.FUMA	0.000121635	12:1485129:C/G	56
ENSG00000256030	CBX3P4	2896097	2903461	-1	pseudogene	100873792	CBX3P4	NA	NA	0	0	0	NA	NA	NA	NA	Yes	GSE86189_GA.po.all.FUMA	0.000121635	12:1485129:C/G	56
ENSG00000004478	FKBP4	2904119	2914576	1	protein_coding	2288	FKBP4	0.927473130653813	NA	0	0	0	NA	NA	NA	NA	Yes	GSE86189_GA.po.all.FUMA	0.000121635	12:1485129:C/G	56
ENSG00000258325	RP4-816N1.6	2906043	2922068	-1	antisense	283440	ITFG2-AS1	NA	NA	0	0	0	NA	NA	NA	NA	Yes	GSE86189_GA.po.all.FUMA	0.000121635	12:1485129:C/G	56
ENSG00000111203	ITFG2	2921788	2968957	1	protein_coding	55846	ITFG2	1.38956896751476e-06	-0.528046936	0	0	0	NA	NA	NA	NA	Yes	GSE86189_GA.po.all.FUMA	0.000121635	12:1485129:C/G	56

ENSG00000053702	NRIP2	2934514	2944710	-1	protein_coding	83714	NRIP2	9.12047384069789e-08	0.195407213	0	0	0	NA	NA	NA	NA	Yes	GSE86189_GA.po.all.FUMA	0.000121635	12:1485129:C/G	56
ENSG00000206044	AC005841.1	2958397	2966213	-1	protein_coding	NA	NA	NA	NA	0	0	0	NA	NA	NA	NA	Yes	GSE86189_GA.po.all.FUMA	0.000121635	12:1485129:C/G	56
ENSG00000111206	FOXM1	2966847	2986206	-1	protein_coding	2305	FOXM1	0.802589769159313	-0.427304082	0	0	0	NA	NA	NA	NA	Yes	GSE86189_GA.po.all.FUMA	0.000121635	12:1485129:C/G	56
ENSG00000222493	Y_RNA	2969115	2969199	1	misc_RNA	NA	NA	NA	NA	0	0	0	NA	NA	NA	NA	Yes	GSE86189_GA.po.all.FUMA	0.000121635	12:1485129:C/G	56
ENSG00000171792	RHNO1	2985424	2998626	1	protein_coding	83695	RHNO1	0.000558184641145266	0.237847533	0	0	0	NA	NA	NA	NA	Yes	GSE86189_GA.po.all.FUMA	0.000121635	12:1485129:C/G	56
ENSG00000078246	TULP3	2986389	3050306	1	protein_coding	7289	TULP3	0.00103688890759594	-0.477883715	0	0	0	NA	NA	NA	NA	Yes	GSE86189_GA.po.all.FUMA	0.000121635	12:1485129:C/G	56
ENSG00000252996	RNU6-1315P	3063406	3063504	-1	snRNA	106481950	RNU6-1315P	NA	NA	0	0	0	NA	NA	NA	NA	Yes	GSE86189_GA.po.all.FUMA	0.000121635	12:1485129:C/G	56
ENSG00000197905	TEAD4	3068496	3149839	1	protein_coding	7004	TEAD4	NA	NA	0	0	0	NA	NA	NA	NA	Yes	GSE86189_GA.po.all.FUMA	0.000121635	12:1485129:C/G	56
ENSG00000011105	TSPAN9	3186521	3395730	1	protein_coding	10867	TSPAN9	0.779777700780573	1.653562548	0	0	0	NA	NA	NA	NA	Yes	GSE86189_GA.po.all.FUMA	0.000121635	12:1485129:C/G	56
ENSG00000233175	CTD-2020K17.3	43315395	43319101	-1	antisense	107985040	LOC107985040	NA	NA	0	0	1	0.00311203	NA	GTEx/v8/Stomach	NA	No	NA	NA	17:43962562:A/G;17:43991509:A/C	73
ENSG00000214425	LRRC37A4P	43578685	43627701	-1	pseudogene	55073	LRRC37A4P	NA	NA	0	0	1	0.000325555	NA	GTEx/v8/Stomach	NA	No	NA	NA	17:43962562:A/G;17:43991509:A/C	73
ENSG00000266918	RP11-798G7.8	43608943	43611204	1	lincRNA	NA	NA	NA	NA	0	0	1	0.0180512	NA	GTEx/v8/Stomach	NA	No	NA	NA	17:43962562:A/G;17:43991509:A/C	73
ENSG00000264070	DND1P1	43663237	43664295	1	pseudogene	644157	DND1P1	NA	NA	0	0	1	0.000732115	NA	GTEx/v8/Stomach	NA	No	NA	NA	17:43962562:A/G;17:43991509:A/C	73
ENSG00000263503	RP11-707O23.5	43678235	43679706	-1	pseudogene	NA	NA	NA	NA	0	0	1	1.1663e-05	1.36659e-55	GTEx/v8/Stomach	NA	No	NA	NA	17:43962562:A/G;17:43991509:A/C	73

ENSG00000204650	CRHR1-IT1	43697694	43725582	1	pseudogene	147081	CRHR1-IT1	NA	NA	0	0	1	0.00144268	NA	GTEx/v8/Stomach	NA	No	NA	NA	17:43962562:A/G;17:43991509:A/C	73
ENSG00000120071	KANSL1	44107282	44302733	-1	protein_coding	284058	KANSL1	0.999733505943003	0.058945245	0	0	1	0.039914	NA	GTEx/v8/Stomach	NA	No	NA	NA	17:43962562:A/G;17:43991509:A/C	73
ENSG00000214401	KANSL1-AS1	44270942	44274089	1	antisense	644246	KANSL1-AS1	NA	NA	0	0	1	0.00301012	NA	GTEx/v8/Stomach	NA	No	NA	NA	17:43962562:A/G;17:43991509:A/C	73
ENSG00000262539	RP11-259G18.3	44336917	44337972	-1	pseudogene	NA	NA	NA	NA	0	0	1	2.53929e-06	6.54799e-59	GTEx/v8/Stomach	NA	No	NA	NA	17:43962562:A/G;17:43991509:A/C	73
ENSG00000176681	LRRC37A	44370099	44415160	1	protein_coding	9884	LRRC37A	0.890487355591061	NA	0	0	1	0.0183545	NA	GTEx/v8/Stomach	NA	No	NA	NA	17:43962562:A/G;17:43991509:A/C	73
ENSG00000238083	LRRC37A2	44588877	44633016	1	protein_coding	474170	LRRC37A2	NA	NA	0	0	1	3.45179e-05	1.58182e-44	GTEx/v8/Stomach	NA	No	NA	NA	17:43962562:A/G;17:43991509:A/C	73
ENSG00000180259	PRNT	4711928	4721314	-1	protein_coding	149830	PRNT	0.105498859424942	NA	0	0	0	NA	NA	NA	NA	Yes	GSE86189_GA.po.all.FUMA	0.000150126	20:6693128:A/G	79
ENSG00000231071	RP5-1068H6.1	4716315	4717319	-1	pseudogene	NA	NA	NA	NA	0	0	0	NA	NA	NA	NA	Yes	GSE86189_GA.po.all.FUMA	0.000150126	20:6693128:A/G	79
ENSG00000101265	RASSF2	4760669	4804291	-1	protein_coding	9770	RASSF2	0.000156231740850229	2.63529488	0	0	0	NA	NA	NA	NA	Yes	GSE86189_GA.po.all.FUMA	0.000150126	20:6693128:A/G	79
ENSG00000089057	SLC23A2	4833002	4990939	-1	protein_coding	9962	SLC23A2	0.511221636863337	-0.26302321	0	0	0	NA	NA	NA	NA	Yes	GSE86189_GA.po.all.FUMA	0.000150126	20:6693128:A/G	79
ENSG00000234573	RP5-1116H23.1	4994166	4994370	-1	pseudogene	NA	NA	NA	NA	0	0	0	NA	NA	NA	NA	Yes	GSE86189_GA.po.all.FUMA	0.000150126	20:6693128:A/G	79
ENSG00000089063	TMEM230	5080486	5093749	-1	protein_coding	29058	TMEM230	0.0138196085002164	-0.318554125	0	0	0	NA	NA	NA	NA	Yes	GSE86189_GA.po.all.FUMA	0.000150126	20:6693128:A/G	79
ENSG00000252367	Y_RNA	5094639	5094729	-1	misc_RNA	NA	NA	NA	NA	0	0	0	NA	NA	NA	NA	Yes	GSE86189_GA.po.all.FUMA	0.000150126	20:6693128:A/G	79

ENSG00000132646	PCNA	5095599	5107272	-1	protein_coding	5111	PCNA	0.937503505123045	-0.064678049	0	0	0	NA	NA	NA	NA	Yes	GSE86189_GA.po.all.FUMA	0.000150126	20:6693128:A/G	79
ENSG00000212517	SNORA26	5102063	5102185	-1	snoRNA	677810	SNORA26	NA	NA	0	0	0	NA	NA	NA	NA	Yes	GSE86189_GA.po.all.FUMA	0.000150126	20:6693128:A/G	79
ENSG00000101290	CDS2	5107432	5178533	1	protein_coding	8760	CDS2	6.78080961217838e-05	-0.414004061	0	0	0	NA	NA	NA	NA	Yes	GSE86189_GA.po.all.FUMA	0.000150126	20:6693128:A/G	79
ENSG00000101292	PROKR2	5282317	5297378	-1	protein_coding	128674	PROKR2	0.000669053120174497	0.520637041	0	0	0	NA	NA	NA	NA	Yes	GSE86189_GA.po.all.FUMA	0.000150126	20:6693128:A/G	79
ENSG00000125772	GPCPD1	5525085	5591672	-1	protein_coding	56261	GPCPD1	0.125571598748616	0.728681343	0	0	0	NA	NA	NA	NA	Yes	GSE86189_GA.po.all.FUMA	0.000150126	20:6693128:A/G	79
ENSG00000171984	C20orf196	5731039	5844558	1	protein_coding	149840	C20orf196	0.333004444219793	0.088267506	0	0	0	NA	NA	NA	NA	Yes	GSE86189_GA.po.all.FUMA	0.000150126	20:6693128:A/G	79
ENSG00000089199	CHGB	5892076	5906007	1	protein_coding	1114	CHGB	3.81635895457789e-07	0.069625991	0	0	0	NA	NA	NA	NA	Yes	GSE86189_GA.po.all.FUMA	0.000150126	20:6693128:A/G	79
ENSG00000089195	TRMT6	5917881	5931182	-1	protein_coding	51605	TRMT6	0.000492531588783058	0.864753047	0	0	0	NA	NA	NA	NA	Yes	GSE86189_GA.po.all.FUMA	0.000150126	20:6693128:A/G	79
ENSG00000125885	MCM8	5931298	5975852	1	protein_coding	84515	MCM8	1.92893351081766e-10	1.170137958	0	0	0	NA	NA	NA	NA	Yes	GSE86189_GA.po.all.FUMA	0.000150126	20:6693128:A/G	79
ENSG00000263755	RN7SL498P	5982379	5982674	1	misc_RNA	106479405	RN7SL498P	NA	NA	0	0	0	NA	NA	NA	NA	Yes	GSE86189_GA.po.all.FUMA	0.000150126	20:6693128:A/G	79
ENSG00000088766	CRLS1	5986736	6020699	1	protein_coding	54675	CRLS1	0.0335080599829462	-0.575320221	0	0	0	NA	NA	NA	NA	Yes	GSE86189_GA.po.all.FUMA	0.000150126	20:6693128:A/G	79
ENSG00000125872	LRRN4	6021424	6034695	-1	protein_coding	164312	LRRN4	1.45171418519099e-08	-0.077009289	0	0	0	NA	NA	NA	NA	Yes	GSE86189_GA.po.all.FUMA	0.000150126	20:6693128:A/G	79
ENSG00000101311	FERMT1	6055492	6104191	-1	protein_coding	55612	FERMT1	7.56849863577804e-07	0.610467254	0	0	0	NA	NA	NA	NA	Yes	GSE86189_GA.po.all.FUMA	0.000150126	20:6693128:A/G	79
ENSG00000228482	RP5-859D4.3	6711905	6716783	-1	lincRNA	NA	NA	NA	NA	1	14.802	NA	NA	NA	NA	NA	No	NA	0.000150126	20:6693128:A/G	79
ENSG00000125845	BMP2	6748311	6760927	1	protein_coding	650	BMP2	0.938086415869115	NA	0	0	0	NA	NA	NA	NA	Yes	GSE86189_GA.po.all.FUMA	0.000150126	20:6693128:A/G	79
ENSG00000101323	HAO1	7863628	7921121	-1	protein_coding	54363	HAO1	0.000672388585939552	-0.306947197	0	0	0	NA	NA	NA	NA	Yes	GSE86189_GA.po.all.FUMA	0.000150126	20:6693128:A/G	79
ENSG00000125827	TMX4	7957995	8000476	-1	protein_coding	56255	TMX4	0.0106621809140241	-0.092549499	0	0	0	NA	NA	NA	NA	Yes	GSE86189_GA.po.all.FUMA	0.000150126	20:6693128:A/G	79
ENSG00000229766	RP5-971N18.3	7999870	8024159	1	antisense	NA	NA	NA	NA	0	0	0	NA	NA	NA	NA	Yes	GSE86189_GA.po.all.FUMA	0.000150126	20:6693128:A/G	79
ENSG00000182621	PLCB1	8112824	8949003	1	protein_coding	23236	PLCB1	0.979044816094075	-0.375923643	0	0	0	NA	NA	NA	NA	Yes	GSE86189_GA.po.all.FUMA	0.000150126	20:6693128:A/G	79
ENSG00000188263	IL17REL	50432942	50451088	-1	protein_coding	400935	IL17REL	0.327459012314709	-1.305449092	0	0	1	0.0266024	NA	GTEx/v8/Stomach	+	No	NA	8.13804e-05	22:51004421:A/G	89

ENSG00000128159	TUBGCP6	50656118	50683421	-1	protein_coding	85378	TUBGCP6	5.08953781746856e-19	-0.506099702	0	0	1	0.0462161	NA	GTEx/v8/Stomach	-	No	NA	8.13804e-05	22:51004421:A/G	89
ENSG00000100429	HDAC10	50683612	50689834	-1	protein_coding	83933	HDAC10	3.2788087078515e-12	NA	0	0	1	0.0102376	NA	GTEx/v8/Stomach	+	No	NA	8.13804e-05	22:51004421:A/G	89
ENSG00000130489	SCO2	50961997	50964868	-1	protein_coding	9997	SCO2	0.000101907145142781	NA	0	0	1	0.027405	NA	GTEx/v8/Stomach	-	No	NA	8.13804e-05	22:51004421:A/G	89
ENSG00000025708	TYMP	50964181	50968485	-1	protein_coding	1890	TYMP	0.000465858647806756	NA	0	0	1	0.0387256	NA	GTEx/v8/Stomach	+	No	NA	8.13804e-05	22:51004421:A/G	89
ENSG00000177989	ODF3B	50968139	50971009	-1	protein_coding	440836	ODF3B	0.00916745822195973	NA	0	0	1	0.0410706	NA	GTEx/v8/Stomach	+	No	NA	8.13804e-05	22:51004421:A/G	89
ENSG00000205560	CPT1B	51007290	51017899	-1	protein_coding	1375	CPT1B	2.03406716145902e-09	0.43084121	0	0	1	0.00054526	NA	GTEx/v8/Stomach	+	No	NA	8.13804e-05	22:51004421:A/G	89
ENSG00000100288	CHKB	51017378	51039884	-1	protein_coding	1120	CHKB	0.000644971493148547	0.337272198	0	0	1	8.15605e-07	6.87297e-15	GTEx/v8/Stomach	+	No	NA	8.13804e-05	22:51004421:A/G	89
ENSG00000184319	RPL23AP82	51195376	51239737	1	pseudogene	284942	RPL23AP82	NA	NA	0	0	1	0.0168171	NA	GTEx/v8/Stomach	+	No	NA	8.13804e-05	22:51004421:A/G	89

Supplementary table S8. MAGMA gene-set analysis (The table displays top 10 gene sets where significant associations are not found after Bonferroni correction $P_{bon} < 0.05$.)

Gene Set	N genes	Beta	Beta STD	SE	*P*	P_{bon}
Curated_gene_sets:bosco_epithelial_differentiation_module	61	0.40672	0.0228	0.11083	0.00012184	1
Curated_gene_sets:reactome_post_translational_protein_modification	1373	0.085183	0.021871	0.024009	0.00019467	1
GO_bp:go_transcription_initiation_from_rna_polymerase_i_promoter	35	0.54009	0.022949	0.15732	0.00029912	1
GO_bp:go_regulation_of_platelet_activation	30	0.5224	0.020554	0.15577	0.00039984	1
GO_bp:go_nucleotide_excision_repair_preincision_complex_assembly	27	0.49838	0.018604	0.15134	0.00049651	1
GO_bp:go_transcription_elongation_from_rna_polymerase_i_promoter	30	0.54986	0.021634	0.16704	0.00049876	1
Curated_gene_sets:eppert_lsc_r	36	0.45018	0.0194	0.13816	0.00056142	1
GO_bp:go_positive_regulation_of_phenotypic_switching	2	2.0904	0.021252	0.64449	0.00059154	1
Curated_gene_sets:lui_thyroid_cancer_cluster_5	15	0.62522	0.017401	0.19282	0.00059357	1
Curated_gene_sets:reactome_post_translational_modification:_synthesis_of_gpi_anchored_proteins	92	0.30288	0.020835	0.095375	0.00074896	1

Supplementary table S9. Gene set enrichment pathway analysis (only significant gene sets included)

Category	GeneSet	N_genes	N_overlap	*p*	adj*P*	genes
GO_bp	GO_ION_TRANSPORT	1663	137	5.181862427345351e-18	3.808668884098833e-14	RNF207:MTOR:RHCE:MFSD2A:LRRC8B:LRRC8C:ABCD3:KCNC4:SLC16A4:GJA5:CTSS:HCN3:SYT11:PSEN2:TACR2:CDH23:PSAP:SLC16A12:HPX:KCNJ11:ABCC8:LINC00610:COMMD9:SYT13:OR5T1:SLC43A1:UCP2:UCP3:TRPC6:UBASH3B:NDUFA9:KCNA1:PTPN6:PHB2:SLC11A2:SLC25A3:ORAI1:P2RX2:MTMR6:SLC25A15:ERO1L:PSEN1:KCNK10:NIPA2:NIPA1:TRPM1:MYO5A:SLC51B:CHRNB4:KIAA1199:PKD1:GRIN2A:CORO1A:ABCC11:SLC7A6:SPG7:TRPV1:RCVRN:PIRT:SLC5A10:SLC47A1:CACNB1:STAC2:GJC1:RPS6KB1:CACNG1:PITPNC1:KCNJ16:KCNJ2:TTYH2:RALBP1:GIPC1:CEACAM1:GRIN2D:KCNJ14:OSR1:NCOA1:ATP6V1B1:SLC4A5:SLC9A4:SCN3A:SLC11A1:PER2:KCNS1:SEMG1:LIME1:SLC37A1:APOL1:MCHR1:SMDT1:ATP5L2:PPARA:PKDREJ:SCO2:CPT1B:MAPK8IP2:SHANK3:SCN5A:GNAI2:CACNA2D2:SLC25A26:ATP2C1:PLSCR4:KCNAB1:FGF12:MFI2:SLC34A2:STIM2:ATP10D:SLC10A4:SLC4A4:MTTP:PLA2G12A:NPY5R:SLC6A19:SLC6A18:SLC12A2:DIAPH1:NDFIP1:PRELID2:PDGFRB:KCNIP1:SLC22A23:EDN1:CNR1:CLDN4:CD36:ABCB1:KEL:LYN:CA2:GEM:KCNV2:UBQLN1:SLC35D2:PTPN3:SLC25A25:SLC34A3
GO_bp	GO_REGULATION_OF_TRANSPORT	1827	139	2.00391851536423e-15	7.364400676263545e-12	RNF207:TARDBP:MTOR:LDLRAP1:TSPAN1:KCNC4:GJA5:GOLPH3L:CTSS:HCN3:SYT11:UHMK1:SEC16B:ABL2:PSEN2:TACR2:GPAM:APBB1:KCNJ11:ABCC8:SYT13:OR5T1:ARL2:UCP2:TRPC6:UBASH3B:CRTAM:HSPA8:KCNA1:PTPN6:NCKAP1L:RAP1B:ORAI1:P2RX2:POSTN:SIAH3:LCP1:IPO5:STXBP6:SCFD1:PSEN1:NUMB:KCNK10:MAPKBP1:MYO5A:ARPP19:SLC51B:CHRNB4:KIAA1199:SH3GL3:GRIN2A:CORO1A:SEPT1:SEPT1:SMPD3:TRPV1:DERL2:RCVRN:PIRT:CACNB1:STAC2:NPEPPS:RPS6KB1:CACNG1:KCNJ16:KCNJ2:AZI1:MBP:SH3GL1:INSR:GIPC1:NPHS1:GSK3A:CEACAM1:CARD8:KCNJ14:OSR1:XPO1:IL1RL1:MERTK:SCN3A:CREB1:SLC11A1:MFF:PER2:KCNS1:SEMG1:LIME1:DNAJC5:MCHR1:PPARA:GTSE1:MAPK8IP2:SHANK3:SCN5A:MYRIP:GNAI2:HYAL3:HYAL2:CACNA2D2:FOXP1:CD200R1:GSK3B:KCNAB1:PYDC2:FGF12:STIM2:NPY5R:TERT:PTGER4:ISL1:MCTP1:C5orf20:TIFAB:DIAPH1:HDAC3:NDFIP1:PDGFRB:KCNIP1:EDN1:FLOT1:C4A:CNR1:TNFAIP3:YWHAG:CD36:ABCB1:KEL:BLK:PPP3CC:LYN:CA2:GEM:ENY2:SCRIB:KCNV2:UBQLN1:PTPN3:LRSAM1:PAEP
GO_bp	GO_TRANSMEMBRANE_TRANSPORT	1574	124	6.213615540971612 6e-15	1.522335807538045e-11	RNF207:MTOR:RHCE:MFSD2A:LRRC8B:LRRC8C:ABCD3:KCNC4:SLC16A4:GJA5:CTSS:HCN3:PSEN2:SLC16A12:KCNJ11:ABCC8:PEX16:SLC43A1:UCP2:UCP3:TRPC6:UBASH3B:HSPA8:KCNA1:PTPN6:PHB2:SLC11A2:SLC25A3:ORAI1:P2RX2:MTMR6:SLC25A15:PSME1:ERO1L:PSEN1:KCNK10:NIPA2:NIPA1:TRPM1:MYO5A:ARPP19:SLC51B:CHRNB4:KIAA1199:PKD1:GRIN2A:CORO1A:ABCC11:LONP2:SLC7A6:SPG7:TRPV1:PIRT:SLC5A10:SLC47A1:SLC47A2:CACNB1:STAC2:GJC1:RPS6KB1:CACNG1:PSMD12:KCNJ16:KCNJ2:TTYH2:RALBP1:SLC14A1:INSR:GSK3A:GRIN2D:KCNJ14:SLC6A16:OSR1:PEX13:ATP6V1B1:SLC4A5:SLC9A4:SCN3A:SLC11A1:PER2:KCNS1:LIME1:SLC37A1:APOL1:SMDT1:ATP5L2:PKDREJ:CPT1B:MAPK8IP2:SHANK3:SCN5A:CACNA2D2:SLC25A26:ATP2C1:KCNAB1:FGF12:SLC34A2:STIM2:ATP10D:SLC10A4:SLC4A4:SLC6A19:SLC6A18:TERT:SLC12A2:DIAPH1:KCNIP1:SLC22A23:EDN1:PEX7:CLDN4:ABCB1:KEL:PIP:LYN:CA2:CDH17:GEM:KCNV2:UBQLN1:SLC35D2:PTPN3:SLC25A25:SLC34A3
GO_bp	GO_CATION_TRANSPORT	1145	98	3.8011283391945607e-14	6.984573323270005e-11	RNF207:RHCE:KCNC4:GJA5:CTSS:HCN3:SYT11:PSEN2:TACR2:CDH23:HPX:KCNJ11:ABCC8:LINC00610:COMMD9:SYT13:OR5T1:UCP2:UCP3:TRPC6:UBASH3B:NDUFA9:KCNA1:PTPN6:PHB2:SLC11A2:SLC25A3:ORAI1:P2RX2:MTMR6:SLC25A15:ERO1L:PSEN1:KCNK10:NIPA2:NIPA1:TRPM1:MYO5A:KIAA1199:PKD1:GRIN2A:CORO1A:SPG7:TRPV1:RCVRN:PIRT:SLC5A10:SLC47A1:CACNB1:STAC2:CACNG1:KCNJ16:KCNJ2:RALBP1:GRIN2D:KCNJ14:OSR1:ATP6V1B1:SLC4A5:SLC9A4:SCN3A:SLC11A1:KCNS1:SEMG1:LIME1:MCHR1:SMDT1:ATP5L2:PKDREJ:SCO2:MAPK8IP2:SHANK3:SCN5A:GNAI2:CACNA2D2:ATP2C1:KCNAB1:FGF12:MFI2:SLC34A2:STIM2:ATP10D:SLC10A4:SLC4A4:SLC12A2:DIAPH1:NDFIP1:PDGFRB:KCNIP1:EDN1:CNR1:KEL:LYN:GEM:KCNV2:UBQLN1:PTPN3:SLC25A25:SLC34A3
GO_bp	GO_ION_TRANSMEMBRANE_TRANSPORT	1125	96	8.392803034826734e-14	1.23374204611953e-10	RNF207:MTOR:RHCE:LRRC8B:LRRC8C:ABCD3:KCNC4:GJA5:CTSS:HCN3:PSEN2:SLC16A12:KCNJ11:ABCC8:SLC43A1:UCP2:UCP3:TRPC6:UBASH3B:KCNA1:PTPN6:PHB2:SLC11A2:SLC25A3:ORAI1:P2RX2:MTMR6:SLC25A15:ERO1L:PSEN1:KCNK10:NIPA2:NIPA1:TRPM1:MYO5A:CHRNB4:KIAA1199:PKD1:GRIN2A:CORO1A:SLC7A6:SPG7:TRPV1:PIRT:SLC5A10:CACNB1:STAC2:GJC1:CACNG1:KCNJ16:KCNJ2:TTYH2:GRIN2D:KCNJ14:OSR1:ATP6V1B1:SLC4A5:SLC9A4:SCN3A:SLC11A1:PER2:KCNS1:LIME1:SLC37A1:APOL1:SMDT1:ATP5L2:PKDREJ:CPT1B:MAPK8IP2:SHANK3:SCN5A:CACNA2D2:ATP2C1:KCNAB1:FGF12:SLC34A2:STIM2:ATP10D:SLC4A4:SLC6A19:SLC6A18:SLC12A2:DIAPH1:KCNIP1:CLDN4:ABCB1:KEL:LYN:GEM:KCNV2:UBQLN1:SLC35D2:PTPN3:SLC25A25:SLC34A3

GO_bp	GO_HOMEOSTATIC_PROCESS	1906	138	1.1562157702114365e-13	1.4163643185090095e-10	EXOSC10:LDLRAP1:MPL:TAL1:TXNDC12:ACOT11:RPE65:BCL10:LRRC8B:LRRC8C:GJA5:CTSK:ARNT:HRNR:FLG:BGLAP:SMG5:TMEM79:ABL2:SOAT1:NUCKS1:GDF2:CDH23:GPAM:HPX:KCNJ11:ABCC8:LINC00610:COMMD9:GYLTL1B:UCP2:DGAT2:TRPC6:YAP1:UBASH3B:LINC01059:GRAMD1B:KCNA1:PTPN6:SLC11A2:NCKAP1L:PRKAB1:P2RX2:POLE:TNFSF13B:PCK2:TXNDC16:ERO1L:PSEN1:TRPM1:MYO5A:KIAA1199:PKD1:GRIN2A:RRN3:SMG1:CORO1A:SLX1A:CALB2:ANKRD11:TRPV1:ASGR2:PLEKHM1:KCNJ2:ZNF236:INSR:PKN1:PTGER1:DEDD2:GRIN2D:FPR2:ATP6V1B1:SLC4A5:DCTN1:IL18R1:SLC9A4:MERTK:POTEE:IGFBP5:SLC11A1:PER2:PCNA:CHMP4B:TGM2:MC3R:LIME1:MCHR1:SMDT1:SCO2:SHANK3:SCN5A:ACKR2:GNAI2:HYAL2:CCDC66:ATP2C1:NPHP3:ACKR4:RBP1:XRN1:FGF12:MFI2:SLC34A2:STIM2:SLC4A4:CXCL6:PLAC8:MTTP:TERT:PTGER4:SLC12A2:DIAPH1:HDAC3:NDFIP1:EDN1:HCRTR2:CNR1:L3MBTL3:TNFAIP3:ESR1:SOD2:CLDN4:RFC2:HSPB1:CD36:PRKAR2B:KEL:PIP:NCAPG2:STAR:LYN:TERF1:CA2:CALB1:ENY2:CYP11B1:CYP11B2:S1PR3:PTPN3:SLC34A3
GO_bp	GO_INTRACELLULAR_TRANSPORT	1815	133	1.4080407881590142e-13	1.4784428275669648e-10	RPL22:ICMT:CLSTN1:TARDBP:CDC42:TMEM50A:LDLRAP1:HOOK1:SSX2IP:ABCD3:SYT11:ARHGEF2:SSR2:LMNA:BGLAP:SMG5:UHMK1:F5:SEC16B:SNRPE:ARL5B:RAB18:IPO7:PEX16:SNX15:VPS51:SYVN1:HSPA8:DDX25:ERC1:PHB2:RAP1B:DAO:SIAH3:LCP1:ARL11:KPNA3:IPO5:SCFD1:HEATR5A:MIA2:CTAGE5:PSEN1:NUMB:CPSF2:HERC2:MYO5A:SLC51B:ANP32A:KIAA1199:PKD1:SMG1:CORO1A:TBC1D10B:LONP2:TRAPPC2L:SPG7:DERL2:RPL19:NPEPPS:KPNB1:SPAG9:RPL38:AZI1:NPLOC4:NAPG:MPPE1:UBXN6:DENND1C:AP1M2:DDX39A:GIPC1:GSK3A:KDELR1:PEX13:XPO1:DCTN1:TMEM87B:MFF:TMEM230:CHMP4B:EIF6:DNAJC5:SYNGR1:FAM109B:ARFGAP3:GTSE1:CPT1B:RPL32:MYRIP:RPL14:TRAK1:HYAL2:TMEM115:PROS1:STX19:IFT57:ADPRH:GSK3B:RAB43:RAB6B:COPB2:AP2M1:LSG1:AREG:AREGB:EIF4E:RPL37:IPO11:HDAC3:TUBB:VPS52:RPS18:RAB44:CNR1:PEX7:ARL4A:LAT2:HSPB1:YWHAG:CD36:BET1:BCAP29:COPG2:EXOC4:CREB3L2:DENND2A:STAR:LYN:RPL7:ENY2:SCRIB:NRBP2:CHMP5:RPL12
GO_bp	GO_SMALL_MOLECULE_METABOLIC_PROCESS	1685	125	3.4959981784918783e-13	3.2119483264894134e-10	ACOT7:MTOR:UBIAD1:LDLRAP1:RIMKLA:ELOVL1:ACOT11:RPE65:DDAH1:ABCD3:EXTL2:AMPD1:PHGDH:HMGCS2:CLK2:FDPS:SOAT1:ADCK3:CREM:ALDH18A1:GPAM:SMPD1:KCNJ11:ASRGL1:TM7SF2:PC:UCP3:MOGAT2:DGAT2:AASDHPPT:FDX1:REXO2:HSPA8:NDUFA9:TPI1:ENO2:PTHLH:DAO:SDS:SDSL:PRKAB1:RNASEH2B:PCK2:PSME1:ERO1L:PLA2G4B:MYO5A:ARPP19:TARSL2:PDXDC1:SULT1A3:DCTPP1:LONP2:COQ9:PLA2G15:SLC7A6:PRPSAP2:BRCA1:KPNB1:PSMD12:IMPA2:INSR:CD320:PDE4A:TECR:PRODH2:SARS2:GSK3A:LIPE:CEACAM1:PLB1:PPP1CB:PEX13:ATP6V1B1:MTHFD2:RPE:IGFBP5:PER2:PANK2:EIF6:UCKL1:LSS:APOL2:APOL1:APOBEC3B:CYP2D6:CYB5R3:PPARA:MIOX:TYMP:CPT1B:ENTPD3:CYP8B1:DHFRL1:GSK3B:CNBP:ACAD11:RBP2:RBP1:GK5:MAN2B2:COQ2:NPY1R:DCTD:MGAT1:EDN1:LRRC16A:GSTA1:CNR1:FUT9:PEX7:AIG1:ASL:TPST1:CD36:PRKAR2B:FAM3C:STAR:RDH10:CYP11B1:CYP11B2:IPPK:DPM2:SLC25A25:CEL
GO_bp	GO_METAL_ION_TRANSPORT	880	79	1.1391561843261264e-12	9.303108838663365e-10	RNF207:KCNC4:GJA5:HCN3:PSEN2:CDH23:HPX:KCNJ11:ABCC8:LINC00610:COMMD9:TRPC6:UBASH3B:NDUFA9:KCNA1:PTPN6:PHB2:SLC11A2:ORAI1:P2RX2:MTMR6:ERO1L:PSEN1:KCNK10:NIPA2:NIPA1:TRPM1:MYO5A:KIAA1199:PKD1:GRIN2A:CORO1A:SPG7:TRPV1:RCVRN:SLC5A10:CACNB1:STAC2:CACNG1:KCNJ16:KCNJ2:GRIN2D:KCNJ14:OSR1:ATP6V1B1:SLC4A5:SLC9A4:SCN3A:SLC11A1:KCNS1:SEMG1:LIME1:MCHR1:SMDT1:PKDREJ:SCO2:SCN5A:GNAI2:CACNA2D2:ATP2C1:KCNAB1:FGF12:MFI2:SLC34A2:STIM2:SLC10A4:SLC4A4:SLC12A2:DIAPH1:NDFIP1:PDGFRB:KCNIP1:KEL:LYN:GEM:KCNV2:UBQLN1:PTPN3:SLC25A25:SLC34A3
GO_bp	GO_CELL_CYCLE	1834	131	1.3446290311093847e-12	9.883023378653978e-10	TARDBP:MTOR:ZBTB17:CDC42:CDC20:TAL1:STIL:FOXE3:SSX2IP:LAMTOR5:PHGDH:HORMAD1:CKS1B:MSTO1:YY1AP1:ARHGEF2:LMNA:PMF1:UHMK1:ASPM:TUBB8:ZMYND11:MCM10:CUL2:REEP3:GRK5:APBB1:IPO7:WEE1:ARL2:SAC3D1:CDCA5:BTG4:RBM7:PTPN6:PHB2:APAF1:PRKAB1:CIT:POLE:ANKLE2:RNASEH2B:IPO5:PSME1:FOXG1:FANCM:DLGAP5:TDRD9:GOLGA8R:MGA:ARPP19:PKD1:SEPT1:SEPT1:SIAH1:SMPD3:MAPK7:RNF112:BRCA1:KPNB1:TUBD1:RPS6KB1:PSMD12:AZI1:CHAF1A:TUBB4A:INSR:GIPC1:ERF:PPP1CB:SPDYA:XPO1:DCTN1:RTKN:ANAPC1:PKP4:CTDSP1:PER2:PCNA:CHMP4B:WBP2NL:GTSE1:NCAPH2:SYCE3:GNAI2:HYAL1:TUSC2:RASSF1:NEK11:GMNC:IL8:EIF4E:TERT:NIPBL:MCIDAS:CCNO:KIF2A:CKS1B:CETN3:HDAC3:PDGFRB:LSM11:EDN1:MDC1:TUBB:CDKN1A:C6orf89:PTP4A1:MEI4:TNFAIP3:HECA:FGFR1OP:SEPT7:MPLKIP:RFC2:YWHAG:ABCB1:MEPCE:PRKAR2B:CEP41:NCAPG2:RB1CC1:TERF1:GEM:GML:SCRIB:CNTLN:CHMP5:KLF4:PTPN3:NTMT1:NACC2:TUBB4B
GO_bp	GO_EPITHELIUM_DEVELOPMENT	1273	100	3.6306150370380326e-12	2.4259109565663217e-09	RNF207:HES2:MTOR:CDC42:STIL:FOXE3:BCL10:RBM15:WDR77:PHGDH:GJA5:CTSK:HRNR:FLG:LCE1F:LCE1E:LCE1D:LCE1C:LCE1B:TMEM79:GDF2:PSAP:ACTA2:DMBT1:KRTAP5-1:KRTAP5-2:KRTAP5-3:KRTAP5-4:KRTAP5-5:EHF:YAP1:PHB2:HOXC13:RAP1B:LGR5:APAF1:ORAI1:TNFRSF19:FNDC3A:PCK2:PSME1:FRMD6:PSEN1:MYO5A:PKD1:TGFB1I1:ESRP2:B9D1:HNF1B:PSMD12:SAFB2:KEAP1:NPHS1:GSK3A:MEGF8:CNFN:CEACAM1:OSR1:SLC4A5:SLC9A4:PKP4:CREB1:IG

						FBP5:VIL1:HES6:PCNA:BFSP1:TGM2:PI3:KRTAP21-3:KRTAP8-1:CELSR1:PLXNB2:TGFBR2:FOXP1:ARL13B:IFT57:GSK3B:NPHP3:DVL3:AP2M1:PSAPL1:AREG:AREGB:MCIDAS:CCNO:CDX1:EDN1:CDKN1A:GSTA2:GSTA1:ESR1:DACT2:HOXA11:CLDN3:RDH10:CA2:CALB1:SCRIB:S1PR3:KLF4
GO_bp	GO_LIPID_METABOLIC_PROCESS	1241	98	4.4696013975791205e-12	2.567685238710121e-09	MTOR:LDLRAP1:A3GALT2:ELOVL1:ACOT11:RPE65:ABCD3:VAV3:HMGCS2:CERS2:FDPS:SOAT1:CREM:RBP3:PSAP:GPAM:SMPD1:TM7SF2:PC:UCP3:MOGAT2:DGAT2:FDX1:LPCAT3:MTMR6:EBPL:PCK2:PLA2G4B:PDXDC1:SMG1:SULT1A3:LONP2:PLA2G15:SMPD3:TRPV1:BRCA1:KPNB1:DGKE:MPPE1:IMPA2:ST8SIA5:PAFAH1B3:LIPE:CEACAM1:FPR2:NCOA1:PLB1:PEX13:ATP6V1B1:CREB1:PER2:PANK2:EIF6:LSS:APOL2:APOL1:NAGA:CYP2D6:CYB5R3:PPARA:CPT1B:ARSA:CYP8B1:NR1I2:CNBP:ACAD11:RBP2:RBP1:GK5:PSAPL1:MTTP:PLA2G12A:PPAP2A:HDAC3:PDGFRB:B3GALT4:GSTA1:CNR1:PEX7:AIG1:ESR1:ITGB8:JAZF1:CD36:PRKAR2B:ESYT2:STAR:LYN:NSMAF:RDH10:CYP11B1:CYP11B2:KLF4:RP11-203J24.9:ST6GALNAC6:ST6GALNAC4:PIP5KL1:DPM2:CEL
GO_bp	GO_CELLULAR_MACROMOLECULE_LOCALIZATION	1886	132	4.541484095677766e-12	2.567685238710121e-09	RPL22:ICMT:CLSTN1:TARDBP:EXOSC10:LDLRAP1:MACF1:SZT2:STIL:SSX2IP:LAMTOR5:GOLPH3L:SYT11:ARHGEF2:SSR2:LMNA:SMG5:UHMK1:SEC16B:ARL5B:RAB18:ZFYVE27:IPO7:KCNJ11:PEX16:ARL2:SNX15:CDCA5:SYVN1:HSPA8:DDX25:KCNA1:PHB2:DAO:SIAH3:LCP1:ARL11:KPNA3:IPO5:STXBP6:FRMD6:PSEN1:NUMB:CPSF2:MOAP1:HERC2:MYO5A:SLC51B:KIAA1199:MESDC2:PKD1:GRIN2A:SMG1:TBC1D10B:LONP2:RABEP1:DERL2:RPL19:STAC2:NPEPPS:KPNB1:RPL38:AZI1:NPLOC4:NAPG:AP1M2:DDX39A:GIPC1:GSK3A:KDELR1:PEX13:XPO1:RTKN:VIL1:MFF:CHMP4B:EIF6:SYNGR1:ARFGAP3:GTSE1:CELSR1:SHANK3:RPL32:MYRIP:RPL14:TRAK1:HYAL2:CCDC66:FAM208A:ARL13B:STX19:IFT57:ADPRH:GSK3B:RAB43:RAB6B:COPB2:DVL3:AP2M1:EIF4E:TERT:NIPBL:RPL37:IPO11:DIAPH1:HDAC3:TSPAN17:FLOT1:RPS18:RAB44:MAP7:PEX7:TNFAIP3:ESR1:SYNE1:ARL4A:HSPB1:YWHAG:CD36:BCAP29:COPG2:EXOC4:PPP3CC:TERF1:RPL7:ENY2:SCRIB:CNTLN:XPA:RPL12:LRSAM1:NACC2
GO_bp	GO_SECRETION	1628	118	7.57703652651981e-12	3.9779441764229e-09	TARDBP:TXLNA:KCNC4:NRAS:GJA5:GOLPH3L:CTSS:HRNR:SYT11:TMEM79:FCGR2A:HSPA6:F5:SELP:RAB18:TACR2:PSAP:GPAM:APBB1:KCNJ11:ABCC8:LINC00610:COMMD9:SYT13:OR5T1:ARL2:UCP2:CRTAM:HSPA8:PTPN6:METTL7A:NCKAP1L:RAP1B:APAF1:POSTN:TNFSF13B:STXBP6:SCFD1:MIA2:CTAGE5:PSEN1:MAPKBP1:MYO5A:SLC51B:CHRNB4:CORO1A:SEPT1:SEPT1:SMPD3:TRPV1:HNF1B:VAT1:KPNB1:PSMD12:MBP:CD97:GIPC1:NPHS1:SYCN:POU2F2:CEACAM1:CARD8:CD33:FPR2:NCOA1:SLC4A5:IL1RL1:SLC9A4:MERTK:CREB1:SLC11A1:PER2:SLPI:DNAJC5:SYNGR1:CYB5R3:ARFGAP3:ARSA:MYRIP:GNAI2:HYAL3:HYAL2:CACNA2D2:FOXP1:PROS1:STX19:CD200R1:GSK3B:ARMC8:PYDC2:CXCL1:PLAC8:MTTP:PLA2G12A:NPY5R:PTGER4:ISL1:MCTP1:C5orf20:TIFAB:DIAPH1:EDN1:TUBB:RAB44:CNR1:TNFAIP3:RNASET2:LAT2:CD36:FAM3C:COPG2:EXOC4:BLK:LYN:CA2:ENY2:SCRIB:CEL:PAEP:TUBB4B:TOR4A
GO_bp	GO_CATION_TRANSMEMBRANE_TRANSPORT	834	73	2.717114479179717e-11	1.3313860947980613e-08	RNF207:RHCE:KCNC4:CTSS:HCN3:PSEN2:KCNJ11:ABCC8:UCP2:UCP3:TRPC6:UBASH3B:KCNA1:PTPN6:PHB2:SLC11A2:SLC25A3:ORAI1:P2RX2:MTMR6:SLC25A15:ERO1L:PSEN1:KCNK10:NIPA2:NIPA1:TRPM1:MYO5A:KIAA1199:PKD1:GRIN2A:CORO1A:SPG7:TRPV1:PIRT:SLC5A10:CACNB1:STAC2:CACNG1:KCNJ16:KCNJ2:GRIN2D:KCNJ14:OSR1:ATP6V1B1:SLC9A4:SCN3A:SLC11A1:KCNS1:LIME1:SMDT1:ATP5L2:PKDREJ:MAPK8IP2:SHANK3:SCN5A:CACNA2D2:ATP2C1:KCNAB1:FGF12:SLC34A2:STIM2:SLC12A2:DIAPH1:KCNIP1:KEL:LYN:GEM:KCNV2:UBQLN1:PTPN3:SLC25A25:SLC34A3
GO_bp	GO_RESPONSE_TO_ABIOTIC_STIMULUS	1211	94	3.181781694464071e-11	1.4616309658944327e-08	MTOR:MPL:ACOT11:RPE65:DNAJB4:BCL10:DDAH1:LRRC8C:CTSS:ARNT:ADAM15:CLK2:PKLR:ARHGEF2:LMNA:BGLAP:HSPA6:NUCKS1:PSEN2:CUL2:TACR2:RGR:FAS:ABCC8:UCP2:UCP3:TRPC6:YAP1:HSPA8:KCNA1:PHB2:SLC11A2:PDE1B:IKBIP:APAF1:P2RX2:POSTN:ERCC5:PSME1:SCFD1:ERO1L:TRPM1:PKD1:GRIN2A:TGFB1I1:N4BP1:TRPV1:RCVRN:PIRT:MFAP4:BRCA1:NPEPPS:RPS6KB1:PSMD12:KCNJ2:PKN1:DNAJB1:PPP1CB:CREB1:IGFBP2:PER2:PCNA:MMP24:PPARA:PKDREJ:SHANK3:RAD18:TGFBR2:HYAL3:HYAL1:HYAL2:CCDC66:GSK3B:PLAC8:TERT:NIPBL:PTGER4:SLC12A2:C5orf20:TIFAB:PDGFRB:CPEB4:EDN1:CDKN1A:MAP7:GTF2H5:CLDN3:ABCB1:TSC22D4:STAR:LYN:CA2:UBQLN1:XPA
GO_bp	GO_BIOLOGICAL_ADHESION	1404	104	4.180170222777942e-11	1.7771244202867616e-08	CLSTN1:FBLIM1:CDC42:MACF1:MPL:SSX2IP:BCL10:VAV3:ANXA9:TNFAIP8L2:ADAM15:BGLAP:PEAR1:SELP:ABL2:CRB1:CSRP1:NID1:CDH23:C10orf54:GPAM:HABP2:ARL2:LRFN4:TENM4:UBASH3B:CDON:OPCML:PTPN6:NCKAP1L:NUAK1:POSTN:FNDC3A:PCDH9:LMO7:TNFSF13B:STXBP6:LRFN5:PSEN1:ACAN:HAPLN3:PKD1:CORO1A:TGFB1I1:MAPK7:MFAP4:TBX21:MYL12A:MBP:MUC16:CD97:IL12RB1:NPHS1:CEACAM1:BCAM:CD33:SIGLEC6:FPR2:PPP1CB:MERTK:RND3:PKP4:IGFBP2:SNED1:MMP24:TGM2:DGCR6:FBLN1:PPARA:CELSR1:PLXNB2:TGFBR2:HYAL1:CD200R1:GSK3B:GP9:ATP2C1:MFI2:IL8:CDH9:CDH6:MEGF10:PCDHGA12:PCDHGC3:PCDHGC4:PCDHGC5:RELL2:NDFIP1:ADAM19:LRRC16A:FLOT1:MUC21:DACT2:THBS2:ITGB8:CLDN3:CLDN4:HSPB1:CD36:LYN:CDH17:SCRIB:KLF4:CEL

GO_bp	GO_REGULATION_OF_RESPONSE_TO_STRESS	1481	108	4.352141437436967e-11	1.7771244202867616e-08	MTOR:SZT2:TXNDC12:BCL10:DDAH1:F3:NRAS:CTSS:CTSK:CERS2:TNFAIP8L2:DCST1:ASH1L:SYT11:ARHGEF2:LMNA:APCS:SELP:ZMYND11:PSAP:FAS:DMBT1:APBB1:HPX:ABCC8:SYVN1:UBASH3B:CRTAM:HSPA8:PTPN6:NUAK1:TNFRSF19:RNASEH2B:PSME1:COCH:LRFN5:MAP3K9:MAPKBP1:SMG1:SHPK:SHPK:DERL2:CLEC10A:MAPK7:BRCA1:NBR1:SPAG9:RNFT1:PSMD12:NPLOC4:PHLPP1:SERPINB4:MUC16:PKN1:DNAJB1:IL12RB1:CXCL17:CEACAM1:PUM2:IL1RL1:IL18RAP:VIL1:PCNA:TGM2:ZBP1:USP25:TAB1:PPARA:MAPK8IP2:TGFBR2:HYAL2:FOXP1:PROS1:CD200R1:GSK3B:DVL3:MAP3K13:PYDC2:CXCL6:NPY5R:TERT:DROSHA:PTGER4:ISL1:MCTP1:C5orf30:HDAC3:RELL2:NDFIP1:WRNIP1:EDN1:FLOT1:IER3:MUC21:CDKN1A:CNR1:TNFAIP3:ESR1:SOD2:HSPB1:CD36:ABCB1:RB1CC1:LYN:SCRIB:UBQLN1:KLF4:LRSAM1:NACC2
GO_bp	GO_INORGANIC_ION_TRANSMEMBRANE_TRANSPORT	821	71	8.748453185968217e-11	3.384270048256126e-08	RNF207:MTOR:KCNC4:HCN3:PSEN2:KCNJ11:ABCC8:UCP2:UCP3:TRPC6:UBASH3B:KCNA1:PTPN6:PHB2:SLC11A2:SLC25A3:ORAI1:MTMR6:ERO1L:PSEN1:KCNK10:NIPA2:NIPA1:TRPM1:MYO5A:KIAA1199:PKD1:GRIN2A:CORO1A:SPG7:TRPV1:SLC5A10:CACNB1:STAC2:CACNG1:KCNJ16:KCNJ2:TTYH2:GRIN2D:KCNJ14:OSR1:ATP6V1B1:SLC9A4:SCN3A:SLC11A1:KCNS1:LIME1:SLC37A1:APOL1:SMDT1:ATP5L2:PKDREJ:SCN5A:CACNA2D2:ATP2C1:KCNAB1:FGF12:SLC34A2:STIM2:SLC12A2:DIAPH1:KCNIP1:CLDN4:KEL:LYN:GEM:KCNV2:UBQLN1:PTPN3:SLC25A25:SLC34A3
GO_bp	GO_REGULATION_OF_ION_TRANSPORT	677	62	1.429824120253392e-10	5.2546036419312164e-08	RNF207:MTOR:KCNC4:GJA5:CTSS:HCN3:SYT11:PSEN2:TACR2:KCNJ11:ABCC8:SYT13:OR5T1:TRPC6:UBASH3B:KCNA1:PTPN6:ORAI1:P2RX2:PSEN1:KCNK10:MYO5A:KIAA1199:GRIN2A:CORO1A:RCVRN:PIRT:CACNB1:STAC2:CACNG1:KCNJ16:KCNJ2:KCNJ14:OSR1:SCN3A:PER2:KCNS1:SEMG1:LIME1:MCHR1:MAPK8IP2:SHANK3:SCN5A:GNAI2:CACNA2D2:KCNAB1:FGF12:STIM2:NPY5R:DIAPH1:PDGFRB:KCNIP1:EDN1:CNR1:ABCB1:KEL:LYN:CA2:GEM:KCNV2:UBQLN1:PTPN3
GO_bp	GO_PROTEIN_CONTAINING_COMPLEX_ASSEMBLY	1891	127	1.7449603483059745e-10	6.0281824903937e-08	MTOR:FBLIM1:CDC42:TAL1:BCL10:LRRC8C:PTBP2:KCNC4:WDR77:GJA5:PRUNE:TMOD4:SYT11:KIAA0907:ARHGEF2:APCS:F5:SELP:SEC16B:SOAT1:FMOD:SNRPE:FAS:ARL2:YAP1:HSPA8:NDUFA9:KCNA1:NCKAP1L:APAF1:PRKAB1:TMEM120B:ULK1:P2RX2:LCP1:KPNA3:KCTD12:ERCC5:STXBP6:SCFD1:FAM179B:PRPF39:TRPM1:CHRNB4:RPL3L:PKD1:RRN3:CORO1A:CD2BP2:DCTPP1:SLC7A6:PRMT7:SMPD3:TRAPPC2L:TRPV1:DHX33:HNF1B:NBR1:SPAG9:KCNJ2:RPL38:DNAI2:C17orf89:NAPG:CHAF1A:TUBB4A:INSR:NPHS1:CD79A:GRWD1:PUM2:TRMT61B:DCTN1:CREB1:VIL1:MFF:COPS8:NDUFAF5:CHMP4B:EIF6:TGM2:KCNS1:SEMG1:SEMG2:NDUFA6:SCO2:MAPK8IP2:CAND2:ZMYND10:SHQ1:GSK3B:NDUFB4:ISY1:PYDC2:SLAIN2:AREG:AREGB:MTTP:TERT:PTGER4:OCLN:ANKRA2:DIAPH1:FCHSD1:THG1L:LRRC16A:FLOT1:PFDN6:TAPBP:TNFAIP3:ESR1:GTF2H5:SOD2:SEPT7:CLDN3:CD36:BET1:PILRB:ASH2L:TERF1:CDH17:LRRC6:KHDRBS3:KCNV2:XPA:RPL12:GTF3C5:NACC2
GO_bp	GO_ANIMAL_ORGAN_MORPHOGENESIS	1030	82	1.8043539427028764e-10	6.0281824903937e-08	RNF207:MTOR:CDC42:STIL:FOXE3:RPE65:OLFM3:RBM15:HIPK1:GJA5:ADAM15:ASH1L:BGLAP:CRB1:ACTA2:YAP1:CDON:C12orf57:PHB2:HOXC13:HOXC11:NRL:PSME1:FOXG1:COCH:SIX6:PSEN1:TRPM1:MYO5A:ACAN:PKD1:ESRP2:SMPD3:ANKRD11:HNF1B:PSMD12:RPL38:FSCN2:ASXL3:INSR:GSK3A:MEGF8:CEACAM1:OSR1:ATP6V1B1:IGFBP5:TGM2:DGCR6:TAB1:PPARA:CELSR1:SHANK3:TGFBR2:SCN5A:HYAL1:HYAL2:CCDC66:LRIG1:ARL13B:IFT57:NPHP3:DVL3:AP2M1:AREG:AREGB:NPY1R:NPY5R:NIPBL:ISL1:C5orf20:TIFAB:PDGFRB:CDX1:EDN1:PEX7:TNFAIP3:ESR1:HOXA11:EXOC4:RDH10:CA2:CALB1:SCRIB
GO_bp	GO_REGULATION_OF_TRANSMEMBRANE_TRANSPORT	553	54	2.1986080383117846e-10	6.912702030221549e-08	RNF207:MTOR:KCNC4:GJA5:CTSS:HCN3:PSEN2:KCNJ11:ABCC8:TRPC6:UBASH3B:KCNA1:PTPN6:PSEN1:KCNK10:MYO5A:ARPP19:KIAA1199:GRIN2A:CORO1A:PIRT:CACNB1:STAC2:RPS6KB1:CACNG1:KCNJ16:KCNJ2:INSR:GSK3A:KCNJ14:OSR1:SCN3A:PER2:KCNS1:LIME1:MAPK8IP2:SHANK3:SCN5A:CACNA2D2:KCNAB1:FGF12:STIM2:TERT:DIAPH1:KCNIP1:EDN1:ABCB1:KEL:LYN:CA2:GEM:KCNV2:UBQLN1:PTPN3
GO_bp	GO_CELL_CYCLE_PROCESS	1370	100	2.2572088261947916e-10	6.912702030221549e-08	MTOR:ZBTB17:CDC42:CDC20:STIL:FOXE3:SSX2IP:LAMTOR5:PHGDH:HORMAD1:MSTO1:ARHGEF2:LMNA:UHMK1:ASPM:TUBB8:MCM10:CUL2:REEP3:APBB1:WEE1:ARL2:SAC3D1:CDCA5:BTG4:PTPN6:PHB2:APAF1:PRKAB1:CIT:POLE:ANKLE2:RNASEH2B:PSME1:FANCM:DLGAP5:TDRD9:GOLGA8R:MGA:ARPP19:PKD1:SEPT1:SEPT1:SMPD3:RNF112:BRCA1:KPNB1:RPS6KB1:PSMD12:AZI1:TUBB4A:INSR:GIPC1:PPP1CB:SPDYA:XPO1:DCTN1:RTKN:ANAPC1:PKP4:CTDSP1:PCNA:CHMP4B:GTSE1:NCAPH2:SYCE3:HYAL1:RASSF1:NEK11:IL8:EIF4E:TERT:NIPBL:CCNO:KIF2A:CETN3:HDAC3:PDGFRB:LSM11:EDN1:MDC1:TUBB:CDKN1A:MEI4:FGFR1OP:SEPT7:RFC2:YWHAG:ABCB1:MEPCE:PRKAR2B:CEP41:TERF1:GEM:GML:CNTLN:CHMP5:KLF4:NTMT1:NACC2:TUBB4B
GO_bp	GO_REGULATION_OF_CELL_DIFFERENTIATION	1844	124	2.699950598669985e-10	7.937854760089757e-08	HES2:MTOR:SPEN:NBL1:CDC42:TRIM62:MACF1:MPL:CDC20:TAL1:FOXE3:RBM15:CTSK:ARNT:CERS2:PRUNE:SEMA6C:ARHGEF2:SEMA4A:BGLAP:APCS:ABL2:ASPM:GDF2:C10orf54:ZFYVE27:APBB1:CTR9:ABCC8:TENM4:TRPC6:YAP1:UBASH3B:CDON:PTPN6:SOX5:PTHLH:NCKAP1L:FBXW8:POSTN:PSME1:FOXG1:PSEN1:NUMB:TCF12:SKOR1:ADAMTS7:SH3GL3:RRN3:TGFB1I1:CCL17:NFATC3:TRPV1:RNF112:HNF1B:IKZF3:RND2:NBR1:TBX21:SPAG9:PSMD12:RNF157:TNRC6C:SKOR2:TCF3:KEAP1:S1PR5:IL

						12RB1:GSK3A:MEGF8:CEACAM1:OSR1:NCOA1:ALK:BCL11A:PLEKHB2:CREB1:IGFBP5:CTDSP1:HES6:PER2:EIF6:C21orf91:FBLN1:PPARA:PLXNB2:SHANK3:TGFBR2:TRAK1:FOXP1:C3orf17:GSK3B:NPHP3:DVL3:MAP3K13:MFI2:AREG:AREGB:EIF4E:TERT:DROSHA:NIPBL:ISL1:MEGF10:NDFIP1:EDN1:LRRC16A:FLOT1:CPNE5:CNR1:SOD2:HOXA11:YWHAG:CD36:CREB3L2:KEL:NCAPG2:ASH2L:STAR:LYN:CA2:S1PR3:KLF4:PAEP:NELFB
GO_bp	GO_RESPONSE_TO_DRUG	1008	80	3.4995220727960354e-10	9.892879705788793e-08	MTOR:ABCD3:VAV3:SETDB1:ADAM15:HCN3:PKLR:SYT11:ARHGEF2:BGLAP:TACR2:PSAP:SLC16A12:SMPD1:KCNJ11:ABCC8:SYT13:OR5T1:TRPC6:YAP1:FDX1:NCKAP1L:PDE1B:RAP1B:P2RX2:SLC25A15:PCK2:PSEN1:CHRNB4:GRIN2A:SULT1A3:SMPD3:TRPV1:MAPK7:RNF112:SLC47A1:SLC47A2:HNF1B:BRCA1:RPS6KB1:RALBP1:CD320:PDE4A:OSR1:NCOA1:BCL11A:XPO1:IL18RAP:CREB1:IGFBP2:SLC11A1:PER2:PCNA:RPN2:CYP2D6:PPARA:TGFBR2:GNAI2:SLC25A26:NR1I2:XRN1:AREG:AREGB:EIF4E:CCNO:PDGFRB:EDN1:CDKN1A:CNR1:TNFAIP3:CLDN3:CLDN4:ABCB1:PRKAR2B:STAR:LYN:TERF1:CA2:CHMP5:KLF4:SLC25A25
GO_bp	GO_INTRACELLULAR_PROTEIN_TRANSPORT	1156	88	3.679847807224029e-10	1.0017363475220968e-07	RPL22:ICMT:CLSTN1:TARDBP:SSX2IP:ARHGEF2:SSR2:LMNA:SMG5:UHMK1:SEC16B:ARL5B:RAB18:IPO7:PEX16:SNX15:SYVN1:HSPA8:DDX25:PHB2:DAO:SIAH3:LCP1:ARL11:KPNA3:IPO5:PSEN1:NUMB:CPSF2:HERC2:SLC51B:KIAA1199:PKD1:SMG1:TBC1D10B:LONP2:DERL2:RPL19:NPEPPS:KPNB1:RPL38:AZI1:NPLOC4:NAPG:AP1M2:DDX39A:GIPC1:GSK3A:KDELR1:PEX13:XPO1:MFF:CHMP4B:EIF6:SYNGR1:ARFGAP3:GTSE1:RPL32:MYRIP:RPL14:TRAK1:HYAL2:STX19:IFT57:ADPRH:GSK3B:RAB43:RAB6B:COPB2:AP2M1:EIF4E:RPL37:IPO11:HDAC3:RPS18:RAB44:PEX7:ARL4A:HSPB1:YWHAG:CD36:BCAP29:COPG2:EXOC4:RPL7:ENY2:SCRIB:RPL12
GO_bp	GO_APOPTOTIC_PROCESS	1956	129	3.936971444513173e-10	1.032497390438524e-07	TARDBP:MTOR:STIL:FOXE3:TXNDC12:TM2D1:DNASE2B:BCL10:F3:VAV3:LAMTOR5:HIPK1:TNFAIP8L2:DAP3:ARHGEF2:LMNA:ARHGEF11:PHLDA3:PSEN2:ZMYND11:CUL2:GDF2:FAS:SFRP5:GPAM:GRK5:SMPD1:APBB1:SYVN1:UCP2:YAP1:ERC1:ATN1:PTPN6:PHB2:DDX47:CSRNP2:NCKAP1L:PDE1B:APAF1:CIT:ANKLE2:TNFRSF19:ERCC5:G2E3:ERO1L:MAP3K9:RBM25:PSEN1:MOAP1:GRIN2A:RRN3:CORO1A:SIAH1:CIAPIN1:TRPV1:RABEP1:MAPK7:HNF1B:IKZF3:BRCA1:KPNB1:RPS6KB1:RNF157:PHLPP1:ARHGEF18:PKN1:DEDD2:GSK3A:CARD8:KDELR1:OSR1:NCOA1:ALK:RTKN:MERTK:CREB1:VIL1:MFF:TGM2:DNAJC5:SYCE3:MAPK8IP2:TGFBR2:GNAI2:HYAL2:FOXP1:SHQ1:C3orf38:IFT57:GSK3B:PLAC8:NPY5R:TERT:CARD6:ISL1:MEGF10:HDAC3:PDGFRB:CPEB4:SNCB:EDN1:IER3:RGL2:CDKN1A:CNR1:TNFAIP3:ESR1:SOD2:SEPT7:HSPB1:YWHAG:BCAP29:PIP:PPP3CC:STAR:RB1CC1:LYN:NSMAF:GML:SCRIB:PUF60:NRBP2:UBQLN1:XPA:KLF4:NAIF1:PAEP:NACC2
GO_bp	GO_CHEMICAL_HOMEOSTASIS	1177	89	4.073799227580571e-10	1.032497390438524e-07	LDLRAP1:GJA5:HRNR:FLG:TMEM79:ABL2:SOAT1:NUCKS1:GDF2:CDH23:GPAM:HPX:KCNJ11:ABCC8:LINC00610:COMMD9:UCP2:DGAT2:TRPC6:UBASH3B:LINC01059:GRAMD1B:KCNA1:PTPN6:SLC11A2:P2RX2:PCK2:ERO1L:PSEN1:TRPM1:MYO5A:KIAA1199:PKD1:GRIN2A:CORO1A:CALB2:TRPV1:ASGR2:KCNJ2:ZNF236:INSR:PTGER1:GRIN2D:FPR2:ATP6V1B1:SLC4A5:SLC9A4:IGFBP5:SLC11A1:TGM2:MC3R:LIME1:MCHR1:SMDT1:SCO2:SCN5A:ACKR2:GNAI2:HYAL2:ATP2C1:ACKR4:RBP1:FGF12:MFI2:SLC34A2:STIM2:SLC4A4:MTTP:PTGER4:SLC12A2:DIAPH1:NDFIP1:EDN1:HCRTR2:CNR1:ESR1:SOD2:CLDN4:CD36:PRKAR2B:KEL:STAR:LYN:CA2:CALB1:ENY2:CYP11B1:CYP11B2:S1PR3:PTPN3:SLC34A3
GO_bp	GO_RESPONSE_TO_OXYGEN_CONTAINING_COMPOUND	1603	111	4.803378774129004e-10	1.1768277996616059e-07	MTOR:TIE1:SZT2:RPE65:BCL10:LAMTOR5:SETDB1:ADAM15:CLK2:HCN3:PKLR:ARHGEF2:BGLAP:SELP:ABL2:NUCKS1:PSAP:GPAM:SMPD1:CTR9:KCNJ11:ABCC8:OR5T1:UCP2:UCP3:DGAT2:TRPC6:YAP1:FDX1:LINC01059:GRAMD1B:NDUFA9:PHB2:RAP1B:P2RX2:POSTN:IPO5:PCK2:PSEN1:MYO5A:GRIN2A:SULT1A3:SMPD3:SHPK:TRPV1:SHPK:GLP2R:MAPK7:RNF112:HNF1B:CACNB1:BRCA1:TRIM25:RPS6KB1:SSTR2:ZNF236:INSR:PTGER1:NFKBIB:GSK3A:CEACAM1:CARD8:FPR2:OSR1:NCOA1:BCL11A:ATP6V1B1:IL18RAP:CREB1:IGFBP2:IGFBP5:SLC11A1:PCNA:EIF6:SLPI:PPARA:TGFBR2:GNAI2:HYAL1:HYAL2:FOXP1:GSK3B:XRN1:PLSCR4:IL8:CXCL6:PF4V1:CXCL1:AREG:AREGB:EIF4E:PTGER4:PDGFRB:CPEB4:EDN1:CDKN1A:CNR1:TNFAIP3:ESR1:SOD2:CRHR2:CLDN3:CLDN4:YWHAG:CD36:PRKAR2B:STAR:LYN:CA2:ENY2:CYP11B1:CYP11B2:CHMP5:KLF4
GO_bp	GO_CELLULAR_RESPONSE_TO_ENDOGENOUS_STIMULUS	1373	99	5.362278670666782e-10	1.2582127811285052e-07	MTOR:C1orf64:NBL1:RPE65:LAMTOR5:TRIM33:CTSS:HCN3:PKLR:ARHGEF2:BGLAP:FMOD:NUCKS1:GDF2:SFRP5:GPAM:SCGB2A1:UCP2:UCP3:YAP1:FDX1:PHB2:SOX5:RAP1B:LGR5:APAF1:POSTN:IPO5:PCK2:PSEN1:MYO5A:SKOR1:ADAMTS7:CORO1A:SULT1A3:TGFB1I1:POLR2C:ESRP2:SMPD3:TRPV1:GLP2R:FAM83G:MAPK7:CACNB1:BRCA1:RPS6KB1:SSTR2:SKOR2:SAFB2:SAFB:INSR:ARHGEF18:GIPC1:GSK3A:MEGF8:CEACAM1:FPR2:NCOA1:FSHR:BCL11A:ATP6V1B1:CREB1:IGFBP2:IGFBP5:VIL1:NCL:TAB1:PPARA:TGFBR2:GNAI2:HYAL1:HYAL2:FOXP1:NR1I2:GSK3B:UBA5:XRN1:FGF12:IL8:EIF4E:PTGER4:ISL1:PPAP2A:DIAPH1:PDGFRB:CPEB4:EDN1:ESR1:CRHR2:YWHAG:CD36:PRKAR2B:STAR:LYN:CA2:CYP11B1:CYP11B2:CHMP5:KLF4
GO_bp	GO_NERVOUS_SYSTEM_PROCESS	1412	101	5.477933196749955e-10	1.2582127811285052e-07	MTOR:RPE65:LCE1D:OR14K1:RBP3:TACR2:CDH23:PSAP:RGR:SFRP5:OR52B4:OR51T1:KCNJ11:ABCC8:OR4C15:OR4C16:OR4P4:OR4S2:OR4C6:OR5T1:OR8H1:OR8K3:OR8K1:OR8J1:OR8U1:OR5R1:TENM4:KCNA1:C12orf57:SLC11A2:PDE1B:OR10P1:P2R

						X2:NRL:COCH:SIX6:PSEN1:KCNK10:TRPM1:MYO5A:CHRNB4:KIAA1199:LINS:GRIN2A:NTAN1:TRPV1:RCVRN:GJC1:RPS6KB1:RPL38:FSCN2:MBP:SH3GL1:INSR:OR1M1:OR7G2:OR7G1:OR7G3:PDE4A:OR10H2:OR10H3:GSK3A:CIC:GRIN2D:ATP6V1B1:DCTN1:SCN3A:CREB1:MMP24:TYMP:MAPK8IP2:SHANK3:SCN5A:CCDC66:LRIG1:GSK3B:UBA5:KCNAB1:FGF12:NPY1R:NIPBL:C5orf20:TIFAB:DIAPH1:GRXCR2:EDN1:OR10C1:CNR1:SOD2:CD36:PRKAR2B:PIP:TAS2R39:TAS2R40:RDH10:CALB1:OR1L3:OR1L4:OR1L6:PBX3:OBP2A
GO_bp	GO_REGULATION_OF_CELL_DEVELOPMENT	930	75	6.217628239198197e-10	1.3848353805486892e-07	HES2:MTOR:SPEN:NBL1:CDC42:MACF1:MPL:CDC20:CERS2:PRUNE:SEMA6C:ARHGEF2:SEMA4A:ABL2:ASPM:ZFYVE27:APBB1:ABCC8:TENM4:TRPC6:YAP1:CDON:PTHLH:FBXW8:POSTN:FOXG1:PSEN1:NUMB:TCF12:SH3GL3:RRN3:TRPV1:RNF112:RND2:SPAG9:RNF157:TCF3:S1PR5:GSK3A:MEGF8:NCOA1:ALK:BCL11A:CREB1:CTDSP1:HES6:PER2:C21orf91:FBLN1:PPARA:PLXNB2:SHANK3:TRAK1:C3orf17:GSK3B:DVL3:MAP3K13:MFI2:EIF4E:TERT:NIPBL:ISL1:EDN1:LRRC16A:CPNE5:CNR1:HOXA11:YWHAG:CREB3L2:KEL:STAR:LYN:S1PR3:KLF4:PAEP
GO_bp	GO_REGULATION_OF_ION_TRANSMEMBRANE_TRANSPORT	467	47	1.218556082095827e-09	2.634231530413038e-07	RNF207:MTOR:KCNC4:GJA5:CTSS:HCN3:PSEN2:KCNJ11:ABCC8:TRPC6:UBASH3B:KCNA1:PTPN6:PSEN1:KCNK10:MYO5A:KIAA1199:GRIN2A:CORO1A:PIRT:CACNB1:STAC2:CACNG1:KCNJ16:KCNJ2:KCNJ14:OSR1:SCN3A:PER2:KCNS1:LIME1:MAPK8IP2:SHANK3:SCN5A:CACNA2D2:KCNAB1:FGF12:STIM2:DIAPH1:KCNIP1:ABCB1:KEL:LYN:GEM:KCNV2:UBQLN1:PTPN3
GO_bp	GO_DEFENSE_RESPONSE	1684	113	1.9987793407246064e-09	4.197436615521673e-07	TRIM62:BCL10:F3:NRAS:CTSS:CTSK:TNFAIP8L2:DCST1:ADAM15:ASH1L:SYT11:ARHGEF2:APCS:SELP:CFHR5:ZMYND11:GPAM:DMBT1:HPX:IPO7:CRTAM:PTPN6:C1S:PSME1:COCH:LRFN5:PSEN1:MAPKBP1:PLA2G4B:CORO1A:CCL17:NFATC3:SHPK:TRPV1:SHPK:CLEC10A:MAPK7:LYZL6:IFI35:TBKBP1:TRIM25:RPS6KB1:PSMD12:NPLOC4:SERPINB4:MUC16:CD97:PTGER1:IL12RB1:CXCL17:CEACAM1:FPR2:PUM2:IL1RL1:IL18R1:IL18RAP:SLC11A1:BPIFA2:TGM2:WFDC12:SEMG1:SEMG2:SLPI:ZBP1:APOL2:APOL1:APOBEC3B:TAB1:PPARA:MAPK8IP2:ACKR2:HYAL3:HYAL1:HYAL2:TUSC2:FOXP1:CD200R1:RAB43:ABCF3:PYDC2:IL8:CXCL6:PF4V1:CXCL1:PLAC8:EIF4E:CFI:NPY5R:DROSHA:PTGER4:ISL1:C5orf30:NDFIP1:WRNIP1:EDN1:HLA-A:TUBB:FLOT1:MUC21:C4A:CNR1:TNFAIP3:ESR1:TPST1:CD36:STAR:LYN:SCRIB:UBQLN1:S1PR3:KLF4:LRSAM1:FCN2:TUBB4B
GO_bp	GO_POSITIVE_REGULATION_OF_MULTICELLULAR_ORGANISMAL_PROCESS	1771	117	2.490637900390234e-09	5.085052379963395e-07	RNF207:CLSTN1:MTOR:SPEN:NBL1:LDLRAP1:MACF1:TIE1:MPL:CDC20:TAL1:FOXE3:BCL10:DDAH1:F3:HIPK1:GJA5:ARNT:ARHGEF2:SEMA4A:SELP:ABL2:ASPM:GDF2:TACR2:C10orf54:ZFYVE27:GPAM:APBB1:ABCC8:UCP2:TENM4:TRPC6:YAP1:CRTAM:CDON:SOX5:HOXC11:NCKAP1L:FBXW8:PRKAB1:POSTN:FOXG1:PSEN1:NUMB:TCF12:CHRNB4:SH3GL3:RRN3:TGFB1I1:TRPV1:DHX33:RNF112:RND2:BRCA1:PLEKHM1:TBX21:SPAG9:RPS6KB1:RNF157:NPLOC4:MBP:TCF3:IL12RB1:GSK3A:MEGF8:CXCL17:CEACAM1:CARD8:OSR1:PUM2:NCOA1:IL1RL1:IL18R1:CREB1:SLC11A1:VIL1:PER2:ZBP1:C21orf91:PLXNB2:SHANK3:TGFBR2:TRAK1:GNAI2:HYAL1:HYAL2:TUSC2:CACNA2D2:FOXP1:DVL3:MAP3K13:IL8:PLAC8:TERT:NIPBL:PTGER4:ISL1:HDAC3:PDGFRB:EDN1:FLOT1:CPNE5:CNR1:TNFAIP3:SOD2:THBS2:ITGB8:HOXA11:HSPB1:CD36:CREB3L2:STAR:LYN:CA2:KLF4:PAEP
GO_bp	GO_NEUROGENESIS	1594	108	2.761848364011049e-09	5.486374452832759e-07	HES2:MTOR:SPEN:NBL1:CDC42:MACF1:CDC20:SZT2:TAL1:RPE65:OLFM3:HIPK1:PHGDH:CERS2:PRUNE:SEMA6C:ARHGEF2:SEMA4A:UHMK1:ABL2:ASPM:CRB1:ZNF488:CDH23:ZFYVE27:APBB1:WEE1:ABCC8:DBX1:TENM4:TRPC6:YAP1:BTG4:CDON:OPCML:KCNA1:C12orf57:SOX5:SLC11A2:NCKAP1L:FBXW8:CIT:ULK1:POSTN:NRL:FOXG1:PSEN1:NUMB:TRPM1:TCF12:SKOR1:SH3GL3:GRIN2A:RRN3:SIAH1:MAPK7:RNF112:RND2:SPAG9:RNF157:FSCN2:SKOR2:TCF3:S1PR5:MEGF8:FPR2:NCOA1:ALK:BCL11A:PEX13:CREB1:CTDSP1:HES6:PER2:MMP24:C21orf91:CELSR1:PLXNB2:MAPK8IP2:SHANK3:HDAC11:TRAK1:CCDC66:C3orf17:GSK3B:DVL3:MAP3K13:AREG:AREGB:EIF4E:TERT:NIPBL:ISL1:FLOT1:CPNE5:CNR1:PEX7:EVX1:YWHAG:NYAP1:CREB3L2:KEL:GFRA2:STAR:LYN:SCRIB:NRBP2:KLF4:PBX3
GO_bp	GO_EMBRYO_DEVELOPMENT	986	76	3.512311488495816e-09	6.793549852748486e-07	RNF207:HES2:ZBTB17:TIE1:TAL1:STIL:BCL10:HIPK1:PHGDH:GJA5:HORMAD1:ARNT:CTR9:TENM4:YAP1:CDON:AKAP3:EMG1:HOXC11:APAF1:FBXW8:POLE:RNASEH2B:FOXG1:PSEN1:PKD1:RRN3:TGFB1I1:ANKRD11:B9D1:MAPK7:RNF112:HNF1B:BRCA1:MBTD1:RPL38:MBP:INSR:KEAP1:MEGF8:TPO:OSR1:NCOA1:ATP6V1B1:AFF3:HES6:RTCB:TAB1:CELSR1:PLXNB2:TGFBR2:HYAL1:LRIG1:ARL13B:IFT57:NPHP3:DVL3:SLC34A2:IL8:NIPBL:ISL1:C5orf20:TIFAB:CDX1:MGAT1:EDN1:VPS52:HOXA10:HOXA11:EVX1:SEPT7:EXOC4:NCAPG2:RDH10:SCRIB:KLF4
GO_bp	GO_MACROMOLECULE_CATABOLIC_PROCESS	1366	96	3.6440480894754298e-09	6.867629091703694e-07	RPL22:TARDBP:EXOSC10:MTOR:CDC20:DNASE2B:OVGP1:CSDE1:CTSS:CTSK:DCST1:UBQLN4:SMG5:F13B:FMOD:CUL2:UBE2L6:PGA3:SYVN1:HSPA8:USP5:SPSB2:APAF1:FBXW8:SIAH3:RNASEH2B:UCHL3:ABHD13:DCAF11:PSME1:FBXO33:PSEN1:HERC2:OTUD7A:ANP32A:ADAMTS7:KIAA1199:ACAN:PKD1:GRIN2A:NTAN1:SMG1:TGFB1I1:LONP2:SIAH1:N4BP1:METTL16:DERL2:RPL19:KPNB1:TBX21:TRIM25:RNFT1:PSMD12:RPL38:TNRC6C:NPLOC4:UBXN6:KEAP1:GIPC1:DEDD2:GSK3A:CEACAM1:CBLC:

						C19orf68:PUM2:PPP1CB:XPO1:ANAPC1:SLC11A1:CHMP4B:USP25:RPL32:DAZL:RPL14:HYAL3:HYAL1:HYAL2:GSK3B:ARMC8:XRN1:AP2M1:DROSHA:RPL37:SKIV2L2:NDFIP1:PRSS16:RPS18:TNFAIP3:RNASET2:HSPB1:RPL7:UBQLN1:PTPN3:RPL12:LRSAM1
GO_bp	GO_CELL_ACTIVATION	1408	98	4.0653733773327674e-09	7.47012358084896e-07	MTOR:CDC42:TXLNA:MPL:BCL10:VAV3:NRAS:CTSS:TNFAIP8L2:HRNR:GON4L:SEMA4A:PEAR1:FCGR2A:HSPA6:SELP:CSRP1:RAB18:C10orf54:PSAP:GPAM:LINC00610:COMMD9:TRPC6:UBASH3B:HSPA8:PTPN6:METTL7A:NCKAP1L:RAP1B:APAF1:LCP1:TNFSF13B:LRFN5:PSEN1:CHRNB4:CORO1A:SHPK:TRPV1:SHPK:IKZF3:VAT1:KPNB1:TBX21:DGKE:PSMD12:MYL12A:TCF3:CD320:CD97:PKN1:IL12RB1:CD79A:POU2F2:CEACAM1:KDELR1:CD33:FPR2:IL1RL1:IL18R1:IL18RAP:MERTK:IGFBP2:SLC11A1:SLPI:DNAJC5:SYNGR1:WBP2NL:CYB5R3:NCAPH2:ARSA:TGFBR2:HYAL2:TUSC2:FOXP1:GP9:ARMC8:IL8:CXCL6:PF4V1:CXCL1:PLAC8:DROSHA:PTGER4:MEGF10:DIAPH1:NDFIP1:PDGFRB:EDN1:TUBB:RAB44:CNR1:TNFAIP3:RNASET2:LAT2:HSPB1:CD36:LYN:CDH17:TUBB4B
GO_bp	GO_REPRODUCTION	1433	99	4.930370480484783e-09	8.838590983308086e-07	MTOR:PHC2:CDC20:HOOK1:RBM15:OVGP1:WDR77:CSDE1:HORMAD1:ARNT:SMCP:ADAM15:ASH1L:ASPM:TUBB8:CREM:TACR2:PSAP:ABCC8:UCP2:TRPC6:RBM7:DDX25:AKAP3:PHB2:PTHLH:LGR5:FBXW8:FNDC3A:FANCM:TDRD9:HERC2:PLA2G4B:PKD1:PRSS21:SIAH1:PRMT7:LYZL6:ZPBP2:SPATA32:RPS6KB1:SSTR2:AZI1:TAF4B:SAFB2:INSR:PAFAH1B3:OSR1:NCOA1:PLB1:SPDYA:FSHR:MERTK:IGFBP2:IGFBP5:PANK2:PCNA:PI3:SEMG1:SEMG2:OSBP2:RTCB:APOL2:WBP2NL:FBLN1:PKDREJ:NCAPH2:SYCE3:MAPK8IP2:DAZL:TGFBR2:HYAL3:PSAPL1:AREG:AREGB:NPY5R:NIPBL:PPAP2A:C5orf20:TIFAB:PDGFRB:ADAM19:SOX30:EDN1:MEI4:CNR1:ESR1:SYNE1:ITGB8:HOXA10:HOXA11:SEPT7:CLDN4:STAR:TERF1:RDH10:LRRC6:SPATA31A4:SPATA31A7:PAPPA:PAEP
GO_bp	GO_CELLULAR_HOMEOSTASIS	967	74	7.663347683637491e-09	1.341085844636561e-06	TXNDC12:LRRC8B:LRRC8C:ABL2:NUCKS1:GDF2:CDH23:HPX:KCNJ11:ABCC8:GYLTL1B:UCP2:DGAT2:TRPC6:UBASH3B:PTPN6:SLC11A2:P2RX2:POLE:PCK2:TXNDC16:ERO1L:PSEN1:TRPM1:MYO5A:KIAA1199:PKD1:GRIN2A:CORO1A:CALB2:TRPV1:KCNJ2:ZNF236:PTGER1:DEDD2:GRIN2D:FPR2:ATP6V1B1:SLC4A5:DCTN1:SLC9A4:SLC11A1:PCNA:TGM2:LIME1:MCHR1:SMDT1:SCO2:SHANK3:ACKR2:ATP2C1:ACKR4:SLC34A2:STIM2:SLC4A4:PTGER4:SLC12A2:DIAPH1:NDFIP1:EDN1:HCRTR2:CNR1:ESR1:RFC2:CD36:KEL:STAR:LYN:TERF1:CA2:CALB1:ENY2:S1PR3:SLC34A3
GO_bp	GO_RESPONSE_TO_LIPID	915	71	8.829658420373816e-09	1.5092555672034314e-06	C1orf64:TIE1:BCL10:ADAM15:CLK2:BGLAP:SELP:ABL2:GPAM:CTR9:KCNJ11:ABCC8:SCGB2A1:UCP2:UCP3:DGAT2:YAP1:FDX1:LINC01059:GRAMD1B:PHB2:POSTN:PCK2:TGFB1I1:SHPK:SHPK:BRCA1:TRIM25:RPS6KB1:SSTR2:SAFB2:SAFB:PTGER1:NFKBIB:CARD8:OSR1:NCOA1:CREB1:IGFBP2:SLC11A1:PCNA:SLPI:PPARA:TGFBR2:FOXP1:NR1I2:UBA5:XRN1:PLSCR4:IL8:CXCL6:PF4V1:CXCL1:AREG:AREGB:EIF4E:PTGER4:ISL1:PPAP2A:PDGFRB:SOX30:EDN1:CDKN1A:CNR1:TNFAIP3:ESR1:CLDN4:CD36:STAR:LYN:CA2:CHMP5:KLF4:PAPPA
GO_bp	GO_RESPONSE_TO_ENDOGENOUS_STIMULUS	1634	108	1.0370985313458476e-08	1.7284557359688804e-06	MTOR:C1orf64:NBL1:RPE65:LAMTOR5:TRIM33:CTSS:HCN3:PKLR:ARHGEF2:BGLAP:FMOD:NUCKS1:GDF2:SFRP5:GPAM:KCNJ11:ABCC8:SCGB2A1:UCP2:UCP3:YAP1:FDX1:PHB2:SOX5:RAP1B:LGR5:APAF1:POSTN:IPO5:PCK2:PSEN1:MYO5A:SKOR1:ADAMTS7:CORO1A:SULT1A3:TGFB1I1:POLR2C:ESRP2:SMPD3:TRPV1:GLP2R:FAM83G:MAPK7:CACNB1:BRCA1:RPS6KB1:SSTR2:SKOR2:SAFB2:SAFB:INSR:ARHGEF18:GIPC1:GSK3A:MEGF8:CEACAM1:FPR2:NCOA1:FSHR:BCL11A:ATP6V1B1:CREB1:IGFBP2:IGFBP5:VIL1:NCL:PCNA:EIF6:TAB1:PPARA:TGFBR2:GNAI2:HYAL1:HYAL2:FOXP1:NR1I2:GSK3B:UBA5:XRN1:FGF12:IL8:AREG:AREGB:EIF4E:PTGER4:ISL1:PPAP2A:DIAPH1:PDGFRB:SOX30:CPEB4:EDN1:CDKN1A:ESR1:CRHR2:CLDN4:YWHAG:CD36:PRKAR2B:STAR:LYN:CA2:CYP11B1:CYP11B2:CHMP5:KLF4:PAPPA
GO_bp	GO_ION_HOMEOSTASIS	810	65	1.058238205695233e-08	1.7284557359688804e-06	GJA5:ABL2:GDF2:CDH23:GPAM:HPX:DGAT2:TRPC6:UBASH3B:KCNA1:PTPN6:SLC11A2:P2RX2:ERO1L:PSEN1:TRPM1:MYO5A:KIAA1199:PKD1:GRIN2A:CORO1A:CALB2:TRPV1:KCNJ2:PTGER1:GRIN2D:FPR2:ATP6V1B1:SLC4A5:SLC9A4:SLC11A1:TGM2:MC3R:LIME1:MCHR1:SMDT1:SCO2:SCN5A:ACKR2:GNAI2:HYAL2:ATP2C1:ACKR4:FGF12:MFI2:SLC34A2:STIM2:SLC4A4:PTGER4:SLC12A2:DIAPH1:NDFIP1:EDN1:HCRTR2:CNR1:ESR1:CD36:KEL:LYN:CA2:CALB1:CYP11B2:S1PR3:PTPN3:SLC34A3
GO_bp	GO_CELL_CELL_SIGNALING	1638	108	1.1791781583218185e-08	1.8548933673572306e-06	RNF207:CLSTN1:TARDBP:MTOR:HTR6:CDC42:MACF1:CDC20:KCNC4:GJA5:ASH1L:SYT11:ASPM:CRB1:TACR2:SFRP5:GRK5:CTR9:KCNJ11:ABCC8:OR5T1:ARL2:UCP2:YAP1:HSPA8:KCNA1:PTHLH:RAP1B:LGR5:P2RX2:PSME1:PSEN1:MYO5A:CHRNB4:MESDC2:PKD1:GRIN2A:TGFB1I1:CCL17:SMPD3:CALB2:TRPV1:HNF1B:CACNB1:GJC1:DGKE:PSMD12:TNRC6C:MBP:SH3GL1:CD97:GIPC1:OR10H2:OR10H3:GSK3A:GRIN2D:CD33:SIGLEC6:MERTK:PKP4:CREB1:IGFBP2:PER2:DNAJC5:SYNGR1:CELSR1:MAPK8IP2:SHANK3:SCN5A:MYRIP:GNAI2:CACNA2D2:STX19:GSK3B:NPHP3:DVL3:AP2M1:SENP2:FGF12:CXCL6:AREG:AREGB:EIF4E:NPY5R:TERT:ISL1:MCTP1:FCHSD1:SNCB:EDN1:FLOT1:HCRTR2:CNR1:TNFAIP3:ESR1:DACT2:CRHR2:YWHAG:PRKAR2B:EXOC4:BLK:STAR:LYN:CA2:CALB1:ENY2:SCRIB:KLF4:CEL

GO_bp	GO_CELLULAR_LIPID_METABOLIC_PROCESS	885	69	1.186122289330474e-08	1.8548933673572306e-06	MTOR:A3GALT2:ELOVL1:ACOT11:RPE65:ABCD3:VAV3:CERS2:SOAT1:CREM:RBP3:PSAP:GPAM:SMPD1:UCP3:MOGAT2:DGAT2:LPCAT3:MTMR6:PCK2:PLA2G4B:PDXDC1:SMG1:LONP2:PLA2G15:SMPD3:DGKE:MPPE1:IMPA2:ST8SIA5:LIPE:FPR2:PLB1:PEX13:ATP6V1B1:PER2:PANK2:NAGA:CYP2D6:PPARA:CPT1B:ARSA:ACAD11:RBP2:RBP1:GK5:PSAPL1:PLA2G12A:PPAP2A:PDGFRB:B3GALT4:GSTA1:CNR1:PEX7:AIG1:ITGB8:CD36:PRKAR2B:ESYT2:STAR:LYN:NSMAF:RDH10:KLF4:RP11-203J24.9:ST6GALNAC6:ST6GALNAC4:PIP5KL1:DPM2:CEL
GO_bp	GO_MITOTIC_CELL_CYCLE	998	75	1.2995269226979476e-08	1.985777738674755e-06	CDC20:TAL1:STIL:CKS1B:MSTO1:ARHGEF2:LMNA:TUBB8:MCM10:CUL2:REEP3:WEE1:CDCA5:BTG4:PTPN6:PHB2:CIT:POLE:ANKLE2:RNASEH2B:PSME1:FOXG1:DLGAP5:ARPP19:PKD1:SMPD3:BRCA1:KPNB1:TUBD1:RPS6KB1:PSMD12:AZI1:TUBB4A:INSR:ERF:PPP1CB:SPDYA:DCTN1:RTKN:ANAPC1:CTDSP1:PCNA:CHMP4B:GTSE1:NCAPH2:HYAL1:NEK11:EIF4E:TERT:NIPBL:MCIDAS:CCNO:KIF2A:CKS1B:PDGFRB:LSM11:EDN1:MDC1:TUBB:CDKN1A:HECA:FGFR1OP:SEPT7:YWHAG:ABCB1:MEPCE:PRKAR2B:CEP41:GEM:GML:SCRIB:CHMP5:KLF4:PTPN3:NACC2:TUBB4B
GO_bp	GO_POSITIVE_REGULATION_OF_CELLULAR_COMPONENT_ORGANIZATION	1188	85	1.32385182578317e-08	1.985777738674755e-06	CLSTN1:MTOR:CDC42:LDLRAP1:MACF1:MPL:CDC20:TSPAN1:TAL1:SETDB1:LMNA:SEMA4A:ABL2:ZFYVE27:APBB1:CTR9:ABCC8:ARL2:CDCA5:PHB2:NCKAP1L:FBXW8:ULK1:LCP1:ERCC5:FAM179B:DLGAP5:PSEN1:MOAP1:MESDC2:RRN3:CORO1A:SLX1A:SMPD3:DHX33:RND2:BRCA1:PLEKHM1:NPEPPS:RNF157:SH3GL1:INSR:NPHS1:GSK3A:MEGF8:DCTN1:CREB1:VIL1:MFF:DSTN:C21orf91:PLXNB2:SHANK3:TRAK1:HYAL1:ZMYND10:FAM208A:GSK3B:DVL3:MAP3K13:MFI2:SLAIN2:NIPBL:ISL1:FCHSD1:PDGFRB:EDN1:LRRC16A:FLOT1:CPNE5:C6orf89:CNR1:ESR1:GTF2H5:THBS2:SEPT7:YWHAG:CD36:CREB3L2:PPP3CC:LYN:TERF1:CDH17:XPA:LRSAM1
GO_bp	GO_G_PROTEIN_COUPLED_RECEPTOR_SIGNALING_PATHWAY	1363	93	2.640076579577436e-08	3.880912571978831e-06	GPR153:HTR6:TM2D1:LPHN2:VAV3:ARHGEF2:ARHGEF11:OR14K1:TACR2:PSAP:RGR:GRK5:OR52B4:OR51T1:OR4C15:OR4C16:OR4P4:OR4S2:OR4C6:OR5T1:OR8H1:OR8K3:OR8K1:OR8J1:OR8U1:OR5R1:PTPN6:GPRC5A:PTHLH:PDE1B:OR10P1:LGR5:MLNR:GPR180:TRPM1:SULT1A3:CCL17:GLP2R:DGKE:SSTR2:INSR:ARHGEF18:OR1M1:OR7G2:OR7G1:OR7G3:PDE4A:S1PR5:RLN3:CD97:PTGER1:GIPC1:OR10H2:OR10H3:GSK3A:TMEM145:FPR2:ALK:FSHR:TGM2:MC3R:MCHR1:CELSR1:ACKR2:GNAI2:GSK3B:ACKR4:PSAPL1:IL8:CXCL6:CXCL1:AREG:AREGB:NPY1R:NPY5R:PTGER4:PPAP2A:SLC12A2:PDGFRB:EDN1:OR10C1:HCRTR2:CNR1:ESR1:CRHR2:GNG11:GPR22:TAS2R39:TAS2R40:CA2:S1PR3:OR1L3:OR1L4:OR1L6
GO_bp	GO_REGULATION_OF_IMMUNE_SYSTEM_PROCESS	1606	105	2.9180684744114447e-08	4.205451624887082e-06	MTOR:NBL1:CDC42:MPL:TAL1:BCL10:VAV3:RBM15:NRAS:CTSS:CTSK:ARNT:TNFAIP8L2:DCST1:BGLAP:APCS:FCGR2A:FCGR3A:FCRLB:SELP:CFHR5:C10orf54:GPAM:DMBT1:HPX:CTR9:NCR3LG1:YAP1:UBASH3B:CRTAM:PTPN6:C1S:NCKAP1L:TNFSF13B:PSME1:COCH:LRFN5:PSEN1:TCF12:CORO1A:NFATC3:SMPD3:SHPK:SHPK:CLEC10A:IKZF3:TBX21:PSMD12:TNRC6C:NPLOC4:PHLPP1:SERPINB4:TCF3:CD320:MUC16:PKN1:IL12RB1:CD79A:CXCL17:CEACAM1:CD33:FPR2:PUM2:IL1RL1:IL18R1:IL18RAP:MERTK:CREB1:IGFBP2:SLC11A1:EIF6:ZBP1:LIME1:TAB1:TGFBR2:FOXP1:PROS1:CD200R1:KLHL6:IL8:CXCL6:CFI:NPY5R:DROSHA:PTGER4:NDFIP1:EDN1:HLA-A:FLOT1:MUC21:C4A:CNR1:TNFAIP3:ESR1:LAT2:CD36:PILRB:PILRA:NCAPG2:BLK:ASH2L:LYN:CA2:SCRIB:UBQLN1:FCN2
GO_bp	GO_NEGATIVE_REGULATION_OF_RESPONSE_TO_STIMULUS	1629	106	3.178414112724799e-08	4.49256610163986e-06	MTOR:NBL1:CDC42:SZT2:TXNDC12:DDAH1:TRIM33:CERS2:SEMA6C:TNFAIP8L2:DCST1:ASH1L:ARHGEF2:LMNA:SEMA4A:PEAR1:APCS:FCRLB:ABL2:PHLDA3:ZMYND11:PSAP:FAS:SFRP5:DUSP8:SMPD1:ABCC8:SYVN1:UCP2:YAP1:UBASH3B:PTPN6:PHB2:GPRC5A:PSME1:LRFN5:PSEN1:OTUD7A:MAPKBP1:SKOR1:RRN3:TGFB1I1:SIAH1:DERL2:EPN2:MAPK7:BRCA1:TBX21:RPS6KB1:PSMD12:NPLOC4:SKOR2:PHLPP1:SERPINB4:EPS15L1:GSK3A:MEGF8:CXCL17:CEACAM1:CBLC:CARD8:OSR1:IL1RL1:IGFBP2:IGFBP5:USP25:FBLN1:PPARA:MAPK8IP2:TGFBR2:GNAI2:HYAL2:NPRL2:FOXP1:PROS1:CD200R1:GSK3B:NPHP3:RASA2:DVL3:PYDC2:IL8:AREG:AREGB:NPY5R:TERT:PTGER4:ISL1:MCTP1:C5orf30:HDAC3:NDFIP1:EDN1:CDKN1A:CNR1:TNFAIP3:ESR1:SOD2:DACT2:HSPB1:RB1CC1:LYN:ENY2:SCRIB:UBQLN1:KLF4:PTPN3
GO_bp	GO_REGULATION_OF_CELL_DEATH	1697	109	3.9264544047123674e-08	5.445177334836962e-06	TARDBP:MTOR:STIL:FOXE3:TXNDC12:BCL10:F3:VAV3:LAMTOR5:TNFAIP8L2:ARHGEF2:LMNA:ARHGEF11:PHLDA3:PSEN2:ZMYND11:GDF2:PSAP:FAS:GPAM:GRK5:SMPD1:APBB1:SYVN1:UCP2:YAP1:ERC1:PHB2:NCKAP1L:APAF1:ANKLE2:ERCC5:MAP3K9:RBM25:PSEN1:MOAP1:GRIN2A:RRN3:CORO1A:SULT1A3:SIAH1:CIAPIN1:TRPV1:MAPK7:HNF1B:IKZF3:BRCA1:RPS6KB1:RNF157:PHLPP1:ARHGEF18:DEDD2:GSK3A:CARD8:OSR1:NCOA1:ALK:RTKN:MERTK:CREB1:VIL1:MFF:CHMP4B:TGM2:DNAJC5:PPARA:SYCE3:MAPK8IP2:RAD18:GNAI2:HYAL2:FOXP1:SHQ1:C3orf38:IFT57:CD200R1:GSK3B:PLAC8:NPY5R:TERT:CARD6:ISL1:HDAC3:PDGFRB:CPEB4:SNCB:EDN1:IER3:RGL2:CDKN1A:CNR1:TNFAIP3:ESR1:SOD2:SEPT7:HSPB1:YWHAG:CD36:PIP:PPP3CC:STAR:RB1CC1:LYN:NSMAF:SCRIB:NRBP2:UBQLN1:KLF4:NACC2

GO_bp	GO_REGULATION_OF_CELL_CYCLE	1201	84	4.3881059561124794e-08	5.9726997735975414e-06	TARDBP:MTOR:ZBTB17:CDC42:CDC20:TAL1:STIL:FOXE3:LAMTOR5:HORMAD1:CKS1B:YY1AP1:UHMK1:ASPM:CUL2:GRK5:APBB1:IPO7:WEE1:CDCA5:BTG4:PTPN6:PHB2:APAF1:PRKAB1:CIT:RNASEH2B:IPO5:PSME1:FOXG1:DLGAP5:MGA:PKD1:SMPD3:RNF112:BRCA1:RPS6KB1:PSMD12:AZI1:TUBB4A:INSR:GIPC1:SPDYA:XPO1:DCTN1:ANAPC1:PKP4:CTDSP1:PER2:PCNA:CHMP4B:GTSE1:HYAL1:RASSF1:NEK11:IL8:EIF4E:TERT:NIPBL:MCIDAS:CCNO:CKS1B:PDGFRB:LSM11:EDN1:MDC1:TUBB:CDKN1A:C6orf89:TNFAIP3:HECA:FGFR1OP:SEPT7:YWHAG:MEPCE:PRKAR2B:CEP41:TERF1:GML:SCRIB:CHMP5:KLF4:PTPN3:NACC2:TUBB4B
GO_bp	GO_BEHAVIOR	592	51	4.4836826261923006e-08	5.991830418638802e-06	MTOR:TAL1:ABL2:TACR2:CDH23:PSAP:ABCC8:OR5T1:C12orf57:SLC11A2:PDE1B:P2RX2:PSEN1:KCNK10:MYO5A:CHRNB4:GRIN2A:NTAN1:TRPV1:DERL2:PIRT:RPS6KB1:INSR:CIC:GRIN2D:NCOA1:ALK:PEX13:ATP6V1B1:DCTN1:CREB1:MC3R:MCHR1:PPARA:CELSR1:MAPK8IP2:SHANK3:KCNAB1:FGF12:EIF4E:NPY1R:NPY5R:C5orf20:TIFAB:HCRTR2:CNR1:ASL:PRKAR2B:STAR:CALB1:PBX3
GO_bp	GO_SMALL_MOLECULE_BIOSYNTHETIC_PROCESS	666	55	5.569114095876083e-08	7.189465755981823e-06	ACOT7:UBIAD1:RIMKLA:ELOVL1:ACOT11:ABCD3:PHGDH:HMGCS2:CLK2:FDPS:ADCK3:ALDH18A1:GPAM:TM7SF2:PC:DGAT2:AASDHPPT:NDUFA9:TPI1:ENO2:SDS:PRKAB1:PCK2:MYO5A:ARPP19:DCTPP1:COQ9:BRCA1:KPNB1:IMPA2:PDE4A:TECR:CEACAM1:PER2:EIF6:UCKL1:LSS:CYP2D6:CYB5R3:PPARA:TYMP:ENTPD3:CYP8B1:DHFRL1:CNBP:RBP1:COQ2:EDN1:ASL:FAM3C:STAR:RDH10:CYP11B1:CYP11B2:IPPK
GO_bp	GO_REGULATION_OF_ANATOMICAL_STRUCTURE_MORPHOGENESIS	1130	80	5.575504055659373e-08	7.189465755981823e-06	RNF207:MTOR:FBLIM1:CDC42:MACF1:TIE1:MPL:DDAH1:F3:HIPK1:SEMA6C:SEMA4A:GDF2:ZFYVE27:SFRP5:ABCC8:TENM4:TRPC6:PHB2:HOXC11:FBXW8:POSTN:TNFSF13B:PSME1:COCH:PSEN1:CORO1A:EPN2:MAPK7:HNF1B:VAT1:RND2:BRCA1:PSMD12:RNF157:MBP:ARHGEF18:PKN1:MEGF8:CEACAM1:ZNF135:OSR1:BCL11A:RND3:VIL1:MFF:FBLN1:CELSR1:PLXNB2:SHANK3:TGFBR2:TRAK1:HYAL1:FOXP1:GSK3B:NPHP3:DVL3:AP2M1:MAP3K13:MFI2:IL8:TERT:ISL1:DIAPH1:CDX1:EDN1:LRRC16A:FLOT1:CPNE5:TNFAIP3:ESR1:THBS2:ITGB8:HOXA11:SEPT7:HSPB1:CD36:KEL:KLF4:PALM2
GO_bp	GO_CELLULAR_RESPONSE_TO_OXYGEN_CONTAINING_COMPOUND	1118	79	7.311724115006456e-08	9.26571935263749e-06	MTOR:SZT2:RPE65:BCL10:LAMTOR5:ADAM15:HCN3:PKLR:ARHGEF2:BGLAP:ABL2:NUCKS1:PSAP:CTR9:KCNJ11:UCP2:DGAT2:TRPC6:YAP1:FDX1:LINC01059:GRAMD1B:PHB2:RAP1B:IPO5:PCK2:PSEN1:MYO5A:SULT1A3:SMPD3:SHPK:TRPV1:SHPK:GLP2R:MAPK7:RNF112:CACNB1:BRCA1:RPS6KB1:SSTR2:ZNF236:INSR:NFKBIB:GSK3A:CEACAM1:CARD8:FPR2:OSR1:BCL11A:ATP6V1B1:IL18RAP:CREB1:IGFBP5:PCNA:GNAI2:GSK3B:XRN1:PLSCR4:IL8:CXCL6:PF4V1:CXCL1:EIF4E:PTGER4:CPEB4:EDN1:TNFAIP3:ESR1:SOD2:CRHR2:YWHAG:CD36:PRKAR2B:STAR:LYN:CA2:ENY2:CYP11B1:CYP11B2:CHMP5:KLF4
GO_bp	GO_EPITHELIAL_CELL_DIFFERENTIATION	762	60	7.564728274796534e-08	9.355440012936492e-06	CDC42:FOXE3:WDR77:CTSK:HRNR:FLG:LCE1F:LCE1E:LCE1D:LCE1C:LCE1B:TMEM79:GDF2:PSAP:ACTA2:DMBT1:KRTAP5-1:KRTAP5-2:KRTAP5-3:KRTAP5-4:KRTAP5-5:EHF:YAP1:RAP1B:FNDC3A:PCK2:FRMD6:TGFB1I1:B9D1:HNF1B:SAFB2:KEAP1:NPHS1:GSK3A:CNFN:CEACAM1:OSR1:SLC4A5:SLC9A4:PKP4:CREB1:VIL1:PCNA:BFSP1:PI3:KRTAP21-3:KRTAP8-1:GSK3B:PSAPL1:MCIDAS:CCNO:CDKN1A:GSTA2:GSTA1:ESR1:DACT2:CLDN3:SCRIB:S1PR3:KLF4
GO_bp	GO_REGULATION_OF_NERVOUS_SYSTEM_DEVELOPMENT	910	68	7.713138710327111e-08	9.355440012936492e-06	HES2:CLSTN1:MTOR:SPEN:NBL1:MACF1:CDC20:CERS2:PRUNE:SEMA6C:ARHGEF2:SEMA4A:ABL2:ASPM:ZFYVE27:APBB1:ABCC8:LRFN4:TENM4:TRPC6:YAP1:CDON:FBXW8:FOXG1:LRFN5:PSEN1:NUMB:TCF12:SH3GL3:RRN3:RNF112:RND2:SPAG9:RNF157:TCF3:S1PR5:MEGF8:NCOA1:ALK:BCL11A:CREB1:CTDSP1:HES6:PER2:C21orf91:CELSR1:PLXNB2:TYMP:SHANK3:TRAK1:C3orf17:GSK3B:NPHP3:DVL3:MAP3K13:EIF4E:TERT:NIPBL:ISL1:CPNE5:CNR1:THBS2:YWHAG:CREB3L2:KEL:STAR:LYN:KLF4
GO_bp	GO_CELLULAR_MACROMOLECULE_CATABOLIC_PROCESS	1139	80	7.764378786246612e-08	9.355440012936492e-06	RPL22:TARDBP:EXOSC10:MTOR:CDC20:DNASE2B:CSDE1:CTSS:CTSK:DCST1:UBQLN4:SMG5:F13B:CUL2:UBE2L6:SYVN1:HSPA8:USP5:SPSB2:APAF1:FBXW8:SIAH3:RNASEH2B:UCHL3:DCAF11:PSME1:FBXO33:PSEN1:HERC2:OTUD7A:ANP32A:ADAMTS7:PKD1:NTAN1:SMG1:TGFB1I1:LONP2:SIAH1:N4BP1:METTL16:DERL2:RPL19:KPNB1:TBX21:TRIM25:RNFT1:PSMD12:RPL38:TNRC6C:NPLOC4:UBXN6:KEAP1:GIPC1:DEDD2:GSK3A:CBLC:C19orf68:PUM2:XPO1:ANAPC1:SLC11A1:CHMP4B:USP25:RPL32:DAZL:RPL14:GSK3B:ARMC8:XRN1:AP2M1:DROSHA:RPL37:SKIV2L2:RPS18:TNFAIP3:RNASET2:HSPB1:RPL7:UBQLN1:RPL12
GO_bp	GO_MICROTUBULE_BASED_PROCESS	731	58	9.62113866670431e-08	1.1405704709722046e-05	CDC42:MACF1:CDC20:STIL:HOOK1:SSX2IP:PRUNE:MSTO1:ARHGEF2:LMNA:ASPM:TUBB8:WEE1:ARL2:SAC3D1:DNAJB13:HSPA8:FAM179B:DLGAP5:GOLGA8R:PKD1:SPG7:DNAH9:BRCA1:KPNB1:TUBD1:DNAI2:KIF19:AZI1:TUBB4A:PEX13:XPO1:DCTN1:CHMP4B:GTSE1:TRAK1:RASSF1:ZMYND10:CCDC66:IFT57:GSK3B:NPHP3:SLAIN2:MCIDAS:KIF2A:CETN3:DIAPH1:HDAC3:TUBB:MAP7:FGFR1OP:HSPB1:COPG2:LRRC6:CNTLN:CHMP5:NTMT1:TUBB4B
GO_bp	GO_ORGANELLE_ASSEMBLY	824	63	1.0348911683537779e-07	1.2073730297460742e-05	CCDC28B:CDC20:STIL:SSX2IP:TMOD4:MSTO1:ARHGEF2:ASPM:TUBB8:SAC3D1:LRFN4:DNAJB13:ULK1:LCP1:SCFD1:FAM179B:PSEN1:GOLGA8R:RPL3L:CORO1A:B9D1:ZPBP2:BRCA1:KPNB1:RPL38:DNAI2:AZI1:TUBB4A:PUM2:DCTN1:VIL1:CHMP4B:EIF6:

						WBP2NL:SHANK3:ZMYND10:NPRL2:CCDC66:ARL13B:IFT57:NPHP3:DZIP1L:GMNC:MCIDAS:CCNO:KIF2A:C5orf30:HDAC3:PDGFRB:EDN1:TUBB:FGFR1OP:SEPT7:YWHAG:PRKAR2B:CEP41:RB1CC1:LRRC6:CHMP5:UBQLN1:RPL12:LRSAM1:TUBB4B
GO_bp	GO_DIVALENT_INORGANIC_CATION_TRANSPORT	473	43	1.1512815382051693e-07	1.3221748915324993e-05	PSEN2:CDH23:TRPC6:UBASH3B:PTPN6:PHB2:SLC11A2:ORAI1:P2RX2:ERO1L:PSEN1:NIPA2:NIPA1:TRPM1:MYO5A:KIAA1199:PKD1:GRIN2A:CORO1A:SPG7:TRPV1:RCVRN:CACNB1:STAC2:CACNG1:KCNJ2:GRIN2D:SLC11A1:SEMG1:LIME1:MCHR1:SMDT1:PKDREJ:GNAI2:CACNA2D2:ATP2C1:STIM2:DIAPH1:PDGFRB:LYN:GEM:UBQLN1:SLC25A25
GO_bp	GO_REGULATION_OF_ESTABLISHMENT_OF_PROTEIN_LOCALIZATION	723	57	1.5318765517605193e-07	1.732198870067664e-05	TARDBP:GJA5:GOLPH3L:SYT11:UHMK1:SEC16B:GPAM:APBB1:KCNJ11:ABCC8:ARL2:UCP2:CRTAM:HSPA8:POSTN:SIAH3:LCP1:IPO5:SCFD1:PSEN1:NUMB:MAPKBP1:SLC51B:KIAA1199:DERL2:NPEPPS:AZI1:MBP:GSK3A:CARD8:XPO1:IL1RL1:MFF:PER2:GTSE1:MYRIP:HYAL2:CACNA2D2:FOXP1:CD200R1:GSK3B:PYDC2:PTGER4:ISL1:HDAC3:NDFIP1:CNR1:TNFAIP3:YWHAG:CD36:BLK:PPP3CC:LYN:TERF1:ENY2:SCRIB:PAEP
GO_bp	GO_METAL_ION_HOMEOSTASIS	653	53	1.678808402194341e-07	1.8695820842618797e-05	ABL2:GDF2:CDH23:HPX:TRPC6:UBASH3B:KCNA1:PTPN6:SLC11A2:P2RX2:ERO1L:PSEN1:TRPM1:MYO5A:KIAA1199:PKD1:GRIN2A:CORO1A:CALB2:TRPV1:KCNJ2:PTGER1:GRIN2D:FPR2:ATP6V1B1:SLC11A1:TGM2:MC3R:LIME1:MCHR1:SMDT1:SCO2:ACKR2:GNAI2:HYAL2:ATP2C1:ACKR4:MFI2:STIM2:PTGER4:SLC12A2:DIAPH1:NDFIP1:EDN1:HCRTR2:CNR1:ESR1:CD36:KEL:LYN:CALB1:CYP11B2:S1PR3
GO_bp	GO_LOCOMOTION	1915	117	1.7316488596148577e-07	1.8996446445028663e-05	MTOR:NBL1:HTR6:TRIM62:MACF1:WDR65:TIE1:TSPAN1:SSX2IP:F3:VAV3:CERS2:SEMA6C:SMCP:ADAM15:ASH1L:ARHGEF2:LMNA:SEMA4A:SELP:ABL2:ASPM:GDF2:TACR2:C10orf54:ABCC8:PC:PTPN6:NCKAP1L:POSTN:LCP1:FOXG1:PSEN1:NUMB:MYO5A:KIAA1199:GRIN2A:PDXDC1:CORO1A:SIAH1:CCL17:SLC7A6:SMPD3:MAPK7:RND2:TBX21:SPAG9:TRIM25:RPS6KB1:FSCN2:RALBP1:INSR:PKN1:GIPC1:MEGF8:CXCL17:CEACAM1:FPR2:PEX13:MERTK:RND3:CREB1:IGFBP5:VIL1:CHMP4B:SEMG1:SEMG2:FBLN1:GTSE1:CELSR1:PLXNB2:TYMP:SHANK3:TGFBR2:CMTM8:ACKR2:GNAI2:HYAL1:HYAL2:FOXP1:PROS1:ARL13B:CD200R1:ACKR4:IL8:CXCL6:PF4V1:CXCL1:TERT:NIPBL:PTGER4:ISL1:PPAP2A:KIF2A:MCTP1:C5orf30:MEGF10:SLC12A2:LECT2:DIAPH1:PDGFRB:EDN1:LRRC16A:PTP4A1:PEX7:TNFAIP3:SOD2:FGFR1OP:ITGB8:SEPT7:HSPB1:GFRA2:LYN:LRRC6:SCRIB:KLF4:PIP5KL1
GO_bp	GO_CELLULAR_CHEMICAL_HOMEOSTASIS	803	61	2.0554703914238234e-07	2.221721673083103e-05	ABL2:NUCKS1:GDF2:CDH23:HPX:KCNJ11:ABCC8:UCP2:DGAT2:TRPC6:UBASH3B:PTPN6:SLC11A2:P2RX2:PCK2:ERO1L:PSEN1:TRPM1:MYO5A:KIAA1199:PKD1:GRIN2A:CORO1A:CALB2:TRPV1:KCNJ2:ZNF236:PTGER1:GRIN2D:FPR2:ATP6V1B1:SLC4A5:SLC9A4:SLC11A1:TGM2:LIME1:MCHR1:SMDT1:SCO2:ACKR2:ATP2C1:ACKR4:SLC34A2:STIM2:SLC4A4:PTGER4:DIAPH1:NDFIP1:EDN1:HCRTR2:CNR1:ESR1:CD36:KEL:STAR:LYN:CA2:CALB1:ENY2:S1PR3:SLC34A3
GO_bp	GO_PROTEIN_LOCALIZATION_TO_ORGANELLE	916	67	2.1053277087799807e-07	2.2291188943054e-05	RPL22:TARDBP:SZT2:STIL:LAMTOR5:SYT11:LMNA:SEC16B:ARL5B:IPO7:PEX16:ARL2:CDCA5:HSPA8:PHB2:DAO:SIAH3:KPNA3:IPO5:PSEN1:NUMB:MOAP1:LONP2:RABEP1:RPL19:NPEPPS:KPNB1:RPL38:AZI1:GSK3A:KDELR1:PEX13:XPO1:RTKN:MFF:CHMP4B:GTSE1:RPL32:RPL14:HYAL2:CCDC66:FAM208A:ARL13B:GSK3B:TERT:NIPBL:RPL37:IPO11:DIAPH1:HDAC3:TSPAN17:RPS18:PEX7:TNFAIP3:ESR1:SYNE1:YWHAG:CD36:BCAP29:PPP3CC:TERF1:RPL7:SCRIB:CNTLN:XPA:RPL12:LRSAM1
GO_bp	GO_SYNAPTIC_SIGNALING	712	56	2.1229703755289524e-07	2.2291188943054e-05	CLSTN1:MTOR:HTR6:CDC20:KCNC4:SYT11:TACR2:OR5T1:HSPA8:KCNA1:RAP1B:P2RX2:PSEN1:MYO5A:CHRNB4:GRIN2A:CALB2:TRPV1:CACNB1:GJC1:DGKE:MBP:SH3GL1:GIPC1:OR10H2:OR10H3:GSK3A:GRIN2D:CREB1:DNAJC5:SYNGR1:MAPK8IP2:SHANK3:GNAI2:CACNA2D2:STX19:GSK3B:FGF12:EIF4E:NPY5R:MCTP1:FCHSD1:SNCB:EDN1:FLOT1:HCRTR2:CNR1:CRHR2:YWHAG:PRKAR2B:EXOC4:STAR:CA2:CALB1:SCRIB:CEL
GO_bp	GO_REGULATION_OF_MITOTIC_CELL_CYCLE	660	53	2.3462121990997853e-07	2.4288253047018905e-05	CDC20:TAL1:CKS1B:WEE1:CDCA5:BTG4:PTPN6:PHB2:RNASEH2B:PSME1:FOXG1:DLGAP5:PKD1:SMPD3:BRCA1:RPS6KB1:PSMD12:AZI1:TUBB4A:INSR:DCTN1:ANAPC1:CTDSP1:PCNA:CHMP4B:GTSE1:HYAL1:NEK11:EIF4E:TERT:NIPBL:MCIDAS:CCNO:CKS1B:PDGFRB:LSM11:EDN1:MDC1:TUBB:CDKN1A:HECA:FGFR1OP:SEPT7:YWHAG:MEPCE:PRKAR2B:CEP41:GML:SCRIB:CHMP5:KLF4:PTPN3:NACC2:TUBB4B
GO_bp	GO_PROTEIN_PHOSPHORYLATION	1949	118	2.4410999267864693e-07	2.4919561752611875e-05	TARDBP:MTOR:TIE1:TAL1:HIPK1:NRAS:CKS1B:CLK2:ASH1L:ARHGEF2:UHMK1:ABL2:ZMYND11:GDF2:PSAP:FAS:GRK5:DUSP8:SMPD1:HPX:IPO7:WEE1:TRPC6:UBASH3B:CDON:ERC1:DYRK4:PTPN6:PHB2:GPRC5A:NCKAP1L:RAP1B:NUAK1:PRKAB1:CIT:ULK1:TNFRSF19:IPO5:PSME1:MAP3K9:PSEN1:KIAA1199:PKD1:SMG1:SULT1A3:CCL17:SMPD3:DHX33:MAPK7:NBR1:SPAG9:RPS6KB1:PSMD12:PPP4R1:PHLPP1:MBP:INSR:PKN1:MAST3:NPHS1:GSK3A:LIPE:CXCL17:CEACAM1:CBLC:FPR2:SPDYA:ALK:FSHR:MERTK:CREB1:SLC11A1:CTDSP1:COPS8:LIME1:TAB1:FBLN1:PLXNB2:MAPK8IP2:SHANK3:TGFBR2:GNAI2:HYAL2:NPRL2:GSK3B:NEK11:RASA2:DVL3:MAP3K13:STK32B:AREG:AREGB:NPY5R:PTGER4:ISL1:CCNO:CKS1B:HDAC3:RELL2:PDGFRB:EDN1:FLOT

						1:STK19:CDKN1A:TNFAIP3:GTF2H5:FGFR1OP:HSPB1:YWHAG:CD36:PILRB:PRKAR2B:NCAPG2:BLK:GFRA2:RB1CC1:LYN:NRBP2:KLF4:PIP5KL1
GO_bp	GO_ANION_TRANSPORT	611	50	2.918444860318427e-07	2.9329692262010028e-05	MTOR:MFSD2A:LRRC8B:LRRC8C:ABCD3:SLC16A4:PSAP:SLC16A12:SLC43A1:SLC25A3:SLC25A15:PSEN1:SLC51B:ABCC11:SLC7A6:TRPV1:RPS6KB1:PITPNC1:TTYH2:GIPC1:CEACAM1:OSR1:NCOA1:SLC4A5:SLC11A1:PER2:SLC37A1:APOL1:PPARA:CPT1B:PLSCR4:SLC34A2:ATP10D:SLC10A4:SLC4A4:MTTP:PLA2G12A:NPY5R:SLC6A19:SLC6A18:SLC12A2:PRELID2:EDN1:CLDN4:CD36:ABCB1:CA2:SLC35D2:SLC25A25:SLC34A3
GO_bp	GO_REGULATION_OF_PROTEIN_LOCALIZATION	982	70	2.9529213978078123e-07	2.9329692262010028e-05	TARDBP:LDLRAP1:GJA5:GOLPH3L:SYT11:LMNA:UHMK1:SEC16B:GPAM:APBB1:KCNJ11:ABCC8:ARL2:CDCA5:UCP2:CRTAM:HSPA8:POSTN:SIAH3:LCP1:IPO5:SCFD1:PSEN1:NUMB:MAPKBP1:SLC51B:KIAA1199:MESDC2:DERL2:B9D1:STAC2:NPEPPS:AZI1:MBP:GSK3A:CARD8:XPO1:IL1RL1:VIL1:MFF:PER2:GTSE1:MYRIP:HYAL2:CACNA2D2:CCDC66:FOXP1:CD200R1:GSK3B:DZIP1L:DVL3:AP2M1:PYDC2:TERT:NIPBL:PTGER4:ISL1:HDAC3:NDFIP1:CNR1:TNFAIP3:YWHAG:CD36:BLK:PPP3CC:LYN:TERF1:ENY2:SCRIB:PAEP
GO_bp	GO_REGULATION_OF_CELLULAR_COMPONENT_BIOGENESIS	909	66	3.370640679710117e-07	3.303227866115915e-05	CLSTN1:MTOR:FBLIM1:CDC42:MACF1:TAL1:STIL:PRUNE:TMOD4:SYT11:ARHGEF2:SEMA4A:SELP:ARL2:LRFN4:HSPA8:NCKAP1L:RAP1B:ULK1:LCP1:ERCC5:STXBP6:SCFD1:LRFN5:FAM179B:CORO1A:SLX1A:SMPD3:DHX33:BRCA1:PLEKHM1:TUBB4A:DNAJB1:NPHS1:DCTN1:CREB1:VIL1:CHMP4B:SHANK3:HYAL1:ZMYND10:NPRL2:SHQ1:GSK3B:PYDC2:SLAIN2:PTGER4:DIMT1:OCLN:ANKRA2:UTP15:FCHSD1:EDN1:LRRC16A:FLOT1:ESR1:GTF2H5:THBS2:SEPT7:CD36:NSMAF:TERF1:CDH17:CHMP5:XPA:LRSAM1
GO_bp	GO_REGULATION_OF_CATION_TRANSMEMBRANE_TRANSPORT	328	33	3.484729251544491e-07	3.3700999998489484e-05	RNF207:CTSS:PSEN2:KCNJ11:ABCC8:UBASH3B:KCNA1:PTPN6:MYO5A:KIAA1199:GRIN2A:CORO1A:PIRT:CACNB1:STAC2:CACNG1:KCNJ2:OSR1:KCNS1:LIME1:MAPK8IP2:SHANK3:SCN5A:KCNAB1:FGF12:STIM2:DIAPH1:KCNIP1:KEL:LYN:GEM:UBQLN1:PTPN3
GO_bp	GO_ORGANONITROGEN_COMPOUND_CATABOLIC_PROCESS	1263	84	3.682392821809041e-07	3.515011329908631e-05	ACOT7:MTOR:CDC20:DDAH1:OVGP1:CTSS:CTSK:DCST1:UBQLN4:F13B:FMOD:CUL2:PSAP:SMPD1:UBE2L6:PGA3:ASRGL1:SYVN1:USP5:SPSB2:DAO:SDS:SDSL:FBXW8:SIAH3:UCHL3:ABHD13:DCAF11:PSME1:FBXO33:PSEN1:HERC2:OTUD7A:ADAMTS7:KIAA1199:ACAN:PKD1:GRIN2A:PDXDC1:NTAN1:SULT1A3:DCTPP1:TGFB1I1:LONP2:SIAH1:N4BP1:PLA2G15:SMPD3:DERL2:NPEPPS:TBX21:TRIM25:RNFT1:PSMD12:NPLOC4:UBXN6:PDE4A:KEAP1:GIPC1:PRODH2:GSK3A:CEACAM1:CBLC:C19orf68:XPO1:ANAPC1:CHMP4B:USP25:APOBEC3B:NAGA:CYP2D6:TYMP:HYAL3:HYAL1:HYAL2:GSK3B:ARMC8:AP2M1:NDFIP1:PRSS16:TNFAIP3:UBQLN1:PTPN3:LRSAM1
GO_bp	GO_CELL_CELL_ADHESION	819	61	4.0142978080907117e-07	3.7827037037777856e-05	CLSTN1:FBLIM1:CDC42:MPL:BCL10:ANXA9:TNFAIP8L2:PEAR1:SELP:CRB1:CSRP1:CDH23:C10orf54:GPAM:LRFN4:TENM4:UBASH3B:PTPN6:NCKAP1L:FNDC3A:PCDH9:TNFSF13B:STXBP6:LRFN5:PSEN1:PKD1:CORO1A:MAPK7:TBX21:MYL12A:MBP:IL12RB1:CEACAM1:PKP4:IGFBP2:MMP24:PPARA:CELSR1:PLXNB2:TGFBR2:CD200R1:ATP2C1:CDH9:CDH6:MEGF10:PCDHGA12:PCDHGC3:PCDHGC4:PCDHGC5:NDFIP1:ADAM19:FLOT1:MUC21:CLDN3:CLDN4:HSPB1:LYN:CDH17:SCRIB:KLF4:CEL
GO_bp	GO_CARBOHYDRATE_DERIVATIVE_METABOLIC_PROCESS	934	67	4.235296641516373e-07	3.9404342171070053e-05	ACOT7:A3GALT2:ELOVL1:ACOT11:EXTL2:AMPD1:HMGCS2:APCS:SOAT1:FMOD:B3GALNT2:CREM:PSAP:GPAM:SMPD1:GYLTL1B:SYVN1:DGAT2:HSPA8:TPI1:PTHLH:RNASEH2B:KDELC1:PSEN1:SLC51B:KIAA1199:ACAN:SULT1A3:SMPD3:SHPK:SHPK:DERL2:ASGR2:PRPSAP2:MPPE1:ST8SIA5:MUC16:TECR:CHST8:ATP6V1B1:RPE:PANK2:RPN2:UCKL1:ATP5L2:TYMP:ARSA:TRAK1:HYAL1:TMEM115:DHFRL1:A4GNT:GK5:MAN2B2:DCTD:PDGFRB:MGAT1:MUC21:B3GALT4:FUT9:ITGB8:TPST1:ESYT2:SLC35D2:RP11-203J24.9:ST6GALNAC6:ST6GALNAC4:DPM2:SLC25A25
GO_bp	GO_POSITIVE_REGULATION_OF_DEVELOPMENTAL_PROCESS	1393	90	4.894627773860492e-07	4.496939267234328e-05	CLSTN1:MTOR:SPEN:NBL1:CDC42:MACF1:TIE1:MPL:CDC20:TAL1:FOXE3:DDAH1:F3:HIPK1:ARNT:ARHGEF2:LMNA:SEMA4A:TMEM79:ABL2:ASPM:GDF2:C10orf54:ZFYVE27:GPAM:APBB1:TENM4:TRPC6:YAP1:CDON:SOX5:HOXC11:NCKAP1L:FBXW8:TNFSF13B:FOXG1:PSEN1:NUMB:TCF12:SH3GL3:RRN3:TGFB1I1:RNF112:RND2:BRCA1:TBX21:SPAG9:RPS6KB1:RNF157:TCF3:INSR:IL12RB1:MEGF8:CEACAM1:OSR1:NCOA1:CREB1:VIL1:MFF:C21orf91:PLXNB2:SHANK3:TGFBR2:TRAK1:HYAL1:CACNA2D2:DVL3:MAP3K13:IL8:TERT:NIPBL:ISL1:PDGFRB:EDN1:LRRC16A:FLOT1:CPNE5:CNR1:TNFAIP3:SOD2:THBS2:ITGB8:HOXA11:HSPB1:CD36:CREB3L2:STAR:LYN:CA2:KLF4
GO_bp	GO_CELLULAR_ION_HOMEOSTASIS	661	52	5.684395415600977e-07	5.1580625067490346e-05	ABL2:GDF2:CDH23:HPX:TRPC6:UBASH3B:PTPN6:SLC11A2:P2RX2:ERO1L:PSEN1:TRPM1:MYO5A:KIAA1199:PKD1:GRIN2A:CORO1A:CALB2:TRPV1:KCNJ2:PTGER1:GRIN2D:FPR2:ATP6V1B1:SLC4A5:SLC9A4:SLC11A1:TGM2:LIME1:MCHR1:SMDT1:SCO2:ACKR2:ATP2C1:ACKR4:SLC34A2:STIM2:SLC4A4:PTGER4:DIAPH1:NDFIP1:EDN1:HCRTR2:CNR1:ESR1:CD36:KEL:LYN:CA2:CALB1:S1PR3:SLC34A3

GO_bp	GO_RESPONSE_TO_PEPTIDE	503	43	6.166892329031328e-07	5.527641294924422e-05	MTOR:RPE65:PKLR:ARHGEF2:BGLAP:NUCKS1:ABCC8:UCP2:UCP3:RAP1B:PCK2:PSEN1:MYO5A:SMPD3:TRPV1:GLP2R:CACNB1:RPS6KB1:INSR:GSK3A:CEACAM1:FPR2:ATP6V1B1:CREB1:IGFBP5:EIF6:PPARA:GNAI2:GSK3B:AREG:AREGB:EDN1:TNFAIP3:CRHR2:YWHAG:CD36:PRKAR2B:STAR:LYN:CA2:CYP11B1:CYP11B2:CHMP5:KLF4
GO_bp	GO_MYELOID_LEUKOCYTE_ACTIVATION	646	51	6.559032695073352e-07	5.80830003720351e-05	NRAS:CTSS:HRNR:FCGR2A:HSPA6:RAB18:PSAP:LINC00610:COMMD9:HSPA8:PTPN6:METTL7A:NCKAP1L:RAP1B:APAF1:LRFN5:PSEN1:CHRNB4:SHPK:TRPV1:SHPK:VAT1:KPNB1:PSMD12:CD97:CEACAM1:CD33:FPR2:IL1RL1:IL18RAP:SLC11A1:SLPI:DNAJC5:SYNGR1:CYB5R3:ARSA:TGFBR2:HYAL2:FOXP1:ARMC8:IL8:CXCL6:CXCL1:PLAC8:DIAPH1:TUBB:RAB44:CNR1:RNASET2:LAT2:CD36:LYN:TUBB4B
GO_bp	GO_IMMUNE_EFFECTOR_PROCESS	1242	82	6.805208228630361e-07	5.906718255858169e-05	MTOR:CDC42:BCL10:VAV3:NRAS:CTSS:HRNR:SEMA4A:APCS:FCGR2A:HSPA6:FCGR3A:CFHR5:ZMYND11:RAB18:PSAP:GPAM:DMBT1:HPX:LINC00610:COMMD9:CRTAM:HSPA8:PTPN6:C1S:METTL7A:NCKAP1L:RAP1B:APAF1:LCP1:PSEN1:CHRNB4:CORO1A:VAT1:KPNB1:TBX21:TRIM25:PSMD12:NPLOC4:SERPINB4:CD97:PKN1:IL12RB1:POU2F2:CEACAM1:KDELR1:CD33:FPR2:PUM2:IL18R1:IL18RAP:SLC11A1:SLPI:DNAJC5:APOBEC3B:SYNGR1:CYB5R3:ARSA:HYAL2:TUSC2:FOXP1:PROS1:ARMC8:ABCF3:CXCL6:CXCL1:PLAC8:CFI:PTGER4:DIAPH1:NDFIP1:HLA-A:TUBB:C4A:RAB44:TNFAIP3:RNASET2:LAT2:CD36:LYN:CDH17:FCN2:TUBB4B
GO_bp	GO_NEURON_DIFFERENTIATION	1343	87	6.830898663237338e-07	5.906718255858169e-05	MTOR:NBL1:CDC42:MACF1:CDC20:SZT2:TAL1:RPE65:OLFM3:HIPK1:PHGDH:CERS2:SEMA6C:ARHGEF2:SEMA4A:UHMK1:ABL2:ASPM:CRB1:CDH23:ZFYVE27:APBB1:WEE1:DBX1:TENM4:TRPC6:BTG4:CDON:OPCML:C12orf57:SLC11A2:NCKAP1L:FBXW8:ULK1:POSTN:NRL:FOXG1:PSEN1:NUMB:TRPM1:TCF12:SKOR1:SH3GL3:RRN3:SIAH1:MAPK7:RNF112:RND2:SPAG9:RNF157:FSCN2:SKOR2:TCF3:S1PR5:MEGF8:NCOA1:ALK:BCL11A:CREB1:CTDSP1:C21orf91:PLXNB2:MAPK8IP2:SHANK3:TRAK1:CCDC66:C3orf17:GSK3B:DVL3:MAP3K13:AREG:AREGB:EIF4E:ISL1:FLOT1:CPNE5:CNR1:EVX1:YWHAG:NYAP1:CREB3L2:KEL:GFRA2:LYN:SCRIB:NRBP2:KLF4:PBX3
GO_bp	GO_RESPONSE_TO_WOUNDING	667	52	7.455512536188381e-07	6.303614848090225e-05	MTOR:CDC42:MACF1:MPL:F3:VAV3:CERS2:ADAM15:SYT11:PEAR1:APCS:F5:SELP:F13B:CSRP1:ABCC8:TRPC6:YAP1:UBASH3B:PTPN6:PHB2:P2RX2:POSTN:GRIN2A:DGKE:RPS6KB1:MYL12A:CEACAM1:MERTK:SLC11A1:VIL1:FBLN1:PPARA:CELSR1:TGFBR2:PROS1:GP9:PF4V1:ISL1:PDGFRB:EDN1:LRRC16A:CDKN1A:C6orf89:TNFAIP3:HSPB1:CD36:PRKAR2B:CREB3L2:LYN:SCRIB:KLF4
GO_bp	GO_NEGATIVE_REGULATION_OF_MULTICELLULAR_ORGANISMAL_PROCESS	1265	83	7.461421656923123e-07	6.303614848090225e-05	MTOR:TRIM62:TIE1:TAL1:FOXE3:ACOT11:RBM15:WDR77:GJA5:CTSK:CERS2:SEMA6C:SYT11:ARHGEF2:LMNA:SEMA4A:APCS:ASPM:GDF2:TACR2:C10orf54:TLL2:SFRP5:CTR9:ABCC8:UBE2L6:TRPC6:UBASH3B:PTPN6:PHB2:PTHLH:NCKAP1L:FOXG1:PSEN1:MAPKBP1:ADAMTS7:NFATC3:TRPV1:EPN2:HNF1B:NBR1:TBX21:TRIM25:NPLOC4:PTGER1:GSK3A:CEACAM1:CARD8:OSR1:BCL11A:IL1RL1:IL18R1:MERTK:IGFBP5:SLC11A1:CTDSP1:PPARA:TGFBR2:GNAI2:HYAL3:TUSC2:PROS1:CD200R1:C3orf17:GSK3B:PYDC2:AREG:AREGB:PLAC8:EIF4E:TERT:PTGER4:ISL1:C5orf30:C5orf20:TIFAB:NDFIP1:EDN1:TNFAIP3:THBS2:NCAPG2:LYN:KLF4:PAEP
GO_bp	GO_POSITIVE_REGULATION_OF_TRANSPORT	950	67	7.707881627126027e-07	6.437832949929124e-05	RNF207:TARDBP:LDLRAP1:TSPAN1:GOLPH3L:CTSS:SEC16B:TACR2:APBB1:KCNJ11:ABCC8:TRPC6:CRTAM:KCNA1:NCKAP1L:ORAI1:P2RX2:POSTN:IPO5:PSEN1:ARPP19:SLC51B:KIAA1199:SMPD3:TRPV1:PIRT:STAC2:NPEPPS:KCNJ2:AZI1:MBP:SH3GL1:INSR:GSK3A:CARD8:IL1RL1:MERTK:CREB1:SLC11A1:MFF:MCHR1:GTSE1:SHANK3:SCN5A:MYRIP:GNAI2:HYAL3:HYAL2:GSK3B:FGF12:STIM2:TERT:PTGER4:ISL1:HDAC3:PDGFRB:EDN1:FLOT1:C4A:CNR1:YWHAG:CD36:ABCB1:BLK:PPP3CC:CA2:PAEP
GO_bp	GO_LIPID_BIOSYNTHETIC_PROCESS	668	52	7.796808531614113e-07	6.438937382849858e-05	ACOT7:MTOR:A3GALT2:MFSD2A:ELOVL1:ACOT11:RPE65:ABCD3:HMGCS2:CERS2:FDPS:GPAM:SMPD1:TM7SF2:MOGAT2:DGAT2:FDX1:LPCAT3:PRKAB1:MTMR6:PCK2:FITM1:MYO5A:SMPD3:BRCA1:KPNB1:DGKE:MPPE1:ST8SIA5:TECR:CEACAM1:FPR2:CREB1:CDS2:EIF6:LSS:CYP2D6:CYB5R3:CHKB:CYP8B1:CNBP:COQ2:PPAP2A:EDN1:B3GALT4:PEX7:STAR:NSMAF:CYP11B1:CYP11B2:RP11-203J24.9:ST6GALNAC6:DPM2
GO_bp	GO_MONOCARBOXYLIC_ACID_METABOLIC_PROCESS	474	41	8.268930980213642e-07	6.752960300507808e-05	ACOT7:MTOR:ELOVL1:ACOT11:ABCD3:PHGDH:CREM:GPAM:PC:UCP3:DGAT2:AASDHPPT:SDS:PRKAB1:PCK2:PLA2G4B:MYO5A:LONP2:BRCA1:TECR:PRODH2:CEACAM1:PEX13:ATP6V1B1:PER2:PANK2:EIF6:CYP2D6:PPARA:CPT1B:CYP8B1:ACAD11:RBP1:EDN1:GSTA1:CNR1:PEX7:CD36:PRKAR2B:STAR:RDH10
GO_bp	GO_PROTEOLYSIS	1744	106	8.610342192803427e-07	6.954507155725845e-05	CDC20:BCL10:F3:LAMTOR5:CTSS:CTSK:FAM63A:DCST1:ADAM15:UBQLN4:PSEN2:CUL2:RBP3:C10orf54:STAMBPL1:FAS:TLL2:HABP2:UBE2L6:PGA3:ASRGL1:NAALADL1:SYVN1:RCE1:USP5:SPSB2:C1S:APAF1:FBXW8:SIAH3:UCHL3:DCAF11:PSME1:FBXO33:PSEN1:PAPLN:HERC2:OTUD7A:JMJD7:ADAMTS7:ACAN:PKD1:PRSS21:GRIN2A:NTAN1:TGFB1I1:LONP2:SIAH1:N4BP1:SPG7:DERL2:BRCA1:ADAM11:NPEPPS:TBX21:TRIM25:RNFT1:PSMD12:NPLOC4:SERPINB4:MBP:UBXN6:KEAP1:GIPC1:GSK3A:CBLC:

						C19orf68:CARD8:ANAPC1:VIL1:TM4SF20:COPS8:CHMP4B:MMP24:WFDC5:WFDC12:PI3:SEMG1:SEMG2:SLPI:USP25:TAB1:FBLN1:PROS1:IFT57:GSK3B:ARMC8:SENP2:MFI2:CFI:ADAM19:PRSS16:C4A:PI16:TNFAIP3:ESR1:CPA1:KEL:PIP:LYN:ENY2:ADAMTSL1:UBQLN1:KLF4:PTPN3:PAPPA
GO_bp	GO_PROTEIN_MODIFICATION_BY_SMALL_PROTEIN_CONJUGATION	896	64	8.863155086887918e-07	7.080890205285456e-05	MTOR:CDC42:TRIM62:CDC20:MED8:BCL10:TRIM33:ARNT:DCST1:RUSC1:CUL2:HECTD2:CTR9:UBE2L6:SYVN1:USP5:SPSB2:FBXW8:SIAH3:UCHL3:LMO7:DCAF11:PSME1:G2E3:FBXO33:FANCM:DCAF4:PSEN1:UBR7:HERC2:KBTBD13:SIAH1:N4BP1:RNF112:BRCA1:NPEPPS:TRIM25:RNFT1:PSMD12:RNF157:KEAP1:DCAF15:FBXO17:GSK3A:CBLC:CTU1:BCL11A:PEX13:ANAPC1:PER2:PCNA:RAD18:CAND2:UBA5:SENP2:KLHL2:UBE2QL1:MARCH3:NDFIP1:TSPAN17:TNFAIP3:UBQLN1:LRSAM1:UBAC1
GO_bp	GO_EXOCYTOSIS	897	64	9.203739926083222e-07	7.273923489968997e-05	TXLNA:NRAS:CTSS:HRNR:SYT11:TMEM79:FCGR2A:HSPA6:F5:SELP:RAB18:PSAP:LINC00610:COMMD9:SYT13:HSPA8:PTPN6:METTL7A:NCKAP1L:RAP1B:APAF1:STXBP6:SCFD1:PSEN1:MYO5A:CHRNB4:CORO1A:SEPT1:SEPT1:SMPD3:VAT1:KPNB1:PSMD12:CD97:GIPC1:SYCN:CEACAM1:CD33:FPR2:SLC11A1:SLPI:DNAJC5:SYNGR1:CYB5R3:ARSA:GNAI2:HYAL3:PROS1:STX19:GSK3B:ARMC8:CXCL1:PLAC8:DIAPH1:TUBB:RAB44:CNR1:RNASET2:LAT2:CD36:FAM3C:EXOC4:LYN:SCRIB:TUBB4B:TOR4A
GO_bp	GO_CALCIUM_ION_TRANSMEMBRANE_TRANSPORT	313	31	1.0931439856904407e-06	8.547455632792275e-05	PSEN2:TRPC6:UBASH3B:PTPN6:PHB2:ORAI1:ERO1L:PSEN1:TRPM1:MYO5A:KIAA1199:PKD1:GRIN2A:CORO1A:SPG7:TRPV1:CACNB1:STAC2:CACNG1:GRIN2D:LIME1:SMDT1:PKDREJ:CACNA2D2:ATP2C1:STIM2:DIAPH1:LYN:GEM:UBQLN1:SLC25A25
GO_bp	GO_POSITIVE_REGULATION_OF_BIOSYNTHETIC_PROCESS	1966	116	1.1063512322616066e-06	8.559664796971377e-05	TARDBP:MTOR:ZBTB17:TAL1:FOXD2:BCL10:DDAH1:RBM15:WDR77:ARNT:ASH1L:ARHGEF2:PMF1:C1orf85:ARHGEF11:NHLH1:UHMK1:SOAT1:NUCKS1:ZNF496:LARP4B:CREM:GDF2:ACTA2:APBB1:ZNF143:CTR9:EHF:DGAT2:YAP1:POU2AF1:CDON:SOX5:CSRNP2:TFCP2:HOXC13:HOXC11:LMO7:NRL:DLGAP5:SIX6:PSEN1:GTF2A1:ARPP19:TCF12:SLC51B:PKD1:RRN3:TGFB1I1:NFATC3:SMPD3:TRPV1:DHX33:RCVRN:MAPK7:HNF1B:IKZF3:BRCA1:TBX21:RPS6KB1:TAF4B:ASXL3:TCF3:SAFB:INSR:PKN1:GIPC1:NFKBIB:POU2F2:FPR2:MYCN:OSR1:NCOA1:CREB1:SLC11A1:NCL:PER2:PCNA:EIF6:WBP2NL:PPARA:CAND2:DAZL:HYAL2:NR1I2:CNBP:DVL3:PLAC8:TERT:NIPBL:ISL1:MCIDAS:DHX29:UTP15:HDAC3:PDGFRB:CDX1:EDN1:ESR1:HOXA10:HOXA11:EVX1:NEUROD6:RFC2:HSPB1:CD36:KLF14:CREB3L2:ASH2L:STAR:NSMAF:RDH10:ENY2:IPPK:KLF4:PBX3
GO_bp	GO_REGULATION_OF_BODY_FLUID_LEVELS	497	42	1.1194443562847047e-06	8.57074585280477e-05	CDC42:MPL:F3:VAV3:GJA5:HRNR:FLG:TMEM79:PEAR1:F5:SELP:F13B:CSRP1:TRPC6:UBASH3B:PTPN6:P2RX2:DGKE:MYL12A:CEACAM1:NCOA1:ATP6V1B1:SLC4A5:MERTK:CREB1:FBLN1:GNAI2:HYAL2:PROS1:GP9:PF4V1:C5orf20:TIFAB:EDN1:LRRC16A:CLDN4:HSPB1:CD36:PRKAR2B:LYN:CYP11B2:CEL
GO_bp	GO_WOUND_HEALING	550	45	1.1351246648761961e-06	8.601202357567054e-05	MTOR:CDC42:MACF1:MPL:F3:VAV3:ADAM15:SYT11:PEAR1:APCS:F5:SELP:F13B:CSRP1:ABCC8:TRPC6:YAP1:UBASH3B:PTPN6:P2RX2:POSTN:DGKE:MYL12A:CEACAM1:MERTK:SLC11A1:VIL1:FBLN1:PPARA:CELSR1:TGFBR2:PROS1:GP9:PF4V1:PDGFRB:EDN1:LRRC16A:CDKN1A:C6orf89:TNFAIP3:HSPB1:CD36:PRKAR2B:LYN:SCRIB
GO_bp	GO_MICROTUBULE_CYTOSKELETON_ORGANIZATION	533	44	1.174581072417132e-06	8.748767622955237e-05	CDC20:STIL:HOOK1:SSX2IP:PRUNE:MSTO1:ARHGEF2:LMNA:ASPM:TUBB8:WEE1:ARL2:SAC3D1:DNAJB13:FAM179B:GOLGA8R:PKD1:BRCA1:KPNB1:TUBD1:DNAI2:KIF19:AZI1:TUBB4A:XPO1:DCTN1:CHMP4B:RASSF1:ZMYND10:CCDC66:GSK3B:SLAIN2:MCIDAS:KIF2A:CETN3:HDAC3:TUBB:MAP7:FGFR1OP:LRRC6:CNTLN:CHMP5:NTMT1:TUBB4B
GO_bp	GO_REGULATION_OF_METAL_ION_TRANSPORT	379	35	1.1784054349286647e-06	8.748767622955237e-05	RNF207:PSEN2:ABCC8:TRPC6:UBASH3B:KCNA1:PTPN6:ORAI1:P2RX2:MYO5A:KIAA1199:CORO1A:RCVRN:CACNB1:STAC2:CACNG1:KCNJ2:OSR1:KCNS1:SEMG1:LIME1:MCHR1:SCN5A:GNAI2:KCNAB1:FGF12:STIM2:DIAPH1:PDGFRB:KCNIP1:KEL:LYN:GEM:UBQLN1:PTPN3
GO_bp	GO_INTERSPECIES_INTERACTION_BETWEEN_ORGANISMS	925	65	1.2720860257923295e-06	9.294701604925396e-05	RPL22:TARDBP:SPEN:CDC42:TRIM62:RBM15:LAMTOR5:SETDB1:FDPS:APCS:NUCKS1:ZMYND11:CUL2:DMBT1:HPX:IPO7:PC:HSPA8:ULK1:KPNA3:IPO5:POLR2C:ASGR2:RPL19:KPNB1:TBKBP1:TRIM25:RPL38:INSR:KEAP1:AP1M2:XPO1:CREB1:PCNA:CHMP4B:SEMG1:SLPI:UCKL1:APOL1:FBLN1:RPL32:RPL14:HYAL3:HYAL1:HYAL2:TUSC2:FAM208A:CD200R1:RAB43:AP2M1:IL8:CXCL6:EIF4E:CFI:RPL37:RPS18:CD36:PILRA:LYN:RPL7:SCRIB:CHMP5:RPL12:LRSAM1:NELFB
GO_bp	GO_TUBE_DEVELOPMENT	1082	73	1.2857517027919745e-06	9.294701604925396e-05	RNF207:TIE1:TAL1:STIL:BCL10:DDAH1:F3:VAV3:RBM15:HIPK1:PHGDH:GJA5:ADAM15:SEMA4A:GDF2:SFRP5:ABCC8:YAP1:CLMP:PHB2:LGR5:APAF1:PSEN1:PKD1:ESRP2:SMPD3:EPN2:MAPK7:HNF1B:BRCA1:GJC1:CIC:MEGF8:CXCL17:CEACAM1:OSR1:CREB1:IGFBP5:NCL:TGM2:TAB1:CELSR1:PLXNB2:TYMP:TGFBR2:HYAL1:FOXP1:ARL13B:IFT57:NPHP3:DVL3:IL8:AREG:AREGB:EIF4E:TERT:NIPBL:ISL1:PDGFRB:EDN1:VPS52:CDKN1A:TNFAIP3:HECA:ESR1:DACT2:THBS2:ITGB8:HOXA11:HSPB1:RDH10:CALB1:SCRIB:KLF4
GO_bp	GO_REGULATION_OF_DEFENSE_RESPONSE	754	56	1.2998281766990779e-06	9.294701604925396e-05	BCL10:NRAS:CTSS:CTSK:TNFAIP8L2:DCST1:ASH1L:SYT11:APCS:DMBT1:HPX:CRTAM:PTPN6:PSME1:COCH:LRFN5:MAPKBP1:SHPK:SHPK:CLEC10A:MAPK7:PSMD12:NPLOC4:SERPINB4:MUC16:IL12RB1:CXCL17:CEACAM1:PUM2:IL1RL1:IL18RAP:TGM2:Z

						BP1:TAB1:PPARA:HYAL2:FOXP1:CD200R1:PYDC2:CXCL6:NPY5R:DROSHA:PTGER4:ISL1:C5orf30:NDFIP1:FLOT1:MUC21:CNR1:TNFAIP3:ESR1:CD36:LYN:SCRIB:UBQLN1:KLF4:LRSAM1
GO_bp	GO_MULTI_ORGANISM_REPRODUCTIVE_PROCESS	984	68	1.3025228099419261e-06	9.294701604925396e-05	MTOR:PHC2:HOOK1:OVGP1:HORMAD1:SMCP:ASH1L:ASPM:TUBB8:CREM:ABCC8:UCP2:TRPC6:DDX25:AKAP3:PTHLH:LGR5:FNDC3A:TDRD9:HERC2:PLA2G4B:PRSS21:SIAH1:PRMT7:LYZL6:ZPBP2:SPATA32:RPS6KB1:SSTR2:AZI1:TAF4B:PAFAH1B3:NCOA1:PLB1:SPDYA:FSHR:MERTK:IGFBP2:IGFBP5:PANK2:PI3:SEMG1:SEMG2:OSBP2:APOL2:WBP2NL:FBLN1:PKDREJ:NCAPH2:SYCE3:MAPK8IP2:DAZL:TGFBR2:HYAL3:PPAP2A:SOX30:EDN1:MEI4:CNR1:ESR1:SYNE1:HOXA10:HOXA11:SEPT7:CLDN4:SPATA31A4:SPATA31A7:PAPPA:PAEP
GO_bp	GO_RIBOSOME_BIOGENESIS	285	29	1.3929000035541103e-06	9.844052909733375e-05	EXOSC10:EBNA1BP2:RPF1:EMG1:DDX47:DHX37:GTF3A:RPL3L:RRN3:METTL16:UTP18:RPL38:NOL10:TRMT61B:XPO1:ESF1:EIF6:DDX27:RPL14:SHQ1:KIAA1239:DROSHA:SKIV2L2:DIMT1:UTP15:WDR46:GTF2H5:RPL7:RPL12
GO_bp	GO_ORGANIC_ANION_TRANSPORT	485	41	1.4829512223752552e-06	0.00010380658556626787	MFSD2A:LRRC8C:ABCD3:SLC16A4:PSAP:SLC16A12:SLC43A1:SLC25A15:PSEN1:SLC51B:ABCC11:SLC7A6:TRPV1:RPS6KB1:PITPNC1:GIPC1:CEACAM1:OSR1:NCOA1:SLC4A5:SLC11A1:PER2:SLC37A1:PPARA:CPT1B:PLSCR4:ATP10D:SLC10A4:SLC4A4:MTTP:PLA2G12A:NPY5R:SLC6A19:SLC6A18:PRELID2:EDN1:CD36:ABCB1:CA2:SLC35D2:SLC25A25
GO_bp	GO_ORGANELLE_LOCALIZATION	685	52	1.6374378499702955e-06	0.00011353932261586482	CDC42:MSTO1:SYT11:ARHGEF2:F5:SEC16B:ASPM:PSEN2:CDCA5:RAP1B:SCFD1:DLGAP5:PSEN1:MYO5A:TRAPPC2L:B9D1:PLEKHM1:KPNB1:AZI1:TUBB4A:AP1M2:GIPC1:PEX13:XPO1:SLC4A5:DCTN1:TMEM230:CHMP4B:EIF6:DNAJC5:ARFGAP3:TRAK1:STX19:GSK3B:AREG:AREGB:TUBB:CNR1:FGFR1OP:LAT2:YWHAG:BET1:PRKAR2B:CEP41:COPG2:EXOC4:ESYT2:LYN:TERF1:GEM:SCRIB:CHMP5:TUBB4B
GO_bp	GO_REGULATION_OF_CYTOSOLIC_CALCIUM_ION_CONCENTRATION	352	33	1.6951282386886679e-06	0.00011644105190992252	ABL2:CDH23:TRPC6:UBASH3B:PTPN6:P2RX2:ERO1L:TRPM1:MYO5A:KIAA1199:PKD1:GRIN2A:CORO1A:CALB2:TRPV1:PTGER1:GRIN2D:FPR2:TGM2:LIME1:MCHR1:ACKR2:ACKR4:PTGER4:DIAPH1:EDN1:HCRTR2:CNR1:ESR1:CD36:LYN:CALB1:S1PR3
GO_bp	GO_CELLULAR_COMPONENT_MORPHOGENESIS	1111	74	1.7378390704463103e-06	0.00011728346677136708	FBLIM1:NBL1:CDC42:MACF1:MPL:SZT2:TAL1:SEMA6C:TMOD4:ARHGEF2:SEMA4A:ZFYVE27:APBB1:WEE1:TENM4:TRPC6:YAP1:C12orf57:PTPN6:SLC11A2:NCKAP1L:FBXW8:ULK1:POSTN:FOXG1:SCFD1:COCH:FRMD6:PSEN1:NUMB:CORO1A:SIAH1:MAPK7:ZPBP2:RND2:RNF157:ARHGEF18:MEGF8:ZNF135:BCL11A:MERTK:RND3:CREB1:VIL1:MFF:PANK2:FBLN1:PLXNB2:MAPK8IP2:SHANK3:TRAK1:ARL13B:GSK3B:DVL3:MAP3K13:MFI2:CDH9:CDH6:ISL1:DIAPH1:PDGFRB:EDN1:LRRC16A:FLOT1:CPNE5:DACT2:SEPT7:CLDN3:NYAP1:KEL:GFRA2:CDH17:SCRIB:PALM2
GO_bp	GO_NEGATIVE_REGULATION_OF_MOLECULAR_FUNCTION	1131	75	1.7393058337522465e-06	0.00011728346677136708	SPOCD1:LAMTOR5:ADAM15:APCS:CFHR5:SFRP5:DUSP8:SMPD1:IPO7:ARL2:UBASH3B:PTPN6:PHB2:GPRC5A:CSRNP2:NCKAP1L:COMMD6:IPO5:PPP1R36:ZFYVE1:PSEN1:PAPLN:ARPP19:PKD1:CD2BP2:PRPSAP2:MAPK7:SERPINB4:KEAP1:PKN1:GSK3A:CEACAM1:CBLC:CARD8:TSKS:OSR1:RTKN:VIL1:PER2:WFDC5:WFDC12:PI3:SLPI:CYP2D6:PPARA:GNAI2:HYAL2:NPRL2:PROS1:GSK3B:KCNAB1:PYDC2:FGF12:ISL1:NDFIP1:SNCB:C4A:CDKN1A:PI16:CNR1:TNFAIP3:ESR1:FGFR1OP:HSPB1:YWHAG:MEPCE:PPP1R35:PRKAR2B:LYN:TERF1:GEM:LY6E:SCRIB:UBQLN1:KLF4
GO_bp	GO_NCRNA_METABOLIC_PROCESS	455	39	1.8964223269763694e-06	0.0001267154918479665	EXOSC10:EBNA1BP2:KTI12:RPF1:NSUN6:ARL5B-AS1:TRUB1:EMG1:DDX47:DHX37:DTD2:TDRD9:MTFMT:TARSL2:METTL16:UTP18:SARS2:CTU1:NOL10:TRMT61B:TPRKB:ESF1:DDX27:RTCB:TRMU:THUMPD3:RPL14:SHQ1:TRMT10A:DROSHA:SKIV2L2:DIMT1:UTP15:THG1L:RPP40:WDR46:GTF2H5:MEPCE:DUS4L:RPL7
GO_bp	GO_CELL_MOTILITY	1691	102	1.952226086644703e-06	0.00012926902465620332	MTOR:NBL1:HTR6:MACF1:WDR65:TIE1:TSPAN1:SSX2IP:F3:VAV3:CERS2:SEMA6C:SMCP:ADAM15:ASH1L:ARHGEF2:LMNA:SEMA4A:SELP:ABL2:ASPM:GDF2:TACR2:C10orf54:ABCC8:PTPN6:NCKAP1L:POSTN:LCP1:FOXG1:PSEN1:NUMB:KIAA1199:PDXDC1:CORO1A:CCL17:SLC7A6:SMPD3:RND2:TBX21:SPAG9:RPS6KB1:FSCN2:INSR:PKN1:GIPC1:MEGF8:CXCL17:CEACAM1:FPR2:PEX13:MERTK:RND3:IGFBP5:VIL1:SEMG1:SEMG2:FBLN1:GTSE1:CELSR1:PLXNB2:TGFBR2:ACKR2:GNAI2:HYAL1:HYAL2:FOXP1:PROS1:ARL13B:CD200R1:ACKR4:IL8:CXCL6:PF4V1:CXCL1:TERT:NIPBL:PTGER4:ISL1:PPAP2A:KIF2A:MCTP1:C5orf30:MEGF10:SLC12A2:DIAPH1:PDGFRB:EDN1:LRRC16A:PTP4A1:PEX7:TNFAIP3:SOD2:FGFR1OP:ITGB8:SEPT7:HSPB1:LYN:LRRC6:SCRIB:KLF4:PIP5KL1
GO_bp	GO_PROTEIN_MODIFICATION_BY_SMALL_PROTEIN_CONJUGATION_OR_REMOVAL	1097	73	2.109273325386352e-06	0.00013750021933845848	MTOR:CDC42:TRIM62:CDC20:MED8:BCL10:TRIM33:ARNT:FAM63A:DCST1:RUSC1:CUL2:STAMBPL1:HECTD2:CTR9:UBE2L6:SYVN1:RCE1:USP5:SPSB2:FBXW8:SIAH3:UCHL3:LMO7:DCAF11:PSME1:G2E3:FBXO33:FANCM:DCAF4:PSEN1:UBR7:HERC2:OTUD7A:KBTBD13:SIAH1:N4BP1:RNF112:BRCA1:NPEPPS:TRIM25:RNFT1:PSMD12:RNF157:KEAP1:DCAF15:FBXO17:GSK3A:CBL

						C:CTU1:BCL11A:PEX13:ANAPC1:COPS8:PER2:PCNA:USP25:TAB1:RAD18:CAND2:UBA5:SENP2:KLHL2:UBE2QL1:MARCH3:NDFIP1:TSPAN17:TNFAIP3:ESR1:ENY2:UBQLN1:LRSAM1:UBAC1
GO_bp	GO_DEVELOPMENTAL_GROWTH	654	50	2.1139489503735793e-06	0.00013750021933845848	MTOR:MACF1:STIL:SEMA6C:ADAM15:SEMA4A:ASPM:PSAP:TLL2:ZFYVE27:GPAM:CTR9:TENM4:YAP1:ULK1:POSTN:COCH:SMPD3:ANKRD11:HNF1B:RND2:RPS6KB1:RNF157:INSR:GSK3A:MEGF8:BCL11A:CREB1:VIL1:PPARA:SHANK3:TGFBR2:CACNA2D2:GSK3B:MAP3K13:PSAPL1:AREG:AREGB:PLAC8:NPY1R:NIPBL:PDGFRB:EDN1:CDKN1A:CPNE5:ESR1:HOXA11:NCAPG2:RDH10:XPA:SLC25A25
GO_bp	GO_EMBRYONIC_ORGAN_DEVELOPMENT	423	37	2.1518057314379622e-06	0.00013873484321113178	RNF207:TAL1:STIL:HIPK1:GJA5:ARNT:YAP1:HOXC11:FBXW8:POLE:FOXG1:PSEN1:PKD1:RNF112:HNF1B:RPL38:MEGF8:TPO:OSR1:NCOA1:ATP6V1B1:CELSR1:TGFBR2:HYAL1:LRIG1:ARL13B:IFT57:NPHP3:IL8:NIPBL:C5orf20:TIFAB:EDN1:VPS52:HOXA11:RDH10:SCRIB
GO_bp	GO_ORGANIC_ACID_METABOLIC_PROCESS	901	63	2.1739385533799977e-06	0.00013894302928124332	ACOT7:MTOR:RIMKLA:ELOVL1:ACOT11:DDAH1:ABCD3:PHGDH:CREM:ALDH18A1:GPAM:ASRGL1:PC:UCP3:DGAT2:AASDHPPT:DAO:SDS:SDSL:PRKAB1:PCK2:PSME1:ERO1L:PLA2G4B:MYO5A:TARSL2:PDXDC1:LONP2:PLA2G15:SLC7A6:BRCA1:PSMD12:TECR:PRODH2:SARS2:LIPE:CEACAM1:PEX13:ATP6V1B1:MTHFD2:PER2:PANK2:EIF6:CYP2D6:CYB5R3:PPARA:CPT1B:CYP8B1:DHFRL1:ACAD11:RBP1:EDN1:LRRC16A:GSTA1:CNR1:PEX7:AIG1:ASL:CD36:PRKAR2B:STAR:RDH10:CEL
GO_bp	GO_SENSORY_PERCEPTION	960	66	2.2137886408114344e-06	0.00014027022853417277	MTOR:RPE65:OR14K1:RBP3:CDH23:PSAP:RGR:SFRP5:OR52B4:OR51T1:OR4C15:OR4C16:OR4P4:OR4S2:OR4C6:OR5T1:OR8H1:OR8K3:OR8K1:OR8J1:OR8U1:OR5R1:KCNA1:OR10P1:P2RX2:NRL:COCH:SIX6:TRPM1:MYO5A:KIAA1199:GRIN2A:TRPV1:RCVRN:GJC1:RPL38:FSCN2:MBP:OR1M1:OR7G2:OR7G1:OR7G3:PDE4A:OR10H2:OR10H3:GRIN2D:ATP6V1B1:MMP24:CCDC66:LRIG1:NPY1R:NIPBL:DIAPH1:GRXCR2:EDN1:OR10C1:CNR1:CD36:PIP:TAS2R39:TAS2R40:RDH10:OR1L3:OR1L4:OR1L6:OBP2A
GO_bp	GO_CELLULAR_RESPONSE_TO_LIPID	601	47	2.304980380908536e-06	0.0001448000495698952	C1orf64:BCL10:ADAM15:BGLAP:ABL2:GPAM:CTR9:SCGB2A1:DGAT2:YAP1:FDX1:LINC01059:GRAMD1B:PHB2:PCK2:TGFB1I1:SHPK:SHPK:BRCA1:RPS6KB1:SSTR2:SAFB2:SAFB:NFKBIB:CARD8:OSR1:NCOA1:CREB1:PPARA:FOXP1:NR1I2:UBA5:PLSCR4:IL8:CXCL6:PF4V1:CXCL1:EIF4E:PTGER4:ISL1:PPAP2A:EDN1:TNFAIP3:ESR1:CD36:STAR:LYN:CHMP5:KLF4
GO_bp	GO_TRANSLATIONAL_INITIATION	187	22	2.431463159709364e-06	0.00015145130698189684	RPL22:MTOR:UHMK1:MTIF3:MTFMT:DHX33:RPL19:RPS6KB1:RPL38:EIF6:RPL32:DAZL:EIF1B:RPL14:EIF4E:RPL37:DHX29:EIF4E1B:RPS18:HSPB1:RPL7:RPL12
GO_bp	GO_EMBRYO_DEVELOPMENT_ENDING_IN_BIRTH_OR_EGG_HATCHING	621	48	2.491064722762624e-06	0.0001537554207421035	HES2:TIE1:STIL:BCL10:PHGDH:HORMAD1:ARNT:CTR9:AKAP3:EMG1:HOXC11:APAF1:FBXW8:RNASEH2B:PSEN1:PKD1:RRN3:ANKRD11:B9D1:HNF1B:BRCA1:MBTD1:KEAP1:MEGF8:OSR1:NCOA1:HES6:RTCB:TAB1:CELSR1:PLXNB2:TGFBR2:HYAL1:ARL13B:IFT57:NPHP3:DVL3:SLC34A2:NIPBL:ISL1:CDX1:MGAT1:EDN1:HOXA11:EVX1:NCAPG2:RDH10:SCRIB
GO_bp	GO_DEVELOPMENTAL_PROCESS_INVOLVED_IN_REPRODUCTION	658	50	2.510292583544547e-06	0.0001537554207421035	MTOR:HOOK1:RBM15:WDR77:CSDE1:HORMAD1:ARNT:ADAM15:ASH1L:ASPM:TUBB8:PSAP:DDX25:PHB2:LGR5:FBXW8:FNDC3A:PKD1:ZPBP2:RPS6KB1:TAF4B:SAFB2:INSR:OSR1:NCOA1:FSHR:MERTK:PANK2:SEMG1:SEMG2:OSBP2:RTCB:DAZL:HYAL3:PSAPL1:AREG:AREGB:NIPBL:C5orf20:TIFAB:PDGFRB:ADAM19:MEI4:ESR1:ITGB8:HOXA10:HOXA11:STAR:RDH10:LRRC6:PAEP
GO_bp	GO_NEGATIVE_REGULATION_OF_SIGNALING	1369	86	2.6992982137917182e-06	0.0001639656353005713	MTOR:NBL1:CDC42:SZT2:TXNDC12:TRIM33:DCST1:ASH1L:SYT11:ARHGEF2:LMNA:PEAR1:ABL2:PHLDA3:ZMYND11:TACR2:FAS:SFRP5:DUSP8:SMPD1:KCNJ11:ABCC8:SYVN1:UCP2:YAP1:UBASH3B:PTPN6:PHB2:GPRC5A:RAP1B:PSME1:PSEN1:OTUD7A:MAPKBP1:SKOR1:RRN3:TGFB1I1:SIAH1:EPN2:MAPK7:BRCA1:RPS6KB1:PSMD12:NPLOC4:SKOR2:PHLPP1:EPS15L1:GSK3A:MEGF8:CEACAM1:CBLC:CARD8:IGFBP2:IGFBP5:FBLN1:MAPK8IP2:SHANK3:TGFBR2:GNAI2:HYAL2:NPRL2:FOXP1:GSK3B:NPHP3:RASA2:DVL3:PYDC2:IL8:AREG:AREGB:NPY5R:TERT:ISL1:HDAC3:EDN1:TNFAIP3:ESR1:SOD2:DACT2:HSPB1:RB1CC1:LYN:ENY2:SCRIB:UBQLN1:KLF4:PTPN3
GO_bp	GO_NCRNA_PROCESSING	377	34	2.8160709205413303e-06	0.00016827740866649413	EXOSC10:EBNA1BP2:KTI12:RPF1:NSUN6:ARL5B-AS1:TRUB1:EMG1:DDX47:DHX37:MTFMT:METTL16:UTP18:CTU1:NOL10:TRMT61B:TPRKB:ESF1:DDX27:RTCB:TRMU:THUMPD3:RPL14:SHQ1:TRMT10A:DROSHA:SKIV2L2:DIMT1:UTP15:THG1L:RPP40:WDR46:GTF2H5:DUS4L:RPL7
GO_bp	GO_CELLULAR_RESPONSE_TO_PEPTIDE	377	34	2.8160709205413303e-06	0.00016827740866649413	RPE65:PKLR:ARHGEF2:BGLAP:NUCKS1:UCP2:RAP1B:PCK2:PSEN1:MYO5A:SMPD3:GLP2R:CACNB1:RPS6KB1:INSR:GSK3A:CEACAM1:FPR2:ATP6V1B1:CREB1:GNAI2:GSK3B:EDN1:CRHR2:YWHAG:CD36:PRKAR2B:STAR:LYN:CA2:CYP11B1:CYP11B2:CHMP5:KLF4
GO_bp	GO_SKELETAL_SYSTEM_DEVELOPMENT	517	42	3.0554490799563445e-06	0.00018110928014257365	TAL1:GJA5:CTSK:ASH1L:BGLAP:GDF2:PTPN6:SOX5:PTHLH:HOXC11:COCH:PSEN1:ADAMTS7:ACAN:HAPLN3:PKD1:SMPD3:ANKRD11:PRPSAP2:MBTD1:RPL38:MEGF8:OSR1:TGFBR2:HYAL3:HYAL1:HYAL2:FOXP1:NIPBL:PTGER4:C5orf20:TIFAB:LECT2:CDX1:EDN1:PEX7:ITGB8:HOXA10:HOXA11:HOXA13:CREB3L2:RDH10

GO_bp	GO_GOLGI_VESICLE_TRANSPORT	363	33	3.3039919787562877e-06	0.0001942747283508697	MACF1:GOLPH3L:SCAMP3:BGLAP:F5:SEC16B:VPS51:STXBP6:SCFD1:MIA2:CTAGE5:MYO5A:TRAPPC2L:RABEP1:NAPG:MPPE1:KDELR1:DCTN1:ARFGAP3:TMEM115:PROS1:RAB43:RAB6B:COPB2:AREG:AREGB:KIF2A:VPS52:TAPBP:BET1:BCAP29:COPG2:EXOC4:CREB3L2:NRBP2
GO_bp	GO_CENTRAL_NERVOUS_SYSTEM_DEVELOPMENT	972	66	3.3457832699088774e-06	0.0001951706907446845	MTOR:HTR6:CDC42:TMEM57:MFSD2A:SZT2:TAL1:STIL:LPHN2:PTBP2:PHGDH:NHLH1:ASPM:RAB18:ZNF488:DBX1:TENM4:CDON:KCNA1:ATN1:C12orf57:APAF1:ANKLE2:FOXG1:PSEN1:NUMB:SH3GL3:ACAN:HAPLN3:PKD1:GRIN2A:HNF1B:SSTR2:MBP:SH3GL1:DNAJB1:CHST8:CIC:PAFAH1B3:GRIN2D:FPR2:NCOA1:ALK:PEX13:DCTN1:POTEE:CREB1:C21orf91:CELSR1:PLXNB2:SHANK3:HDAC11:TGFBR2:SCN5A:ARL13B:GSK3B:KCNAB1:NIPBL:ISL1:EVX1:NEUROD6:PPP3CC:STAR:LYN:SCRIB:PBX3
GO_bp	GO_RESPONSE_TO_HORMONE	973	66	3.461241186778585e-06	0.00020031592695135905	MTOR:C1orf64:RPE65:CTSS:PKLR:BGLAP:NUCKS1:GPAM:KCNJ11:ABCC8:SCGB2A1:UCP2:UCP3:YAP1:PHB2:RAP1B:LGR5:POSTN:PCK2:MYO5A:TGFB1I1:TRPV1:GLP2R:BRCA1:RPS6KB1:SSTR2:SAFB2:SAFB:INSR:GSK3A:CEACAM1:NCOA1:FSHR:ATP6V1B1:CREB1:IGFBP2:IGFBP5:PCNA:EIF6:PPARA:TGFBR2:GNAI2:FOXP1:NR1I2:UBA5:XRN1:AREG:AREGB:EIF4E:PTGER4:ISL1:PPAP2A:PDGFRB:SOX30:EDN1:CDKN1A:ESR1:CRHR2:CLDN4:YWHAG:PRKAR2B:STAR:LYN:CA2:CYP11B1:CYP11B2:PAPPA
GO_bp	GO_REGULATION_OF_CELLULAR_COMPONENT_MOVEMENT	1053	70	3.531743025381188e-06	0.00020197692322486343	RNF207:MTOR:NBL1:MACF1:TIE1:SSX2IP:F3:GJA5:CERS2:SEMA6C:ADAM15:ARHGEF2:LMNA:SEMA4A:SELP:ABL2:GDF2:TACR2:C10orf54:ABCC8:NCKAP1L:POSTN:NUMB:KIAA1199:CORO1A:SMPD3:RND2:SPAG9:RPS6KB1:KCNJ2:INSR:PKN1:MEGF8:CXCL17:CEACAM1:FPR2:RND3:IGFBP5:VIL1:SEMG1:SEMG2:FBLN1:GTSE1:PLXNB2:TGFBR2:SCN5A:GNAI2:HYAL1:HYAL2:FOXP1:CD200R1:IL8:TERT:NIPBL:PTGER4:KIF2A:MCTP1:C5orf30:DIAPH1:PDGFRB:EDN1:LRRC16A:PTP4A1:SOD2:FGFR1OP:SEPT7:HSPB1:LYN:KLF4:PIP5KL1
GO_bp	GO_POSITIVE_REGULATION_OF_CELL_DEVELOPMENT	538	43	3.544901101497603e-06	0.00020197692322486343	MTOR:SPEN:NBL1:CDC42:MACF1:MPL:ARHGEF2:ABL2:ASPM:ZFYVE27:APBB1:TENM4:TRPC6:CDON:FBXW8:FOXG1:PSEN1:NUMB:TCF12:SH3GL3:RRN3:RNF112:RND2:SPAG9:RNF157:TCF3:MEGF8:NCOA1:C21orf91:PLXNB2:SHANK3:TRAK1:DVL3:MAP3K13:NIPBL:EDN1:LRRC16A:CPNE5:CNR1:HOXA11:CREB3L2:STAR:LYN
GO_bp	GO_REGULATION_OF_INTRACELLULAR_SIGNAL_TRANSDUCTION	1824	107	3.8022670003053966e-06	0.00021208741851291556	MTOR:HTR6:CDC42:TRIM62:SZT2:TXNDC12:SSX2IP:BCL10:F3:VAV3:LAMTOR5:HIPK1:ASH1L:ARHGEF2:ARHGEF11:SELP:ABL2:PHLDA3:ZMYND11:GDF2:PSAP:FAS:SFRP5:DUSP8:SMPD1:SYVN1:CDON:PTPN6:PHB2:RAP1B:NUAK1:PRKAB1:P2RX2:TNFRSF19:MAP3K9:PSEN1:OTUD7A:MAPKBP1:MYO5A:RRN3:SIAH1:CCL17:DHX33:MAPK7:BRCA1:NBR1:SPAG9:TRIM25:NPLOC4:RALBP1:TAF4B:PHLPP1:INSR:ARHGEF18:PDE4A:CC2D1A:PKN1:GSK3A:CXCL17:CEACAM1:CBLC:CARD8:FPR2:PUM2:ALK:FSHR:IL18R1:IGFBP5:COPS8:RALGAPA2:TGM2:LIME1:TAB1:FBLN1:MAPK8IP2:GNAI2:HYAL2:NPRL2:GSK3B:ATP2C1:RASA2:DVL3:MAP3K13:PYDC2:AREG:AREGB:NPY5R:HDAC3:RELL2:ARAP3:NDFIP1:PDGFRB:EDN1:FLOT1:IER3:RGL2:TNFAIP3:PLEKHG1:ESR1:SOD2:RFC2:HSPB1:CD36:RB1CC1:LYN:UBQLN1:KLF4:NACC2
GO_bp	GO_ORGANIC_HYDROXY_COMPOUND_METABOLIC_PROCESS	486	40	3.8052075211989203e-06	0.00021208741851291556	LDLRAP1:RPE65:HMGCS2:FDPS:SOAT1:GPAM:SMPD1:TM7SF2:MOGAT2:DGAT2:FDX1:PDE1B:DAO:EBPL:PCK2:MYO5A:GRIN2A:KPNB1:IMPA2:GIPC1:LIPE:TPO:PLB1:PANK2:LSS:APOL2:APOL1:CYB5R3:MIOX:CYP8B1:CNBP:GK5:COQ2:SNCB:STAR:RDH10:CYP11B1:CYP11B2:IPPK:DPM2
GO_bp	GO_CELLULAR_RESPONSE_TO_UV_B	8	5	3.8089169039054224e-06	0.00021208741851291556	MFAP4:HYAL3:HYAL1:HYAL2:CDKN1A
GO_bp	GO_POSITIVE_REGULATION_OF_MOLECULAR_FUNCTION	1740	103	3.9437146862883785e-06	0.00021794212740014723	RNF207:MTOR:TRIM62:CDC20:TAL1:DNAJB4:BCL10:F3:VAV3:CTSS:CKS1B:ARHGEF2:ARHGEF11:ABL2:PSEN2:AGAP4:AGAP8:C10orf54:PSAP:FAS:KCNJ11:ABCC8:CDCA5:TRPC6:ERC1:KCNA1:PHB2:GPRC5A:NCKAP1L:APAF1:ORAI1:PSME1:MAP3K9:PSEN1:KIAA1199:PKD1:GRIN2A:TBC1D10B:CCL17:RABEP1:DHX33:RCVRN:PIRT:STAC2:SPAG9:TRIM25:RALBP1:MBP:TCF3:INSR:PKN1:DNAJB1:GSK3A:CXCL17:CARD8:FPR2:SPDYA:ALK:IL18R1:IL18RAP:PKP4:SLC11A1:COPS8:PCNA:RALGAPA2:MMP24:SEMG1:SEMG2:TAB1:ARFGAP3:FBLN1:SHANK3:TGFBR2:GNAI2:NPRL2:IFT57:ADPRH:GSK3B:RASA2:DVL3:MAP3K13:STIM2:CXCL1:TERT:NIPBL:ISL1:CKS1B:ARAP3:PDGFRB:WRNIP1:EDN1:FLOT1:CDKN1A:C6orf89:ESR1:RFC2:CD36:ABCB1:PILRB:PRKAR2B:NCAPG2:LYN:NSMAF:SCRIB:KLF4
GO_bp	GO_CELLULAR_RESPONSE_TO_NITROGEN_COMPOUND	632	48	4.015595407527671e-06	0.00021953531277557194	MTOR:RPE65:LAMTOR5:HCN3:PKLR:ARHGEF2:BGLAP:NUCKS1:UCP2:FDX1:RAP1B:IPO5:PCK2:PSEN1:MYO5A:SULT1A3:SMPD3:TRPV1:GLP2R:CACNB1:BRCA1:RPS6KB1:INSR:GSK3A:CEACAM1:FPR2:BCL11A:ATP6V1B1:CREB1:IGFBP5:GNAI2:GSK3B:XRN1:DIAPH1:CPEB4:EDN1:FLOT1:CRHR2:YWHAG:CD36:PRKAR2B:STAR:LYN:CA2:CYP11B1:CYP11B2:CHMP5:KLF4
GO_bp	GO_EMBRYONIC_MORPHOGENESIS	577	45	4.032281255061526e-06	0.00021953531277557194	RNF207:ZBTB17:TAL1:STIL:BCL10:HIPK1:GJA5:CTR9:TENM4:YAP1:CDON:HOXC11:APAF1:FOXG1:PSEN1:TGFB1I1:B9D1:MAPK7:HNF1B:RPL38:MBP:MEGF8:OSR1:NCOA1:ATP6V1B1:AFF3:CELSR1:PLXNB2:TGFBR2:HYAL1:LRIG1:ARL13B:IFT57:NPHP3:DVL3:NIPBL:C5orf20:TIFAB:EDN1:HOXA10:HOXA11:EXOC4:RDH10:SCRIB:KLF4

GO_bp	GO_CELL_PROJECTION_ORGANIZATION	1509	92	4.107210568247466e-06	0.00022197057115160937	MTOR:NBL1:CDC42:CCDC28B:MACF1:CDC20:SZT2:SSX2IP:VAV3:PHGDH:CERS2:SEMA6C:SEMA4A:UHMK1:ABL2:CDH23:ZFYVE27:APBB1:WEE1:DNAJB13:TRPC6:C12orf57:EMP1:SLC11A2:NCKAP1L:FBXW8:ULK1:POSTN:FOXG1:FAM179B:PSEN1:NUMB:RRN3:CORO1A:SIAH1:DNAH9:B9D1:MAPK7:RND2:PLEKHM1:TUBD1:DNAI2:KIF19:RNF157:AZI1:TUBB4A:INSR:MEGF8:BCL11A:DCTN1:CREB1:VIL1:C21orf91:PLXNB2:MAPK8IP2:SHANK3:TRAK1:ZMYND10:CCDC66:ARL13B:IFT57:GSK3B:NPHP3:DZIP1L:DVL3:MAP3K13:GMNC:AREG:AREGB:ISL1:MCIDAS:CCNO:C5orf30:LRRC16A:TUBB:FLOT1:CPNE5:CNR1:FGFR1OP:SEPT7:YWHAG:NYAP1:PRKAR2B:GPR22:CEP41:CREB3L2:KEL:GFRA2:LYN:LRRC6:SCRIB:KLF4:TUBB4B
GO_bp	GO_DIVALENT_INORGANIC_CATION_HOMEOSTASIS	506	41	4.2482494223890475e-06	0.00022791703105517883	ABL2:CDH23:TRPC6:UBASH3B:KCNA1:PTPN6:P2RX2:ERO1L:PSEN1:TRPM1:MYO5A:KIAA1199:PKD1:GRIN2A:CORO1A:CALB2:TRPV1:PTGER1:GRIN2D:FPR2:ATP6V1B1:SLC11A1:TGM2:LIME1:MCHR1:SMDT1:ACKR2:ATP2C1:ACKR4:STIM2:PTGER4:DIAPH1:EDN1:HCRTR2:CNR1:ESR1:CD36:KEL:LYN:CALB1:S1PR3
GO_bp	GO_G_PROTEIN_COUPLED_RECEPTOR_SIGNALING_PATHWAY_COUPLED_TO_CYCLIC_NUCLEOTIDE_SECOND_MESSENGER	254	26	4.301352676321619e-06	0.0002290937838475645	HTR6:LPHN2:PSAP:GRK5:OR5T1:PTHLH:LGR5:GLP2R:SSTR2:CD97:PTGER1:OR10H2:OR10H3:GSK3A:FPR2:FSHR:MC3R:MCHR1:GNAI2:PSAPL1:NPY1R:PTGER4:EDN1:CNR1:CRHR2:S1PR3
GO_bp	GO_RESPONSE_TO_CYTOKINE	1182	76	4.5468414007454064e-06	0.00024042650572286863	CDC42:LDLRAP1:TXLNA:TRIM62:MPL:F3:RBM15:HIPK1:DCST1:ARHGEF2:HPX:CTR9:KCNJ11:HSPA8:PTPN6:PDE1B:RAP1B:TNFRSF19:POSTN:LCP1:TNFSF13B:PSME1:ADAMTS7:CORO1A:BOLA2B:CCL17:SMPD3:SHPK:TRPV1:SHPK:MAPK7:IFI35:BRCA1:TRIM25:RPS6KB1:PSMD12:KEAP1:GIPC1:IL12RB1:GSK3A:CEACAM1:CARD8:AFF3:IL1RL1:IL18R1:IL18RAP:CREB1:SLC11A1:NCL:ZBP1:SYNGR1:TAB1:ACKR2:HYAL3:HYAL1:HYAL2:GSK3B:RAB43:ACKR4:MRAS:PYDC2:IL8:CXCL6:PF4V1:CXCL1:OCLN:SLC12A2:EDN1:HLA-A:CDKN1A:GSTA2:TNFAIP3:SOD2:CD36:STAR:SCRIB:KLF4
GO_bp	GO_RESPONSE_TO_STEROID_HORMONE	386	34	4.710759315142387e-06	0.0002459313082356026	C1orf64:BGLAP:GPAM:SCGB2A1:UCP3:YAP1:PHB2:PCK2:TGFB1I1:BRCA1:RPS6KB1:SSTR2:SAFB2:SAFB:NCOA1:IGFBP2:PCNA:PPARA:TGFBR2:FOXP1:NR1I2:UBA5:AREG:AREGB:EIF4E:ISL1:PPAP2A:SOX30:EDN1:CDKN1A:ESR1:CLDN4:STAR:CA2:PAPPA
GO_bp	GO_CELL_CELL_ADHESION_VIA_PLASMA_MEMBRANE_ADHESION_MOLECULES	271	27	4.717865913091152e-06	0.0002459313082356026	CLSTN1:SELP:CRB1:CDH23:LRFN4:TENM4:PCDH9:LRFN5:PKD1:MAPK7:MBP:CEACAM1:MMP24:CELSR1:PLXNB2:TGFBR2:ATP2C1:CDH9:CDH6:PCDHGA12:PCDHGC3:PCDHGC4:PCDHGC5:CLDN3:CLDN4:CDH17:KLF4
GO_bp	GO_CYTOKINE_PRODUCTION	750	54	4.961938469711913e-06	0.0002568327306505814	BCL10:ARNT:ASH1L:SYT11:ARHGEF2:GDF2:C10orf54:GPAM:ABCC8:UBE2L6:CRTAM:PTPN6:NCKAP1L:POSTN:MAPKBP1:NFATC3:DHX33:BRCA1:TBX21:TRIM25:NPLOC4:MBP:IL12RB1:CXCL17:CEACAM1:CARD8:KDELR1:PUM2:IL1RL1:IL18R1:IL18RAP:MERTK:CREB1:SLC11A1:ZBP1:HYAL2:TUSC2:FOXP1:CD200R1:PYDC2:CXCL6:PTGER4:ISL1:C5orf30:NDFIP1:FLOT1:TNFAIP3:ITGB8:HSPB1:CD36:LYN:S1PR3:KLF4:PAEP
GO_bp	GO_ORGANONITROGEN_COMPOUND_BIOSYNTHETIC_PROCESS	1814	106	5.006318671883658e-06	0.0002573177778905237	RPL22:ACOT7:MTOR:A3GALT2:MFSD2A:RIMKLA:ELOVL1:BCL10:EXTL2:DPH5:AMPD1:PHGDH:CERS2:DAP3:UHMK1:SOAT1:FMOD:B3GALNT2:LARP4B:ALDH18A1:GPAM:SMPD1:APBB1:BBOX1:GYLTL1B:SYVN1:MRPL49:AASDHPPT:DDX25:SLC11A2:DAO:MTIF3:SLC25A15:KDELC1:PSEN1:MTFMT:SLC51B:KIAA1199:ACAN:TARSL2:RPL3L:SMPD3:METTL16:DHX33:ASGR2:RCVRN:PRPSAP2:RPL19:RPS6KB1:RPL38:TNRC6C:MPPE1:ST8SIA5:MUC16:TECR:CHST8:SARS2:MRPS12:PUM2:IGFBP5:NCL:PER2:PANK2:CDS2:EIF6:RPN2:UCKL1:ATP5L2:TYMP:CHKB:RPL32:DAZL:EIF1B:RPL14:TRAK1:HYAL1:TMEM115:DHFRL1:A4GNT:MRPS22:XRN1:EIF4E:DCTD:RPL37:DHX29:PPAP2A:PDGFRB:CPEB4:EIF4E1B:MGAT1:MUC21:RPS18:B3GALT4:GSTA2:GSTA1:FUT9:ASL:HSPB1:NSMAF:RPL7:SLC35D2:KLF4:RPL12:RP11-203J24.9:ST6GALNAC6:ST6GALNAC4:DPM2
GO_bp	GO_POSITIVE_REGULATION_OF_NERVOUS_SYSTEM_DEVELOPMENT	530	42	5.656492662057763e-06	0.00028871681295919834	CLSTN1:MTOR:SPEN:NBL1:MACF1:CDC20:ARHGEF2:SEMA4A:ABL2:ASPM:ZFYVE27:APBB1:TENM4:TRPC6:CDON:FBXW8:FOXG1:PSEN1:NUMB:TCF12:SH3GL3:RRN3:RNF112:RND2:SPAG9:RNF157:TCF3:MEGF8:NCOA1:C21orf91:PLXNB2:SHANK3:TRAK1:DVL3:MAP3K13:NIPBL:CPNE5:CNR1:THBS2:CREB3L2:STAR:LYN
GO_bp	GO_CYTOSKELETON_ORGANIZATION	1293	81	5.838423043129386e-06	0.0002959476508069034	MTOR:CDC42:MACF1:CDC20:STIL:HOOK1:SSX2IP:PRUNE:TMOD4:MSTO1:ARHGEF2:LMNA:ARHGEF11:ABL2:ASPM:TUBB8:WEE1:ARL2:SAC3D1:DNAJB13:NCKAP1L:CIT:LCP1:FAM179B:FRMD6:GOLGA8R:PKD1:CORO1A:RND2:BRCA1:KPNB1:TUBD1:DNAI2:KIF19:AZI1:FSCN2:TUBB4A:ARHGEF18:MAST3:NPHS1:ZNF135:XPO1:DCTN1:RTKN:RND3:VIL1:BFSP1:DSTN:CHMP4B:CELSR1:SHANK3:NAT6:RASSF1:ZMYND10:CCDC66:GSK3B:ATP2C1:MRAS:SLAIN2:CXCL1:PTGER4:MCIDAS:KIF2A:CETN3:DIAPH1:

						HDAC3:FCHSD1:ARAP3:PDGFRB:EDN1:LRRC16A:TUBB:MAP7:TNFAIP3:SYNE1:FGFR1OP:LRRC6:CNTLN:CHMP5:NTMT1:TUBB4B
GO_bp	GO_REGULATION_OF_ORGANELLE_ORGANIZATION	1254	79	6.216925075489949e-06	0.0003129753377044598	EXOSC10:MTOR:CDC42:CDC20:TAL1:STIL:HORMAD1:SETDB1:PRUNE:TMOD4:SYT11:ARHGEF2:UBQLN4:LMNA:SMG5:ABL2:CTR9:ARL2:CDCA5:LRFN4:PHB2:NCKAP1L:CIT:ULK1:SIAH3:LCP1:STXBP6:SCFD1:FAM179B:DLGAP5:MOAP1:PKD1:SMG1:CORO1A:SLX1A:SMPD3:VAT1:RND2:BRCA1:NPEPPS:AZI1:TUBB4A:INSR:NPHS1:GSK3A:XPO1:DCTN1:ANAPC1:RND3:VIL1:MFF:DSTN:CHMP4B:CELSR1:SHANK3:NAT6:RASSF1:ZMYND10:NPRL2:FAM208A:GSK3B:XRN1:SLAIN2:NIPBL:PTGER4:ISL1:CCNO:DIAPH1:FCHSD1:PDGFRB:EDN1:LRRC16A:C6orf89:SEPT7:YWHAG:PPP3CC:TERF1:CHMP5:LRSAM1
GO_bp	GO_ESTABLISHMENT_OF_PROTEIN_LOCALIZATION_TO_ORGANELLE	552	43	6.742870780927005e-06	0.00033714353904635023	RPL22:TARDBP:LMNA:IPO7:PEX16:HSPA8:PHB2:DAO:SIAH3:KPNA3:IPO5:PSEN1:NUMB:MOAP1:LONP2:RPL19:NPEPPS:KPNB1:RPL38:GSK3A:PEX13:MFF:CHMP4B:RPL32:RPL14:HYAL2:TERT:NIPBL:RPL37:IPO11:HDAC3:TSPAN17:RPS18:PEX7:TNFAIP3:YWHAG:CD36:PPP3CC:TERF1:RPL7:SCRIB:RPL12:LRSAM1
GO_bp	GO_CELL_ACTIVATION_INVOLVED_IN_IMMUNE_RESPONSE	702	51	7.050546723145449e-06	0.00035014539469675033	MTOR:NRAS:CTSS:HRNR:SEMA4A:FCGR2A:HSPA6:RAB18:PSAP:LINC00610:COMMD9:HSPA8:PTPN6:METTL7A:NCKAP1L:RAP1B:APAF1:LCP1:PSEN1:CHRNB4:CORO1A:VAT1:KPNB1:TBX21:PSMD12:CD97:IL12RB1:CEACAM1:CD33:FPR2:IL18R1:SLC11A1:SLPI:DNAJC5:SYNGR1:CYB5R3:ARSA:FOXP1:ARMC8:CXCL1:PLAC8:PTGER4:DIAPH1:NDFIP1:TUBB:RAB44:RNASET2:LAT2:CD36:LYN:CDH17:TUBB4B
GO_bp	GO_POSITIVE_REGULATION_OF_PROTEIN_METABOLIC_PROCESS	1615	96	7.155795362895506e-06	0.0003529872209213555	MTOR:CDC20:TAL1:BCL10:F3:ARNT:CKS1B:ARHGEF2:LMNA:UHMK1:SOAT1:LARP4B:GDF2:C10orf54:PSAP:FAS:SMPD1:HPX:CTR9:TRPC6:CDON:USP5:PHB2:GPRC5A:NCKAP1L:RAP1B:APAF1:FBXW8:ANKLE2:TNFRSF19:PSME1:FANCM:MAP3K9:PSEN1:SLC51B:KIAA1199:PKD1:GRIN2A:CCL17:DHX33:MAPK7:BRCA1:SPAG9:RPS6KB1:RNFT1:MBP:INSR:KEAP1:PKN1:GSK3A:CXCL17:CARD8:FPR2:SPDYA:ALK:FSHR:SLC11A1:COPS8:EIF6:SEMG1:SEMG2:TAB1:FBLN1:MAPK8IP2:DAZL:TGFBR2:GNAI2:IFT57:GSK3B:DVL3:MAP3K13:SENP2:MFI2:AREG:AREGB:NPY5R:NIPBL:ISL1:DHX29:CKS1B:HDAC3:RELL2:NDFIP1:PDGFRB:EDN1:FLOT1:CDKN1A:C6orf89:TNFAIP3:HSPB1:CD36:PILRB:PRKAR2B:NCAPG2:RB1CC1:LYN:UBQLN1:KLF4
GO_bp	GO_REGULATION_OF_NEURON_DIFFERENTIATION	646	48	7.209108321898514e-06	0.0003532463077730272	MTOR:NBL1:MACF1:CDC20:CERS2:SEMA6C:ARHGEF2:SEMA4A:ABL2:ASPM:ZFYVE27:APBB1:TRPC6:CDON:FBXW8:FOXG1:PSEN1:TCF12:SH3GL3:RRN3:RNF112:RND2:SPAG9:RNF157:TCF3:S1PR5:MEGF8:NCOA1:ALK:BCL11A:CTDSP1:C21orf91:PLXNB2:SHANK3:TRAK1:C3orf17:GSK3B:DVL3:MAP3K13:EIF4E:ISL1:CPNE5:CNR1:YWHAG:CREB3L2:KEL:LYN:KLF4
GO_bp	GO_LEUKOCYTE_MEDIATED_IMMUNITY	857	59	7.263229830681418e-06	0.00035354131957290343	BCL10:NRAS:CTSS:HRNR:APCS:FCGR2A:HSPA6:RAB18:PSAP:HPX:LINC00610:COMMD9:CRTAM:HSPA8:PTPN6:C1S:METTL7A:NCKAP1L:RAP1B:APAF1:PSEN1:CHRNB4:CORO1A:VAT1:KPNB1:TBX21:PSMD12:SERPINB4:CD97:IL12RB1:POU2F2:CEACAM1:KDELR1:CD33:FPR2:IL18R1:IL18RAP:SLC11A1:SLPI:DNAJC5:SYNGR1:CYB5R3:ARSA:TUSC2:ARMC8:CXCL6:CXCL1:PLAC8:CFI:DIAPH1:NDFIP1:HLA-A:TUBB:C4A:RAB44:RNASET2:LAT2:CD36:LYN:TUBB4B
GO_bp	GO_REGULATION_OF_PROTEIN_COMPLEX_ASSEMBLY	429	36	7.447387588591581e-06	0.00036012038668518497	MTOR:FBLIM1:CDC42:TAL1:PRUNE:TMOD4:ARHGEF2:SELP:ARL2:HSPA8:NCKAP1L:ULK1:LCP1:ERCC5:STXBP6:FAM179B:CORO1A:DHX33:TUBB4A:NPHS1:DCTN1:CREB1:VIL1:GSK3B:PYDC2:SLAIN2:PTGER4:ANKRA2:FCHSD1:LRRC16A:ESR1:GTF2H5:CD36:TERF1:CDH17:XPA
GO_bp	GO_POSITIVE_REGULATION_OF_NEURON_DIFFERENTIATION	361	32	7.787062248139054e-06	0.0003740843629007977	MTOR:NBL1:MACF1:ARHGEF2:ABL2:ZFYVE27:APBB1:TRPC6:CDON:FBXW8:FOXG1:PSEN1:TCF12:SH3GL3:RRN3:RNF112:RND2:SPAG9:RNF157:TCF3:MEGF8:NCOA1:C21orf91:PLXNB2:SHANK3:TRAK1:DVL3:MAP3K13:CPNE5:CNR1:CREB3L2:LYN
GO_bp	GO_REGULATION_OF_CELL_POPULATION_PROLIFERATION	1684	99	8.101123521267664e-06	0.000383849146205493	MTOR:ZBTB17:LDLRAP1:MPL:CDC20:TAL1:FOXE3:DDAH1:F3:PTBP2:VAV3:WDR77:HIPK1:NRAS:ARNT:CERS2:ARHGEF2:LMNA:ABL2:ASPM:CUL2:GDF2:C10orf54:SFRP5:GPAM:GRK5:ABCC8:PRKRIR:YAP1:BTG4:CDON:PTPN6:PHB2:PTHLH:NCKAP1L:LGR5:NUAK1:RNASEH2B:TNFSF13B:FOXG1:CORO1A:TGFB1I1:ESRP2:SMPD3:DERL2:GLP2R:IKZF3:BRCA1:RPS6KB1:SSTR2:AZI1:INSR:CD320:PKN1:IL12RB1:CEACAM1:CD33:OSR1:SPDYA:ALK:CREB1:IGFBP2:IGFBP5:COPS8:PER2:TGM2:FBLN1:TGFBR2:SCN5A:GNAI2:HYAL1:TMEM115:FOXP1:IFT57:CNBP:A4GNT:IL8:CXCL1:AREG:AREGB:PLAC8:NPY5R:TERT:ISL1:CCNO:PPAP2A:NDFIP1:PDGFRB:EDN1:CDKN1A:TNFAIP3:ESR1:SOD2:FGFR1OP:ASH2L:LYN:GML:S1PR3:KLF4:NACC2
GO_bp	GO_POSITIVE_REGULATION_OF_SIGNALING	1813	105	8.128614137692338e-06	0.000383849146205493	RNF207:CLSTN1:TARDBP:MTOR:C1orf64:HTR6:LDLRAP1:TRIM62:MACF1:BCL10:F3:VAV3:LAMTOR5:ARNT:RUSC1:SELP:ASPM:NUCKS1:GDF2:TACR2:PSAP:FAS:HPX:YAP1:CDON:PTPN6:PHB2:RAP1B:LGR5:APAF1:P2RX2:TNFRSF19:PSME1:MAP3K9:PSEN1:MOAP1:CHRNB4:GRIN2A:TGFB1I1:SIAH1:CCL17:CALB2:DHX33:EPN2:SPAG9:TRIM25:TUBD1:PSMD12:INSR:CC2D1A:PKN1:GIPC1:DEDD2:GSK3A:CXCL17:GRIN2D:FPR2:PUM2:ALK:FSHR:IL18R1:CREB1:IGFBP5:MFF:COPS8:TGM2:ZBP1:TAB1:MAPK8IP2:SHANK3:MYRIP:GNAI2:HYAL2:FOXP1:C3orf17:GSK3B:ATP2C1:DVL3:MAP3K13:AREG:AREGB:NPY5R:TERT:ISL1:HDAC3:R

						ELL2:NDFIP1:PDGFRB:EDN1:FLOT1:RGL2:CNR1:TNFAIP3:ESR1:CRHR2:YWHAG:CD36:KLF14:BLK:PPP3CC:RB1CC1:LYN:CA2:CALB1:SCRIB:NACC2
GO_bp	GO_GROWTH	979	65	8.147002286810464e-06	0.000383849146205493	CLSTN1:MTOR:CDC42:MACF1:STIL:SEMA6C:ADAM15:SEMA4A:ARHGEF11:ASPM:GDF2:PSAP:TLL2:ZFYVE27:GPAM:APBB1:CTR9:TENM4:YAP1:ULK1:POSTN:COCH:SMPD3:ANKRD11:DERL2:HNF1B:RND2:RPS6KB1:RNF157:SAFB:INSR:GSK3A:MEGF8:CEACAM1:BCL11A:CREB1:IGFBP2:IGFBP5:VIL1:PPARA:SHANK3:TGFBR2:HYAL1:HYAL2:CACNA2D2:GSK3B:MAP3K13:PSAPL1:AREG:AREGB:PLAC8:NPY1R:NIPBL:PDGFRB:EDN1:CDKN1A:CPNE5:ESR1:FGFR1OP:HOXA11:CD36:NCAPG2:RDH10:XPA:NAIF1:SLC25A25
GO_bp	GO_CELLULAR_RESPONSE_TO_HORMONE_STIMULUS	689	50	8.905246299555133e-06	0.00041615917408457524	C1orf64:RPE65:CTSS:PKLR:BGLAP:NUCKS1:GPAM:SCGB2A1:UCP2:UCP3:YAP1:PHB2:RAP1B:LGR5:PCK2:MYO5A:TGFB1I1:GLP2R:BRCA1:RPS6KB1:SSTR2:SAFB2:SAFB:INSR:GSK3A:CEACAM1:NCOA1:FSHR:ATP6V1B1:CREB1:IGFBP2:PPARA:GNAI2:FOXP1:NR1I2:UBA5:EIF4E:PTGER4:ISL1:PPAP2A:EDN1:ESR1:CRHR2:YWHAG:PRKAR2B:STAR:LYN:CA2:CYP11B1:CYP11B2
GO_bp	GO_REGULATION_OF_CELL_CYCLE_PROCESS	785	55	8.946006735423522e-06	0.00041615917408457524	ZBTB17:CDC42:CDC20:STIL:FOXE3:HORMAD1:WEE1:CDCA5:PTPN6:PHB2:APAF1:CIT:RNASEH2B:PSME1:DLGAP5:MGA:PKD1:SMPD3:RNF112:BRCA1:PSMD12:AZI1:TUBB4A:INSR:GIPC1:XPO1:DCTN1:ANAPC1:PKP4:CTDSP1:PCNA:CHMP4B:GTSE1:HYAL1:NEK11:TERT:NIPBL:CCNO:PDGFRB:LSM11:EDN1:TUBB:CDKN1A:FGFR1OP:SEPT7:YWHAG:MEPCE:PRKAR2B:CEP41:TERF1:GML:CHMP5:KLF4:NACC2:TUBB4B
GO_bp	GO_CARBOHYDRATE_METABOLIC_PROCESS	559	43	9.195491166864809e-06	0.00042507459167582607	MTOR:A3GALT2:OVGP1:ARNT:CLK2:CREM:KCNJ11:PC:MOGAT2:DGAT2:TPI1:ENO2:SDS:PCK2:ARPP19:SMPD3:SHPK:SHPK:IMPA2:ST8SIA5:INSR:CHST8:GSK3A:PPP1CB:RPE:IGFBP5:PER2:EIF6:NAGA:PPARA:MIOX:HYAL3:HYAL1:HYAL2:GSK3B:A4GNT:GK5:MAN2B2:COQ2:NPY1R:FUT9:FAM3C:IPPK:RP11-203J24.9:ST6GALNAC6
GO_bp	GO_EPITHELIAL_TUBE_MORPHOGENESIS	314	29	9.39489314497085e-06	0.0004315779038470985	RNF207:STIL:BCL10:RBM15:GDF2:YAP1:PHB2:LGR5:APAF1:PSEN1:PKD1:ESRP2:HNF1B:MEGF8:OSR1:CELSR1:PLXNB2:TGFBR2:FOXP1:ARL13B:IFT57:NPHP3:DVL3:AREG:AREGB:EDN1:ESR1:HOXA11:RDH10:SCRIB
GO_bp	GO_MORPHOGENESIS_OF_AN_EPITHELIUM	526	41	1.0781371873998052e-05	0.0004921930638129545	RNF207:MTOR:CDC42:STIL:BCL10:RBM15:TMEM79:GDF2:YAP1:PHB2:LGR5:APAF1:PSME1:PSEN1:PKD1:TGFB1I1:ESRP2:HNF1B:PSMD12:MEGF8:CEACAM1:OSR1:IGFBP5:TGM2:CELSR1:PLXNB2:TGFBR2:FOXP1:ARL13B:IFT57:NPHP3:DVL3:AP2M1:AREG:AREGB:EDN1:ESR1:HOXA11:RDH10:CA2:SCRIB:KLF4
GO_bp	GO_MEMBRANE_LIPID_METABOLIC_PROCESS	205	22	1.0869864950486208e-05	0.0004931697986794669	A3GALT2:ELOVL1:CERS2:CREM:PSAP:SMPD1:PDXDC1:PLA2G15:SMPD3:MPPE1:ST8SIA5:NAGA:ARSA:PSAPL1:PPAP2A:B3GALT4:ITGB8:ESYT2:NSMAF:RP11-203J24.9:ST6GALNAC6:ST6GALNAC4:DPM2
GO_bp	GO_INFLAMMATORY_RESPONSE	714	51	1.1185775562853767e-05	0.0005043892661777619	F3:TNFAIP8L2:ASH1L:APCS:SELP:LRFN5:PSEN1:PLA2G4B:CCL17:NFATC3:SHPK:TRPV1:SHPK:MAPK7:CD97:PTGER1:CXCL17:FPR2:IL1RL1:IL18R1:IL18RAP:SLC11A1:TGM2:APOL2:PPARA:ACKR2:HYAL3:HYAL1:HYAL2:TUSC2:FOXP1:CD200R1:PYDC2:IL8:CXCL6:PF4V1:CXCL1:NPY5R:DROSHA:PTGER4:ISL1:C5orf30:NDFIP1:C4A:CNR1:TNFAIP3:ESR1:TPST1:CD36:LYN:S1PR3:KLF4
GO_bp	GO_ORGANIC_CYCLIC_COMPOUND_CATABOLIC_PROCESS	601	45	1.1376909196698595e-05	0.0005073683305986794	RPL22:ACOT7:TARDBP:EXOSC10:MTOR:DNASE2B:CSDE1:SMG5:HSPA8:APAF1:RNASEH2B:PSME1:ANP32A:SMG1:SULT1A3:DCTPP1:METTL16:RPL19:KPNB1:PSMD12:RPL38:TNRC6C:PDE4A:DEDD2:GSK3A:PUM2:XPO1:SLC11A1:APOBEC3B:TYMP:RPL32:DAZL:ENTPD3:RPL14:XRN1:DROSHA:RPL37:SKIV2L2:MGAT1:RPS18:RNASET2:HSPB1:RPL7:RPL12:CEL
GO_bp	GO_RESPONSE_TO_OXYGEN_LEVELS	385	33	1.1389901299154026e-05	0.0005073683305986794	MTOR:MPL:DDAH1:ARNT:ADAM15:PKLR:LMNA:PSEN2:CUL2:FAS:UCP2:UCP3:TRPC6:PHB2:SLC11A2:APAF1:P2RX2:POSTN:PSME1:SCFD1:ERO1L:NPEPPS:PSMD12:CREB1:PPARA:TGFBR2:TERT:PDGFRB:CPEB4:EDN1:CDKN1A:CLDN3:UBQLN1
GO_bp	GO_NEGATIVE_REGULATION_OF_PHOSPHORUS_METABOLIC_PROCESS	546	42	1.1629787189828531e-05	0.0005143059704676095	TARDBP:MTOR:SPOCD1:ASH1L:ZMYND11:DUSP8:SMPD1:IPO7:UBASH3B:PTPN6:GPRC5A:CSRNP2:NCKAP1L:ANKLE2:IPO5:PPP1R36:ZFYVE1:PSEN1:ARPP19:CD2BP2:PKN1:CEACAM1:CBLC:TSKS:CTDSP1:FBLN1:GNAI2:HYAL2:NPRL2:GSK3B:HDAC3:CDKN1A:TNFAIP3:FGFR1OP:HSPB1:YWHAG:PPP1R35:PRKAR2B:GFRA2:LYN:KLF4:PIP5KL1
GO_bp	GO_IMPORT_INTO_CELL	773	54	1.1685591437835483e-05	0.0005143059704676095	CDC42:LDLRAP1:TSPAN1:SYT11:PEAR1:ABL2:DMBT1:HPX:KCNJ11:SLC11A2:NCKAP1L:HEATR5A:PSEN1:NUMB:SH3GL3:TRPV1:RABEP1:CLEC10A:ASGR2:EPN2:RPS6KB1:KCNJ16:KCNJ2:RALBP1:SH3GL1:DENND1C:INSR:EPS15L1:CEACAM1:KCNJ14:FPR2:SLC9A4:PER2:APOL1:TGFBR2:ACKR2:ACKR4:AP2M1:IL8:CFI:MCTP1:MARCH3:MEGF10:SLC12A2:SNCB:LRRC16A:FLOT1:CD36:ESYT2:PPP3CC:SCRIB:LRSAM1:CEL:FCN2
GO_bp	GO_RESPONSE_TO_ORGANIC_CYCLIC_COMPOUND	912	61	1.2316576153634568e-05	0.0005388502067215123	MTOR:C1orf64:ABCD3:HCN3:PKLR:BGLAP:GPAM:SMPD1:KCNJ11:OR5T1:SCGB2A1:UCP3:YAP1:FDX1:LINC01059:GRAMD1B:PHB2:RAP1B:P2RX2:POSTN:PCK2:SULT1A3:TGFB1I1:LONP2:TRPV1:HNF1B:BRCA1:TRIM25:RPS6KB1:SSTR2:SAFB2:SAFB:NCOA1:IGFBP2:IGFBP5:PCNA:PPARA:TGFBR2:FOXP1:NR1I2:UBA5:XRN1:AREG:AREGB:EIF4E:ISL1:PPAP2A:DIAPH1:PDGFRB:SOX30:EDN1:FLOT1:CDKN1A:CNR1:ESR1:CLDN4:CD36:PRKAR2B:STAR:LYN:CA2:KLF4:PAPPA

GO_bp	GO_NEGATIVE_REGULATION_OF_DEVELOPMENTAL_PROCESS	1013	66	1.2669237821187045e-05	0.0005486986513542685	TRIM62:TIE1:TAL1:FOXE3:RBM15:WDR77:CTSK:CERS2:SEMA6C:ARHGEF2:SEMA4A:APCS:ASPM:GDF2:TLL2:SFRP5:CTR9:ABCC8:TRPC6:YAP1:UBASH3B:PHB2:PTHLH:POSTN:FOXG1:PSEN1:SKOR1:ADAMTS7:TGFB1I1:CCL17:NFATC3:TRPV1:EPN2:HNF1B:VAT1:NBR1:TBX21:SKOR2:GSK3A:CEACAM1:OSR1:BCL11A:IGFBP5:CTDSP1:FBLN1:PPARA:TGFBR2:HYAL3:C3orf17:GSK3B:NPHP3:MFI2:AREG:AREGB:PLAC8:EIF4E:TERT:ISL1:NDFIP1:CDKN1A:THBS2:NCAPG2:LYN:S1PR3:KLF4:PAEP:NELFB
GO_bp	GO_MYELOID_LEUKOCYTE_MEDIATED_IMMUNITY	548	42	1.2690989214996687e-05	0.0005486986513542685	NRAS:CTSS:HRNR:FCGR2A:HSPA6:RAB18:PSAP:LINC00610:COMMD9:HSPA8:PTPN6:METTL7A:NCKAP1L:RAP1B:APAF1:PSEN1:CHRNB4:VAT1:KPNB1:PSMD12:CD97:CEACAM1:CD33:FPR2:SLC11A1:SLPI:DNAJC5:SYNGR1:CYB5R3:ARSA:TUSC2:ARMC8:CXCL6:CXCL1:PLAC8:DIAPH1:TUBB:RAB44:RNASET2:LAT2:CD36:LYN:TUBB4B
GO_bp	GO_TUBE_MORPHOGENESIS	894	60	1.3093736169719299e-05	0.0005628009406282856	RNF207:TIE1:TAL1:STIL:BCL10:DDAH1:F3:VAV3:RBM15:HIPK1:GJA5:ADAM15:SEMA4A:GDF2:SFRP5:ABCC8:YAP1:PHB2:LGR5:APAF1:PSEN1:PKD1:ESRP2:EPN2:MAPK7:HNF1B:BRCA1:GJC1:MEGF8:CXCL17:CEACAM1:OSR1:NCL:TGM2:CELSR1:PLXNB2:TYMP:TGFBR2:HYAL1:FOXP1:ARL13B:IFT57:NPHP3:DVL3:IL8:AREG:AREGB:TERT:NIPBL:ISL1:PDGFRB:EDN1:TNFAIP3:ESR1:THBS2:ITGB8:HOXA11:HSPB1:RDH10:SCRIB:KLF4
GO_bp	GO_MULTICELLULAR_ORGANISMAL_HOMEOSTASIS	477	38	1.3770968783269615e-05	0.0005884687241687887	ACOT11:RPE65:CTSK:HRNR:FLG:BGLAP:TMEM79:CDH23:UCP2:YAP1:UBASH3B:SLC11A2:PRKAB1:CORO1A:ANKRD11:TRPV1:PLEKHM1:ATP6V1B1:IL18R1:POTEE:SLC11A1:PER2:CHMP4B:MC3R:HYAL2:CCDC66:NPHP3:PLAC8:HDAC3:CNR1:TNFAIP3:CLDN4:HSPB1:CD36:PRKAR2B:PIP:CA2:CYP11B2
GO_bp	GO_REGULATION_OF_RESPONSE_TO_EXTERNAL_STIMULUS	779	54	1.4487377657696885e-05	0.0006155041952836538	MTOR:NBL1:F3:CERS2:SEMA6C:TNFAIP8L2:ASH1L:SYT11:SEMA4A:APCS:SELP:ABCC8:UBASH3B:NCKAP1L:LRFN5:MAPKBP1:SHPK:SHPK:MAPK7:NPLOC4:IL12RB1:MEGF8:CXCL17:CEACAM1:CARD8:FPR2:PUM2:IL1RL1:TGM2:PPARA:TGFBR2:HYAL2:FOXP1:PROS1:CD200R1:PYDC2:IL8:CXCL6:NPY5R:DROSHA:PTGER4:ISL1:C5orf30:NDFIP1:PDGFRB:EDN1:CDKN1A:CNR1:TNFAIP3:ESR1:HSPB1:CD36:LYN:KLF4:LRSAM1
GO_bp	GO_PEPTIDE_SECRETION	591	44	1.6284877541397857e-05	0.0006878956892487026	TARDBP:GJA5:GOLPH3L:SYT11:TACR2:GPAM:APBB1:KCNJ11:ABCC8:ARL2:UCP2:CRTAM:POSTN:TNFSF13B:MIA2:CTAGE5:MAPKBP1:MYO5A:SMPD3:TRPV1:HNF1B:MBP:POU2F2:CARD8:IL1RL1:PER2:ARFGAP3:MYRIP:HYAL2:CACNA2D2:FOXP1:CD200R1:PYDC2:MTTP:PTGER4:ISL1:EDN1:CNR1:TNFAIP3:CD36:COPG2:BLK:LYN:ENY2:PAEP
GO_bp	GO_REGULATION_OF_CELLULAR_LOCALIZATION	881	59	1.6419354078997875e-05	0.0006896128713179108	TARDBP:LDLRAP1:KCNC4:SYT11:LMNA:UHMK1:SEC16B:TACR2:KCNJ11:CDCA5:UCP2:UBASH3B:PTPN6:RAP1B:P2RX2:SIAH3:LCP1:IPO5:SCFD1:PSEN1:NUMB:MYO5A:SLC51B:CHRNB4:KIAA1199:MESDC2:CORO1A:DERL2:STAC2:NPEPPS:AZI1:SH3GL1:GIPC1:GSK3A:XPO1:VIL1:MFF:PER2:LIME1:DNAJC5:GTSE1:HYAL2:CCDC66:GSK3B:DVL3:AP2M1:TERT:NIPBL:MCTP1:DIAPH1:HDAC3:CNR1:YWHAG:CD36:PPP3CC:LYN:TERF1:ENY2:SCRIB
GO_bp	GO_COAGULATION	341	30	1.7074253427989216e-05	0.0007112271142316166	CDC42:MPL:F3:VAV3:PEAR1:F5:SELP:F13B:CSRP1:TRPC6:UBASH3B:PTPN6:P2RX2:PSEN1:DGKE:MYL12A:CEACAM1:MERTK:SEMG1:SEMG2:FBLN1:PROS1:GP9:PF4V1:EDN1:LRRC16A:HSPB1:CD36:PRKAR2B:LYN
GO_bp	GO_CELL_CYCLE_PHASE_TRANSITION	611	45	1.7127510097822604e-05	0.0007112271142316166	CDC20:MCM10:CUL2:WEE1:CDCA5:PTPN6:PHB2:CIT:POLE:RNASEH2B:PSME1:DLGAP5:ARPP19:PKD1:BRCA1:RPS6KB1:PSMD12:AZI1:TUBB4A:PPP1CB:SPDYA:DCTN1:ANAPC1:CTDSP1:PCNA:CHMP4B:GTSE1:HYAL1:NEK11:EIF4E:TERT:LSM11:TUBB:CDKN1A:FGFR1OP:SEPT7:YWHAG:ABCB1:MEPCE:PRKAR2B:CEP41:GML:KLF4:NACC2:TUBB4B
GO_bp	GO_RIBONUCLEOPROTEIN_COMPLEX_BIOGENESIS	483	38	1.81807710137477e-05	0.000750722848039582	EXOSC10:EBNA1BP2:RPF1:PTBP2:WDR77:KIAA0907:SNRPE:EMG1:DDX47:DHX37:GTF3A:PRPF39:RPL3L:RRN3:CD2BP2:PRMT7:METTL16:UTP18:RPL38:NOL10:PUM2:TRMT61B:XPO1:ESF1:EIF6:DDX27:RPL14:SHQ1:ISY1:KIAA1239:DROSHA:SKIV2L2:DIMT1:UTP15:WDR46:GTF2H5:RPL7:RPL12
GO_bp	GO_RESPONSE_TO_NITROGEN_COMPOUND	968	63	2.0491578318539945e-05	0.0008414139700629531	MTOR:RPE65:LAMTOR5:HCN3:PKLR:ARHGEF2:BGLAP:NUCKS1:SMPD1:KCNJ11:ABCC8:OR5T1:UCP2:UCP3:FDX1:PDE1B:RAP1B:P2RX2:IPO5:PCK2:PSEN1:MYO5A:GRIN2A:SULT1A3:SMPD3:TRPV1:GLP2R:CACNB1:BRCA1:RPS6KB1:INSR:GSK3A:CEACAM1:FPR2:BCL11A:ATP6V1B1:CREB1:IGFBP5:PCNA:EIF6:PPARA:GNAI2:GSK3B:XRN1:AREG:AREGB:DIAPH1:CPEB4:EDN1:FLOT1:CDKN1A:CNR1:TNFAIP3:CRHR2:YWHAG:CD36:PRKAR2B:STAR:LYN:CA2:CYP11B1:CYP11B2:CHMP5:KLF4
GO_bp	GO_MEMBRANE_ORGANIZATION	869	58	2.1269332786391877e-05	0.0008623112186637404	CLSTN1:TARDBP:CDC42:LDLRAP1:TIE1:VAV3:GOLPH3L:SYT11:LMNA:F5:SEC16B:CRB1:REEP3:PSAP:HSPA8:LINC01059:GRAMD1B:NCKAP1L:ANKLE2:STXBP6:SCFD1:NUMB:MOAP1:MESDC2:CORO1A:TRAPPC2L:SPG7:RABEP1:EPN2:LYZL6:NAPG:MBP:EPS15L1:GSK3A:DCTN1:CHMP4B:SYNGR1:ARFGAP3:SHANK3:HYAL2:STX19:GSK3B:PLSCR4:AP2M1:ATP10D:AREG:AREGB:MEGF10:FCHSD1:FLOT1:TAPBP:YWHAG:CD36:ABCB1:BET1:PPP3CC:SCRIB:CHMP5:NAIF1:CEL
GO_bp	GO_KETONE_BIOSYNTHETIC_PROCESS	42	9	2.1329624490913397e-05	0.0008623112186637404	UBIAD1:ADCK3:NDUFA9:TPI1:COQ9:COQ2:STAR:CYP11B1:CYP11B2

GO_bp	GO_ORGANIC_ACID_TRANSPORT	328	29	2.1358911053592662e-05	0.0008623112186637404	MFSD2A:LRRC8C:ABCD3:SLC16A4:PSAP:SLC16A12:SLC43A1:SLC25A15:PSEN1:SLC51B:SLC7A6:TRPV1:RPS6KB1:GIPC1:CEACAM1:OSR1:NCOA1:SLC11A1:PER2:PPARA:CPT1B:SLC10A4:PLA2G12A:NPY5R:SLC6A19:SLC6A18:EDN1:CD36:SLC35D2
GO_bp	GO_RESPONSE_TO_BIOTIC_STIMULUS	1010	65	2.1469789525913537e-05	0.0008623112186637404	BCL10:LAMTOR5:ADAM15:SYT11:SELP:ZMYND11:ACTA2:GPAM:DMBT1:CTR9:ABCC8:CRTAM:APAF1:COCH:MAPKBP1:SHPK:SHPK:LYZL6:IKZF3:TBX21:TRIM25:RPS6KB1:NPLOC4:CCDC130:PTGER1:IL12RB1:NFKBIB:CEACAM1:CARD8:PUM2:SLC11A1:VIL1:BPIFA2:WFDC12:PI3:SEMG1:SEMG2:SLPI:APOBEC3B:HYAL3:HYAL1:HYAL2:TUSC2:FOXP1:GSK3B:PLSCR4:KLHL6:ABCF3:IL8:CXCL6:PF4V1:CXCL1:PLAC8:DROSHA:PTGER4:EDN1:CNR1:TNFAIP3:HSPB1:CD36:STAR:LYN:SCRIB:CHMP5:LRSAM1:FCN2
GO_bp	GO_REGULATION_OF_TRANS_SYNAPTIC_SIGNALING	433	35	2.1900277623130787e-05	0.0008748208724457135	CLSTN1:MTOR:CDC20:KCNC4:SYT11:TACR2:RAP1B:PSEN1:CHRNB4:GRIN2A:CALB2:DGKE:SH3GL1:GIPC1:GRIN2D:CREB1:SYNGR1:MAPK8IP2:SHANK3:GNAI2:CACNA2D2:GSK3B:EIF4E:NPY5R:MCTP1:EDN1:FLOT1:CNR1:CRHR2:YWHAG:PRKAR2B:STAR:CA2:CALB1:CEL
GO_bp	GO_NEGATIVE_REGULATION_OF_DEFENSE_RESPONSE	215	22	2.29708979158394e-05	0.0009126275658455113	TNFAIP8L2:DCST1:ASH1L:APCS:LRFN5:MAPKBP1:MAPK7:NPLOC4:SERPINB4:CXCL17:CEACAM1:PPARA:CD200R1:PYDC2:NPY5R:PTGER4:ISL1:C5orf30:NDFIP1:TNFAIP3:SCRIB:KLF4
GO_bp	GO_SEXUAL_REPRODUCTION	813	55	2.3848361388717e-05	0.000942394925844462	MTOR:PHC2:HOOK1:OVGP1:HORMAD1:SMCP:ASH1L:ASPM:TUBB8:CREM:TRPC6:DDX25:AKAP3:LGR5:FNDC3A:TDRD9:HERC2:PRSS21:SIAH1:PRMT7:LYZL6:ZPBP2:SPATA32:RPS6KB1:SSTR2:AZI1:TAF4B:PAFAH1B3:NCOA1:PLB1:SPDYA:FSHR:MERTK:PANK2:PI3:SEMG1:SEMG2:OSBP2:WBP2NL:PKDREJ:NCAPH2:SYCE3:MAPK8IP2:DAZL:HYAL3:PPAP2A:SOX30:MEI4:CNR1:SYNE1:HOXA10:HOXA11:SEPT7:SPATA31A4:SPATA31A7:PAEP
GO_bp	GO_TISSUE_MORPHOGENESIS	659	47	2.531028994614744e-05	0.0009948162091132817	RNF207:MTOR:CDC42:TAL1:STIL:BCL10:RBM15:TMEM79:GDF2:ACTA2:YAP1:PHB2:LGR5:APAF1:PSME1:COCH:PSEN1:PKD1:TGFB1I1:ESRP2:HNF1B:PSMD12:MEGF8:CEACAM1:OSR1:IGFBP5:TGM2:CELSR1:PLXNB2:TGFBR2:FOXP1:ARL13B:IFT57:NPHP3:DVL3:AP2M1:AREG:AREGB:ISL1:EDN1:VPS52:ESR1:HOXA11:EXOC4:RDH10:CA2:SCRIB:KLF4
GO_bp	GO_RESPONSE_TO_NUTRIENT	217	22	2.6507188020731897e-05	0.001036318255065848	MTOR:SETDB1:PKLR:BGLAP:KCNJ11:UCP3:APAF1:P2RX2:POSTN:TRPV1:TRIM25:RPS6KB1:NCOA1:BCL11A:CREB1:IGFBP2:PCNA:TGFBR2:GNAI2:SLC6A19:CNR1:STAR
GO_bp	GO_POSITIVE_REGULATION_OF_CELL_CYCLE	402	33	2.7338133761530506e-05	0.0010572821238470896	ZBTB17:CDC42:TAL1:FOXE3:CKS1B:CDCA5:PHB2:CIT:FOXG1:DLGAP5:PKD1:SMPD3:RNF112:BRCA1:RPS6KB1:INSR:GIPC1:SPDYA:PKP4:PCNA:GTSE1:HYAL1:EIF4E:TERT:NIPBL:CCNO:CKS1B:PDGFRB:LSM11:EDN1:CDKN1A:C6orf89:MEPCE:GML
GO_bp	GO_POSITIVE_REGULATION_OF_GENE_EXPRESSION	1955	109	2.742009114763105e-05	0.0010572821238470896	RNF207:TARDBP:MTOR:ZBTB17:TAL1:FOXD2:BCL10:RBM15:ARNT:ASH1L:ARHGEF2:LMNA:C1orf85:ARHGEF11:NHLH1:UHMK1:SEC16B:NUCKS1:ZNF496:LARP4B:CREM:GDF2:C10orf54:ACTA2:RBM20:APBB1:ZNF143:CTR9:EHF:YAP1:POU2AF1:CDON:SOX5:CSRNP2:TFCP2:HOXC13:HOXC11:PRKAB1:LMO7:NRL:DLGAP5:SIX6:PSEN1:TCF12:PKD1:RRN3:TGFB1I1:NFATC3:DHX33:MAPK7:HNF1B:IKZF3:BRCA1:TBX21:RPS6KB1:TNRC6C:TAF4B:ASXL3:TCF3:SAFB:INSR:POU2F2:GSK3A:MYCN:OSR1:NCOA1:BCL11A:CREB1:SLC11A1:NCL:EIF6:PPARA:CAND2:DAZL:HYAL2:NR1I2:GSK3B:CNBP:DVL3:MFI2:PLAC8:TERT:DROSHA:NIPBL:ISL1:MCIDAS:DHX29:DIMT1:UTP15:HDAC3:CDX1:ADAM19:EDN1:ESR1:ITGB8:HOXA10:HOXA11:EVX1:NEUROD6:CD36:KLF14:CREB3L2:PIP:ASH2L:STAR:ENY2:IPPK:KLF4:PBX3
GO_bp	GO_REGULATION_OF_CELLULAR_COMPONENT_SIZE	367	31	2.747495042922369e-05	0.0010572821238470896	MTOR:MACF1:LRRC8B:LRRC8C:VAV3:LAMTOR5:SEMA6C:TMOD4:SEMA4A:ZFYVE27:NCKAP1L:CIT:CORO1A:RND2:PEX11G:NPHS1:MEGF8:CREB1:VIL1:DSTN:SHANK3:NAT6:GSK3B:AP2M1:MAP3K13:SLC12A2:FCHSD1:EDN1:LRRC16A:KEL:RB1CC1
GO_bp	GO_CELLULAR_AMIDE_METABOLIC_PROCESS	1020	65	2.8937113040512068e-05	0.001103540436761833	RPL22:ACOT7:MTOR:ELOVL1:ACOT11:DPH5:HMGCS2:CERS2:DAP3:UHMK1:PSEN2:LARP4B:GPAM:SMPD1:MRPL49:DGAT2:DDX25:MTIF3:SLC25A15:PSEN1:MTFMT:TARSL2:RPL3L:PLA2G15:SMPD3:METTL16:DHX33:RPL19:RPS6KB1:RPL38:TNRC6C:TECR:SARS2:MRPS12:GSK3A:PUM2:MTHFD2:IGFBP5:NCL:PER2:EIF6:RPL32:DAZL:EIF1B:RPL14:DHFRL1:MRPS22:XRN1:EIF4E:RPL37:DHX29:CPEB4:EIF4E1B:RPS18:B3GALT4:TAPBP:GSTA2:GSTA1:ITGB8:ASL:HSPB1:NSMAF:RPL7:RPL12:RP11-203J24.9:ST6GALNAC6
GO_bp	GO_REGULATION_OF_PROTEIN_MODIFICATION_PROCESS	1826	103	2.897732031224949e-05	0.001103540436761833	TARDBP:MTOR:CDC20:TAL1:BCL10:ARNT:CKS1B:ASH1L:ARHGEF2:LMNA:ZMYND11:GDF2:PSAP:FAS:DUSP8:SMPD1:HPX:IPO7:CTR9:TRPC6:UBASH3B:CDON:PTPN6:PHB2:GPRC5A:NCKAP1L:RAP1B:NUAK1:ANKLE2:TNFRSF19:IPO5:FANCM:PPP1R36:MAP3K9:PSEN1:ARPP19:SLC51B:KIAA1199:PKD1:N4BP1:CCL17:SMPD3:DHX33:BRCA1:NBR1:SPAG9:PPP4R1:PHLPP1:INSR:PKN1:GSK3A:CXCL17:CEACAM1:CBLC:FPR2:SPDYA:ALK:FSHR:SLC11A1:CTDSP1:COPS8:PER2:LIME1:TAB1:FBLN1:PLXNB2:MAPK8IP2:TGFBR2:GNAI2:HYAL2:GSK3B:DVL3:MAP3K13:SENP2:AREG:AREGB:NPY5R:NIPBL:ISL1:CCNO:CKS1B:HDAC3:RELL2:NDFIP

						1:PDGFRB:EDN1:FLOT1:CDKN1A:C6orf89:TNFAIP3:FGFR1OP:HSPB1:YWHAG:CD36:PILRB:PPP1R35:PRKAR2B:NCAPG2:GFRA2:RB1CC1:LYN:KIAA0020:UBQLN1:KLF4:PIP5KL1
GO_bp	GO_EMBRYONIC_ORGAN_MORPHOGENESIS	284	26	3.09496131428006e-05	0.0011722536983518501	RNF207:STIL:HIPK1:YAP1:HOXC11:FOXG1:PSEN1:HNF1B:RPL38:MEGF8:OSR1:ATP6V1B1:CELSR1:TGFBR2:HYAL1:LRIG1:ARL13B:IFT57:NPHP3:NIPBL:C5orf20:TIFAB:EDN1:HOXA11:RDH10:SCRIB
GO_bp	GO_NEGATIVE_REGULATION_OF_TRANSPORT	495	38	3.110060832362051e-05	0.0011722536983518501	MTOR:SYT11:TACR2:KCNJ11:ABCC8:UCP2:RAP1B:SIAH3:STXBP6:MAPKBP1:SH3GL3:CORO1A:DERL2:GSK3A:CEACAM1:CARD8:OSR1:SEMG1:GNAI2:CD200R1:KCNAB1:PYDC2:FGF12:NPY5R:PTGER4:MCTP1:C5orf20:TIFAB:NDFIP1:EDN1:CNR1:TNFAIP3:CD36:KEL:GEM:ENY2:UBQLN1:LRSAM1
GO_bp	GO_REGULATION_OF_SECRETION	782	53	3.173908355488578e-05	0.0011864224583052968	TARDBP:KCNC4:GJA5:GOLPH3L:SYT11:TACR2:GPAM:APBB1:KCNJ11:ABCC8:SYT13:OR5T1:ARL2:UCP2:CRTAM:RAP1B:POSTN:STXBP6:MAPKBP1:CHRNB4:SEPT1:SEPT1:SMPD3:TRPV1:MBP:GIPC1:NPHS1:CEACAM1:CARD8:IL1RL1:CREB1:PER2:MYRIP:GNAI2:HYAL3:HYAL2:CACNA2D2:FOXP1:CD200R1:GSK3B:PYDC2:NPY5R:PTGER4:ISL1:MCTP1:C5orf20:TIFAB:EDN1:CNR1:TNFAIP3:BLK:LYN:ENY2:PAEP
GO_bp	GO_MONOVALENT_INORGANIC_CATION_TRANSPORT	514	39	3.186479355388039e-05	0.0011864224583052968	RNF207:KCNC4:GJA5:HCN3:KCNJ11:ABCC8:LINC00610:COMMD9:UCP2:UCP3:NDUFA9:KCNA1:SLC11A2:SLC25A3:MTMR6:KCNK10:SLC5A10:KCNJ16:KCNJ2:KCNJ14:OSR1:ATP6V1B1:SLC4A5:SLC9A4:SCN3A:KCNS1:ATP5L2:SCN5A:ATP2C1:KCNAB1:FGF12:SLC34A2:SLC10A4:SLC4A4:SLC12A2:KCNIP1:KEL:KCNV2:PTPN3:SLC34A3
GO_bp	GO_NEGATIVE_REGULATION_OF_CELL_DEATH	1003	64	3.1996417907948715e-05	0.0011864224583052968	MTOR:STIL:FOXE3:TXNDC12:BCL10:LAMTOR5:ARHGEF2:LMNA:ZMYND11:PSAP:FAS:GPAM:GRK5:SYVN1:UCP2:YAP1:ERC1:PHB2:NCKAP1L:ANKLE2:ERCC5:PSEN1:RRN3:CORO1A:SULT1A3:CIAPIN1:MAPK7:HNF1B:BRCA1:RPS6KB1:RNF157:OSR1:MERTK:CREB1:VIL1:CHMP4B:DNAJC5:PPARA:MAPK8IP2:RAD18:GNAI2:FOXP1:CD200R1:GSK3B:PLAC8:NPY5R:TERT:ISL1:HDAC3:PDGFRB:CPEB4:SNCB:EDN1:IER3:RGL2:CDKN1A:TNFAIP3:SOD2:HSPB1:PIP:STAR:RB1CC1:NRBP2:KLF4
GO_bp	GO_FATTY_ACID_CATABOLIC_PROCESS	103	14	3.213157751882681e-05	0.0011864224583052968	MTOR:ABCD3:PCK2:LONP2:PLA2G15:LIPE:PEX13:PPARA:CPT1B:ACAD11:CNR1:PEX7:AIG1:CEL
GO_bp	GO_POSITIVE_REGULATION_OF_RNA_BIOSYNTHETIC_PROCESS	1592	92	3.228360430762712e-05	0.0011864224583052968	TARDBP:MTOR:ZBTB17:TAL1:FOXD2:BCL10:RBM15:WDR77:ARNT:ASH1L:ARHGEF2:PMF1:C1orf85:ARHGEF11:NHLH1:NUCKS1:ZNF496:CREM:GDF2:ACTA2:APBB1:ZNF143:CTR9:EHF:YAP1:POU2AF1:CDON:SOX5:CSRNP2:TFCP2:HOXC13:HOXC11:LMO7:NRL:DLGAP5:SIX6:PSEN1:GTF2A1:TCF12:PKD1:RRN3:TGFB1I1:NFATC3:DHX33:MAPK7:HNF1B:IKZF3:BRCA1:TBX21:TAF4B:ASXL3:TCF3:SAFB:INSR:PKN1:NFKBIB:POU2F2:MYCN:OSR1:NCOA1:CREB1:SLC11A1:NCL:PER2:WBP2NL:PPARA:CAND2:HYAL2:NR1I2:CNBP:DVL3:PLAC8:TERT:NIPBL:ISL1:MCIDAS:UTP15:HDAC3:CDX1:EDN1:ESR1:HOXA10:HOXA11:EVX1:NEUROD6:KLF14:CREB3L2:ASH2L:ENY2:IPPK:KLF4:PBX3
GO_bp	GO_REGULATION_OF_CELL_SIZE	174	19	3.3228937753264155e-05	0.0012128794955951364	MTOR:MACF1:LRRC8B:LRRC8C:VAV3:LAMTOR5:SEMA6C:SEMA4A:ZFYVE27:RND2:MEGF8:CREB1:SHANK3:GSK3B:MAP3K13:SLC12A2:EDN1:KEL:RB1CC1
GO_bp	GO_EPIDERMIS_DEVELOPMENT	460	36	3.3333558926560214e-05	0.0012128794955951364	CTSK:HRNR:FLG:LCE1F:LCE1E:LCE1D:LCE1C:LCE1B:TMEM79:PSAP:KRTAP5-1:KRTAP5-2:KRTAP5-3:KRTAP5-4:KRTAP5-5:YAP1:EMP1:PTHLH:HOXC13:LGR5:TNFRSF19:MYO5A:INSR:KEAP1:CNFN:PKP4:IGFBP5:PI3:KRTAP21-3:KRTAP8-1:PPARA:CELSR1:ATP2C1:RBP2:SCRIB:KLF4
GO_bp	GO_SENSORY_ORGAN_MORPHOGENESIS	253	24	3.4814082810251334e-05	0.0012605098948539277	FOXE3:RPE65:OLFM3:HIPK1:CRB1:CDON:C12orf57:HOXC13:NRL:FOXG1:TRPM1:RPL38:FSCN2:OSR1:ATP6V1B1:CELSR1:CCDC66:LRIG1:NIPBL:C5orf20:TIFAB:EDN1:CALB1:SCRIB
GO_bp	GO_SENSORY_ORGAN_DEVELOPMENT	535	40	3.5290465553268004e-05	0.0012714947147868618	FOXE3:RPE65:OLFM3:HIPK1:CRB1:RAB18:CDH23:PSAP:CDON:C12orf57:HOXC13:LGR5:NRL:FOXG1:SIX6:PSEN1:TRPM1:B9D1:RPL38:FSCN2:OSR1:ATP6V1B1:SLC4A5:MERTK:BFSP1:CELSR1:SCO2:TGFBR2:CCDC66:LRIG1:NIPBL:C5orf20:TIFAB:PDGFRB:EDN1:RDH10:CALB1:SCRIB:KLF4:SLC25A25
GO_bp	GO_REGULATION_OF_DEVELOPMENTAL_GROWTH	338	29	3.706582036957753e-05	0.0013289452669092432	MTOR:MACF1:SEMA6C:SEMA4A:TLL2:ZFYVE27:GPAM:YAP1:HNF1B:RND2:RPS6KB1:RNF157:INSR:GSK3A:MEGF8:BCL11A:CREB1:VIL1:PPARA:TGFBR2:CACNA2D2:GSK3B:MAP3K13:PLAC8:NPY1R:NIPBL:EDN1:CDKN1A:CPNE5
GO_bp	GO_RNA_CATABOLIC_PROCESS	391	32	3.808881498348874e-05	0.0013589941268380692	RPL22:TARDBP:EXOSC10:MTOR:CSDE1:SMG5:HSPA8:RNASEH2B:PSME1:ANP32A:SMG1:METTL16:RPL19:PSMD12:RPL38:TNRC6C:DEDD2:PUM2:XPO1:SLC11A1:RPL32:DAZL:RPL14:XRN1:DROSHA:RPL37:SKIV2L2:RPS18:RNASET2:HSPB1:RPL7:RPL12
GO_bp	GO_REGULATION_OF_IMMUNE_RESPONSE	1072	67	3.970363440604436e-05	0.0014097667289102707	CDC42:BCL10:VAV3:NRAS:CTSS:CTSK:DCST1:APCS:FCGR2A:FCGR3A:FCRLB:CFHR5:DMBT1:HPX:NCR3LG1:CRTAM:PTPN6:C1S:NCKAP1L:TNFSF13B:PSME1:COCH:PSEN1:NFATC3:CLEC10A:TBX21:PSMD12:NPLOC4:SERPINB4:MUC16:PKN1:IL12RB1:CD7

						9A:CEACAM1:CD33:FPR2:PUM2:IL1RL1:IL18R1:IL18RAP:SLC11A1:ZBP1:LIME1:TAB1:FOXP1:PROS1:CD200R1:KLHL6:CFI:NPY5R:NDFIP1:HLA-A:FLOT1:MUC21:C4A:CNR1:TNFAIP3:ESR1:LAT2:CD36:PILRB:PILRA:BLK:LYN:SCRIB:UBQLN1:FCN2
GO_bp	GO_RESPONSE_TO_ENDOPLASMIC_RETICULUM_STRESS	272	25	4.084129457495279e-05	0.0014431899765668414	TARDBP:ZBTB17:TXNDC12:EXTL2:LMNA:SYVN1:APAF1:ERO1L:PLA2G4B:SULT1A3:DERL2:TRIM25:RNFT1:NPLOC4:UBXN6:GSK3A:DCTN1:USP25:GSK3B:UBA5:SRPRB:IL8:FLOT1:CREB3L2:UBQLN1
GO_bp	GO_POSITIVE_REGULATION_OF_CELLULAR_COMPONENT_BIOGENESIS	521	39	4.281516133983893e-05	0.001505700649989551	CLSTN1:MTOR:CDC42:TAL1:SEMA4A:ARL2:NCKAP1L:ULK1:LCP1:ERCC5:FAM179B:CORO1A:SLX1A:SMPD3:DHX33:PLEKHM1:NPHS1:DCTN1:CREB1:VIL1:HYAL1:ZMYND10:GSK3B:SLAIN2:DIMT1:UTP15:FCHSD1:EDN1:LRRC16A:FLOT1:ESR1:GTF2H5:THBS2:SEPT7:CD36:TERF1:CDH17:XPA:LRSAM1
GO_bp	GO_SPHINGOLIPID_METABOLIC_PROCESS	80	12	4.33409230232234e-05	0.001516932305812819	CREM:PSAP:SMPD1:PDXDC1:PLA2G15:SMPD3:ARSA:PSAPL1:ITGB8:ESYT2:NSMAF:RP11-203J24.9:ST6GALNAC6
GO_bp	GO_RNA_MODIFICATION	163	18	4.5664013572723635e-05	0.001581386677510715	KTI12:RBM15:NSUN6:ARL5B-AS1:TRUB1:EMG1:MTFMT:METTL16:CTU1:TRMT61B:TPRKB:APOBEC3B:TRMU:THUMPD3:TRMT10A:DIMT1:THG1L:MEPCE:DUS4L
GO_bp	GO_REGULATION_OF_CALCIUM_ION_TRANSPORT	241	23	4.572845395710462e-05	0.001581386677510715	PSEN2:TRPC6:UBASH3B:PTPN6:ORAI1:P2RX2:MYO5A:KIAA1199:CORO1A:RCVRN:CACNB1:STAC2:CACNG1:SEMG1:LIME1:MCHR1:GNAI2:STIM2:DIAPH1:PDGFRB:LYN:GEM:UBQLN1
GO_bp	GO_NEGATIVE_REGULATION_OF_CATALYTIC_ACTIVITY	793	53	4.5827940450310516e-05	0.001581386677510715	SPOCD1:LAMTOR5:APCS:DUSP8:SMPD1:IPO7:ARL2:UBASH3B:PTPN6:GPRC5A:CSRNP2:NCKAP1L:IPO5:PPP1R36:ZFYVE1:PSEN1:PAPLN:ARPP19:CD2BP2:PRPSAP2:MAPK7:SERPINB4:PKN1:GSK3A:CEACAM1:CBLC:CARD8:TSKS:RTKN:VIL1:WFDC5:WFDC12:PI3:SLPI:GNAI2:HYAL2:NPRL2:PROS1:GSK3B:SNCB:C4A:CDKN1A:PI16:CNR1:TNFAIP3:FGFR1OP:HSPB1:YWHAG:PPP1R35:PRKAR2B:LYN:TERF1:KLF4
GO_bp	GO_COGNITION	291	26	4.6738428191945915e-05	0.001605268444910292	MTOR:LCE1D:TACR2:OR52B4:ABCC8:C12orf57:SLC11A2:PDE1B:PSEN1:KCNK10:LINS:GRIN2A:NTAN1:RPS6KB1:INSR:CIC:CREB1:MAPK8IP2:SHANK3:KCNAB1:NIPBL:C5orf20:TIFAB:CNR1:PRKAR2B:CALB1
GO_bp	GO_REGULATION_OF_CELL_ADHESION	676	47	4.7296116686992773e-05	0.0016168672448809158	CDC42:MACF1:BCL10:VAV3:TNFAIP8L2:ADAM15:ABL2:NID1:C10orf54:GPAM:ARL2:UBASH3B:PTPN6:NCKAP1L:NUAK1:POSTN:LMO7:TNFSF13B:PKD1:CORO1A:MAPK7:TBX21:MBP:IL12RB1:CEACAM1:PPP1CB:PKP4:IGFBP2:TGM2:FBLN1:PPARA:PLXNB2:TGFBR2:HYAL1:GSK3B:MFI2:IL8:RELL2:NDFIP1:ADAM19:LRRC16A:FLOT1:MUC21:DACT2:CD36:LYN:KLF4
GO_bp	GO_NEGATIVE_REGULATION_OF_RESPONSE_TO_EXTERNAL_STIMULUS	343	29	4.8323075642267364e-05	0.0016389251216316712	NBL1:CERS2:SEMA6C:TNFAIP8L2:ASH1L:SEMA4A:APCS:ABCC8:UBASH3B:LRFN5:MAPKBP1:MAPK7:NPLOC4:CXCL17:CEACAM1:CARD8:PPARA:PROS1:CD200R1:PYDC2:NPY5R:PTGER4:ISL1:C5orf30:NDFIP1:EDN1:CDKN1A:TNFAIP3:KLF4
GO_bp	GO_SMALL_MOLECULE_CATABOLIC_PROCESS	396	32	4.8610296124585624e-05	0.0016389251216316712	MTOR:DDAH1:ABCD3:ASRGL1:TPI1:DAO:SDS:SDSL:PCK2:SULT1A3:LONP2:PLA2G15:IMPA2:PRODH2:GSK3A:LIPE:PEX13:RPE:APOBEC3B:PPARA:MIOX:TYMP:CPT1B:ENTPD3:ACAD11:GK5:MGAT1:CNR1:FUT9:PEX7:AIG1:CEL
GO_bp	GO_REGULATION_OF_APOPTOTIC_SIGNALING_PATHWAY	396	32	4.8610296124585624e-05	0.0016389251216316712	TXNDC12:BCL10:ARHGEF2:LMNA:ZMYND11:FAS:SYVN1:YAP1:APAF1:PSEN1:MOAP1:RRN3:SIAH1:MAPK7:BRCA1:RPS6KB1:DEDD2:GSK3A:MFF:MAPK8IP2:GNAI2:HYAL2:GSK3B:TERT:TNFAIP3:SOD2:HSPB1:YWHAG:PPP3CC:RB1CC1:UBQLN1:NACC2
GO_bp	GO_REGULATION_OF_PHOSPHORUS_METABOLIC_PROCESS	1677	95	5.066834486609487e-05	0.0017005129441360607	TARDBP:MTOR:SPOCD1:TAL1:BCL10:VAV3:CKS1B:ASH1L:ARHGEF2:SMG5:ZMYND11:GDF2:PSAP:FAS:DUSP8:SMPD1:HPX:IPO7:TRPC6:UBASH3B:CDON:PTPN6:PHB2:GPRC5A:CSRNP2:NCKAP1L:RAP1B:NUAK1:ANKLE2:TNFRSF19:IPO5:PPP1R36:MAP3K9:ZFYVE1:PSEN1:ARPP19:KIAA1199:PKD1:CD2BP2:CCL17:SMPD3:DHX33:NBR1:SPAG9:PPP4R1:PHLPP1:INSR:PKN1:CXCL17:CEACAM1:CBLC:TSKS:FPR2:SPDYA:ALK:FSHR:SLC11A1:CTDSP1:COPS8:LIME1:TAB1:FBLN1:PLXNB2:MAPK8IP2:TGFBR2:GNAI2:HYAL2:NPRL2:GSK3B:DVL3:MAP3K13:AREG:AREGB:NPY5R:ISL1:CCNO:CKS1B:HDAC3:RELL2:PDGFRB:EDN1:FLOT1:CDKN1A:TNFAIP3:FGFR1OP:HSPB1:YWHAG:CD36:PILRB:PPP1R35:PRKAR2B:NCAPG2:GFRA2:RB1CC1:LYN:KLF4:PIP5KL1
GO_bp	GO_RESPONSE_TO_ESTROGEN	70	11	5.780421381760327e-05	0.0019311862343608366	BGLAP:BRCA1:TRIM25:IGFBP2:TGFBR2:SLC34A2:PDGFRB:ESR1:ASH2L:STAR:CA2

GO_bp	GO_REGULATION_OF_LIPID_METABOLIC_PROCESS	383	31	6.142029257689464e-05	0.0020427110879645957	MTOR:LDLRAP1:VAV3:HMGCS2:FDPS:PSAP:GPAM:TM7SF2:DGAT2:LONP2:SMPD3:BRCA1:KPNB1:CEACAM1:FPR2:NCOA1:CREB1:PANK2:EIF6:LSS:PPARA:PSAPL1:PPAP2A:HDAC3:PDGFRB:CNR1:CD36:STAR:LYN:NSMAF:KLF4
GO_bp	GO_STEROL_METABOLIC_PROCESS	96	13	6.337004140234516e-05	0.0020980621815641304	LDLRAP1:SOAT1:SMPD1:DGAT2:FDX1:EBPL:LIPE:APOL2:APOL1:CYP8B1:STAR:CYP11B1:CYP11B2
GO_bp	GO_RESPONSE_TO_TOXIC_SUBSTANCE	513	38	6.65544674120715e-05	0.0021920581056609897	UBIAD1:SZT2:SETDB1:HCN3:BGLAP:PSAP:KCNJ11:TRPC6:PDE1B:SCFD1:CHRNB4:GRIN2A:SMPD3:MAPK7:RNF112:RPS6KB1:MBP:TPO:IL18RAP:CREB1:PCNA:PPARA:XRN1:AREG:AREGB:PDGFRB:EDN1:CDKN1A:GSTA1:CNR1:TNFAIP3:SOD2:INMT:CLDN3:CD36:STAR:LYN:XPA:KLF4
GO_bp	GO_SECOND_MESSENGER_MEDIATED_SIGNALING	439	34	6.680558036300159e-05	0.0021920581056609897	MTOR:LPHN2:DDAH1:SELP:OR5T1:PTHLH:LGR5:P2RX2:PLA2G4B:MYO5A:GRIN2A:SULT1A3:NFATC3:MAPK7:PDE4A:CD97:PTGER1:GSK3A:GRIN2D:FPR2:FSHR:MC3R:ACKR2:GNAI2:GSK3B:ACKR4:IL8:PTGER4:MCTP1:EDN1:LAT2:CD36:PRKAR2B:PPP3CC
GO_bp	GO_PROTEIN_DEPHOSPHORYLATION	315	27	6.878311499344681e-05	0.002246915089785929	MTOR:PTP4A2:DUSP8:SMPD1:UBASH3B:PTPN6:NCKAP1L:NUAK1:ANKLE2:MTMR6:DLGAP5:PPP1R36:ARPP19:PPP4R1:PHLPP1:PPP1CB:DUSP11:CTDSP1:TAB1:GNAI2:GSK3B:PPAP2A:PDGFRB:PTP4A1:PPP1R35:PPP3CC:PTPN3
GO_bp	GO_IMMUNE_SYSTEM_DEVELOPMENT	968	61	6.909745253063451e-05	0.002247195911947627	MTOR:CDC42:MPL:TAL1:RBM15:HIPK1:ARNT:GON4L:SEMA4A:BGLAP:APCS:C10orf54:CTR9:YAP1:UBASH3B:PTPN6:SLC11A2:NCKAP1L:PDE1B:ARL11:PSME1:PSEN1:TCF12:CIAPIN1:SLC7A6OS:SMPD3:IKZF3:TBX21:PSMD12:TNRC6C:PHLPP1:TCF3:PKN1:IL12RB1:CD79A:POU2F2:CEACAM1:KDELR1:TPO:IL18R1:MERTK:CREB1:EIF6:NCAPH2:TGFBR2:HYAL2:TUSC2:FOXP1:DROSHA:PTGER4:NDFIP1:PDGFRB:L3MBTL3:TNFAIP3:DACT2:NCAPG2:ASH2L:LYN:CA2:CDH17:KLF4
GO_bp	GO_POSITIVE_REGULATION_OF_PROTEIN_COMPLEX_ASSEMBLY	248	23	7.10814021841796e-05	0.0023015343879018504	MTOR:TAL1:ARL2:NCKAP1L:LCP1:ERCC5:FAM179B:CORO1A:DHX33:NPHS1:DCTN1:CREB1:VIL1:GSK3B:SLAIN2:FCHSD1:LRRC16A:ESR1:GTF2H5:CD36:TERF1:CDH17:XPA
GO_bp	GO_NEURON_DEVELOPMENT	1094	67	7.276156875962084e-05	0.0023456032034351454	MTOR:NBL1:CDC42:MACF1:CDC20:SZT2:RPE65:OLFM3:PHGDH:CERS2:SEMA6C:SEMA4A:UHMK1:ABL2:CRB1:CDH23:ZFYVE27:APBB1:WEE1:TENM4:TRPC6:OPCML:C12orf57:SLC11A2:NCKAP1L:FBXW8:ULK1:POSTN:NRL:FOXG1:PSEN1:NUMB:TRPM1:SKOR1:RRN3:SIAH1:MAPK7:RND2:RNF157:FSCN2:SKOR2:MEGF8:ALK:BCL11A:CREB1:C21orf91:PLXNB2:MAPK8IP2:SHANK3:TRAK1:CCDC66:GSK3B:DVL3:MAP3K13:AREG:AREGB:ISL1:FLOT1:CPNE5:CNR1:NYAP1:CREB3L2:KEL:GFRA2:LYN:SCRIB:KLF4:PBX3
GO_bp	GO_CELLULAR_RESPONSE_TO_STEROID_HORMONE_STIMULUS	249	23	7.557952826237514e-05	0.002425805819774923	C1orf64:GPAM:SCGB2A1:YAP1:PHB2:PCK2:TGFB1I1:BRCA1:RPS6KB1:SSTR2:SAFB2:SAFB:NCOA1:PPARA:FOXP1:NR1I2:UBA5:EIF4E:ISL1:PPAP2A:EDN1:ESR1:STAR
GO_bp	GO_LIPID_LOCALIZATION	406	32	7.789494726800303e-05	0.0024892515757383576	LDLRAP1:MFSD2A:ABCD3:SOAT1:PSAP:VPS51:DGAT2:LINC01059:GRAMD1B:FITM1:SLC51B:RPS6KB1:PITPNC1:CEACAM1:NCOA1:OSBP2:APOL2:APOL1:PPARA:CPT1B:PLSCR4:ATP10D:SLC10A4:MTTP:PLA2G12A:PRELID2:EDN1:CD36:ABCB1:MEST:ESYT2:STAR:CEL
GO_bp	GO_RESPONSE_TO_PEPTIDE_HORMONE	425	33	8.084991637288073e-05	0.002572497339137114	MTOR:RPE65:PKLR:BGLAP:NUCKS1:ABCC8:UCP2:UCP3:RAP1B:PCK2:MYO5A:TRPV1:GLP2R:RPS6KB1:INSR:GSK3A:CEACAM1:ATP6V1B1:CREB1:IGFBP5:EIF6:PPARA:GNAI2:AREG:AREGB:EDN1:CRHR2:YWHAG:PRKAR2B:STAR:LYN:CA2:CYP11B1:CYP11B2
GO_bp	GO_REGULATION_OF_CELLULAR_PROTEIN_LOCALIZATION	519	38	8.480368637627008e-05	0.0026866685123516595	TARDBP:LDLRAP1:SYT11:LMNA:UHMK1:SEC16B:KCNJ11:CDCA5:SIAH3:LCP1:IPO5:PSEN1:NUMB:SLC51B:KIAA1199:MESDC2:DERL2:STAC2:NPEPPS:AZI1:GSK3A:XPO1:VIL1:MFF:GTSE1:HYAL2:CCDC66:GSK3B:DVL3:AP2M1:TERT:NIPBL:HDAC3:YWHAG:CD36:PPP3CC:TERF1:SCRIB
GO_bp	GO_REGULATION_OF_INTRACELLULAR_ESTROGEN_RECEPTOR_SIGNALING_PATHWAY	39	8	8.536805538400085e-05	0.0026929408028858637	C1orf64:GPAM:YAP1:PHB2:BRCA1:UBA5:ISL1:ESR1
GO_bp	GO_NEGATIVE_REGULATION_OF_CELL_DIFFERENTIATION	733	49	8.691984587306052e-05	0.0027301746460127985	TRIM62:TAL1:FOXE3:RBM15:CERS2:SEMA6C:ARHGEF2:SEMA4A:APCS:ASPM:CTR9:ABCC8:TRPC6:YAP1:UBASH3B:PTHLH:POSTN:FOXG1:PSEN1:SKOR1:ADAMTS7:TGFB1I1:CCL17:NFATC3:TRPV1:NBR1:TBX21:SKOR2:GSK3A:CEACAM1:OSR1:BCL11A:IGFBP5:CTDSP1:FBLN1:PPARA:C3orf17:GSK3B:NPHP3:MFI2:AREG:AREGB:EIF4E:TERT:ISL1:NCAPG2:LYN:S1PR3:PAEP:NELFB

GO_bp	GO_POSITIVE_REGULATION_OF_SMOOTH_MUSCLE_CELL_PROLIFERATION	99	13	8.753968655421839e-05	0.002737943387972362	MTOR:LDLRAP1:SMPD3:RPS6KB1:IGFBP5:TGM2:TGFBR2:GNAI2:FOXP1:NPY5R:TERT:PDGFRB:EDN1
GO_bp	GO_REGULATION_OF_INTRACELLULAR_PROTEIN_TRANSPORT	235	22	8.807570049664585e-05	0.0027430355875014707	TARDBP:UHMK1:SEC16B:SIAH3:LCP1:IPO5:PSEN1:NUMB:SLC51B:KIAA1199:DERL2:NPEPPS:AZI1:GSK3A:XPO1:MFF:GTSE1:HYAL2:GSK3B:HDAC3:CD36:SCRIB
GO_bp	GO_CARBOHYDRATE_DERIVATIVE_BIOSYNTHETIC_PROCESS	655	45	8.94077978158861e-05	0.0027623325034062166	ACOT7:A3GALT2:ELOVL1:EXTL2:AMPD1:SOAT1:FMOD:B3GALNT2:GYLTL1B:SYVN1:TPI1:KDELC1:PSEN1:SLC51B:KIAA1199:ACAN:SMPD3:ASGR2:PRPSAP2:MPPE1:ST8SIA5:MUC16:TECR:CHST8:PANK2:RPN2:UCKL1:ATP5L2:TYMP:TRAK1:HYAL1:TMEM115:DHFRL1:A4GNT:GK5:DCTD:PDGFRB:MGAT1:MUC21:B3GALT4:FUT9:SLC35D2:RP11-203J24.9:ST6GALNAC6:ST6GALNAC4:DPM2
GO_bp	GO_TRNA_MODIFICATION	86	12	8.944695725315367e-05	0.0027623325034062166	KTI12:NSUN6:ARL5B-AS1:TRUB1:MTFMT:CTU1:TRMT61B:TPRKB:TRMU:THUMPD3:TRMT10A:THG1L:DUS4L
GO_bp	GO_LIPOSACCHARIDE_METABOLIC_PROCESS	50	9	9.148647066406877e-05	0.0028018037120465075	CREM:PSAP:SMPD1:SMPD3:ARSA:ITGB8:ESYT2:RP11-203J24.9:ST6GALNAC6:ST6GALNAC4
GO_bp	GO_RESPONSE_TO_EXTRACELLULAR_STIMULUS	521	38	9.182669176404119e-05	0.0028018037120465075	MTOR:SZT2:BCL10:SETDB1:PKLR:BGLAP:FAS:KCNJ11:UCP2:UCP3:HSPA8:APAF1:ULK1:P2RX2:POSTN:PCK2:ZFYVE1:DCTPP1:TRPV1:TRIM25:RPS6KB1:SSTR2:NCOA1:BCL11A:CREB1:IGFBP2:PCNA:PPARA:TGFBR2:GNAI2:NPRL2:SLC6A19:CPEB4:CDKN1A:CNR1:STAR:LYN:SLC25A25
GO_bp	GO_NUCLEAR_TRANSPORT	338	28	9.194203915784001e-05	0.0028018037120465075	TARDBP:LMNA:SMG5:UHMK1:SNRPE:IPO7:DDX25:PHB2:KPNA3:IPO5:PSEN1:CPSF2:ANP32A:PKD1:SMG1:KPNB1:DDX39A:XPO1:EIF6:GTSE1:HYAL2:GSK3B:LSG1:EIF4E:IPO11:HDAC3:CD36:ENY2
GO_bp	GO_REGULATION_OF_CATION_CHANNEL_ACTIVITY	172	18	9.22498637163612e-05	0.0028018037120465075	RNF207:CTSS:KCNJ11:ABCC8:KCNA1:MYO5A:GRIN2A:PIRT:CACNB1:STAC2:KCNS1:MAPK8IP2:SHANK3:KCNAB1:FGF12:STIM2:GEM:UBQLN1
GO_bp	GO_PHASIC_SMOOTH_MUSCLE_CONTRACTION	21	6	9.348211324653164e-05	0.0028275454006666976	TACR2:P2RX2:SSTR2:C5orf20:TIFAB:EDN1
GO_bp	GO_POSITIVE_REGULATION_OF_IMMUNE_SYSTEM_PROCESS	1147	69	9.739731731072059e-05	0.0029338945993188374	CDC42:MPL:TAL1:BCL10:VAV3:NRAS:CTSS:CTSK:ARNT:APCS:FCGR2A:FCGR3A:SELP:CFHR5:C10orf54:GPAM:DMBT1:HPX:CRTAM:PTPN6:C1S:NCKAP1L:TNFSF13B:PSME1:COCH:PSEN1:CORO1A:CLEC10A:TBX21:PSMD12:NPLOC4:CD320:MUC16:IL12RB1:CD79A:CXCL17:CEACAM1:FPR2:PUM2:IL1RL1:IL18R1:IL18RAP:CREB1:IGFBP2:SLC11A1:ZBP1:LIME1:TAB1:TGFBR2:FOXP1:PROS1:KLHL6:IL8:CFI:EDN1:HLA-A:FLOT1:MUC21:C4A:CNR1:TNFAIP3:ESR1:LAT2:CD36:BLK:LYN:CA2:UBQLN1:FCN2
GO_bp	GO_POSITIVE_REGULATION_OF_ORGANELLE_ORGANIZATION	619	43	9.938703434753048e-05	0.0029816110304259143	MTOR:CDC42:TAL1:SETDB1:LMNA:CTR9:ARL2:CDCA5:PHB2:NCKAP1L:ULK1:LCP1:FAM179B:DLGAP5:MOAP1:CORO1A:SLX1A:SMPD3:BRCA1:NPEPPS:INSR:NPHS1:GSK3A:DCTN1:VIL1:MFF:DSTN:ZMYND10:FAM208A:GSK3B:SLAIN2:NIPBL:ISL1:FCHSD1:PDGFRB:EDN1:LRRC16A:C6orf89:SEPT7:YWHAG:PPP3CC:TERF1:LRSAM1
GO_bp	GO_CELLULAR_RESPONSE_TO_DRUG	358	29	0.00010297175674935793	0.003067274759433216	ADAM15:HCN3:ARHGEF2:PSAP:KCNJ11:TRPC6:FDX1:RAP1B:PCK2:SULT1A3:SMPD3:TRPV1:MAPK7:RNF112:BRCA1:RPS6KB1:PDE4A:NCOA1:BCL11A:IL18RAP:PCNA:GNAI2:XRN1:EIF4E:EDN1:TNFAIP3:STAR:CHMP5:KLF4
GO_bp	GO_ANION_TRANSMEMBRANE_TRANSPORT	271	24	0.00010307712456870807	0.003067274759433216	MTOR:LRRC8B:LRRC8C:ABCD3:SLC16A12:SLC43A1:SLC25A3:SLC25A15:PSEN1:SLC7A6:TTYH2:OSR1:SLC4A5:PER2:SLC37A1:APOL1:CPT1B:SLC4A4:SLC6A19:SLC6A18:SLC12A2:CLDN4:ABCB1:SLC35D2
GO_bp	GO_INTRACELLULAR_STEROID_HORMONE_RECEPTOR_SIGNALING_PATHWAY	129	15	0.00010687389600875957	0.0031674319986467053	C1orf64:GPAM:SCGB2A1:YAP1:PHB2:TGFB1I1:BRCA1:SAFB2:SAFB:NCOA1:FOXP1:UBA5:ISL1:PPAP2A:ESR1
GO_bp	GO_ALCOHOL_METABOLIC_PROCESS	222	21	0.00010890793297995088	0.0032147522385648154	LDLRAP1:RPE65:SOAT1:SMPD1:MOGAT2:DGAT2:FDX1:IMPA2:LIPE:PLB1:APOL2:APOL1:MIOX:GK5:COQ2:STAR:RDH10:CYP11B1:CYP11B2:IPPK:DPM2

GO_bp	GO_ANATOMICAL_STRUCTURE_FORMATION_INVOLVED_IN_MORPHOGENESIS	1152	69	0.00011068439735622004	0.003254121282272869	HES2:TIE1:MPL:TAL1:STIL:BCL10:DDAH1:F3:VAV3:RBM15:HIPK1:GJA5:TMOD4:ADAM15:SEMA4A:GDF2:CTR9:ABCC8:TENM4:CDON:PTPN6:HOXC11:APAF1:TNFSF13B:PSEN1:SMPD3:EPN2:MAPK7:HNF1B:ZPBP2:BRCA1:PKN1:NPHS1:CXCL17:CEACAM1:OSR1:NCL:HES6:TGM2:PPARA:CELSR1:PLXNB2:TYMP:TGFBR2:HYAL1:IFT57:NPHP3:KLHL6:DVL3:IL8:TERT:ISL1:C5orf20:TIFAB:PDGFRB:CDX1:EDN1:FLOT1:TNFAIP3:THBS2:ITGB8:HOXA11:HSPB1:CD36:EXOC4:RDH10:CALB1:SCRIB:KLF4
GO_bp	GO_SKIN_DEVELOPMENT	414	32	0.00011189388553555848	0.003276573938989461	CTSK:HRNR:FLG:LCE1F:LCE1E:LCE1D:LCE1C:LCE1B:ASH1L:TMEM79:PSAP:KRTAP5-1:KRTAP5-2:KRTAP5-3:KRTAP5-4:KRTAP5-5:YAP1:HOXC13:LGR5:TNFRSF19:PSEN1:MYO5A:PKD1:CNFN:PKP4:IGFBP5:PI3:KRTAP21-3:KRTAP8-1:CELSR1:DACT2:CLDN4
GO_bp	GO_POSITIVE_REGULATION_OF_CELL_POPULATION_PROLIFERATION	965	60	0.00011377349063232415	0.003318393476776121	MTOR:LDLRAP1:MPL:CDC20:FOXE3:F3:VAV3:WDR77:HIPK1:NRAS:ARNT:ASPM:GDF2:GPAM:GRK5:YAP1:CDON:PTPN6:PTHLH:NCKAP1L:RNASEH2B:TNFSF13B:FOXG1:CORO1A:ESRP2:SMPD3:DERL2:GLP2R:RPS6KB1:AZI1:INSR:CD320:IL12RB1:OSR1:SPDYA:IGFBP2:IGFBP5:TGM2:TGFBR2:SCN5A:GNAI2:HYAL1:FOXP1:CNBP:AREG:AREGB:PLAC8:NPY5R:TERT:ISL1:CCNO:PDGFRB:EDN1:CDKN1A:TNFAIP3:ESR1:FGFR1OP:ASH2L:LYN:S1PR3:NACC2
GO_bp	GO_CELLULAR_LIPID_CATABOLIC_PROCESS	207	20	0.00011784344793341564	0.0034077262518616863	MTOR:ABCD3:PSAP:SMPD1:PCK2:PLA2G4B:PDXDC1:LONP2:PLA2G15:SMPD3:LIPE:PEX13:NAGA:PPARA:CPT1B:ACAD11:CNR1:PEX7:AIG1:CEL
GO_bp	GO_REGULATION_OF_SMALL_MOLECULE_METABOLIC_PROCESS	343	28	0.00011801091306003229	0.0034077262518616863	MTOR:LDLRAP1:CLK2:FDPS:GPAM:TM7SF2:DGAT2:PSME1:ARPP19:LONP2:BRCA1:KPNB1:PSMD12:INSR:CD320:GSK3A:CEACAM1:PPP1CB:IGFBP5:PANK2:EIF6:LSS:PPARA:GSK3B:CNR1:FAM3C:STAR:RDH10
GO_bp	GO_MULTICELLULAR_ORGANISM_REPRODUCTION	823	53	0.00011822723730948708	0.0034077262518616863	MTOR:PHC2:HOOK1:HORMAD1:SMCP:ASH1L:ASPM:TUBB8:CREM:DDX25:LGR5:FNDC3A:TDRD9:HERC2:PRSS21:SIAH1:PRMT7:LYZL6:ZPBP2:SPATA32:RPS6KB1:SSTR2:AZI1:TAF4B:PAFAH1B3:NCOA1:SPDYA:FSHR:MERTK:PANK2:PCNA:SEMG1:SEMG2:OSBP2:APOL2:NCAPH2:SYCE3:MAPK8IP2:DAZL:HYAL3:NPY5R:PPAP2A:SOX30:EDN1:MEI4:CNR1:ESR1:SYNE1:HOXA10:HOXA11:SEPT7:SPATA31A4:SPATA31A7:PAEP
GO_bp	GO_DEPHOSPHORYLATION	471	35	0.00011911009905041769	0.003419762609455352	MTOR:SPOCD1:PTP4A2:PRUNE:SMG5:DUSP8:SMPD1:UBASH3B:PTPN6:CSRNP2:NCKAP1L:NUAK1:ANKLE2:MTMR6:DLGAP5:PPP1R36:ZFYVE1:ARPP19:CD2BP2:PPP4R1:IMPA2:PHLPP1:TSKS:PPP1CB:DUSP11:CTDSP1:TAB1:GNAI2:GSK3B:PPAP2A:PDGFRB:PTP4A1:PPP1R35:PPP3CC:PTPN3
GO_bp	GO_FATTY_ACID_METABOLIC_PROCESS	257	23	0.00012173295411292089	0.0034814677538131072	MTOR:ELOVL1:ACOT11:ABCD3:CREM:GPAM:UCP3:DGAT2:PLA2G4B:LONP2:PEX13:ATP6V1B1:PER2:PANK2:CYP2D6:PPARA:CPT1B:ACAD11:GSTA1:CNR1:PEX7:CD36:PRKAR2B
GO_bp	GO_NEGATIVE_REGULATION_OF_SECRETION	224	21	0.0001235970061765345	0.0035210775015408085	SYT11:TACR2:KCNJ11:ABCC8:UCP2:RAP1B:STXBP6:MAPKBP1:CEACAM1:CARD8:GNAI2:CD200R1:PYDC2:NPY5R:PTGER4:C5orf20:TIFAB:EDN1:CNR1:TNFAIP3:ENY2
GO_bp	GO_ESTABLISHMENT_OF_ORGANELLE_LOCALIZATION	491	36	0.00012514145585501945	0.0035422864623127184	CDC42:SYT11:ARHGEF2:F5:SEC16B:CDCA5:RAP1B:SCFD1:DLGAP5:PSEN1:MYO5A:TRAPPC2L:KPNB1:AP1M2:GIPC1:XPO1:DCTN1:TMEM230:CHMP4B:EIF6:DNAJC5:ARFGAP3:TRAK1:STX19:GSK3B:AREG:AREGB:CNR1:LAT2:BET1:COPG2:EXOC4:LYN:TERF1:GEM:SCRIB:CHMP5
GO_bp	GO_CARBOHYDRATE_BIOSYNTHETIC_PROCESS	208	20	0.00012585295159174337	0.0035422864623127184	MTOR:CLK2:PC:DGAT2:TPI1:ENO2:SDS:PCK2:ARPP19:SMPD3:IMPA2:INSR:CHST8:GSK3A:PPP1CB:PER2:PPARA:GSK3B:FAM3C:RP11-203J24.9:ST6GALNAC6
GO_bp	GO_RRNA_METABOLIC_PROCESS	208	20	0.00012585295159174337	0.0035422864623127184	EXOSC10:EBNA1BP2:RPF1:EMG1:DDX47:DHX37:METTL16:UTP18:NOL10:TRMT61B:ESF1:DDX27:RPL14:SHQ1:SKIV2L2:DIMT1:UTP15:WDR46:GTF2H5:RPL7
GO_bp	GO_REGULATION_OF_CELLULAR_CATABOLIC_PROCESS	805	52	0.00012660910018669755	0.0035422864623127184	TARDBP:MTOR:LAMTOR5:ARNT:UBQLN4:ABL2:F13B:PSAP:HSPA8:USP5:APAF1:FBXW8:PRKAB1:ULK1:PSME1:SCFD1:PSEN1:ANP32A:PKD1:LONP2:N4BP1:METTL16:DERL2:RNFT1:PSMD12:TNRC6C:INSR:KEAP1:GIPC1:GSK3A:PUM2:PPP1CB:XPO1:ATP6V1B1:SLC11A1:CHMP4B:USP25:PPARA:DAZL:NPRL2:GSK3B:XRN1:CNR1:TNFAIP3:HSPB1:EXOC4:RB1CC1:NRBP2:UBQLN1:XPA:PTPN3:LRSAM1
GO_bp	GO_PROTEIN_CATABOLIC_PROCESS	907	57	0.00012675120266506735	0.0035422864623127184	MTOR:CDC20:CTSS:CTSK:DCST1:UBQLN4:F13B:CUL2:UBE2L6:PGA3:SYVN1:USP5:SPSB2:FBXW8:SIAH3:UCHL3:ABHD13:DCAF11:PSME1:FBXO33:PSEN1:HERC2:OTUD7A:ADAMTS7:PKD1:GRIN2A:NTAN1:TGFB1I1:LONP2:SIAH1:N4BP1:DERL2:TBX21:TRIM25:RNFT1:PSMD12:NPLOC4:UBXN6:KEAP1:GIPC1:GSK3A:CEACAM1:CBLC:C19orf68:XPO1:ANAPC1:CHMP4B:USP25:GSK3B:ARMC8:AP2M1:NDFIP1:PRSS16:TNFAIP3:UBQLN1:PTPN3:LRSAM1
GO_bp	GO_POSITIVE_REGULATION_OF_CELL_DIFFERENTIATION	970	60	0.00013069590680761558	0.003638692859984752	MTOR:SPEN:NBL1:CDC42:MACF1:MPL:TAL1:ARNT:ARHGEF2:ABL2:ASPM:GDF2:C10orf54:ZFYVE27:APBB1:TENM4:TRPC6:CDON:SOX5:NCKAP1L:FBXW8:FOXG1:PSEN1:NUMB:TCF12:SH3GL3:RRN3:TGFB1I1:RNF112:RND2:SPAG9:RNF157:TCF3:IL12RB

						1:MEGF8:CEACAM1:NCOA1:CREB1:C21orf91:PLXNB2:SHANK3:TGFBR2:TRAK1:DVL3:MAP3K13:TERT:NIPBL:ISL1:EDN1:LRRC16A:FLOT1:CPNE5:CNR1:SOD2:HOXA11:CD36:CREB3L2:STAR:LYN:CA2
GO_bp	GO_ENDOPLASMIC_RETICULUM_TO_GOLGI_VESICLE_MEDIATED_TRANSPORT	209	20	0.0001343391079942309	0.003726009221726782	BGLAP:F5:SEC16B:SCFD1:MIA2:CTAGE5:TRAPPC2L:MPPE1:KDELR1:DCTN1:ARFGAP3:TMEM115:PROS1:RAB43:COPB2:AREG:AREGB:BET1:BCAP29:COPG2:CREB3L2:NRBP2
GO_bp	GO_REGULATION_OF_CELL_MORPHOGENESIS	475	35	0.0001402501242150163	0.0038753323796254504	FBLIM1:CDC42:MACF1:MPL:SEMA6C:SEMA4A:ZFYVE27:TRPC6:FBXW8:POSTN:COCH:PSEN1:CORO1A:RND2:RNF157:ARHGEF18:MEGF8:ZNF135:BCL11A:RND3:VIL1:FBLN1:PLXNB2:SHANK3:TRAK1:GSK3B:DVL3:MAP3K13:MFI2:DIAPH1:LRRC16A:CPNE5:SEPT7:KEL:PALM2
GO_bp	GO_APOPTOTIC_SIGNALING_PATHWAY	591	41	0.00014671837064539833	0.004038876495294674	TXNDC12:TM2D1:BCL10:HIPK1:DAP3:ARHGEF2:LMNA:PHLDA3:ZMYND11:CUL2:FAS:SYVN1:YAP1:DDX47:APAF1:ERO1L:PSEN1:MOAP1:RRN3:SIAH1:MAPK7:BRCA1:RPS6KB1:DEDD2:GSK3A:MFF:MAPK8IP2:GNAI2:HYAL2:GSK3B:TERT:CDKN1A:TNFAIP3:SOD2:HSPB1:YWHAG:PPP3CC:RB1CC1:UBQLN1:XPA:NACC2
GO_bp	GO_NEGATIVE_REGULATION_OF_PROTEIN_METABOLIC_PROCESS	1123	67	0.00015478666172185317	0.004245081953938884	TARDBP:MTOR:LAMTOR5:ASH1L:APCS:F13B:ZMYND11:DUSP8:SMPD1:IPO7:UBASH3B:PTPN6:GPRC5A:NCKAP1L:IPO5:PPP1R36:PSEN1:PAPLN:ARPP19:N4BP1:METTL16:DERL2:BRCA1:TNRC6C:SERPINB4:PKN1:GIPC1:CEACAM1:CBLC:CARD8:IGFBP5:CTDSP1:VIL1:TM4SF20:NCL:PER2:EIF6:WFDC5:WFDC12:PI3:SLPI:USP25:FBLN1:GNAI2:HYAL2:PROS1:GSK3B:XRN1:SENP2:EIF4E:HDAC3:CPEB4:EDN1:C4A:CDKN1A:PI16:TNFAIP3:FGFR1OP:HSPB1:YWHAG:PPP1R35:PRKAR2B:GFRA2:LYN:KLF4:PTPN3:PIP5KL1
GO_bp	GO_ENDOMEMBRANE_SYSTEM_ORGANIZATION	422	32	0.00015869250322399545	0.004336021928239281	TARDBP:CDC42:HOOK1:GOLPH3L:SYT11:LMNA:SEC16B:CRB1:RAB18:REEP3:PSAP:ZFYVE27:VPS51:FBXW8:CIT:ANKLE2:GOLGA8G:GOLGA8J:GOLGA8R:GOLGA8H:ZPBP2:NPLOC4:DCTN1:CREB1:CHMP4B:SYNGR1:FAM109B:RAB43:PLSCR4:FLOT1:SYNE1:CHMP5
GO_bp	GO_STEROID_METABOLIC_PROCESS	314	26	0.00016211002211677974	0.004412995046512337	LDLRAP1:HMGCS2:FDPS:SOAT1:GPAM:SMPD1:TM7SF2:DGAT2:FDX1:EBPL:SULT1A3:KPNB1:LIPE:PANK2:LSS:APOL2:APOL1:CYP2D6:CYB5R3:CYP8B1:NR1I2:CNBP:ESR1:STAR:CYP11B1:CYP11B2
GO_bp	GO_REPRODUCTIVE_SYSTEM_DEVELOPMENT	423	32	0.00016563278567603508	0.0044839830792532415	RBM15:WDR77:CSDE1:ARNT:ADAM15:ASH1L:ASPM:PSAP:FBXW8:FNDC3A:PKD1:SAFB2:INSR:OSR1:NCOA1:FSHR:MERTK:RTCB:HYAL3:PSAPL1:NIPBL:C5orf20:TIFAB:PDGFRB:ADAM19:ESR1:ITGB8:HOXA10:HOXA11:STAR:RDH10:LRRC6
GO_bp	GO_POSITIVE_REGULATION_OF_LOCOMOTION	575	40	0.00016593787721862338	0.0044839830792532415	MTOR:F3:SEMA6C:ARHGEF2:SEMA4A:SELP:TACR2:C10orf54:PC:NCKAP1L:POSTN:NUMB:KIAA1199:CORO1A:SPAG9:RPS6KB1:INSR:MEGF8:CXCL17:FPR2:IGFBP5:VIL1:CHMP4B:GTSE1:TGFBR2:GNAI2:HYAL1:FOXP1:IL8:TERT:NIPBL:DIAPH1:PDGFRB:EDN1:LRRC16A:PTP4A1:SOD2:FGFR1OP:HSPB1:LYN
GO_bp	GO_ENDOCYTOSIS	634	43	0.00016789863135422661	0.004520347767229178	CDC42:LDLRAP1:TSPAN1:SYT11:PEAR1:ABL2:DMBT1:HPX:NCKAP1L:HEATR5A:NUMB:SH3GL3:RABEP1:CLEC10A:ASGR2:EPN2:RALBP1:SH3GL1:DENND1C:INSR:EPS15L1:CEACAM1:FPR2:APOL1:TGFBR2:ACKR2:ACKR4:AP2M1:IL8:CFI:MCTP1:MARCH3:MEGF10:SNCB:LRRC16A:FLOT1:CD36:ESYT2:PPP3CC:SCRIB:LRSAM1:CEL:FCN2
GO_bp	GO_AGING	315	26	0.0001705062144633956	0.004573798088707875	MTOR:LMNA:BGLAP:UCP2:UCP3:TRPC6:APAF1:NUAK1:FOXG1:ERO1L:PSEN1:RPS6KB1:CREB1:IGFBP2:IGFBP5:RPN2:TGFBR2:HYAL2:NPY5R:TERT:SLC12A2:PDGFRB:EDN1:CDKN1A:CNR1:SOD2
GO_bp	GO_REGULATION_OF_ANATOMICAL_STRUCTURE_SIZE	499	36	0.00017160155640847855	0.004586441598553881	MTOR:MACF1:LRRC8B:LRRC8C:VAV3:LAMTOR5:GJA5:SEMA6C:TMOD4:SEMA4A:ACTA2:ZFYVE27:NCKAP1L:CIT:CORO1A:HNF1B:RND2:PEX11G:NPHS1:MEGF8:CREB1:VIL1:PER2:DSTN:SHANK3:NAT6:GSK3B:AP2M1:MAP3K13:SLC12A2:FCHSD1:EDN1:LRRC16A:SOD2:KEL:RB1CC1
GO_bp	GO_SMOOTH_MUSCLE_CELL_PROLIFERATION	165	17	0.00017270873352740493	0.004599308664588501	MTOR:LDLRAP1:SMPD3:RPS6KB1:IGFBP5:TGM2:TGFBR2:GNAI2:FOXP1:NPY5R:TERT:PDGFRB:EDN1:CDKN1A:TNFAIP3:SOD2:KLF4
GO_bp	GO_POSITIVE_REGULATION_OF_DEVELOPMENTAL_GROWTH	181	18	0.000176504579833412	0.00468342477175299	MTOR:MACF1:ZFYVE27:GPAM:YAP1:RND2:RPS6KB1:RNF157:INSR:MEGF8:CREB1:VIL1:TGFBR2:CACNA2D2:MAP3K13:NIPBL:EDN1:CPNE5
GO_bp	GO_REGULATION_OF_RESPONSE_TO_WOUNDING	166	17	0.00018578512813088638	0.0049119449343957376	MTOR:F3:CERS2:APCS:SELP:ABCC8:UBASH3B:CEACAM1:VIL1:TGFBR2:PROS1:EDN1:CDKN1A:TNFAIP3:CD36:LYN:KLF4

GO_bp	GO_PROTEIN_COMPLEX_OLIGOMERIZATION	559	39	0.00018761307361128958	0.004942494949974834	BCL10:LRRC8C:KCNC4:GJA5:SYT11:KCNA1:APAF1:PRKAB1:TMEM120B:P2RX2:KCTD12:TRPM1:CHRNB4:PKD1:DCTPP1:SMPD3:TRAPPC2L:TRPV1:NBR1:SPAG9:KCNJ2:INSR:CD79A:TRMT61B:MFF:CHMP4B:TGM2:KCNS1:SEMG1:SEMG2:THG1L:FLOT1:TNFAIP3:SOD2:SEPT7:CLDN3:KHDRBS3:KCNV2:NACC2
GO_bp	GO_RNA_METABOLIC_PROCESS	1542	86	0.00019131821517014751	0.005020243285581659	TARDBP:EXOSC10:EBNA1BP2:FOXE3:KTI12:RPF1:PTBP2:RBM15:WDR77:BCAS2:ARNT:SCNM1:CLK2:KIAA0907:SNRPE:NSUN6:ARL5B-AS1:RBM20:TRUB1:CTR9:RBM7:REXO2:HSPA8:EMG1:DDX47:TFCP2:DHX37:WBP4:RNASEH2B:DTD2:PRPF39:RBM25:CPSF2:TDRD9:AQR:MTFMT:TARSL2:CD2BP2:POLR2C:ESRP2:PRMT7:METTL16:UTP18:SAFB2:SAFB:DDX39A:SARS2:DEDD2:CTU1:NOL10:MYCN:PUM2:TRMT61B:ZNF638:TPRKB:DUSP11:RBMS1:ESF1:DDX27:RTCB:APOBEC3B:TRMU:THUMPD3:RPL14:SHQ1:ISY1:DBR1:XRN1:TRMT10A:TERT:DROSHA:SKIV2L2:DIMT1:UTP15:THG1L:LSM11:RPP40:WDR46:PPIL1:ESR1:GTF2H5:RNASET2:MEPCE:DUS4L:RPL7:KHDRBS3:PUF60
GO_bp	GO_NEGATIVE_REGULATION_OF_INFLAMMATORY_RESPONSE	151	16	0.00019193038955761174	0.005020243285581659	TNFAIP8L2:ASH1L:APCS:LRFN5:MAPK7:CXCL17:PPARA:CD200R1:PYDC2:NPY5R:PTGER4:ISL1:C5orf30:NDFIP1:TNFAIP3:KLF4
GO_bp	GO_REGULATION_OF_WOUND_HEALING	136	15	0.00019354917843505915	0.0050446328421903715	MTOR:F3:APCS:SELP:ABCC8:UBASH3B:CEACAM1:VIL1:TGFBR2:PROS1:EDN1:CDKN1A:TNFAIP3:CD36:LYN
GO_bp	GO_MRNA_METABOLIC_PROCESS	560	39	0.00019452432891407085	0.00505213363080714	TARDBP:EXOSC10:FOXE3:PTBP2:RBM15:WDR77:BCAS2:ARNT:SCNM1:KIAA0907:SNRPE:RBM20:CTR9:RBM7:HSPA8:DDX47:TFCP2:WBP4:PRPF39:RBM25:CPSF2:AQR:CD2BP2:POLR2C:ESRP2:PRMT7:METTL16:SAFB2:SAFB:DDX39A:ISY1:DBR1:XRN1:SKIV2L2:LSM11:PPIL1:GTF2H5:KHDRBS3:PUF60
GO_bp	GO_REGULATION_OF_CYTOSKELETON_ORGANIZATION	522	37	0.00019898640043118038	0.005149824095666112	MTOR:CDC42:STIL:PRUNE:TMOD4:ARHGEF2:ABL2:ARL2:NCKAP1L:CIT:FAM179B:PKD1:CORO1A:RND2:BRCA1:AZI1:TUBB4A:NPHS1:XPO1:DCTN1:RND3:VIL1:DSTN:CHMP4B:CELSR1:SHANK3:NAT6:RASSF1:GSK3B:SLAIN2:PTGER4:DIAPH1:FCHSD1:PDGFRB:EDN1:LRRC16A:CHMP5
GO_bp	GO_HORMONE_MEDIATED_SIGNALING_PATHWAY	232	21	0.00020139116130997147	0.005177031228572884	C1orf64:GPAM:SCGB2A1:YAP1:PHB2:LGR5:TGFB1I1:BRCA1:SSTR2:SAFB2:SAFB:NCOA1:FSHR:PPARA:FOXP1:NR1I2:UBA5:ISL1:PPAP2A:ESR1:CRHR2
GO_bp	GO_REGULATION_OF_SYSTEM_PROCESS	600	41	0.00020144638522065917	0.005177031228572884	RNF207:MTOR:GJA5:LMNA:TACR2:KCNJ11:ABCC8:TENM4:KCNA1:C12orf57:CHRNB4:GRIN2A:TRPV1:CACNB1:GJC1:RPS6KB1:CACNG1:KCNJ2:SSTR2:SH3GL1:PTGER1:NPHS1:GSK3A:GRIN2D:KCNJ14:IGFBP5:PER2:MC3R:PPARA:TYMP:SHANK3:SCN5A:GNAI2:CACNA2D2:FGF12:C5orf20:TIFAB:KCNIP1:EDN1:KLF4:PBX3
GO_bp	GO_INNATE_IMMUNE_RESPONSE	966	59	0.00020622685516170106	0.0052641381842277206	TRIM62:BCL10:NRAS:CTSS:CTSK:TNFAIP8L2:DCST1:ADAM15:ARHGEF2:APCS:CFHR5:DMBT1:HPX:IPO7:CRTAM:PTPN6:C1S:PSME1:COCH:CORO1A:CCL17:CLEC10A:IFI35:TBKBP1:TRIM25:RPS6KB1:PSMD12:NPLOC4:SERPINB4:MUC16:IL12RB1:CEACAM1:PUM2:IL18RAP:SLC11A1:SLPI:ZBP1:APOL1:APOBEC3B:TAB1:RAB43:PYDC2:CFI:WRNIP1:EDN1:HLA-A:TUBB:FLOT1:MUC21:C4A:TNFAIP3:ESR1:CD36:STAR:LYN:SCRIB:UBQLN1:FCN2:TUBB4B
GO_bp	GO_MONOCARBOXYLIC_ACID_CATABOLIC_PROCESS	122	14	0.00020626827170851475	0.0052641381842277206	MTOR:ABCD3:PCK2:LONP2:PLA2G15:LIPE:PEX13:PPARA:CPT1B:ACAD11:CNR1:PEX7:AIG1:CEL
GO_bp	GO_REGULATION_OF_GROWTH	680	45	0.0002072677317329578	0.005271341966218823	CLSTN1:MTOR:CDC42:MACF1:SEMA6C:ADAM15:SEMA4A:ARHGEF11:GDF2:TLL2:ZFYVE27:GPAM:APBB1:YAP1:DERL2:HNF1B:RND2:RPS6KB1:RNF157:SAFB:INSR:GSK3A:MEGF8:CEACAM1:BCL11A:CREB1:IGFBP2:IGFBP5:VIL1:PPARA:TGFBR2:HYAL1:HYAL2:CACNA2D2:GSK3B:MAP3K13:PLAC8:NPY1R:NIPBL:EDN1:CDKN1A:CPNE5:FGFR1OP:CD36:NAIF1
GO_bp	GO_CIRCULATORY_SYSTEM_DEVELOPMENT	1136	67	0.00021378683261337336	0.005418390412787222	RNF207:MTOR:TIE1:TAL1:STIL:DDAH1:F3:VAV3:RBM15:HIPK1:GJA5:ADAM15:LMNA:SEMA4A:GDF2:ACTA2:RBM20:ABCC8:TENM4:YAP1:FBXW8:PSEN1:ACAN:PKD1:NFATC3:EPN2:B9D1:MAPK7:BRCA1:GJC1:INSR:GSK3A:MEGF8:CXCL17:CEACAM1:OSR1:CREB1:NCL:PCNA:TAB1:PPARA:TYMP:TGFBR2:SCN5A:HYAL1:ARL13B:IFT57:NPHP3:KCNAB1:FGF12:IL8:NPY1R:NPY5R:TERT:NIPBL:ISL1:PDGFRB:EDN1:CDKN1A:POPDC3:TNFAIP3:SOD2:THBS2:ITGB8:HSPB1:RB1CC1:KLF4
GO_bp	GO_PLATELET_ACTIVATION	153	16	0.00022339348776946523	0.005642412835414327	MPL:VAV3:PEAR1:SELP:CSRP1:TRPC6:UBASH3B:PTPN6:DGKE:MYL12A:CEACAM1:MERTK:GP9:PF4V1:HSPB1:LYN

GO_bp	GO_REGULATION_OF_CELL_CYCLE_PHASE_TRANSITION	468	34	0.00022547482080882946	0.005675479222414029	CDC20:WEE1:CDCA5:PTPN6:PHB2:RNASEH2B:PSME1:DLGAP5:PKD1:BRCA1:PSMD12:AZI1:TUBB4A:DCTN1:ANAPC1:CTDSP1:PCNA:GTSE1:HYAL1:NEK11:TERT:LSM11:TUBB:CDKN1A:FGFR1OP:SEPT7:YWHAG:MEPCE:PRKAR2B:CEP41:GML:KLF4:NACC2:TUBB4B
GO_bp	GO_QUINONE_BIOSYNTHETIC_PROCESS	16	5	0.00023083830178295803	0.005790653645408674	UBIAD1:ADCK3:NDUFA9:COQ9:COQ2
GO_bp	GO_ANATOMICAL_STRUCTURE_HOMEOSTASIS	432	32	0.00024142344115028345	0.006035586028757086	EXOSC10:RPE65:CTSK:BGLAP:SMG5:CDH23:GYLTL1B:YAP1:UBASH3B:POLE:SMG1:CORO1A:SLX1A:CALB2:ANKRD11:PLEKHM1:DCTN1:POTEE:PCNA:CHMP4B:CCDC66:NPHP3:XRN1:TERT:CNR1:TNFAIP3:RFC2:HSPB1:PIP:TERF1:CA2:CALB1
GO_bp	GO_REGULATION_OF_TRANSPORTER_ACTIVITY	270	23	0.0002510306210301899	0.006254491744311511	RNF207:CTSS:KCNJ11:ABCC8:TRPC6:KCNA1:MYO5A:GRIN2A:PIRT:CACNB1:STAC2:OSR1:KCNS1:MAPK8IP2:SHANK3:KCNAB1:FGF12:STIM2:NDFIP1:ABCB1:GEM:UBQLN1:PTPN3
GO_bp	GO_REGULATION_OF_PROTEIN_STABILITY	273	23	0.00029418960451040943	0.007305045922809153	TSPAN1:LMNA:SYVN1:HSPA8:PHB2:SIAH3:SLC51B:SIAH1:ASGR2:NAPG:GIPC1:CREB1:USP25:LSS:GTSE1:DVL3:SENP2:AFM:TERT:HDAC3:FLOT1:CDKN1A:DPM2
GO_bp	GO_PLATELET_AGGREGATION	58	9	0.0002970939127112832	0.007327651873919233	MPL:PEAR1:CSRP1:UBASH3B:PTPN6:MYL12A:CEACAM1:HSPB1:LYN
GO_bp	GO_INTRACELLULAR_ESTROGEN_RECEPTOR_SIGNALING_PATHWAY	58	9	0.0002970939127112832	0.007327651873919233	C1orf64:GPAM:YAP1:PHB2:BRCA1:SAFB:UBA5:ISL1:ESR1
GO_bp	GO_MULTI_MULTICELLULAR_ORGANISM_PROCESS	222	20	0.00030037135261661165	0.0073837105074652035	MTOR:OVGP1:SMCP:ASH1L:ABCC8:UCP2:PTHLH:PLA2G4B:IGFBP2:IGFBP5:SEMG1:APOL2:FBLN1:TGFBR2:HYAL3:EDN1:CNR1:ESR1:CLDN4:PAPPA
GO_bp	GO_RESPONSE_TO_INORGANIC_SUBSTANCE	553	38	0.0003033860186493404	0.007432957456908841	A3GALT2:SZT2:LCE1D:PKLR:SYT11:BGLAP:PSAP:APBB1:ABCC8:SYT13:UCP2:UCP3:TRPC6:KCNA1:SLC11A2:SMPD3:MAPK7:RNF112:IL18RAP:CREB1:IGFBP2:PCNA:SCN5A:ARL13B:AREG:AREGB:TERT:PDGFRB:SNCB:EDN1:CPNE5:TNFAIP3:SOD2:CD36:STAR:CA2:CYP11B1:CYP11B2:KLF4
GO_bp	GO_REGULATION_OF_HYDROLASE_ACTIVITY	1261	72	0.00032368496556645174	0.007903935205692426	MTOR:SPOCD1:DNAJB4:BCL10:F3:VAV3:LAMTOR5:ARHGEF11:APCS:ABL2:AGAP4:AGAP8:C10orf54:PSAP:FAS:ARL2:CSRNP2:NCKAP1L:APAF1:NUAK1:ANKLE2:IPO5:PSME1:PPP1R36:ZFYVE1:PAPLN:ARPP19:GRIN2A:CD2BP2:TBC1D10B:CCL17:RABEP1:MAPK7:RALBP1:PPP4R1:SERPINB4:MBP:DNAJB1:CARD8:TSKS:RTKN:PKP4:VIL1:PCNA:RALGAPA2:WFDC5:WFDC12:PI3:SEMG1:SEMG2:SLPI:ARFGAP3:FBLN1:PLXNB2:GNAI2:NPRL2:PROS1:IFT57:ADPRH:GSK3B:RASA2:DVL3:ARAP3:PDGFRB:C4A:C6orf89:PI16:ESR1:PPP1R35:LYN:TERF1:SCRIB:KLF4
GO_bp	GO_NEGATIVE_REGULATION_OF_TRANSFERASE_ACTIVITY	275	23	0.0003264670139790365	0.007945472028959994	DUSP8:SMPD1:IPO7:UBASH3B:PTPN6:GPRC5A:IPO5:PSEN1:PKN1:GSK3A:CEACAM1:CBLC:HYAL2:NPRL2:GSK3B:CDKN1A:TNFAIP3:FGFR1OP:HSPB1:YWHAG:PRKAR2B:LYN:TERF1
GO_bp	GO_SECONDARY_ALCOHOL_METABOLIC_PROCESS	85	11	0.0003412813774015883	0.008251375407571296	LDLRAP1:SOAT1:SMPD1:DGAT2:FDX1:LIPE:APOL2:APOL1:STAR:CYP11B1:CYP11B2
GO_bp	GO_RETROGRADE_TRANSPORT_ENDOSOME_TO_GOLGI	85	11	0.0003412813774015883	0.008251375407571296	VPS51:ERC1:HEATR5A:TBC1D10B:SPAG9:DCTN1:TMEM87B:FAM109B:RAB6B:VPS52:DENND2A
GO_bp	GO_CELLULAR_COMPONENT_DISASSEMBLY	537	37	0.0003436080841646112	0.008280391536425876	DNASE2B:CTSS:CTSK:TMOD4:ADAM15:DAP3:ARHGEF2:UBQLN4:TLL2:ABCC8:MRPL49:HSPA8:APAF1:MTIF3:LCP1:KPNB1:KIF19:EPG5:SH3GL1:INSR:MRPS12:DEDD2:GSK3A:GRWD1:DCTN1:VIL1:DSTN:GSK3B:MRPS22:DVL3:MFI2:KIF2A:LRRC16A:FLOT1:RB1CC1:CHMP5:UBQLN1
GO_bp	GO_PERISTALSIS	10	4	0.0003490242137353736	0.008360600780382558	P2RX2:SSTR2:C5orf20:TIFAB

GO_bp	GO_POSITIVE_REGULATION_OF_TRANSCRIPTION_BY_RNA_POLYMERASE_II	1178	68	0.0003492114883778837	0.008360600780382558	TARDBP:ZBTB17:TAL1:FOXD2:RBM15:ARNT:ASH1L:ARHGEF2:C1orf85:NHLH1:NUCKS1:CREM:GDF2:APBB1:ZNF143:CTR9:EHF:YAP1:POU2AF1:CDON:SOX5:CSRNP2:TFCP2:HOXC13:HOXC11:LMO7:NRL:DLGAP5:SIX6:TCF12:PKD1:NFATC3:MAPK7:HNF1B:IKZF3:BRCA1:TAF4B:ASXL3:TCF3:SAFB:POU2F2:MYCN:OSR1:NCOA1:CREB1:SLC11A1:NCL:PPARA:HYAL2:NR1I2:CNBP:PLAC8:TERT:NIPBL:ISL1:MCIDAS:HDAC3:CDX1:EDN1:ESR1:HOXA10:EVX1:NEUROD6:KLF14:CREB3L2:ASH2L:KLF4:PBX3
GO_bp	GO_EMBRYONIC_HEART_TUBE_DEVELOPMENT	72	10	0.0003530378226946938	0.008424766223396102	RNF207:STIL:GJA5:YAP1:PSEN1:MEGF8:TGFBR2:ARL13B:IFT57:NPHP3
GO_bp	GO_RESPONSE_TO_REACTIVE_OXYGEN_SPECIES	225	20	0.00035774643537782194	0.008509502589084115	SZT2:PSAP:UCP2:UCP3:TRPC6:SMPD3:MAPK7:RNF112:IL18RAP:PCNA:HYAL1:HYAL2:AREG:AREGB:PDGFRB:EDN1:TNFAIP3:SOD2:CD36:STAR:KLF4
GO_bp	GO_REGULATION_OF_CELLULAR_RESPONSE_TO_STRESS	718	46	0.00036171088093872887	0.00855060556365763	MTOR:SZT2:TXNDC12:DDAH1:CERS2:ARHGEF2:ZMYND11:PSAP:FAS:APBB1:SYVN1:HSPA8:NUAK1:TNFRSF19:RNASEH2B:MAP3K9:SMG1:DERL2:MAPK7:BRCA1:NBR1:SPAG9:RNFT1:PHLPP1:PKN1:DNAJB1:PCNA:USP25:MAPK8IP2:GSK3B:DVL3:MAP3K13:TERT:HDAC3:RELL2:WRNIP1:EDN1:IER3:SOD2:HSPB1:CD36:RB1CC1:LYN:UBQLN1:KLF4:NACC2
GO_bp	GO_INTRACELLULAR_RECEPTOR_SIGNALING_PATHWAY	277	23	0.0003618130906083337	0.00855060556365763	C1orf64:ARNT:CREM:GPAM:SCGB2A1:YAP1:PHB2:TGFB1I1:BRCA1:NPLOC4:SAFB2:SAFB:PUM2:NCOA1:TAB1:PPARA:FOXP1:NR1I2:UBA5:ISL1:PPAP2A:TNFAIP3:ESR1
GO_bp	GO_POSITIVE_REGULATION_OF_CATALYTIC_ACTIVITY	1397	78	0.0003633201696653201	0.00855060556365763	MTOR:CDC20:TAL1:DNAJB4:BCL10:F3:VAV3:CKS1B:ARHGEF11:ABL2:PSEN2:AGAP4:AGAP8:C10orf54:PSAP:FAS:GPRC5A:NCKAP1L:APAF1:ORAI1:PSME1:MAP3K9:PSEN1:KIAA1199:PKD1:GRIN2A:TBC1D10B:CCL17:RABEP1:RCVRN:SPAG9:RALBP1:MBP:INSR:PKN1:DNAJB1:GSK3A:CXCL17:CARD8:FPR2:SPDYA:ALK:PKP4:SLC11A1:COPS8:PCNA:RALGAPA2:MMP24:SEMG1:SEMG2:TAB1:ARFGAP3:FBLN1:TGFBR2:GNAI2:NPRL2:IFT57:ADPRH:GSK3B:RASA2:DVL3:MAP3K13:CXCL1:TERT:CKS1B:ARAP3:PDGFRB:WRNIP1:EDN1:CDKN1A:C6orf89:ESR1:RFC2:PILRB:PRKAR2B:NCAPG2:LYN:NSMAF:SCRIB:KLF4
GO_bp	GO_POSITIVE_REGULATION_OF_CELL_DEATH	698	45	0.000364788088740521	0.00855060556365763	MTOR:BCL10:F3:VAV3:ARHGEF2:ARHGEF11:PHLDA3:PSEN2:FAS:SMPD1:APBB1:UCP2:APAF1:MAP3K9:PSEN1:MOAP1:GRIN2A:SIAH1:TRPV1:ARHGEF18:DEDD2:GSK3A:CARD8:NCOA1:CREB1:MFF:TGM2:SYCE3:HYAL2:SHQ1:C3orf38:IFT57:GSK3B:PDGFRB:CDKN1A:CNR1:SOD2:SEPT7:YWHAG:CD36:PPP3CC:LYN:NSMAF:SCRIB:NACC2
GO_bp	GO_CELLULAR_KETONE_METABOLIC_PROCESS	192	18	0.00036529117646101985	0.00855060556365763	MTOR:UBIAD1:ADCK3:DGAT2:NDUFA9:TPI1:PSME1:LONP2:COQ9:PSMD12:PANK2:PPARA:COQ2:CNR1:STAR:RDH10:CYP11B1:CYP11B2
GO_bp	GO_SEX_DIFFERENTIATION	260	22	0.0003712075282622097	0.008661508992784893	CSDE1:ADAM15:ASPM:FNDC3A:PKD1:SAFB2:INSR:OSR1:NCOA1:FSHR:MERTK:HYAL3:NIPBL:C5orf20:TIFAB:PDGFRB:ESR1:HOXA10:HOXA11:STAR:RDH10:LRRC6
GO_bp	GO_NEGATIVE_REGULATION_OF_INTRACELLULAR_SIGNAL_TRANSDUCTION	521	36	0.0003890621459388473	0.009049388521045974	MTOR:SZT2:TXNDC12:ASH1L:ARHGEF2:ABL2:PHLDA3:ZMYND11:SFRP5:DUSP8:SMPD1:SYVN1:PTPN6:OTUD7A:MAPKBP1:RRN3:MAPK7:NPLOC4:PHLPP1:GSK3A:CBLC:CARD8:FBLN1:GNAI2:HYAL2:NPRL2:GSK3B:RASA2:PYDC2:HDAC3:TNFAIP3:ESR1:SOD2:HSPB1:LYN:KLF4
GO_bp	GO_MEMORY	115	13	0.00039781951436031876	0.00922389094810202 8	MTOR:ABCC8:PSEN1:KCNK10:GRIN2A:NTAN1:RPS6KB1:INSR:CIC:CREB1:SHANK3:CNR1:CALB1
GO_bp	GO_TRNA_PROCESSING	130	14	0.0003996468641973495	0.009237120917768928	KTI12:NSUN6:ARL5B-AS1:TRUB1:MTFMT:CTU1:TRMT61B:TPRKB:RTCB:TRMU:THUMPD3:TRMT10A:THG1L:RPP40:DUS4L
GO_bp	GO_CELLULAR_RESPONSE_TO_PEPTIDE_HORMONE_STIMULUS	315	25	0.00040627619731490146	0.009360909248478138	RPE65:PKLR:BGLAP:NUCKS1:UCP2:RAP1B:PCK2:MYO5A:GLP2R:RPS6KB1:INSR:GSK3A:CEACAM1:ATP6V1B1:CREB1:GNAI2:EDN1:CRHR2:YWHAG:PRKAR2B:STAR:LYN:CA2:CYP11B1:CYP11B2
GO_bp	GO_I_KAPPAB_KINASE_NF_KAPPAB_SIGNALING	262	22	0.0004123067991039193	0.009470171791918147	TRIM62:BCL10:ASH1L:ZMYND11:ERC1:TNFRSF19:OTUD7A:MAPKBP1:TRIM25:CC2D1A:CARD8:TGM2:LIME1:TAB1:ATP2C1:TIFAB:NDFIP1:IRAK1BP1:TNFAIP3:ESR1:HSPB1:CD36
GO_bp	GO_LEUKOCYTE_HOMEOSTASIS	87	11	0.00041844967938393343	0.009572157728435546	MPL:BCL10:GPAM:NCKAP1L:TNFSF13B:CORO1A:PKN1:MERTK:CXCL6:TNFAIP3:LYN

GO_bp	GO_REGULATION_OF_CALCIUM_ION_TRANSMEMBRANE_TRANSPORT	146	15	0.0004193516719124144	0.009572157728435546	PSEN2:UBASH3B:PTPN6:MYO5A:KIAA1199:CORO1A:CACNB1:STAC2:CACNG1:LIME1:STIM2:DIAPH1:LYN:GEM:UBQLN1
GO_bp	GO_TRNA_METABOLIC_PROCESS	178	17	0.000424100722449343	0.00961709826330857	KTI12:NSUN6:ARL5B-AS1:TRUB1:DTD2:MTFMT:TARSL2:SARS2:CTU1:TRMT61B:TPRKB:RTCB:TRMU:THUMPD3:TRMT10A:THG1L:RPP40:DUS4L
GO_bp	GO_RESPONSE_TO_UV_B	18	5	0.00042524584157486875	0.00961709826330857	MFAP4:HYAL3:HYAL1:HYAL2:CDKN1A
GO_bp	GO_PEROXISOMAL_MEMBRANE_TRANSPORT	18	5	0.00042524584157486875	0.00961709826330857	ABCD3:PEX16:LONP2:PEX13:PEX7
GO_bp	GO_CILIUM_ORGANIZATION	371	28	0.0004266117432738624	0.009618393598352419	CCDC28B:SSX2IP:DNAJB13:FAM179B:B9D1:DNAI2:KIF19:AZI1:TUBB4A:DCTN1:ZMYND10:CCDC66:ARL13B:IFT57:NPHP3:DZIP1L:GMNC:MCIDAS:CCNO:C5orf30:TUBB:FGFR1OP:SEPT7:YWHAG:PRKAR2B:CEP41:LRRC6:TUBB4B
GO_bp	GO_REGULATION_OF_EMBRYONIC_DEVELOPMENT	131	14	0.0004322419789679078	0.009715530719920864	RNF207:TENM4:MAPK7:HNF1B:MBP:INSR:OSR1:CELSR1:NPHP3:DVL3:NIPBL:CDX1:SEPT7:KLF4
GO_bp	GO_RESPONSE_TO_TOPOLOGICALLY_INCORRECT_PROTEIN	195	18	0.00044035878336634116	0.009867795907751853	ZBTB17:DNAJB4:EXTL2:LMNA:HSPA6:SYVN1:HSPA8:ERO1L:PLA2G4B:SULT1A3:DERL2:DNAJB1:GSK3A:DCTN1:SRPRB:IL8:HSPB1:CREB3L2
GO_bp	GO_REGULATION_OF_INTRACELLULAR_STEROID_HORMONE_RECEPTOR_SIGNALING_PATHWAY	74	10	0.000442037846543317	0.009870002357383762	C1orf64:GPAM:YAP1:PHB2:BRCA1:SAFB2:FOXP1:UBA5:ISL1:ESR1
GO_bp	GO_HEART_DEVELOPMENT	564	38	0.00044314296298457706	0.009870002357383762	RNF207:MTOR:STIL:RBM15:GJA5:ADAM15:LMNA:RBM20:TENM4:YAP1:PSEN1:ACAN:PKD1:GJC1:INSR:GSK3A:MEGF8:OSR1:CREB1:PCNA:TAB1:PPARA:TGFBR2:SCN5A:ARL13B:IFT57:NPHP3:KCNAB1:FGF12:NPY1R:NPY5R:NIPBL:ISL1:PDGFRB:EDN1:CDKN1A:POPDC3:RB1CC1
GO_bp	GO_DRUG_TRANSPORT	212	19	0.000448202796387892	0.009952539436407873	SYT11:TACR2:SLC16A12:SYT13:OR5T1:SLC25A15:PSEN1:SLC47A1:SLC47A2:RALBP1:CD320:SLC11A1:PER2:SLC25A26:NR1I2:EDN1:CNR1:CA2:SLC25A25
GO_bp	GO_POSITIVE_REGULATION_OF_IMMUNE_RESPONSE	871	53	0.00046236024596344616	0.01023598737298593	CDC42:BCL10:VAV3:NRAS:CTSS:CTSK:APCS:FCGR2A:FCGR3A:CFHR5:DMBT1:HPX:CRTAM:PTPN6:C1S:NCKAP1L:TNFSF13B:PSME1:COCH:PSEN1:CLEC10A:TBX21:PSMD12:NPLOC4:MUC16:IL12RB1:CD79A:CEACAM1:FPR2:PUM2:IL18R1:IL18RAP:SLC11A1:ZBP1:LIME1:TAB1:FOXP1:PROS1:KLHL6:CFI:HLA-A:FLOT1:MUC21:C4A:CNR1:TNFAIP3:ESR1:LAT2:CD36:BLK:LYN:UBQLN1:FCN2
GO_bp	GO_RENAL_SYSTEM_PROCESS	117	13	0.00047046480911217936	0.010353042955013528	GJA5:PSAP:CHRNB4:TRPV1:PKN1:ATP6V1B1:SLC4A5:GNAI2:HYAL2:EDN1:CLDN4:PRKAR2B:CYP11B2
GO_bp	GO_ENDOPLASMIC_RETICULUM_UNFOLDED_PROTEIN_RESPONSE	117	13	0.00047046480911217936	0.010353042955013528	ZBTB17:EXTL2:LMNA:SYVN1:ERO1L:PLA2G4B:SULT1A3:DERL2:GSK3A:DCTN1:SRPRB:IL8:CREB3L2
GO_bp	GO_LEUKOCYTE_DIFFERENTIATION	507	35	0.00047302792824627807	0.010378373948089981	MTOR:CDC42:TAL1:GON4L:SEMA4A:BGLAP:APCS:C10orf54:UBASH3B:PTPN6:NCKAP1L:PDE1B:PSEN1:IKZF3:TBX21:TCF3:IL12RB1:CD79A:POU2F2:CEACAM1:KDELR1:IL18R1:MERTK:CREB1:NCAPH2:TGFBR2:TUSC2:FOXP1:DROSHA:PTGER4:NDFIP1:L3MBTL3:LYN:CA2:CDH17
GO_bp	GO_GLYCOSYLATION	266	22	0.0005065769159768066	0.011015799800087362	A3GALT2:EXTL2:B3GALNT2:GYLTL1B:SYVN1:KDELC1:PSEN1:SLC51B:ASGR2:ST8SIA5:MUC16:RPN2:TRAK1:TMEM115:A4GNT:MGAT1:MUC21:B3GALT4:FUT9:RP11-203J24.9:ST6GALNAC6:ST6GALNAC4:DPM2

GO_bp	GO_POSITIVE_REGULATION_OF_ION_TRANSPORT	266	22	0.0005065769159768066	0.011015799800087362	RNF207:CTSS:TACR2:KCNJ11:ABCC8:TRPC6:KCNA1:ORAI1:P2RX2:PSEN1:KIAA1199:PIRT:STAC2:KCNJ2:MCHR1:SHANK3:SCN5A:FGF12:STIM2:PDGFRB:EDN1:ABCB1
GO_bp	GO_POSITIVE_REGULATION_OF_GROWTH	266	22	0.0005065769159768066	0.011015799800087362	MTOR:CDC42:MACF1:ZFYVE27:GPAM:YAP1:DERL2:RND2:RPS6KB1:RNF157:INSR:MEGF8:CREB1:VIL1:TGFBR2:HYAL1:CACNA2D2:MAP3K13:NIPBL:EDN1:CPNE5:FGFR1OP
GO_bp	GO_STEROID_HORMONE_MEDIATED_SIGNALING_PATHWAY	181	17	0.000514243014500527	0.011149516686073373	C1orf64:GPAM:SCGB2A1:YAP1:PHB2:TGFB1I1:BRCA1:SAFB2:SAFB:NCOA1:PPARA:FOXP1:NR1I2:UBA5:ISL1:PPAP2A:ESR1
GO_bp	GO_POSITIVE_REGULATION_OF_TRANSMEMBRANE_TRANSPORT	198	18	0.0005284091772266572	0.011422963095929207	RNF207:CTSS:KCNJ11:ABCC8:TRPC6:KCNA1:PSEN1:ARPP19:KIAA1199:PIRT:STAC2:KCNJ2:INSR:SHANK3:STIM2:TERT:ABCB1:CA2
GO_bp	GO_MUSCLE_CELL_PROLIFERATION	232	20	0.000530044958233765	0.011424722706798162	MTOR:LDLRAP1:TENM4:YAP1:SMPD3:RPS6KB1:IGFBP5:TGM2:TGFBR2:GNAI2:FOXP1:NPY5R:TERT:MEGF10:PDGFRB:EDN1:CDKN1A:TNFAIP3:SOD2:KLF4
GO_bp	GO_EMBRYONIC_SKELETAL_JOINT_MORPHOGENESIS	11	4	0.0005321351994808021	0.01143623893621022	HOXC11:OSR1:HYAL1:HOXA11
GO_bp	GO_PEPTIDYL_AMINO_ACID_MODIFICATION	1219	69	0.0005406274110327102	0.011584873093558073	MTOR:TIE1:TAL1:DPH5:ARNT:SETDB1:CLK2:ASH1L:ARHGEF2:LMNA:UHMK1:ABL2:APBB1:HPX:WEE1:CTR9:SYVN1:TRPC6:DYRK4:PTPN6:GPRC5A:ULK1:KDELC1:FKBP3:PSEN1:KIAA1199:PKD1:SMG1:PRMT7:ASGR2:MAPK7:BRCA1:RPS6KB1:INSR:PKN1:MAST3:GSK3A:CBLC:NCOA1:ALK:BCL11A:MERTK:METTL21A:TTLL4:NDUFAF5:RPN2:TGM2:TGFBR2:NAT6:HYAL2:GSK3B:MAP3K13:SENP2:STK32B:AREG:AREGB:ISL1:PDGFRB:MGAT1:PPIL1:FGFR1OP:TPST1:CD36:CEP41:NCAPG2:BLK:ASH2L:LYN:DPM2:NTMT1
GO_bp	GO_CELL_CYCLE_G1_S_PHASE_TRANSITION	286	23	0.0005657157613546857	0.012087240831270175	MCM10:CUL2:WEE1:PTPN6:PHB2:POLE:PSME1:PKD1:RPS6KB1:SPDYA:CTDSP1:PCNA:GTSE1:HYAL1:EIF4E:TERT:LSM11:CDKN1A:SEPT7:MEPCE:GML:KLF4:NACC2
GO_bp	GO_RESPONSE_TO_OXIDATIVE_STRESS	435	31	0.0005751973080956333	0.012254203520298273	ANGPTL7:SZT2:ARNT:PSAP:UCP2:UCP3:TRPC6:ERO1L:PSEN1:SMPD3:MAPK7:RNF112:TPO:IL18RAP:PCNA:NDUFA6:HYAL1:HYAL2:NDUFB4:AREG:AREGB:MCTP1:PDGFRB:EDN1:TNFAIP3:SOD2:HSPB1:CD36:STAR:UBQLN1:XPA:KLF4
GO_bp	GO_PROTEIN_IMPORT	183	17	0.0005830975033432283	0.012386608813794012	TARDBP:LMNA:IPO7:PEX16:HSPA8:PHB2:KPNA3:IPO5:PSEN1:LONP2:KPNB1:PEX13:HYAL2:IPO11:HDAC3:PEX7:CD36
GO_bp	GO_CONNECTIVE_TISSUE_DEVELOPMENT	269	22	0.000589103517346532	0.012478129257916457	CTSK:GDF2:ACTA2:DGAT2:SOX5:PTHLH:COCH:ADAMTS7:ACAN:PKD1:SMPD3:OSR1:TGFBR2:HYAL3:HYAL1:HYAL2:PDGFRB:EDN1:ITGB8:HOXA11:CREB3L2:SLC25A25
GO_bp	GO_EPITHELIAL_CELL_DEVELOPMENT	200	18	0.0005952024977833393	0.01254709871977931	TMEM79:PSAP:ACTA2:RAP1B:FNDC3A:FRMD6:NPHS1:GSK3A:SLC4A5:SLC9A4:BFSP1:GSK3B:CDKN1A:ESR1:DACT2:CLDN3:SCRIB:S1PR3
GO_bp	GO_PROTEIN_LOCALIZATION_TO_MEMBRANE	613	40	0.0005966371312923026	0.01254709871977931	RPL22:ICMT:CLSTN1:LDLRAP1:MACF1:GOLPH3L:SSR2:ARL5B:ZFYVE27:KCNJ11:PEX16:NUMB:MOAP1:MYO5A:SLC51B:KIAA1199:MESDC2:GRIN2A:RABEP1:RPL19:STAC2:RPL38:VIL1:MFF:CHMP4B:SHANK3:RPL32:RPL14:ARL13B:AP2M1:RPL37:FLOT1:RPS18:MAP7:YWHAG:EXOC4:PPP3CC:RPL7:SCRIB:RPL12
GO_bp	GO_PATTERN_SPECIFICATION_PROCESS	436	31	0.0005974808914180624	0.01254709871977931	RNF207:HES2:NBL1:STIL:HIPK1:DBX1:CDON:HOXC13:HOXC11:FOXG1:PSEN1:HNF1B:DNAI2:MEGF8:OSR1:HES6:CELSR1:TGFBR2:ARL13B:IFT57:NPHP3:ISL1:C5orf20:TIFAB:CDX1:EDN1:HOXA10:HOXA11:EVX1:LRRC6:PBX3
GO_bp	GO_CELLULAR_PROTEIN_CATABOLIC_PROCESS	756	47	0.00060169465873605	0.012599589007720707	CDC20:CTSS:CTSK:DCST1:UBQLN4:F13B:CUL2:UBE2L6:SYVN1:USP5:SPSB2:FBXW8:SIAH3:UCHL3:DCAF11:PSME1:FBXO33:PSEN1:HERC2:OTUD7A:ADAMTS7:PKD1:NTAN1:TGFB1I1:LONP2:SIAH1:N4BP1:DERL2:TBX21:TRIM25:RNFT1:PSMD12:NPLOC4:UBXN6:KEAP1:GIPC1:GSK3A:CBLC:C19orf68:ANAPC1:CHMP4B:USP25:GSK3B:ARMC8:AP2M1:TNFAIP3:UBQLN1
GO_bp	GO_HOMOTYPIC_CELL_CELL_ADHESION	77	10	0.0006098773001412507	0.012734653852381228	MPL:PEAR1:CSRP1:UBASH3B:PTPN6:MYL12A:CEACAM1:MEGF10:HSPB1:LYN
GO_bp	GO_SENSORY_SYSTEM_DEVELOPMENT	361	27	0.0006141515154978918	0.012758790077697113	FOXE3:RPE65:OLFM3:HIPK1:CRB1:RAB18:CDON:C12orf57:NRL:SIX6:PSEN1:TRPM1:B9D1:FSCN2:SLC4A5:MERTK:BFSP1:SCO2:TGFBR2:CCDC66:NIPBL:ISL1:PDGFRB:RDH10:CALB1:KLF4:SLC25A25

GO_bp	GO_RNA_SPLICING	456	32	0.0006145049914972488	0.012758790077697113	TARDBP:PTBP2:RBM15:WDR77:BCAS2:SCNM1:CLK2:KIAA0907:SNRPE:RBM20:RBM7:HSPA8:DDX47:WBP4:PRPF39:RBM25:CPSF2:AQR:CD2BP2:POLR2C:ESRP2:PRMT7:METTL16:DDX39A:ZNF638:RTCB:ISY1:DBR1:SKIV2L2:PPIL1:KHDRBS3:PUF60
GO_bp	GO_NEGATIVE_REGULATION_OF_CYTOKINE_PRODUCTION	270	22	0.0006190938549827151	0.012817858687670297	SYT11:C10orf54:UBE2L6:PTPN6:NCKAP1L:MAPKBP1:TBX21:TRIM25:NPLOC4:CEACAM1:CARD8:IL1RL1:MERTK:SLC11A1:TUSC2:CD200R1:PYDC2:PTGER4:C5orf30:NDFIP1:TNFAIP3:KLF4
GO_bp	GO_IRE1_MEDIATED_UNFOLDED_PROTEIN_RESPONSE	64	9	0.0006283059630125116	0.012972047270061687	ZBTB17:EXTL2:LMNA:SYVN1:PLA2G4B:SULT1A3:GSK3A:DCTN1:SRPRB
GO_bp	GO_CALCIUM_MEDIATED_SIGNALING	218	19	0.0006316036990409461	0.013003605568490067	MTOR:SELP:P2RX2:PLA2G4B:MYO5A:GRIN2A:SULT1A3:NFATC3:MAPK7:GRIN2D:FPR2:ACKR2:GSK3B:ACKR4:IL8:MCTP1:EDN1:LAT2:PPP3CC
GO_bp	GO_SPINDLE_ORGANIZATION	168	16	0.0006371732391342271	0.013008953632323803	CDC20:STIL:MSTO1:ASPM:TUBB8:SAC3D1:GOLGA8R:PKD1:KPNB1:DCTN1:CHMP4B:KIF2A:HDAC3:TUBB:CHMP5:NTMT1
GO_bp	GO_CYTOSOLIC_CALCIUM_ION_TRANSPORT	168	16	0.0006371732391342271	0.013008953632323803	PSEN2:UBASH3B:PTPN6:P2RX2:ERO1L:TRPM1:MYO5A:KIAA1199:GRIN2A:CORO1A:TRPV1:GRIN2D:LIME1:SMDT1:DIAPH1:LYN
GO_bp	GO_INORGANIC_ANION_TRANSPORT	168	16	0.0006371732391342271	0.013008953632323803	MTOR:LRRC8B:LRRC8C:SLC25A3:TTYH2:SLC4A5:SLC11A1:SLC37A1:APOL1:SLC34A2:SLC4A4:SLC12A2:CLDN4:ABCB1:CA2:SLC34A3
GO_bp	GO_LIPID_CATABOLIC_PROCESS	325	25	0.0006416540734471548	0.013064148032788332	MTOR:ABCD3:PSAP:SMPD1:PCK2:PLA2G4B:PDXDC1:LONP2:PLA2G15:SMPD3:PAFAH1B3:LIPE:PLB1:PEX13:NAGA:PPARA:CPT1B:PLCXD2:ACAD11:LIPH:PLA2G12A:CNR1:PEX7:AIG1:CEL
GO_bp	GO_SPINDLE_ASSEMBLY	106	12	0.0006478424673525859	0.013117471446395334	CDC20:MSTO1:ASPM:TUBB8:SAC3D1:GOLGA8R:KPNB1:CHMP4B:KIF2A:HDAC3:TUBB:CHMP5
GO_bp	GO_RESPONSE_TO_RETINOIC_ACID	106	12	0.0006478424673525859	0.013117471446395334	TIE1:CLK2:ABL2:YAP1:PHB2:OSR1:NCOA1:CREB1:IGFBP2:PDGFRB:LYN:KLF4
GO_bp	GO_NUCLEIC_ACID_PHOSPHODIESTER_BOND_HYDROLYSIS	289	23	0.0006530103427212055	0.013185785766485878	EXOSC10:DNASE2B:HORMAD1:REXO2:APAF1:POLE:RNASEH2B:ERCC5:FANCM:CPSF2:SLX1A:KPNB1:PCNA:ASTE1:DBR1:XRN1:DROSHA:RPP40:MEI4:GTF2H5:RNASET2:RFC2:XPA
GO_bp	GO_REGULATION_OF_RNA_METABOLIC_PROCESS	219	19	0.0006677416668624058	0.013446304798462145	PTBP2:RBM15:CLK2:RBM20:CTR9:RBM7:RBM25:ESRP2:METTL16:SAFB2:SAFB:MYCN:SHQ1:TERT:DROSHA:DIMT1:UTP15:ESR1:KHDRBS3
GO_bp	GO_REGULATION_OF_MULTI_ORGANISM_PROCESS	401	29	0.0006755475092579883	0.013547625201846068	TARDBP:TRIM62:OVGP1:SETDB1:SYT11:APCS:NUCKS1:PC:MAPKBP1:POLR2C:TRIM25:NPLOC4:IL12RB1:CARD8:PUM2:PLB1:CHMP4B:SLPI:HYAL3:FAM208A:FOXP1:IL8:CXCL6:CNR1:TNFAIP3:CD36:LRSAM1:PAEP:NELFB
GO_bp	GO_VESICLE_TARGETING	92	11	0.0006774217569892359	0.013547625201846068	F5:SEC16B:SCFD1:PSEN1:TRAPPC2L:AP1M2:ARFGAP3:AREG:AREGB:BET1:EXOC4:SCRIB
GO_bp	GO_INTERLEUKIN_6_PRODUCTION	137	14	0.0006792964457383255	0.013547625201846068	BCL10:ASH1L:SYT11:ARHGEF2:PTPN6:NCKAP1L:MBP:IL18RAP:HYAL2:CD200R1:ISL1:TNFAIP3:CD36:PAEP
GO_bp	GO_RESPONSE_TO_TUMOR_NECROSIS_FACTOR	308	24	0.0006801460815620679	0.013547625201846068	HIPK1:ARHGEF2:KCNJ11:TNFRSF19:POSTN:TNFSF13B:PSME1:ADAMTS7:CCL17:SMPD3:TRPV1:BRCA1:RPS6KB1:PSMD12:CARD8:AFF3:HYAL3:HYAL1:HYAL2:PYDC2:IL8:OCLN:EDN1:TNFAIP3
GO_bp	GO_NEGATIVE_REGULATION_OF_PHOSPHORYLATION	441	31	0.0007206842644510125	0.014246451281117407	TARDBP:MTOR:ASH1L:ZMYND11:DUSP8:SMPD1:IPO7:UBASH3B:PTPN6:GPRC5A:ANKLE2:IPO5:PSEN1:PKN1:CEACAM1:CBLC:CTDSP1:FBLN1:HYAL2:NPRL2:HDAC3:CDKN1A:TNFAIP3:FGFR1OP:HSPB1:YWHAG:PRKAR2B:GFRA2:LYN:KLF4:PIP5KL1
GO_bp	GO_CELLULAR_RESPONSE_TO_ORGANIC_CYCLIC_COMPOUND	539	36	0.0007225207508810142	0.014246451281117407	C1orf64:HCN3:PKLR:BGLAP:GPAM:SCGB2A1:YAP1:FDX1:LINC01059:GRAMD1B:PHB2:RAP1B:PCK2:SULT1A3:TGFB1I1:TRPV1:BRCA1:RPS6KB1:SSTR2:SAFB2:SAFB:NCOA1:IGFBP5:PPARA:FOXP1:NR1I2:UBA5:XRN1:EIF4E:ISL1:PPAP2A:DIAPH1:EDN1:FLOT1:ESR1:STAR:KLF4

GO_bp	GO_BRANCH_ELONGATION_OF_AN_EPITHELIUM	20	5	0.0007227600354142013	0.014246451281117407	YAP1:HNF1B:AREG:AREGB:ESR1:RDH10
GO_bp	GO_NEGATIVE_REGULATION_OF_PROTEIN_MODIFICATION_PROCESS	599	39	0.0007229831738580671	0.014246451281117407	TARDBP:MTOR:ASH1L:ZMYND11:DUSP8:SMPD1:IPO7:UBASH3B:PTPN6:GPRC5A:NCKAP1L:IPO5:PPP1R36:PSEN1:ARPP19:N4BP1:BRCA1:PKN1:CEACAM1:CBLC:CTDSP1:PER2:FBLN1:GNAI2:HYAL2:GSK3B:SENP2:HDAC3:CDKN1A:TNFAIP3:FGFR1OP:HSPB1:YWHAG:PPP1R35:PRKAR2B:GFRA2:LYN:KLF4:PIP5KL1
GO_bp	GO_SIGNAL_TRANSDUCTION_BY_PROTEIN_PHOSPHORYLATION	952	56	0.0007262860177246826	0.01427326799539149	NRAS:ASH1L:ZMYND11:GDF2:PSAP:FAS:DUSP8:SMPD1:CDON:PTPN6:PHB2:RAP1B:TNFRSF19:PSME1:MAP3K9:PSEN1:SULT1A3:CCL17:DHX33:MAPK7:NBR1:SPAG9:PSMD12:PHLPP1:MBP:INSR:PKN1:NPHS1:CXCL17:CEACAM1:CBLC:FPR2:ALK:FSHR:LIME1:TAB1:FBLN1:MAPK8IP2:SHANK3:GNAI2:HYAL2:RASA2:DVL3:MAP3K13:AREG:AREGB:NPY5R:PTGER4:HDAC3:RELL2:PDGFRB:EDN1:CD36:GFRA2:RB1CC1:LYN:KLF4
GO_bp	GO_SINGLE_FERTILIZATION	138	14	0.0007303116987606548	0.014314109295708834	OVGP1:SMCP:ASH1L:TRPC6:AKAP3:LYZL6:ZPBP2:PLB1:WBP2NL:PKDREJ:HYAL3:HOXA10:HOXA11:PAEP
GO_bp	GO_POSITIVE_REGULATION_OF_PROTEIN_MODIFICATION_PROCESS	1212	68	0.00074539685447084	0.014570922554150727	MTOR:CDC20:TAL1:BCL10:ARNT:CKS1B:ARHGEF2:LMNA:GDF2:PSAP:FAS:SMPD1:HPX:CTR9:TRPC6:CDON:PHB2:GPRC5A:NCKAP1L:RAP1B:ANKLE2:TNFRSF19:FANCM:MAP3K9:PSEN1:SLC51B:KIAA1199:PKD1:CCL17:DHX33:BRCA1:SPAG9:INSR:PKN1:GSK3A:CXCL17:FPR2:SPDYA:ALK:FSHR:SLC11A1:COPS8:TAB1:MAPK8IP2:TGFBR2:GNAI2:DVL3:MAP3K13:SENP2:AREG:AREGB:NPY5R:NIPBL:ISL1:CKS1B:HDAC3:RELL2:NDFIP1:PDGFRB:EDN1:FLOT1:CDKN1A:C6orf89:CD36:PILRB:PRKAR2B:NCAPG2:RB1CC1:LYN:UBQLN1
GO_bp	GO_HEAD_DEVELOPMENT	764	47	0.0007510747500630144	0.01464296926515426	MTOR:HTR6:CDC42:TMEM57:MFSD2A:SZT2:STIL:LPHN2:PTBP2:PHGDH:ASPM:RAB18:CDON:KCNA1:C12orf57:APAF1:FOXG1:PSEN1:NUMB:GRIN2A:ANKRD11:HNF1B:SSTR2:MBP:DNAJB1:CIC:PAFAH1B3:GRIN2D:NCOA1:ALK:PEX13:POTEE:CREB1:C21orf91:PLXNB2:SHANK3:TGFBR2:SCN5A:ARL13B:GSK3B:KCNAB1:NIPBL:ISL1:NEUROD6:PPP3CC:STAR:SCRIB
GO_bp	GO_B_CELL_HOMEOSTASIS	30	6	0.0007656591907560603	0.014746968884828309	BCL10:NCKAP1L:TNFSF13B:PKN1:TNFAIP3:LYN
GO_bp	GO_LEUKOCYTE_MEDIATED_CYTOTOXICITY	108	12	0.0007665763829337904	0.014746968884828309	CRTAM:PTPN6:CORO1A:SERPINB4:IL12RB1:CEACAM1:IL18RAP:TUSC2:CXCL6:HLA-A:TUBB:TUBB4B
GO_bp	GO_T_HELPER_1_TYPE_IMMUNE_RESPONSE	41	7	0.0007690334667533676	0.014746968884828309	MTOR:SEMA4A:IL12RB1:IL1RL1:IL18R1:IL18RAP:SLC11A1
GO_bp	GO_CIRCULATORY_SYSTEM_PROCESS	541	36	0.0007719069304655046	0.014746968884828309	RNF207:MTOR:DDAH1:GJA5:F5:TACR2:ACTA2:KCNJ11:YAP1:P2RX2:POSTN:TRPV1:CACNB1:GJC1:CACNG1:KCNJ2:GSK3A:CEACAM1:KCNJ14:SLC4A5:PER2:MC3R:CYB5R3:PPARA:SCN5A:GNAI2:CACNA2D2:FGF12:NPY1R:KCNIP1:EDN1:CNR1:SOD2:KEL:CYP11B1:CYP11B2
GO_bp	GO_FERTILIZATION	171	16	0.0007721196135954693	0.014746968884828309	OVGP1:SMCP:ASH1L:TRPC6:AKAP3:FNDC3A:TDRD9:LYZL6:ZPBP2:PLB1:WBP2NL:PKDREJ:HYAL3:HOXA10:HOXA11:PAEP
GO_bp	GO_REGULATION_OF_ACTION_POTENTIAL	53	8	0.0007723560491238122	0.014746968884828309	RNF207:GJA5:KCNJ2:SCN5A:FGF12:CNR1:CD36:PTPN3
GO_bp	GO_SPHINGOLIPID_MEDIATED_SIGNALING_PATHWAY	12	4	0.0007744666652440446	0.014746968884828309	SMPD3:S1PR5:KLF14:S1PR3
GO_bp	GO_MANGANESE_ION_TRANSPORT	12	4	0.0007744666652440446	0.014746968884828309	TRPC6:SLC11A2:SLC11A1:ATP2C1
GO_bp	GO_CHEMOKINE_C_X_C_MOTIF_LIGAND_2_PRODUCTION	12	4	0.0007744666652440446	0.014746968884828309	POSTN:MBP:FOXP1:KLF4

GO_bp	GO_DETECTION_OF_STIMULUS	683	43	0.0007845407527681625	0.014900192591333319	RPE65:OR14K1:RGR:OR52B4:OR51T1:OR4C15:OR4C16:OR4P4:OR4S2:OR4C6:OR5T1:OR8H1:OR8K3:OR8K1:OR8J1:OR8U1:OR5R1:CRTAM:KCNA1:OR10P1:P2RX2:PKD1:TRPV1:RCVRN:OR1M1:OR7G2:OR7G1:OR7G3:OR10H2:OR10H3:PCNA:MMP24:PKDREJ:RAD18:CCDC66:OR10C1:RFC2:PIP:TAS2R39:TAS2R40:OR1L3:OR1L4:OR1L6
GO_bp	GO_POSITIVE_REGULATION_OF_CATION_CHANNEL_ACTIVITY	66	9	0.0007897472045985198	0.014960417406698765	RNF207:CTSS:KCNJ11:ABCC8:KCNA1:PIRT:STAC2:SHANK3:STIM2
GO_bp	GO_REGULATION_OF_PHOSPHATASE_ACTIVITY	172	16	0.0008221946202554261	0.015535039740044682	MTOR:SPOCD1:CSRNP2:NCKAP1L:NUAK1:ANKLE2:PPP1R36:ZFYVE1:ARPP19:CD2BP2:PPP4R1:TSKS:GNAI2:GSK3B:PDGFRB:PPP1R35
GO_bp	GO_NEGATIVE_REGULATION_OF_APOPTOTIC_SIGNALING_PATHWAY	223	19	0.0008306767552365658	0.015655061925612203	TXNDC12:ARHGEF2:LMNA:ZMYND11:FAS:SYVN1:YAP1:PSEN1:RRN3:MAPK7:BRCA1:RPS6KB1:MAPK8IP2:GNAI2:TERT:TNFAIP3:SOD2:HSPB1:RB1CC1
GO_bp	GO_REGULATION_OF_DEPHOSPHORYLATION	206	18	0.0008409225230415181	0.01580762287558864	MTOR:SPOCD1:SMG5:SMPD1:CSRNP2:NCKAP1L:NUAK1:ANKLE2:PPP1R36:ZFYVE1:ARPP19:CD2BP2:PPP4R1:TSKS:GNAI2:GSK3B:PDGFRB:PPP1R35
GO_bp	GO_PROTEIN_TARGETING	426	30	0.0008452740275039245	0.015848888015698583	RPL22:ICMT:SSR2:PEX16:HSPA8:DAO:SIAH3:SLC51B:KIAA1199:LONP2:RPL19:NPEPPS:RPL38:AP1M2:GIPC1:GSK3A:PEX13:MFF:CHMP4B:SYNGR1:RPL32:RPL14:TRAK1:RPL37:RPS18:PEX7:YWHAG:EXOC4:RPL7:RPL12
GO_bp	GO_REGULATION_OF_CATABOLIC_PROCESS	959	56	0.0008603282192026024	0.016090107916384547	TARDBP:MTOR:LAMTOR5:ARNT:UBQLN4:ABL2:F13B:C10orf54:PSAP:HSPA8:USP5:APAF1:FBXW8:PRKAB1:ULK1:PSME1:SCFD1:PSEN1:ANP32A:PKD1:GRIN2A:LONP2:N4BP1:METTL16:DERL2:RNFT1:PSMD12:TNRC6C:INSR:KEAP1:GIPC1:GSK3A:PUM2:PPP1CB:XPO1:ATP6V1B1:SLC11A1:CHMP4B:EIF6:USP25:PPARA:DAZL:NPRL2:GSK3B:XRN1:NDFIP1:CNR1:TNFAIP3:HSPB1:EXOC4:RB1CC1:NRBP2:UBQLN1:XPA:PTPN3:LRSAM1
GO_bp	GO_REGULATION_OF_NEUROTRANSMITTER_LEVELS	332	25	0.0008703421735968321	0.016194974622624595	MTOR:DDAH1:KCNC4:SYT11:TACR2:HSPA8:PDE1B:RAP1B:PSEN1:CHRNB4:GRIN2A:TRPV1:INSR:GIPC1:PER2:DNAJC5:STX19:GSK3B:MCTP1:EDN1:FLOT1:CNR1:CD36:SCRIB:KLF4
GO_bp	GO_NEGATIVE_REGULATION_OF_CELL_DEVELOPMENT	332	25	0.0008703421735968321	0.016194974622624595	CERS2:SEMA6C:ARHGEF2:SEMA4A:ASPM:ABCC8:TRPC6:PTHLH:POSTN:FOXG1:PSEN1:TRPV1:GSK3A:BCL11A:CTDSP1:FBLN1:PPARA:C3orf17:GSK3B:MFI2:EIF4E:TERT:ISL1:S1PR3:PAEP
GO_bp	GO_EXTRINSIC_APOPTOTIC_SIGNALING_PATHWAY	224	19	0.0008763726026324057	0.016239665713193067	BCL10:HIPK1:ARHGEF2:LMNA:ZMYND11:FAS:YAP1:DDX47:MOAP1:MAPK7:BRCA1:RPS6KB1:DEDD2:GSK3A:HYAL2:GSK3B:TERT:TNFAIP3:RB1CC1
GO_bp	GO_POSITIVE_REGULATION_OF_MITOTIC_NUCLEAR_DIVISION	54	8	0.0008771628963452581	0.016239665713193067	CDCA5:PHB2:DLGAP5:SMPD3:INSR:NIPBL:PDGFRB:EDN1
GO_bp	GO_RESPONSE_TO_CHEMOKINE	95	11	0.0008886282029394634	0.01638250398238457	MPL:RBM15:CCL17:ACKR2:ACKR4:IL8:CXCL6:PF4V1:CXCL1:SLC12A2:EDN1
GO_bp	GO_CARTILAGE_DEVELOPMENT	207	18	0.0008893359304723052	0.01638250398238457	CTSK:GDF2:SOX5:PTHLH:COCH:ADAMTS7:ACAN:PKD1:SMPD3:OSR1:TGFBR2:HYAL3:HYAL1:HYAL2:EDN1:ITGB8:HOXA11:CREB3L2
GO_bp	GO_POSITIVE_REGULATION_OF_NITRIC_OXIDE_BIOSYNTHETIC_PROCESS	42	7	0.0008932013230430358	0.016384824064683053	MTOR:DDAH1:TRPV1:INSR:EDN1:CD36:KLF4
GO_bp	GO_GAMETE_GENERATION	667	42	0.0008939203333248849	0.016384824064683053	MTOR:PHC2:HOOK1:HORMAD1:ASPM:TUBB8:CREM:DDX25:LGR5:FNDC3A:TDRD9:HERC2:PRSS21:SIAH1:PRMT7:ZPBP2:SPATA32:RPS6KB1:SSTR2:AZI1:TAF4B:PAFAH1B3:SPDYA:FSHR:MERTK:PANK2:SEMG1:SEMG2:OSBP2:NCAPH2:SYCE3:DAZL:PPAP2A:SOX30:MEI4:CNR1:SYNE1:HOXA10:HOXA11:SEPT7:SPATA31A4:SPATA31A7:PAEP

GO_bp	GO_CELLULAR_RESPONSE_TO_TOPOLOGICALLY_INCORRECT_PROTEIN	157	15	0.0008981639375986527	0.01642165408296044	ZBTB17:EXTL2:LMNA:HSPA6:SYVN1:HSPA8:ERO1L:PLA2G4B:SULT1A3:DERL2:GSK3A:DCTN1:SRPRB:IL8:CREB3L2
GO_bp	GO_AMINO_ACID_TRANSPORT	141	14	0.0009031994084458731	0.016437037483528986	LRRC8C:SLC16A12:SLC43A1:SLC25A15:PSEN1:SLC7A6:TRPV1:GIPC1:OSR1:SLC11A1:PER2:NPY5R:SLC6A19:SLC6A18
GO_bp	GO_POSITIVE_REGULATION_OF_CELL_CYCLE_PROCESS	296	23	0.0009034779786864913	0.016437037483528986	ZBTB17:CDC42:CDCA5:PHB2:CIT:DLGAP5:SMPD3:RNF112:BRCA1:INSR:GIPC1:PKP4:PCNA:GTSE1:HYAL1:TERT:NIPBL:PDGFRB:LSM11:EDN1:CDKN1A:MEPCE:GML
GO_bp	GO_DNA_STRAND_ELONGATION	21	5	0.0009194093860492342	0.016685577746819436	NUCKS1:POLE:PCNA:TERT:WRNIP1
GO_bp	GO_ORGANIC_ACID_BIOSYNTHETIC_PROCESS	297	23	0.0009452970776702631	0.017040788410678288	ACOT7:RIMKLA:ELOVL1:ACOT11:ABCD3:PHGDH:ALDH18A1:AASDHPPT:SDS:PRKAB1:MYO5A:BRCA1:TECR:CEACAM1:PER2:EIF6:CYP2D6:CYP8B1:RBP1:EDN1:ASL:STAR:RDH10
GO_bp	GO_CELL_GROWTH	468	32	0.0009459376423886723	0.017040788410678288	CLSTN1:MTOR:CDC42:MACF1:SEMA6C:ADAM15:SEMA4A:ARHGEF11:GDF2:ZFYVE27:APBB1:ULK1:POSTN:DERL2:RND2:RNF157:GSK3A:MEGF8:CEACAM1:BCL11A:IGFBP5:PPARA:TGFBR2:HYAL1:HYAL2:GSK3B:MAP3K13:EDN1:CDKN1A:CPNE5:FGFR1OP:NAIF1
GO_bp	GO_REGULATION_OF_HEMOPOIESIS	468	32	0.0009459376423886723	0.017040788410678288	MTOR:MPL:TAL1:RBM15:ARNT:BGLAP:APCS:C10orf54:CTR9:YAP1:UBASH3B:PTPN6:NCKAP1L:PSME1:TCF12:IKZF3:TBX21:PSMD12:TNRC6C:TCF3:IL12RB1:CEACAM1:CREB1:EIF6:TGFBR2:FOXP1:DROSHA:NDFIP1:NCAPG2:ASH2L:LYN:CA2
GO_bp	GO_GOLGI_ORGANIZATION	142	14	0.0009679859373223602	0.017390779425784042	CDC42:HOOK1:GOLPH3L:SEC16B:VPS51:FBXW8:CIT:GOLGA8G:GOLGA8J:GOLGA8R:GOLGA8H:NPLOC4:RAB43:SYNE1
GO_bp	GO_MONOSACCHARIDE_BIOSYNTHETIC_PROCESS	96	11	0.0009700978999416948	0.017390779425784042	CLK2:PC:DGAT2:TPI1:ENO2:SDS:PCK2:ARPP19:PER2:PPARA:FAM3C
GO_bp	GO_CHEMOKINE_C_C_MOTIF_LIGAND_2_SECRETION	6	3	0.00097543523684809	0.01740157522046957	POSTN:MBP:FOXP1
GO_bp	GO_MANGANESE_ION_TRANSMEMBRANE_TRANSPORT	6	3	0.00097543523684809	0.01740157522046957	SLC11A2:SLC11A1:ATP2C1
GO_bp	GO_REGULATION_OF_MEMBRANE_POTENTIAL	430	30	0.0009797419424420527	0.017436085416341614	RNF207:GJA5:HCN3:KCNJ11:UCP2:KCNA1:P2RX2:PSEN1:KCNK10:CHRNB4:GRIN2A:TRPV1:GJC1:KCNJ2:SH3GL1:GSK3A:GRIN2D:SCN3A:PANK2:MAPK8IP2:SHANK3:SCN5A:TUSC2:GSK3B:FGF12:EDN1:CNR1:POPDC3:CD36:PTPN3
GO_bp	GO_POSITIVE_REGULATION_OF_CELL_PROJECTION_ORGANIZATION	373	27	0.0010014012393169108	0.01777850026323501	MTOR:CDC42:MACF1:ABL2:ZFYVE27:APBB1:FBXW8:PSEN1:RRN3:RND2:PLEKHM1:RNF157:MEGF8:VIL1:C21orf91:PLXNB2:SHANK3:TRAK1:ZMYND10:DVL3:MAP3K13:LRRC16A:CPNE5:CNR1:SEPT7:CREB3L2:LYN
GO_bp	GO_REGULATION_OF_NUCLEOBASE_CONTAINING_COMPOUND_METABOLIC_PROCESS	510	34	0.0010309758152149487	0.01825945118513222	EXOSC10:PTBP2:RBM15:CLK2:SMG5:NUCKS1:RBM20:APBB1:CTR9:RBM7:APAF1:RBM25:SMG1:SLX1A:ESRP2:METTL16:BRCA1:TBX21:SAFB2:SAFB:GSK3A:MYCN:PCNA:SHQ1:XRN1:TERT:DROSHA:DIMT1:UTP15:NDFIP1:WRNIP1:ESR1:TERF1:KHDRBS3
GO_bp	GO_NEGATIVE_REGULATION_OF_IMMUNE_SYSTEM_PROCESS	451	31	0.0010355114978485957	0.018295695935546103	NBL1:TAL1:BCL10:RBM15:TNFAIP8L2:DCST1:APCS:FCRLB:C10orf54:GPAM:CTR9:UBASH3B:PTPN6:LRFN5:TBX21:NPLOC4:SERPINB4:PKN1:CEACAM1:IL1RL1:MERTK:CD200R1:NPY5R:PTGER4:NDFIP1:CNR1:TNFAIP3:NCAPG2:LYN:SCRIB:UBQLN1
GO_bp	GO_NUCLEOBASE_CONTAINING_SMALL_MOLE	355	26	0.0010394058532997592	0.01832046288190223	ACOT7:ACOT11:EXTL2:AMPD1:HMGCS2:GPAM:DGAT2:REXO2:HSPA8:PTHLH:RNASEH2B:SULT1A3:DCTPP1:PRPSAP2:PDE4A:GSK3A:ATP6V1B1:UCKL1:APOBEC3B:TYMP:ENTPD3:DHFRL1:DCTD:MGAT1:TPST1:SLC25A25

	CULE_METABOLIC_PROCESS					
GO_bp	GO_REGULATION_OF_CARBOHYDRATE_BIOSYNTHETIC_PROCESS	97	11	0.0010576387296793257	0.018568707835695697	MTOR:CLK2:DGAT2:ARPP19:SMPD3:INSR:GSK3A:PPP1CB:PPARA:GSK3B:FAM3C
GO_bp	GO_REGULATION_OF_CELL_CYCLE_G1_S_PHASE_TRANSITION	193	17	0.0010585426643750335	0.018568707835695697	WEE1:PTPN6:PHB2:PSME1:PKD1:CTDSP1:PCNA:GTSE1:HYAL1:TERT:LSM11:CDKN1A:SEPT7:MEPCE:GML:KLF4:NACC2
GO_bp	GO_CELL_PART_MORPHOGENESIS	673	42	0.0010619856850047702	0.018584749487583478	NBL1:CDC42:MACF1:SZT2:SEMA6C:SEMA4A:ZFYVE27:APBB1:WEE1:TRPC6:C12orf57:SLC11A2:NCKAP1L:FBXW8:ULK1:POSTN:FOXG1:PSEN1:NUMB:SIAH1:MAPK7:RND2:RNF157:MEGF8:BCL11A:CREB1:VIL1:MFF:PANK2:PLXNB2:MAPK8IP2:SHANK3:TRAK1:GSK3B:DVL3:MAP3K13:ISL1:FLOT1:CPNE5:NYAP1:KEL:GFRA2
GO_bp	GO_RESPONSE_TO_MOLECULE_OF_BACTERIAL_ORIGIN	337	25	0.0010742555124540272	0.01868402049960369	BCL10:SELP:CTR9:ABCC8:SHPK:SHPK:RPS6KB1:PTGER1:NFKBIB:CARD8:SLC11A1:SLPI:FOXP1:PLSCR4:IL8:CXCL6:PF4V1:CXCL1:PTGER4:EDN1:CNR1:TNFAIP3:CD36:STAR:LYN:CHMP5
GO_bp	GO_CELL_SURFACE_RECEPTOR_SIGNALING_PATHWAY_INVOLVED_IN_CELL_CELL_SIGNALING	612	39	0.0010761932696389438	0.01868402049960369	CDC42:MACF1:ASPM:SFRP5:GRK5:CTR9:YAP1:LGR5:P2RX2:PSME1:PSEN1:CHRNB4:MESDC2:PKD1:GRIN2A:TGFB1I1:TRPV1:HNF1B:PSMD12:TNRC6C:SH3GL1:GSK3A:GRIN2D:MERTK:IGFBP2:CELSR1:MAPK8IP2:SHANK3:GSK3B:NPHP3:DVL3:AP2M1:SENP2:TERT:ISL1:TNFAIP3:ESR1:DACT2:KLF4
GO_bp	GO_DEVELOPMENTAL_GROWTH_INVOLVED_IN_MORPHOGENESIS	228	19	0.0010813588056956588	0.01868402049960369	MACF1:SEMA6C:SEMA4A:ZFYVE27:YAP1:ULK1:POSTN:HNF1B:RND2:RNF157:MEGF8:BCL11A:TGFBR2:GSK3B:MAP3K13:AREG:AREGB:CPNE5:ESR1:RDH10
GO_bp	GO_PEPTIDYL_TYROSINE_MODIFICATION	356	26	0.0010821907882107723	0.01868402049960369	MTOR:TIE1:TAL1:CLK2:ARHGEF2:ABL2:HPX:WEE1:DYRK4:PTPN6:GPRC5A:PSEN1:INSR:CBLC:ALK:MERTK:HYAL2:AREG:AREGB:ISL1:PDGFRB:FGFR1OP:TPST1:CD36:NCAPG2:BLK:LYN
GO_bp	GO_NEGATIVE_REGULATION_OF_MUSCLE_ADAPTATION	13	4	0.001085452619500786	0.01868402049960369	MTOR:LMNA:IGFBP5:KLF4
GO_bp	GO_PROTEIN_IMPORT_INTO_PEROXISOME_MATRIX	13	4	0.001085452619500786	0.01868402049960369	PEX16:LONP2:PEX13:PEX7
GO_bp	GO_REGULATION_OF_INTEGRIN_ACTIVATION	13	4	0.001085452619500786	0.01868402049960369	FBLIM1:SELP:PTGER4:CDH17
GO_bp	GO_CHAPERONE_COFACTOR_DEPENDENT_PROTEIN_REFOLDING	32	6	0.0010959683886225082	0.018820952468166906	DNAJB4:HSPA6:DNAJB13:HSPA8:ERO1L:DNAJB1
GO_bp	GO_ORGANOPHOSPHATE_METABOLIC_PROCESS	532	35	0.0011025729817269	0.018884571397519725	ACOT7:ACOT11:VAV3:AMPD1:HMGCS2:GPAM:SMPD1:DGAT2:REXO2:HSPA8:LPCAT3:PTHLH:MTMR6:RNASEH2B:PLA2G4B:SMG1:SULT1A3:PLA2G15:SMPD3:IMPA2:FPR2:PLB1:ATP6V1B1:MIOX:PLA2G12A:DCTD:PPAP2A:PDGFRB:TPST1:LYN:IPPK:KLF4:PIP5KL1:DPM2:SLC25A25
GO_bp	GO_RESPONSE_TO_ANTIBIOTIC	319	24	0.00110481165999095	0.018884571397519725	SETDB1:BGLAP:PSAP:TRPC6:FDX1:GRIN2A:SMPD3:MAPK7:RNF112:RPS6KB1:IL18RAP:PCNA:HYAL3:HYAL1:HYAL2:XRN1:AREG:AREGB:PDGFRB:EDN1:CNR1:TNFAIP3:CLDN3:STAR:KLF4
GO_bp	GO_REGULATION_OF_MICROTUBULE_BASED_PROCESS	211	18	0.0011075547420358173	0.018887534463951874	MACF1:STIL:PRUNE:ARHGEF2:ARL2:FAM179B:PKD1:BRCA1:AZI1:TUBB4A:XPO1:DCTN1:CHMP4B:RASSF1:GSK3B:SLAIN2:DIAPH1:CHMP5

GO_bp	GO_MEMBRANE_DOCKING	177	16	0.001116026463719646	0.018987950250785646	PSEN2:PEX16:SCFD1:B9D1:AZI1:TUBB4A:DCTN1:STX19:TUBB:FGFR1OP:YWHAG:PRKAR2B:CEP41:EXOC4:ESYT2:TUBB4B
GO_bp	GO_AMIDE_BIOSYNTHETIC_PROCESS	843	50	0.0011438689472506001	0.01941671307688663	RPL22:MTOR:ELOVL1:DPH5:CERS2:DAP3:UHMK1:LARP4B:SMPD1:MRPL49:DDX25:MTIF3:SLC25A15:MTFMT:TARSL2:RPL3L:SMPD3:METTL16:DHX33:RPL19:RPS6KB1:RPL38:TNRC6C:TECR:SARS2:MRPS12:PUM2:IGFBP5:NCL:PER2:EIF6:RPL32:DAZL:EIF1B:RPL14:MRPS22:XRN1:EIF4E:RPL37:DHX29:CPEB4:EIF4E1B:RPS18:B3GALT4:ASL:HSPB1:NSMAF:RPL7:RPL12:RP11-203J24.9:ST6GALNAC6
GO_bp	GO_UTERUS_DEVELOPMENT	22	5	0.001153219680195366	0.019454090222131633	ASH1L:NIPBL:ESR1:HOXA10:HOXA11
GO_bp	GO_POSITIVE_REGULATION_OF_TRANSCRIPTION_BY_RNA_POLYMERASE_I	22	5	0.001153219680195366	0.019454090222131633	MTOR:DHX33:NCL:UTP15:IPPK
GO_bp	GO_NEGATIVE_REGULATION_OF_KINASE_ACTIVITY	247	20	0.001154011338346856	0.019454090222131633	DUSP8:SMPD1:IPO7:UBASH3B:PTPN6:GPRC5A:IPO5:PSEN1:PKN1:CEACAM1:CBLC:HYAL2:NPRL2:CDKN1A:TNFAIP3:FGFR1OP:HSPB1:YWHAG:PRKAR2B:LYN
GO_bp	GO_GLAND_DEVELOPMENT	435	30	0.0011737840283507124	0.01974213411528086	WDR77:ASH1L:PSAP:UCP2:PHB2:CIT:ORAI1:PCK2:PSEN1:PKD1:ESRP2:HNF1B:RPL19:INSR:CEACAM1:NCOA1:CREB1:IGFBP5:PCNA:TGM2:TGFBR2:NPHP3:PSAPL1:AREG:AREGB:ISL1:TNFAIP3:ESR1:RB1CC1:SCRIB:PTPN3
GO_bp	GO_REGULATION_OF_BLOOD_PRESSURE	178	16	0.0011843648085915813	0.019874614938694343	DDAH1:GJA5:ACTA2:P2RX2:POSTN:TRPV1:GSK3A:SLC4A5:MC3R:PPARA:NPY1R:EDN1:CNR1:SOD2:CYP11B1:CYP11B2
GO_bp	GO_APOPTOTIC_CELL_CLEARANCE	44	7	0.001188611439034563	0.01990044208861968	PEAR1:MERTK:TGM2:MEGF10:C4A:CD36:FCN2
GO_bp	GO_MALE_GAMETE_GENERATION	535	35	0.0012135961141438755	0.020272571452176102	PHC2:HOOK1:HORMAD1:ASPM:CREM:DDX25:FNDC3A:TDRD9:HERC2:PRSS21:SIAH1:ZPBP2:SPATA32:SSTR2:AZI1:TAF4B:PAFAH1B3:SPDYA:FSHR:MERTK:PANK2:SEMG1:SEMG2:OSBP2:SYCE3:DAZL:SOX30:MEI4:CNR1:SYNE1:HOXA10:HOXA11:SEPT7:SPATA31A4:SPATA31A7:PAEP
GO_bp	GO_LOCOMOTORY_BEHAVIOR	196	17	0.0012535185878870597	0.02084470954970563	TAL1:CDH23:PSAP:PDE1B:MYO5A:CHRNB4:NTAN1:GRIN2D:ALK:PEX13:MC3R:CELSR1:FGF12:NPY1R:ASL:CALB1:PBX3
GO_bp	GO_MORPHOGENESIS_OF_A_BRANCHING_STRUCTURE	196	17	0.0012535185878870597	0.02084470954970563	RBM15:GDF2:YAP1:PHB2:PKD1:ESRP2:HNF1B:BCL11A:TGM2:CELSR1:TGFBR2:MAP3K13:AREG:AREGB:EDN1:ESR1:HOXA11:RDH10
GO_bp	GO_PHOSPHATIDYLINOSITOL_3_KINASE_SIGNALING	146	14	0.0012675868744738323	0.02102178347808258	PEAR1:SELP:PTPN6:INSR:CEACAM1:FSHR:LIME1:PDGFRB:EDN1:IER3:RGL2:NYAP1:LYN:KLF4
GO_bp	GO_NEUROTRANSMITTER_TRANSPORT	267	21	0.0012698873284719273	0.02102178347808258	KCNC4:SYT11:TACR2:HSPA8:RAP1B:PSEN1:CHRNB4:GIPC1:SLC6A16:PER2:DNAJC5:STX19:GSK3B:SLC6A19:SLC6A18:MCTP1:EDN1:FLOT1:CNR1:LYN:SCRIB
GO_bp	GO_REGIONALIZATION	342	25	0.0013182476548382236	0.021736960202930575	HES2:NBL1:HIPK1:DBX1:CDON:HOXC13:HOXC11:FOXG1:PSEN1:HNF1B:MEGF8:OSR1:HES6:CELSR1:ARL13B:IFT57:ISL1:C5orf20:TIFAB:CDX1:EDN1:HOXA10:HOXA11:EVX1:PBX3
GO_bp	GO_RESPONSE_TO_KETONE	197	17	0.0013249194790357684	0.021736960202930575	ADAM15:BGLAP:KCNJ11:YAP1:FDX1:PCK2:RPS6KB1:NCOA1:PCNA:XRN1:EIF4E:PTGER4:EDN1:CDKN1A:CLDN4:STAR:KLF4
GO_bp	GO_MONOCARBOXYLIC_ACID_BIOSYNTHETIC_PROCESS	197	17	0.0013249194790357684	0.021736960202930575	ACOT7:ELOVL1:ABCD3:SDS:PRKAB1:MYO5A:BRCA1:TECR:CEACAM1:PER2:EIF6:CYP2D6:CYP8B1:RBP1:EDN1:STAR:RDH10
GO_bp	GO_PROTEIN_TARGETING_TO_MEMBRANE	197	17	0.0013249194790357684	0.021736960202930575	RPL22:ICMT:SSR2:PEX16:SLC51B:KIAA1199:RPL19:RPL38:MFF:CHMP4B:RPL32:RPL14:RPL37:RPS18:EXOC4:RPL7:RPL12

GO_bp	GO_AMINO_ACID_TRANSMEMBRANE_TRANSPORT	85	10	0.0013281856634700102	0.021742014758362082	LRRC8C:SLC16A12:SLC43A1:SLC25A15:PSEN1:SLC7A6:OSR1:PER2:SLC6A19:SLC6A18
GO_bp	GO_REGULATION_OF_MYELOID_CELL_DIFFERENTIATION	250	20	0.0013351894116523982	0.02175973874865882	MTOR:MPL:TAL1:RBM15:ARNT:BGLAP:APCS:CTR9:UBASH3B:NCKAP1L:TNRC6C:CEACAM1:CREB1:EIF6:FOXP1:NDFIP1:NCAPG2:ASH2L:LYN:CA2
GO_bp	GO_ORGANIC_ACID_CATABOLIC_PROCESS	250	20	0.0013351894116523982	0.02175973874865882	MTOR:DDAH1:ABCD3:ASRGL1:DAO:SDS:SDSL:PCK2:LONP2:PLA2G15:PRODH2:LIPE:PEX13:PPARA:CPT1B:ACAD11:CNR1:PEX7:AIG1:CEL
GO_bp	GO_RIBOSOMAL_LARGE_SUBUNIT_BIOGENESIS	71	9	0.0013444666434004751	0.02186245537387941	EBNA1BP2:GTF3A:RPL3L:RPL38:EIF6:RPL14:KIAA1239:RPL7:RPL12
GO_bp	GO_T_CELL_ACTIVATION_INVOLVED_IN_IMMUNE_RESPONSE	100	11	0.001360142690797581	0.02205724115559294	MTOR:SEMA4A:LCP1:PSEN1:TBX21:IL12RB1:CEACAM1:IL18R1:SLC11A1:FOXP1:PTGER4
GO_bp	GO_NEGATIVE_REGULATION_OF_I_KAPPAB_KINASE_NF_KAPPAB_SIGNALING	45	7	0.0013624472768216593	0.02205724115559294	ASH1L:ZMYND11:OTUD7A:MAPKBP1:CARD8:TNFAIP3:ESR1
GO_bp	GO_CELLULAR_PROCESS_INVOLVED_IN_REPRODUCTION_IN_MULTICELLULAR_ORGANISM	343	25	0.0013723929717623395	0.02216942492846856	MTOR:HOOK1:HORMAD1:ASPM:TUBB8:DDX25:LGR5:FNDC3A:TDRD9:PRMT7:LYZL6:ZPBP2:RPS6KB1:TAF4B:SPDYA:FSHR:PANK2:SEMG1:SEMG2:OSBP2:NCAPH2:DAZL:PPAP2A:MEI4:PAEP
GO_bp	GO_SENSORY_PERCEPTION_OF_CHEMICAL_STIMULUS	519	34	0.0013815469328334654	0.022268355167381515	OR14K1:OR52B4:OR51T1:OR4C15:OR4C16:OR4P4:OR4S2:OR4C6:OR5T1:OR8H1:OR8K3:OR8K1:OR8J1:OR8U1:OR5R1:OR10P1:P2RX2:TRPV1:OR1M1:OR7G2:OR7G1:OR7G3:PDE4A:OR10H2:OR10H3:OR10C1:CD36:PIP:TAS2R39:TAS2R40:OR1L3:OR1L4:OR1L6:OBP2A
GO_bp	GO_RESPONSE_TO_INSULIN	269	21	0.0013929644335955072	0.02240325730180958	MTOR:RPE65:PKLR:BGLAP:NUCKS1:ABCC8:UCP2:UCP3:PCK2:MYO5A:RPS6KB1:INSR:GSK3A:CEACAM1:ATP6V1B1:EIF6:PPARA:GNAI2:YWHAG:STAR:LYN
GO_bp	GO_HEART_MORPHOGENESIS	251	20	0.0014007360914874705	0.022479061730202855	RNF207:MTOR:STIL:RBM15:GJA5:ADAM15:YAP1:PSEN1:INSR:GSK3A:MEGF8:TAB1:TGFBR2:ARL13B:IFT57:NPHP3:NPY1R:NPY5R:NIPBL:ISL1
GO_bp	GO_SIGNAL_RELEASE	460	31	0.0014153508722611152	0.022634082391884004	TARDBP:KCNC4:SYT11:TACR2:KCNJ11:ABCC8:ARL2:UCP2:HSPA8:RAP1B:PSEN1:MYO5A:CHRNB4:SMPD3:HNF1B:GIPC1:CREB1:PER2:DNAJC5:MYRIP:CACNA2D2:STX19:GSK3B:ISL1:MCTP1:EDN1:CNR1:BLK:LYN:ENY2:SCRIB
GO_bp	GO_CELL_MORPHOGENESIS_INVOLVED_IN_DIFFERENTIATION	725	44	0.0014165548163628085	0.022634082391884004	CDC42:MACF1:MPL:SZT2:TAL1:SEMA6C:SEMA4A:ZFYVE27:APBB1:TRPC6:C12orf57:PTPN6:SLC11A2:FBXW8:ULK1:POSTN:FOXG1:COCH:FRMD6:PSEN1:NUMB:SIAH1:MAPK7:RND2:MEGF8:MERTK:CREB1:FBLN1:PLXNB2:MAPK8IP2:SHANK3:TRAK1:ARL13B:GSK3B:MAP3K13:MFI2:ISL1:LRRC16A:FLOT1:DACT2:CLDN3:KEL:GFRA2:SCRIB
GO_bp	GO_ESTABLISHMENT_OF_PROTEIN_LOCALIZATION_TO_MEMBRANE	325	24	0.0014210586916770967	0.022656792589645685	RPL22:ICMT:CLSTN1:MACF1:GOLPH3L:SSR2:PEX16:MOAP1:SLC51B:KIAA1199:RPL19:RPL38:MFF:CHMP4B:RPL32:RPL14:RPL37:RPS18:YWHAG:EXOC4:PPP3CC:RPL7:SCRIB:RPL12
GO_bp	GO_RETROGRADE_VESICLE_MEDIATED_TRANSPORT_GOLGI_TO_ENDOPLASMIC_RETICULUM	86	10	0.0014531815279700527	0.0231187970358872	GOLPH3L:SCFD1:KDELR1:ARFGAP3:TMEM115:RAB6B:COPB2:KIF2A:TAPBP:COPG2
GO_bp	GO_REGULATION_OF_MUSCLE_SYSTEM_PROCESS	252	20	0.001469004473314035	0.0232576355757327	RNF207:MTOR:GJA5:LMNA:TACR2:ABCC8:KCNA1:C12orf57:CHRNB4:RPS6KB1:KCNJ2:SSTR2:GSK3A:IGFBP5:PPARA:SCN5A:C5orf20:TIFAB:EDN1:KLF4

GO_bp	GO_NEUROBLAST_DIVISION	14	4	0.0014745657385430528	0.0232576355757327	ARHGEF2:ASPM:SOX5:NUMB
GO_bp	GO_ALDITOL_METABOLIC_PROCESS	14	4	0.0014745657385430528	0.0232576355757327	MOGAT2:DGAT2:GK5:COQ2
GO_bp	GO_EMBRYONIC_SKELETAL_JOINT_DEVELOPMENT	14	4	0.0014745657385430528	0.0232576355757327	HOXC11:OSR1:HYAL1:HOXA11
GO_bp	GO_RESPONSE_TO_CORTICOSTEROID	165	15	0.0014852094536765776	0.02336602278141951	BGLAP:UCP3:PCK2:RPS6KB1:SSTR2:IGFBP2:PCNA:AREG:AREGB:EIF4E:ISL1:SOX30:EDN1:CDKN1A:STAR:PAPPA
GO_bp	GO_REGULATION_OF_PROTEOLYSIS	706	43	0.001489997152203093	0.02336602278141951	BCL10:F3:LAMTOR5:UBQLN4:C10orf54:FAS:USP5:APAF1:FBXW8:PSME1:PSEN1:PAPLN:PKD1:GRIN2A:N4BP1:DERL2:RNFT1:SERPINB4:MBP:KEAP1:GIPC1:GSK3A:CARD8:VIL1:TM4SF20:WFDC5:WFDC12:PI3:SEMG1:SEMG2:SLPI:USP25:FBLN1:PROS1:IFT57:GSK3B:MFI2:C4A:PI16:LYN:UBQLN1:KLF4:PTPN3
GO_bp	GO_INOSITOL_LIPID_MEDIATED_SIGNALING	182	16	0.0014941538377234243	0.02336602278141951	PEAR1:SELP:PTPN6:PIRT:RPS6KB1:INSR:CEACAM1:FSHR:LIME1:PDGFRB:EDN1:IER3:RGL2:NYAP1:LYN:KLF4
GO_bp	GO_NEGATIVE_REGULATION_OF_ESTABLISHMENT_OF_PROTEIN_LOCALIZATION	182	16	0.0014941538377234243	0.02336602278141951	SYT11:KCNJ11:ABCC8:UCP2:SIAH3:MAPKBP1:DERL2:CARD8:CD200R1:PYDC2:PTGER4:NDFIP1:TNFAIP3:CD36:TERF1:ENY2
GO_bp	GO_MUSCLE_SYSTEM_PROCESS	462	31	0.0015145376321694876	0.023634504451052512	RNF207:MTOR:GJA5:TMOD4:LMNA:ARHGEF11:TACR2:ACTA2:ABCC8:KCNA1:C12orf57:P2RX2:CHRNB4:MYLPF:TRPV1:GJC1:RPS6KB1:KCNJ2:SSTR2:MYL12A:MYL12B:GSK3A:IGFBP5:PPARA:SCN5A:FGF12:C5orf20:TIFAB:EDN1:CALD1:KLF4
GO_bp	GO_POSITIVE_REGULATION_OF_NEURON_PROJECTION_DEVELOPMENT	271	21	0.0015260883024846145	0.02376429877809728	MTOR:MACF1:ABL2:ZFYVE27:APBB1:FBXW8:PSEN1:RRN3:RND2:RNF157:MEGF8:C21orf91:PLXNB2:SHANK3:TRAK1:DVL3:MAP3K13:CPNE5:CNR1:CREB3L2:LYN
GO_bp	GO_POTASSIUM_ION_TRANSPORT	235	19	0.0015389923025911842	0.02388111144064006	RNF207:KCNC4:GJA5:HCN3:KCNJ11:ABCC8:KCNA1:MTMR6:KCNK10:KCNJ16:KCNJ2:KCNJ14:SLC9A4:KCNS1:KCNAB1:SLC12A2:KCNIP1:KEL:KCNV2
GO_bp	GO_POSITIVE_REGULATION_OF_INTRACELLULAR_PROTEIN_TRANSPORT	149	14	0.001540088003110665	0.02388111144064006	TARDBP:SEC16B:IPO5:PSEN1:SLC51B:KIAA1199:NPEPPS:AZI1:GSK3A:MFF:GTSE1:HYAL2:GSK3B:HDAC3
GO_bp	GO_RESPONSE_TO_NICOTINE	46	7	0.0015555116333744417	0.0240694958006361	KCNJ11:CHRNB4:CREB1:PPARA:EDN1:CNR1:STAR
GO_bp	GO_MICROTUBULE_ORGANIZING_CENTER_ORGANIZATION	133	13	0.0015656158220169018	0.024161826449867294	STIL:SSX2IP:ARL2:SAC3D1:BRCA1:AZI1:XPO1:DCTN1:CHMP4B:MCIDAS:CETN3:CNTLN:CHMP5
GO_bp	GO_DETECTION_OF_STIMULUS_INVOLVED_IN_SENSORY_PERCEPTION	523	34	0.0015680532267464897	0.024161826449867294	RPE65:OR14K1:OR52B4:OR51T1:OR4C15:OR4C16:OR4P4:OR4S2:OR4C6:OR5T1:OR8H1:OR8K3:OR8K1:OR8J1:OR8U1:OR5R1:KCNA1:OR10P1:TRPV1:OR1M1:OR7G2:OR7G1:OR7G3:OR10H2:OR10H3:MMP24:CCDC66:OR10C1:PIP:TAS2R39:TAS2R40:OR1L3:OR1L4:OR1L6
GO_bp	GO_POSITIVE_REGULATION_OF_MITOTIC_CELL_CYCLE	166	15	0.0015772301727755176	0.024252388639958277	TAL1:CDCA5:PHB2:DLGAP5:SMPD3:RPS6KB1:INSR:HYAL1:EIF4E:TERT:NIPBL:PDGFRB:LSM11:EDN1:MEPCE

GO_bp	GO_CARBOHYDRATE_DERIVATIVE_CATABOLIC_PROCESS	183	16	0.0015814406721446255	0.024259007929798838	ACOT7:OVGP1:FMOD:PSAP:KIAA1199:ACAN:DCTPP1:PDE4A:GSK3A:APOBEC3B:NAGA:TYMP:HYAL3:HYAL1:HYAL2:MGAT1
GO_bp	GO_CHAPERONE_MEDIATED_PROTEIN_FOLDING	59	8	0.0015847597811195955	0.024259007929798838	DNAJB4:HSPA6:DNAJB13:HSPA8:ERO1L:DNAJB1:DNAJC5:HSPB1
GO_bp	GO_CD4_POSITIVE_ALPHA_BETA_T_CELL_ACTIVATION	87	10	0.001587562287650781	0.024259007929798838	MTOR:SEMA4A:C10orf54:NCKAP1L:TBX21:IL12RB1:IL18R1:TGFBR2:FOXP1:PTGER4
GO_bp	GO_RESPONSE_TO_TEMPERATURE_STIMULUS	236	19	0.0016161566460582276	0.02464471234134434	MTOR:ACOT11:DNAJB4:PKLR:HSPA6:UCP2:UCP3:HSPA8:ERO1L:TGFB1I1:TRPV1:PIRT:RPS6KB1:DNAJB1:MMP24:GSK3B:PLAC8:CDKN1A:LYN
GO_bp	GO_TRANSCRIPTION_COUPLED_NUCLEOTIDE_EXCISION_REPAIR	73	9	0.0016394704622684875	0.024870384226994814	ERCC5:AQR:POLR2C:COPS8:PCNA:ISY1:GTF2H5:RFC2:XPA
GO_bp	GO_BRANCHING_MORPHOGENESIS_OF_AN_EPITHELIAL_TUBE	150	14	0.0016411069864071407	0.024870384226994814	RBM15:GDF2:YAP1:PHB2:PKD1:ESRP2:HNF1B:CELSR1:TGFBR2:AREG:AREGB:EDN1:ESR1:HOXA11:RDH10
GO_bp	GO_POSITIVE_REGULATION_OF_ION_TRANSMEMBRANE_TRANSPORT	150	14	0.0016411069864071407	0.024870384226994814	RNF207:CTSS:KCNJ11:ABCC8:TRPC6:KCNA1:PSEN1:KIAA1199:PIRT:STAC2:KCNJ2:SHANK3:STIM2:ABCB1
GO_bp	GO_VITAMIN_A_METABOLIC_PROCESS	7	3	0.001659230664099555	0.02499046184658141	RPE65:RBP2:RBP1
GO_bp	GO_RECOGNITION_OF_APOPTOTIC_CELL	7	3	0.001659230664099555	0.02499046184658141	PEAR1:MEGF10:FCN2
GO_bp	GO_MICTURITION	7	3	0.001659230664099555	0.02499046184658141	PSAP:CHRNB4:TRPV1
GO_bp	GO_RESPONSE_TO_BACTERIUM	669	41	0.0016908329097382808	0.02541435968625023	BCL10:SYT11:SELP:DMBT1:CTR9:ABCC8:COCH:MAPKBP1:SHPK:SHPK:LYZL6:IKZF3:RPS6KB1:PTGER1:NFKBIB:CARD8:SLC11A1:VIL1:BPIFA2:WFDC12:SEMG1:SEMG2:SLPI:TUSC2:FOXP1:PLSCR4:KLHL6:IL8:CXCL6:PF4V1:CXCL1:PLAC8:DROSHA:PTGER4:EDN1:CNR1:TNFAIP3:CD36:STAR:LYN:CHMP5:FCN2
GO_bp	GO_RESPONSE_TO_RADIATION	446	30	0.001721593143075975	0.025823897146139627	MTOR:RPE65:CLK2:NUCKS1:RGR:ABCC8:YAP1:PDE1B:IKBIP:ERCC5:TRPM1:GRIN2A:N4BP1:RCVRN:MFAP4:BRCA1:PPP1CB:CREB1:PER2:PCNA:RAD18:HYAL3:HYAL1:HYAL2:CCDC66:NIPBL:CDKN1A:GTF2H5:STAR:XPA
GO_bp	GO_PROTEASOMAL_PROTEIN_CATABOLIC_PROCESS	466	31	0.0017310980449726588	0.025871978822904988	CDC20:UBQLN4:CUL2:SYVN1:USP5:SPSB2:FBXW8:SIAH3:DCAF11:PSME1:FBXO33:PSEN1:HERC2:PKD1:SIAH1:N4BP1:DERL2:TBX21:TRIM25:RNFT1:PSMD12:NPLOC4:UBXN6:KEAP1:GIPC1:GSK3A:ANAPC1:USP25:GSK3B:ARMC8:UBQLN1
GO_bp	GO_ADENYLATE_CYCLASE_INHIBITING_G_PROTEIN_COUPLED_RECEPTOR_SIGNALING_PATHWAY	88	10	0.001731838582431191	0.025871978822904988	PSAP:OR5T1:SSTR2:FPR2:MCHR1:GNAI2:PSAPL1:NPY1R:EDN1:S1PR3
GO_bp	GO_REGULATION_OF_INFLAMMATORY_RESPONSE	349	25	0.0017391769215865762	0.025928905423248143	TNFAIP8L2:ASH1L:APCS:LRFN5:SHPK:SHPK:MAPK7:CXCL17:IL1RL1:TGM2:PPARA:HYAL2:FOXP1:CD200R1:PYDC2:NPY5R:DROSHA:PTGER4:ISL1:C5orf30:NDFIP1:CNR1:TNFAIP3:ESR1:LYN:KLF4

GO_bp	GO_CERAMIDE_METABOLIC_PROCESS	24	5	0.0017487417162350522	0.026018727964225982	PLA2G15:SMPD3:ITGB8:NSMAF:RP11-203J24.9:ST6GALNAC6
GO_bp	GO_PHOTORECEPTOR_CELL_DEVELOPMENT	47	7	0.001769216464698715	0.026206145096650924	RPE65:OLFM3:CRB1:NRL:TRPM1:FSCN2:CCDC66
GO_bp	GO_PEPTIDE_CROSS_LINKING	60	8	0.0017692373170907314	0.026206145096650924	FLG:LCE1F:LCE1E:LCE1D:LCE1C:LCE1B:TGM2:PI3
GO_bp	GO_SKELETAL_SYSTEM_MORPHOGENESIS	238	19	0.0017803521522823593	0.026206145096650924	HOXC11:COCH:PSEN1:ACAN:PKD1:SMPD3:ANKRD11:MEGF8:OSR1:TGFBR2:HYAL1:HYAL2:NIPBL:C5orf20:TIFAB:CDX1:PEX7:HOXA11:RDH10
GO_bp	GO_RESPONSE_TO_FLUID_SHEAR_STRESS	35	6	0.001783487357229312	0.026206145096650924	PKD1:MAPK7:HDAC3:PDGFRB:CA2:KLF4
GO_bp	GO_REGULATION_OF_GLIAL_CELL_PROLIFERATION	35	6	0.001783487357229312	0.026206145096650924	MTOR:CERS2:ABCC8:CREB1:TERT:LYN
GO_bp	GO_EYE_PHOTORECEPTOR_CELL_DEVELOPMENT	35	6	0.001783487357229312	0.026206145096650924	OLFM3:CRB1:NRL:TRPM1:FSCN2:CCDC66
GO_bp	GO_ORGANIC_ACID_TRANSMEMBRANE_TRANSPORT	135	13	0.0017914342167662494	0.026206145096650924	LRRC8C:ABCD3:SLC16A12:SLC43A1:SLC25A15:PSEN1:SLC7A6:OSR1:PER2:CPT1B:SLC6A19:SLC6A18:SLC35D2
GO_bp	GO_NUCLEAR_TRANSCRIBED_MRNA_CATABOLIC_PROCESS_NONSENSE_MEDIATED_DECAY	119	12	0.0017914752938120522	0.026206145096650924	RPL22:EXOSC10:SMG5:SMG1:RPL19:RPL38:RPL32:RPL14:RPL37:RPS18:RPL7:RPL12
GO_bp	GO_RNA_SPLICING_VIA_TRANSESTERIFICATION_REACTIONS	369	26	0.00179342734470958	0.026206145096650924	PTBP2:RBM15:WDR77:BCAS2:SCNM1:KIAA0907:SNRPE:RBM7:HSPA8:WBP4:PRPF39:RBM25:CPSF2:AQR:CD2BP2:POLR2C:ESRP2:PRMT7:METTL16:DDX39A:ISY1:DBR1:SKIV2L2:PPIL1:KHDRBS3:PUF60
GO_bp	GO_NEGATIVE_REGULATION_OF_EXTRINSIC_APOPTOTIC_SIGNALING_PATHWAY	104	11	0.0018704038627872525	0.027276722998980766	ARHGEF2:LMNA:ZMYND11:FAS:YAP1:MAPK7:BRCA1:RPS6KB1:TERT:TNFAIP3:RB1CC1
GO_bp	GO_CHROMOSOME_SEGREGATION	313	23	0.0018801238116812199	0.027364178249221716	CDC42:CDC20:HORMAD1:MSTO1:PMF1:CDCA5:PHB2:FANCM:DLGAP5:BRCA1:KPNB1:PUM2:ANAPC1:CHMP4B:NCAPH2:SYCE3:RAD18:NIPBL:MEI4:TERF1:GEM:CHMP5:NTMT1
GO_bp	GO_REGULATION_OF_RESPONSE_TO_BIOTIC_STIMULUS	136	13	0.0019140468919260183	0.027763647593435685	SYT11:CRTAM:MAPKBP1:NPLOC4:IL12RB1:CEACAM1:CARD8:PUM2:FOXP1:CXCL6:TNFAIP3:CD36:LRSAM1
GO_bp	GO_NEGATIVE_REGULATION_OF_CELL_CYCLE	632	39	0.0019151250788941349	0.027763647593435685	MTOR:ZBTB17:CDC20:FOXE3:LAMTOR5:HORMAD1:UHMK1:CUL2:APBB1:IPO7:WEE1:BTG4:APAF1:PRKAB1:RNASEH2B:IPO5:PSME1:MGA:PKD1:RNF112:BRCA1:PSMD12:CTDSP1:PCNA:GTSE1:RASSF1:NEK11:IL8:MDC1:CDKN1A:TNFAIP3:HECA:SEPT7:TERF1:GML:SCRIB:KLF4:PTPN3:NACC2
GO_bp	GO_GERMINAL_CENTER_FORMATION	15	4	0.0019512189121662113	0.02823121851264105	TNFSF13B:PKN1:KLHL6:TNFAIP3
GO_bp	GO_AMMONIUM_ION_METABOLIC_PROCESS	61	8	0.0019702573700406518	0.028450671256972086	SMPD1:LPCAT3:PDE1B:PLA2G4B:GRIN2A:SMPD3:PLB1:PLA2G12A
GO_bp	GO_GLIAL_CELL_PROLIFERATION	48	7	0.0020050156274261832	0.02883926587393825	MTOR:CERS2:ABCC8:CREB1:AREG:AREGB:TERT:LYN

GO_bp	GO_CALCIUM_DEPENDENT_CELL_CELL_ADHESION_VIA_PLASMA_MEMBRANE_CELL_ADHESION_MOLECULES	48	7	0.0020050156274261832	0.02883926587393825	SELP:CDH23:ATP2C1:CDH9:CDH6:PCDHGC3:CDH17
GO_bp	GO_ESTABLISHMENT_OR_MAINTENANCE_OF_CELL_POLARITY	205	17	0.002030000868226687	0.029141614026301072	CDC42:ARHGEF2:LMNA:ARHGEF11:CRB1:SFRP5:WEE1:NCKAP1L:RAP1B:PKD1:RND2:FSCN2:DCTN1:RND3:GSK3B:MAP7:SCRIB
GO_bp	GO_REGULATION_OF_PEPTIDE_SECRETION	471	31	0.0020389980262634395	0.029205263581627526	TARDBP:GJA5:GOLPH3L:SYT11:GPAM:APBB1:KCNJ11:ABCC8:ARL2:UCP2:CRTAM:POSTN:MAPKBP1:MBP:CARD8:IL1RL1:PER2:MYRIP:HYAL2:CACNA2D2:FOXP1:CD200R1:PYDC2:PTGER4:ISL1:CNR1:TNFAIP3:BLK:LYN:ENY2:PAEP
GO_bp	GO_NEGATIVE_REGULATION_OF_PROTEIN_SERINE_THREONINE_KINASE_ACTIVITY	137	13	0.0020434965356871163	0.029205263581627526	DUSP8:SMPD1:IPO7:PTPN6:IPO5:CBLC:HYAL2:CDKN1A:TNFAIP3:HSPB1:YWHAG:PRKAR2B:LYN
GO_bp	GO_METANEPHROS_DEVELOPMENT	90	10	0.0020521953108750325	0.029205263581627526	YAP1:HOXC11:PKD1:HNF1B:OSR1:NIPBL:PDGFRB:HOXA11:RDH10:CALB1
GO_bp	GO_KERATINIZATION	223	18	0.0020543022138369295	0.029205263581627526	HRNR:FLG:LCE1F:LCE1E:LCE1D:LCE1C:LCE1B:TMEM79:KRTAP5-1:KRTAP5-2:KRTAP5-3:KRTAP5-4:KRTAP5-5:CNFN:PKP4:PI3:KRTAP21-3:KRTAP8-1
GO_bp	GO_DEVELOPMENTAL_CELL_GROWTH	223	18	0.0020543022138369295	0.029205263581627526	MTOR:MACF1:SEMA6C:SEMA4A:ZFYVE27:ULK1:POSTN:RND2:RNF157:GSK3A:MEGF8:BCL11A:PPARA:TGFBR2:GSK3B:MAP3K13:EDN1:CPNE5
GO_bp	GO_REGULATION_OF_MUSCLE_ORGAN_DEVELOPMENT	154	14	0.002101958804060553	0.02980276817265282	MTOR:TLL2:YAP1:CDON:RPS6KB1:GSK3A:CREB1:PPARA:TGFBR2:MEGF10:C5orf20:TIFAB:EDN1:FLOT1
GO_bp	GO_NEGATIVE_REGULATION_OF_HYDROLASE_ACTIVITY	452	30	0.0021044403648444645	0.02980276817265282	SPOCD1:LAMTOR5:APCS:ARL2:CSRNP2:NCKAP1L:IPO5:PPP1R36:ZFYVE1:PAPLN:ARPP19:CD2BP2:MAPK7:SERPINB4:CARD8:TSKS:RTKN:VIL1:WFDC5:WFDC12:PI3:SLPI:GNAI2:PROS1:GSK3B:C4A:PI16:PPP1R35:TERF1:KLF4
GO_bp	GO_HISTONE_MRNA_METABOLIC_PROCESS	25	5	0.0021188839218881416	0.029949609280534306	EXOSC10:SNRPE:CPSF2:XRN1:LSM11
GO_bp	GO_CELLULAR_CARBOHYDRATE_METABOLIC_PROCESS	260	20	0.0021243416202572224	0.02996911882704527	MTOR:CLK2:MOGAT2:DGAT2:TPI1:PCK2:IMPA2:INSR:GSK3A:PPP1CB:RPE:IGFBP5:PER2:PPARA:MIOX:GSK3B:GK5:COQ2:FAM3C:IPPK
GO_bp	GO_POSITIVE_REGULATION_OF_TRANSPORTER_ACTIVITY	106	11	0.002178558019182934	0.030675098545966596	RNF207:CTSS:KCNJ11:ABCC8:TRPC6:KCNA1:PIRT:STAC2:SHANK3:STIM2:ABCB1
GO_bp	GO_DNA_BIOSYNTHETIC_PROCESS	189	16	0.002199944653736403	0.0309170042159896	EXOSC10:SMG5:GDF2:UBE2L6:POLE:SMPD3:TRIM25:NPLOC4:PCNA:XRN1:TERT:PDGFRB:WRNIP1:RFC2:TERF1:KLF4
GO_bp	GO_RESPONSE_TO_ACID_CHEMICAL	336	24	0.0022049440469015104	0.030928127375431488	MTOR:TIE1:LAMTOR5:CLK2:ABL2:UCP2:DGAT2:YAP1:PHB2:IPO5:RPS6KB1:OSR1:NCOA1:BCL11A:CREB1:IGFBP2:PCNA:PTGER4:PDGFRB:CPEB4:EDN1:CD36:LYN:KLF4
GO_bp	GO_REGULATION_OF_SYSTEMIC_ARTERIAL_BLOOD_PRESSURE	91	10	0.002229371312621055	0.031211198376694768	DDAH1:GJA5:P2RX2:POSTN:TRPV1:GSK3A:SLC4A5:EDN1:SOD2:CYP11B2
GO_bp	GO_RESPONSE_TO_MECHANICAL_STIMULUS	207	17	0.002248584080298695	0.03142032887869849	BCL10:BGLAP:FAS:KCNA1:POSTN:PKD1:RPS6KB1:KCNJ2:IGFBP2:PKDREJ:SHANK3:TGFBR2:PTGER4:C5orf20:TIFAB:EDN1:XPA

GO_bp	GO_PROTEIN_HOMOO LIGOMERIZATION	337	24	0.0022916422 09197782	0.0319612338 4744535	BCL10:KCNC4:SYT11:KCNA1:APAF1:P2RX2:KCTD12:DCTPP1:SMPD3:TRPV1:SPAG9:KCNJ2:CD79A:TRMT61B:MFF:CHMP4B:TGM2:KCNS1:THG1L:FLOT1:SOD2:CLDN3:KCNV2:NACC2
GO_bp	GO_REGULATION_OF_INNATE_IMMUNE_RESPONSE	435	29	0.0023167998 50780945	0.0322509070 1371201	BCL10:NRAS:CTSS:CTSK:DCST1:DMBT1:HPX:CRTAM:PTPN6:PSME1:COCH:CLEC10A:PSMD12:NPLOC4:SERPINB4:MUC16:CEACAM1:PUM2:IL18RAP:ZBP1:TAB1:FLOT1:MUC21:TNFAIP3:ESR1:CD36:LYN:SCRIB:UBQLN1
GO_bp	GO_AMMONIUM_TRANSPORT	107	11	0.0023473892 963927793	0.0326149552 5233824	RHCE:SYT11:TACR2:SYT13:OR5T1:PSEN1:RALBP1:SLC12A2:EDN1:CNR1:LYN
GO_bp	GO_MUSCLE_CONTRACTION	357	25	0.0023564851 363929544	0.0326795580 2356267	RNF207:MTOR:GJA5:TMOD4:ARHGEF11:TACR2:ACTA2:KCNA1:C12orf57:P2RX2:CHRNB4:MYLPF:TRPV1:GJC1:RPS6KB1:KCNJ2:SSTR2:MYL12A:MYL12B:SCN5A:FGF12:C5orf20:TIFAB:EDN1:CALD1
GO_bp	GO_MALE_SEX_DIFFERENTIATION	156	14	0.0023697172 75815113	0.0327395149 9481406	CSDE1:ADAM15:ASPM:FNDC3A:SAFB2:INSR:NCOA1:FSHR:PDGFRB:ESR1:HOXA10:HOXA11:STAR:LRRC6
GO_bp	GO_REGULATION_OF_EXTRINSIC_APOPTOTIC_SIGNALING_PATHWAY	156	14	0.0023697172 75815113	0.0327395149 9481406	BCL10:ARHGEF2:LMNA:ZMYND11:FAS:YAP1:MAPK7:BRCA1:RPS6KB1:DEDD2:HYAL2:TERT:TNFAIP3:RB1CC1
GO_bp	GO_POSITIVE_REGULATION_OF_CELLULAR_PROTEIN_LOCALIZATION	319	23	0.0023927803 123176532	0.0329961262 5803893	TARDBP:SYT11:SEC16B:KCNJ11:IPO5:PSEN1:SLC51B:KIAA1199:MESDC2:STAC2:NPEPPS:AZI1:GSK3A:VIL1:MFF:GTSE1:HYAL2:GSK3B:TERT:NIPBL:HDAC3:YWHAG:PPP3CC
GO_bp	GO_DETERMINATION_OF_HEART_LEFT_RIGHT_ASYMMETRY	63	8	0.0024259912 73424501	0.0333290389 90037536	RNF207:STIL:PSEN1:MEGF8:TGFBR2:ARL13B:IFT57:NPHP3
GO_bp	GO_LYMPHOCYTE_HOMEOSTASIS	63	8	0.0024259912 73424501	0.0333290389 90037536	BCL10:GPAM:NCKAP1L:TNFSF13B:CORO1A:PKN1:TNFAIP3:LYN
GO_bp	GO_PROTEIN_LOCALIZATION_TO_ENDOPLASMIC_RETICULUM	140	13	0.0024757476 12776712	0.0338247192 3812132	RPL22:SEC16B:RPL19:RPL38:KDELR1:CHMP4B:RPL32:RPL14:RPL37:RPS18:BCAP29:RPL7:RPL12
GO_bp	GO_HEPATICOBILIARY_SYSTEM_DEVELOPMENT	140	13	0.0024757476 12776712	0.0338247192 3812132	UCP2:CIT:PCK2:PKD1:HNF1B:RPL19:CEACAM1:PCNA:NPHP3:NIPBL:TNFAIP3:RB1CC1:PTPN3
GO_bp	GO_RAS_PROTEIN_SIGNAL_TRANSDUCTION	437	29	0.0024758774 081781315	0.0338247192 3812132	CDC42:SSX2IP:VAV3:NRAS:SETDB1:RIT1:ARHGEF2:ARHGEF11:ABL2:RAB18:RAP1B:RND2:KPNB1:ARHGEF18:RTKN:RND3:CELSR1:RASSF1:RAB43:RAB6B:MRAS:RASA2:PDGFRB:FLOT1:RGL2:CDKN1A:RAB44:PLEKHG1:LYN
GO_bp	GO_REGULATION_OF_REPRODUCTIVE_PROCESS	157	14	0.0025137854 349594857	0.0342788922 9490208	CDC20:OVGP1:WDR77:HORMAD1:ASPM:TACR2:INSR:PLB1:SEMG1:SEMG2:HYAL3:CNR1:ESR1:PAEP
GO_bp	GO_HYALURONAN_CATABOLIC_PROCESS	16	4	0.0025246791 15320403	0.0343636879 58527706	KIAA1199:HYAL3:HYAL1:HYAL2
GO_bp	GO_REGULATION_OF_ANION_TRANSMEMBRANE_TRANSPORT	26	5	0.0025430033 169663246	0.0344853770 8432193	MTOR:PSEN1:OSR1:PER2:ABCB1
GO_bp	GO_POSITIVE_REGULATION_OF_TRANSLATIONAL_INITIATION	26	5	0.0025430033 169663246	0.0344853770 8432193	MTOR:UHMK1:RPS6KB1:DAZL:DHX29
GO_bp	GO_RESPONSE_TO_INTERLEUKIN_12	50	7	0.0025489061 85573981	0.0345017688 10255545	CDC42:RAP1B:LCP1:BOLA2B:IL12RB1:GSTA2:SOD2

GO_bp	GO_GLYCOPROTEIN_BIOSYNTHETIC_PROCESS	340	24	0.002569321896784835	0.03471418371575099	EXTL2:SOAT1:B3GALNT2:GYLTL1B:SYVN1:KDELC1:PSEN1:SLC51B:ACAN:ASGR2:ST8SIA5:MUC16:CHST8:RPN2:TRAK1:TMEM115:A4GNT:MGAT1:MUC21:B3GALT4:FUT9:RP11-203J24.9:ST6GALNAC6:ST6GALNAC4:DPM2
GO_bp	GO_RIBOSOMAL_PROTEIN_IMPORT_INTO_NUCLEUS	8	3	0.002580604812509506	0.03480265205861444	IPO5:KPNB1:IPO11
GO_bp	GO_METHYLATION	360	25	0.002631756826994637	0.03542749574800473	ICMT:DPH5:RBM15:SETDB1:ASH1L:LMNA:NSUN6:ARL5B-AS1:CTR9:EMG1:METTL7A:TDRD9:CIAPIN1:PRMT7:METTL16:BRCA1:TRMT61B:METTL21A:NDUFAF5:THUMPD3:TRMT10A:DIMT1:INMT:MEPCE:ASH2L:NTMT1
GO_bp	GO_MODIFICATION_DEPENDENT_MACROMOLECULE_CATABOLIC_PROCESS	623	38	0.0026460185395787024	0.03555436246051821	EXOSC10:CDC20:DCST1:UBQLN4:CUL2:UBE2L6:SYVN1:USP5:SPSB2:FBXW8:SIAH3:UCHL3:DCAF11:PSME1:FBXO33:PSEN1:HERC2:OTUD7A:NTAN1:TGFB1I1:SIAH1:N4BP1:DERL2:TBX21:TRIM25:PSMD12:NPLOC4:KEAP1:GIPC1:GSK3A:CBLC:C19orf68:ANAPC1:USP25:GSK3B:ARMC8:TNFAIP3:UBQLN1
GO_bp	GO_RESPONSE_TO_ACTIVITY	64	8	0.002682803460540633	0.0359828566331636	MTOR:BGLAP:UCP3:POSTN:CREB1:EDN1:STAR:SLC25A25
GO_bp	GO_REGULATION_OF_MAPK_CASCADE	751	44	0.002747247193313895	0.03678008537496745	ASH1L:ZMYND11:GDF2:PSAP:FAS:DUSP8:SMPD1:CDON:PTPN6:PHB2:RAP1B:TNFRSF19:MAP3K9:PSEN1:CCL17:DHX33:NBR1:SPAG9:PHLPP1:INSR:PKN1:CXCL17:CEACAM1:CBLC:FPR2:ALK:FSHR:LIME1:TAB1:FBLN1:MAPK8IP2:GNAI2:HYAL2:DVL3:MAP3K13:NPY5R:HDAC3:RELL2:PDGFRB:EDN1:CD36:RB1CC1:LYN:KLF4
GO_bp	GO_LYMPHOCYTE_ACTIVATION	709	42	0.002781131173956279	0.03716602568832482	MTOR:CDC42:TXLNA:MPL:BCL10:VAV3:TNFAIP8L2:GON4L:SEMA4A:C10orf54:GPAM:PTPN6:NCKAP1L:LCP1:TNFSF13B:PSEN1:CORO1A:IKZF3:TBX21:TCF3:CD320:PKN1:IL12RB1:CD79A:POU2F2:CEACAM1:KDELR1:IL18R1:MERTK:IGFBP2:SLC11A1:NCAPH2:TGFBR2:TUSC2:FOXP1:DROSHA:PTGER4:NDFIP1:TNFAIP3:LAT2:LYN:CDH17
GO_bp	GO_ENZYME_LINKED_RECEPTOR_PROTEIN_SIGNALING_PATHWAY	1034	57	0.0028131432908954897	0.037525595622653084	NBL1:CDC42:TIE1:RPE65:F3:VAV3:TRIM33:ARNT:FMOD:NUCKS1:PSEN2:GDF2:ZFYVE27:SFRP5:UBASH3B:AKAP3:GPRC5A:NCKAP1L:PSEN1:SKOR1:TGFB1I1:POLR2C:ESRP2:SMPD3:FAM83G:EPN2:RPS6KB1:SKOR2:INSR:ARHGEF18:GIPC1:EPS15L1:GSK3A:MEGF8:CEACAM1:CBLC:ALK:ATP6V1B1:MERTK:CREB1:IGFBP2:IGFBP5:VIL1:TAB1:TGFBR2:GNAI2:AP2M1:FGF12:AREG:AREGB:PDGFRB:DACT2:HSPB1:PILRB:BLK:GFRA2:LYN:PTPN3
GO_bp	GO_PROTEIN_LOCALIZATION_TO_NUCLEUS	248	19	0.0028284176237105127	0.03766099553310193	TARDBP:LMNA:IPO7:ARL2:PHB2:KPNA3:IPO5:PSEN1:KPNB1:XPO1:GTSE1:HYAL2:GSK3B:TERT:IPO11:HDAC3:SYNE1:CD36:XPA
GO_bp	GO_ADAPTIVE_IMMUNE_RESPONSE_BASED_ON_SOMATIC_RECOMBINATION_OF_IMMUNE_RECEPTORS_BUILT_FROM_IMMUNOGLOBULIN_SUPERFAMILY_DOMAINS	343	24	0.002875006919409095	0.03821211728328544	MTOR:BCL10:SEMA4A:APCS:HPX:PTPN6:C1S:TNFSF13B:TBX21:PKN1:IL12RB1:POU2F2:CEACAM1:KDELR1:IL1RL1:IL18R1:IL18RAP:SLC11A1:KLHL6:CFI:NDFIP1:HLA-A:C4A:TNFAIP3
GO_bp	GO_CELL_CYCLE_G2_M_PHASE_TRANSITION	267	20	0.0028856132392235	0.03828385795720708	WEE1:CIT:RNASEH2B:PSME1:ARPP19:BRCA1:PSMD12:AZI1:TUBB4A:PPP1CB:DCTN1:GTSE1:TUBB:CDKN1A:FGFR1OP:YWHAG:ABCB1:PRKAR2B:CEP41:TUBB4B
GO_bp	GO_MUSCLE_TISSUE_DEVELOPMENT	402	27	0.0029003176785941374	0.038409612500300734	MTOR:GJA5:LMNA:TLL2:TENM4:YAP1:CDON:P2RX2:MYLPF:GJC1:RPS6KB1:NPHS1:GSK3A:OSR1:CREB1:IGFBP5:PPARA:TGFBR2:KCNAB1:ISL1:MEGF10:PDGFRB:EDN1:FLOT1:POPDC3:KEL:GTF3C5
GO_bp	GO_NATURAL_KILLER_CELL_MEDIATED_IMMUNITY	65	8	0.0029603522479144466	0.03913415291757407	CRTAM:PTPN6:CORO1A:SERPINB4:CEACAM1:IL18RAP:TUBB:TUBB4B
GO_bp	GO_IN_UTERO_EMBRYONIC_DEVELOPMENT	344	24	0.002983499290741847	0.03924171840053913	TIE1:STIL:HORMAD1:ARNT:CTR9:AKAP3:EMG1:FBXW8:RNASEH2B:PKD1:RRN3:ANKRD11:B9D1:HNF1B:KEAP1:NCOA1:RTCB:TAB1:TGFBR2:SLC34A2:MGAT1:EDN1:NCAPG2:RDH10

GO_bp	GO_MONOCARBOXYLIC_ACID_TRANSPORT	160	14	0.0029898452114696483	0.03924171840053913	MFSD2A:ABCD3:SLC16A4:SLC16A12:SLC51B:RPS6KB1:CEACAM1:NCOA1:PPARA:CPT1B:SLC10A4:PLA2G12A:EDN1:CD36
GO_bp	GO_MODIFICATION_OF_MORPHOLOGY_OR_PHYSIOLOGY_OF_OTHER_ORGANISM	160	14	0.0029898452114696483	0.03924171840053913	TARDBP:APCS:NUCKS1:PC:KPNA3:KPNB1:INSR:SEMG1:SLPI:APOL1:HYAL2:TUSC2:CXCL6:SCRIB
GO_bp	GO_CELL_KILLING	160	14	0.0029898452114696483	0.03924171840053913	CRTAM:PTPN6:CORO1A:SERPINB4:IL12RB1:CEACAM1:IL18RAP:SEMG1:APOL1:TUSC2:CXCL6:HLA-A:TUBB:TUBB4B
GO_bp	GO_MAINTENANCE_OF_LOCATION	325	23	0.0030192774332223516	0.03955737813580086	NBL1:SOAT1:ASPM:ARL2:DGAT2:UBASH3B:PTPN6:FITM1:ERO1L:MYO5A:KIAA1199:PKD1:CORO1A:TRPV1:KEAP1:KDELR1:LIME1:PPARA:DIAPH1:SYNE1:CD36:MEST:LYN
GO_bp	GO_CELLULAR_RESPONSE_TO_DNA_DAMAGE_STIMULUS	841	48	0.003028814877572777	0.03961172482234859	VAV3:HIPK1:PHLDA3:NUCKS1:MCM10:APBB1:UBE2L6:CDCA5:YAP1:NUAK1:POLE:RNASEH2B:ERCC5:FANCM:PSEN1:MOAP1:HERC2:AQR:SMG1:SLX1A:ZNF771:POLR2C:BRCA1:TRIM25:C17orf70:NPLOC4:CHAF1A:SPDYA:COPS8:PCNA:GTSE1:RAD18:FOXP1:ISY1:ASTE1:NEK11:NIPBL:WRNIP1:MDC1:IER3:CDKN1A:GTF2H5:RFC2:ASH2L:LYN:GML:XPA:NACC2
GO_bp	GO_CILIARY_BASAL_BODY_PLASMA_MEMBRANE_DOCKING	95	10	0.003064837807309686	0.03994070546759963	B9D1:AZI1:TUBB4A:DCTN1:TUBB:FGFR1OP:YWHAG:PRKAR2B:CEP41:TUBB4B
GO_bp	GO_RESPONSE_TO_ANTINEOPLASTIC_AGENT	95	10	0.003064837807309686	0.03994070546759963	ADAM15:HCN3:PCK2:BRCA1:RPS6KB1:PCNA:XRN1:EIF4E:EDN1:STAR
GO_bp	GO_ACTIVATION_OF_IMMUNE_RESPONSE	692	41	0.003089552633925602	0.04019152541478438	CDC42:BCL10:VAV3:NRAS:CTSS:CTSK:APCS:FCGR2A:FCGR3A:CFHR5:DMBT1:PTPN6:C1S:NCKAP1L:PSME1:PSEN1:CLEC10A:PSMD12:NPLOC4:MUC16:CD79A:CEACAM1:FPR2:PUM2:LIME1:TAB1:FOXP1:PROS1:KLHL6:CFI:FLOT1:MUC21:C4A:TNFAIP3:ESR1:LAT2:CD36:BLK:LYN:UBQLN1:FCN2
GO_bp	GO_REGULATION_OF_SUPRAMOLECULAR_FIBER_ORGANIZATION	345	24	0.0030954282981923136	0.04019681623977651	MTOR:CDC42:PRUNE:TMOD4:ARHGEF2:ARL2:HSPA8:NCKAP1L:CIT:FAM179B:PSEN1:CORO1A:TUBB4A:NPHS1:DCTN1:VIL1:DSTN:SHANK3:NAT6:SLAIN2:PTGER4:FCHSD1:EDN1:LRRC16A
GO_bp	GO_PEROXISOME_ORGANIZATION	80	9	0.003102100713167395	0.04021241665216994	ABCD3:SEC16B:PEX16:DAO:LONP2:PEX11G:PEX13:MFF:PEX7
GO_bp	GO_NEGATIVE_REGULATION_OF_DEPHOSPHORYLATION	111	11	0.003131761673860371	0.04045421494353907	SPOCD1:CSRNP2:NCKAP1L:PPP1R36:ZFYVE1:ARPP19:CD2BP2:TSKS:GNAI2:GSK3B:PPP1R35
GO_bp	GO_MODIFICATION_OF_MORPHOLOGY_OR_PHYSIOLOGY_OF_OTHER_ORGANISM_INVOLVED_IN_SYMBIOTIC_INTERACTION	111	11	0.003131761673860371	0.04045421494353907	TARDBP:APCS:NUCKS1:PC:KPNA3:KPNB1:INSR:HYAL2:TUSC2:CXCL6:SCRIB
GO_bp	GO_DEVELOPMENT_OF_PRIMARY_SEXUAL_CHARACTERISTICS	214	17	0.0031744695507372554	0.04093394947003303	CSDE1:ADAM15:ASPM:FNDC3A:SAFB2:INSR:OSR1:NCOA1:FSHR:HYAL3:PDGFRB:ESR1:HOXA10:HOXA11:STAR:RDH10:LRRC6
GO_bp	GO_POSTREPLICATION_REPAIR	52	7	0.0031995499959976835	0.04096984750972644	UBE2L6:BRCA1:TRIM25:NPLOC4:PCNA:RAD18:RFC2
GO_bp	GO_POSITIVE_REGULATION_OF_REACTIVE_OXYGEN_SPECIES_BIOSYNTHETIC_PROCESS	52	7	0.0031995499959976835	0.04096984750972644	MTOR:DDAH1:TRPV1:INSR:EDN1:CD36:KLF4

GO_bp	GO_NEGATIVE_REGULATION_OF_ERBB_SIGNALING_PATHWAY	52	7	0.0031995499959976835	0.04096984750972644	CDC42:GPRC5A:PSEN1:EPS15L1:CBLC:AREG:AREGB:PTPN3
GO_bp	GO_DNA_SYNTHESIS_INVOLVED_IN_DNA_REPAIR	52	7	0.0031995499959976835	0.04096984750972644	UBE2L6:POLE:TRIM25:NPLOC4:PCNA:WRNIP1:RFC2
GO_bp	GO_REGULATION_OF_INTRACELLULAR_TRANSPORT	425	28	0.0032155463760639657	0.04110307106794808	TARDBP:LDLRAP1:UHMK1:SEC16B:RAP1B:SIAH3:LCP1:IPO5:SCFD1:PSEN1:NUMB:SLC51B:KIAA1199:DERL2:NPEPPS:AZI1:GIPC1:GSK3A:XPO1:MFF:GTSE1:HYAL2:GSK3B:HDAC3:CNR1:CD36:LYN:SCRIB
GO_bp	GO_POSITIVE_REGULATION_OF_NUCLEAR_DIVISION	66	8	0.0032597460470094016	0.04159571778735956	CDCA5:PHB2:DLGAP5:SMPD3:INSR:NIPBL:PDGFRB:EDN1
GO_bp	GO_CYTOKINE_MEDIATED_SIGNALING_PATHWAY	780	45	0.003295029653628154	0.0419730813763725	CDC42:TXLNA:TRIM62:MPL:F3:RBM15:HIPK1:DCST1:HPX:CTR9:HSPA8:PTPN6:RAP1B:TNFRSF19:LCP1:TNFSF13B:PSME1:BOLA2B:CCL17:IFI35:TRIM25:PSMD12:IL12RB1:CEACAM1:CARD8:IL1RL1:IL18R1:IL18RAP:ZBP1:TAB1:ACKR2:ACKR4:PYDC2:IL8:CXCL6:PF4V1:CXCL1:EDN1:HLA-A:CDKN1A:GSTA2:TNFAIP3:SOD2:CD36:SCRIB
GO_bp	GO_OXIDATION_REDUCTION_PROCESS	954	53	0.0033157181259488404	0.04216354364312107	MTOR:TXNDC12:RPE65:ABCD3:PHGDH:ALDH18A1:BBOX1:TM7SF2:DGAT2:FDX1:NDUFA9:DAO:ERO1L:JMJD7:LONP2:CIAPIN1:COQ9:CYB5B:VAT1:INSR:TECR:PRODH2:GSK3A:TPO:PPP1CB:PEX13:MTHFD2:PER2:PANK2:NDUFAF5:NDUFA6:CYP2D6:CYB5R3:PPARA:MIOX:SCO2:CPT1B:CYP8B1:CYB561D2:XXcos-LUCA11.4:DHFRL1:GSK3B:NDUFB4:ACAD11:KCNAB1:C5orf63:CNR1:PEX7:SOD2:DUS4L:RDH10:CYP11B1:CYP11B2:SLC25A25
GO_bp	GO_EAR_DEVELOPMENT	215	17	0.0033294168943655655	0.04226461860723127	CDH23:PSAP:LGR5:FOXG1:RPL38:OSR1:ATP6V1B1:CELSR1:LRIG1:NIPBL:C5orf20:TIFAB:PDGFRB:EDN1:RDH10:CALB1:SCRIB
GO_bp	GO_HEMATOPOIETIC_PROGENITOR_CELL_DIFFERENTIATION	162	14	0.003346455872676471	0.04240767355891735	TAL1:YAP1:PTPN6:ARL11:PSME1:PSEN1:TCF12:SLC7A6OS:SMPD3:PSMD12:TCF3:HYAL2:PDGFRB:DACT2
GO_bp	GO_MOLTING_CYCLE	112	11	0.0033575106490019023	0.04247453230665057	TMEM79:SNRPE:HOXC13:LGR5:TNFRSF19:MYO5A:IGFBP5:CELSR1:LRIG1:TERT:NIPBL
GO_bp	GO_REGULATION_OF_COAGULATION	81	9	0.003375816534744666	0.04255960811384785	F3:SELP:UBASH3B:PSEN1:CEACAM1:PROS1:EDN1:CD36:LYN
GO_bp	GO_RNA_METHYLATION	81	9	0.003375816534744666	0.04255960811384785	RBM15:NSUN6:ARL5B-AS1:EMG1:METTL16:TRMT61B:THUMPD3:TRMT10A:DIMT1:MEPCE
GO_bp	GO_REGULATION_OF_TRANSFERASE_ACTIVITY	956	53	0.003458035196451567	0.04352150461287503	MTOR:CDC20:TAL1:BCL10:VAV3:CKS1B:DUSP8:SMPD1:IPO7:UBASH3B:PTPN6:GPRC5A:NCKAP1L:IPO5:MAP3K9:PSEN1:KIAA1199:PKD1:SPAG9:INSR:PKN1:GSK3A:CXCL17:CEACAM1:CBLC:FPR2:SPDYA:ALK:SLC11A1:COPS8:LIME1:TAB1:TGFBR2:HYAL2:NPRL2:GSK3B:DVL3:MAP3K13:CCNO:CKS1B:PDGFRB:EDN1:CDKN1A:TNFAIP3:FGFR1OP:RFC2:HSPB1:YWHAG:PILRB:PRKAR2B:NCAPG2:LYN:TERF1:KLF4
GO_bp	GO_UROGENITAL_SYSTEM_DEVELOPMENT	329	23	0.00350949324255799	0.044093633047523464	WDR77:NID1:PSAP:ACTA2:YAP1:HOXC11:LGR5:APAF1:PKD1:HNF1B:NPHS1:OSR1:HYAL2:NPHP3:PSAPL1:NIPBL:PDGFRB:ESR1:DACT2:HOXA11:RDH10:CA2:CALB1
GO_bp	GO_CELLULAR_PROTEIN_CONTAINING_COMPLEX_ASSEMBLY	1112	60	0.003541524143983494	0.04436112609538955	MTOR:PTBP2:WDR77:PRUNE:TMOD4:KIAA0907:APCS:FMOD:SNRPE:ARL2:NDUFA9:NCKAP1L:ERCC5:STXBP6:FAM179B:PRPF39:RPL3L:RRN3:CORO1A:CD2BP2:PRMT7:DHX33:HNF1B:RPL38:DNAI2:C17orf89:CHAF1A:TUBB4A:NPHS1:GRWD1:PUM2:DCTN1:CREB1:VIL1:COPS8:NDUFAF5:EIF6:NDUFA6:SCO2:MAPK8IP2:ZMYND10:SHQ1:NDUFB4:ISY1:PYDC2:SLAIN2:DIAPH1:FCHSD1:LRRC16A:PFDN6:TAPBP:ESR1:GTF2H5:CD36:PILRB:TERF1:LRRC6:XPA:RPL12:GTF3C5
GO_bp	GO_MYELOID_CELL_DIFFERENTIATION	408	27	0.003545664011942238	0.04436112609538955	MTOR:CDC42:MPL:TAL1:RBM15:ARNT:BGLAP:APCS:CTR9:UBASH3B:PTPN6:SLC11A2:NCKAP1L:PDE1B:PSEN1:TNRC6C:CEACAM1:CREB1:EIF6:TGFBR2:FOXP1:NDFIP1:L3MBTL3:NCAPG2:ASH2L:LYN:CA2
GO_bp	GO_RESPONSE_TO_LIGHT_STIMULUS	310	22	0.003548890087631164	0.04436112609538955	MTOR:RPE65:RGR:ABCC8:PDE1B:ERCC5:TRPM1:GRIN2A:N4BP1:RCVRN:MFAP4:PPP1CB:CREB1:PER2:PCNA:RAD18:HYAL3:HYAL1:HYAL2:CCDC66:CDKN1A:XPA

GO_bp	GO_REGULATION_OF_RESPONSE_TO_DRUG	97	10	0.0035671218109179503	0.044437873407198195	SYT11:TACR2:PSAP:SYT13:OR5T1:PSEN1:OSR1:NCOA1:PER2:CNR1
GO_bp	GO_DIGESTIVE_SYSTEM_PROCESS	97	10	0.0035671218109179503	0.044437873407198195	MOGAT2:TRPV1:PTGER1:SLC9A4:VIL1:TYMP:C5orf20:TIFAB:CD36:CEL
GO_bp	GO_EXCRETION	67	8	0.0035821143604461347	0.044549137985243806	TACR2:PSAP:CHRNB4:TRPV1:NPHS1:ATP6V1B1:GNAI2:EDN1
GO_bp	GO_LIPID_MODIFICATION	235	18	0.003604558689906375	0.0447525445452903	MTOR:A3GALT2:ABCD3:SOAT1:DGAT2:MTMR6:SMG1:LONP2:DGKE:IMPA2:PEX13:PPARA:CPT1B:ACAD11:PPAP2A:CNR1:PEX7:PIP5KL1
GO_bp	GO_SODIUM_ION_TRANSPORT	217	17	0.0036581064247497496	0.045264448185034785	HCN3:LINC00610:COMMD9:NDUFA9:SLC5A10:OSR1:ATP6V1B1:SLC4A5:SLC9A4:SCN3A:SCN5A:FGF12:SLC34A2:SLC10A4:SLC4A4:SLC12A2:PTPN3:SLC34A3
GO_bp	GO_CYTOKINE_SECRETION	217	17	0.0036581064247497496	0.045264448185034785	SYT11:GPAM:CRTAM:POSTN:MAPKBP1:MBP:CARD8:IL1RL1:HYAL2:FOXP1:CD200R1:PYDC2:PTGER4:TNFAIP3:CD36:LYN:PAEP
GO_bp	GO_SENSORY_PERCEPTION_OF_MECHANICAL_STIMULUS	164	14	0.003737067561874981	0.04579125547000767	CDH23:PSAP:KCNA1:P2RX2:COCH:KIAA1199:TRPV1:RPL38:MBP:ATP6V1B1:LRIG1:NIPBL:DIAPH1:GRXCR2
GO_bp	GO_ACTION_POTENTIAL	130	12	0.0037423930657218135	0.04579125547000767	RNF207:GJA5:KCNA1:CHRNB4:GJC1:KCNJ2:SCN3A:SCN5A:FGF12:CNR1:CD36:PTPN3
GO_bp	GO_VITAMIN_METABOLIC_PROCESS	130	12	0.0037423930657218135	0.04579125547000767	UBIAD1:RPE65:PC:AASDHPPT:CD320:MTHFD2:PANK2:CYB5R3:DHFRL1:RBP2:RBP1:RDH10
GO_bp	GO_PEROXISOME_FISSION	9	3	0.003762982082161175	0.04579125547000767	SEC16B:PEX11G:MFF
GO_bp	GO_MULTICELLULAR_ORGANISMAL_IRON_ION_HOMEOSTASIS	9	3	0.003762982082161175	0.04579125547000767	SLC11A2:SLC11A1:HYAL2
GO_bp	GO_MESONEPHRIC_DUCT_DEVELOPMENT	9	3	0.003762982082161175	0.04579125547000767	PKD1:HNF1B:OSR1
GO_bp	GO_RETINAL_ROD_CELL_DEVELOPMENT	9	3	0.003762982082161175	0.04579125547000767	NRL:TRPM1:CCDC66
GO_bp	GO_RESPONSE_TO_L_GLUTAMATE	9	3	0.003762982082161175	0.04579125547000767	BCL11A:CREB1:PCNA
GO_bp	GO_REGULATION_OF_TOLL_LIKE_RECEPTOR_3_SIGNALING_PATHWAY	9	3	0.003762982082161175	0.04579125547000767	FLOT1:TNFAIP3:UBQLN1
GO_bp	GO_REGULATION_OF_GERMINAL_CENTER_FORMATION	9	3	0.003762982082161175	0.04579125547000767	TNFSF13B:PKN1:TNFAIP3
GO_bp	GO_REGULATION_OF_CELL_SHAPE	147	13	0.003779987569729824	0.04592216303721357	FBLIM1:CDC42:SEMA4A:COCH:CORO1A:RND2:ARHGEF18:RND3:VIL1:PLXNB2:DIAPH1:SEPT7:PALM2
GO_bp	GO_REGULATION_OF_PROTEASOMAL_PROTEIN_CATABOLIC_PROCESS	182	15	0.003828859610319935	0.04643913883803882	UBQLN4:USP5:FBXW8:PSME1:PSEN1:PKD1:N4BP1:DERL2:RNFT1:KEAP1:GIPC1:GSK3A:USP25:GSK3B:UBQLN1

GO_bp	GO_REGULATION_OF_PHOSPHOPROTEIN_PHOSPHATASE_ACTIVITY	114	11	0.003848086771732027	0.04659544937764481	MTOR:NCKAP1L:NUAK1:ANKLE2:PPP1R36:ARPP19:PPP4R1:GNAI2:GSK3B:PDGFRB:PPP1R35
GO_bp	GO_HOMOPHILIC_CELL_ADHESION_VIA_PLASMA_MEMBRANE_ADHESION_MOLECULES	165	14	0.003945849108565313	0.04769844961768717	CLSTN1:CDH23:PCDH9:PKD1:CEACAM1:CELSR1:PLXNB2:CDH9:CDH6:PCDHGA12:PCDHGC3:PCDHGC4:PCDHGC5:CDH17
GO_bp	GO_REGULATION_OF_HORMONE_LEVELS	513	32	0.003952157254036937	0.04769844961768717	TARDBP:RPE65:ARNT:TACR2:KCNJ11:ABCC8:ARL2:UCP2:DGAT2:FDX1:MYO5A:SMPD3:HNF1B:SAFB:CHST8:TPO:PLB1:CREB1:PER2:MYRIP:CACNA2D2:ISL1:EDN1:CNR1:ESR1:BLK:STAR:LYN:RDH10:ENY2:CYP11B1:CYP11B2
GO_bp	GO_EPIDERMAL_CELL_DIFFERENTIATION	352	24	0.0039824427890776245	0.04783465597639646	CTSK:HRNR:FLG:LCE1F:LCE1E:LCE1D:LCE1C:LCE1B:TMEM79:PSAP:KRTAP5-1:KRTAP5-2:KRTAP5-3:KRTAP5-4:KRTAP5-5:YAP1:KEAP1:CNFN:PKP4:PI3:KRTAP21-3:KRTAP8-1:SCRIB:KLF4
GO_bp	GO_NUCLEAR_ENVELOPE_REASSEMBLY	18	4	0.003997920204849549	0.04783465597639646	LMNA:REEP3:ANKLE2:CHMP4B
GO_bp	GO_REGULATION_OF_FATTY_ACID_BETA_OXIDATION	18	4	0.003997920204849549	0.04783465597639646	MTOR:LONP2:PPARA:CNR1
GO_bp	GO_REGULATION_OF_DEFENSE_RESPONSE_TO_BACTERIUM	18	4	0.003997920204849549	0.04783465597639646	SYT11:MAPKBP1:FOXP1:CXCL6
GO_bp	GO_EYE_MORPHOGENESIS	148	13	0.004004905411736852	0.04783465597639646	FOXE3:RPE65:OLFM3:HIPK1:CRB1:CDON:C12orf57:NRL:TRPM1:FSCN2:CCDC66:NIPBL:CALB1
GO_bp	GO_MULTICELLULAR_ORGANISM_GROWTH	148	13	0.004004905411736852	0.04783465597639646	MTOR:STIL:GPAM:SMPD3:ANKRD11:CREB1:VIL1:CACNA2D2:PLAC8:NPY1R:NIPBL:XPA:SLC25A25
GO_bp	GO_REGULATION_OF_LEUKOCYTE_PROLIFERATION	219	17	0.004013109842483305	0.04783465597639646	MPL:VAV3:C10orf54:GPAM:PTPN6:NCKAP1L:TNFSF13B:CORO1A:IKZF3:CD320:PKN1:IL12RB1:IGFBP2:TGFBR2:NDFIP1:TNFAIP3:LYN
GO_bp	GO_RESPONSE_TO_GROWTH_FACTOR	724	42	0.004015507855433554	0.04783465597639646	NBL1:TRIM33:ARNT:BGLAP:FMOD:GDF2:ZFYVE27:SFRP5:SOX5:APAF1:POSTN:SKOR1:ADAMTS7:CORO1A:TGFB1I1:POLR2C:ESRP2:SMPD3:TRPV1:FAM83G:EPN2:MAPK7:RPS6KB1:SKOR2:INSR:ARHGEF18:GIPC1:MEGF8:CREB1:VIL1:NCL:TAB1:TGFBR2:HYAL1:HYAL2:FGF12:IL8:PDGFRB:EDN1:HSPB1:STAR:KLF4
GO_bp	GO_MULTICELLULAR_ORGANISMAL_SIGNALING	201	16	0.004047235156514445	0.04813459288087569	RNF207:GJA5:KCNJ11:KCNA1:CHRNB4:CACNB1:GJC1:CACNG1:KCNJ2:KCNJ14:SCN3A:TYMP:SCN5A:CACNA2D2:FGF12:KCNIP1
GO_bp	GO_DE_NOVO_PROTEIN_FOLDING	41	6	0.004080805317365206	0.04845544278293096	DNAJB4:HSPA6:DNAJB13:HSPA8:ERO1L:DNAJB1
GO_bp	GO_STEM_CELL_PROLIFERATION	115	11	0.004113949295625721	0.04877020535943396	ARHGEF2:ASPM:YAP1:KCNA1:SOX5:FOXG1:NUMB:FBLN1:PLXNB2:TERT:ABCB1
GO_bp	GO_CELL_FATE_COMMITMENT	257	19	0.004171683199639593	0.04934551108845276	MTOR:TAL1:ARHGEF2:CTR9:DBX1:TENM4:CDON:SOX5:FOXG1:PSEN1:TGFB1I1:HNF1B:TBX21:TCF3:IL12RB1:ISL1:HOXA11:EVX1:KLF4
GO_bp	GO_LIPOPROTEIN_METABOLIC_PROCESS	29	5	0.0041826195113069484	0.04934551108845276	DGAT2:APOL2:APOL1:PPARA:MTTP
GO_bp	GO_NEGATIVE_REGULATION_OF_PROTEIN_TYROSINE_KINASE_ACTIVITY	29	5	0.0041826195113069484	0.04934551108845276	GPRC5A:PSEN1:CBLC:HYAL2:FGFR1OP

GO_bp	GO_ENDOSOMAL_TRANSPORT	220	17	0.004200961144415457	0.049482475018355135	TMEM50A:VPS51:ERC1:HEATR5A:TBC1D10B:SPAG9:UBXN6:DENND1C:DCTN1:TMEM87B:CHMP4B:FAM109B:RAB6B:VPS52:DENND2A:SCRIB:CHMP5
GO_bp	GO_MORPHOGENESIS_OF_EMBRYONIC_EPITHELIUM	149	13	0.004240512958104427	0.04977461718137696	STIL:BCL10:APAF1:TGFB1I1:HNF1B:OSR1:CELSR1:PLXNB2:IFT57:NPHP3:DVL3:RDH10:SCRIB
GO_bp	GO_VESICLE_MEDIATED_TRANSPORT_IN_SYNAPSE	202	16	0.004246079588125626	0.04977461718137696	CLSTN1:SYT11:RAP1B:PSEN1:NUMB:SH3GL1:GIPC1:TMEM230:DNAJC5:STX19:GSK3B:SNCB:CNR1:PPP3CC:SCRIB:CEL
GO_bp	GO_MYELOID_LEUKOCYTE_DIFFERENTIATION	202	16	0.004246079588125626	0.04977461718137696	MTOR:CDC42:TAL1:BGLAP:APCS:UBASH3B:PDE1B:PSEN1:CEACAM1:CREB1:TGFBR2:FOXP1:NDFIP1:L3MBTL3:LYN:CA2
GWAScatalog	Body	mass	index	1365	116	2.4548587794681247e-16
GWAScatalog	Blood	protein	levels	1935	144	3.578216889817222e-15
GWAScatalog	Body	fat	distribution	(leg	fat	ratio)
GWAScatalog	Body	fat	distribution	(trunk	fat	ratio)
GWAScatalog	Rhegmatogenous	retinal	detachment	20	11	2.5201947989951598e-11
GWAScatalog	Crohn's	disease	630	60	6.012267862228627e-11	1.81871102832416e-08
GWAScatalog	Inflammatory	bowel	disease	730	64	4.3221413998414945e-10
GWAScatalog	Sleep	duration	(short	sleep)	99	18
GWAScatalog	Multiple	sclerosis	243	28	1.6906193577182752e-07	3.4094157047318546e-05
GWAScatalog	Heel	bone	mineral	density	834	62
GWAScatalog	Autism	15	7	5.180172029829482e-07	8.547283849218645e-05	TRIM33:BCAS2:DENND2C:AMPD1:NRAS:CSDE1:CDH9
GWAScatalog	Systolic	blood	pressure	793	54	2.360747090459456e-05
GWAScatalog	Intelligence	55	10	3.430445076254477e-05	0.004568384230168469	CSMD2:ZNF638:AFF3:WBP2NL:NAGA:FAM109B:NDUFA6:CYP2D6:DAZL:EXOC4
GWAScatalog	Frontotemporal	dementia	18	6	3.523822546686423e-05	0.004568384230168469
GWAScatalog	Mean	corpuscular	hemoglobin	concentration	121	15
GWAScatalog	Chronic	inflammatory	diseases	(ankylosing	spondylitis,	Crohn's
GWAScatalog	Asthma	or	allergic	disease	(pleiotropy)	170
GWAScatalog	Hip	circumference	adjusted	for	BMI	218
GWAScatalog	Clozapine-induced	agranulocytosis	70	10	0.000279492747338168	0.025693357428199514
GWAScatalog	Red	cell	distribution	width	221	20

GWAScatalog	Beard	thickness	36	7	0.0003362792943386724	0.02754256295346535
GWAScatalog	Plasma	omega-6	polyunsaturated	fatty	acid	levels
GWAScatalog	Polymyositis	10	4	0.0003490242137353736	0.02754256295346535	RPL38:IL18R1:FAM167A:BLK
GWAScatalog	Iris	color	(L*	coordinate)	27	6
GWAScatalog	Lung	cancer	214	19	0.0005033885316813388	0.0365460074000652
GWAScatalog	Height	898	54	0.000541580656651125	0.03689921170563887	ACOT11:PHGDH:DCST1:CRB1:GRK5:ERC1:SOX5:DLEU1:TNFSF13B:SIX6:CPSF2:SH3GL3:ACAN:PKD1:PDXDC1:NTAN1:SIAH1:NPEPPS:C17orf67:DGKE:TRIM25:INSR:ZNF638:AFF3:TTLL4:RALGAPA2:MMP24:EIF6:ZNFX1:CHKB:VGLL4:HDAC11:CMTM8:FAM208A:FOXP1:RASA2:FNDC3B:SENP2:C3orf65:NIPBL:SLC12A2:HLA-A:NRM:POPDC3:L3MBTL3:ESR1:ARL4A:JAZF1:PRKAR2B:LYN:CYP11B1:IPPK:SLC35D2:PAPPA
GWAScatalog	Chronic	lymphocytic	leukemia	76	10	0.0005489138931417352
GWAScatalog	Primary	biliary	cholangitis	105	12	0.000594513525343263
GWAScatalog	Prostate	cancer	381	28	0.0006469339452652606	0.03741844196844301
GWAScatalog	Chronotype	556	37	0.000657127174933989	0.03741844196844301	HTR6:LPHN2:PTBP2:TRIM33:BCAS2:GOLPH3L:LARP4B:UBASH3B:SOX5:CCER1:PCDH9:LRFN5:NFATC3:TUBD1:PITPNC1:SKOR2:POU2F2:FSHR:BCL11A:RBMS1:HES6:PER2:RAD18:CACNA2D2:FOXP1:CD200R1:MRAS:C3orf55:STIM2:TMEM161B:ZNF608:HLA-A:HCRTR2:EXOC4:CALB1:KHDRBS3:IZUMO3
GWAScatalog	Selective	IgA	deficiency	40	7	0.0006589108394981873
GWAScatalog	Weight	185	17	0.000659719087046929	0.03741844196844301	UBIAD1:SEC16B:NUCKS1:DLEU1:PDXDC1:NTAN1:PRMT7:CHST8:CBLC:RBMS1:NEK11:RASA2:NIPBL:MGAT1:PPIL1:L3MBTL3:PALM2
GWAScatalog	Offspring	birth	weight	153	15	0.0006876647733658853
GWAScatalog	Spherical	equivalent	(joint	analysis	main	effects
GWAScatalog	Benign	prostatic	hyperplasia	and	lower	urinary
GWAScatalog	Systemic	lupus	erythematosus	and	Systemic	sclerosis
GWAScatalog	Diffusing	capacity	of	the	lung	for
GWAScatalog	Waist-to-hip	ratio	adjusted	for	BMI	x
GO_cc	GO_INTRINSIC_COMPONENT_OF_PLASMA_MEMBRANE	1697	131	4.685533984802733e-15	4.6902195187875354e-12	CLSTN1:HTR6:RHCE:MFSD2A:TIE1:MPL:TSPAN1:TM2D1:LPHN2:LRRC8B:LRRC8C:F3:OLFM3:KCNC4:SLC16A4:GJA5:SEMA6C:HCN3:SYT11:SEMA4A:SELP:PSEN2:TACR2:RGR:SLC16A12:KCNJ11:ABCC8:SYT13:OR5T1:SLC43A1:TM7SF2:RCE1:LRFN4:TENM4:TRPC6:CDON:KCNA1:GPRC5A:SLC11A2:NCKAP1L:LGR5:SLC25A3:ORAI1:P2RX2:PCDH9:LRFN5:PSEN1:KCNK10:TRPM1:CHRNB4:PKD1:GRIN2A:ABCC11:SLC7A6:TRPV1:CACNG1:KCNJ16:KCNJ2:SSTR2:SLC14A1:INSR:CD320:CD97:PTGER1:OR10H2:OR10H3:IL12RB1:NPHS1:CEACAM1:BCAM:GRIN2D:KCNJ14:CD33:SIGLEC6:TPO:ALK:FSHR:SLC4A5:IL18R1:IL18RAP:MERTK:SCN3A:SLC11A1:MMP24:TGM2:KCNS1:MC3R:MCHR1:CELSR1:PLXNB2:SHANK3:TGFBR2:SCN5A:ACKR2:HYAL2:GP9:ACKR4:KCNAB1:MFI2:SLC34A2:SLC4A4:NPY1R:NPY5R:SLC6A19:SLC6A18:CDH9:PPAP2A:SLC12A2:PCDHGA12:PCDHGC3:PCDHGC4:PCDHGC5:PDGFRB:KCNIP1:TSPAN17:HCRTR2:CNR1:ITGB8:CRHR2:CLDN3:CLDN4:CD36:PILRB:GPR22:BCAP29:ESYT2:LYN:SCRIB:KCNV2:S1PR3:CEL
GO_cc	GO_NEURON_PART	1709	129	4.0335970502345616e-14	2.018815323642398e-11	CLSTN1:MTOR:HTR6:CDC42:TMEM50A:TMEM57:LDLRAP1:MPL:LPHN2:PTBP2:KCNC4:HCN3:RUSC1:SYT11:ARHGEF2:BGLAP:UHMK1:CRB1:PSEN2:CDH23:ZFYVE27:APBB1:KCNJ11:ABCC8:ASRGL1:LRFN4:TENM4:HSPA8:ERC1:KCNA1:ENO2:PHB2:PDE1

						B:CIT:ULK1:P2RX2:PCDH9:KCTD12:ABHD13:LRFN5:ERO1L:PSEN1:MYO5A:SKOR1:CHRNB4:SH3GL3:GRIN2A:CORO1A:SEPT1:SEPT1:CALB2:SPG7:TRPV1:RCVRN:RNF112:TBX21:RPS6KB1:KCNJ2:RPL38:FSCN2:PHLPP1:MBP:SH3GL1:TUBB4A:INSR:GIPC1:DNAJB1:OR10H2:OR10H3:GSK3A:GRIN2D:KCNJ14:NCOA1:BCL11A:ATP6V1B1:DCTN1:MERTK:FAM168B:PKP4:SCN3A:CREB1:MFF:TMEM230:DNAJC5:OSBP2:SYNGR1:MAPK8IP2:SHANK3:SCN5A:MYRIP:RPL14:TRAK1:GNAI2:CCDC66:STX19:IFT57:GSK3B:RAB6B:XRN1:KCNAB1:NPY5R:CDH9:DROSHA:MCTP1:FCHSD1:NDFIP1:GRXCR2:KCNIP1:CPEB4:SNCB:FLOT1:C4A:RPS18:CPNE5:CNR1:MAP7:CRHR2:HSPB1:YWHAG:PRKAR2B:EXOC4:PPP3CC:STAR:LYN:RPL7:CA2:CALB1:SCRIB:RPL12:CEL
GO_cc	GO_ENDOPLASMIC_RETICULUM	1884	133	2.0461196289007765e-12	6.827219161765591e-10	ICMT:CLSTN1:MTOR:UBIAD1:CDC42:TMEM50A:TMEM57:MFSD2A:ELOVL1:TXNDC12:RPE65:LRRC8B:LRRC8C:EXTL2:CERS2:SSR2:UBQLN4:BGLAP:FCRLB:F5:SEC16B:TOR3A:SOAT1:PSEN2:B3GALNT2:RAB18:REEP3:LRIT1:ZFYVE27:KCNJ11:PEX16:TM7SF2:SYVN1:RCE1:MOGAT2:DGAT2:LINC01059:GRAMD1B:KCNA1:LPCAT3:METTL7A:IKBIP:ULK1:ANKLE2:EBPL:KDELC1:FITM1:SCFD1:MIA2:CTAGE5:TXNDC16:ERO1L:ZFYVE1:PSEN1:MYO5A:ANP32A:ADAMTS7:KIAA1199:MESDC2:PKD1:GRIN2A:NOMO1:PDXDC1:TRAPPC2L:DERL2:ASGR2:GJC1:KPNB1:RNFT1:KCNJ2:NPLOC4:MPPE1:CD320:KEAP1:TECR:KDELR1:IGFBP5:TM4SF20:TMEM230:CDS2:RPN2:TGM2:USP25:SLC37A1:LSS:OSBP2:RTCB:APOL2:APOL1:CYP2D6:CYB5R3:LMF2:ARSA:SCN5A:CYP8B1:HYAL3:HYAL2:CYB561D2:XXcos-LUCA11.4:PROS1:RAB43:CNBP:SRPRB:COPB2:LSG1:MFI2:STIM2:ATP10D:AREG:AREGB:MTTP:UTP15:MCTP1:CPEB4:EDN1:HLA-A:C4A:B3GALT4:TAPBP:PTP4A1:RNASET2:ERMARD:BET1:BCAP29:MEST:COPG2:CREB3L2:GIMAP5:ESYT2:RB1CC1:RDH10:COL22A1:KIAA0020:ADAMTSL1:UBQLN1:PAPPA-AS1:DPM2
GO_cc	GO_ENVELOPE	1166	92	2.1771115801925254e-11	5.448221729431795e-09	MTOR:UBIAD1:TMEM57:RBM15:CERS2:SMCP:MSTO1:DAP3:LMNA:PSEN2:ADCK3:ALDH18A1:GPAM:GRK5:IPO7:KCNJ11:ARL2:TM7SF2:MRPL49:UCP2:UCP3:REXO2:NDUFA9:PHB2:SLC11A2:LETMD1:SLC25A3:DAO:TMEM120B:BRI3BP:ULK1:P2RX2:MTMR6:SLC25A15:KPNA3:LMO7:IPO5:PSEN1:MOAP1:PLA2G4B:NPIPA1:CIAPIN1:COQ9:CYB5B:SPG7:VAT1:KPNB1:UTP18:RPS6KB1:ENTHD2:PHLPP1:INSR:PRODH2:MRPS12:PUM2:XPO1:DCTN1:MFF:PANK2:CDS2:NDUFAF5:CHMP4B:RTCB:SMDT1:NDUFA6:CYB5R3:ATP5L2:SCO2:CPT1B:TRAK1:ACKR2:SLC25A26:DHFRL1:NDUFB4:ACAD11:MRPS22:SENP2:COQ2:IPO11:PRELID2:NRM:TUBB:CNR1:SYNE1:GIMAP5:STAR:RB1CC1:LYN:ENY2:CYP11B1:CYP11B2:SLC25A25
GO_cc	GO_ENDOPLASMIC_RETICULUM_PART	1344	101	3.61576924706283e-11	7.238770032619785e-09	ICMT:CLSTN1:MTOR:UBIAD1:CDC42:TMEM57:MFSD2A:ELOVL1:TXNDC12:RPE65:LRRC8B:LRRC8C:EXTL2:CERS2:SSR2:UBQLN4:BGLAP:F5:SEC16B:TOR3A:SOAT1:PSEN2:B3GALNT2:RAB18:REEP3:LRIT1:ZFYVE27:PEX16:TM7SF2:SYVN1:RCE1:MOGAT2:DGAT2:LINC01059:GRAMD1B:LPCAT3:IKBIP:ULK1:ANKLE2:EBPL:KDELC1:FITM1:SCFD1:MIA2:CTAGE5:TXNDC16:ERO1L:PSEN1:ADAMTS7:NOMO1:DERL2:ASGR2:GJC1:KPNB1:RNFT1:NPLOC4:MPPE1:TECR:KDELR1:IGFBP5:TM4SF20:CDS2:RPN2:SLC37A1:LSS:OSBP2:RTCB:APOL2:APOL1:CYP2D6:CYB5R3:LMF2:ARSA:CYP8B1:CYB561D2:XXcos-LUCA11.4:PROS1:RAB43:SRPRB:COPB2:MFI2:STIM2:ATP10D:AREG:AREGB:MTTP:MCTP1:EDN1:HLA-A:C4A:TAPBP:RNASET2:ERMARD:BET1:BCAP29:MEST:COPG2:CREB3L2:ESYT2:RB1CC1:RDH10:COL22A1:ADAMTSL1:PAPPA-AS1:DPM2
GO_cc	GO_WHOLE_MEMBRANE	1647	116	6.815028063103599e-11	1.1369738485277837e-08	CLSTN1:MTOR:LDLRAP1:SZT2:TSPAN1:BCL10:ABCD3:LAMTOR5:NRAS:SCAMP3:MSTO1:SYT11:TMEM79:C1orf85:FCGR2A:SELP:SEC16B:RAB18:PSAP:FAS:ZFYVE27:GPAM:DMBT1:ABCC8:PEX16:HSPA8:PHB2:SLC11A2:LETMD1:NCKAP1L:RAP1B:DAO:ORAI1:BRI3BP:ULK1:PSEN1:MOAP1:PLA2G4B:CHRNB4:KIAA1199:SH3GL3:PKD1:CORO1A:MYLPF:ABCC11:CYB5B:RABEP1:EPN2:VAT1:RND2:PLEKHM1:RPS6KB1:ENTHD2:NAPG:SH3GL1:UBXN6:INSR:PEX11G:CD320:AP1M2:CD97:SYCN:CD79A:LIPE:CEACAM1:CBLC:KDELR1:CD33:FPR2:PEX13:XPO1:ATP6V1B1:PLEKHB2:SLC11A1:MFF:CHMP4B:RPN2:DNAJC5:SYNGR1:TAB1:CYB5R3:ARFGAP3:CPT1B:TGFBR2:SCN5A:GNAI2:HYAL3:HYAL2:NPRL2:RAB43:COPB2:AP2M1:MAN2B2:AREG:AREGB:MCTP1:MARCH3:DIAPH1:NDFIP1:TTC1:HLA-A:TUBB:FLOT1:VPS52:TAPBP:RAB44:CNR1:PEX7:SYNE1:LAT2:CD36:PRKAR2B:COPG2:GIMAP5:RB1CC1:LYN:CHMP5
GO_cc	GO_VACUOLE	760	65	8.642776401965537e-10	1.2359170254810718e-07	MTOR:SZT2:TSPAN1:DNASE2B:BCL10:LAMTOR5:CTSS:CTSK:HRNR:SYT11:UBQLN4:TMEM79:C1orf85:FMOD:PSAP:SMPD1:HSPA8:USP5:SLC11A2:RAP1B:ULK1:ZFYVE1:PSEN1:MYO5A:ACAN:MYLPF:ABCC11:PLA2G15:VAT1:NBR1:PLEKHM1:NAPG:UBXN6:AP1M2:ATP6V1B1:SLC11A1:TMEM230:RPN2:DNAJC5:SYNGR1:NAGA:CYB5R3:ARSA:HYAL3:HYAL1:HYAL2:NPRL2:AP2M1:MAN2B2:PSAPL1:PLAC8:MARCH3:PDGFRB:PRSS16:TUBB:FLOT1:RAB44:TNFAIP3:RNASET2:LAT2:GIMAP5:RB1CC1:LYN:UBQLN1:TUBB4B

GO_cc	GO_NUCLEAR_OUTER_MEMBRANE_ENDOPLASMIC_RETICULUM_MEMBRANE_NETWORK	1080	82	1.6816428503261398e-09	2.1041556164705825e-07	ICMT:CLSTN1:MTOR:UBIAD1:CDC42:TMEM57:MFSD2A:ELOVL1:RPE65:LRRC8B:LRRC8C:EXTL2:CERS2:SSR2:UBQLN4:SEC16B:SOAT1:PSEN2:B3GALNT2:RAB18:REEP3:LRIT1:ZFYVE27:PEX16:TM7SF2:SYVN1:RCE1:MOGAT2:DGAT2:LINC01059:GRAMD1B:LPCAT3:IKBIP:ULK1:ANKLE2:EBPL:FITM1:SCFD1:MIA2:CTAGE5:ERO1L:PSEN1:NOMO1:DERL2:GJC1:RNFT1:NPLOC4:TECR:KDELR1:XPO1:TM4SF20:CDS2:RPN2:SLC37A1:LSS:RTCB:APOL2:CYP2D6:CYB5R3:LMF2:CYP8B1:CYB561D2:XXcos-LUCA11.4:PROS1:RAB43:SRPRB:COPB2:STIM2:ATP10D:AREG:AREGB:MCTP1:HLA-A:TAPBP:SYNE1:ERMARD:BET1:BCAP29:MEST:COPG2:CREB3L2:ESYT2:RB1CC1:RDH10:PAPPA-AS1:DPM2
GO_cc	GO_GOLGI_APPARATUS	1547	106	2.141235783940168e-09	2.3815300219156755e-07	CLSTN1:MTOR:UBIAD1:CDC42:A3GALT2:MACF1:MPL:OLFM3:WDR77:NRAS:CSDE1:GOLPH3L:SCAMP3:RUSC1:ASH1L:ARHGEF2:PMF1:BGLAP:TMEM79:UHMK1:F5:SEC16B:FMOD:PSEN2:B3GALNT2:ARL5B:RAB18:OPALIN:EHF:LINC00610:COMMD9:GYLTL1B:ARL2:ZFPL1:VPS51:ERC1:PTHLH:LGR5:FBXW8:CIT:POSTN:FNDC3A:SCFD1:ZFYVE1:PSEN1:GOLGA8G:GOLGA8J:GOLGA8R:GOLGA8Q:GOLGA8H:MYO5A:ACAN:PKD1:PDXDC1:SMPD3:TRAPPC2L:KCNJ2:ENTHD2:MPPE1:ST8SIA5:MUC16:AP1M2:CHST8:KDELR1:TMEM87B:RND3:TMEM230:MMP24:FAM109B:ARFGAP3:HYAL2:TMEM115:PROS1:IFT57:RAB43:ATP2C1:UBA5:RAB6B:A4GNT:COPB2:AREG:AREGB:MTTP:HDAC3:NDFIP1:PDGFRB:ADAM19:MGAT1:HLA-A:MUC21:VPS52:B3GALT4:TAPBP:C6orf89:FUT9:ESR1:SYNE1:MPLKIP:TPST1:CD36:BET1:FAM3C:COPG2:LYN:SLC35D2:RP11-203J24.9:ST6GALNAC6:ST6GALNAC4:UBAC1
GO_cc	GO_MITOCHONDRION	1552	105	5.016643216744843e-09	5.021659859961588e-07	ACOT7:MTOR:UBIAD1:ABCD3:HMGCS2:SMCP:MSTO1:DAP3:ADCK3:ECHDC3:ALDH18A1:GPAM:TRUB1:KCNJ11:ABCC8:BBOX1:ARL2:MRPL49:PC:UCP2:UCP3:DGAT2:FDX1:REXO2:NDUFA9:PHB2:SLC11A2:LETMD1:SLC25A3:DAO:SDS:BRI3BP:ULK1:MTIF3:SLC25A15:SIAH3:PCK2:DLGAP5:PSEN1:MOAP1:PLA2G4B:MTFMT:DCTPP1:CIAPIN1:COQ9:CYB5B:SPG7:TRPV1:VAT1:NBR1:RPS6KB1:C17orf89:NAPG:PRODH2:SARS2:MRPS12:GSK3A:TRMT61B:BOLA3:MTHFD2:CREB1:MFF:PANK2:CDS2:NDUFAF5:TGM2:DNAJC5:SMDT1:NDUFA6:CYP2D6:CYB5R3:ATP5L2:TRMU:SCO2:CPT1B:TRAK1:TUSC2:SLC25A26:DHFRL1:GTPBP8:GSK3B:NDUFB4:ACAD11:MRPS22:GK5:COQ2:TERT:DIMT1:PRELID2:THG1L:CNR1:SOD2:YWHAG:GIMAP5:PPP3CC:STAR:LYN:ENY2:POU5F1B:TMEM71:CYP11B1:CYP11B2:NAIF1:SLC25A25:NACC2
GO_cc	GO_CYTOSKELETAL_PART	1639	109	6.3581710022658426e-09	5.307340797220781e-07	FBLIM1:CDC42:LDLRAP1:CCDC28B:MACF1:CDC20:STIL:HOOK1:SSX2IP:BCL10:HIPK1:BCAS2:TMOD4:FLG:RUSC1:ARHGEF2:LMNA:HSPA6:ASPM:PSEN2:TUBB8:REEP3:KRTAP5-1:KRTAP5-2:KRTAP5-3:KRTAP5-4:KRTAP5-5:ARL2:SAC3D1:DNAJB13:TTC12:CLMP:LCP1:FAM179B:DLGAP5:PSEN1:HERC2:MAPKBP1:MYO5A:MESDC1:CORO1A:MYLPF:SEPT1:SEPT1:CALB2:DNAH9:B9D1:BRCA1:SPAG9:TUBD1:DNAI2:KIF19:AZI1:MYL12A:MYL12B:SH3GL1:UBXN6:TUBB4A:KEAP1:CC2D1A:TSKS:DCTN1:RTKN:MZT2A:PKP4:VIL1:TTLL4:PCNA:BFSP1:DSTN:KRTAP21-3:KRTAP8-1:SPATC1L:GTSE1:RAD18:MYRIP:ACKR2:GNAI2:RASSF1:ZMYND10:CCDC66:ARL13B:IFT57:GSK3B:SRPRB:DZIP1L:SLAIN2:KIF2A:CETN3:DIAPH1:HDAC3:FCHSD1:LRRC16A:TUBB:FLOT1:PTP4A1:MAP7:FGFR1OP:TCTE3:SEPT7:MPLKIP:HSPB1:PRKAR2B:CEP41:CALD1:TERF1:CALB1:GEM:CNTLN:TUBB4B
GO_cc	GO_SYNAPSE	1169	85	6.362446510154783e-09	5.307340797220781e-07	CLSTN1:MTOR:CDC42:TMEM57:OLFM3:KCNC4:HCN3:RUSC1:SYT11:ARHGEF2:PSEN2:RAB18:APBB1:ABCC8:LRFN4:HSPA8:ERC1:KCNA1:PHB2:P2RX2:POSTN:KCTD12:LRFN5:PSEN1:NUMB:MYO5A:CHRNB4:SH3GL3:ACAN:GRIN2A:CORO1A:SEPT1:SEPT1:CALB2:TRPV1:RNF112:DGKE:RPS6KB1:KCNJ2:RPL38:NAPG:SH3GL1:GIPC1:DNAJB1:GSK3A:GRIN2D:BCL11A:ATP6V1B1:PKP4:MFF:TMEM230:DNAJC5:SYNGR1:MAPK8IP2:SHANK3:MYRIP:RPL14:STX19:GSK3B:RAB6B:XRN1:EIF4E:CDH9:DROSHA:ACTBL2:MCTP1:FCHSD1:NDFIP1:CPEB4:SNCB:FLOT1:C4A:RPS18:CNR1:SYNE1:CRHR2:YWHAG:PRKAR2B:EXOC4:PPP3CC:LYN:RPL7:CALB1:SCRIB:RPL12:CEL
GO_cc	GO_SECRETORY_VESICLE	983	73	3.2871252826272724e-08	2.5310864676229994e-06	NRAS:CTSS:HRNR:ADAM15:SYT11:FCGR2A:HSPA6:F5:SELP:PSEN2:RAB18:PSAP:DMBT1:SMPD1:KCNJ11:ABCC8:LINC00610:COMMD9:SYT13:HSPA8:AKAP3:PTPN6:METTL7A:NCKAP1L:RAP1B:APAF1:FNDC3A:PSEN1:MYO5A:CHRNB4:SH3GL3:GRIN2A:SEPT1:SEPT1:RNF112:ZPBP2:VAT1:RND2:KPNB1:SPAG9:PSMD12:AZI1:CD97:GIPC1:SYCN:CEACAM1:CD33:FPR2:ATP6V1B1:SLC11A1:MFF:TMEM230:SLPI:DNAJC5:SYNGR1:CYB5R3:ARSA:MYRIP:HYAL3:PROS1:STX19:ARMC8:CXCL1:PLAC8:MCTP1:DIAPH1:EDN1:TUBB:RAB44:RNASET2:THBS2:CD36:FAM3C:TUBB4B:TOR4A
GO_cc	GO_CELL_JUNCTION	1275	88	3.7878519618698157e-08	2.7083141527369183e-06	RPL22:CLSTN1:FBLIM1:CDC42:TSPAN1:SSX2IP:OLFM3:GJA5:PRUNE:ADAM15:RUSC1:ASH1L:SYT11:ARHGEF2:CRB1:CSRP1:OPALIN:KCNJ11:ARL2:TRPC6:REXO2:HSPA8:CLMP:KCNA1:ATN1:PTPN6:RAP1B:LCP1:PCDH9:LMO7:KCTD12:STXBP6:FRMD6:PSEN1:NUMB:CHRNB4:GRIN2A:CORO1A:ARMC5:TGFB1I1:CALB2:TRPV1:RNF112:RPL19:GJC1:RPS6KB1:KCNJ2:RPL38:SH3GL1:ARHGEF18:CD97:NPHS1:CEACAM1:GRIN2D:PPP1CB:RND3:PKP4:MFF:TM4SF20:TMEM230:TGM2:SYNGR1:NCAPH2:SHANK3:S

						CN5A:CDH9:CDH6:OCLN:MCTP1:PCDHGA12:NDFIP1:PDGFRB:CPEB4:MDC1:FLOT1:C4A:RPS18:ITGB8:CLDN3:CLDN4:HSPB1:YWHAG:LYN:RPL7:CDH17:SCRIB:PUF60:RPL12
GO_cc	GO_GOLGI_APPARATUS_PART	964	71	6.998913588505151e-08	4.54956519167952e-06	CLSTN1:MTOR:UBIAD1:CDC42:A3GALT2:NRAS:GOLPH3L:SCAMP3:BGLAP:TMEM79:F5:SEC16B:FMOD:PSEN2:B3GALNT2:ARL5B:GYLTL1B:VPS51:ERC1:LGR5:CIT:POSTN:FNDC3A:SCFD1:ZFYVE1:PSEN1:GOLGA8G:GOLGA8J:GOLGA8R:GOLGA8H:ACAN:PKD1:SMPD3:TRAPPC2L:ENTHD2:MPPE1:ST8SIA5:MUC16:AP1M2:CHST8:KDELR1:TMEM87B:RND3:TMEM230:MMP24:FAM109B:ARFGAP3:HYAL2:TMEM115:PROS1:RAB43:ATP2C1:RAB6B:A4GNT:COPB2:AREG:AREGB:NDFIP1:MGAT1:HLA-A:MUC21:VPS52:B3GALT4:TAPBP:C6orf89:FUT9:TPST1:BET1:COPG2:SLC35D2:RP11-203J24.9:ST6GALNAC6:ST6GALNAC4
GO_cc	GO_MITOCHONDRIAL_ENVELOPE	725	58	7.27203227441282e-08	4.54956519167952e-06	MTOR:UBIAD1:SMCP:MSTO1:DAP3:ADCK3:ALDH18A1:GPAM:ARL2:MRPL49:UCP2:UCP3:REXO2:NDUFA9:PHB2:SLC11A2:LETMD1:SLC25A3:DAO:BRI3BP:ULK1:SLC25A15:PSEN1:MOAP1:PLA2G4B:CIAPIN1:COQ9:CYB5B:SPG7:VAT1:RPS6KB1:PRODH2:MRPS12:MFF:PANK2:CDS2:NDUFAF5:SMDT1:NDUFA6:CYB5R3:ATP5L2:SCO2:CPT1B:TRAK1:SLC25A26:DHFRL1:NDUFB4:ACAD11:MRPS22:COQ2:PRELID2:CNR1:GIMAP5:STAR:LYN:CYP11B1:CYP11B2:SLC25A25
GO_cc	GO_GOLGI_MEMBRANE	747	59	8.733946666729687e-08	5.142753301997891e-06	CLSTN1:MTOR:UBIAD1:CDC42:A3GALT2:NRAS:GOLPH3L:SCAMP3:TMEM79:F5:SEC16B:PSEN2:B3GALNT2:GYLTL1B:VPS51:ERC1:LGR5:FNDC3A:SCFD1:PSEN1:GOLGA8G:GOLGA8J:GOLGA8R:GOLGA8H:PKD1:SMPD3:TRAPPC2L:ENTHD2:ST8SIA5:AP1M2:CHST8:KDELR1:TMEM87B:RND3:MMP24:ARFGAP3:HYAL2:TMEM115:PROS1:RAB43:ATP2C1:RAB6B:A4GNT:COPB2:AREG:AREGB:NDFIP1:MGAT1:HLA-A:VPS52:B3GALT4:TAPBP:C6orf89:FUT9:TPST1:BET1:COPG2:SLC35D2:RP11-203J24.9:ST6GALNAC6:ST6GALNAC4
GO_cc	GO_CYTOPLASMIC_VESICLE_PART	1483	96	1.8461149288797799e-07	9.871689694481054e-06	LDLRAP1:LAMTOR5:NRAS:CTSS:CTSK:HRNR:SCAMP3:SYT11:FCGR2A:HSPA6:F5:SELP:SEC16B:RAB18:PSAP:ZFYVE27:DMBT1:HPX:ABCC8:LINC00610:COMMD9:PGA3:SNX15:VPS51:HSPA8:PTPN6:GPRC5A:METTL7A:SLC11A2:NCKAP1L:RAP1B:APAF1:PSEN1:PLA2G4B:CHRNB4:KIAA1199:SH3GL3:PKD1:CORO1A:ABCC11:RABEP1:EPN2:VAT1:RND2:PLEKHM1:KPNB1:PSMD12:ENTHD2:SH3GL1:UBXN6:INSR:CD320:AP1M2:CD97:SYCN:CEACAM1:KDELR1:CD33:FPR2:ATP6V1B1:PLEKHB2:SLC11A1:CHMP4B:SLPI:DNAJC5:SYNGR1:TAB1:CYB5R3:ARFGAP3:ARSA:HYAL3:CYB561D2:XXcos-LUCA11.4:PROS1:ZPLD1:RAB43:ARMC8:COPB2:AP2M1:CXCL1:AREG:AREGB:PLAC8:MCTP1:MARCH3:DIAPH1:NDFIP1:HLA-A:TUBB:VPS52:TAPBP:RAB44:RNASET2:CD36:FAM3C:COPG2:CHMP5:TUBB4B:TOR4A
GO_cc	GO_SYNAPSE_PART	932	68	1.8737472946567433e-07	9.871689694481054e-06	CLSTN1:MTOR:CDC42:KCNC4:HCN3:RUSC1:SYT11:ARHGEF2:PSEN2:APBB1:ABCC8:LRFN4:HSPA8:ERC1:KCNA1:PHB2:P2RX2:KCTD12:LRFN5:PSEN1:MYO5A:CHRNB4:SH3GL3:GRIN2A:SEPT1:SEPT1:CALB2:TRPV1:RNF112:KCNJ2:RPL38:SH3GL1:GIPC1:DNAJB1:GSK3A:GRIN2D:BCL11A:ATP6V1B1:PKP4:MFF:TMEM230:DNAJC5:SYNGR1:MAPK8IP2:SHANK3:RPL14:STX19:GSK3B:RAB6B:EIF4E:CDH9:DROSHA:MCTP1:CPEB4:SNCB:FLOT1:RPS18:CNR1:SYNE1:CRHR2:YWHAG:PRKAR2B:PPP3CC:LYN:RPL7:CALB1:SCRIB:RPL12:CEL
GO_cc	GO_PLASMA_MEMBRANE_REGION	1185	81	1.9844425762362695e-07	9.93213509406253e-06	CLSTN1:LDLRAP1:MACF1:KCNC4:RUSC1:SYT11:ARHGEF2:ASPM:CRB1:PSEN2:APBB1:KCNJ11:NAALADL1:LRFN4:ERC1:KCNA1:SLC11A2:CIT:ORAI1:P2RX2:LCP1:LMO7:KCTD12:LRFN5:PSEN1:NUMB:SLC51B:CHRNB4:PKD1:GRIN2A:SLC7A6:CALB2:TRPV1:RNF112:KCNJ16:SLC14A1:PHLPP1:INSR:ARHGEF18:PDE4A:PKN1:LIPE:CEACAM1:GRIN2D:PLB1:ATP6V1B1:SLC4A5:SLC9A4:IGFBP2:MCHR1:SHANK3:TGFBR2:SCN5A:HYAL2:ZMYND10:ARL13B:STX19:SLC34A2:SLC4A4:MTTP:SLC6A19:SLC6A18:CDH9:OCLN:SLC12A2:DIAPH1:PDGFRB:CPEB4:FLOT1:CNR1:MAP7:SYNE1:SEPT7:CLDN3:CLDN4:CD36:ABCB1:CA2:CDH17:SCRIB:SLC34A3
GO_cc	GO_ENDOSOME	885	65	2.7614962964466875e-07	1.3163132346395877e-05	CLSTN1:LDLRAP1:PTP4A2:C1orf210:LAMTOR5:CTSS:CTSK:SCAMP3:RUSC1:SYT11:PSEN2:PSAP:STAMBPL1:ZFYVE27:SMPD1:KCNJ11:PGA3:VPS51:SLC11A2:UHRF1BP1L:ULK1:PSEN1:NUMB:NIPA2:NIPA1:PLA2G4B:MYO5A:SH3GL3:CORO1A:SIAH1:RABEP1:DERL2:RNF112:RND2:NBR1:PLEKHM1:SH3GL1:UBXN6:INSR:CD320:PKN1:CD79A:PLEKHB2:SLC11A1:TMEM230:CHMP4B:TAB1:FAM109B:DENND6B:TRAK1:ACKR2:HYAL3:ACKR4:AP2M1:MCTP1:MARCH3:FCHSD1:NDFIP1:LRRC16A:PRSS16:HLA-A:FLOT1:VPS52:PTP4A1:CHMP5
GO_cc	GO_CELL_PROJECTION_PART	1438	93	3.0076735285510685e-07	1.368491455490736e-05	CLSTN1:MTOR:HTR6:CDC42:TMEM57:MACF1:SSX2IP:PTBP2:KCNC4:HCN3:SYT11:ARHGEF2:BGLAP:UHMK1:TACR2:ZFYVE27:APBB1:KCNJ11:DNAJB13:HSPA8:AKAP3:KCNA1:SLC11A2:LCP1:PCDH9:ABHD13:FAM179B:ERO1L:PSEN1:MYO5A:SKOR1:PKD1:CALB2:SPG7:TRPV1:RCVRN:DNAH9:B9D1:LYZL6:KCNJ2:DNAI2:KIF19:AZI1:PHLPP1:MBP:TUBB4A:INSR:PDE4A:GIPC1:DNAJB1:OR10H2:OR10H3:GSK3A:CEACAM1:KCNJ14:PLB1:DCTN1:MERTK:VIL1:OSBP2:MCHR1:SHANK3:MYRIP:TRAK1:GNAI2:HYAL

						3:CCDC66:ARL13B:IFT57:GSK3B:DZIP1L:XRN1:KCNAB1:SLC34A2:SLC6A19:SLC6A18:KIF2A:C5orf30:DIAPH1:NDFIP1:KCNIP1:CPEB4:C4A:CNR1:CRHR2:SEPT7:HSPB1:CD36:PRKAR2B:CEP41:EXOC4:CALB1:SLC34A3
GO_cc	GO_SOMATODENDRITIC_COMPARTMENT	818	61	3.8526984830871306e-07	1.676761383291399e-05	CLSTN1:MTOR:HTR6:CDC42:TMEM50A:MPL:PTBP2:KCNC4:HCN3:SYT11:ARHGEF2:BGLAP:UHMK1:PSEN2:ZFYVE27:APBB1:KCNJ11:HSPA8:KCNA1:ENO2:PDE1B:CIT:P2RX2:ABHD13:ERO1L:PSEN1:MYO5A:SKOR1:CALB2:TRPV1:RCVRN:TBX21:KCNJ2:MBP:TUBB4A:INSR:GIPC1:DNAJB1:OR10H2:OR10H3:GSK3A:KCNJ14:DCTN1:OSBP2:MAPK8IP2:SHANK3:TRAK1:GNAI2:IFT57:GSK3B:XRN1:KCNAB1:NDFIP1:KCNIP1:CPEB4:C4A:CPNE5:CRHR2:PRKAR2B:STAR:CALB1
GO_cc	GO_MICROTUBULE_CYTOSKELETON	1169	79	4.4162629605602475e-07	1.8419496764670033e-05	CDC42:CCDC28B:MACF1:CDC20:STIL:HOOK1:SSX2IP:BCL10:HIPK1:BCAS2:RUSC1:ARHGEF2:HSPA6:ASPM:PSEN2:TUBB8:REEP3:ARL2:SAC3D1:TTC12:CLMP:NUAK1:FAM179B:DLGAP5:PSEN1:HERC2:MAPKBP1:MYO5A:SEPT1:SEPT1:POLR2C:DNAH9:B9D1:BRCA1:SPAG9:TUBD1:DNAI2:KIF19:AZI1:UBXN6:TUBB4A:KEAP1:CC2D1A:TSKS:DCTN1:MZT2A:PKP4:TTLL4:PCNA:SPATC1L:GTSE1:RAD18:GNAI2:RASSF1:ZMYND10:CCDC66:IFT57:GSK3B:SRPRB:DZIP1L:SLAIN2:KIF2A:CETN3:DIAPH1:HDAC3:TUBB:FLOT1:PTP4A1:MAP7:FGFR1OP:TCTE3:SEPT7:MPLKIP:HSPB1:PRKAR2B:CEP41:TERF1:GEM:CNTLN:TUBB4B
GO_cc	GO_MEMBRANE_PROTEIN_COMPLEX	1153	78	5.018766389881994e-07	2.0095140625087506e-05	HOOK1:LRRC8B:LRRC8C:OLFM3:KCNC4:LAMTOR5:GJA5:FAS:KCNJ11:ABCC8:SYVN1:TRPC6:NDUFA9:KCNA1:PTPN6:PSEN1:CHRNB4:PKD1:GRIN2A:SPG7:DERL2:EPN2:CACNB1:GJC1:CACNG1:KCNJ16:KCNJ2:TTYH2:ENTHD2:NPLOC4:NAPG:INSR:AP1M2:EPS15L1:IL12RB1:CD79A:CEACAM1:GRIN2D:KCNJ14:ATP6V1B1:DCTN1:IL18R1:IL18RAP:SCN3A:CHMP4B:RPN2:KCNS1:LIME1:SMDT1:NDUFA6:ATP5L2:ARFGAP3:SHANK3:SCN5A:GNAI2:CACNA2D2:STX19:NDUFB4:COPB2:KCNAB1:AP2M1:CDH9:CDH6:KCNIP1:HLA-A:FLOT1:TAPBP:SYNE1:ITGB8:CLDN4:GNG11:BET1:COPG2:LYN:CDH17:SCRIB:KCNV2:DPM2
GO_cc	GO_CELL_BODY	557	46	6.689820926060328e-07	2.575581056533226e-05	MTOR:CDC42:TMEM50A:MPL:RPE65:PTBP2:KCNC4:HCN3:SYT11:ARHGEF2:BGLAP:PSEN2:ACTA2:APBB1:KCNJ11:KCNA1:ENO2:PDE1B:CIT:P2RX2:PSEN1:MYO5A:SKOR1:TRPV1:RNF112:ZPBP2:TBX21:KCNJ2:RNF157:MBP:TUBB4A:INSR:DNAJB1:GSK3A:KCNJ14:DCTN1:MAPK8IP2:GNAI2:XRN1:KCNAB1:TUBB:C4A:CPNE5:PRKAR2B:STAR:CALB1
GO_cc	GO_SECRETORY_GRANULE	831	60	1.3729848249487843e-06	5.090214110273085e-05	NRAS:CTSS:HRNR:ADAM15:FCGR2A:HSPA6:F5:SELP:RAB18:PSAP:DMBT1:SMPD1:KCNJ11:LINC00610:COMMD9:HSPA8:AKAP3:PTPN6:METTL7A:NCKAP1L:RAP1B:APAF1:FNDC3A:PSEN1:MYO5A:CHRNB4:SH3GL3:ZPBP2:VAT1:RND2:KPNB1:SPAG9:PSMD12:AZI1:CD97:SYCN:CEACAM1:CD33:FPR2:SLC11A1:SLPI:DNAJC5:SYNGR1:CYB5R3:ARSA:MYRIP:HYAL3:PROS1:ARMC8:CXCL1:PLAC8:DIAPH1:EDN1:TUBB:RAB44:RNASET2:THBS2:CD36:FAM3C:TUBB4B:TOR4A
GO_cc	GO_MITOCHONDRIAL_PART	1019	69	2.2119622538860633e-06	7.733484356491368e-05	MTOR:UBIAD1:HMGCS2:SMCP:MSTO1:DAP3:ADCK3:ALDH18A1:GPAM:ARL2:MRPL49:PC:UCP2:UCP3:FDX1:REXO2:NDUFA9:PHB2:SLC11A2:LETMD1:SLC25A3:DAO:BRI3BP:ULK1:SLC25A15:PCK2:PSEN1:MOAP1:PLA2G4B:CIAPIN1:COQ9:CYB5B:SPG7:VAT1:RPS6KB1:PRODH2:SARS2:MRPS12:TRMT61B:MTHFD2:CREB1:MFF:PANK2:CDS2:NDUFAF5:SMDT1:NDUFA6:CYB5R3:ATP5L2:SCO2:CPT1B:TRAK1:SLC25A26:DHFRL1:NDUFB4:ACAD11:MRPS22:COQ2:TERT:DIMT1:PRELID2:CNR1:SOD2:GIMAP5:STAR:LYN:CYP11B1:CYP11B2:SLC25A25
GO_cc	GO_NEURON_PROJECTION	1301	83	2.2404699933891077e-06	7.733484356491368e-05	CLSTN1:MTOR:HTR6:CDC42:TMEM57:LDLRAP1:MPL:LPHN2:PTBP2:KCNC4:HCN3:SYT11:ARHGEF2:BGLAP:UHMK1:CDH23:ZFYVE27:APBB1:KCNJ11:TENM4:HSPA8:KCNA1:PHB2:ULK1:PCDH9:ABHD13:ERO1L:PSEN1:MYO5A:SKOR1:CHRNB4:GRIN2A:CORO1A:CALB2:SPG7:TRPV1:RCVRN:RPS6KB1:KCNJ2:FSCN2:PHLPP1:MBP:TUBB4A:INSR:GIPC1:DNAJB1:OR10H2:OR10H3:GSK3A:KCNJ14:NCOA1:DCTN1:MERTK:FAM168B:SCN3A:CREB1:OSBP2:SHANK3:SCN5A:MYRIP:TRAK1:GNAI2:CCDC66:IFT57:GSK3B:XRN1:KCNAB1:NPY5R:NDFIP1:GRXCR2:KCNIP1:CPEB4:C4A:CPNE5:CNR1:MAP7:CRHR2:HSPB1:PRKAR2B:EXOC4:STAR:CA2:CALB1
GO_cc	GO_PLASMA_MEMBRANE_PROTEIN_COMPLEX	570	45	2.9338774824060437e-06	9.638024424657313e-05	OLFM3:KCNC4:GJA5:FAS:KCNJ11:ABCC8:KCNA1:PTPN6:PSEN1:CHRNB4:GRIN2A:CACNB1:GJC1:CACNG1:KCNJ16:KCNJ2:INSR:EPS15L1:IL12RB1:CD79A:CEACAM1:GRIN2D:KCNJ14:IL18R1:IL18RAP:SCN3A:KCNS1:LIME1:SHANK3:SCN5A:GNAI2:CACNA2D2:KCNAB1:AP2M1:CDH9:CDH6:KCNIP1:HLA-A:FLOT1:ITGB8:GNG11:LYN:CDH17:SCRIB:KCNV2
GO_cc	GO_INTRINSIC_COMPONENT_OF_ORGANELLE_MEMBRANE	378	34	2.984802768874892e-06	9.638024424657313e-05	CLSTN1:UBIAD1:ELOVL1:SYT11:LRIT1:ZFYVE27:PEX16:TM7SF2:SYVN1:RCE1:DGAT2:SLC25A3:P2RX2:ANKLE2:FITM1:SPG7:DERL2:PEX11G:TECR:PEX13:MFF:DNAJC5:SLC37A1:SYNGR1:SMDT1:SCO2:COQ2:HLA-A:TAPBP:CNR1:TPST1:BET1:ESYT2:DPM2
GO_cc	GO_EXTRINSIC_COMPONENT_OF_MEMBRANE	289	28	5.418720739905948e-06	0.00016950435814518293	ADCK3:DMBT1:ULK1:ZFYVE1:NUMB:RNF112:STAC2:ENTHD2:UBXN6:MUC16:OSR1:ATP6V1B1:NDUFAF5:SHANK3:GNAI2:KCNAB1:CDH9:CDH6:KCNIP1:GNG11:ESYT2:BLK:GFRA2:RB1CC1:LYN:CDH17:GML:SCRIB

GO_cc	GO_PERINUCLEAR_REGION_OF_CYTOPLASM	697	51	5.78992461214634e-06	0.00017562771323510566	RNF207:CDC20:TSPAN1:BCL10:HRNR:LCE1D:SYT11:UBQLN4:PSEN2:APBB1:MOGAT2:DGAT2:ATN1:SLC11A2:FBXW8:LCP1:TNFSF13B:ZFYVE1:ANP32A:TRAPPC2L:ASGR2:SPATA32:SPAG9:RPS6KB1:PDE4A:PUM2:FAM168B:PKP4:COPS8:PER2:KCNS1:OSBP2:WBP2NL:SCN5A:MYRIP:TRAK1:HYAL2:RASA2:MRFAP1:EIF4E:NDFIP1:CPEB4:WRNIP1:VPS52:CDKN1A:MAP7:FGFR1OP:PRKAR2B:CREB3L2:LYN:UBQLN1
GO_cc	GO_RECYCLING_ENDOSOME	171	20	7.505931478469246e-06	0.0002209834532337563	LDLRAP1:C1orf210:SCAMP3:SYT11:ZFYVE27:VPS51:SLC11A2:ULK1:MYO5A:RABEP1:PLEKHB2:TMEM230:FAM109B:DENND6B:ACKR2:ACKR4:MCTP1:FCHSD1:HLA-A:VPS52
GO_cc	GO_EARLY_ENDOSOME	345	31	8.181515300668995e-06	0.00023399133759913326	LDLRAP1:PTP4A2:C1orf210:RUSC1:PSEN2:SLC11A2:UHRF1BP1L:PSEN1:NUMB:NIPA2:NIPA1:PLA2G4B:MYO5A:SH3GL3:CORO1A:SIAH1:RABEP1:DERL2:RND2:SH3GL1:UBXN6:TMEM230:FAM109B:TRAK1:ACKR2:HYAL3:ACKR4:MARCH3:HLA-A:FLOT1:PTP4A1
GO_cc	GO_TRANSPORTER_COMPLEX	334	30	1.1453916560731897e-05	0.0003184825132581286	LRRC8B:LRRC8C:OLFM3:KCNC4:KCNJ11:ABCC8:TRPC6:KCNA1:CHRNB4:PKD1:GRIN2A:CACNB1:CACNG1:KCNJ16:KCNJ2:TTYH2:GRIN2D:KCNJ14:PEX13:SCN3A:KCNS1:SMDT1:SHANK3:SCN5A:CACNA2D2:KCNAB1:KCNIP1:CLDN4:SCRIB:KCNV2
GO_cc	GO_VACUOLAR_PART	553	42	1.5745930524764538e-05	0.00042599125554835957	MTOR:SZT2:TSPAN1:LAMTOR5:CTSS:CTSK:HRNR:TMEM79:C1orf85:FMOD:PSAP:SMPD1:HSPA8:SLC11A2:RAP1B:ULK1:PSEN1:ACAN:MYLPF:ABCC11:VAT1:PLEKHM1:NAPG:UBXN6:AP1M2:ATP6V1B1:RPN2:DNAJC5:SYNGR1:CYB5R3:ARSA:HYAL1:NPRL2:AP2M1:MAN2B2:PLAC8:PDGFRB:TUBB:FLOT1:RAB44:RNASET2:TUBB4B
GO_cc	GO_POSTSYNAPSE	610	45	1.6450628600582695e-05	0.00043334419024166525	CLSTN1:MTOR:CDC42:RUSC1:SYT11:ARHGEF2:APBB1:LRFN4:HSPA8:KCNA1:PHB2:P2RX2:KCTD12:LRFN5:MYO5A:CHRNB4:SH3GL3:GRIN2A:TRPV1:RNF112:KCNJ2:RPL38:SH3GL1:GIPC1:DNAJB1:GSK3A:GRIN2D:BCL11A:PKP4:MAPK8IP2:SHANK3:RPL14:GSK3B:EIF4E:CDH9:DROSHA:CPEB4:RPS18:SYNE1:PRKAR2B:LYN:RPL7:CALB1:SCRIB:RPL12
GO_cc	GO_APICAL_PLASMA_MEMBRANE	313	28	2.3803046135248273e-05	0.0006109448508047056	ASPM:CRB1:PSEN2:NAALADL1:KCNA1:SLC11A2:P2RX2:LMO7:ARHGEF18:CEACAM1:PLB1:ATP6V1B1:SLC4A5:SLC9A4:IGFBP2:HYAL2:ZMYND10:SLC34A2:SLC6A19:SLC6A18:OCLN:SLC12A2:PDGFRB:SEPT7:CLDN4:CD36:ABCB1:SLC34A3
GO_cc	GO_MICROTUBULE_ORGANIZING_CENTER	722	50	3.0435734946727465e-05	0.0007616542670418548	CDC42:CCDC28B:CDC20:STIL:HOOK1:SSX2IP:HIPK1:BCAS2:HSPA6:ASPM:PSEN2:ARL2:SAC3D1:TTC12:FAM179B:DLGAP5:PSEN1:HERC2:SEPT1:SEPT1:B9D1:BRCA1:SPAG9:TUBD1:AZI1:UBXN6:KEAP1:CC2D1A:TSKS:DCTN1:MZT2A:PCNA:SPATC1L:RAD18:GNAI2:RASSF1:ZMYND10:CCDC66:IFT57:GSK3B:DZIP1L:SLAIN2:KIF2A:CETN3:DIAPH1:FLOT1:FGFR1OP:MPLKIP:PRKAR2B:CEP41:CNTLN
GO_cc	GO_CATION_CHANNEL_COMPLEX	220	22	3.2732153470164295e-05	0.0007844863081567816	OLFM3:KCNC4:KCNJ11:ABCC8:TRPC6:KCNA1:PKD1:GRIN2A:CACNB1:CACNG1:KCNJ16:KCNJ2:GRIN2D:KCNJ14:SCN3A:KCNS1:SMDT1:SCN5A:CACNA2D2:KCNAB1:KCNIP1:KCNV2
GO_cc	GO_ANCHORING_JUNCTION	552	41	3.2915509433151675e-05	0.0007844863081567816	RPL22:FBLIM1:CDC42:SSX2IP:PRUNE:ADAM15:ARHGEF2:CRB1:CSRP1:ARL2:REXO2:HSPA8:LCP1:LMO7:STXBP6:NUMB:ARMC5:TGFB1I1:RPL19:RPL38:CD97:CEACAM1:PPP1CB:RND3:PKP4:TM4SF20:TGM2:CDH9:CDH6:PDGFRB:MDC1:FLOT1:RPS18:ITGB8:HSPB1:YWHAG:LYN:RPL7:CDH17:SCRIB:RPL12
GO_cc	GO_ORGANELLE_INNER_MEMBRANE	516	39	3.469790884822948e-05	0.0008077350408622723	DAP3:PSEN2:ADCK3:ALDH18A1:TM7SF2:MRPL49:UCP2:UCP3:NDUFA9:PHB2:LETMD1:SLC25A3:TMEM120B:P2RX2:SLC25A15:PSEN1:PLA2G4B:COQ9:SPG7:PRODH2:MRPS12:CDS2:NDUFAF5:SMDT1:NDUFA6:ATP5L2:SCO2:SLC25A26:DHFRL1:NDUFB4:ACAD11:MRPS22:COQ2:NRM:STAR:LYN:CYP11B1:CYP11B2:SLC25A25
GO_cc	GO_NUCLEAR_ENVELOPE	462	36	3.648675399260715e-05	0.0008300736533318126	TMEM57:RBM15:CERS2:LMNA:PSEN2:GRK5:IPO7:KCNJ11:TM7SF2:TMEM120B:P2RX2:MTMR6:KPNA3:LMO7:IPO5:PSEN1:NPIPA1:KPNB1:UTP18:ENTHD2:PHLPP1:INSR:PUM2:XPO1:DCTN1:CHMP4B:RTCB:ACKR2:NDUFB4:SENP2:IPO11:NRM:TUBB:SYNE1:RB1CC1:ENY2
GO_cc	GO_NUCLEOPLASM_PART	1113	69	3.932097537810429e-05	0.0008670570061169214	TARDBP:MTOR:MED8:TAL1:RBM15:HIPK1:BCAS2:ARNT:FAM63A:SCNM1:CLK2:GON4L:LMNA:SFMBT2:FAS:GRK5:APBB1:CTR9:C12orf57:WBP4:FANCM:RBM25:GTF2A1:CPSF2:MGA:TCF12:CD2BP2:N4BP1:POLR2C:MAPK7:RNF112:NBR1:TRIM25:ENTHD2:C17orf70:TAF4B:TCEB3C:TCEB3B:TCF3:SAFB2:DDX39A:XPO1:ZNF638:DUSP11:AFF3:PCNA:SLC2A4RG:TAB1:RAD18:HDAC11:NR1I2:SENP2:LSG1:TERT:MCIDAS:KIF2A:HDAC3:LSM11:LRRC16A:MDC1:STK19:CDKN1A:GTF2H5:NCAPG2:ASH2L:TERF1:ENY2:NACC2:NELFB
GO_cc	GO_NUCLEAR_BODY	769	52	3.984477750387451e-05	0.0008670570061169214	TARDBP:MTOR:RBM15:HIPK1:BCAS2:ARNT:FAM63A:SCNM1:CLK2:GON4L:LMNA:SFMBT2:FAS:GRK5:APBB1:CTR9:C12orf57:WBP4:RBM25:TCF12:CD2BP2:N4BP1:MAPK7:RNF112:NBR1:TRIM25:ENTHD2:TCF3:SAFB2:DDX39A:XPO1:ZNF638:DUSP11:AFF3:PCNA:SLC2A4RG:TAB1:RAD18:NR1I2:SENP2:LSG1:TERT:MCIDAS:KIF2A:LSM11:LRRC16A:MDC1:STK19:CDKN1A:NCAPG2:TERF1:NACC2

GO_cc	GO_ORGANELLE_SUBCOMPARTMENT	375	31	4.13927943968226e-05	0.0008815784508770091	A3GALT2:GOLPH3L:SCAMP3:TMEM79:ARL5B:RAB18:ZFYVE27:VPS51:LGR5:CIT:POSTN:SCFD1:ZFYVE1:GOLGA8G:GOLGA8J:GOLGA8R:GOLGA8H:SMPD3:KPNB1:ENTHD2:AP1M2:TMEM230:MMP24:FAM109B:ARFGAP3:TMEM115:RAB43:ATP2C1:VPS52:FUT9:BET1
GO_cc	GO_PRESYNAPSE	484	37	4.292832685481602e-05	0.0008952344829514758	KCNC4:HCN3:SYT11:PSEN2:APBB1:ABCC8:HSPA8:ERC1:KCNA1:PHB2:KCTD12:PSEN1:SH3GL3:GRIN2A:SEPT1:SEPT1:CALB2:RNF112:SH3GL1:GIPC1:ATP6V1B1:MFF:TMEM230:DNAJC5:SYNGR1:STX19:RAB6B:CDH9:MCTP1:SNCB:FLOT1:CNR1:CRHR2:YWHAG:PPP3CC:CALB1:SCRIB:CEL
GO_cc	GO_CYTOPLASMIC_REGION	488	37	5.099891972251624e-05	0.001041835074331403	CDC42:UHMK1:PSEN2:DNAJB13:ABHD13:STXBP6:PSEN1:CORO1A:SEPT1:SEPT1:CALB2:SPG7:DNAH9:RND2:DNAI2:KIF19:MYL12B:TUBB4A:GIPC1:DCTN1:RTKN:RND3:NCL:BFSP1:DSTN:MYRIP:TRAK1:ARL13B:IFT57:KCNAB1:FCHSD1:NDFIP1:FLOT1:SEPT7:HSPB1:EXOC4:CALD1:CALB1
GO_cc	GO_MICROTUBULE_ORGANIZING_CENTER_PART	180	19	5.302521758238044e-05	0.0010615648559992564	STIL:SSX2IP:HSPA6:HERC2:BRCA1:TUBD1:AZI1:TSKS:DCTN1:MZT2A:ZMYND10:CCDC66:DZIP1L:KIF2A:CETN3:FLOT1:FGFR1OP:CEP41:CNTLN
GO_cc	GO_VESICLE_MEMBRANE	780	52	5.7429774573645656e-05	0.001127200085259202	LDLRAP1:NRAS:SYT11:FCGR2A:SELP:SEC16B:RAB18:PSAP:DMBT1:ABCC8:SNX15:HSPA8:GPRC5A:NCKAP1L:RAP1B:FNDC3A:PSEN1:CHRNB4:KIAA1199:CORO1A:ABCC11:EPN2:RND2:ENTHD2:AP1M2:CD97:GIPC1:SYCN:CEACAM1:KDELR1:CD33:FPR2:ATP6V1B1:SLC11A1:DNAJC5:SYNGR1:ARFGAP3:HYAL3:CYB561D2:XXcos-LUCA11.4:ZPLD1:RAB43:COPB2:AP2M1:AREG:AREGB:MCTP1:MARCH3:DIAPH1:HLA-A:TAPBP:RAB44:CD36:COPG2
GO_cc	GO_CYTOSOLIC_PART	246	23	6.279545607926699e-05	0.0012088125295258894	RPL22:MTOR:UBQLN4:HSPA8:ERC1:ENO2:APAF1:RPL3L:DHX33:RPL19:RPL38:CARD8:CTU1:CYB5R3:RPL32:RPL14:EIF4E:RPL37:RPS18:PPP3CC:RPL7:CALB1:RPL12
GO_cc	GO_VACUOLAR_LUMEN	170	18	7.929288776863306e-05	0.001470248941171335	CTSS:CTSK:HRNR:FMOD:PSAP:SMPD1:HSPA8:ACAN:VAT1:CYB5R3:ARSA:HYAL1:MAN2B2:PLAC8:PDGFRB:TUBB:RNASET2:TUBB4B
GO_cc	GO_CATALYTIC_COMPLEX	1351	79	7.931412869455753e-05	0.001470248941171335	EXOSC10:PHC2:CDC20:TAL1:F3:RBM15:WDR77:BCAS2:CKS1B:YY1AP1:UBQLN4:SNRPE:CUL2:CTR9:UBE2L6:SYVN1:HSPA8:ERC1:NDUFA9:SPSB2:ENO2:FBXW8:PRKAB1:ULK1:POLE:LMO7:DCAF11:PSME1:FBXO33:DCAF4:GTF2A1:AQR:MGA:POLR2C:SPG7:DNAH9:PRPSAP2:BRCA1:PSMD12:DNAI2:PPP4R1:TAF4B:INSR:KEAP1:DCAF15:FBXO17:PPP1CB:TRMT61B:DCTN1:ANAPC1:RPN2:ZNFX1:NDUFA6:RAD18:HDAC11:GNAI2:NDUFB4:ISY1:ARMC8:KLHL2:TERT:DROSHA:CCNO:SKIV2L2:CKS1B:HDAC3:TSPAN17:RPP40:CDKN1A:PPIL1:GTF2H5:TCTE3:HSPB1:GNG11:PPP3CC:ASH2L:RB1CC1:ENY2:UBQLN1:DPM2
GO_cc	GO_OUTER_MEMBRANE	205	20	0.00010316294053496241	0.001877565517736316	MTOR:MSTO1:GPAM:PHB2:SLC11A2:LETMD1:DAO:BRI3BP:ULK1:PSEN1:MOAP1:CYB5B:VAT1:RPS6KB1:MFF:CYB5R3:CPT1B:CNR1:SYNE1:GIMAP5
GO_cc	GO_APICAL_PART_OF_CELL	379	30	0.00011907206703609585	0.002128413198270213	ASPM:CRB1:PSEN2:NAALADL1:KCNA1:SLC11A2:P2RX2:LMO7:NUMB:ARHGEF18:CEACAM1:PLB1:ATP6V1B1:SLC4A5:SLC9A4:IGFBP2:HYAL2:ZMYND10:SLC34A2:SLC6A19:SLC6A18:OCLN:SLC12A2:PDGFRB:SEPT7:CLDN4:CD36:ABCB1:CA2:SLC34A3
GO_cc	GO_DENDRITIC_TREE	588	41	0.0001317125989009415	0.002313058096488464	CLSTN1:MTOR:HTR6:CDC42:HCN3:SYT11:ARHGEF2:BGLAP:UHMK1:ZFYVE27:APBB1:HSPA8:KCNA1:ABHD13:ERO1L:PSEN1:SKOR1:CALB2:RCVRN:KCNJ2:GIPC1:DNAJB1:OR10H2:OR10H3:GSK3A:KCNJ14:OSBP2:SHANK3:TRAK1:GNAI2:IFT57:GSK3B:XRN1:KCNAB1:NDFIP1:KCNIP1:CPEB4:C4A:CRHR2:PRKAR2B:CALB1
GO_cc	GO_CORNIFIED_ENVELOPE	65	10	0.00014942887717590096	0.0025789363112599458	HRNR:FLG:LCE1F:LCE1E:LCE1D:LCE1C:LCE1B:CNFN:PKP4:PI3
GO_cc	GO_NUCLEOLUS	1342	77	0.00017529002882371903	0.002973988455127843	EXOSC10:FBLIM1:EBNA1BP2:RPF1:BCAS2:YY1AP1:NUCKS1:LARP4B:MCM10:CUL2:WEE1:ARL2:SNX15:VPS51:REXO2:HSPA8:PTPN6:EMG1:DDX47:GPRC5A:NUAK1:DHX37:IPO5:G2E3:RRN3:CD2BP2:ZNF771:N4BP1:CIAPIN1:PRMT7:DHX33:PLEKHM1:UTP18:TAF4B:SLC14A1:CC2D1A:DNAJB1:DEDD2:GRWD1:NOL10:MYCN:PPP1CB:XPO1:DUSP11:MOB1A:NCL:PER2:ESF1:EIF6:DDX27:THUMPD3:C3orf17:NEK11:TRMT10A:TERT:DROSHA:SKIV2L2:KIF2A:DIMT1:IPO11:UTP15:CETN3:RPP40:WDR46:CDKN1A:C6orf89:L3MBTL3:SYNE1:GTF2H5:ARL4A:JAZF1:TERF1:RPL7:KIAA0020:IPPK:RPL12:NACC2
GO_cc	GO_CENTROSOME	500	36	0.000178384926284149	0.0029760551868405526	CDC42:CCDC28B:CDC20:STIL:HOOK1:SSX2IP:HIPK1:BCAS2:ASPM:PSEN2:ARL2:SAC3D1:TTC12:DLGAP5:PSEN1:B9D1:AZI1:DCTN1:MZT2A:PCNA:SPATC1L:RAD18:GNAI2:ZMYND10:CCDC66:IFT57:GSK3B:SLAIN2:KIF2A:CETN3:FLOT1:FGFR1OP:MPLKIP:PRKAR2B:CEP41:CNTLN
GO_cc	GO_TRANSCRIPTION_FACTOR_COMPLEX	354	28	0.0001998816520060322	0.0032800251419350533	MED8:TAL1:FOXE3:ARNT:PMF1:CREM:YAP1:POU2AF1:SIX6:GTF2A1:TCF12:SKOR1:NFATC3:SNAI3:HNF1B:TAF4B:TCF3:CREB1:HES6:PPARA:HYAL2:NR1I2:GTF2H5:HOXA10:HOXA11:KLF4:PBX3:GTF3C5

GO_cc	GO_LYSOSOMAL_LUMEN	94	12	0.00021215252341340955	0.003422144167518657	CTSS:CTSK:FMOD:PSAP:SMPD1:HSPA8:ACAN:ARSA:HYAL1:MAN2B2:PDGFRB:RNASET2
GO_cc	GO_CELL_CORTEX	302	25	0.0002153797028508246	0.003422144167518657	CDC42:PSEN2:STXBP6:PSEN1:CORO1A:SEPT1:SEPT1:CALB2:RND2:MYL12B:GIPC1:DCTN1:RTKN:RND3:NCL:BFSP1:DSTN:MYRIP:TRAK1:FCHSD1:NDFIP1:FLOT1:SEPT7:EXOC4:CALD1:CALB1
GO_cc	GO_INTRINSIC_COMPONENT_OF_ENDOPLASMIC_RETICULUM_MEMBRANE	155	16	0.00025923412691891635	0.004054583766341176	ELOVL1:LRIT1:ZFYVE27:TM7SF2:SYVN1:RCE1:DGAT2:ANKLE2:FITM1:DERL2:TECR:SLC37A1:HLA-A:TAPBP:ESYT2:DPM2
GO_cc	GO_NEURON_TO_NEURON_SYNAPSE	342	27	0.00026519057139825694	0.0040839347995331576	CLSTN1:RUSC1:SYT11:ARHGEF2:LRFN4:PHB2:LRFN5:SH3GL3:GRIN2A:RNF112:RPL38:SH3GL1:DNAJB1:GRIN2D:BCL11A:PKP4:MAPK8IP2:SHANK3:RPL14:DROSHA:CPEB4:RPS18:LYN:RPL7:CALB1:SCRIB:RPL12
GO_cc	GO_RECEPTOR_COMPLEX	398	30	0.00027724105529617043	0.004204822671991918	TIE1:OLFM3:TM7SF2:PTPN6:GPRC5A:P2RX2:CHRNB4:GRIN2A:INSR:IL12RB1:CD79A:CEACAM1:GRIN2D:ALK:FSHR:IL18R1:IL18RAP:MERTK:LIME1:PLXNB2:SHANK3:TGFBR2:CD200R1:MTTP:PDGFRB:ITGB8:CD36:GFRA2:LYN:SCRIB
GO_cc	GO_SPERM_PART	189	18	0.00030157862924672557	0.004505674744417498	ADAM15:TACR2:KCNJ11:DNAJB13:AKAP3:FNDC3A:SH3GL3:LYZL6:ZPBP2:RND2:SPAG9:DNAI2:AZI1:DNAJB1:WBP2NL:HYAL3:KIF2A:SEPT7
GO_cc	GO_SIDE_OF_MEMBRANE	554	38	0.00031422099896488497	0.0045242623305318825	LDLRAP1:F3:FCGR3A:SELP:DMBT1:HSPA8:TRPV1:STAC2:DNAI2:INSR:IL12RB1:CD79A:BCAM:CD33:IL1RL1:PKP4:CHMP4B:SHANK3:TGFBR2:ACKR2:GNAI2:HYAL2:CD200R1:ACKR4:KCNAB1:KCNIP1:HLA-A:FLOT1:TAPBP:PTP4A1:CD36:GNG11:ESYT2:BLK:GFRA2:LYN:GEM:PTPN3
GO_cc	GO_CELL_CELL_CONTACT_ZONE	71	10	0.0003144755677109142	0.0045242623305318825	GJA5:OPALIN:KCNJ11:PCDH9:GJC1:KCNJ2:PKP4:SCN5A:FLOT1:SCRIB
GO_cc	GO_CELL_SURFACE	857	53	0.00031638198115607573	0.0045242623305318825	CLSTN1:MPL:F3:ANXA9:ADAM15:FCGR3A:SELP:FAS:DMBT1:LRFN4:CLMP:KCNA1:PHB2:SLC11A2:LMO7:LRFN5:PSEN1:ADAMTS7:PKD1:GRIN2A:TRPV1:LYZL6:RPS6KB1:DNAI2:MBP:INSR:IL12RB1:CD79A:CEACAM1:BCAM:CD33:TPO:IL1RL1:PLXNB2:TGFBR2:SCN5A:ACKR2:HYAL2:CD200R1:ACKR4:MFI2:AREG:AREGB:CDH9:CDH6:PDGFRB:HLA-A:FLOT1:ITGB8:CD36:ABCB1:GFRA2:CDH17:CEL
GO_cc	GO_GLUTAMATERGIC_SYNAPSE	350	27	0.00038157794176365417	0.00537971154514673	CLSTN1:MTOR:ARHGEF2:LRFN4:KCNA1:PHB2:LRFN5:NUMB:MYO5A:SH3GL3:ACAN:GRIN2A:CORO1A:DGKE:SH3GL1:GIPC1:DNAJB1:GSK3B:EIF4E:ACTBL2:FLOT1:CNR1:PRKAR2B:PPP3CC:LYN:CALB1:SCRIB
GO_cc	GO_CELL_SUBSTRATE_JUNCTION	409	30	0.0004372198923063771	0.0060048074440720136	RPL22:FBLIM1:CDC42:PRUNE:ARHGEF2:CSRP1:ARL2:REXO2:HSPA8:LCP1:LMO7:NUMB:ARMC5:TGFB1I1:RPL19:RPL38:CD97:PPP1CB:RND3:TM4SF20:TGM2:PDGFRB:MDC1:FLOT1:RPS18:ITGB8:HSPB1:YWHAG:RPL7:RPL12
GO_cc	GO_CELL_CELL_JUNCTION	447	32	0.0004379130303868701	0.0060048074440720136	SSX2IP:GJA5:ASH1L:ARHGEF2:CRB1:OPALIN:KCNJ11:TRPC6:CLMP:PTPN6:RAP1B:PCDH9:STXBP6:FRMD6:CORO1A:CALB2:GJC1:KCNJ2:NPHS1:CEACAM1:PKP4:SCN5A:CDH9:CDH6:OCLN:PCDHGA12:FLOT1:CLDN3:CLDN4:LYN:CDH17:SCRIB
GO_cc	GO_SUPRAMOLECULAR_COMPLEX	936	56	0.00048728586164946977	0.006591529020420531	LDLRAP1:MACF1:HOOK1:BCL10:TMOD4:FLG:RUSC1:ARHGEF2:LMNA:ASPM:PSEN2:TUBB8:REEP3:ACTA2:KRTAP5-1:KRTAP5-2:KRTAP5-3:KRTAP5-4:KRTAP5-5:CLMP:LCP1:FAM179B:MYO5A:CORO1A:MYLPF:DNAH9:MFAP4:NBR1:TUBD1:DNAI2:KIF19:TUBB4A:KEAP1:DCTN1:TTLL4:BFSP1:KRTAP21-3:KRTAP8-1:FBLN1:GTSE1:SCO2:SCN5A:ACKR2:RASSF1:CCDC66:SRPRB:SLAIN2:KIF2A:LRRC16A:TUBB:MAP7:SYNE1:TCTE3:HSPB1:CALD1:TUBB4B
GO_cc	GO_CYTOSOLIC_LARGE_RIBOSOMAL_SUBUNIT	62	9	0.0004948930969392568	0.006605173200482614	RPL22:RPL3L:RPL19:RPL38:RPL32:RPL14:RPL37:RPL7:RPL12
GO_cc	GO_BASOLATERAL_PLASMA_MEMBRANE	214	19	0.0005033885316813388	0.006630156844908161	LDLRAP1:ORAI1:NUMB:SLC51B:PKD1:SLC7A6:KCNJ16:SLC14A1:CEACAM1:ATP6V1B1:SLC9A4:SLC4A4:MTTP:FLOT1:MAP7:CLDN4:CA2:CDH17:SCRIB
GO_cc	GO_NUCLEAR_MEMBRANE	302	24	0.0005149750890453727	0.006694676157589845	TMEM57:RBM15:CERS2:LMNA:PSEN2:GRK5:TM7SF2:TMEM120B:P2RX2:IPO5:PSEN1:NPIPA1:KPNB1:UTP18:ENTHD2:PHLPP1:PUM2:XPO1:ACKR2:NDUFB4:SENP2:NRM:SYNE1:RB1CC1
GO_cc	GO_CILIUM	593	39	0.0005978502188827303	0.007672411142328373	HTR6:SSX2IP:ADAM15:TACR2:DNAJB13:AKAP3:FAM179B:PSEN1:MYO5A:PKD1:DNAH9:B9D1:LYZL6:TUBD1:DNAI2:KIF19:AZI1:PHLPP1:TUBB4A:DCTN1:MERTK:TTLL4:DAW1:MCHR1:WBP2NL:SHANK3:MYRIP:HYAL3:CCDC66:ARL13B:IFT57:NPHP3:DZIP1L:KIF2A:C5orf30:SEPT7:PRKAR2B:CEP41:LRRC6

GO_cc	GO_AXON	600	39	0.0007459501325190374	0.009451849147488055	TMEM57:LDLRAP1:PTBP2:KCNC4:HCN3:SYT11:UHMK1:ZFYVE27:APBB1:KCNJ11:HSPA8:KCNA1:PHB2:ULK1:PCDH9:PSEN1:MYO5A:CORO1A:SPG7:MBP:TUBB4A:INSR:DCTN1:FAM168B:SCN3A:CREB1:SCN5A:TRAK1:GSK3B:KCNAB1:CPEB4:C4A:CNR1:MAP7:CRHR2:HSPB1:EXOC4:CA2:CALB1
GO_cc	GO_CILIARY_PART	443	31	0.0007758895535483997	0.00970831803877435	SSX2IP:TACR2:DNAJB13:AKAP3:FAM179B:PSEN1:MYO5A:PKD1:DNAH9:B9D1:LYZL6:DNAI2:KIF19:AZI1:PHLPP1:TUBB4A:DCTN1:MERTK:MCHR1:SHANK3:MYRIP:HYAL3:CCDC66:ARL13B:IFT57:DZIP1L:KIF2A:C5orf30:SEPT7:PRKAR2B:CEP41
GO_cc	GO_TRANSPORT_VESICLE	387	28	0.0008223671880545809	0.010162834015341179	OVGP1:SYT11:SEC16B:PSEN2:ABCC8:SYT13:PSEN1:GRIN2A:SEPT1:SEPT1:RNF112:GIPC1:SYCN:CEACAM1:KDELR1:ATP6V1B1:MFF:TMEM230:DNAJC5:SYNGR1:MYRIP:STX19:COPB2:AREG:AREGB:MCTP1:EDN1:HLA-A:BET1:COPG2
GO_cc	GO_POLYMERIC_CYTOSKELETAL_FIBER	706	44	0.0008412791506744146	0.010269761339330353	LDLRAP1:MACF1:HOOK1:BCL10:FLG:RUSC1:ARHGEF2:LMNA:ASPM:TUBB8:REEP3:KRTAP5-1:KRTAP5-2:KRTAP5-3:KRTAP5-4:KRTAP5-5:CLMP:LCP1:FAM179B:MYO5A:CORO1A:DNAH9:TUBD1:DNAI2:KIF19:TUBB4A:KEAP1:DCTN1:TTLL4:BFSP1:KRTAP21-3:KRTAP8-1:GTSE1:ACKR2:RASSF1:CCDC66:SRPRB:SLAIN2:KIF2A:LRRC16A:TUBB:MAP7:TCTE3:TUBB4B
GO_cc	GO_TETHERING_COMPLEX	67	9	0.0008822797343001588	0.010640506193186253	CDC42:HOOK1:VPS51:STXBP6:TRAPPC2L:MYRIP:TMEM115:VPS52:EXOC4
GO_cc	GO_TRANSFERASE_COMPLEX	772	47	0.000932501200461581	0.011112305972167173	PHC2:CDC20:RBM15:WDR77:CKS1B:SNRPE:CUL2:CTR9:UBE2L6:SYVN1:HSPA8:ERC1:SPSB2:FBXW8:PRKAB1:ULK1:POLE:LMO7:DCAF11:FBXO33:DCAF4:GTF2A1:MGA:POLR2C:PRPSAP2:BRCA1:TAF4B:INSR:KEAP1:DCAF15:FBXO17:TRMT61B:ANAPC1:RPN2:ZNFX1:RAD18:ARMC8:KLHL2:TERT:CCNO:CKS1B:TSPAN17:CDKN1A:GTF2H5:ASH2L:RB1CC1:ENY2:DPM2
GO_cc	GO_SECRETORY_GRANULE_MEMBRANE	297	23	0.0009452970776702631	0.011132263232328628	NRAS:FCGR2A:SELP:RAB18:PSAP:DMBT1:NCKAP1L:RAP1B:PSEN1:CHRNB4:RND2:CD97:SYCN:CEACAM1:CD33:FPR2:SLC11A1:DNAJC5:SYNGR1:HYAL3:DIAPH1:RAB44:CD36
GO_cc	GO_UBIQUITIN_LIGASE_COMPLEX	281	22	0.0010470366459480291	0.012187019565046244	PHC2:CDC20:CKS1B:CUL2:UBE2L6:SYVN1:HSPA8:SPSB2:FBXW8:LMO7:DCAF11:FBXO33:DCAF4:BRCA1:KEAP1:DCAF15:FBXO17:ANAPC1:RAD18:ARMC8:KLHL2:CKS1B:TSPAN17
GO_cc	GO_LATERAL_PLASMA_MEMBRANE	56	8	0.001120905756410944	0.012896858185831667	ARL2:PKD1:CEACAM1:ATP6V1B1:SCN5A:OCLN:CLDN3:CLDN4
GO_cc	GO_POTASSIUM_CHANNEL_COMPLEX	98	11	0.0011515903848905698	0.013099340628130231	KCNC4:KCNJ11:ABCC8:KCNA1:KCNJ16:KCNJ2:KCNJ14:KCNS1:KCNAB1:KCNIP1:KCNV2
GO_cc	GO_GOLGI_CISTERNA	115	12	0.001334477453418199	0.015009122818782215	A3GALT2:GOLPH3L:CIT:SCFD1:GOLGA8G:GOLGA8J:GOLGA8R:GOLGA8H:SMPD3:TMEM115:FUT9:BET1
GO_cc	GO_CLUSTER_OF_ACTIN_BASED_CELL_PROJECTIONS	148	14	0.0014443022890814436	0.01606385101522806	CDH23:SLC11A2:CALB2:FSCN2:GIPC1:PLB1:VIL1:SLC34A2:SLC6A19:SLC6A18:GRXCR2:CD36:CALB1:SLC34A3
GO_cc	GO_PHAGOCYTIC_CUP	24	5	0.0017487417162350522	0.01902707019512269	SYT11:PEAR1:LCP1:CORO1A:MEGF10
GO_cc	GO_REGION_OF_CYTOSOL	24	5	0.0017487417162350522	0.01902707019512269	MTOR:HSPA8:EIF4E:PPP3CC:CALB1
GO_cc	GO_MICROBODY	135	13	0.0017914342167662494	0.0192819962471292	SZT2:ABCD3:PEX16:DAO:MYO5A:LONP2:PEX11G:CD33:PEX13:MFF:ACAD11:TTC1:PEX7
GO_cc	GO_NUCLEAR_SPECK	389	27	0.0018362175585621935	0.01955376357575272	TARDBP:RBM15:HIPK1:BCAS2:SCNM1:CLK2:LMNA:SFMBT2:GRK5:APBB1:CTR9:C12orf57:WBP4:RBM25:TCF12:CD2BP2:ENTHD2:TCF3:DDX39A:ZNF638:DUSP11:SLC2A4RG:TAB1:TERT:LRRC16A:STK19:NCAPG2
GO_cc	GO_MICROTUBULE	410	28	0.0019353906593304337	0.020392905789365937	MACF1:HOOK1:BCL10:RUSC1:ARHGEF2:ASPM:TUBB8:REEP3:CLMP:FAM179B:MYO5A:DNAH9:TUBD1:DNAI2:KIF19:TUBB4A:DCTN1:TTLL4:GTSE1:RASSF1:CCDC66:SRPRB:SLAIN2:KIF2A:TUBB:MAP7:TCTE3:TUBB4B
GO_cc	GO_PLASMA_MEMBRANE_RECEPTOR_COMPLEX	187	16	0.0019746150579141777	0.020589475760125958	OLFM3:PTPN6:CHRNB4:GRIN2A:INSR:IL12RB1:CD79A:CEACAM1:GRIN2D:IL18R1:IL18RAP:LIME1:SHANK3:ITGB8:LYN:SCRIB

GO_cc	GO_ACTIN_CYTOSKELETON	491	32	0.0020347231370552977	0.020961709246794975	FBLIM1:MACF1:TMOD4:ARHGEF2:ABL2:ACTA2:LCP1:MYO5A:MESDC1:CORO1A:MYLPF:CALB2:FSCN2:MYL12A:MYL12B:SH3GL1:KEAP1:DCTN1:RTKN:VIL1:DSTN:MYRIP:ACKR2:TRMT10A:KLHL2:FCHSD1:LRRC16A:FLOT1:SEPT7:CALD1:DENND2A:CALB1
GO_cc	GO_TRANS_GOLGI_NETWORK_MEMBRANE	90	10	0.0020521953108750325	0.020961709246794975	GOLPH3L:SCAMP3:TMEM79:VPS51:LGR5:ENTHD2:AP1M2:MMP24:RAB43:VPS52
GO_cc	GO_AZUROPHIL_GRANULE	154	14	0.002101958804060553	0.021253139018834483	HRNR:PSAP:RAP1B:PSEN1:VAT1:DNAJC5:SYNGR1:CYB5R3:ARSA:PLAC8:TUBB:RAB44:RNASET2:TUBB4B
GO_cc	GO_ACTIN_BASED_CELL_PROJECTION	206	17	0.002136955005137684	0.021390919601428214	CDC42:CRB1:CDH23:ACTA2:APBB1:LCP1:MYO5A:CALB2:FSCN2:CEACAM1:ATP6V1B1:VIL1:HYAL2:GRXCR2:EXOC4:CA2:CALB1
GO_cc	GO_CYTOPLASMIC_SIDE_OF_MEMBRANE	173	15	0.002364272674024852	0.023432048977216603	LDLRAP1:STAC2:PKP4:CHMP4B:SHANK3:GNAI2:KCNAB1:KCNIP1:PTP4A1:GNG11:ESYT2:BLK:LYN:GEM:PTPN3
GO_cc	GO_BRUSH_BORDER_MEMBRANE	50	7	0.002548906185573981	0.025014265605485832	SLC11A2:PLB1:SLC34A2:SLC6A19:SLC6A18:CD36:SLC34A3
GO_cc	GO_VACUOLAR_MEMBRANE	399	27	0.0026169924566408985	0.025420590273909895	MTOR:SZT2:TSPAN1:LAMTOR5:TMEM79:C1orf85:PSAP:HSPA8:SLC11A2:RAP1B:ULK1:PSEN1:MYLPF:ABCC11:PLEKHM1:NAPG:UBXN6:AP1M2:ATP6V1B1:RPN2:DNAJC5:SYNGR1:NPRL2:AP2M1:MAN2B2:FLOT1:RAB44
GO_cc	GO_COATED_VESICLE_MEMBRANE	175	15	0.002641100288198431	0.025420590273909895	LDLRAP1:SEC16B:HSPA8:KIAA1199:EPN2:ENTHD2:AP1M2:KDELR1:DNAJC5:ARFGAP3:COPB2:AP2M1:AREG:AREGB:HLA-A:COPG2
GO_cc	GO_CELL_CORTEX_PART	176	15	0.002789241381769403	0.026590767839534974	STXBP6:CORO1A:SEPT1:SEPT1:CALB2:MYL12B:DCTN1:RTKN:DSTN:MYRIP:FCHSD1:FLOT1:SEPT7:EXOC4:CALD1:CALB1
GO_cc	GO_NUCLEAR_TRANSCRIPTION_FACTOR_COMPLEX	195	16	0.003008053174640719	0.028406237998258112	MED8:ARNT:YAP1:POU2AF1:GTF2A1:TCF12:NFATC3:TAF4B:TCF3:CREB1:PPARA:HYAL2:NR1I2:GTF2H5:KLF4:GTF3C5
GO_cc	GO_POSTSYNAPTIC_CYTOSOL	17	4	0.003203992568515157	0.029973799636296	MTOR:HSPA8:EIF4E:CALB1
GO_cc	GO_9PLUS2_MOTILE_CILIUM	96	10	0.003308412214872295	0.030664079880436734	TACR2:DNAJB13:AKAP3:DNAH9:LYZL6:DNAI2:WBP2NL:HYAL3:KIF2A:SEPT7
GO_cc	GO_EXTRINSIC_COMPONENT_OF_PLASMA_MEMBRANE	163	14	0.0035373677993012203	0.03219910232871693	NUMB:STAC2:SHANK3:GNAI2:KCNAB1:CDH9:CDH6:KCNIP1:GNG11:ESYT2:BLK:LYN:CDH17:SCRIB
GO_cc	GO_REPLISOME	28	5	0.0035705298286589204	0.03219910232871693	BCAS2:ERCC5:PCNA:WRNIP1:XPA
GO_cc	GO_GOLGI_CIS_CISTERNA	28	5	0.0035705298286589204	0.03219910232871693	GOLGA8G:GOLGA8J:GOLGA8R:GOLGA8H:SMPD3
GO_cc	GO_CUTICULAR_PLATE	9	3	0.003762982082161175	0.03348466864866862	CALB2:FCHSD1:CALB1
GO_cc	GO_GOLGI_STACK	147	13	0.003779987569729824	0.03348466864866862	A3GALT2:GOLPH3L:CIT:SCFD1:ZFYVE1:GOLGA8G:GOLGA8J:GOLGA8R:GOLGA8H:SMPD3:TMEM115:FUT9:BET1
GO_cc	GO_REPLICATION_FORK	68	8	0.003928606223261378	0.034495919556882804	BCAS2:MCM10:ERCC5:PCNA:RAD18:WRNIP1:RFC2:XPA
GO_cc	GO_CORTICAL_ACTIN_CYTOSKELETON	83	9	0.003979964705232893	0.03449929418150344	CORO1A:CALB2:RTKN:DSTN:MYRIP:FCHSD1:FLOT1:CALD1:CALB1
GO_cc	GO_COPI_COATED_VESICLE_MEMBRANE	18	4	0.003997920204849549	0.03449929418150344	KDELR1:ARFGAP3:COPB2:COPG2

GO_cc	GO_CENTRIOLAR_SATELLITE	29	5	0.0041826195113069484	0.035784633596737225	SSX2IP:AZI1:ZMYND10:CCDC66:FLOT1
GO_cc	GO_FIBRILLAR_CENTER	133	12	0.004498938009960164	0.038164719898051896	FBLIM1:YY1AP1:NUAK1:CD2BP2:PRMT7:TAF4B:CC2D1A:DUSP11:IPO11:UTP15:JAZF1:TERF1
GO_cc	GO_VESICLE_LUMEN	338	23	0.00486041280906687	0.040884648923327195	HRNR:HSPA6:F5:HPX:LINC00610:COMMD9:HSPA8:PTPN6:APAF1:VAT1:KPNB1:PSMD12:SLPI:CYB5R3:ARSA:PROS1:ARMC8:CXCL1:PLAC8:TUBB:RNASET2:FAM3C:TUBB4B:TOR4A
GO_cc	GO_CENTRIOLE	135	12	0.005067906972149549	0.042136666414620425	STIL:HSPA6:HERC2:TUBD1:TSKS:DCTN1:DZIP1L:KIF2A:CETN3:FGFR1OP:CEP41:CNTLN
GO_cc	GO_EXTRACELLULAR_MATRIX	522	32	0.005093443192976096	0.042136666414620425	F3:CTSS:HRNR:FLG:APCS:FMOD:NID1:RBP3:PSAP:DMBT1:HPX:CDON:POSTN:COCH:ACAN:HAPLN3:TGFB1I1:MFAP4:ADAM11:PODNL1:BCAM:MMP24:TGM2:PI3:SLPI:DGCR6:FBLN1:LRIG1:RELL2:ADAM19:THBS2:COL22A1
GO_cc	GO_SPERM_HEAD	10	3	0.005226106883109447	0.04287977860649637	TACR2:DNAJB1:WBP2NL
GO_cc	GO_COATED_VESICLE	282	20	0.005302740601391218	0.043154823918639104	LDLRAP1:F5:SEC16B:HSPA8:NUMB:KIAA1199:EPN2:ENTHD2:DENND1C:AP1M2:KDELR1:DNAJC5:FAM109B:ARFGAP3:COPB2:AP2M1:AREG:AREGB:EDN1:HLA-A:COPG2
GO_cc	GO_EXOCYTIC_VESICLE	208	16	0.005613672379582457	0.04531682299969387	SYT11:PSEN2:ABCC8:SYT13:PSEN1:GRIN2A:SEPT1:SEPT1:RNF112:GIPC1:ATP6V1B1:MFF:TMEM230:DNAJC5:SYNGR1:STX19:MCTP1
GO_cc	GO_MIDBODY	172	14	0.005687325032656954	0.04554409886151689	CDC42:ASPM:CIT:SEPT1:SEPT1:KEAP1:PKN1:PKP4:CHMP4B:GNAI2:C6orf89:SEPT7:MPLKIP:EXOC4:GEM
GO_cc	GO_EXTRINSIC_COMPONENT_OF_CYTOPLASMIC_SIDE_OF_PLASMA_MEMBRANE	88	9	0.005861778987836179	0.04656857751447631	STAC2:SHANK3:GNAI2:KCNAB1:KCNIP1:GNG11:ESYT2:BLK:LYN
GO_cc	GO_GABA_ERGIC_SYNAPSE	73	8	0.006064293114465191	0.047798089823461856	CLSTN1:LRFN4:PHB2:LRFN5:ACAN:FLOT1:CNR1:CALB1
GO_cc	GO_CULLIN_RING_UBIQUITIN_LIGASE_COMPLEX	156	13	0.006220156192965271	0.04864356522779872	CDC20:CKS1B:CUL2:SPSB2:FBXW8:DCAF11:FBXO33:DCAF4:KEAP1:DCAF15:FBXO17:ANAPC1:KLHL2:CKS1B
Hallmark_gene_sets	HALLMARK_EPITHELIAL_MESENCHYMAL_TRANSITION	199	20	6.834118037378431e-05	0.0034170590186892156	FMOD:ACTA2:FAS:ENO2:PTHLH:POSTN:IGFBP2:TGM2:FBLN1:IL8:CXCL6:CXCL1:AREG:AREGB:CDH6:PDGFRB:TNFAIP3:THBS2:MEST:CALD1:GEM
Hallmark_gene_sets	HALLMARK_UV_RESPONSE_DN	144	15	0.00036163047185891483	0.005952024977833393	LPHN2:DDAH1:F3:GRK5:CDON:FAM179B:RND3:IGFBP5:TGFBR2:ATP2C1:RASA2:NIPBL:PDGFRB:SYNE1:PRKAR2B
Hallmark_gene_sets	HALLMARK_PEROXISOME	104	12	0.0005449170053470866	0.005952024977833393	BCL10:ABCD3:FDPS:ABCC8:LONP2:SIAH1:PEX13:NR1I2:CNBP:SOD2:ABCB1:CEL
Hallmark_gene_sets	HALLMARK_INTERFERON_GAMMA_RESPONSE	200	18	0.0005952024977833393	0.005952024977833393	SELP:FAS:UBE2L6:PTPN6:C1S:PSME1:IFI35:TRIM25:MTHFD2:ZNFX1:ZBP1:HLA-A:TAPBP:CDKN1A:TNFAIP3:SOD2:ARL4A:LY6E
Hallmark_gene_sets	HALLMARK_INFLAMMATORY_RESPONSE	200	18	0.0005952024977833393	0.005952024977833393	F3:SLC11A2:PSEN1:CCL17:KCNJ2:IL18R1:IL18RAP:ATP2C1:SLC4A4:IL8:CXCL6:PTGER4:EDN1:TAPBP:CDKN1A:ITGB8:LYN:LY6E
Hallmark_gene_sets	HALLMARK_APOPTOSIS	161	15	0.0011607693438899641	0.009673077865749702	BCL10:DAP3:LMNA:PSEN2:FAS:WEE1:ENO2:EMP1:PSEN1:BRCA1:PDGFRB:IER3:CDKN1A:SOD2:HSPB1
Hallmark_gene_sets	HALLMARK_ESTROGEN_RESPONSE_LATE	200	17	0.0015600931094926875	0.009750581934329297	NBL1:CDC20:STIL:HMGCS2:ANXA9:PTPN6:RABEP1:IMPA2:CHST8:CACNA2D2:AREG:AREGB:NPY1R:PRKAR2B:MEST:CA2:KLF4:FAM102A
Hallmark_gene_sets	HALLMARK_E2F_TARGETS	200	17	0.0015600931094926875	0.009750581934329297	CDC20:CKS1B:IPO7:WEE1:CIT:POLE:DLGAP5:UBR7:DCTPP1:BRCA1:DDX39A:XPO1:MTHFD2:PCNA:CKS1B:TUBB:CDKN1A:RFC2

Hallmark_gene_sets	HALLMARK_BILE_ACID_METABOLISM	112	11	0.0033575106490019023	0.017527659000310315	ABCD3:BBOX1:PEX16:LONP2:PEX11G:LIPE:PEX13:CYP8B1:NR1I2:RBP1:PEX7
Hallmark_gene_sets	HALLMARK_COMPLEMENT	200	16	0.0038560849800682692	0.017527659000310315	F3:CTSS:F5:CSRP1:RCE1:FDX1:C1S:PSEN1:GNAI2:GP9:CXCL1:KIF2A:TNFAIP3:CD36:LYN:CA2
Hallmark_gene_sets	HALLMARK_P53_PATHWAY	200	16	0.0038560849800682692	0.017527659000310315	PHLDA3:ADCK3:FAS:CSRNP2:APAF1:PRKAB1:ERCC5:PITPNC1:TPRKB:PCNA:PLXNB2:ANKRA2:HDAC3:IER3:CDKN1A:KLF4
Hallmark_gene_sets	HALLMARK_KRAS_SIGNALING_UP	199	15	0.008582982398427712	0.03448555519580487	TSPAN1:PTBP2:CTSS:EMP1:LCP1:ERO1L:MYCN:SLPI:MAP7:TNFAIP3:LAT2:ABCB1:GNG11:CA2:KLF4
Hallmark_gene_sets	HALLMARK_ESTROGEN_RESPONSE_EARLY	200	15	0.008966244350909267	0.03448555519580487	NBL1:ANXA9:FAM63A:ESRP2:CALB2:GREB1:TGM2:SLC37A1:SYNGR1:CELSR1:LRIG1:AREG:AREGB:NPY1R:KLF4:FAM102A
Hallmark_gene_sets	HALLMARK_UNFOLDED_PROTEIN_RESPONSE	113	10	0.010323309609710086	0.03686896289182173	EXOSC10:ZBTB17:CKS1B:ALDH18A1:ERO1L:MTHFD2:DCTN1:SRPRB:EIF4E:SKIV2L2:CKS1B
Cancer_modules	MODULE_136	489	47	5.334983067664287e-09	2.2993777021633077e-06	TAL1:F3:DPH5:GJA5:SELP:ADCK3:CDH23:STAMBPL1:TRUB1:ZNF143:SLC43A1:RBM7:SLC11A2:LMO7:KDELC1:NRL:COCH:PRPF39:MAPKBP1:RFX7:SIAH1:SPAG9:PITPNC1:MYL12A:EPS15L1:FPR2:GREB1:PPP1CB:ZNF638:IL1RL1:IL18R1:TYMP:HYAL2:C3orf17:IL8:MTTP:C5orf20:WRNIP1:EDN1:WDR46:PLEKHG1:ZNF117:RFC2:ZMAT4:DIRAS2:NELFB:TOR4A
Cancer_modules	MODULE_69	524	48	1.6942680599067022e-08	3.6511476690989433e-06	TAL1:F3:GJA5:SELP:ABL2:ADCK3:STAMBPL1:ZNF143:SLC43A1:RBM7:SLC11A2:LMO7:KDELC1:NRL:COCH:PRPF39:MAPKBP1:RFX7:SIAH1:SPAG9:PITPNC1:TTYH2:MYL12A:CHAF1A:EPS15L1:FPR2:GREB1:PPP1CB:ZNF638:IL1RL1:IL18R1:TYMP:HYAL2:C3orf17:IL8:MTTP:NPY1R:C5orf20:WRNIP1:EDN1:WDR46:PLEKHG1:ZNF117:RFC2:ZMAT4:DIRAS2:NELFB:TOR4A
Cancer_modules	MODULE_37	469	42	2.4208415201244025e-07	3.477942317245392e-05	TAL1:GJA5:ABL2:ADCK3:STAMBPL1:ZNF143:SLC43A1:RBM7:SLC11A2:LMO7:KDELC1:NRL:COCH:MAPKBP1:SIAH1:SPAG9:PITPNC1:MYL12A:CHAF1A:EPS15L1:FPR2:GREB1:PPP1CB:ZNF638:IL1RL1:IL18R1:HYAL2:C3orf17:IL8:MTTP:NPY1R:C5orf20:WRNIP1:EDN1:WDR46:PLEKHG1:ZNF117:RFC2:ZMAT4:DIRAS2:NELFB:TOR4A
Cancer_modules	MODULE_88	829	61	6.021865298890539e-07	6.488559859554557e-05	CDC20:TSPAN1:PDZK1IP1:SLC16A4:HMGCS2:SEMA6C:HCN3:APCS:HSPA6:LRIT1:DMBT1:APBB1:BBOX1:SLC43A1:UBE2L6:TM7SF2:C1S:LGR5:DAO:SDS:PCK2:SIX6:CHRNB4:CORO1A:ASGR2:RCVRN:HNF1B:SSTR2:NFKBIB:CD79A:BCAM:CD33:FPR2:IGFBP2:TGM2:SLPI:APOL1:ARFGAP3:GTSE1:SCO2:HYAL1:RBP1:SLC4A4:CFI:OCLN:LECT2:ADAM19:PRSS16:IER3:CDKN1A:GSTA2:ESR1:ARL4A:CLDN3:CLDN4:PIP:CA2:ST6GALNAC4:CEL:FCN2:PAEP
Cancer_modules	MODULE_163	562	45	2.0216533679757273e-06	0.0001742665203195077	ICMT:TXNDC12:HOOK1:RBM15:LAMTOR5:SEMA6C:CRB1:TLL2:KCNJ11:BBOX1:DAO:RFX7:RASL12:ADAMTS7:SIAH1:METTL16:TAF4B:S1PR5:SLC6A16:FPR2:HPCAL1:MYCN:KIAA1841:RBMS1:VIL1:ZNF343:SEMG1:TYMP:C3orf55:STK32B:ATP10D:C4orf17:TERT:CDX1:WDR46:HCRTR2:INMT:BCAP29:CPA1:GIMAP5:STAR:ADAMTSL1:SPATA31A4:SPATA31A7:ZNF79:LRSAM1
Cancer_modules	MODULE_55	826	58	4.808033745922994e-06	0.00034537709074880177	ICMT:CDC20:PDZK1IP1:SLC16A4:HMGCS2:SEMA6C:HCN3:APCS:HSPA6:LRIT1:DMBT1:APBB1:BBOX1:SLC43A1:UBE2L6:TM7SF2:C1S:LGR5:DAO:SDS:PCK2:SIX6:CHRNB4:ASGR2:RCVRN:HNF1B:SSTR2:NFKBIB:POU2F2:BCAM:CD33:FPR2:IGFBP2:TGM2:SLPI:APOL1:ARFGAP3:GTSE1:SCO2:TGFBR2:HYAL1:RBP1:SLC4A4:CFI:OCLN:LECT2:ADAM19:PRSS16:IER3:GSTA2:ESR1:CLDN3:PIP:CA2:ST6GALNAC4:CEL:FCN2:PAEP
Cancer_modules	MODULE_105	200	22	7.319116892089127e-06	0.0003629798717063516	ACOT7:CKS1B:ACTA2:WEE1:EMG1:EMP1:PSEN1:TCF12:HELZ:PPP4R1:CHAF1A:KEAP1:ERF:PAFAH1B3:PCNA:RBP1:CKS1B:MGAT1:THBS2:HSPB1:FAM3C:PUF60:KIAA0020
Cancer_modules	MODULE_255	448	37	8.01510391230578e-06	0.0003629798717063516	TAL1:GJA5:SEMA4A:SELP:ADCK3:STAMBPL1:ZNF143:SLC43A1:RBM7:METTL7A:SLC11A2:KDELC1:NRL:COCH:PRPF39:MAPKBP1:SIAH1:PITPNC1:MYL12A:DCAF15:EPS15L1:FPR2:PPP1CB:IL1RL1:IL18R1:C3orf17:IL8:C5orf20:WRNIP1:WDR46:PLEKHG1:ZNF117:ZMAT4:GEM:DIRAS2:NELFB:TOR4A
Cancer_modules	MODULE_179	466	38	8.136241509667602e-06	0.0003629798717063516	TAL1:GJA5:SEMA4A:SELP:ADCK3:STAMBPL1:ZNF143:SLC43A1:RBM7:METTL7A:SLC11A2:KDELC1:NRL:COCH:PRPF39:MAPKBP1:SIAH1:PITPNC1:MYL12A:DCAF15:EPS15L1:FPR2:GREB1:PPP1CB:IL1RL1:IL18R1:C3orf17:IL8:C5orf20:WRNIP1:WDR46:PLEKHG1:ZNF117:ZMAT4:GEM:DIRAS2:NELFB:TOR4A
Cancer_modules	MODULE_95	557	43	8.421806768128807e-06	0.0003629798717063516	ICMT:TXNDC12:HOOK1:RBM15:LAMTOR5:SEMA6C:CRB1:TLL2:KCNJ11:BBOX1:DAO:RFX7:RASL12:ADAMTS7:SIAH1:METTL16:TAF4B:S1PR5:SLC6A16:FPR2:HPCAL1:MYCN:KIAA1841:RBMS1:VIL1:ZNF343:SEMG1:TYMP:C3orf55:STK32B:ATP10D:C4orf17:TERT:CDX1:WDR46:HCRTR2:INMT:BCAP29:CPA1:GIMAP5:STAR:ADAMTSL1:ZNF79

Cancer_modules	MODULE_205	305	28	1.4848390858244885e-05	0.0005817869509003223	EXTL2:VAV3:SEMA6C:LCE1B:KCNJ11:STXBP6:FANCM:DLGAP5:B9D1:SLC47A1:LYZL6:CACNB1:NAPG:TUBB4A:MUC16:NPHS1:ZNF83:GREB1:TTLL4:THUMPD3:MYRIP:CACNA2D2:CD200R1:FCHSD1:PRSS16:CNR1:ZNF117:CEP41
Cancer_modules	MODULE_459	429	35	1.803346987963122e-05	0.0006477021265100881	TAL1:GJA5:SEMA4A:ADCK3:STAMBPL1:ZNF143:SLC43A1:RBM7:METTL7A:SLC11A2:KDELC1:NRL:COCH:PRPF39:MAPKBP1:SIAH1:PITPNC1:MYL12A:DCAF15:EPS15L1:FPR2:IL1RL1:IL18R1:C3orf17:IL8:C5orf20:WRNIP1:WDR46:PLEKHG1:ZNF117:ZMAT4:GEM:DIRAS2:NELFB:TOR4A
Cancer_modules	MODULE_5	433	35	2.1900277623130787e-05	0.0006742156896835264	TSPAN1:F3:FCGR2A:FCGR3A:CSRP1:GRK5:DMBT1:UBE2L6:C1S:GPRC5A:POSTN:LCP1:CORO1A:MFAP4:PPP4R1:CD97:BCAM:RBMS1:TGM2:SLPI:FBLN1:TYMP:TGFBR2:CACNA2D2:IL8:CXCL1:PDGFRB:ADAM19:IER3:CDKN1A:TNFAIP3:GNG11:CALD1:LYN:ST6GALNAC4
Cancer_modules	MODULE_378	433	35	2.1900277623130787e-05	0.0006742156896835264	TAL1:GJA5:SEMA4A:ADCK3:STAMBPL1:ZNF143:SLC43A1:RBM7:METTL7A:SLC11A2:KDELC1:NRL:COCH:PRPF39:MAPKBP1:SIAH1:PITPNC1:MYL12A:DCAF15:EPS15L1:FPR2:IL1RL1:IL18R1:C3orf17:IL8:C5orf20:WRNIP1:WDR46:PLEKHG1:ZNF117:ZMAT4:GEM:DIRAS2:NELFB:TOR4A
Cancer_modules	MODULE_317	438	35	2.7794075986059643e-05	0.0007986164499994471	TAL1:GJA5:SEMA4A:ADCK3:STAMBPL1:ZNF143:SLC43A1:RBM7:METTL7A:SLC11A2:KDELC1:NRL:COCH:PRPF39:MAPKBP1:SIAH1:PITPNC1:MYL12A:DCAF15:EPS15L1:FPR2:IL1RL1:IL18R1:C3orf17:IL8:C5orf20:WRNIP1:WDR46:PLEKHG1:ZNF117:ZMAT4:GEM:DIRAS2:NELFB:TOR4A
Cancer_modules	MODULE_27	354	30	3.457293347136313e-05	0.0008667661314653399	TIE1:MPL:LPHN2:F3:FCGR2A:FCGR3A:SELP:RGR:FAS:OPCML:GPRC5A:CHRNB4:ASGR2:PTGER1:IL12RB1:CD79A:FPR2:IL18R1:MERTK:PPARA:HYAL2:CFI:NPY1R:PDGFRB:HLA-A:CDKN1A:HCRTR2:CNR1:ITGB8:CLDN4
Cancer_modules	MODULE_408	18	6	3.523822546686423e-05	0.0008667661314653399	PSAP:SMPD1:NAGA:ARSA:HYAL1:NSMAF
Cancer_modules	MODULE_13	517	39	3.619904957395851e-05	0.0008667661314653399	HTR6:SZT2:ADAM15:FAM189B:PMF1:FCGR2A:LRIT1:DMBT1:ZNF143:ABCC8:SAC3D1:AKAP3:LRRC23:LGR5:WBP4:GRIN2A:CIAPIN1:CALB2:ASGR2:DNAH9:LYZL6:TECR:CD33:ATP6V1B1:PI3:GTSE1:ACKR2:IFRD2:RASSF1:KCNAB1:PCDHGC3:MGAT1:PRSS16:MDC1:ESR1:CRHR2:PPP3CC:LY6E:ST6GALNAC4
Cancer_modules	MODULE_36	161	18	3.874581223056444e-05	0.000878918161651225	EXOSC10:SPEN:TM2D1:CSRNP2:UCHL3:ARPP19:CYB5B:TTLL4:PER2:PCNA:CDS2:TRAK1:PROS1:COQ2:NIPBL:ZNF117:ASH2L:NSMAF
Cancer_modules	MODULE_41	562	41	4.924373419379838e-05	0.001061202471876355	SZT2:FCGR2A:LRIT1:DMBT1:ZNF143:ABCC8:SAC3D1:RCE1:AKAP3:HOXC11:LGR5:SIX6:CHRNB4:CIAPIN1:CALB2:ASGR2:DNAH9:CACNB1:PDE4A:C19orf57:CD33:FPR2:IL1RL1:PI3:NDUFA6:CYP2D6:ARFGAP3:GTSE1:ACKR2:CACNA2D2:GP9:KCNAB1:C5orf30:ADAM19:PRSS16:ESR1:CRHR2:DUS4L:PPP3CC:ST6GALNAC4:FCN2
Cancer_modules	MODULE_1	366	30	6.377913451972327e-05	0.0013089908084762253	C1orf63:CTSK:LMNA:CSRP1:FMOD:SLC43A1:C1S:EMP1:ULK1:PKD1:MFAP4:BCAM:IGFBP2:TGM2:SLPI:FBLN1:TGFBR2:RBP1:PDGFRB:ADAM19:IER3:CDKN1A:TNFAIP3:SYNE1:THBS2:GNG11:CALD1:STAR:GEM:KLF4
Cancer_modules	MODULE_532	405	32	7.437818692063649e-05	0.001457136298308833	TAL1:GJA5:ADCK3:STAMBPL1:ZNF143:RBM7:METTL7A:SLC11A2:KDELC1:NRL:COCH:PRPF39:MAPKBP1:SIAH1:MYL12A:DCAF15:EPS15L1:FPR2:IL1RL1:IL18R1:C3orf17:IL8:C5orf20:WRNIP1:WDR46:PLEKHG1:ZNF117:ZMAT4:GEM:DIRAS2:NELFB:TOR4A
Cancer_modules	MODULE_84	541	39	9.559451617015776e-05	0.0017913581073625215	CTSS:TNFAIP8L2:SEMA4A:FCGR2A:HSPA6:FCGR3A:C10orf54:LRIT1:ACTA2:UBE2L6:C1S:SDS:LCP1:KIAA0226L:FOXG1:SIX6:CORO1A:IFI35:CD79A:FPR2:TPO:IGFBP2:TGM2:PI3:SLPI:TYMP:TGFBR2:RBP1:IL8:CXCL1:PLAC8:ADAM19:HLA-A:IER3:CDKN1A:TNFAIP3:SYNE1:LYN:ST6GALNAC4
Cancer_modules	MODULE_45	576	40	0.00017200992038119998	0.003031965359328901	HTR6:CTSS:SEMA6C:FCGR2A:HSPA6:FCGR3A:LRIT1:SLC43A1:UBE2L6:PTPN6:LCP1:SIX6:CORO1A:IFI35:IMPA2:CD97:PKN1:MAST3:NFKBIB:CD79A:FPR2:SLC11A1:PI3:SLPI:SCO2:TYMP:TGFBR2:ACKR2:KCNAB1:IL8:ADAM19:IER3:TNFAIP3:SYNE1:SOD2:CRHR2:GNG11:PPP3CC:LYN:ST6GALNAC4
Cancer_modules	MODULE_48	334	27	0.0001814166566440918	0.003031965359328901	ICMT:TXNDC12:HOOK1:RBM15:SLC16A4:LAMTOR5:TLL2:RFX7:SIAH1:METTL16:S1PR5:HPCAL1:KIAA1841:RBMS1:VIL1:ZNF343:TYMP:TERT:CDX1:WDR46:INMT:BCAP29:CPA1:GIMAP5:STAR:ADAMTSL1:ZNF79
Cancer_modules	MODULE_100	539	38	0.00018304269626003624	0.003031965359328901	F3:DMBT1:DUSP8:APBB1:ABCC8:TM7SF2:TENM4:ENO2:LGR5:PCDH9:FOXG1:SIX6:SH3GL3:PKD1:CORO1A:CALB2:EPN2:SSTR2:MBP:BCL11A:RND3:IGFBP2:SYNGR1:SCO2:MAPK8IP2:CACNA2D2:RBP1:C5orf30:SNCB:CDKN1A:MAP7:SYNE1:THBS2:CA2:CALB1:KHDRBS3:ST6GALNAC4:CEL
Cancer_modules	MODULE_137	540	38	0.00018993750510877105	0.003031965359328901	F3:DMBT1:DUSP8:APBB1:ABCC8:TM7SF2:TENM4:ENO2:LGR5:PCDH9:FOXG1:SIX6:SH3GL3:PKD1:CORO1A:CALB2:EPN2:SSTR2:MBP:BCL11A:RND3:IGFBP2:SYNGR1:SCO2:MAPK8IP2:CACNA2D2:RBP1:C5orf30:SNCB:CDKN1A:MAP7:SYNE1:THBS2:CA2:CALB1:KHDRBS3:ST6GALNAC4:CEL

Cancer_modules	MODULE_87	44	8	0.00020819742695905988	0.0032047532506912432	CDC42:NRAS:RIT1:ARL2:RAP1B:PCK2:GEM:TUBB4B
Cancer_modules	MODULE_9	124	14	0.0002448023921989298	0.0035213464473496034	ICMT:TXNDC12:HOOK1:SLC16A4:HSPA8:RFX7:METTL16:RBMS1:VIL1:TERT:CDX1:BCAP29:STAR:ZNF79
Cancer_modules	MODULE_66	547	38	0.0002451053211612253	0.0035213464473496034	F3:DMBT1:DUSP8:APBB1:ABCC8:TM7SF2:TENM4:ENO2:LGR5:PCDH9:FOXG1:SIX6:SH3GL3:PKD1:CORO1A:CALB2:EPN2:SSTR2:MBP:BCL11A:RND3:IGFBP2:SYNGR1:SCO2:MAPK8IP2:CACNA2D2:RBP1:C5orf30:SNCB:CDKN1A:MAP7:SYNE1:THBS2:CA2:CALB1:KHDRBS3:ST6GALNAC4:CEL
Cancer_modules	MODULE_33	379	29	0.0002711826032698672	0.0037703129680423473	PDZK1IP1:ACTA2:LPCAT3:EMP1:SDS:PKD1:CORO1A:CCL17:SLC7A6:CLEC10A:MAPK7:CACNG1:ERF:PAFAH1B3:CEACAM1:MERTK:SLPI:MAPK8IP2:MAP7:SOD2:ARL4A:RFC2:CD36:CPA1:MEST:CALD1:BLK:KLF4:PTPN3
Cancer_modules	MODULE_212	326	26	0.00029180133271459286	0.003930199199999673	ABCD3:VAV3:CTSS:SOAT1:BBOX1:UBE2L6:HSPA8:PHB2:METTL7A:DAO:SDS:SLC25A15:PCK2:SULT1A3:CD320:RND3:TMEM230:PCNA:ARSA:HYAL1:PPAP2A:FLOT1:GSTA2:MAP7:CA2:CYP11B2
Cancer_modules	MODULE_112	258	22	0.0003337381365785769	0.004272195831491601	F3:CTSK:FCGR3A:GRK5:ZNF143:C1S:DAO:SDS:CCL17:ASGR2:RCVRN:C19orf57:POU2F2:CEACAM1:RND3:CYP2D6:RBP1:NPY1R:ISL1:THBS2:CRHR2:CDH17
Cancer_modules	MODULE_64	517	36	0.00033701776860954624	0.004272195831491601	HTR6:F3:SLC16A4:FCGR2A:FCGR3A:FAS:GRK5:GPRC5A:NCKAP1L:CHRNB4:CCL17:ASGR2:CACNG1:KCNJ2:SSTR2:CD79A:CEACAM1:FPR2:TPO:IL18R1:PPARA:ACKR2:GP9:SLC4A4:IL8:CXCL1:AREG:AREGB:CFI:NPY1R:HLA-A:CDKN1A:CNR1:CRHR2:CLDN4:GNG11:LY6E
Cancer_modules	MODULE_117	719	46	0.0003727359374123195	0.00458997682927742	MPL:RPE65:BCAS2:AMPD1:CTSS:APCS:HSPA6:F5:F13B:FMOD:RGR:BBOX1:SCGB2A1:TRPC6:KCNA1:DAO:CORO1A:CACNG1:FSCN2:SLC14A1:ST8SIA5:PTGER1:IL18R1:IGFBP2:BFSP1:KCNS1:SEMG1:MAPK8IP2:HYAL1:AFM:MTTP:TERT:CDH6:CDX1:C4A:GSTA2:CNR1:CLDN4:CD36:PIP:BLK:CA2:CDH17:LRRC6:KCNV2:CEL
Cancer_modules	MODULE_342	211	19	0.0004226334471832419	0.00505986154822159	FBLIM1:NBL1:HOOK1:VAV3:ANXA9:FMOD:CDCA5:GPRC5A:STXBP6:UBXN6:RTKN:IGFBP2:SLPI:HYAL1:OCLN:CDKN1A:MAP7:CLDN4:CDH17
Cancer_modules	MODULE_146	102	12	0.0004560923306723385	0.0053128593113453484	HTR6:RGR:OPCML:CHRNB4:SSTR2:PTGER1:OR10H3:FPR2:ACKR2:NPY1R:HCRTR2:CNR1
Cancer_modules	MODULE_18	450	32	0.0004910023515286761	0.0055690003554963	ICMT:ACOT7:CDC20:FDPS:PMF1:SLC43A1:LGR5:IPO5:DLGAP5:TCF12:CIAPIN1:CYB5B:VAT1:CHAF1A:DDX39A:NFKBIB:RND3:COPS8:PCNA:APOBEC3B:GTSE1:ACKR2:IFRD2:IL8:CXCL1:C5orf30:MDC1:IER3:PTP4A1:THBS2:GNG11:ST6GALNAC4
Cancer_modules	MODULE_180	118	13	0.0005108106066533516	0.00564511208891268	HOOK1:VAV3:ANXA9:UCP2:METTL7A:ESRP2:UBXN6:BCAM:ISY1:NPY1R:OCLN:MAP7:CLDN4
Cancer_modules	MODULE_49	288	23	0.0006226943275708999	0.006709531379576447	SMCP:NHLH1:WEE1:SYT13:KCNA1:DAO:KLHL28:ZFYVE1:SH3GL3:ADAM11:TAF4B:SH3GL1:KCNJ14:NOL10:ZNF343:NDUFAF5:TYMP:C3orf55:STK32B:IPO11:CDX1:KCNIP1:HCRTR2
Cancer_modules	MODULE_3	382	28	0.0006736688215241973	0.007081738099437294	ACOT7:CDC20:EXTL2:SEMA6C:FDPS:DAP3:IPO7:NDUFA9:EMP1:DLGAP5:CYB5B:VAT1:PPP4R1:SH3GL1:CHAF1A:ERF:RND3:IGFBP2:PCNA:TGM2:APOBEC3B:TGFBR2:IER3:CDKN1A:PTP4A1:GNG11:MEST:CALD1
Cancer_modules	MODULE_545	20	5	0.0007227600354142013	0.007416894649131448	DAO:IL1RL1:IL18R1:SOX30:GSTA2
Cancer_modules	MODULE_94	370	27	0.0008887130754842072	0.008907798500783565	ARHGEF11:SELP:PSEN2:APBB1:CDON:SLC11A2:SDS:SIX6:CCL17:CLEC10A:HNF1B:KEAP1:NFKBIB:CARD8:GREB1:APOL1:SCO2:ENTPD3:HYAL1:ABCF3:MAP3K13:CDX1:SNCB:IER3:B3GALT4:TPST1:KIAA0020
Cancer_modules	MODULE_6	411	29	0.000991485548706895	0.00971205162483345	C1orf63:TSPAN1:F3:HMGCS2:FMOD:DMBT1:C1S:GPRC5A:MFAP4:CD79A:IGFBP2:TGM2:PI3:SLPI:FBLN1:TGFBR2:IL8:CXCL1:SLC12A2:PDGFRB:IER3:CDKN1A:TNFAIP3:CLDN3:CLDN4:PIP:LYN:GEM:ST6GALNAC4
Cancer_modules	MODULE_480	97	11	0.0010576387296793257	0.009916290324570828	PTHLH:PSEN1:TRPM1:IFI35:DNAJB1:FAM49A:SNED1:SYNGR1:SRPRB:CFI:PILRB
Cancer_modules	MODULE_83	318	24	0.0010585032744754388	0.009916290324570828	DAP3:SNRPE:IPO7:FAU:HSPA8:ATN1:PHB2:RAP1B:RPL19:RPL38:MYL12A:MYL12B:XPO1:RBMS1:NCL:SEMG2:RPL32:RPL14:RPL37:RPS18:HSPB1:YWHAG:RPL7:RPL12
Cancer_modules	MODULE_176	228	19	0.0010813588056956588	0.009916290324570828	F3:CTSK:FCGR3A:GRK5:ZNF143:C1S:SDS:CCL17:ASGR2:C19orf57:POU2F2:CEACAM1:RND3:CYP2D6:NPY1R:ISL1:THBS2:CRHR2:CDH17

Cancer_modules	MODULE_11	537	35	0.0012929263351339699	0.011609401050890438	F3:DMBT1:DUSP8:APBB1:ABCC8:TM7SF2:TENM4:ENO2:LGR5:PCDH9:FOXG1:MOAP1:SH3GL3:PKD1:CORO1A:CALB2:EPN2:SSTR2:MBP:BCL11A:IGFBP2:SYNGR1:SCO2:MAPK8IP2:CACNA2D2:RBP1:C5orf30:SNCB:MAP7:SYNE1:THBS2:CA2:CALB1:KHDRBS3:ST6GALNAC4
Cancer_modules	MODULE_8	419	29	0.0013301908883687802	0.011700250467080496	CDC20:ABCD3:SEMA6C:FDPS:DAP3:IPO7:WEE1:SLC43A1:NDUFA9:SLC11A2:IPO5:DCAF11:ARPP19:ASGR2:CHAF1A:DDX39A:RND3:VIL1:PCNA:RBP1:IL8:CXCL1:AREG:AREGB:OCLN:IER3:PTP4A1:ARL4A:MEST:KIAA0020
Cancer_modules	MODULE_72	307	23	0.0014641958944668426	0.012621368610304183	CDC20:ABCD3:DAP3:CREM:SAC3D1:AKAP3:C1S:ULK1:MTMR6:FOXG1:ARPP19:CALB2:UTP18:PPP4R1:SAFB2:DDX39A:NFKBIB:IGFBP2:PCNA:RBP1:PDGFRB:ARL4A:KHDRBS3
Cancer_modules	MODULE_118	403	28	0.0015070195140548372	0.012735792363875193	CDC20:FDPS:LRIT1:DMBT1:ENO2:EMP1:LGR5:LCP1:DLGAP5:SIX6:CORO1A:CHAF1A:CD79A:PAFAH1B3:CD33:RND3:IGFBP2:PCNA:TGM2:GTSE1:TGFBR2:RBP1:CXCL1:C5orf30:ADAM19:IER3:CDKN1A:CALD1
Cancer_modules	MODULE_324	133	13	0.0015656158220169018	0.012826154801249965	CRB1:C1S:CALB2:RCVRN:MYL12A:FBLN1:SCO2:ADPRH:CDH6:TNFAIP3:THBS2:CA2:CDH17
Cancer_modules	MODULE_334	166	15	0.0015772301727755176	0.012826154801249965	TRIM33:GRK5:PTHLH:PSEN1:ARPP19:IFI35:DNAJB1:FAM49A:SNED1:SYNGR1:SRPRB:RPS18:PILRB:PRKAR2B:ESYT2
Cancer_modules	MODULE_432	15	4	0.0019512189121662113	0.015573617613771058	HMGCS2:FDPS:LSS:CNBP
Cancer_modules	MODULE_114	336	24	0.0022049440469015104	0.01727874334935547	FDPS:SNRPE:FAU:UCP2:HSPA8:TPI1:PHB2:RPL19:RPL38:MYL12B:TPRKB:RPE:NCL:TMEM230:PCNA:SEMG2:TPD52L2:RPL32:RPL14:HLA-A:HSPB1:YWHAG:RPL7:RPL12
Cancer_modules	MODULE_99	356	25	0.0022703512164610916	0.01747359596954876	FOXD2:RPE65:KCNC4:SLC16A4:AMPD1:F5:APBB1:BBOX1:SCGB2A1:TRPC6:KCNA1:GTF2A1:RPL3L:CD2BP2:EPN2:DGKE:FSCN2:IL12RB1:KCNS1:SEMG1:PPARA:AFM:THBS2:ASL:KCNV2
Cancer_modules	MODULE_96	37	6	0.0023964541869204103	0.01812055709759117	LPHN2:APCS:SELP:CLEC10A:ASGR2:FCN2
Cancer_modules	MODULE_345	124	12	0.0025358079061885697	0.0185242916536826	FCRLA:UBE2L6:KIAA0226L:KCTD12:IFI35:CD79A:POU2F2:FOXP1:NEK11:ADAM19:SLC22A23:HLA-A
Cancer_modules	MODULE_289	124	12	0.0025358079061885697	0.0185242916536826	LPHN2:RGR:GRK5:PTPN6:CCL17:PTGER1:OR10H3:FPR2:IL8:CXCL1:NPY1R:HCRTR2
Cancer_modules	MODULE_23	562	35	0.002736933189888369	0.01939524911987673	HMGCS2:ANXA9:SEMA6C:HCN3:APCS:SLC43A1:UBE2L6:SAC3D1:TM7SF2:PC:C1S:DAO:SDS:PCK2:CLEC10A:ASGR2:NFKBIB:IGFBP2:SLC11A1:TGM2:SLPI:APOL1:CYP2D6:SCO2:ARSA:HYAL1:RBP1:LECT2:FLOT1:GSTA2:ESR1:CLDN3:ST6GALNAC4:FCN2:PAEP
Cancer_modules	MODULE_63	211	17	0.002745035258265616	0.01939524911987673	TIE1:KCNC4:GJA5:FCGR2A:FCGR3A:KCNJ11:KCNA1:GPRC5A:CHRNB4:ASGR2:CACNG1:KCNJ2:MERTK:HYAL2:PDGFRB:ITGB8:GFRA2
Cancer_modules	MODULE_330	27	5	0.0030254364526428636	0.021031663082081844	GJA5:KCNA1:CACNG1:DSTN:CALD1
Cancer_modules	MODULE_346	17	4	0.003203992568515157	0.021919377730635438	PSAP:SMPD1:ARSA:NSMAF
Cancer_modules	MODULE_169	96	10	0.003308412214872295	0.022280088509530612	TNFAIP8L2:ULK1:HEATR5A:ATP2C1:KLHL6:STIM2:ZNF608:PCDHGC3:IER3:PRKAR2B
Cancer_modules	MODULE_248	129	12	0.0035143058202303954	0.02330255090029693	RPE65:ARHGEF11:ABL2:PSEN2:PTPN6:RCVRN:DGKE:FSCN2:MERTK:IL8:CXCL1:PTPN3
Cancer_modules	MODULE_104	349	24	0.0035792255778440323	0.023373427637133	SZT2:LRIT1:DMBT1:ZNF143:ABCC8:RCE1:AKAP3:HOXC11:SIX6:CHRNB4:CALB2:RCVRN:DNAH9:SSTR2:PDE4A:C19orf57:CD33:PI3:SLPI:ARFGAP3:GTSE1:C5orf30:PRSS16:FCN2
Cancer_modules	MODULE_129	218	17	0.0038322232875210274	0.024652063237635264	TSPAN1:SLC16A4:GRK5:HPX:BBOX1:KCNA1:PCDH9:CCL17:CEACAM1:KCNS1:SEMG1:CELSR1:MAPK8IP2:THBS2:TPST1:GNG11:KHDRBS3
Cancer_modules	MODULE_427	115	11	0.004113949295625721	0.026075178623745383	PTHLH:PSEN1:TRPM1:IFI35:DNAJB1:FAM49A:SNED1:SYNGR1:SRPRB:CFI:PILRB

Cancer_modules	MODULE_98	393	26	0.0041760781274914624	0.026085357578968408	FOXD2:SETDB1:CLK2:SNRPE:IPO7:WEE1:ATN1:EMG1:POLE:FOXG1:TCF12:ANP32A:KPNB1:PSMD12:CHAF1A:SAFB:MYCN:COPS8:PCNA:CCNO:CDKN1A:MAP7:HOXA13:ZNF117:RFC2:KHDRBS3
Cancer_modules	MODULE_32	239	18	0.004298775592947615	0.026468175436577458	RPL22:RIT1:SNRPE:RAP1B:RPL19:RPL38:CHAF1A:RBMS1:NCL:EIF6:RPL32:GNAI2:RPL37:RNASET2:RPL7:GEM:RPL12:TUBB4B
Cancer_modules	MODULE_151	316	22	0.004448414861417988	0.02700375782072046	FDPS:SNRPE:UCP2:HSPA8:TPI1:PHB2:RPL19:RPL38:MYL12B:TPRKB:RPE:NCL:TMEM230:PCNA:SEMG2:TPD52L2:RPL32:RPL14:HLA-A:YWHAG:RPL7:RPL12
Cancer_modules	MODULE_53	397	26	0.004759113447244982	0.02848858188559149	CDC20:FDPS:LRIT1:DMBT1:WEE1:UBE2L6:SAC3D1:ENO2:LGR5:LCP1:PCDH9:DLGAP5:SIX6:CORO1A:SSTR2:CHAF1A:CD79A:PAFAH1B3:CD33:IGFBP2:PCNA:GTSE1:C5orf30:ADAM19:IER3:ST6GALNAC4
Cancer_modules	MODULE_436	135	12	0.005067906972149549	0.029921478150636376	FCRLA:UBE2L6:KIAA0226L:KCTD12:IFI35:CD79A:POU2F2:FOXP1:NEK11:ADAM19:SLC22A23:HLA-A
Cancer_modules	MODULE_2	380	25	0.0052552564296603	0.030441004874890445	C1orf63:SYT11:HSPA6:CSRP1:UBE2L6:ENO2:C1S:EMP1:ULK1:PCDH9:MOAP1:MFAP4:MBP:TGM2:SLPI:SYNGR1:FBLN1:TGFBR2:PDGFRB:IER3:CDKN1A:TNFAIP3:THBS2:GNG11:GEM
Cancer_modules	MODULE_206	153	13	0.0052971586209206115	0.030441004874890445	SYT11:CUL2:LINC01059:GRAMD1B:DDX25:KDELC1:COCH:ANKRD11:PLEKHM1:NCL:KLHL6:CCNO:FAM167A:ENY2
Cancer_modules	MODULE_199	57	7	0.005378504134975533	0.030501780028611244	TIE1:MPL:F3:GRK5:MERTK:PDGFRB:CDKN1A
Cancer_modules	MODULE_207	226	17	0.0054856282811564915	0.03070526998933049	MED8:MSTO1:BBOX1:AKAP3:FOXG1:TRPM1:AQR:MGA:SLC7A6OS:PRPSAP2:ST8SIA5:ZNF83:SLC4A5:IFT57:MAP3K13:DUS4L:GIMAP5
Cancer_modules	MODULE_52	425	27	0.00606513566858627	0.033513762476419004	CDC20:SEMA6C:FDPS:WEE1:EMP1:LGR5:DLGAP5:PPP4R1:CHAF1A:RND3:IGFBP2:PCNA:TGM2:GTSE1:TGFBR2:RBP1:IL8:CXCL1:OCLN:C5orf30:IER3:CDKN1A:THBS2:GNG11:MEST:CALD1:ST6GALNAC4
Cancer_modules	MODULE_332	45	6	0.0065112213312429135	0.035523245490705006	GTF3A:UCHL3:IPO5:ERCC5:TXNDC16:PUM2
Cancer_modules	MODULE_279	141	12	0.007125077204284036	0.03819489005047304	TRIM33:PTHLH:PSEN1:IFI35:FAM49A:SNED1:SYNGR1:SRPRB:RPS18:PILRB:PRKAR2B:ESYT2
Cancer_modules	MODULE_259	46	6	0.007254577786021945	0.03819489005047304	TIE1:F3:GRK5:MERTK:PDGFRB:CDKN1A
Cancer_modules	MODULE_238	159	13	0.007266777225380021	0.03819489005047304	CSMD2:TMEM79:FCRLA:SELP:NAALADL1:PTPN6:KIAA0226L:COQ9:FOXP1:NEK11:PLAC8:BLK:DIRAS2
Cancer_modules	MODULE_419	33	5	0.007386228047446154	0.0383549914270999	MACF1:CORO1A:DSTN:CALD1:PIP
Cancer_modules	MODULE_389	216	16	0.007970892928449838	0.04089827204954619	MED8:SYT11:BBOX1:LINC01059:GRAMD1B:AKAP3:FOXG1:TRPM1:AQR:SLC7A6OS:ANKRD11:PRPSAP2:ZNF83:SLC4A5:IFT57:DUS4L:FAM167A
Cancer_modules	MODULE_75	397	25	0.0089364756839241	0.045313188467897496	F3:CTSS:FCGR2A:FCGR3A:SELP:FAS:C1S:EMP1:CCL17:CD79A:CEACAM1:IL18R1:IL8:CXCL6:CXCL1:AREG:AREGB:CFI:NPY1R:PTGER4:EDN1:HLA-A:CDKN1A:GEM:LY6E:FCN2
Cancer_modules	MODULE_139	79	8	0.009675217439121073	0.04819264953777657	VAV3:ANXA9:UCP2:UBXN6:BCAM:ISY1:OCLN:CLDN4
Cancer_modules	MODULE_456	112	10	0.009727982621314529	0.04819264953777657	NRAS:CREM:PCNA:ATP2C1:KLHL6:PTGER4:ZNF608:CDKN1A:TNFAIP3:PRKAR2B
Positional_gene_sets	chr1q22	66	27	1.5876801504268735e-21	4.747163649776352e-19	FAM189B:SCAMP3:CLK2:HCN3:PKLR:FDPS:RUSC1:ASH1L:MSTO1:YY1AP1:DAP3:GON4L:SYT11:RIT1:KIAA0907:ARHGEF2:SSR2:UBQLN4:LMNA:SEMA4A:PMF1-BGLAP:PMF1:BGLAP:PAQR6:SMG5:TMEM79:C1orf85
Positional_gene_sets	chr22q13	270	38	2.960406961908785e-12	4.4258084080536337e-10	APOBEC3B:SYNGR1:TAB1:MCHR1:WBP2NL:NAGA:FAM109B:SMDT1:NDUFA6:CYP2D6:CYB5R3:ATP5L2:ARFGAP3:KIAA1644:FBLN1:PPARA:CDPF1:PKDREJ:TTC38:GTSE1:TRMU:CELSR1:PLXNB2:DENND6B:MIOX:LMF2:NCAPH2:SCO2:TYMP:ODF3B:KLHDC7B:SYCE3:CPT1B:CHKB-CPT1B:CHKB:MAPK8IP2:ARSA:SHANK3

Positional_gene_sets	chr1q21	317	29	1.1259998675926317e-05	0.0011222465347006563	GJA5:GOLPH3L:HORMAD1:CTSS:CTSK:ARNT:SETDB1:CERS2:ANXA9:FAM63A:PRUNE:SEMA6C:TNFAIP8L2:SCNM1:LYSMD1:TMOD4:HRNR:FLG:KPRP:LCE1F:LCE1E:LCE1D:LCE1C:LCE1B:SMCP:CKS1B:DCST2:DCST1:ADAM15:CKS1B
Positional_gene_sets	chr3q22	103	14	3.213157751882681e-05	0.002401835419532304	ATP2C1:ASTE1:NEK11:ACAD11:NPHP3:ACKR4:UBA5:SRPRB:RAB6B:DZIP1L:A4GNT:DBR1:ARMC8:MRAS
Positional_gene_sets	chr14q12	86	12	8.944695725315367e-05	0.005348928043738589	NRL:DCAF11:FITM1:PSME1:STXBP6:FOXG1:C14orf23:G2E3:SCFD1:COCH:HEATR5A:DTD2
Positional_gene_sets	chr9q34	285	23	0.0005389703497416623	0.026858689095459506	RP11-203J24.9:ST6GALNAC6:ST6GALNAC4:PIP5KL1:DPM2:FAM102A:NAIF1:SLC25A25:NTMT1:C9orf50:GTF3C5:CEL:FCN2:OBP2A:PAEP:UBAC1:NACC2:RNF224:SLC34A3:TUBB4B:FAM166A:C9orf173:NELFB:TOR4A
Positional_gene_sets	chr14q21	68	9	0.000983425389140527	0.042006313050431086	MIA2:CTAGE5:FBXO33:LRFN5:C14orf28:KLHL28:FAM179B:PRPF39:FKBP3:FANCM
BioCarta	BIOCARTA_PTDINS_PATHWAY	23	6	0.00016303418301790638	0.047116878892174946	ARHGEF2:RPS6KB1:GSK3A:GSK3B:AP2M1:LYN
BioCarta	BIOCARTA_CARM_ER_PATHWAY	26	6	0.00033758246204784015	0.0487806657659129	SPEN:PHB2:GTF2A1:BRCA1:HDAC3:ESR1
Wikipathways	Vitamin D Receptor Pathway	184	20	2.230384445721354e-05	0.012110987540266951	LCE1F:LCE1D:BGLAP:KRTAP5-1:KRTAP5-4:PTHLH:CD97:CEACAM1:IL1RL1:IGFBP5:KRTAP8-1:CYP2D6:TRAK1:SLC34A2:PTGER4:CDKN1A:TNFAIP3:DACT2:ABCB1:KLF4
Wikipathways	MAPK Signaling Pathway	249	23	7.557952826237514e-05	0.018144614827210116	CDC42:NRAS:HSPA6:FAS:DUSP8:HSPA8:RAP1B:PLA2G4B:NFATC3:MAPK7:CACNB1:CACNG1:TAB1:MAPK8IP2:TGFBR2:CACNA2D2:MRAS:RASA2:MAP3K13:FGF12:PDGFRB:HSPB1:PPP3CC
Wikipathways	Cytoplasmic Ribosomal Proteins	87	12	0.00010024649075806694	0.018144614827210116	RPL22:FAU:RPL3L:RPL19:RPS6KB1:RPL38:RPL32:RPL14:RPL37:RPS18:RPL7:RPL12
GO_mf	GO_RIBONUCLEOTIDE_BINDING	1885	138	5.0104523780794994e-14	8.242194161940776e-11	ACOT7:MTOR:CDC42:RIMKLA:TIE1:KTI12:ACOT11:ABCD3:HIPK1:NRAS:CLK2:HCN3:PKLR:DAP3:RIT1:HSPA6:UHMK1:TOR3A:ABL2:SOAT1:ADCK3:TUBB8:ARL5B:RAB18:ACTA2:ALDH18A1:GRK5:WEE1:KCNJ11:ABCC8:ARL2:PC:HSPA8:DDX25:DYRK4:DDX47:RAP1B:APAF1:NUAK1:CIT:DHX37:ULK1:P2RX2:ARL11:PCK2:FANCM:MAP3K9:TDRD9:AQR:MYO5A:RASL12:TARSL2:SMG1:SEPT1:SEPT1:ABCC11:LONP2:SPG7:SHPK:TRPV1:SHPK:DHX33:DNAH9:MAPK7:RNF112:RND2:DGKE:TUBD1:RPS6KB1:HELZ:KIF19:TUBB4A:INSR:PDE4A:DDX39A:PKN1:MAST3:SARS2:GSK3A:ALK:ATP6V1B1:RTKN:MERTK:RND3:TTLL4:PANK2:TGM2:DDX27:UCKL1:RTCB:CYB5R3:TRMU:CHKB:TGFBR2:ENTPD3:GNAI2:NAT6:ARL13B:GTPBP8:GSK3B:RAB43:ATP2C1:NEK11:UBA5:SRPRB:RAB6B:MRAS:GK5:ABCF3:MAP3K13:LSG1:STK32B:ATP10D:UBE2QL1:DHX29:SKIV2L2:ACTBL2:KIF2A:PDGFRB:THG1L:WRNIP1:TUBB:STK19:RAB44:POPDC3:ARL4A:SEPT7:RFC2:ABCB1:PRKAR2B:GIMAP5:BLK:LYN:GEM:NRBP2:DIRAS2:IPPK:PIP5KL1:TUBB4B:TOR4A
GO_mf	GO_TRANSPORTER_ACTIVITY	1241	102	1.41012994068657e-13	1.1598318762147038e-10	RHCE:MFSD2A:LRRC8B:LRRC8C:ABCD3:KCNC4:SLC16A4:GJA5:HCN3:SLC16A12:HPX:IPO7:KCNJ11:ABCC8:SLC43A1:UCP2:UCP3:TRPC6:LINC01059:GRAMD1B:KCNA1:SLC11A2:SLC25A3:ORAI1:P2RX2:MTMR6:SLC25A15:KPNA3:IPO5:MIA2:CTAGE5:PSEN1:KCNK10:NIPA2:NIPA1:TRPM1:SLC51B:CHRNB4:PKD1:GRIN2A:ABCC11:SLC7A6:TRPV1:SLC5A10:SLC47A1:SLC47A2:CACNB1:GJC1:KPNB1:CACNG1:PITPNC1:KCNJ16:KCNJ2:TTYH2:RALBP1:SLC14A1:CD320:CEACAM1:GRIN2D:KCNJ14:SLC6A16:XPO1:ATP6V1B1:SLC4A5:SLC9A4:SCN3A:SLC11A1:KCNS1:SLC37A1:OSBP2:APOL1:ATP5L2:ARFGAP3:PKDREJ:SCN5A:CACNA2D2:SLC25A26:ATP2C1:PLSCR4:KCNAB1:AP2M1:SLC34A2:STIM2:ATP10D:SLC10A4:SLC4A4:MTTP:SLC6A19:SLC6A18:IPO11:SLC12A2:PRELID2:KCNIP1:SLC22A23:TAPBP:CNR1:CLDN4:ABCB1:STAR:CDH17:KCNV2:SLC35D2:SLC25A25:SLC34A3
GO_mf	GO_IDENTICAL_PROTEIN_BINDING	1706	124	1.760541995911437e-12	9.65363861091438e-10	ACOT7:TARDBP:MTOR:NBL1:CDC42:PHC2:STIL:HOOK1:BCL10:ABCD3:ARNT:ANXA9:CLK2:SYT11:UBQLN4:TMEM79:APCS:CFHR5:MCM10:C10orf54:PSAP:FAS:ALDH18A1:BBOX1:PC:DGAT2:TENM4:TRPC6:UBASH3B:TPI1:C1S:APAF1:UHRF1BP1L:SDS:SDSL:ORAI1:ULK1:P2RX2:LCP1:KCTD12:ERCC5:MAP3K9:MYO5A:TCF12:MESDC2:SH3GL3:CORO1A:SEPT1:SEPT1:DCTPP1:SIAH1:COQ9:TRPV1:RABEP1:HNF1B:IKZF3:BRCA1:RPS6KB1:KCNJ2:AZI1:IMPA2:TCF3:SH3GL1:CHAF1A:SAFB2:KEAP1:DDX39A:GIPC1:CD79A:PAFAH1B3:CEACAM1:CARD8:ALK:CREB1:RPE:SLC11A1:VIL1:MFF:NCL:PCNA:CHMP4B:NAGA:FAM109B:FBLN1:TYMP:RAD18:TMEM115:CCDC66:FOXP1:CGGBP1:MAP3K13:KLHL2:DCTD:TERT:CDH9:CDH6:DROSHA:MCIDAS:LECT2:THG1L:W

						RNIP1:MDC1:PEX7:TNFAIP3:ESR1:SYNE1:SOD2:FGFR1OP:SEPT7:ASL:TPST1:CLDN3:CLDN4:HSPB1:YWHAG:ESYT2:TERF1:RPL7:CDH17:KHDRBS3:PUF60:IZUMO3:UBQLN1:XPA:NACC2
GO_mf	GO_TRANSMEMBRANE_TRANSPORTER_ACTIVITY	1038	85	1.9991887912100587e-11	8.221663903851366e-09	RHCE:MFSD2A:LRRC8B:LRRC8C:ABCD3:KCNC4:SLC16A4:GJA5:HCN3:SLC16A12:HPX:KCNJ11:ABCC8:SLC43A1:UCP2:UCP3:TRPC6:KCNA1:SLC11A2:SLC25A3:ORAI1:P2RX2:MTMR6:SLC25A15:PSEN1:KCNK10:NIPA2:NIPA1:TRPM1:SLC51B:CHRNB4:PKD1:GRIN2A:ABCC11:SLC7A6:TRPV1:SLC5A10:SLC47A1:SLC47A2:CACNB1:GJC1:CACNG1:KCNJ16:KCNJ2:TTYH2:RALBP1:SLC14A1:CD320:CEACAM1:GRIN2D:KCNJ14:SLC6A16:ATP6V1B1:SLC4A5:SLC9A4:SCN3A:SLC11A1:KCNS1:SLC37A1:APOL1:ATP5L2:PKDREJ:SCN5A:CACNA2D2:SLC25A26:ATP2C1:KCNAB1:SLC34A2:STIM2:SLC10A4:SLC4A4:SLC6A19:SLC6A18:SLC12A2:KCNIP1:SLC22A23:TAPBP:CNR1:CLDN4:ABCB1:CDH17:KCNV2:SLC35D2:SLC25A25:SLC34A3
GO_mf	GO_RNA_BINDING	1875	127	1.0156824665669745e-10	3.341595315005346e-08	RPL22:TARDBP:EXOSC10:SPEN:MACF1:EBNA1BP2:RPF1:C1orf52:PTBP2:RBM15:CSDE1:FDPS:DAP3:KIAA0907:SMG5:UHMK1:CSRP1:SNRPE:NUCKS1:LARP4B:NSUN6:ARL5B-AS1:ALDH18A1:RBM20:TRUB1:FAU:RBM7:HSPA8:DDX25:EMG1:DDX47:DHX37:GTF3A:MTIF3:FNDC3A:KCTD12:IPO5:DTD2:PRPF39:FKBP3:RBM25:CPSF2:TDRD9:AQR:MYO5A:ANP32A:RPL3L:SMG1:CORO1A:ESRP2:PABPN1L:METTL16:DHX33:RPL19:BRCA1:KPNB1:UTP18:TRIM25:HELZ:RPL38:TNRC6C:SAFB2:SAFB:DDX39A:SARS2:MRPS12:GRWD1:CTU1:NOL10:PUM2:XPO1:ZNF638:DUSP11:RBMS1:NCL:ESF1:EIF6:SLPI:DDX27:ZNFX1:ZBP1:TPD52L2:RTCB:APOBEC3B:TRMU:THUMPD3:RPL32:DAZL:EIF1B:RPL14:FAM208A:DHFRL1:ISY1:CNBP:DBR1:XRN1:FNDC3B:EIF4E:TRMT10A:TERT:DROSHA:RPL37:DHX29:SKIV2L2:DIMT1:UTP15:DIAPH1:THG1L:LSM11:CPEB4:EIF4E1B:HLA-A:RPS18:WDR46:SYNE1:RNASET2:SRRM3:HSPB1:YWHAG:MEPCE:RPL7:KHDRBS3:PUF60:KIAA0020:RBM18:RPL12:TUBB4B:NELFB
GO_mf	GO_TRANSITION_METAL_ION_BINDING	1072	84	2.337141807100393e-10	6.407663787800245e-08	RNF207:TRIM62:PHC2:C1orf123:EXTL2:TRIM33:SETDB1:HRNR:FLG:ARHGEF2:F5:ABL2:CSRP1:ZMYND11:TLL2:RBM20:SMPD1:BBOX1:GYLTL1B:ZFPL1:TRIM77:FDX1:USP5:SLC11A2:POLE:WBP4:PCK2:ZFYVE1:UBR7:HERC2:OTUD7A:GRIN2A:SIAH1:SNAI3:SPG7:RNF112:VAT1:BRCA1:NBR1:NPEPPS:KPNB1:MBTD1:TRIM25:MPPE1:CBLC:ZNF638:DYTN:MMP24:SEMG1:SEMG2:ZNFX1:APOBEC3B:CYP2D6:PPARA:MIOX:SCO2:SHANK3:MYRIP:CYP8B1:RASSF1:NR1I2:CNBP:MFI2:TRIM60:DCTD:MARCH3:SNCB:MGAT1:ZFAND3:L3MBTL3:TNFAIP3:ESR1:SOD2:ZNF117:ZNF92:ZCWPW1:CPA1:ZMAT4:CA2:CALB1:CYP11B1:CYP11B2:KLF4:PAPPA
GO_mf	GO_METAL_ION_TRANSMEMBRANE_TRANSPORTER_ACTIVITY	438	46	4.6196419756899933e-10	1.0856158642871484e-07	KCNC4:HCN3:KCNJ11:ABCC8:TRPC6:KCNA1:SLC11A2:ORAI1:MTMR6:PSEN1:KCNK10:NIPA2:NIPA1:TRPM1:PKD1:GRIN2A:TRPV1:SLC5A10:CACNB1:CACNG1:KCNJ16:KCNJ2:GRIN2D:KCNJ14:SLC6A16:SLC4A5:SLC9A4:SCN3A:SLC11A1:KCNS1:PKDREJ:SCN5A:CACNA2D2:ATP2C1:KCNAB1:SLC34A2:STIM2:SLC10A4:SLC4A4:SLC6A19:SLC6A18:SLC12A2:KCNIP1:CNR1:KCNV2:SLC34A3
GO_mf	GO_DNA_BINDING_TRANSCRIPTION_FACTOR_ACTIVITY	1691	113	2.5211359520599554e-09	4.6986789051901354e-07	HES2:TARDBP:ZBTB17:ZNF362:TAL1:FOXE3:FOXD2:ARNT:ASH1L:GON4L:C1orf85:ETV3L:NHLH1:ZBTB41:NUCKS1:ZNF496:CREM:ZNF143:DBX1:EHF:PRKRIR:SOX5:CSRNP2:TFCP2:HOXC13:HOXC12:HOXC11:GTF3A:NRL:FOXG1:SIX6:ZNF770:MGA:RFX7:TCF12:SKOR1:BOLA2B:ZNF48:ZNF771:ZNF720:NFATC3:ZNF469:SNAI3:HNF1B:IKZF3:TBX21:TAF4B:SKOR2:ZNF236:TCF3:ZNF442:CC2D1A:RFX1:ZNF737:POU2F2:ZNF526:ERF:CIC:ZNF766:ZNF480:ZNF534:ZNF83:ZNF611:ZNF765:ZSCAN1:ZNF135:MYCN:OSR1:NCOA1:BCL11A:BOLA3:AFF3:CREB1:HES6:ZNF343:ZNFX1:SLC2A4RG:PPARA:ZNF860:ZNF619:ZNF620:ZNF662:FOXP1:CGGBP1:ZNF654:NR1I2:CNBP:ISL1:CDX1:SOX30:ZFP62:ZNF311:ESR1:HOXA10:HOXA11:HOXA13:EVX1:JAZF1:NEUROD6:ZNF716:ZNF117:ZNF92:TSC22D4:KLF14:CREB3L2:ZNF467:TERF1:POU5F1B:ZNF34:FOXB2:KLF4:PBX3:ZNF79
GO_mf	GO_ION_TRANSMEMBRANE_TRANSPORTER_ACTIVITY	870	70	2.5707057839946028e-09	4.6986789051901354e-07	RHCE:MFSD2A:LRRC8B:LRRC8C:KCNC4:SLC16A4:HCN3:SLC16A12:KCNJ11:ABCC8:SLC43A1:TRPC6:KCNA1:SLC11A2:SLC25A3:ORAI1:P2RX2:MTMR6:SLC25A15:PSEN1:KCNK10:NIPA2:NIPA1:TRPM1:SLC51B:CHRNB4:PKD1:GRIN2A:ABCC11:SLC7A6:TRPV1:SLC5A10:CACNB1:GJC1:CACNG1:KCNJ16:KCNJ2:TTYH2:CEACAM1:GRIN2D:KCNJ14:SLC6A16:ATP6V1B1:SLC4A5:SLC9A4:SCN3A:SLC11A1:KCNS1:SLC37A1:APOL1:ATP5L2:PKDREJ:SCN5A:CACNA2D2:ATP2C1:KCNAB1:SLC34A2:STIM2:SLC10A4:SLC4A4:SLC6A19:SLC6A18:SLC12A2:KCNIP1:CNR1:CLDN4:KCNV2:SLC35D2:SLC25A25:SLC34A3
GO_mf	GO_CALCIUM_ION_BINDING	693	59	6.139693556414294e-09	1.0099795900301513e-06	CLSTN1:MACF1:ANXA9:HRNR:FLG:BGLAP:APCS:SELP:CRB1:NID1:CDH23:TLL2:HABP2:SYT13:C1S:LCP1:PCDH9:PLA2G4B:MYO5A:MYLPF:CALB2:RCVRN:MYL12A:MYL12B:TUBB4A:CD320:CD97:EPS15L1:MEGF8:CBLC:TPO:HPCAL1:VIL1:SNED1:FBLN1:PKDREJ:CELSR1:ARSA:EFHB:PROS1:PLSCR4:STIM2:PLA2G12A:CDH9:CDH6:CETN3:MCTP1:PCDHGA12:PCDHGC3:PCDHGC4:PCDHGC5:KCNIP1:SNCB:RAB44:THBS2:ESYT2:CALB1:CDH17:SLC25A25

GO_mf	GO_ZINC_ION_BINDING	806	64	2.0411389293789333e-08	2.948394402623213e-06	RNF207:TRIM62:PHC2:C1orf123:TRIM33:SETDB1:ARHGEF2:CSRP1:ZMYND11:TLL2:RBM20:SMPD1:BBOX1:ZFPL1:TRIM77:USP5:SLC11A2:POLE:WBP4:ZFYVE1:UBR7:HERC2:OTUD7A:GRIN2A:SIAH1:SPG7:RNF112:VAT1:BRCA1:NBR1:NPEPPS:KPNB1:MBTD1:TRIM25:CBLC:ZNF638:DYTN:MMP24:SEMG1:SEMG2:ZNFX1:APOBEC3B:PPARA:SHANK3:MYRIP:RASSF1:NR1I2:CNBP:TRIM60:DCTD:MARCH3:ZFAND3:L3MBTL3:TNFAIP3:ESR1:ZNF117:ZNF92:ZCWPW1:CPA1:ZMAT4:CA2:CALB1:KLF4:PAPPA
GO_mf	GO_ADENYL_NUCLEOTIDE_BINDING	1536	102	2.150804427445505e-08	2.948394402623213e-06	ACOT7:MTOR:RIMKLA:TIE1:KTI12:ACOT11:ABCD3:HIPK1:CLK2:HCN3:PKLR:HSPA6:UHMK1:TOR3A:ABL2:SOAT1:ADCK3:ACTA2:ALDH18A1:GRK5:WEE1:KCNJ11:ABCC8:PC:HSPA8:DDX25:DYRK4:DDX47:APAF1:NUAK1:CIT:DHX37:ULK1:P2RX2:FANCM:MAP3K9:TDRD9:AQR:MYO5A:TARSL2:SMG1:ABCC11:LONP2:SPG7:SHPK:TRPV1:SHPK:DHX33:DNAH9:MAPK7:DGKE:RPS6KB1:HELZ:KIF19:INSR:PDE4A:DDX39A:PKN1:MAST3:SARS2:GSK3A:ALK:ATP6V1B1:MERTK:TTLL4:PANK2:DDX27:UCKL1:RTCB:CYB5R3:TRMU:CHKB:TGFBR2:ENTPD3:NAT6:GSK3B:ATP2C1:NEK11:UBA5:GK5:ABCF3:MAP3K13:STK32B:ATP10D:UBE2QL1:DHX29:SKIV2L2:ACTBL2:KIF2A:PDGFRB:THG1L:WRNIP1:STK19:POPDC3:RFC2:ABCB1:PRKAR2B:BLK:LYN:NRBP2:IPPK:PIP5KL1:TOR4A
GO_mf	GO_TRANSMEMBRANE_SIGNALING_RECEPTOR_ACTIVITY	1263	88	2.471785575841804e-08	3.127759440199821e-06	GPR153:HTR6:TIE1:MPL:TM2D1:LPHN2:F3:ANXA9:OR14K1:TACR2:RGR:FAS:OR52B4:OR51T1:ABCC8:OR4C15:OR4C16:OR4P4:OR4S2:OR4C6:OR5T1:OR8H1:OR8K3:OR8K1:OR8J1:OR8U1:OR5R1:PTPN6:GPRC5A:OR10P1:LGR5:P2RX2:TNFRSF19:MLNR:CHRNB4:PKD1:GRIN2A:TRPV1:GLP2R:B9D1:SSTR2:INSR:OR1M1:OR7G2:OR7G1:OR7G3:S1PR5:CD97:PTGER1:OR10H2:OR10H3:IL12RB1:CD79A:BCAM:GRIN2D:FPR2:ALK:FSHR:IL1RL1:IL18R1:IL18RAP:MERTK:MC3R:MCHR1:CELSR1:PLXNB2:TGFBR2:ACKR2:CD200R1:ACKR4:NPY1R:NPY5R:PTGER4:PDGFRB:OR10C1:HCRTR2:CNR1:CRHR2:CLDN3:CLDN4:GPR22:TAS2R39:TAS2R40:GFRA2:S1PR3:OR1L3:OR1L4:OR1L6
GO_mf	GO_MOLECULAR_TRANSDUCER_ACTIVITY	1529	101	3.2816197731410436e-08	3.8559032334407265e-06	GPR153:HTR6:TIE1:MPL:TM2D1:LPHN2:F3:ARNT:ANXA9:PAQR6:PEAR1:OR14K1:TACR2:RGR:FAS:DMBT1:OR52B4:OR51T1:ABCC8:OR4C15:OR4C16:OR4P4:OR4S2:OR4C6:OR5T1:OR8H1:OR8K3:OR8K1:OR8J1:OR8U1:OR5R1:PTPN6:GPRC5A:OR10P1:LGR5:PRKAB1:P2RX2:TNFRSF19:MLNR:FKBP3:CHRNB4:PKD1:GRIN2A:TRPV1:GLP2R:B9D1:SSTR2:INSR:OR1M1:OR7G2:OR7G1:OR7G3:S1PR5:CD97:PTGER1:OR10H2:OR10H3:IL12RB1:CD79A:BCAM:GRIN2D:CD33:FPR2:ALK:FSHR:IL1RL1:IL18R1:IL18RAP:MERTK:MC3R:MCHR1:PPARA:CELSR1:PLXNB2:TGFBR2:ACKR2:CD200R1:NR1I2:ACKR4:NPY1R:NPY5R:PTGER4:PDGFRB:OR10C1:HCRTR2:CNR1:ESR1:ITGB8:CRHR2:CLDN3:CLDN4:CD36:GPR22:TAS2R39:TAS2R40:GFRA2:S1PR3:OR1L3:OR1L4:OR1L6:CEL
GO_mf	GO_PROTEIN_DOMAIN_SPECIFIC_BINDING	698	57	4.699326430227365e-08	5.153594651816011e-06	MTOR:CDC42:SSX2IP:BCL10:GJA5:ADAM15:FAM189B:PMF1:CTR9:HSPA8:ERC1:KCNA1:ATN1:PTPN6:CIT:NRL:PSEN1:MYO5A:TCF12:PKD1:RABEP1:KPNB1:RPS6KB1:SSTR2:SH3GL1:CHAF1A:INSR:KEAP1:GIPC1:POU2F2:CBLC:CARD8:XPO1:TGM2:WBP2NL:PPARA:SHANK3:SCN5A:TRAK1:PLSCR4:KCNAB1:NIPBL:OCLN:NDFIP1:ADAM19:MDC1:TUBB:PPIL1:LAT2:YWHAG:PRKAR2B:EXOC4:LYN:TERF1:KHDRBS3:CNTLN:XPA
GO_mf	GO_CATION_TRANSMEMBRANE_TRANSPORTER_ACTIVITY	637	53	7.618267711170904e-08	7.832531490547586e-06	RHCE:KCNC4:HCN3:KCNJ11:ABCC8:TRPC6:KCNA1:SLC11A2:SLC25A3:ORAI1:P2RX2:MTMR6:SLC25A15:PSEN1:KCNK10:NIPA2:NIPA1:TRPM1:CHRNB4:PKD1:GRIN2A:TRPV1:SLC5A10:CACNB1:CACNG1:KCNJ16:KCNJ2:GRIN2D:KCNJ14:SLC6A16:ATP6V1B1:SLC4A5:SLC9A4:SCN3A:SLC11A1:KCNS1:ATP5L2:PKDREJ:SCN5A:CACNA2D2:ATP2C1:KCNAB1:SLC34A2:STIM2:SLC10A4:SLC4A4:SLC6A19:SLC6A18:SLC12A2:KCNIP1:CNR1:KCNV2:SLC34A3
GO_mf	GO_GUANYL_NUCLEOTIDE_BINDING	392	38	1.2215654165060254e-07	1.1546406874334293e-05	CDC42:NRAS:DAP3:RIT1:TUBB8:ARL5B:RAB18:ARL2:RAP1B:ARL11:PCK2:RASL12:SEPT1:SEPT1:RNF112:RND2:TUBD1:TUBB4A:INSR:RTKN:RND3:TGM2:GNAI2:ARL13B:GTPBP8:RAB43:SRPRB:RAB6B:MRAS:LSG1:THG1L:TUBB:RAB44:ARL4A:SEPT7:GIMAP5:GEM:DIRAS2:TUBB4B
GO_mf	GO_SEQUENCE_SPECIFIC_DNA_BINDING	1114	78	1.283668790176512e-07	1.1546406874334293e-05	HES2:TARDBP:MTOR:ZBTB17:MED8:TAL1:FOXE3:FOXD2:WDR77:ARNT:SMG5:ETV3L:NHLH1:MCM10:CREM:ZNF143:DBX1:EHF:YAP1:POU2AF1:CSRNP2:TFCP2:HOXC13:HOXC12:HOXC11:NRL:FOXG1:SIX6:RFX7:TCF12:RRN3:SMG1:NFATC3:SNAI3:DHX33:HNF1B:IKZF3:TBX21:TCF3:SAFB2:SAFB:CC2D1A:RFX1:POU2F2:ERF:GRWD1:MYCN:OSR1:BCL11A:CREB1:NCL:HES6:PER2:PPARA:FOXP1:CGGBP1:NR1I2:CNBP:TERT:ISL1:CDX1:SOX30:ESR1:GTF2H5:HOXA10:HOXA11:HOXA13:EVX1:NEUROD6:KLF14:CREB3L2:TERF1:POU5F1B:FOXB2:KLF4:PBX3:GTF3C5:NACC2
GO_mf	GO_DRUG_BINDING	1718	108	1.333627541716423e-07	1.1546406874334293e-05	MTOR:RIMKLA:TIE1:KTI12:ABCD3:OVGP1:HIPK1:CLK2:PKLR:HSPA6:UHMK1:TOR3A:ABL2:ADCK3:ACTA2:ALDH18A1:GRK5:WEE1:KCNJ11:ABCC8:OR5T1:PC:HSPA8:DDX25:DYRK4:DDX47:APAF1:NUAK1:SDS:SDSL:CIT:DHX37:ULK1:P2RX2:FKBP3:FANCM:MAP3K9:TDRD9:AQR:MYO5A:CHRNB4:TARSL2:PDXDC1:SMG1:ABCC11:LONP2:SPG7:SHPK:TRPV1:SHPK:DHX33:DNAH9:MAPK7:DGKE:RPS6KB1:HELZ:KIF19:INSR:CD320:DDX39A:PKN1:MAST3:SARS2:GSK3A:ALK:ATP6V1B1:MERTK:TTLL4:PANK2:DDX

						27:UCKL1:RTCB:CYP2D6:CYB5R3:PPARA:TRMU:CHKB:TGFBR2:ENTPD3:CYP8B1:NR1I2:GSK3B:ATP2C1:NEK11:UBA5:GK5:ABCF3:MAP3K13:STK32B:ATP10D:UBE2QL1:DHX29:SKIV2L2:ACTBL2:KIF2A:PDGFRB:THG1L:WRNIP1:STK19:PPIL1:CNR1:RFC2:ABCB1:BLK:LYN:NRBP2:IPPK:PIP5KL1:TOR4A
GO_mf	GO_HYDROLASE_ACTIVITY_ACTING_ON_ACID_ANHYDRIDES	921	67	2.563525906878496e-07	2.1085000584075627e-05	CDC42:MACF1:ABCD3:NRAS:PRUNE:RIT1:HSPA6:TOR3A:TUBB8:RAB18:ABCC8:ARL2:HSPA8:DDX25:DDX47:RAP1B:DHX37:FANCM:TDRD9:AQR:MYO5A:RASL12:SEPT1:SEPT1:DCTPP1:ABCC11:LONP2:DHX33:DNAH9:RNF112:RND2:TUBD1:HELZ:DNAI2:KIF19:RALBP1:TUBB4A:CD320:DDX39A:DCTN1:RND3:DDX27:ATP5L2:RAD18:ENTPD3:GNAI2:RAB43:ATP2C1:RAB6B:MRAS:ABCF3:LSG1:ATP10D:DHX29:SKIV2L2:KIF2A:WRNIP1:TUBB:TAPBP:RAB44:TCTE3:SEPT7:RFC2:ABCB1:GNG11:GEM:DIRAS2:TUBB4B
GO_mf	GO_CATION_CHANNEL_ACTIVITY	317	32	4.842857797620957e-07	3.79357194146975e-05	KCNC4:HCN3:KCNJ11:ABCC8:TRPC6:KCNA1:ORAI1:P2RX2:MTMR6:PSEN1:KCNK10:TRPM1:CHRNB4:PKD1:GRIN2A:TRPV1:CACNB1:CACNG1:KCNJ16:KCNJ2:GRIN2D:KCNJ14:SCN3A:KCNS1:PKDREJ:SCN5A:CACNA2D2:KCNAB1:STIM2:KCNIP1:CNR1:KCNV2
GO_mf	GO_AMIDE_BINDING	350	34	5.275836202746484e-07	3.9448866152354395e-05	ACOT7:CLSTN1:LDLRAP1:ACOT11:TM2D1:SOAT1:PSAP:APBB1:PC:PHB2:KPNA3:IPO5:KIAA1199:GRIN2A:GLP2R:NPEPPS:KPNB1:RPS6KB1:SSTR2:INSR:KDELR1:FPR2:MC3R:MCHR1:MAPK8IP2:NAT6:AP2M1:HLA-A:TAPBP:PPIL1:HCRTR2:PEX7:CRHR2:CD36
GO_mf	GO_PASSIVE_TRANSMEMBRANE_TRANSPORTER_ACTIVITY	452	40	6.294888983168507e-07	4.5022140770922585e-05	LRRC8B:LRRC8C:KCNC4:GJA5:HCN3:KCNJ11:ABCC8:TRPC6:KCNA1:ORAI1:P2RX2:MTMR6:PSEN1:KCNK10:TRPM1:CHRNB4:PKD1:GRIN2A:TRPV1:CACNB1:GJC1:CACNG1:KCNJ16:KCNJ2:TTYH2:SLC14A1:GRIN2D:KCNJ14:SCN3A:KCNS1:APOL1:PKDREJ:SCN5A:CACNA2D2:KCNAB1:STIM2:KCNIP1:CNR1:CLDN4:KCNV2
GO_mf	GO_PROTEIN_CONTAINING_COMPLEX_BINDING	1096	74	1.0583669979294356e-06	7.254223798308007e-05	MTOR:LDLRAP1:MACF1:CDC20:NRAS:CTSS:CTSK:TMOD4:ADAM15:APCS:FCGR2A:FCGR3A:UHMK1:ABL2:NID1:CUL2:APBB1:CDCA5:HSPA8:NDUFA9:LETMD1:NCKAP1L:RAP1B:SLC25A3:UHRF1BP1L:ULK1:MTIF3:LCP1:ERCC5:SCFD1:COCH:MYO5A:CORO1A:CD2BP2:PRMT7:DHX33:HNF1B:ADAM11:AZI1:FSCN2:INSR:NCOA1:ATP6V1B1:DCTN1:VIL1:PCNA:DSTN:EIF6:RPN2:APOL2:TAB1:FBLN1:PPARA:MAPK8IP2:RAD18:EIF1B:GNAI2:MAP3K13:NIPBL:DHX29:RELL2:CPEB4:LRRC16A:TUBB:TAPBP:CDKN1A:SYNE1:ITGB8:CD36:PIP:LYN:CDH17:S1PR3:NACC2
GO_mf	GO_KINASE_BINDING	722	54	1.6244924921879136e-06	0.00010689160598596471	MTOR:CDC42:BCL10:SIKE1:CKS1B:ACTA2:FAS:GRK5:PTPN6:PRKAB1:PKD1:MAPK7:RND2:NBR1:SPAG9:TCF3:PKN1:GSK3A:LIPE:CEACAM1:CBLC:TSKS:MYCN:PPP1CB:SPDYA:BCL11A:TPRKB:DCTN1:RND3:PCNA:LIME1:TAB1:MAPK8IP2:TGFBR2:SCN5A:HYAL2:GSK3B:MAP3K13:CCNO:CKS1B:ANKRA2:SLC12A2:PDGFRB:CDKN1A:TNFAIP3:ESR1:FGFR1OP:DACT2:HSPB1:YWHAG:SPDYE3:PRKAR2B:RB1CC1:CNTLN:UBQLN1
GO_mf	GO_HYDROLASE_ACTIVITY_ACTING_ON_ESTER_BONDS	723	54	1.6930596907979582e-06	0.0001071185842831785	ACOT7:EXOSC10:PTP4A2:ACOT11:RPE65:DNASE2B:PRUNE:DUSP8:SMPD1:REXO2:UBASH3B:PTPN6:PDE1B:POLE:MTMR6:RNASEH2B:ERCC5:ABHD13:DTD2:FANCM:DLGAP5:PLA2G4B:SLX1A:PLA2G15:SMPD3:PPP4R1:MPPE1:IMPA2:PHLPP1:PDE4A:PAFAH1B3:LIPE:PLB1:PPP1CB:DUSP11:CTDSP1:TAB1:ARSA:PLCXD2:ASTE1:DBR1:XRN1:LIPH:PLA2G12A:DROSHA:PPAP2A:RPP40:PTP4A1:RNASET2:PRKAR2B:PPP3CC:CA2:PTPN3:CEL
GO_mf	GO_LIPID_BINDING	726	54	1.915344789767616e-06	0.00011669415478398992	LDLRAP1:ACOT11:RPE65:F3:GOLPH3L:ANXA9:SYT11:PAQR6:SELP:SOAT1:PHLDA3:RBP3:PSAP:GRK5:SYT13:SNX15:LINC01059:GRAMD1B:STXBP6:ZFYVE1:PLA2G4B:SH3GL3:COQ9:PLA2G15:TRPV1:PIRT:EPN2:PITPNC1:KCNJ2:MPPE1:SH3GL1:DENND1C:PLEKHB2:VIL1:BPIFA2:OSBP2:BPIFC:APOL2:APOL1:PPARA:RBP2:RBP1:RASA2:AP2M1:MTTP:DROSHA:ARAP3:CPNE5:ESR1:CD36:ESYT2:STAR:LYN:CALB1:S1PR3
GO_mf	GO_TRANSCRIPTION_FACTOR_BINDING	639	48	5.397079033833952e-06	0.0003170783932377447	HES2:MTOR:SPEN:ZBTB17:C1orf64:TAL1:BCL10:ARNT:ARHGEF2:NUCKS1:CREM:APBB1:YAP1:ATN1:PHB2:TFCP2:COMMD6:GTF2A1:TCF12:TGFB1I1:NFATC3:DHX33:BRCA1:TAF4B:TCF3:KEAP1:PKN1:NCOA1:CREB1:HES6:PER2:PCNA:PPARA:CAND2:HDAC11:HYAL1:FOXP1:NR1I2:GSK3B:EIF4E:TERT:ISL1:HDAC3:ESR1:DACT2:NCAPG2:KLF4:GTF3C5
GO_mf	GO_ACTIVE_TRANSMEMBRANE_TRANSPORTER_ACTIVITY	356	32	5.845144411462854e-06	0.0003315607778226343	MFSD2A:ABCD3:SLC16A4:SLC16A12:ABCC8:SLC11A2:SLC25A3:ABCC11:SLC7A6:SLC5A10:SLC47A1:SLC47A2:RALBP1:CD320:SLC6A16:SLC4A5:SLC9A4:SLC11A1:SLC37A1:ATP5L2:ATP2C1:SLC34A2:SLC10A4:SLC4A4:SLC6A19:SLC6A18:SLC12A2:TAPBP:ABCB1:CDH17:SLC35D2:SLC34A3
GO_mf	GO_MONOVALENT_INORGANIC_CATION_TRANSMEMBRANE_TRANSPORTER_ACTIVITY	374	33	6.230429209289509e-06	0.0003351152696231635	KCNC4:HCN3:KCNJ11:ABCC8:KCNA1:SLC11A2:SLC25A3:MTMR6:KCNK10:SLC5A10:KCNJ16:KCNJ2:KCNJ14:SLC6A16:ATP6V1B1:SLC4A5:SLC9A4:SCN3A:SLC11A1:KCNS1:ATP5L2:SCN5A:ATP2C1:KCNAB1:SLC34A2:SLC10A4:SLC4A4:SLC6A19:SLC6A18:SLC12A2:KCNIP1:KCNV2:SLC34A3

GO_mf	GO_CADHERIN_BINDING	324	30	6.315242163111287e-06	0.0003351152696231635	MACF1:HSPA8:ERC1:GPRC5A:STXBP6:PSEN1:NUMB:PDXDC1:EPN2:TRIM25:SH3GL1:CC2D1A:GIPC1:DNAJB1:EPS15L1:PKP4:CHMP4B:MMP24:RPL14:ABCF3:CDH9:CDH6:DHX29:SEPT7:CALD1:ESYT2:CDH17:SCRIB:PUF60:CHMP5
GO_mf	GO_STRUCTURAL_MOLECULE_ACTIVITY	817	57	7.002690339137044e-06	0.00035998205024626365	RPL22:LDLRAP1:MACF1:LAMTOR5:HRNR:FLG:LCE1F:LCE1E:LCE1D:LCE1C:LCE1B:DAP3:LMNA:BGLAP:FMOD:NID1:TUBB8:MRPL49:POSTN:ACAN:RPL3L:MYLPF:SEPT1:SEPT1:MFAP4:RPL19:SPAG9:TUBD1:RPL38:MBP:TUBB4A:MRPS12:DEDD2:BFSP1:PI3:FBLN1:MAPK8IP2:SHANK3:RPL32:CMTM8:RPL14:MRPS22:COPB2:RPL37:TUBB:RPS18:MAP7:THBS2:SEPT7:CLDN3:CLDN4:COPG2:RB1CC1:RPL7:COL22A1:IPPK:RPL12:TUBB4B
GO_mf	GO_MOLECULAR_FUNCTION_REGULATOR	1787	104	7.319695501765078e-06	0.0003648757303152592	NBL1:TXLNA:CDC20:DNAJB4:BCL10:VAV3:LAMTOR5:DENND2C:SEMA6C:CKS1B:ARHGEF2:SEMA4A:ARHGEF11:AGAP4:GDF2:AGAP8:C10orf54:PSAP:IPO7:ARL2:GPRC5A:PTHLH:NCKAP1L:APAF1:ANKLE2:IPO5:TNFSF13B:PSME1:PPP1R36:PAPLN:HERC2:ARPP19:TBC1D10B:CCL17:TRPV1:RABEP1:RCVRN:PRPSAP2:CACNG1:RALBP1:PPP4R1:SERPINB4:DENND1C:ARHGEF18:CD320:RLN3:DNAJB1:CARD8:SPDYA:ALK:RTKN:VIL1:PCNA:RALGAPA2:MMP24:KCNS1:WFDC5:WFDC12:PI3:SLPI:TAB1:ARFGAP3:FBLN1:DENND6B:TYMP:CMTM8:HYAL2:NPRL2:PROS1:ADPRH:RASA2:KCNAB1:FGF12:STIM2:IL8:CXCL6:PF4V1:CXCL1:AREG:AREGB:CCNO:CKS1B:ARAP3:KCNIP1:SNCB:WRNIP1:EDN1:C4A:RGL2:CDKN1A:PI16:PLEKHG1:ESR1:FGFR1OP:HSPB1:YWHAG:PPP1R35:PRKAR2B:FAM3C:DENND2A:NCAPG2:NSMAF:GEM:LY6E:KLF4:PTPN3:DPM2
GO_mf	GO_CELL_ADHESION_MOLECULE_BINDING	487	39	9.464299481534428e-06	0.0004579050778565922	MACF1:ADAM15:TENM4:HSPA8:ERC1:PTPN6:GPRC5A:POSTN:LCP1:STXBP6:PSEN1:NUMB:PDXDC1:EPN2:ADAM11:TRIM25:SH3GL1:CC2D1A:GIPC1:DNAJB1:EPS15L1:PKP4:CHMP4B:MMP24:RPL14:ABCF3:CDH9:CDH6:DHX29:ITGB8:SEPT7:CALD1:ESYT2:LYN:CDH17:SCRIB:PUF60:CHMP5:S1PR3
GO_mf	GO_CATALYTIC_ACTIVITY_ACTING_ON_RNA	349	31	1.0296612467037777e-05	0.0004839407859507755	EXOSC10:NSUN6:ARL5B-AS1:REXO2:DDX25:EMG1:DHX37:RNASEH2B:DTD2:TDRD9:MTFMT:TARSL2:POLR2C:METTL16:DHX33:SARS2:TRMT61B:RTCB:THUMPD3:ISY1:DBR1:XRN1:TRMT10A:TERT:DROSHA:DHX29:SKIV2L2:DIMT1:RPP40:RNASET2:MEPCE:DUS4L
GO_mf	GO_G_PROTEIN_COUPLED_RECEPTOR_ACTIVITY	857	58	1.4270684678666877e-05	0.0006520910082335282	GPR153:HTR6:TM2D1:LPHN2:OR14K1:TACR2:RGR:OR52B4:OR51T1:OR4C15:OR4C16:OR4P4:OR4S2:OR4C6:OR5T1:OR8H1:OR8K3:OR8K1:OR8J1:OR8U1:OR5R1:GPRC5A:OR10P1:LGR5:MLNR:GLP2R:SSTR2:OR1M1:OR7G2:OR7G1:OR7G3:S1PR5:CD97:PTGER1:OR10H2:OR10H3:FPR2:FSHR:MC3R:MCHR1:CELSR1:ACKR2:ACKR4:NPY1R:NPY5R:PTGER4:PDGFRB:OR10C1:HCRTR2:CNR1:CRHR2:GPR22:TAS2R39:TAS2R40:S1PR3:OR1L3:OR1L4:OR1L6
GO_mf	GO_PEPTIDE_BINDING	290	27	1.629178941510919e-05	0.0007243241510230977	CLSTN1:LDLRAP1:TM2D1:APBB1:KPNA3:IPO5:KIAA1199:GRIN2A:GLP2R:NPEPPS:KPNB1:RPS6KB1:SSTR2:INSR:KDELR1:FPR2:MC3R:MCHR1:MAPK8IP2:AP2M1:HLA-A:TAPBP:PPIL1:HCRTR2:PEX7:CRHR2:CD36
GO_mf	GO_DOUBLE_STRANDED_DNA_BINDING	952	62	2.331746525507425e-05	0.00100940079854203	HES2:TARDBP:MTOR:ZBTB17:MED8:TAL1:ARNT:NHLH1:NUCKS1:ZMYND11:MCM10:CREM:ZNF143:EHF:YAP1:POU2AF1:TFCP2:HOXC11:ERCC5:NRL:SIX6:RFX7:TCF12:RRN3:NFATC3:SNAI3:DHX33:HNF1B:IKZF3:TCF3:SAFB:CC2D1A:RFX1:POU2F2:GRWD1:MYCN:OSR1:BCL11A:ZNF638:AFF3:RBMS1:CREB1:HES6:PER2:PCNA:ZBP1:PPARA:CGGBP1:NR1I2:CNBP:ISL1:CDX1:ESR1:GTF2H5:HOXA10:NEUROD6:CREB3L2:TERF1:KLF4:PBX3:GTF3C5:NACC2
GO_mf	GO_ENZYME_REGULATOR_ACTIVITY	1019	65	2.8094619804454074e-05	0.0011844610320863244	CDC20:DNAJB4:BCL10:VAV3:CKS1B:ARHGEF11:AGAP4:AGAP8:C10orf54:PSAP:IPO7:ARL2:GPRC5A:NCKAP1L:APAF1:ANKLE2:IPO5:PSME1:PPP1R36:PAPLN:ARPP19:TBC1D10B:RABEP1:RCVRN:PRPSAP2:RALBP1:PPP4R1:SERPINB4:DNAJB1:CARD8:SPDYA:ALK:RTKN:VIL1:PCNA:RALGAPA2:MMP24:WFDC5:WFDC12:PI3:SLPI:TAB1:ARFGAP3:FBLN1:HYAL2:NPRL2:PROS1:ADPRH:RASA2:CXCL1:CCNO:CKS1B:ARAP3:SNCB:WRNIP1:C4A:CDKN1A:PI16:ESR1:FGFR1OP:HSPB1:YWHAG:PPP1R35:PRKAR2B:NSMAF:KLF4:DPM2
GO_mf	GO_SECONDARY_ACTIVE_TRANSMEMBRANE_TRANSPORTER_ACTIVITY	234	23	2.8801484062889345e-05	0.0011844610320863244	MFSD2A:SLC16A4:SLC16A12:SLC11A2:SLC25A3:SLC7A6:SLC5A10:SLC47A1:SLC47A2:SLC6A16:SLC4A5:SLC9A4:SLC11A1:SLC37A1:SLC34A2:SLC10A4:SLC4A4:SLC6A19:SLC6A18:SLC12A2:CDH17:SLC35D2:SLC34A3
GO_mf	GO_PROTEIN_DIMERIZATION_ACTIVITY	1319	79	3.603700080185378e-05	0.0014458747882694994	ACOT7:HES2:NBL1:TAL1:ABCD3:ARNT:ANXA9:SYT11:NHLH1:CFHR5:PSAP:SCGB2A1:DGAT2:PRKRIR:TENM4:TRPC6:YAP1:TPI1:UHRF1BP1L:DAO:SDS:ERCC5:MAP3K9:GTF2A1:MGA:TCF12:SLC51B:CHRNB4:CORO1A:COQ9:POLR2C:RABEP1:HNF1B:IKZF3:AZI1:IMPA2:TAF4B:TCF3:KEAP1:GIPC1:CD79A:PAFAH1B3:CEACAM1:CARD8:MYCN:NCOA1:RPE:SLC11A1:VIL1:MFF:HES6:RALGAPA2:CHMP4B:NAGA:FAM109B:CELSR1:TYMP:CCDC66:DVL3:MAP3K13:MTTP:TERT:CDH9:CDH6:DROSHA:FLOT1:PEX7:SYNE1:FGFR1OP:NEUROD6:TPST1:HSPB1:PIP:TERF1:RPL7:CDH17:IZUMO3:XPA:NACC2

GO_mf	GO_VOLTAGE_GATED_ION_CHANNEL_ACTIVITY	194	20	4.775886221750899e-05	0.0018705554368524356	KCNC4:HCN3:KCNJ11:KCNA1:KCNK10:GRIN2A:CACNB1:CACNG1:KCNJ16:KCNJ2:GRIN2D:KCNJ14:SCN3A:KCNS1:SCN5A:CACNA2D2:KCNAB1:KCNIP1:CNR1:KCNV2
GO_mf	GO_PROTEIN_HOMODIMERIZATION_ACTIVITY	846	55	6.930271248578388e-05	0.002651231675328244	ACOT7:NBL1:ABCD3:ARNT:ANXA9:SYT11:CFHR5:PSAP:DGAT2:TENM4:TRPC6:TPI1:UHRF1BP1L:SDS:ERCC5:MAP3K9:TCF12:CORO1A:COQ9:RABEP1:HNF1B:IKZF3:AZI1:IMPA2:TCF3:KEAP1:GIPC1:CD79A:CEACAM1:CARD8:RPE:SLC11A1:VIL1:MFF:CHMP4B:NAGA:FAM109B:TYMP:CCDC66:MAP3K13:TERT:CDH9:CDH6:DROSHA:PEX7:SYNE1:FGFR1OP:TPST1:HSPB1:TERF1:RPL7:CDH17:IZUMO3:XPA:NACC2
GO_mf	GO_VOLTAGE_GATED_CATION_CHANNEL_ACTIVITY	139	16	7.210681220896502e-05	0.002695811501903351	KCNC4:HCN3:KCNJ11:KCNA1:GRIN2A:CACNB1:CACNG1:KCNJ16:KCNJ2:GRIN2D:KCNJ14:KCNS1:CACNA2D2:KCNAB1:CNR1:KCNV2
GO_mf	GO_PROTEIN_C_TERMINUS_BINDING	187	19	8.882493351037764e-05	0.003197172656338141	CDC20:BCL10:KCNJ11:PEX16:YAP1:PHB2:KPNA3:SH3GL3:CORO1A:SIAH1:SH3GL1:BCAM:NCL:MCHR1:FBLN1:SHANK3:TERT:NIPBL:MDC1
GO_mf	GO_UNFOLDED_PROTEIN_BINDING	127	15	8.940422017723677e-05	0.003197172656338141	DNAJB4:APCS:HSPA6:SYVN1:DNAJB13:HSPA8:SPG7:CHAF1A:DNAJB1:SHQ1:KIAA1239:TTC1:PFDN6:TAPBP:TUBB4B
GO_mf	GO_TRANSFERASE_ACTIVITY_TRANSFERRING_ONE_CARBON_GROUPS	224	21	0.0001235970061765345	0.00423611775962378	ICMT:DPH5:SETDB1:ASH1L:NSUN6:ARL5B-AS1:EMG1:METTL7A:MTFMT:CIAPIN1:PRMT7:METTL16:TRMT61B:METTL21A:NDUFAF5:THUMPD3:TRMT10A:DIMT1:INMT:MEPCE:ASH2L:NTMT1
GO_mf	GO_HYALURONONGLUCOSAMINIDASE_ACTIVITY	8	4	0.00012360708356349022	0.00423611775962378	KIAA1199:HYAL3:HYAL1:HYAL2
GO_mf	GO_HEXOSAMINIDASE_ACTIVITY	15	5	0.0001637695120151678	0.005497976474794919	KIAA1199:NAGA:HYAL3:HYAL1:HYAL2
GO_mf	GO_POTASSIUM_CHANNEL_ACTIVITY	121	14	0.00018904443044785222	0.006219561761734338	KCNC4:HCN3:KCNJ11:ABCC8:KCNA1:MTMR6:KCNK10:KCNJ16:KCNJ2:KCNJ14:KCNS1:KCNAB1:KCNIP1:KCNV2
GO_mf	GO_DNA_BINDING_TRANSCRIPTION_FACTOR_BINDING	336	27	0.00019978323961737893	0.006443988807266438	C1orf64:BCL10:ARNT:CREM:YAP1:PHB2:COMMD6:GTF2A1:TCF12:TGFB1I1:DHX33:BRCA1:TAF4B:TCF3:PKN1:NCOA1:CREB1:PCNA:PPARA:FOXP1:GSK3B:EIF4E:ISL1:HDAC3:ESR1:NCAPG2:KLF4
GO_mf	GO_GTPASE_ACTIVITY	319	26	0.00020808847441764058	0.006582798854173437	CDC42:NRAS:RIT1:TUBB8:RAB18:ARL2:RAP1B:RASL12:SEPT1:SEPT1:RNF112:RND2:TUBD1:TUBB4A:RND3:GNAI2:RAB43:RAB6B:MRAS:LSG1:TUBB:RAB44:SEPT7:GNG11:GEM:DIRAS2:TUBB4B
GO_mf	GO_TRANSFERASE_ACTIVITY_TRANSFERRING_PHOSPHORUS_CONTAINING_GROUPS	908	56	0.00023247677315172083	0.007215552676124165	MTOR:TIE1:HIPK1:CLK2:PKLR:UHMK1:ABL2:ADCK3:ALDH18A1:GRK5:WEE1:AASDHPPT:DYRK4:NUAK1:PRKAB1:CIT:ULK1:POLE:MAP3K9:SMG1:POLR2C:SHPK:SHPK:PRPSAP2:MAPK7:DGKE:RPS6KB1:INSR:PKN1:MAST3:GSK3A:CTU1:ALK:MERTK:PANK2:CDS2:UCKL1:CHKB:TGFBR2:NPRL2:GSK3B:NEK11:GK5:MAP3K13:STK32B:TERT:CCNO:PDGFRB:THG1L:STK19:CDKN1A:BLK:LYN:TERF1:NRBP2:IPPK:PIP5KL1
GO_mf	GO_CALCIUM_ION_TRANSMEMBRANE_TRANSPORTER_ACTIVITY	139	15	0.00024623822078986336	0.007501145799987504	TRPC6:ORAI1:PSEN1:TRPM1:PKD1:GRIN2A:TRPV1:CACNB1:CACNG1:GRIN2D:PKDREJ:CACNA2D2:ATP2C1:STIM2:CNR1
GO_mf	GO_S_ADENOSYLMETHIONINE_DEPENDENT_METHYLTRANSFERASE_ACTIVITY	155	16	0.00025923412691891635	0.007753457068756681	ICMT:DPH5:SETDB1:ASH1L:NSUN6:ARL5B-AS1:EMG1:PRMT7:METTL16:TRMT61B:METTL21A:THUMPD3:TRMT10A:DIMT1:INMT:MEPCE:ASH2L
GO_mf	GO_POTASSIUM_ION_TRANSMEMBRANE_TRANSPORTER_ACTIVITY	157	16	0.0002999469401231943	0.008810941366118832	KCNC4:HCN3:KCNJ11:ABCC8:KCNA1:MTMR6:KCNK10:KCNJ16:KCNJ2:KCNJ14:SLC9A4:KCNS1:KCNAB1:SLC12A2:KCNIP1:KCNV2

GO_mf	GO_SEQUENCE_SPECIFIC_DOUBLE_STRANDED_DNA_BINDING	860	53	0.00034360157461017093	0.009679120305546188	HES2:TARDBP:MTOR:ZBTB17:MED8:TAL1:ARNT:NHLH1:MCM10:CREM:ZNF143:EHF:YAP1:POU2AF1:TFCP2:HOXC11:NRL:SIX6:RFX7:TCF12:RRN3:NFATC3:SNAI3:DHX33:HNF1B:IKZF3:TCF3:SAFB:CC2D1A:RFX1:POU2F2:GRWD1:MYCN:OSR1:BCL11A:CREB1:HES6:PER2:PPARA:CGGBP1:NR1I2:CNBP:ISL1:CDX1:ESR1:GTF2H5:HOXA10:NEUROD6:CREB3L2:KLF4:PBX3:GTF3C5:NACC2
GO_mf	GO_TRANSCRIPTION_COREGULATOR_ACTIVITY	557	38	0.00034883035223732926	0.009679120305546188	HES2:SPEN:MED8:BCL10:WDR77:ARNT:GON4L:PMF1:ZMYND11:SFMBT2:YAP1:POU2AF1:ATN1:NRL:GTF2A1:SKOR1:TGFB1I1:BRCA1:SKOR2:TCF3:PKN1:DNAJB1:NFKBIB:ERF:NCOA1:CREB1:HES6:PER2:WBP2NL:PPARA:HYAL2:NR1I2:ISL1:MCIDAS:HDAC3:JAZF1:ENY2:NACC2
GO_mf	GO_RDNA_BINDING	10	4	0.0003490242137353736	0.009679120305546188	MTOR:DHX33:GTF2H5:GTF3C5
GO_mf	GO_SOLUTE_SODIUM_SYMPORTER_ACTIVITY	72	10	0.0003530378226946938	0.009679120305546188	SLC5A10:SLC6A16:SLC4A5:SLC34A2:SLC10A4:SLC4A4:SLC6A19:SLC6A18:SLC12A2:SLC34A3
GO_mf	GO_SYMPORTER_ACTIVITY	144	15	0.00036163047185891483	0.009752166003408442	MFSD2A:SLC16A4:SLC16A12:SLC11A2:SLC25A3:SLC5A10:SLC6A16:SLC4A5:SLC34A2:SLC10A4:SLC4A4:SLC6A19:SLC6A18:SLC12A2:SLC34A3
GO_mf	GO_CARGO_RECEPTOR_ACTIVITY	86	11	0.0003782170923098814	0.010034953497576693	LDLRAP1:DMBT1:ASGR2:INSR:FPR2:ACKR2:ACKR4:AP2M1:CFI:MEGF10:CD36
GO_mf	GO_SOLUTE_CATION_SYMPORTER_ACTIVITY	102	12	0.0004560923306723385	0.01190907752311106	SLC11A2:SLC25A3:SLC5A10:SLC6A16:SLC4A5:SLC34A2:SLC10A4:SLC4A4:SLC6A19:SLC6A18:SLC12A2:SLC34A3
GO_mf	GO_DNA_BINDING_TRANSCRIPTION_ACTIVATOR_ACTIVITY	414	30	0.0005336914615283291	0.013567946835429615	TARDBP:ZBTB17:FOXD2:NHLH1:NUCKS1:ZNF143:EHF:CSRNP2:TFCP2:HOXC13:HOXC11:NRL:SIX6:TCF12:NFATC3:IKZF3:POU2F2:MYCN:CREB1:PPARA:NR1I2:ISL1:CDX1:ESR1:HOXA10:NEUROD6:KLF14:CREB3L2:KLF4:PBX3
GO_mf	GO_ANION_TRANSMEMBRANE_TRANSPORTER_ACTIVITY	321	25	0.0005361194798194072	0.013567946835429615	MFSD2A:LRRC8B:LRRC8C:SLC16A4:SLC16A12:SLC43A1:SLC25A15:SLC51B:ABCC11:SLC7A6:TTYH2:CEACAM1:SLC4A5:SLC37A1:APOL1:SLC34A2:SLC10A4:SLC4A4:SLC6A19:SLC6A18:SLC12A2:CLDN4:SLC35D2:SLC25A25:SLC34A3
GO_mf	GO_UBIQUITIN_BINDING	76	10	0.0005489138931417352	0.013681262942699309	CKS1B:UBE2L6:USP5:UCHL3:NBR1:RNFT1:NPLOC4:USP25:CKS1B:TNFAIP3:HSPB1
GO_mf	GO_SIGNALING_RECEPTOR_BINDING	1619	87	0.0005712589207436051	0.013914699585548775	C1orf64:NBL1:CDC42:LDLRAP1:TXLNA:VAV3:ARNT:SEMA6C:ADAM15:SEMA4A:ARHGEF11:GDF2:PSAP:CRTAM:HSPA8:PHB2:PTHLH:DAO:LCP1:TNFSF13B:ARPP19:MESDC2:TGFB1I1:LONP2:CCL17:RABEP1:BRCA1:ADAM11:ASXL3:SH3GL1:INSR:CD320:RLN3:PKN1:PTGER1:GIPC1:IL12RB1:GSK3A:CBLC:FPR2:NCOA1:IGFBP2:PCNA:APOL2:MCHR1:TYMP:SHANK3:TGFBR2:CMTM8:TRAK1:GNAI2:HYAL2:FOXP1:PLSCR4:DVL3:AP2M1:FGF12:IL8:CXCL6:PF4V1:CXCL1:AREG:AREGB:ISL1:ANKRA2:MEGF10:DIAPH1:PDGFRB:EDN1:HLA-A:TUBB:FLOT1:TAPBP:MAP7:ESR1:ITGB8:YWHAG:CD36:PILRB:PILRA:FAM3C:BLK:LYN:CDH17:SCRIB:S1PR3:CEL:TUBB4B
GO_mf	GO_SMALL_GTPASE_BINDING	435	31	0.0005751973080956333	0.013914699585548775	VAV3:DENND2C:SIKE1:ARHGEF2:ARHGEF11:IPO7:ERC1:NCKAP1L:ULK1:IPO5:STXBP6:MYO5A:TBC1D10B:KPNB1:RALBP1:DENND1C:ARHGEF18:PKN1:XPO1:RTKN:DENND6B:MYRIP:ADPRH:DVL3:IPO11:DIAPH1:VPS52:RGL2:PLEKHG1:EXOC4:DENND2A
GO_mf	GO_KINASE_ACTIVITY	758	47	0.0006363195487964488	0.015090477545626492	MTOR:TIE1:HIPK1:CLK2:PKLR:UHMK1:ABL2:ADCK3:ALDH18A1:GRK5:WEE1:DYRK4:NUAK1:PRKAB1:CIT:ULK1:MAP3K9:SMG1:SHPK:SHPK:MAPK7:DGKE:RPS6KB1:INSR:PKN1:MAST3:GSK3A:ALK:MERTK:PANK2:UCKL1:CHKB:TGFBR2:NPRL2:GSK3B:NEK11:GK5:MAP3K13:STK32B:CCNO:PDGFRB:STK19:CDKN1A:BLK:LYN:NRBP2:IPPK:PIP5KL1
GO_mf	GO_PEPTIDE_RECEPTOR_ACTIVITY	152	15	0.000642147980664957	0.015090477545626492	TACR2:LGR5:MLNR:GLP2R:SSTR2:FPR2:FSHR:MC3R:MCHR1:ACKR2:ACKR4:NPY1R:NPY5R:HCRTR2:CRHR2
GO_mf	GO_MHC_CLASS_I_PROTEIN_BINDING	20	5	0.0007227600354142013	0.01674563744023044	TUBB:TAPBP:PILRB:PILRA:TUBB4B
GO_mf	GO_TRANSCRIPTION_COFACTOR_BINDING	41	7	0.0007690334667533676	0.01748408651489202	ZBTB17:CREB1:PER2:PPARA:TERT:ESR1:KLF4

GO_mf	GO_ATPASE_ACTIVITY	443	31	0.0007758895535483997	0.01748408651489202	MACF1:ABCD3:HSPA6:TOR3A:ABCC8:HSPA8:DDX25:DHX37:FANCM:TDRD9:ABCC11:LONP2:DHX33:DNAH9:DNAI2:KIF19:RALBP1:CD320:DDX39A:ATP5L2:RAD18:ATP2C1:ABCF3:ATP10D:DHX29:SKIV2L2:KIF2A:WRNIP1:TAPBP:RFC2:ABCB1
GO_mf	GO_UBIQUITIN_LIKE_PROTEIN_BINDING	96	11	0.0009700978999416948	0.021394546194868108	CKS1B:UBE2L6:USP5:UCHL3:HERC2:NBR1:RNFT1:NPLOC4:USP25:CKS1B:TNFAIP3:HSPB1
GO_mf	GO_MANGANESE_ION_TRANSMEMBRANE_TRANSPORTER_ACTIVITY	6	3	0.00097543523684809	0.021394546194868108	SLC11A2:SLC11A1:ATP2C1
GO_mf	GO_GTPASE_BINDING	531	35	0.0010675941536475524	0.023107794509871365	VAV3:LAMTOR5:DENND2C:SIKE1:ARHGEF2:ARHGEF11:IPO7:ERC1:NCKAP1L:ULK1:LCP1:IPO5:STXBP6:HERC2:MYO5A:TBC1D10B:KPNB1:RALBP1:SH3GL1:DENND1C:ARHGEF18:PKN1:XPO1:RTKN:DENND6B:MYRIP:ADPRH:DVL3:IPO11:DIAPH1:VPS52:RGL2:PLEKHG1:EXOC4:DENND2A
GO_mf	GO_ATPASE_ACTIVITY_COUPLED	356	26	0.0010821907882107723	0.023119530475411956	ABCD3:HSPA6:ABCC8:HSPA8:DDX25:DHX37:FANCM:TDRD9:ABCC11:LONP2:DHX33:DNAH9:DNAI2:KIF19:RALBP1:CD320:ATP5L2:RAD18:ATP2C1:ATP10D:DHX29:SKIV2L2:WRNIP1:TAPBP:RFC2:ABCB1
GO_mf	GO_TRANSCRIPTION_COACTIVATOR_BINDING	22	5	0.001153219680195366	0.024294114228305026	ZBTB17:CREB1:PPARA:TERT:ESR1
GO_mf	GO_GATED_CHANNEL_ACTIVITY	339	25	0.0011667082212985392	0.024294114228305026	KCNC4:HCN3:KCNJ11:KCNA1:P2RX2:MTMR6:KCNK10:CHRNB4:GRIN2A:TRPV1:CACNB1:CACNG1:KCNJ16:KCNJ2:TTYH2:GRIN2D:KCNJ14:SCN3A:KCNS1:SCN5A:CACNA2D2:KCNAB1:KCNIP1:CNR1:KCNV2
GO_mf	GO_VOLTAGE_GATED_POTASSIUM_CHANNEL_ACTIVITY	84	10	0.0012120792769231773	0.024923380131732835	KCNC4:HCN3:KCNJ11:KCNA1:KCNJ16:KCNJ2:KCNJ14:KCNS1:KCNAB1:KCNV2
GO_mf	GO_LIPID_TRANSPORTER_ACTIVITY	146	14	0.0012675868744738323	0.025742968006289557	MFSD2A:ABCD3:LINC01059:GRAMD1B:SLC51B:PITPNC1:CEACAM1:OSBP2:PLSCR4:ATP10D:SLC10A4:MTTP:PRELID2:ABCB1:STAR
GO_mf	GO_UBIQUITIN_LIKE_PROTEIN_LIGASE_BINDING	306	23	0.0014031806785506952	0.028149173368486508	BCL10:SCAMP3:SYT11:SMG5:CUL2:APBB1:UBE2L6:UBASH3B:HSPA8:TPI1:MOAP1:HERC2:BRCA1:NPLOC4:USP25:RAD18:SCN5A:GSK3B:ANKRA2:TUBB:CDKN1A:PRKAR2B:LYN
GO_mf	GO_HYDROLASE_ACTIVITY_ACTING_ON_GLYCOSYL_BONDS	116	12	0.0014385112449786265	0.02828425316117827	OVGP1:PSAP:SMPD1:KIAA1199:LYZL6:PCNA:NAGA:HYAL3:HYAL1:HYAL2:ADPRH:MAN2B2
GO_mf	GO_SODIUM_ION_TRANSMEMBRANE_TRANSPORTER_ACTIVITY	148	14	0.0014443022890814436	0.02828425316117827	HCN3:SLC5A10:SLC6A16:SLC4A5:SLC9A4:SCN3A:SCN5A:SLC34A2:SLC10A4:SLC4A4:SLC6A19:SLC6A18:SLC12A2:SLC34A3
GO_mf	GO_PEPTIDASE_ACTIVITY	623	39	0.0014853081169438757	0.028745080616149124	F3:CTSS:CTSK:FAM63A:ADAM15:PSEN2:RBP3:STAMBPL1:TLL2:HABP2:PGA3:ASRGL1:NAALADL1:RCE1:USP5:C1S:UCHL3:PSEN1:PAPLN:OTUD7A:JMJD7:ADAMTS7:PRSS21:LONP2:SPG7:ADAM11:NPEPPS:MMP24:USP25:SENP2:CFI:ADAM19:PRSS16:TNFAIP3:CPA1:KEL:PIP:ADAMTSL1:PAPPA
GO_mf	GO_PHOSPHOLIPID_BINDING	423	29	0.0015342860791049337	0.02926634121838413	LDLRAP1:RPE65:F3:GOLPH3L:ANXA9:SYT11:PHLDA3:PSAP:GRK5:SYT13:SNX15:LINC01059:GRAMD1B:STXBP6:ZFYVE1:PLA2G4B:PLA2G15:TRPV1:PIRT:PITPNC1:KCNJ2:MPPE1:DENND1C:PLEKHB2:VIL1:BPIFC:RASA2:ARAP3:CPNE5:ESYT2
GO_mf	GO_SIGNAL_SEQUENCE_BINDING	46	7	0.0015555116333744417	0.02926634121838413	KPNA3:IPO5:KIAA1199:KPNB1:KDELR1:AP2M1:PEX7
GO_mf	GO_CARBOXYLIC_ESTER_HYDROLASE_ACTIVITY	133	13	0.0015656158220169018	0.02926634121838413	ACOT7:ACOT11:RPE65:DTD2:PLA2G4B:PLA2G15:PAFAH1B3:LIPE:PLB1:LIPH:PLA2G12A:CA2:CEL
GO_mf	GO_O_ACYLTRANSFERASE_ACTIVITY	47	7	0.001769216464698715	0.032700686341903214	SOAT1:GPAM:MOGAT2:DGAT2:LPCAT3:PLA2G15:CPT1B

GO_mf	GO_UBIQUITIN_LIKE_PROTEIN_TRANSFERASE_ACTIVITY	408	28	0.001803477782573218	0.032963566137032704	CDC42:TRIM62:TRIM33:DCST1:CUL2:HECTD2:UBE2L6:SYVN1:SPSB2:FBXW8:SIAH3:LMO7:G2E3:UBR7:HERC2:SIAH1:RNF112:BRCA1:TRIM25:RNFT1:RNF157:CBLC:ANAPC1:RAD18:UBE2QL1:TSPAN17:TNFAIP3:LRSAM1
GO_mf	GO_STEROID_HORMONE_RECEPTOR_BINDING	89	10	0.0018865360905982553	0.034102767791583846	C1orf64:PHB2:TGFB1I1:BRCA1:PKN1:NCOA1:PCNA:FOXP1:ISL1:ESR1
GO_mf	GO_REGULATORY_REGION_NUCLEIC_ACID_BINDING	934	53	0.002149021144662786	0.038425432423590034	HES2:MTOR:ZBTB17:MED8:TAL1:ARNT:C1orf85:NHLH1:CREM:ZNF143:EHF:YAP1:POU2AF1:TFCP2:HOXC11:NRL:SIX6:RFX7:TCF12:RRN3:NFATC3:SNAI3:HNF1B:IKZF3:BRCA1:TBX21:TCF3:SAFB:CC2D1A:RFX1:POU2F2:MYCN:OSR1:NCOA1:BCL11A:CREB1:HES6:PER2:PPARA:CGGBP1:NR1I2:CNBP:ISL1:CDX1:ESR1:HOXA10:NEUROD6:CREB3L2:ASH2L:KLF4:PBX3:GTF3C5:NACC2
GO_mf	GO_MANGANESE_ION_BINDING	62	8	0.0021888298549811126	0.03871639904778419	EXTL2:ABL2:GYLTL1B:SLC11A2:PCK2:MPPE1:MGAT1:SOD2
GO_mf	GO_PROTEIN_CONTAINING_COMPLEX_SCAFFOLD_ACTIVITY	91	10	0.002229371312621055	0.039013997970868464	LDLRAP1:LAMTOR5:SEPT1:SEPT1:SPAG9:DEDD2:MAPK8IP2:SHANK3:SEPT7:RB1CC1:IPPK
GO_mf	GO_ION_CHANNEL_BINDING	123	12	0.0023697105990414732	0.04085798645272027	RNF207:KCNJ11:ABCC8:PKD1:PIRT:SH3GL1:SCN5A:KCNAB1:AP2M1:FGF12:DIAPH1:LYN
GO_mf	GO_AMYLOID_BETA_BINDING	77	9	0.0023844174464809397	0.04085798645272027	CLSTN1:LDLRAP1:TM2D1:APBB1:GRIN2A:INSR:FPR2:MAPK8IP2:CD36
GO_mf	GO_RNA_POLYMERASE_II_SPECIFIC_DNA_BINDING_TRANSCRIPTION_FACTOR_BINDING	263	20	0.0024267457786056397	0.04115460624542554	C1orf64:BCL10:ARNT:PHB2:COMMD6:GTF2A1:TGFB1I1:BRCA1:TAF4B:PKN1:NCOA1:CREB1:PCNA:PPARA:FOXP1:GSK3B:ISL1:HDAC3:ESR1:KLF4
GO_mf	GO_ALKALI_METAL_ION_BINDING	16	4	0.002524679115320403	0.042030642738397406	PKLR:KCNJ11:ADPRH:SLC34A2
GO_mf	GO_RRNA_ADENINE_METHYLTRANSFERASE_ACTIVITY	8	3	0.002580604812509506	0.042030642738397406	METTL16:TRMT61B:DIMT1
GO_mf	GO_COMPLEMENT_COMPONENT_C1Q_BINDING	8	3	0.002580604812509506	0.042030642738397406	APCS:MEGF10:C4A
GO_mf	GO_INSULIN_LIKE_GROWTH_FACTOR_II_BINDING	8	3	0.002580604812509506	0.042030642738397406	INSR:IGFBP2:IGFBP5
GO_mf	GO_PROTEASE_BINDING	125	12	0.0027112553310004164	0.04372563744603613	BCL10:F3:PSAP:SYVN1:LONP2:SERPINB4:MBP:SEMG2:GSK3B:DVL3:FLOT1:TNFAIP3
GO_mf	GO_PHOSPHORIC_ESTER_HYDROLASE_ACTIVITY	364	25	0.0030408459338173615	0.04856496661290834	PTP4A2:PRUNE:DUSP8:SMPD1:UBASH3B:PTPN6:PDE1B:MTMR6:DLGAP5:SMPD3:PPP4R1:MPPE1:IMPA2:PHLPP1:PDE4A:PPP1CB:DUSP11:CTDSP1:TAB1:PLCXD2:PPAP2A:PTP4A1:PRKAR2B:PPP3CC:PTPN3
Cancer_gene_neighborhoods	GNF2_FBL	147	18	1.119720675127091e-05	0.004781207282792679	RPL22:TARDBP:RBM15:DAP3:SNRPE:GTF3A:RPL19:KPNB1:RPL38:XPO1:NCL:RPL32:RPL14:RPL37:DIMT1:RPL7:KIAA0020:RPL12
Cancer_gene_neighborhoods	GCM_DFFA	129	16	2.8834050685407496e-05	0.0061560698213345005	TARDBP:C1orf52:CSDE1:KIAA0907:LINC00610:COMMD9:KPNA3:IPO5:RBM25:UBR7:SMG1:TBC1D10B:FAM168B:ARMC8:ZFP62:VPS52:MEPCE
Cancer_gene_neighborhoods	GCM_DEAF1	27	7	4.807975863029308e-05	0.006843352311711715	GPR153:MOAP1:HERC2:NAPG:FAM168B:YWHAG:UBQLN1

Cancer_gene_neighborhoods	MORF_AATF	209	20	0.0001343391079942309	0.011758583513655801	CLSTN1:MTOR:TXLNA:DAP3:HSPA8:USP5:PHB2:COQ9:POLR2C:SAFB:DDX39A:PUM2:XPO1:NCL:EIF6:RTCB:SEPT7:ASH2L:PUF60:NELFB
Cancer_gene_neighborhoods	MORF_DAP3	195	19	0.0001545615560006274	0.011758583513655801	FDPS:DAP3:SSR2:SNRPE:HSPA8:PHB2:SLC25A3:PUM2:XPO1:NCL:EIF6:RPN2:RTCB:VGLL4:CNBP:AP2M1:RPP40:PUF60:NELFB
Cancer_gene_neighborhoods	GCM_RAN	196	19	0.00016522599784996443	0.011758583513655801	HIPK1:TRIM33:IPO7:SNX15:CSRNP2:KPNA3:IPO5:MOAP1:UBR7:HERC2:ARPP19:TBC1D10B:SPAG9:PITPNC1:NAPG:FAM168B:GSK3B:VPS52:YWHAG
Cancer_gene_neighborhoods	MORF_EIF4A2	138	15	0.00022744706158972687	0.013874270756973337	RPL22:PTP4A2:DAP3:ZFPL1:FAU:SLC25A3:RPL19:PUM2:XPO1:FAM168B:NCL:RPL14:SEPT7:TERF1:RPL7
Cancer_gene_neighborhoods	MORF_SUPT3H	327	26	0.00030591727781152054	0.015605148018150576	LPHN2:FAS:ABCC8:TENM4:OPCML:ERC1:AKAP3:HOXC11:WBP4:SIX6:CHRNB4:CACNB1:BRCA1:OR10H3:FSHR:CYP2D6:DAZL:ENTPD3:NR1I2:SLC4A4:ISL1:LECT2:HCRTR2:ABCB1:EXOC4:RB1CC1
Cancer_gene_neighborhoods	MORF_CDC10	146	15	0.0004193516719124144	0.015605148018150576	RPL22:CLSTN1:PTP4A2:TXLNA:CUL2:HSPA8:POLR2C:SAFB:PUM2:XPO1:RTCB:DIAPH1:SEPT7:ASH2L:TERF1
Cancer_gene_neighborhoods	MORF_DDB1	245	21	0.0004206329371108261	0.015605148018150576	CLSTN1:TXLNA:ADAM15:FAM189B:DAP3:HSPA8:USP5:PHB2:PDXDC1:POLR2C:DDX39A:EIF6:RTCB:VGLL4:DIMT1:CETN3:MGAT1:STK19:SEPT7:PUF60:NELFB
Cancer_gene_neighborhoods	MORF_ERCC4	320	25	0.0005122395567698657	0.015605148018150576	TIE1:FAS:ABCC8:TENM4:ERC1:HOXC11:WBP4:SIX6:CHRNB4:CACNB1:BRCA1:PDE4A:OR10H3:FPR2:FSHR:CYP2D6:GTSE1:ENTPD3:NR1I2:ISL1:LECT2:HCRTR2:ABCB1:EXOC4:RB1CC1
Cancer_gene_neighborhoods	GCM_NF2	302	24	0.0005149750890453727	0.015605148018150576	C1orf52:ASH1L:UBQLN4:SMG5:NUCKS1:AGAP4:AGAP8:LINC00610:COMMD9:SNX15:DDX47:CSRNP2:DHX37:IPO5:GTF2A1:UBR7:TBC1D10B:CIAPIN1:NAPG:SAFB:EPS15L1:FAM168B:ACAD11:ANKRA2:VPS52:MEPCE
Cancer_gene_neighborhoods	GNF2_DAP3	119	13	0.0005540452006474264	0.015605148018150576	RPL22:TARDBP:RBM15:DAP3:SNRPE:GTF3A:RPL19:RPL38:RPL32:RPL14:RPL37:RPL7:RPL12
Cancer_gene_neighborhoods	GNF2_ST13	64	9	0.0006283059630125116	0.015605148018150576	RPL22:SNRPE:RPL19:RPL38:RPL32:RPL14:RPL37:RPL7:RPL12
Cancer_gene_neighborhoods	MORF_PTPRB	253	21	0.0006409234216007166	0.015605148018150576	LPHN2:FAS:ABCC8:TENM4:ERC1:WBP4:SIX6:NIPA2:CHRNB4:OR10H3:FPR2:TPO:FSHR:DAZL:NR1I2:ISL1:LECT2:HCRTR2:ABCB1:EXOC4:RB1CC1
Cancer_gene_neighborhoods	GNF2_EIF3S6	121	13	0.0006498381056076144	0.015605148018150576	RPL22:DAP3:FAU:DDX47:GTF3A:RPL19:RPL38:RPL32:RPL14:RPL37:RPS18:RPL7:RPL12
Cancer_gene_neighborhoods	MORF_CCNF	78	10	0.0006763263993141517	0.015605148018150576	EXTL2:PDXDC1:SMG1:BRCA1:KPNB1:GTSE1:COQ2:EIF4E:DIMT1:CETN3
Cancer_gene_neighborhoods	GCM_GSPT1	169	16	0.0006797188333333953	0.015605148018150576	TARDBP:TRIM33:KIAA0907:AGAP4:AGAP8:LINC00610:COMMD9:SNX15:CSRNP2:IPO5:UBR7:TBC1D10B:NAPG:FAM168B:ATP2C1:ANKRA2:VPS52:MEPCE
Cancer_gene_neighborhoods	MORF_ACTG1	138	14	0.0007303116987606548	0.015605148018150576	RPL22:SSR2:ZFPL1:FAU:SLC25A3:RPL19:RPL38:NCL:RPL32:RPL14:RPL37:RPS18:RPL7:RPL12
Cancer_gene_neighborhoods	MORF_UBE2I	238	20	0.0007309202818805891	0.015605148018150576	RPL22:TARDBP:PTP4A2:DAP3:SSR2:SNRPE:ZFPL1:FAU:PHB2:SLC25A3:RPL19:XPO1:FAM168B:NCL:EIF6:RPL14:CNBP:AP2M1:RPL7:PUF60
Cancer_gene_neighborhoods	MORF_PRKAG1	223	19	0.0008306767552365658	0.016890427356476837	CLSTN1:TXLNA:ADAM15:FAM189B:DAP3:HSPA8:USP5:PDXDC1:COQ9:POLR2C:RPN2:RTCB:VGLL4:DIMT1:CETN3:MGAT1:STK19:SEPT7:NELFB
Cancer_gene_neighborhoods	GCM_MLL	175	16	0.0009893100056247038	0.018557145343792908	TARDBP:C1orf52:KIAA0907:LINC00610:COMMD9:DDX47:CSRNP2:IPO5:RBM25:UBR7:SMG1:TBC1D10B:SAFB:FAM168B:CREB1:ANKRA2:MEPCE
Cancer_gene_neighborhoods	MORF_RPA2	192	17	0.0009995652058717491	0.018557145343792908	CLSTN1:TXLNA:ADAM15:FAM189B:USP5:PHB2:COQ9:GSK3A:XPO1:EIF6:RTCB:IFRD2:DIAPH1:MGAT1:SEPT7:ASH2L:NELFB
Cancer_gene_neighborhoods	GCM_CSNK1D	32	6	0.0010959683886225082	0.01882431959912639	GPR153:FAM168B:CHMP4B:VPS52:ZFAND3:UBQLN1
Cancer_gene_neighborhoods	MORF_PPP2CA	128	13	0.0011021264402298824	0.01882431959912639	TARDBP:HSPA8:RAP1B:SLC25A3:SAFB:DDX39A:XPO1:FAM168B:NCL:RTCB:CNBP:SEPT7:PUF60

Cancer_gene_neighborhoods	MORF_NPM1	162	15	0.0012356773436303398	0.01963690025264167	RPL22:SNRPE:FAU:PHB2:SLC25A3:RPL19:RPL38:NCL:RPL32:RPL14:RPL37:TUBB:RPS18:RPL7:RPL12
Cancer_gene_neighborhoods	MORF_RAN	267	21	0.0012698873284719273	0.01963690025264167	TARDBP:FDPS:DAP3:SSR2:SNRPE:FAU:HSPA8:TPI1:PHB2:SLC25A3:UTP18:MRPS12:XPO1:NCL:PCNA:RPN2:RPL14:CNBP:AP2M1:RPL7:PUF60
Cancer_gene_neighborhoods	MORF_GNB1	304	23	0.0012876655903371588	0.01963690025264167	CLSTN1:TARDBP:PTP4A2:TXLNA:SNRPE:PHB2:SLC25A3:ANP32A:POLR2C:SAFB:PKN1:PUM2:XPO1:FAM168B:NCL:RTCB:TRAK1:DCTD:SKIV2L2:DIAPH1:MDC1:SEPT7:TERF1
Cancer_gene_neighborhoods	GNF2_NPM1	72	9	0.0014860993320104999	0.021881531543740805	TARDBP:SNRPE:XPO1:NCL:RPL14:RPL37:DIMT1:RPL7:KIAA0020
Cancer_gene_neighborhoods	MORF_XRCC5	235	19	0.0015389923025911842	0.021904990440214524	TARDBP:TXLNA:DAP3:SNRPE:PHB2:SLC25A3:ANP32A:COQ9:CHAF1A:SAFB:PUM2:XPO1:FAM168B:NCL:DCTD:SKIV2L2:DIAPH1:SEPT7:TERF1
Cancer_gene_neighborhoods	MORF_RAD23A	348	25	0.0016727748007114539	0.02304112386786422	ACOT7:TARDBP:SCAMP3:SNRPE:PHB2:SLC25A3:ERCC5:ANP32A:SAFB:DDX39A:PKN1:MRPS12:XPO1:FAM168B:NCL:RTCB:DCTD:SKIV2L2:DIAPH1:TTC1:MDC1:NSMAF:TERF1:PUF60:UBAC1
Cancer_gene_neighborhoods	GCM_MYST2	172	15	0.0022351454202916505	0.02982522170201671	TARDBP:PTBP2:HIPK1:CSDE1:ARL5B:IPO7:LINC00610:COMMD9:CSRNP2:KPNA3:RBM25:UBR7:SPAG9:FAM168B:GSK3B:ZFAND3
Cancer_gene_neighborhoods	MORF_MLLT10	301	22	0.0024879390939457294	0.03219242403378262	TIE1:FAS:ABCC8:TENM4:ERC1:AKAP3:WBP4:SIX6:MGA:CHRNB4:CACNB1:BRCA1:OR10H3:FSHR:CYP2D6:DAZL:NR1I2:LECT2:HCRTR2:ABCB1:EXOC4:RB1CC1
Cancer_gene_neighborhoods	MORF_ACP1	211	17	0.002745035258265616	0.032793195739494914	RPL22:TARDBP:PTP4A2:SNRPE:ZFPL1:PHB2:SLC25A3:ERCC5:ANP32A:SAFB:XPO1:FAM168B:NCL:RPL14:DIAPH1:TTC1:TERF1
Cancer_gene_neighborhoods	GNF2_TPT1	38	6	0.0027565066306163484	0.032793195739494914	RPL19:RPL38:RPL32:RPL37:RPS18:RPL12
Cancer_gene_neighborhoods	GNF2_STAT6	79	9	0.0028461722026213777	0.032793195739494914	PTPN6:LCP1:CORO1A:MOB1A:HLA-A:TAPBP:RNASET2:LAT2:LYN
Cancer_gene_neighborhoods	GCM_CALM1	110	11	0.002918364023655285	0.032793195739494914	DDAH1:CSRNP2:NUAK1:MOAP1:HERC2:NAPG:FAM168B:GSK3B:NDFIP1:VPS52:YWHAG
Cancer_gene_neighborhoods	GNF2_HDAC1	110	11	0.002918364023655285	0.032793195739494914	RPL22:TARDBP:RPF1:RBM15:CLK2:DAP3:DDX47:GTF3A:KPNB1:OSR1:TAPBP
Cancer_gene_neighborhoods	MORF_RAD51L3	386	26	0.0032998831845490197	0.0361294902513444	LPHN2:FAS:ABCC8:TENM4:OPCML:ERC1:AKAP3:HOXC11:WBP4:SIX6:MGA:CHRNB4:CACNB1:BRCA1:OR10H3:FSHR:CYP2D6:DAZL:NR1I2:SLC4A4:ISL1:LECT2:HCRTR2:ABCB1:EXOC4:RB1CC1
Cancer_gene_neighborhoods	MORF_FOSL1	407	27	0.003430372276152025	0.036619224047922866	TIE1:FAS:ABCC8:TENM4:ERC1:AKAP3:HOXC11:WBP4:SIX6:MGA:CHRNB4:SMG1:CACNB1:BRCA1:PDE4A:FPR2:FSHR:ATP6V1B1:CYP2D6:GTSE1:NR1I2:LECT2:HCRTR2:ESR1:ABCB1:EXOC4:RB1CC1
Cancer_gene_neighborhoods	MORF_MAGEA8	259	19	0.004532540820056844	0.044584916574863624	LPHN2:FAS:ABCC8:TENM4:AKAP3:WBP4:SIX6:CHRNB4:OR10H3:FSHR:DAZL:NR1I2:SLC4A4:ISL1:LECT2:HCRTR2:ABCB1:EXOC4:RB1CC1
Cancer_gene_neighborhoods	MORF_CTBP1	168	14	0.004629698248438317	0.044584916574863624	TARDBP:DAP3:SSR2:SNRPE:SLC25A3:MRPS12:XPO1:FAM168B:NCL:CNBP:AP2M1:DIAPH1:VPS52:PUF60
Cancer_gene_neighborhoods	GCM_BMPR2	85	9	0.0046654705303718555	0.044584916574863624	MACF1:IPO7:CSRNP2:HERC2:FAM168B:GSK3B:NDFIP1:VPS52:ZFAND3
Cancer_gene_neighborhoods	GCM_HBP1	70	8	0.004698644603908344	0.044584916574863624	TARDBP:BTF3L4:C1orf52:KIAA0907:LINC00610:COMMD9:RBM25:RRN3:SLAIN2
Cancer_gene_neighborhoods	GCM_TPT1	70	8	0.004698644603908344	0.044584916574863624	SLC25A3:RPL19:RPL38:NCL:RPL32:RPL14:RPS18:RPL7
Cancer_gene_neighborhoods	MORF_PTPRR	102	10	0.005110747280173332	0.04643168273689389	TENM4:AKAP3:HOXC11:SIX6:FSHR:DAZL:LECT2:HCRTR2:ABCB1:EXOC4
Cancer_gene_neighborhoods	MORF_TPT1	102	10	0.005110747280173332	0.04643168273689389	RPL22:ZFPL1:FAU:RPL19:RPL38:RPL32:RPL37:RPS18:RPL7:RPL12

Cancer_gene_neighborhoods	GNF2_PRDX2	31	5	0.0056249634517049445	0.04856975577889039	TAL1:DCAF11:KEL:CA2:UBAC1
Cancer_gene_neighborhoods	GNF2_GLTSCR2	31	5	0.0056249634517049445	0.04856975577889039	FAU:RPL19:RPL38:ARHGEF18:RPL12
Cancer_gene_neighborhoods	GCM_MAP4K4	172	14	0.005687325032656954	0.04856975577889039	MACF1:HIPK1:IPO7:IPO5:HERC2:TBC1D10B:SPAG9:PITPNC1:TTYH2:NAPG:FAM168B:VPS52:ZFAND3:YWHAG
Cancer_gene_neighborhoods	GNF2_TST	104	10	0.005857633585642788	0.048632005989794405	HMGCS2:PKLR:APCS:HABP2:HPX:SDS:PCK2:ASGR2:CYP2D6:ASL
Cancer_gene_neighborhoods	GCM_PTPRD	58	7	0.005922398855900021	0.048632005989794405	MOAP1:NIPA1:HERC2:ARPP19:RABEP1:TTYH2:PKP4
Curated_gene_sets	ZWANG_TRANSIENTLY_UP_BY_2ND_EGF_PULSE_ONLY	1878	139	1.7320949510152234e-14	9.528254325534744e-11	ANGPTL7:TMEM82:PHC2:MACF1:MFSD2A:RIMKLA:FAM151A:DNAJB4:SSX2IP:DDAH1:AMPD1:LCE1F:LCE1C:LRRC71:APCS:HSPA6:SEC16B:FMOD:OR14K1:TUBB8:CDH23:OPALIN:TLL2:DUSP8:KRTAP5-3:KRTAP5-5:OR4C15:OR5R1:SCGB1D2:SAC3D1:NAALADL1:TMEM262:UCP2:POU2AF1:HSPA8:DDX25:AKAP3:SOX5:OR10P1:CLLU1OS:P2RX2:PCDH9:TNFSF13B:FOXG1:KCNK10:TDRD9:TRPM1:OTUD7A:SLC51B:MESDC1:PRSS21:ZNF48:ZNF771:TGFB1I1:SNAI3:RNF166:ASGR2:GLP2R:SLC5A10:MFAP4:STAC2:ZPBP2:RND2:ADAM11:GJC1:RNFT1:CACNG1:KCNJ2:SSTR2:ACTL9:MUC16:OR1M1:PDE4A:PODNL1:RLN3:CD97:OR10H3:NPHS1:CD79A:POU2F2:TMEM145:CBLC:C19orf68:TSKS:CTU1:HPCAL1:POTEE:RBMS1:CTDSP1:AC016757.3:ZNF343:TGM2:WFDC5:ZBP1:TPD52L2:UCKL1:OSBP2:SYNGR1:MCHR1:WBP2NL:KIAA1644:TYMP:CAND2:HDAC11:MYRIP:ZNF662:ZPLD1:PLCXD2:RAB43:RBP2:RBP1:TM4SF18:LIPH:C3orf65:MFI2:C4orf17:FCHSD1:PI16:GSTA2:CNR1:DACT2:THBS2:HOXA10:SRRM3:ABCB1:SPDYE3:NYAP1:PRKAR2B:GPR22:KLF14:KEL:COL22A1:GML:ADAMTSL1:SPATA31A4:SPATA31A7:OR1L3:RP11-203J24.9:ST6GALNAC6:PIP5KL1:OBP2A
Curated_gene_sets	GRAESSMANN_APOPTOSIS_BY_DOXORUBICIN_DN	1720	126	6.693343567394517e-13	1.006674666095127e-09	RPL22:CLSTN1:EXOSC10:SPEN:IQCC:C1orf123:LRRC8C:ABCD3:PTBP2:VAV3:WDR77:NRAS:CSDE1:PRUNE:FDPS:ASH1L:PMF1:TMEM79:SEC16B:ADCK3:RAB18:CUL2:STAMBPL1:ALDH18A1:GPAM:GRK5:APBB1:WEE1:SNX15:RCE1:PRKRIR:TENM4:ERC1:EMG1:EMP1:RASSF8:LETMD1:POLE:EBPL:KPNA3:PCK2:PRPF39:FANCM:FRMD6:DLGAP5:RBM25:NUMB:MGA:TCF12:MTFMT:RASL12:ZNF771:DCTPP1:NFATC3:PLA2G15:PRMT7:CYB5B:EPN2:GJC1:KPNB1:TTYH2:AZI1:TCF3:UBXN6:KEAP1:PAFAH1B3:KDELR1:OSR1:PUM2:XPO1:ZNF638:DUSP11:MTHFD2:ANAPC1:PKP4:RBMS1:CREB1:IGFBP2:IGFBP5:CTDSP1:PER2:TPD52L2:LSS:NAGA:CPT1B:RAD18:TRAK1:IFRD2:RASSF1:GSK3B:NDUFB4:SRPRB:SLAIN2:SLC4A4:CXCL6:NIPBL:KIF2A:DIMT1:IPO11:CETN3:HDAC3:NDFIP1:PDGFRB:RPP40:LRRC16A:NRM:TUBB:FLOT1:IER3:ZFAND3:PEX7:SOD2:MEPCE:TSC22D4:FAM3C:CEP41:COPG2:EXOC4:ZNF467:NCAPG2:PPP3CC:NUDCD1:KIAA0020:CNTLN:XPA:RBM18
Curated_gene_sets	KRIGE_RESPONSE_TO_TOSEDOSTAT_24HR_DN	1025	88	6.812007974971272e-13	1.006674666095127e-09	RNF207:ACOT7:TARDBP:UBIAD1:EBNA1BP2:KTI12:ABCD3:WDR77:ADAM15:BGLAP:ADCK3:B3GALNT2:FAS:ALDH18A1:IPO7:NCR3LG1:ZNHIT2:UBASH3B:SLC11A2:IKBIP:TMEM120B:DHX37:EBPL:DLEU1:KCTD12:TNFSF13B:DTD2:DCAF4:RBM25:AQR:MTFMT:DCTPP1:CIAPIN1:SLC7A6:TRAPPC2L:METTL16:SHPK:SHPK:DHX33:C17orf89:TAF4B:TCF3:CD320:PDE4A:DNAJB1:MRPS12:GRWD1:NOL10:BCL11A:BOLA3:PKP4:METTL21A:NCL:HES6:ESF1:RALGAPA2:LSS:SYNGR1:TRMU:SCO2:RAD18:IFRD2:NPRL2:IFT57:DBR1:ARMC8:ZAR1:EIF4E:TRMT10A:PTGER4:DIMT1:UTP15:RPP40:WDR46:PFDN6:SOD2:RNASET2:FGFR1OP:ARL4A:SEPT7:HSPB1:PRKAR2B:CREB3L2:ESYT2:RDH10:CA2:LY6E:SCRIB:KIAA0020
Curated_gene_sets	PEREZ_TP53_TARGETS	1185	97	7.319939400800778e-13	1.006674666095127e-09	HES2:FBLIM1:LDLRAP1:TXLNA:DDAH1:KCNC4:DENND2C:SIKE1:ANXA9:FAM63A:FLG:ASH1L:BGLAP:PEAR1:TOR3A:ADCK3:B3GALNT2:ECHDC3:GRK5:WEE1:SAC3D1:LINC01059:GRAMD1B:ENO2:LPCAT3:HOXC13:APAF1:NUAK1:ORAI1:ULK1:LMO7:ABHD13:KLHL28:ZFYVE1:KCNK10:MOAP1:ADAMTS7:KIAA1199:MESDC1:NPIPA1:ABCC11:LONP2:ESRP2:RNF166:FAM83G:SLC47A1:TTYH2:TNRC6C:TTC39C:INSR:S1PR5:PODNL1:DEDD2:CEACAM1:ZNF765:GREB1:FAM49A:AFF3:MERTK:HES6:ZNF343:WFDC5:ZNFX1:DNAJC5:UCKL1:APOL2:APOBEC3B:MCHR1:KIAA1644:TRAK1:CYB561D2:CACNA2D2:XXcos-LUCA11.4:ZNF654:C3orf55:KLHL6:DVL3:UBE2QL1:ANKRA2:MARCH3:MEGF10:ZFP62:SLC22A23:C4A:CDKN1A:MAP7:FGFR1OP:HOXA11:LAT2:ABCB1:GNG11:CREB3L2:FAM167A:COL22A1:NRBP2:S1PR3:FAM102A:SLC25A25:CEL
Curated_gene_sets	NUYTTEN_NIPP1_TARGETS_DN	846	76	2.988648506760995e-12	3.2881110871384467e-09	PHC2:SSX2IP:SLC16A4:HIPK1:TRIM33:SIKE1:ARNT:PHLDA3:NUCKS1:TUBB8:LARP4B:RAB18:REEP3:SMPD1:EHF:TM7SF2:UCP2:AASDHPPT:ERC1:GPRC5A:ULK1:IPO5:ABHD13:COCH:ERO1L:UBR7:ARPP19:ANP32A:KIAA1199:DCTPP1:NFATC3:KPNB1:RPS6KB1:PSMD12:KCNJ2:TTYH2:UBALD2:RALBP1:IMPA2:TCF3:TUBB4A:PDE4A:KEAP1:DDX39A:GIPC1:CEACAM1:PUM2:NCOA1:

						PPP1CB:TPRKB:DSTN:RALGAPA2:SLPI:SLC2A4RG:TPD52L2:OSBP2:TTC38:ENTPD3:GNAI2:C3orf38:SLAIN2:KIF2A:DIMT1:SLC12A2:HOXA10:HOXA13:YWHAG:CEP41:DENND2A:ESYT2:RB1CC1:RDH10:CALB1:SLC35D2:RBM18:TUBB4B
Curated_gene_sets	HESSON_TUMOR_SUPPRESSOR_CLUSTER_3P21_3	8	8	3.9306915519575485e-12	3.603789037886412e-09	HYAL2:TUSC2:RASSF1:ZMYND10:NPRL2:CYB561D2:TMEM115:CACNA2D2:XXcos-LUCA11.4
Curated_gene_sets	PUJANA_ATM_PCC_NETWORK	1381	105	7.260831968013364e-12	5.705976665148788e-09	RPL22:EXOSC10:C1orf63:PTP4A2:CDC20:ACOT11:NRAS:SIKE1:CTSS:ARNT:CLK2:DAP3:KIAA0907:FCGR2A:SNRPE:LARP4B:CUL2:LRIT1:FAS:CTR9:SNX15:TMEM262:UCP2:PRKRIR:POU2AF1:HSPA8:OPCML:KCNA1:PTPN6:EMG1:RASSF8:TFCP2:HOXC11:NCKAP1L:RAP1B:POLE:GTF3A:LCP1:DLEU1:RNASEH2B:TGDS:ERCC5:PSME1:SCFD1:SIX6:GTF2A1:ANP32A:CORO1A:CIAPIN1:NFATC3:SLC7A6:RABEP1:PRPSAP2:HNF1B:RPL19:BRCA1:NBR1:UTP18:RPS6KB1:FSCN2:RALBP1:TAF4B:TCF3:CHAF1A:SAFB:ARHGEF18:C19orf57:DDX39A:PKN1:CD79A:POU2F2:PAFAH1B3:PEX13:XPO1:DUSP11:MOB1A:MTHFD2:NCL:PCNA:BFSP1:DDX27:LSS:TAB1:FBLN1:GTSE1:CHKB:RPL14:ACKR2:RASSF1:NPRL2:CNBP:RPL37:DIMT1:MARCH3:DIAPH1:ADAM19:PFDN6:ZNF117:CD36:BET1:PPP3CC:RB1CC1:LYN:NSMAF:RPL7
Curated_gene_sets	CHEN_METABOLIC_SYNDROM_NETWORK	1211	95	1.3919300335524534e-11	9.571258893215058e-09	RNF207:CDC42:LDLRAP1:ELOVL1:CDCP2:RPE65:BCL10:CTSS:CTSK:PRUNE:LMNA:FCGR2A:ABL2:SOAT1:FMOD:NID1:C10orf54:PSAP:STAMBPL1:ALDH18A1:SLC43A1:UBASH3B:SPSB2:PTPN6:RASSF8:SLC11A2:NCKAP1L:POSTN:LHFP:LCP1:KIAA0226L:ARL11:RNASEH2B:TNFSF13B:FITM1:FRMD6:MYO5A:CORO1A:TGFB1I1:SLC7A6:CLEC10A:MAPK7:VAT1:TMEM106A:SSTR2:MYL12A:MYL12B:TTC39C:DENND1C:TUBB4A:S1PR5:CD97:PRODH2:CD33:HPCAL1:PLB1:FSHR:MTHFD2:IL1RL1:PLEKHB2:MZT2A:IGFBP2:SLC11A1:PCNA:SLPI:APOL2:SYNGR1:CYP2D6:PLXNB2:TGFBR2:RASSF1:LRIG1:CD200R1:ADPRH:KLHL6:AP2M1:MFI2:PLAC8:DROSHA:ACTBL2:C5orf30:TIFAB:PDGFRB:TUBB:THBS2:TPST1:LAT2:CD36:LYN:CA2:NRBP2:LRSAM1:ST6GALNAC4:PIP5KL1:TOR4A
Curated_gene_sets	PILON_KLF1_TARGETS_DN	1984	134	3.5732857758115494e-11	2.1840716725265925e-08	ACOT7:MTOR:CDC42:LDLRAP1:PTP4A2:TXLNA:IQCC:EBNA1BP2:ELOVL1:TXNDC12:BTF3L4:C1orf123:BCL10:EXTL2:NRAS:PHGDH:CKS1B:SEMA4A:FCGR2A:F5:SOAT1:NUCKS1:REEP3:C10orf54:STAMBPL1:ZNF143:WEE1:KCNJ11:SYVN1:PHB2:NCKAP1L:RAP1B:APAF1:UHRF1BP1L:CIT:BRI3BP:POLE:ANKLE2:MTMR6:WBP4:LCP1:KPNA3:RNASEH2B:KCTD12:IPO5:KDELC1:ABHD13:HEATR5A:FBXO33:FKBP3:GTF2A1:UBR7:AQR:JMJD7-PLA2G4B:ARPP19:RFX7:ANP32A:CORO1A:NFATC3:PLA2G15:RNF166:DHX33:NPEPPS:KPNB1:TRIM25:TNRC6C:MYL12B:PPP4R1:MPPE1:IMPA2:ZNF236:MBP:SAFB2:SAFB:ARHGEF18:DDX39A:MAST3:CEACAM1:GRWD1:CD33:FPR2:PUM2:PPP1CB:BCL11A:PEX13:XPO1:DUSP11:MOB1A:IL18RAP:ANAPC1:FAM168B:RBMS1:RPE:PANK2:C21orf91:RAD18:RPL14:CGGBP1:GSK3B:CNBP:DBR1:ARMC8:COPB2:RASA2:KLHL6:STIM2:COMMD8:SLAIN2:PLAC8:DCTD:DROSHA:CKS1B:UTP15:CETN3:C5orf30:ZNF608:TIFAB:ARAP3:RPP40:RAB44:C6orf89:PTP4A1:MAP7:PEX7:HECA:SYNE1:RNASET2:FAM3C:ESYT2:NSMAF:TERF1:LY6E:KIAA0020:TSTD2:RBM18
Curated_gene_sets	BLALOCK_ALZHEIMERS_DISEASE_UP	1662	117	5.7749329957746485e-11	3.1767906409756344e-08	SPEN:ZBTB17:LDLRAP1:PTP4A2:TXLNA:PHC2:MACF1:DPH5:RBM15:CSDE1:CTSS:SCAMP3:FDPS:SSR2:LMNA:BGLAP:PHLDA3:NUCKS1:ADCK3:NID1:GRK5:ZFPL1:PC:TPI1:C1S:EMP1:METTL7A:LGR5:DAO:DLEU1:PCDH9:NUMB:KCNK10:RASL12:PKD1:CD2BP2:TGFB1I1:N4BP1:NFATC3:SLC7A6:TRPV1:RABEP1:VAT1:BRCA1:NPEPPS:KPNB1:SPAG9:TUBD1:HELZ:PITPNC1:RALBP1:SLC14A1:SERPINB4:ZNF236:MBP:TCF3:INSR:ARHGEF18:DDX39A:PKN1:GIPC1:ERF:BCAM:KCNJ14:ZNF83:MYCN:MOB1A:RBMS1:IGFBP5:SLC11A1:CTDSP1:PER2:SNED1:DDX27:SLC2A4RG:TPD52L2:C21orf91:LSS:DGCR6:APOL2:NAGA:FBLN1:GTSE1:PLXNB2:TYMP:CPT1B:VGLL4:IFRD2:FAM208A:CGGBP1:GTPBP8:GSK3B:A4GNT:ARMC8:MRAS:ATP10D:SLC4A4:AFM:NIPBL:PDGFRB:MGAT1:C4A:TAPBP:ZFAND3:MAP7:TNFAIP3:SOD2:RNASET2:ITGB8:LAT2:PILRA:TSC22D4:CALD1:GIMAP5:GEM:SCRIB:ST6GALNAC4
Curated_gene_sets	GEORGES_TARGETS_OF_MIR192_AND_MIR215	890	75	8.511421028476702e-11	4.256484279786394e-08	CLSTN1:TARDBP:UBIAD1:IQCC:PHC2:MACF1:RIMKLA:CDC20:ELOVL1:STIL:BTF3L4:DNAJB4:SAMD13:LRRC8C:PTBP2:EXTL2:LAMTOR5:HCN3:PMF1:TOR3A:SOAT1:ASPM:NUCKS1:ZMYND11:MCM10:STAMBPL1:ASRGL1:AASDHPPT:UBASH3B:CDON:RASSF8:CIT:LHFP:KCTD12:GPR180:BIVM:TNFSF13B:G2E3:COCH:FANCM:GPR137C:DLGAP5:MOAP1:NIPA1:HERC2:TRPV1:HNF1B:BRCA1:TMEM106A:SPAG9:RPS6KB1:PITPNC1:ZNF611:PPP1CB:KIAA1841:XPO1:PKP4:FBXO36:HES6:C21orf91:SYNGR1:LRIG1:DHFRL1:NPHP3:FNDC3B:MFI2:CCNO:DIMT1:C5orf30:PPP1R18:MDC1:FGFR1OP:HOXA10:HOXA13:KHDRBS3
Curated_gene_sets	DODD_NASOPHARYNGEAL_CARCINOMA_UP	1762	121	1.2527629017544878e-10	5.448802361419523e-08	NBL1:LDLRAP1:TMEM125:TSPAN1:PDZK1IP1:F3:HIPK1:DENND2C:ARNT:ANXA9:FAM63A:PKLR:LRRC71:FCRLA:C10orf54:RBM20:SCGB2A1:ASRGL1:TM7SF2:DNAJB13:UCP2:POU2AF1:BTG4:C11orf88:TTC12:C11orf71:LRRC23:GPRC5A:MORN3:TNFRS

						F19:PCDH9:LMO7:PPP1R36:MAP3K9:NUMB:C14orf142:TRPM1:C15orf26:SH3GL3:SMPD3:SPG7:DNAH9:EPN2:B9D1:KCNJ16:DNAI2:KIF19:MYL12B:IMPA2:TTC39C:ASXL3:PHLPP1:SERPINB4:MBP:PEX11G:MUC16:AP1M2:CNFN:CXCL17:TPO:C2orf81:WDR54:AFF3:CCDC74A:DAW1:FBXO36:PER2:SNED1:RALGAPA2:MMP24:PI3:SLPI:SLC37A1:SYNGR1:SMDT1:TTC38:DENND6B:ODF3B:CAND2:EFHB:CMTM8:ENTPD3:TRAK1:ZNF662:TUSC2:RASSF1:ZMYND10:CYB561D2:XXcos-LUCA11.4:FOXP1:IFT57:CD200R1:RAB43:NEK11:DZIP1L:KLHL6:MAP3K13:LIPH:SLC34A2:SLAIN2:CXCL1:PLAC8:CCNO:C5orf63:B3GALT4:C6orf89:GSTA1:SYNE1:ASL:CLDN3:ABCB1:ZCWPW1:BLK:LYN:RDH10:LRRC6:PTPN3:RP11-203J24.9:ST6GALNAC6:FAM102A:TUBB4B:FAM166A:TOR4A
Curated_gene_sets	DANG_BOUND_BY_MYC	1059	84	1.287664619132045e-10	5.448802361419523e-08	RPL22:MTOR:ELOVL1:LAMTOR5:NRAS:CSDE1:SCAMP3:DAP3:LMNA:PSEN2:NID1:PSAP:TLL2:PEX16:SLC43A1:ZFPL1:UCP3:PRKRIR:HSPA8:ENO2:PTPN6:PHB2:EMP1:SLC25A3:PRKAB1:KPNA3:DLEU1:ERO1L:AQR:ARPP19:TCF12:PLA2G15:SHPK:SHPK:MAPK7:CACNB1:RPL19:NBR1:TRIM25:RPS6KB1:RNFT1:IMPA2:INSR:KEAP1:CD97:NFKBIB:ERF:PPP1CB:CREB1:CTDSP1:NCL:PCNA:SLC2A4RG:DGCR6:NAGA:NDUFA6:TYMP:RPL32:IFRD2:TUSC2:NPRL2:CYB561D2:XXcos-LUCA11.4:GSK3B:NDUFB4:MFI2:EIF4E:TERT:RPL37:SLC12A2:HDAC3:VPS52:WDR46:PFDN6:RGL2:PTP4A1:RNASET2:RFC2:BLK:ASH2L:UBQLN1:PAPPA:ST6GALNAC4:DPM2:GTF3C5:UBAC1
Curated_gene_sets	DIAZ_CHRONIC_MEYLOGENOUS_LEUKEMIA_UP	1397	102	1.453421610480902e-10	5.710908770896744e-08	ICMT:ACOT7:TARDBP:TMEM50A:PTP4A2:TIE1:TAL1:TM2D1:ABCD3:VAV3:CSDE1:CERS2:FDPS:LMNA:FCGR2A:ZMYND11:FAS:ALDH18A1:IPO7:SLC43A1:MRPL49:PRKRIR:AASDHPPT:RBM7:TPI1:PTPN6:PHB2:LPCAT3:TFCP2:PRKAB1:SLC25A15:KPNA3:DLEU1:IPO5:PCK2:SCFD1:FKBP3:ARPP19:TCF12:ANP32A:NOMO1:RRN3:POLR2C:NFATC3:PRPSAP2:BRCA1:NBR1:NPEPPS:KPNB1:RNFT1:PSMD12:RALBP1:PPP4R1:KEAP1:PUM2:NCOA1:XPO1:DUSP11:MTHFD2:ANAPC1:PKP4:RBMS1:COPS8:DSTN:RPN2:TGM2:LSS:RTCB:EIF1B:RASSF1:CGGBP1:CNBP:ATP2C1:COPB2:AP2M1:EIF4E:PLA2G12A:DCTD:KIF2A:CETN3:HDAC3:NDFIP1:C4A:MAP7:SOD2:HOXA10:SEPT7:ASL:CD36:PILRB:PRKAR2B:BCAP29:MEST:KEL:ASH2L:STAR:LYN:NSMAF:TERF1:LY6E:PBX3:UBAC1
Curated_gene_sets	KRIGE_RESPONSE_TO_TOSEDOSTAT_6HR_DN	931	75	6.521503774204708e-10	2.3916528174600063e-07	RNF207:ACOT7:TARDBP:UBIAD1:EBNA1BP2:ABCD3:WDR77:TNFAIP8L2:ADAM15:ADCK3:B3GALNT2:ALDH18A1:IPO7:NCR3LG1:ZNHIT2:UBASH3B:SLC11A2:IKBIP:TMEM120B:DHX37:EBPL:DLEU1:KCTD12:TNFSF13B:DTD2:DCAF4:RBM25:AQR:MTFMT:DCTPP1:CIAPIN1:SLC7A6:TRAPPC2L:METTL16:DHX33:C17orf89:TCF3:PDE4A:DNAJB1:MRPS12:GRWD1:NOL10:NCOA1:KIAA1841:BOLA3:IL18R1:METTL21A:NCL:HES6:ESF1:RALGAPA2:SYNGR1:TRMU:SCO2:RAD18:NPRL2:IFT57:DBR1:ZAR1:EIF4E:TRMT10A:DIMT1:WDR46:PFDN6:SOD2:RNASET2:FGFR1OP:SEPT7:HSPB1:PRKAR2B:CREB3L2:RDH10:CA2:LY6E:SCRIB
Curated_gene_sets	PUJANA_BRCA1_PCC_NETWORK	1617	111	7.87427830512692e-10	2.7072753097814494e-07	RPL22:TARDBP:EXOSC10:MTOR:PTP4A2:EBNA1BP2:CDC20:STIL:LAMTOR5:WDR77:BCAS2:NRAS:SIKE1:ARNT:CKS1B:FDPS:DAP3:KIAA0907:SNRPE:LARP4B:CUL2:FAS:IPO7:WEE1:CTR9:SNX15:SAC3D1:UCP2:PRKRIR:POU2AF1:HSPA8:KCNA1:PHB2:RASSF8:NCKAP1L:RAP1B:POLE:ANKLE2:GTF3A:LCP1:DLEU1:RNASEH2B:UCHL3:TGDS:IPO5:NRL:SCFD1:DLGAP5:GTF2A1:ANP32A:CORO1A:CIAPIN1:NFATC3:SLC7A6:CYB5B:RABEP1:PRPSAP2:RPL19:BRCA1:KPNB1:UTP18:DGKE:RPS6KB1:TAF4B:TCF3:CHAF1A:SAFB:DDX39A:PKN1:IL12RB1:CD79A:PAFAH1B3:XPO1:DUSP11:MOB1A:MTHFD2:NCL:PCNA:BFSP1:DDX27:FBLN1:GTSE1:RPL14:ACKR2:IFRD2:RASSF1:NR1I2:CNBP:RPL37:SKIV2L2:KIF2A:DIMT1:CKS1B:CETN3:DIAPH1:HDAC3:ADAM19:RPP40:PFDN6:FGFR1OP:RFC2:BET1:BLK:PPP3CC:ASH2L:RB1CC1:NSMAF:RPL7:KIAA0020:SPATA31A4:SPATA31A7:RPL12:ST6GALNAC4
Curated_gene_sets	BYSTRYKH_HEMATOPOIESIS_STEM_CELL_QTL_TRANS	762	65	9.607716916896374e-10	3.1089441623439385e-07	MTOR:TIE1:CDC20:GJA5:SETDB1:CERS2:FAM63A:LMNA:UHMK1:TOR3A:CSRP1:NUCKS1:CUL2:PSAP:SMPD1:YAP1:GTF3A:FANCM:ERO1L:TRPM1:MYO5A:ARPP19:PDXDC1:RRN3:SMG1:POLR2C:MAPK7:NPEPPS:RPS6KB1:IMPA2:MBP:TCF3:CD320:GIPC1:CD79A:GSK3A:LIPE:CEACAM1:KDELR1:GRIN2D:TPO:ALK:RTKN:IL1RL1:IL18R1:IGFBP2:IGFBP5:CDS2:BPIFA2:SYNGR1:FBLN1:PLXNB2:UBA5:SLC4A4:CDH6:SNCB:EDN1:IER3:WDR46:RGL2:ESR1:YWHAG:STAR:RBM18:NACC2
Curated_gene_sets	MARSON_BOUND_BY_FOXP3_STIMULATED	977	77	1.0328187338044182e-09	3.156408808143391e-07	C1orf63:TMEM50A:TCEANC2:DNAJB4:SAMD13:BCL10:GOLPH3L:SETDB1:CERS2:ANXA9:FAM63A:PRUNE:SCNM1:LYSMD1:ASH1L:DAP3:KIAA0907:SEMA4A:SMG5:TMEM79:NSUN6:ARL5B-AS1:ARL5B:REEP3:HECTD2:UCP2:AASDHPPT:POU2AF1:CRTAM:SLC11A2:LETMD1:IKBIP:APAF1:UHRF1BP1L:TNFRSF19:SLC25A15:ARL11:KPNA3:ABHD13:FRMD6:SIX6:PSEN1:CPSF2:TMEM251:NFATC3:SNAI3:ZPBP2:BRCA1:NBR1:KPNB1:SPAG9:UBALD2:PPP4R1:DDX39A:PTGER1:PPP1CB:XPO1:CREB1:CHMP4B:THUMPD3:DBR1:ARMC8:LIPH:ATP10D:EIF4E:PTGER4:ACTBL2:NDFIP1:FLOT1:IER3:TCTE3:ERMARD:MPLKIP:GPR22:GIMAP5:LRRC6:TMEM71:XPA
Curated_gene_sets	NUYTTEN_EZH2_TARGETS_DN	1017	79	1.2075946871683974e-09	3.496304407428081e-07	CDC42:CDC20:STIL:TCEANC2:HOOK1:SSX2IP:DDAH1:DPH5:SLC16A4:PHGDH:PRUNE:CKS1B:PMF1:BGLAP:ASPM:ADCK3:LARP4B:MCM10:REEP3:ALDH18A1:GPAM:WEE1:ASRGL1:SAC3D1:CDCA5:C12orf57:METTL7A:BRI3BP:TNFRSF19:MTMR6:MTIF3:C

						OMMD6:IPO5:PCK2:COCH:DLGAP5:UBR7:ARPP19:ANP32A:TGFB1I1:CYB5B:SPG7:DHX33:BRCA1:NBR1:GJC1:UTP18:PITPNC1:AZI1:IMPA2:PHLPP1:CHAF1A:CD320:WDR54:BFSP1:SLC2A4RG:CYB5R3:GTSE1:RAD18:CMTM8:CGGBP1:ATP2C1:SRPRB:MAN2B2:KLHL2:CKS1B:ANKRA2:CETN3:SLC12A2:MDC1:FLOT1:RPS18:MAP7:AIG1:FGFR1OP:ERMARD:LAT2:PPP1R35:CEP41:NUDCD1
Curated_gene_sets	NAKAMURA_TUMOR_ZONE_PERIPHERAL_VS_CENTRAL_DN	632	56	3.6623803802100757e-09	1.0073377235767814e-06	RPL22:MACF1:TSPAN1:HSPA6:ZNF496:EHF:ENO2:C1S:LETMD1:IKBIP:FNDC3A:COMMD6:KLHL28:ERO1L:GOLGA8G:ANP32A:KIAA1199:MESDC2:LONP2:NBR1:SERPINB4:INSR:PODNL1:DNAJB1:BCL11A:IL1RL1:IGFBP5:RALGAPA2:CHMP4B:PI3:SLPI:DNAJC5:ARFGAP3:PPARA:PLXNB2:CPT1B:LRIG1:RAB43:ACAD11:NPHP3:FNDC3B:MAP3K13:CFI:RPL37:CARD6:SLC22A23:RGL2:TAPBP:PTP4A1:SOD2:ITGB8:PILRB:CREB3L2:ESYT2:RB1CC1:CALB1
Curated_gene_sets	REACTOME_POST_TRANSLATIONAL_PROTEIN_MODIFICATION	1427	99	3.984067672995674e-09	1.0436360128166287e-06	ICMT:PTP4A2:PHC2:CDC20:BCL10:DPH5:BGLAP:F5:SEC16B:B3GALNT2:TUBB8:RAB18:CUL2:STAMBPL1:CTR9:LINC00610:COMMD9:GYLTL1B:SYVN1:RCE1:HSPA8:OPCML:USP5:SPSB2:FBXW8:COMMD6:UCHL3:LMO7:DCAF11:PSME1:SCFD1:MIA2:CTAGE5:DCAF4:HERC2:OTUD7A:KBTBD13:ADAMTS7:PRSS21:TRAPPC2L:DERL2:ASGR2:BRCA1:TRIM25:PSMD12:NAPG:ST8SIA5:SAFB:TUBB4A:MUC16:KEAP1:CHST8:FBXO17:KDELR1:PUM2:NCOA1:PEX13:DCTN1:METTL21A:IGFBP5:TTLL4:COPS8:PCNA:RPN2:USP25:APOL1:TAB1:ARFGAP3:PPARA:ARSA:RAD18:TGFBR2:TMEM115:PROS1:NR1I2:RAB43:RAB6B:A4GNT:COPB2:SENP2:MFI2:COMMD8:AREG:AREGB:KLHL2:MGAT1:HLA-A:MDC1:MUC21:C4A:RAB44:TNFAIP3:ESR1:THBS2:BET1:COPG2:LY6E:ADAMTSL1:RP11-203J24.9:ST6GALNAC6:ST6GALNAC4:DPM2:TUBB4B
Curated_gene_sets	BENPORATH_MYC_MAX_TARGETS	774	64	4.375252025713915e-09	1.0940118815205566e-06	MTOR:ELOVL1:LAMTOR5:TRIM33:CSDE1:SCAMP3:DAP3:ALDH18A1:TLL2:PEX16:SLC43A1:SNX15:ZFPL1:PRKRIR:ENO2:PTPN6:PHB2:PRKAB1:KPNA3:DLEU1:IPO5:DCAF11:ERO1L:JMJD7-PLA2G4B:ARPP19:TCF12:PLA2G15:SHPK:SHPK:MAPK7:CACNB1:RPL19:NBR1:TRIM25:RPS6KB1:RNFT1:KEAP1:CD97:NFKBIB:PPP1CB:CREB1:NCL:PCNA:DGCR6:NAGA:NDUFA6:RPL32:RASSF1:NPRL2:CYB561D2:XXcos-LUCA11.4:NDUFB4:RPL37:SLC12A2:HLA-A:VPS52:WDR46:RGL2:PTP4A1:RNASET2:BLK:ASH2L:UBQLN1:ST6GALNAC4:DPM2:GTF3C5
Curated_gene_sets	LASTOWSKA_NEUROBLASTOMA_COPY_NUMBER_DN	795	65	5.142931649174588e-09	1.2300550870482353e-06	ICMT:GPR153:ACOT7:CLSTN1:EXOSC10:MTOR:UBIAD1:FBLIM1:SPEN:ZBTB17:CDC42:C1orf63:TMEM50A:RHCE:LDLRAP1:PTP4A2:TXLNA:CCDC28B:TRIM62:ZNF362:PHC2:MACF1:EBNA1BP2:ELOVL1:MED8:SZT2:TXNDC12:BTF3L4:C1orf123:TM2D1:SSX2IP:C1orf52:BCL10:REEP3:STAMBPL1:HECTD2:ZFYVE27:GPAM:TRUB1:ASRGL1:DGAT2:PRKRIR:RP11-111M22.2:TENM4:AASDHPPT:FDX1:C11orf71:RBM7:HSPA8:CDON:RAD18:THUMPD3:VGLL4:RPL32:CMTM8:MYRIP:EIF1B:TRAK1:KRBOX1:KRBOX1:HYAL2:TUSC2:TMEM115:CCDC66:MRFAP1:SLAIN2
Curated_gene_sets	BERENJENO_TRANSFORMED_BY_RHOA_UP	540	50	6.028619872817342e-09	1.3818099133486749e-06	ACOT7:TARDBP:PHC2:EBNA1BP2:CDC20:LRRC8C:CKS1B:PMF1:ASPM:NID1:MCM10:FAM171A1:IPO7:CDCA5:TPI1:EMG1:SLC11A2:IPO5:DLGAP5:RRN3:BOLA2B:DCTPP1:BRCA1:KPNB1:UTP18:GRWD1:MYCN:XPO1:MTHFD2:IL1RL1:PKP4:PCNA:ESF1:EIF6:GTSE1:IFRD2:MRPS22:SLC4A4:CXCL6:PTGER4:IPO11:CKS1B:DIAPH1:IER3:PFDN6:CDKN1A:TNFAIP3:SOD2:KIAA0020:KLF4:PBX3
Curated_gene_sets	YOSHIMURA_MAPK8_TARGETS_UP	1171	85	6.8803777942855335e-09	1.5139583298545889e-06	RHCE:C1orf210:HOOK1:LPHN2:KCNC4:AMPD1:HMGCS2:GJA5:CTSS:ARNT:TMEM79:FCGR2A:TACR2:GPAM:APBB1:KCNJ11:ABCC8:SCGB1D2:NAALADL1:UCP2:UCP3:KCNA1:PDE1B:SDS:ARL11:PSME1:FOXG1:MAP3K9:PSEN1:CHRNB4:ACAN:CALB2:TRPV1:DNAH9:PRPSAP2:RNF112:HNF1B:CACNG1:KCNJ16:KCNJ2:SSTR2:MYL12B:RALBP1:TCF3:PDE4A:PTGER1:SYCN:GRIN2D:MYCN:SLC9A4:SCN3A:IGFBP2:BFSP1:BPIFA2:KCNS1:SEMG1:SEMG2:MC3R:SYNGR1:CYP2D6:PPARA:CHKB:MAPK8IP2:RAD18:RPL32:HYAL2:GSK3B:MRAS:RBP2:CXCL6:SLC6A19:PTGER4:ISL1:OCLN:IER3:CDKN1A:DACT2:CLDN3:HSPB1:GNG11:PIP:NCAPG2:CA2:CDH17:PTPN3
Curated_gene_sets	MILI_PSEUDOPODIA_HAPTOTAXIS_DN	665	57	8.735968487902083e-09	1.8483293327672831e-06	ICMT:CLSTN1:EXOSC10:UBIAD1:CDC20:ELOVL1:LPHN2:CERS2:SCAMP3:C1orf85:PEAR1:SOAT1:NID1:FAM171A1:REEP3:ZFYVE27:SMPD1:CTR9:SYVN1:PHB2:GPRC5A:SLC11A2:ORAI1:ANKLE2:LHFP:KDELC1:SCFD1:PSEN1:PLA2G15:SLC7A6:SMPD3:ANKRD11:DERL2:GJC1:TTLL4:CDS2:PLXNB2:LMF2:HYAL2:CYB561D2:TMEM115:XXcos-LUCA11.4:ATP10D:CXCL6:C5orf22:PPAP2A:PCDHGC3:PDGFRB:MGAT1:SLC22A23:NRM:CREB3L2:ESYT2:LY6E:PUF60:S1PR3:RPL12:ST6GALNAC4

Curated_gene_sets	NIKOLSKY_BREAST_CANCER_17P11_AMPLICON	10	7	1.1404898701315671e-08	2.323642509479167e-06	EPN2:B9D1:MAPK7:MFAP4:RNF112:SLC47A1:SLC47A2
Curated_gene_sets	HAN_SATB1_TARGETS_UP	421	42	1.1829659018608667e-08	2.3241055093345097e-06	DNAJB4:DDAH1:F3:WDR77:REEP3:GRK5:UCP2:ENO2:PCDH9:STXBP6:KIAA1199:MBP:AP1M2:IL18R1:RND3:IGFBP5:CDS2:PI3:SLPI:LSS:APOL2:SYNGR1:NAGA:NDUFA6:CYB5R3:ARFGAP3:TTC38:CELSR1:PLXNB2:NCAPH2:SCO2:ARSA:HYAL3:ARMC8:FNDC3B:IL8:CXCL1:CDH6:ASL:CALD1:PAEP:NACC2
Curated_gene_sets	KIM_WT1_TARGETS_DN	471	45	1.3462676926863135e-08	2.5537305439542798e-06	C1orf63:CDC20:ELOVL1:DNAJB4:ABCD3:LAMTOR5:GOLPH3L:KIAA0907:SOAT1:ASPM:CREM:GRK5:CTR9:SAC3D1:AASDHPPT:APAF1:NUAK1:TMEM120B:DLEU1:DLGAP5:RBM25:GOLGA8G:NFATC3:PRMT7:DERL2:NBR1:TRIM25:DNAJB1:TECR:PKP4:GTSE1:TRMU:CPT1B:ATP10D:PLA2G12A:KLHL2:CETN3:GTF2H5:SOD2:ARL4A:RFC2:PILRB:MEPCE:ZNF34:CHMP5
Curated_gene_sets	BRUINS_UVC_RESPONSE_VIA_TP53_GROUP_A	859	67	1.9035701427366493e-08	3.3981004429223137e-06	CCDC28B:PDZK1IP1:DNAJB4:ADAM15:MSTO1:APCS:SEC16B:CSRP1:C10orf54:ACTA2:HPX:KCNJ11:DBX1:UBE2L6:UCP2:TENM4:DDX25:RASSF8:DCAF11:DTD2:ERO1L:MYO5A:KIAA1199:ACAN:PDXDC1:SLX1A:GLP2R:DGKE:HELZ:KCNJ2:SSTR2:MYL12A:TUBB4A:MUC16:AFF3:RND3:TM4SF20:SLPI:CYP2D6:KIAA1644:CELSR1:CACNA2D2:GP9:SRPRB:RASA2:XRN1:STK32B:SLC4A4:TMEM161B:LECT2:PRELID2:TTC1:C5orf47:IER3:TAPBP:PTP4A1:MPLKIP:CLDN3:CLDN4:LAT2:YWHAG:ZNF467:NRBP2:FOXB2:IPPK:RBM18:CEL
Curated_gene_sets	GRAESSMANN_APOPTOSIS_BY_DOXORUBICIN_UP	1121	81	1.9149448051370972e-08	3.3981004429223137e-06	ICMT:LDLRAP1:TM2D1:SSX2IP:RBM15:PHGDH:ADAM15:FAM189B:RUSC1:UHMK1:TOR3A:PHLDA3:CREM:FAS:CTR9:SAC3D1:SYVN1:DGAT2:ENO2:C1S:PTHLH:PDE1B:IKBIP:APAF1:ULK1:KCTD12:ERCC5:FBXO33:DCAF4:GTF2A1:MOAP1:MAPKBP1:MYO5A:ADAMTS7:CORO1A:SIAH1:ESRP2:STAC2:IFI35:UBALD2:ENTHD2:DDX39A:PTGER1:DNAJB1:NCOA1:RPE:HES6:DSTN:TGM2:ZBP1:C21orf91:APOL2:GTSE1:PLXNB2:KRBOX1:KRBOX1:NAT6:HYAL1:HYAL2:RAB43:ACAD11:DBR1:PSAPL1:AREG:AREGB:PLA2G12A:PTGER4:SLC12A2:CPEB4:WDR46:TAPBP:CDKN1A:PTP4A1:IRAK1BP1:EXOC4:RDH10:GEM:ZNF34:IPPK:ST6GALNAC4:FAM102A:SLC25A25:TUBB4B
Curated_gene_sets	MONNIER_POSTRADIATION_TUMOR_ESCAPE_UP	397	40	1.9915592038875692e-08	3.4236147439329745e-06	TARDBP:UBIAD1:TXLNA:SSX2IP:HIPK1:GON4L:KIAA0907:SMG5:ZFYVE27:ASRGL1:PC:YAP1:FDX1:SLC11A2:LETMD1:LHFP:RBM25:SLC7A6:DHX33:FAM83G:VAT1:KPNB1:NPLOC4:TTC39C:SLC14A1:GRWD1:IL1RL1:IL18R1:RASSF1:LRIG1:SENP2:CXCL6:C5orf22:NIPBL:SLC12A2:SLC22A23:WDR46:PRKAR2B:ASH2L:FAM102A
Curated_gene_sets	SCHLOSSER_SERUM_RESPONSE_DN	700	58	2.1606634571962427e-08	3.6017605084959186e-06	C1orf63:PTP4A2:SLC16A4:LAMTOR5:HIPK1:PHGDH:DAP3:NHLH1:FCGR2A:SNRPE:ZMYND11:UBE2L6:POU2AF1:ATN1:NCKAP1L:RAP1B:SLC25A3:APAF1:FAM179B:TMEM251:NIPA2:TCF12:ANP32A:CYB5B:PRPSAP2:PSMD12:MYL12A:RALBP1:MPPE1:PHLPP1:TCF3:INSR:MEGF8:BCAM:DUSP11:PKP4:IGFBP5:COPS8:DSTN:RPN2:TGM2:RTCB:FBLN1:FAM208A:LRIG1:KCNAB1:CXCL1:COQ2:ADAM19:PTP4A1:BET1:PRKAR2B:FAM3C:PPP3CC:RB1CC1:TERF1:XPA:KLF4
Curated_gene_sets	REACTOME_METABOLISM_OF_RNA	668	56	2.5346066225308102e-08	4.100844420747643e-06	RPL22:EXOSC10:EBNA1BP2:WDR77:BCAS2:SMG5:SNRPE:NSUN6:ARL5B-AS1:FAU:HSPA8:EMG1:DDX47:DHX37:WBP4:PSME1:CPSF2:AQR:ANP32A:RPL3L:SMG1:CD2BP2:POLR2C:RPL19:UTP18:PSMD12:RPL38:DDX39A:CTU1:TRMT61B:XPO1:TPRKB:NCL:RTCB:APOBEC3B:TRMU:RPL32:RPL14:ISY1:XRN1:EIF4E:TRMT10A:RPL37:SKIV2L2:DIMT1:UTP15:THG1L:LSM11:RPP40:RPS18:WDR46:PPIL1:GTF2H5:HSPB1:RPL7:PUF60:RPL12
Curated_gene_sets	FORTSCHEGGER_PHF8_TARGETS_DN	778	62	2.9780379916523607e-08	4.6806248548798956e-06	RPL22:FBLIM1:DPH5:HIPK1:CTSS:PAQR6:HSPA6:UHMK1:ABL2:SOAT1:ZMYND11:STAMBPL1:ASRGL1:AASDHPPT:ERC1:SPSB2:ENO2:EMP1:METTL7A:RAP1B:KDELC1:DCAF11:STXBP6:FRMD6:TMEM251:MAPKBP1:ARPP19:PRSS21:SLX1A:SLC7A6:FAM211A:PLEKHM1:NAPG:MEGF8:CEACAM1:PPP1CB:WDR54:FAM168B:PLEKHB2:PKP4:C2CD2:LSS:OSBP2:HYAL1:CGGBP1:MRFAP1:DCTD:PTGER4:DIMT1:CPEB4:EDN1:PPP1R18:TUBB:ITGB8:MPLKIP:ASL:LAT2:DENND2A:ENY2:NRBP2:XPA:PBX3
Curated_gene_sets	CHARAFE_BREAST_CANCER_LUMINAL_VS_BASAL_DN	454	43	3.611253704401853e-08	5.518196285531832e-06	FBLIM1:PDZK1IP1:TM2D1:DNAJB4:ABCD3:F3:SOAT1:FAM171A1:STAMBPL1:FAS:YAP1:RBM7:REXO2:UBASH3B:CLMP:C1S:EMP1:FRMD6:GJC1:MYL12A:RALBP1:PPP4R1:MBP:CD97:RND3:RBMS1:COPS8:PI3:SLPI:CYB5R3:TGFBR2:FNDC3B:ATP10D:CXCL1:CFI:DCTD:CARD6:ARAP3:TNFAIP3:ITGB8:CALD1:ESYT2:LYN
Curated_gene_sets	GOBERT_OLIGODENDROCYTE_DIFFERENTIATION_DN	1064	77	4.051963555688119e-08	6.024284194551444e-06	ICMT:DNAJB4:LRRC8B:LRRC8C:GOLPH3L:CTSK:FAM63A:PRUNE:RUSC1:SYT11:SMG5:SEC16B:SOAT1:ADCK3:ARL5B:REEP3:ZFYVE27:DUSP8:IPO7:VPS51:LRFN4:UCP2:DGAT2:UBASH3B:DDX25:KCNA1:SDSL:BRI3BP:ULK1:LHFP:TGDS:GPR180:ZFYVE1:RBM25:TCF12:FAM211A:VAT1:RND2:BRCA1:HELZ:TTYH2:IMPA2:TTC39C:MBP:TUBB4A:GIPC1:NFKBIB:PKP4:RBMS1:SNED1:C21orf91:SYNGR1:NAGA:KIAA1644:PPARA:DENND6B:RAD18:LRIG1:PROS1:GSK3B:XRN1:KLHL2:PCDHGC4:LSM11:SLC22A23:C4A:FUT9:PLEKHG1:SYNE1:ERMARD:ARL4A:ITGB8:TERF1:TMEM71:RBM18:FAM102A:TOR4A

Curated_gene_sets	WANG_TUMOR_INVASIVENESS_UP	361	37	4.307285083868531e-08	6.235361906937049e-06	TARDBP:CDC42:PTP4A2:ABCD3:CKS1B:CLK2:NUCKS1:IPO7:FAU:PC:GTF3A:POSTN:IPO5:FKBP3:CPSF2:ARPP19:SMG1:CLEC10A:RPL19:KPNB1:RALBP1:CD79A:PEX13:NCL:PCNA:RPL14:LRIG1:PROS1:ABCF3:PLAC8:CKS1B:TUBB:SEPT7:YWHAG:CALD1:ASH2L:ENY2:TUBB4B
Curated_gene_sets	IWANAGA_CARCINOGENESIS_BY_KRAS_PTEN_DN	347	36	4.7999336559754126e-08	6.7703679593642936e-06	GPR153:MTOR:LPHN2:ARHGEF2:B3GALNT2:NID1:VPS51:CDON:PSEN1:MYO5A:KIAA1199:PDZD9:CORO1A:TGFB1I1:SNAI3:BRCA1:HELZ:INSR:CD97:MAST3:ERF:TMEM87B:TGM2:DDX27:NEK11:ATP10D:ANKRA2:RPP40:MAP7:INMT:ABCB1:ZMAT4:ZNF34:IPPK:XPA:KLF4
Curated_gene_sets	MORI_SMALL_PRE_BII_LYMPHOCYTE_DN	71	15	4.9973269230804934e-08	6.870823827825141e-06	RHCE:STIL:LCP1:PCK2:NFATC3:DERL2:DNAJB1:XPO1:RPN2:IFRD2:EIF4E:CDKN1A:CD36:CA2:TUBB4B
Curated_gene_sets	MARSON_BOUND_BY_E2F4_UNSTIMULATED	682	56	5.120955770602269e-08	6.870823827825141e-06	TXLNA:CDC20:MED8:STIL:TXNDC12:BTF3L4:CKS1B:FAM189B:SCAMP3:PMF1:ASPM:NUCKS1:MCM10:LINC00610:COMMD9:CDCA5:ZFPL1:USP5:SLC11A2:CIT:RNASEH2B:FKBP3:FANCM:DLGAP5:GTF2A1:NTAN1:CD2BP2:SIAH1:DERL2:VAT1:RND2:KPNB1:TCF3:UBXN6:C19orf57:CC2D1A:SARS2:MRPS12:SPDYA:RPN2:SYNGR1:GTSE1:SHQ1:DBR1:ARMC8:DROSHA:C5orf22:IPO11:CKS1B:PCDHGC4:PCDHGC5:NRM:MDC1:TUBB:PPIL1:C6orf89:NUDCD1:ENY2
Curated_gene_sets	REACTOME_DISEASE	1072	77	5.5131802394218296e-08	7.22095345168083e-06	RPL22:MTOR:NRAS:LMNA:FMOD:PSEN2:KCNJ11:ABCC8:FAU:SYVN1:PC:FDX1:SLC11A2:RAP1B:KPNA3:IPO5:PSME1:PSEN1:GTF2A1:ADAMTS7:SH3GL3:ACAN:RPL3L:POLR2C:DERL2:RPL19:KPNB1:PSMD12:RPL38:TAF4B:TCEB3C:TCEB3B:SH3GL1:CD320:MUC16:AP1M2:GSK3A:XPO1:CREB1:CHMP4B:RPL32:HDAC11:TGFBR2:RPL14:TRAK1:HYAL1:GSK3B:RBP1:RASA2:AP2M1:SLC34A2:SLC4A4:AREG:AREGB:SLC6A19:SLC6A18:RPL37:HDAC3:PDGFRB:HLA-A:MUC21:RPS18:CDKN1A:ESR1:GTF2H5:SOD2:FGFR1OP:THBS2:CD36:RPL7:CYP11B1:CYP11B2:ADAMTSL1:CHMP5:RPL12:DPM2:SLC34A3:NELFB
Curated_gene_sets	WAKABAYASHI_ADIPOGENESIS_PPARG_RXRA_BOUND_8D	829	64	5.767466588964587e-08	7.378333419975394e-06	PTP4A2:TXLNA:TXNDC12:BTF3L4:ABCD3:HIPK1:CKS1B:DCST1:MSTO1:LMNA:SEMA4A:ADCK3:NID1:STAMBPL1:PEX16:GYLTL1B:PC:UCP2:TENM4:YAP1:FDX1:USP5:ATN1:C12orf57:PTPN6:LETMD1:POLE:UCHL3:ZNF770:MAPKBP1:LONP2:PLA2G15:SNAI3:RABEP1:CLEC10A:RNFT1:TTYH2:MYL12A:UBXN6:PEX11G:GSK3A:LIPE:MOB1A:RBMS1:LIME1:TRAK1:GNAI2:HYAL2:ARMC8:FNDC3B:NIPBL:CKS1B:CDKN1A:RNASET2:YWHAG:CD36:FAM3C:CREB3L2:UBQLN1:PALM2:FAM102A:SLC25A25:TUBB4B:FAM166A:TOR4A
Curated_gene_sets	KRIGE_RESPONSE_TO_TOSEDOSTAT_6HR_UP	926	69	6.892343500053198e-08	8.616995816771056e-06	C1orf63:KTI12:TM2D1:PHGDH:CTSS:RIT1:SEMA4A:BGLAP:ZBTB41:C10orf54:FAS:UBE2L6:METTL7A:LETMD1:CSRNP2:UHRF1BP1L:MTMR6:PCK2:MIA2:CTAGE5:NUMB:MYO5A:TCF12:PKD1:NPIPA1:N4BP1:SHPK:SHPK:NBR1:UBALD2:MYL12A:TAF4B:CD320:CCDC130:DCAF15:FPR2:NOL10:BCL11A:MTHFD2:IL18RAP:TMEM87B:PKP4:ESF1:LSS:ARFGAP3:CELSR1:CHKB:IFRD2:IFT57:NPHP3:ARMC8:IL8:KLHL2:PTGER4:RPL37:ANKRA2:UTP15:CPEB4:RPP40:ZNF311:HLA-A:FLOT1:PFDN6:CDKN1A:MAP7:HECA:ARL4A:ESYT2:KIAA0020:KLF4:TOR4A
Curated_gene_sets	DACOSTA_UV_RESPONSE_VIA_ERCC3_DN	856	65	8.348775878593418e-08	1.0205914690698309e-05	SPEN:MACF1:STIL:F3:TRIM33:SETDB1:ZMYND11:LARP4B:FAM171A1:CUL2:GRK5:IPO7:YAP1:ERC1:SLC11A2:NUAK1:ANKLE2:WBP4:FNDC3A:KPNA3:MIA2:CTAGE5:FAM179B:DLGAP5:NUMB:AQR:TCF12:KIAA1199:N4BP1:NFATC3:BRCA1:NPEPPS:SPAG9:UTP18:HELZ:PHLPP1:HPCAL1:PUM2:NCOA1:XPO1:ZNF638:PKP4:RBMS1:CREB1:CDS2:C2CD2:TGFBR2:TRAK1:FAM208A:LRIG1:GSK3B:ATP2C1:EIF4E:NIPBL:SKIV2L2:KIF2A:IER3:THBS2:TPST1:CREB3L2:RB1CC1:LYN:NSMAF:TERF1:KIAA0020:PBX3
Curated_gene_sets	ACEVEDO_LIVER_CANCER_UP	971	71	9.251683243307222e-08	1.0836834230784386e-05	C1orf63:CDC20:TM2D1:RPF1:PTBP2:DPH5:NRAS:GOLPH3L:SCNM1:CKS1B:ADAM15:KIAA0907:SSR2:PMF1:CFHR5:F13B:ASPM:PHLDA3:RAB18:STAMBPL1:AASDHPPT:METTL7A:APAF1:PRKAB1:MTMR6:LCP1:KPNA3:STXBP6:C14orf142:ARPP19:NTAN1:CIAPIN1:KPNB1:SH3GL1:DNAJB1:ZNF83:TRMT61B:XPO1:RPN2:DDX27:ZNFX1:TYMP:MYRIP:EIF1B:RPL14:TUSC2:CYB561D2:XXcos-LUCA11.4:COPB2:FNDC3B:DVL3:SENP2:KLHL2:DROSHA:DHX29:CKS1B:PDGFRB:MDC1:PTP4A1:MPLKIP:HSPB1:YWHAG:ABCB1:GNG11:BET1:SPDYE3:ZNF467:GIMAP5:RB1CC1:RPL7:NRBP2:KIAA0020:CHMP5
Curated_gene_sets	SMIRNOV_RESPONSE_TO_IR_6HR_UP	168	23	9.405174967435372e-08	1.0836834230784386e-05	CDC42:TMEM57:DNAJB4:LMNA:PHLDA3:ADCK3:ACTA2:FAS:SAC3D1:MRPL49:METTL7A:APAF1:PRKAB1:RFX7:SLC7A6:CEACAM1:APOBEC3B:PLXNB2:ZNF654:ANKRA2:IER3:CDKN1A:ZNF79
Curated_gene_sets	SMID_BREAST_CANCER_BASAL_UP	659	54	9.455881532042367e-08	1.0836834230784386e-05	CDC20:PDZK1IP1:DNAJB4:PHGDH:ARNT:HSPA6:ASPM:MCM10:FAM171A1:BBOX1:LRFN4:LGR5:STXBP6:COCH:DLGAP5:ACAN:CALB2:GJC1:IMPA2:SERPINB4:CHAF1A:MUC16:MRPS12:FBXO17:CEACAM1:BCL11A:RND3:RBMS1:TTLL4:PI3:SLPI:APOBEC3B:GTSE1:CAND2:HYAL1:RAB6B:MRAS:RBP1:RASA2:FNDC3B:SLC34A2:IL8:CXCL1:EDN1:PRSS16:MDC1:GSTA1:FUT9:SOD2:ITGB8:CALD1:DENND2A:LYN:KHDRBS3

Curated_gene_sets	NIKOLSKY_BREAST_CANCER_1Q21_AMPLICON	38	11	9.674666723525015e-08	1.0861294213492063e-05	CKS1B:DCST2:DCST1:ADAM15:FAM189B:SCAMP3:CLK2:HCN3:PKLR:FDPS:RUSC1:CKS1B
Curated_gene_sets	LEE_BMP2_TARGETS_UP	750	59	1.0018424648011429e-07	1.1022270797742173e-05	RPL22:CLSTN1:ANGPTL7:FBLIM1:NBL1:CCDC28B:TIE1:TSPAN1:PDZK1IP1:F3:VAV3:RUSC1:SEMA4A:C1orf85:F5:ADCK3:C10orf54:EHF:ATN1:C1S:METTL7A:PCK2:STXBP6:CORO1A:TGFB1I1:MFAP4:VAT1:RND2:PKN1:LIPE:CXCL17:GREB1:MYCN:RTKN:IGFBP5:NAGA:CYP2D6:CYB5R3:PLXNB2:CHKB:HDAC11:NAT6:LRIG1:RAB6B:PLSCR4:KCNAB1:ZNF608:FLOT1:IER3:TAPBP:ZFAND3:FUT9:ITGB8:CLDN3:ABCB1:ZNF467:CA2:CALB1:ADAMTSL1
Curated_gene_sets	REACTOME_SIGNALING_BY_GPCR	1171	81	1.2173043033957428e-07	1.313017837839212e-05	HTR6:CDC42:RPE65:VAV3:NRAS:ARHGEF2:ARHGEF11:OR14K1:RBP3:REEP3:TACR2:PSAP:RGR:GRK5:OR51T1:OR4C15:OR4C16:OR4P4:OR4S2:OR4C6:OR5T1:OR8H1:OR8K3:OR8K1:OR8J1:OR8U1:OR5R1:TRPC6:PTHLH:PDE1B:OR10P1:MLNR:GLP2R:RCVRN:MAPK7:DGKE:SSTR2:ARHGEF18:OR1M1:OR7G2:OR7G1:OR7G3:PDE4A:S1PR5:RLN3:CD97:PTGER1:OR10H2:OR10H3:FPR2:PLB1:FSHR:CREB1:MC3R:MCHR1:GNAI2:ACKR4:RBP2:RBP1:IL8:CXCL6:CXCL1:NPY1R:NPY5R:PTGER4:EDN1:OR10C1:HCRTR2:CNR1:CRHR2:GNG11:PRKAR2B:KEL:TAS2R39:TAS2R40:PPP3CC:RDH10:S1PR3:OR1L3:OR1L4:OR1L6
Curated_gene_sets	FEVR_CTNNB1_TARGETS_UP	687	55	1.540013389715576e-07	1.6043574242164133e-05	CLSTN1:DDAH1:PKLR:LMNA:REEP3:DUSP8:DGAT2:EMP1:SLC11A2:TNFSF13B:MIA2:CTAGE5:ERO1L:SLC51B:PKD1:SMG1:CORO1A:FAM83G:SLC47A1:HNF1B:VAT1:TRIM25:ENTHD2:SYCN:IL1RL1:CDS2:ZBP1:LSS:CYP2D6:ARFGAP3:PLXNB2:TYMP:HYAL2:ACKR4:LIPH:CXCL6:MTTP:OCLN:C5orf30:NDFIP1:PDGFRB:SLC22A23:FLOT1:VPS52:B3GALT4:CDKN1A:C6orf89:TNFAIP3:PLEKHG1:CLDN3:CD36:ABCB1:CPA1:ZNF467:LY6E:CEL
Curated_gene_sets	REACTOME_DEVELOPMENTAL_BIOLOGY	1100	77	1.5625570881326777e-07	1.6043574242164133e-05	RPL22:CDC42:MED8:TAL1:VAV3:NRAS:FLG:LCE1F:LCE1E:LCE1D:LCE1C:LCE1B:PKLR:SEMA4A:ARHGEF11:ABL2:PSEN2:TUBB8:CUL2:KRTAP5-1:KRTAP5-2:KRTAP5-3:KRTAP5-4:KRTAP5-5:FAU:TRPC6:HSPA8:CDON:PSME1:PSEN1:NUMB:TCF12:RPL3L:SIAH1:POLR2C:MAPK7:HNF1B:CACNB1:RPL19:ADAM11:SPAG9:PSMD12:RPL38:MYL12A:MYL12B:TCF3:TUBB4A:NCOA1:ZNF638:PKP4:SCN3A:CREB1:PI3:KRTAP21-3:KRTAP8-1:PPARA:SHANK3:RPL32:SCN5A:RPL14:FOXP1:GSK3B:AP2M1:RPL37:ISL1:HDAC3:RPS18:CDKN1A:CD36:ZNF467:GFRA2:ASH2L:LYN:RPL7:KLF4:RPL12:TUBB4B
Curated_gene_sets	TOYOTA_TARGETS_OF_MIR34B_AND_MIR34C	445	41	1.5749009436045507e-07	1.6043574242164133e-05	IQCC:STIL:HOOK1:LRRC8C:TRIM33:PHGDH:HCN3:BGLAP:FAM171A1:AGAP4:ZNF488:AGAP8:HECTD2:WEE1:PRKRIR:CDON:USP5:BRI3BP:POLE:ANKLE2:SMIM2:PDXDC1:KPNB1:PPP4R1:CC2D1A:NOL10:TTLL4:C2CD2:GTSE1:SHQ1:ARL13B:COQ2:DIAPH1:PRELID2:TUBB:FLOT1:C6orf89:ESR1:RFC2:SPDYE3:LY6E:PTPN3
Curated_gene_sets	BUYTAERT_PHOTODYNAMIC_THERAPY_STRESS_UP	821	62	2.0073015975042433e-07	2.0076665614310623e-05	CDC42:DNAJB4:PTBP2:TRIM33:NRAS:GOLPH3L:GON4L:RIT1:ARHGEF2:HSPA6:ABL2:ZNF143:GPRC5A:PTHLH:ULK1:WBP4:TGDS:MAP3K9:RBM25:PSEN1:NUMB:ARPP19:PKD1:NPIPA1:N4BP1:PLA2G15:RABEP1:MAPK7:VAT1:GJC1:PLEKHM1:SPAG9:TRIM25:MBP:INSR:ZNF442:DNAJB1:ZNF83:NCOA1:MTHFD2:PLEKHB2:RND3:PER2:LSS:ARFGAP3:RASSF1:GSK3B:IL8:AREG:AREGB:NIPBL:PPAP2A:IER3:CDKN1A:PTP4A1:HECA:SOD2:BET1:RB1CC1:NSMAF:GEM:KLF4:FAM102A
Curated_gene_sets	RAO_BOUND_BY_SALL4_ISOFORM_B	500	44	2.0601397370208347e-07	2.0237194095270736e-05	BCL10:VAV3:TOR3A:F13B:ZBTB41:RAB18:IPO7:OR8J1:ASRGL1:YAP1:SLC25A3:TNFRSF19:LHFP:IPO5:G2E3:MOAP1:COQ9:EPN2:MBTD1:MYL12B:OR7G2:OR7G1:TTLL4:TMEM230:EFHB:CGGBP1:IFT57:SENP2:MRFAP1:TRMT10A:NPY1R:NPY5R:PPAP2A:ACTBL2:SOX30:KLHL31:FUT9:L3MBTL3:MAP7:COPG2:CREB3L2:PPP3CC:RB1CC1:NSMAF
Curated_gene_sets	GRESHOCK_CANCER_COPY_NUMBER_UP	323	33	2.447869191759722e-07	2.36240849541583e-05	RPL22:TAL1:STIL:BCL10:RBM15:TRIM33:NRAS:ARNT:ABL2:FAS:POU2AF1:ERC1:HOXC13:HOXC11:LHFP:LCP1:ERCC5:TCF12:RABEP1:BRCA1:TCF3:SH3GL1:MYCN:ALK:BCL11A:AFF3:CNBP:PDGFRB:FGFR1OP:HOXA11:HOXA13:JAZF1:XPA
Curated_gene_sets	ONKEN_UVEAL_MELANOMA_UP	789	60	2.492968034336468e-07	2.3644512339456744e-05	MACF1:TM2D1:TRIM33:CTSS:CTSK:SETDB1:CERS2:PRUNE:SOAT1:CREM:PSAP:EMP1:SLC11A2:GTF3A:LHFP:KCTD12:DLGAP5:RBM25:NUMB:NIPA2:HERC2:TCF12:NPIPA1:NTAN1:RRN3:SULT1A3:KPNB1:PITPNC1:SAFB2:PDE4A:CD97:DDX39A:GREB1:ALK:RBMS1:COPS8:PCNA:C2CD2:NDUFA6:CELSR1:SCO2:TYMP:COQ2:PTGER4:PCDHGC3:IER3:C4A:TAPBP:CDKN1A:SOD2:PILRB:RB1CC1:LYN:NSMAF:TERF1:GEM:ENY2:LY6E:KIAA0020:NACC2
Curated_gene_sets	MOHANKUMAR_HOXA1_TARGETS_UP	406	38	2.9834860397937765e-07	2.7817214754077228e-05	ICMT:CDC20:HOOK1:DDAH1:ABCD3:WDR77:HIPK1:PRUNE:WEE1:UBE2L6:PRKRIR:HSPA8:USP5:UBR7:CYB5B:TBX21:HELZ:AP1M2:DNAJB1:PPP1CB:XPO1:DCTN1:PKP4:COPS8:PCNA:RPN2:COPB2:MDC1:TUBB:IER3:WDR46:PTP4A1:SEPT7:CD36:MEPCE:MEST:CNTLN:TUBB4B
Curated_gene_sets	ZHOU_INFLAMMATORY_RESPONSE_LIVE_UP	441	40	3.3420817964554857e-07	3.0641319937169375e-05	MACF1:MFSD2A:F3:SEMA6C:ABL2:HECTD2:CDON:ANKLE2:PCDH9:NRL:KLHL28:MESDC1:GRIN2A:ARMC5:ABCC11:TCEB3B:BCL11A:AFF3:RND3:BFSP1:LSS:ODF3B:GSK3B:RAB6B:KCNAB1:FNDC3B:CXCL1:EIF4E:CPEB4:C5orf47:SLC22A23:EDN1:IER3:TNFAIP3:SOD2:TCTE3:ITGB8:ZCWPW1:PPP3CC:S1PR3

Curated_gene_sets	FULCHER_INFLAMMATORY_RESPONSE_LECTIN_VS_LPS_UP	583	48	4.208060307612948e-07	3.794842582324398e-05	ACOT7:MFSD2A:TM2D1:LRRC8C:F3:TNFAIP8L2:SSR2:FCGR2A:HSPA6:FCRLB:ABL2:SOAT1:CSRP1:EHF:EMG1:ORAI1:GTF3A:FNDC3A:ERO1L:C14orf142:MYO5A:ANP32A:DCTPP1:CCL17:CIAPIN1:COQ9:CYB5B:TRAPPC2L:SHPK:SHPK:C17orf89:TCF3:MRPS12:BOLA3:MTHFD2:TGM2:SMDT1:ATP2C1:SRPRB:MAP3K13:IL8:CXCL1:KIF2A:MARCH3:TIFAB:ADAM19:EDN1:LAT2:CALD1
Curated_gene_sets	ENK_UV_RESPONSE_KERATINOCYTE_UP	533	45	4.814833645931317e-07	4.2719999816561576e-05	TSPAN1:HMGCS2:SCAMP3:CSRP1:DUSP8:SMPD1:NAALADL1:FAU:PHB2:ULK1:PSME1:MOAP1:HNF1B:RPL19:VAT1:SH3GL1:TUBB4A:TECR:MRPS12:MYCN:DCTN1:RND3:EIF6:TGM2:DGCR6:EIF1B:RPL14:CGGBP1:CXCL1:AREG:AREGB:HLA-A:IER3:CDKN1A:TNFAIP3:EVX1:ASL:CLDN4:RFC2:HSPB1:GEM:CYP11B1:LY6E:RPL12:DPM2:TUBB4B
Curated_gene_sets	MARSON_BOUND_BY_FOXP3_UNSTIMULATED	1177	79	5.768056919202283e-07	5.0365208115129774e-05	C1orf63:TMEM50A:LDLRAP1:TCEANC2:DNAJB4:BCL10:LRRC8C:EXTL2:TRIM33:GOLPH3L:CTSS:SETDB1:CERS2:ANXA9:FAM63A:PRUNE:SCNM1:LYSMD1:SEMA4A:FCGR2A:FCRLA:ADCK3:MCM10:RAB18:C10orf54:WEE1:POU2AF1:C11orf71:RBM7:LPCAT3:EMP1:TNFRSF19:SLC25A15:KPNA3:PSEN1:PAPLN:CPSF2:TMEM251:NIPA1:MTFMT:TBC1D10B:MYLPF:NFATC3:DHX33:FAM211A:ZPBP2:KPNB1:HELZ:KCNJ2:UBALD2:FSCN2:MBP:PODNL1:PPP1CB:XPO1:MOB1A:MTHFD2:CREB1:METTL21A:CTDSP1:TGFBR2:ISY1:DBR1:ARMC8:ATP10D:NDFIP1:ARL4A:MPLKIP:TSC22D4:NYAP1:GPR22:GIMAP5:TERF1:LRRC6:TMEM71:XPA:NAIF1:TUBB4B:TOR4A
Curated_gene_sets	RIZKI_TUMOR_INVASIVENESS_3D_DN	259	28	6.289132641840252e-07	5.405706041056754e-05	ICMT:ZBTB17:NBL1:CDC42:HSPA6:SNRPE:UBE2L6:POSTN:PSME1:TRPM1:JMJD7-PLA2G4B:PRSS21:NOMO1:SMG1:RCVRN:MYL12B:SLC14A1:HPCAL1:RTCB:CYP2D6:PROS1:SOX30:EVX1:YWHAG:ABCB1:DUS4L:MEST:CALB1
Curated_gene_sets	GOLDRATH_ANTIGEN_RESPONSE	353	34	6.42106464208302e-07	5.4341963993997996e-05	ACOT7:RHCE:CDC20:SSX2IP:CKS1B:LMNA:ASPM:MCM10:WEE1:CDCA5:TPI1:EMP1:RNASEH2B:FANCM:DLGAP5:N4BP1:BRCA1:CHAF1A:IL12RB1:MTHFD2:IL18R1:IL18RAP:PLEKHB2:PCNA:DSTN:SLPI:GTSE1:PTGER4:CKS1B:IER3:C6orf89:LAT2:LYN:CA2:GEM
Curated_gene_sets	ZWANG_CLASS_3_TRANSIENTLY_INDUCED_BY_EGF	230	26	6.670591335714848e-07	5.559836808752633e-05	ARL5B:DMBT1:WEE1:EMP1:FNDC3A:LMO7:FRMD6:MBP:RND3:RBMS1:NCL:PER2:APOBEC3B:PROS1:RASA2:IL8:CXCL1:PLAC8:EDN1:IER3:PTP4A1:TNFAIP3:CLDN4:GEM:IPPK:KLF4
Curated_gene_sets	REACTOME_VESICLE_MEDIATED_TRANSPORT	722	55	7.425294660837074e-07	6.025118249350279e-05	LDLRAP1:DENND2C:GJA5:SYT11:F5:SEC16B:TUBB8:RAB18:HPX:VPS51:HSPA8:PRKAB1:ULK1:SCFD1:MIA2:CTAGE5:MYO5A:SH3GL3:TBC1D10B:TRAPPC2L:RABEP1:EPN2:GJC1:KIF19:NAPG:SH3GL1:DENND1C:TUBB4A:AP1M2:EPS15L1:PAFAH1B3:KDELR1:DCTN1:COPS8:RALGAPA2:CHMP4B:APOL1:ARFGAP3:DENND6B:TMEM115:RAB43:RAB6B:COPB2:AP2M1:AREG:AREGB:KIF2A:VPS52:YWHAG:CD36:BET1:COPG2:EXOC4:DENND2A:CHMP5:UBQLN1:TUBB4B
Curated_gene_sets	RODRIGUES_THYROID_CARCINOMA_ANAPLASTIC_DN	524	44	7.447882947751663e-07	6.025118249350279e-05	LPHN2:SAMD13:SLC16A4:CTSS:CTSK:CSRP1:FMOD:ECHDC3:ACTA2:GRK5:DMBT1:C11orf71:DDX25:CDON:SLC25A3:LHFP:SLC25A15:PCDH9:KCTD12:STXBP6:SULT1A3:LONP2:NFATC3:B9D1:PITPNC1:PDE4A:RFX1:BCAM:RALGAPA2:SMDT1:VGLL4:HDAC11:LRIG1:FOXP1:PLSCR4:KCNAB1:SLC4A4:PLA2G12A:PPAP2A:NDFIP1:RNASET2:CREB3L2:ENY2:SLC35D2
Curated_gene_sets	KINSEY_TARGETS_OF_EWSR1_FLII_FUSION_UP	1286	84	7.660507868364047e-07	6.101273261798834e-05	ICMT:ACOT7:CDC20:MED8:HOOK1:SSX2IP:DENND2C:AMPD1:NRAS:PRUNE:SOAT1:ASPM:NUCKS1:ZNF496:MCM10:FAS:GPAM:GRK5:IPO7:WEE1:ASRGL1:CDCA5:AASDHPPT:FDX1:HSPA8:NCKAP1L:BRI3BP:MTMR6:KIAA0226L:TNFSF13B:PCK2:G2E3:LRFN5:ERO1L:DLGAP5:GTF2A1:CPSF2:C14orf142:UBR7:NIPA1:MTFMT:ANP32A:SH3GL3:DCTPP1:CIAPIN1:COQ9:NFATC3:CYB5B:DHX33:BRCA1:KPNB1:IMPA2:CHAF1A:SAFB:DDX39A:DNAJB1:TRMT61B:TPRKB:MTHFD2:PKP4:METTL21A:RPE:MFF:PER2:PCNA:ESF1:GTSE1:TRMU:RAD18:THUMPD3:ENTPD3:DBR1:STK32B:NPY1R:KIF2A:DIMT1:MDC1:PTP4A1:RFC2:DUS4L:NCAPG2:NUDCD1:TMEM71:UBQLN1
Curated_gene_sets	TIEN_INTESTINE_PROBIOTICS_24HR_UP	560	46	7.763845270422076e-07	6.101273261798834e-05	ICMT:TARDBP:CDC42:TMEM50A:ABCD3:HMGCS2:FDPS:SNRPE:CUL2:LINC00610:COMMD9:SAC3D1:AASDHPPT:NDUFA9:EMG1:FKBP3:DLGAP5:NTAN1:DCTPP1:CIAPIN1:CYB5B:TRAPPC2L:SPG7:IMPA2:SAFB2:TRMT61B:PLEKHB2:PCNA:LSS:APOBEC3B:NDUFA6:CYB5R3:GTSE1:ATP2C1:MRPS22:COMMD8:EIF4E:DROSHA:DHX29:DIMT1:CETN3:TTC1:GTF2H5:RFC2:CALD1:CHMP5:UBAC1
Curated_gene_sets	KRIEG_HYPOXIA_NOT_VIA_KDM3A	743	56	8.242032487836062e-07	6.385833903603687e-05	PTP4A2:PHC2:RIMKLA:RIT1:SOAT1:NID1:ZNF496:FAS:ALDH18A1:CDCA5:TPI1:ENO2:GPRC5A:IKBIP:NUAK1:TMEM120B:POLE:ANKLE2:LHFP:ERCC5:NIPA1:NTAN1:ZNF48:SPG7:MAPK7:HNF1B:UBALD2:TCF3:SH3GL1:FBXO17:CIC:WDR54:CTDSP1:CDS2:BFSP1:MMP24:APOL2:APOL1:RASSF1:GSK3B:FGF12:TMEM44:CXCL6:CXCL1:PRELID2:KCNIP1:TUBB:IER3:TNFAIP3:SOD2:RNASET2:NCAPG2:PPP3CC:POU5F1B:PALM2:TUBB4B
Curated_gene_sets	KOINUMA_TARGETS_OF_SMAD2_OR_SMAD3	839	61	8.947485737331896e-07	6.83612764459205e-05	CLSTN1:ZNF362:F3:EXTL2:DENND2C:SNRPE:NUCKS1:B3GALNT2:ZNF143:WEE1:EHF:PC:YAP1:GPRC5A:EMP1:PTHLH:WBP4:DCAF11:FRMD6:RBM25:NPIPA1:SMG1:SULT1A3:SLC7A6:EPN2:SPAG9:MBTD1:TRIM25:HELZ:PEX13:IL1RL1:TMEM87B:COPS8:

						DSTN:CHMP4B:TGM2:APOBEC3B:ARFGAP3:PLCXD2:RASA2:MFI2:CXCL6:COQ2:EIF4E:OCLN:ADAM19:EDN1:MDC1:TUBB:FLOT1:IER3:CDKN1A:PTP4A1:ARL4A:HOXA10:CLDN4:RFC2:CALD1:UBQLN1:ST6GALNAC4:GTF3C5
Curated_gene_sets	LIM_MAMMARY_STEM_CELL_DN	408	37	9.147310657682111e-07	6.893062455877985e-05	RPL22:TMEM125:C1orf210:PDZK1IP1:GOLPH3L:DMBT1:SNX15:TM7SF2:C11orf71:PTPN6:SDSL:ORAI1:RNASEH2B:PSME1:IFI35:PEX11G:AP1M2:RFX1:PKN1:GIPC1:PAFAH1B3:CXCL17:CEACAM1:CBLC:TGM2:SLC37A1:SYNGR1:THUMPD3:HDAC11:LIPH:C5orf22:ANKRA2:TSPAN17:ESR1:CLDN3:CA2:SLC35D2
Curated_gene_sets	REACTOME_RRNA_PROCESSING_IN_THE_NUCLEUS_AND_CYTOSOL	191	23	9.630799776101587e-07	7.159328320045247e-05	RPL22:EXOSC10:EBNA1BP2:FAU:EMG1:DDX47:DHX37:RPL3L:RPL19:UTP18:RPL38:NCL:RPL32:RPL14:RPL37:SKIV2L2:DIMT1:UTP15:RPP40:RPS18:WDR46:RPL7:RPL12
Curated_gene_sets	NUYTTEN_NIPP1_TARGETS_UP	766	57	9.865972521070224e-07	7.236361978454306e-05	RHCE:SPOCD1:PTP4A2:TXNDC12:TM2D1:DNAJB4:SAMD13:BCL10:ARL5B:CREM:FDX1:LHFP:WBP4:EBPL:TGDS:DTD2:RBM25:NUMB:TCF12:DHX33:ZNF480:ZNF765:PEX13:MOB1A:PLEKHB2:DAW1:RPN2:TGM2:ZNFX1:APOL2:CDPF1:VGLL4:PROS1:UBA5:ARMC8:GK5:XRN1:COMMD8:SLC4A4:IL8:CXCL6:CXCL1:CDH6:ISL1:C5orf30:LSM11:PPIL1:TNFAIP3:JAZF1:FAM3C:MEST:CALD1:PPP3CC:GEM:NRBP2:CHMP5:PBX3
Curated_gene_sets	PURBEY_TARGETS_OF_CTBP1_NOT_SATB1_UP	297	30	1.0761789867909583e-06	7.702176185144766e-05	HES2:EBNA1BP2:SETDB1:PAQR6:ACTA2:GPAM:FAU:PRKRIR:AKAP3:DLGAP5:SMPD3:KPNB1:INSR:SARS2:LIPE:ZNF135:IL18R1:USP25:SPATC1L:TRMU:ATP2C1:SENP2:CXCL6:CXCL1:NDFIP1:IER3:PTP4A1:MEPCE:XPA:PAPPA
Curated_gene_sets	PATIL_LIVER_CANCER	657	51	1.0781086461664188e-06	7.702176185144766e-05	CLSTN1:CDC20:HORMAD1:ARNT:FAM63A:PRUNE:CKS1B:ADAM15:FAM189B:SCAMP3:CLK2:FDPS:RUSC1:MSTO1:YY1AP1:GON4L:SMG5:ABL2:SNRPE:NUCKS1:ALDH18A1:ARL2:SAC3D1:CDCA5:LGR5:STXBP6:DLGAP5:CPSF2:VAT1:RNF157:CHAF1A:PAFAH1B3:MYCN:XPO1:PCNA:OSBP2:MAPK8IP2:HDAC11:DVL3:CKS1B:PDGFRB:NRM:TUBB:PFDN6:ARL4A:DUS4L:CREB3L2:NCAPG2:RB1CC1:RDH10:PUF60:DPM2
Curated_gene_sets	WEI_MYCN_TARGETS_WITH_E_BOX	788	58	1.123660576122143e-06	7.924688242625524e-05	CDC42:SPOCD1:SSX2IP:WDR77:ARNT:TOR3A:MCM10:CREM:STAMBPL1:CDCA5:LRFN4:AASDHPPT:REXO2:HSPA8:DDX25:USP5:EMP1:DHX37:P2RX2:UCHL3:G2E3:ERO1L:MYO5A:MTFMT:MESDC2:DCTPP1:DHX33:KPNB1:UTP18:PSMD12:TAF4B:MBP:NFKBIB:MRPS12:MYCN:ALK:TPRKB:METTL21A:COPS8:ESF1:NDUFAF5:HYAL2:SRPRB:LSG1:COMMD8:DIMT1:IPO11:SLC12A2:DIAPH1:RPP40:TUBB:POPDC3:LYN:CA2:GEM:NUDCD1:KIAA0020:UBQLN1
Curated_gene_sets	SPIELMAN_LYMPHOBLAST_EUROPEAN_VS_ASIAN_UP	484	41	1.4076125364639047e-06	9.801615902642962e-05	ICMT:ACOT7:CDC42:MACF1:CDC20:TRIM33:FDPS:PMF1:ACTA2:FAU:UCP2:PTPN6:APAF1:POLE:ERCC5:PCK2:FKBP3:HERC2:PKD1:CD2BP2:CYB5B:SPG7:RPL19:RPS6KB1:SAFB:CD97:PKN1:GIPC1:TECR:LSS:CYB5R3:TUSC2:NDUFB4:DROSHA:RPL37:PCDHGC3:TUBB:PTP4A1:FGFR1OP:HSPB1:BLK
Curated_gene_sets	DACOSTA_UV_RESPONSE_VIA_ERCC3_COMMON_DN	467	40	1.4321225436974943e-06	9.847632641099896e-05	SPEN:STIL:F3:TRIM33:ZMYND11:LARP4B:FAM171A1:IPO7:YAP1:NUAK1:FNDC3A:KPNA3:NUMB:TCF12:KIAA1199:BRCA1:NPEPPS:UTP18:HELZ:PHLPP1:PUM2:NCOA1:XPO1:ZNF638:PKP4:RBMS1:CREB1:CDS2:C2CD2:TRAK1:FAM208A:GSK3B:EIF4E:SKIV2L2:KIF2A:THBS2:CREB3L2:LYN:KIAA0020:PBX3
Curated_gene_sets	CASORELLI_ACUTE_PROMYELOCYTIC_LEUKEMIA_DN	664	51	1.4672413218650485e-06	9.964561125406952e-05	TARDBP:CDC20:TAL1:STIL:DDAH1:CKS1B:FDPS:LMNA:ASPM:CSRP1:LARP4B:FAM171A1:TM7SF2:TPI1:ENO2:LPCAT3:DLEU1:KDELC1:STXBP6:ERO1L:DLGAP5:CD2BP2:PRMT7:BRCA1:KPNB1:CHAF1A:SAFB:KEAP1:CC2D1A:TECR:PAFAH1B3:PLEKHB2:RPE:LSS:GTSE1:NCAPH2:SHQ1:PROS1:AP2M1:SKIV2L2:CKS1B:THG1L:LRRC16A:TUBB:FLOT1:WDR46:MAP7:HOXA10:ASL:PRKAR2B:DUS4L:ASH2L
Curated_gene_sets	BILD_E2F3_ONCOGENIC_SIGNATURE	241	26	1.6237781584109056e-06	0.00010893175182217551	SSX2IP:DDAH1:FAM171A1:NCR3LG1:SAC3D1:ENO2:BRI3BP:POLE:RBM25:NIPA1:NFATC3:INSR:METTL21A:PCNA:SHANK3:LRIG1:MRAS:DVL3:UBE2QL1:CCNO:C5orf30:TNFAIP3:HECA:KHDRBS3:KIAA0020:ANKRD18B
Curated_gene_sets	SENGUPTA_NASOPHARYNGEAL_CARCINOMA_DN	354	33	1.91871462912448e-06	0.00012628566446483414	TSPAN1:PDZK1IP1:FCRLA:SCGB2A1:POU2AF1:C11orf88:TTC12:MORN3:C14orf142:C15orf26:DNAH9:B9D1:DNAI2:IMPA2:SERPINB4:MUC16:WDR54:CCDC74A:DAW1:PI3:SLPI:SYNGR1:SMDT1:ODF3B:EFHB:TRAK1:RASSF1:IFT57:NEK11:CXCL1:CCNO:GSTA1:TUBB4B
Curated_gene_sets	CHICAS_RB1_TARGETS_SENESCENT	561	45	1.9283758980269162e-06	0.00012628566446483414	ACOT7:SPEN:PTP4A2:PHGDH:CTSS:NUCKS1:WEE1:EHF:UBE2L6:SAC3D1:DGAT2:ENO2:EMP1:BRI3BP:POSTN:KCTD12:LRFN5:C14orf142:UBR7:RASL12:KIAA1199:GJC1:KPNB1:PITPNC1:SLC14A1:SERPINB4:POU2F2:ZNF83:KIAA1841:WDR54:IGFBP5:PCNA:PI3:SLC2A4RG:LRIG1:CXCL6:CXCL1:AREG:AREGB:TNFAIP3:RNASET2:THBS2:RFC2:NCAPG2:RDH10:GEM
Curated_gene_sets	KAYO_CALORIE_RESTRICTION_MUSCLE_UP	93	15	1.968088478448813e-06	0.0001273700555287873	HSPA6:FAS:TCF12:CALB2:VAT1:SAFB:PAFAH1B3:CYB5R3:CHKB:TGFBR2:RBP1:RASA2:ITGB8:CALB1:SCRIB

Curated_gene_sets	SMID_BREAST_CANCER_LUMINAL_B_DN	581	46	2.116916683881281e-06	0.00013540882183756892	TIE1:PDZK1IP1:DNAJB4:PHGDH:FAM171A1:FAS:BBOX1:POU2AF1:C1S:STXBP6:CORO1A:CALB2:CLEC10A:MFAP4:IMPA2:MUC16:FBXO17:CD79A:MYCN:FAM49A:BCL11A:RND3:RBMS1:TTLL4:PI3:SLPI:SYNGR1:FBLN1:PROS1:MRAS:FNDC3B:SLC34A2:IL8:CXCL1:PLAC8:CFI:PTGER4:EDN1:GSTA1:TNFAIP3:SOD2:CALD1:GIMAP5:LYN:GEM:KHDRBS3
Curated_gene_sets	LU_AGING_BRAIN_UP	260	27	2.156342273879964e-06	0.00013591841593682774	PTP4A2:ABCD3:CSRP1:BBOX1:METTL7A:RAP1B:VAT1:MYL12A:SLC14A1:PHLPP1:INSR:GIPC1:GREB1:TGM2:TPD52L2:TGFBR2:FAM208A:LRIG1:SLC4A4:NIPBL:PPAP2A:CETN3:HCRTR2:GNG11:TSC22D4:CALD1:CREB3L2
Curated_gene_sets	LIN_MELANOMA_COPY_NUMBER_UP	71	13	2.1742993278387277e-06	0.00013591841593682774	ARNT:SETDB1:CERS2:FAM63A:PRUNE:NAGA:RPP40:ARL4A:CPA1:CEP41:MEST:COPG2:KLF14
Curated_gene_sets	LEE_BMP2_TARGETS_DN	882	62	2.2035262362796742e-06	0.00013619772837948862	TARDBP:MFSD2A:EBNA1BP2:MED8:ACOT11:WDR77:PHGDH:CKS1B:NUCKS1:MCM10:TRUB1:WEE1:SLC11A2:CSRNP2:UCHL3:FKBP3:MOAP1:NOMO1:RRN3:DCTPP1:CIAPIN1:SLC7A6:PRMT7:METTL16:DHX33:BRCA1:KPNB1:UTP18:RPS6KB1:PSMD12:TAF4B:DDX39A:DNAJB1:GRWD1:NOL10:XPO1:MTHFD2:RND3:TTLL4:COPS8:ESF1:DDX27:THUMPD3:CAND2:SHQ1:DBR1:MRPS22:EIF4E:DCTD:DHX29:SKIV2L2:DIMT1:CKS1B:UTP15:RPP40:WDR46:YWHAG:DUS4L:NUDCD1:KIAA0020:UBQLN1:RPL12:ST6GALNAC4
Curated_gene_sets	REACTOME_RRNA_PROCESSING	201	23	2.340077341693069e-06	0.0001430307272961508	RPL22:EXOSC10:EBNA1BP2:FAU:EMG1:DDX47:DHX37:RPL3L:RPL19:UTP18:RPL38:NCL:RPL32:RPL14:RPL37:SKIV2L2:DIMT1:UTP15:RPP40:RPS18:WDR46:RPL7:RPL12
Curated_gene_sets	POMEROY_MEDULLOBLASTOMA_DESMOPLASIC_VS_CLASSIC_DN	61	12	2.4175905952396394e-06	0.0001461446798287171	NHLH1:NID1:C1S:TCF12:NFATC3:MYCN:RND3:IGFBP5:RPS18:CNR1:RPL7:PBX3
Curated_gene_sets	DODD_NASOPHARYNGEAL_CARCINOMA_DN	1389	87	2.6510567966977043e-06	0.0001585159069416747	RPL22:CCDC28B:TXNDC12:SSX2IP:PTBP2:DPH5:RBM15:NRAS:SIKE1:PHGDH:CKS1B:FAM189B:C1orf85:FCGR2A:ASPM:SNRPE:NUCKS1:B3GALNT2:NID1:MCM10:FAM171A1:ACTA2:FAS:TRUB1:SAC3D1:CDCA5:TPI1:DDX47:TFCP2:IKBIP:NUAK1:BRI3BP:ANKLE2:POSTN:IPO5:KDELC1:FANCM:DLGAP5:ARPP19:TCF12:NTAN1:RRN3:SMG1:DHX33:BRCA1:KPNB1:MBTD1:UTP18:RPS6KB1:SSTR2:TAF4B:TCF3:CHAF1A:DDX39A:TRMT61B:XPO1:MTHFD2:RTKN:ANAPC1:PKP4:METTL21A:RPE:TTLL4:MFF:NCL:PCNA:NDUFAF5:RPN2:SHANK3:RAD18:ATP2C1:UBA5:FNDC3B:DCTD:CKS1B:TUBB:THBS2:HOXA10:ZNF117:ZNF92:YWHAG:PILRA:CALD1:NCAPG2:STAR:NUDCD1:ENY2:COL22A1
Curated_gene_sets	KORKOLA_EMBRYONAL_CARCINOMA_UP	42	10	2.6900791131949584e-06	0.00015911962582457488	NDUFA9:TPI1:SPSB2:ENO2:C12orf57:PTPN6:EMG1:PHB2:DDX47:GPRC5A
Curated_gene_sets	KIM_BIPOLAR_DISORDER_OLIGODENDROCYTE_DENSITY_CORR_UP	681	51	3.024194974633022e-06	0.00017697975058996015	CLSTN1:C1orf123:LAMTOR5:SCAMP3:FDPS:RUSC1:SYT11:ARHGEF2:SNRPE:PSAP:VPS51:FAU:AASDHPPT:TPI1:ENO2:PHB2:CSRNP2:SLC25A3:MTMR6:PSME1:FOXG1:MOAP1:PDXDC1:CORO1A:PSMD12:MYL12B:TUBB4A:ARHGEF18:PAFAH1B3:HPCAL1:PUM2:DCTN1:NCL:DSTN:EIF6:RTCB:NDUFA6:CAND2:RPL32:MYRIP:CNBP:RPL37:TTC1:VPS52:RPS18:ARL4A:RPL7:PUF60:RPL12:FAM102A:TUBB4B
Curated_gene_sets	SENGUPTA_NASOPHARYNGEAL_CARCINOMA_WITH_LMP1_UP	396	35	3.1836989058549907e-06	0.0001843529229590348	LPHN2:F5:ASPM:IPO7:EHF:SCGB2A1:PTHLH:TNFRSF19:POSTN:LMO7:G2E3:RBM25:SPAG9:MOB1A:MTHFD2:PKP4:RPE:IGFBP5:ESF1:KRBOX1:KRBOX1:ZPLD1:FNDC3B:IL8:MCTP1:C5orf30:MEGF10:SLC12A2:CPEB4:CNR1:POPDC3:PRKAR2B:MEST:CALB1:PAPPA:CEL
Curated_gene_sets	VANTVEER_BREAST_CANCER_ESR1_DN	266	27	3.3261038744536167e-06	0.000190592681389264	CDC20:PDZK1IP1:STIL:LRRC8C:PHGDH:FAM171A1:FDX1:UBASH3B:UCHL3:GPR180:ADAMTS7:HAPLN3:IMPA2:FAM49A:PPP1CB:BCL11A:RBMS1:C21orf91:APOBEC3B:RAB6B:MRAS:FNDC3B:TNFAIP3:PLEKHG1:SOD2:CREB3L2:LYN
Curated_gene_sets	BONOME_OVARIAN_CANCER_SURVIVAL_SUBOPTIMAL_DEBULKING	504	41	3.856132979366012e-06	0.00021868646927311784	ICMT:ACOT7:NBL1:TM2D1:DNAJB4:SSX2IP:WDR77:ADAM15:ARHGEF2:NID1:ZFPL1:LRFN4:WBP4:FNDC3A:RNASEH2B:DCAF11:ADAMTS7:TGFB1I1:SLC47A1:C17orf70:CHAF1A:PDE4A:AP1M2:CC2D1A:DCAF15:MAST3:CHST8:FAM49A:MERTK:IGFBP5:COPS8:TYMP:ARMC8:KCNAB1:DHX29:DIMT1:ANKRA2:SYNE1:SEPT7:CREB3L2:RB1CC1
Curated_gene_sets	ZHOU_INFLAMMATORY_RESPONSE_FIMA_UP	505	41	4.047796791474934e-06	0.00022721357295820015	MFSD2A:SEMA6C:DCST2:ABL2:HECTD2:TLL2:CDON:ERC1:POSTN:NRL:STXBP6:KLHL28:KIAA1199:MESDC1:ABCC11:FPR2:BCL11A:AFF3:LSS:TAB1:ODF3B:CYP8B1:GSK3B:RAB6B:KCNAB1:LIPH:IL8:CXCL1:EIF4E:C5orf47:SLC22A23:EDN1:IER3:TNFAIP3:ITGB8:SEPT7:ZCWPW1:TSGA13:GEM:S1PR3:PAPPA
Curated_gene_sets	GARY_CD5_TARGETS_DN	435	37	4.112483179485371e-06	0.00022851282798332347	TARDBP:EBNA1BP2:DNAJB4:RPF1:WDR77:SOAT1:ARL5B:CUL2:CREM:EMG1:SLC11A2:IKBIP:BRI3BP:WBP4:DLEU1:IPO5:RBM25:ANP32A:MESDC2:DCTPP1:CYB5B:IFI35:KPNB1:MRPS12:BCL11A:AFF3:DDX27:ACKR4:TRMT10A:DHX29:IPO11:UTP15:RPP40:PPIL1:SOD2:RFC2:KIAA0020

Curated_gene_sets	KORKOLA_SEMINOMA_UP	44	10	4.23624180001397e-06	0.0002313823769560609	ERC1:NDUFA9:TPI1:SPSB2:ENO2:ATN1:PTPN6:EMG1:PHB2:GPRC5A
Curated_gene_sets	MILI_PSEUDOPODIA_HAPTOTAXIS_UP	506	41	4.2482494223890475e-06	0.0002313823769560609	PTP4A2:BTF3L4:DNAJB4:ZMYND11:CUL2:AASDHPPT:RBM7:MTMR6:WBP4:FNDC3A:KPNA3:FAM179B:FKBP3:RBM25:MAPKBP1:MYO5A:ARPP19:TCF12:ANKRD11:RABEP1:PPP1CB:KIAA1841:XPO1:PKP4:RPE:MFF:ESF1:CYB5R3:THUMPD3:C3orf38:MRPS22:COMMD8:NIPBL:KIF2A:CETN3:TTC1:PTP4A1:IRAK1BP1:SEPT7:ZNF34:KIAA0020
Curated_gene_sets	WONG_ADULT_TISSUE_STEM_MODULE	709	52	4.403299700372779e-06	0.00023747599658579076	MACF1:TIE1:MPL:TAL1:DNAJB4:F3:ASH1L:SELP:NID1:ZMYND11:REEP3:DUSP8:EHF:TRPC6:KCNA1:EMP1:LGR5:POSTN:LHFP:PCK2:FRMD6:TXNDC16:RABEP1:PHLPP1:CD97:MYCN:IL18R1:IGFBP5:TGM2:SHANK3:CAND2:HDAC11:CMTM8:CACNA2D2:RBP1:PTGER4:PPAP2A:SLC12A2:PPP1R18:FLOT1:IER3:CDKN1A:MAP7:HOXA10:TPST1:ABCB1:CALD1:GEM:KHDRBS3:KLF4:PBX3:NACC2
Curated_gene_sets	BRUINS_UVC_RESPONSE_LATE	1101	72	4.5416310593897054e-06	0.00024022608132406512	TARDBP:PTP4A2:MFSD2A:EBNA1BP2:BTF3L4:C1orf123:DDAH1:DPH5:BCAS2:SETDB1:SCNM1:UHMK1:SELP:CSRP1:FMOD:NID1:CREM:ACTA2:LINC00610:COMMD9:MRPL49:FDX1:C11orf88:IKBIP:UCHL3:DTD2:GTF2A1:MYO5A:PKD1:CD2BP2:RABEP1:FAM83G:MFAP4:IFI35:PSMD12:AZI1:MYL12A:IMPA2:TTC39C:EPG5:CHAF1A:DNAJB1:SARS2:GRIN2D:CTU1:HPCAL1:TPRKB:MTHFD2:WDR54:RTKN:PLEKHB2:RBMS1:DNAJC5:CDPF1:TGFBR2:HYAL2:SHQ1:GSK3B:SRPRB:MRPS22:RASA2:AREG:AREGB:PLAC8:PPAP2A:SLC12A2:TAPBP:PEX7:THBS2:MPLKIP:TPST1:ABCB1:GNG11:UBQLN1
Curated_gene_sets	REACTOME_INNATE_IMMUNE_SYSTEM	1101	72	4.5416310593897054e-06	0.00024022608132406512	CDC42:BCL10:VAV3:NRAS:SIKE1:CTSS:CTSK:HRNR:FCGR2A:HSPA6:FCGR3A:CFHR5:RAB18:PSAP:LINC00610:COMMD9:UBE2L6:HSPA8:PTPN6:C1S:METTL7A:NCKAP1L:RAP1B:APAF1:PSME1:PSEN1:MYO5A:CHRNB4:CCL17:NFATC3:CLEC10A:MAPK7:VAT1:KPNB1:TRIM25:PSMD12:MUC16:CD97:NFKBIB:CEACAM1:CD33:FPR2:ATP6V1B1:CREB1:SLC11A1:BPIFA2:PI3:SEMG1:SLPI:ZBP1:DNAJC5:SYNGR1:TAB1:CYB5R3:ARSA:PROS1:ARMC8:CXCL1:PLAC8:CFI:DIAPH1:HLA-A:TUBB:MUC21:C4A:RAB44:TNFAIP3:RNASET2:LAT2:CD36:LYN:FCN2:TUBB4B
Curated_gene_sets	LIU_PROSTATE_CANCER_DN	490	40	4.634317247919682e-06	0.00024177051500229242	NBL1:MFSD2A:SSX2IP:VAV3:CSRP1:NID1:ACTA2:GRK5:EHF:CLMP:ENO2:C1S:METTL7A:C12orf75:PCDH9:COCH:C14orf28:FRMD6:RASL12:TGFB1I1:SLC47A1:GJC1:SLC14A1:PDE4A:FBXO17:CXCL17:RBMS1:SEMG1:SEMG2:SYNGR1:MRAS:RBP1:MFI2:UBE2QL1:GSTA1:JAZF1:TPST1:HSPB1:CALD1:PPP3CC
Curated_gene_sets	CHICAS_RB1_TARGETS_CONFLUENT	562	44	4.658730156379385e-06	0.00024177051500229242	ACOT7:SPOCD1:PHGDH:LMNA:PEAR1:C10orf54:ACTA2:SMPD1:TENM4:C1S:GPRC5A:METTL7A:C12orf75:TNFRSF19:POSTN:LHFP:LMO7:PCK2:STXBP6:UBR7:ZNF469:MFAP4:SLC14A1:EPG5:MTHFD2:AFF3:TGM2:SLC2A4RG:APOBEC3B:FBLN1:CAND2:TGFBR2:GNAI2:RBP1:CDH6:ADAM19:TUBB:FLOT1:ARL4A:JAZF1:HSPB1:MEST:LY6E:TUBB4B
Curated_gene_sets	LINDGREN_BLADDER_CANCER_CLUSTER_2B	386	34	4.710759315142387e-06	0.00024218585974390908	LRRC8C:GJA5:CSRP1:ACTA2:GRK5:CRTAM:C1S:NUAK1:POSTN:LCP1:STXBP6:JMJD7-PLA2G4B:MYO5A:CLEC10A:MAPK7:CD97:RND3:SLC2A4RG:LRIG1:PROS1:RBP1:KLHL6:PDGFRB:ADAM19:PPP1R18:C4A:TNFAIP3:THBS2:TPST1:LAT2:GNG11:GEM:KLF4:PAPPA
Curated_gene_sets	DOUGLAS_BMI1_TARGETS_UP	563	44	4.874034573510034e-06	0.0002473021148487896	ICMT:PTP4A2:MACF1:MFSD2A:RUSC1:ASH1L:DAP3:RIT1:NID1:CDH23:CDON:HOXC11:PDE1B:LGR5:C12orf75:ULK1:MTMR6:GTF3A:KCTD12:TNFSF13B:CORO1A:POLR2C:CYB5B:MFAP4:RND2:TCF3:TUBB4A:CD320:PDE4A:FAM49A:UCKL1:IFRD2:HYAL2:ARL13B:RBP1:IPO11:THG1L:MGAT1:PTP4A1:ZNF92:BET1:PRKAR2B:NUDCD1:PAPPA
Curated_gene_sets	OSMAN_BLADDER_CANCER_DN	421	36	4.90018733294275e-06	0.0002473021148487896	RPL22:DPH5:CSDE1:PRUNE:ARHGEF2:FCRLA:ABL2:PEX16:FAU:C12orf57:IKBIP:KIAA0226L:EBPL:JMJD7-PLA2G4B:ARPP19:SIAH1:SLC7A6:PRPSAP2:RPL19:TUBD1:PITPNC1:RPL38:MYL12A:SAFB:EPS15L1:DEDD2:RPL32:TGFBR2:RPL14:MAP3K13:IL8:CXCL6:RPL37:RPS18:RPL12:FAM102A
Curated_gene_sets	GRAESSMANN_APOPTOSIS_BY_SERUM_DEPRIVATION_UP	547	43	5.378189940283721e-06	0.00026895838965000684	EBNA1BP2:WDR77:TOR3A:CREM:TACR2:FAS:ABCC8:DDX25:CDON:PTHLH:METTL7A:PDE1B:FNDC3A:KCTD12:TXNDC16:IFI35:KPNB1:TRIM25:OSR1:TPRKB:RND3:RPE:NCL:ZBP1:USP25:APOL2:KRBOX1:KRBOX1:LRIG1:CXCL6:TRMT10A:KLHL2:PTGER4:DIMT1:PDGFRB:SOD2:SEPT7:CLDN3:HSPB1:MEPCE:MEST:GFRA2:NACC2:TUBB4B
Curated_gene_sets	MILI_PSEUDOPODIA_CHEMOTAXIS_DN	423	36	5.447844977007918e-06	0.00026998734431099596	CLSTN1:TMEM57:ELOVL1:LRRC8C:F3:SMG5:FAM171A1:PSAP:ZFYVE27:SYVN1:TENM4:PHB2:C1S:SLC11A2:KDELC1:SCFD1:ERO1L:PKD1:SLC7A6:ANKRD11:SNED1:CDS2:RPN2:LIME1:NAGA:PLXNB2:GSK3B:DIAPH1:PDGFRB:TAPBP:C6orf89:PI16:CALD1:LY6E:PUF60:RPL12
Curated_gene_sets	WANG_LMO4_TARGETS_DN	355	32	5.514730658585687e-06	0.00027086190493642734	TARDBP:FBLIM1:CDC42:RPF1:RBM15:BCAS2:RAB18:PSAP:ACTA2:FDX1:GPRC5A:RASSF8:MTIF3:RBM25:CPSF2:RRN3:VAT1:NBR1:TUBD1:RNFT1:INSR:PLEKHB2:DDX27:ZNFX1:THUMPD3:TRMT10A:OCLN:PFDN6:BCAP29:EXOC4:RB1CC1:UBQLN1

Curated_gene_sets	REACTOME_NEUTROPHIL_DEGRANULATION	476	39	5.566331332607457e-06	0.00027097689080242144	NRAS:CTSS:HRNR:FCGR2A:HSPA6:RAB18:PSAP:LINC00610:COMMD9:HSPA8:PTPN6:METTL7A:NCKAP1L:RAP1B:APAF1:PSEN1:CHRNB4:VAT1:KPNB1:PSMD12:CD97:CEACAM1:CD33:FPR2:SLC11A1:SLPI:DNAJC5:SYNGR1:CYB5R3:ARSA:ARMC8:CXCL1:PLAC8:DIAPH1:HLA-A:TUBB:RAB44:RNASET2:CD36:TUBB4B
Curated_gene_sets	PECE_MAMMARY_STEM_CELL_DN	140	18	5.630395368324051e-06	0.0002716912712381632	BTF3L4:RUSC1:ARHGEF2:NID1:REXO2:EMP1:KLHL28:SLC7A6:RABEP1:NPEPPS:KPNB1:PUM2:SNED1:TMEM230:SHQ1:DCTD:CALD1:NSMAF
Curated_gene_sets	MCBRYAN_PUBERTAL_BREAST_4_5WK_UP	258	26	5.715393451844718e-06	0.0002733946032921547	RPL22:C1orf210:PDZK1IP1:F3:STAMBPL1:ACTA2:EHF:IKBIP:TXNDC16:ESRP2:CEACAM1:ATP6V1B1:RND3:IGFBP2:TGM2:SLPI:HDAC11:ATP2C1:RBP1:AREG:AREGB:PLAC8:OCLN:THBS2:INMT:CLDN3:CALD1
Curated_gene_sets	YAGI_AML_WITH_INV_16_TRANSLOCATION	407	35	5.831813799050504e-06	0.00027655868714290366	SPEN:HSPA6:TLL2:ZNF143:WEE1:SAC3D1:UCP3:OPCML:EMP1:PTHLH:TFCP2:RNASEH2B:RBM25:MAPKBP1:NFATC3:CLEC10A:RPL19:TAF4B:POU2F2:BCAM:KCNS1:RPL32:IFRD2:COPB2:MFI2:RPL37:PRSS16:RPS18:HSPB1:DUS4L:MEST:ASH2L:RB1CC1:LYN:LY6E
Curated_gene_sets	ENK_UV_RESPONSE_EPIDERMIS_DN	513	41	5.9301309189991235e-06	0.00027881752295225794	HOOK1:DNAJB4:BCL10:DDAH1:ABCD3:F3:VAV3:PHGDH:ANXA9:DUSP8:BBOX1:EHF:PRKRIR:YAP1:EMP1:POSTN:HERC2:NFATC3:NBR1:SPAG9:RPS6KB1:IMPA2:INSR:DNAJB1:TECR:ERF:NCOA1:BCL11A:PEX13:SLC2A4RG:LSS:DGCR6:PROS1:AREG:AREGB:PTGER4:PPAP2A:TNFAIP3:HECA:RNASET2:HSPB1:PBX3
Curated_gene_sets	BHATI_G2M_ARREST_BY_2METHOXYESTRADIOL_UP	115	16	6.628701520347161e-06	0.00030902107680872657	CDC20:PTBP2:NRAS:ASPM:ASRGL1:NDUFA9:DLGAP5:UBR7:PLEKHB2:PCNA:IER3:SOD2:FGFR1OP:GNG11:FAM3C:NACC2
Curated_gene_sets	FLECHNER_BIOPSY_KIDNEY_TRANSPLANT_REJECTED_VS_OK_DN	552	43	6.742870780927005e-06	0.0003095778057600758	EBNA1BP2:PDZK1IP1:ABCD3:SLC16A4:LAMTOR5:HIPK1:ZMYND11:WEE1:BBOX1:PC:HSPA8:METTL7A:FNDC3A:DLEU1:PCK2:MIA2:CTAGE5:PDXDC1:SMG1:PRPSAP2:VAT1:IMPA2:TECR:PRODH2:FAM168B:PLEKHB2:IGFBP2:IGFBP5:DSTN:CYB5R3:SLC4A4:AFM:GSTA2:GSTA1:PEX7:ZNF117:HSPB1:ABCB1:FAM3C:CREB3L2:TERF1:CA2:CALB1:PTPN3
Curated_gene_sets	GRAESSMANN_RESPONSE_TO_MC_AND_SERUM_DEPRIVATION_UP	214	23	6.7531969989473e-06	0.0003095778057600758	EBNA1BP2:TOR3A:TACR2:FAS:ABCC8:CDON:PTHLH:METTL7A:KCTD12:TXNDC16:IFI35:TRIM25:OSR1:NCL:ZBP1:USP25:APOL2:PDGFRB:SOD2:MEPCE:MEST:NACC2:TUBB4B
Curated_gene_sets	FARMER_BREAST_CANCER_APOCRINE_VS_LUMINAL	327	30	7.574293322136115e-06	0.0003428936483235919	ACOT7:PDZK1IP1:NRAS:ANXA9:SCGB1D2:UCP2:YAP1:ENO2:METTL7A:ANKLE2:UCHL3:ERO1L:IFI35:NBR1:NPEPPS:KPNB1:GREB1:MZT2A:PI3:SLPI:CACNA2D2:STK32B:IL8:NPY1R:C5orf30:MARCH3:ESR1:PRKAR2B:PIP:FCN2
Curated_gene_sets	BROWNE_HCMV_INFECTION_24HR_DN	143	18	7.604621904286168e-06	0.0003428936483235919	DDAH1:SLC16A4:PKLR:FDPS:ACTA2:TM7SF2:CDON:KIAA1199:B9D1:SLC14A1:IGFBP5:TGFBR2:PDGFRB:CDKN1A:HOXA11:MEST:CALD1:BLK
Curated_gene_sets	LANDEMAINE_LUNG_METASTASIS	21	7	7.674025968973518e-06	0.0003432098931327099	C1orf210:SIKE1:HORMAD1:GYLTL1B:CALB2:C21orf91:ITGB8
Curated_gene_sets	CHARAFE_BREAST_CANCER_LUMINAL_VS_MESENCHYMAL_DN	465	38	7.747520474049085e-06	0.00034370250103019366	FBLIM1:CDC20:STIL:DNAJB4:EXTL2:SOAT1:FAM171A1:STAMBPL1:ACTA2:FAS:GRK5:UBASH3B:CLMP:C1S:IKBIP:LHFP:KCTD12:IPO5:TGFB1I1:DHX33:KPNB1:SPAG9:RALBP1:PPP4R1:PDE4A:CD97:TGFBR2:PROS1:ATP10D:ARAP3:PPP1R18:TUBB:POPDC3:TNFAIP3:GNG11:CALD1:LYN:PAPPA
Curated_gene_sets	MASSARWEH_TAMOXIFEN_RESISTANCE_UP	575	44	8.286045583857159e-06	0.00036465229405438585	RNF207:C1orf64:TMEM50A:TMEM57:TRIM62:TSPAN1:DENND2C:HMGCS2:SFMBT2:AGAP4:AGAP8:FAS:SMPD1:REXO2:EMP1:SLC11A2:HOXC13:NUAK1:PSEN1:PDXDC1:BCAM:GRIN2D:NCOA1:TMEM87B:RND3:RBMS1:IGFBP5:SLC37A1:RAD18:TRAK1:IFT57:SLC12A2:ARAP3:EDN1:IER3:B3GALT4:FUT9:L3MBTL3:HOXA10:CD36:BCAP29:CREB3L2:RDH10:KLF4:PBX3
Curated_gene_sets	VECCHI_GASTRIC_CANCER_EARLY_UP	432	36	8.682542617333606e-06	0.00037906878522184255	EBNA1BP2:CDC20:PDZK1IP1:WDR77:CKS1B:ASPM:MCM10:ARL5B:STAMBPL1:SAC3D1:CDCA5:HSPA8:GPRC5A:UCHL3:DLGAP5:KIAA1199:NPIPA1:BRCA1:PI3:GTSE1:SHANK3:RAD18:SHQ1:MFI2:IL8:CXCL1:CKS1B:WRNIP1:SOD2:HOXA10:ZNF117:CLDN3:HSPB1:NCAPG2:CDH17:NUDCD1:SCRIB
Curated_gene_sets	GRAESSMANN_RESPONSE_TO_MC_AND_DOXORUBICIN_UP	596	45	9.226289636717158e-06	0.00039963637237465426	ICMT:LDLRAP1:SSX2IP:PHGDH:RUSC1:TOR3A:PHLDA3:FAS:CTR9:SAC3D1:DGAT2:ENO2:PTHLH:APAF1:ULK1:KCTD12:ERCC5:FBXO33:DCAF4:MAPKBP1:ADAMTS7:CORO1A:NFATC3:UBALD2:ENTHD2:PTGER1:DNAJB1:NCOA1:RPE:HES6:TGM2:GTSE1:PLXNB2:NAT6:HYAL2:RAB43:ACAD11:PSAPL1:CDKN1A:PTP4A1:IRAK1BP1:EXOC4:GEM:ST6GALNAC4:FAM102A
Curated_gene_sets	BROWNE_HCMV_INFECTION_20HR_UP	235	24	1.0294603874871947e-05	0.0004415031797356912	TRIM33:SIKE1:DUSP8:SOX5:PCDH9:GTF2A1:SMG1:NFATC3:SAFB2:PDE4A:ERF:FAM49A:IGFBP2:PPARA:TRMU:HYAL2:CGGBP1:ABCF3:MAP3K13:PCDHGA12:ABCB1:LYN:CA2:KHDRBS3

Curated_gene_sets	MACAEVA_PBMC_RESPONSE_TO_IR	106	15	1.0353373965806974e-05	0.0004415031797356912	F5:ACTA2:FAS:METTL7A:IKBIP:PRKAB1:SLC7A6:PTGER1:PCNA:CDS2:ANKRA2:CDKN1A:PTP4A1:PAPPA-AS1:ZNF79
Curated_gene_sets	KIM_ALL_DISORDERS_OLIGODENDROCYTE_NUMBER_CORR_UP	755	53	1.2278960210733738e-05	0.0005194634691436184	ACOT7:CLSTN1:NBL1:LAMTOR5:SCAMP3:FDPS:RUSC1:SYT11:CSRP1:SNRPE:FAM171A1:PSAP:SAC3D1:VPS51:FAU:MRPL49:TPI1:ENO2:SLC25A3:NUAK1:MTMR6:GTF3A:FOXG1:CORO1A:SH3GL1:TUBB4A:ARHGEF18:DNAJB1:TECR:MAST3:PAFAH1B3:HPCAL1:PUM2:NCL:DSTN:EIF6:MAPK8IP2:RPL32:MYRIP:TUSC2:AP2M1:RPL37:TTC1:SNCB:VPS52:ARL4A:TSC22D4:ZMAT4:CA2:LY6E:PUF60:FAM102A:TUBB4B
Curated_gene_sets	LOPEZ_MBD_TARGETS	952	63	1.2370426187568445e-05	0.0005194634691436184	HES2:HTR6:CDC42:TMEM50A:EBNA1BP2:BTF3L4:BCAS2:SIKE1:CTSK:FAM189B:YY1AP1:ARHGEF2:ZNF496:GRK5:IPO7:SLC43A1:ARL2:NAALADL1:TM7SF2:ZNHIT2:RCE1:FDX1:RBM7:HSPA8:USP5:RAP1B:FNDC3A:COMMD6:CORO1A:TGFB1I1:PLA2G15:KPNB1:PSMD12:EPS15L1:POU2F2:KDELR1:PPP1CB:IGFBP2:VIL1:PCNA:EIF6:TPD52L2:C2CD2:HDAC11:CYP8B1:HYAL1:CNBP:COPB2:MRFAP1:AREG:AREGB:TERT:RPL37:PFDN6:CDKN1A:PTP4A1:SEPT7:PPP3CC:STAR:PUF60:IPPK:XPA:ZNF79:UBAC1
Curated_gene_sets	SPIELMAN_LYMPHOBLAST_EUROPEAN_VS_ASIAN_DN	587	44	1.3799561191171295e-05	0.00057508625842904	CSDE1:CTSS:SCAMP3:DAP3:RIT1:SSR2:CREM:PSAP:FAS:GRK5:IPO7:UBE2L6:MRPL49:RBM7:HSPA8:PRKAB1:WBP4:LCP1:DLEU1:TGDS:IPO5:SCFD1:MYO5A:ANP32A:POLR2C:NFATC3:SPAG9:PSMD12:HPCAL1:PUM2:CREB1:RPN2:RTCB:APOL1:CHKB:CGGBP1:COPB2:KIF2A:SLC12A2:TTC1:STK19:BET1:ASH2L:PBX3
Curated_gene_sets	LASTOWSKA_COAMPLIFIED_WITH_MYCN	40	9	1.3995669709103768e-05	0.0005788735268404499	TPO:HPCAL1:NOL10:GREB1:MYCN:FAM49A:PLB1:TRMT61B:ALK
Curated_gene_sets	ZHOU_INFLAMMATORY_RESPONSE_LIVE_DN	372	32	1.4300941586821193e-05	0.0005870856691724132	LDLRAP1:ZNF362:DPH5:OLFM3:VAV3:TACR2:C10orf54:AASDHPPT:SPSB2:MTIF3:KIAA0226L:ARL11:MOAP1:UBR7:RASL12:ANP32A:COQ9:NFATC3:PLA2G15:SNAI3:DNAH9:MBP:PDE4A:MAST3:DEDD2:TRMT61B:THUMPD3:VGLL4:MFI2:ARAP3:CALB1:ST6GALNAC4
Curated_gene_sets	REACTOME_AXON_GUIDANCE	551	42	1.4450828749580831e-05	0.0005888445107514381	RPL22:CDC42:VAV3:NRAS:SEMA4A:ARHGEF11:ABL2:PSEN2:TUBB8:CUL2:FAU:TRPC6:HSPA8:PSME1:PSEN1:NUMB:RPL3L:SIAH1:MAPK7:CACNB1:RPL19:PSMD12:RPL38:MYL12A:MYL12B:TUBB4A:SCN3A:CREB1:SHANK3:RPL32:SCN5A:RPL14:GSK3B:AP2M1:RPL37:ISL1:RPS18:GFRA2:LYN:RPL7:RPL12:TUBB4B
Curated_gene_sets	GOZGIT_ESR1_TARGETS_DN	760	53	1.4717734834633286e-05	0.0005953107303332184	MACF1:TSPAN1:VAV3:CTSS:LMNA:C1orf85:DUSP8:UCP2:UCP3:HOXC13:NUAK1:ULK1:KIAA0226L:FRMD6:GPR137C:MYO5A:KIAA1199:GRIN2A:RABEP1:VAT1:EPS15L1:CEACAM1:GREB1:AFF3:RND3:IGFBP5:VIL1:TGM2:SLC37A1:APOL1:FOXP1:ZPLD1:PLCXD2:C3orf65:AREG:AREGB:EIF4E:UBE2QL1:PPAP2A:SLC12A2:EDN1:C4A:GSTA1:FUT9:TNFAIP3:ESR1:ARL4A:ITGB8:HOXA10:CLDN3:CREB3L2:SPATA31A4:SPATA31A7:DIRAS2:FAM102A
Curated_gene_sets	BLALOCK_ALZHEIMERS_DISEASE_DN	1244	77	1.500496991224859e-05	0.0006024988283743029	ACOT7:CDC42:TMEM50A:TM2D1:LPHN2:LRRC8B:LAMTOR5:BCAS2:RUSC1:CUL2:IPO7:AASDHPPT:HSPA8:TPI1:ENO2:DDX47:LETMD1:SLC25A3:WBP4:KPNA3:UCHL3:IPO5:FOXG1:SCFD1:FKBP3:NUMB:MOAP1:HERC2:MGA:ARPP19:GRIN2A:NPEPPS:KPNB1:UTP18:PSMD12:RPL38:MYL12A:MYL12B:PUM2:BCL11A:SCN3A:RPE:MFF:COPS8:PCNA:CDS2:EIF6:RPN2:USP25:SYNGR1:EIF1B:ENTPD3:RPL14:NDUFB4:ATP2C1:KCNAB1:AP2M1:PTGER4:KIF2A:DIMT1:NDFIP1:TTC1:KCNIP1:CNR1:PEX7:RNASET2:NEUROD6:ABCB1:BET1:PRKAR2B:GPR22:BCAP29:ASH2L:RB1CC1:ENY2:DIRAS2:TUBB4B
Curated_gene_sets	DAVICIONI_TARGETS_OF_PAX_FOXO1_FUSIONS_UP	257	25	1.5777267768271645e-05	0.000625807915673903	DDAH1:VAV3:CSRP1:PSEN2:NID1:CREM:EMP1:RAP1B:NTAN1:TGFB1I1:HPCAL1:MYCN:FAM49A:ALK:SCN3A:APOBEC3B:STK32B:MARCH3:ADAM19:TNFAIP3:HOXA11:PRKAR2B:FAM3C:DENND2A:KLF4
Curated_gene_sets	KRIGE_RESPONSE_TO_TOSEDOSTAT_24HR_UP	762	53	1.581299768745183e-05	0.000625807915673903	C1orf63:TM2D1:PHGDH:CTSS:TNFAIP8L2:RIT1:SEMA4A:ZBTB41:C10orf54:UBE2L6:METTL7A:LETMD1:CSRNP2:UHRF1BP1L:MTMR6:PCK2:MIA2:CTAGE5:NUMB:MYO5A:TCF12:PKD1:NPIPA1:N4BP1:NBR1:UBALD2:MYL12A:CCDC130:DCAF15:FPR2:NOL10:NCOA1:KIAA1841:MTHFD2:IL18R1:IL18RAP:TMEM87B:ARFGAP3:CELSR1:CHKB:NPHP3:IL8:KLHL2:RPL37:ANKRA2:CPEB4:ZNF311:HLA-A:FLOT1:CDKN1A:MAP7:HECA:KLF4:TOR4A
Curated_gene_sets	HOFFMANN_LARGE_TO_SMALL_PRE_BII_LYMPHOCYTE_UP	151	18	1.624145831953094e-05	0.0006381733015409978	CDC20:SSX2IP:CKS1B:NUCKS1:MCM10:CDCA5:CIT:IPO5:PCK2:DLGAP5:ANP32A:DCTPP1:XPO1:MTHFD2:NCAPH2:CKS1B:TUBB:CDKN1A:TUBB4B
Curated_gene_sets	BLUM_RESPONSE_TO_SALIRASIB_DN	341	30	1.7074253427989216e-05	0.0006661380716834657	CDC20:STIL:ABCD3:VAV3:LMNA:TPI1:LPCAT3:POLE:FOXG1:COCH:FKBP3:ARPP19:ANP32A:BRCA1:NPEPPS:KPNB1:CHAF1A:PDE4A:DDX39A:TECR:PAFAH1B3:PCNA:BFSP1:DBR1:PDGFRB:TUBB:RFC2:HSPB1:MEST:TUBB4B

Curated_gene_sets	CHARAFE_BREAST_CANCER_LUMINAL_VS_MESENCHYMAL_UP	446	36	1.7340516767177874e-05	0.0006717618502552499	TMEM125:TSPAN1:HOOK1:VAV3:ANXA9:FAM63A:RUSC1:UBQLN4:SEMA4A:TMEM79:EHF:TM7SF2:PTPN6:METTL7A:ULK1:PCK2:DCAF11:KLHL28:PDXDC1:ABCC11:ESRP2:AP1M2:CBLC:IGFBP2:PER2:SLC37A1:SYNGR1:PLA2G12A:OCLN:RGL2:MAP7:ESR1:CLDN3:CLDN4:ZNF467:FAM102A
Curated_gene_sets	RIZKI_TUMOR_INVASIVENESS_2D_DN	62	11	1.7866708388012046e-05	0.0006873060338633165	NBL1:BBOX1:PRKRIR:ULK1:SMG1:MFAP4:TRIM25:CBLC:VIL1:NCAPH2:RPL12
Curated_gene_sets	LINDGREN_BLADDER_CANCER_CLUSTER_1_DN	378	32	1.9667965467836107e-05	0.0007513435974900445	ACOT7:TMEM125:CDCP2:CERS2:PRUNE:CKS1B:ADAM15:GRK5:ENO2:UHRF1BP1L:CIT:POLE:DLGAP5:PDXDC1:BRCA1:PAFAH1B3:MTHFD2:APOBEC3B:GTSE1:KLHL6:CXCL1:PLAC8:CKS1B:SLC12A2:PDGFRB:ADAM19:MGAT1:CLDN4:CREB3L2:NCAPG2:CA2:GEM:PAPPA
Curated_gene_sets	PILON_KLF1_TARGETS_UP	485	38	1.9916662542451573e-05	0.0007555969699725939	DNAJB4:DENND2C:SETDB1:CLK2:LARP4B:DUSP8:HPX:ABCC8:C1S:LHFP:CPSF2:BOLA2B:CIAPIN1:RNF157:RALBP1:NAPG:INSR:PUM2:MTHFD2:RBMS1:IGFBP2:C2CD2:CYP2D6:RAD18:RBP1:AFM:TRMT10A:CFI:KIF2A:LECT2:CPEB4:SLC22A23:GNG11:PRKAR2B:CALD1:KEL:NUDCD1:NTMT1
Curated_gene_sets	BLUM_RESPONSE_TO_SALIRASIB_UP	245	24	2.0617700300065004e-05	0.0007768354065113533	PTP4A2:PHGDH:DAP3:ARHGEF2:ZFPL1:CSRNP2:SCFD1:PSEN1:MYO5A:PKD1:RRN3:SIAH1:NAPG:MTHFD2:USP25:EIF1B:DCTD:MGAT1:STK19:CDKN1A:TNFAIP3:DUS4L:NSMAF:RPL12
Curated_gene_sets	FISCHER_DIRECT_P53_TARGETS_META_ANALYSIS	311	28	2.1194418188273805e-05	0.0007931326153312531	BCL10:LCE1E:LMNA:F5:PHLDA3:ADCK3:ACTA2:FAS:TRUB1:SAC3D1:MRPL49:YAP1:IKBIP:APAF1:PRKAB1:ENTHD2:C17orf89:POU2F2:GREB1:PCNA:PLXNB2:ANKRA2:IER3:CDKN1A:PTP4A1:SCRIB:ZNF79:CEL
Curated_gene_sets	NUYTTEN_EZH2_TARGETS_UP	1031	66	2.1905356255853093e-05	0.000814198410563837	ICMT:CDC42:PTP4A2:DNAJB4:BCL10:PTBP2:ABL2:SOAT1:PSEN2:ARL5B:FAS:GRK5:UBE2L6:FDX1:TPI1:C1S:NUAK1:LHFP:UCHL3:GPR180:ABHD13:FRMD6:RBM25:PAPLN:RABEP1:SPAG9:TRIM25:TMEM145:CEACAM1:PPP1CB:RND3:FBXO36:RPN2:TGM2:PI3:ZNFX1:LSS:APOL2:PLXNB2:TYMP:VGLL4:TGFBR2:ZNF654:ARMC8:MRAS:XRN1:FNDC3B:MAP3K13:SLC4A4:IL8:CXCL1:EDN1:TAPBP:CDKN1A:TNFAIP3:SYNE1:SOD2:JAZF1:FAM3C:MEST:PPP3CC:GEM:CHMP5:KLF4:PBX3:FAM102A
Curated_gene_sets	WEST_ADRENOCORTICAL_TUMOR_UP	295	27	2.208783821679291e-05	0.0008154711277219986	ICMT:ACOT7:PTP4A2:MED8:EXTL2:WDR77:NRAS:FDPS:DAP3:ASPM:TFCP2:KPNA3:PLA2G15:CHAF1A:DCAF15:DDX39A:DNAJB1:XPO1:MTHFD2:PLEKHB2:RPN2:TPD52L2:LSS:SRPRB:TNFAIP3:FGFR1OP:TUBB4B
Curated_gene_sets	COLINA_TARGETS_OF_4EBP1_AND_4EBP2	346	30	2.2517930456583453e-05	0.0008258075696111039	CDC20:DDAH1:F3:TOR3A:GRK5:ENO2:C1S:POLE:POSTN:DLGAP5:IFI35:TRIM25:GRWD1:OSR1:XPO1:IGFBP2:TGM2:ZBP1:ATP10D:CXCL6:SKIV2L2:SLC12A2:TSPAN17:C4A:AIG1:ABCB1:GNG11:KHDRBS3:LY6E:UBQLN1
Curated_gene_sets	SENESE_HDAC3_TARGETS_DN	544	41	2.3602299414199206e-05	0.0008493476200255673	CDC42:PHGDH:MSTO1:NID1:CTR9:UCP2:YAP1:DYRK4:TMEM120B:BRI3BP:STXBP6:C14orf28:GTF2A1:ANP32A:TGFB1I1:NFATC3:CYB5B:EPN2:KPNB1:TCF3:AP1M2:BCAM:FAM49A:NCOA1:PKP4:DAW1:C21orf91:CYB5R3:FBLN1:COMMD8:PLAC8:HDAC3:NDFIP1:SYNE1:SEPT7:LAT2:BET1:FAM3C:CEP41:RP11-203J24.9:ST6GALNAC6:NACC2
Curated_gene_sets	FOSTER_KDM1A_TARGETS_DN	200	21	2.3754251277706616e-05	0.0008493476200255673	CCDC28B:CDC20:DDX25:DLGAP5:UBR7:ARPP19:CORO1A:SNAI3:TNRC6C:BCAM:DUSP11:MTHFD2:MZT2A:TGM2:DDX27:CHKB:NAT6:RASSF1:AP2M1:ANKRA2:ZCWPW1
Curated_gene_sets	SMID_BREAST_CANCER_BASAL_DN	696	49	2.3971539747684398e-05	0.0008493476200255673	PTP4A2:TSPAN1:VAV3:HMGCS2:ANXA9:HPX:ABCC8:SCGB1D2:TM7SF2:UCP2:TTC12:GPRC5A:PTHLH:AQR:JMJD7-PLA2G4B:LONP2:SMPD3:RABEP1:B9D1:MFAP4:CACNG1:SSTR2:LIPE:BCAM:GREB1:SNED1:FBLN1:CELSR1:HDAC11:ENTPD3:TRAK1:RASSF1:CACNA2D2:LRIG1:KCNAB1:LRRC31:STK32B:AREG:AREGB:NPY1R:CCNO:C5orf30:C4A:CNR1:ESR1:HSPB1:CD36:ABCB1:PIP:LRRC6
Curated_gene_sets	GOBERT_OLIGODENDROCYTE_DIFFERENTIATION_UP	563	42	2.397441453063157e-05	0.0008493476200255673	CDC20:TAL1:STIL:LPHN2:DDAH1:CKS1B:PMF1:ASPM:NUCKS1:MCM10:STAMBPL1:WEE1:CDCA5:PRKRIR:PTPN6:EMP1:LGR5:CIT:POLE:DLGAP5:DCTPP1:AZI1:IMPA2:PAFAH1B3:GRWD1:XPO1:TTLL4:NCL:GTSE1:NCAPH2:RAD18:CAND2:IFRD2:SHQ1:ISY1:DCTD:CDH6:CKS1B:MDC1:RFC2:ABCB1:NCAPG2:TERF1
Curated_gene_sets	UEDA_PERIFERAL_CLOCK	170	19	2.4000029402775862e-05	0.0008493476200255673	FDPS:PSEN2:MCM10:FDX1:SLC11A2:LMO7:ANP32A:MESDC2:LONP2:CYB5B:RBMS1:HES6:PER2:PPARA:CYP8B1:PLA2G12A:TUBB:C6orf89:PTP4A1
Curated_gene_sets	RODRIGUES_THYROID_CARCINOMA_POORLY_DIFFERENTIATED_DN	774	53	2.4128683441647558e-05	0.0008493476200255673	ZNF362:TM2D1:SAMD13:CTSK:GON4L:CSRP1:FMOD:ACTA2:VPS51:CDON:GPRC5A:EMP1:METTL7A:SLC25A3:KCTD12:MIA2:CTAGE5:LINS:TARSL2:LONP2:MBP:RFX1:DEDD2:BCAM:ZNF611:PPP1CB:PKP4:SNED1:RALGAPA2:SLPI:DNAJC5:FBLN1:CHKB:VGLL4:HDAC11:TGFBR2:TRAK1:TUSC2:LRIG1:FOXP1:NDUFB4:MAN2B2:SLAIN2:SLC4A4:DCTD:PTGER4:PPAP2A:OCLN:C4A:PEX7:TNFAIP3:RNASET2:ITGB8:ENY2

Curated_gene_sets	MIKKELSEN_MEF_HCP_WITH_H3K27ME3	582	43	2.4240606497730244e-05	0.0008493476200255673	HES2:RIMKLA:TAL1:FOXE3:PAQR6:SFRP5:ABCC8:DBX1:GYLTL1B:HOXC13:LRFN5:SIX6:OTUD7A:SKOR1:ACAN:GRIN2A:CALB2:SLC47A1:IKZF3:ADAM11:TBX21:TUBB4A:AP1M2:CNFN:CBLC:ALK:MMP24:KCNS1:CELSR1:ODF3B:MAPK8IP2:SCN5A:MYRIP:CACNA2D2:FGF12:STK32B:SLC34A2:NPY5R:CDX1:KCNIP1:SNCB:ZMAT4:CALB1
Curated_gene_sets	MARKEY_RB1_ACUTE_LOF_UP	232	23	2.513502632409528e-05	0.0008751125304357477	TARDBP:CKS1B:NUCKS1:ZMYND11:FAS:LMO7:TGDS:IPO5:DLGAP5:CPSF2:DCTPP1:BRCA1:GSK3A:XPO1:PCNA:DSTN:FBLN1:IFRD2:NIPBL:PTGER4:KIF2A:CKS1B:SLC12A2:TUBB
Curated_gene_sets	CREIGHTON_ENDOCRINE_THERAPY_RESISTANCE_3	717	50	2.5452986964852415e-05	0.0008806093163122838	NBL1:TMEM57:RIMKLA:MED8:TSPAN1:LPHN2:LRRC8B:HMGCS2:GOLPH3L:CTSS:F5:SOAT1:FAM171A1:EHF:SCGB2A1:SLC11A2:PRKAB1:TNFRSF19:NIPA1:PDXDC1:CXCL17:CEACAM1:MYCN:TTLL4:SLPI:SLC37A1:ARFGAP3:PPARA:ENTPD3:TRAK1:RAB6B:GK5:LRRC31:TMEM44:STIM2:IL8:KLHL2:MARCH3:SLC12A2:CPEB4:GSTA1:POPDC3:TPST1:FAM3C:CREB3L2:LYN:RDH10:LRRC6:SLC35D2:PTPN3
Curated_gene_sets	GARY_CD5_TARGETS_UP	473	37	2.6355864053768714e-05	0.0009061475509986357	CDC42:TM2D1:PHGDH:UHMK1:TOR3A:SLC43A1:ARL2:UCP2:ENO2:PTPN6:SOX5:LETMD1:LHFP:PCK2:ANKRD11:MBP:UBXN6:PKN1:POU2F2:KDELR1:HPCAL1:LSS:PPARA:CMTM8:TRAK1:GNAI2:GSK3B:PPP1R18:FLOT1:CDKN1A:ZFAND3:TNFAIP3:ASL:PILRB:LRSAM1:ST6GALNAC4:FAM102A
Curated_gene_sets	GAVIN_FOXP3_TARGETS_CLUSTER_P3	157	18	2.7635606686834653e-05	0.0009442451700886796	SEMA4A:PEAR1:PSEN2:CREM:DNAJB13:DGAT2:POU2AF1:IKBIP:DHX37:RNF166:PHLPP1:MBP:POU2F2:IL18R1:PKP4:CD200R1:ZNF608:ITGB8
Curated_gene_sets	SWEET_KRAS_ONCOGENIC_SIGNATURE	89	13	2.8137387333400744e-05	0.000954415195057806	CTSS:FCGR2A:PHB2:PSEN1:HNF1B:PAFAH1B3:CEACAM1:KDELR1:MYCN:RPL14:SLC12A2:CDKN1A:FAM3C
Curated_gene_sets	BROWNE_HCMV_INFECTION_18HR_UP	172	19	2.828025391645562e-05	0.000954415195057806	OVGP1:KIAA0907:HSPA6:CREM:PTHLH:TGDS:TMEM251:SH3GL3:HELZ:ERF:MTHFD2:IL1RL1:MRAS:IL8:NPY1R:FGFR1OP:LYN:TERF1:PTPN3
Curated_gene_sets	IGLESIAS_E2F_TARGETS_UP	143	17	2.8644294856762838e-05	0.000958460728405252	CDC42:CTSS:CKS1B:LMNA:CSRP1:ACTA2:EMP1:POSTN:LCP1:IGFBP5:PCNA:DSTN:ARSA:GNAI2:RBP1:CKS1B:TUBB:SEPT7
Curated_gene_sets	CHEN_LVAD_SUPPORT_OF_FAILING_HEART_UP	102	14	2.8748594834914848e-05	0.000958460728405252	DDAH1:NID1:METTL7A:POSTN:MFAP4:INSR:MTHFD2:RND3:HYAL2:FNDC3B:AREG:AREGB:PPAP2A:CDKN1A:CALD1
Curated_gene_sets	JOHNSTONE_PARVB_TARGETS_3_DN	859	57	2.937726205765074e-05	0.0009735199914405827	TMEM50A:EBNA1BP2:CDC20:BCL10:PTBP2:RBM15:CKS1B:RIT1:UHMK1:ASPM:NUCKS1:CREM:ALDH18A1:YAP1:TPI1:UHRF1BP1L:KPNA3:UCHL3:G2E3:COCH:DTD2:FRMD6:DLGAP5:MYO5A:TCF12:PSMD12:RALBP1:ZNF765:PPP1CB:TPRKB:MTHFD2:PLEKHB2:RBMS1:RPE:COPS8:PER2:PANK2:PCNA:NDUFA6:EIF1B:ATP2C1:MRPS22:GK5:KCNAB1:MAP3K13:EIF4E:C5orf22:KIF2A:DIMT1:CKS1B:UTP15:CETN3:FLOT1:RFC2:PRKAR2B:NCAPG2:NUDCD1:ENY2
Curated_gene_sets	CREIGHTON_ENDOCRINE_THERAPY_RESISTANCE_1	531	40	2.98521402023602e-05	0.000983333073372356	DPH5:CLK2:ARHGEF2:UBQLN4:ASPM:SNRPE:ARL2:TRPC6:ERC1:METTL7A:NUAK1:GTF3A:COMMD6:IPO5:PCK2:FKBP3:FRMD6:DCAF4:MYO5A:NTAN1:DCTPP1:DHX33:RPL19:EPS15L1:GREB1:AFF3:DDX27:HDAC11:UBA5:AREG:AREGB:NPY1R:PTGER4:PPP1R18:TUBB:ESR1:ARL4A:HSPB1:PPP1R35:MEST:CA2
Curated_gene_sets	IVANOVA_HEMATOPOIESIS_STEM_CELL_AND_PROGENITOR	550	41	3.0315769756775248e-05	0.0009926610085239324	HES2:MTOR:MPL:FAM189B:SYT11:ARHGEF2:ARL5B:CREM:TACR2:ASRGL1:UBASH3B:FKBP3:ARPP19:SEPT1:SEPT1:SLC7A6:MBTD1:TAF4B:INSR:IL12RB1:POU2F2:KDELR1:KCNJ14:TSKS:MYCN:PKP4:PER2:C21orf91:C2CD2:SYNGR1:TYMP:CAND2:DVL3:KIAA1239:C4A:SOD2:ITGB8:ABCB1:GIMAP5:KHDRBS3:ZNF34:KLF4
Curated_gene_sets	GAVIN_FOXP3_TARGETS_CLUSTER_P6	90	13	3.176639040587919e-05	0.0010340054060517243	ACOT7:CDC20:CKS1B:ASPM:MCM10:CDCA5:TPI1:FBXW8:IL12RB1:TGFBR2:DCTD:CKS1B:ABCB1:NCAPG2
Curated_gene_sets	NABA_MATRISOME	1024	65	3.2545858424913754e-05	0.0010531456893850032	ANGPTL7:VWA5B1:CDCP2:OVGP1:CTSS:CTSK:ANXA9:SEMA6C:HRNR:FLG:ADAM15:SEMA4A:BGLAP:F13B:FMOD:NID1:GDF2:TLL2:SFRP5:HABP2:DMBT1:HPX:POSTN:TNFSF13B:COCH:PAPLN:ADAMTS7:ACAN:HAPLN3:CCL17:CLEC10A:MFAP4:ADAM11:SERPINB4:MUC16:PODNL1:MEGF8:TPO:IGFBP2:IGFBP5:SNED1:MMP24:TGM2:PI3:SLPI:FBLN1:PLXNB2:HYAL3:HYAL1:HYAL2:ZPLD1:FGF12:IL8:CXCL6:PF4V1:CXCL1:AREG:AREGB:MEGF10:ADAM19:MUC21:THBS2:COL22A1:ADAMTSL1:PAPPA:FCN2
Curated_gene_sets	BONOME_OVARIAN_CANCER_SURVIVAL_OPTIMAL_DEBULKING	236	23	3.294207147992749e-05	0.0010597329544507668	ELOVL1:ARNT:ANXA9:FLG:TRPC6:CSRNP2:PCK2:KIAA1199:MYL12B:SLC14A1:TCF3:SH3GL1:TUBB4A:NCOA1:ACKR4:STK32B:COQ2:CFI:MCTP1:FLOT1:STK19:HOXA10:LY6E

Curated_gene_sets	RODWELL_AGING_KIDNEY_UP	497	38	3.393152655194006e-05 0.0010852170207105948	LDLRAP1:TSPAN1:PRUNE:C10orf54:FAS:FAU:PTPN6:C1S:IKBIP:NUAK1:KIAA0226L:CORO1A:RNF166:CLEC10A:TCF3:CD97:FAM49A:LMF2:TYMP:ODF3B:SCN5A:MRAS:RBP1:TM4SF18:CXCL1:CDH6:MGAT1:HLA-A:PPP1R18:C4A:WDR46:TAPBP:JAZF1:CLDN3:LYN:LY6E:RPL12:TOR4A
Curated_gene_sets	FISCHER_DREAM_TARGETS	966	62	3.5743589708541174e-05 0.001136563508593555	CDC20:STIL:TXNDC12:PTBP2:CKS1B:PMF1:ASPM:NUCKS1:MCM10:WEE1:CDCA5:PRKRIR:HSPA8:EMG1:CIT:POLE:KPNA3:DLEU1:RNASEH2B:GPR180:G2E3:FANCM:GPR137C:DLGAP5:UBR7:METTL16:BRCA1:KPNB1:SPAG9:TUBD1:CHAF1A:DCAF15:DDX39A:PAFAH1B3:GRWD1:ANAPC1:PKP4:TTLL4:PCNA:ZNFX1:APOBEC3B:GTSE1:NCAPH2:RAD18:IFRD2:RASSF1:ARL13B:EIF4E:NIPBL:KIF2A:IPO11:CKS1B:ANKRA2:UTP15:C5orf30:MDC1:TUBB:WDR46:ZNF92:RFC2:EXOC4:NCAPG2:PBX3
Curated_gene_sets	LU_EZH2_TARGETS_UP	287	26	3.700572486644865e-05 0.001169933864886977	C1orf210:PHGDH:LMNA:TOR3A:ECHDC3:FAM25E:FAM25D:PEX16:UBE2L6:PCK2:SH3GL3:POLR2C:IFI35:WDR54:C2CD2:NCAPH2:HYAL3:HYAL2:CYB561D2:XXcos-LUCA11.4:SLC25A26:TSPAN17:FLOT1:IER3:STK19:PFDN6:SOD2:DENND2A
Curated_gene_sets	REACTOME_ASPARAGINE_N_LINKED_GLYCOSYLATION	304	27	3.7405271118324993e-05 0.001175522075418401	F5:SEC16B:TUBB8:SYVN1:SCFD1:MIA2:CTAGE5:TRAPPC2L:DERL2:ASGR2:NAPG:ST8SIA5:TUBB4A:CHST8:KDELR1:DCTN1:RPN2:ARFGAP3:TMEM115:COPB2:AREG:AREGB:MGAT1:BET1:COPG2:RP11-203J24.9:ST6GALNAC6:ST6GALNAC4:DPM2:TUBB4B
Curated_gene_sets	CONCANNON_APOPTOSIS_BY_EPOXOMICIN_UP	238	23	3.760986825552419e-05 0.001175522075418401	DNAJB4:DAP3:RIT1:ARHGEF2:LMNA:HSPA6:CREM:ACTA2:FAS:C1S:GTF2A1:SMG1:DGKE:PSMD12:DNAJB1:TGFBR2:LRIG1:TTC1:CDKN1A:HSPB1:MEST:GEM:NACC2
Curated_gene_sets	XU_GH1_AUTOCRINE_TARGETS_DN	132	16	3.836443173697329e-05 0.0011923318586728252	HOOK1:IPO7:SCGB2A1:RBM7:CIT:ULK1:GPR180:RPS6KB1:DDX39A:FOXP1:GP9:RPL37:SLC22A23:CA2:PTPN3:FAM102A
Curated_gene_sets	BROWNE_HCMV_INFECTION_48HR_DN	482	37	3.934560663553172e-05 0.0012159560792250562	ARHGEF11:ZFPL1:UCP3:DYRK4:POSTN:LMO7:CORO1A:TGFB1I1:SPG7:IFI35:GJC1:TCF3:RFX1:PTGER1:SIGLEC6:FPR2:ZNF135:DCTN1:FAM168B:IGFBP5:PER2:SNED1:UCKL1:DGCR6:NDUFA6:TRAK1:HYAL1:ATP2C1:MAN2B2:CXCL6:CDH6:PTP4A1:THBS2:CRHR2:PIP:LY6E:CEL
Curated_gene_sets	REACTOME_NEURONAL_SYSTEM	410	33	4.03643380429586e-05 0.0012404705227615379	KCNC4:NRAS:HCN3:TUBB8:KCNJ11:ABCC8:LRFN4:HSPA8:KCNA1:PRKAB1:KCNK10:CHRNB4:GRIN2A:CACNB1:GJC1:KCNJ16:KCNJ2:TUBB4A:GRIN2D:KCNJ14:CREB1:KCNS1:DNAJC5:SHANK3:GNAI2:CACNA2D2:KCNAB1:AP2M1:FLOT1:GNG11:PRKAR2B:KCNV2:TUBB4B
Curated_gene_sets	CHEMNITZ_RESPONSE_TO_PROSTAGLANDIN_E2_UP	147	17	4.0905373504913205e-05 0.001250113664725153	LRRC8C:SCNM1:ASPM:STAMBPL1:DLGAP5:TGFB1I1:GJC1:CHAF1A:FBXO17:BOLA3:MTHFD2:ANAPC1:MZT2A:GTSE1:ARAP3:ADAM19:NSMAF
Curated_gene_sets	WANG_SMARCE1_TARGETS_UP	290	26	4.41105388385904e-05 0.0013406191942048938	FBLIM1:SPOCD1:KCNC4:NID1:ACTA2:TLL2:C1S:TNFRSF19:POSTN:KLHL28:ACAN:SERPINB4:BCAM:RBMS1:IGFBP5:MFF:KLHDC7B:PROS1:PLSCR4:ATP10D:CFI:RPL37:ITGB8:CALD1:S1PR3:FAM102A
Curated_gene_sets	MARTENS_TRETINOIN_RESPONSE_DN	835	55	4.9063701366742896e-05 0.001482963852848641	TARDBP:IQCC:ELOVL1:C1orf52:CERS2:SCNM1:SNRPE:WEE1:SLC43A1:TM7SF2:ZNHIT2:HSPA8:PHB2:LETMD1:HOXC11:ORAI1:COCH:C14orf142:ZNF770:TGFB1I1:RABEP1:FAM211A:MAPK7:RPL19:MYL12A:OR7G2:KEAP1:CARD8:GRWD1:KCNJ14:PCNA:SLC2A4RG:SCO2:ODF3B:THUMPD3:CAND2:RPL32:RPL14:LSMEM2:HYAL3:RASSF1:CYB561D2:XXcos-LUCA11.4:GTPBP8:CNBP:IL8:RPL37:PTP4A1:HOXA11:MPLKIP:LAT2:HSPB1:MEPCE:RPL7:ZNF79:FAM166A
Curated_gene_sets	REACTOME_INFECTIOUS_DISEASE	379	31	5.05155256863282e-05 0.0015185022229535052	RPL22:FAU:KPNA3:IPO5:PSME1:GTF2A1:SH3GL3:RPL3L:POLR2C:RPL19:KPNB1:PSMD12:RPL38:TAF4B:TCEB3C:TCEB3B:SH3GL1:AP1M2:XPO1:CHMP4B:RPL32:RPL14:AP2M1:RPL37:HLA-A:RPS18:GTF2H5:RPL7:CHMP5:RPL12:NELFB
Curated_gene_sets	SENESE_HDAC3_TARGETS_UP	488	37	5.099891972251624e-05 0.0015206954066572292	ASH1L:UHMK1:CREM:FAS:WEE1:UBASH3B:EMP1:SLC25A15:KLHL28:SMG1:METTL16:SLC14A1:SERPINB4:ZNF480:PLEKHB2:IGFBP5:PI3:TGFBR2:GSK3B:RAB43:TM4SF18:SLAIN2:IL8:UBE2QL1:ADAM19:CPEB4:FLOT1:TNFAIP3:SOD2:ITGB8:ZNF92:ESYT2:CALB1:GEM:KHDRBS3:ADAMTSL1:PAPPA
Curated_gene_sets	BOCHKIS_FOXA2_TARGETS	415	33	5.1141365248425265e-05 0.0015206954066572292	ZBTB17:DNASE2B:F13B:PSEN2:ADCK3:GPAM:HABP2:HPX:UBE2L6:DCAF11:PSME1:COCH:MIA2:CTAGE5:MAPKBP1:POLR2C:ESRP2:BRCA1:MERTK:PER2:CYP2D6:PPARA:TUSC2:PROS1:STX19:NR1I2:RAB43:CFI:WDR46:CALD1:GIMAP5:LYN:CHMP5:SLC25A25
Curated_gene_sets	FORTSCHEGGER_PHF8_TARGETS_UP	276	25	5.188042417810205e-05 0.0015343774914179536	DNAJB4:DDAH1:VAV3:PHGDH:TMEM79:CRB1:FAM171A1:UCP3:LINC01059:GRAMD1B:GPR180:PCK2:DCAF4:MESDC2:GLP2R:KPNB1:MBTD1:INSR:MTHFD2:TGFBR2:TUSC2:GSK3B:TRMT10A:PTP4A1:MAP7:BET1

Curated_gene_sets	SCHAEFFER_PROSTATE_DEVELOPMENT_48HR_DN	398	32	5.350943111332127e-05	0.0015740929441410711	TMEM57:RIMKLA:LMNA:C10orf54:SYT13:DGAT2:TRPC6:UBASH3B:ERC1:PTHLH:PDE1B:NUAK1:STXBP6:LRFN5:TGFB1I1:MFAP4:SLC14A1:MYCN:FAM49A:RND3:PKP4:PER2:FBLN1:TGFBR2:KCNAB1:PLA2G12A:CDH6:IPO11:PDGFRB:ESR1:ZMAT4:PAPPA
Curated_gene_sets	GRAESSMANN_APOPTOSIS_BY_SERUM_DEPRIVATION_DN	228	22	5.6219523671366095e-05	0.0016450191474265154	C1orf123:F3:FCRLA:DGAT2:TENM4:ENO2:ERO1L:ANP32A:ARMC5:KCNJ14:BCL11A:HES6:PER2:TGM2:NAT6:HYAL1:COPB2:MAN2B2:KIF2A:CRHR2:CLDN3:ST6GALNAC4
Curated_gene_sets	GRUETZMANN_PANCREATIC_CANCER_DN	197	20	5.931590173571169e-05	0.001726437965334127	PSEN2:SMPD1:SLC25A3:PCK2:INSR:NCOA1:DSTN:LSS:LMF2:MAPK8IP2:ARSA:RPL32:RBP1:KCNAB1:NPY1R:PTP4A1:EVX1:PRKAR2B:MEST:PBX3
Curated_gene_sets	MEISSNER_BRAIN_HCP_WITH_H3K27ME3	263	24	6.460011686040866e-05	0.0018703433834163583	HES2:FOXE3:FOXD2:SFRP5:DBX1:GYLTL1B:HOXC13:HOXC12:HOXC11:SIX6:CALB2:SLC47A1:HNF1B:IKZF3:TBX21:CBLC:CELSR1:CDX1:HOXA10:HOXA11:HOXA13:EVX1:CLDN3:FOXB2
Curated_gene_sets	BENPORATH_ES_WITH_H3K27ME3	1112	68	6.738846825952953e-05	0.001928430790665339	HES2:NBL1:HTR6:CSMD2:RIMKLA:TAL1:FOXE3:FOXD2:DDAH1:VAV3:KCNC4:ADAM15:LRRC71:HSPA6:CDH23:SFRP5:GRK5:DUSP8:ABCC8:DDX25:KCNA1:PTHLH:HOXC12:HOXC11:PDE1B:LGR5:POLE:STXBP6:FOXG1:LRFN5:SIX6:KIAA1199:ACAN:SMPD3:HNF1B:IKZF3:TBX21:SSTR2:ST8SIA5:CHST8:GRIN2D:OSR1:OSBP2:MAPK8IP2:ENTPD3:KCNAB1:STK32B:SLC10A4:NPY1R:NPY5R:CDH6:PTGER4:ISL1:PCDHGC4:CDX1:HLA-A:FUT9:ESR1:SYNE1:THBS2:HOXA10:HOXA13:EVX1:GIMAP5:FAM167A:ANKRD18B:KLF4:PAPPA
Curated_gene_sets	NIKOLSKY_BREAST_CANCER_17Q21_Q25_AMPLICON	332	28	6.755203708465702e-05	0.001928430790665339	SPAG9:MBTD1:UTP18:C17orf67:DGKE:TRIM25:TUBD1:RPS6KB1:RNFT1:CACNG1:HELZ:PSMD12:PITPNC1:KCNJ16:KCNJ2:SSTR2:RPL38:TTYH2:DNAI2:KIF19:RNF157:TNRC6C:AZI1:ENTHD2:C17orf89:FSCN2:C17orf70:NPLOC4
Curated_gene_sets	RICKMAN_TUMOR_DIFFERENTIATED_MODERATELY_VS_POORLY_UP	124	15	6.787106629483651e-05	0.001928430790665339	NBL1:CTSK:EMG1:IPO5:HEATR5A:ZNF469:VAT1:AP1M2:ANAPC1:NAGA:ENTPD3:TRAK1:LIPH:CARD6:CPEB4
Curated_gene_sets	SARRIO_EPITHELIAL_MESENCHYMAL_TRANSITION_DN	153	17	6.800864813471655e-05	0.001928430790665339	C1orf63:PDZK1IP1:VAV3:PSAP:PC:C1S:PCDH9:PSME1:TCF3:CXCL1:RPL37:ZNF608:TNFAIP3:SOD2:CALD1:CA2:GEM
Curated_gene_sets	WHITE_NEUROBLASTOMA_WITH_1P36.3_DELETION	20	6	6.896551687914575e-05	0.0019455349146265681	RPL22:RNF207:ICMT:GPR153:ACOT7:HES2
Curated_gene_sets	BUYTAERT_PHOTODYNAMIC_THERAPY_STRESS_DN	648	45	6.979453365811336e-05	0.0019588761717004165	ICMT:TARDBP:CCDC28B:IQCC:TSPAN1:SSX2IP:EXTL2:WDR77:TOR3A:NID1:MCM10:SAC3D1:TM7SF2:UCP2:C11orf71:LCP1:DLEU1:KDELC1:TMEM251:UBR7:ANP32A:CORO1A:DCTPP1:PRMT7:HNF1B:MPPE1:PKP4:IGFBP5:TTLL4:TGM2:DGCR6:APOL1:TGFBR2:HYAL2:TUSC2:ASTE1:PCDHGC3:EDN1:TUBB:RNASET2:HOXA10:SEPT7:CALD1:XPA:DPM2
Curated_gene_sets	BENPORATH_SUZ12_TARGETS	1031	64	7.142376410915385e-05	0.001994427037383022	HES2:NBL1:HTR6:RIMKLA:TAL1:FOXE3:FOXD2:DDAH1:LRRC8C:KCNC4:LRRC71:HSPA6:ZMYND11:CDH23:TLL2:SFRP5:ABCC8:TRPC6:KCNA1:PTHLH:HOXC13:HOXC12:HOXC11:LGR5:POLE:SIAH3:MLNR:STXBP6:FOXG1:COCH:LRFN5:SIX6:KIAA1199:ACAN:ANKRD11:HNF1B:IKZF3:TBX21:KCNJ2:SSTR2:CHST8:GRIN2D:HPCAL1:OSR1:IGFBP5:DGCR6:MAPK8IP2:KCNAB1:STK32B:SLC10A4:ZAR1:CXCL1:NPY1R:NPY5R:CDH6:PTGER4:ISL1:KLF14:GIMAP5:GFRA2:RDH10:ANKRD18B:KLF4:PAPPA
Curated_gene_sets	REACTOME_TRANSPORT_OF_SMALL_MOLECULES	728	49	7.362417201037705e-05	0.002040310147522486	LDLRAP1:LRRC8B:LRRC8C:ABCD3:SOAT1:KCNJ11:SLC43A1:ARL2:TRPC6:PHB2:SLC11A2:PSME1:NIPA2:NIPA1:TRPM1:ABCC11:SLC7A6:SPG7:TRPV1:DERL2:SLC5A10:SLC47A1:SLC47A2:PSMD12:TTYH2:SLC14A1:ATP6V1B1:SLC4A5:SLC9A4:SLC11A1:SMDT1:LMF2:SLC25A26:ATP2C1:AP2M1:SLC34A2:ATP10D:SLC4A4:MTTP:SLC6A19:SLC6A18:SLC12A2:ABCB1:GNG11:PRKAR2B:PIP:CA2:SLC35D2:SLC34A3
Curated_gene_sets	REACTOME_ER_TO_GOLGI_ANTEROGRADE_TRANSPORT	154	17	7.380871102653604e-05	0.002040310147522486	F5:SEC16B:TUBB8:SCFD1:MIA2:CTAGE5:TRAPPC2L:NAPG:TUBB4A:KDELR1:DCTN1:ARFGAP3:TMEM115:COPB2:AREG:AREGB:BET1:COPG2:TUBB4B
Curated_gene_sets	REACTOME_SRP_DEPENDENT_COTRANSLATI	111	14	7.430611311830338e-05	0.0020437896413189344	RPL22:SSR2:FAU:RPL3L:RPL19:RPL38:RPN2:RPL32:RPL14:SRPRB:RPL37:RPS18:RPL7:RPL12

	ONAL_PROTEIN_TARGETING_TO_MEMBRANE					
Curated_gene_sets	REACTOME_TRANSPORT_TO_THE_GOLGI_AND_SUBSEQUENT_MODIFICATION	185	19	7.688834298868121e-05	0.0021042924118444546	F5:SEC16B:TUBB8:SCFD1:MIA2:CTAGE5:TRAPPC2L:NAPG:TUBB4A:CHST8:KDELR1:DCTN1:ARFGAP3:TMEM115:COPB2:AREG:AREGB:MGAT1:BET1:COPG2:TUBB4B
Curated_gene_sets	KEGG_MAPK_SIGNALING_PATHWAY	267	24	8.184002071467294e-05	0.00222872254431394	CDC42:NRAS:HSPA6:FAS:DUSP8:HSPA8:RAP1B:PLA2G4B:JMJD7-PLA2G4B:MAPK7:CACNB1:CACNG1:TAB1:MAPK8IP2:TGFBR2:CACNA2D2:MRAS:RASA2:MAP3K13:FGF12:PLA2G12A:PDGFRB:HSPB1:PPP3CC
Curated_gene_sets	BOQUEST_STEM_CELL_CULTURED_VS_FRESH_UP	426	33	8.454662007375558e-05	0.002291088458254825	DDAH1:LRRC8B:CTSS:CTSK:HSPA6:ASPM:SLC43A1:GPRC5A:METTL7A:LMO7:COCH:DLGAP5:RASL12:KIAA1199:ACAN:MFAP4:DNAJB1:PER2:TGM2:KCNAB1:IL8:CXCL1:CFI:NPY1R:POPDC3:TNFAIP3:ESR1:SOD2:PRKAR2B:GEM:KLF4:PAPPA:NACC2
Curated_gene_sets	DEBIASI_APOPTOSIS_BY_REOVIRUS_INFECTION_UP	302	26	8.645811495195176e-05	0.0023314024036798363	TM2D1:BCL10:NRAS:SIKE1:CREM:DUSP8:WEE1:UBE2L6:PRKRIR:YAP1:TGDS:ARPP19:N4BP1:IFI35:BRCA1:PEX13:RND3:RPE:IL8:CXCL1:EIF4E:CETN3:TNFAIP3:FGFR1OP:BET1:GEM
Curated_gene_sets	DAVICIONI_MOLECULAR_ARMS_VS_ERMS_UP	337	28	8.739608020481106e-05	0.0023451992058861736	ACOT7:PTBP2:ARHGEF2:NHLH1:PSEN2:ASRGL1:CSRNP2:TMEM120B:PSME1:HERC2:ARPP19:COQ9:TRPV1:NPEPPS:CHST8:MYCN:NCOA1:ALK:BCL11A:PKP4:CDS2:PPAP2A:MARCH3:MDC1:CNR1:POPDC3:PRKAR2B:TSTD2
Curated_gene_sets	REACTOME_G_ALPHA_S_SIGNALLING_EVENTS	539	39	8.844847023440018e-05	0.0023619176444632786	HTR6:OR14K1:REEP3:GRK5:OR51T1:OR4C15:OR4C16:OR4P4:OR4S2:OR4C6:OR5T1:OR8H1:OR8K3:OR8K1:OR8J1:OR8U1:OR5R1:PTHLH:PDE1B:OR10P1:GLP2R:OR1M1:OR7G2:OR7G1:OR7G3:PDE4A:RLN3:OR10H2:OR10H3:FSHR:MC3R:GNAI2:PTGER4:OR10C1:CRHR2:GNG11:OR1L3:OR1L4:OR1L6
Curated_gene_sets	REACTOME_IRE1ALPHA_ACTIVATES_CHAPERONES	50	9	9.148647066406877e-05	0.0024312419088069674	ZBTB17:EXTL2:LMNA:SYVN1:PLA2G4B:SULT1A3:GSK3A:DCTN1:SRPRB
Curated_gene_sets	MOOTHA_HUMAN_MITODB_6_2002	430	33	0.00010090726070750257	0.002668705967076787	MTOR:HMGCS2:SMCP:DAP3:ALDH18A1:MRPL49:PC:UCP2:UCP3:FDX1:REXO2:NDUFA9:SLC25A3:APAF1:SDS:SLC25A15:PCK2:NPIPA1:CYB5B:SPG7:MRPS12:MTHFD2:NDUFA6:CYB5R3:LMF2:NCAPH2:CPT1B:NDUFB4:MRPS22:SOD2:STAR:CYP11B1:CYP11B2
Curated_gene_sets	MIKKELSEN_NPC_HCP_WITH_H3K27ME3	341	28	0.00010688006048284884	0.0028131445584504856	HES2:FOXE3:SFRP5:DBX1:TRPC6:HOXC13:LRFN5:SIX6:SKOR1:SNAI3:STAC2:IKZF3:TBX21:S1PR5:ALK:BCL11A:CELSR1:SCN5A:CACNA2D2:ISL1:CPNE5:DACT2:HOXA11:HOXA13:EVX1:FAM167A:FOXB2:PAPPA
Curated_gene_sets	BROWNE_HCMV_INFECTION_14HR_DN	289	25	0.00010863586668342817	0.0028457423934549445	C1orf123:LPHN2:LMNA:FAS:GRK5:TM7SF2:DYRK4:CIT:SPAG9:FSCN2:PTGER1:ZNF135:APOBEC3B:CAND2:TRAK1:RASA2:TERT:CDH6:NIPBL:SYNE1:HOXA11:ASL:CLDN4:GNG11:BLK
Curated_gene_sets	LINSLEY_MIR16_TARGETS	206	20	0.00011028750394436304	0.0028753154464357398	MACF1:F3:FAM189B:UHMK1:TRUB1:WEE1:RCE1:SLC11A2:COMMD6:LMO7:PLEKHM1:CDS2:ZNFX1:DNAJC5:IFRD2:TUSC2:IPPK:RP11-203J24.9:ST6GALNAC6:UBAC1:NACC2
Curated_gene_sets	LIM_MAMMARY_STEM_CELL_UP	470	35	0.00011429526872275526	0.002965746571905079	NBL1:TIE1:LRRC8C:PHLDA3:FMOD:C10orf54:ACTA2:FAS:GYLTL1B:CLMP:POSTN:LHFP:KDELC1:RASL12:PKD1:TGFB1I1:STAC2:GJC1:TTYH2:ERF:OSR1:RND3:IGFBP2:PROS1:DZIP1L:PPAP2A:PCDHGC3:PPP1R18:CDKN1A:TPST1:CD36:GNG11:MEST:CALD1:GEM
Curated_gene_sets	DAZARD_RESPONSE_TO_UV_NHEK_UP	223	21	0.00011604689378770585	0.0029970608578693426	HMGCS2:CSRP1:SNRPE:PCK2:CHRNB4:RPL38:TUBB4A:CEACAM1:IL1RL1:TYMP:EIF1B:IL8:CXCL1:RPL37:IER3:TNFAIP3:CLDN4:GEM:CYP11B1:LY6E:TUBB4B
Curated_gene_sets	KARLSSON_TGFB1_TARGETS_DN	191	19	0.00011770611918450871	0.0030257072973550584	C1orf63:TXNDC12:SSX2IP:KCNC4:SIKE1:ARNT:SYT11:DUSP8:TPI1:NBR1:TECR:ANAPC1:PCNA:TGM2:USP25:PLXNB2:NDFIP1:C4A:LY6E
Curated_gene_sets	DELACROIX_RARG_BOUND_MEF	361	29	0.00011899088098857584	0.003044506215433282	NBL1:DPH5:CERS2:ANXA9:NID1:ECHDC3:YAP1:SPSB2:GPRC5A:EMP1:RPL3L:SHPK:SHPK:CACNB1:RPL19:PSMD12:RFX1:PKN1:LIPE:OSR1:RND3:ZBP1:CYP2D6:ARSA:NDFIP1:HOXA10:HOXA13:INMT:CPA1:RB1CC1
Curated_gene_sets	OKAWA_NEUROBLASTOMA_1P36_31_DELETION	22	6	0.0001244543859643768	0.0031378805758907405	RPL22:RNF207:ICMT:GPR153:ACOT7:HES2

Curated_gene_sets	MCBRYAN_PUBERTAL_BREAST_6_7WK_DN	76	11	0.0001245821739779981	0.0031378805758907405	FBLIM1:CDC20:F3:ACTA2:EHF:MBP:IGFBP2:SLPI:IFT57:SLC4A4:AREG:AREGB
Curated_gene_sets	BAELDE_DIABETIC_NEPHROPATHY_DN	435	33	0.00012534148674474675	0.0031378805758907405	F3:GJA5:GRK5:UBE2L6:AASDHPPT:C1S:GPRC5A:LETMD1:POSTN:N4BP1:NFATC3:IFI35:MYL12A:NAPG:ARHGEF18:NPHS1:BCAM:XPO1:MTHFD2:RBMS1:IGFBP2:DSTN:RPN2:APOL1:PLXNB2:CAND2:LRIG1:DCTD:PTGER4:TAPBP:PRKAR2B:CALD1:CREB3L2
Curated_gene_sets	PASINI_SUZ12_TARGETS_DN	309	26	0.00012539961231400706	0.0031378805758907405	PHC2:ELOVL1:F3:SYT11:SOAT1:CSRP1:REEP3:ACTA2:GPRC5A:NUAK1:TNFRSF19:LHFP:KDELC1:TCF12:TGFB1I1:VAT1:RND3:RBMS1:IGFBP5:RBP1:ARAP3:PPP1R18:IER3:CDKN1A:CALD1:RDH10
Curated_gene_sets	STEIN_ESRRA_TARGETS	529	38	0.00012549240623449607	0.0031378805758907405	HOOK1:SIKE1:FAM63A:FDX1:RBM7:NDUFA9:LPCAT3:METTL7A:LETMD1:SLC25A3:ULK1:UBR7:DCTPP1:CIAPIN1:COQ9:TUBD1:IMPA2:CCDC130:DCAF15:MRPS12:PLEKHB2:MFF:SYNGR1:TGFBR2:GTPBP8:MRPS22:PLA2G12A:DCTD:MARCH3:IER3:SOD2:RNASET2:CLDN3:PRKAR2B:TERF1:CA2:ST6GALNAC4:UBAC1
Curated_gene_sets	RICKMAN_METASTASIS_UP	345	28	0.00013016787873848946	0.0032400610902281923	RPL22:RBM15:ZMYND11:DYRK4:LRRC23:RASSF8:SLC11A2:RBM25:MGA:RASL12:PDXDC1:NBR1:PKP4:CREB1:METTL21A:RPE:MFF:NCL:COPS8:SNED1:CAND2:C3orf17:SENP2:ZNF92:TPST1:PILRB:MEST:GEM
Curated_gene_sets	CAIRO_HEPATOBLASTOMA_CLASSES_UP	608	42	0.00013437174861999284	0.003315116673301418	RPL22:CDC20:DPH5:WDR77:ASPM:MCM10:ALDH18A1:IPO7:TPI1:EMG1:PHB2:SLC25A3:IPO5:DLGAP5:CIAPIN1:B9D1:RPL19:KPNB1:UTP18:RPS6KB1:CHAF1A:DDX39A:KDELR1:MYCN:NCL:ESF1:RPN2:DDX27:TPD52L2:TRMU:RPL14:COMMD8:THG1L:RPP40:WDR46:RFC2:CREB3L2:NCAPG2:RB1CC1:ENY2:SCRIB:PUF60
Curated_gene_sets	REACTOME_ADAPTIVE_IMMUNE_SYSTEM	807	52	0.00013456007772308414	0.003315116673301418	MTOR:CDC42:CDC20:BCL10:NRAS:CTSS:CTSK:FCGR3A:TUBB8:CUL2:HECTD2:NCR3LG1:UBE2L6:CRTAM:SPSB2:PTPN6:RAP1B:FBXW8:ORAI1:LMO7:PSME1:HERC2:KBTBD13:SIAH1:NFATC3:NPEPPS:PSMD12:TUBB4A:KEAP1:AP1M2:NFKBIB:FBXO17:CD79A:CD33:SIGLEC6:DCTN1:ANAPC1:CD200R1:UBA5:AP2M1:KLHL2:KIF2A:HLA-A:TAPBP:CD36:PILRB:PILRA:BLK:LYN:LRSAM1:UBAC1:TUBB4B
Curated_gene_sets	FARMER_BREAST_CANCER_BASAL_VS_LULMINAL	328	27	0.0001349911170368147	0.003315116673301418	CDC20:TSPAN1:STIL:PHGDH:ANXA9:MCM10:FAM171A1:BBOX1:TM7SF2:LONP2:RABEP1:GREB1:PPP1CB:BCL11A:MTHFD2:TTLL4:PI3:CELSR1:CACNA2D2:IL8:AREG:AREGB:C5orf30:ESR1:SOD2:HSPB1:LYN:KIAA0020
Curated_gene_sets	MORI_SMALL_PRE_BII_LYMPHOCYTE_UP	77	11	0.0001404590659066933	0.0034340680957898662	FCRLA:POU2AF1:SLC11A2:MYO5A:TCF12:CACNG1:PAFAH1B3:DUSP11:NCAPH2:FGF12:PSAPL1
Curated_gene_sets	SANSOM_APC_TARGETS_DN	348	28	0.00015050677382309898	0.0036634414283224226	NBL1:SETDB1:FAS:KCNJ11:PC:MOGAT2:POU2AF1:SLC11A2:DAO:ERCC5:NUMB:CORO1A:KEAP1:IL12RB1:CD79A:SLC4A5:MERTK:SLPI:CYB5R3:PPARA:ARSA:CGGBP1:RBP2:SLC4A4:MTTP:PPAP2A:LAT2:ZNF467
Curated_gene_sets	IVANOVA_HEMATOPOIESIS_EARLY_PROGENITOR	440	33	0.0001549698170162416	0.003755457988574207	RPL22:TXNDC12:EXTL2:DPH5:ABL2:CSRP1:ECHDC3:CUL2:WEE1:ASRGL1:ARL2:FDX1:BRI3BP:ANKLE2:SLC25A15:KDELC1:RFX7:CYB5B:METTL16:RNF157:IMPA2:MRPS12:TTLL4:NCL:TRAK1:DCTD:DIMT1:LSM11:L3MBTL3:PEX7:ZNF34:ADAMTSL1:DIRAS2
Curated_gene_sets	SCHUETZ_BREAST_CANCER_DUCTAL_INVASIVE_UP	349	28	0.00015789100426213764	0.003801644514165182	NBL1:CTSS:CTSK:FCGR2A:NID1:FAS:GRK5:C1S:EMP1:NUAK1:POSTN:LHFP:KCTD12:KIAA1199:TGFB1I1:KCNJ2:MERTK:TGM2:TGFBR2:PROS1:PLSCR4:ATP10D:PDGFRB:ADAM19:THBS2:CALD1:LYN:GEM
Curated_gene_sets	ZHENG_BOUND_BY_FOXP3	478	35	0.0001582578792481052	0.003801644514165182	MACF1:LRRC8C:SELP:CREM:REEP3:FAS:UBASH3B:LCP1:HEATR5A:AQR:TCF12:ANKRD11:SPG7:ZPBP2:EPS15L1:C21orf91:VGLL4:TGFBR2:LRIG1:C3orf38:IFT57:CD200R1:GPRIN3:NIPBL:PTGER4:ZNF608:L3MBTL3:TNFAIP3:ARL4A:PRKAR2B:FAM3C:ESYT2:PPP3CC:NSMAF:TMEM71
Curated_gene_sets	DURAND_STROMA_NS_UP	164	17	0.00016044269096839313	0.0038207586277797865	FBLIM1:MFSD2A:OVGP1:CTSK:HECTD2:UCP2:GPRC5A:PCDH9:LMO7:TNFSF13B:OSR1:RALGAPA2:RAB6B:AIG1:ZNF34:S1PR3:KLF4
Curated_gene_sets	PHONG_TNF_RESPONSE_VIA_P38_PARTIAL	164	17	0.00016044269096839313	0.0038207586277797865	DNAJB4:GPRC5A:PTHLH:LGR5:LHFP:DLEU1:CREB1:FNDC3B:IL8:CXCL1:PLAC8:PTGER4:IER3:TAPBP:CDKN1A:TNFAIP3:TPST1
Curated_gene_sets	BIOCARTA_PTDINS_PATHWAY	23	6	0.00016303418301790638	0.003865737244747858	ARHGEF2:RPS6KB1:GSK3A:GSK3B:AP2M1:LYN
Curated_gene_sets	CHEMNITZ_RESPONSE_TO_PROSTAGLANDIN_E2_DN	386	30	0.0001640311644839952	0.003872684273933294	ICMT:CLSTN1:NBL1:TMEM125:C1orf210:TSPAN1:SAMD13:ASRGL1:C12orf75:FNDC3A:LMO7:STXBP6:MIA2:CTAGE5:CYB5B:PRPSAP2:EPN2:RND2:BCL11A:WFDC5:SLPI:LIME1:GK5:OCLN:CDKN1A:MAP7:ITGB8:CLDN4:EXOC4:KLF4:PAPPA

Curated_gene_sets	WAMUNYOKOLI_OVARIAN_CANCER_LMP_DN	196	19	0.00016522599784996443	0.0038842231374899756	C1orf63:BTF3L4:EXTL2:CTSK:ARNT:NUCKS1:AASDHPPT:CDON:C1S:KIAA0226L:SPAG9:XPO1:RBMS1:PROS1:PLSCR4:PLA2G12A:PPAP2A:SOD2:CALD1
Curated_gene_sets	MEISSNER_NPC_HCP_WITH_H3K4ME2	480	35	0.00017139014498065153	0.003994988082790526	GPR153:HTR6:MFSD2A:RIMKLA:PEAR1:CSRP1:SLC16A12:TLL2:SYT13:UCP2:CLMP:PTHLH:OTUD7A:CALB2:FAM211A:ADAM11:MBP:GRIN2D:BCL11A:UCKL1:SLC37A1:OSBP2:RAB43:FGF12:TERT:SOX30:SNCB:B3GALT4:POPDC3:MAP7:PEX7:AIG1:ZMAT4:LY6E:DIRAS2
Curated_gene_sets	ENK_UV_RESPONSE_KERATINOCYTE_DN	480	35	0.00017139014498065153	0.003994988082790526	PTP4A2:MACF1:ZMYND11:IPO7:AASDHPPT:LPCAT3:PTHLH:SLC11A2:DLEU1:TGDS:IPO5:ERCC5:SCFD1:MIA2:CTAGE5:MYO5A:ANP32A:SIAH1:CYB5B:NPEPPS:GSK3A:PUM2:XPO1:ZNF638:RND3:CREB1:TGFBR2:TUSC2:GSK3B:ATP2C1:KIF2A:CETN3:BET1:ASH2L:TERF1:XPA
Curated_gene_sets	GRAHAM_CML_DIVIDING_VS_NORMAL_QUIESCENT_DN	92	12	0.00017265906918288002	0.004007584555168873	CREM:EMP1:PCDH9:TGFB1I1:BCL11A:IL18R1:IL8:CXCL6:CXCL1:AREG:AREGB:PTGER4:SOD2
Curated_gene_sets	MUELLER_PLURINET	298	25	0.000175446219449245	0.004055166610043264	WDR77:PHGDH:CKS1B:PMF1:SNRPE:MCM10:WEE1:HSPA8:PTPN6:ERCC5:FKBP3:ANP32A:DCTPP1:KPNB1:CHAF1A:NFKBIB:MRPS12:ANAPC1:NCL:PCNA:CKS1B:DIAPH1:RPP40:PFDN6:ARL4A:RFC2
Curated_gene_sets	SWEET_LUNG_CANCER_KRAS_DN	406	31	0.00017653254759879278	0.004063203114397318	MACF1:TIE1:NID1:ZMYND11:ACTA2:FAS:GPAM:GRK5:TNFRSF19:POSTN:KCTD12:NUMB:SIAH1:TCF3:CD97:PPP1CB:SLC4A5:IGFBP2:IGFBP5:ACKR2:ACKR4:RBP1:PPAP2A:PDGFRB:EDN1:INMT:GNG11:GFRA2:CA2:TMEM71:KLF4
Curated_gene_sets	DACOSTA_UV_RESPONSE_VIA_ERCC3_UP	316	26	0.0001792868285938176	0.004109403517060794	C1orf63:C1orf123:CREM:SMPD1:SAC3D1:ZFPL1:TM7SF2:CSRNP2:PCDH9:MOAP1:CYB5B:TRIM25:MRPS12:PAFAH1B3:PER2:PCNA:LMF2:SCO2:HYAL2:NPRL2:ABCF3:MGAT1:HLA-A:ASL:RFC2:LY6E
Curated_gene_sets	BRUINS_UVC_RESPONSE_VIA_TP53_GROUP_D	264	23	0.0001810979299577441	0.004133691753931744	SPEN:RHCE:HOOK1:EXTL2:PHGDH:FMOD:RBM20:TPI1:LGR5:TNFSF13B:RBM25:MBP:CNFN:PPP1CB:TRAK1:GSK3B:UBA5:UBE2QL1:SLC22A23:LAT2:CPA1:BLK:IZUMO3
Curated_gene_sets	SWEET_LUNG_CANCER_KRAS_UP	463	34	0.0001847316734069966	0.004188540942000025	ELOVL1:F3:CTSS:CTSK:SOAT1:PSAP:ZNF143:TPI1:PHB2:LPCAT3:LCP1:PSME1:MIA2:CTAGE5:PSEN1:HNF1B:MBTD1:PAFAH1B3:CEACAM1:KDELR1:FPR2:VIL1:NCL:SLPI:CYB5R3:PLXNB2:SLC34A2:SLC4A4:AREG:AREGB:SLC12A2:RPS18:CDKN1A:RNASET2:CLDN3:FAM3C
Curated_gene_sets	SASAKI_ADULT_T_CELL_LEUKEMIA	166	17	0.00018578512813088638	0.004188540942000025	ICMT:EXTL2:SYT11:FAS:EMP1:PTHLH:TFCP2:DLGAP5:NOMO1:CORO1A:SSTR2:MRPS12:MYCN:PCNA:ATP2C1:CETN3:TUBB
Curated_gene_sets	MIKKELSEN_IPS_LCP_WITH_H3K4ME3	166	17	0.00018578512813088638	0.004188540942000025	ELOVL1:ACOT11:PKLR:SEMA4A:C1orf85:ARHGEF11:F5:PTPN6:MYLPF:SEPT1:SEPT1:PIRT:IFI35:PDE4A:PODNL1:CD79A:PDGFRB:NYAP1
Curated_gene_sets	HAMAI_APOPTOSIS_VIA_TRAIL_UP	657	44	0.00018722769372307713	0.004203834870084275	TRIM33:BCAS2:PHGDH:ASPM:FMOD:NID1:CUL2:ASRGL1:EMP1:WBP4:KIAA0226L:IPO5:ERCC5:PRPF39:DLGAP5:TRPM1:MYO5A:RABEP1:BRCA1:PSMD12:RALBP1:ZNF83:ZNF611:XPO1:ZNF638:CREB1:NCL:ESF1:USP25:RAD18:THUMPD3:FAM208A:ZNF654:PROS1:NIPBL:DHX29:SKIV2L2:CETN3:TTC1:ZNF117:BCAP29:CALD1:RB1CC1:KIAA0020
Curated_gene_sets	YAO_TEMPORAL_RESPONSE_TO_PROGESTERONE_CLUSTER_2	55	9	0.00019621037420174136	0.004387614912535687	TARDBP:PKLR:TACR2:CDON:SOX5:DTD2:MYLPF:ESR1:THBS2
Curated_gene_sets	HAMAI_APOPTOSIS_VIA_TRAIL_DN	199	19	0.00020119327183029702	0.004480826673435076	UBIAD1:FOXD2:VAV3:ECHDC3:SAC3D1:PCK2:OTUD7A:SH3GL3:PITPNC1:SSTR2:ERF:GRWD1:FBLN1:TYMP:MAPK8IP2:TERT:ARAP3:MEPCE:TSC22D4
Curated_gene_sets	ACEVEDO_LIVER_TUMOR_VS_NORMAL_ADJACENT_TISSUE_UP	862	54	0.0002040343523222371	0.004525778113405751	CDC20:TM2D1:DPH5:NRAS:GOLPH3L:SCNM1:CKS1B:SCAMP3:KIAA0907:SSR2:LMNA:C1orf85:F5:CFHR5:F13B:ASPM:PSEN2:CUL2:STAMBPL1:CTR9:REXO2:C12orf57:BIVM:STXBP6:ARPP19:DCTPP1:POLR2C:XPO1:ESF1:NAGA:ARFGAP3:ARSA:EIF1B:IFRD2:NAT6:TUSC2:LRIG1:COPB2:DVL3:SENP2:LSG1:DROSHA:DHX29:CKS1B:MDC1:FLOT1:PFDN6:GSTA2:GSTA1:MPLKIP:GNG11:CREB3L2:RDH10:CHMP5:TUBB4B
Curated_gene_sets	LEI_MYB_TARGETS	319	26	0.00020808847441764058	0.004576555287874042	TARDBP:CTSK:HSPA6:ACTA2:TM7SF2:MRPL49:PTHLH:NFATC3:RPS6KB1:MYL12A:SERPINB4:DNAJB1:MRPS12:PCNA:TGM2:PI3:IL8:FLOT1:CDKN1A:CLDN3:HSPB1:GNG11:CREB3L2:LY6E:PUF60:DPM2
Curated_gene_sets	VANOEVELEN_MYOGENESIS_SIN3A_TARGETS	216	20	0.00020923252396130138	0.004576555287874042	CKS1B:DAP3:C10orf54:USP5:LPCAT3:UCHL3:C14orf142:SLC7A6OS:MBTD1:SH3GL1:ERF:PKP4:CREB1:LRIG1:DCTD:CDH6:CKS1B:NRM:STK19:VPS52:SOD2
Curated_gene_sets	KEGG_INSULIN_SIGNALING_PATHWAY	137	15	0.00020990732410294704	0.004576555287874042	MTOR:NRAS:PKLR:PRKAB1:PCK2:RPS6KB1:INSR:LIPE:CBLC:PPP1CB:GSK3B:EIF4E:EIF4E1B:FLOT1:PRKAR2B

Curated_gene_sets	GRAHAM_CML_QUIESCENT_VS_CML_DIVIDING_UP	24	6	0.00021048327355610482	0.004576555287874042	EMP1:PCDH9:IL8:CXCL6:CXCL1:SOD2
Curated_gene_sets	HASLINGER_B_CLL_WITH_13Q14_DELETION	24	6	0.00021048327355610482	0.004576555287874042	DUSP8:DLEU1:TUBB4A:TUBB:ARL4A:KLF4
Curated_gene_sets	REACTOME_CELL_CYCLE	641	43	0.00021256713964114867	0.004603668642385665	CDC20:CKS1B:LMNA:PMF1:TUBB8:MCM10:WEE1:CDCA5:POLE:ANKLE2:PSME1:HERC2:ARPP19:BRCA1:PSMD12:AZI1:TUBB4A:PPP1CB:XPO1:DCTN1:ANAPC1:MZT2A:PCNA:GTSE1:NCAPH2:SYCE3:GSK3B:TERT:NIPBL:KIF2A:CKS1B:MDC1:TUBB:CDKN1A:SYNE1:FGFR1OP:RFC2:YWHAG:PRKAR2B:CEP41:NCAPG2:LYN:TERF1:TUBB4B
Curated_gene_sets	BRUINS_UVC_RESPONSE_EARLY_LATE	302	25	0.0002153797028508246	0.004646289197577985	STIL:TCEANC2:ABCD3:SEMA6C:STAMBPL1:KPNA3:TGDS:TXNDC16:KIAA1199:ANKRD11:DHX33:B9D1:TUBD1:ARHGEF18:CCDC130:C19orf57:POU2F2:RND3:RAD18:LRIG1:NIPBL:C5orf63:CALD1:TUBB4B:TOR4A
Curated_gene_sets	CUI_TCF21_TARGETS_2_DN	823	52	0.00021642935565963412	0.004650694865170497	MACF1:TCEANC2:DNAJB4:CTSS:ARNT:ASH1L:NID1:GRK5:RAP1B:MTMR6:FNDC3A:LMO7:HEATR5A:FRMD6:GTF2A1:CORO1A:EPN2:IFI35:RNFT1:PHLPP1:ARHGEF18:CD97:FAM49A:PUM2:MERTK:RBMS1:PER2:USP25:TGFBR2:FAM208A:PROS1:ARL13B:RAB6B:ARMC8:RASA2:PLSCR4:FNDC3B:SLAIN2:GPRIN3:PTGER4:PPAP2A:SLC12A2:CPEB4:TNFAIP3:PLEKHG1:ITGB8:CD36:ABCB1:NSMAF:GEM:KLF4:FAM102A
Curated_gene_sets	WARTERS_RESPONSE_TO_IR_SKIN	81	11	0.00022230661024193885	0.0047583994666961305	WDR65:DNAJB4:FAS:TMEM120B:C17orf89:POU2F2:FOXP1:ANKRA2:CDKN1A:PAPPA:ZNF79
Curated_gene_sets	WILCOX_RESPONSE_TO_PROGESTERONE_UP	153	16	0.00022339348776946523	0.004763130140386931	CDC20:FDPS:ASPM:TLL2:PTHLH:METTL7A:DLGAP5:TMEM251:GOLGA8G:CYB5B:KCNJ16:LSS:ACKR4:AREG:AREGB:NPY1R:MAP7
Curated_gene_sets	FULCHER_INFLAMMATORY_RESPONSE_LECTIN_VS_LPS_DN	449	33	0.00022448342664567299	0.004767889304933773	HIPK1:CTSK:SEMA4A:FCGR3A:FAS:UBE2L6:LPCAT3:C1S:METTL7A:SDS:MTIF3:KCTD12:KIAA1199:NPIPA1:PDXDC1:NTAN1:PLA2G15:METTL16:MBP:CD97:FPR2:RBMS1:APOBEC3B:TGFBR2:XRN1:PTGER4:SLC22A23:RNASET2:CD36:GIMAP5:LYN:LY6E:KLF4
Curated_gene_sets	LY_AGING_OLD_DN	56	9	0.00022605342173147557	0.0047750782260805	CDC20:CKS1B:POSTN:PSMD12:SAFB:DDX39A:PCNA:TGFBR2:IL8:CKS1B
Curated_gene_sets	GAUSSMANN_MLL_AF4_FUSION_TARGETS_G_UP	234	21	0.0002265579743695711	0.0047750782260805	PDZK1IP1:TCEANC2:SYT11:LRRC71:SELP:ACTA2:SYT13:UBE2L6:CRTAM:CLMP:NIPA1:ANP32A:PRMT7:RNFT1:ST8SIA5:MUC16:FAM49A:RBP1:GML:PTPN3:CEL
Curated_gene_sets	JOHNSTONE_PARVB_TARGETS_2_DN	339	27	0.0002304356204208251	0.004821647892252785	PTP4A2:HIPK1:SOAT1:CREM:WEE1:EMP1:RASSF8:LHFP:IPO5:PRPF39:FRMD6:ERO1L:GTF2A1:ARPP19:RFX7:TTC39C:TECR:ZNF83:ZNF654:ATP2C1:RASA2:XRN1:WRNIP1:MDC1:PPIL1:POPDC3:TPST1
Curated_gene_sets	PID_MAPK_TRK_PATHWAY	34	7	0.00023139702664147157	0.004821647892252785	NRAS:RUSC1:RIT1:RAP1B:TRPV1:MAPK7:CREB1
Curated_gene_sets	SCHOEN_NFKB_SIGNALING	34	7	0.00023139702664147157	0.004821647892252785	CTSS:IGFBP5:IL8:PTGER4:EDN1:TPST1:KLF4
Curated_gene_sets	MARTINEZ_RB1_TARGETS_UP	664	44	0.00023554644272528723	0.004889588609176623	LRRC8C:HMGCS2:UBQLN4:ZMYND11:PSAP:WEE1:CTR9:PC:UCP2:UCP3:TENM4:ERC1:PHB2:SOX5:SLC11A2:LGR5:TNFRSF19:LCP1:ERO1L:MGA:ARPP19:TCF12:RPL3L:SIAH1:SAFB2:DNAJB1:TECR:LIPE:CEACAM1:TPO:PEX13:MOB1A:RBMS1:RPN2:FBLN1:PKDREJ:CPT1B:TGFBR2:GSK3B:IER3:CDKN1A:CD36:CALD1:STAR
Curated_gene_sets	CUI_TCF21_TARGETS_2_UP	413	31	0.0002382088956220533	0.004907895039072097	PHGDH:CKS1B:SOAT1:ASPM:FMOD:ALDH18A1:APBB1:CDCA5:LRFN4:EMG1:BRI3BP:POSTN:PCDH9:DCTPP1:PRMT7:PAFAH1B3:GRWD1:GREB1:MYCN:IGFBP2:TGM2:CELSR1:NCAPH2:RAD18:HDAC11:RBP1:CDH6:CKS1B:UTP15:CPNE5:ESR1:RPL12
Curated_gene_sets	HORIUCHI_WTAP_TARGETS_UP	304	25	0.0002382126841360207	0.004907895039072097	LRRC8B:RIT1:C1orf85:ACTA2:SLC11A2:LETMD1:BIVM:MESDC2:NPIPA1:KCNJ2:CEACAM1:C2CD2:APOL1:ATP2C1:FNDC3B:KLHL6:CXCL6:CXCL1:CFI:MARCH3:TAPBP:TNFAIP3:SOD2:KLF4:PAPPA
Curated_gene_sets	HOSHIDA_LIVER_CANCER_SUBCLASS_S1	235	21	0.00024014735637164017	0.004908570827712384	NBL1:CDC20:CTSS:ADAM15:RIT1:FCGR2A:ACTA2:POSTN:LCP1:CORO1A:PPP4R1:PKN1:PPP1CB:MTHFD2:IGFBP5:GNAI2:CXCL1:IER3:LYN:NSMAF:GEM
Curated_gene_sets	REACTOME_INFLUENZA_INFECTION	154	16	0.00024073665027786735	0.004908570827712384	RPL22:FAU:KPNA3:IPO5:RPL3L:POLR2C:RPL19:KPNB1:RPL38:XPO1:RPL32:RPL14:RPL37:RPS18:RPL7:RPL12

Curated_gene_sets	SMID_BREAST_CANCER_RELAPSE_IN_BONE_DN	322	26	0.000240922400196754	0.004908570827712384	PDZK1IP1:DNAJB4:PHGDH:BBOX1:POU2AF1:LGR5:STXBP6:COCH:ACAN:IMPA2:MUC16:FBXO17:CEACAM1:HPCAL1:BCL11A:TTLL4:PI3:SLPI:MRAS:RBP1:IL8:EDN1:MDC1:SOD2:KHDRBS3:OBP2A
Curated_gene_sets	TIEN_INTESTINE_PROBIOTICS_6HR_DN	170	17	0.0002471130060967092	0.00501612046692988	RBM15:CTR9:NDUFA9:NTAN1:N4BP1:SLC7A6:RBMS1:NDUFA6:EIF1B:ATP2C1:FNDC3B:DHX29:DIMT1:CETN3:TTC1:CALD1:CHMP5
Curated_gene_sets	BENPORATH_CYCLING_GENES	646	43	0.0002507439239975084	0.005058312990062545	CDC42:CDC20:STIL:DNAJB4:HORMAD1:CKS1B:FAM189B:LMNA:NUCKS1:CTR9:ZNHIT2:HSPA8:CIT:KDELC1:BIVM:G2E3:DLGAP5:PSEN1:C14orf142:UBR7:BRCA1:KPNB1:TUBD1:CHAF1A:INSR:CD97:DNAJB1:CIC:PANK2:PCNA:ZNFX1:TTC38:GTSE1:RAD18:EIF4E:NIPBL:CKS1B:HDAC3:MDC1:PTP4A1:ARL4A:RFC2:MEPCE:TUBB4B
Curated_gene_sets	KEGG_NEUROACTIVE_LIGAND_RECEPTOR_INTERACTION	270	23	0.0002510306210301899	0.005058312990062545	HTR6:TACR2:P2RX2:MLNR:CHRNB4:GRIN2A:TRPV1:GLP2R:SSTR2:S1PR5:PTGER1:GRIN2D:FPR2:FSHR:MC3R:MCHR1:NPY1R:NPY5R:PTGER4:HCRTR2:CNR1:CRHR2:S1PR3
Curated_gene_sets	LU_EZH2_TARGETS_DN	396	30	0.0002545461239602423	0.005110431488705449	STIL:DDAH1:TRIM33:CLK2:STAMBPL1:CTR9:ASRGL1:APAF1:LHFP:KPNA3:NIPA2:JMJD7-PLA2G4B:RP11-347C12.1:SLC7A6:HNF1B:GJC1:PSMD12:ZNF611:CREB1:METTL21A:TGFBR2:LRIG1:SHQ1:ATP2C1:PLSCR4:SENP2:TNFAIP3:PILRB:CALD1:TERF1
Curated_gene_sets	SHETH_LIVER_CANCER_VS_TXNIP_LOSS_PAM1	254	22	0.00026860974992971376	0.005373171761321292	CDC20:BCL10:HIPK1:PHGDH:LMNA:CDCA5:TRPC6:YAP1:SH3GL3:FAM83G:SSTR2:CD320:PAFAH1B3:FPR2:GREB1:RND3:TGM2:LSS:AIG1:FAM3C:RPL12:TUBB4B
Curated_gene_sets	YORDY_RECIPROCAL_REGULATION_BY_ETS1_AND_SP100_DN	70	10	0.000279492747338168	0.00557061450401182	HSPA8:RASSF8:PCDH9:LMO7:BRCA1:DNAJB1:FAM208A:CPEB4:CA2:KLF4
Curated_gene_sets	FEVR_CTNNB1_TARGETS_DN	553	38	0.0003033860186493404	0.006025005373971198	CDC20:BCAS2:PHGDH:SYT11:ADCK3:SFRP5:WEE1:EHF:AASDHPPT:HSPA8:POLE:UCHL3:G2E3:FKBP3:TCF12:BRCA1:IMPA2:GRWD1:PPP1CB:XPO1:CREB1:NCL:PCNA:RPL32:LRIG1:MRPS22:CFI:DCTD:CETN3:SLC12A2:ZFP62:NRM:TUBB:RFC2:RB1CC1:TERF1:CA2:RPL12
Curated_gene_sets	MCLACHLAN_DENTAL_CARIES_UP	257	22	0.00031627839250931717	0.006248473369247598	CTSS:FCGR2A:UCP2:POU2AF1:LCP1:CORO1A:CD79A:MTHFD2:PI3:SLPI:SCO2:TYMP:IL8:CXCL1:PLAC8:CFI:PTGER4:IER3:C4A:TNFAIP3:SOD2:LYN
Curated_gene_sets	YIH_RESPONSE_TO_ARSENITE_C4	17	5	0.0003169103926595309	0.006248473369247598	CDC42:MYCN:IL8:EIF4E:TAPBP
Curated_gene_sets	GRUETZMANN_PANCREATIC_CANCER_UP	346	27	0.0003187179174677388	0.0062616687999643966	NBL1:CTSK:CKS1B:ARHGEF2:FCGR3A:NID1:NDUFA9:TFCP2:POSTN:LCP1:MYL12A:RALBP1:MRPS12:XPO1:IGFBP5:COPS8:SLPI:AREG:AREGB:CKS1B:PDGFRB:TUBB:TNFAIP3:THBS2:ARL4A:CALD1:GEM:PUF60:KLF4
Curated_gene_sets	SHEDDEN_LUNG_CANCER_POOR_SURVIVAL_A6	458	33	0.0003206284606570356	0.006274422017453708	ACOT7:CDC20:MED8:SSX2IP:NRAS:ASPM:MCM10:LRFN4:NDUFA9:ANKLE2:IPO5:FOXG1:ERO1L:DLGAP5:ARPP19:PSMD12:MUC16:TPRKB:MOB1A:MTHFD2:IL18RAP:PCNA:APOBEC3B:GTSE1:TRMU:COMMD8:KIF2A:FUT9:SOD2:FGFR1OP:RFC2:NCAPG2:UBAC1
Curated_gene_sets	BOYAULT_LIVER_CANCER_SUBCLASS_G3_UP	190	18	0.00032164824739537275	0.006274422017453708	PTP4A2:EBNA1BP2:PTBP2:NRAS:CSDE1:RIT1:IPO7:IPO5:ARPP19:CYB5B:NPEPPS:KPNB1:UTP18:XPO1:TPRKB:TPD52L2:C5orf22:KIF2A
Curated_gene_sets	REACTOME_G_ALPHA_I_SIGNALLING_EVENTS	402	30	0.00032809020865505586	0.006377470804987499	RPE65:RBP3:PSAP:RGR:PDE1B:RCVRN:SSTR2:PDE4A:S1PR5:RLN3:FPR2:PLB1:CREB1:MCHR1:GNAI2:RBP2:RBP1:IL8:CXCL6:CXCL1:NPY1R:NPY5R:CNR1:GNG11:PRKAR2B:TAS2R39:TAS2R40:PPP3CC:RDH10:S1PR3
Curated_gene_sets	REACTOME_MUSCLE_CONTRACTION	207	19	0.00033258174482375045	0.006402463997118901	HIPK1:TMOD4:ACTA2:KCNJ11:ORAI1:KCNK10:MYLPF:CACNB1:CACNG1:KCNJ2:MYL12A:MYL12B:KCNJ14:SCN3A:SCN5A:CACNA2D2:FGF12:KCNIP1:CALD1
Curated_gene_sets	PEREZ_TP53_AND_TP63_TARGETS	207	19	0.00033258174482375045	0.006402463997118901	C10orf54:GRK5:TM7SF2:APAF1:NUAK1:ULK1:ZFYVE1:NPIPA1:TNRC6C:INSR:DEDD2:WFDC5:KIAA1644:SHANK3:PLCXD2:UBE2QL1:MARCH3:MAP7:STAR
Curated_gene_sets	REACTOME_ORGANELLE_BIOGENESIS_AND_MAINTENANCE	293	24	0.00033286760646718884	0.006402463997118901	TUBB8:PRKAB1:PKD1:B9D1:AZI1:TUBB4A:NCOA1:DCTN1:CREB1:MCHR1:PPARA:ARL13B:IFT57:NPHP3:HDAC3:TUBB:SOD2:FGFR1OP:TCTE3:YWHAG:PRKAR2B:CEP41:EXOC4:TUBB4B

Curated_gene_sets	BIOCARTA_CARM_ER_PATHWAY	26	6	0.00033758246204784015	0.00647052656350233	SPEN:PHB2:GTF2A1:BRCA1:HDAC3:ESR1
Curated_gene_sets	MULLIGHAN_NPM1_MUTATED_SIGNATURE_2_UP	128	14	0.00034070621470994814	0.006507725302498002	SSX2IP:ASRGL1:RCE1:METTL7A:PRKAB1:LHFP:SPG7:SH3GL1:ALK:MCHR1:NIPBL:C5orf30:HOXA10:PBX3
Curated_gene_sets	MULLIGHAN_MLL_SIGNATURE_2_DN	276	23	0.0003437416211965482	0.0065419952475548	NBL1:MACF1:TIE1:LRRC8B:VAV3:FAM171A1:PTHLH:LGR5:POLE:ANKLE2:DLEU1:TGFB1I1:VGLL4:TGFBR2:HYAL2:ATP2C1:MRPS22:STK32B:IL8:AREG:AREGB:C5orf30:RPP40:ABCB1
Curated_gene_sets	REACTOME_POTASSIUM_CHANNELS	99	12	0.0003458805980442907	0.0065419952475548	KCNC4:HCN3:KCNJ11:ABCC8:KCNA1:KCNK10:KCNJ16:KCNJ2:KCNJ14:KCNS1:KCNAB1:KCNV2
Curated_gene_sets	SERVITJA_LIVER_HNF1A_TARGETS_DN	159	16	0.0003460680998070254	0.0065419952475548	APCS:F13B:MCM10:HABP2:LCP1:SULT1A3:PRODH2:MYCN:HES6:CELSR1:AFM:CFI:LECT2:ESR1:LY6E:NRBP2
Curated_gene_sets	VANTVEER_BREAST_CANCER_ESR1_UP	208	19	0.0003533640044371127	0.006657039001399167	VAV3:CERS2:FAM63A:FAM179B:MOAP1:AQR:GREB1:AFF3:SYNGR1:CELSR1:HDAC11:TUSC2:LRIG1:MAN2B2:TMEM161B:C5orf30:C4A:ESR1:FAM102A
Curated_gene_sets	BENPORATH_NANOG_TARGETS	987	59	0.0003564863840783743	0.0066929406102905695	ICMT:TAL1:TCEANC2:EXTL2:WDR77:PHGDH:SCNM1:FDPS:UBQLN4:SMG5:SNRPE:NUCKS1:PSEN2:HECTD2:WEE1:YAP1:RBM7:CLMP:METTL7A:KPNA3:DCAF11:PRPF39:DLGAP5:ZNF770:ANP32A:RRN3:TBC1D10B:CYB5B:NBR1:SPAG9:UBALD2:TAF4B:RFX1:DDX39A:EPS15L1:GSK3A:OSR1:MZT2A:IGFBP2:PCNA:CDS2:SMDT1:FBLN1:RPL32:CNBP:RBP1:SENP2:SLAIN2:PPAP2A:CETN3:VPS52:RPS18:B3GALT4:THBS2:EXOC4:CALD1:TERF1:CA2:CALB1
Curated_gene_sets	REACTOME_METABOLISM_OF_LIPIDS	738	47	0.0003579692854476688	0.00669792190220281	ACOT7:MFSD2A:ELOVL1:MED8:ACOT11:HMGCS2:ARNT:CERS2:TNFAIP8L2:FDPS:PSAP:GPAM:SMPD1:TM7SF2:MOGAT2:DGAT2:FDX1:LPCAT3:MTMR6:FITM1:PLA2G4B:PLA2G15:SMPD3:KPNB1:TECR:LIPE:NCOA1:PLB1:PPP1CB:CDS2:LSS:CYP2D6:PPARA:CPT1B:CHKB:ARSA:CYP8B1:ACAD11:LIPH:PLA2G12A:PPAP2A:HDAC3:CD36:ESYT2:STAR:CYP11B1:CYP11B2
Curated_gene_sets	REACTOME_NONSENSE_MEDIATED_DECAY_NMD	114	13	0.00036522513076086774	0.006810520150222148	RPL22:SMG5:FAU:RPL3L:SMG1:RPL19:RPL38:RPL32:RPL14:RPL37:RPS18:RPL7:RPL12
Curated_gene_sets	DING_LUNG_CANCER_EXPRESSION_BY_COPY_NUMBER	100	12	0.000379791939346152	0.007058227899808048	MTMR6:GTF3A:FBXO33:NAPG:DBR1:ARMC8:SENP2:DCTD:NIPBL:ASH2L:RB1CC1:TERF1
Curated_gene_sets	JIANG_AGING_CEREBRAL_CORTEX_DN	48	8	0.0003881070831714425	0.007188474964734361	CSDE1:ARPP19:PPP1CB:DCTN1:NCL:AP2M1:SEPT7:TUBB4B
Curated_gene_sets	TORCHIA_TARGETS_OF_EWSR1_FLI1_FUSION_UP	261	22	0.0003912856473643167	0.007223028007218477	SAMD13:DENND2C:HMGCS2:RUSC1:ARHGEF2:CREM:GRK5:SLC43A1:PC:IL1RL1:IL18R1:ARSA:SHANK3:PLSCR4:KLHL6:STIM2:DIAPH1:CPNE5:MEST:SLC35D2:PBX3:FAM102A
Curated_gene_sets	MEISSNER_BRAIN_HCP_WITH_H3K4ME3_AND_H3K27ME3	1055	62	0.00039751421667595844	0.007313463899446312	NBL1:LDLRAP1:MFSD2A:STIL:F3:PEAR1:ASPM:SFMBT2:C10orf54:SLC16A12:TLL2:SYT13:SLC43A1:UCP2:TENM4:PTHLH:PDE1B:LGR5:P2RX2:LHFP:STXBP6:COCH:ADAMTS7:KIAA1199:ESRP2:STAC2:GJC1:TRIM25:PDE4A:AP1M2:CD97:CHST8:MYCN:IGFBP2:IGFBP5:CTDSP1:SNED1:BFSP1:PPARA:PKDREJ:ODF3B:TGFBR2:CMTM8:SCN5A:CACNA2D2:PROS1:SLC4A4:AREG:AREGB:NPY5R:PTGER4:ISL1:MCIDAS:CCNO:OCLN:CPNE5:POPDC3:LYN:S1PR3:DIRAS2:KLF4:PTPN3:PAPPA
Curated_gene_sets	YAGI_AML_FAB_MARKERS	194	18	0.00041397750086505386	0.007590967440862204	CTR9:TM7SF2:TRPC6:ANKLE2:LCP1:DLEU1:CYB5B:MPPE1:GIPC1:PAFAH1B3:PCNA:TGM2:TYMP:CACNA2D2:PROS1:GP9:KIF2A:KEL
Curated_gene_sets	KEGG_RIBOSOME	87	11	0.00041844967938393343	0.007605449534491672	RPL22:FAU:RPL3L:RPL19:RPL38:RPL32:RPL14:RPL37:RPS18:RPL7:RPL12
Curated_gene_sets	WEI_MIR34A_TARGETS	146	15	0.0004193516719124144	0.007605449534491672	GOLPH3L:PMF1:SNX15:ZFPL1:ARPP19:CYB5B:METTL16:NOL10:PKP4:PER2:RAB43:NPHP3:PDGFRB:FAM167A:CALB1
Curated_gene_sets	REACTOME_ENDOGENOUS_STEROLS	27	6	0.0004202975201755078	0.007605449534491672	ARNT:FDX1:NCOA1:CYP8B1:CYP11B1:CYP11B2

Curated_gene_sets	NGUYEN_NOTCH1_TARGETS_UP	27	6	0.0004202975201755078	0.007605449534491672	ABCC8:PHB2:PTHLH:BRCA1:RBP1:FLOT1
Curated_gene_sets	ACOSTA_PROLIFERATION_INDEPENDENT_MYC_TARGETS_DN	116	13	0.0004328512593496304	0.007794234999844542	RHCE:RIT1:ARHGEF2:CREM:MIA2:CTAGE5:CD97:BCAM:NAGA:ARFGAP3:ESR1:ARL4A:CALB1:TOR4A
Curated_gene_sets	WAKABAYASHI_ADIPOGENESIS_PPARG_BOUND_8D	623	41	0.0004335640628890074	0.007794234999844542	MTOR:UBIAD1:IQCC:F3:SETDB1:CERS2:PRUNE:ARHGEF2:PHLDA3:UCP3:SPSB2:EMG1:PHB2:LPCAT3:CSRNP2:BRI3BP:BIVM:ERO1L:NUMB:CIAPIN1:COQ9:BRCA1:SPAG9:TRIM25:SAFB2:INSR:KEAP1:CHST8:CTU1:EIF6:DNAJC5:CYB5R3:ARSA:TUSC2:GSK3B:PLA2G12A:PRELID2:TNFAIP3:PRKAR2B:ZNF34:XPA
Curated_gene_sets	RAMALHO_STEMNESS_UP	195	18	0.00044035878336634116	0.007890598264815123	RPL22:DPH5:RAB18:YAP1:ERCC5:RRN3:DCTPP1:SLC7A6:RNFT1:PSMD12:GRWD1:XPO1:ESF1:LSG1:KIF2A:CDKN1A:PEX7:ABCB1
Curated_gene_sets	FRASOR_RESPONSE_TO_SERM_OR_FULVESTRANT_DN	49	8	0.00044869565454736746	0.007962176760209898	CDC20:CKS1B:WEE1:POLE:CHAF1A:TTLL4:CKS1B:HOXA11:RFC2
Curated_gene_sets	REACTOME_SIGNALING_BY_EGFR	49	8	0.00044869565454736746	0.007962176760209898	CDC42:NRAS:SH3GL3:SH3GL1:EPS15L1:LRIG1:AREG:AREGB:PTPN3
Curated_gene_sets	PRAMOONJAGO_SOX4_TARGETS_DN	49	8	0.00044869565454736746	0.007962176760209898	ACOT7:ADAM15:UCP2:HSPA8:DLEU1:DLGAP5:MRPS12:LY6E
Curated_gene_sets	ACEVEDO_NORMAL_TISSUE_ADJACENT_TO_LIVER_TUMOR_DN	354	27	0.0004551231112846677	0.008050264421790859	TMEM82:PHGDH:ARNT:SMG5:TM7SF2:DGAT2:EMP1:GTF3A:SLC25A15:TGDS:FITM1:CYB5B:RPL19:TTC39C:TECR:NPRL2:SRPRB:DIAPH1:GSTA2:GSTA1:CLDN3:MEPCE:KHDRBS3:PBX3:RPL12:UBAC1:TUBB4B
Curated_gene_sets	MOOTHA_MITOCHONDRIA	449	32	0.00047270625078496114	0.00830785011363601	HMGCS2:SMCP:PKLR:DAP3:ADCK3:MRPL49:PC:UCP2:UCP3:FDX1:SLC25A3:APAF1:SLC25A15:PCK2:NPIPA1:CYB5B:SPG7:MRPS12:TPO:MTHFD2:NDUFA6:CYB5R3:LMF2:NCAPH2:TYMP:CPT1B:NDUFB4:MRPS22:SOD2:STAR:CYP11B1:CYP11B2
Curated_gene_sets	SENESE_HDAC1_TARGETS_UP	449	32	0.00047270625078496114	0.00830785011363601	DDAH1:UHMK1:CREM:REEP3:FAS:TRUB1:RASSF8:PTHLH:LCP1:RRN3:SLC7A6:PLEKHM1:EPG5:MOB1A:PLEKHB2:TGM2:PI3:TGFBR2:GSK3B:SLAIN2:IL8:DIMT1:CPEB4:CPNE5:TNFAIP3:SOD2:ESYT2:NSMAF:CA2:GEM:COL22A1:ST6GALNAC4
Curated_gene_sets	HOFFMANN_IMMATURE_TO_MATURE_B_LYMPHOCYTE_UP	38	7	0.00047617448717821574	0.008315669377674174	BGLAP:ARL2:LRFN4:PCK2:RAB6B:PBX3:FAM102A
Curated_gene_sets	REACTOME_HSF1_DEPENDENT_TRANSACTIVATION	38	7	0.00047617448717821574	0.008315669377674174	MTOR:HSPA6:HSPA8:DNAJB1:DEDD2:HSPB1:GML
Curated_gene_sets	REACTOME_EXTRA_NUCLEAR_ESTROGEN_SIGNALING	75	10	0.0004930807721906339	0.00855658463034914	NRAS:UHMK1:XPO1:CREB1:GNAI2:AREG:AREGB:ESR1:HSPB1:GNG11:S1PR3
Curated_gene_sets	KEGG_B_CELL_RECEPTOR_SIGNALING_PATHWAY	75	10	0.0004930807721906339	0.00855658463034914	BCL10:VAV3:NRAS:PTPN6:NFATC3:NFKBIB:CD79A:GSK3B:PPP3CC:LYN
Curated_gene_sets	REACTOME_GOLGI_TO_ER_RETROGRADE_TRANSPORT	133	14	0.0005042720706121963	0.008723272517099659	TUBB8:RAB18:KIF19:NAPG:TUBB4A:PAFAH1B3:KDELR1:DCTN1:ARFGAP3:RAB6B:COPB2:KIF2A:COPG2:TUBB4B
Curated_gene_sets	MORI_IMMATURE_B_LYMPHOCYTE_DN	89	11	0.0005097282682425991	0.008778285986887948	CDC20:CKS1B:MCM10:CDCA5:PCK2:DLGAP5:MTHFD2:PCNA:CKS1B:TUBB:CDKN1A:TUBB4B
Curated_gene_sets	KOKKINAKIS_METHIONINE_DEPRIVATION_96HR_UP	118	13	0.0005108106066533516	0.008778285986887948	FAS:WEE1:APAF1:BRCA1:IGFBP2:IGFBP5:DVL3:IL8:DCTD:PDGFRB:CDKN1A:GTF2H5:HSPB1

Curated_gene_sets	REACTOME_CLASS_A_1_RHODOPSIN_LIKE_RECEPTORS	320	25	0.0005122395567698657	0.008778285986887948	HTR6:TACR2:PSAP:RGR:MLNR:SSTR2:S1PR5:RLN3:PTGER1:FPR2:FSHR:MC3R:MCHR1:ACKR4:IL8:CXCL6:CXCL1:NPY1R:NPY5R:PTGER4:EDN1:HCRTR2:CNR1:KEL:S1PR3
Curated_gene_sets	ZHU_CMV_8_HR_DN	50	8	0.0005167354991170225	0.008822155058792325	LMNA:WEE1:ERCC5:ANP32A:ANAPC1:RBMS1:IGFBP2:FBLN1
Curated_gene_sets	REACTOME_ATTENUATION_PHASE	28	6	0.0005180069231030579	0.008822155058792325	HSPA6:HSPA8:DNAJB1:DEDD2:HSPB1:GML
Curated_gene_sets	SCHAEFFER_PROSTATE_DEVELOPMENT_6HR_DN	452	32	0.0005294814285389216	0.008989744871582123	TMEM57:TXLNA:ANXA9:GON4L:ARHGEF2:C1orf85:CSRP1:NUCKS1:PSAP:SYT13:MIA2:CTAGE5:RFX7:CD2BP2:PSMD12:TCF3:GIPC1:AFF3:RND3:PKP4:GNAI2:FAM208A:SENP2:DHX29:KIF2A:MGAT1:PPIL1:L3MBTL3:HOXA13:SEPT7:CALD1:KHDRBS3:S1PR3
Curated_gene_sets	KORKOLA_CHORIOCARCINOMA_DN	11	4	0.0005321351994808021	0.009007002253365823	AKAP3:LRRC23:SOX5:RASSF8
Curated_gene_sets	ZHANG_RESPONSE_TO_IKK_INHIBITOR_AND_TNF_DN	104	12	0.0005449170053470866	0.009195056584093017	ZNF362:SSX2IP:MCM10:FDX1:PTHLH:DLEU1:RFX7:PHLPP1:ERF:THUMPD3:HOXA10:SEPT7
Curated_gene_sets	REACTOME_GPCR_LIGAND_BINDING	453	32	0.0005497007754850258	0.009247412739887237	HTR6:TACR2:PSAP:RGR:PTHLH:MLNR:GLP2R:SSTR2:S1PR5:RLN3:CD97:PTGER1:FPR2:FSHR:MC3R:MCHR1:ACKR4:IL8:CXCL6:CXCL1:NPY1R:NPY5R:PTGER4:EDN1:HCRTR2:CNR1:CRHR2:GNG11:KEL:TAS2R39:TAS2R40:S1PR3
Curated_gene_sets	GRAHAM_NORMAL_QUIESCENT_VS_NORMAL_DIVIDING_UP	63	9	0.000558345102421188	0.009306356419494245	CREM:EMP1:PCDH9:TGFB1I1:IL18R1:CXCL6:CXCL1:AREG:AREGB:SOD2
Curated_gene_sets	PID_MYC_REPRESS_PATHWAY	63	9	0.000558345102421188	0.009306356419494245	ZBTB17:CSDE1:HMGCS2:BRCA1:CREB1:SLC11A1:HDAC3:PDGFRB:CDKN1A
Curated_gene_sets	WIERENGA_STAT5A_TARGETS_UP	199	18	0.0005609499854872166	0.009306356419494245	SEMA6C:FCGR2A:HSPA6:NAALADL1:EMP1:COCH:CALB2:IL18R1:IL18RAP:IGFBP5:HES6:TGM2:OSBP2:FNDC3B:IL8:ARL4A:CA2:UBAC1
Curated_gene_sets	REACTOME_MITOTIC_G2_G2_M_PHASES	199	18	0.0005609499854872166	0.009306356419494245	TUBB8:WEE1:PSME1:PSMD12:AZI1:TUBB4A:PPP1CB:XPO1:DCTN1:MZT2A:GTSE1:TUBB:CDKN1A:FGFR1OP:YWHAG:PRKAR2B:CEP41:TUBB4B
Curated_gene_sets	AMIT_EGF_RESPONSE_60_MCF10A	39	7	0.0005616633941596236	0.009306356419494245	RND3:IL8:PTGER4:IER3:TNFAIP3:CALD1:SLC35D2
Curated_gene_sets	BENPORATH_SOX2_TARGETS	734	46	0.0005783465208115549	0.009554006639592683	ICMT:TAL1:TXNDC12:BTF3L4:WDR77:SCNM1:UBQLN4:NUCKS1:PSEN2:HECTD2:KPNA3:DCAF11:ZNF770:ANP32A:SH3GL3:RRN3:TBC1D10B:VAT1:NBR1:SPAG9:UBALD2:AZI1:RFX1:DDX39A:GSK3A:IGFBP2:PER2:USP25:FOXP1:GSK3B:SENP2:LSG1:PPAP2A:CETN3:MDC1:TUBB:VPS52:RPS18:B3GALT4:THBS2:CEP41:RPL7:RDH10:CA2:TSTD2:PTPN3
Curated_gene_sets	BOYAULT_LIVER_CANCER_SUBCLASS_G123_DN	51	8	0.0005928890556106683	0.009764918248246365	FAS:SLC25A15:PCK2:PRODH2:HYAL1:NR1I2:SLC4A4:CFI
Curated_gene_sets	JIANG_HYPOXIA_NORMAL	200	18	0.0005952024977833393	0.009772842504899385	CDC42:CSDE1:ARHGEF2:SYT13:ENO2:GPRC5A:MESDC1:PKD1:CORO1A:CD2BP2:MAPK7:KPNB1:NCL:TGM2:HLA-A:GSTA1:SOD2:KHDRBS3
Curated_gene_sets	YAO_TEMPORAL_RESPONSE_TO_PROGESTERONE_CLUSTER_13	167	16	0.0005969233015172138	0.009772842504899385	LAMTOR5:DAP3:CSRP1:PC:NDUFA9:FKBP3:CYB5B:PRPSAP2:B9D1:CD320:PAFAH1B3:CETN3:PEX7:FAM3C:CALB1:NACC2
Curated_gene_sets	KEGG_GLYCEROPHOSPHOLIPID_METABOLISM	77	10	0.0006098773001412507	0.00992584327833438	GPAM:LPCAT3:PLA2G4B:JMJD7-PLA2G4B:PLA2G15:DGKE:CDS2:CHKB:PLA2G12A:PPAP2A
Curated_gene_sets	MAHAJAN_RESPONSE_TO_IL1A_UP	77	10	0.0006098773001412507	0.00992584327833438	KCNA1:TRIM25:SSTR2:SERPINB4:MERTK:PPARA:MAP3K13:EDN1:SOD2:GFRA2

Curated_gene_sets	YANG_BCL3_TARGETS_UP	324	25	0.0006137009882476609	0.009958611021682544	ACOT7:CDC20:GPAM:HPX:UBE2L6:UCP2:UCP3:DGAT2:CLMP:PTPN6:DLGAP5:IFI35:SSTR2:PEX11G:LIPE:MERTK:IGFBP5:APOL2:CPT1B:TGFBR2:ACAD11:NPY1R:UBE2QL1:EDN1:CNR1
Curated_gene_sets	REACTOME_UNFOLDED_PROTEIN_RESPONSE_UPR	91	11	0.0006170696964712247	0.00998382470673002	ZBTB17:EXTL2:LMNA:SYVN1:PLA2G4B:SULT1A3:GSK3A:DCTN1:SRPRB:IL8:CREB3L2
Curated_gene_sets	FLORIO_NEOCORTEX_BASAL_RADIAL_GLIA_UP	380	28	0.0006211331896824413	0.010020098757897682	HES2:FBLIM1:STIL:ACOT11:LPHN2:VAV3:CRB1:FMOD:SFMBT2:SLC16A12:EHF:OR8K1:PGA3:POU2AF1:EBPL:ACAN:SLC14A1:CEACAM1:GREB1:PLB1:IL1RL1:SLC9A4:BPIFC:APOL1:SAMD7:ZNF608:ZNF716:POU5F1B
Curated_gene_sets	CHYLA_CBFA2T3_TARGETS_DN	235	20	0.0006235207994411824	0.01002920443779516	RHCE:LPHN2:LMNA:BGLAP:DUSP8:PC:SLC11A2:NIPA1:TARSL2:MYLPF:PDE4A:BCAM:KCNAB1:PPAP2A:SLC22A23:CLDN4:CREB3L2:KEL:GML:UBAC1
Curated_gene_sets	PID_ERA_GENOMIC_PATHWAY	64	9	0.0006283059630125116	0.010076708753737104	APBB1:PHB2:ANP32A:BRCA1:SAFB:GREB1:NCOA1:PCNA:ESR1
Curated_gene_sets	REACTOME_CILIUM_ASSEMBLY	201	18	0.000631239882282075	0.010077743504662917	TUBB8:PKD1:B9D1:AZI1:TUBB4A:DCTN1:MCHR1:ARL13B:IFT57:NPHP3:TUBB:FGFR1OP:TCTE3:YWHAG:PRKAR2B:CEP41:EXOC4:TUBB4B
Curated_gene_sets	REACTOME_CELL_CYCLE_MITOTIC	535	36	0.0006320344499379578	0.010077743504662917	CDC20:CKS1B:LMNA:PMF1:TUBB8:MCM10:WEE1:CDCA5:POLE:ANKLE2:PSME1:ARPP19:PSMD12:AZI1:TUBB4A:PPP1CB:XPO1:DCTN1:ANAPC1:MZT2A:PCNA:GTSE1:NCAPH2:GSK3B:NIPBL:KIF2A:CKS1B:TUBB:CDKN1A:FGFR1OP:RFC2:YWHAG:PRKAR2B:CEP41:NCAPG2:LYN:TUBB4B
Curated_gene_sets	KEGG_LYSOSOME	121	13	0.0006498381056076144	0.010331674621235512	DNASE2B:CTSS:CTSK:PSAP:SMPD1:SLC11A2:PLA2G15:AP1M2:SLC11A1:NAGA:ARSA:HYAL1:PSAPL1
Curated_gene_sets	PUJANA_CHEK2_PCC_NETWORK	760	47	0.0006727064248355477	0.010646181618252218	TARDBP:CDC20:STIL:WDR77:SIKE1:CKS1B:FDPS:SNRPE:LARP4B:WEE1:CTR9:PRKRIR:POU2AF1:HSPA8:POLE:GTF3A:DLEU1:RNASEH2B:UCHL3:TGDS:IPO5:NRL:DLGAP5:SLC7A6:PRPSAP2:BRCA1:UTP18:TCF3:CHAF1A:DDX39A:PAFAH1B3:XPO1:DUSP11:MOB1A:MTHFD2:NCL:PCNA:FBLN1:GTSE1:NR1I2:CNBP:EIF4E:DIMT1:CKS1B:PFDN6:BET1:RB1CC1:KIAA0020
Curated_gene_sets	REACTOME_TRANSCRIPTION_COUPLED_NUCLEOTIDE_EXCISION_REPAIR_TC_NER	78	10	0.0006763263993141517	0.010646181618252218	POLE:ERCC5:AQR:POLR2C:COPS8:PCNA:ISY1:GTF2H5:RFC2:XPA
Curated_gene_sets	BONCI_TARGETS_OF_MIR15A_AND_MIR16_1	92	11	0.0006774217569892359	0.010646181618252218	CDC42:MACF1:WEE1:ARL2:SOX5:KPNA3:PCDH9:KCNJ2:KCNAB1:PTPN3:PAPPA
Curated_gene_sets	BROWNE_HCMV_INFECTION_1HR_UP	52	8	0.0006778525837319672	0.010646181618252218	TLL2:SOX5:VAT1:FSCN2:IGFBP5:IL8:CXCL1:FUT9
Curated_gene_sets	EPPERT_PROGENITOR	137	14	0.0006792964457383255	0.010646181618252218	TAL1:SSX2IP:DDAH1:FAM171A1:ASRGL1:IPO5:RFX7:NOMO1:MYCN:MTHFD2:ARMC8:SKIV2L2:C5orf30:PRKAR2B
Curated_gene_sets	GENTILE_UV_HIGH_DOSE_DN	290	23	0.0006846012680546136	0.01068315057975123	TARDBP:SPEN:HTR6:PHC2:WEE1:EMG1:SIAH1:N4BP1:PUM2:XPO1:DUSP11:RND3:PKP4:RBMS1:C2CD2:EIF4E:KIF2A:C5orf30:RPP40:TNFAIP3:ZNF117:TPST1:GEM
Curated_gene_sets	GRAESSMANN_RESPONSE_TO_MC_AND_DOXORUBICIN_DN	740	46	0.0006855393845941073	0.01068315057975123	EXOSC10:SPEN:C1orf123:LRRC8C:WDR77:NRAS:CSDE1:PMF1:STAMBPL1:ALDH18A1:GPAM:APBB1:RCE1:ERC1:POLE:KPNA3:PRPF39:FANCM:NUMB:MGA:ZNF771:DCTPP1:CYB5B:EPN2:KPNB1:TCF3:KEAP1:DUSP11:MTHFD2:CPT1B:IFRD2:RASSF1:SRPRB:CXCL6:DIMT1:CETN3:RPP40:TUBB:FLOT1:IER3:ZFAND3:CEP41:NCAPG2:NUDCD1:KIAA0020:RBM18
Curated_gene_sets	LU_AGING_BRAIN_DN	153	15	0.0006876647733658853	0.010685999769168742	MTOR:HSPA8:FOXG1:GRIN2A:CORO1A:CALB2:NAPG:MRPS12:MEGF8:FAM49A:PLEKHB2:FGF12:C5orf30:GFRA2:CALB1
Curated_gene_sets	GROSS_HYPOXIA_VIA_HIF1A_DN	107	12	0.0007051214195620579	0.010873285092287651	SSX2IP:SOAT1:ZNF143:ENO2:LMO7:NPEPPS:MERTK:DNAJC5:EIF1B:KLHL2:EDN1:KLF4
Curated_gene_sets	REACTOME_DUAL_INCISION_IN_TC_NER	65	9	0.0007052683518302777	0.010873285092287651	POLE:ERCC5:AQR:POLR2C:PCNA:ISY1:GTF2H5:RFC2:XPA

Curated_gene_sets	OSWALD_HEMATOPOIETIC_STEM_CELL_IN_COLLAGEN_GEL_UP	220	19	0.0007056467511264663	0.010873285092287651	MTOR:HOOK1:F3:HIPK1:KPNB1:KCNJ2:PKP4:ARMC8:IL8:CXCL1:AREG:AREGB:PTGER4:SLC12A2:IER3:CDKN1A:TNFAIP3:SOD2:ARL4A:KLF4
Curated_gene_sets	RAY_TUMORIGENESIS_BY_ERBB2_CDC25A_DN	255	21	0.0007095454801737436	0.010902820353172525	RPL22:CLSTN1:TMEM82:TXLNA:CCDC28B:IQCC:EBNA1BP2:ELOVL1:TOR3A:BBOX1:SYT13:LHFP:ZNF770:MGA:TCF12:MBP:DEDD2:PLB1:PER2:CDS2:CHMP4B
Curated_gene_sets	KORKOLA_YOLK_SAC_TUMOR_UP	20	5	0.0007227600354142013	0.011074938592795326	TPI1:PTPN6:EMG1:PHB2:DDX47
Curated_gene_sets	SMID_BREAST_CANCER_NORMAL_LIKE_UP	480	33	0.0007248240900593029	0.01107571477615618	TIE1:ACTA2:BBOX1:POU2AF1:C1S:NCKAP1L:LHFP:KIAA0226L:KCTD12:RRN3:CORO1A:CLEC10A:MFAP4:MBP:MAST3:CD79A:FAM49A:BCL11A:IL18R1:RBMS1:ZBP1:LIME1:PROS1:PLAC8:CFI:PTGER4:SYNE1:ABCB1:GNG11:GIMAP5:PPP3CC:LYN:GEM
Curated_gene_sets	MORI_PRE_BI_LYMPHOCYTE_UP	79	10	0.0007486320406796811	0.011407825085260183	CDC20:PHGDH:CKS1B:NUCKS1:CDCA5:EMP1:MTHFD2:CKS1B:TUBB:CDKN1A:TUBB4B
Curated_gene_sets	ZHOU_INFLAMMATORY_RESPONSE_FIMA_DN	274	22	0.0007527075820913092	0.011438244223989756	LDLRAP1:ZNF362:CSMD2:SLC16A4:C10orf54:GPAM:SPSB2:MTIF3:GPR180:NFATC3:RNF166:SHPK:SHPK:MBP:MUC16:OR10H2:AFF3:RPE:MCHR1:VGLL4:ACKR4:PCDHGC5:ARAP3
Curated_gene_sets	LIM_MAMMARY_LUMINAL_MATURE_UP	108	12	0.0007665763829337904	0.011590282467425411	C1orf210:TSPAN1:ACOT11:HMGCS2:FAM63A:ABCC8:PTPN6:FBXO36:HES6:TGM2:HDAC11:ESR1
Curated_gene_sets	MIKKELSEN_ES_ICP_WITH_H3K27ME3	41	7	0.0007690334667533676	0.011590282467425411	WDR65:DUSP8:C11orf88:RNF112:MC3R:CYP8B1:PRSS16
Curated_gene_sets	EPPERT_LSC_R	41	7	0.0007690334667533676	0.011590282467425411	LRRC8B:CSDE1:SETDB1:PAQR6:ARPP19:C2CD2:VGLL4
Curated_gene_sets	PAPASPYRIDONOS_UNSTABLE_ATEROSCLEROTIC_PLAQUE_UP	53	8	0.0007723560491238122	0.011608553623579484	CTSS:FCGR3A:NCKAP1L:PLEKHB2:RBMS1:RPN2:SOD2:RNASET2
Curated_gene_sets	HEDVAT_ELF4_TARGETS_UP	12	4	0.0007744666652440446	0.011608558925088526	CREM:IL8:ABCB1:CA2
Curated_gene_sets	DUTERTRE_ESTRADIOL_RESPONSE_24HR_DN	502	34	0.0007874294437445437	0.011770786331626997	FBLIM1:RIT1:PHLDA3:CSRP1:SFMBT2:HOXC13:NUAK1:PCDH9:LMO7:FRMD6:MYL12A:RND3:RBMS1:TGFBR2:ENTPD3:ZNF654:NPHP3:UBA5:XRN1:LIPH:SENP2:ATP10D:PTGER4:CPEB4:EDN1:CDKN1A:ITGB8:ZNF117:CLDN4:BCAP29:CALD1:CREB3L2:PPP3CC:S1PR3
Curated_gene_sets	REACTOME_PEPTIDE_LIGAND_BINDING_RECEPTORS	188	17	0.0007907570287524478	0.011788494350046655	TACR2:PSAP:MLNR:SSTR2:RLN3:FPR2:MC3R:MCHR1:ACKR4:IL8:CXCL6:CXCL1:NPY1R:NPY5R:EDN1:HCRTR2:KEL
Curated_gene_sets	SENESE_HDAC1_AND_HDAC2_TARGETS_UP	240	20	0.0008110709281829777	0.012020889894116316	TIE1:CREM:REEP3:UBASH3B:SMG1:POU2F2:AFF3:PLEKHB2:TGM2:PI3:PPARA:TGFBR2:MFI2:IL8:MEGF10:CPEB4:TNFAIP3:ESYT2:CA2:GEM
Curated_gene_sets	REACTOME_NONSENSE_MEDIATED_DECAY_NMD_INDEPENDENT_OF_THE_EXON_JUNCTION_COMPLEX_EJC	94	11	0.0008129014798420777	0.012020889894116316	RPL22:FAU:RPL3L:RPL19:RPL38:RPL32:RPL14:RPL37:RPS18:RPL7:RPL12
Curated_gene_sets	REACTOME_RECRUITMENT_OF_NUMA_TO_MITOTIC_CENTROSOMES	94	11	0.0008129014798420777	0.012020889894116316	TUBB8:AZI1:TUBB4A:DCTN1:MZT2A:TUBB:FGFR1OP:YWHAG:PRKAR2B:CEP41:TUBB4B
Curated_gene_sets	WANG_RESPONSE_TO_GSK3_INHIBITOR_SB216763_UP	387	28	0.0008223671880545809	0.01210191644106416	C1orf63:DPH5:CTSS:ARNT:PSAP:LETMD1:PDE1B:TNFSF13B:MYO5A:PITPNC1:DENND1C:SIGLEC6:MTHFD2:SIRPB2:ODF3B:CPT1B:PROS1:ACAD11:ATP10D:THG1L:CPEB4:ZFAND3:ZNF117:ERV3-1:CD36:PRKAR2B:GFRA2:SLC35D2

Curated_gene_sets	DURCHDEWALD_SKIN_CARCINOGENESIS_DN	258	21	0.0008243651160260182	0.01210191644106416	FBLIM1:MACF1:HOOK1:ARNT:LARP4B:UBASH3B:LETMD1:DHX37:RBM25:SMG1:N4BP1:FAM83G:NPLOC4:FAM168B:SLPI:NAT6:GSK3B:ATP2C1:RBP1:STIM2:CPEB4
Curated_gene_sets	YAO_HOXA10_TARGETS_VIA_PROGESTERONE_UP	80	10	0.0008271806184039491	0.01210191644106416	NBL1:DTD2:PRPF39:PROS1:RBP1:KCNAB1:ARL4A:INMT:GEM:PBX3
Curated_gene_sets	SMID_BREAST_CANCER_RELAPSE_IN_BRAIN_DN	80	10	0.0008271806184039491	0.01210191644106416	VAV3:MAPKBP1:JMJD7-PLA2G4B:CHST8:GREB1:CELSR1:STK32B:CCNO:C4A:ESR1
Curated_gene_sets	RUIZ_TNC_TARGETS_UP	156	15	0.0008410094473528465	0.012255046878943947	C1orf63:EXTL2:SLC16A4:PSAP:METTL7A:KCTD12:TCF12:NBR1:IMPA2:C21orf91:VGLL4:LRIG1:PROS1:PPAP2A:PDGFRB
Curated_gene_sets	ROSTY_CERVICAL_CANCER_PROLIFERATION_CLUSTER	140	14	0.0008421028395275063	0.012255046878943947	CDC20:STIL:ASPM:MCM10:SAC3D1:SLC25A15:DLGAP5:BRCA1:PAFAH1B3:PCNA:APOBEC3B:GTSE1:COMMD8:CA2
Curated_gene_sets	TORCHIA_TARGETS_OF_EWSR1_FLI1_FUSION_DN	313	24	0.000851254251338931	0.012323025359514368	ACOT7:HOOK1:LPHN2:PHGDH:F5:ZNF496:NCKAP1L:LCP1:KCTD12:SLC7A6:CD33:HPCAL1:MYCN:PER2:TGM2:ODF3B:GK5:PLAC8:MEI4:L3MBTL3:KHDRBS3:ZNF34:CNTLN:PTPN3
Curated_gene_sets	HORIUCHI_WTAP_TARGETS_DN	313	24	0.000851254251338931	0.012323025359514368	CDC20:LRRC8C:ABCD3:CKS1B:FDPS:MSTO1:UHMK1:TOR3A:ASPM:NUCKS1:ARL5B:C12orf57:EBPL:DLGAP5:KPNB1:PEX13:C21orf91:APOBEC3B:GTSE1:ATP2C1:SRPRB:IPO11:CKS1B:BCAP29:ENY2
Curated_gene_sets	OUILLETTE_CLL_13Q14_DELETION_DN	54	8	0.0008771628963452581	0.012664758773740854	DDAH1:GRK5:ENO2:DLEU1:RNASEH2B:PHLPP1:PLA2G12A:ESYT2
Curated_gene_sets	DOUGLAS_BMI1_TARGETS_DN	314	24	0.0008896285926011355	0.012811117507588605	SLC16A4:GOLPH3L:SOAT1:ZBTB41:ARL5B:ACTA2:WEE1:PEX16:GPRC5A:APAF1:LCP1:KLHL28:ZFYVE1:SHPK:SHPK:IFI35:ANAPC1:C2CD2:CGGBP1:PLSCR4:LIPH:ATP10D:ISL1:CPEB4:TNFAIP3
Curated_gene_sets	MARKEY_RB1_CHRONIC_LOF_UP	110	12	0.000902960948211316	0.012935385875287626	SLC25A15:ERCC5:IL1RL1:PCNA:LSS:PROS1:AREG:AREGB:CDKN1A:HOXA11:TPST1:FAM3C:KLF4
Curated_gene_sets	DOANE_BREAST_CANCER_ESR1_UP	110	12	0.000902960948211316	0.012935385875287626	TSPAN1:VAV3:ANXA9:SCGB1D2:GPRC5A:GREB1:CELSR1:STK32B:AREG:AREGB:C4A:ESR1:PIP
Curated_gene_sets	REACTOME_CENTROSOME_MATURATION	81	10	0.0009123741406203889	0.012972863352226426	AZI1:TUBB4A:DCTN1:MZT2A:TUBB:FGFR1OP:YWHAG:PRKAR2B:CEP41:TUBB4B
Curated_gene_sets	OUYANG_PROSTATE_CANCER_MARKERS	21	5	0.0009194093860492342	0.012972863352226426	VAV3:INSR:CEACAM1:IGFBP2:CDKN1A
Curated_gene_sets	VALK_AML_CLUSTER_13	31	6	0.0009194618193521982	0.012972863352226426	NBL1:GRK5:LCP1:CACNA2D2:STK32B:LAT2
Curated_gene_sets	REACTOME_EGFR_DOWNREGULATION	31	6	0.0009194618193521982	0.012972863352226426	CDC42:SH3GL3:SH3GL1:EPS15L1:AREG:AREGB:PTPN3
Curated_gene_sets	KOYAMA_SEMA3B_TARGETS_DN	352	26	0.000919726723753555	0.012972863352226426	GPR153:CCDC28B:RIMKLA:NRAS:CKS1B:SCAMP3:ASH1L:DAP3:GON4L:SSR2:UHRF1BP1L:COCH:TDRD9:IMPA2:DDX27:SYNGR1:CDPF1:CELSR1:TERT:RPL37:CKS1B:PRKAR2B:TSGA13:EXOC4:CALD1:KHDRBS3:FAM102A
Curated_gene_sets	PEREZ_TP63_TARGETS	352	26	0.000919726723753555	0.012972863352226426	HSPA6:C10orf54:GRK5:TM7SF2:DGAT2:APAF1:NUAK1:ULK1:KIAA0226L:ZFYVE1:KIAA1199:NPIPA1:TNRC6C:INSR:DEDD2:WFDC5:KIAA1644:SHANK3:PLCXD2:UBE2QL1:MARCH3:MAP7:HSPB1:STAR:KHDRBS3:CYP11B2
Curated_gene_sets	CHICAS_RB1_TARGETS_GROWING	243	20	0.0009454429103403052	0.013301487083841481	CDC20:PHGDH:LMNA:PEAR1:ASPM:POSTN:PCK2:STXBP6:UBR7:IMPA2:SLC14A1:APOBEC3B:FBLN1:GTSE1:RBP1:CDH6:ARL4A:MEST:NCAPG2:S1PR3
Curated_gene_sets	MISSIAGLIA_REGULATED_BY_METHYLATION_UP	126	13	0.0009520503420555964	0.013360277886856724	PSAP:UBE2L6:PSEN1:BCL11A:SLPI:MYRIP:HYAL1:MFI2:AREG:AREGB:CDKN1A:TNFAIP3:SOD2:LY6E

Curated_gene_sets	MARTORIATI_MDM4_TARGETS_NEUROEPITHELIUM_DN	158	15	0.0009585696349726162	0.013417535781130693	TAL1:SYT11:DBX1:PCDH9:SKOR1:CORO1A:RND2:BCL11A:KIAA1841:SLC4A5:IGFBP5:PLCXD2:CNR1:ITGB8:ENY2
Curated_gene_sets	BIOCARTA_CREM_PATHWAY	6	3	0.00097543523684809	0.013618957456602396	CREM:FSHR:XPO1
Curated_gene_sets	LAU_APOPTOSIS_CDKN2A_UP	55	8	0.0009930697085609205	0.013830067004540819	BCL10:FAS:APAF1:SIAH1:BRCA1:PCNA:CDKN1A:TNFAIP3
Curated_gene_sets	HUMMEL_BURKITTS_LYMPHOMA_UP	43	7	0.0010326364826067215	0.014344781037423168	PTBP2:LHFP:DLEU1:RNASEH2B:TCF3:TERT:PRKAR2B
Curated_gene_sets	GRYDER_PAX3FOXO1_ENHANCERS_IN_TADS	1010	58	0.0010397763310732438	0.014407580849455702	MFSD2A:TM2D1:ABCD3:DPH5:FCRLA:CSRP1:SFMBT2:NSUN6:ARL5B-AS1:ZNF488:HECTD2:RBM20:WEE1:CTR9:FAU:TTC12:REXO2:CDON:CSRNP2:TNFRSF19:GTF3A:STXBP6:HERC2:AQR:TCF12:NAPG:CHST8:PAFAH1B3:HPCAL1:MYCN:FAM49A:NCOA1:ALK:BCL11A:RBMS1:COPS8:NDUFA6:SLC25A26:LRIG1:FOXP1:XRN1:SENP2:STIM2:DCTD:DROSHA:C5orf22:SKIV2L2:MEGF10:ADAM19:ZFAND3:TNFAIP3:ITGB8:TPST1:GNG11:MEST:NCAPG2:ESYT2:KHDRBS3:CNTLN
Curated_gene_sets	BHAT_ESR1_TARGETS_VIA_AKT1_UP	281	22	0.0010470366459480291	0.014471730124020372	UBIAD1:MFSD2A:UBQLN4:LRFN4:TMEM120B:SLC7A6:TBKBP1:C17orf70:ARHGEF18:CHST8:HPCAL1:GREB1:RTKN:TGM2:LRIG1:ARAP3:RPP40:IER3:WDR46:NUDCD1:FAM102A:SLC25A25
Curated_gene_sets	KORKOLA_EMBRYONAL_CARCINOMA_DN	13	4	0.001085452619500786	0.014965099899433142	AKAP3:LRRC23:SOX5:RASSF8
Curated_gene_sets	BOSCO_EPITHELIAL_DIFFERENTIATION_MODULE	69	9	0.0010937657539267325	0.015042013530877389	F3:DENND2C:ANXA9:GPRC5A:LMO7:MUC16:MUC21:RDH10:PTPN3
Curated_gene_sets	IZADPANAH_STEM_CELL_ADIPOSE_VS_BONE_UP	128	13	0.0011021264402298824	0.015112455677431394	LMNA:STAMBPL1:ORAI1:PPP4R1:DDX39A:HPCAL1:CDH6:MARCH3:HOXA10:HOXA11:ABCB1:LYN:KHDRBS3
Curated_gene_sets	NGUYEN_NOTCH1_TARGETS_DN	83	10	0.0011043823272727541	0.015112455677431394	F3:FNDC3A:ERCC5:HPCAL1:MTHFD2:RPE:SLC12A2:BET1:KLF4:PBX3
Curated_gene_sets	DELACROIX_RAR_BOUND_ES	453	31	0.0011111973281538673	0.015167981394973757	SPEN:NBL1:RHCE:TMEM57:C1orf210:DENND2C:REEP3:WEE1:PTPN6:GPRC5A:DCAF4:ZNF770:ANP32A:C15orf26:SLX1A:MYLPF:FAM211A:VAT1:MBP:CD97:LIPE:MYCN:IGFBP2:HYAL1:RBP1:PDGFRB:CDX1:DACT2:YWHAG:RB1CC1:NACC2
Curated_gene_sets	KEGG_PATHOGENIC_ESCHERICHIA_COLI_INFECTION	56	8	0.001120905756410944	0.015262630113902484	CDC42:ARHGEF2:TUBB8:TUBB4A:NCL:OCLN:TUBB:TUBB4B
Curated_gene_sets	RIGGI_EWING_SARCOMA_PROGENITOR_UP	434	30	0.001132502185604046	0.015382455612365082	FBLIM1:OLFM3:UHMK1:FMOD:CREM:GRK5:OPCML:KIAA0226L:PCDH9:LRFN5:TCF12:SH3GL3:NFATC3:MFAP4:CEACAM1:ALK:IGFBP2:IGFBP5:TM4SF20:ENTPD3:STK32B:NPY1R:PTGER4:L3MBTL3:RNASET2:CD36:GFRA2:STAR:S1PR3:PALM2
Curated_gene_sets	HOSHIDA_LIVER_CANCER_SURVIVAL_DN	113	12	0.0011450009070447578	0.01551391623067294	ZBTB17:PKLR:HABP2:TM7SF2:ERCC5:DCAF11:CREB1:PROS1:ATP2C1:SLC4A4:RFC2:XPA
Curated_gene_sets	SMID_BREAST_CANCER_RELAPSE_IN_BONE_UP	98	11	0.0011515903848905698	0.015564861688656078	TSPAN1:HMGCS2:ANXA9:HPX:SCGB2A1:SCGB1D2:TTC12:JMJD7-PLA2G4B:CELSR1:CCNO:ESR1
Curated_gene_sets	VERHAAK_GLIOBLASTOMA_CLASSICAL	161	15	0.0011607693438899641	0.015650470982202676	VAV3:LHFP:KEAP1:CC2D1A:CD97:SARS2:FBXO17:MEGF8:SLC4A4:CDH6:ARAP3:ADAM19:LRRC16A:ITGB8:DENND2A
Curated_gene_sets	REACTOME_GENE_EXPRESSION_TRANSCRIPTION	1478	79	0.0011679829568241944	0.01570922798408287	MTOR:PHC2:MED8:TAL1:LAMTOR5:HIPK1:TRIM33:CTSK:BGLAP:SNRPE:ZNF496:FAS:GPAM:ZNF143:CTR9:YAP1:APAF1:NUAK1:PRKAB1:GTF3A:PSME1:FOXG1:GTF2A1:CPSF2:TDRD9:ZNF770:MGA:TCF12:GRIN2A:RRN3:ZNF771:ZNF720:POLR2C:BRCA1:PSMD12:TNRC6C:TAF4B:TCEB3C:TCEB3B:TCF3:ZNF442:DDX39A:ZNF737:POU2F2:ZNF480:ZNF611:ZNF135:CREB1:ZNF343:P

						CNA:PPARA:HDAC11:ZNF860:ZNF619:ZNF620:ZNF662:NR1I2:GSK3B:DROSHA:OCLN:HDAC3:LSM11:ZNF311:MDC1:CDKN1A:ESR1:GTF2H5:SOD2:ZNF716:ZNF92:RFC2:YWHAG:BLK:ASH2L:ZNF34:KLF4:ZNF79:GTF3C5:NELFB
Curated_gene_sets	DANG_MYC_TARGETS_UP	129	13	0.0011842678011802	0.015889407742176294	RPL22:NRAS:HSPA8:EMP1:SLC25A3:RPL19:IMPA2:NCL:PCNA:RPL32:EIF4E:TERT:RFC2
Curated_gene_sets	KERLEY_RESPONSE_TO_CISPLATIN_UP	44	7	0.001188611439034563	0.015908884491798374	LMNA:PHLDA3:ACTA2:FAS:PDE4A:IER3:CDKN1A
Curated_gene_sets	HSIAO_HOUSEKEEPING_GENES	397	28	0.0012076152766897026	0.016066621933384092	PTP4A2:SSR2:FMOD:PSAP:FAS:PTPN6:PHB2:SLC25A3:PSME1:PSEN1:NPIPA1:RPL19:RPL38:XPO1:NCL:DSTN:RPN2:CYB5R3:CHKB:RPL32:RPL14:HYAL2:DVL3:HLA-A:RPS18:HSPB1:RPL7:RPL12
Curated_gene_sets	HSIAO_LIVER_SPECIFIC_GENES	248	20	0.0012119118684344764	0.016066621933384092	HMGCS2:APCS:F5:F13B:HABP2:HPX:C1S:SDS:PCK2:ASGR2:PROS1:AFM:MTTP:CFI:C4A:RGL2:GSTA2:GSTA1:ASL:FCN2
Curated_gene_sets	QI_PLASMACYTOMA_UP	248	20	0.0012119118684344764	0.016066621933384092	SEMA4A:FAS:UBASH3B:PTPN6:SLC11A2:APAF1:FBXW8:LCP1:TNFSF13B:IFI35:DNAJB1:CD79A:MYCN:MOB1A:IL18RAP:TGM2:PLAC8:TNFAIP3:GNG11:BLK
Curated_gene_sets	SCHUETZ_BREAST_CANCER_DUCTAL_INVASIVE_DN	84	10	0.0012120792769231773	0.016066621933384092	PDZK1IP1:HOOK1:ABCD3:VAV3:ANXA9:CELSR1:AREG:AREGB:PEX7:CLDN3:PRKAR2B
Curated_gene_sets	IVANOVA_HEMATOPOIESIS_LATE_PROGENITOR	496	33	0.001252420103914753	0.016490876200459012	CDC42:CTSS:TNFAIP8L2:FDPS:DAP3:SOAT1:STAMBPL1:RNASEH2B:FBXO33:ERO1L:TRAPPC2L:CLEC10A:MAPK7:KCNJ2:NAPG:SLC14A1:CD33:DUSP11:IL1RL1:RPE:SLPI:IFRD2:FNDC3B:MAP3K13:C5orf22:CETN3:C5orf63:PRSS16:RAB44:PPIL1:PRKAR2B:BCAP29:NCAPG2
Curated_gene_sets	GOTZMANN_EPITHELIAL_TO_MESENCHYMAL_TRANSITION_DN	196	17	0.0012535185878870597	0.016490876200459012	ABCD3:RAB18:DMBT1:UCP2:PTHLH:PSME1:NTAN1:POLR2C:NPEPPS:TRIM25:PPP1CB:MTHFD2:KCNAB1:CXCL6:CDKN1A:ABCB1:KLF4
Curated_gene_sets	DEURIG_T_CELL_PROLYMPHOCYTIC_LEUKEMIA_DN	322	24	0.0012543782814896476	0.016490876200459012	TARDBP:CDC42:BCAS2:SYT11:CUL2:CREM:FAS:CTR9:CRTAM:DERL2:IKZF3:TBX21:MBP:NCOA1:IL18R1:TGFBR2:CACNA2D2:FAM208A:KLHL2:PPAP2A:SYNE1:ABCB1:GNG11:PPP3CC
Curated_gene_sets	WANG_MLL_TARGETS	285	22	0.0012560765548068219	0.016490876200459012	F5:CLMP:SOX5:PTHLH:HOXC13:POSTN:KIAA0226L:FNDC3A:STXBP6:HNF1B:KCNJ2:IL1RL1:SNED1:PLAC8:B3GALT4:ESR1:THBS2:HOXA10:HOXA13:ZCWPW1:FAM3C:S1PR3
Curated_gene_sets	ZWANG_CLASS_1_TRANSIENTLY_INDUCED_BY_EGF	537	35	0.0012929263351339699	0.01693425659421897	BCL10:LRRC8C:DENND2C:ARHGEF2:ABL2:CSRP1:REEP3:STAMBPL1:ENO2:GPRC5A:RASSF8:CSRNP2:FBXO33:DCAF4:ARPP19:ACAN:LINS:FAM83G:SERPINB4:SLPI:ARFGAP3:ARL13B:NPHP3:AREG:AREGB:PTGER4:ACTBL2:C5orf30:ITGB8:CALD1:PIP:LYN:POU5F1B:S1PR3:PALM2:SLC25A25
Curated_gene_sets	DUTERTRE_ESTRADIOL_RESPONSE_24HR_UP	323	24	0.0013079617939509524	0.017090493654451755	STIL:ASPM:MCM10:CDCA5:CIT:TMEM120B:BRI3BP:POLE:DLGAP5:BRCA1:IMPA2:CHAF1A:CD320:EPS15L1:GREB1:PCNA:GTSE1:NCAPH2:RAD18:LRIG1:AREG:AREGB:NPY1R:RFC2:NCAPG2
Curated_gene_sets	IVANOVA_HEMATOPOIESIS_MATURE_CELL	286	22	0.0013136066209707405	0.01712357825109015	RHCE:ACTA2:PC:C1S:G2E3:GTF2A1:NIPA2:DERL2:DEDD2:FPR2:MTHFD2:RBMS1:SLC11A1:USP25:RAB43:C5orf30:CPEB4:TSPAN17:SLC22A23:CLDN4:KEL:UBAC1
Curated_gene_sets	LIN_APC_TARGETS	71	9	0.0013444666434004751	0.01748442318048703	LMNA:PCDH9:GIPC1:PCNA:TGM2:CYB5R3:SCN5A:HDAC3:LY6E
Curated_gene_sets	HUANG_GATA2_TARGETS_UP	147	14	0.0013535374967413847	0.01756087209805273	TMEM50A:TNFAIP8L2:FCGR2A:C12orf57:KCTD12:MYL12B:GREB1:HES6:NDUFA6:TGFBR2:PLAC8:CDKN1A:RAB44:LAT2
Curated_gene_sets	PID_HNF3B_PATHWAY	45	7	0.0013624472768216593	0.017634876399519876	PKLR:KCNJ11:ABCC8:UCP2:HNF1B:CREB1:CPT1B
Curated_gene_sets	ACEVEDO_LIVER_CANCER_WITH_H3K27ME3_DN	215	18	0.0013697887250649476	0.01767962541496024	KCNC4:PHGDH:APCS:OR4C16:YAP1:ULK1:SCFD1:COCH:PRPF39:SLC51B:NOMO1:APOL2:STK32B:PCDHGC3:PCDHGC4:PCDHGC5:PDGFRB:KCNIP1

Curated_gene_sets	HATADA_METHYLATED_IN_LUNG_CANCER_UP	362	26	0.0013723323127046033	0.01767962541496024	FBLIM1:KCNC4:BCAS2:LRRC71:TLL2:POU2AF1:CLMP:HOXC12:LRFN5:GRIN2A:TGFB1I1:SLC47A1:HNF1B:RALBP1:NCL:TYMP:CPT1B:CACNA2D2:LRIG1:CNBP:STK32B:ISL1:PCDHGC4:ESR1:HOXA13:MEPCE
Curated_gene_sets	LEE_RECENT_THYMIC_EMIGRANT	233	19	0.0013940029726076922	0.017916846617558213	VAV3:SIKE1:RAB18:RBM7:UBASH3B:HERC2:ZNF720:MBTD1:TAF4B:BCL11A:PEX13:METTL21A:PLCXD2:RASA2:PLAC8:PTGER4:PFDN6:TNFAIP3:FAM3C
Curated_gene_sets	LIU_CMYB_TARGETS_UP	164	15	0.0013977274848986344	0.017922841245751487	ACOT7:TSPAN1:ASRGL1:SAC3D1:SPG7:IMPA2:PHLPP1:HPCAL1:GREB1:NCOA1:USP25:ANKRA2:MARCH3:WDR46:PRKAR2B
Curated_gene_sets	KEGG_ENDOCYTOSIS	181	16	0.0014109527426765022	0.01805035124991497	CDC42:LDLRAP1:HSPA6:GRK5:HSPA8:SH3GL3:RABEP1:EPN2:SH3GL1:CBLC:CHMP4B:ARFGAP3:AP2M1:ARAP3:HLA-A:CHMP5
Curated_gene_sets	MATZUK_IMPLANTATION_AND_UTERINE	23	5	0.0014282712072451436	0.018229512554653212	DLGAP5:NCOA1:TERT:HOXA10:HOXA11
Curated_gene_sets	REACTOME_SELENOAMINO_ACID_METABOLISM	116	12	0.0014385112449786265	0.018317709163489408	RPL22:FAU:RPL3L:RPL19:RPL38:RPL32:RPL14:RPL37:RPS18:INMT:RPL7:RPL12
Curated_gene_sets	TAKEDA_TARGETS_OF_NUP98_HOXA9_FUSION_6HR_UP	86	10	0.0014531815279700527	0.018461781952340092	OLFM3:PCDH9:HEATR5A:FRMD6:CDH9:MCTP1:C5orf30:EDN1:ITGB8:PBX3
Curated_gene_sets	TARTE_PLASMA_CELL_VS_PLASMABLAST_DN	307	23	0.0014641958944668426	0.018558851648530186	ACOT7:EBNA1BP2:CDC20:STIL:ABCD3:CKS1B:FDPS:ACTA2:FAS:NDUFA9:UCHL3:IPO5:DLGAP5:MYO5A:ANP32A:CORO1A:CHAF1A:DDX39A:MTHFD2:PCNA:KIF2A:CKS1B:CDKN1A:SOD2
Curated_gene_sets	AFFAR_YY1_TARGETS_DN	252	20	0.001469004473314035	0.018573496472363667	FDPS:ASPM:FMOD:ALDH18A1:ZNF143:UCP3:LGR5:MTHFD2:IL1RL1:CREB1:IGFBP5:SEMG1:MCHR1:RAD18:SLC25A26:LRIG1:SLC4A4:CXCL6:THBS2:TERF1
Curated_gene_sets	NIKOLSKY_BREAST_CANCER_14Q22_AMPLICON	14	4	0.0014745657385430528	0.018573496472363667	FRMD6:TXNDC16:GPR137C:ERO1L
Curated_gene_sets	REACTOME_COPI_MEDIATED_ANTEROGRADE_TRANSPORT	101	11	0.001475480450540433	0.018573496472363667	TUBB8:NAPG:TUBB4A:KDELR1:DCTN1:ARFGAP3:TMEM115:COPB2:BET1:COPG2:TUBB4B
Curated_gene_sets	REACTOME_AURKA_ACTIVATION_BY_TPX2	72	9	0.0014860993320104999	0.018664457592214065	AZI1:TUBB4A:DCTN1:TUBB:FGFR1OP:YWHAG:PRKAR2B:CEP41:TUBB4B
Curated_gene_sets	YOKOE_CANCER_TESTIS_ANTIGENS	34	6	0.0015257322161828992	0.01911857157453788	OVGP1:TDRD9:PDXDC1:TUBB4A:GREB1:PTGER4
Curated_gene_sets	NIKOLSKY_BREAST_CANCER_20Q12_Q13_AMPLICON	149	14	0.001540088003110665	0.019254600238890383	KCNS1:WFDC5:WFDC12:PI3:SEMG1:SEMG2:SLPI:MC3R:ZBP1:LIME1:SLC2A4RG:TPD52L2:DNAJC5:UCKL1
Curated_gene_sets	PARENT_MTOR_SIGNALING_UP	563	36	0.0015452431323206574	0.01927524369817673	GPR153:CLSTN1:MTOR:PHGDH:ARNT:PRUNE:CLK2:FDPS:BGLAP:ECHDC3:SMPD1:RCE1:AASDHPPT:DYRK4:PTPN6:SLC11A2:NCKAP1L:PCDH9:PCK2:VAT1:NBR1:NPEPPS:SPAG9:HELZ:PDE4A:PRODH2:TAB1:PPARA:TTC38:NCAPH2:NPRL2:SLC4A4:SYNE1:ZCWPW1:SCRIB:ZNF34
Curated_gene_sets	RASHI_RESPONSE_TO_IONIZING_RADIATION_2	117	12	0.001549145123403569	0.01928019756525573	BCL10:PHLDA3:ACTA2:SLC11A2:APAF1:N4BP1:NFKBIB:OCLN:EDN1:CDKN1A:TNFAIP3:KLF4
Curated_gene_sets	BASAKI_YBX1_TARGETS_UP	290	22	0.001566869440422262	0.019412947729195638	CDC20:MED8:WDR77:RUSC1:SMG5:ASPM:MCM10:CREM:SAC3D1:FRMD6:CHAF1A:DDX39A:PEX13:TPRKB:RND3:GTSE1:IFRD2:AREG:AREGB:PLAC8:MDC1:RFC2:TUBB4B
Curated_gene_sets	GRADE_COLON_AND_RECTAL_CANCER_UP	290	22	0.001566869440422262	0.019412947729195638	DAP3:DGAT2:TPI1:GTF3A:KPNA3:TGDS:SH3GL1:DDX39A:PAFAH1B3:MTHFD2:RTKN:COPS8:PCNA:EIF6:DDX27:GSK3B:CNBP:SLC12A2:MDC1:SOD2:RFC2:LY6E

Curated_gene_sets	NABA_MATRISOME_ASSOCIATED	750	45	0.0015740094105207998	0.019450289853823934	ANGPTL7:OVGP1:CTSS:CTSK:ANXA9:SEMA6C:HRNR:FLG:ADAM15:SEMA4A:F13B:GDF2:TLL2:SFRP5:HABP2:HPX:TNFSF13B:ADAMTS7:CCL17:CLEC10A:ADAM11:SERPINB4:MUC16:MEGF8:TPO:MMP24:TGM2:PI3:SLPI:PLXNB2:HYAL3:HYAL1:HYAL2:FGF12:IL8:CXCL6:PF4V1:CXCL1:AREG:AREGB:MEGF10:ADAM19:MUC21:ADAMTSL1:PAPPA:FCN2
Curated_gene_sets	LEE_LIVER_CANCER_CIPROFIBRATE_UP	59	8	0.0015847597811195955	0.019450289853823934	APCS:UCP2:BRCA1:IGFBP2:TGM2:CHKB:CDKN1A:CD36
Curated_gene_sets	STEIN_ESRRA_TARGETS_UP	385	27	0.0015853342690217366	0.019450289853823934	HOOK1:SIKE1:FAM63A:FDX1:RBM7:NDUFA9:LPCAT3:METTL7A:LETMD1:SLC25A3:DCTPP1:CIAPIN1:COQ9:IMPA2:CCDC130:MRPS12:PLEKHB2:MFF:SYNGR1:GTPBP8:MRPS22:PLA2G12A:MARCH3:SOD2:RNASET2:ST6GALNAC4:UBAC1
Curated_gene_sets	REACTOME_REGULATION_OF_PLK1_ACTIVITY_AT_G2_M_TRANSITION	87	10	0.001587562287650781	0.019450289853823934	AZI1:TUBB4A:PPP1CB:DCTN1:TUBB:FGFR1OP:YWHAG:PRKAR2B:CEP41:TUBB4B
Curated_gene_sets	BAELDE_DIABETIC_NEPHROPATHY_UP	87	10	0.001587562287650781	0.019450289853823934	TIE1:BBOX1:MIA2:CTAGE5:SPG7:VAT1:IMPA2:IGFBP5:HYAL1:HYAL2:C4A
Curated_gene_sets	REACTOME_TRANSLATION	291	22	0.001636333672342239	0.02000327007012146	RPL22:DAP3:SSR2:FAU:MRPL49:MTIF3:MTFMT:RPL3L:RPL19:RPL38:SARS2:MRPS12:RPN2:RPL32:RPL14:SRPRB:MRPS22:EIF4E:RPL37:RPS18:RPL7:RPL12
Curated_gene_sets	BORCZUK_MALIGNANT_MESOTHELIOMA_UP	310	23	0.0016610652908287588	0.02016689220088481	LAMTOR5:HIPK1:CKS1B:SCAMP3:RIT1:ZMYND11:IPO7:WEE1:HSPA8:EMG1:RAP1B:SLC25A3:PRPSAP2:MOB1A:PCNA:KIF2A:CKS1B:C5orf30:THBS2:RFC2:BET1:CALD1:TERF1:PUF60
Curated_gene_sets	HOLLERN_EMT_BREAST_TUMOR_DN	118	12	0.0016666924272373492	0.02016689220088481	C1orf210:TMEM79:EHF:ESRP2:AP1M2:DEDD2:CEACAM1:CBLC:BCL11A:OCLN:MAP7:CLDN3
Curated_gene_sets	DAIRKEE_CANCER_PRONE_RESPONSE_BPA_E2	118	12	0.0016666924272373492	0.02016689220088481	PTP4A2:EBNA1BP2:POLR2C:KPNB1:RPS6KB1:CHAF1A:DDX39A:FBXO17:HDAC11:GSK3B:EIF4E:BCAP29
Curated_gene_sets	REACTOME_EUKARYOTIC_TRANSLATION_INITIATION	118	12	0.0016666924272373492	0.02016689220088481	RPL22:FAU:RPL3L:RPL19:RPL38:RPL32:RPL14:EIF4E:RPL37:RPS18:RPL7:RPL12
Curated_gene_sets	GINESTIER_BREAST_CANCER_ZNF217_AMPLIFIED_DN	329	24	0.0016726334940486388	0.02016689220088481	RNF207:TARDBP:MTOR:SZT2:SETDB1:SEMA6C:MSTO1:KIAA0907:ARHGEF2:LMNA:SMG5:ARHGEF11:ATN1:DHX33:ENTHD2:TCF3:SAFB2:CIC:ZNF611:ZNF638:RTKN:TRMU:IFRD2:ABCF3
Curated_gene_sets	KIM_WT1_TARGETS_8HR_UP	167	15	0.0016739709824138043	0.02016689220088481	SPEN:NBL1:DNAJB4:HIPK1:HSPA6:ENO2:EMP1:PTHLH:TGFB1I1:DNAJB1:AREG:AREGB:ADAM19:SYNE1:LYN:GEM
Curated_gene_sets	ZHANG_BREAST_CANCER_PROGENITORS_DN	134	13	0.0016753807918204616	0.02016689220088481	ANXA9:ALDH18A1:FNDC3A:PRPF39:RNF166:VAT1:CEACAM1:HDAC11:ACAD11:ARMC8:RP11-766F14.2:TAPBP:GTF2H5
Curated_gene_sets	NABA_ECM_REGULATORS	237	19	0.001696573977766598	0.020377409283174792	CTSS:CTSK:ADAM15:F13B:TLL2:HABP2:ADAMTS7:ADAM11:SERPINB4:MMP24:TGM2:PI3:SLPI:HYAL3:HYAL1:HYAL2:ADAM19:ADAMTSL1:PAPPA
Curated_gene_sets	MEISSNER_NPC_HCP_WITH_H3_UNMETHYLATED	526	34	0.0017219247129851736	0.02063683626608157	HORMAD1:C10orf54:ABCC8:GYLTL1B:P2RX2:GRIN2A:ESRP2:FAM83G:SLC47A1:SPATA32:TRIM25:TUBB4A:AP1M2:CD97:FBXO17:CBLC:MYCN:BFSP1:FAM109B:PKDREJ:TTC38:ODF3B:DAZL:PLSCR4:SLC34A2:NPY5R:CDX1:KCNIP1:C5orf47:CLDN3:ABCB1:GFRA2:S1PR3:C9orf50
Curated_gene_sets	XU_HGF_TARGETS_REPRESSED_BY_AKT1_DN	88	10	0.001731838582431191	0.020666478691166498	SIKE1:FMOD:ATN1:WBP4:STXBP6:N4BP1:BRCA1:RPL38:GSTA1:PPP3CC
Curated_gene_sets	CHANDRAN_METASTASIS_UP	202	17	0.0017356686339427235	0.020666478691166498	TM2D1:HSPA6:DUSP8:KLHL28:SMG1:HES6:OSBP2:NCAPH2:GSK3B:RAB6B:RPL37:CPEB4:AIG1:THBS2:LYN:TERF1:CNTLN
Curated_gene_sets	REACTOME_INTRA_GOLGI_AND_RETROGRADE_GOLGI_TO_ER_TRAFFIC	202	17	0.0017356686339427235	0.020666478691166498	TUBB8:RAB18:VPS51:KIF19:NAPG:TUBB4A:PAFAH1B3:KDELR1:DCTN1:ARFGAP3:RAB43:RAB6B:COPB2:KIF2A:VPS52:COPG2:TUBB4B

Curated_gene_sets	BURTON_ADIPOGENESIS_7	47	7	0.001769216464698715	0.02089369862918787	XPO1:PLXNB2:PROS1:RBP1:PLAC8:S1PR3:PBX3
Curated_gene_sets	REACTOME_INTERLEUKIN_12_SIGNALING	47	7	0.001769216464698715	0.02089369862918787	CDC42:RAP1B:LCP1:BOLA2B:IL12RB1:GSTA2:SOD2
Curated_gene_sets	PID_LKB1_PATHWAY	47	7	0.001769216464698715	0.02089369862918787	MTOR:PSEN2:PRKAB1:CREB1:GSK3B:ESR1:YWHAG
Curated_gene_sets	RAO_BOUND_BY_SALL4	220	18	0.0017699442939831938	0.02089369862918787	TMEM57:DDAH1:DENND2C:ARNT:REEP3:STAMBPL1:WEE1:ANP32A:NBR1:NPEPPS:TRIM25:ARHGEF18:MYCN:FBXO36:LIPH:ZNF608:YWHAG:ZNF34
Curated_gene_sets	LOPEZ_TRANSLATION_VIA_FN1_SIGNALING	35	6	0.001783487357229312	0.020914663804170664	RBM25:ANP32A:SSTR2:RALBP1:TGFBR2:CALD1
Curated_gene_sets	PICCALUGA_ANGIOIMMUNOBLASTIC_LYMPHOMA_DN	135	13	0.0017914342167662494	0.020914663804170664	PTP4A2:HIPK1:CREM:FBXO33:MOAP1:FOXP1:AREG:AREGB:EIF4E:PTGER4:PTP4A1:TNFAIP3:HECA:KLF4
Curated_gene_sets	HOLLERN_EMT_BREAST_TUMOR_UP	135	13	0.0017914342167662494	0.020914663804170664	NBL1:APBB1:RAP1B:IKBIP:PKD1:PLA2G15:TTYH2:RNF157:OSR1:IL18R1:TGFBR2:ADPRH:MRAS
Curated_gene_sets	ZHU_CMV_ALL_DN	119	12	0.0017914752938120522	0.020914663804170664	LMNA:ACTA2:WEE1:POSTN:ERCC5:ANP32A:ANAPC1:RBMS1:IGFBP2:IGFBP5:FBLN1:THBS2
Curated_gene_sets	REACTOME_METABOLISM_OF_AMINO_ACIDS_AND_DERIVATIVES	369	26	0.00179342734470958	0.020914663804170664	RPL22:RIMKLA:PHGDH:ALDH18A1:BBOX1:ASRGL1:FAU:DAO:SDS:SDSL:SLC25A15:PSME1:RPL3L:RPL19:PSMD12:RPL38:PRODH2:TPO:RPL32:RPL14:RPL37:RPS18:INMT:ASL:RPL7:RPL12
Curated_gene_sets	FLECHNER_BIOPSY_KIDNEY_TRANSPLANT_OK_VS_DONOR_UP	568	36	0.0017945321424411114	0.020914663804170664	RPL22:TARDBP:C1orf63:PTP4A2:SLC16A4:HIPK1:ZMYND11:ACTA2:WEE1:BBOX1:HSPA8:C1S:RAP1B:GTF3A:FNDC3A:SCFD1:ANP32A:SMG1:SIAH1:N4BP1:VAT1:RALBP1:ARHGEF18:XPO1:FAM168B:CREB1:DSTN:CYB5R3:TGFBR2:CNBP:DCTD:HLA-A:TAPBP:FAM3C:CREB3L2:TERF1
Curated_gene_sets	REACTOME_GENERIC_TRANSCRIPTION_PATHWAY	1211	66	0.0018100320098467838	0.021050710541579615	MTOR:PHC2:MED8:TAL1:LAMTOR5:HIPK1:TRIM33:CTSK:BGLAP:ZNF496:FAS:GPAM:YAP1:APAF1:NUAK1:PRKAB1:PSME1:FOXG1:ZNF770:MGA:TCF12:GRIN2A:ZNF771:ZNF720:POLR2C:BRCA1:PSMD12:TNRC6C:TAF4B:TCEB3C:TCEB3B:TCF3:ZNF442:ZNF737:ZNF480:ZNF611:ZNF135:CREB1:ZNF343:PCNA:PPARA:HDAC11:ZNF860:ZNF619:ZNF620:ZNF662:NR1I2:GSK3B:OCLN:HDAC3:ZNF311:MDC1:CDKN1A:ESR1:GTF2H5:SOD2:ZNF716:ZNF92:RFC2:YWHAG:BLK:ASH2L:ZNF34:KLF4:ZNF79:NELFB
Curated_gene_sets	ACEVEDO_METHYLATED_IN_LIVER_CANCER_DN	841	49	0.0018338593635420536	0.021265332188738162	LPHN2:HRNR:FLG:MSTO1:FCGR2A:FMOD:TUBB8:LARP4B:C10orf131:CC2D2B:ABCC8:OR8K3:SLC43A1:NCKAP1L:OR10P1:HEATR5A:FBXO33:TXNDC16:TDRD9:MYO5A:ARMC5:ADAM11:TTYH2:OR10H2:ERF:GRWD1:TPO:POTEE:BPIFC:HDAC11:FOXP1:STK32B:NPY5R:SLC6A19:SLC6A18:OCLN:ARAP3:OR10C1:RAB44:C6orf89:CNR1:ZNF92:CPA1:MEST:GML:SPATA31A4:SPATA31A7:DIRAS2:PTPN3:FCN2:OBP2A
Curated_gene_sets	BERENJENO_TRANSFORMED_BY_RHOA_DN	389	27	0.0018362175585621935	0.021265332188738162	RPL22:C1orf63:C1orf85:CSRP1:PSAP:ATN1:POSTN:LMO7:PCK2:TXNDC16:COQ9:MYL12B:HES6:LSS:NAGA:NDUFA6:ARFGAP3:ARSA:GNAI2:SLC12A2:NDFIP1:PDGFRB:TAPBP:THBS2:NRBP2:XPA:NACC2
Curated_gene_sets	REACTOME_SIGNALING_BY_RECEPTOR_TYROSINE_KINASES	468	31	0.0018490649942257036	0.02136913137234369	MTOR:CDC42:VAV3:NRAS:RIT1:PSEN2:YAP1:PTPN6:NCKAP1L:RAP1B:PSEN1:SH3GL3:POLR2C:ESRP2:MAPK7:SH3GL1:INSR:EPS15L1:ATP6V1B1:CREB1:LRIG1:AP2M1:AREG:AREGB:DIAPH1:PDGFRB:ESR1:THBS2:HSPB1:LYN:PTPN3:NELFB
Curated_gene_sets	YOSHIMURA_MAPK8_TARGETS_DN	351	25	0.0018787781198309132	0.021617680822144374	RPL22:CDC42:HOOK1:RUSC1:SOAT1:FMOD:PSEN2:PSAP:GPAM:SCGB1D2:FDX1:EMP1:SCFD1:CYB5B:RABEP1:KPNB1:RPS6KB1:RALBP1:TCF3:IGFBP5:RPN2:LSS:SLC4A4:PCDHGC3:EDN1
Curated_gene_sets	MARTORIATI_MDM4_TARGETS_NEUROEPITHELIUM_UP	169	15	0.0018823612277417115	0.021617680822144374	SOAT1:PHLDA3:TPI1:C12orf75:ERCC5:ERO1L:NOMO1:GTSE1:PROS1:PLA2G12A:PRELID2:CDKN1A:TCTE3:EXOC4:CALD1
Curated_gene_sets	CHIANG_LIVER_CANCER_SUBCLASS_CTNNB1_DN	169	15	0.0018823612277417115	0.021617680822144374	F3:HABP2:ASRGL1:POU2AF1:SDSL:KCTD12:TNFSF13B:PAPLN:ZNF83:RALGAPA2:SLPI:SLC12A2:THBS2:TPST1:GEM

Curated_gene_sets	BRUINS_UVC_RESPONSE_VIA_TP53_GROUP_C	89	10	0.0018865360905982553	0.021620489654960422	MTOR:COCH:MESDC1:SNAI3:DHX33:IL1RL1:NAGA:CXCL6:SNCB:GEM
Curated_gene_sets	MCCABE_BOUND_BY_HOXC6	449	30	0.0019047488231589671	0.021783832175046734	ARHGEF11:STAMBPL1:ALDH18A1:ZFYVE27:DUSP8:APBB1:OR4C6:OR8K3:LRRC23:PTHLH:COCH:PSEN1:RFX7:MESDC1:PKD1:SMG1:CD2BP2:LYZL6:DGKE:ASXL3:AP1M2:DDX39A:CARD8:ZNF534:FAM49A:ZNFX1:FAM109B:SMDT1:MEGF10:SEPT7
Curated_gene_sets	CHUNG_BLISTER_CYTOTOXICITY_UP	136	13	0.0019140468919260183	0.02184475508814321	SYT11:TPI1:SLC11A2:PCK2:TCF12:NTAN1:PDE4A:WDR54:COPS8:MDC1:CDKN1A:TNFAIP3:TUBB4B
Curated_gene_sets	CERVERA_SDHB_TARGETS_1_UP	120	12	0.001923824621280222	0.021910888699094206	RIMKLA:UBASH3B:DYRK4:PTHLH:TNFRSF19:PCDH9:MFAP4:CCDC74A:IGFBP2:CAND2:LIPH:EDN1
Curated_gene_sets	ENK_UV_RESPONSE_EPIDERMIS_UP	295	22	0.001941079595116631	0.022040359827158785	EBNA1BP2:CDC20:CERS2:FAM189B:PHLDA3:EHF:PRKAB1:POLE:SLC25A15:JMJD7-PLA2G4B:ANP32A:SPG7:PITPNC1:PAFAH1B3:BCL11A:TPD52L2:SCO2:CHKB:ENTPD3:THBS2:CD36:CA2
Curated_gene_sets	KEGG_GLYCOSPHINGOLIPID_BIOSYNTHESIS_GANGLIO_SERIES	15	4	0.0019512189121662113	0.022040359827158785	ST8SIA5:B3GALT4:RP11-203J24.9:ST6GALNAC6:ST6GALNAC4
Curated_gene_sets	GARGALOVIC_RESPONSE_TO_OXIDIZED_PHOSPHOLIPIDS_SALMON_DN	15	4	0.0019512189121662113	0.022040359827158785	TRIM25:FAM49A:VGLL4:LYN
Curated_gene_sets	NIKOLSKY_BREAST_CANCER_12Q24_AMPLICON	15	4	0.0019512189121662113	0.022040359827158785	ULK1:P2RX2:POLE:ANKLE2
Curated_gene_sets	BROWNE_HCMV_INFECTION_16HR_UP	222	18	0.0019555342396253414	0.022043839861022547	STIL:TRIM33:SIKE1:CREM:PRKRIR:ENO2:PTHLH:MOAP1:SMG1:RND2:FAM49A:PLEKHB2:MAP3K13:ISL1:ARL4A:ABCB1:LYN:KHDRBS3
Curated_gene_sets	SENGUPTA_EBNA1_ANTICORRELATED	153	14	0.001977771133557067	0.022248914122080625	ZBTB17:ANXA9:SYT11:NHLH1:HPX:FITM1:SMPD3:SPG7:EPN2:PLEKHM1:NAT6:FCHSD1:HLA-A:B3GALT4
Curated_gene_sets	LEIN_OLIGODENDROCYTE_MARKERS	75	9	0.0019842035589654953	0.022275721995651407	CERS2:SYT11:CSRP1:OPALIN:TTYH2:MBP:S1PR5:CA2:NRBP2
Curated_gene_sets	REACTOME_REGULATION_OF_EXPRESSION_OF_SLITS_AND_ROBOS	170	15	0.001994396356862277	0.022344550629530315	RPL22:CUL2:FAU:PSME1:RPL3L:RPL19:PSMD12:RPL38:RPL32:RPL14:RPL37:ISL1:RPS18:RPL7:RPL12
Curated_gene_sets	REACTOME_DNA_DAMAGE_BYPASS	48	7	0.0020050156274261832	0.022372395469516092	UBE2L6:POLE:TRIM25:NPLOC4:PCNA:RAD18:RFC2
Curated_gene_sets	RADAEVA_RESPONSE_TO_IFNA1_UP	48	7	0.0020050156274261832	0.022372395469516092	UBE2L6:PSME1:IFI35:BRCA1:CYB561D2:XXcos-LUCA11.4:TUBB:RFC2
Curated_gene_sets	MATZUK_SPERMATID_DIFFERENTIATION	36	6	0.0020729289697125506	0.023036731843209576	CREM:DDX25:FNDC3A:PANK2:SLC12A2:MAP7
Curated_gene_sets	UZONYI_RESPONSE_TO_LEUKOTRIENE_AND_THROMBIN	36	6	0.0020729289697125506	0.023036731843209576	F3:CREM:KCNJ2:IL8:GEM:KLF4
Curated_gene_sets	RODRIGUES_THYROID_CARCINOMA_POORLY_DIFFERENTIATED_UP	635	39	0.0020806131354273428	0.023075509794326234	ACOT7:STIL:SSX2IP:ABCD3:NRAS:SIKE1:PHGDH:CKS1B:SOAT1:ARL5B:REEP3:TRUB1:ENO2:IKBIP:CIT:GPR180:ERO1L:DLGAP5:CPSF2:C14orf142:BRCA1:DDX39A:MRPS12:MOB1A:MTHFD2:FAM168B:RPE:COPS8:PCNA:GTSE1:RAD18:CFI:C5orf22:SKIV2L2:KIF2A:CKS1B:UTP15:TMEM161B:RFC2:LY6E
Curated_gene_sets	JOHNSTONE_PARVB_TARGETS_3_UP	432	29	0.002094620325472024	0.023184117525999203	NBL1:CTSS:PSAP:GRK5:DMBT1:TM7SF2:SYVN1:ENO2:LPCAT3:C1S:NPIPA1:NTAN1:IFI35:VAT1:HPCAL1:TMEM87B:TGM2:CELSR1:TGFBR2:MFI2:AREG:AREGB:PCDHGC3:NDFIP1:TAPBP:PTP4A1:RNASET2:CLDN4:CALD1:KLF4

Curated_gene_sets	VALK_AML_CLUSTER_8	25	5	0.0021188839218881416	0.023405583241579653	RHCE:TAL1:DCAF11:BCAM:OSBP2
Curated_gene_sets	STARK_PREFRONTAL_CORTEX_22Q11_DELETION_DN	513	33	0.0021528171296153085	0.023732759579185997	PHC2:C1orf123:SCNM1:FDPS:DAP3:RIT1:SAC3D1:TM7SF2:NDUFA9:TPI1:GTF3A:DCAF11:FKBP3:NIPA1:PDXDC1:NTAN1:TRAPPC2L:MRPS12:MZT2A:CHMP4B:EIF6:NPRL2:NDUFB4:MRPS22:AP2M1:TMEM44:COMMD8:EIF4E:PFDN6:BCAP29:DPM2:FAM102A:UBAC1
Curated_gene_sets	GAVIN_FOXP3_TARGETS_CLUSTER_P2	76	9	0.002177003663218556	0.023920654019012614	IQCC:FCGR2A:NCKAP1L:TBX21:KIAA1841:LIME1:CD200R1:RAB6B:ABCB1
Curated_gene_sets	REACTOME_TRANSPORT_OF_INORGANIC_CATIONS_ANIONS_AND_AMINO_ACIDS_OLIGOPEPTIDES	106	11	0.002178558019182934	0.023920654019012614	SLC43A1:SLC7A6:SLC4A5:SLC9A4:SLC25A26:SLC34A2:SLC4A4:SLC6A19:SLC6A18:SLC12A2:SLC34A3
Curated_gene_sets	HAHTOLA_MYCOSIS_FUNGOIDES_CD4_UP	62	8	0.0021888298549811126	0.02398556380926514	FAM49A:IL8:CXCL1:MCTP1:IER3:CDKN1A:SOD2:KLF4
Curated_gene_sets	BRUINS_UVC_RESPONSE_VIA_TP53_GROUP_B	534	34	0.002197802386420709	0.024011697500404664	ELOVL1:BCL10:F3:HCN3:SEMA4A:ZBTB41:ECHDC3:FAS:SMPD1:ZFPL1:VPS51:C11orf88:APAF1:ULK1:ERCC5:FRMD6:KIAA1199:PKD1:CLEC10A:SH3GL1:UBXN6:DNAJB1:METTL21A:RPE:RASSF1:NR1I2:AREG:AREGB:MTTP:TSPAN17:INMT:GNG11:KEL:UBQLN1:PAPPA
Curated_gene_sets	KEGG_CHEMOKINE_SIGNALING_PATHWAY	189	16	0.002199944653736403	0.024011697500404664	CDC42:VAV3:NRAS:GRK5:RAP1B:CCL17:NFKBIB:GSK3A:GNAI2:GSK3B:IL8:CXCL6:PF4V1:CXCL1:GNG11:LYN
Curated_gene_sets	JAATINEN_HEMATOPOIETIC_STEM_CELL_UP	317	23	0.002210129556767122	0.024075094439160272	SAMD13:DDAH1:VAV3:IPO7:EMP1:EBPL:KDELC1:BIVM:FRMD6:BCL11A:SCN3A:SHANK3:ATP2C1:TMEM44:AREG:AREGB:PLA2G12A:IPO11:MAP7:HOXA10:ZNF117:HSPB1:MEST:KHDRBS3
Curated_gene_sets	ROZANOV_MMP14_TARGETS_UP	261	20	0.0022213935560606787	0.02414997223693635	CDC42:SLC16A4:PAQR6:FCRLA:ALDH18A1:ENO2:FRMD6:ERO1L:TTYH2:IGFBP2:DSTN:KIAA1644:LRIG1:TMEM44:COMMD8:PLAC8:MEGF10:WDR46:CALD1:PAEP
Curated_gene_sets	UDAYAKUMAR_MED1_TARGETS_DN	243	19	0.0022535974076727827	0.02445175412151475	C1orf63:PTBP2:PHLDA3:CREM:FAS:GRK5:HOXC13:MAP3K9:SPAG9:PHLPP1:ERF:CPT1B:RASSF1:PFDN6:CDKN1A:ARL4A:PILRB:RB1CC1:KLF4
Curated_gene_sets	MIYAGAWA_TARGETS_OF_EWSR1_ETS_FUSIONS_UP	262	20	0.0023221580877823707	0.02511711237682609	TRIM62:HIPK1:NID1:FAS:GRK5:SLC43A1:UBE2L6:KIAA0226L:KCTD12:TCF12:SH3GL3:IMPA2:CD79A:CEACAM1:IGFBP2:TGM2:PPARA:TMEM44:HOXA13:STAR
Curated_gene_sets	REACTOME_CARDIAC_CONDUCTION	139	13	0.0023240520268686567	0.02511711237682609	HIPK1:KCNJ11:ORAI1:KCNK10:CACNB1:CACNG1:KCNJ2:KCNJ14:SCN3A:SCN5A:CACNA2D2:FGF12:KCNIP1
Curated_gene_sets	HELLER_SILENCED_BY_METHYLATION_DN	107	11	0.0023473892963927793	0.025319585332268	DNAJB4:DDAH1:PHGDH:SYT11:HSPA6:MIA2:CTAGE5:PKD1:SLC47A1:DNAJB1:CEACAM1:CA2
Curated_gene_sets	MARTENS_TRETINOIN_RESPONSE_UP	852	49	0.0023763527185648235	0.025581832299070635	ANGPTL7:C1orf210:KCNC4:GJA5:SEMA6C:FMOD:ECHDC3:GDF2:CDH23:C10orf54:OR8U1:USP5:ENO2:HOXC13:MLNR:SIX6:CHRNB4:CACNG1:AZI1:TCEB3B:MUC16:PTGER1:CHST8:CD79A:BCAM:ZSCAN1:HPCAL1:ITPRIPL1:TTLL4:TGM2:LSS:APOL1:CYP2D6:PKDREJ:PLXNB2:DENND6B:SCN5A:SLC6A19:SLC6A18:LECT2:SNCB:EIF4E1B:DACT2:CLDN3:ZNF467:GEM:CYP11B1:NRBP2:ZNF34
Curated_gene_sets	MCBRYAN_PUBERTAL_BREAST_3_4WK_DN	37	6	0.0023964541869204103	0.02569765006286389	PHGDH:ADCK3:TXNDC16:MBP:RPL14:CDKN1A
Curated_gene_sets	BERNARD_PPAPDC1B_TARGETS_UP	37	6	0.0023964541869204103	0.02569765006286389	SMPD1:WBP4:SCFD1:ANP32A:NPEPPS:KLHL2
Curated_gene_sets	REACTOME_MISCELLANEOUS_TRANSPORT_AND_BINDING_EVENTS	26	5	0.0025430033169663246	0.027068596383865773	LRRC8B:LRRC8C:NIPA2:NIPA1:PIP

Curated_gene_sets	TOMIDA_METASTASIS_UP	26	5	0.0025430033169663246	0.027068596383865773	GNAI2:MRPS22:CXCL1:TUBB:IER3
Curated_gene_sets	REACTOME_CONSTITUTIVE_SIGNALING_BY_AKT1_E17K_IN_CANCER	26	5	0.0025430033169663246	0.027068596383865773	MTOR:GSK3A:CREB1:GSK3B:CDKN1A
Curated_gene_sets	CHEN_NEUROBLASTOMA_COPY_NUMBER_GAINS	50	7	0.002548906185573981	0.027068596383865773	TPO:HPCAL1:MYCN:ALK:TERT:HDAC3:ARAP3
Curated_gene_sets	WONG_PROTEASOME_GENE_MODULE	50	7	0.002548906185573981	0.027068596383865773	CDC20:UCHL3:PSME1:UBR7:SIAH1:PSMD12:USP25
Curated_gene_sets	MYLLYKANGAS_AMPLIFICATION_HOT_SPOT_29	8	3	0.002580604812509506	0.027299821295413064	HOXA11:HOXA13:JAZF1
Curated_gene_sets	BIOCARTA_EPHA4_PATHWAY	8	3	0.002580604812509506	0.027299821295413064	SELP:RAP1B:LYN
Curated_gene_sets	DUTERTRE_ESTRADIOL_RESPONSE_6HR_UP	228	18	0.002613261050449087	0.02759222464207376	MACF1:ANXA9:MCM10:TMEM120B:BRI3BP:SLC47A1:BRCA1:CHAF1A:GREB1:PCNA:TGM2:RPL32:LRIG1:AREG:AREGB:NPY1R:WDR46:DPM2:FAM102A
Curated_gene_sets	WEST_ADRENOCORTICAL_TUMOR_DN	540	34	0.0026254197130605892	0.02761459625534666	NBL1:CSRP1:ADCK3:ZMYND11:CREM:FAS:VPS51:C1S:POSTN:LHFP:CD2BP2:SLC47A1:IFI35:NPEPPS:RPL38:IGFBP5:CTDSP1:DDX27:FBLN1:FGF12:NPY1R:CDH6:PTGER4:PCDHGC3:IER3:ESR1:GTF2H5:ABCB1:MEPCE:CALD1:GEM:CYP11B1:CYP11B2:LY6E
Curated_gene_sets	ACEVEDO_LIVER_CANCER_DN	540	34	0.0026254197130605892	0.02761459625534666	TMEM82:HOOK1:PHGDH:ARNT:TOR3A:BBOX1:TM7SF2:DGAT2:LPCAT3:POLE:EBPL:TGDS:FITM1:TRAPPC2L:UBXN6:PEX11G:PRODH2:SARS2:RND3:ACAD11:PLSCR4:MAN2B2:CFI:LECT2:DIAPH1:IER3:MAP7:AIG1:ESR1:INMT:CLDN3:LY6E:PBX3:RPL12
Curated_gene_sets	TAKEDA_TARGETS_OF_NUP98_HOXA9_FUSION_16D_UP	175	15	0.002641100288198431	0.027726512758357955	B3GALNT2:C12orf75:TDRD9:SIGLEC6:AFF3:CFI:C5orf20:TIFAB:ADAM19:ABCB1:PRKAR2B:CDH17:GEM:LY6E:S1PR3
Curated_gene_sets	DAVICIONI_RHABDOMYOSARCOMA_PAX_FOXO1_FUSION_UP	64	8	0.002682803460540633	0.028110670164636233	VAV3:NTAN1:MYCN:FAM49A:ALK:TNFAIP3:PRKAR2B:FAM3C
Curated_gene_sets	LIU_VMYB_TARGETS_UP	125	12	0.0027112553310004164	0.028288036486350084	ABCD3:ASRGL1:SAC3D1:TPI1:IMPA2:PHLPP1:NCOA1:USP25:ANKRA2:MARCH3:WDR46:PRKAR2B
Curated_gene_sets	KIM_WT1_TARGETS_8HR_DN	125	12	0.0027112553310004164	0.028288036486350084	YY1AP1:REXO2:GPRC5A:SLC11A2:SIX6:ANP32A:NFATC3:ERF:SEMG1:PLAC8:SOD2:CALD1
Curated_gene_sets	YAMAZAKI_TCEB3_TARGETS_DN	193	16	0.0027151578376282213	0.028288036486350084	EBNA1BP2:CUL2:ULK1:IPO5:G2E3:FKBP3:SEPT1:SEPT1:IGFBP2:DIAPH1:TUBB:SOD2:ARL4A:ASH2L:ZNF34:TUBB4B:NELFB
Curated_gene_sets	BENPORATH_MYC_TARGETS_WITH_EBOX	229	18	0.0027390636238120865	0.028483154999225498	RPL22:LMNA:PSEN2:WEE1:UCP3:HSPA8:TCF12:RPL19:IMPA2:INSR:NCL:IFRD2:GSK3B:MFI2:EIF4E:TERT:HDAC3:BLK
Curated_gene_sets	GAUSSMANN_MLL_AF4_FUSION_TARGETS_D_UP	38	6	0.0027565066306163484	0.028502900328985965	GPR153:F5:C10orf54:SLC16A12:KCNAB1:POPDC3
Curated_gene_sets	REACTOME_GENE_AND_PROTEIN_EXPRESSION_BY_JAK_STAT_SIGNALING_AFTER_INTERLEUKIN_12_STIMULATION	38	6	0.0027565066306163484	0.028502900328985965	CDC42:RAP1B:LCP1:BOLA2B:GSTA2:SOD2

Curated_gene_sets	HU_GENOTOXIN_ACTION_DIRECT_VS_INDIRECT_4HR	38	6	0.0027565066306163484	0.028502900328985965	C1orf63:CLK2:DMBT1:KCNJ2:TMEM230:ACKR4
Curated_gene_sets	LIM_MAMMARY_LUMINAL_MATURE_DN	94	10	0.0028357631119053637	0.029189905565239677	FAM171A1:FAS:KCTD12:RASL12:STAC2:FAM49A:BCL11A:MERTK:TPST1:CALD1
Curated_gene_sets	HECKER_IFNB1_TARGETS	94	10	0.0028357631119053637	0.029189905565239677	PDZK1IP1:IFI35:ZBP1:APOBEC3B:SCO2:TYMP:PROS1:GP9:IL8:LY6E
Curated_gene_sets	QUINTENS_EMBRYONIC_BRAIN_RESPONSE_TO_IR	79	9	0.0028461722026213777	0.029189905565239677	DENND2C:PHLDA3:FAS:APAF1:ERCC5:DCAF4:HPCAL1:GTSE1:CDKN1A
Curated_gene_sets	MEISSNER_NPC_HCP_WITH_H3K27ME3	79	9	0.0028461722026213777	0.029189905565239677	HES2:SFRP5:HOXC13:HOXC12:HOXC11:SIX6:HNF1B:HOXA10:HOXA11
Curated_gene_sets	PLASARI_TGFB1_TARGETS_10HR_UP	194	16	0.002858502357583988	0.029189905565239677	MFSD2A:LRRC8C:HECTD2:PTHLH:NUAK1:OTUD7A:CCL17:ZNF469:IL18R1:CDH6:MEGF10:IER3:TNFAIP3:ABCB1:CREB3L2:KHDRBS3
Curated_gene_sets	CHIARADONNA_NEOPLASTIC_TRANSFORMATION_KRAS_CDC25_DN	51	7	0.0028600907288973253	0.029189905565239677	NID1:FAS:IL1RL1:SLPI:PROS1:CXCL6:SLC12A2
Curated_gene_sets	ROESSLER_LIVER_CANCER_METASTASIS_DN	51	7	0.0028600907288973253	0.029189905565239677	HIPK1:TRIM33:PRUNE:MOAP1:N4BP1:TRMU:ARAP3
Curated_gene_sets	RICKMAN_TUMOR_DIFFERENTIATED_WELL_VS_MODERATELY_DN	110	11	0.002918364023655285	0.02972948239653282	LRFN4:NUMB:NIPA2:IMPA2:WFDC5:WFDC12:PI3:AREG:AREGB:CDKN1A:PTP4A1:IPPK
Curated_gene_sets	MIKKELSEN_ES_ICP_WITH_H3K4ME3	711	42	0.002923953565144621	0.029731365179039855	TMEM82:CCDC28B:TMEM125:DENND2C:GOLPH3L:SCNM1:LYSMD1:FAM189B:ECHDC3:NSUN6:ARL5B-AS1:TACR2:TTC12:EMP1:BIVM:FITM1:DCAF4:CHRNB4:HAPLN3:TGFB1I1:RPL19:TMEM106A:TBKBP1:CACNG1:C19orf68:CTU1:SLC11A1:CYP2D6:MIOX:CPT1B:EFHB:ENTPD3:TRAK1:HYAL1:LIPH:LSG1:GPRIN3:MEGF10:PLEKHG1:SRRM3:GNG11:EXOC4:CALD1
Curated_gene_sets	PLASARI_TGFB1_TARGETS_10HR_DN	249	19	0.002956968402804676	0.029990603528135124	SLC16A4:SYT11:METTL7A:PCDH9:LMO7:SLC14A1:OSR1:RND3:IGFBP5:CAND2:ZNF608:SLC12A2:PLEKHG1:CD36:PRKAR2B:MEST:ZNF467:S1PR3:DIRAS2
Curated_gene_sets	JAZAG_TGFB1_SIGNALING_VIA_SMAD4_DN	65	8	0.0029603522479144466	0.029990603528135124	SCAMP3:PKLR:MAPK7:POU2F2:VIL1:RASSF1:PDGFRB:CLDN4
Curated_gene_sets	STARK_PREFRONTAL_CORTEX_22Q11_DELETION_UP	195	16	0.003008053174640719	0.03041783182665183	RPL22:OPCML:ATN1:TNFRSF19:FNDC3A:RBM25:ANKRD11:SAFB2:FAM49A:TRAK1:GSK3B:SLC4A4:GPRIN3:LSM11:CNR1:NACC2
Curated_gene_sets	VALK_AML_CLUSTER_7	27	5	0.0030254364526428636	0.030537478763281455	RHCE:PDZK1IP1:TAL1:OSBP2:KEL
Curated_gene_sets	DEURIG_T_CELL_PROLYMPHOCYTIC_LEUKEMIA_UP	364	25	0.0030408459338173615	0.03063680124895477	SSX2IP:ARL2:RNASEH2B:KCTD12:STXBP6:SIX6:PKD1:TBC1D10B:N4BP1:SLC7A6:TAF4B:EPS15L1:CIC:MERTK:IGFBP5:SLC11A1:IFRD2:CXCL1:IER3:MEST:NCAPG2:SCRIB:PUF60:KLF4:ST6GALNAC4
Curated_gene_sets	REACTOME_SLC_MEDIATED_TRANSMEMBRANE_TRANSPORT	250	19	0.003090361480033076	0.03107290340940860 7	SLC43A1:ARL2:SLC11A2:SLC7A6:SLC5A10:SLC47A1:SLC47A2:SLC14A1:SLC4A5:SLC9A4:SLC11A1:SLC25A26:SLC34A2:SLC4A4:SLC6A19:SLC6A18:SLC12A2:SLC35D2:SLC34A3
Curated_gene_sets	OISHI_CHOLANGIOMA_STEM_CELL_LIKE_UP	345	24	0.0030954282981923136	0.031072903409408607	HOOK1:PHGDH:SETDB1:CERS2:ANXA9:SCNM1:SCAMP3:GON4L:SSR2:SMG5:APCS:SNRPE:PSEN2:HABP2:AASDHPPT:RBM7:HNF1B:FBXO17:MEGF8:SLC6A16:ZNF480:NDUFAF5:SYNGR1:PBX3

Curated_gene_sets	KIM_ALL_DISORDERS_CALB1_CORR_UP	546	34	0.003122585170982205	0.03128841716862133	ACOT7:CLSTN1:SCAMP3:RUSC1:SYT11:PSAP:DUSP8:AASDHPPT:OPCML:ENO2:CSRNP2:SLC25A3:FOXG1:FAM179B:MOAP1:ARPP19:GRIN2A:PSMD12:TECR:MAST3:FAM49A:PUM2:DCTN1:FAM168B:COPS8:DSTN:RTCB:KCNAB1:ABCF3:SNCB:PRKAR2B:CALB1:KHDRBS3:TUBB4B
Curated_gene_sets	WATANABE_RECTAL_CANCER_RADIOTHERAPY_RESPONSIVE_UP	111	11	0.003131761673860371	0.031323310850738	C1orf63:ABCD3:HIPK1:TRIM33:PDXDC1:CYB5B:RPL38:IMPA2:DDX27:GNAI2:TMEM115
Curated_gene_sets	REACTOME_FORMATION_OF_FIBRIN_CLOT_CLOTTING_CASCADE	39	6	0.003155569037410446	0.03139020845351693	F3:F5:F13B:PROS1:GP9:PF4V1
Curated_gene_sets	MIKKELSEN_MEF_ICP_WITH_H3K4ME3_AND_H3K27ME3	39	6	0.003155569037410446	0.03139020845351693	RNF207:OPCML:CORO1A:KCNJ2:MCHR1:NPY1R
Curated_gene_sets	REACTOME_TRANSLESION_SYNTHESIS_BY_Y_FAMILY_DNA_POLYMERASES_BYPASSES_LESIONS_ON_DNA_TEMPLATE	39	6	0.003155569037410446	0.03139020845351693	UBE2L6:POLE:TRIM25:NPLOC4:PCNA:RFC2
Curated_gene_sets	FERREIRA_EWINGS_SARCOMA_UNSTABLE_VS_STABLE_UP	161	14	0.0031640429134834696	0.03141740306488748	DAP3:ZFYVE27:NDUFA9:NCKAP1L:FANCM:DLGAP5:GJC1:CHAF1A:PDE4A:ESF1:LSS:KRBOX1:KRBOX1:C5orf30:NCAPG2
Curated_gene_sets	LOCKWOOD_AMPLIFIED_IN_LUNG_CANCER	214	17	0.0031744695507372554	0.03141740306488748	CTSK:ARNT:SETDB1:CERS2:CKS1B:RIT1:SSR2:PMF1:ARHGEF11:CUL2:PC:COCH:DVL3:AP2M1:C5orf22:CKS1B:LY6E:PUF60
Curated_gene_sets	HAN_SATB1_TARGETS_DN	385	26	0.0031884218481425444	0.03141740306488748	DNAJB4:LRRC8B:PRUNE:ADAM15:LMNA:LARP4B:PC:LRRC23:EMP1:METTL7A:ANKLE2:STXBP6:FOXG1:POLR2C:CALB2:RABEP1:GRWD1:PI3:LSS:CXCL1:AREG:AREGB:NPY1R:MDC1:CLDN4:KHDRBS3:PAEP
Curated_gene_sets	REACTOME_COPI_INDEPENDENT_GOLGI_TO_ER_RETROGRADE_TRAFFIC	52	7	0.0031995499959976835	0.03141740306488748	TUBB8:RAB18:TUBB4A:PAFAH1B3:DCTN1:RAB6B:TUBB4B
Curated_gene_sets	ROSS_AML_WITH_CBFB_MYH11_FUSION	52	7	0.0031995499959976835	0.03141740306488748	CTSS:KCTD12:CLEC10A:HPCAL1:NAGA:PLXNB2:NACC2
Curated_gene_sets	KEGG_STEROID_BIOSYNTHESIS	17	4	0.003203992568515157	0.03141740306488748	SOAT1:TM7SF2:LSS:CEL
Curated_gene_sets	GENTILE_UV_RESPONSE_CLUSTER_D1	17	4	0.003203992568515157	0.03141740306488748	WEE1:RND3:ZNF117:GEM
Curated_gene_sets	REACTOME_HYALURONAN_METABOLISM	17	4	0.003203992568515157	0.03141740306488748	KIAA1199:HYAL3:HYAL1:HYAL2
Curated_gene_sets	VANHARANTA_UTERINE_FIBROID_WITH_7Q_DELETION_UP	66	8	0.0032597460470094016	0.031907229545549316	PHGDH:GON4L:EMG1:UBR7:TRMT61B:IGFBP5:NPRL2:PTP4A1
Curated_gene_sets	ONKEN_UVEAL_MELANOMA_DN	527	33	0.003268702903805713	0.031938072244822785	RPL22:GPR153:PTP4A2:CSDE1:ARL2:VPS51:ERC1:LETMD1:POSTN:IPO5:TUBB4A:DSTN:CAND2:RPL32:EIF1B:RPL14:TRAK1:HYAL2:CGGBP1:IFT57:GTPBP8:NDUFB4:CNBP:RAB6B:PLSCR4:DVL3:AP2M1:LSG1:PTP4A1:GNG11:FAM3C:MEST:SLC35D2
Curated_gene_sets	GAVIN_FOXP3_TARGETS_CLUSTER_T4	96	10	0.003308412214872295	0.03226875105321364	HIPK1:SYT11:SEMA4A:PLEKHB2:RPN2:APOL2:LRIG1:ATP10D:GPRIN3:ITGB8

Curated_gene_sets	CAIRO_LIVER_DEVELOPMENT_UP	162	14	0.003346455872676471	0.03258204204529782	LPHN2:PTBP2:LRFN4:UCHL3:FKBP3:ARPP19:SH3GL3:GJC1:PAFAH1B3:GRWD1:MYCN:IGFBP5:SLC12A2:MEST
Curated_gene_sets	GOLDRATH_HOMEOSTATIC_PROLIFERATION	145	13	0.0033608034350262345	0.03266392172452176	RAB18:IPO7:KCTD12:CPSF2:SIAH1:UBALD2:IMPA2:CREB1:C3orf38:CETN3:C5orf30:ZFP62:NACC2
Curated_gene_sets	HINATA_NFKB_TARGETS_FIBROBLAST_UP	81	9	0.003375816534744666	0.03275196959017709	RABEP1:RND3:IL8:CXCL6:CXCL1:EDN1:IER3:TNFAIP3:SOD2
Curated_gene_sets	RODRIGUES_THYROID_CARCINOMA_ANAPLASTIC_UP	717	42	0.0033909809963504458	0.03284117334669683	CDC20:MED8:STIL:SSX2IP:NRAS:SIKE1:UHMK1:ASPM:STAMBPL1:FAS:TLL2:TRUB1:REXO2:G2E3:CPSF2:RPS6KB1:PSMD12:TTC39C:DDX39A:MOB1A:MTHFD2:ANAPC1:FAM168B:RND3:RBMS1:RPE:ESF1:TGM2:DDX27:GSK3B:XRN1:IL8:CXCL1:AREG:AREGB:NIPBL:KIF2A:UTP15:PCDHGC3:IER3:ZNF92:ESYT2:KIAA0020
Curated_gene_sets	BLALOCK_ALZHEIMERS_DISEASE_INCIPIENT_UP	387	26	0.003414619265411262	0.033011986957868805	CSDE1:CTSS:SSR2:NUCKS1:PC:DAO:KCNK10:RABEP1:TUBD1:SLC14A1:MBP:TCF3:GIPC1:ERF:KCNJ14:ZNF83:CTDSP1:DGCR6:APOL2:PLXNB2:FAM208A:GSK3B:ATP10D:RNASET2:TSC22D4:ST6GALNAC4
Curated_gene_sets	BENPORATH_OCT4_TARGETS	290	21	0.003427667247991591	0.033055169316766164	ICMT:TAL1:PHGDH:SMG5:NUCKS1:WEE1:TCF12:SPAG9:NPLOC4:OSR1:RPL32:DBR1:LSG1:PPAP2A:VPS52:RPS18:B3GALT4:THBS2:RDH10:CA2:TSTD2
Curated_gene_sets	SHEN_SMARCA2_TARGETS_UP	427	28	0.003431103741114975	0.033055169316766164	MACF1:HIPK1:ZMYND11:CUL2:CTR9:AASDHPPT:TFCP2:SCFD1:NUMB:HERC2:ANP32A:NBR1:NPEPPS:PSMD12:PUM2:NCOA1:XPO1:PKP4:COPS8:TMEM230:CNBP:ARMC8:DHX29:CETN3:NDFIP1:GTF2H5:CHMP5:XPA
Curated_gene_sets	REACTOME_NEDDYLATION	234	18	0.0034463450944002254	0.03314046497073258	CUL2:LINC00610:COMMD9:SPSB2:FBXW8:COMMD6:UCHL3:LMO7:DCAF11:PSME1:DCAF4:KBTBD13:PSMD12:KEAP1:FBXO17:PUM2:COPS8:COMMD8:KLHL2
Curated_gene_sets	SARRIO_EPITHELIAL_MESENCHYMAL_TRANSITION_UP	180	15	0.003452006258540223	0.03314046497073258	STIL:LRRC8C:F3:CKS1B:ASPM:STAMBPL1:CDCA5:GPRC5A:HOXC13:CIT:BRCA1:PCNA:CKS1B:TUBB:RFC2:TUBB4B
Curated_gene_sets	REACTOME_SIGNALING_BY_ROBO_RECEPTORS	216	17	0.003490567260212456	0.03338779643400548	RPL22:CDC42:ABL2:CUL2:FAU:PSME1:RPL3L:RPL19:PSMD12:RPL38:RPL32:RPL14:RPL37:ISL1:RPS18:RPL7:RPL12
Curated_gene_sets	KIM_WT1_TARGETS_12HR_DN	216	17	0.003490567260212456	0.03338779643400548	YY1AP1:REXO2:GPRC5A:UCHL3:RBM25:ANP32A:PPP1CB:MOB1A:RND3:TGFBR2:ARMC8:PLAC8:SOD2:HOXA10:LAT2:FAM3C:CALD1
Curated_gene_sets	REACTOME_MITOTIC_PROMETAPHASE	198	16	0.003495977230682995	0.03338779643400548	CDC20:PMF1:TUBB8:CDCA5:AZI1:TUBB4A:XPO1:DCTN1:MZT2A:KIF2A:TUBB:FGFR1OP:YWHAG:PRKAR2B:CEP41:TUBB4B
Curated_gene_sets	REACTOME_ANCHORING_OF_THE_BASAL_BODY_TO_THE_PLASMA_MEMBRANE	97	10	0.0035671218109179503	0.03379967598081336	B9D1:AZI1:TUBB4A:DCTN1:TUBB:FGFR1OP:YWHAG:PRKAR2B:CEP41:TUBB4B
Curated_gene_sets	BECKER_TAMOXIFEN_RESISTANCE_DN	53	7	0.0035689055967236516	0.03379967598081336	BCAS2:EMP1:IGFBP5:RBP1:IL8:AREG:AREGB:KHDRBS3
Curated_gene_sets	OHM_METHYLATED_IN_ADULT_CANCERS	28	5	0.0035705298286589204	0.03379967598081336	SFRP5:BRCA1:RASSF1:RBP1:ESR1
Curated_gene_sets	LEE_CALORIE_RESTRICTION_NEOCORTEX_UP	67	8	0.0035821143604461347	0.03379967598081336	SELP:CIT:ZNF771:PCNA:OSBP2:NAT6:NPRL2:SLC12A2
Curated_gene_sets	ELVIDGE_HIF1A_TARGETS_UP	67	8	0.0035821143604461347	0.03379967598081336	WDR77:TOR3A:PSEN2:CORO1A:SLC7A6:MRPS12:LSG1:RPP40
Curated_gene_sets	SETLUR_PROSTATE_CANCER_TMPRSS2_ERG_FUSION_UP	67	8	0.0035821143604461347	0.03379967598081336	ALDH18A1:ABCC8:SAFB:RFX1:NCOA1:OCLN:MAP7:KHDRBS3
Curated_gene_sets	PID_TELOMERASE_PATHWAY	67	8	0.0035821143604461347	0.03379967598081336	MTOR:SMG5:RPS6KB1:NCL:ZNFX1:TERT:ESR1:TERF1

Curated_gene_sets	BOYAULT_LIVER_CANCER_SUBCLASS_G12_UP	40	6	0.0035961557782105682	0.033816158864848435	SCAMP3:YY1AP1:TOR3A:NUAK1:MFF:CREB3L2
Curated_gene_sets	VANTVEER_BREAST_CANCER_BRCA1_UP	40	6	0.0035961557782105682	0.033816158864848435	POU2AF1:REXO2:SLC11A2:DCAF4:PPP1CB:KIAA0020
Curated_gene_sets	ALFANO_MYC_TARGETS	235	18	0.003604558689906375	0.033823654275555184	NBL1:EBNA1BP2:CTSK:CSRP1:SMPD1:EMP1:KCTD12:TGFB1I1:RND2:MYL12A:PAFAH1B3:TGM2:SLPI:GNAI2:RBP1:CDX1:FLOT1:LY6E
Curated_gene_sets	ONDER_CDH1_TARGETS_2_DN	469	30	0.0036092501472006714	0.033823654275555184	TSPAN1:HOOK1:LPHN2:F3:EHF:GPRC5A:PTHLH:MAP3K9:CORO1A:ESRP2:PITPNC1:MBP:AP1M2:CEACAM1:CBLC:PI3:SLPI:CELSR1:IL8:CXCL1:AREG:AREGB:EDN1:IER3:POPDC3:MAP7:TNFAIP3:CLDN4:LYN:CA2:PTPN3
Curated_gene_sets	MOREAUX_MULTIPLE_MYELOMA_BY_TACI_UP	369	25	0.0036266534811245353	0.033928946938207596	ZNF362:ASH1L:LMNA:HSPA6:FMOD:DUSP8:APAF1:NUAK1:DAO:KCNK10:CALB2:TRPV1:ADAM11:SSTR2:MBP:POU2F2:CEACAM1:SYNGR1:MAPK8IP2:CXCL1:TIFAB:KCNIP1:PI16:CYP11B1:TOR4A
Curated_gene_sets	MULLIGHAN_NPM1_SIGNATURE_3_UP	330	23	0.0036419730812633465	0.03401442091685852	C1orf63:SSX2IP:ARHGEF11:ASRGL1:METTL7A:SDS:PRKAB1:LHFP:SIX6:RBM25:NIPA2:POLR2C:SPG7:SH3GL1:ALK:MCHR1:GTSE1:NIPBL:C5orf30:PCDHGC3:CNR1:HOXA10:PBX3
Curated_gene_sets	BOQUEST_STEM_CELL_DN	217	17	0.0036581064247497496	0.03410719227550572	TIE1:SELP:CREM:PITPNC1:CD320:CARD8:IL18R1:PKP4:TGM2:MYRIP:AREG:AREGB:CFI:KLHL2:MCTP1:ARAP3:GNG11:GIMAP5
Curated_gene_sets	MANALO_HYPOXIA_DN	292	21	0.0037110164049495367	0.03454196487923418	UBIAD1:EBNA1BP2:STIL:WDR77:TOR3A:MCM10:AASDHPPT:EMG1:SLC11A2:SLC25A15:PRMT7:BRCA1:CHAF1A:MRPS12:GRWD1:TTLL4:COQ2:RPP40:RFC2:KIAA0020:TUBB4B
Curated_gene_sets	PURBEY_TARGETS_OF_CTBP1_AND_SATB1_DN	164	14	0.003737067561874981	0.034716533312876385	MSTO1:ALDH18A1:MYO5A:MAPK7:RTKN:HYAL3:HYAL2:GP9:SRPRB:FGF12:C5orf22:WDR46:HSPB1:PILRB
Curated_gene_sets	KOKKINAKIS_METHIONINE_DEPRIVATION_48HR_UP	130	12	0.0037423930657218135	0.034716533312876385	FAS:WEE1:APAF1:MAP3K9:BRCA1:IGFBP2:IL8:DCTD:PDGFRB:CDKN1A:GTF2H5:HSPB1
Curated_gene_sets	SASSON_FSH_RESPONSE	9	3	0.003762982082161175	0.03484876167334785	FDX1:NPY1R:STAR
Curated_gene_sets	REACTOME_CELLULAR_RESPONSES_TO_EXTERNAL_STIMULI	532	33	0.0037720292144714823	0.03487383648539096	MTOR:PHC2:LAMTOR5:ARNT:HSPA6:CSRP1:TUBB8:CUL2:HSPA8:PRKAB1:ULK1:PSME1:ERO1L:MAPK7:PSMD12:TNRC6C:TUBB4A:DNAJB1:DEDD2:ERF:DCTN1:ANAPC1:CHMP4B:GSK3B:IL8:SNCB:CDKN1A:SOD2:HSPB1:RB1CC1:TERF1:GML:TUBB4B
Curated_gene_sets	SANSOM_APC_TARGETS_REQUIRE_MYC	218	17	0.0038322232875210274	0.03537090655143149	DDAH1:PHGDH:PMF1:PTHLH:POLE:BIVM:AQR:MAPK7:RNF157:GIPC1:GRWD1:BCL11A:WDR54:HES6:GTSE1:DENND6B:NTMT1
Curated_gene_sets	TAKAO_RESPONSE_TO_UVB_RADIATION_DN	98	10	0.0038416140185223505	0.03539818880383827	EXOSC10:KCNC4:SETDB1:PSEN2:PHB2:DNAJB1:PUM2:LSS:TNFAIP3:KIAA0020
Curated_gene_sets	FISCHER_G1_S_CELL_CYCLE	200	16	0.0038560849800682692	0.03547211283504272	ICMT:MCM10:SMPD1:OPCML:POLE:UBR7:BRCA1:CHAF1A:INSR:PEX13:PANK2:PCNA:PLSCR4:LIPH:ANKRA2:RFC2
Curated_gene_sets	NABA_CORE_MATRISOME	274	20	0.003863270938592679	0.035478887200664985	VWA5B1:CDCP2:BGLAP:FMOD:NID1:DMBT1:POSTN:COCH:PAPLN:ACAN:HAPLN3:MFAP4:PODNL1:IGFBP2:IGFBP5:SNED1:FBLN1:ZPLD1:THBS2:COL22A1
Curated_gene_sets	AMIT_EGF_RESPONSE_120_HELA	68	8	0.003928606223261378	0.03601877139026807	BCL10:F3:IL8:CXCL1:AREG:AREGB:CDKN1A:LAT2:GEM
Curated_gene_sets	PASQUALUCCI_LYMPHOMA_BY_GC_STAGE_DN	165	14	0.003945849108565313	0.036116665467916455	PHC2:FCRLA:NUCKS1:TNFRSF19:LCP1:RFX7:CORO1A:ARHGEF18:CD79A:PUM2:BCL11A:AFF3:USP25:DIRAS2
Curated_gene_sets	ZWANG_CLASS_2_TRANSIENTLY_INDUCED_BY_EGF	54	7	0.003969803445773517	0.036128359477699906	SPOCD1:F3:HSPA8:PTHLH:HOXC13:OSR1:DZIP1L

Curated_gene_sets	WESTON_VEGFA_TARGETS_6HR	54	7	0.003969803445773517	0.036128359477699906	C1S:PRKAB1:POSTN:ISL1:ESR1:MEST:GEM
Curated_gene_sets	LEE_TARGETS_OF_PTCH1_AND_SUFU_UP	54	7	0.003969803445773517	0.036128359477699906	CTSS:POLE:SSTR2:MYCN:MTHFD2:RND3:C4A
Curated_gene_sets	SANA_TNF_SIGNALING_UP	83	9	0.003979964705232893	0.036128359477699906	C1S:SPAG9:APOL1:IL8:CXCL6:HLA-A:TAPBP:TNFAIP3:SOD2
Curated_gene_sets	GHANDHI_BYSTANDER_IRRADIATION_UP	83	9	0.003979964705232893	0.036128359477699906	DNAJB4:LRRC8B:POU2F2:IL8:CXCL1:MCTP1:POPDC3:TNFAIP3:SOD2
Curated_gene_sets	NADERI_BREAST_CANCER_PROGNOSIS_DN	18	4	0.003997920204849549	0.03623156350391658	SSR2:REXO2:C1S:FBLN1
Curated_gene_sets	DURAND_STROMA_S_UP	294	21	0.0040136387748893184	0.03631418898135878	LPHN2:PEAR1:SELP:SLC43A1:UBE2L6:MOGAT2:UBASH3B:SOX5:PDE1B:POSTN:IL1RL1:IL18RAP:FBLN1:SLC4A4:PPAP2A:PDGFRB:POPDC3:MEST:RB1CC1:NRBP2:PAPPA
Curated_gene_sets	REACTOME_DUAL_INCISION_IN_GG_NER	41	6	0.004080805317365206	0.03674060564783306	POLE:ERCC5:PCNA:GTF2H5:RFC2:XPA
Curated_gene_sets	KAMMINGA_EZH2_TARGETS	41	6	0.004080805317365206	0.03674060564783306	SETDB1:CUL2:CDCA5:IPO5:XPO1:PCNA
Curated_gene_sets	XU_CREBBP_TARGETS_DN	41	6	0.004080805317365206	0.03674060564783306	ARHGEF2:ANP32A:CD97:PER2:KRTAP8-1:DIAPH1
Curated_gene_sets	LAIHO_COLORECTAL_CANCER_SERRATED_UP	115	11	0.004113949295625721	0.03697848868502793	MACF1:SNRPE:HSPA8:C1S:EMP1:POSTN:ARPP19:MYL12B:PLEKHB2:AP2M1:BCAP29
Curated_gene_sets	WINZEN_DEGRADED_VIA_KHSRP	99	10	0.004132547715908382	0.03702466609969383	F3:PTHLH:SLC4A4:IL8:PTGER4:ANKRA2:CPEB4:EDN1:GEM:PAPPA
Curated_gene_sets	REACTOME_COPI_DEPENDENT_GOLGI_TO_ER_RETROGRADE_TRAFFIC	99	10	0.004132547715908382	0.03702466609969383	TUBB8:KIF19:NAPG:TUBB4A:KDELR1:ARFGAP3:COPB2:KIF2A:COPG2:TUBB4B
Curated_gene_sets	CHYLA_CBFA2T3_TARGETS_UP	373	25	0.0041611642340474465	0.03717058147285868	WDR65:ARHGEF2:FCGR2A:MOGAT2:UBASH3B:GPRC5A:EMP1:RASSF8:LGR5:LCP1:ARL11:SLC7A6:CLEC10A:VAT1:SSTR2:TTC39C:CD33:DSTN:SLPI:BPIFC:FNDC3B:MCTP1:TIFAB:PRSS16:RAB44
Curated_gene_sets	REACTOME_MYOGENESIS	29	5	0.0041826195113069484	0.03717058147285868	CDC42:CDON:TCF12:SPAG9:TCF3
Curated_gene_sets	MARSON_FOXP3_TARGETS_STIMULATED_UP	29	5	0.0041826195113069484	0.03717058147285868	POU2AF1:CRTAM:TNFRSF19:MBP:PTGER4
Curated_gene_sets	AIYAR_COBRA1_TARGETS_DN	29	5	0.0041826195113069484	0.03717058147285868	METTL7A:PSMD12:CLDN4:HSPB1:NELFB
Curated_gene_sets	CHUANG_OXIDATIVE_STRESS_RESPONSE_UP	29	5	0.0041826195113069484	0.03717058147285868	FAS:MIA2:CTAGE5:PSMD12:RPL38:EDN1
Curated_gene_sets	KEGG_TIGHT_JUNCTION	132	12	0.004234277361604004	0.037495428984213715	CDC42:NRAS:ASH1L:MYLPF:MYL12A:MYL12B:GNAI2:MRAS:OCLN:CLDN3:CLDN4:EXOC4
Curated_gene_sets	TONKS_TARGETS_OF_RUNX1_RUNX1T1_FUSION_HSC_UP	184	15	0.004238683538702347	0.037495428984213715	C1orf63:NID1:CREM:ACTA2:EMP1:COCH:TGFB1I1:DGKE:CEACAM1:IL1RL1:TGFBR2:IL8:HSPB1:CA2:SLC35D2
Curated_gene_sets	THUM_SYSTOLIC_HEART_FAILURE_DN	202	16	0.004246079588125626	0.037495428984213715	CCDC28B:TM2D1:B3GALNT2:RAB18:LMO7:SPAG9:BCL11A:PER2:CPEB4:CPNE5:KLHL31:AIG1:SYNE1:FGFR1OP:CD36:RPL7

Curated_gene_sets	CREIGHTON_ENDOCRINE_THERAPY_RESISTANCE_2	454	29	0.004246437421771523	0.037495428984213715	TXLNA:WDR65:STIL:DNAJB13:TTC12:DDX25:LRRC23:COCH:FAM179B:C14orf142:HERC2:KIAA1841:CCDC74A:HES6:ZNFX1:DENND6B:EFHB:RASSF1:ZMYND10:IFT57:NEK11:MFI2:CCNO:AIG1:FGFR1OP:JAZF1:CEP41:NACC2:TUBB4B
Curated_gene_sets	PID_MTOR_4PATHWAY	69	8	0.004300388579868542	0.03791095765682187	MTOR:NRAS:ULK1:RRN3:RPS6KB1:EIF4E:YWHAG:RB1CC1
Curated_gene_sets	WONG_EMBRYONIC_STEM_CELL_CORE	335	23	0.004368895132314468	0.03845326739657902	RPL22:EBNA1BP2:CDC20:WDR77:CKS1B:FDPS:DAP3:WEE1:CDCA5:YAP1:NDUFA9:DLGAP5:UTP18:CHAF1A:XPO1:MTHFD2:NCL:PCNA:EIF6:GTSE1:RAD18:CKS1B:RPP40:TERF1
Curated_gene_sets	IWANAGA_CARCINOGENESIS_BY_KRAS_UP	167	14	0.004391858212597177	0.03851496244687487	CKS1B:SOAT1:PC:G2E3:NIPA1:DERL2:FAM83G:PAFAH1B3:TGFBR2:ACKR4:CXCL6:CKS1B:PEX7:FAM3C:PUF60
Curated_gene_sets	REACTOME_REGULATION_OF_CHOLESTEROL_BIOSYNTHESIS_BY_SREBP_SREBF	55	7	0.004403910448842809	0.03851496244687487	FDPS:GPAM:TM7SF2:KPNB1:NCOA1:LSS:PPARA
Curated_gene_sets	MADAN_DPPA4_TARGETS	55	7	0.004403910448842809	0.03851496244687487	HOOK1:HORMAD1:PRPF39:CALB2:DAZL:PLEKHG1:ABCB1
Curated_gene_sets	BROWNE_HCMV_INFECTION_10HR_DN	55	7	0.004403910448842809	0.03851496244687487	EMP1:MGA:ANP32A:RBMS1:NIPBL:HOXA11:BLK
Curated_gene_sets	WANG_LMO4_TARGETS_UP	355	24	0.004423031897812107	0.038620791222006984	SOAT1:NUCKS1:WEE1:PHB2:GTF2A1:NPIPA1:RALBP1:TCF3:RFX1:BCAM:PLGLB1:CREB1:RPE:IGFBP2:CTDSP1:DSTN:SLC2A4RG:C21orf91:VGLL4:FOXP1:C5orf30:CPEB4:TUBB:CREB3L2
Curated_gene_sets	WESTON_VEGFA_TARGETS	100	10	0.0044405927186670375	0.038712679152753365	TIE1:C1S:PRKAB1:POSTN:CEACAM1:CXCL1:ISL1:ESR1:MEST:GEM
Curated_gene_sets	LEE_LIVER_CANCER_SURVIVAL_UP	185	15	0.004456605807100914	0.03879080465959197	ANXA9:PKLR:APCS:CFHR5:F13B:ADCK3:PC:PHLPP1:INSR:CYP2D6:CAND2:MYRIP:HYAL1:STK32B:PPAP2A
Curated_gene_sets	SMID_BREAST_CANCER_ERBB2_UP	150	13	0.004487171918932963	0.038995154385545384	TSPAN1:HMGCS2:HPX:SCGB1D2:TM7SF2:UCP2:LPCAT3:ANP32A:PRMT7:SLPI:SLC37A1:LRRC31:PIP
Curated_gene_sets	PASQUALUCCI_LYMPHOMA_BY_GC_STAGE_UP	278	20	0.004535968773447376	0.0393570413607792	EXTL2:HIPK1:SSR2:PHLDA3:GYLTL1B:YAP1:REXO2:ERO1L:NOMO1:CD2BP2:FBXO17:KDELR1:TGM2:FNDC3B:PLA2G12A:TERT:AIG1:TPST1:ABCB1:CREB3L2
Curated_gene_sets	REACTOME_ACTIVATION_OF_GENE_EXPRESSION_BY_SREBF_SREBP	42	6	0.0046120726496789895	0.039766475933987654	FDPS:GPAM:TM7SF2:NCOA1:LSS:PPARA
Curated_gene_sets	KORKOLA_TERATOMA	42	6	0.0046120726496789895	0.039766475933987654	CDC20:EHF:EMP1:DLGAP5:MFAP4:MYCN
Curated_gene_sets	PAPASPYRIDONOS_UNSTABLE_ATEROSCLEROTIC_PLAQUE_DN	42	6	0.0046120726496789895	0.039766475933987654	FMOD:GRK5:LHFP:RASL12:DSTN:HSPB1
Curated_gene_sets	JOHANSSON_BRAIN_CANCER_EARLY_VS_LATE_DN	42	6	0.0046120726496789895	0.039766475933987654	ACOT7:CLSTN1:PLEKHB2:IGFBP5:OSBP2:HDAC11
Curated_gene_sets	SMID_BREAST_CANCER_LUMINAL_B_UP	168	14	0.004629698248438317	0.0398559781919549	ANXA9:ABCC8:TTC12:RABEP1:B9D1:SSTR2:GREB1:CELSR1:HDAC11:RASSF1:CACNA2D2:STK32B:NPY1R:ESR1
Curated_gene_sets	BERTUCCI_MEDULLARY_VS_DUCTAL_BREAST_CANCER_UP	204	16	0.004667762412378576	0.04012087661014773	RNF207:CTSS:MCM10:CUL2:PSAP:STAMBPL1:PTPN6:EMG1:PHB2:C1S:IL12RB1:GRWD1:C2CD2:GTSE1:TYMP:SOD2

Curated_gene_sets	VERHAAK_AML_WITH_NPM1_MUTATED_UP	186	15	0.004683554486331635	0.04013547199704938	LMNA:FCGR2A:FCGR3A:KCNJ2:SCO2:TYMP:MCTP1:IER3:TNFAIP3:SOD2:HOXA10:CD36:PILRA:PRKAR2B:PBX3
Curated_gene_sets	ZHENG_GLIOBLASTOMA_PLASTICITY_UP	241	18	0.004685125845630699	0.04013547199704938	ACOT7:TRIM62:CDC20:F3:CKS1B:ASPM:REEP3:CIT:POLE:MBP:DDX39A:MTHFD2:RND3:RBP1:CKS1B:IER3:C4A:CD36:LYN
Curated_gene_sets	ROSS_AML_OF_FAB_M7_TYPE	70	8	0.004698644603908344	0.04013547199704938	TAL1:PRUNE:PCDH9:STXBP6:MRPS12:PROS1:ARMC8:KEL
Curated_gene_sets	FURUKAWA_DUSP6_TARGETS_PCI35_DN	70	8	0.004698644603908344	0.04013547199704938	EBNA1BP2:STIL:DLGAP5:CALB2:HPCAL1:MAP7:LAT2:KHDRBS3
Curated_gene_sets	REACTOME_OLFACTORY_SIGNALING_PATHWAY	397	26	0.004759113447244982	0.040535199103290225	OR14K1:REEP3:OR51T1:OR4C15:OR4C16:OR4P4:OR4S2:OR4C6:OR5T1:OR8H1:OR8K3:OR8K1:OR8J1:OR8U1:OR5R1:OR10P1:OR1M1:OR7G2:OR7G1:OR7G3:OR10H2:OR10H3:OR10C1:OR1L3:OR1L4:OR1L6
Curated_gene_sets	RICKMAN_TUMOR_DIFFERENTIATED_WELL_VS_POORLY_DN	377	25	0.00476017789869578	0.040535199103290225	HES2:FBLIM1:LRRC8C:TMEM79:LRFN4:PTHLH:COCH:FRMD6:ERO1L:NUMB:KCNK10:MYO5A:KIAA1199:N4BP1:PLEKHM1:NPEPPS:PSMD12:AP1M2:GIPC1:PLEKHB2:CARD6:CPEB4:CDKN1A:CD36:IPPK
Curated_gene_sets	KIM_RESPONSE_TO_TSA_AND_DECITABINE_UP	134	12	0.0047766649492990325	0.040550052293354906	TSPAN1:TM7SF2:SMPD3:RND2:TGM2:DAZL:HYAL1:NR1I2:CDKN1A:MAP7:RNASET2:KCNV2
Curated_gene_sets	KEGG_UBIQUITIN_MEDIATED_PROTEOLYSIS	134	12	0.0047766649492990325	0.040550052293354906	CDC20:CUL2:UBE2L6:SYVN1:FBXW8:HERC2:SIAH1:BRCA1:KEAP1:CBLC:ANAPC1:UBE2QL1
Curated_gene_sets	DAZARD_UV_RESPONSE_CLUSTER_G2	30	5	0.004866011575118868	0.040987590317816444	CSRP1:IL8:IER3:CLDN4:LY6E
Curated_gene_sets	PID_P38_ALPHA_BETA_PATHWAY	30	5	0.004866011575118868	0.040987590317816444	CDC42:DUSP8:TAB1:BLK:LYN
Curated_gene_sets	SHIN_B_CELL_LYMPHOMA_CLUSTER_2	30	5	0.004866011575118868	0.040987590317816444	PSEN2:POU2AF1:HOXC13:TNFSF13B:HOXA11
Curated_gene_sets	TIEN_INTESTINE_PROBIOTICS_2HR_UP	30	5	0.004866011575118868	0.040987590317816444	PTP4A2:HSPA8:ANP32A:KDELR1:ST6GALNAC4
Curated_gene_sets	GRADE_COLON_VS_RECTAL_CANCER_DN	56	7	0.004872911119405918	0.040987590317816444	LINC01059:GRAMD1B:PTHLH:ULK1:TRPM1:ANP32A:TCF3:FGF12
Curated_gene_sets	MORI_LARGE_PRE_BII_LYMPHOCYTE_DN	56	7	0.004872911119405918	0.040987590317816444	MACF1:CTSS:SOAT1:PTPN6:LCP1:RGL2:LAT2
Curated_gene_sets	BIOCARTA_IGF1MTOR_PATHWAY	19	4	0.004914882551091455	0.04112172033790459	MTOR:RPS6KB1:GSK3B:EIF4E
Curated_gene_sets	SHIN_B_CELL_LYMPHOMA_CLUSTER_9	19	4	0.004914882551091455	0.04112172033790459	TAL1:IKZF3:CD79A:BCL11A
Curated_gene_sets	KOBAYASHI_RESPONSE_TO_ROMIDEPSIN	19	4	0.004914882551091455	0.04112172033790459	SYT11:MERTK:IL8:RB1CC1
Curated_gene_sets	MULLIGHAN_NPM1_MUTATED_SIGNATURE_1_UP	261	19	0.004918758767922417	0.04112172033790459	SSX2IP:ASRGL1:METTL7A:SDS:LHFP:SIX6:RBM25:POLR2C:SPG7:SH3GL1:ALK:MCHR1:GTSE1:NIPBL:C5orf30:PCDHGC3:CNR1:HOXA10:PBX3
Curated_gene_sets	MORI_MATURE_B_LYMPHOCYTE_UP	86	9	0.00504093288115946	0.04201002773885544	MACF1:CTSS:CERS2:BGLAP:ATN1:PTPN6:LCP1:LAT2:FAM102A
Curated_gene_sets	ALCALA_APOPTOSIS	86	9	0.00504093288115946	0.04201002773885544	ELOVL1:SCAMP3:RIT1:FAS:HSPA8:SLC25A3:XPO1:MFF:HLA-A

Curated_gene_sets	BENPORATH_PRC2_TARGETS	648	38	0.005047923711213133	0.04201002773885544	HES2:RIMKLA:TAL1:FOXD2:DDAH1:KCNC4:LRRC71:HSPA6:CDH23:SFRP5:ABCC8:KCNA1:PTHLH:HOXC12:HOXC11:LGR5:POLE:STXBP6:FOXG1:LRFN5:SIX6:KIAA1199:HNF1B:IKZF3:TBX21:SSTR2:CHST8:OSR1:KCNAB1:STK32B:SLC10A4:NPY1R:PTGER4:ISL1:GIMAP5:ANKRD18B:KLF4:PAPPA
Curated_gene_sets	MIKKELSEN_IPS_WITH_HCP_H3K27ME3	102	10	0.005110747280173332	0.04239138402334573	DBX1:OSR1:KCNS1:ZMYND10:FGF12:NPY5R:KCNIP1:HOXA11:CALB1:PAPPA
Curated_gene_sets	BILD_MYC_ONCOGENIC_SIGNATURE	206	16	0.005122990027200795	0.04239138402334573	UBIAD1:HSPA6:ABL2:NCR3LG1:PC:GPRC5A:EMP1:DCAF4:TAF4B:ANAPC1:NCL:TGM2:OSBP2:LSG1:FCHSD1:RPP40
Curated_gene_sets	REACTOME_TRANSLOCATION_OF_SLC2A4_GLUT4_TO_THE_PLASMA_MEMBRANE	71	8	0.005124571964283751	0.04239138402334573	TUBB8:PRKAB1:MYO5A:TUBB4A:RALGAPA2:YWHAG:EXOC4:TUBB4B
Curated_gene_sets	BASSO_CD40_SIGNALING_DN	71	8	0.005124571964283751	0.04239138402334573	PTP4A2:POU2AF1:METTL7A:TCF3:ARHGEF18:CNR1:PRKAR2B:CDH17
Curated_gene_sets	OSAWA_TNF_TARGETS	10	3	0.005226106883109447	0.04316638733331091	HABP2:IL8:IER3
Curated_gene_sets	DAZARD_RESPONSE_TO_UV_NHEK_DN	301	21	0.005239094435695215	0.04320878334446683	SPEN:PHC2:LPHN2:FLG:WEE1:MTMR6:FNDC3A:FAM179B:N4BP1:HELZ:PHLPP1:KEAP1:PUM2:XPO1:ZNF638:PER2:KIF2A:MDC1:CREB3L2:XPA:PTPN3
Curated_gene_sets	MIKKELSEN_MEF_HCP_WITH_H3_UNMETHYLATED	225	17	0.005251725567552426	0.043248117286086664	OLFM3:TNFAIP8L2:RBP3:C10orf54:KCNJ11:PTPN6:TNFSF13B:TRPM1:RCVRN:SSTR2:FSCN2:MYCN:WBP2NL:DROSHA:C5orf47:KCNV2:ADAMTSL1
Curated_gene_sets	MARTORIATI_MDM4_TARGETS_FETAL_LIVER_DN	503	31	0.005350364994865034	0.04399455580979455	TMEM57:NRAS:ARNT:UBQLN4:NUCKS1:ZMYND11:PSAP:ZNF143:FBXW8:POSTN:LCP1:KPNA3:RBM25:SEPT1:SEPT1:NFATC3:METTL16:TBKBP1:RPS6KB1:PPP4R1:SAFB2:BCL11A:FAM168B:NCL:PLXNB2:DHX29:UTP15:YWHAG:PRKAR2B:CA2:S1PR3:PBX3
Curated_gene_sets	BILD_SRC_ONCOGENIC_SIGNATURE	57	7	0.005378504134975533	0.04409411512146111	EXTL2:TRIM33:ASH1L:PTHLH:LSS:SLC12A2:TNFAIP3
Curated_gene_sets	REACTOME_INTERLEUKIN_12_FAMILY_SIGNALING	57	7	0.005378504134975533	0.04409411512146111	CDC42:RAP1B:LCP1:BOLA2B:IL12RB1:GSTA2:SOD2
Curated_gene_sets	MEISSNER_NPC_HCP_WITH_H3K4ME2_AND_H3K27ME3	341	23	0.005397028986256692	0.04418014353184235	FOXE3:KCNC4:DBX1:KCNA1:ACAN:STAC2:IKZF3:TBX21:ALK:MMP24:CELSR1:SCN5A:MYRIP:CACNA2D2:ISL1:MCIDAS:OCLN:CPNE5:DACT2:HOXA13:EVX1:FOXB2:PAPPA
Curated_gene_sets	WIERENGA_STAT5A_TARGETS_DN	189	15	0.005421274570147742	0.044312676687047144	MPL:ACOT11:DENND2C:SELP:UBASH3B:CORO1A:SLC37A1:PROS1:GP9:TMEM44:PF4V1:SLC12A2:ARAP3:SLC22A23:GNG11
Curated_gene_sets	REACTOME_INTRACELLULAR_SIGNALING_BY_SECOND_MESSENGERS	302	21	0.005437019160944241	0.04432908496952656	MTOR:PHC2:LAMTOR5:ATN1:PDE1B:PSME1:PSMD12:TNRC6C:PHLPP1:INSR:GSK3A:IL1RL1:CREB1:GSK3B:AREG:AREGB:HDAC3:PDGFRB:IER3:CDKN1A:ESR1:PRKAR2B
Curated_gene_sets	LI_DCP2_BOUND_MRNA	87	9	0.00543939871922022	0.04432908496952656	SPEN:PHGDH:TRAPPC2L:CHAF1A:UBXN6:MRPS12:DGCR6:SCO2:RFC2
Curated_gene_sets	BROWNE_HCMV_INFECTION_8HR_UP	103	10	0.005474246112690997	0.044547082641883395	C1orf63:DDAH1:KIAA0907:TLL2:DUSP8:DNAJB1:ERF:IGFBP2:PCDHGA12:SOD2
Curated_gene_sets	MARKEY_RB1_ACUTE_LOF_DN	226	17	0.0054856282811564915	0.04457376835249906	ARNT:TOR3A:UBE2L6:PTPN6:NCKAP1L:LCP1:FNDC3A:ERO1L:IFI35:IGFBP5:HES6:ZNFX1:DNAJC5:PLAC8:TAPBP:SOD2:LAT2
Curated_gene_sets	HUANG_GATA2_TARGETS_DN	72	8	0.005579381043071507	0.045268694864212915	FDPS:NOMO1:SIAH1:MTHFD2:DCTD:IPO11:FAM3C:IPPK

Curated_gene_sets	RIZKI_TUMOR_INVASIVENESS_3D_UP	208	16	0.005613672379582457	0.045370856228488125	TARDBP:LAMTOR5:BCAS2:SMCP:DAO:POLE:LHFP:TCF12:RPS6KB1:EPS15L1:DCTN1:ZMYND10:FGF12:IL8:PPAP2A:CPA1
Curated_gene_sets	REACTOME_INWARDLY_RECTIFYING_KPLUS_CHANNELS	31	5	0.0056249634517049445	0.045370856228488125	KCNJ11:ABCC8:KCNJ16:KCNJ2:KCNJ14
Curated_gene_sets	VALK_AML_CLUSTER_15	31	5	0.0056249634517049445	0.045370856228488125	LINC01059:GRAMD1B:ATN1:NDFIP1:HOXA10:HSPB1
Curated_gene_sets	REACTOME_HSF1_ACTIVATION	31	5	0.0056249634517049445	0.045370856228488125	HSPA6:DNAJB1:DEDD2:HSPB1:GML
Curated_gene_sets	DELYS_THYROID_CANCER_UP	443	28	0.005637696758589968	0.04540698370278684	ACOT7:PDZK1IP1:SLC16A4:CTSS:FCGR2A:FAS:PC:UCP2:OPCML:NCKAP1L:CCL17:SH3GL1:CD97:TGM2:SLPI:PLXNB2:PROS1:IL8:AREG:AREGB:CFI:KLHL2:IER3:C4A:CDKN1A:THBS2:LYN:LY6E:PBX3
Curated_gene_sets	FLORIO_NEOCORTEX_BASAL_RADIAL_GLIA_DN	190	15	0.005687063517853365	0.04573762633291135	CDC20:PHGDH:CKS1B:ASPM:NID1:WEE1:CDCA5:EMP1:DLGAP5:GRIN2A:MFAP4:BRCA1:GTSE1:CKS1B:NCAPG2:GEM
Curated_gene_sets	KEGG_NUCLEOTIDE_EXCISION_REPAIR	44	6	0.005824718123852771	0.046708125946522	POLE:ERCC5:PCNA:GTF2H5:RFC2:XPA
Curated_gene_sets	REACTOME_PHASE_0_RAPID_DEPOLARISATION	44	6	0.005824718123852771	0.046708125946522	CACNB1:CACNG1:SCN3A:SCN5A:CACNA2D2:FGF12
Curated_gene_sets	MATSUDA_NATURAL_KILLER_DIFFERENTIATION	465	29	0.00588531749292682	0.047125373404061775	EXOSC10:FBLIM1:EBNA1BP2:DPH5:PHGDH:CSRP1:HABP2:CDCA5:TPI1:POSTN:KCTD12:GTF2A1:MYO5A:CORO1A:CYB5B:TBX21:GRWD1:MYCN:WDR54:MERTK:NCL:TAB1:PFDN6:HOXA13:CD36:ABCB1:PPP3CC:GEM:KLF4
Curated_gene_sets	LIM_MAMMARY_LUMINAL_PROGENITOR_UP	58	7	0.005922398855900021	0.047284638760966634	PDZK1IP1:BBOX1:HAPLN3:PLB1:ATP6V1B1:MFI2:SLC34A2
Curated_gene_sets	LIANG_SILENCED_BY_METHYLATION_2	58	7	0.005922398855900021	0.047284638760966634	C1S:PI3:SLPI:CXCL6:AREG:AREGB:TNFAIP3:SOD2
Curated_gene_sets	MOROSETTI_FACIOSCAPULOHUMERAL_MUSCULAR_DISTROPHY_UP	20	4	0.005962913179869605	0.04747031172570578	LPHN2:NID1:POSTN:THBS2
Curated_gene_sets	MASRI_RESISTANCE_TO_TAMOXIFEN_AND_AROMATASE_INHIBITORS_UP	20	4	0.005962913179869605	0.04747031172570578	METTL7A:GREB1:AREG:AREGB:NPY1R
Curated_gene_sets	SENGUPTA_NASOPHARYNGEAL_CARCINOMA_WITH_LMP1_DN	173	14	0.005979920837860631	0.04748845446957802	PDZK1IP1:GRIN2A:SERPINB4:CXCL17:GREB1:PI3:EFHB:PROS1:SLC4A4:CXCL6:NPY1R:GSTA1:ABCB1:RDH10
Curated_gene_sets	MITSIADES_RESPONSE_TO_APLIDIN_UP	445	28	0.005982457543613447	0.04748845446957802	TRIM33:BCAS2:AMPD1:CTSS:YY1AP1:TOR3A:CREM:MRPL49:PTHLH:MTMR6:TGDS:PLEKHM1:PDE4A:CCDC130:PEX13:DUSP11:RBMS1:DDX27:CPT1B:IFT57:MAP3K13:AREG:AREGB:ANKRA2:BET1:GEM:ZNF34:TSTD2:KLF4
Curated_gene_sets	HOSHIDA_LIVER_CANCER_SUBCLASS_S3	266	19	0.006003922774379993	0.04759017173179301	HMGCS2:PKLR:APCS:F5:SOAT1:C1S:DAO:SDS:PCK2:ASGR2:INSR:SLPI:ARSA:PROS1:C4A:GSTA2:SOD2:ASL:CA2
Curated_gene_sets	ADDYA_ERYTHROID_DIFFERENTIATION_BY_HEMIN	73	8	0.006064293114465191	0.04799953442111225	DNAJB4:FCGR2A:CREM:UCP2:MOAP1:NCOA1:IL8:IER3
Curated_gene_sets	REACTOME_CARGO_RECOGNITION_FOR_CLAT	105	10	0.006261625523432473	0.04941922812683219	LDLRAP1:SYT11:SH3GL3:EPN2:SH3GL1:EPS15L1:COPS8:AP2M1:AREG:AREGB:UBQLN1

	HRIN_MEDIATED_ENDOCYTOSIS					
Curated_gene_sets	JAZAG_TGFB1_SIGNALING_VIA_SMAD4_UP	105	10	0.006261625523432473	0.04941922812683219	UBIAD1:PDZK1IP1:ERCC5:MYLPF:IFI35:OR10H2:NPRL2:FGF12:CDH6:PILRA
Curated_gene_sets	RAO_BOUND_BY_SALL4_ISOFORM_A	174	14	0.0062844560628107955	0.04945750043136221	DNAJB4:LMNA:OR4C15:OR8K3:ULK1:ERCC5:PSEN1:RFX7:CIAPIN1:OR7G1:MFF:STX19:NIPBL:SEPT7
Curated_gene_sets	KEGG_HUNTINGTONS_DISEASE	174	14	0.0062844560628107955	0.04945750043136221	NDUFA9:APAF1:POLR2C:DNAI2:TAF4B:DCTN1:CREB1:TGM2:NDUFA6:IFT57:NDUFB4:AP2M1:SOD2:CREB3L2
Curated_gene_sets	LIU_VAV3_PROSTATE_CARCINOGENESIS_UP	89	9	0.006308993814432157	0.04957967853313042	CTSS:CTSK:NID1:TNFSF13B:MBP:TGM2:FBLN1:TGFBR2:CD36
Curated_gene_sets	REACTOME_DISEASES_OF_SIGNAL_TRANSDUCTION	386	25	0.00637440322424692	0.0499019664761155	MTOR:NRAS:LMNA:PSEN2:SYVN1:RAP1B:PSME1:PSEN1:POLR2C:DERL2:PSMD12:GSK3A:CREB1:HDAC11:TGFBR2:TRAK1:GSK3B:RBP1:RASA2:AREG:AREGB:HDAC3:PDGFRB:CDKN1A:ESR1:FGFR1OP
Curated_gene_sets	MIKKELSEN_ES_LCP_WITH_H3K4ME3	139	12	0.006377219129741718	0.0499019664761155	C1orf210:ACOT11:PKLR:C1orf85:ARHGEF11:F5:SULT1A3:MYLPF:SEPT1:SEPT1:PIRT:NYAP1:GIMAP5
Curated_gene_sets	GARGALOVIC_RESPONSE_TO_OXIDIZED_PHOSPHOLIPIDS_BLUE_UP	139	12	0.006377219129741718	0.0499019664761155	TMEM57:F3:HSPA6:CREM:EMP1:KLHL28:UBALD2:DNAJB1:DEDD2:IL8:CPEB4:PPP3CC
Oncogenic_signatures	PDGF_ERK_DN.V1_DN	149	20	8.732837082873976e-07	0.00016505062086631816	BCL10:DUSP8:SAC3D1:TBKBP1:PHLPP1:PDE4A:GIPC1:DNAJB1:ERF:CIC:MYCN:NCAPH2:SCO2:TMEM115:ISL1:MGAT1:PRSS16:IRAK1BP1:LRRC6:FAM102A
Oncogenic_signatures	BRCA1_DN.V1_UP	130	17	8.077272877840025e-06	0.0007633022869558824	SLC16A4:GJA5:APBB1:SLC43A1:BTG4:DDX25:PRSS21:KCNJ16:DENND1C:DAZL:CDX1:THBS2:NEUROD6:PIP:STAR:GML:PAEP
Oncogenic_signatures	PRC1_BMI_UP.V1_UP	184	20	2.230384445721354e-05	0.001405142200804453	LRRC8B:TRPC6:FOXG1:KLHL28:TTLL4:SLPI:LIME1:ACKR2:HYAL3:FOXP1:NR1I2:LRRC31:FGF12:IL8:MCTP1:EDN1:TNFAIP3:NEUROD6:ZNF117:CLDN3
Oncogenic_signatures	IL2_UP.V1_DN	190	20	3.548096298566802e-05	0.0015578863269390829	ZNF362:GOLPH3L:FCGR2A:F13B:MOGAT2:POU2AF1:METTL7A:NUAK1:TRPM1:RASL12:CHRNB4:RPL3L:SCN3A:KCNS1:NR1I2:A4GNT:ISL1:PRSS16:FUT9:CEL
Oncogenic_signatures	PRC2_EZH2_UP.V1_UP	192	20	4.121392399309743e-05	0.0015578863269390829	PTBP2:DUSP8:KCNA1:PTHLH:PCDH9:KCTD12:RASL12:SLC14A1:IL1RL1:SLPI:KIAA1644:HYAL3:IL8:MCTP1:EDN1:B3GALT4:POPDC3:SLC35D2:KLF4:TOR4A
Oncogenic_signatures	CYCLIN_D1_KE_.V1_UP	188	19	9.53854498732237e-05	0.0025940788242272738	HTR6:DYRK4:TCF12:CHRNB4:PKD1:POLR2C:TRIM25:PKN1:FSHR:LMF2:TGFBR2:GNAI2:IFRD2:SKIV2L2:CDKN1A:EVX1:FAM3C:KLF4:PTPN3
Oncogenic_signatures	E2F1_UP.V1_UP	189	19	0.00010237014674557378	0.0025940788242272738	NBL1:STIL:SSX2IP:RBM15:LARP4B:MCM10:DMBT1:KPNA3:FANCM:DLGAP5:DCTPP1:RND2:GRWD1:BOLA3:PKP4:EIF6:MFI2:KIF2A:PBX3
Oncogenic_signatures	CYCLIN_D1_KE_.V1_DN	190	19	0.00010980227827417031	0.0025940788242272738	TXLNA:DNAJB4:F3:ARNT:SMCP:HPX:UCP2:TFCP2:NCKAP1L:DAO:FKBP3:POU2F2:PAFAH1B3:ALK:ATP6V1B1:DAZL:CCNO:ARL4A:BET1
Oncogenic_signatures	CSR_EARLY_UP.V1_UP	153	16	0.00022339348776946523	0.0040527679171816966	TXNDC12:F3:HECTD2:PRKRIR:CSRNP2:FRMD6:MESDC1:DHX33:CD33:HPCAL1:NCL:C21orf91:VGLL4:PTP4A1:YWHAG:KIAA0020
Oncogenic_signatures	TBK1.DF_UP	287	24	0.00024553695472131457	0.0040527679171816966	TMEM50A:EXTL2:GOLPH3L:RIT1:CREM:ENO2:PTHLH:CORO1A:CIAPIN1:CD97:MAST3:SLC37A1:SCO2:PROS1:IFT57:ATP10D:IL8:ISL1:ARAP3:TNFAIP3:TPST1:CLDN4:GNG11:PPP3CC
Oncogenic_signatures	STK33_SKM_UP	272	23	0.0002791296240924263	0.0040527679171816966	DNAJB4:CTSK:RIT1:ARL5B:C10orf54:ACTA2:DUSP8:KLHL28:GPR137C:TGFB1I1:KCNJ2:KIAA1841:RND3:PLXNB2:PLCXD2:FCHSD1:CPEB4:CDKN1A:ITGB8:ZNF117:CD36:GNG11:TMEM71
Oncogenic_signatures	LTE2_UP.V1_UP	188	18	0.00028260639282676696	0.0040527679171816966	HMGCS2:SOAT1:EHF:ASRGL1:UHRF1BP1L:PCDH9:NUMB:MYO5A:KIAA1199:LONP2:MBP:IGFBP5:PROS1:PTGER4:SLC12A2:EDN1:CREB3L2:PIP
Oncogenic_signatures	STK33_NOMO_UP	290	24	0.0002862809045334194	0.0040527679171816966	PTBP2:NRAS:RIT1:FCRLB:ZMYND11:ARL5B:HECTD2:EMP1:SLC11A2:NUAK1:FNDC3A:KLHL28:NPEPPS:KCNJ2:MERTK:RND3:CXCL6:CXCL1:GPRIN3:C5orf20:CPEB4:IER3:GNG11:PTPN3

Oncogenic_signatures	E2F1_UP.V1_DN	190	18	0.00032164824739537275	0.0040527679171816966	C1orf63:PDZK1IP1:F3:PSEN2:FAS:SLC11A2:PKD1:TGFB1I1:LIPE:RTKN:IL1RL1:HES6:TGM2:SLPI:CYP2D6:LRIG1:CXCL1:ASL
Oncogenic_signatures	PRC2_EZH2_UP.V1_DN	190	18	0.00032164824739537275	0.0040527679171816966	IQCC:SYT11:MCM10:PCK2:DLGAP5:LINS:CORO1A:CYB5B:METTL16:CD320:AFF3:SCN3A:KCNS1:CACNA2D2:THBS2:HOXA10:PILRB:CDH17
Oncogenic_signatures	VEGF_A_UP.V1_DN	193	18	0.0003889750474891028	0.004581253870410146	BCL10:DLEU1:UCHL3:IPO5:DLGAP5:MYO5A:TRIM25:AZI1:IL1RL1:RND3:TRMU:CYB561D2:XXcos-LUCA11.4:RASA2:DHX29:C5orf30:ZNF117:MEST:KIAA0020
Oncogenic_signatures	BMI1_DN.V1_UP	147	15	0.00045105596239613995	0.004581253870410146	TIE1:F3:KCNC4:CTSS:ACTA2:GPRC5A:PTHLH:LCP1:CD97:LRIG1:CXCL1:MARCH3:EDN1:ITGB8:PAPPA
Oncogenic_signatures	KRAS.600.LUNG.BREAST_UP.V1_DN	282	23	0.00046523516913147574	0.004581253870410146	VAV3:PAQR6:NSUN6:ARL5B-AS1:BBOX1:NAALADL1:METTL7A:SDS:SH3GL3:TBKBP1:FSCN2:IMPA2:DENND1C:MAST3:PRODH2:MEGF8:ATP6V1B1:IL18R1:IGFBP2:ZNF343:PLAC8:LECT2:CD36:CNTLN
Oncogenic_signatures	MEK_UP.V1_UP	196	18	0.00046818119991437734	0.004581253870410146	LPHN2:ENO2:LHFP:PCDH9:ERO1L:MAP3K9:NUMB:MYO5A:KIAA1199:CORO1A:TGFB1I1:BCAM:PTGER4:PPAP2A:SLC12A2:EDN1:CLDN4:KLF4
Oncogenic_signatures	P53_DN.V2_UP	148	15	0.000484788769355571	0.004581253870410146	SLC16A4:RGR:POU2AF1:NCKAP1L:RND2:OR10H3:CHST8:CYP2D6:IL8:CXCL6:PF4V1:CXCL1:IER3:TNFAIP3:EVX1
Oncogenic_signatures	STK33_UP	284	23	0.0005133340941441874	0.004620006847297687	RIT1:ZMYND11:ARL5B:C10orf54:DUSP8:EMP1:NUAK1:FNDC3A:KLHL28:GPR137C:KCNJ2:KIAA1841:RND3:PLCXD2:CXCL1:GPRIN3:CPEB4:CDKN1A:ITGB8:ZNF117:CD36:GNG11:TMEM71
Oncogenic_signatures	AKT_UP_MTOR_DN.V1_DN	184	17	0.0006203947222402901	0.005329754659246129	NID1:FAS:SFRP5:WEE1:TNFRSF19:KCTD12:ZNF771:SARS2:ERF:RTKN:MERTK:CDPF1:TYMP:IFRD2:PI16:GNG11:S1PR3
Oncogenic_signatures	JNK_DN.V1_UP	185	17	0.000659719087046929	0.005421169889211722	HTR6:MPL:ABL2:YAP1:C1S:RASSF8:HNF1B:IL12RB1:KCNS1:ARMC8:MRAS:IL8:CCNO:MAP7:SYNE1:ARL4A:ZNF79
Oncogenic_signatures	PRC1_BMI_UP.V1_DN	187	17	0.0007448072372530595	0.00563074271363313	FLG:DDX25:OPCML:ADAMTS7:CORO1A:ZNF771:RND2:OR10H3:FBXO17:LIPE:TM4SF20:NAT6:HYAL1:CACNA2D2:HCRTR2:HOXA10:PILRA
Oncogenic_signatures	ATF2_UP.V1_UP	187	17	0.0007448072372530595	0.00563074271363313	DNASE2B:HSPA6:APAF1:FOXG1:DCAF4:ANKRD11:PTGER1:NFKBIB:PAFAH1B3:LIPE:RBP1:OR10C1:TSC22D4:CEP41:DENND2A:FAM102A:GTF3C5
Oncogenic_signatures	RPS14_DN.V1_UP	189	17	0.0008391060566439802	0.005894719876692545	MPL:CTSS:F5:ACTA2:CRTAM:EMP1:KCTD12:SH3GL3:SCN3A:ACKR4:IL8:PF4V1:PTGER4:LECT2:CDKN1A:PILRA:CALB1
Oncogenic_signatures	MEL18_DN.V1_UP	140	14	0.0008421028395275063	0.005894719876692545	TIE1:F3:CTSS:PTHLH:LCP1:ERO1L:CD97:C2CD2:LRIG1:CXCL1:ADAM19:EDN1:ITGB8:PAPPA
Oncogenic_signatures	STK33_NOMO_DN	278	22	0.0009105924426227912	0.006146498987703841	SAMD13:DDAH1:SLC16A4:RGR:SLC43A1:KDELC1:BIVM:HEATR5A:PRSS21:SEPT1:SEPT1:SLC47A1:VAT1:FPR2:AFF3:IL18R1:IL18RAP:HES6:SIRPB2:CMTM8:HOXA10:ASL:STAR
Oncogenic_signatures	ESC_V6.5_UP_EARLY.V1_UP	176	16	0.00105105255271295	0.006849963188370606	GPR153:SSX2IP:FAM189B:HIGD1C:NRL:OTUD7A:MGA:ZNF771:AZI1:MAST3:HES6:PKDREJ:HDAC11:LRIG1:TERT:OCLN
Oncogenic_signatures	VEGF_A_UP.V1_UP	194	17	0.001120450052947898	0.007058835333571758	TLL2:DUSP8:UBE2L6:TM7SF2:AKAP3:ENO2:PKD1:LONP2:CACNB1:CD79A:CIC:MERTK:PER2:SLC12A2:EVX1:CPA1:LY6E
Oncogenic_signatures	BMI1_DN_MEL18_DN.V1_UP	145	14	0.0011862492522314726	0.007232293828120914	F3:WEE1:PTHLH:LCP1:CD97:C2CD2:IL8:CXCL1:MARCH3:ADAM19:EDN1:ARL4A:ITGB8:PAPPA
Oncogenic_signatures	MYC_UP.V1_UP	179	16	0.001256204352453708	0.007419456956679713	UBIAD1:HSPA6:ADCK3:SAC3D1:DGAT2:SLC25A15:DCAF4:DCTPP1:TTYH2:TAF4B:CD320:ESF1:SPATC1L:SLC25A26:UTP15:RPP40
Oncogenic_signatures	BMI1_DN_MEL18_DN.V1_DN	147	14	0.0013535374967413847	0.007752078390427931	TSPAN1:VAV3:SLC16A4:CREM:UBE2L6:METTL7A:HOXC13:KCNJ16:SLPI:SLC4A4:KLHL2:MAP7:CA2:LY6E
Oncogenic_signatures	IL15_UP.V1_DN	181	16	0.0014109527426765022	0.007843237304878204	KCNC4:GOLPH3L:FLG:SELP:RASL12:RPL3L:TGFB1I1:DNAI2:C19orf57:SCN3A:KCNS1:SLPI:RAB6B:NPY5R:PRSS16:GEM

Oncogenic_signatures	BCAT_BILD_ET_AL_DN	46	7	0.0015555116333744417	0.008399762820221986	ASH1L:PTHLH:ZNF236:ARHGEF18:LSS:ATP10D:TNFAIP3
Oncogenic_signatures	PGF_UP.V1_DN	185	16	0.001768897830236353	0.009286713608740853	CDC20:PKLR:LRRC23:PDE1B:FOXG1:PKD1:SSTR2:ERF:BCAM:IGFBP2:IGFBP5:ENTPD3:TNFAIP3:GFRA2:LY6E:FCN2
Oncogenic_signatures	BCAT_GDS748_UP	48	7	0.0020050156274261832	0.010163706453812238	DDAH1:ASRGL1:KCTD12:PLEKHB2:ABCB1:FAM3C:CA2
Oncogenic_signatures	KRAS.LUNG.BREAST_UP.V1_UP	137	13	0.0020434965356871163	0.010163706453812238	TSPAN1:NRL:DNAH9:KCNJ14:IL1RL1:OSBP2:IL8:CXCL1:TERT:ADAM19:TNFAIP3:LAT2:CALB1
Oncogenic_signatures	CRX_DN.V1_UP	138	13	0.002180067758587886	0.01056494375315668	RNF207:RPE65:RGR:RCVRN:TTC39C:KCNJ14:C5orf30:NRM:IER3:TNFAIP3:FAM3C:PPP3CC:SLC25A25
Oncogenic_signatures	KRAS.PROSTATE_UP.V1_DN	140	13	0.002475747612776712	0.011247832188105772	FOXD2:OPCML:SDS:TUBB4A:MUC16:NPHS1:KCNJ14:VIL1:PI3:PF4V1:CFI:EDN1:ABCB1
Oncogenic_signatures	PKCA_DN.V1_UP	174	15	0.0024995182640235045	0.011247832188105772	BBOX1:SMPD3:SLC47A1:PRODH2:CXCL17:PER2:CYP2D6:FBLN1:TYMP:PSAPL1:LECT2:GSTA2:CD36:CREB3L2:CA2
Oncogenic_signatures	WNT_UP.V1_UP	174	15	0.0024995182640235045	0.011247832188105772	NBL1:PKLR:PMF1:DNAJB13:BTG4:POSTN:MAPKBP1:TGFB1I1:RNF112:TCF3:GRIN2D:FPR2:KRTAP8-1:ACKR4:CXCL1
Oncogenic_signatures	KRAS.DF.V1_DN	192	16	0.0025778218647861876	0.011330426335920686	FAM63A:HPX:MOGAT2:LCP1:PTGER1:OR10H3:BCAM:SIGLEC6:IGFBP2:PER2:RAB6B:DVL3:MAP7:SYNE1:HOXA11:KLF4
Oncogenic_signatures	PRC2_EED_UP.V1_DN	193	16	0.0027151578376282213	0.011662837075266679	MCM10:PC:ENO2:CORO1A:PRMT7:MAPK7:AZI1:FBXO17:LIPE:AFF3:KCNS1:LSS:GNAI2:RASSF1:MRAS:PCDHGC3
Oncogenic_signatures	ATM_DN.V1_DN	145	13	0.0033608034350262345	0.014115374427110186	KCNC4:TLL2:SCGB2A1:OPCML:MIA2:CTAGE5:GTF2A1:PRSS21:DNAH9:TCEB3B:GRIN2D:MRAS:GPR22:FCN2
Oncogenic_signatures	AKT_UP_MTOR_DN.V1_UP	181	15	0.0036364388051035733	0.014941020307925552	BCAS2:TOR3A:DUSP8:VAT1:GIPC1:CEACAM1:MYCN:WFDC12:LSS:DAZL:RASSF1:MAN2B2:CXCL1:EDN1:RP11-203J24.9:ST6GALNAC6
Oncogenic_signatures	ESC_J1_UP_EARLY.V1_UP	182	15	0.003828859610319935	0.015396903539371655	RPL22:ANGPTL7:C1orf210:TSPAN1:ADCK3:C10orf54:TXNDC16:CEACAM1:CD33:HES6:NAGA:TYMP:CPT1B:PTGER4:PDGFRB
Oncogenic_signatures	AKT_UP.V1_DN	186	15	0.004683554486331635	0.017356701919934884	HMGCS2:NID1:FAS:RCE1:C1S:TNFRSF19:KCTD12:ZNF771:TCF3:RTKN:MERTK:CDPF1:IFRD2:PI16:S1PR3
Oncogenic_signatures	ATF2_UP.V1_DN	186	15	0.004683554486331635	0.017356701919934884	ADAM15:FMOD:PC:SLC25A15:KCTD12:ERO1L:KIAA1199:NPIPA1:MFAP4:RPS6KB1:KCNJ2:IGFBP2:PLSCR4:KEL:PIP
Oncogenic_signatures	GCNP_SHH_UP_LATE.V1_UP	186	15	0.004683554486331635	0.017356701919934884	TARDBP:SSX2IP:MCM10:CREM:LRRC23:FANCM:FRMD6:DLGAP5:SLC7A6:DDX39A:XPO1:ZNF638:MOB1A:PFDN6:NTMT1
Oncogenic_signatures	ESC_J1_UP_LATE.V1_DN	186	15	0.004683554486331635	0.017356701919934884	POLE:CORO1A:MAPK7:SLC47A1:PPP4R1:NPHS1:DDX27:DBR1:MRAS:DIMT1:TSPAN17:ZFP62:RPP40:EDN1:KLF4
Oncogenic_signatures	PRC2_EED_UP.V1_UP	188	15	0.005165613465941052	0.018775018174285747	C1orf63:PTBP2:RGR:DUSP8:ZNHIT2:P2RX2:KLHL28:RASL12:RPL3L:IL1RL1:SLPI:C2CD2:PLSCR4:MCTP1:SLC35D2
Oncogenic_signatures	HOXA9_DN.V1_DN	190	15	0.005687063517853365	0.019542818270441565	FCGR2A:HSPA6:MCM10:LPCAT3:COCH:ERO1L:NFATC3:METTL16:MFAP4:TAF4B:PHLPP1:IGFBP2:PER2:LSS:APOBEC3B
Oncogenic_signatures	ERBB2_UP.V1_UP	190	15	0.005687063517853365	0.019542818270441565	TSPAN1:ENO2:KIAA1199:BCAM:CDS2:APOBEC3B:GNAI2:IL8:PTGER4:PPAP2A:SLC12A2:EDN1:B3GALT4:CREB3L2:ZNF467
Oncogenic_signatures	CYCLIN_D1_UP.V1_DN	190	15	0.005687063517853365	0.019542818270441565	TXLNA:DNAJB4:ARNT:PSEN2:SMPD1:UCP2:TFCP2:NCKAP1L:ASGR2:PAFAH1B3:PUM2:PPARA:MAPK8IP2:DAZL:CCNO
Oncogenic_signatures	NFE2L2.V2	465	29	0.00588531749292682	0.019772931028245868	ICMT:RIMKLA:ACOT11:PHGDH:F5:MCM10:ZNF143:OR8H1:C1S:SDS:PCK2:CCL17:GLP2R:SLC47A1:OR10H3:ZNF135:MTHFD2:IL18R1:VIL1:TM4SF20:CYP8B1:NR1I2:MFI2:CFI:TERT:GSTA2:ITGB8:SPDYE3:PIP

Oncogenic_signatures	HOXA9_DN.V1_UP	191	15	0.005963264913280499	0.019772931028245868	ACTA2:FAS:UHRF1BP1L:FNDC3A:TMEM251:TYMP:IFT57:FNDC3B:IL8:PTGER4:DHX29:ANKRA2:NDFIP1:SLC35D2:KLF4
Oncogenic_signatures	TBK1.DF_DN	286	20	0.006173100332422697	0.020115792462549825	HOOK1:SIKE1:EHF:SNX15:GTF2A1:RFX7:RRN3:SMG1:TBC1D10B:ANKRD11:RPS6KB1:MBP:PAFAH1B3:IL1RL1:PKP4:ESF1:ZNF654:GTPBP8:C5orf22:NSMAF
Oncogenic_signatures	E2F3_UP.V1_DN	158	13	0.006903497299629556	0.022114593044576036	MFSD2A:HSPA6:PC:GPRC5A:NCKAP1L:NRL:KLHL28:CBLC:ZNF135:ESF1:TGM2:RAB43:NACC2
Oncogenic_signatures	SNF5_DN.V1_UP	178	14	0.007628824805777791	0.024030798138200044	FDPS:FMOD:C1S:LGR5:GTF3A:LCP1:GTF2A1:TMEM106A:ZNFX1:FBLN1:TAPBP:MEST:NCAPG2:TERF1
Oncogenic_signatures	CAMP_UP.V1_DN	199	15	0.008582982398427712	0.026593174972177665	DDAH1:FAM189B:SYT11:HSPA8:ENO2:NPEPPS:DEDD2:IGFBP5:EIF4E:UBE2QL1:SEPT7:RPL7:GEM:PAEP:TUBB4B
Oncogenic_signatures	DCA_UP.V1_DN	182	14	0.00919197660045408	0.028020702862674534	TXLNA:ASPM:CDON:NUMB:PKD1:NPIPA1:LONP2:ALK:TMEM115:ATP10D:MCTP1:SNCB:VPS52:CNTLN
Oncogenic_signatures	SIRNA_EIF4GI_UP	95	9	0.0095662153368492	0.028698646010547602	CSDE1:SETDB1:PSAP:LETMD1:SLC25A3:PSME1:KIF2A:HSPB1:CA2
Oncogenic_signatures	RELA_DN.V1_UP	147	12	0.009788212728539525	0.028905815713968285	HOOK1:F3:KCNC4:CRB1:DUSP8:ZNHIT2:KCNA1:LMO7:KCNJ16:MC3R:WRNIP1:PIP
Oncogenic_signatures	ATF2_S_UP.V1_DN	185	14	0.010521757216888201	0.03059403252295185	RHCE:FMOD:TENM4:ENO2:POLE:KCTD12:KLHL28:ERO1L:KIAA1199:NPIPA1:CCL17:IGFBP2:PLSCR4:HCRTR2
Oncogenic_signatures	NOTCH_DN.V1_UP	186	14	0.010996959764468728	0.03149129387097863	ANGPTL7:RHCE:IQCC:TGFB1I1:DGKE:OR10H3:CHST8:PKDREJ:NAT6:PF4V1:CDKN1A:GNG11:CALB1:GTF3C5
Oncogenic_signatures	LEF1_UP.V1_DN	187	14	0.011488753105382473	0.03193197554290129	TSPAN1:PDZK1IP1:NSUN6:ARL5B-AS1:EHF:LGR5:COCH:HNF1B:AP1M2:MYRIP:ENTPD3:PROS1:PLSCR4:PLAC8:CDH17
Oncogenic_signatures	CYCLIN_D1_UP.V1_UP	187	14	0.011488753105382473	0.03193197554290129	HTR6:RGR:DYRK4:PDE1B:PKD1:RCVRN:TRIM25:CACNG1:FSHR:NAT6:PCDHGA12:EVX1:KLF4:PTPN3
Oncogenic_signatures	SRC_UP.V1_DN	169	13	0.01179132072490233	0.032297965463862904	PTBP2:SOAT1:LINC00610:COMMD9:PC:C14orf142:RRN3:PRR19:ZNF135:KIAA1841:NPY5R:PTGER4:PPIL1:S1PR3
Oncogenic_signatures	CAHOY_NEURONAL	100	9	0.013136432210324834	0.03546836696787705	SYT13:ENO2:BCL11A:KIAA1239:CDH9:CPNE5:NEUROD6:GPR22:CALB1
Oncogenic_signatures	P53_DN.V1_UP	191	14	0.013629337746560433	0.03628091315633693	BCL10:F3:YAP1:KIAA1199:CAND2:LRIG1:RBP1:AREG:AREGB:CNR1:ARL4A:CLDN3:MEST:CALB1:KHDRBS3
Oncogenic_signatures	MEK_UP.V1_DN	194	14	0.015427424856982197	0.039942236958488156	PTBP2:ANXA9:UBE2L6:AQR:TCF12:CALB2:IFI35:NPEPPS:GREB1:LRIG1:IFT57:NPY1R:CETN3:PRKAR2B
Oncogenic_signatures	RAF_UP.V1_DN	194	14	0.015427424856982197	0.039942236958488156	PTBP2:VAV3:TCF12:RRN3:CALB2:GREB1:RND3:PKP4:IGFBP2:IGFBP5:LRIG1:SHQ1:IFT57:NPY1R
Oncogenic_signatures	MTOR_UP.N4.V1_UP	195	14	0.016065501338401574	0.041032158823755376	EBNA1BP2:PHGDH:RIT1:FAS:SLC43A1:OPCML:DLEU1:PCK2:MFAP4:SSTR2:HYAL3:COQ2:ARL4A:KIAA0020
Oncogenic_signatures	KRAS.600.LUNG.BREAST_UP.V1_UP	274	18	0.016312532489003752	0.04110758187228946	TSPAN1:SFRP5:NRL:GRIN2A:DNAH9:CACNG1:NPHS1:KCNJ14:IL1RL1:OSBP2:IL8:CXCL1:TERT:ADAM19:TNFAIP3:LAT2:GNG11:CALB1
Oncogenic_signatures	CRX_NRL_DN.V1_UP	140	11	0.01694413487392985	0.042137388041746604	RNF207:PRKAB1:NRL:TTC39C:KCNJ14:PPARA:NRM:IER3:TNFAIP3:MEST:SLC25A25
Oncogenic_signatures	ERBB2_UP.V1_DN	197	14	0.017401997384437824	0.042713993579983754	STIL:PTBP2:AASDHPPT:RRN3:CALB2:NPEPPS:GREB1:TRMT61B:LRIG1:IFT57:PLA2G12A:NPY1R:CETN3:PRKAR2B
Oncogenic_signatures	ESC_J1_UP_EARLY.V1_DN	179	13	0.018238823484086977	0.0441940722883646	KTI12:UHRF1BP1L:FBXO33:ZNF48:MAPK7:ERF:ZBP1:HYAL2:DBR1:MRAS:DIMT1:EDN1:KLF4

Oncogenic_signatures	CSR_EARLY_UP.V1_DN	126	10	0.020819566816131424	0.04944886787405056	FAM63A:WEE1:ZFYVE1:PAPLN:MPPE1:CC2D1A:GTPBP8:NPHP3:HOXA10:ZNF467
Oncogenic_signatures	PDGF_UP.V1_UP	145	11	0.021403568644137165	0.04944886787405056	EXOSC10:ACOT11:NRAS:FDX1:NCKAP1L:MOB1A:AREG:AREGB:WDR46:CDKN1A:GNG11:FAM3C
Oncogenic_signatures	MTOR_UP.V1_DN	183	13	0.02145400616757749	0.04944886787405056	ADCK3:B3GALNT2:RCE1:HIGD1C:TCF3:MYCN:HYAL3:RBP1:CXCL1:PTGER4:ISL1:PI16:S1PR3
Oncogenic_signatures	JAK2_DN.V1_UP	183	13	0.02145400616757749	0.04944886787405056	FOXE3:RBP3:ACTA2:SMIM2:ASGR2:SERPINB4:ZNF236:AFF3:TYMP:LRIG1:NEK11:SNCB:LRRC6
Computational_gene_sets	MODULE_136	489	47	5.334983067664287e-09	4.577415472055958e-06	TAL1:F3:DPH5:GJA5:SELP:ADCK3:CDH23:STAMBPL1:TRUB1:ZNF143:SLC43A1:RBM7:SLC11A2:LMO7:KDELC1:NRL:COCH:PRPF39:MAPKBP1:RFX7:SIAH1:SPAG9:PITPNC1:MYL12A:EPS15L1:FPR2:GREB1:PPP1CB:ZNF638:IL1RL1:IL18R1:TYMP:HYAL2:C3orf17:IL8:MTTP:C5orf20:WRNIP1:EDN1:WDR46:PLEKHG1:ZNF117:RFC2:ZMAT4:DIRAS2:NELFB:TOR4A
Computational_gene_sets	MODULE_69	524	48	1.6942680599067022e-08	7.2684099769997525e-06	TAL1:F3:GJA5:SELP:ABL2:ADCK3:STAMBPL1:ZNF143:SLC43A1:RBM7:SLC11A2:LMO7:KDELC1:NRL:COCH:PRPF39:MAPKBP1:RFX7:SIAH1:SPAG9:PITPNC1:TTYH2:MYL12A:CHAF1A:EPS15L1:FPR2:GREB1:PPP1CB:ZNF638:IL1RL1:IL18R1:TYMP:HYAL2:C3orf17:IL8:MTTP:NPY1R:C5orf20:WRNIP1:EDN1:WDR46:PLEKHG1:ZNF117:RFC2:ZMAT4:DIRAS2:NELFB:TOR4A
Computational_gene_sets	MODULE_37	469	42	2.4208415201244025e-07	6.923606747555791e-05	TAL1:GJA5:ABL2:ADCK3:STAMBPL1:ZNF143:SLC43A1:RBM7:SLC11A2:LMO7:KDELC1:NRL:COCH:MAPKBP1:SIAH1:SPAG9:PITPNC1:MYL12A:CHAF1A:EPS15L1:FPR2:GREB1:PPP1CB:ZNF638:IL1RL1:IL18R1:HYAL2:C3orf17:IL8:MTTP:NPY1R:C5orf20:WRNIP1:EDN1:WDR46:PLEKHG1:ZNF117:RFC2:ZMAT4:DIRAS2:NELFB:TOR4A
Computational_gene_sets	MODULE_88	829	61	6.021865298890539e-07	0.00012916901066120206	CDC20:TSPAN1:PDZK1IP1:SLC16A4:HMGCS2:SEMA6C:HCN3:APCS:HSPA6:LRIT1:DMBT1:APBB1:BBOX1:SLC43A1:UBE2L6:TM7SF2:C1S:LGR5:DAO:SDS:PCK2:SIX6:CHRNB4:CORO1A:ASGR2:RCVRN:HNF1B:SSTR2:NFKBIB:CD79A:BCAM:CD33:FPR2:IGFBP2:TGM2:SLPI:APOL1:ARFGAP3:GTSE1:SCO2:HYAL1:RBP1:SLC4A4:CFI:OCLN:LECT2:ADAM19:PRSS16:IER3:CDKN1A:GSTA2:ESR1:ARL4A:CLDN3:CLDN4:PIP:CA2:ST6GALNAC4:CEL:FCN2:PAEP
Computational_gene_sets	MODULE_163	562	45	2.0216533679757273e-06	0.0003469157179446348	ICMT:TXNDC12:HOOK1:RBM15:LAMTOR5:SEMA6C:CRB1:TLL2:KCNJ11:BBOX1:DAO:RFX7:RASL12:ADAMTS7:SIAH1:METTL16:TAF4B:S1PR5:SLC6A16:FPR2:HPCAL1:MYCN:KIAA1841:RBMS1:VIL1:ZNF343:SEMG1:TYMP:C3orf55:STK32B:ATP10D:C4orf17:TERT:CDX1:WDR46:HCRTR2:INMT:BCAP29:CPA1:GIMAP5:STAR:ADAMTSL1:SPATA31A4:SPATA31A7:ZNF79:LRSAM1
Computational_gene_sets	MODULE_55	826	58	4.808033745922994e-06	0.0006875488256669882	ICMT:CDC20:PDZK1IP1:SLC16A4:HMGCS2:SEMA6C:HCN3:APCS:HSPA6:LRIT1:DMBT1:APBB1:BBOX1:SLC43A1:UBE2L6:TM7SF2:C1S:LGR5:DAO:SDS:PCK2:SIX6:CHRNB4:ASGR2:RCVRN:HNF1B:SSTR2:NFKBIB:POU2F2:BCAM:CD33:FPR2:IGFBP2:TGM2:SLPI:APOL1:ARFGAP3:GTSE1:SCO2:TGFBR2:HYAL1:RBP1:SLC4A4:CFI:OCLN:LECT2:ADAM19:PRSS16:IER3:GSTA2:ESR1:CLDN3:PIP:CA2:ST6GALNAC4:CEL:FCN2:PAEP
Computational_gene_sets	MODULE_105	200	22	7.319116892089127e-06	0.0007225910207054515	ACOT7:CKS1B:ACTA2:WEE1:EMG1:EMP1:PSEN1:TCF12:HELZ:PPP4R1:CHAF1A:KEAP1:ERF:PAFAH1B3:PCNA:RBP1:CKS1B:MGAT1:THBS2:HSPB1:FAM3C:PUF60:KIAA0020
Computational_gene_sets	MODULE_255	448	37	8.01510391230578e-06	0.0007225910207054515	TAL1:GJA5:SEMA4A:SELP:ADCK3:STAMBPL1:ZNF143:SLC43A1:RBM7:METTL7A:SLC11A2:KDELC1:NRL:COCH:PRPF39:MAPKBP1:SIAH1:PITPNC1:MYL12A:DCAF15:EPS15L1:FPR2:PPP1CB:IL1RL1:IL18R1:C3orf17:IL8:C5orf20:WRNIP1:WDR46:PLEKHG1:ZNF117:ZMAT4:GEM:DIRAS2:NELFB:TOR4A
Computational_gene_sets	MODULE_179	466	38	8.136241509667602e-06	0.0007225910207054515	TAL1:GJA5:SEMA4A:SELP:ADCK3:STAMBPL1:ZNF143:SLC43A1:RBM7:METTL7A:SLC11A2:KDELC1:NRL:COCH:PRPF39:MAPKBP1:SIAH1:PITPNC1:MYL12A:DCAF15:EPS15L1:FPR2:GREB1:PPP1CB:IL1RL1:IL18R1:C3orf17:IL8:C5orf20:WRNIP1:WDR46:PLEKHG1:ZNF117:ZMAT4:GEM:DIRAS2:NELFB:TOR4A
Computational_gene_sets	MODULE_95	557	43	8.421806768128807e-06	0.0007225910207054515	ICMT:TXNDC12:HOOK1:RBM15:LAMTOR5:SEMA6C:CRB1:TLL2:KCNJ11:BBOX1:DAO:RFX7:RASL12:ADAMTS7:SIAH1:METTL16:TAF4B:S1PR5:SLC6A16:FPR2:HPCAL1:MYCN:KIAA1841:RBMS1:VIL1:ZNF343:SEMG1:TYMP:C3orf55:STK32B:ATP10D:C4orf17:TERT:CDX1:WDR46:HCRTR2:INMT:BCAP29:CPA1:GIMAP5:STAR:ADAMTSL1:ZNF79
Computational_gene_sets	GNF2_FBL	147	18	1.119720675127091e-05	0.000873382126599131	RPL22:TARDBP:RBM15:DAP3:SNRPE:GTF3A:RPL19:KPNB1:RPL38:XPO1:NCL:RPL32:RPL14:RPL37:DIMT1:RPL7:KIAA0020:RPL12
Computational_gene_sets	MODULE_205	305	28	1.4848390858244885e-05	0.0010616599463645093	EXTL2:VAV3:SEMA6C:LCE1B:KCNJ11:STXBP6:FANCM:DLGAP5:B9D1:SLC47A1:LYZL6:CACNB1:NAPG:TUBB4A:MUC16:NPHS1:ZNF83:GREB1:TTLL4:THUMPD3:MYRIP:CACNA2D2:CD200R1:FCHSD1:PRSS16:CNR1:ZNF117:CEP41

Computational_gene_sets	MODULE_459	429	35	1.803346987963122e-05	0.0011902090120556605	TAL1:GJA5:SEMA4A:ADCK3:STAMBPL1:ZNF143:SLC43A1:RBM7:METTL7A:SLC11A2:KDELC1:NRL:COCH:PRPF39:MAPKBP1:SIAH1:PITPNC1:MYL12A:DCAF15:EPS15L1:FPR2:IL1RL1:IL18R1:C3orf17:IL8:C5orf20:WRNIP1:WDR46:PLEKHG1:ZNF117:ZMAT4:GEM:DIRAS2:NELFB:TOR4A
Computational_gene_sets	MODULE_378	433	35	2.1900277623130787e-05	0.001252695880043081	TAL1:GJA5:SEMA4A:ADCK3:STAMBPL1:ZNF143:SLC43A1:RBM7:METTL7A:SLC11A2:KDELC1:NRL:COCH:PRPF39:MAPKBP1:SIAH1:PITPNC1:MYL12A:DCAF15:EPS15L1:FPR2:IL1RL1:IL18R1:C3orf17:IL8:C5orf20:WRNIP1:WDR46:PLEKHG1:ZNF117:ZMAT4:GEM:DIRAS2:NELFB:TOR4A
Computational_gene_sets	MODULE_5	433	35	2.1900277623130787e-05	0.001252695880043081	TSPAN1:F3:FCGR2A:FCGR3A:CSRP1:GRK5:DMBT1:UBE2L6:C1S:GPRC5A:POSTN:LCP1:CORO1A:MFAP4:PPP4R1:CD97:BCAM:RBMS1:TGM2:SLPI:FBLN1:TYMP:TGFBR2:CACNA2D2:IL8:CXCL1:PDGFRB:ADAM19:IER3:CDKN1A:TNFAIP3:GNG11:CALD1:LYN:ST6GALNAC4
Computational_gene_sets	MODULE_317	438	35	2.7794075986059643e-05	0.001455271499298802	TAL1:GJA5:SEMA4A:ADCK3:STAMBPL1:ZNF143:SLC43A1:RBM7:METTL7A:SLC11A2:KDELC1:NRL:COCH:PRPF39:MAPKBP1:SIAH1:PITPNC1:MYL12A:DCAF15:EPS15L1:FPR2:IL1RL1:IL18R1:C3orf17:IL8:C5orf20:WRNIP1:WDR46:PLEKHG1:ZNF117:ZMAT4:GEM:DIRAS2:NELFB:TOR4A
Computational_gene_sets	GCM_DFFA	129	16	2.8834050685407496e-05	0.001455271499298802	TARDBP:C1orf52:CSDE1:KIAA0907:LINC00610:COMMD9:KPNA3:IPO5:RBM25:UBR7:SMG1:TBC1D10B:FAM168B:ARMC8:ZFP62:VPS52:MEPCE
Computational_gene_sets	MODULE_27	354	30	3.457293347136313e-05	0.00155293922672282	TIE1:MPL:LPHN2:F3:FCGR2A:FCGR3A:SELP:RGR:FAS:OPCML:GPRC5A:CHRNB4:ASGR2:PTGER1:IL12RB1:CD79A:FPR2:IL18R1:MERTK:PPARA:HYAL2:CFI:NPY1R:PDGFRB:HLA-A:CDKN1A:HCRTR2:CNR1:ITGB8:CLDN4
Computational_gene_sets	MODULE_408	18	6	3.523822546686423e-05	0.00155293922672282	PSAP:SMPD1:NAGA:ARSA:HYAL1:NSMAF
Computational_gene_sets	MODULE_13	517	39	3.619904957395851e-05	0.00155293922672282	HTR6:SZT2:ADAM15:FAM189B:PMF1:FCGR2A:LRIT1:DMBT1:ZNF143:ABCC8:SAC3D1:AKAP3:LRRC23:LGR5:WBP4:GRIN2A:CIAPIN1:CALB2:ASGR2:DNAH9:LYZL6:TECR:CD33:ATP6V1B1:PI3:GTSE1:ACKR2:IFRD2:RASSF1:KCNAB1:PCDHGC3:MGAT1:PRSS16:MDC1:ESR1:CRHR2:PPP3CC:LY6E:ST6GALNAC4
Computational_gene_sets	MODULE_36	161	18	3.874581223056444e-05	0.0015830431854202043	EXOSC10:SPEN:TM2D1:CSRNP2:UCHL3:ARPP19:CYB5B:TTLL4:PER2:PCNA:CDS2:TRAK1:PROS1:COQ2:NIPBL:ZNF117:ASH2L:NSMAF
Computational_gene_sets	GCM_DEAF1	27	7	4.807975863029308e-05	0.0018370053886208263	GPR153:MOAP1:HERC2:NAPG:FAM168B:YWHAG:UBQLN1
Computational_gene_sets	MODULE_41	562	41	4.924373419379838e-05	0.0018370053886208263	SZT2:FCGR2A:LRIT1:DMBT1:ZNF143:ABCC8:SAC3D1:RCE1:AKAP3:HOXC11:LGR5:SIX6:CHRNB4:CIAPIN1:CALB2:ASGR2:DNAH9:CACNB1:PDE4A:C19orf57:CD33:FPR2:IL1RL1:PI3:NDUFA6:CYP2D6:ARFGAP3:GTSE1:ACKR2:CACNA2D2:GP9:KCNAB1:C5orf30:ADAM19:PRSS16:ESR1:CRHR2:DUS4L:PPP3CC:ST6GALNAC4:FCN2
Computational_gene_sets	MODULE_1	366	30	6.377913451972327e-05	0.002280104059080107	C1orf63:CTSK:LMNA:CSRP1:FMOD:SLC43A1:C1S:EMP1:ULK1:PKD1:MFAP4:BCAM:IGFBP2:TGM2:SLPI:FBLN1:TGFBR2:RBP1:PDGFRB:ADAM19:IER3:CDKN1A:TNFAIP3:SYNE1:THBS2:GNG11:CALD1:STAR:GEM:KLF4
Computational_gene_sets	MODULE_532	405	32	7.437818692063649e-05	0.0025526593751162446	TAL1:GJA5:ADCK3:STAMBPL1:ZNF143:RBM7:METTL7A:SLC11A2:KDELC1:NRL:COCH:PRPF39:MAPKBP1:SIAH1:MYL12A:DCAF15:EPS15L1:FPR2:IL1RL1:IL18R1:C3orf17:IL8:C5orf20:WRNIP1:WDR46:PLEKHG1:ZNF117:ZMAT4:GEM:DIRAS2:NELFB:TOR4A
Computational_gene_sets	MODULE_84	541	39	9.559451617015776e-05	0.0031546190336152057	CTSS:TNFAIP8L2:SEMA4A:FCGR2A:HSPA6:FCGR3A:C10orf54:LRIT1:ACTA2:UBE2L6:C1S:SDS:LCP1:KIAA0226L:FOXG1:SIX6:CORO1A:IFI35:CD79A:FPR2:TPO:IGFBP2:TGM2:PI3:SLPI:TYMP:TGFBR2:RBP1:IL8:CXCL1:PLAC8:ADAM19:HLA-A:IER3:CDKN1A:TNFAIP3:SYNE1:LYN:ST6GALNAC4
Computational_gene_sets	MORF_AATF	209	20	0.0001343391079942309	0.00426899832070556	CLSTN1:MTOR:TXLNA:DAP3:HSPA8:USP5:PHB2:COQ9:POLR2C:SAFB:DDX39A:PUM2:XPO1:NCL:EIF6:RTCB:SEPT7:ASH2L:PUF60:NELFB
Computational_gene_sets	MORF_DAP3	195	19	0.0001545615560006274	0.00473620768030494	FDPS:DAP3:SSR2:SNRPE:HSPA8:PHB2:SLC25A3:PUM2:XPO1:NCL:EIF6:RPN2:RTCB:VGLL4:CNBP:AP2M1:RPP40:PUF60:NELFB
Computational_gene_sets	GCM_RAN	196	19	0.00016522599784996443	0.004888410557078258	HIPK1:TRIM33:IPO7:SNX15:CSRNP2:KPNA3:IPO5:MOAP1:UBR7:HERC2:ARPP19:TBC1D10B:SPAG9:PITPNC1:NAPG:FAM168B:GSK3B:VPS52:YWHAG
Computational_gene_sets	MODULE_45	576	40	0.00017200992038119998	0.004907832293472221	HTR6:CTSS:SEMA6C:FCGR2A:HSPA6:FCGR3A:LRIT1:SLC43A1:UBE2L6:PTPN6:LCP1:SIX6:CORO1A:IFI35:IMPA2:CD97:PKN1:MAST3:NFKBIB:CD79A:FPR2:SLC11A1:PI3:SLPI:SCO2:TYMP:TGFBR2:ACKR2:KCNAB1:IL8:ADAM19:IER3:TNFAIP3:SYNE1:SOD2:CRHR2:GNG11:PPP3CC:LYN:ST6GALNAC4

Computational_gene_sets	MODULE_48	334	27	0.0001814166566440918	0.004907832293472221	ICMT:TXNDC12:HOOK1:RBM15:SLC16A4:LAMTOR5:TLL2:RFX7:SIAH1:METTL16:S1PR5:HPCAL1:KIAA1841:RBMS1:VIL1:ZNF343:TYMP:TERT:CDX1:WDR46:INMT:BCAP29:CPA1:GIMAP5:STAR:ADAMTSL1:ZNF79
Computational_gene_sets	MODULE_100	539	38	0.00018304269626003624	0.004907832293472221	F3:DMBT1:DUSP8:APBB1:ABCC8:TM7SF2:TENM4:ENO2:LGR5:PCDH9:FOXG1:SIX6:SH3GL3:PKD1:CORO1A:CALB2:EPN2:SSTR2:MBP:BCL11A:RND3:IGFBP2:SYNGR1:SCO2:MAPK8IP2:CACNA2D2:RBP1:C5orf30:SNCB:CDKN1A:MAP7:SYNE1:THBS2:CA2:CALB1:KHDRBS3:ST6GALNAC4:CEL
Computational_gene_sets	MODULE_137	540	38	0.00018993750510877105	0.004938375132828047	F3:DMBT1:DUSP8:APBB1:ABCC8:TM7SF2:TENM4:ENO2:LGR5:PCDH9:FOXG1:SIX6:SH3GL3:PKD1:CORO1A:CALB2:EPN2:SSTR2:MBP:BCL11A:RND3:IGFBP2:SYNGR1:SCO2:MAPK8IP2:CACNA2D2:RBP1:C5orf30:SNCB:CDKN1A:MAP7:SYNE1:THBS2:CA2:CALB1:KHDRBS3:ST6GALNAC4:CEL
Computational_gene_sets	MODULE_87	44	8	0.00020819742695905988	0.005253923303849217	CDC42:NRAS:RIT1:ARL2:RAP1B:PCK2:GEM:TUBB4B
Computational_gene_sets	MORF_EIF4A2	138	15	0.00022744706158972687	0.005575702252685304	RPL22:PTP4A2:DAP3:ZFPL1:FAU:SLC25A3:RPL19:PUM2:XPO1:FAM168B:NCL:RPL14:SEPT7:TERF1:RPL7
Computational_gene_sets	MODULE_9	124	14	0.0002448023921989298	0.00568379366368463	ICMT:TXNDC12:HOOK1:SLC16A4:HSPA8:RFX7:METTL16:RBMS1:VIL1:TERT:CDX1:BCAP29:STAR:ZNF79
Computational_gene_sets	MODULE_66	547	38	0.0002451053211612253	0.00568379366368463	F3:DMBT1:DUSP8:APBB1:ABCC8:TM7SF2:TENM4:ENO2:LGR5:PCDH9:FOXG1:SIX6:SH3GL3:PKD1:CORO1A:CALB2:EPN2:SSTR2:MBP:BCL11A:RND3:IGFBP2:SYNGR1:SCO2:MAPK8IP2:CACNA2D2:RBP1:C5orf30:SNCB:CDKN1A:MAP7:SYNE1:THBS2:CA2:CALB1:KHDRBS3:ST6GALNAC4:CEL
Computational_gene_sets	MODULE_33	379	29	0.0002711826032698672	0.0061230177264617386	PDZK1IP1:ACTA2:LPCAT3:EMP1:SDS:PKD1:CORO1A:CCL17:SLC7A6:CLEC10A:MAPK7:CACNG1:ERF:PAFAH1B3:CEACAM1:MERTK:SLPI:MAPK8IP2:MAP7:SOD2:ARL4A:RFC2:CD36:CPA1:MEST:CALD1:BLK:KLF4:PTPN3
Computational_gene_sets	MODULE_212	326	26	0.00029180133271459286	0.006419629319721043	ABCD3:VAV3:CTSS:SOAT1:BBOX1:UBE2L6:HSPA8:PHB2:METTL7A:DAO:SDS:SLC25A15:PCK2:SULT1A3:CD320:RND3:TMEM230:PCNA:ARSA:HYAL1:PPAP2A:FLOT1:GSTA2:MAP7:CA2:CYP11B2
Computational_gene_sets	MORF_SUPT3H	327	26	0.00030591727781152054	0.006561925609057115	LPHN2:FAS:ABCC8:TENM4:OPCML:ERC1:AKAP3:HOXC11:WBP4:SIX6:CHRNB4:CACNB1:BRCA1:OR10H3:FSHR:CYP2D6:DAZL:ENTPD3:NR1I2:SLC4A4:ISL1:LECT2:HCRTR2:ABCB1:EXOC4:RB1CC1
Computational_gene_sets	MODULE_112	258	22	0.0003337381365785769	0.006884791558737873	F3:CTSK:FCGR3A:GRK5:ZNF143:C1S:DAO:SDS:CCL17:ASGR2:RCVRN:C19orf57:POU2F2:CEACAM1:RND3:CYP2D6:RBP1:NPY1R:ISL1:THBS2:CRHR2:CDH17
Computational_gene_sets	MODULE_64	517	36	0.00033701776860954624	0.006884791558737873	HTR6:F3:SLC16A4:FCGR2A:FCGR3A:FAS:GRK5:GPRC5A:NCKAP1L:CHRNB4:CCL17:ASGR2:CACNG1:KCNJ2:SSTR2:CD79A:CEACAM1:FPR2:TPO:IL18R1:PPARA:ACKR2:GP9:SLC4A4:IL8:CXCL1:AREG:AREGB:CFI:NPY1R:HLA-A:CDKN1A:CNR1:CRHR2:CLDN4:GNG11:LY6E
Computational_gene_sets	MODULE_117	719	46	0.0003727359374123195	0.007437382193017909	MPL:RPE65:BCAS2:AMPD1:CTSS:APCS:HSPA6:F5:F13B:FMOD:RGR:BBOX1:SCGB2A1:TRPC6:KCNA1:DAO:CORO1A:CACNG1:FSCN2:SLC14A1:ST8SIA5:PTGER1:IL18R1:IGFBP2:BFSP1:KCNS1:SEMG1:MAPK8IP2:HYAL1:AFM:MTTP:TERT:CDH6:CDX1:C4A:GSTA2:CNR1:CLDN4:CD36:PIP:BLK:CA2:CDH17:LRRC6:KCNV2:CEL
Computational_gene_sets	MORF_CDC10	146	15	0.0004193516719124144	0.007883032558330902	RPL22:CLSTN1:PTP4A2:TXLNA:CUL2:HSPA8:POLR2C:SAFB:PUM2:XPO1:RTCB:DIAPH1:SEPT7:ASH2L:TERF1
Computational_gene_sets	MORF_DDB1	245	21	0.0004206329371108261	0.007883032558330902	CLSTN1:TXLNA:ADAM15:FAM189B:DAP3:HSPA8:USP5:PHB2:PDXDC1:POLR2C:DDX39A:EIF6:RTCB:VGLL4:DIMT1:CETN3:MGAT1:STK19:SEPT7:PUF60:NELFB
Computational_gene_sets	MODULE_342	211	19	0.0004226334471832419	0.007883032558330902	FBLIM1:NBL1:HOOK1:VAV3:ANXA9:FMOD:CDCA5:GPRC5A:STXBP6:UBXN6:RTKN:IGFBP2:SLPI:HYAL1:OCLN:CDKN1A:MAP7:CLDN4:CDH17
Computational_gene_sets	MODULE_146	102	12	0.0004560923306723385	0.008326111057805669	HTR6:RGR:OPCML:CHRNB4:SSTR2:PTGER1:OR10H3:FPR2:ACKR2:NPY1R:HCRTR2:CNR1
Computational_gene_sets	MODULE_18	450	32	0.0004910023515286761	0.008663698556880976	ICMT:ACOT7:CDC20:FDPS:PMF1:SLC43A1:LGR5:IPO5:DLGAP5:TCF12:CIAPIN1:CYB5B:VAT1:CHAF1A:DDX39A:NFKBIB:RND3:COPS8:PCNA:APOBEC3B:GTSE1:ACKR2:IFRD2:IL8:CXCL1:C5orf30:MDC1:IER3:PTP4A1:THBS2:GNG11:ST6GALNAC4
Computational_gene_sets	MODULE_180	118	13	0.0005108106066533516	0.008663698556880976	HOOK1:VAV3:ANXA9:UCP2:METTL7A:ESRP2:UBXN6:BCAM:ISY1:NPY1R:OCLN:MAP7:CLDN4

Computational_gene_sets MORF_ERCC4	320	25	0.0005122395567698657	0.008663698556880976	TIE1:FAS:ABCC8:TENM4:ERC1:HOXC11:WBP4:SIX6:CHRNB4:CACNB1:BRCA1:PDE4A:OR10H3:FPR2:FSHR:CYP2D6:GTSE1:ENTPD3:NR1I2:ISL1:LECT2:HCRTR2:ABCB1:EXOC4:RB1CC1
Computational_gene_sets GCM_NF2	302	24	0.0005149750890453727	0.008663698556880976	C1orf52:ASH1L:UBQLN4:SMG5:NUCKS1:AGAP4:AGAP8:LINC00610:COMMD9:SNX15:DDX47:CSRNP2:DHX37:IPO5:GTF2A1:UBR7:TBC1D10B:CIAPIN1:NAPG:SAFB:EPS15L1:FAM168B:ACAD11:ANKRA2:VPS52:MEPCE
Computational_gene_sets GNF2_DAP3	119	13	0.0005540452006474264	0.009141745810682535	RPL22:TARDBP:RBM15:DAP3:SNRPE:GTF3A:RPL19:RPL38:RPL32:RPL14:RPL37:RPL7:RPL12
Computational_gene_sets MODULE_49	288	23	0.0006226943275708999	0.00988472472881446	SMCP:NHLH1:WEE1:SYT13:KCNA1:DAO:KLHL28:ZFYVE1:SH3GL3:ADAM11:TAF4B:SH3GL1:KCNJ14:NOL10:ZNF343:NDUFAF5:TYMP:C3orf55:STK32B:IPO11:CDX1:KCNIP1:HCRTR2
Computational_gene_sets GNF2_ST13	64	9	0.0006283059630125116	0.00988472472881446	RPL22:SNRPE:RPL19:RPL38:RPL32:RPL14:RPL37:RPL7:RPL12
Computational_gene_sets MORF_PTPRB	253	21	0.0006409234216007166	0.00988472472881446	LPHN2:FAS:ABCC8:TENM4:ERC1:WBP4:SIX6:NIPA2:CHRNB4:OR10H3:FPR2:TPO:FSHR:DAZL:NR1I2:ISL1:LECT2:HCRTR2:ABCB1:EXOC4:RB1CC1
Computational_gene_sets GNF2_EIF3S6	121	13	0.0006498381056076144	0.00988472472881446	RPL22:DAP3:FAU:DDX47:GTF3A:RPL19:RPL38:RPL32:RPL14:RPL37:RPS18:RPL7:RPL12
Computational_gene_sets MODULE_3	382	28	0.0006736688215241973	0.00988472472881446	ACOT7:CDC20:EXTL2:SEMA6C:FDPS:DAP3:IPO7:NDUFA9:EMP1:DLGAP5:CYB5B:VAT1:PPP4R1:SH3GL1:CHAF1A:ERF:RND3:IGFBP2:PCNA:TGM2:APOBEC3B:TGFBR2:IER3:CDKN1A:PTP4A1:GNG11:MEST:CALD1
Computational_gene_sets MORF_CCNF	78	10	0.0006763263993141517	0.00988472472881446	EXTL2:PDXDC1:SMG1:BRCA1:KPNB1:GTSE1:COQ2:EIF4E:DIMT1:CETN3
Computational_gene_sets GCM_GSPT1	169	16	0.0006797188333333953	0.00988472472881446	TARDBP:TRIM33:KIAA0907:AGAP4:AGAP8:LINC00610:COMMD9:SNX15:CSRNP2:IPO5:UBR7:TBC1D10B:NAPG:FAM168B:ATP2C1:ANKRA2:VPS52:MEPCE
Computational_gene_sets MODULE_545	20	5	0.0007227600354142013	0.010114993578282992	DAO:IL1RL1:IL18R1:SOX30:GSTA2
Computational_gene_sets MORF_ACTG1	138	14	0.0007303116987606548	0.010114993578282992	RPL22:SSR2:ZFPL1:FAU:SLC25A3:RPL19:RPL38:NCL:RPL32:RPL14:RPL37:RPS18:RPL7:RPL12
Computational_gene_sets MORF_UBE2I	238	20	0.0007309202818805891	0.010114993578282992	RPL22:TARDBP:PTP4A2:DAP3:SSR2:SNRPE:ZFPL1:FAU:PHB2:SLC25A3:RPL19:XPO1:FAM168B:NCL:EIF6:RPL14:CNBP:AP2M1:RPL7:PUF60
Computational_gene_sets MORF_PRKAG1	223	19	0.0008306767552365658	0.011313026285602754	CLSTN1:TXLNA:ADAM15:FAM189B:DAP3:HSPA8:USP5:PDXDC1:COQ9:POLR2C:RPN2:RTCB:VGLL4:DIMT1:CETN3:MGAT1:STK19:SEPT7:NELFB
Computational_gene_sets MODULE_94	370	27	0.0008887130754842072	0.011914309668210152	ARHGEF11:SELP:PSEN2:APBB1:CDON:SLC11A2:SDS:SIX6:CCL17:CLEC10A:HNF1B:KEAP1:NFKBIB:CARD8:GREB1:APOL1:SCO2:ENTPD3:HYAL1:ABCF3:MAP3K13:CDX1:SNCB:IER3:B3GALT4:TPST1:KIAA0020
Computational_gene_sets GCM_MLL	175	16	0.0009893100056247038	0.012800402188626278	TARDBP:C1orf52:KIAA0907:LINC00610:COMMD9:DDX47:CSRNP2:IPO5:RBM25:UBR7:SMG1:TBC1D10B:SAFB:FAM168B:CREB1:ANKRA2:MEPCE
Computational_gene_sets MODULE_6	411	29	0.000991485548706895	0.012800402188626278	C1orf63:TSPAN1:F3:HMGCS2:FMOD:DMBT1:C1S:GPRC5A:MFAP4:CD79A:IGFBP2:TGM2:PI3:SLPI:FBLN1:TGFBR2:IL8:CXCL1:SLC12A2:PDGFRB:IER3:CDKN1A:TNFAIP3:CLDN3:CLDN4:PIP:LYN:GEM:ST6GALNAC4
Computational_gene_sets MORF_RPA2	192	17	0.0009995652058717491	0.012800402188626278	CLSTN1:TXLNA:ADAM15:FAM189B:USP5:PHB2:COQ9:GSK3A:XPO1:EIF6:RTCB:IFRD2:DIAPH1:MGAT1:SEPT7:ASH2L:NELFB
Computational_gene_sets MODULE_480	97	11	0.0010576387296793257	0.013133673412739431	PTHLH:PSEN1:TRPM1:IFI35:DNAJB1:FAM49A:SNED1:SYNGR1:SRPRB:CFI:PILRB
Computational_gene_sets MODULE_83	318	24	0.0010585032744754388	0.013133673412739431	DAP3:SNRPE:IPO7:FAU:HSPA8:ATN1:PHB2:RAP1B:RPL19:RPL38:MYL12A:MYL12B:XPO1:RBMS1:NCL:SEMG2:RPL32:RPL14:RPL37:RPS18:HSPB1:YWHAG:RPL7:RPL12
Computational_gene_sets MODULE_176	228	19	0.0010813588056956588	0.013133673412739431	F3:CTSK:FCGR3A:GRK5:ZNF143:C1S:SDS:CCL17:ASGR2:C19orf57:POU2F2:CEACAM1:RND3:CYP2D6:NPY1R:ISL1:THBS2:CRHR2:CDH17
Computational_gene_sets GCM_CSNK1D	32	6	0.0010959683886225082	0.013133673412739431	GPR153:FAM168B:CHMP4B:VPS52:ZFAND3:UBQLN1

Computational_gene_sets	MORF_PPP2CA	128	13	0.0011021264402298824	0.013133673412739431	TARDBP:HSPA8:RAP1B:SLC25A3:SAFB:DDX39A:XPO1:FAM168B:NCL:RTCB:CNBP:SEPT7:PUF60
Computational_gene_sets	MORF_NPM1	162	15	0.0012356773436303398	0.014523440559381253	RPL22:SNRPE:FAU:PHB2:SLC25A3:RPL19:RPL38:NCL:RPL32:RPL14:RPL37:TUBB:RPS18:RPL7:RPL12
Computational_gene_sets	MORF_RAN	267	21	0.0012698873284719273	0.014596457836117713	TARDBP:FDPS:DAP3:SSR2:SNRPE:FAU:HSPA8:TPI1:PHB2:SLC25A3:UTP18:MRPS12:XPO1:NCL:PCNA:RPN2:RPL14:CNBP:AP2M1:RPL7:PUF60
Computational_gene_sets	MORF_GNB1	304	23	0.0012876655903371588	0.014596457836117713	CLSTN1:TARDBP:PTP4A2:TXLNA:SNRPE:PHB2:SLC25A3:ANP32A:POLR2C:SAFB:PKN1:PUM2:XPO1:FAM168B:NCL:RTCB:TRAK1:DCTD:SKIV2L2:DIAPH1:MDC1:SEPT7:TERF1
Computational_gene_sets	MODULE_11	537	35	0.0012929263351339699	0.014596457836117713	F3:DMBT1:DUSP8:APBB1:ABCC8:TM7SF2:TENM4:ENO2:LGR5:PCDH9:FOXG1:MOAP1:SH3GL3:PKD1:CORO1A:CALB2:EPN2:SSTR2:MBP:BCL11A:IGFBP2:SYNGR1:SCO2:MAPK8IP2:CACNA2D2:RBP1:C5orf30:SNCB:MAP7:SYNE1:THBS2:CA2:CALB1:KHDRBS3:ST6GALNAC4
Computational_gene_sets	MODULE_8	419	29	0.0013301908883687802	0.01482212704182355	CDC20:ABCD3:SEMA6C:FDPS:DAP3:IPO7:WEE1:SLC43A1:NDUFA9:SLC11A2:IPO5:DCAF11:ARPP19:ASGR2:CHAF1A:DDX39A:RND3:VIL1:PCNA:RBP1:IL8:CXCL1:AREG:AREGB:OCLN:IER3:PTP4A1:ARL4A:MEST:KIAA0020
Computational_gene_sets	MODULE_72	307	23	0.0014641958944668426	0.01610615483913527	CDC20:ABCD3:DAP3:CREM:SAC3D1:AKAP3:C1S:ULK1:MTMR6:FOXG1:ARPP19:CALB2:UTP18:PPP4R1:SAFB2:DDX39A:NFKBIB:IGFBP2:PCNA:RBP1:PDGFRB:ARL4A:KHDRBS3
Computational_gene_sets	GNF2_NPM1	72	9	0.0014860993320104999	0.016140167428671	TARDBP:SNRPE:XPO1:NCL:RPL14:RPL37:DIMT1:RPL7:KIAA0020
Computational_gene_sets	MODULE_118	403	28	0.0015070195140548372	0.016162784288238127	CDC20:FDPS:LRIT1:DMBT1:ENO2:EMP1:LGR5:LCP1:DLGAP5:SIX6:CORO1A:CHAF1A:CD79A:PAFAH1B3:CD33:RND3:IGFBP2:PCNA:TGM2:GTSE1:TGFBR2:RBP1:CXCL1:C5orf30:ADAM19:IER3:CDKN1A:CALD1
Computational_gene_sets	MORF_XRCC5	235	19	0.0015389923025911842	0.016301918464484397	TARDBP:TXLNA:DAP3:SNRPE:PHB2:SLC25A3:ANP32A:COQ9:CHAF1A:SAFB:PUM2:XPO1:FAM168B:NCL:DCTD:SKIV2L2:DIAPH1:SEPT7:TERF1
Computational_gene_sets	MODULE_324	133	13	0.0015656158220169018	0.01630437937640234	CRB1:C1S:CALB2:RCVRN:MYL12A:FBLN1:SCO2:ADPRH:CDH6:TNFAIP3:THBS2:CA2:CDH17
Computational_gene_sets	MODULE_334	166	15	0.0015772301727755176	0.01630437937640234	TRIM33:GRK5:PTHLH:PSEN1:ARPP19:IFI35:DNAJB1:FAM49A:SNED1:SYNGR1:SRPRB:RPS18:PILRB:PRKAR2B:ESYT2
Computational_gene_sets	MORF_RAD23A	348	25	0.0016727748007114539	0.017086199750124136	ACOT7:TARDBP:SCAMP3:SNRPE:PHB2:SLC25A3:ERCC5:ANP32A:SAFB:DDX39A:PKN1:MRPS12:XPO1:FAM168B:NCL:RTCB:DCTD:SKIV2L2:DIAPH1:TTC1:MDC1:NSMAF:TERF1:PUF60:UBAC1
Computational_gene_sets	MODULE_432	15	4	0.0019512189121662113	0.019695833254571873	HMGCS2:FDPS:LSS:CNBP
Computational_gene_sets	MODULE_114	336	24	0.0022049440469015104	0.02199816270048251	FDPS:SNRPE:FAU:UCP2:HSPA8:TPI1:PHB2:RPL19:RPL38:MYL12B:TPRKB:RPE:NCL:TMEM230:PCNA:SEMG2:TPD52L2:RPL32:RPL14:HLA-A:HSPB1:YWHAG:RPL7:RPL12
Computational_gene_sets	GCM_MYST2	172	15	0.0022351454202916505	0.022043158282876278	TARDBP:PTBP2:HIPK1:CSDE1:ARL5B:IPO7:LINC00610:COMMD9:CSRNP2:KPNA3:RBM25:UBR7:SPAG9:FAM168B:GSK3B:ZFAND3
Computational_gene_sets	MODULE_99	356	25	0.0022703512164610916	0.022135924360495645	FOXD2:RPE65:KCNC4:SLC16A4:AMPD1:F5:APBB1:BBOX1:SCGB2A1:TRPC6:KCNA1:GTF2A1:RPL3L:CD2BP2:EPN2:DGKE:FSCN2:IL12RB1:KCNS1:SEMG1:PPARA:AFM:THBS2:ASL:KCNV2
Computational_gene_sets	MODULE_96	37	6	0.0023964541869204103	0.023102895419974293	LPHN2:APCS:SELP:CLEC10A:ASGR2:FCN2
Computational_gene_sets	MORF_MLLT10	301	22	0.0024879390939457294	0.02364916503814992	TIE1:FAS:ABCC8:TENM4:ERC1:AKAP3:WBP4:SIX6:MGA:CHRNB4:CACNB1:BRCA1:OR10H3:FSHR:CYP2D6:DAZL:NR1I2:LECT2:HCRTR2:ABCB1:EXOC4:RB1CC1
Computational_gene_sets	MODULE_289	124	12	0.0025358079061885697	0.02364916503814992	LPHN2:RGR:GRK5:PTPN6:CCL17:PTGER1:OR10H3:FPR2:IL8:CXCL1:NPY1R:HCRTR2
Computational_gene_sets	MODULE_345	124	12	0.0025358079061885697	0.02364916503814992	FCRLA:UBE2L6:KIAA0226L:KCTD12:IFI35:CD79A:POU2F2:FOXP1:NEK11:ADAM19:SLC22A23:HLA-A

Computational_gene_sets	MODULE_23	562	35	0.0027369331 89888369	0.0246362780 11133612	HMGCS2:ANXA9:SEMA6C:HCN3:APCS:SLC43A1:UBE2L6:SAC3D1:TM7SF2:PC:C1S:DAO:SDS:PCK2:CLEC10A:ASGR2:NFKBIB:IGFBP2:SLC11A1:TGM2:SLPI:APOL1:CYP2D6:SCO2:ARSA:HYAL1:RBP1:LECT2:FLOT1:GSTA2:ESR1:CLDN3:ST6GALNAC4:FCN2:PAEP
Computational_gene_sets	MODULE_63	211	17	0.0027450352 58265616	0.0246362780 11133612	TIE1:KCNC4:GJA5:FCGR2A:FCGR3A:KCNJ11:KCNA1:GPRC5A:CHRNB4:ASGR2:CACNG1:KCNJ2:MERTK:HYAL2:PDGFRB:ITGB8:GFRA2
Computational_gene_sets	MORF_ACP1	211	17	0.0027450352 58265616	0.0246362780 11133612	RPL22:TARDBP:PTP4A2:SNRPE:ZFPL1:PHB2:SLC25A3:ERCC5:ANP32A:SAFB:XPO1:FAM168B:NCL:RPL14:DIAPH1:TTC1:TERF1
Computational_gene_sets	GNF2_TPT1	38	6	0.0027565066 306163484	0.0246362780 11133612	RPL19:RPL38:RPL32:RPL37:RPS18:RPL12
Computational_gene_sets	GNF2_STAT6	79	9	0.0028461722 026213777	0.0251754201 01537546	PTPN6:LCP1:CORO1A:MOB1A:HLA-A:TAPBP:RNASET2:LAT2:LYN
Computational_gene_sets	GNF2_HDAC1	110	11	0.0029183640 23655285	0.0252924882 05012467	RPL22:TARDBP:RPF1:RBM15:CLK2:DAP3:DDX47:GTF3A:KPNB1:OSR1:TAPBP
Computational_gene_sets	GCM_CALM1	110	11	0.0029183640 23655285	0.0252924882 05012467	DDAH1:CSRNP2:NUAK1:MOAP1:HERC2:NAPG:FAM168B:GSK3B:NDFIP1:VPS52:YWHAG
Computational_gene_sets	MODULE_330	27	5	0.0030254364 526428636	0.0259582447 6367577	GJA5:KCNA1:CACNG1:DSTN:CALD1
Computational_gene_sets	MODULE_346	17	4	0.0032039925 68515157	0.0272180754 8302975	PSAP:SMPD1:ARSA:NSMAF
Computational_gene_sets	MORF_RAD51L3	386	26	0.0032998831 845490197	0.0275593949 54955622	LPHN2:FAS:ABCC8:TENM4:OPCML:ERC1:AKAP3:HOXC11:WBP4:SIX6:MGA:CHRNB4:CACNB1:BRCA1:OR10H3:FSHR:CYP2D6:DAZL:NR1I2:SLC4A4:ISL1:LECT2:HCRTR2:ABCB1:EXOC4:RB1CC1
Computational_gene_sets	MODULE_169	96	10	0.0033084122 14872295	0.0275593949 54955622	TNFAIP8L2:ULK1:HEATR5A:ATP2C1:KLHL6:STIM2:ZNF608:PCDHGC3:IER3:PRKAR2B
Computational_gene_sets	MORF_FOSL1	407	27	0.0034303722 76152025	0.0283005712 78254204	TIE1:FAS:ABCC8:TENM4:ERC1:AKAP3:HOXC11:WBP4:SIX6:MGA:CHRNB4:SMG1:CACNB1:BRCA1:PDE4A:FPR2:FSHR:ATP6V1B1:CYP2D6:GTSE1:NR1I2:LECT2:HCRTR2:ESR1:ABCB1:EXOC4:RB1CC1
Computational_gene_sets	MODULE_248	129	12	0.0035143058 202303954	0.0287168989 8816837	RPE65:ARHGEF11:ABL2:PSEN2:PTPN6:RCVRN:DGKE:FSCN2:MERTK:IL8:CXCL1:PTPN3
Computational_gene_sets	MODULE_104	349	24	0.0035792255 778440323	0.0289714674 13114904	SZT2:LRIT1:DMBT1:ZNF143:ABCC8:RCE1:AKAP3:HOXC11:SIX6:CHRNB4:CALB2:RCVRN:DNAH9:SSTR2:PDE4A:C19orf57:CD33:PI3:SLPI:ARFGAP3:GTSE1:C5orf30:PRSS16:FCN2
Computational_gene_sets	MODULE_129	218	17	0.0038322232 875210274	0.0307294166 41991043	TSPAN1:SLC16A4:GRK5:HPX:BBOX1:KCNA1:PCDH9:CCL17:CEACAM1:KCNS1:SEMG1:CELSR1:MAPK8IP2:THBS2:TPST1:GNG11:KHDRBS3
Computational_gene_sets	MODULE_427	115	11	0.0041139492 95625721	0.0326830416 263599	PTHLH:PSEN1:TRPM1:IFI35:DNAJB1:FAM49A:SNED1:SYNGR1:SRPRB:CFI:PILRB
Computational_gene_sets	MODULE_98	393	26	0.0041760781 274914624	0.0328722480 1273096	FOXD2:SETDB1:CLK2:SNRPE:IPO7:WEE1:ATN1:EMG1:POLE:FOXG1:TCF12:ANP32A:KPNB1:PSMD12:CHAF1A:SAFB:MYCN:COPS8:PCNA:CCNO:CDKN1A:MAP7:HOXA13:ZNF117:RFC2:KHDRBS3
Computational_gene_sets	MODULE_32	239	18	0.0042987755 92947615	0.0335304496 249914	RPL22:RIT1:SNRPE:RAP1B:RPL19:RPL38:CHAF1A:RBMS1:NCL:EIF6:RPL32:GNAI2:RPL37:RNASET2:RPL7:GEM:RPL12:TUBB4B
Computational_gene_sets	MODULE_151	316	22	0.0044484148 61417988	0.0343850446 04474176	FDPS:SNRPE:UCP2:HSPA8:TPI1:PHB2:RPL19:RPL38:MYL12B:TPRKB:RPE:NCL:TMEM230:PCNA:SEMG2:TPD52L2:RPL32:RPL14:HLA-A:YWHAG:RPL7:RPL12
Computational_gene_sets	MORF_MAGEA8	259	19	0.0045325408 20056844	0.0347225002 10792616	LPHN2:FAS:ABCC8:TENM4:AKAP3:WBP4:SIX6:CHRNB4:OR10H3:FSHR:DAZL:NR1I2:SLC4A4:ISL1:LECT2:HCRTR2:ABCB1:EXOC4:RB1CC1
Computational_gene_sets	MORF_CTBP1	168	14	0.0046296982 48438317	0.0347537678 4614965	TARDBP:DAP3:SSR2:SNRPE:SLC25A3:MRPS12:XPO1:FAM168B:NCL:CNBP:AP2M1:DIAPH1:VPS52:PUF60

Computational_gene_sets	GCM_BMPR2	85	9	0.0046654705303718555	0.03475376784614965	MACF1:IPO7:CSRNP2:HERC2:FAM168B:GSK3B:NDFIP1:VPS52:ZFAND3
Computational_gene_sets	GCM_HBP1	70	8	0.004698644603908344	0.03475376784614965	TARDBP:BTF3L4:C1orf52:KIAA0907:LINC00610:COMMD9:RBM25:RRN3:SLAIN2
Computational_gene_sets	GCM_TPT1	70	8	0.004698644603908344	0.03475376784614965	SLC25A3:RPL19:RPL38:NCL:RPL32:RPL14:RPS18:RPL7
Computational_gene_sets	MODULE_53	397	26	0.004759113447244982	0.03490016527979654	CDC20:FDPS:LRIT1:DMBT1:WEE1:UBE2L6:SAC3D1:ENO2:LGR5:LCP1:PCDH9:DLGAP5:SIX6:CORO1A:SSTR2:CHAF1A:CD79A:PAFAH1B3:CD33:IGFBP2:PCNA:GTSE1:C5orf30:ADAM19:IER3:ST6GALNAC4
Computational_gene_sets	MODULE_436	135	12	0.005067906972149549	0.03654184305323932	FCRLA:UBE2L6:KIAA0226L:KCTD12:IFI35:CD79A:POU2F2:FOXP1:NEK11:ADAM19:SLC22A23:HLA-A
Computational_gene_sets	MORF_TPT1	102	10	0.005110747280173332	0.03654184305323932	RPL22:ZFPL1:FAU:RPL19:RPL38:RPL32:RPL37:RPS18:RPL7:RPL12
Computational_gene_sets	MORF_PTPRR	102	10	0.005110747280173332	0.03654184305323932	TENM4:AKAP3:HOXC11:SIX6:FSHR:DAZL:LECT2:HCRTR2:ABCB1:EXOC4
Computational_gene_sets	MODULE_2	380	25	0.0052552564296603	0.03725378767827775	C1orf63:SYT11:HSPA6:CSRP1:UBE2L6:ENO2:C1S:EMP1:ULK1:PCDH9:MOAP1:MFAP4:MBP:TGM2:SLPI:SYNGR1:FBLN1:TGFBR2:PDGFRB:IER3:CDKN1A:TNFAIP3:THBS2:GNG11:GEM
Computational_gene_sets	MODULE_206	153	13	0.0052971586209206115	0.03725378767827775	SYT11:CUL2:LINC01059:GRAMD1B:DDX25:KDELC1:COCH:ANKRD11:PLEKHM1:NCL:KLHL6:CCNO:FAM167A:ENY2
Computational_gene_sets	MODULE_199	57	7	0.005378504134975533	0.037518345917146395	TIE1:MPL:F3:GRK5:MERTK:PDGFRB:CDKN1A
Computational_gene_sets	MODULE_207	226	17	0.0054856282811564915	0.037957008590582826	MED8:MSTO1:BBOX1:AKAP3:FOXG1:TRPM1:AQR:MGA:SLC7A6OS:PRPSAP2:ST8SIA5:ZNF83:SLC4A5:IFT57:MAP3K13:DUS4L:GIMAP5
Computational_gene_sets	GNF2_PRDX2	31	5	0.0056249634517049445	0.03830332255208605	TAL1:DCAF11:KEL:CA2:UBAC1
Computational_gene_sets	GNF2_GLTSCR2	31	5	0.0056249634517049445	0.03830332255208605	FAU:RPL19:RPL38:ARHGEF18:RPL12
Computational_gene_sets	GCM_MAP4K4	172	14	0.005687325032656954	0.038423030535587925	MACF1:HIPK1:IPO7:IPO5:HERC2:TBC1D10B:SPAG9:PITPNC1:TTYH2:NAPG:FAM168B:VPS52:ZFAND3:YWHAG
Computational_gene_sets	GNF2_TST	104	10	0.005857633585642788	0.03926445012876181	HMGCS2:PKLR:APCS:HABP2:HPX:SDS:PCK2:ASGR2:CYP2D6:ASL
Computational_gene_sets	GCM_PTPRD	58	7	0.005922398855900021	0.039390838902032696	MOAP1:NIPA1:HERC2:ARPP19:RABEP1:TTYH2:PKP4
Computational_gene_sets	MODULE_52	425	27	0.00606513566858627	0.04002989541266938	CDC20:SEMA6C:FDPS:WEE1:EMP1:LGR5:DLGAP5:PPP4R1:CHAF1A:RND3:IGFBP2:PCNA:TGM2:GTSE1:TGFBR2:RBP1:IL8:CXCL1:OCLN:C5orf30:IER3:CDKN1A:THBS2:GNG11:MEST:CALD1:ST6GALNAC4
Computational_gene_sets	MODULE_332	45	6	0.0065112213312429135	0.04264601452065969	GTF3A:UCHL3:IPO5:ERCC5:TXNDC16:PUM2
Computational_gene_sets	MORF_CDC16	74	8	0.006580538483493933	0.04277350014271056	TARDBP:ERCC5:ANP32A:SAFB:DCTD:SKIV2L2:DIAPH1:MDC1
Computational_gene_sets	MORF_CAMK4	289	20	0.006899845582627819	0.04451178578868172	FAS:ABCC8:TENM4:ERC1:AKAP3:HOXC11:WBP4:SIX6:BRCA1:OR10H3:FSHR:CYP2D6:DAZL:NR1I2:ISL1:LECT2:HCRTR2:ABCB1:EXOC4:RB1CC1
Computational_gene_sets	MORF_ATF2	329	22	0.0070570995002047785	0.045186502769967905	LPHN2:FAS:ABCC8:TENM4:AKAP3:HOXC11:WBP4:SIX6:MGA:CHRNB4:BRCA1:SLC14A1:OR10H3:FSHR:DAZL:NR1I2:ISL1:LECT2:HCRTR2:ABCB1:EXOC4:RB1CC1
Computational_gene_sets	MODULE_279	141	12	0.007125077204284036	0.04528382400944965	TRIM33:PTHLH:PSEN1:IFI35:FAM49A:SNED1:SYNGR1:SRPRB:RPS18:PILRB:PRKAR2B:ESYT2

Computational_gene_sets	MODULE_259	46	6	0.007254577786021945	0.0455101814552997	TIE1:F3:GRK5:MERTK:PDGFRB:CDKN1A
Computational_gene_sets	MODULE_238	159	13	0.007266777225380021	0.0455101814552997	CSMD2:TMEM79:FCRLA:SELP:NAALADL1:PTPN6:KIAA0226L:COQ9:FOXP1:NEK11:PLAC8:BLK:DIRAS2
Computational_gene_sets	MODULE_419	33	5	0.007386228047446154	0.04592307003412174	MACF1:CORO1A:DSTN:CALD1:PIP
Computational_gene_sets	MORF_PRKAR1A	142	12	0.007523992067510768	0.04644305894909524	DAP3:SSR2:SNRPE:HSPA8:KDELR1:PUM2:ZNF638:RPN2:RTCB:VGLL4:AP2M1:PUF60
Computational_gene_sets	MORF_HDAC1	254	18	0.0079488058768915	0.04850373143695008	TARDBP:SCAMP3:SNRPE:UCP2:PHB2:SLC25A3:ANP32A:PKN1:MRPS12:XPO1:FAM168B:NCL:RPL14:DIAPH1:TTC1:VPS52:PUF60:UBAC1
Computational_gene_sets	MODULE_389	216	16	0.007970892928449838	0.04850373143695008	MED8:SYT11:BBOX1:LINC01059:GRAMD1B:AKAP3:FOXG1:TRPM1:AQR:SLC7A6OS:ANKRD11:PRPSAP2:ZNF83:SLC4A5:IFT57:DUS4L:FAM167A
KEGG	KEGG_MAPK_SIGNALING_PATHWAY	267	24	8.184002071467294e-05	0.015222243852929166	CDC42:NRAS:HSPA6:FAS:DUSP8:HSPA8:RAP1B:PLA2G4B:JMJD7-PLA2G4B:MAPK7:CACNB1:CACNG1:TAB1:MAPK8IP2:TGFBR2:CACNA2D2:MRAS:RASA2:MAP3K13:FGF12:PLA2G12A:PDGFRB:HSPB1:PPP3CC
KEGG	KEGG_INSULIN_SIGNALING_PATHWAY	137	15	0.00020990732410294704	0.015563898503871774	MTOR:NRAS:PKLR:PRKAB1:PCK2:RPS6KB1:INSR:LIPE:CBLC:PPP1CB:GSK3B:EIF4E:EIF4E1B:FLOT1:PRKAR2B
KEGG	KEGG_NEUROACTIVE_LIGAND_RECEPTOR_INTERACTION	270	23	0.0002510306210301899	0.015563898503871774	HTR6:TACR2:P2RX2:MLNR:CHRNB4:GRIN2A:TRPV1:GLP2R:SSTR2:S1PR5:PTGER1:GRIN2D:FPR2:FSHR:MC3R:MCHR1:NPY1R:NPY5R:PTGER4:HCRTR2:CNR1:CRHR2:S1PR3
KEGG	KEGG_RIBOSOME	87	11	0.00041844967938393343	0.017267126806145183	RPL22:FAU:RPL3L:RPL19:RPL38:RPL32:RPL14:RPL37:RPS18:RPL7:RPL12
KEGG	KEGG_B_CELL_RECEPTOR_SIGNALING_PATHWAY	75	10	0.0004930807721906339	0.017267126806145183	BCL10:VAV3:NRAS:PTPN6:NFATC3:NFKBIB:CD79A:GSK3B:PPP3CC:LYN
KEGG	KEGG_GLYCEROPHOSPHOLIPID_METABOLISM	77	10	0.0006098773001412507	0.017267126806145183	GPAM:LPCAT3:PLA2G4B:JMJD7-PLA2G4B:PLA2G15:DGKE:CDS2:CHKB:PLA2G12A:PPAP2A
KEGG	KEGG_LYSOSOME	121	13	0.0006498381056076144	0.017267126806145183	DNASE2B:CTSS:CTSK:PSAP:SMPD1:SLC11A2:PLA2G15:AP1M2:SLC11A1:NAGA:ARSA:HYAL1:PSAPL1
KEGG	KEGG_PATHOGENIC_ESCHERICHIA_COLI_INFECTION	56	8	0.001120905756410944	0.026061058836554445	CDC42:ARHGEF2:TUBB8:TUBB4A:NCL:OCLN:TUBB:TUBB4B
KEGG	KEGG_ENDOCYTOSIS	181	16	0.0014109527426765022	0.02915969001531438	CDC42:LDLRAP1:HSPA6:GRK5:HSPA8:SH3GL3:RABEP1:EPN2:SH3GL1:CBLC:CHMP4B:ARFGAP3:AP2M1:ARAP3:HLA-A:CHMP5
KEGG	KEGG_GLYCOSPHINGOLIPID_BIOSYNTHESIS_GANGLIO_SERIES	15	4	0.0019512189121662113	0.03629267176629153	ST8SIA5:B3GALT4:RP11-203J24.9:ST6GALNAC6:ST6GALNAC4
KEGG	KEGG_CHEMOKINE_SIGNALING_PATHWAY	189	16	0.002199944653736403	0.03719906414499735	CDC42:VAV3:NRAS:GRK5:RAP1B:CCL17:NFKBIB:GSK3A:GNAI2:GSK3B:IL8:CXCL6:PF4V1:CXCL1:GNG11:LYN
KEGG	KEGG_STEROID_BIOSYNTHESIS	17	4	0.003203992568515157	0.049661884811984934	SOAT1:TM7SF2:LSS:CEL
microRNA_targets	GCACTTT_MIR175P_MIR20A_MIR106A_MIR106B_MIR20B_MIR519D	596	56	4.335315509194943e-10	9.581047275320823e-08	NBL1:C1orf63:LDLRAP1:ZNF362:TAL1:SSX2IP:F3:OLFM3:SIKE1:ARHGEF11:NHLH1:ZBTB41:REEP3:DUSP8:WEE1:PTHLH:ULK1:FNDC3A:KPNA3:C14orf28:FRMD6:MAP3K9:NIPA1:PKD1:RABEP1:DERL2:PLEKHM1:NPLOC4:ZNF236:ARHGEF18:CC2D1A:CIC:

						MYCN:OSR1:MMP24:ZNFX1:SLC2A4RG:C2CD2:PPARA:TGFBR2:TUSC2:LRIG1:XRN1:FNDC3B:SLC22A23:CDKN1A:L3MBTL3:MAP7:HECA:ITGB8:JAZF1:CALD1:RB1CC1:PTPN3:PBX3:FAM102A
microRNA_targets	ACCAAAG_MIR9	500	49	1.3293701952018135e-09	1.4689540656980037e-07	ICMT:ACOT7:LDLRAP1:ZNF362:PTBP2:VAV3:HIPK1:PRUNE:ARHGEF2:LMNA:ZBTB41:GPAM:UBASH3B:OPCML:HOXC13:LHFP:KCTD12:FOXG1:FAM179B:FRMD6:MESDC1:CALB2:HNF1B:ADAM11:KCNJ2:DNAJB1:NCOA1:BCL11A:MTHFD2:PANK2:C2CD2:PPARA:VGLL4:FOXP1:ZNF654:XRN1:FNDC3B:AP2M1:FGF12:MRFAP1:EIF4E:DIAPH1:PDGFRB:CPEB4:MAP7:HOXA11:BCAP29:ESYT2:SCRIB
microRNA_targets	TTTTGAG_MIR373	226	30	2.3308776348226434e-09	1.7170798576526805e-07	TARDBP:C1orf63:KTI12:TCEANC2:SSX2IP:TRIM33:CSDE1:PRUNE:ALDH18A1:CCER1:KPNA3:KCTD12:IPO5:NPLOC4:ZNF236:MBP:KDELR1:MYCN:ALK:XPO1:PKP4:ARMC8:MRFAP1:SLC4A4:ISL1:NDFIP1:ZFAND3:PTP4A1:IPPK:PALM2
microRNA_targets	TTTGCAC_MIR19A_MIR19B	515	49	3.5318867287540584e-09	1.9513674176366171e-07	SPEN:MACF1:RIMKLA:TXNDC12:BTF3L4:F3:HIPK1:TRIM33:SYT11:KIAA0907:PHLDA3:B3GALNT2:ZMYND11:RAB18:REEP3:PSAP:WEE1:UCP3:SOX5:RAP1B:FNDC3A:KPNA3:FAM179B:NIPA1:ARPP19:GRIN2A:ESRP2:EPN2:KCNJ2:CC2D1A:RFX1:DNAJB1:MYCN:RBMS1:CHMP4B:PPARA:VGLL4:TGFBR2:RASSF1:LRIG1:FOXP1:ATP2C1:ARMC8:C5orf30:CPEB4:PTP4A1:L3MBTL3:ESR1:SEPT7
microRNA_targets	CAGTATT_MIR200B_MIR200C_MIR429	465	44	2.5842968957772332e-08	1.142259227933537e-06	TARDBP:BTF3L4:HIPK1:TRIM33:SLC16A12:HECTD2:SYVN1:PTHLH:RAP1B:LHFP:LCP1:LMO7:FOXG1:FBXO33:FAM179B:NIPA1:GOLGA8G:PKD1:SIAH1:HNF1B:NBR1:RPS6KB1:KCNJ2:SLC14A1:MYCN:PUM2:AFF3:RND3:COPS8:USP25:FNDC3B:OCLN:TUBB:JAZF1:YWHAG:NYAP1:PRKAR2B:BCAP29:CEP41:RDH10:GEM:UBQLN1:KLF4:PALM2
microRNA_targets	TGAATGT_MIR181A_MIR181B_MIR181C_MIR181D	486	45	3.432473621738352e-08	1.2642944506736263e-06	TARDBP:C1orf63:MED8:SSX2IP:ZBTB41:PSAP:CDON:SOX5:RAP1B:SLC25A3:FNDC3A:IPO5:FBXO33:FAM179B:KCNK10:GOLGA8G:ADAM11:NPEPPS:KPNB1:RPS6KB1:PPP1CB:BCL11A:CREB1:PER2:CACNA2D2:FOXP1:CGGBP1:FNDC3B:SENP2:KLHL2:NIPBL:CPEB4:CNR1:HECA:ESR1:SYNE1:ITGB8:HOXA11:JAZF1:YWHAG:FAM3C:NSMAF:PALM2:PBX3:SLC25A25
microRNA_targets	AAGCACA_MIR218	400	37	5.669838630848443e-07	1.79004905345358e-05	OLFM3:CSDE1:HCN3:ZBTB41:LARP4B:ARL5B:HECTD2:GPAM:SYT13:SOX5:LHFP:NUMB:ARPP19:RABEP1:VAT1:SPAG9:RPS6KB1:ZNF236:CHST8:PUM2:PPP1CB:PKP4:MMP24:SYNGR1:GNAI2:ZNF654:NPY1R:SLC12A2:PTP4A1:L3MBTL3:HOXA10:PRKAR2B:FAM3C:ZMAT4:CA2:DIRAS2:PALM2
microRNA_targets	CAGTGTT_MIR141_MIR200A	309	30	2.4369434014071794e-06	6.555652084112813e-05	ACOT7:HIPK1:RBM7:SOX5:HOXC13:SLC25A3:KPNA3:PCDH9:FRMD6:PSEN1:TCF12:SIAH1:SPAG9:RNFT1:KCNJ2:TTYH2:PHLPP1:ARHGEF18:FAM168B:RBMS1:MYRIP:FOXP1:PTP4A1:CNR1:HOXA11:JAZF1:SEPT7:YWHAG:NUDCD1:PAPPA
microRNA_targets	AGCACTT_MIR93_MIR302A_MIR302B_MIR302C_MIR302D_MIR372_MIR373_MIR520E_MIR520A_MIR526B_MIR520B_MIR520C_MIR520D	344	32	2.8540242453023872e-06	6.555652084112813e-05	TARDBP:ZNF362:TAL1:SSX2IP:OLFM3:ZBTB41:ZMYND11:REEP3:WEE1:OPCML:ULK1:FNDC3A:RABEP1:DERL2:PLEKHM1:ARHGEF18:CC2D1A:MYCN:BCL11A:MMP24:ZNFX1:C2CD2:TUSC2:ZNF654:FNDC3B:SLC22A23:ESR1:ITGB8:JAZF1:NEUROD6:RB1CC1:PBX3
microRNA_targets	AGGTGCA_MIR500	96	15	2.96635840910082e-06	6.555652084112813e-05	HIPK1:CIT:KPNA3:FOXG1:KCNK10:CACNB1:PPP4R1:ZNF236:NCOA1:RBMS1:CREB1:SLC25A26:FNDC3B:C5orf30:PPP3CC
microRNA_targets	TGCTGCT_MIR15A_MIR16_MIR15B_MIR195_MIR424_MIR497	603	45	1.2359923492854038e-05	0.00024832209926552206	SPEN:CDC42:ZNF362:LPHN2:CSDE1:PHLDA3:CUL2:C10orf54:WEE1:ARL2:PTHLH:HOXC11:PCDH9:ABHD13:RASL12:ARMC5:SIAH1:N4BP1:NFATC3:PLA2G15:CACNB1:VAT1:HELZ:KCNJ2:PEX13:TMEM87B:CDS2:USP25:LRIG1:MRAS:GK5:KCNAB1:SLC4A4:PPAP2A:SLC12A2:FCHSD1:STK19:MAP7:SYNE1:HOXA10:BCAP29:LY6E:PTPN3:PAPPA:PBX3
microRNA_targets	CACTGTG_MIR128A_MIR128B	338	30	1.441375932097792e-05	0.00026033290812700773	ACOT11:ARHGEF11:GPAM:IPO7:WEE1:LETMD1:RAP1B:SLC25A3:ULK1:FBXO33:KCNK10:KIAA1199:SMG1:RPS6KB1:C17orf70:BCL11A:RND3:ARMC8:KCNAB1:STIM2:NIPBL:ISL1:MGAT1:RGL2:CNR1:HOXA13:NEUROD6:DIRAS2:TSTD2:KLF4
microRNA_targets	CATTTCA_MIR203	289	27	1.531370047805928e-05	0.00026033290812700773	TARDBP:SPEN:VAV3:HIPK1:PHLDA3:ZMYND11:CSRNP2:NUAK1:GPR180:FOXG1:TCF12:VAT1:KPNB1:PPP1CB:IGFBP5:TUSC2:SLC4A4:EIF4E:ISL1:KIF2A:C5orf30:ZNF608:SLC12A2:CPEB4:EDN1:PTP4A1:PTPN3
microRNA_targets	TGCCTTA_MIR124A	555	42	1.714699887856542e-05	0.000270677625154497	ICMT:MACF1:PTBP2:HIPK1:CERS2:SEMA6C:LMNA:TOR3A:LARP4B:CDON:LPCAT3:KCTD12:PSEN1:KCNK10:EPN2:VAT1:RPS6KB1:KCNJ2:NPLOC4:TCF3:PDE4A:RFX1:ERF:RBMS1:CREB1:TPD52L2:OSBP2:PPARA:LMF2:MYRIP:CACNA2D2:LRIG1:RAB43:ZNF608:DIAPH1:NDFIP1:CPNE5:MAP7:JAZF1:ESYT2:KLF4:PALM2
microRNA_targets	TTGCCAA_MIR182	329	29	2.2598351110214158e-05	0.0003329490396904886	LPHN2:KIAA0907:FAM171A1:PC:PTHLH:NUAK1:KPNA3:ABHD13:NUMB:GOLGA8G:PDXDC1:N4BP1:TMEM145:KDELR1:BCL11A:C2CD2:TMEM115:RAB6B:FNDC3B:ISL1:FLOT1:PPIL1:THBS2:HOXA10:JAZF1:SEPT7:YWHAG:EXOC4:KHDRBS3

microRNA_targets	ACTGTGA_MIR27A_MIR27B	475	37	2.884556027329701e-05	0.0003984293012749149	CDC42:MFSD2A:ACOT11:C1orf52:VAV3:GPAM:WEE1:APAF1:CIT:KPNA3:FBXO33:KIAA1199:MESDC1:N4BP1:NPEPPS:RPS6KB1:RNFT1:INSR:NCOA1:RND3:CREB1:EIF6:PPARA:ARMC8:STIM2:PTGER4:ISL1:ADAM19:CPEB4:RGL2:HOXA10:HOXA13:NEUROD6:MEPCE:PTPN3:PALM2:PAPPA
microRNA_targets	ATTCTTT_MIR186	270	25	3.615719792590539e-05	0.0004543480664158418	ICMT:TARDBP:CDC42:LPHN2:CSDE1:ZBTB41:SLC25A3:FNDC3A:FBXO33:PRPF39:SMPD3:EPN2:RPS6KB1:PPP1CB:TGFBR2:CD200R1:FNDC3B:EIF4E:ZNF608:CPEB4:ZFAND3:SEPT7:RB1CC1:CALB1:PAPPA
microRNA_targets	ACTTTAT_MIR1425P	287	26	3.700572486644865e-05	0.0004543480664158418	SPEN:HIPK1:CUL2:HSPA8:SOX5:ULK1:FNDC3A:UCHL3:C14orf28:TCF12:CIC:MYCN:OSR1:MMP24:C2CD2:CELSR1:TGFBR2:RAB6B:FNDC3B:SLC4A4:CPEB4:SLC22A23:ZFAND3:HOXA13:PAPPA:PBX3
microRNA_targets	GTACTGT_MIR101	256	24	4.208221039710549e-05	0.0004894825525137007	TAL1:C1orf52:CERS2:ZBTB41:EMP1:RAP1B:FAM179B:UBR7:N4BP1:ANKRD11:KPNB1:MPPE1:PDE4A:MYCN:PUM2:IGFBP5:MYRIP:STIM2:TMEM161B:NDFIP1:ZFAND3:MEPCE:KHDRBS3:PALM2
microRNA_targets	CTTTGTA_MIR524	435	34	5.5813768017808166e-05	0.0006167421365967802	SPEN:CDC42:LPHN2:VAV3:LAMTOR5:HIPK1:CSDE1:KIAA0907:CREM:RCE1:HOXC13:RAP1B:PRKAB1:PCDH9:GPR180:C14orf28:ARPP19:HNF1B:PUM2:NCOA1:PPP1CB:CACNA2D2:FOXP1:ARMC8:EIF4E:CDH9:C5orf30:CPEB4:CNR1:L3MBTL3:ARL4A:JAZF1:DIRAS2:PALM2
microRNA_targets	TGTGTGA_MIR377	199	20	6.834118037378431e-05	0.0006841288547522529	MACF1:CDON:SLC11A2:IPO5:NPEPPS:KPNB1:RPS6KB1:NCOA1:C21orf91:CACNA2D2:GTPBP8:ARMC8:MRFAP1:EIF4E:KLHL2:KIF2A:MARCH3:FCHSD1:CPEB4:JAZF1
microRNA_targets	AAGCCAT_MIR135A_MIR135B	333	28	7.116131528919515e-05	0.0006841288547522529	LDLRAD2:ZNF362:EXTL2:ARHGEF2:SSR2:NUCKS1:GRK5:ZNF143:LCP1:KPNA3:KCTD12:MAPKBP1:SIAH1:RPS6KB1:PITPNC1:RALBP1:ZNF236:PDE4A:CIC:NCOA1:BCL11A:CHMP4B:VGLL4:MRAS:HOXA10:YWHAG:DIRAS2:KLF4
microRNA_targets	GTGCCTT_MIR506	727	49	7.11989305850761e-05	0.0006841288547522529	LDLRAP1:TAL1:PTBP2:HIPK1:NRAS:CERS2:NHLH1:TOR3A:SFMBT2:FAM171A1:ARL5B:HECTD2:SFRP5:GPAM:DGAT2:CLMP:KPNA3:PCDH9:PSEN1:KCNK10:UBR7:NIPA1:N4BP1:SMPD3:MAPK7:VAT1:RALBP1:CCDC130:RFX1:BCL11A:CTDSP1:TPD52L2:LMF2:MYRIP:GNAI2:LRIG1:XRN1:CDH9:KIF2A:SLC12A2:ADAM19:FLOT1:ZFAND3:FGFR1OP:TSC22D4:ESYT2:RB1CC1:NSMAF:RDH10
microRNA_targets	AACTGGA_MIR145	234	22	8.271459122565269e-05	0.0007616635275362185	PTP4A2:SCAMP3:LARP4B:HOXC11:NUAK1:FNDC3A:FKBP3:GOLGA8G:MYO5A:RTKN:SCN3A:CHKB:TGFBR2:RASA2:XRN1:FNDC3B:SENP2:SLC4A4:ADAM19:CPEB4:LRRC16A:SLC25A25
microRNA_targets	TATTATA_MIR374	285	25	8.706592800558513e-05	0.0007696628035693726	CSMD2:LPHN2:LARP4B:DUSP8:NUAK1:FOXG1:NUMB:HERC2:TCF12:KIAA1199:ZNF236:NCOA1:BOLA3:SCN5A:FOXP1:DZIP1L:FNDC3B:NIPBL:SKIV2L2:TMEM161B:CPEB4:SYNE1:HOXA10:HOXA11:PAPPA
microRNA_targets	GTGCAAT_MIR25_MIR32_MIR92_MIR363_MIR367	307	26	0.00011292909154568938	0.0009598972781383597	RAP1B:GPR180:FOXG1:KCNK10:MOAP1:HERC2:GOLGA8G:NOMO1:HNF1B:PITPNC1:RFX1:CIC:BCL11A:SCN3A:CREB1:PER2:FNDC3B:PTGER4:C5orf30:ADAM19:CPEB4:WRNIP1:CEP41:CREB3L2:NSMAF:KLF4
microRNA_targets	ATGTTAA_MIR302C	241	22	0.00012723457357125621	0.0010414385466388008	PTP4A2:SSX2IP:HORMAD1:CREM:RAP1B:MTMR6:PCDH9:FOXG1:FBXO33:NUMB:FAM49A:BCL11A:PPARA:FOXP1:CGGBP1:ATP2C1:MRFAP1:ARL4A:HOXA13:KHDRBS3:UBQLN1:PTPN3
microRNA_targets	ACTGTAG_MIR139	123	14	0.00022482627475624867	0.00177452166861182	CDC42:SSX2IP:HIPK1:SOX5:SLC25A3:LRFN5:TCF12:PDE4A:DNAJC5:CACNA2D2:FOXP1:CPEB4:ZFAND3:YWHAG
microRNA_targets	GTGCCAA_MIR96	304	25	0.0002382126841360207	0.0018153449377262265	MTOR:SPEN:DDAH1:DENND2C:ZBTB41:SOX5:LCP1:ARPP19:DERL2:CACNB1:VAT1:KPNB1:ALK:CTDSP1:CELSR1:MYRIP:CACNA2D2:LRIG1:NIPBL:CPEB4:PTP4A1:JAZF1:CREB3L2:PAPPA:SLC25A25
microRNA_targets	GTGCCAT_MIR183	172	17	0.00028388829875920714	0.0020913104675261596	PSEN2:HECTD2:MTMR6:FRMD6:KCNK10:ARPP19:TCF12:SMPD3:ARHGEF18:KCNJ14:PKP4:CTDSP1:FOXP1:SNCB:SLC22A23:L3MBTL3:PALM2
microRNA_targets	CCTGCTG_MIR214	241	21	0.00033773462116018564	0.0024077210089161623	NBL1:PHC2:ZBTB41:IPO7:ARL2:SLC25A3:KPNA3:TBC1D10B:ARMC5:KCNJ14:BCL11A:PANK2:NDUFAF5:CHMP4B:GNAI2:IPO11:CPEB4:ZFAND3:JAZF1:LY6E:PAPPA
microRNA_targets	TGGTGCT_MIR29A_MIR29B_MIR29C	519	36	0.00036220840041567136	0.00250150176537073	SPEN:CDC42:LDLRAP1:ZNF362:ARNT:SETDB1:LYSMD1:CLK2:ZBTB41:LARP4B:GPAM:PTHLH:CSRNP2:KDELC1:MAPKBP1:ARPP19:KIAA1199:SMPD3:TTYH2:C17orf70:POU2F2:MYCN:BCL11A:RND3:MMP24:RAB6B:ARMC8:ISL1:PDGFRB:ADAM19:LSM11:PTP4A1:CNR1:SYNE1:FAM167A:KLF4
microRNA_targets	CTACCTC_LET7A_LET7B_LET7C_LET7D_LET7E_LET7F_MIR98_LET7G_LET7I	390	29	0.00043328903923031236	0.0029017235657545163	ZNF362:MED8:NRAS:ADAM15:SYT11:HOXC11:FNDC3A:C14orf28:NIPA1:ARPP19:PLA2G15:SNAI3:TBKBP1:RNFT1:DCAF15:NCOA1:TTLL4:SCN5A:TUSC2:LRIG1:CD200R1:NPHP3:DVL3:SLC4A4:LSM11:RDH10:SLC35D2:PAPPA:PBX3

microRNA_targets	CTGAGCC_MIR24	232	20	0.000530044958233765	0.0034452922285194724	SZT2:TRIM33:CLK2:SEMA4A:PSAP:RAP1B:TNFRSF19:KPNA3:DCAF11:ACAN:MAPK7:HNF1B:CACNB1:IGFBP5:PER2:MCHR1:PTGER4:TMEM161B:ADAM19:RGL2
microRNA_targets	AAGCACT_MIR520F	238	20	0.0007309202818805891	0.004615239494160292	TARDBP:OLFM3:UHMK1:ZBTB41:OPCML:LETMD1:FNDC3A:RABEP1:DERL2:KPNB1:CC2D1A:PANK2:MMP24:ZNFX1:ZNF654:SLC22A23:ESR1:JAZF1:RB1CC1:PBX3
microRNA_targets	CACTTTG_MIR520G_MIR520H	239	20	0.0007700977085949294	0.004727544266652206	ZNF362:MACF1:LAMTOR5:TRIM33:CKS1B:NHLH1:FNDC3A:KPNA3:MAP3K9:PLEKHM1:TBKBP1:CIC:MYCN:SLC2A4RG:ARMC8:EIF4E:CKS1B:CDKN1A:ZFAND3:CNR1:JAZF1
microRNA_targets	TTTGTAG_MIR520D	337	25	0.0010742555124540272	0.006337584405480184	TAL1:RCE1:BIVM:COCH:PRPF39:UBR7:RPS6KB1:PHLPP1:RFX1:KDELR1:PPP1CB:SCN3A:VGLL4:SCN5A:TUSC2:C4orf17:NIPBL:NDFIP1:CPEB4:PTP4A1:ARL4A:JAZF1:TPST1:CNTLN:DIRAS2
microRNA_targets	GACTGTT_MIR212_MIR132	160	15	0.0010897203955124298	0.006337584405480184	PTBP2:CSDE1:ARHGEF11:SOX5:ANP32A:BRCA1:PSMD12:KCNJ2:SCN3A:ISL1:LSM11:YWHAG:MEPCE:FAM167A:PALM2
microRNA_targets	TGCACTT_MIR519C_MIR519B_MIR519A	447	30	0.0017808788817423764	0.010091646996540133	NBL1:C1orf63:BTF3L4:ARHGEF11:NHLH1:LARP4B:DUSP8:WEE1:SOX5:PTHLH:KPNA3:FOXG1:C14orf28:FRMD6:MAP3K9:NIPA1:N4BP1:CACNB1:ZNF236:CIC:ZNFX1:SLC2A4RG:TGFBR2:LRIG1:ATP2C1:L3MBTL3:MAP7:HECA:CALD1:PUF60
microRNA_targets	GTCTTCC_MIR7	169	15	0.0018823612277417115	0.010400045783272957	SLC25A15:MAP3K9:TCF12:SMG1:TBC1D10B:ADAM11:RPS6KB1:KCNJ2:FAM168B:CGGBP1:SLC4A4:IPO11:SRRM3:KLF4:PBX3
microRNA_targets	GGTGAAG_MIR412	62	8	0.0021888298549811126	0.011798326779288435	RNF207:CSDE1:CLK2:BRCA1:PPP1CB:PKP4:ARMC8:EIF4E
microRNA_targets	CACCAGC_MIR138	225	18	0.002264447323854241	0.011915306156471125	MACF1:CLK2:ZMYND11:CLMP:ERC1:CIT:SMG1:NCOA1:BCL11A:AFF3:CPT1B:CHKB:CPEB4:SNCB:PTP4A1:L3MBTL3:JAZF1:PAPPA
microRNA_targets	GTGACTT_MIR224	156	14	0.002369717275815113	0.012179244603607906	CDC42:HABP2:NUAK1:CIT:KCTD12:PHLPP1:PUM2:AFF3:RND3:SYNGR1:CGGBP1:AP2M1:SLC4A4:UBQLN1
microRNA_targets	ACATTCC_MIR1_MIR206	301	22	0.0024879390939457294	0.012496239540045596	CDC42:PHC2:CREM:FNDC3A:FBXO33:MAPKBP1:SLC7A6:CYB5B:ZNF236:ARHGEF18:PUM2:NCL:DNAJC5:VGLL4:FOXP1:FNDC3B:EIF4E:KIF2A:EDN1:BET1:UBQLN1:SLC25A25
microRNA_targets	TGTTTAC_MIR30A5P_MIR30C_MIR30D_MIR30B_MIR30E5P	581	36	0.0026120577992638826	0.01282810608082929	SPEN:SSX2IP:EXTL2:ZBTB41:PSEN2:CUL2:REEP3:TLL2:RAP1B:CIT:KIAA0226L:FNDC3A:KPNA3:FOXG1:FKBP3:FRMD6:HERC2:RASL12:TBC1D10B:VAT1:FAM49A:XPO1:SCN3A:PER2:GNAI2:STIM2:ANKRA2:ZNF608:ADAM19:CPEB4:PTP4A1:HOXA11:NEUROD6:SEPT7:CEP41:PALM2
microRNA_targets	AACATTC_MIR4093P	144	13	0.0031658434047389986	0.014872659991758328	PCDH9:KCTD12:FBXO33:RPS6KB1:ZNF236:ARHGEF18:PUM2:FOXP1:NIPBL:ISL1:EDN1:BET1:PALM2
microRNA_targets	CAGTCAC_MIR134	52	7	0.0031995499959976835	0.014872659991758328	OPCML:KPNA3:MYCN:AFF3:VGLL4:CDH9:TAPBP
microRNA_targets	CAAGGAT_MIR362	66	8	0.0032597460470094016	0.014872659991758328	GPR180:SMG1:NPEPPS:BCL11A:TMEM115:DVL3:ISL1:CPEB4
microRNA_targets	GTGCAAA_MIR507	128	12	0.003297558097720172	0.014872659991758328	AGAP4:AGAP8:HOXC11:PCDH9:FAM179B:RPS6KB1:NCOA1:PPP1CB:BCL11A:SCN3A:DAZL:NDFIP1:CPEB4
microRNA_targets	CTCAGGG_MIR125B_MIR125A	329	23	0.00350949324255799	0.015163263696752634	PHC2:CSDE1:SYVN1:RBM7:SLC25A15:ZFYVE1:NIPA1:GRIN2A:SMG1:SLC7A6:CACNB1:ADAM11:ZNF236:MEGF8:DCTN1:FNDC3B:DVL3:ATP10D:SLC4A4:TMEM161B:FCHSD1:NRM:SRRM3
microRNA_targets	ACACTAC_MIR1423P	129	12	0.0035143058202303954	0.015163263696752634	TARDBP:ZBTB41:EHF:CRTAM:FNDC3A:GTF2A1:SMG1:ANKRD11:XPO1:MRFAP1:RGL2:S1PR3
microRNA_targets	CTTTGCA_MIR527	235	18	0.003604558689906375	0.015163263696752634	TARDBP:SPEN:PHLDA3:ULK1:FNDC3A:KPNA3:ARPP19:TCF12:ESRP2:RPS6KB1:RFX1:MYCN:MMP24:CPEB4:L3MBTL3:HOXA13:CALD1:CREB3L2
microRNA_targets	CTACTGT_MIR199A	181	15	0.0036364388051035733	0.015163263696752634	MTOR:CDC42:SEMA6C:HECTD2:RBM25:GOLGA8G:SLC7A6:ZNF638:FOXP1:ZNF654:FGF12:SLC4A4:TMEM161B:CPEB4:ITGB8
microRNA_targets	TAGCTTT_MIR9	236	18	0.003768738675287587	0.01540938149204724	TARDBP:ZNF362:RIMKLA:HIPK1:TRIM33:ZMYND11:TLL2:CIT:FBXO33:KPNB1:AFF3:GNAI2:GK5:CPEB4:MAP7:JAZF1:CEP41:CALD1

microRNA_targets	CAGCTTT_MIR320	255	19	0.003834913945984607	0.01540938149204724	HIPK1:RAB18:HECTD2:IPO7:ULK1:ABHD13:ARPP19:N4BP1:METTL16:HELZ:XPO1:TPD52L2:USP25:TRMU:RAD18:WRNIP1:CALD1:PALM2:PBX3
microRNA_targets	GTTTGTT_MIR495	256	19	0.004000365605748551	0.015787157122686246	TARDBP:ZNF362:SSX2IP:HIPK1:SOX5:CYB5B:KCNJ2:NCOA1:BCL11A:ANAPC1:RND3:PER2:ARMC8:ZFAND3:CNR1:THBS2:NEUROD6:SEPT7:S1PR3
microRNA_targets	TAATAAT_MIR126	221	17	0.004395975277290372	0.017044044496160916	LDLRAD2:CUL2:ATN1:UHRF1BP1L:NUAK1:FNDC3A:MIA2:CTAGE5:TCF12:SPAG9:PHLPP1:PLEKHB2:DBR1:DVL3:MARCH3:NDFIP1:HECA:TSC22D4
microRNA_targets	AAGTCCA_MIR422B_MIR422A	71	8	0.005124571964283751	0.019526386277701875	FMOD:HECTD2:RAP1B:UHRF1BP1L:FOXG1:GNAI2:FNDC3B:EDN1
microRNA_targets	TACTTGA_MIR26A_MIR26B	301	21	0.005239094435695215	0.01962440458116343	SSX2IP:ULK1:PCDH9:CPSF2:ARPP19:STAC2:PITPNC1:KCNJ2:MOB1A:USP25:CELSR1:GSK3B:MRAS:SLC4A4:ZNF608:SLC12A2:ADAM19:LSM11:SLC22A23:LRRC16A:PTP4A1
microRNA_targets	GTGTTGA_MIR505	104	10	0.005857633585642788	0.02123693698871796	HIPK1:ZMYND11:SLC25A3:PCDH9:EPN2:KCNJ2:MYCN:CREB1:CGGBP1:SLC12A2
microRNA_targets	ATGTCAC_MIR489	88	9	0.005861778987836179	0.02123693698871796	IPO5:FAM211A:NCOA1:PPP1CB:BCL11A:XPO1:SCN3A:SOX30:ITGB8
microRNA_targets	ATATGCA_MIR448	210	16	0.006141770704987958	0.0218924407387474	C1orf63:HECTD2:KPNA3:PCDH9:FBXO33:BCL11A:SCN5A:ARMC8:PCDHGA12:PCDHGC3:PCDHGC4:PCDHGC5:WRNIP1:HOXA11:PRKAR2B:PAPPA
microRNA_targets	ATGTAGC_MIR221_MIR222	140	12	0.006742982084067288	0.023653953025061437	HIPK1:ARNT:HECTD2:FNDC3A:TCF12:MESDC1:ADAM11:VGLL4:GNAI2:SNCB:ESR1:YWHAG
microRNA_targets	GTGTCAA_MIR514	60	7	0.007131963155969782	0.024256694588566854	TAL1:SYT11:ZBTB41:TCF12:VAT1:SCN3A:PPIL1
microRNA_targets	GAGCCTG_MIR484	107	10	0.0071343219378137805	0.024256694588566854	PHC2:HIPK1:TRIM33:BTG4:ZFYVE1:BCL11A:PTGER4:IPO11:CEP41:PALM2
microRNA_targets	TCCAGAT_MIR5165P	108	10	0.007604491855699829	0.02546352575923731	C1orf63:SMG5:C10orf131:CC2D2B:RAP1B:CLLU1OS:FBXO33:SPAG9:RALBP1:NCOA1:PBX3
microRNA_targets	TGCAAAC_MIR452	109	10	0.00809819570832634	0.026319136052060602	CLK2:PCDH9:FAM179B:RPS6KB1:RFX1:MFF:ATP2C1:DIAPH1:SEPT7:CNTLN
microRNA_targets	AAACCAC_MIR140	109	10	0.00809819570832634	0.026319136052060602	UBASH3B:CIT:SIAH1:TTYH2:BCL11A:IGFBP5:DAZL:ZNF654:ZNF608:MGAT1
microRNA_targets	GAGCTGG_MIR337	162	13	0.008447990083320148	0.027058055194402214	ACOT7:MSTO1:ZMYND11:OPCML:MAP3K9:UBR7:KIAA1199:SMG1:FAM211A:ARAP3:PDGFRB:ZFAND3:BCAP29
microRNA_targets	ACTGCAG_MIR173P	110	10	0.008616178876588866	0.027202507596087708	CSDE1:UHMK1:CTR9:KPNA3:FAM168B:VGLL4:FOXP1:ARMC8:FGFR1OP:YWHAG
microRNA_targets	GGGACCA_MIR133A_MIR133B	200	15	0.008966244350909267	0.02790901410635138	CDC42:PTBP2:CERS2:ARHGEF2:RCE1:TPI1:FOXG1:NIPA1:CHKB:PCDHGA12:PCDHGC3:PCDHGC4:PCDHGC5:PAPPA:SLC25A25
microRNA_targets	ATGCTGG_MIR338	114	10	0.010945927190554174	0.033597915404339894	TRIM33:ARNT:C10orf131:CC2D2B:NRL:FBXO33:LINS:PPP4R1:MYCN:NDUFAF5:ESYT2
microRNA_targets	CTCCAAG_MIR432	83	8	0.012841748023696076	0.038877072784066206	FAM171A1:ARL5B:PDE1B:PITPNC1:PDE4A:CDPF1:CPT1B:CHKB
microRNA_targets	ATAAGCT_MIR21	118	10	0.013724424137344671	0.04098780722098882	YAP1:SOX5:CCER1:RNFT1:OSR1:PER2:PPARA:DAZL:TGFBR2:ZNF654
microRNA_targets	ATAGGAA_MIR202	102	9	0.014811155196772369	0.043643537313155914	TARDBP:ZNF362:TRIM33:TCF12:ESRP2:BCL11A:TGFBR2:ZFAND3:ESR1
microRNA_targets	CTCTGGA_MIR520A_MIR525	157	12	0.015867932312153558	0.04614227685507811	TRIM33:UBASH3B:USP5:SLC11A2:SMG1:HNF1B:PUM2:SLPI:CCNO:SEPT7:GFRA2:PBX3

microRNA_targets	AATGTGA_MIR23A_MIR23B	420	25	0.01701338757900404	0.048830631882596016	LPHN2:FAS:GRK5:PRKRIR:HOXC11:NUAK1:IPO5:RBM25:PUM2:NCOA1:PPP1CB:BCL11A:PKP4:TGFBR2:TUSC2:LRIG1:SLC4A4:PTGER4:ADAM19:CPEB4:HOXA11:YWHAG:BET1:CA2:NACC2
TF_targets	TGGAAA_NFAT_Q4_01	1924	142	1.0951109944004451e-14	6.680177065842715e-12	ANGPTL7:PTP4A2:EBNA1BP2:WDR65:TMEM125:TAL1:FOXE3:CDCP2:DNASE2B:EXTL2:NRAS:PHGDH:CTSK:CKS1B:RIT1:ARHGEF2:FCRLA:UHMK1:SOAT1:FMOD:NSUN6:ARL5B-AS1:CREM:HECTD2:GRK5:SMPD1:HPX:ZNF143:PEX16:SLC43A1:SNX15:YAP1:POU2AF1:UBASH3B:OPCML:KCNA1:USP5:TPI1:C1S:EMP1:SOX5:RASSF8:HOXC11:PDE1B:DHX37:TNFRSF19:WBP4:DCAF11:FOXG1:FRMD6:SIX6:NUMB:TCF12:RASL12:ANP32A:C15orf26:N4BP1:POLR2C:SMPD3:TRPV1:FAM211A:HNF1B:CACNB1:STAC2:RND2:NPEPPS:SPAG9:UTP18:TRIM25:PITPNC1:KCNJ2:UBALD2:NAPG:IMPA2:PTGER1:TECR:GRWD1:KCNJ14:GREB1:BCL11A:XPO1:RTKN:AFF3:IL18R1:PKP4:SCN3A:CREB1:IGFBP5:DAW1:NCL:PER2:DSTN:ZNFX1:UCKL1:USP25:C21orf91:WBP2NL:CYP2D6:PPARA:TYMP:VGLL4:HYAL3:NAT6:HYAL2:FAM208A:FOXP1:ZNF654:CD200R1:ATP2C1:ACKR4:FGF12:AREG:AREGB:CDH6:CKS1B:PCDHGC3:DIAPH1:FCHSD1:ARAP3:CPEB4:FLOT1:IER3:RGL2:CDKN1A:ZFAND3:CNR1:MAP7:AIG1:ESR1:ITGB8:HOXA10:HOXA11:HOXA13:SEPT7:MEPCE:GPR22:CEP41:KLF14:CALD1:ESYT2:CALB1:LRRC6:KIAA0020:ADAMTSL1:PALM2
TF_targets	CTTTGA_LEF1_Q2	1244	99	2.2060450111480745e-12	6.728437284001627e-10	ELOVL1:TCEANC2:LPHN2:C1orf52:DPH5:TRIM33:GOLPH3L:ARNT:FAM63A:PRUNE:SCNM1:LYSMD1:SCAMP3:SOAT1:CFHR5:ZBTB41:CREM:ZNF488:TLL2:HABP2:GRK5:UCP2:PRKRIR:POU2AF1:OPCML:USP5:SPSB2:EMG1:PHB2:LPCAT3:C1S:EMP1:SOX5:PTHLH:HOXC11:DAO:TNFRSF19:LMO7:TNFSF13B:PCK2:PAPLN:GTF2A1:TRPM1:PLA2G4B:ARPP19:TCF12:CD2BP2:POLR2C:ESRP2:HNF1B:STAC2:IKZF3:ZPBP2:SPATA32:TBX21:SPAG9:KCNJ2:TTYH2:UBALD2:MPPE1:TTC39C:SLC14A1:MBP:KCNJ14:AFF3:IL1RL1:IL18R1:IL18RAP:SCN3A:PANK2:CHMP4B:WBP2NL:VGLL4:CACNA2D2:RBP2:STIM2:SLC4A4:GPRIN3:EIF4E:CDH9:CDH6:ISL1:PCDHGC3:CDX1:SLC22A23:HOXA10:HOXA11:HOXA13:MPLKIP:NYAP1:CREB3L2:ZMAT4:TMEM71:KIAA0020:CNTLN:ADAMTSL1:PALM2:PBX3:SLC34A3
TF_targets	CAGCTG_AP4_Q5	1535	113	8.359902052245964e-12	1.6998467506233458e-09	TRIM62:MFSD2A:EBNA1BP2:WDR65:TMEM125:TXNDC12:HOOK1:SSX2IP:CSDE1:GOLPH3L:ARNT:FAM63A:TMOD4:RIT1:ARHGEF2:SEMA4A:SMG5:TMEM79:FCRLA:FCRLB:CRB1:B3GALNT2:CREM:RGR:HECTD2:GRK5:SYVN1:OPCML:USP5:TPI1:PTPN6:SOX5:HOXC12:DAO:TNFRSF19:WBP4:DLEU1:PCDH9:FITM1:STXBP6:SCFD1:LRFN5:PRPF39:ZFYVE1:OTUD7A:TCF12:RASL12:ANP32A:MESDC1:MYLPF:ARMC5:RNF166:TRPV1:SLC5A10:EPN2:CACNB1:VAT1:RPS6KB1:KCNJ16:KCNJ2:UBALD2:TNRC6C:C17orf70:MBP:TUBB4A:INSR:ERF:MEGF8:GRIN2D:MYCN:OSR1:ALK:DCTN1:RTKN:FBXO36:HES6:WBP2NL:LMF2:NCAPH2:HDAC11:TGFBR2:SCN5A:CYP8B1:HYAL2:TUSC2:RASSF1:SLC25A26:FOXP1:GSK3B:ARMC8:DVL3:MAP3K13:FGF12:TRMT10A:CDH6:NIPBL:ISL1:MEGF10:SLC12A2:CDX1:CPEB4:ZFAND3:PEX7:ESR1:ITGB8:SEPT7:SRRM3:TSC22D4:STAR:RDH10:GEM:COL22A1:PALM2
TF_targets	SCGGAAGY_ELK1_02	1248	96	3.196695161612652e-11	4.874960121459294e-09	EBNA1BP2:WDR65:C1orf210:TIE1:MED8:SZT2:TXNDC12:KTI12:TCEANC2:TM2D1:RPF1:C1orf52:EXTL2:NRAS:CTSK:SETDB1:FAM63A:CKS1B:GON4L:SEMA4A:UHMK1:ZBTB41:SNRPE:ARL5B:ZFYVE27:CTR9:PEX16:ZNHIT2:MRPL49:USP5:SPSB2:C12orf57:EMG1:PHB2:DDX47:PTHLH:TFCP2:PRKAB1:DHX37:COMMD6:TGDS:SCFD1:FBXO33:GTF2A1:CD2BP2:ZNF48:RPL19:PSMD12:CCDC130:C19orf57:DNAJB1:NFKBIB:GRWD1:RPE:FBXO36:PER2:TMEM230:TYMP:THUMPD3:RPL32:NPRL2:CYB561D2:XXcos-LUCA11.4:SHQ1:CGGBP1:C3orf38:ATP2C1:ACAD11:UBA5:DBR1:TRMT10A:NIPBL:RPL37:CKS1B:CETN3:SLC12A2:DIAPH1:THG1L:CPEB4:VPS52:RPS18:B3GALT4:WDR46:PFDN6:PPIL1:C6orf89:ZFAND3:TCTE3:EVX1:SEPT7:RFC2:PPP1R35:CEP41:RB1CC1:PUF60:IPPK:TSTD2:ZNF79
TF_targets	RTAAACA_FREAC2_01	938	78	6.676695149003098e-11	8.145568081783779e-09	CLSTN1:SPEN:TMEM57:ZNF362:MED8:SZT2:TXNDC12:TCEANC2:DNAJB4:HIPK1:NRAS:CSDE1:ARHGEF2:CRB1:ADCK3:RBP3:LRIT1:GRK5:WEE1:POU2AF1:KCNA1:EMP1:SOX5:CCER1:ULK1:TNFRSF19:WBP4:SIAH3:PCDH9:KCTD12:DCAF11:FOXG1:COCH:FRMD6:ZFYVE1:GTF2A1:TCF12:ANP32A:C15orf26:LINS:SMG1:CD2BP2:NFATC3:PITPNC1:KCNJ2:KIF19:PHLPP1:INSR:FAM49A:OSR1:PPP1CB:BCL11A:UCKL1:CYB5R3:EFHB:RAB43:CNBP:MAP3K13:FGF12:GPRIN3:MTTP:ISL1:OCLN:EDN1:WDR46:PFDN6:CDKN1A:HOXA10:HOXA11:NEUROD6:ABCB1:NYAP1:KLF14:CALD1:TAS2R39:ZMAT4:PAPPA:SLC34A3
TF_targets	AACTTT_UNKNOWN	1928	129	1.5745319281263533e-10	1.600774126928459e-08	GPR153:TMEM57:ZNF362:PHC2:TAL1:HOOK1:LPHN2:C1orf52:PTBP2:EXTL2:HIPK1:NRAS:GOLPH3L:CTSS:LCE1F:CKS1B:RIT1:ARHGEF2:LMNA:PMF1:ARHGEF11:NHLH1:SOAT1:CFHR5:ARL5B:CREM:HECTD2:WEE1:EHF:SPSB2:SOX5:PTHLH:HOXC12:HOXC11:RAP1B:TNFRSF19:POSTN:WBP4:SIAH3:PCDH9:PCK2:STXBP6:ERO1L:PLA2G4B:TCF12:ANP32A:ACAN:CALB2:FAM211A:CACNB1:STAC2:IKZF3:RND2:SPAG9:UTP18:PITPNC1:KCNJ2:RALBP1:IMPA2:CHST8:ERF:CIC:TMEM145:HPCAL1:GREB1:FAM49A:OSR1:ALK:BCL11A:XPO1:AFF3:IL18R1:MERTK:RBMS1:CREB1:CTDSP1:MMP24:MC3R:USP25:C21orf91:MCHR1:SCN5A:IFT5

						7:RBP2:FGF12:STK32B:GPRIN3:EIF4E:CDH9:NIPBL:ISL1:CKS1B:C5orf30:MARCH3:SLC12A2:PCDHGC3:DIAPH1:PRELID2:KCNIP1:CPNE5:ZFAND3:HCRTR2:FUT9:ESR1:SYNE1:ARL4A:HOXA10:HOXA11:EVX1:JAZF1:NEUROD6:MPLKIP:HSPB1:TSC22D4:PRKAR2B:GPR22:DUS4L:COPG2:KLF14:CALD1:CREB3L2:ZMAT4:RB1CC1:CALB1:GEM:KIAA0020:ADAMTSL1:KLF4:PALM2:PBX3
TF_targets	COUP_01	266	34	5.637939843518058e-10	4.9130618636371654e-08	ACOT7:MED8:SZT2:SCNM1:LYSMD1:CKS1B:PKLR:LMNA:CFHR5:LRFN4:EMG1:PHB2:SLC11A2:WBP4:LINS:MYLPF:ASGR2:HNF1B:ZPBP2:PITPNC1:CIC:DCTN1:RBMS1:BPIFA2:FBLN1:RBP2:FGF12:MTTP:CKS1B:CDX1:C4A:HOXA10:NYAP1:CREB3L2:NUDCD1
TF_targets	TGACCTY_ERR1_Q2	1064	80	4.132095649504e-09	3.116496643953119e-07	GPR153:CDCP2:EXTL2:WDR77:ANXA9:SCNM1:LYSMD1:ADAM15:RBP3:HECTD2:TLL2:SMPD1:HPX:ZNF143:LRFN4:DNAJB13:CRTAM:TPI1:SOX5:CSRNP2:HOXC13:SLC25A3:PRKAB1:WBP4:LRFN5:SIX6:HNF1B:STAC2:SPATA32:SPAG9:ST8SIA5:TUBB4A:INSR:RFX1:PKN1:PPP1CB:ATP6V1B1:DCTN1:RTKN:RND3:RBMS1:RPE:MFF:BPIFA2:USP25:SYNGR1:FBLN1:RPL32:SCN5A:RASSF1:PLCXD2:NR1I2:RAB6B:AP2M1:ABCF3:FGF12:EIF4E:TRMT10A:MTTP:PLA2G12A:CDH6:TMEM161B:DIAPH1:RELL2:NDFIP1:CDKN1A:HOXA10:CLDN4:YWHAG:NYAP1:PPP3CC:ZMAT4:RB1CC1:CA2:TMEM71:CNTLN:NTMT1:CEL:UBAC1:NACC2
TF_targets	HNF4_01	274	33	4.598109802553782e-09	3.116496643953119e-07	ACOT7:C1orf210:MED8:SZT2:PTBP2:SCNM1:LYSMD1:PKLR:SEC16B:F13B:PEX16:LRFN4:TPI1:ATN1:SOX5:CCER1:PAPLN:LINS:HNF1B:INSR:PKP4:RBMS1:CREB1:PPARA:RBP2:CDH6:SNCB:C4A:HOXA10:NYAP1:CREB3L2:CALB1:SLC25A25
TF_targets	TGANTCA_AP1_C	1143	82	2.1372258107817594e-08	1.303707744576873e-06	ZNF362:MACF1:C1orf210:ELOVL1:LPHN2:F3:NRAS:GJA5:ADAM15:HCN3:RIT1:ARHGEF2:LMNA:SEC16B:ZBTB41:CDH23:EHF:POU2AF1:ENO2:EMP1:RASSF8:PDE1B:NUAK1:ORAI1:PCDH9:KCNK10:TCF12:PDZD9:TBC1D10B:ZNF771:N4BP1:CALB2:ZPBP2:VAT1:NPEPPS:SPAG9:TRIM25:PSMD12:PITPNC1:SSTR2:UBALD2:TUBB4A:PKN1:GIPC1:FAM49A:SLC11A1:VIL1:DSTN:CHMP4B:ZNFX1:SYNGR1:TGFBR2:SCN5A:ZNF654:AP2M1:ABCF3:FGF12:GPRIN3:IPO11:MCTP1:DIAPH1:HDAC3:RELL2:FCHSD1:PDGFRB:TTC1:SNCB:CDKN1A:PTP4A1:POPDC3:AIG1:ESR1:HOXA11:JAZF1:CLDN4:YWHAG:RB1CC1:ADAMTSL1:UBQLN1:PAPPA:LRSAM1:TOR4A
TF_targets	OLF1_01	277	31	7.364402900071531e-08	3.867759019391135e-06	VWA5B1:IPO7:PTPN6:SOX5:PTHLH:HOXC13:SIAH3:C14orf142:UBR7:MESDC1:SMPD3:RND2:TBX21:RPS6KB1:KCNJ2:CD79A:GRIN2D:GRWD1:FAM49A:AFF3:SPATC1L:WBP2NL:CMTM8:GSK3B:FGF12:STIM2:TRMT10A:PDGFRB:CDX1:YWHAG:TSC22D4
TF_targets	YTATTTTNR_MEF2_02	708	57	7.608706267654693e-08	3.867759019391135e-06	RNF207:ANGPTL7:ZNF362:C1orf180:LRRC8C:HIPK1:TRIM33:DENND2C:CTSK:PRUNE:TMOD4:LMNA:CTR9:KCNJ11:OPCML:SIAH3:PCDH9:FITM1:MIA2:CTAGE5:SIX6:MYLPF:POLR2C:EPN2:ADAM11:UBALD2:SLC14A1:GRIN2D:BCL11A:CREB1:TM4SF20:ZNFX1:USP25:PPARA:CPT1B:FOXP1:GP9:CNBP:ATP2C1:DZIP1L:FGF12:CXCL1:MARCH3:EDN1:LRRC16A:CDKN1A:SYNE1:HOXA10:JAZF1:LAT2:YWHAG:GNG11:PRKAR2B:KLF14:LYN:TMEM71:CNTLN:PBX3
TF_targets	GCANCTGNY_MYOD_Q6	932	69	8.805083086017468e-08	3.976508018588658e-06	ANGPTL7:FBLIM1:MED8:SZT2:SSX2IP:NRAS:CSDE1:GJA5:FAM189B:NHLH1:FCRLA:FCRLB:SOAT1:CRB1:ARL5B:GRK5:OPCML:SOX5:TNFRSF19:PCDH9:FITM1:SCFD1:LRFN5:FRMD6:SIX6:PSEN1:TCF12:ANP32A:GRIN2A:MYLPF:TRPV1:CACNB1:ADAM11:NPEPPS:KCNJ2:FSCN2:TUBB4A:RFX1:PKN1:ERF:CNFN:GRIN2D:OSR1:ALK:XPO1:RTKN:PKP4:IGFBP5:HES6:SLC37A1:SPATC1L:SYNGR1:MCHR1:WBP2NL:HDAC11:HYAL2:GSK3B:ARMC8:DVL3:FGF12:ISL1:MEGF10:VPS52:RPS18:MAP7:SYNE1:NEUROD6:CLDN3:COL22A1
TF_targets	WTGAAAT_UNKNOWN	623	52	9.126411845941183e-08	3.976508018588658e-06	DNAJB4:DPH5:NRAS:GOLPH3L:RIT1:LMNA:NHLH1:CREM:CDH23:TLL2:TTC12:OPCML:EMP1:SOX5:PTHLH:HOXC11:TNFRSF19:LHFP:SIAH3:UCHL3:FOXG1:KCNK10:MOAP1:ANKRD11:ZPBP2:ADAM11:TUBD1:RPS6KB1:PKN1:GRWD1:MTHFD2:SLC4A5:PKP4:CREB1:IGFBP5:C2CD2:SAMD7:GPRIN3:TRMT10A:C5orf20:PCDHGC3:NDFIP1:LRRC16A:HECA:HOXA13:JAZF1:NEUROD6:GPR22:KLF14:KIAA0020:ADAMTSL1:PAPPA
TF_targets	GGGTGGRR_PAX4_03	1311	88	1.296779469845164e-07	5.07448162236824e-06	ANGPTL7:SPEN:NBL1:MFSD2A:TIE1:TSPAN1:TAL1:SIKE1:SEMA6C:CKS1B:ARHGEF2:NHLH1:AGAP4:AGAP8:DUSP8:ZNF143:EHF:SYVN1:MRPL49:LRFN4:AASDHPPT:CLMP:KCNA1:ENO2:ATN1:PTPN6:HOXC11:KCTD12:PSME1:LRFN5:KLHL28:FAM179B:ZFYVE1:KCNK10:MESDC1:ACAN:LINS:CORO1A:CD2BP2:DERL2:LYZL6:HNF1B:CACNB1:ADAM11:KPNB1:SPAG9:UTP18:RNFT1:PITPNC1:RALBP1:TUBB4A:C19orf57:RFX1:CD97:PTGER1:ERF:GRIN2D:KCNJ14:OSR1:DCTN1:RTKN:IGFBP5:FBXO36:WFDC5:LIME1:GNAI2:HYAL1:HYAL2:RAB6B:ARMC8:EIF4E:ISL1:CCNO:CKS1B:FCHSD1:CDX1:TSPAN17:CDKN1A:CPNE5:HOXA10:EVX1:SEPT7:YWHAG:ZCWPW1:MEPCE:NYAP1:PRKAR2B:KEL:ZMAT4:CHMP5
TF_targets	RYTTCCTG_ETS2_B	1115	78	1.331011573080194e-07	5.07448162236824e-06	FBLIM1:LDLRAP1:MPL:ELOVL1:DNASE2B:EXTL2:GJA5:GOLPH3L:CTSS:CTSK:SEMA6C:CKS1B:ADAM15:ASH1L:YY1AP1:DAP3:ARHGEF2:CREM:PSAP:TLL2:GRK5:IPO7:ZNF143:SLC43A1:LRFN4:TRPC6:PTPN6:LCP1:TNFSF13B:MOAP1:TCF12:ADAMTS7:SMG1:SEPT1:SEPT1:CLEC10A:KPNB1:TRIM25:PITPNC1:CD79A:ERF:RTKN:IL18RAP:SCN3A:SLPI:LIME1:UCKL1:EFHB:TGFBR2:CMTM8:HYAL2:RASSF1:NPRL2:CYB561D2:XXcos-

						LUCA11.4:FOXP1:STX19:CD200R1:RAB43:RASA2:DROSHA:C5orf22:CARD6:CKS1B:TMEM161B:PDGFRB:SOX30:CDKN1A:C6orf89:AIG1:THBS2:HOXA10:HOXA11:GNG11:TSC22D4:CALD1:ZMAT4:LYN:TMEM71:CNTLN:ADAMTSL1
TF_targets	AP1_Q6	259	29	1.8994132532239973e-07	6.815541673333167e-06	RIT1:LMNA:REXO2:ORAI1:PCDH9:UCHL3:KCNK10:TCF12:PDZD9:ZNF771:PSMD12:PITPNC1:DSTN:ZNFX1:TGFBR2:FGF12:EIF4E:DIAPH1:HDAC3:RELL2:PDGFRB:SNCB:CDKN1A:PTP4A1:HOXA11:RB1CC1:UBQLN1:PAPPA:TOR4A
TF_targets	ALPHACP1_01	261	29	2.2379882159166209e-07	7.5842933983841035e-06	ZNF362:FDPS:ASPM:EHF:USP5:C12orf57:LPCAT3:SDS:DLEU1:NRL:DCAF11:DLGAP5:LINS:UTP18:ERF:KIAA1841:XPO1:AFF3:PCNA:MAP3K13:CDH9:CDH6:NIPBL:ISL1:THG1L:HOXA10:CALD1:ADAMTSL1:SLC25A25
TF_targets	MEIS1_01	249	28	2.8087026384498139e-07	9.017413734125605e-06	TCEANC2:DNASE2B:CTSK:ASH1L:YY1AP1:DAP3:SEMA4A:ABL2:POU2AF1:LPCAT3:TNFRSF19:FITM1:FKBP3:FRMD6:SMPD3:NPEPPS:SPAG9:RPS6KB1:EPG5:HES6:SMDT1:ARFGAP3:AP2M1:FGF12:HCRTR2:KLF14:TMEM71:SLC25A25
TF_targets	GATTGGY_NFY_Q6_01	1179	80	3.173289421308664e-07	9.678532734991425e-06	GPR153:MTOR:TMEM57:ZNF362:TAL1:STIL:GOLPH3L:CKS1B:FDPS:TLL2:SFRP5:WEE1:EHF:PEX16:TM7SF2:LRFN4:AASDHPPT:BTG4:C11orf88:KCNA1:C12orf57:SLC11A2:TFCP2:HOXC11:C12orf75:CIT:SIAH3:DLEU1:PCDH9:KDELC1:BIVM:DCAF11:FKBP3:SIX6:NUMB:LINS:ANKRD11:DERL2:MAPK7:BRCA1:KPNB1:UTP18:KCNJ2:UBALD2:TNRC6C:ENTHD2:CCDC130:DNAJB1:TECR:SARS2:MRPS12:SPDYA:MOB1A:PANK2:DSTN:RPN2:TGFBR2:RPL14:GNAI2:CGGBP1:PLCXD2:GSK3B:MAP3K13:FGF12:ATP10D:EIF4E:CDH9:CDH6:DROSHA:C5orf22:CKS1B:CETN3:PDGFRB:CPEB4:PPIL1:THBS2:CEP41:ZMAT4:TERF1:ADAMTSL1:PBX3
TF_targets	COUP_DR1_Q6	253	28	3.9000364230268537e-07	1.1328677228792289e-05	MED8:SZT2:HMGCS2:SCNM1:LYSMD1:CKS1B:SMG5:TMEM79:PEX16:ATN1:EMG1:PHB2:SLC11A2:HOXC13:PAPLN:KCNK10:INSR:RFX1:PRODH2:BCAM:DCTN1:RBMS1:BPIFA2:FBLN1:HDAC11:MTTP:CKS1B:NYAP1:NUDCD1
TF_targets	AP1_Q4	271	29	4.942282990411734e-07	1.3703602837050717e-05	ZNF362:NRAS:LMNA:CREM:REXO2:PCDH9:UCHL3:TCF12:TBC1D10B:ZNF771:RABEP1:ZPBP2:PITPNC1:ST8SIA5:DSTN:ZNFX1:TGFBR2:FGF12:EIF4E:DIAPH1:HDAC3:RELL2:PDGFRB:EDN1:CDKN1A:HOXA11:RB1CC1:UBQLN1:PAPPA
TF_targets	VDR_Q6	276	29	7.223058462653482e-07	1.9156807227037493e-05	TMEM57:TRIM33:SEMA4A:LPCAT3:SOX5:TCF12:MESDC1:LINS:GRIN2A:CD2BP2:RND2:KPNB1:C17orf70:PPP4R1:PODNL1:PKN1:BCAM:GREB1:XPO1:RND3:FBXO36:CHMP4B:ZPLD1:SLC4A4:EIF4E:NIPBL:CDX1:SNCB:KLF4
TF_targets	DR1_Q3	263	28	8.568102823126999e-07	2.1777261342114455e-05	MED8:SZT2:HMGCS2:SCNM1:LYSMD1:SMG5:TMEM79:SEC16B:SPSB2:ATN1:EMG1:PHB2:SOX5:HOXC13:PAPLN:KCNK10:TTYH2:INSR:PRODH2:BCAM:DCTN1:RBMS1:BPIFA2:WBP2NL:FBLN1:MTTP:CDX1:NUDCD1
TF_targets	FREAC2_01	264	28	9.246267388729003e-07	2.256089242849877e-05	TMEM57:ZNF362:MED8:SZT2:TXNDC12:HIPK1:WEE1:EMP1:HOXC12:SIAH3:FOXG1:C15orf26:SH3GL3:PITPNC1:PHLPP1:BCL11A:DSTN:FGF12:MTTP:NIPBL:VPS52:RPS18:CDKN1A:HOXA11:NYAP1:CALD1:PAPPA:SLC34A3
TF_targets	CETS1P54_01	265	28	9.973663613674457e-07	2.3399749247466997e-05	MTOR:EBNA1BP2:WDR65:MED8:SZT2:EXTL2:NRAS:CKS1B:UHMK1:ARL5B:IPO7:CTR9:SYVN1:DDX47:TFCP2:PSMD12:TYMP:CGGBP1:STX19:ACAD11:UBA5:NIPBL:CKS1B:CPEB4:VPS52:RPS18:RGL2:PPIL1:TMEM71
TF_targets	RNGTGGGC_UNKNOWN	786	58	1.036849175910621e-06	2.342511101131403e-05	RPL22:PTBP2:NRAS:CSDE1:SEMA4A:SMG5:TMEM79:DMBT1:IPO7:PTPN6:HOXC11:UCHL3:FOXG1:SH3GL3:PKD1:GRIN2A:TGFB1I1:VAT1:UTP18:ENTHD2:SERPINB4:SAFB:ERF:PAFAH1B3:MEGF8:KDELR1:KCNJ14:PEX13:ATP6V1B1:DUSP11:DCTN1:RTKN:PKP4:FBXO36:LIME1:UCKL1:LSMEM2:FOXP1:CGGBP1:FGF12:GPRIN3:MTTP:DIAPH1:HDAC3:RELL2:PDGFRB:ARL4A:ITGB8:HOXA10:HOXA11:EVX1:NEUROD6:YWHAG:ZCWPW1:MEPCE:CEP41:CALD1:CREB3L2
TF_targets	NFE2_01	275	28	2.0769534791338783e-06	4.524791508113092e-05	TMEM57:RIT1:REXO2:PDE1B:PCDH9:TBC1D10B:ZNF771:N4BP1:CALB2:VAT1:PSMD12:SLC11A1:SYNGR1:ABCF3:FGF12:IPO11:DIAPH1:HDAC3:RELL2:SNCB:CDKN1A:HOXA11:JAZF1:YWHAG:ZCWPW1:MEPCE:RB1CC1:NUDCD1
TF_targets	LEF1_Q2	232	25	2.610283530908673e-06	5.3088456312981254e-05	RIMKLA:ELOVL1:TCEANC2:LPHN2:C1orf52:DPH5:TRIM33:GOLPH3L:ARNT:FAM63A:PRUNE:SCNM1:LYSMD1:SCAMP3:CFHR5:ZBTB41:CREM:ZNF488:TLL2:HABP2:GRK5:SYT13:UCP2:DGAT2:PRKRIR
TF_targets	TGACAGNY_MEIS1_01	848	60	2.6109076875236683e-06	5.3088456312981254e-05	ZBTB17:PHC2:TMEM125:EXTL2:DENND2C:GOLPH3L:CTSK:FAM63A:SEMA6C:ASH1L:YY1AP1:DAP3:SEMA4A:SMG5:TMEM79:ABL2:APBB1:SYVN1:MRPL49:UCP3:POU2AF1:LPCAT3:SOX5:DHX37:TNFRSF19:LHFP:FITM1:G2E3:COCH:FKBP3:FRMD6:KCNK10:HNF1B:SPAG9:RPS6KB1:RALBP1:TUBB4A:FAM49A:OSR1:XPO1:ZNF638:ITPRIPL1:RBMS1:VIL1:HES6:SMDT1:ATP2C1:AP2M1:MAP3K13:FGF12:EIF4E:NIPBL:SLC12A2:PCDHGC3:EVX1:KLF14:CALD1:TMEM71:CNTLN:PBX3
TF_targets	HNF4_DR1_Q3	265	27	3.0976441928439167e-06	6.095364379467062e-05	MPL:MED8:SZT2:SCNM1:LYSMD1:SCAMP3:PKLR:SEC16B:GRK5:PEX16:SPSB2:ATN1:SOX5:PAPLN:KCNK10:ZPBP2:TTYH2:INSR:PRODH2:DCTN1:RBMS1:BPIFA2:FBLN1:SENP2:MTTP:CDX1:NUDCD1
TF_targets	AP4_01	266	27	3.3261038744536167e-06	6.340385510677207e-05	ANGPTL7:RUSC1:CRB1:UCP3:OPCML:DCAF11:FKBP3:MOAP1:ESRP2:DERL2:EPN2:KCNJ2:TNRC6C:GRIN2D:BCL11A:BOLA3:HES6:HYAL2:FOXP1:GSK3B:EIF4E:NIPBL:HOXA10:HOXA11:EVX1:SRRM3:CNTLN
TF_targets	AFP1_Q6	267	27	3.5699110244185025e-06	6.598926439076626e-05	PMF1:SEC16B:SOAT1:HABP2:ZNF143:BTG4:C11orf88:TTC12:C12orf75:SIAH3:LRFN5:SMG1:ADAM11:PUM2:AFF3:PKP4:RBMS1:FOXP1:ZNF654:FGF12:SLC4A4:NIPBL:PCDHGC3:ESR1:JAZF1:CALD1:TMEM71

TF_targets	MAZ_Q6	192	22	3.7642085607666318e-06	6.753433006081309e-05	CLSTN1:ZBTB17:TMEM57:ZNF362:PHC2:TRIM33:NRAS:CSDE1:SETDB1:CKS1B:FAM189B:FDPS:SYT11:ARHGEF2:LMNA:ZNF496:CUL2:CREM:GRK5:DUSP8:APBB1:ZNF143:CKS1B
TF_targets	NFKB_C	269	27	4.107317485173665e-06	6.959621294322043e-05	TAL1:FOXD2:ASH1L:YY1AP1:DAP3:ARHGEF2:GRK5:EHF:SLC43A1:SNX15:OPCML:SOX5:PTHLH:ANP32A:ACAN:SMPD3:NFKBIB:GRIN2D:BCL11A:IL18R1:RAD18:CGGBP1:MAP3K13:ITGB8:HOXA11:PAPPA:SLC34A3
TF_targets	ETS1_B	269	27	4.107317485173665e-06	6.959621294322043e-05	TSPAN1:EXTL2:GJA5:ADAM15:POU2AF1:LCP1:ARPP19:CORO1A:SEPT1:SEPT1:GLP2R:ADAM11:UBALD2:CD79A:FBXO36:LIME1:HYAL2:FOXP1:RAB43:RASA2:NIPBL:TMEM161B:MCTP1:HOXA10:TSC22D4:LYN:TMEM71:TOR4A
TF_targets	TATAAA_TATA_01	1306	82	4.701236189564006e-06	7.75068669090282e-05	RNF207:GPR153:SPEN:TMEM57:HOOK1:AMPD1:NRAS:CSDE1:HMGCS2:SCAMP3:RIT1:ARHGEF2:BGLAP:FCRLA:CREM:PSAP:DMBT1:ZNF143:LPCAT3:SOX5:PTHLH:HOXC12:POSTN:WBP4:LCP1:COCH:MIA2:CTAGE5:LRFN5:KLHL28:FAM179B:GTF2A1:PLA2G4B:TCF12:KIAA1199:PRPSAP2:ADAM11:RLN3:TECR:KCNJ14:PUM2:RND3:RBMS1:FBXO36:DSTN:UCKL1:KRTAP8-1:FOXP1:ZNF654:IFT57:RBP2:XRN1:MAP3K13:STIM2:AFM:AREG:AREGB:NPY5R:NIPBL:C5orf30:LECT2:CDX1:SOX30:CPEB4:EDN1:B3GALT4:CNR1:HECA:AIG1:SYNE1:THBS2:HOXA10:HOXA11:NEUROD6:CD36:ABCB1:KLF14:CALD1:BLK:CA2:GEM:KIAA0020:CNTLN:PAPPA
TF_targets	HNF4_Q6	272	27	5.0533009377852214e-06	7.969320797457411e-05	C1orf210:SLC16A4:PKLR:YY1AP1:DAP3:ARHGEF2:SEC16B:C1S:EMP1:CCER1:KCTD12:FOXG1:NUMB:INSR:TM4SF20:SLPI:PROS1:DZIP1L:MTTP:NIPBL:PCDHGC3:C4A:DACT2:HOXA10:HOXA11:EVX1:CREB3L2
TF_targets	TTCYRGAA_UNKNOWN	337	31	5.095139526243262e-06	7.969320797457411e-05	ACOT7:ELOVL1:DNAJB4:SELP:RAB18:HSPA8:PTHLH:FBXO33:DCAF4:TBC1D10B:NFATC3:RNFT1:PKN1:GRIN2D:XPO1:SCN3A:PER2:DAZL:ATP2C1:PLSCR4:KCNAB1:C4orf17:MEGF10:VPS52:RPS18:CDKN1A:ESR1:SOD2:HSPB1:YWHAG:GPR22
TF_targets	E4F1_Q6	292	28	6.591059559072677e-06	0.00010051365827585832	RUSC1:FAU:MRPL49:ERC1:HOXC11:LGR5:CCER1:GTF2A1:PSMD12:SSTR2:CCDC130:RFX1:ERF:OSR1:TRMT61B:PKP4:CREB1:ABCF3:MAP3K13:ATP10D:PTP4A1:NEUROD6:CLDN3:YWHAG:CALD1:GEM:SLC25A25:NTMT1
TF_targets	CAGGTA_AREB6_01	799	56	7.362686827117593e-06	0.0001069942088563149	ICMT:ZNF362:SCAMP3:FDPS:BTG4:C11orf88:CRTAM:KCNA1:SOX5:PTHLH:CCER1:ULK1:TNFRSF19:FOXG1:MIA2:CTAGE5:LRFN5:FKBP3:PLA2G4B:TCF12:ANP32A:C15orf26:CORO1A:ESRP2:CACNB1:KCNJ2:PPP4R1:CIC:PAFAH1B3:KCNJ14:GREB1:FAM49A:BCL11A:DCTN1:IGFBP5:UCKL1:USP25:CYB5R3:FOXP1:RAB43:KCNAB1:STIM2:CDH6:TMEM161B:DIAPH1:FLOT1:IER3:PTP4A1:ITGB8:HOXA13:EVX1:PILRB:GPR22:KLF14:RB1CC1:ADAMTSL1:SLC34A3
TF_targets	SREBP1_Q6	246	25	7.406490050953394e-06	0.0001069942088563149	MACF1:ANXA9:DUSP8:PEX16:ERC1:ENO2:EMP1:HOXC11:ZFYVE1:KCNK10:ANP32A:CORO1A:MYLPF:TGFB1I1:RNF112:HNF1B:CACNB1:KPNB1:ERF:GRWD1:DCTN1:HYAL1:ESR1:HOXA11:EVX1
TF_targets	NFY_01	262	26	7.542214722658263e-06	0.0001069942088563149	TMEM57:STIL:FDPS:ADCK3:EHF:TM7SF2:C12orf57:CIT:DLEU1:PCDH9:DCAF11:NUMB:LINS:DERL2:UTP18:KCNJ2:UBALD2:SPDYA:XPO1:MOB1A:DSTN:RPN2:GSK3B:CDH6:ISL1:PDGFRB
TF_targets	LMO2COM_02	247	25	7.951038125864854e-06	0.0001102303012903991	TMEM57:RBP3:SFRP5:MOGAT2:SOX5:HOXC11:RAP1B:WBP4:SIAH3:PCDH9:TNFSF13B:RNF112:PKN1:IGFBP5:MFF:HYAL3:NAT6:RAB43:SAMD7:ISL1:CPEB4:PTP4A1:HOXA10:PILRB:ADAMTSL1
TF_targets	PPAR_DR1_Q2	265	26	9.245607046499728e-06	0.00011999617656095392	ACOT7:MED8:SZT2:HMGCS2:SCNM1:LYSMD1:SMG5:TMEM79:RCE1:LRFN4:ATN1:EMG1:PHB2:LRFN5:PAPLN:INSR:CHST8:PRODH2:DCTN1:RTKN:RBMS1:WBP2NL:RBP2:MTTP:CDX1:SLC34A3
TF_targets	STAT4_01	265	26	9.245607046499728e-06	0.00011999617656095392	TRIM62:EXTL2:VAV3:NRAS:CTSK:RASSF8:HOXC13:TNFRSF19:FOXG1:KCNK10:TDRD9:PITPNC1:TTYH2:ERF:OSR1:PKP4:RBMS1:STX19:CD200R1:FGF12:STIM2:NDFIP1:CPEB4:EDN1:LRRC16A:ABCB1
TF_targets	AP1_Q2	265	26	9.245607046499728e-06	0.00011999617656095392	ZNF362:NRAS:LMNA:CREM:ORAI1:PCDH9:UCHL3:KCNK10:TCF12:TBC1D10B:ZNF771:ZPBP2:PITPNC1:ST8SIA5:DSTN:ZNFX1:TGFBR2:FGF12:HDAC3:RELL2:PDGFRB:SNCB:HOXA11:HOXA13:UBQLN1:PAPPA
TF_targets	HIF1_Q5	250	25	9.810454917736921e-06	0.00012467453124624003	TMEM57:RIMKLA:MPL:TRIM33:ARL5B:WEE1:C11orf71:RBM7:FKBP3:ERO1L:MESDC1:KPNB1:RNFT1:PITPNC1:PHLPP1:ERF:C19orf68:BCL11A:CHMP4B:LMF2:NCAPH2:HYAL2:CACNA2D2:HDAC3:RELL2
TF_targets	SMAD_Q6	255	25	1.3803840392987234e-05	0.00017184372734126965	RHCE:ZNF362:CKS1B:SYT11:SEMA4A:CREM:HPX:SYVN1:KCNA1:CSRNP2:HOXC13:GTF2A1:SMG1:GRIN2D:RBMS1:IGFBP5:WBP2NL:EIF4E:CKS1B:ARAP3:RGL2:EVX1:NYAP1:CALD1:RPL7:RDH10
TF_targets	IRF1_01	256	25	1.4760689463645309e-05	0.00017922136920135736	ZNF362:F3:NRAS:RIT1:CUL2:USP5:KPNA3:TNFSF13B:ZPBP2:TCEB3B:HPCAL1:NCOA1:BCL11A:CREB1:VGLL4:CD200R1:C3orf17:XRN1:TAPBP:ESR1:HOXA10:HOXA13:YWHAG:BLK:KIAA0020
TF_targets	CACGTG_MYC_Q2	1059	68	1.49840816873266e-05	0.00017922136920135736	RPL22:UBIAD1:EBNA1BP2:WDR65:TXNDC12:WDR77:NRAS:CSDE1:SCAMP3:RUSC1:PSEN2:NID1:REEP3:ALDH18A1:IPO7:WEE1:SLC43A1:ARL2:LRFN4:REXO2:HOXC13:HOXC12:HOXC11:CCER1:DCAF11:MIA2:CTAGE5:FBXO33:KLHL28:FAM179B:GTF2A1:ACAN:GRIN2A:SMG1:ZNF771:DCTPP1:NFATC3:ESRP2:RPL19:IKZF3:UTP18:UBXN6:PKN1:ERF:OSR1:XPO1:NCL:C21orf91:CYP2

						D6:DAZL:CMTM8:IFRD2:HYAL2:GSK3B:KCNAB1:COMMD8:EIF4E:DIAPH1:CPEB4:SNCB:TUBB:WDR46:PFDN6:MAP7:HOXA11:NEUROD6:ZCWPW1:MEPCE:NTMT1
TF_targets	CREB_Q2_01	226	23	1.651681934288563e-05	0.00019375499613769685	CDC42:SYT11:CREM:ZFYVE27:LGR5:SLC25A3:GTF2A1:PDXDC1:RNF166:SPAG9:PITPNC1:ERF:OSR1:DCTN1:SENP2:AREG:AREGB:EVX1:NEUROD6:CALD1:FAM167A:GEM:RBM18:SLC25A25
TF_targets	HNF4_01_B	258	25	1.685686801683691e-05	0.0001940130092503871	ACOT7:C1orf210:LPHN2:SCNM1:LYSMD1:SMG5:TMEM79:SEC16B:PEX16:KCNK10:TCF12:LINS:ESRP2:HNF1B:INSR:PRODH2:BCL11A:RBMS1:CHMP4B:FBLN1:PPARA:RBP2:HOXA10:EVX1:NYAP1
TF_targets	TNCATNTCCYR_UNKNOWN	138	17	1.7966487224507397e-05	0.00020295476309165763	DNASE2B:SCNM1:LYSMD1:IPO7:DDX25:DDX47:CSRNP2:HOXC12:NTAN1:SMPD3:GSK3A:ZNFX1:DROSHA:C5orf22:TTC1:ABCB1:BET1
TF_targets	E2F_Q2	182	20	1.900790270089603e-05	0.00021038421800077762	RNF207:CLSTN1:CDC42:TMEM57:ZNF362:MED8:SZT2:TAL1:STIL:LPHN2:DDAH1:HIPK1:TRIM33:CSDE1:YY1AP1:DAP3:UBQLN4:CSRP1:NSUN6:ARL5B-AS1:HECTD2
TF_targets	GTGACGY_E4F1_Q6	671	48	1.9313960996792698e-05	0.00021038421800077762	DNAJB4:CSDE1:RUSC1:SOAT1:RAB18:ARL2:FAU:MRPL49:RCE1:FDX1:ERC1:TFCP2:LGR5:SLC25A3:DCAF11:GTF2A1:MESDC1:PDXDC1:SSTR2:PHLPP1:PEX11G:CCDC130:ERF:KDELR1:GRWD1:OSR1:TRMT61B:BCL11A:PKP4:CREB1:CTDSP1:IFT57:MAP3K13:SENP2:DHX29:SKIV2L2:IPO11:CDKN1A:PTP4A1:EVX1:NEUROD6:CLDN3:YWHAG:GEM:RBM18:SLC25A25:NTMT1:SLC34A3
TF_targets	AP4_Q6	229	23	2.0419479714478324e-05	0.00021852425659353995	TRIM62:TMEM125:TXNDC12:CSDE1:GOLPH3L:ARNT:FAM63A:TMOD4:ARHGEF2:SYVN1:HOXC12:RASL12:MESDC1:TRPV1:VAT1:RPS6KB1:UBALD2:RTKN:CTDSP1:CHMP4B:MAP3K13:FGF12:PALM2
TF_targets	FOXO1_01	248	24	2.517335492681564e-05	0.00026029013886267954	MED8:SZT2:LPHN2:HIPK1:CRB1:CREM:EMP1:WBP4:SIAH3:ZFYVE1:SH3GL3:LINS:SPAG9:TCEB3B:OSR1:PPP1CB:ZNF654:FGF12:WDR46:PFDN6:HOXA11:TAS2R39:ZMAT4:PAPPA
TF_targets	GNCF_01	76	12	2.562088662750113e-05	0.00026029013886267954	HOXC13:ZFYVE1:ESRP2:HNF1B:STAC2:TUBD1:RPS6KB1:KCNJ2:INSR:BCL11A:ARMC8:ITGB8
TF_targets	CTGCAGY_UNKNOWN	776	53	2.5854853948875327e-05	0.00026029013886267954	NBL1:SZT2:DNAJB4:C1orf52:NRAS:RUSC1:FCRLB:UHMK1:ADCK3:DUSP8:OPCML:ENO2:NUAK1:PCDH9:LRFN5:KLHL28:FAM179B:GTF2A1:MOAP1:PLA2G4B:GRIN2A:MAPK7:HNF1B:RND2:ADAM11:SPAG9:KCNJ2:CHST8:GRIN2D:PUM2:BCL11A:KIAA1841:XPO1:RTKN:DSTN:WBP2NL:FOXP1:GSK3B:ATP2C1:KCNAB1:DVL3:FGF12:CDH6:NIPBL:HDAC3:RELL2:PDGFRB:KCNIP1:PTP4A1:DACT2:HOXA13:EVX1:GEM
TF_targets	LMO2COM_01	265	25	2.648944991597125e-05	0.00026029013886267954	RIMKLA:NHLH1:ARL5B:ANP32A:MESDC1:MYLPF:ESRP2:SMPD3:NPEPPS:RPS6KB1:FSCN2:KEAP1:PKN1:CD79A:OSR1:DCTN1:IGFBP5:HES6:FGF12:HCRTR2:MAP7:SYNE1:HOXA11:EVX1:CALB1
TF_targets	FOXO1_02	249	24	2.6882424177621e-05	0.00026029013886267954	CLSTN1:TMEM50A:TXNDC12:KCNA1:HOXC11:ULK1:SIAH3:FOXG1:PSEN1:C15orf26:SH3GL3:PITPNC1:KCNJ16:RFX1:WBP2NL:ACKR4:WDR46:PFDN6:CDKN1A:ITGB8:HOXA10:HOXA11:ZMAT4:TMEM71
TF_targets	SP3_Q3	249	24	2.6882424177621e-05	0.00026029013886267954	TMEM125:MPL:C1orf52:TMOD4:KCNJ11:ADAMTS7:C15orf26:LINS:GRIN2A:RNF112:ADAM11:PLEKHM1:CNFN:KDELR1:CTDSP1:SCN5A:FOXP1:GSK3B:HDAC3:RELL2:CDX1:ITGB8:KLF14:FAM166A
TF_targets	ZIC2_01	250	24	2.869537666690642e-05	0.0002735028088564518	MACF1:TRIM33:CSDE1:SEMA6C:ZMYND11:HPX:KCNA1:UBALD2:MEGF8:GRIN2D:MYCN:PKP4:FOXP1:ATP2C1:RAB6B:FGF12:NIPBL:PI16:HOXA10:YWHAG:ZCWPW1:MEPCE:TSC22D4:CALB1
TF_targets	MYC_Q2	188	20	3.0470866887357442e-05	0.0002859573661736622	RPL22:UBIAD1:EBNA1BP2:WDR65:TXNDC12:WDR77:NRAS:CSDE1:RUSC1:PSEN2:NID1:ALDH18A1:IPO7:WEE1:SLC43A1:REXO2:HOXC13:HOXC12:HOXC11:CCER1
TF_targets	AML1_Q6	268	25	3.196255595416016e-05	0.0002910023751050403	MPL:FOXD2:DNASE2B:HIPK1:NRAS:CTSK:ARNT:ARHGEF2:SEMA4A:BGLAP:CREM:TLL2:GRK5:BTG4:C11orf88:REXO2:AKAP3:NDUFA9:ATN1:EMP1:SOX5:MIA2:CTAGE5:GTF2A1:MOAP1:TCF12
TF_targets	AML1_01	268	25	3.196255595416016e-05	0.0002910023751050403	MPL:FOXD2:DNASE2B:HIPK1:NRAS:CTSK:ARNT:ARHGEF2:SEMA4A:BGLAP:CREM:TLL2:GRK5:BTG4:C11orf88:REXO2:AKAP3:NDUFA9:ATN1:EMP1:SOX5:MIA2:CTAGE5:GTF2A1:MOAP1:TCF12
TF_targets	TAL1BETAE47_01	252	24	3.26552988427089e-05	0.00029293723961841805	VWA5B1:LRRC8C:UBQLN4:PMF1:NHLH1:CDH23:SOX5:FNDC3A:SIX6:MOAP1:TCF12:HNF1B:KCNJ2:C19orf57:AFF3:SCN5A:GNAI2:FOXP1:CDH6:MAP7:ARL4A:HOXA11:CEP41:TMEM71
TF_targets	TGTTTGY_HNF3_Q6	744	51	3.319033344364464e-05	0.00029342178841482944	RPE65:LPHN2:GOLPH3L:TMOD4:ADAM15:RBP3:HABP2:DUSP8:EHF:PEX16:TPI1:SOX5:HOXC13:HOXC11:ULK1:GTF2A1:ESRP2:GLP2R:EPN2:IKZF3:BRCA1:RPS6KB1:KCNJ2:UBALD2:SLC14A1:PAFAH1B3:PUM2:BCL11A:DCTN1:AFF3:CTDSP1:WBP2NL:ENTPD3:TMEM115:FOXP1:ATP2C1:GPRIN3:TERT:CDH6:NIPBL:DHX29:SKIV2L2:MARCH3:SNCB:TAPBP:HCRTR2:ESR1:HOXA10:HOXA11:YWHAG:CALD1

TF_targets	T3R_Q6	254	24	3.7100396407 65376e-05	0.0003233034 544095542	LDLRAP1:ZNF362:SEMA6C:CREM:APBB1:ZNF143:SOX5:DAO:SIAH3:PLA2G4B:ESRP2:RNF112:CXCL17:BPIFA2:SYNGR1:CACNA 2D2:MAP3K13:FGF12:EIF4E:RELL2:SNCB:RGL2:NYAP1:CNBD1
TF_targets	AP1_01	271	25	3.8435059996 027e-05	0.0003302167 1264192213	HCN3:RIT1:LMNA:CDH23:REXO2:ULK1:KCNK10:ZNF771:CALB2:ZPBP2:VAT1:PSMD12:TUBB4A:FAM49A:SLC11A1:SYNGR1:TG FBR2:ABCF3:FGF12:HDAC3:RELL2:SNCB:CDKN1A:YWHAG:RB1CC1
TF_targets	CTTTAAR_UNKNOWN	990	63	3.9813642312 5982e-05	0.0003334890 711290851	NBL1:TMEM57:ZNF362:TXNDC12:CSDE1:GJA5:GOLPH3L:PRUNE:LMNA:CDH23:HECTD2:GRK5:EHF:SNX15:CRTAM:ERC1:EMP 1:SOX5:HOXC13:LGR5:CCER1:C12orf75:TNFRSF19:PCDH9:NRL:STXBP6:FOXG1:MIA2:CTAGE5:FKBP3:GTF2A1:NIPA2:TRPM1: HAPLN3:IKZF3:VAT1:GJC1:KIF19:PPP4R1:ERF:ITPRIPL1:AFF3:HYAL2:DVL3:MAP3K13:STIM2:SLC6A18:EDN1:PI16:HOXA10:JAZ F1:NEUROD6:YWHAG:ABCB1:GNG11:PRKAR2B:GPR22:TSGA13:ASH2L:RPL7:RDH10:CNTLN:PAPPA:PBX3
TF_targets	ACTAYRNNNCCCR_UNKNOWN	464	36	3.9909347856 4315e-05	0.0003334890 711290851	CSDE1:FAM63A:PRUNE:SCAMP3:FDPS:UBQLN4:TRUB1:IPO7:LINC00610:COMMD9:DDX25:USP5:KDELC1:BIVM:KLHL28:FAM 179B:ZFYVE1:NTAN1:BRCA1:RALBP1:NFKBIB:GSK3A:C19orf68:GRWD1:TTLL4:CHMP4B:ZNFX1:NPRL2:CYB561D2:XXcos-LUCA11.4:AP2M1:RPP40:TUBB:YWHAG:BET1:RPL7:RDH10:NUDCD1
TF_targets	TGCTGAY_UNKNOWN	539	40	4.1619909266 330587e-05	0.0003379723 961648653	SZT2:DNAJB4:DNASE2B:FAM63A:PRUNE:NHLH1:F5:SNX15:KCNA1:ULK1:WBP4:SIAH3:MOAP1:CHRNB4:TBC1D10B:PITPNC1: PKN1:NPHS1:CEACAM1:OSR1:ITPRIPL1:PKP4:MAPK8IP2:VGLL4:CGGBP1:ZNF654:DHFRL1:SAMD7:ABCF3:MAP3K13:PCDHGC 3:DIAPH1:SNCB:LRRC16A:GSTA2:PTP4A1:YWHAG:TMEM71:KCNV2:CNTLN
TF_targets	MSX1_01	177	19	4.2102708080 92132e-05	0.0003379723 961648653	DNAJB4:HABP2:POSTN:FOXG1:ZNF770:ADAM11:KCNJ2:FSCN2:MPPE1:AFF3:WBP2NL:FOXP1:KCNAB1:PCDHGC3:AIG1:HOXA 11:NEUROD6:CEP41:ADAMTSL1
TF_targets	PPARA_02	133	16	4.2108036243 49142e-05	0.0003379723 961648653	ZBTB17:TMEM125:STIL:SCAMP3:SMG5:TMEM79:MIA2:CTAGE5:LINS:KPNB1:C19orf57:RFX1:ERF:DCTN1:CTDSP1:CREB3L2:C DH17
TF_targets	RCGCANGCGY_NRF1_Q6	952	61	4.3209266398 34118e-05	0.0003423071 75363482	ICMT:ZBTB17:VAV3:CSDE1:SIKE1:HORMAD1:CKS1B:SCAMP3:RAB18:GRK5:PEX16:ASRGL1:MRPL49:LETMD1:SLC25A3:UCHL3 :GTF2A1:ZNF770:MTFMT:ANP32A:PDXDC1:ESRP2:B9D1:MAPK7:VAT1:UTP18:RALBP1:NAPG:PHLPP1:SAFB:PEX11G:CD320:T ECR:PPP1CB:ZNF638:TTLL4:DAW1:PCNA:CDS2:ESF1:NDUFAF5:RTCB:ARFGAP3:RASSF1:ZMYND10:NPRL2:CYB561D2:TMEM1 15:XXcos-LUCA11.4:ATP2C1:ABCF3:FGF12:STIM2:IPO11:CKS1B:TMEM161B:ZFAND3:ZCWPW1:MEPCE:RPL7:RDH10:CHMP5:TSTD2
TF_targets	OCT1_Q5_01	274	25	4.6064439943 364153e-05	0.0003602475 4314682223	ZNF362:NRAS:SEMA6C:TLL2:EHF:SYVN1:C12orf57:PTHLH:HOXC11:NRL:FOXG1:TCF12:SH3GL3:HNF1B:ALK:KRTAP8-1:FOXP1:MRAS:FGF12:ISL1:FCHSD1:HOXA10:CEP41:TAS2R40:LYN
TF_targets	TTANTCA_UNKNOWN	962	61	5.8065431525 49408e-05	0.0004442121 057475857	MFSD2A:TCEANC2:LPHN2:TRIM33:GOLPH3L:NHLH1:ABL2:CREM:REEP3:TTC12:TPI1:EMP1:SOX5:RASSF8:HOXC11:CCER1:UL K1:WBP4:PCDH9:UCHL3:DCAF11:NIPA2:GRIN2A:RABEP1:SLC47A1:STAC2:ZPBP2:ADAM11:SPAG9:PITPNC1:DNAJB1:GRWD1: ALK:ITPRIPL1:IL18RAP:ATP5L2:VGLL4:CMTM8:RASSF1:FOXP1:ZNF654:A4GNT:RASA2:MAP3K13:FGF12:ATP10D:GPRIN3:MTT P:CDH9:NIPBL:ISL1:CPEB4:EDN1:TAPBP:CPNE5:SOD2:JAZF1:ZCWPW1:MEPCE:GIMAP5:ADAMTSL1
TF_targets	AP1_C	278	25	5.8347507817 5262e-05	0.0004442121 057475857	ADAM15:HCN3:RIT1:LMNA:CDH23:REXO2:EMP1:NUAK1:ULK1:PCDH9:ZNF771:CALB2:VAT1:PITPNC1:TUBB4A:SYNGR1:AP2 M1:ABCF3:HDAC3:PDGFRB:TTC1:SNCB:AIG1:HOXA11:RB1CC1
TF_targets	IRF_Q6	245	23	5.8985541910 74498e-05	0.0004442121 057475857	F3:HIPK1:ADAM15:UBE2L6:SLC11A2:TNFSF13B:MIA2:CTAGE5:CYB5B:IFI35:GJC1:NPEPPS:TCEB3B:HPCAL1:BCL11A:ZBP1:ISL1 :PCDHGC3:PDGFRB:CPEB4:TUBB:TAPBP:ESR1:KLF14
TF_targets	YGTCCTTGR_UNKNOWN	97	13	7.0686139789 86029e-05	0.0005224054 859319224	SYT13:ATN1:RASSF8:ESRP2:ADAM11:KCNJ16:ST8SIA5:CHST8:HDAC3:RELL2:SNCB:YWHAG:CNBD1
TF_targets	MEF2_Q6_01	248	23	7.1081402184 1796e-05	0.0005224054 859319224	TMEM57:ZNF362:LRRC8C:HIPK1:TRIM33:SOX5:WBP4:KPNA3:PCDH9:FITM1:ADAM11:DCTN1:SCN3A:CPT1B:FOXP1:DZIP1L:F GF12:NIPBL:ESR1:JAZF1:LYN:TMEM71:CNTLN
TF_targets	AP1_Q4_01	266	24	7.7183332538 29162e-05	0.0005474631 726553243	NRAS:ADAM15:HCN3:RIT1:CDH23:REXO2:EMP1:CALB2:ZPBP2:TUBB4A:FAM49A:SYNGR1:AP2M1:ABCF3:FGF12:MCTP1:HDA C3:RELL2:TTC1:SNCB:CDKN1A:HOXA11:RB1CC1:PAPPA
TF_targets	OCT_Q6	266	24	7.7183332538 29162e-05	0.0005474631 726553243	ZNF362:NRAS:SEMA6C:TLL2:EHF:SYVN1:C12orf57:PTHLH:HOXC11:SIAH3:NRL:FOXG1:TCF12:SH3GL3:HNF1B:ALK:FOXP1:MR AS:FGF12:FCHSD1:HOXA10:CEP41:TAS2R40:LYN
TF_targets	OCT1_06	266	24	7.7183332538 29162e-05	0.0005474631 726553243	HIPK1:NRAS:CTSK:SEMA6C:HABP2:PRKRIR:SOX5:HOXC11:PPP1R36:HNF1B:TCEB3B:CNFN:PKP4:SCN3A:KRTAP8-1:FOXP1:FGF12:SLC4A4:GPRIN3:EIF4E:C5orf20:HOXA11:NEUROD6:CD36

TF_targets	CREB_Q4_01	217	21	7.87277345647196e-05	0.0005519990584422869	CDC42:RUSC1:CREM:ZFYVE27:LGR5:GTF2A1:MESDC1:RNF166:SPAG9:OSR1:DCTN1:PKP4:MAP3K13:SENP2:AREG:AREGB:TRMT10A:NEUROD6:CREB3L2:GEM:RBM18:SLC25A25
TF_targets	TGCGCANK_UNKNOWN	557	40	8.497248352960663e-05	0.0005879451169328641	ICMT:TXNDC12:SIKE1:RAB18:REEP3:ASRGL1:PRKRIR:C11orf71:RBM7:LPCAT3:DLEU1:ABHD13:NUMB:GTF2A1:MTFMT:PDXDC1:B9D1:RPL19:VAT1:TUBD1:RPS6KB1:RALBP1:SAFB:TECR:EPS15L1:OSR1:CREB1:DAW1:ZNFX1:NDUFA6:RPL14:NPRL2:CYB561D2:XXcos-LUCA11.4:CNBP:ARMC8:FGF12:COQ2:ZCWPW1:MEPCE:CHMP5
TF_targets	OCT1_B	268	24	8.674600085894716e-05	0.0005879451169328641	ZNF362:NRAS:TLL2:EHF:C12orf57:PTHLH:SIAH3:KCTD12:NRL:FOXG1:TRPM1:TCF12:SH3GL3:HNF1B:ALK:FOXP1:MRAS:FGF12:HOXA10:HOXA13:NEUROD6:CEP41:TAS2R40:LYN
TF_targets	MYB_Q5_01	268	24	8.674600085894716e-05	0.0005879451169328641	ZNF362:CSDE1:FAM63A:PRUNE:FCRLA:SEC16B:FMOD:GRK5:SYVN1:USP5:SOX5:PCDH9:LRFN5:TCF12:SPAG9:C19orf57:XPO1:HES6:SLC37A1:WBP2NL:IFT57:FGF12:NEUROD6:GPR22
TF_targets	SPZ1_01	235	22	8.807570049664585e-05	0.0005903975527797139	TMEM57:ZNF362:CUL2:GRK5:ORAI1:GTF2A1:HNF1B:STAC2:KPNB1:UTP18:PITPNC1:BCL11A:XPO1:DCTN1:GNAI2:HYAL2:CGGBP1:EIF4E:HOXA10:NYAP1:KCNV2:ADAMTSL1
TF_targets	GATA6_01	271	24	0.00010307712456870807	0.0006760972686764723	PHC2:NRAS:SFRP5:SYVN1:MOGAT2:POU2AF1:CDON:ENO2:SOX5:PCDH9:C15orf26:RNF112:SERPINB4:GREB1:PUM2:IGFBP5:FOXP1:MTTP:CARD6:ISL1:LECT2:CPA1:TMEM71:CNTLN
TF_targets	AP1FJ_Q2	271	24	0.00010307712456870807	0.0006760972686764723	LMNA:REXO2:ORAI1:PCDH9:KCNK10:TCF12:ZNF771:ZPBP2:PITPNC1:ST8SIA5:GRIN2D:DSTN:ZNFX1:TGFBR2:FGF12:DIAPH1:HDAC3:RELL2:PDGFRB:SNCB:ITGB8:HOXA11:UBQLN1:PAPPA
TF_targets	HNF6_Q6	238	22	0.00010606088995007583	0.0006882674773355985	LPHN2:NRAS:HABP2:MTMR6:SIAH3:GRIN2A:PPP1CB:DCTN1:UCKL1:ZPLD1:ASTE1:NEK11:KCNAB1:CDH9:CDH6:NIPBL:CCNO:SLC12A2:EDN1:HOXA11:JAZF1:CNBD1
TF_targets	USF2_Q6	255	23	0.00010830968756059758	0.0006954622043364686	RPL22:UBIAD1:EBNA1BP2:WDR65:TXNDC12:WDR77:NRAS:CSDE1:SCAMP3:RUSC1:PSEN2:NID1:REEP3:ALDH18A1:IPO7:WEE1:SLC43A1:ARL2:LRFN4:REXO2:HOXC13:HOXC12:HOXC11
TF_targets	AREB6_02	256	23	0.00011484716384754368	0.0007222347417216665	TXNDC12:DNASE2B:AMPD1:FDPS:EHF:POU2AF1:LPCAT3:LCP1:SMG1:EPN2:HNF1B:VAT1:ADAM11:OSR1:DCTN1:AFF3:SCN5A:ARMC8:OCLN:EDN1:MAP7:NYAP1:ZNF467
TF_targets	HNF1_Q6	256	23	0.00011484716384754368	0.0007222347417216665	F13B:HABP2:C13orf35:FOXG1:MIA2:CTAGE5:TCF12:HNF1B:ADAM11:PRODH2:FAM49A:PPP1CB:BCL11A:RBMS1:SLC4A4:AFM:MTTP:CFI:ISL1:SLC12A2:HOXA10:KLF14:CDH17:CNTLN
TF_targets	TCF11MAFG_01	207	20	0.00011784344793341564	0.0007284708561493648	ARHGEF11:HECTD2:REXO2:SOX5:PCDH9:ERO1L:GTF2A1:ARPP19:RABEP1:NPEPPS:SLC11A1:FOXP1:CD200R1:FGF12:SNCB:TUBB:PTP4A1:POPDC3:JAZF1:NEUROD6
TF_targets	TAATTA_CHX10_01	823	53	0.00011822723730948708	0.0007284708561493648	TAL1:DNAJB4:DNASE2B:CLK2:ARHGEF2:PMF1:SOAT1:ARL5B:ZNF488:REEP3:HABP2:SOX5:HOXC12:PRKAB1:SIAH3:TNFSF13B:NRL:FOXG1:MIA2:CTAGE5:LRFN5:STAC2:ADAM11:PITPNC1:TTC39C:TUBB4A:CHST8:PRODH2:HPCAL1:ALK:AFF3:KRTAP8-1:RTCB:RASA2:CDH6:PPAP2A:OCLN:FCHSD1:CPEB4:LRRC16A:TUBB:FLOT1:IER3:POPDC3:AIG1:HOXA11:CLDN4:CD36:GPR22:CEP41:KLF14:CALD1:CA2:CNTLN
TF_targets	AP4_Q5	274	24	0.00012209368051892578	0.0007373974764014329	TRIM62:TXNDC12:GOLPH3L:RIT1:ARHGEF2:B3GALNT2:OPCML:TNFRSF19:RASL12:ANP32A:MESDC1:VAT1:RPS6KB1:KCNJ2:UBALD2:MBP:INSR:GRIN2D:RTKN:CHMP4B:TGFBR2:MAP3K13:ZFAND3:ESR1
TF_targets	NRF2_01	274	24	0.00012209368051892578	0.0007373974764014329	MED8:SZT2:KTI12:NRAS:CKS1B:UHMK1:ZBTB41:ARL5B:SYVN1:EMG1:PHB2:TFCP2:CD2BP2:PSMD12:MEGF8:TYMP:GNAI2:ACAD11:UBA5:NIPBL:RPL37:CKS1B:CPEB4:VPS52:RPS18
TF_targets	MYCMAX_01	258	23	0.0001289829561138923	0.0007713686591124931	UBIAD1:NRAS:CSDE1:IPO7:SLC43A1:REXO2:HOXC11:DCAF11:MIA2:CTAGE5:KLHL28:FAM179B:ZNF771:DCTPP1:ESRP2:OSR1:NCL:CYP2D6:DAZL:IFRD2:EIF4E:DIAPH1:MAP7:HOXA11
TF_targets	TCF4_Q5	242	22	0.00013508158109140013	0.0007999977132597482	TRIM33:FAM63A:PRUNE:SCNM1:LYSMD1:SCAMP3:TLL2:HABP2:UCP2:PRKRIR:LPCAT3:SOX5:HOXC11:TNFRSF19:TRPM1:PANK2:EIF4E:CDX1:HOXA10:HOXA11:ZMAT4:CNTLN
TF_targets	CEBP_01	276	24	0.00013644633468176136	0.0008003102322680234	NRAS:FAM63A:PRUNE:OPALIN:PEX16:SOX5:SIAH3:KPNA3:PCDH9:LRFN5:ARMC5:CACNB1:HPCAL1:PPP1CB:RBMS1:IGFBP5:PER2:CYP2D6:FOXP1:CPEB4:HOXA10:HOXA11:NUDCD1:CNTLN
TF_targets	CATTGTYY_SOX9_B1	367	29	0.00015787881378021161	0.0009172007276755151	ZNF362:DENND2C:SEMA6C:ADAM15:GRK5:WEE1:SOX5:KPNA3:CORO1A:ANKRD11:SPAG9:MBP:CHST8:GREB1:FAM49A:BCL11A:RBMS1:PER2:OSBP2:HYAL2:EIF4E:CDX1:EDN1:CDKN1A:ZFAND3:HOXA10:GNG11:CALD1:RDH10
TF_targets	IRF1_Q6	262	23	0.0001619639919924652	0.0009233461225738669	NRAS:CTSS:ADAM15:CUL2:CSRNP2:TNFRSF19:PCDH9:TNFSF13B:FOXG1:EPN2:TUBD1:HPCAL1:FAM49A:VGLL4:ATP2C1:FGF12:CDH6:PCDHGC3:CDX1:TAPBP:HOXA10:HOXA13:CALD1

TF_targets	STAT1_02	262	23	0.0001619639919924652	0.0009233461225738669	SZT2:TXNDC12:NRAS:SIKE1:FAM63A:SCNM1:LYSMD1:CKS1B:UHMK1:ZBTB41:LRFN4:DDX25:DDX47:CSRNP2:FOXG1:SMPD3:GSK3A:ERF:ZNFX1:CKS1B:TTC1:SYNE1:BET1:NUDCD1
TF_targets	TAL1BETAITF2_01	264	23	0.0001810979299577441	0.0010228679377242954	MACF1:LPHN2:DDAH1:UBQLN4:NHLH1:CDH23:SOX5:FNDC3A:FRMD6:MOAP1:TCF12:DNAH9:KCNJ2:C19orf57:AFF3:FBXO36:FOXP1:MAP7:ARL4A:HOXA11:INMT:NEUROD6:TMEM71
TF_targets	OCT_C	266	23	0.00020220411798474653	0.0011213137451881398	ZNF362:NRAS:SEMA6C:TLL2:EHF:C12orf57:HOXC11:SIAH3:NRL:STXBP6:FOXG1:SH3GL3:HNF1B:ALK:PER2:KRTAP8-1:MRAS:FGF12:ISL1:FCHSD1:HOXA10:TAS2R40:LYN
TF_targets	PAX2_02	266	23	0.00020220411798474653	0.0011213137451881398	CDC20:LPHN2:HIPK1:PHGDH:CRB1:ZNF143:HOXC12:WBP4:KCNK10:TCF12:RPS6KB1:CHST8:ERF:OSR1:XPO1:PER2:IFRD2:CACNA2D2:LSG1:RGL2:HOXA10:NYAP1:ADAMTSL1
TF_targets	ACTWSNACTNY_UNKNOWN	108	13	0.00021348171263555962	0.0011630843532147313	NUMB:TBC1D10B:ARMC5:KCNJ2:C19orf68:GRWD1:BCL11A:XPO1:GNAI2:TMEM115:AP2M1:TUBB:COPG2
TF_targets	ZID_01	267	23	0.0002135499140328687	0.0011630843532147313	TMEM57:CSDE1:ANXA9:SEMA6C:FAU:MRPL49:STXBP6:KCNK10:SIAH1:PHLPP1:ERF:TMEM145:BCAM:CHMP4B:SMDT1:FOXP1:ZNF654:AP2M1:ISL1:TSC22D4:RB1CC1:CNTLN:PAPPA
TF_targets	PAX4_01	268	23	0.00022545395485538267	0.001206063161297642	ZFYVE27:LINS:GRIN2A:SMG1:KPNB1:CIC:MEGF8:BCL11A:XPO1:CTDSP1:FBXO36:PER2:CPT1B:CHKB:ZNF654:RAB43:NIPBL:CDX1:ITGB8:HOXA10:EVX1:KLF14:ADAMTSL1
TF_targets	YY1_01	251	22	0.00022737256319545709	0.001206063161297642	DNAJB4:DNASE2B:DDAH1:NRAS:CSDE1:ASH1L:YY1AP1:DAP3:CREM:SIAH3:GTF2A1:ZPBP2:RBMS1:SCN3A:CTDSP1:DSTN:GNAI2:ZNF654:CDH6:PEX7:CALD1:PBX3
TF_targets	MYOD_Q6	251	22	0.00022737256319545709	0.001206063161297642	FBLIM1:NBL1:C1orf210:ELOVL1:LRRC8C:GOLPH3L:ARHGEF2:NHLH1:ABL2:CDON:ESRP2:ADAM11:NPEPPS:FSCN2:FAM49A:OSR1:PKP4:HES6:SYNGR1:MAP3K13:ARL4A:JAZF1
TF_targets	SP1_Q2_01	252	22	0.00024045164768614713	0.0012536367956286303	CLSTN1:TAL1:C1orf52:HIPK1:PHLDA3:CTR9:MRPL49:ERC1:PTHLH:CSRNP2:TCF12:ANP32A:LINS:GRIN2A:SPAG9:UTP18:S1PR5:CREB1:OSBP2:HYAL2:CGGBP1:FGF12
TF_targets	ISRE_01	252	22	0.00024045164768614713	0.0012536367956286303	ADAM15:ERC1:SLC11A2:KPNA3:TNFSF13B:MIA2:CTAGE5:CYB5B:IKZF3:TBX21:KCNJ16:TECR:HPCAL1:BCL11A:ANAPC1:ZBP1:KRTAP8-1:CACNA2D2:PCDHGC3:TAPBP:ESR1:YWHAG:KLF14
TF_targets	TGCCAAR_NF1_Q6	726	47	0.0002491377076588182	0.001287915268405755	FBLIM1:MFSD2A:MPL:DNAJB4:RIT1:LMNA:PAQR6:SOAT1:FMOD:HECTD2:DMBT1:BBOX1:EHF:TPI1:PTHLH:POSTN:SIAH3:PRPF39:PAPLN:MOAP1:ANP32A:TARSL2:CD2BP2:SPAG9:CXCL17:FAM49A:OSR1:PUM2:MFF:PER2:ZNFX1:SYNGR1:ZNF654:ARMC8:KLHL2:CPEB4:EDN1:C4A:PI16:PEX7:ESR1:HOXA10:GPR22:COPG2:CALD1:TMEM71:PALM2
TF_targets	ICSBP_Q6	253	22	0.0002541880961004631	0.0013029809968175	CTSS:ARL5B:EMP1:SLC11A2:TNFRSF19:TNFSF13B:CYB5B:ZPBP2:IFI35:HPCAL1:BCL11A:PKP4:ZBP1:VGLL4:XRN1:NIPBL:PCDHGC3:CPEB4:TAPBP:ESR1:HOXA13:GPR22
TF_targets	RGAGGAARY_PU1_Q6	511	36	0.00027054757083832425	0.001375283485094815	ACOT7:TIE1:ADAM15:FCRLA:SEC16B:POU2AF1:PTPN6:SOX5:PTHLH:NCKAP1L:PCDH9:ZFYVE1:PSEN1:TCF12:ANP32A:LINS:TBC1D10B:SEPT1:SEPT1:UBALD2:PKN1:ITPRIPL1:ZNFX1:CYB5R3:VGLL4:HYAL3:NAT6:CD200R1:NIPBL:C5orf20:NDFIP1:CPEB4:TUBB:ZFAND3:EVX1:LYN:CHMP5
TF_targets	EN1_01	111	13	0.0002807093478232901	0.0014027111508534463	NRAS:CREM:TTC12:HOXC11:FNDC3A:PCDH9:RNFT1:RPL38:OSR1:RTKN:ISL1:HOXA11:RDH10
TF_targets	P53_02	255	22	0.00028374537812589275	0.0014027111508534463	ZNF362:VAV3:HIPK1:SOAT1:SYT13:SOX5:RAP1B:FOXG1:GRIN2A:PITPNC1:NAPG:BCAM:BCL11A:ZNF654:NIPBL:CDKN1A:RAB44:CNR1:HOXA11:CALD1:NUDCD1:NRBP2
TF_targets	NFKAPPAB_01	255	22	0.00028374537812589275	0.0014027111508534463	TAL1:ASH1L:YY1AP1:DAP3:GRK5:EHF:OPCML:SOX5:SLC11A2:ORAI1:ACAN:SMPD3:NFKBIB:C19orf68:GRIN2D:RBMS1:CREB1:BFSP1:CXCL6:NIPBL:SLC12A2:HOXA11
TF_targets	RGAANNTTC_HSF1_01	455	33	0.00028514128312430713	0.0014027111508534463	ACOT7:SPEN:LRRC8B:HIPK1:ADAM15:ZBTB41:CUL2:CREM:TRPC6:HSPA8:PTHLH:KLHL28:FAM179B:PSEN1:MESDC1:NFATC3:SPAG9:EPG5:DNAJB1:CHST8:GREB1:XPO1:IGFBP5:DAZL:CACNA2D2:ZNF654:ATP2C1:RAB6B:TRMT10A:POPDC3:HSPB1:YWHAG:TMEM71
TF_targets	HNF4ALPHA_Q6	273	23	0.00029418960451040943	0.001435645270010798	C1orf210:LPHN2:SCNM1:LYSMD1:SEC16B:GRK5:PEX16:ZFYVE1:PAPLN:KCNK10:LINS:TARSL2:MYLPF:N4BP1:ESRP2:HNF1B:RBMS1:PPARA:RBP2:MTTP:CFI:ZCWPW1:MEPCE
TF_targets	TAL1ALPHAE47_01	256	22	0.0002996246974091307	0.0014505640112664263	MACF1:LPHN2:ARHGEF2:UBQLN4:PMF1:NHLH1:CDH23:HOXC12:SIX6:TCF12:KCNJ2:AFF3:HES6:SCN5A:FOXP1:CDH6:MARCH3:MAP7:ARL4A:HOXA11:NEUROD6:TMEM71

TF_targets	RYAAAKNNNNNNTTGW_UNKNOWN	84	11	0.0003074248995737313	0.0014766077853541426	HIPK1:GRK5:PC:SOX5:HOXC11:SMG1:PKP4:GSK3B:CALD1:NUDCD1:ADAMTSL1
TF_targets	AP1_Q2_01	274	23	0.0003099593505352391	0.0014771500298944988	C1orf210:NRAS:ADAM15:HCN3:RIT1:LMNA:REXO2:ENO2:NUAK1:ORAI1:PCDH9:KCNK10:TCF12:PDZD9:TUBB4A:EIF4E:TTC1:SNCB:CDKN1A:PTP4A1:CLDN4:RB1CC1:ADAMTSL1
TF_targets	NRF2_Q4	257	22	0.00031627839250931717	0.0014955799955866935	TMEM57:ADAM15:CDH23:REXO2:NUAK1:WBP4:MIA2:CTAGE5:GTF2A1:ZNF771:RABEP1:PSMD12:PITPNC1:TNRC6C:SLC11A1:PER2:CNBP:FGF12:FCHSD1:TTC1:TUBB:YWHAG:RB1CC1
TF_targets	YATTNATC_UNKNOWN	383	29	0.00032250718432484736	0.0015086733221758505	DNAJB4:OVGP1:SMG5:TMEM79:AGAP4:AGAP8:GRK5:EHF:TRPC6:SIAH3:FNDC3A:KCTD12:VAT1:PITPNC1:PKP4:WBP2NL:PLCXD2:RAB43:EIF4E:CDH9:MCTP1:CPEB4:LRRC16A:HCRTR2:HOXA10:EVX1:NEUROD6:CALD1:CALB1:CNTLN
TF_targets	EVI1_05	174	17	0.0003253190393726715	0.0015086733221758505	HIPK1:NRAS:TMOD4:HECTD2:EHF:SOX5:DCAF11:HNF1B:SEMG1:UCKL1:CDH9:ISL1:JAZF1:NEUROD6:GPR22:KLF14:CNTLN
TF_targets	NF1_Q6_01	275	23	0.0003264670139790365	0.0015086733221758505	FBLIM1:RPE65:DNAJB4:HIPK1:CSDE1:LMNA:SOAT1:BBOX1:LPCAT3:POSTN:ANP32A:NFATC3:CACNB1:ALK:RBP2:CFI:KLHL2:PCDHGC3:ITGB8:JAZF1:COPG2:NUDCD1:ADAMTSL1
TF_targets	NFKAPPAB65_01	241	21	0.00033773462116018564	0.001549008412839949	ASH1L:YY1AP1:DAP3:ARHGEF2:GRK5:EHF:SOX5:PTHLH:ORAI1:ACAN:SMPD3:NFKBIB:RBMS1:CREB1:CTDSP1:CYP2D6:STX19:FGF12:SLC12A2:FLOT1:IER3
TF_targets	AP4_Q6_01	259	22	0.00035203661151422014	0.001602554724057271	HOOK1:GOLPH3L:TMOD4:ARHGEF2:GRK5:HOXC12:FITM1:RASL12:KCNJ2:TUBB4A:ERF:GRIN2D:MYCN:ALK:RTKN:HES6:HYAL2:DVL3:MAP3K13:FGF12:CDH6:ISL1
TF_targets	FAC1_01	225	20	0.00035774643537782194	0.001616483893188677	ZNF362:HIPK1:SOX5:HOXC11:GTF2A1:IKZF3:BRCA1:RFX1:BCL11A:AFF3:RBMS1:FOXP1:FGF12:PDGFRB:TAPBP:ITGB8:GNG11:ZCWPW1:MEPCE:PAPPA
TF_targets	STTTCRNTTT_IRF_Q6	192	18	0.00036529117646101985	0.001638438365008986	SOX5:TNFSF13B:PSME1:TCF12:TCEB3B:IL12RB1:HPCAL1:BCL11A:CREB1:IGFBP5:ZBP1:VGLL4:CD200R1:TAPBP:ESR1:HOXA13:CLDN3:YWHAG
TF_targets	HNF1_01	244	21	0.00039840057347516973	0.0017440887713915296	PDZK1IP1:APCS:HABP2:ZNF143:BTG4:C11orf88:WBP4:HNF1B:ADAM11:BCL11A:RBMS1:TM4SF20:DZIP1L:SLC4A4:AFM:CFI:ISL1:LECT2:HOXA10:CALD1:CDH17
TF_targets	RACTNNRTTTNC_UNKNOWN	130	14	0.0003996468641973495	0.0017440887713915296	FAM63A:PRUNE:SCNM1:LYSMD1:POU2AF1:DDX25:CSRNP2:SMPD3:GSK3A:CHMP4B:ZNFX1:NEUROD6:BET1:CEP41
TF_targets	SRY_01	227	20	0.0004011046108375643	0.0017440887713915296	CLSTN1:NBL1:TMEM50A:TMEM57:ZNF362:MED8:SZT2:TAL1:NRAS:CSDE1:CTSK:RBP3:HECTD2:HABP2:GRK5:EHF:OPCML:KCNA1:SOX5:RASSF8
TF_targets	PPARA_01	37	7	0.0004013820139522287	0.0017440887713915296	HMGCS2:PEX16:ATN1:PAPLN:CACNB1:RPL19:CD36
TF_targets	WTTGKCTG_UNKNOWN	522	36	0.00040314183076427165	0.0017440887713915296	GPR153:ZNF362:TMEM125:RBP3:TLL2:PEX16:LPCAT3:C1S:SLC11A2:CCER1:SIAH3:DLEU1:GTF2A1:HERC2:ANP32A:ANKRD11:STAC2:KPNB1:PITPNC1:CCDC130:CHST8:CIC:XPO1:AFF3:PCNA:DAZL:FOXP1:NIPBL:ISL1:PCDHGC3:CDKN1A:HOXA10:CALD1:ADAMTSL1:PALM2:SLC25A25
TF_targets	CCAWYNNGAAR_UNKNOWN	147	15	0.00045105596239613995	0.0019376347680397563	MFSD2A:MED8:SZT2:GOLPH3L:CKS1B:NUCKS1:EMG1:PHB2:KPNB1:FAM49A:RBMS1:PCNA:VGLL4:GNAI2:CKS1B:TERF1
TF_targets	BACH1_01	264	22	0.0004573269924372443	0.0019508354222847483	NRAS:HCN3:LMNA:CDH23:REXO2:ENO2:NUAK1:PDZD9:ZNF771:PSMD12:TUBB4A:CHMP4B:SYNGR1:FGF12:EIF4E:DIAPH1:HDAC3:RELL2:SNCB:CDKN1A:RB1CC1:ADAMTSL1
TF_targets	HNF1_C	247	21	0.000468361645399997	0.0019840319700972094	ZNF362:PKLR:APCS:HABP2:ZNF143:IKBIP:APAF1:SIAH3:GRIN2A:HNF1B:ADAM11:UBALD2:MIOX:RASA2:AFM:ISL1:SLC12A2:HOXA10:KLF14:CDH17:CNTLN
TF_targets	RSRFC4_01	248	21	0.0004939452958800166	0.002077976761978001	GPR153:ZNF362:AMPD1:ZNF143:SOX5:WBP4:LCP1:KPNA3:PCDH9:FITM1:ADAM11:TTYH2:LSMEM2:FOXP1:FGF12:TRMT10A:CPEB4:ESR1:THBS2:KLF14:TMEM71
TF_targets	ATF_01	266	22	0.0005065769159768066	0.002102121896230286	CDC42:RCE1:LGR5:PCDH9:GTF2A1:PDXDC1:RNF166:SPAG9:SSTR2:RFX1:ERF:OSR1:DCTN1:CHMP4B:MAP3K13:IPO11:EVX1:NEUROD6:CLDN3:CALD1:GEM:SLC25A25
TF_targets	ZIC1_01	266	22	0.0005065769159768066	0.002102121896230286	ZNF362:SZT2:LMNA:FCGR3A:ZMYND11:ARL5B:KCNA1:HOXC11:UCHL3:KCNK10:LINS:UTP18:GRIN2D:KCNJ14:DUSP11:DCTN1:CGGBP1:ZNF654:HOXA10:YWHAG:TSC22D4:STAR

TF_targets	USF_C	284	23	0.0005133340941441874	0.002115768901540232	RPL22:TXNDC12:RUSC1:PSEN2:WEE1:HOXC13:HOXC12:HOXC11:KLHL28:GTF2A1:GRIN2A:ZNF771:RPL19:IKZF3:UTP18:OSR1:C21orf91:CYP2D6:DAZL:HYAL2:EIF4E:WDR46:PFDN6
TF_targets	MEF2_02	232	20	0.000530044958233765	0.0021527325976753134	GPR153:TMEM57:ZNF362:TRIM33:AMPD1:PRKRIR:SOX5:KPNA3:FITM1:IKZF3:ADAM11:CPT1B:FOXP1:DZIP1L:FGF12:TRMT10A:CPEB4:ESR1:LYN:TMEM71
TF_targets	CEBPB_01	267	22	0.0005328895446704465	0.0021527325976753134	CSDE1:EHF:LRFN4:C1S:DDX47:PDE1B:SIAH3:TNFSF13B:ACAN:TBC1D10B:ANKRD11:CACNB1:SPAG9:S1PR5:PPP1CB:AFF3:IGFBP5:C2CD2:FOXP1:KLHL6:PCDHGC3:CEP41
TF_targets	AP1_Q6_01	267	22	0.0005328895446704465	0.0021527325976753134	LMNA:REXO2:NUAK1:ORAI1:PCDH9:KCNK10:TBC1D10B:ZNF771:VAT1:PSMD12:PITPNC1:SLC11A1:VIL1:ZNFX1:SYNGR1:MCTP1:DIAPH1:PDGFRB:HOXA11:RB1CC1:ADAMTSL1:UBQLN1
TF_targets	TGACATY_UNKNOWN	671	43	0.0005502673827342206	0.0022083098912360167	HOOK1:DNAJB4:HIPK1:BCAS2:GOLPH3L:CREM:HABP2:SYT13:AKAP3:PTHLH:HOXC12:TNFRSF19:FOXG1:DTD2:LRFN5:HERC2:TRPM1:CORO1A:ESRP2:SMPD3:STAC2:RND2:ADAM11:PITPNC1:TTC39C:BCL11A:FBXO36:MCHR1:HYAL1:ZNF654:GSK3B:KCNAB1:FGF12:CXCL6:MAP7:PEX7:HOXA10:NEUROD6:PRKAR2B:GPR22:KLF14:CALD1:GEM
TF_targets	MGGAAGTG_GABP_B	775	48	0.000574280939275577	0.0022896168167196207	ELOVL1:SZT2:TXNDC12:EXTL2:NRAS:CTSS:FAM63A:CKS1B:ZBTB41:ARL5B:DMBT1:CTR9:HSPA8:EMG1:PHB2:DDX47:LCP1:ARPP19:TCF12:CD2BP2:SEPT1:SEPT1:NFATC3:PKN1:ERF:GRWD1:ITPRIPL1:CTDSP1:PER2:LIME1:CYB5R3:HYAL2:FOXP1:C3orf38:ATP2C1:NIPBL:RPL37:CKS1B:DIAPH1:CPEB4:VPS52:RPS18:RGL2:ZFAND3:PEX7:HOXA11:PILRB:PILRA:ZNF79:TOR4A
TF_targets	PR_02	135	14	0.0005862640348814794	0.002318407390847642	PHC2:AKAP3:SOX5:KCNK10:PDZD9:EPG5:MBP:DSTN:ZNF654:NIPBL:CPEB4:CDKN1A:CD36:ADAMTSL1
TF_targets	OCT1_Q6	269	22	0.000589103517346532	0.002318407390847642	ZNF362:SEMA6C:TLL2:EHF:SYVN1:C12orf57:SOX5:KPNA3:NRL:DTD2:SH3GL3:HNF1B:STAC2:SCN3A:KRTAP8-1:MRAS:NIPBL:ISL1:HOXA10:TAS2R40:LYN:PTPN3
TF_targets	TCCCRNNRTGC_UNKNOWN	217	19	0.0005971646569628185	0.002320193890110314	FAM189B:IPO7:CSRNP2:SMG1:SMPD3:KPNB1:MBP:GSK3A:C19orf68:IFRD2:TMEM115:ISL1:TTC1:BET1:EXOC4:RB1CC1:RPL7:RDH10:UBAC1
TF_targets	RSRFC4_Q2	217	19	0.0005971646569628185	0.002320193890110314	GPR153:TRIM33:AMPD1:ZNF143:SOX5:WBP4:KPNA3:PCDH9:FITM1:ADAM11:TTYH2:FGF12:TRMT10A:CPEB4:ESR1:THBS2:KLF14:LYN:TMEM71
TF_targets	WGTTNNNNNAAA_UNKNOWN	555	37	0.0006358193299806191	0.002443520544852732	ZNF362:DNAJB4:C1orf52:NRAS:TMOD4:RIT1:C10orf131:CC2D2B:EHF:HOXC13:HOXC11:TNFRSF19:FNDC3A:PCDH9:LRFN5:PAPLN:LINS:ADAM11:HPCAL1:ALK:XPO1:CTDSP1:RAB43:ATP2C1:FGF12:TRMT10A:CFI:FLOT1:IER3:CDKN1A:PTP4A1:ARL4A:HOXA11:INMT:SEPT7:YWHAG:CALD1:COL22A1
TF_targets	MYB_Q3	253	21	0.0006409234216007166	0.002443520544852732	ZNF362:CSDE1:FAM63A:PRUNE:LMNA:SEC16B:GRK5:OPCML:USP5:SOX5:PCDH9:LINS:SPAG9:XPO1:FOXP1:FGF12:NIPBL:C4A:HOXA11:NEUROD6:LYN
TF_targets	FOXM1_01	253	21	0.0006409234216007166	0.002443520544852732	GPR153:DNASE2B:ARHGEF11:ZBTB41:FAU:MRPL49:SOX5:CSRNP2:SIAH3:PCDH9:STXBP6:FOXG1:G2E3:TCF12:GSK3A:RTKN:NPHP3:KLHL2:ISL1:CEP41:KLF14
TF_targets	TGIF_01	254	21	0.0006744795894591921	0.002539707096111773	TMEM125:GOLPH3L:ASH1L:YY1AP1:DAP3:ABL2:LPCAT3:TNFRSF19:LHFP:FITM1:FKBP3:HNF1B:RPS6KB1:TUBB4A:DCTN1:SMDT1:MAP3K13:NIPBL:SLC12A2:KLF14:KIAA0020
TF_targets	E47_02	254	21	0.0006744795894591921	0.002539707096111773	TAL1:DDAH1:POU2AF1:TPI1:MESDC1:MYLPF:ESRP2:HNF1B:MEGF8:AFF3:HES6:SCN5A:HDAC3:RELL2:MGAT1:AIG1:ARL4A:CEP41:CALB1:COL22A1:CEL
TF_targets	HIF1_Q3	237	20	0.000693473575968795	0.002595207860987515	RPL22:TMEM57:MPL:TRIM33:C11orf71:RBM7:FKBP3:ERO1L:MESDC1:GRIN2A:ZNF771:UTP18:ERF:C19orf68:BCL11A:LMF2:NCAPH2:HYAL2:SNCB:NTMT1
TF_targets	CP2_02	255	21	0.0007095454801737436	0.002607365921120383	RNF207:GPR153:TMEM57:TRIM62:MOGAT2:PTHLH:STAC2:PKN1:DNAJB1:HPCAL1:CREB1:CHMP4B:TGFBR2:RASSF1:GSK3B:ATP10D:CLDN4:YWHAG:CALD1:LYN:PAPPA
TF_targets	WCAANNNYCAG_UNKNOWN	255	21	0.0007095454801737436	0.002607365921120383	NRAS:SCNM1:LYSMD1:ARHGEF2:HECTD2:RALBP1:PHLPP1:GRWD1:CREB1:FBXO36:NPRL2:CYB561D2:TMEM115:XXcos-LUCA11.4:PROS1:GTPBP8:TRMT10A:RPP40:NEUROD6:YWHAG:RPL7:RDH10
TF_targets	ZIC3_01	255	21	0.0007095454801737436	0.002607365921120383	ZNF362:TAL1:CTSK:LMNA:PAQR6:NHLH1:NUCKS1:ZMYND11:ARL5B:ANP32A:SAFB:MEGF8:GRIN2D:MYCN:BCL11A:RBMS1:CTDSP1:FOXP1:CETN3:HOXA10:HOXA11
TF_targets	POU1F1_Q6	238	20	0.0007309202818805891	0.0026698285745338883	LMNA:NHLH1:APBB1:PTHLH:STAC2:SARS2:MRPS12:PKP4:ZNFX1:VGLL4:SLC25A26:MAP3K13:FGF12:EIF4E:NIPBL:DIAPH1:FCHSD1:THBS2:ZCWPW1:MEPCE

TF_targets	MTF1_Q4	256	21	0.0007461766990412848	0.002709332061995141	ZNF362:SEMA6C:PAQR6:NUAK1:KCTD12:ANP32A:ZNF48:STAC2:SPATA32:PHLPP1:C19orf68:ANAPC1:CREB1:GNAI2:CNBP:HDAC3:RELL2:ZCWPW1:MEPCE:CEP41:UBAC1
TF_targets	CRX_Q4	275	22	0.0007897719793299346	0.0028506562567530186	LPHN2:LRIT1:BTG4:C11orf88:CLMP:KCNA1:FOXG1:MOAP1:ZPBP2:BCL11A:DCTN1:MERTK:EFHB:TRMT10A:NIPBL:PCDHGC3:CPEB4:SYNE1:MEPCE:CNTLN:ADAMTSL1:PALM2
TF_targets	TEL2_Q6	240	20	0.0008110709281829777	0.0029103133305389203	RPF1:EXTL2:CTSS:FAM63A:CKS1B:ZBTB41:CTR9:SYVN1:PTPN6:LCP1:TCF12:CD2BP2:CD79A:LIME1:HYAL2:CGGBP1:RAB43:CKS1B:PPIL1:LYN:TMEM71
TF_targets	CEBP_Q2_01	276	22	0.0008284045300497528	0.0029551272709377146	CSDE1:RGR:WEE1:SOX5:SIAH3:TNFSF13B:TCF12:ACAN:ASGR2:SPAG9:S1PR5:ERF:PPP1CB:C2CD2:FOXP1:MTTP:PCDHGC3:ESR1:HOXA10:YWHAG:CEP41:CALD1
TF_targets	CATRRAGC_UNKNOWN	140	14	0.0008421028395275063	0.002986527512277784	TMEM57:C14orf142:UBR7:TBC1D10B:NFATC3:TBX21:SPAG9:ZNF654:GPRIN3:DIAPH1:TSPAN17:HOXA11:SRRM3:ADAMTSL1
TF_targets	GGCKCATGS_UNKNOWN	67	9	0.0008822797343001588	0.003110928542908074	ZBTB17:GON4L:PRPF39:KCNK10:ESF1:NDUFAF5:ZFAND3:RPL7:RDH10
TF_targets	GGGYGTGNY_UNKNOWN	688	43	0.0009057440801244823	0.00317530970618353	TMEM50A:TIE1:PKLR:UBQLN4:ZBTB41:GRK5:APBB1:WEE1:SLC43A1:POU2AF1:ATN1:PCDH9:UCHL3:TGFB1I1:PLA2G15:VAT1:KPNB1:RALBP1:DNAJB1:GSK3A:PAFAH1B3:OSR1:BCL11A:XPO1:RTKN:LMF2:NCAPH2:VGLL4:GNAI2:ATP2C1:RAB6B:SENP2:ATP10D:TUBB:ZFAND3:SYNE1:ARL4A:CLDN3:GNG11:BET1:NYAP1:GPR22:IPPK
TF_targets	STAT5A_02	142	14	0.0009679859373223602	0.0033741224100950844	TAL1:TCEANC2:GJA5:CREM:DDX25:EMP1:PCDH9:FOXP1:IPO11:ARL4A:HOXA11:SEPT7:PILRB:KLF4
TF_targets	PEA3_Q6	262	21	0.0010021427205541527	0.0034733355655570063	TMEM57:ELOVL1:TXNDC12:EXTL2:HIPK1:TRIM33:GOLPH3L:CTSS:FAM63A:PRUNE:TNFAIP8L2:FCGR3A:SELP:DMBT1:IPO7:EHF:SLC43A1:LRFN4:POU2AF1:C12orf57:PTPN6
TF_targets	IRF2_01	128	13	0.0011021264402298824	0.0037982888618091994	ASH1L:YY1AP1:DAP3:CUL2:USP5:TNFSF13B:PSME1:HPCAL1:BCL11A:VGLL4:TAPBP:ESR1:BLK
TF_targets	GATA1_04	247	20	0.001154011338346856	0.0039547579572560805	DNAJB4:HIPK1:ALDH18A1:SFRP5:MOGAT2:ENO2:ATN1:HOXC12:TNFRSF19:SLC14A1:IFRD2:PLCXD2:ISL1:SYNE1:HOXA10:HOXA13:NEUROD6:CALD1:ADAMTSL1:PAPPA
TF_targets	CDPCR1_01	129	13	0.0011842678011802	0.0040171976656587765	NUCKS1:CUL2:CREM:KCNA1:EMP1:HOXC11:SIAH3:FOXG1:PLA2G15:KCNS1:FGF12:FCHSD1:HOXA10
TF_targets	TGASTMAGC_NFE2_01	195	17	0.0011854025898665242	0.0040171976656587765	ADAM15:RIT1:CDH23:ZNF771:CALB2:RABEP1:VAT1:PSMD12:TUBB4A:IL18RAP:ABCF3:EIF4E:HDAC3:RELL2:TTC1:SNCB:RB1CC1
TF_targets	S8_01	248	20	0.0012119118684344764	0.004084343865994645	PHC2:EHF:SOX5:PRKAB1:SIAH3:LCP1:LRFN5:TRPM1:LINS:GRIN2A:ADAM11:GRWD1:SCN3A:KCNS1:FOXP1:JAZF1:CD36:CALD1:CNTLN:SLC25A25
TF_targets	AACWWCAANK_UNKNOWN	146	14	0.0012675868744738323	0.004248505458401306	LPHN2:SOX5:TNFRSF19:NFATC3:IKZF3:GRWD1:AFF3:UCKL1:TMEM115:TRMT10A:ZCWPW1:MEPCE:RPL7:RDH10
TF_targets	AAANWWTGC_UNKNOWN	197	17	0.0013249194790357684	0.004386096163484918	NRAS:SEMA6C:PRKRIR:SOX5:DCAF11:SPAG9:BCL11A:DSTN:KRTAP8-1:FOXP1:GPRIN3:PDGFRB:ESR1:THBS2:TSC22D4:KLF14:PPP3CC
TF_targets	CEBPB_02	268	21	0.0013302094922044423	0.004386096163484918	ARNT:NHLH1:RGR:EHF:REXO2:SOX5:HOXC13:KPNA3:TRPM1:TCF12:ACAN:GLP2R:KCNJ2:ERF:PPP1CB:BCL11A:FOXP1:TRMT10A:RAB44:ESR1:UBQLN1
TF_targets	HEB_Q6	268	21	0.0013302094922044423	0.004386096163484918	ZNF362:MACF1:C1orf210:HOOK1:GOLPH3L:TMOD4:ARHGEF2:SOX5:RASSF8:HOXC12:FITM1:RASL12:MESDC1:TBC1D10B:KCNJ2:C17orf70:GRIN2D:ALK:RTKN:DVL3:TSC22D4
TF_targets	CREB_Q2	270	21	0.0014582306270393849	0.004782369260720564	CDC42:RUSC1:CREM:ALDH18A1:ZFYVE27:LGR5:GTF2A1:MESDC1:SSTR2:CCDC130:ERF:OSR1:PKP4:IFT57:MAP3K13:SENP2:PTP4A1:EVX1:NEUROD6:FAM167A:GEM
TF_targets	ETS_Q4	252	20	0.001469004473314035	0.00479193972578375	EXTL2:GJA5:ZBTB41:LRFN4:LCP1:TCF12:CORO1A:CD2BP2:SEPT1:SEPT1:PKN1:CD79A:ITPRIPL1:SCN3A:LIME1:HYAL2:FOXP1:RAB43:NIPBL:HOXA10:LYN
TF_targets	CCCNNGGGAR_OLF1_01	326	24	0.001480688923456927	0.004804362996323008	ANXA9:FCRLA:GRK5:POU2AF1:CDON:OPCML:ERC1:PTHLH:MESDC1:TBC1D10B:SMPD3:VAT1:GJC1:TBX21:KIF19:GRIN2D:SPATC1L:STIM2:CCNO:NDFIP1:SNCB:ZFAND3:HOXA11:CA2

TF_targets	PR_01	149	14	0.0015400880 03110665	0.0049706544 01574104	TIE1:NHLH1:PCDH9:PDZD9:EPG5:MBP:TUBB4A:GREB1:RTKN:DSTN:ZNF654:NIPBL:CD36:ADAMTSL1
TF_targets	LYF1_01	272	21	0.0015966194 587957935	0.0051259887 887654424	ANGPTL7:ANXA9:SCNM1:LYSMD1:C10orf131:CC2D2B:ENO2:HOXC11:TNFRSF19:ZFYVE1:MYLPF:STAC2:TBX21:SPAG9:IMPA2:ATP2C1:RASA2:CFI:PDGFRB:EVX1:CALD1:CNTLN
TF_targets	ATF3_Q6	254	20	0.0016140677 084562443	0.0051548759 27530413	CDC42:RUSC1:CREM:RCE1:AKAP3:LGR5:ORAI1:TNFRSF19:RNF166:ADAM11:PITPNC1:RFX1:OSR1:DCTN1:PKP4:MAP3K13:AREG:AREGB:ISL1:CDKN1A:GEM
TF_targets	OCT1_05	256	20	0.0017711221 899950865	0.0056270027 91130222	SEMA6C:TLL2:EHF:SOX5:HOXC11:NRL:LRFN5:TRPM1:TCF12:SH3GL3:HNF1B:KRTAP8-1:MRAS:ISL1:CDX1:EDN1:HOXA10:CEP41:TAS2R40:LYN
TF_targets	IRF7_01	257	20	0.0018543873 231155604	0.0058308055 00518	ZNF362:MPL:CUL2:SOX5:PDE1B:KPNA3:DLEU1:PSME1:TCF12:GJC1:TUBD1:RPS6KB1:PHLPP1:CHST8:TMEM115:TAPBP:ESR1:HOXA10:CALD1:RDH10
TF_targets	FREAC3_01	257	20	0.0018543873 231155604	0.0058308055 00518	ZNF362:TXNDC12:DENND2C:HOXC12:ULK1:TNFRSF19:PRPF39:SH3GL3:PHLPP1:PPP1CB:BCL11A:RPN2:NIPBL:CDKN1A:HOXA10:HOXA11:HOXA13:CALD1:ZMAT4:PAPPA
TF_targets	ETF_Q6	120	12	0.0019238246 21280222	0.0060181180 460560785	CLSTN1:MTOR:ZBTB17:ZNF362:TRIM33:RUSC1:ASH1L:YY1AP1:DAP3:ZBTB41:ZNF496:CUL2
TF_targets	TGATTTRY_GFI1_01	295	22	0.0019410795 95116631	0.0060411150 66434412	PHC2:VAV3:CRB1:DMBT1:CDON:USP5:SOX5:LHFP:LCP1:KPNA3:SIX6:TCF12:MRAS:CDH6:NIPBL:PPAP2A:PCDHGC3:ESR1:ITGB8:JAZF1:CD36:SLC25A25
TF_targets	LBP1_Q6	222	18	0.0019555342 396253414	0.0060552075 44017555	TRIM62:TXNDC12:SSX2IP:CSDE1:TMOD4:SMG5:TMEM79:FCRLA:FCRLB:CRB1:RGR:GRK5:SOX5:PCDH9:FITM1:STXBP6:SCFD1:LRFN5
TF_targets	TEF_Q6	259	20	0.0020308938 23821491	0.0062253529 27292007	GPR153:ARNT:CRB1:ZFYVE27:C12orf57:HOXC11:LMO7:MIA2:CTAGE5:SPAG9:KCNJ16:ERF:PUM2:FOXP1:ZPLD1:FGF12:C5orf30:NDFIP1:HOXA11:TSGA13:RB1CC1
TF_targets	NFKB_Q6	259	20	0.0020308938 23821491	0.0062253529 27292007	SPEN:TAL1:FOXD2:SLC16A4:ASH1L:YY1AP1:DAP3:ARHGEF2:GRK5:EHF:SOX5:PTHLH:SLC11A2:NFKBIB:PAFAH1B3:XPO1:BFSP1:NIPBL:HOXA11:PAPPA
TF_targets	CREBP1_Q2	260	20	0.0021243416 202572224	0.0064470069 07248287	CSDE1:RUSC1:CREM:RCE1:ERC1:LGR5:GTF2A1:MESDC1:CCDC130:ERF:OSR1:BCL11A:SMDT1:MAP3K13:SENP2:ATP10D:NEUROD6:FAM167A:GEM:SLC25A25
TF_targets	ER_Q6_02	260	20	0.0021243416 202572224	0.0064470069 07248287	SMPD1:HPX:HOXC13:SLC25A3:LCP1:STAC2:SPAG9:UTP18:EPG5:RFX1:CTU1:ATP6V1B1:FGF12:RELL2:CDKN1A:DACT2:ITGB8:HOXA10:ZMAT4:CNTLN
TF_targets	AREB6_03	261	20	0.0022213935 560606787	0.0067081686 593911585	RIMKLA:SSX2IP:DDAH1:KCNJ11:SYVN1:FITM1:MESDC1:HNF1B:VAT1:NPEPPS:RPS6KB1:MEGF8:FAM49A:OSR1:DSTN:VGLL4:HDAC3:RELL2:HOXA11:CEL
TF_targets	ATF4_Q2	262	20	0.0023221580 877823707	0.0069779134 65749981	TMEM57:CDH23:ZFYVE27:PCDH9:GTF2A1:MESDC1:PSMD12:KCNJ2:ST8SIA5:CCDC130:XPO1:DCTN1:CPT1B:GSK3B:CNBP:AP2M1:MAP3K13:FAM167A:GEM:RBM18
TF_targets	HSF_Q6	208	17	0.0023650449 266779706	0.0070171239 72073877	ACOT7:SEMA6C:CKS1B:PRKAB1:PSEN1:GTF2A1:CORO1A:DNAJB1:ITPRIPL1:BPIFA2:ATP2C1:CKS1B:KCNIP1:TSPAN17:EDN1:YWHAG:KLF14:CREB3L2
TF_targets	RTTTNNNYTGGM_UNKNOWN	156	14	0.0023697172 75815113	0.0070171239 72073877	SLC11A2:ESRP2:SPAG9:PUM2:BCL11A:BPIFA2:FOXP1:ZNF654:ATP10D:PTP4A1:ESR1:HOXA11:CALD1:PALM2
TF_targets	PAX5_01	156	14	0.0023697172 75815113	0.0070171239 72073877	GOLPH3L:ZNF143:HOXC13:SH3GL3:GIPC1:C19orf68:KIAA1841:GSK3B:EIF4E:KCNIP1:HOXA10:NEUROD6:CALD1:CREB3L2
TF_targets	FOXO4_02	263	20	0.0024267457 786056397	0.0071168986 7764154	GPR153:TMEM57:ZNF362:TXNDC12:FAM63A:PRUNE:EMP1:SH3GL3:NFATC3:PITPNC1:PHLPP1:INSR:SCN3A:RBP2:FGF12:CNR1:ITGB8:ZMAT4:TMEM71:PAPPA
TF_targets	CDX2_Q5	263	20	0.0024267457 786056397	0.0071168986 7764154	GPR153:ZNF362:RPE65:TMOD4:PMF1:HECTD2:TTC12:HOXC12:CCER1:ADAM11:RPL38:FAM49A:FOXP1:PLSCR4:MARCH3:ARL4A:HOXA11:CNTLN:ADAMTSL1:PAPPA
TF_targets	LEF1_Q6	264	20	0.0025352693 008012504	0.0073995898 253050845	SYT11:HABP2:WEE1:LPCAT3:SOX5:TFCP2:HOXC11:TNFRSF19:FRMD6:TRPM1:MESDC1:TUBB4A:AFF3:CDX1:ADAM19:PTP4A1:HOXA10:CLDN4:ZMAT4:CNTLN
TF_targets	CAGNYGKNAAA_UNKNOWN	78	9	0.0026072119 311310576	0.0075733298 95190215	HECTD2:GRK5:TPI1:TNFRSF19:UBALD2:PPP1CB:AFF3:CACNA2D2:SOD2

TF_targets	NCX_01	175	15	0.002641100288198431	0.007618794794392287	TRIM62:NRAS:LCE1F:HOXC11:PRKAB1:PCDH9:CACNA2D2:CDH6:ISL1:ESR1:EVX1:NEUROD6:ZCWPW1:MEPCE:KLF4
TF_targets	DR4_Q2	265	20	0.0026478434367396145	0.007618794794392287	TMEM125:SEMA6C:SCAMP3:POU2AF1:ENO2:LPCAT3:SOX5:STAC2:SPATA32:NPEPPS:TCEB3B:CIC:PUM2:FOXP1:RAB6B:NIPBL:RELL2:EDN1:KLF14:RB1CC1
TF_targets	TST1_01	266	20	0.002764585081171535	0.007917356335749467	ZNF362:LMNA:TTC12:HOXC12:SLC25A3:SIAH3:FOXG1:G2E3:NIPA2:MESDC1:RAD18:CACNA2D2:ISL1:THBS2:ARL4A:JAZF1:NEUROD6:TSGA13:KLF14:TMEM71
TF_targets	IK3_01	230	18	0.002869878485385861	0.008149185536695996	ZNF362:TAL1:ADAM15:HCN3:EHF:C1S:DNAH9:NAPG:CHST8:BCAM:PPP1CB:XPO1:PKP4:USP25:MAP3K13:CXCL6:ARAP3:MPLKIP
TF_targets	HNF3ALPHA_Q6	212	17	0.0028824565918728067	0.008149185536695996	DNASE2B:NRAS:ARHGEF2:RBP3:GTF2A1:ESRP2:PAFAH1B3:PUM2:PPP1CB:AFF3:FOXP1:MAP3K13:GPRIN3:CCNO:CDKN1A:HOXA11:CALD1
TF_targets	E12_Q6	267	20	0.0028856132392235	0.008149185536695996	FBLIM1:NBL1:ELOVL1:NHLH1:ARL5B:ESRP2:HNF1B:NPEPPS:FSCN2:PKN1:MEGF8:FAM49A:OSR1:IGFBP5:HES6:PPARA:MAP7:AIG1:CALB1:COL22A1
TF_targets	GGAANCGGAANY_UNKNOWN	110	11	0.002918364023655285	0.008203696103362782	EBNA1BP2:WDR65:MED8:SZT2:TM2D1:SNRPE:PRKAB1:COMMD6:ZNF48:RPL38:THUMPD3
TF_targets	CTAWWWATA_RSRFC4_Q2	363	25	0.0029338442527167465	0.00820938070714319	RNF207:GPR153:TMEM57:AMPD1:CSDE1:SOX5:FITM1:MIA2:CTAGE5:CACNB1:ADAM11:KCNJ2:TTYH2:GRIN2D:CPT1B:LSMEM2:FOXP1:PLSCR4:FGF12:TRMT10A:CPEB4:ESR1:THBS2:HSPB1:CD36:ADAMTSL1
TF_targets	E2F1_Q3_01	249	19	0.002956968402804676	0.008236304683611199	WEE1:KCNA1:DLEU1:GTF2A1:GRIN2A:DCTPP1:RND2:KPNB1:UBALD2:CTDSP1:CGGBP1:ISL1:CPNE5:C6orf89:HOXA13:ZCWPW1:MEPCE:PUF60:KLF4
TF_targets	AML_Q6	268	20	0.003011049025376473	0.00834881775218022	RNF207:CTSK:FAM63A:PRUNE:SEMA4A:CREM:BTG4:SOX5:KIAA1199:HAPLN3:KPNB1:PITPNC1:UBALD2:C17orf70:GRIN2D:HPCAL1:IL18RAP:ENTPD3:TSPAN17:PAPPA
TF_targets	HFH1_01	250	19	0.003090361480033076	0.00852995702633564	ARHGEF2:KRTAP5-5:POU2AF1:KCTD12:ZFYVE1:GTF2A1:LINS:CD2BP2:RPS6KB1:FAM49A:PPP1CB:BCL11A:WBP2NL:CNBP:NIPBL:WDR46:PFDN6:HOXA10:HOXA11
TF_targets	YATGNWAAT_OCT_C	365	25	0.0031511283821880165	0.008658505915021125	GPR153:PHC2:PDZK1IP1:NRAS:SEMA6C:C12orf57:SOX5:HOXC11:SIAH3:NRL:FOXG1:LRFN5:SH3GL3:HNF1B:UTP18:TUBB4A:ALK:FOXP1:MRAS:FGF12:ISL1:FCHSD1:HOXA10:GPR22:LYN
TF_targets	MEIS1BHOXA9_01	144	13	0.0031658434047389986	0.008659930389644794	ZNF362:LPHN2:HIPK1:NRAS:TTC12:HOXC12:FRMD6:ACAN:TCF3:NIPBL:HOXA11:KLF14:KCNV2
TF_targets	NF1_Q6	270	20	0.0032756384619828617	0.008920265454506901	RPE65:HIPK1:SOAT1:SNX15:LPCAT3:NFATC3:CACNB1:ERF:ALK:BCL11A:BPIFA2:CYB5R3:RBP2:CFI:KLHL2:C5orf30:PCDHGC3:JAZF1:NUDCD1:ADAMTSL1
TF_targets	AP2GAMMA_01	252	19	0.003372233391361405	0.009142499416579809	ZNF362:PHC2:SEMA6C:FCRLA:CDON:SOX5:SMG1:NFATC3:SPAG9:PKN1:ERF:CIC:SCN5A:CPEB4:TSPAN17:C6orf89:ZFAND3:SRRM3:NYAP1
TF_targets	AREB6_01	271	20	0.003415044849068305	0.009217598928901177	TMEM125:EHF:PC:LRFN4:BTG4:C11orf88:CRTAM:KIAA1199:ACAN:SMG1:ESRP2:VAT1:OSR1:AFF3:SAMD7:DIAPH1:MAP7:ITGB8:ADAMTSL1:CEL
TF_targets	TGACCTTG_SF1_Q6	253	19	0.0035209976264195695	0.009399187176711324	HOXC13:LRFN5:HNF1B:STAC2:ST8SIA5:TUBB4A:INSR:ATP6V1B1:RBMS1:SYNGR1:SCN5A:TMEM161B:NDFIP1:NYAP1:PPP3CC:CA2:CNTLN:NTMT1:CEL
TF_targets	STAT1_03	253	19	0.0035209976264195695	0.009399187176711324	SCNM1:LYSMD1:GRK5:LRFN4:DDX25:DDX47:CSRNP2:FITM1:FOXG1:SMPD3:BRCA1:GSK3A:ERF:ZNFX1:ARMC8:TTC1:SYNE1:BET1:NUDCD1
TF_targets	SMTTTTGT_UNKNOWN	408	27	0.003545664011942238	0.009399187176711324	GPR153:TARDBP:ZNF143:EHF:SOX5:WBP4:LCP1:KCNK10:SMPD3:EPN2:SAFB:HPCAL1:BCL11A:ZNF638:DSTN:FOXP1:GSK3B:GPRIN3:NIPBL:ISL1:IPO11:CPEB4:PTP4A1:ITGB8:NEUROD6:YWHAG:CALD1
TF_targets	BACH2_01	272	20	0.0035593643242955997	0.009399187176711324	RIT1:LMNA:CDH23:REXO2:ENO2:LPCAT3:NUAK1:CALB2:TUBB4A:NOL10:CHMP4B:ABCF3:EIF4E:HDAC3:RELL2:SNCB:CDKN1A:ZCWPW1:MEPCE:UBQLN1
TF_targets	CACCCBINDINGFACTOR_Q6	272	20	0.0035593643242955997	0.009399187176711324	SZT2:TRIM33:CREM:GRK5:C1S:KCNK10:LINS:SPAG9:UTP18:BCL11A:XPO1:DCTN1:RTKN:IGFBP5:USP25:GNAI2:EIF4E:CDKN1A:SEPT7:ADAMTSL1

TF_targets	FXR_IR1_Q6	113	11	0.0035961146 4067375	0.0094553014 25909429	MACF1:OVGP1:TTC12:SOX5:DTD2:BRCA1:ERF:WBP2NL:IFRD2:GPRIN3:TRMT10A
TF_targets	HEN1_01	200	16	0.0038560849 800682692	0.0100521873 41203608	TMEM125:PSME1:TCF12:SLC5A10:MFAP4:PTGER1:GIPC1:GRIN2D:RTKN:CHMP4B:SPATC1L:HYAL2:FOXP1:MGAT1:ARL4A:HOXA11
TF_targets	EGR2_01	200	16	0.0038560849 800682692	0.0100521873 41203608	SYVN1:UBASH3B:SH3GL3:PKD1:NFATC3:ESRP2:ERF:OSR1:SCN5A:GNAI2:CGGBP1:GPRIN3:DIAPH1:PDGFRB:ITGB8:KLF4
TF_targets	MEF2_03	237	18	0.0039390535 817647124	0.0102247773 82453084	TMEM57:AMPD1:SOX5:KPNA3:FITM1:MIA2:CTAGE5:ADAM11:CREB1:CPT1B:FOXP1:FGF12:TRMT10A:CPEB4:ESR1:THBS2:HOXA11:LYN:TMEM71
TF_targets	P300_01	256	19	0.0040003656 05748551	0.0103079843 93077972	TMEM57:PTBP2:CKS1B:ADAM15:HCN3:FDPS:GRK5:EMP1:CSRNP2:ORAI1:MOAP1:C15orf26:SMPD3:PUM2:PPP1CB:BCL11A:FOXP1:FGF12:CKS1B:TOR4A
TF_targets	CGTSACG_PAX3_B	148	13	0.0040049054 11736852	0.0103079843 93077972	SOAT1:IPO7:SLC25A3:DCAF11:PDXDC1:PHLPP1:PEX11G:CCDC130:BCL11A:EIF4E:CDKN1A:GEM:RBM18
TF_targets	HEN1_02	201	16	0.0040472351 56514445	0.0103731657 37284923	TXNDC12:ADAM15:SMG5:TMEM79:CRB1:USP5:PSME1:MFAP4:GIPC1:GRIN2D:CHMP4B:SPATC1L:HYAL2:MEGF10:MGAT1:ARL4A
TF_targets	ELK1_02	257	19	0.0041716831 99639593	0.0106030281 32417298	C1orf210:MED8:SZT2:C1orf52:EXTL2:NRAS:CKS1B:ARL5B:SYVN1:RPL19:PSMD12:FBXO36:CGGBP1:NIPBL:RPL37:CKS1B:CPEB4:VPS52:RPS18:UBAC1
TF_targets	CEBPA_01	257	19	0.0041716831 99639593	0.0106030281 32417298	CSDE1:ARNT:F5:SLC16A12:EHF:USP5:RASSF8:SIAH3:TNFSF13B:KCNJ2:PUM2:PPP1CB:PROS1:SAMD7:MTTP:MCTP1:ESR1:CLDN4:RB1CC1
TF_targets	FOXD3_01	202	16	0.0042460795 88125626	0.0107473383 76583536	NRAS:RBP3:SIAH1:ANKRD11:IKZF3:RPS6KB1:FAM49A:PPP1CB:AFF3:FLOT1:IER3:HOXA10:HOXA11:EVX1:YWHAG:CALD1
TF_targets	MYOGENIN_Q6	258	19	0.0043490223 14124291	0.0109624116 1824718	FBLIM1:NBL1:TRIM62:TMEM125:DDAH1:GJA5:CDON:FITM1:EPN2:CACNB1:ADAM11:FSCN2:TUBB4A:PKN1:ERF:CIC:FAM49A:OSR1:RTKN
TF_targets	CEBP_Q3	259	19	0.0045325408 20056844	0.0112936365 37971018	ARNT:NSUN6:ARL5B-AS1:RASSF8:KPNA3:MIA2:CTAGE5:TRPM1:TCF12:ANP32A:SPAG9:PUM2:BCL11A:ZNF654:CD200R1:TRMT10A:PDGFRB:CALD1:TMEM71:CNTLN:PALM2
TF_targets	STAT5A_01	259	19	0.0045325408 20056844	0.0112936365 37971018	GPR153:TAL1:CREM:SMPD1:APBB1:FRMD6:GSK3A:USP25:PROS1:STX19:EIF4E:MTTP:PRELID2:SNCB:BET1:PRKAR2B:CEP41:NUDCD1:KLF4
TF_targets	USF_02	278	20	0.0045359687 73447376	0.0112936365 37971018	CSDE1:DUSP8:UBE2L6:REXO2:HOXC11:LHFP:KLHL28:FAM179B:GTF2A1:ARPP19:TCF12:ESRP2:SHPK:SHPK:XPO1:COMMD8:EIF4E:CPEB4:C4A:HOXA11:NYAP1
TF_targets	ER_Q6_01	279	20	0.0047184791 34698709	0.0116626044 4307083	LRFN4:LCP1:ESRP2:TNRC6C:C19orf57:CIC:CXCL17:GREB1:PKP4:RBMS1:PPARA:EIF4E:NIPBL:ISL1:ITGB8:HOXA10:NYAP1:CEP41:CALD1:TMEM71
TF_targets	CREB_02	260	19	0.0047223988 48259828	0.0116626044 4307083	CDC42:ZFYVE27:RCE1:ERC1:LGR5:GTF2A1:MESDC1:RNF166:SPAG9:ERF:KDELR1:NOL10:DCTN1:MAP3K13:TRMT10A:EVX1:NEUROD6:GEM:RBM18
TF_targets	SP1_Q6	261	19	0.0049187587 67922417	0.0120499712 78846083	CLSTN1:HIPK1:CTR9:MRPL49:ERC1:PPP1R36:ANP32A:GRIN2A:ZNF48:SPAG9:UTP18:S1PR5:CCDC74A:CREB1:FBXO36:HYAL2:CGGBP1:FGF12:RPL7
TF_targets	AMEF2_Q6	261	19	0.0049187587 67922417	0.0120499712 78846083	TMEM57:ZNF362:ARHGEF2:SOX5:KPNA3:GTF2A1:TECR:DCTN1:AFF3:GNAI2:LSMEM2:SLC4A4:EDN1:HOXA10:JAZF1:ZCWPW1:MEPCE:LYN:CNTLN
TF_targets	E4BP4_01	224	17	0.0050260201 00466724	0.0122634890 45138807	CLSTN1:CDC42:TMEM57:ARNT:SYT11:WEE1:PTHLH:PCK2:EPN2:ERF:MIOX:VGLL4:NIPBL:FUT9:HOXA10:NEUROD6:YWHAG
TF_targets	PXR_Q2	262	19	0.0051217851 58319144	0.0124473663 21014652	MACF1:TMEM125:TAL1:CSDE1:CKS1B:ADAM15:SEMA4A:IPO7:AKAP3:HOXC13:PSME1:STAC2:SYNGR1:IFRD2:GSK3B:TRMT10A:CKS1B:ARL4A:HOXA10:COPG2
TF_targets	TATA_C	282	20	0.0053027406 01391218	0.0128359990 74796203	GJA5:ARHGEF2:GRK5:EHF:SOX5:FOXG1:PLA2G4B:CTDSP1:FBXO36:UCKL1:CYB561D2:XXcos-LUCA11.4:FOXP1:RBP2:PPAP2A:SLC12A2:PDGFRB:CDX1:HOXA10:PRKAR2B:CA2

TF_targets	CDC5_01	264	19	0.0055485065 54010216	0.0133636602 45844095	HIPK1:NRAS:GOLPH3L:LMNA:HOXC12:LRFN5:RBMS1:FOXP1:FGF12:MTTP:CDH6:PCDHGC3:CPEB4:HOXA11:MPLKIP:YWHAG:CALD1:LYN:KLF4
TF_targets	YNTTTNNNANGCARM_UNKNOWN	72	8	0.0055793810 43071507	0.0133636602 45844095	TLL2:GRK5:C1S:KCTD12:FOXG1:FGF12:HCRTR2:EVX1
TF_targets	TTCYNRGAA_STAT5B_01	342	23	0.0055864481 35557778	0.0133636602 45844095	GPR153:EXTL2:CREM:SMPD1:STXBP6:MIA2:CTAGE5:FRMD6:SMPD3:ADAM11:TRIM25:GSK3A:USP25:PROS1:AP2M1:EIF4E:MTTP:IPO11:HOXA11:BET1:PRKAR2B:CEP41:NUDCD1:KLF4
TF_targets	TCANNTGAY_SREBP1_01	484	30	0.0056146153 20451614	0.0133785755 68263612	CTSS:ANXA9:C1orf85:PSAP:DUSP8:LINC00610:COMMD9:UCP3:SOX5:LCP1:ZFYVE1:SHPK:SHPK:RABEP1:ADAM11:NPEPPS:PKN1:GIPC1:FSHR:FBXO36:SYNGR1:WBP2NL:VGLL4:FOXP1:CGGBP1:GSK3B:KCNAB1:NEUROD6:NYAP1:GEM:UBQLN1:NTMT1
TF_targets	TAAWWATAG_RSRFC4_Q2	172	14	0.0056873250 32656954	0.0134990983 26539853	RNF207:SOX5:WBP4:PCDH9:FITM1:MIA2:CTAGE5:CPT1B:LSMEM2:FOXP1:DZIP1L:CDKN1A:ESR1:KLF14:TMEM71
TF_targets	E2F_Q3	227	17	0.0057279436 0031671	0.0135428123 88345708	RHCE:ZNF362:SYT11:IPO7:WEE1:KCNA1:SLC25A3:SMG1:DCTPP1:KPNB1:C19orf68:CTDSP1:NCL:PCNA:NIPBL:CPNE5:KLF4
TF_targets	FOXO4_01	246	18	0.0057770651 94702582	0.0136062153 23430792	MED8:SZT2:TXNDC12:LPHN2:CRB1:DCAF11:FOXG1:SH3GL3:NFATC3:PITPNC1:WBP2NL:CACNA2D2:ZNF654:SLC4A4:ISL1:HOXA10:HOXA11:NYAP1
TF_targets	GATA1_05	285	20	0.0059452603 61514648	0.0139484954 63553598	SEC16B:MOGAT2:ENO2:SOX5:TFCP2:PCDH9:RABEP1:SLC14A1:PUM2:GSK3B:ARMC8:ABCF3:CXCL6:CPEB4:HOXA11:HOXA13:KEL:CNTLN:PAPPA:SLC25A25
TF_targets	GABP_B	266	19	0.0060039227 74379993	0.0139594236 42883006	EBNA1BP2:WDR65:EXTL2:NRAS:SCAMP3:ZBTB41:EMG1:PHB2:PRKAB1:CD2BP2:FBXO36:PER2:RPL32:NIPBL:DIAPH1:THG1L:CPEB4:TSTD2:ZNF79
TF_targets	CEBPDELTA_Q6	247	18	0.0060185711 77177428	0.0139594236 42883006	CDC42:DNASE2B:SYT11:TTC12:HOXC13:PDE1B:SIAH3:DTD2:TBC1D10B:ANKRD11:UBALD2:S1PR5:C2CD2:FOXP1:STX19:LRRC16A:HOXA11:GPR22
TF_targets	CACBINDINGPROTEIN_Q6	247	18	0.0060185711 77177428	0.0139594236 42883006	GRK5:SYVN1:CLMP:LRFN5:ZFYVE1:ACAN:KPNB1:RALBP1:RFX1:KCNJ14:PPP1CB:BCL11A:DCTN1:IGFBP5:FBXO36:GNAI2:RAB6B:HOXA10
TF_targets	GATA1_03	248	18	0.0062682353 68632524	0.0144834226 32067575	SSX2IP:NRAS:SFRP5:HABP2:EHF:ENO2:SOX5:HOXC11:PCDH9:GREB1:FOXP1:AP2M1:GPRIN3:TMEM161B:KLF14:ZMAT4:ADAMTSL1:PAPPA
TF_targets	ARGGGTTAA_UNKNOWN	122	11	0.0064095504 5581714	0.0147540595 39805494	TMEM125:NUMB:TGFB1I1:PEX13:RTKN:CREB1:DSTN:HYAL3:NAT6:ISL1:ESR1
TF_targets	TTAYRTAA_E4BP4_01	268	19	0.0064894269 40041418	0.0148260315 85862416	CLSTN1:ANGPTL7:TMEM57:ARNT:HECTD2:WEE1:PTHLH:C15orf26:EPN2:ERF:BCL11A:ZNF638:PER2:MIOX:NIPBL:MCTP1:FUT9:NEUROD6:YWHAG
TF_targets	TCF1P_Q6	268	19	0.0064894269 40041418	0.0148260315 85862416	TAL1:TRIM33:GOLPH3L:PRUNE:DUSP8:PCDH9:FOXG1:ZPBP2:SPAG9:BCAM:ZNF654:PPAP2A:MCTP1:CPEB4:EDN1:CDKN1A:HOXA11:TSC22D4:CNTLN
TF_targets	RGTTAMWNATT_HNF1_01	74	8	0.0065805384 83493933	0.0149780913 24370519	F13B:HABP2:HNF1B:RBMS1:DZIP1L:CFI:SLC12A2:HOXA10
TF_targets	E2F1DP1RB_01	232	17	0.0070734328 04038879	0.0160401264 32950618	RHCE:ZNF362:HCN3:SOAT1:IPO7:WEE1:C14orf142:UBR7:DCTPP1:KPNB1:C19orf57:C19orf68:CTDSP1:NCL:PCNA:NIPBL:KLF4
TF_targets	MMEF2_Q6	271	19	0.0072772245 17278719	0.0164411368 7237044	GPR153:TMEM57:LPHN2:NHLH1:SOX5:KPNA3:MYLPF:ZPBP2:ADAM11:DCTN1:NIPBL:MCTP1:ESR1:HOXA10:HOXA11:HSPB1:ZCWPW1:MEPCE:LYN
TF_targets	MYCMAX_B	272	19	0.0075564314 08770028	0.0170089415 47415928	ZNF362:RIMKLA:IPO7:WEE1:CCER1:LRFN5:GTF2A1:ZNF771:KPNB1:SPAG9:RND3:NCL:CYP2D6:DAZL:CNBP:ISL1:DIAPH1:CPNE5:TSC22D4
TF_targets	KCCGNSWTTT_UNKNOWN	108	10	0.0076044918 55699829	0.0170541912 94032704	PTBP2:YY1AP1:DAP3:SYT11:RFX1:BCL11A:XPO1:NIPBL:RGL2:GNG11
TF_targets	LFA1_Q6	254	18	0.0079488058 768915	0.0177366424 24941627	TAL1:C1orf52:SYVN1:SOX5:DTD2:RASL12:GRIN2A:PDZD9:CACNB1:CIC:OSR1:DCTN1:IFRD2:GSK3B:KCNAB1:NIPBL:ARAP3:CREB3L2
TF_targets	MZF1_02	235	17	0.0079960273 22719586	0.0177366424 24941627	CSDE1:GRK5:EHF:HOXC11:WBP4:FRMD6:HNF1B:VAT1:UTP18:GRIN2D:XPO1:FOXP1:CGGBP1:CDX1:SOX30:HOXA10:ADAMTSL1

TF_targets	PU1_Q6	235	17	0.0079960273 22719586	0.0177366424 24941627	ADAM15:FCRLA:FAU:MRPL49:NCKAP1L:PCDH9:SEPT1:SEPT1:ESRP2:ADAM11:PKN1:ERF:ITPRIPL1:ZNFX1:KCNAB1:FGF12:LECT2:LYN
TF_targets	HSF1_01	274	19	0.0081408738 03943658	0.0179331812 4451101	ACOT7:FOXD2:LRRC8B:HIPK1:CREM:HSPA8:PTHLH:TNFRSF19:NFATC3:DNAJB1:CHST8:GREB1:IGFBP5:DAZL:CACNA2D2:RAB6B:STIM2:HSPB1:YWHAG
TF_targets	GCCATNTTG_YY1_Q6	435	27	0.0081434282 04474673	0.0179331812 4451101	TARDBP:UBIAD1:WDR77:YY1AP1:DAP3:SYT11:PMF1:APBB1:ZNF143:HSPA8:EMG1:PHB2:KLHL28:FAM179B:CPSF2:METTL16:PRPSAP2:ADAM11:SAFB:RFX1:ZNF638:RBMS1:LMF2:NCAPH2:GNAI2:FOXP1:ARMC8
TF_targets	YNGTTNNNATT_UNKNOWN	375	24	0.0085184456 16873607	0.0186915533 3198885	TAL1:HIPK1:TMOD4:WEE1:EMP1:SOX5:HOXC12:MIA2:CTAGE5:NUMB:TCF12:KCNJ2:TCEB3B:FAM49A:ZNF638:DCTN1:ITPRIPL1:PKP4:VGLL4:NIPBL:CPEB4:NEUROD6:CEP41:EXOC4:CALD1
TF_targets	NERF_Q2	256	18	0.0085835053 80440372	0.0187668038 78382175	FBLIM1:MPL:EXTL2:TNFAIP8L2:PEX16:SLC43A1:USP5:PTPN6:TCF12:CORO1A:NFATC3:C17orf70:CD79A:FBXO36:EFHB:HYAL2:ARAP3:LYN
TF_targets	CREL_01	257	18	0.0089157765 06642941	0.0193545326 30078983	ARHGEF2:GRK5:EHF:SOX5:ORAI1:ACAN:SMPD3:IKZF3:RBMS1:CREB1:CTDSP1:CYP2D6:STX19:FGF12:SLC12A2:FLOT1:IER3:ZFAND3
TF_targets	MYOD_Q6_01	257	18	0.0089157765 06642941	0.0193545326 30078983	NHLH1:ARL5B:APBB1:CTR9:HOXC12:ESRP2:SPAG9:KCNJ2:FSCN2:PKN1:PPARA:HYAL2:GSK3B:ARL4A:JAZF1:SRRM3:TSC22D4:CALB1
TF_targets	CDP_01	94	9	0.0089502953 60461876	0.0193605679 78304058	PHC2:SOAT1:SIAH3:C13orf35:ADAM11:FGF12:NEUROD6:KLF14:CNTLN
TF_targets	CEBPGAMMA_Q6	258	18	0.0092582830 71265262	0.0198857488 50252852	NRAS:SCAMP3:SOAT1:OPALIN:HABP2:KCNA1:USP5:NCKAP1L:WBP4:MOAP1:SPAG9:TCEB3B:FAM208A:NIPBL:YWHAG:GPR22:CNTLN:RBM18
TF_targets	STAT_01	258	18	0.0092582830 71265262	0.0198857488 50252852	GPR153:TAL1:SCAMP3:CREM:STXBP6:TRIM25:GSK3A:CXCL17:IL18RAP:USP25:AP2M1:EIF4E:IPO11:SNCB:BET1:CEP41:RDH10:NUDCD1
TF_targets	GR_01	201	15	0.0093629386 41691762	0.0200399739 34849034	TIE1:KCNK10:TCF12:EPG5:MBP:TUBB4A:PUM2:DSTN:ZNF654:NIPBL:ISL1:CPEB4:CDKN1A:CD36:ADAMTSL1
TF_targets	TGAYRTCA_ATF3_Q6	546	32	0.0095584670 7241921	0.0203869402 59355658	HOOK1:DNAJB4:HIPK1:RUSC1:CREM:ZFYVE27:RCE1:AKAP3:PTHLH:LGR5:ORAI1:TNFRSF19:DTD2:RND2:ADAM11:SPAG9:PITPNC1:ACTL9:OSR1:DCTN1:PKP4:SYNGR1:MAP3K13:SENP2:FGF12:AREG:AREGB:NEUROD6:GPR22:GEM:UBQLN1:RBM18:SLC25A25
TF_targets	CREBP1_01	183	14	0.0096196355 6049335	0.0204459153 02790742	CLSTN1:CDC42:TMEM57:SYT11:WEE1:PTHLH:EPN2:ERF:BCL11A:ZNF638:PER2:MCTP1:NEUROD6:YWHAG
TF_targets	YCATTAA_UNKNOWN	568	33	0.0097112334 27862872	0.0205689319 13181775	ZBTB17:HOOK1:NRAS:LMNA:SEMA4A:EHF:OPCML:SOX5:HOXC13:CCER1:PRKAB1:ARPP19:SLC5A10:STAC2:VAT1:TUBB4A:PTGER1:CHST8:OSR1:RBMS1:GSK3B:TRMT10A:MTTP:CDH6:CDX1:LSM11:CPNE5:HOXA10:JAZF1:NEUROD6:KLF14:CNTLN:SLC34A3
TF_targets	SOX9_B1	240	17	0.0097462243 20410975	0.0205716153 47580258	TAL1:DENND2C:SEMA6C:GRK5:WEE1:CDON:SOX5:G2E3:ZFYVE1:N4BP1:MBP:FAM49A:USP25:HYAL2:HOXA10:NEUROD6:CD36
TF_targets	SRY_02	260	18	0.0099748761 15560822	0.0209816359 67214144	TMEM50A:ZNF362:MED8:SZT2:LPHN2:SEMA6C:OPCML:SOX5:CSRNP2:ULK1:TNFRSF19:ZFYVE1:CXCL17:C21orf91:CDH9:NIPBL:YWHAG:CD36
TF_targets	NMYC_01	280	19	0.0101162018 83501017	0.0211626663 75157235	RPL22:NRAS:PSEN2:ALDH18A1:WEE1:SLC43A1:HOXC13:MIA2:CTAGE5:KLHL28:FAM179B:GRIN2A:DCTPP1:UTP18:OSR1:CMTM8:HYAL2:TUBB:MAP7:NEUROD6
TF_targets	E2F_Q6_01	241	17	0.0101303255 4351789	0.0211626663 75157235	ZNF362:IPO7:WEE1:DLEU1:SMG1:DCTPP1:RND2:KPNB1:C19orf68:NCL:PCNA:CNBP:MAP3K13:NIPBL:SNCB:CDKN1A:PUF60
TF_targets	TMTCGCGANR_UNKNOWN	166	13	0.0102518211 63299773	0.0213433819 44071198	ICMT:WDR77:CSDE1:SMG5:TMEM79:COQ9:DBR1:SLC12A2:SEPT7:ASH2L:PUF60:RPL12:LRSAM1
TF_targets	AAAYWAACM_HFH4_01	261	18	0.0103494033 2669471	0.0214732518 0028494	FAM63A:ARHGEF2:RBP3:HOXC12:ANKRD11:IKZF3:ADAM11:RPS6KB1:FAM49A:PPP1CB:CNBP:NIPBL:RGL2:HOXA10:HOXA11:INMT:CD36:CALD1
TF_targets	TAANNYSGCG_UNKNOWN	80	8	0.0104055637 42270143	0.0215165894 3316877	CLSTN1:IKZF3:BRCA1:RND3:ISL1:HOXA10:SEPT7:PBX3

TF_targets	OCT1_04	242	17	0.0105263567 73826715	0.0216928298 37953703	ZNF362:LAMTOR5:LMNA:EHF:OPCML:C12orf57:SOX5:FOXG1:HNF1B:PKP4:UCKL1:FOXP1:ACKR4:ISL1:CPEB4:HOXA10:JAZF1
TF_targets	ARP1_01	167	13	0.0107464933 6092507	0.0220719223 91125567	MED8:SZT2:TXNDC12:PMF1:EMP1:KLHL28:FAM179B:PITPNC1:ATP2C1:CFI:FCHSD1:CDX1:STAR
TF_targets	ZF5_B	243	17	0.0109345681 42481614	0.0223319405 94628525	RNF207:SPEN:ZBTB17:LPHN2:TRIM33:GON4L:CDON:METTL16:UTP18:TMEM145:MTHFD2:LMF2:NCAPH2:FGF12:PCDHGC3:DIAPH1:NYAP1
TF_targets	WYAAANNRNNNGCG_UNKNOWN	65	7	0.0109463118 65235949	0.0223319405 94628525	PHC2:LGR5:ULK1:MESDC1:RFX1:ERF:HYAL2
TF_targets	CREBP1CJUN_01	263	18	0.0111320319 62681765	0.0226351316 5745292	RUSC1:SYT11:CREM:ZFYVE27:RCE1:LGR5:SPAG9:OSR1:DCTN1:PKP4:MAP3K13:SENP2:AREG:AREGB:HCRTR2:NEUROD6:GEM:RBM18:SLC25A25
TF_targets	NFAT_Q6	244	17	0.0113552112 3672035	0.0230122221 07639247	ZNF362:ARL5B:ZNF143:EMP1:SOX5:RASL12:NAPG:SAFB:PTGER1:PER2:WBP2NL:FOXP1:ZNF654:GSK3B:CPEB4:EDN1:ZFAND3
TF_targets	WGGAATGY_TEF1_Q6	385	24	0.0115072147 36814502	0.0232430496 33963068	RNF207:TMEM125:HOOK1:PRUNE:CKS1B:LMNA:PAQR6:NID1:HOXC13:ESRP2:ANKRD11:GLP2R:BCAM:BCL11A:PKP4:DVL3:AP2M1:CKS1B:C5orf20:DIAPH1:TSPAN17:EDN1:ESR1:HOXA10:ZNF467
TF_targets	RFX1_02	284	19	0.0116337873 57702144	0.0234211560 6666108	EBNA1BP2:WDR65:SCNM1:LYSMD1:GYLTL1B:MRPL49:ATN1:ZFYVE1:KCNJ16:TUBB4A:GRIN2D:GRWD1:HPCAL1:FAM49A:CCDC74A:DAW1:PER2:CEP41:LRRC6
TF_targets	SMAD4_Q6	245	17	0.0117885389 956088	0.0235770779 912176	TXNDC12:PTBP2:APBB1:SYVN1:POU2AF1:SIAH3:DCTN1:CREB1:HYAL2:PLCXD2:AP2M1:HOXA10:EVX1:CALD1:NUDCD1:ADAMTSL1:PBX3
TF_targets	USF_Q6_01	245	17	0.0117885389 956088	0.0235770779 912176	NRAS:IPO7:WEE1:SYT13:SLC43A1:HOXC12:HOXC11:DCAF11:KLHL28:FAM179B:GTF2A1:RPL19:PKN1:EIF4E:DIAPH1:RGL2:KLF4
TF_targets	HAND1E47_01	285	19	0.0120401107 14185493	0.0240015278 94291344	VWA5B1:ASH1L:DMBT1:SOX5:PDZD9:RNF112:ADAM11:GRIN2D:BCL11A:GSK3B:ACAD11:UBA5:DVL3:EDN1:PI16:NEUROD6:CALD1:CREB3L2:CNTLN
TF_targets	CREB_01	267	18	0.0128379005 9524431	0.0254257122 17853988	CDC42:RUSC1:SYT11:CREM:ZFYVE27:RCE1:LGR5:SPAG9:OSR1:DCTN1:PKP4:MAP3K13:SENP2:AREG:AREGB:NEUROD6:GEM:RBM18:SLC25A25
TF_targets	GATA_C	267	18	0.0128379005 9524431	0.0254257122 17853988	RHCE:C1orf180:PKLR:MOGAT2:SOX5:TNFRSF19:C15orf26:IL1RL1:IGFBP5:HYAL3:NAT6:CDH9:ISL1:C5orf30:EDN1:KLF14:CNTLN:SLC34A3
TF_targets	TGGNNNNNNKCCAR_UNKNOWN	431	26	0.0130128444 33318169	0.0256887867 4538538	MPL:RPE65:TMOD4:ARHGEF2:IPO7:PTHLH:STXBP6:ADAMTS7:MYLPF:ESRP2:CACNB1:FSCN2:MYCN:FAM49A:IGFBP5:PPARA:LSMEM2:ARL13B:PLCXD2:CFI:PCDHGC3:PDGFRB:HOXA10:TSC22D4:KLF14:NUDCD1
TF_targets	GGGNNTTTCC_NFKB_Q6_01	135	11	0.0132176099 29318832	0.0260088453 4478867	SOX5:ORAI1:N4BP1:SMPD3:PKN1:RBMS1:CREB1:SLC12A2:CPEB4:ZFAND3:GEM
TF_targets	RREB1_01	210	15	0.0135905057 38632078	0.0266566189 7287964	TRIM62:CTSK:KCNA1:HOXC11:NUAK1:KPNB1:RALBP1:GRIN2D:PPP1CB:DCTN1:RBMS1:IGFBP5:FBXO36:ITGB8:NYAP1
TF_targets	YY1_Q6	249	17	0.0136537970 47622762	0.0266949237 1490348	TARDBP:UBIAD1:TXNDC12:WDR77:ARNT:CLK2:ASH1L:YY1AP1:DAP3:SYT11:PMF1:APBB1:BCL11A:ARMC8:CPEB4:SEPT7:PBX3
TF_targets	FREAC4_01	154	12	0.0138052775 00299728	0.0269048539 14322156	TCEANC2:GOLPH3L:EMP1:ULK1:PHLPP1:PUM2:PER2:CDKN1A:HOXA11:EVX1:CALD1:SLC34A3
TF_targets	GGAMTNNNNNTCCY_UNKNOWN	119	10	0.0144948254 54820105	0.0281587373 4853587	SLC16A4:FAM63A:PRUNE:TRUB1:GRK5:IPO7:NTAN1:GSK3A:TTLL4:TMEM115
TF_targets	TAAYNRNNTCC_UNKNOWN	174	13	0.0147474195 84666474	0.0285584950 68719204	HECTD2:ZNF143:HOXC11:CCER1:TNFRSF19:SIAH3:KCNJ16:TCEB3B:GRIN2D:WBP2NL:PPARA:ISL1:TAS2R40
TF_targets	YTAATTAA_LHX3_01	193	14	0.0148089580 3571738	0.0285869126 63884814	TAL1:CLK2:HOXC12:PRKAB1:NRL:LRFN5:ADAM11:PITPNC1:CHST8:RTCB:FLOT1:IER3:CD36:KLF14
TF_targets	VDR_Q3	232	16	0.0150244709 74498343	0.0289114425 6922394	ZNF362:TXNDC12:ARHGEF2:NHLH1:GRK5:CCER1:ORAI1:GTF2A1:BCAM:XPO1:FBXO36:NDUFA6:GSK3B:RAB6B:EIF4E:YWHAG

TF_targets	GATA4_Q3	252	17	0.01519848688547722	0.02915433018912297	ARHGEF2:FCRLA:SOX5:HOXC11:TNFRSF19:PSEN1:GTF2A1:ERF:ALK:SCN3A:ZNF654:CXCL6:CPEB4:CNR1:EVX1:YWHAG:TMEM71
TF_targets	CCANNAGRKGGC_UNKNOWN	120	10	0.015297037358780897	0.02925138805284121	DENND2C:GRK5:OPCML:LETMD1:RPL38:PUM2:ALK:SCN3A:STK32B:HOXA10
TF_targets	RNCTGNYNRNCTGNY_UNKNOWN	86	8	0.015671237250851493	0.029793133125048443	ANXA9:EHF:MOAP1:PLCXD2:C4A:HOXA10:CALD1:ADAMTSL1
TF_targets	POU3F2_01	103	9	0.015705114576053524	0.029793133125048443	NRAS:RIT1:PTHLH:SIAH3:KCNK10:STAC2:SYNGR1:TAPBP:HOXA11
TF_targets	HMGIY_Q6	253	17	0.015742525549892673	0.029793133125048443	PTBP2:HCN3:F13B:HECTD2:SOX5:PTHLH:ANKRD11:IKZF3:ZPBP2:BCL11A:IL18RAP:VGLL4:FOXP1:ZNF654:C3orf17:KCNAB1:TERT
TF_targets	CREB_Q4	273	18	0.015775708195722372	0.029793133125048443	CDC42:RUSC1:LGR5:GTF2A1:MESDC1:KCNJ2:SSTR2:CCDC130:ERF:OSR1:PKP4:IFT57:MAP3K13:SENP2:EVX1:NEUROD6:FAM167A:GEM
TF_targets	RP58_01	214	15	0.015888763624301118	0.029914030280320008	PMF1:HABP2:HOXC12:NUAK1:MOAP1:TCF12:ANP32A:GRIN2D:FAM49A:RBMS1:TGFBR2:FOXP1:CDH6:CREB3L2:PALM2
TF_targets	TGACGTCA_ATF3_Q6	234	16	0.016169373053938584	0.030348669424315494	RUSC1:CREM:ZFYVE27:RCE1:LGR5:SPAG9:OSR1:DCTN1:PKP4:MAP3K13:SENP2:AREG:AREGB:NEUROD6:GEM:RBM18:SLC25A25
TF_targets	CCAWNWWNNNGGC_UNKNOWN	87	8	0.016707372798321435	0.031100558773450963	CSDE1:YY1AP1:DAP3:EIF1B:ENTPD3:HOXA10:ZCWPW1:MEPCE
TF_targets	GTGGGTGK_UNKNOWN	295	19	0.016752456598897302	0.031100558773450963	LCE1F:SCAMP3:EHF:UBASH3B:PTHLH:C15orf26:KPNB1:UTP18:RNFT1:MYCN:BCL11A:ITPRIPL1:PER2:GNAI2:CGGBP1:SLC12A2:ZCWPW1:MEPCE:CHMP5
TF_targets	ELK1_01	275	18	0.01686346168992298	0.031100558773450963	PHC2:EBNA1BP2:WDR65:TIE1:CKS1B:EMG1:PHB2:NCKAP1L:PRKAB1:LCP1:UTP18:ZNFX1:TGFBR2:STX19:DBR1:CKS1B:TMEM161B:CPEB4:EVX1
TF_targets	RRAGTTGT_UNKNOWN	255	17	0.016875876973790605	0.031100558773450963	NRAS:ARHGEF11:TLL2:IPO7:ERC1:KCNA1:SIAH3:MOAP1:SPAG9:GRWD1:TMEM115:NIPBL:CNR1:HOXA11:RPL7:RDH10:KLF4
TF_targets	STAT5B_01	255	17	0.016875876973790605	0.031100558773450963	TAL1:CREM:SMPD1:APBB1:FRMD6:ADAM11:TRIM25:GSK3A:USP25:PROS1:AP2M1:MTTP:SNCB:BET1:PRKAR2B:CEP41:KLF4
TF_targets	E47_01	255	17	0.016875876973790605	0.031100558773450963	NBL1:MACF1:CORO1A:C19orf57:ERF:HES6:CHMP4B:SCN5A:CACNA2D2:FOXP1:ISL1:HDAC3:RELL2:MGAT1:AIG1:ARL4A:CALB1
TF_targets	AR_01	159	12	0.017367017472747694	0.031909279091494255	SLC43A1:HOXC12:FAM49A:RTKN:PER2:DSTN:ZNF654:STX19:TMEM161B:CDKN1A:MPLKIP:CD36
TF_targets	COREBINDINGFACTOR_Q6	276	18	0.017428729802499376	0.03192650204061447	MPL:NRAS:ARHGEF2:BTG4:C11orf88:NDUFA9:EMP1:SOX5:MIA2:CTAGE5:LINS:PITPNC1:UBALD2:TUBB4A:HPCAL1:ENTPD3:GSK3B:KCNV2:PALM2
TF_targets	TAXCREB_02	27	4	0.01754774965744899	0.0320482853025266	ZNF362:NOL10:BCL11A:DIAPH1
TF_targets	GATA2_01	105	9	0.0176108221061804	0.032067467118716546	DPH5:GRIN2A:KPNB1:MFF:SHQ1:HDAC3:RELL2:HOXA10:NELFB
TF_targets	CDPCR3HD_01	237	16	0.018011630394101016	0.032517734772427434	NUCKS1:CREM:HABP2:EHF:KCNA1:EMP1:SOX5:SIAH3:DTD2:MOAP1:PLA2G15:KCNS1:IFRD2:HOXA10:NEUROD6:YWHAG
TF_targets	NFKB_Q6_01	237	16	0.018011630394101016	0.032517734772427434	ASH1L:YY1AP1:DAP3:ARHGEF2:ARL5B:GRK5:EHF:SOX5:PTHLH:ORAI1:SMPD3:NFKBIB:GRIN2D:RBMS1:CREB1:PAPPA
TF_targets	PAX4_03	257	17	0.01807133129156213	0.032517734772427434	ANGPTL7:MPL:NHLH1:DUSP8:SYVN1:ACAN:KPNB1:SPAG9:TUBB4A:GSK3A:HPCAL1:BCL11A:DCTN1:IGFBP5:NIPBL:EVX1:PRKAR2B
TF_targets	TCF11_01	257	17	0.01807133129156213	0.032517734772427434	CTSK:PKLR:RIT1:HECTD2:KCNA1:C15orf26:SPAG9:PHLPP1:FBXO36:EIF4E:NIPBL:CDX1:CPEB4:TSPAN17:PI16:AIG1:CNTLN

TF_targets	AHRARNT_01	142	11	0.018635748043923154	0.033434724431744486	SPEN:CKS1B:SOAT1:C12orf57:GTF2A1:KIAA1199:PITPNC1:HPCAL1:BCL11A:NIPBL:CKS1B:JAZF1
TF_targets	PBX1_01	259	17	0.01933097315637279	0.03458033321227977	ZNF143:ZNHIT2:CCER1:SIAH3:SIX6:STAC2:KIF19:PTGER1:FAM49A:UCKL1:GSK3B:TAPBP:HOXA11:CALD1:NUDCD1:CNTLN:PAPPA
TF_targets	SF1_Q6	260	17	0.01998551492377976	0.035646678665221206	SMG5:TMEM79:APBB1:IPO7:HOXC13:LRFN5:EPN2:HNF1B:STAC2:SPAG9:ST8SIA5:SAFB:TUBB4A:INSR:C19orf57:ATP6V1B1:RBMS1
TF_targets	GATAAGR_GATA_C	301	19	0.02020342260283308	0.03593028509541743	RHCE:PKLR:SOAT1:MOGAT2:ENO2:SOX5:RAP1B:TNFRSF19:NFATC3:SPAG9:EPG5:IGFBP5:CYB5R3:HYAL3:NAT6:ABCF3:ISL1:EDN1:SLC34A3
TF_targets	ETS2_B	281	18	0.020478346608062713	0.036313347182901905	TSPAN1:ZNF143:POU2AF1:LCP1:PSEN1:TCF12:SAFB:RTKN:ZNFX1:STX19:RAB43:NIPBL:ARAP3:HOXA10:EVX1:GNG11:LYN:TMEM71
TF_targets	PAX4_04	221	15	0.02061634247439116	0.03641820475975079	NRAS:FAM63A:PRUNE:PHLDA3:EMP1:HOXC11:TCF12:C15orf26:ERF:BCL11A:FOXP1:GSK3B:NIPBL:CALD1:PAPPA
TF_targets	WHN_B	261	17	0.02065688335553078	0.03641820475975079	TMEM57:ZNF362:DNAJB4:EXTL2:CSDE1:HOXC11:GRIN2A:DNAH9:GJC1:HPCAL1:ALK:ARMC8:SENP2:SNCB:PRSS16:SRRM3:PAPPA
TF_targets	DBP_Q6	262	17	0.021345337284934297	0.03725101809940197	TMEM57:HECTD2:OPALIN:USP5:PCDH9:C13orf35:ACAN:ESRP2:UBALD2:PUM2:AFF3:ATP2C1:GPRIN3:DHX29:SKIV2L2:TAPBP:CALD1
TF_targets	HNF3B_01	222	15	0.021370298058035263	0.03725101809940197	RIMKLA:ABL2:PCDH9:FOXG1:GTF2A1:PPP1CB:AFF3:GPRIN3:CPEB4:FLOT1:IER3:HOXA11:CALD1:ZMAT4:CNTLN
TF_targets	AP2ALPHA_01	242	16	0.021434602217852608	0.03725101809940197	ZNF362:PHC2:ELOVL1:SEMA6C:SOX5:SMG1:NFATC3:SPAG9:PKN1:ERF:CIC:SCN5A:TSPAN17:C6orf89:ZFAND3:NYAP1
TF_targets	E2F1_Q6_01	242	16	0.021434602217852608	0.03725101809940197	RNF207:ZNF362:HCN3:IPO7:WEE1:DLEU1:C14orf142:UBR7:ERF:NCL:PCNA:NIPBL:ZCWPW1:MEPCE:RB1CC1:TSTD2
TF_targets	SMAD3_Q6	242	16	0.021434602217852608	0.03725101809940197	ELOVL1:TXNDC12:ANXA9:RUSC1:SMG5:TMEM79:PTPN6:SIAH3:MFAP4:GRIN2D:FOXP1:ZNF654:PLCXD2:EDN1:CDKN1A:THBS2
TF_targets	ER_Q6	283	18	0.02180675116436075	0.03779010855187516	TMEM57:DNAJB4:FAM63A:PRUNE:LRFN4:SLC25A3:LCP1:KCNJ2:CIC:CTU1:GREB1:FAM49A:OSR1:PPARA:DACT2:ITGB8:RB1CC1:UBQLN1
TF_targets	NKX62_Q2	243	16	0.022174692724061956	0.03831887411240168	MRPL49:HOXC11:LRFN5:POLR2C:IKZF3:ADAM11:BCL11A:XPO1:KCNS1:FLOT1:IER3:HOXA10:HOXA11:CLDN4:ABCB1:CALD1
TF_targets	HFH4_01	204	14	0.022752826891068573	0.03902377869144676	TXNDC12:NRAS:TMOD4:RBP3:SIAH3:RPS6KB1:CHST8:PPP1CB:FBXO36:EIF4E:CPEB4:TSPAN17:HOXA10:HOXA11
TF_targets	GATA_Q6	204	14	0.022752826891068573	0.03902377869144676	RHCE:PKLR:SFRP5:MOGAT2:ENO2:RAP1B:PCDH9:TNFSF13B:DCAF11:IGFBP5:CYB5R3:ISL1:EDN1:CPA1
TF_targets	MAF_Q6	264	17	0.02277453313795909	0.03902377869144676	SEMA4A:CUL2:MRPL49:POU2AF1:ERC1:NUMB:HNF1B:STAC2:KPNB1:SPAG9:UTP18:KCNJ16:WDR54:HYAL2:AP2M1:CREB3L2:SLC34A3
TF_targets	SRF_Q5_01	224	15	0.02294041480398175	0.03919790764826013	FOXE3:NRAS:CSRP1:KCNA1:LCP1:MYLPF:TGFB1I1:CHST8:DSTN:FOXP1:DVL3:CXCL6:ITGB8:CALD1:ADAMTSL1
TF_targets	NKX25_02	265	17	0.023515788488819207	0.040068801615027134	DNAJB4:LPHN2:CTSK:BBOX1:PRKAB1:PCDH9:KCTD12:NRL:TRPM1:TCF12:LINS:VGLL4:SLC4A4:EIF4E:CEP41:UBQLN1:SLC25A25
TF_targets	GATA3_01	245	16	0.023712673576901048	0.04029172947607142	RHCE:TSPAN1:SOX5:HOXC11:DCAF11:OSR1:IL1RL1:MFF:ATP2C1:ISL1:EDN1:ESR1:ARL4A:HOXA10:NEUROD6:PILRB
TF_targets	TGANNYRGCA_TCF11MAFG_01	307	19	0.024177812137007364	0.04096795945437359	SZT2:CDON:SOX5:FOXG1:MIA2:CTAGE5:SIX6:CACNB1:CD200R1:CNBP:KCNAB1:SAMD7:ABCF3:IPO11:FCHSD1:SNCB:TSPAN17:ESR1:JAZF1:YWHAG
TF_targets	CDP_02	111	9	0.024334200897547648	0.04111873281857082	LPHN2:FAM63A:SIAH3:LCP1:SLC5A10:UCKL1:CDH9:CPEB4:HOXA11

TF_targets	GKCGCNNNNNNNTGAYG_UNKNOWN	60	6	0.024968625949496157	0.041790065688042166	GPAM:FAU:MRPL49:RCE1:TRMT61B:SENP2
TF_targets	PR_Q2	267	17	0.02505288929967686	0.041790065688042166	FBLIM1:SOX5:HOXC12:SIAH3:FITM1:TCF12:PDZD9:UTP18:TMEM145:CACNA2D2:FAM208A:CDH6:KCNIP1:CPEB4:RGL2:SEPT7:ADAMTSL1
TF_targets	GFI1_01	267	17	0.02505288929967686	0.041790065688042166	DNAJB4:ARHGEF2:CREM:CDON:USP5:SOX5:PTHLH:DCAF11:RFX7:TCF12:RPS6KB1:RAB6B:CDH6:EDN1:HOXA10:JAZF1:TMEM71
TF_targets	TTF1_Q6	267	17	0.02505288929967686	0.041790065688042166	TXNDC12:C1orf52:PTBP2:ZFYVE27:ATN1:SLC25A3:CORO1A:PITPNC1:HPCAL1:DCTN1:CREB1:ZNF654:EIF4E:PCDHGC3:ESR1:HOXA10:HOXA13
TF_targets	FOXJ2_01	187	13	0.025074039412825298	0.041790065688042166	C12orf57:KCNK10:RPS6KB1:FAM49A:PPP1CB:UCKL1:CPEB4:TSPAN17:FLOT1:IER3:HOXA10:HOXA11:CNTLN
TF_targets	E2F1_Q3	247	16	0.025329618288407862	0.042066974526256636	ZNF362:HCN3:SYT11:SOAT1:IPO7:KCNA1:SLC25A3:C14orf142:UBR7:SMG1:DCTPP1:C19orf57:NCL:PCNA:NIPBL:CPNE5
TF_targets	CTCNANGTGNY_UNKNOWN	94	8	0.025378109222397447	0.042066974526256636	ALDH18A1:LRFN5:FRMD6:SMPD3:IKZF3:C17orf70:TUBB:HOXA13
TF_targets	CHX10_01	227	15	0.025455925204817747	0.04208161077219194	DNAJB4:SEMA6C:RBP3:HOXC12:PRKAB1:SIAH3:PCDH9:LRFN5:TUBB4A:CHST8:AFF3:CDH9:CDH6:PPAP2A:CA2
TF_targets	KTGGYRSGAA_UNKNOWN	77	7	0.02576822814072393	0.04248275450227459	WEE1:DERL2:C19orf68:ZNF654:CNBP:ZCWPW1:MEPCE
TF_targets	MYOD_01	268	17	0.025849240998604688	0.0425014474640131	TXNDC12:LRRC8C:ARHGEF2:NHLH1:ARL5B:ESRP2:MEGF8:HES6:SCN5A:HYAL2:CACNA2D2:MGAT1:ARL4A:HOXA10:CEP41:CALB1:KIAA0020
TF_targets	YKACATTT_UNKNOWN	289	18	0.02618829135962889	0.04294316593917641	GOLPH3L:TTC12:DDX25:DDX47:NRL:LRFN5:TRPM1:SMPD3:PITPNC1:CHMP4B:ZNFX1:MCHR1:ZNF654:SAMD7:TTC1:HOXA10:BET1:CNTLN
TF_targets	TFIII_Q6	208	14	0.026315858209450198	0.04295844366016405	LMNA:ZNF143:HOXC11:PCDH9:FOXG1:TCF12:ANP32A:ADAM11:KPNB1:UTP18:OSR1:BCL11A:XPO1:FBXO36
TF_targets	CART1_01	228	15	0.026338455621149764	0.04295844366016405	ZNF362:NRAS:SOX5:LRFN5:CALB2:TRPV1:ADAM11:BCL11A:CDH6:HOXA11:NEUROD6:CLDN4:CALD1:CNTLN:PAPPA
TF_targets	OCT1_07	169	12	0.02650162564590056	0.04310931105066491	ABL2:C11orf88:LRFN5:ANKRD11:MBP:TUBB4A:ISL1:CPEB4:HOXA10:JAZF1:NEUROD6:CD36
TF_targets	SOX5_01	269	17	0.026664461926587012	0.04325883450855872	ZNF362:VAV3:DENND2C:SEMA6C:SOX5:CSRNP2:G2E3:ZFYVE1:TCF12:SPAG9:TUBB4A:BCL11A:USP25:C21orf91:NIPBL:ISL1:CPEB4
TF_targets	MYAATNNNNNNNGGC_UNKNOWN	113	9	0.02693339994706933	0.043579241293666554	CDC20:PAQR6:BBOX1:SOX5:CSRNP2:HOXC11:ANP32A:DERL2:PANK2
TF_targets	AAAYRNCTG_UNKNOWN	375	22	0.027642687603571067	0.044608569942270776	PHC2:DNAJB4:POU2AF1:SOX5:LHFP:LMO7:FOXG1:COCH:MOAP1:C15orf26:ESRP2:PLEKHM1:FAM49A:SCN5A:TUSC2:ZNF654:FGF12:SOX30:MGAT1:HOXA10:SEPT7:CNTLN
TF_targets	NFY_C	250	16	0.027908011961968505	0.044917908434830574	ZNF362:FDPS:EHF:TFCP2:SIAH3:DLEU1:DCAF11:DERL2:TECR:RPN2:GNAI2:GSK3B:CDH9:CDH6:BLK:ADAMTSL1
TF_targets	HOXA4_Q2	271	17	0.02835250664569041	0.0455132343522925	PHC2:DNAJB4:ARHGEF2:BBOX1:SNX15:UBASH3B:SOX5:PRKAB1:PCDH9:STAC2:SPAG9:TUBB4A:CHST8:ALK:ZNF654:NDFIP1:CPEB4
TF_targets	E2A_Q2	251	16	0.028809378397079238	0.04612525150188539	MACF1:TMOD4:ARHGEF2:ARL5B:GYLTL1B:FITM1:ARPP19:ESRP2:EPN2:KCNJ2:FSCN2:FAM49A:SPATC1L:SCN5A:EVX1:JAZF1
TF_targets	ATF_B	191	13	0.029125463580928226	0.04650924812661314	RUSC1:CREM:RCE1:LGR5:SPAG9:OSR1:DCTN1:PKP4:MAP3K13:AREG:AREGB:NEUROD6:GEM:SLC25A25
TF_targets	HNF3_Q6	192	13	0.030208729731686413	0.048088091922948724	DNASE2B:NRAS:ARHGEF2:RBP3:GTF2A1:PPP1CB:AFF3:MAP3K13:GPRIN3:CDKN1A:HOXA10:HOXA11:CALD1

TF_targets	CCTNTMAGA_UNKNOWN	134	10	0.030271848030184116	0.048088091922948724	TMEM57:MPL:LMNA:CSRNP2:NRL:GJC1:HYAL2:SAMD7:DVL3:NIPBL
TF_targets	CRGAARNNNNCGA_UNKNOWN	47	5	0.031096617708868714	0.049269965720545233	PTBP2:EXTL2:CREB1:EIF1B:GSK3B
TF_targets	XBP1_01	135	10	0.03163618707803356	0.04999501066735874	CDC42:TMEM125:DNAJB4:REEP3:GTF2A1:KDELR1:ARFGAP3:MAP3K13:HCRTR2:HOXA11
Reactome	REACTOME_POST_TRANSLATIONAL_PROTEIN_MODIFICATION	1427	99	3.984067672995674e-09	5.972117441820517e-06	ICMT:PTP4A2:PHC2:CDC20:BCL10:DPH5:BGLAP:F5:SEC16B:B3GALNT2:TUBB8:RAB18:CUL2:STAMBPL1:CTR9:LINC00610:COMMD9:GYLTL1B:SYVN1:RCE1:HSPA8:OPCML:USP5:SPSB2:FBXW8:COMMD6:UCHL3:LMO7:DCAF11:PSME1:SCFD1:MIA2:CTAGE5:DCAF4:HERC2:OTUD7A:KBTBD13:ADAMTS7:PRSS21:TRAPPC2L:DERL2:ASGR2:BRCA1:TRIM25:PSMD12:NAPG:ST8SIA5:SAFB:TUBB4A:MUC16:KEAP1:CHST8:FBXO17:KDELR1:PUM2:NCOA1:PEX13:DCTN1:METTL21A:IGFBP5:TTLL4:COPS8:PCNA:RPN2:USP25:APOL1:TAB1:ARFGAP3:PPARA:ARSA:RAD18:TGFBR2:TMEM115:PROS1:NR1I2:RAB43:RAB6B:A4GNT:COPB2:SENP2:MFI2:COMMD8:AREG:AREGB:KLHL2:MGAT1:HLA-A:MDC1:MUC21:C4A:RAB44:TNFAIP3:ESR1:THBS2:BET1:COPG2:LY6E:ADAMTSL1:RP11-203J24.9:ST6GALNAC6:ST6GALNAC4:DPM2:TUBB4B
Reactome	REACTOME_METABOLISM_OF_RNA	668	56	2.5346066225308102e-08	1.8996876635868425e-05	RPL22:EXOSC10:EBNA1BP2:WDR77:BCAS2:SMG5:SNRPE:NSUN6:ARL5B-AS1:FAU:HSPA8:EMG1:DDX47:DHX37:WBP4:PSME1:CPSF2:AQR:ANP32A:RPL3L:SMG1:CD2BP2:POLR2C:RPL19:UTP18:PSMD12:RPL38:DDX39A:CTU1:TRMT61B:XPO1:TPRKB:NCL:RTCB:APOBEC3B:TRMU:RPL32:RPL14:ISY1:XRN1:EIF4E:TRMT10A:RPL37:SKIV2L2:DIMT1:UTP15:THG1L:LSM11:RPP40:RPS18:WDR46:PPIL1:GTF2H5:HSPB1:RPL7:PUF60:RPL12
Reactome	REACTOME_DISEASE	1072	77	5.5131802394218296e-08	2.7547523929644406e-05	RPL22:MTOR:NRAS:LMNA:FMOD:PSEN2:KCNJ11:ABCC8:FAU:SYVN1:PC:FDX1:SLC11A2:RAP1B:KPNA3:IPO5:PSME1:PSEN1:GTF2A1:ADAMTS7:SH3GL3:ACAN:RPL3L:POLR2C:DERL2:RPL19:KPNB1:PSMD12:RPL38:TAF4B:TCEB3C:TCEB3B:SH3GL1:CD320:MUC16:AP1M2:GSK3A:XPO1:CREB1:CHMP4B:RPL32:HDAC11:TGFBR2:RPL14:TRAK1:HYAL1:GSK3B:RBP1:RASA2:AP2M1:SLC34A2:SLC4A4:AREG:AREGB:SLC6A19:SLC6A18:RPL37:HDAC3:PDGFRB:HLA-A:MUC21:RPS18:CDKN1A:ESR1:GTF2H5:SOD2:FGFR1OP:THBS2:CD36:RPL7:CYP11B1:CYP11B2:ADAMTSL1:CHMP5:RPL12:DPM2:SLC34A3:NELFB
Reactome	REACTOME_SIGNALING_BY_GPCR	1171	81	1.2173043033957428e-07	4.5618478769755465e-05	HTR6:CDC42:RPE65:VAV3:NRAS:ARHGEF2:ARHGEF11:OR14K1:RBP3:REEP3:TACR2:PSAP:RGR:GRK5:OR51T1:OR4C15:OR4C16:OR4P4:OR4S2:OR4C6:OR5T1:OR8H1:OR8K3:OR8K1:OR8J1:OR8U1:OR5R1:TRPC6:PTHLH:PDE1B:OR10P1:MLNR:GLP2R:RCVRN:MAPK7:DGKE:SSTR2:ARHGEF18:OR1M1:OR7G2:OR7G1:OR7G3:PDE4A:S1PR5:RLN3:CD97:PTGER1:OR10H2:OR10H3:FPR2:PLB1:FSHR:CREB1:MC3R:MCHR1:GNAI2:ACKR4:RBP2:RBP1:IL8:CXCL6:CXCL1:NPY1R:NPY5R:PTGER4:EDN1:OR10C1:HCRTR2:CNR1:CRHR2:GNG11:PRKAR2B:KEL:TAS2R39:TAS2R40:PPP3CC:RDH10:S1PR3:OR1L3:OR1L4:OR1L6
Reactome	REACTOME_DEVELOPMENTAL_BIOLOGY	1100	77	1.5625570881326777e-07	4.684546150221768e-05	RPL22:CDC42:MED8:TAL1:VAV3:NRAS:FLG:LCE1F:LCE1E:LCE1D:LCE1C:LCE1B:PKLR:SEMA4A:ARHGEF11:ABL2:PSEN2:TUBB8:CUL2:KRTAP5-1:KRTAP5-2:KRTAP5-3:KRTAP5-4:KRTAP5-5:FAU:TRPC6:HSPA8:CDON:PSME1:PSEN1:NUMB:TCF12:RPL3L:SIAH1:POLR2C:MAPK7:HNF1B:CACNB1:RPL19:ADAM11:SPAG9:PSMD12:RPL38:MYL12A:MYL12B:TCF3:TUBB4A:NCOA1:ZNF638:PKP4:SCN3A:CREB1:PI3:KRTAP21-3:KRTAP8-1:PPARA:SHANK3:RPL32:SCN5A:RPL14:FOXP1:GSK3B:AP2M1:RPL37:ISL1:HDAC3:RPS18:CDKN1A:CD36:ZNF467:GFRA2:ASH2L:LYN:RPL7:KLF4:RPL12:TUBB4B
Reactome	REACTOME_VESICLE_MEDIATED_TRANSPORT	722	55	7.425294660837074e-07	0.0001855086116099129	LDLRAP1:DENND2C:GJA5:SYT11:F5:SEC16B:TUBB8:RAB18:HPX:VPS51:HSPA8:PRKAB1:ULK1:SCFD1:MIA2:CTAGE5:MYO5A:SH3GL3:TBC1D10B:TRAPPC2L:RABEP1:EPN2:GJC1:KIF19:NAPG:SH3GL1:DENND1C:TUBB4A:AP1M2:EPS15L1:PAFAH1B3:KDELR1:DCTN1:COPS8:RALGAPA2:CHMP4B:APOL1:ARFGAP3:DENND6B:TMEM115:RAB43:RAB6B:COPB2:AP2M1:AREG:AREGB:KIF2A:VPS52:YWHAG:CD36:BET1:COPG2:EXOC4:DENND2A:CHMP5:UBQLN1:TUBB4B
Reactome	REACTOME_RRNA_PROCESSING_IN_THE_NUCLEUS_AND_CYTOSOL	191	23	9.630799776101587e-07	0.00020623669806251827	RPL22:EXOSC10:EBNA1BP2:FAU:EMG1:DDX47:DHX37:RPL3L:RPL19:UTP18:RPL38:NCL:RPL32:RPL14:RPL37:SKIV2L2:DIMT1:UTP15:RPP40:RPS18:WDR46:RPL7:RPL12
Reactome	REACTOME_RRNA_PROCESSING	201	23	2.340077341693069e-06	0.0004384719918997389	RPL22:EXOSC10:EBNA1BP2:FAU:EMG1:DDX47:DHX37:RPL3L:RPL19:UTP18:RPL38:NCL:RPL32:RPL14:RPL37:SKIV2L2:DIMT1:UTP15:RPP40:RPS18:WDR46:RPL7:RPL12

Reactome	REACTOME_INNATE_IMMUNE_SYSTEM	1101	72	4.5416310593897054e-06	0.0007564338842250188	CDC42:BCL10:VAV3:NRAS:SIKE1:CTSS:CTSK:HRNR:FCGR2A:HSPA6:FCGR3A:CFHR5:RAB18:PSAP:LINC00610:COMMD9:UBE2L6:HSPA8:PTPN6:C1S:METTL7A:NCKAP1L:RAP1B:APAF1:PSME1:PSEN1:MYO5A:CHRNB4:CCL17:NFATC3:CLEC10A:MAPK7:VAT1:KPNB1:TRIM25:PSMD12:MUC16:CD97:NFKBIB:CEACAM1:CD33:FPR2:ATP6V1B1:CREB1:SLC11A1:BPIFA2:PI3:SEMG1:SLPI:ZBP1:DNAJC5:SYNGR1:TAB1:CYB5R3:ARSA:PROS1:ARMC8:CXCL1:PLAC8:CFI:DIAPH1:HLA-A:TUBB:MUC21:C4A:RAB44:TNFAIP3:RNASET2:LAT2:CD36:LYN:FCN2:TUBB4B
Reactome	REACTOME_NEUTROPHIL_DEGRANULATION	476	39	5.566331332607457e-06	0.0008343930667578579	NRAS:CTSS:HRNR:FCGR2A:HSPA6:RAB18:PSAP:LINC00610:COMMD9:HSPA8:PTPN6:METTL7A:NCKAP1L:RAP1B:APAF1:PSEN1:CHRNB4:VAT1:KPNB1:PSMD12:CD97:CEACAM1:CD33:FPR2:SLC11A1:SLPI:DNAJC5:SYNGR1:CYB5R3:ARSA:ARMC8:CXCL1:PLAC8:DIAPH1:HLA-A:TUBB:RAB44:RNASET2:CD36:TUBB4B
Reactome	REACTOME_AXON_GUIDANCE	551	42	1.4450828749580831e-05	0.001969253845056515	RPL22:CDC42:VAV3:NRAS:SEMA4A:ARHGEF11:ABL2:PSEN2:TUBB8:CUL2:FAU:TRPC6:HSPA8:PSME1:PSEN1:NUMB:RPL3L:SIAH1:MAPK7:CACNB1:RPL19:PSMD12:RPL38:MYL12A:MYL12B:TUBB4A:SCN3A:CREB1:SHANK3:RPL32:SCN5A:RPL14:GSK3B:AP2M1:RPL37:ISL1:RPS18:GFRA2:LYN:RPL7:RPL12:TUBB4B
Reactome	REACTOME_ASPARAGINE_N_LINKED_GLYCOSYLATION	304	27	3.7405271118324993e-05	0.004654318671261149	F5:SEC16B:TUBB8:SYVN1:SCFD1:MIA2:CTAGE5:TRAPPC2L:DERL2:ASGR2:NAPG:ST8SIA5:TUBB4A:CHST8:KDELR1:DCTN1:RPN2:ARFGAP3:TMEM115:COPB2:AREG:AREGB:MGAT1:BET1:COPG2:RP11-203J24.9:ST6GALNAC6:ST6GALNAC4:DPM2:TUBB4B
Reactome	REACTOME_NEURONAL_SYSTEM	410	33	4.03643380429586e-05	0.004654318671261149	KCNC4:NRAS:HCN3:TUBB8:KCNJ11:ABCC8:LRFN4:HSPA8:KCNA1:PRKAB1:KCNK10:CHRNB4:GRIN2A:CACNB1:GJC1:KCNJ16:KCNJ2:TUBB4A:GRIN2D:KCNJ14:CREB1:KCNS1:DNAJC5:SHANK3:GNAI2:CACNA2D2:KCNAB1:AP2M1:FLOT1:GNG11:PRKAR2B:KCNV2:TUBB4B
Reactome	REACTOME_INFECTIOUS_DISEASE	379	31	5.05155256863282e-05	0.005408769500271855	RPL22:FAU:KPNA3:IPO5:PSME1:GTF2A1:SH3GL3:RPL3L:POLR2C:RPL19:KPNB1:PSMD12:RPL38:TAF4B:TCEB3C:TCEB3B:SH3GL1:AP1M2:XPO1:CHMP4B:RPL32:RPL14:AP2M1:RPL37:HLA-A:RPS18:GTF2H5:RPL7:CHMP5:RPL12:NELFB
Reactome	REACTOME_TRANSPORT_OF_SMALL_MOLECULES	728	49	7.362417201037705e-05	0.006403090341112952	LDLRAP1:LRRC8B:LRRC8C:ABCD3:SOAT1:KCNJ11:SLC43A1:ARL2:TRPC6:PHB2:SLC11A2:PSME1:NIPA2:NIPA1:TRPM1:ABCC11:SLC7A6:SPG7:TRPV1:DERL2:SLC5A10:SLC47A1:SLC47A2:PSMD12:TTYH2:SLC14A1:ATP6V1B1:SLC4A5:SLC9A4:SLC11A1:SMDT1:LMF2:SLC25A26:ATP2C1:AP2M1:SLC34A2:ATP10D:SLC4A4:MTTP:SLC6A19:SLC6A18:SLC12A2:ABCB1:GNG11:PRKAR2B:PIP:CA2:SLC35D2:SLC34A3
Reactome	REACTOME_ER_TO_GOLGI_ANTEROGRADE_TRANSPORT	154	17	7.380871102653604e-05	0.006403090341112952	F5:SEC16B:TUBB8:SCFD1:MIA2:CTAGE5:TRAPPC2L:NAPG:TUBB4A:KDELR1:DCTN1:ARFGAP3:TMEM115:COPB2:AREG:AREGB:BET1:COPG2:TUBB4B
Reactome	REACTOME_SRP_DEPENDENT_COTRANSLATIONAL_PROTEIN_TARGETING_TO_MEMBRANE	111	14	7.430611311830338e-05	0.006403090341112952	RPL22:SSR2:FAU:RPL3L:RPL19:RPL38:RPN2:RPL32:RPL14:SRPRB:RPL37:RPS18:RPL7:RPL12
Reactome	REACTOME_TRANSPORT_TO_THE_GOLGI_AND_SUBSEQUENT_MODIFICATION	185	19	7.688834298868121e-05	0.006403090341112952	F5:SEC16B:TUBB8:SCFD1:MIA2:CTAGE5:TRAPPC2L:NAPG:TUBB4A:CHST8:KDELR1:DCTN1:ARFGAP3:TMEM115:COPB2:AREG:AREGB:MGAT1:BET1:COPG2:TUBB4B
Reactome	REACTOME_G_ALPHA_S_SIGNALLING_EVENTS	539	39	8.844847023440018e-05	0.006856910976271955	HTR6:OR14K1:REEP3:GRK5:OR51T1:OR4C15:OR4C16:OR4P4:OR4S2:OR4C6:OR5T1:OR8H1:OR8K3:OR8K1:OR8J1:OR8U1:OR5R1:PTHLH:PDE1B:OR10P1:GLP2R:OR1M1:OR7G2:OR7G1:OR7G3:PDE4A:RLN3:OR10H2:OR10H3:FSHR:MC3R:GNAI2:PTGER4:OR10C1:CRHR2:GNG11:OR1L3:OR1L4:OR1L6
Reactome	REACTOME_IRE1ALPHA_ACTIVATES_CHAPERONES	50	9	9.148647066406877e-05	0.006856910976271955	ZBTB17:EXTL2:LMNA:SYVN1:PLA2G4B:SULT1A3:GSK3A:DCTN1:SRPRB
Reactome	REACTOME_ADAPTIVE_IMMUNE_SYSTEM	807	52	0.00013456007772308414	0.00960502650032872	MTOR:CDC42:CDC20:BCL10:NRAS:CTSS:CTSK:FCGR3A:TUBB8:CUL2:HECTD2:NCR3LG1:UBE2L6:CRTAM:SPSB2:PTPN6:RAP1B:FBXW8:ORAI1:LMO7:PSME1:HERC2:KBTBD13:SIAH1:NFATC3:NPEPPS:PSMD12:TUBB4A:KEAP1:AP1M2:NFKBIB:FBXO17:CD79A:CD33:SIGLEC6:DCTN1:ANAPC1:CD200R1:UBA5:AP2M1:KLHL2:KIF2A:HLA-A:TAPBP:CD36:PILRB:PILRA:BLK:LYN:LRSAM1:UBAC1:TUBB4B

Reactome	REACTOME_CELL_CYCLE	641	43	0.00021256713964114867	0.014483551923730993	CDC20:CKS1B:LMNA:PMF1:TUBB8:MCM10:WEE1:CDCA5:POLE:ANKLE2:PSME1:HERC2:ARPP19:BRCA1:PSMD12:AZI1:TUBB4A:PPP1CB:XPO1:DCTN1:ANAPC1:MZT2A:PCNA:GTSE1:NCAPH2:SYCE3:GSK3B:TERT:NIPBL:KIF2A:CKS1B:MDC1:TUBB:CDKN1A:SYNE1:FGFR1OP:RFC2:YWHAG:PRKAR2B:CEP41:NCAPG2:LYN:TERF1:TUBB4B
Reactome	REACTOME_INFLUENZA_INFECTION	154	16	0.00024073665027786735	0.015689749511587962	RPL22:FAU:KPNA3:IPO5:RPL3L:POLR2C:RPL19:KPNB1:RPL38:XPO1:RPL32:RPL14:RPL37:RPS18:RPL7:RPL12
Reactome	REACTOME_G_ALPHA_I_SIGNALLING_EVENTS	402	30	0.00032809020865505586	0.01887836106932899	RPE65:RBP3:PSAP:RGR:PDE1B:RCVRN:SSTR2:PDE4A:S1PR5:RLN3:FPR2:PLB1:CREB1:MCHR1:GNAI2:RBP2:RBP1:IL8:CXCL6:CXCL1:NPY1R:NPY5R:CNR1:GNG11:PRKAR2B:TAS2R39:TAS2R40:PPP3CC:RDH10:S1PR3
Reactome	REACTOME_MUSCLE_CONTRACTION	207	19	0.00033258174482375045	0.01887836106932899	HIPK1:TMOD4:ACTA2:KCNJ11:ORAI1:KCNK10:MYLPF:CACNB1:CACNG1:KCNJ2:MYL12A:MYL12B:KCNJ14:SCN3A:SCN5A:CACNA2D2:FGF12:KCNIP1:CALD1
Reactome	REACTOME_ORGANELLE_BIOGENESIS_AND_MAINTENANCE	293	24	0.00033286760646718884	0.01887836106932899	TUBB8:PRKAB1:PKD1:B9D1:AZI1:TUBB4A:NCOA1:DCTN1:CREB1:MCHR1:PPARA:ARL13B:IFT57:NPHP3:HDAC3:TUBB:SOD2:FGFR1OP:TCTE3:YWHAG:PRKAR2B:CEP41:EXOC4:TUBB4B
Reactome	REACTOME_POTASSIUM_CHANNELS	99	12	0.0003458805980442907	0.01887836106932899	KCNC4:HCN3:KCNJ11:ABCC8:KCNA1:KCNK10:KCNJ16:KCNJ2:KCNJ14:KCNS1:KCNAB1:KCNV2
Reactome	REACTOME_METABOLISM_OF_LIPIDS	738	47	0.0003579692854476688	0.01887836106932899	ACOT7:MFSD2A:ELOVL1:MED8:ACOT11:HMGCS2:ARNT:CERS2:TNFAIP8L2:FDPS:PSAP:GPAM:SMPD1:TM7SF2:MOGAT2:DGAT2:FDX1:LPCAT3:MTMR6:FITM1:PLA2G4B:PLA2G15:SMPD3:KPNB1:TECR:LIPE:NCOA1:PLB1:PPP1CB:CDS2:LSS:CYP2D6:PPARA:CPT1B:CHKB:ARSA:CYP8B1:ACAD11:LIPH:PLA2G12A:PPAP2A:HDAC3:CD36:ESYT2:STAR:CYP11B1:CYP11B2
Reactome	REACTOME_NONSENSE_MEDIATED_DECAY_NMD	114	13	0.00036522513076086774	0.01887836106932899	RPL22:SMG5:FAU:RPL3L:SMG1:RPL19:RPL38:RPL32:RPL14:RPL37:RPS18:RPL7:RPL12
Reactome	REACTOME_ENDOGENOUS_STEROLS	27	6	0.0004202975201755078	0.021000866091436206	ARNT:FDX1:NCOA1:CYP8B1:CYP11B1:CYP11B2
Reactome	REACTOME_SIGNALING_BY_EGFR	49	8	0.00044869565454736746	0.021569232714763437	CDC42:NRAS:SH3GL3:SH3GL1:EPS15L1:LRIG1:AREG:AREGB:PTPN3
Reactome	REACTOME_HSF1_DEPENDENT_TRANSACTIVATION	38	7	0.00047617448717821574	0.021569232714763437	MTOR:HSPA6:HSPA8:DNAJB1:DEDD2:HSPB1:GML
Reactome	REACTOME_EXTRA_NUCLEAR_ESTROGEN_SIGNALING	75	10	0.0004930807721906339	0.021569232714763437	NRAS:UHMK1:XPO1:CREB1:GNAI2:AREG:AREGB:ESR1:HSPB1:GNG11:S1PR3
Reactome	REACTOME_GOLGI_TO_ER_RETROGRADE_TRANSPORT	133	14	0.0005042720706121963	0.021569232714763437	TUBB8:RAB18:KIF19:NAPG:TUBB4A:PAFAH1B3:KDELR1:DCTN1:ARFGAP3:RAB6B:COPB2:KIF2A:COPG2:TUBB4B
Reactome	REACTOME_CLASS_A_1_RHODOPSIN_LIKE_RECEPTORS	320	25	0.0005122395567698657	0.021569232714763437	HTR6:TACR2:PSAP:RGR:MLNR:SSTR2:S1PR5:RLN3:PTGER1:FPR2:FSHR:MC3R:MCHR1:ACKR4:IL8:CXCL6:CXCL1:NPY1R:NPY5R:PTGER4:EDN1:HCRTR2:CNR1:KEL:S1PR3
Reactome	REACTOME_ATTENUATION_PHASE	28	6	0.0005180069231030579	0.021569232714763437	HSPA6:HSPA8:DNAJB1:DEDD2:HSPB1:GML
Reactome	REACTOME_GPCR_LIGAND_BINDING	453	32	0.0005497007754850258	0.02212800074329836	HTR6:TACR2:PSAP:RGR:PTHLH:MLNR:GLP2R:SSTR2:S1PR5:RLN3:CD97:PTGER1:FPR2:FSHR:MC3R:MCHR1:ACKR4:IL8:CXCL6:CXCL1:NPY1R:NPY5R:PTGER4:EDN1:HCRTR2:CNR1:CRHR2:GNG11:KEL:TAS2R39:TAS2R40:S1PR3
Reactome	REACTOME_MITOTIC_G2_G2_M_PHASES	199	18	0.0005609499854872166	0.02212800074329836	TUBB8:WEE1:PSME1:PSMD12:AZI1:TUBB4A:PPP1CB:XPO1:DCTN1:MZT2A:GTSE1:TUBB:CDKN1A:FGFR1OP:YWHAG:PRKAR2B:CEP41:TUBB4B

Reactome	REACTOME_UNFOLDED_PROTEIN_RESPONSE_UPR	91	11	0.0006170696964712247	0.023107796108707286	ZBTB17:EXTL2:LMNA:SYVN1:PLA2G4B:SULT1A3:GSK3A:DCTN1:SRPRB:IL8:CREB3L2
Reactome	REACTOME_CILIUM_ASSEMBLY	201	18	0.000631239882282075	0.023107796108707286	TUBB8:PKD1:B9D1:AZI1:TUBB4A:DCTN1:MCHR1:ARL13B:IFT57:NPHP3:TUBB:FGFR1OP:TCTE3:YWHAG:PRKAR2B:CEP41:EXOC4:TUBB4B
Reactome	REACTOME_CELL_CYCLE_MITOTIC	535	36	0.0006320344499379578	0.023107796108707286	CDC20:CKS1B:LMNA:PMF1:TUBB8:MCM10:WEE1:CDCA5:POLE:ANKLE2:PSME1:ARPP19:PSMD12:AZI1:TUBB4A:PPP1CB:XPO1:DCTN1:ANAPC1:MZT2A:PCNA:GTSE1:NCAPH2:GSK3B:NIPBL:KIF2A:CKS1B:TUBB:CDKN1A:FGFR1OP:RFC2:YWHAG:PRKAR2B:CEP41:NCAPG2:LYN:TUBB4B
Reactome	REACTOME_TRANSCRIPTION_COUPLED_NUCLEOTIDE_EXCISION_REPAIR_TC_NER	78	10	0.0006763263993141517	0.024138411251712222	POLE:ERCC5:AQR:POLR2C:COPS8:PCNA:ISY1:GTF2H5:RFC2:XPA
Reactome	REACTOME_DUAL_INCISION_IN_TC_NER	65	9	0.0007052683518302777	0.02458598277659503	POLE:ERCC5:AQR:POLR2C:PCNA:ISY1:GTF2H5:RFC2:XPA
Reactome	REACTOME_PEPTIDE_LIGAND_BINDING_RECEPTORS	188	17	0.0007907570287524478	0.026489985180071184	TACR2:PSAP:MLNR:SSTR2:RLN3:FPR2:MC3R:MCHR1:ACKR4:IL8:CXCL6:CXCL1:NPY1R:NPY5R:EDN1:HCRTR2:KEL
Reactome	REACTOME_NONSENSE_MEDIATED_DECAY_NMD_INDEPENDENT_OF_THE_EXON_JUNCTION_COMPLEX_EJC	94	11	0.0008129014798420777	0.026489985180071184	RPL22:FAU:RPL3L:RPL19:RPL38:RPL32:RPL14:RPL37:RPS18:RPL7:RPL12
Reactome	REACTOME_RECRUITMENT_OF_NUMA_TO_MITOTIC_CENTROSOMES	94	11	0.0008129014798420777	0.026489985180071184	TUBB8:AZI1:TUBB4A:DCTN1:MZT2A:TUBB:FGFR1OP:YWHAG:PRKAR2B:CEP41:TUBB4B
Reactome	REACTOME_CENTROSOME_MATURATION	81	10	0.0009123741406203889	0.028714026400186353	AZI1:TUBB4A:DCTN1:MZT2A:TUBB:FGFR1OP:YWHAG:PRKAR2B:CEP41:TUBB4B
Reactome	REACTOME_EGFR_DOWNREGULATION	31	6	0.0009194618193521982	0.028714026400186353	CDC42:SH3GL3:SH3GL1:EPS15L1:AREG:AREGB:PTPN3
Reactome	REACTOME_GENE_EXPRESSION_TRANSCRIPTION	1478	79	0.0011679829568241944	0.03573074392407077	MTOR:PHC2:MED8:TAL1:LAMTOR5:HIPK1:TRIM33:CTSK:BGLAP:SNRPE:ZNF496:FAS:GPAM:ZNF143:CTR9:YAP1:APAF1:NUAK1:PRKAB1:GTF3A:PSME1:FOXG1:GTF2A1:CPSF2:TDRD9:ZNF770:MGA:TCF12:GRIN2A:RRN3:ZNF771:ZNF720:POLR2C:BRCA1:PSMD12:TNRC6C:TAF4B:TCEB3C:TCEB3B:TCF3:ZNF442:DDX39A:ZNF737:POU2F2:ZNF480:ZNF611:ZNF135:CREB1:ZNF343:PCNA:PPARA:HDAC11:ZNF860:ZNF619:ZNF620:ZNF662:NR1I2:GSK3B:DROSHA:OCLN:HDAC3:LSM11:ZNF311:MDC1:CDKN1A:ESR1:GTF2H5:SOD2:ZNF716:ZNF92:RFC2:YWHAG:BLK:ASH2L:ZNF34:KLF4:ZNF79:GTF3C5:NELFB
Reactome	REACTOME_SELENOAMINO_ACID_METABOLISM	116	12	0.0014385112449786265	0.04283967112853345	RPL22:FAU:RPL3L:RPL19:RPL38:RPL32:RPL14:RPL37:RPS18:INMT:RPL7:RPL12
Reactome	REACTOME_COPI_MEDIATED_ANTEROGRADE_TRANSPORT	101	11	0.001475480450540433	0.04283967112853345	TUBB8:NAPG:TUBB4A:KDELR1:DCTN1:ARFGAP3:TMEM115:COPB2:BET1:COPG2:TUBB4B
Reactome	REACTOME_AURKA_ACTIVATION_BY_TPX2	72	9	0.0014860993320104999	0.04283967112853345	AZI1:TUBB4A:DCTN1:TUBB:FGFR1OP:YWHAG:PRKAR2B:CEP41:TUBB4B
Reactome	REACTOME_REGULATION_OF_PLK1_ACTIVITY	87	10	0.001587562287650781	0.044901054135632466	AZI1:TUBB4A:PPP1CB:DCTN1:TUBB:FGFR1OP:YWHAG:PRKAR2B:CEP41:TUBB4B

	_AT_G2_M_TRANSITION					
Reactome	REACTOME_TRANSLATION	291	22	0.001636333672342239	0.045423410645204006	RPL22:DAP3:SSR2:FAU:MRPL49:MTIF3:MTFMT:RPL3L:RPL19:RPL38:SARS2:MRPS12:RPN2:RPL32:RPL14:SRPRB:MRPS22:EIF4E:RPL37:RPS18:RPL7:RPL12
Reactome	REACTOME_EUKARYOTIC_TRANSLATION_INITIATION	118	12	0.0016666924272373492	0.04542494451688703	RPL22:FAU:RPL3L:RPL19:RPL38:RPL32:RPL14:EIF4E:RPL37:RPS18:RPL7:RPL12
Reactome	REACTOME_INTRA_GOLGI_AND_RETROGRADE_GOLGI_TO_ER_TRAFFIC	202	17	0.0017356686339427235	0.0459870844535649	TUBB8:RAB18:VPS51:KIF19:NAPG:TUBB4A:PAFAH1B3:KDELR1:DCTN1:ARFGAP3:RAB43:RAB6B:COPB2:KIF2A:VPS52:COPG2:TUBB4B
Reactome	REACTOME_INTERLEUKIN_12_SIGNALING	47	7	0.001769216464698715	0.0459870844535649	CDC42:RAP1B:LCP1:BOLA2B:IL12RB1:GSTA2:SOD2
Reactome	REACTOME_METABOLISM_OF_AMINO_ACIDS_AND_DERIVATIVES	369	26	0.00179342734470958	0.0459870844535649	RPL22:RIMKLA:PHGDH:ALDH18A1:BBOX1:ASRGL1:FAU:DAO:SDS:SDSL:SLC25A15:PSME1:RPL3L:RPL19:PSMD12:RPL38:PRODH2:TPO:RPL32:RPL14:RPL37:RPS18:INMT:ASL:RPL7:RPL12
Reactome	REACTOME_GENERIC_TRANSCRIPTION_PATHWAY	1211	66	0.0018100320098467838	0.0459870844535649	MTOR:PHC2:MED8:TAL1:LAMTOR5:HIPK1:TRIM33:CTSK:BGLAP:ZNF496:FAS:GPAM:YAP1:APAF1:NUAK1:PRKAB1:PSME1:FOXG1:ZNF770:MGA:TCF12:GRIN2A:ZNF771:ZNF720:POLR2C:BRCA1:PSMD12:TNRC6C:TAF4B:TCEB3C:TCEB3B:TCF3:ZNF442:ZNF737:ZNF480:ZNF611:ZNF135:CREB1:ZNF343:PCNA:PPARA:HDAC11:ZNF860:ZNF619:ZNF620:ZNF662:NR1I2:GSK3B:OCLN:HDAC3:ZNF311:MDC1:CDKN1A:ESR1:GTF2H5:SOD2:ZNF716:ZNF92:RFC2:YWHAG:BLK:ASH2L:ZNF34:KLF4:ZNF79:NELFB
Reactome	REACTOME_SIGNALING_BY_RECEPTOR_TYROSINE_KINASES	468	31	0.0018490649942257036	0.046195807105738826	MTOR:CDC42:VAV3:NRAS:RIT1:PSEN2:YAP1:PTPN6:NCKAP1L:RAP1B:PSEN1:SH3GL3:POLR2C:ESRP2:MAPK7:SH3GL1:INSR:EPS15L1:ATP6V1B1:CREB1:LRIG1:AP2M1:AREG:AREGB:DIAPH1:PDGFRB:ESR1:THBS2:HSPB1:LYN:PTPN3:NELFB
Reactome	REACTOME_REGULATION_OF_EXPRESSION_OF_SLITS_AND_ROBOS	170	15	0.001994396356862277	0.04847610363728788	RPL22:CUL2:FAU:PSME1:RPL3L:RPL19:PSMD12:RPL38:RPL32:RPL14:RPL37:ISL1:RPS18:RPL7:RPL12
Reactome	REACTOME_DNA_DAMAGE_BYPASS	48	7	0.0020050156274261832	0.04847610363728788	UBE2L6:POLE:TRIM25:NPLOC4:PCNA:RAD18:RFC2
Canonical_Pathways	REACTOME_POST_TRANSLATIONAL_PROTEIN_MODIFICATION	1427	99	3.984067672995674e-09	8.760964812917489e-06	ICMT:PTP4A2:PHC2:CDC20:BCL10:DPH5:BGLAP:F5:SEC16B:B3GALNT2:TUBB8:RAB18:CUL2:STAMBPL1:CTR9:LINC00610:COMMD9:GYLTL1B:SYVN1:RCE1:HSPA8:OPCML:USP5:SPSB2:FBXW8:COMMD6:UCHL3:LMO7:DCAF11:PSME1:SCFD1:MIA2:CTAGE5:DCAF4:HERC2:OTUD7A:KBTBD13:ADAMTS7:PRSS21:TRAPPC2L:DERL2:ASGR2:BRCA1:TRIM25:PSMD12:NAPG:ST8SIA5:SAFB:TUBB4A:MUC16:KEAP1:CHST8:FBXO17:KDELR1:PUM2:NCOA1:PEX13:DCTN1:METTL21A:IGFBP5:TTLL4:COPS8:PCNA:RPN2:USP25:APOL1:TAB1:ARFGAP3:PPARA:ARSA:RAD18:TGFBR2:TMEM115:PROS1:NR1I2:RAB43:RAB6B:A4GNT:COPB2:SENP2:MFI2:COMMD8:AREG:AREGB:KLHL2:MGAT1:HLA-A:MDC1:MUC21:C4A:RAB44:TNFAIP3:ESR1:THBS2:BET1:COPG2:LY6E:ADAMTSL1:RP11-203J24.9:ST6GALNAC6:ST6GALNAC4:DPM2:TUBB4B
Canonical_Pathways	REACTOME_METABOLISM_OF_RNA	668	56	2.5346066225308102e-08	2.786799981472626e-05	RPL22:EXOSC10:EBNA1BP2:WDR77:BCAS2:SMG5:SNRPE:NSUN6:ARL5B-AS1:FAU:HSPA8:EMG1:DDX47:DHX37:WBP4:PSME1:CPSF2:AQR:ANP32A:RPL3L:SMG1:CD2BP2:POLR2C:RPL19:UTP18:PSMD12:RPL38:DDX39A:CTU1:TRMT61B:XPO1:TPRKB:NCL:RTCB:APOBEC3B:TRMU:RPL32:RPL14:ISY1:XRN1:EIF4E:TRMT10A:RPL37:SKIV2L2:DIMT1:UTP15:THG1L:LSM11:RPP40:RPS18:WDR46:PPIL1:GTF2H5:HSPB1:RPL7:PUF60:RPL12
Canonical_Pathways	REACTOME_DISEASE	1072	77	5.5131802394218296e-08	4.041161115496201e-05	RPL22:MTOR:NRAS:LMNA:FMOD:PSEN2:KCNJ11:ABCC8:FAU:SYVN1:PC:FDX1:SLC11A2:RAP1B:KPNA3:IPO5:PSME1:PSEN1:GTF2A1:ADAMTS7:SH3GL3:ACAN:RPL3L:POLR2C:DERL2:RPL19:KPNB1:PSMD12:RPL38:TAF4B:TCEB3C:TCEB3B:SH3GL1:CD320:MUC16:AP1M2:GSK3A:XPO1:CREB1:CHMP4B:RPL32:HDAC11:TGFBR2:RPL14:TRAK1:HYAL1:GSK3B:RBP1:RASA2:AP2M1:SLC34A2:SLC4A4:AREG:AREGB:SLC6A19:SLC6A18:RPL37:HDAC3:PDGFRB:HLA-

						A:MUC21:RPS18:CDKN1A:ESR1:GTF2H5:SOD2:FGFR1OP:THBS2:CD36:RPL7:CYP11B1:CYP11B2:ADAMTSL1:CHMP5:RPL12:DPM2:SLC34A3:NELFB
Canonical_Pathways	REACTOME_SIGNALING_BY_GPCR	1171	81	1.2173043033957428e-07	6.692130407918096e-05	HTR6:CDC42:RPE65:VAV3:NRAS:ARHGEF2:ARHGEF11:OR14K1:RBP3:REEP3:TACR2:PSAP:RGR:GRK5:OR51T1:OR4C15:OR4C16:OR4P4:OR4S2:OR4C6:OR5T1:OR8H1:OR8K3:OR8K1:OR8J1:OR8U1:OR5R1:TRPC6:PTHLH:PDE1B:OR10P1:MLNR:GLP2R:RCVRN:MAPK7:DGKE:SSTR2:ARHGEF18:OR1M1:OR7G2:OR7G1:OR7G3:PDE4A:S1PR5:RLN3:CD97:PTGER1:OR10H2:OR10H3:FPR2:PLB1:FSHR:CREB1:MC3R:MCHR1:GNAI2:ACKR4:RBP2:RBP1:IL8:CXCL6:CXCL1:NPY1R:NPY5R:PTGER4:EDN1:OR10C1:HCRTR2:CNR1:CRHR2:GNG11:PRKAR2B:KEL:TAS2R39:TAS2R40:PPP3CC:RDH10:S1PR3:OR1L3:OR1L4:OR1L6
Canonical_Pathways	REACTOME_DEVELOPMENTAL_BIOLOGY	1100	77	1.5625570881326777e-07	6.872126073607517e-05	RPL22:CDC42:MED8:TAL1:VAV3:NRAS:FLG:LCE1F:LCE1E:LCE1D:LCE1C:LCE1B:PKLR:SEMA4A:ARHGEF11:ABL2:PSEN2:TUBB8:CUL2:KRTAP5-1:KRTAP5-2:KRTAP5-3:KRTAP5-4:KRTAP5-5:FAU:TRPC6:HSPA8:CDON:PSME1:PSEN1:NUMB:TCF12:RPL3L:SIAH1:POLR2C:MAPK7:HNF1B:CACNB1:RPL19:ADAM11:SPAG9:PSMD12:RPL38:MYL12A:MYL12B:TCF3:TUBB4A:NCOA1:ZNF638:PKP4:SCN3A:CREB1:PI3:KRTAP21-3:KRTAP8-1:PPARA:SHANK3:RPL32:SCN5A:RPL14:FOXP1:GSK3B:AP2M1:RPL37:ISL1:HDAC3:RPS18:CDKN1A:CD36:ZNF467:GFRA2:ASH2L:LYN:RPL7:KLF4:RPL12:TUBB4B
Canonical_Pathways	REACTOME_VESICLE_MEDIATED_TRANSPORT	722	55	7.425294660837074e-07	0.00027213704931967875	LDLRAP1:DENND2C:GJA5:SYT11:F5:SEC16B:TUBB8:RAB18:HPX:VPS51:HSPA8:PRKAB1:ULK1:SCFD1:MIA2:CTAGE5:MYO5A:SH3GL3:TBC1D10B:TRAPPC2L:RABEP1:EPN2:GJC1:KIF19:NAPG:SH3GL1:DENND1C:TUBB4A:AP1M2:EPS15L1:PAFAH1B3:KDELR1:DCTN1:COPS8:RALGAPA2:CHMP4B:APOL1:ARFGAP3:DENND6B:TMEM115:RAB43:RAB6B:COPB2:AP2M1:AREG:AREGB:KIF2A:VPS52:YWHAG:CD36:BET1:COPG2:EXOC4:DENND2A:CHMP5:UBQLN1:TUBB4B
Canonical_Pathways	REACTOME_RRNA_PROCESSING_IN_THE_NUCLEUS_AND_CYTOSOL	191	23	9.630799776101587e-07	0.0003025446958235342	RPL22:EXOSC10:EBNA1BP2:FAU:EMG1:DDX47:DHX37:RPL3L:RPL19:UTP18:RPL38:NCL:RPL32:RPL14:RPL37:SKIV2L2:DIMT1:UTP15:RPP40:RPS18:WDR46:RPL7:RPL12
Canonical_Pathways	REACTOME_RRNA_PROCESSING	201	23	2.340077341693069e-06	0.0006432287592978824	RPL22:EXOSC10:EBNA1BP2:FAU:EMG1:DDX47:DHX37:RPL3L:RPL19:UTP18:RPL38:NCL:RPL32:RPL14:RPL37:SKIV2L2:DIMT1:UTP15:RPP40:RPS18:WDR46:RPL7:RPL12
Canonical_Pathways	REACTOME_INNATE_IMMUNE_SYSTEM	1101	72	4.5416310593897054e-06	0.0011096718555108848	CDC42:BCL10:VAV3:NRAS:SIKE1:CTSS:CTSK:HRNR:FCGR2A:HSPA6:FCGR3A:CFHR5:RAB18:PSAP:LINC00610:COMMD9:UBE2L6:HSPA8:PTPN6:C1S:METTL7A:NCKAP1L:RAP1B:APAF1:PSME1:PSEN1:MYO5A:CHRNB4:CCL17:NFATC3:CLEC10A:MAPK7:VAT1:KPNB1:TRIM25:PSMD12:MUC16:CD97:NFKBIB:CEACAM1:CD33:FPR2:ATP6V1B1:CREB1:SLC11A1:BPIFA2:PI3:SEMG1:SLPI:ZBP1:DNAJC5:SYNGR1:TAB1:CYB5R3:ARSA:PROS1:ARMC8:CXCL1:PLAC8:CFI:DIAPH1:HLA-A:TUBB:MUC21:C4A:RAB44:TNFAIP3:RNASET2:LAT2:CD36:LYN:FCN2:TUBB4B
Canonical_Pathways	REACTOME_NEUTROPHIL_DEGRANULATION	476	39	5.566331332607457e-06	0.0012240362600403798	NRAS:CTSS:HRNR:FCGR2A:HSPA6:RAB18:PSAP:LINC00610:COMMD9:HSPA8:PTPN6:METTL7A:NCKAP1L:RAP1B:APAF1:PSEN1:CHRNB4:VAT1:KPNB1:PSMD12:CD97:CEACAM1:CD33:FPR2:SLC11A1:SLPI:DNAJC5:SYNGR1:CYB5R3:ARSA:ARMC8:CXCL1:PLAC8:DIAPH1:HLA-A:TUBB:RAB44:RNASET2:CD36:TUBB4B
Canonical_Pathways	REACTOME_AXON_GUIDANCE	551	42	1.4450828749580831e-05	0.002888852038211659	RPL22:CDC42:VAV3:NRAS:SEMA4A:ARHGEF11:ABL2:PSEN2:TUBB8:CUL2:FAU:TRPC6:HSPA8:PSME1:PSEN1:NUMB:RPL3L:SIAH1:MAPK7:CACNB1:RPL19:PSMD12:RPL38:MYL12A:MYL12B:TUBB4A:SCN3A:CREB1:SHANK3:RPL32:SCN5A:RPL14:GSK3B:AP2M1:RPL37:ISL1:RPS18:GFRA2:LYN:RPL7:RPL12:TUBB4B
Canonical_Pathways	NABA_MATRISOME	1024	65	3.2545858424913754e-05	0.005964028556365446	ANGPTL7:VWA5B1:CDCP2:OVGP1:CTSS:CTSK:ANXA9:SEMA6C:HRNR:FLG:ADAM15:SEMA4A:BGLAP:F13B:FMOD:NID1:GDF2:TLL2:SFRP5:HABP2:DMBT1:HPX:POSTN:TNFSF13B:COCH:PAPLN:ADAMTS7:ACAN:HAPLN3:CCL17:CLEC10A:MFAP4:ADAM11:SERPINB4:MUC16:PODNL1:MEGF8:TPO:IGFBP2:IGFBP5:SNED1:MMP24:TGM2:PI3:SLPI:FBLN1:PLXNB2:HYAL3:HYAL1:HYAL2:ZPLD1:FGF12:IL8:CXCL6:PF4V1:CXCL1:AREG:AREGB:MEGF10:ADAM19:MUC21:THBS2:COL22A1:ADAMTSL1:PAPPA:FCN2
Canonical_Pathways	REACTOME_ASPARAGINE_N_LINKED_GLYCOSYLATION	304	27	3.7405271118324993e-05	0.006327245476092051	F5:SEC16B:TUBB8:SYVN1:SCFD1:MIA2:CTAGE5:TRAPPC2L:DERL2:ASGR2:NAPG:ST8SIA5:TUBB4A:CHST8:KDELR1:DCTN1:RPN2:ARFGAP3:TMEM115:COPB2:AREG:AREGB:MGAT1:BET1:COPG2:RP11-203J24.9:ST6GALNAC6:ST6GALNAC4:DPM2:TUBB4B
Canonical_Pathways	REACTOME_NEURONAL_SYSTEM	410	33	4.03643380429586e-05	0.006340084239747568	KCNC4:NRAS:HCN3:TUBB8:KCNJ11:ABCC8:LRFN4:HSPA8:KCNA1:PRKAB1:KCNK10:CHRNB4:GRIN2A:CACNB1:GJC1:KCNJ16:KCNJ2:TUBB4A:GRIN2D:KCNJ14:CREB1:KCNS1:DNAJC5:SHANK3:GNAI2:CACNA2D2:KCNAB1:AP2M1:FLOT1:GNG11:PRKAR2B:KCNV2:TUBB4B

Canonical_Pathways	REACTOME_INFECTIOUS_DISEASE	379	31	5.05155256863282e-05	0.007405576065615714	RPL22:FAU:KPNA3:IPO5:PSME1:GTF2A1:SH3GL3:RPL3L:POLR2C:RPL19:KPNB1:PSMD12:RPL38:TAF4B:TCEB3C:TCEB3B:SH3GL1:AP1M2:XPO1:CHMP4B:RPL32:RPL14:AP2M1:RPL37:HLA-A:RPS18:GTF2H5:RPL7:CHMP5:RPL12:NELFB
Canonical_Pathways	REACTOME_TRANSPORT_OF_SMALL_MOLECULES	728	49	7.362417201037705e-05	0.008898814012216314	LDLRAP1:LRRC8B:LRRC8C:ABCD3:SOAT1:KCNJ11:SLC43A1:ARL2:TRPC6:PHB2:SLC11A2:PSME1:NIPA2:NIPA1:TRPM1:ABCC11:SLC7A6:SPG7:TRPV1:DERL2:SLC5A10:SLC47A1:SLC47A2:PSMD12:TTYH2:SLC14A1:ATP6V1B1:SLC4A5:SLC9A4:SLC11A1:SMDT1:LMF2:SLC25A26:ATP2C1:AP2M1:SLC34A2:ATP10D:SLC4A4:MTTP:SLC6A19:SLC6A18:SLC12A2:ABCB1:GNG11:PRKAR2B:PIP:CA2:SLC35D2:SLC34A3
Canonical_Pathways	REACTOME_ER_TO_GOLGI_ANTEROGRADE_TRANSPORT	154	17	7.380871102653604e-05	0.008898814012216314	F5:SEC16B:TUBB8:SCFD1:MIA2:CTAGE5:TRAPPC2L:NAPG:TUBB4A:KDELR1:DCTN1:ARFGAP3:TMEM115:COPB2:AREG:AREGB:BET1:COPG2:TUBB4B
Canonical_Pathways	REACTOME_SRP_DEPENDENT_COTRANSLATIONAL_PROTEIN_TARGETING_TO_MEMBRANE	111	14	7.430611311830338e-05	0.008898814012216314	RPL22:SSR2:FAU:RPL3L:RPL19:RPL38:RPN2:RPL32:RPL14:SRPRB:RPL37:RPS18:RPL7:RPL12
Canonical_Pathways	REACTOME_TRANSPORT_TO_THE_GOLGI_AND_SUBSEQUENT_MODIFICATION	185	19	7.688834298868121e-05	0.008898814012216314	F5:SEC16B:TUBB8:SCFD1:MIA2:CTAGE5:TRAPPC2L:NAPG:TUBB4A:CHST8:KDELR1:DCTN1:ARFGAP3:TMEM115:COPB2:AREG:AREGB:MGAT1:BET1:COPG2:TUBB4B
Canonical_Pathways	KEGG_MAPK_SIGNALING_PATHWAY	267	24	8.184002071467294e-05	0.00899831027757829	CDC42:NRAS:HSPA6:FAS:DUSP8:HSPA8:RAP1B:PLA2G4B:JMJD7-PLA2G4B:MAPK7:CACNB1:CACNG1:TAB1:MAPK8IP2:TGFBR2:CACNA2D2:MRAS:RASA2:MAP3K13:FGF12:PLA2G12A:PDGFRB:HSPB1:PPP3CC
Canonical_Pathways	REACTOME_G_ALPHA_S_SIGNALLING_EVENTS	539	39	8.844847023440018e-05	0.009144488590467601	HTR6:OR14K1:REEP3:GRK5:OR51T1:OR4C15:OR4C16:OR4P4:OR4S2:OR4C6:OR5T1:OR8H1:OR8K3:OR8K1:OR8J1:OR8U1:OR5R1:PTHLH:PDE1B:OR10P1:GLP2R:OR1M1:OR7G2:OR7G1:OR7G3:PDE4A:RLN3:OR10H2:OR10H3:FSHR:MC3R:GNAI2:PTGER4:OR10C1:CRHR2:GNG11:OR1L3:OR1L4:OR1L6
Canonical_Pathways	REACTOME_IRE1ALPHA_ACTIVATES_CHAPERONES	50	9	9.148647066406877e-05	0.009144488590467601	ZBTB17:EXTL2:LMNA:SYVN1:PLA2G4B:SULT1A3:GSK3A:DCTN1:SRPRB
Canonical_Pathways	REACTOME_ADAPTIVE_IMMUNE_SYSTEM	807	52	0.00013456007772308414	0.012865113517959218	MTOR:CDC42:CDC20:BCL10:NRAS:CTSS:CTSK:FCGR3A:TUBB8:CUL2:HECTD2:NCR3LG1:UBE2L6:CRTAM:SPSB2:PTPN6:RAP1B:FBXW8:ORAI1:LMO7:PSME1:HERC2:KBTBD13:SIAH1:NFATC3:NPEPPS:PSMD12:TUBB4A:KEAP1:AP1M2:NFKBIB:FBXO17:CD79A:CD33:SIGLEC6:DCTN1:ANAPC1:CD200R1:UBA5:AP2M1:KLHL2:KIF2A:HLA-A:TAPBP:CD36:PILRB:PILRA:BLK:LYN:LRSAM1:UBAC1:TUBB4B
Canonical_Pathways	BIOCARTA_PTDINS_PATHWAY	23	6	0.00016303418301790638	0.014938007019015672	ARHGEF2:RPS6KB1:GSK3A:GSK3B:AP2M1:LYN
Canonical_Pathways	KEGG_INSULIN_SIGNALING_PATHWAY	137	15	0.00020990732410294704	0.017978274618110995	MTOR:NRAS:PKLR:PRKAB1:PCK2:RPS6KB1:INSR:LIPE:CBLC:PPP1CB:GSK3B:EIF4E:EIF4E1B:FLOT1:PRKAR2B
Canonical_Pathways	REACTOME_CELL_CYCLE	641	43	0.00021256713964114867	0.017978274618110995	CDC20:CKS1B:LMNA:PMF1:TUBB8:MCM10:WEE1:CDCA5:POLE:ANKLE2:PSME1:HERC2:ARPP19:BRCA1:PSMD12:AZI1:TUBB4A:PPP1CB:XPO1:DCTN1:ANAPC1:MZT2A:PCNA:GTSE1:NCAPH2:SYCE3:GSK3B:TERT:NIPBL:KIF2A:CKS1B:MDC1:TUBB:CDKN1A:SYNE1:FGFR1OP:RFC2:YWHAG:PRKAR2B:CEP41:NCAPG2:LYN:TERF1:TUBB4B
Canonical_Pathways	PID_MAPK_TRK_PATHWAY	34	7	0.00023139702664147157	0.018846002280910962	NRAS:RUSC1:RIT1:RAP1B:TRPV1:MAPK7:CREB1
Canonical_Pathways	REACTOME_INFLUENZA_INFECTION	154	16	0.00024073665027786735	0.01890642478432251	RPL22:FAU:KPNA3:IPO5:RPL3L:POLR2C:RPL19:KPNB1:RPL38:XPO1:RPL32:RPL14:RPL37:RPS18:RPL7:RPL12
Canonical_Pathways	KEGG_NEUROACTIVE_LIGAND_RECEPTOR_INTERACTION	270	23	0.0002510306210301899	0.019035046056737503	HTR6:TACR2:P2RX2:MLNR:CHRNB4:GRIN2A:TRPV1:GLP2R:SSTR2:S1PR5:PTGER1:GRIN2D:FPR2:FSHR:MC3R:MCHR1:NPY1R:NPY5R:PTGER4:HCRTR2:CNR1:CRHR2:S1PR3

Canonical_Pathways	REACTOME_G_ALPHA_I_SIGNALLING_EVENTS	402	30	0.00032809020865505586	0.02230916840397634	RPE65:RBP3:PSAP:RGR:PDE1B:RCVRN:SSTR2:PDE4A:S1PR5:RLN3:FPR2:PLB1:CREB1:MCHR1:GNAI2:RBP2:RBP1:IL8:CXCL6:CXCL1:NPY1R:NPY5R:CNR1:GNG11:PRKAR2B:TAS2R39:TAS2R40:PPP3CC:RDH10:S1PR3
Canonical_Pathways	REACTOME_MUSCLE_CONTRACTION	207	19	0.00033258174482375045	0.02230916840397634	HIPK1:TMOD4:ACTA2:KCNJ11:ORAI1:KCNK10:MYLPF:CACNB1:CACNG1:KCNJ2:MYL12A:MYL12B:KCNJ14:SCN3A:SCN5A:CACNA2D2:FGF12:KCNIP1:CALD1
Canonical_Pathways	REACTOME_ORGANELLE_BIOGENESIS_AND_MAINTENANCE	293	24	0.00033286760646718884	0.02230916840397634	TUBB8:PRKAB1:PKD1:B9D1:AZI1:TUBB4A:NCOA1:DCTN1:CREB1:MCHR1:PPARA:ARL13B:IFT57:NPHP3:HDAC3:TUBB:SOD2:FGFR1OP:TCTE3:YWHAG:PRKAR2B:CEP41:EXOC4:TUBB4B
Canonical_Pathways	BIOCARTA_CARM_ER_PATHWAY	26	6	0.00033758246204784015	0.02230916840397634	SPEN:PHB2:GTF2A1:BRCA1:HDAC3:ESR1
Canonical_Pathways	REACTOME_POTASSIUM_CHANNELS	99	12	0.0003458805980442907	0.02230916840397634	KCNC4:HCN3:KCNJ11:ABCC8:KCNA1:KCNK10:KCNJ16:KCNJ2:KCNJ14:KCNS1:KCNAB1:KCNV2
Canonical_Pathways	REACTOME_METABOLISM_OF_LIPIDS	738	47	0.0003579692854476688	0.02230916840397634	ACOT7:MFSD2A:ELOVL1:MED8:ACOT11:HMGCS2:ARNT:CERS2:TNFAIP8L2:FDPS:PSAP:GPAM:SMPD1:TM7SF2:MOGAT2:DGAT2:FDX1:LPCAT3:MTMR6:FITM1:PLA2G4B:PLA2G15:SMPD3:KPNB1:TECR:LIPE:NCOA1:PLB1:PPP1CB:CDS2:LSS:CYP2D6:PPARA:CPT1B:CHKB:ARSA:CYP8B1:ACAD11:LIPH:PLA2G12A:PPAP2A:HDAC3:CD36:ESYT2:STAR:CYP11B1:CYP11B2
Canonical_Pathways	REACTOME_NONSENSE_MEDIATED_DECAY_NMD	114	13	0.00036522513076086774	0.02230916840397634	RPL22:SMG5:FAU:RPL3L:SMG1:RPL19:RPL38:RPL32:RPL14:RPL37:RPS18:RPL7:RPL12
Canonical_Pathways	KEGG_RIBOSOME	87	11	0.00041844967938393343	0.0243219538648932	RPL22:FAU:RPL3L:RPL19:RPL38:RPL32:RPL14:RPL37:RPS18:RPL7:RPL12
Canonical_Pathways	REACTOME_ENDOGENOUS_STEROLS	27	6	0.0004202975201755078	0.0243219538648932	ARNT:FDX1:NCOA1:CYP8B1:CYP11B1:CYP11B2
Canonical_Pathways	REACTOME_SIGNALING_BY_EGFR	49	8	0.00044869565454736746	0.025299531906401565	CDC42:NRAS:SH3GL3:SH3GL1:EPS15L1:LRIG1:AREG:AREGB:PTPN3
Canonical_Pathways	REACTOME_HSF1_DEPENDENT_TRANSACTIVATION	38	7	0.00047617448717821574	0.025313271642302758	MTOR:HSPA6:HSPA8:DNAJB1:DEDD2:HSPB1:GML
Canonical_Pathways	KEGG_B_CELL_RECEPTOR_SIGNALING_PATHWAY	75	10	0.0004930807721906339	0.025313271642302758	BCL10:VAV3:NRAS:PTPN6:NFATC3:NFKBIB:CD79A:GSK3B:PPP3CC:LYN
Canonical_Pathways	REACTOME_EXTRA_NUCLEAR_ESTROGEN_SIGNALING	75	10	0.0004930807721906339	0.025313271642302758	NRAS:UHMK1:XPO1:CREB1:GNAI2:AREG:AREGB:ESR1:HSPB1:GNG11:S1PR3
Canonical_Pathways	REACTOME_GOLGI_TO_ER_RETROGRADE_TRANSPORT	133	14	0.0005042720706121963	0.025313271642302758	TUBB8:RAB18:KIF19:NAPG:TUBB4A:PAFAH1B3:KDELR1:DCTN1:ARFGAP3:RAB6B:COPB2:KIF2A:COPG2:TUBB4B
Canonical_Pathways	REACTOME_CLASS_A_1_RHODOPSIN_LIKE_RECEPTORS	320	25	0.0005122395567698657	0.025313271642302758	HTR6:TACR2:PSAP:RGR:MLNR:SSTR2:S1PR5:RLN3:PTGER1:FPR2:FSHR:MC3R:MCHR1:ACKR4:IL8:CXCL6:CXCL1:NPY1R:NPY5R:PTGER4:EDN1:HCRTR2:CNR1:KEL:S1PR3
Canonical_Pathways	REACTOME_ATTENUATION_PHASE	28	6	0.0005180069231030579	0.025313271642302758	HSPA6:HSPA8:DNAJB1:DEDD2:HSPB1:GML
Canonical_Pathways	REACTOME_GPCR_LIGAND_BINDING	453	32	0.0005497007754850258	0.02569852121013311	HTR6:TACR2:PSAP:RGR:PTHLH:MLNR:GLP2R:SSTR2:S1PR5:RLN3:CD97:PTGER1:FPR2:FSHR:MC3R:MCHR1:ACKR4:IL8:CXCL6:CXCL1:NPY1R:NPY5R:PTGER4:EDN1:HCRTR2:CNR1:CRHR2:GNG11:KEL:TAS2R39:TAS2R40:S1PR3
Canonical_Pathways	PID_MYC_REPRESS_PATHWAY	63	9	0.000558345102421188	0.02569852121013311	ZBTB17:CSDE1:HMGCS2:BRCA1:CREB1:SLC11A1:HDAC3:PDGFRB:CDKN1A

Canonical_Pathways	REACTOME_MITOTIC_G2_G2_M_PHASES	199	18	0.0005609499854872166	0.02569852121013311	TUBB8:WEE1:PSME1:PSMD12:AZI1:TUBB4A:PPP1CB:XPO1:DCTN1:MZT2A:GTSE1:TUBB:CDKN1A:FGFR1OP:YWHAG:PRKAR2B:CEP41:TUBB4B
Canonical_Pathways	KEGG_GLYCEROPHOSPHOLIPID_METABOLISM	77	10	0.0006098773001412507	0.02622346708327489	GPAM:LPCAT3:PLA2G4B:JMJD7-PLA2G4B:PLA2G15:DGKE:CDS2:CHKB:PLA2G12A:PPAP2A
Canonical_Pathways	REACTOME_UNFOLDED_PROTEIN_RESPONSE_UPR	91	11	0.0006170696964712247	0.02622346708327489	ZBTB17:EXTL2:LMNA:SYVN1:PLA2G4B:SULT1A3:GSK3A:DCTN1:SRPRB:IL8:CREB3L2
Canonical_Pathways	PID_ERA_GENOMIC_PATHWAY	64	9	0.0006283059630125116	0.02622346708327489	APBB1:PHB2:ANP32A:BRCA1:SAFB:GREB1:NCOA1:PCNA:ESR1
Canonical_Pathways	REACTOME_CILIUM_ASSEMBLY	201	18	0.000631239882282075	0.02622346708327489	TUBB8:PKD1:B9D1:AZI1:TUBB4A:DCTN1:MCHR1:ARL13B:IFT57:NPHP3:TUBB:FGFR1OP:TCTE3:YWHAG:PRKAR2B:CEP41:EXOC4:TUBB4B
Canonical_Pathways	REACTOME_CELL_CYCLE_MITOTIC	535	36	0.0006320344499379578	0.02622346708327489	CDC20:CKS1B:LMNA:PMF1:TUBB8:MCM10:WEE1:CDCA5:POLE:ANKLE2:PSME1:ARPP19:PSMD12:AZI1:TUBB4A:PPP1CB:XPO1:DCTN1:ANAPC1:MZT2A:PCNA:GTSE1:NCAPH2:GSK3B:NIPBL:KIF2A:CKS1B:TUBB:CDKN1A:FGFR1OP:RFC2:YWHAG:PRKAR2B:CEP41:NCAPG2:LYN:TUBB4B
Canonical_Pathways	KEGG_LYSOSOME	121	13	0.0006498381056076144	0.026462851745021183	DNASE2B:CTSS:CTSK:PSAP:SMPD1:SLC11A2:PLA2G15:AP1M2:SLC11A1:NAGA:ARSA:HYAL1:PSAPL1
Canonical_Pathways	REACTOME_TRANSCRIPTION_COUPLED_NUCLEOTIDE_EXCISION_REPAIR_TC_NER	78	10	0.0006763263993141517	0.027040759128942174	POLE:ERCC5:AQR:POLR2C:COPS8:PCNA:ISY1:GTF2H5:RFC2:XPA
Canonical_Pathways	REACTOME_DUAL_INCISION_IN_TC_NER	65	9	0.0007052683518302777	0.027694376887049656	POLE:ERCC5:AQR:POLR2C:PCNA:ISY1:GTF2H5:RFC2:XPA
Canonical_Pathways	REACTOME_PEPTIDE_LIGAND_BINDING_RECEPTORS	188	17	0.0007907570287524478	0.0302978026130971	TACR2:PSAP:MLNR:SSTR2:RLN3:FPR2:MC3R:MCHR1:ACKR4:IL8:CXCL6:CXCL1:NPY1R:NPY5R:EDN1:HCRTR2:KEL
Canonical_Pathways	REACTOME_NONSENSE_MEDIATED_DECAY_NMD_INDEPENDENT_OF_THE_EXON_JUNCTION_COMPLEX_EJC	94	11	0.0008129014798420777	0.0302978026130971	RPL22:FAU:RPL3L:RPL19:RPL38:RPL32:RPL14:RPL37:RPS18:RPL7:RPL12
Canonical_Pathways	REACTOME_RECRUITMENT_OF_NUMA_TO_MITOTIC_CENTROSOMES	94	11	0.0008129014798420777	0.0302978026130971	TUBB8:AZI1:TUBB4A:DCTN1:MZT2A:TUBB:FGFR1OP:YWHAG:PRKAR2B:CEP41:TUBB4B
Canonical_Pathways	REACTOME_CENTROSOME_MATURATION	81	10	0.0009123741406203889	0.033145844930417764	AZI1:TUBB4A:DCTN1:MZT2A:TUBB:FGFR1OP:YWHAG:PRKAR2B:CEP41:TUBB4B
Canonical_Pathways	REACTOME_EGFR_DOWNREGULATION	31	6	0.0009194618193521982	0.033145844930417764	CDC42:SH3GL3:SH3GL1:EPS15L1:AREG:AREGB:PTPN3
Canonical_Pathways	BIOCARTA_CREM_PATHWAY	6	3	0.00097543523684809	0.03459648525530565	CREM:FSHR:XPO1
Canonical_Pathways	KEGG_PATHOGENIC_ESCHERICHIA_COLI_INFECTION	56	8	0.001120905756410944	0.039124948545201044	CDC42:ARHGEF2:TUBB8:TUBB4A:NCL:OCLN:TUBB:TUBB4B

Canonical_Pathways	REACTOME_GENE_EXPRESSION_TRANSCRIPTION	1478	79	0.0011679829568241944	0.040131164407131305	MTOR:PHC2:MED8:TAL1:LAMTOR5:HIPK1:TRIM33:CTSK:BGLAP:SNRPE:ZNF496:FAS:GPAM:ZNF143:CTR9:YAP1:APAF1:NUAK1:PRKAB1:GTF3A:PSME1:FOXG1:GTF2A1:CPSF2:TDRD9:ZNF770:MGA:TCF12:GRIN2A:RRN3:ZNF771:ZNF720:POLR2C:BRCA1:PSMD12:TNRC6C:TAF4B:TCEB3C:TCEB3B:TCF3:ZNF442:DDX39A:ZNF737:POU2F2:ZNF480:ZNF611:ZNF135:CREB1:ZNF343:PCNA:PPARA:HDAC11:ZNF860:ZNF619:ZNF620:ZNF662:NR1I2:GSK3B:DROSHA:OCLN:HDAC3:LSM11:ZNF311:MDC1:CDKN1A:ESR1:GTF2H5:SOD2:ZNF716:ZNF92:RFC2:YWHAG:BLK:ASH2L:ZNF34:KLF4:ZNF79:GTF3C5:NELFB
Canonical_Pathways	PID_HNF3B_PATHWAY	45	7	0.0013624472768216593	0.04609263941124352	PKLR:KCNJ11:ABCC8:UCP2:HNF1B:CREB1:CPT1B
Canonical_Pathways	KEGG_ENDOCYTOSIS	181	16	0.0014109527426765022	0.047010380017358	CDC42:LDLRAP1:HSPA6:GRK5:HSPA8:SH3GL3:RABEP1:EPN2:SH3GL1:CBLC:CHMP4B:ARFGAP3:AP2M1:ARAP3:HLA-A:CHMP5
Canonical_Pathways	REACTOME_SELENOAMINO_ACID_METABOLISM	116	12	0.0014385112449786265	0.04721322727922388	RPL22:FAU:RPL3L:RPL19:RPL38:RPL32:RPL14:RPL37:RPS18:INMT:RPL7:RPL12
Canonical_Pathways	REACTOME_COPI_MEDIATED_ANTEROGRADE_TRANSPORT	101	11	0.001475480450540433	0.047361339581030275	TUBB8:NAPG:TUBB4A:KDELR1:DCTN1:ARFGAP3:TMEM115:COPB2:BET1:COPG2:TUBB4B
Canonical_Pathways	REACTOME_AURKA_ACTIVATION_BY_TPX2	72	9	0.0014860993320104999	0.047361339581030275	AZI1:TUBB4A:DCTN1:TUBB:FGFR1OP:YWHAG:PRKAR2B:CEP41:TUBB4B
Canonical_Pathways	NABA_MATRISOME_ASSOCIATED	750	45	0.0015740094105207998	0.04916971085273334	ANGPTL7:OVGP1:CTSS:CTSK:ANXA9:SEMA6C:HRNR:FLG:ADAM15:SEMA4A:F13B:GDF2:TLL2:SFRP5:HABP2:HPX:TNFSF13B:ADAMTS7:CCL17:CLEC10A:ADAM11:SERPINB4:MUC16:MEGF8:TPO:MMP24:TGM2:PI3:SLPI:PLXNB2:HYAL3:HYAL1:HYAL2:FGF12:IL8:CXCL6:PF4V1:CXCL1:AREG:AREGB:MEGF10:ADAM19:MUC21:ADAMTSL1:PAPPA:FCN2
Canonical_Pathways	REACTOME_REGULATION_OF_PLK1_ACTIVITY_AT_G2_M_TRANSITION	87	10	0.001587562287650781	0.04916971085273334	AZI1:TUBB4A:PPP1CB:DCTN1:TUBB:FGFR1OP:YWHAG:PRKAR2B:CEP41:TUBB4B
Canonical_Pathways	REACTOME_TRANSLATION	291	22	0.001636333672342239	0.04997635757611922	RP+G1:G5914L22:DAP3:SSR2:FAU:MRPL49:MTIF3:MTFMT:RPL3L:RPL19:RPL38:SARS2:MRPS12:RPN2:RPL32:RPL14:SRPRB:MRPS22:EIF4E:RPL37:RPS18:RPL7:RPL12

Supplementary table S10. MAGMA tissue expression analysis
MEAN_SAMPLE_SIZE = 3686
TOTAL_GENES = 17399
TEST_DIRECTION = one-sided, positive (set), one-sided, positive (covar)
CONDITIONED_INTERNAL = gene size, gene density, inverse mac, log(gene size), log(gene density), log(inverse mac)
CONDITIONED_HIDDEN = Average (covar)

VARIABLE	TYPE	NGENES	BETA	BETA_STD	SE	*P*	FULL_NAME
Adipose_Subcutaneous	COVAR	17399	-0.01294	-0.025853	0.010402	0.89324	Adipose_Subcutaneous
Adipose_Visceral_Omentum	COVAR	17399	-0.0010494	-0.0020404	0.010869	0.53846	Adipose_Visceral_Omentum
Adrenal_Gland	COVAR	17399	0.016119	0.031145	0.0098179	0.050329	Adrenal_Gland
Artery_Aorta	COVAR	17399	0.00070453	0.001439	0.0099587	0.4718	Artery_Aorta
Artery_Coronary	COVAR	17399	-0.0020363	-0.004058	0.011512	0.5702	Artery_Coronary
Artery_Tibial	COVAR	17399	-0.0063005	-0.012983	0.0098072	0.7397	Artery_Tibial
Bladder	COVAR	17399	0.0023925	0.004669	0.012805	0.4259	Bladder
Brain_Amygdala	COVAR	17399	-0.0064782	-0.011048	0.0076257	0.8022	Brain_Amygdala
Brain_Anterior_cingulate_cor...	COVAR	17399	-0.0052447	-0.0092737	0.0070305	0.77215	Brain_Anterior_cingulate_cortex_BA24
Brain_Caudate_basal_ganglia	COVAR	17399	-0.0026846	-0.0046423	0.0076298	0.63752	Brain_Caudate_basal_ganglia
Brain_Cerebellar_Hemisphere	COVAR	17399	-0.0079416	-0.015949	0.0059931	0.90742	Brain_Cerebellar_Hemisphere
Brain_Cerebellum	COVAR	17399	-0.0079696	-0.015836	0.0061858	0.90118	Brain_Cerebellum
Brain_Cortex	COVAR	17399	-0.005627	-0.010273	0.0069459	0.79105	Brain_Cortex
Brain_Frontal_Cortex_BA9	COVAR	17399	-0.0065636	-0.012107	0.0067126	0.83591	Brain_Frontal_Cortex_BA9
Brain_Hippocampus	COVAR	17399	-0.00633	-0.010731	0.0077605	0.79265	Brain_Hippocampus
Brain_Hypothalamus	COVAR	17399	-0.004953	-0.0085361	0.0078495	0.73597	Brain_Hypothalamus
Brain_Nucleus_accumbens_basa...	COVAR	17399	-0.0028157	-0.0048905	0.0073823	0.64855	Brain_Nucleus_accumbens_basal_ganglia
Brain_Putamen_basal_ganglia	COVAR	17399	-0.00061099	-0.0010407	0.0076989	0.53163	Brain_Putamen_basal_ganglia
Brain_Spinal_cord_cervical_c...	COVAR	17399	-0.0051716	-0.0093057	0.0083165	0.73297	Brain_Spinal_cord_cervical_c-1
Brain_Substantia_nigra	COVAR	17399	-0.0050275	-0.0086635	0.0082836	0.72804	Brain_Substantia_nigra
Breast_Mammary_Tissue	COVAR	17399	-0.009244	-0.017798	0.012263	0.77451	Breast_Mammary_Tissue
Cells_Cultured_fibroblasts	COVAR	17399	-0.0046121	-0.0098879	0.006451	0.76267	Cells_Cultured_fibroblasts
Cells_EBV-transformed_lympho...	COVAR	17399	-0.0026886	-0.0058677	0.0047844	0.71292	Cells_EBV-transformed_lymphocytes
Cervix_Ectocervix	COVAR	17399	0.0030409	0.005967	0.011957	0.39963	Cervix_Ectocervix
Cervix_Endocervix	COVAR	17399	0.00017862	0.00035406	0.011556	0.49383	Cervix_Endocervix
Colon_Sigmoid	COVAR	17399	-0.018053	-0.035357	0.012169	0.93102	Colon_Sigmoid
Colon_Transverse	COVAR	17399	0.00096804	0.001782	0.011123	0.46533	Colon_Transverse
Esophagus_Gastroesophageal_J...	COVAR	17399	-0.020531	-0.040458	0.012486	0.94994	Esophagus_Gastroesophageal_Junction
Esophagus_Mucosa	COVAR	17399	0.0060087	0.011627	0.0075853	0.21414	Esophagus_Mucosa
Esophagus_Muscularis	COVAR	17399	-0.018449	-0.036427	0.012079	0.93665	Esophagus_Muscularis
Fallopian_Tube	COVAR	17399	-0.0011643	-0.0022619	0.011684	0.53969	Fallopian_Tube
Heart_Atrial_Appendage	COVAR	17399	0.0027782	0.0049919	0.009452	0.38441	Heart_Atrial_Appendage
Heart_Left_Ventricle	COVAR	17399	0.0062665	0.010467	0.0091111	0.2458	Heart_Left_Ventricle
Kidney_Cortex	COVAR	17399	0.020144	0.035434	0.008836	0.011319	Kidney_Cortex

Kidney_Medulla	COVAR	17399	0.01529	0.028307	0.0091026	0.046517	Kidney_Medulla
Liver	COVAR	17399	0.021399	0.038556	0.0062982	0.00034083	Liver
Lung	COVAR	17399	0.0043288	0.0083798	0.0090158	0.31557	Lung
Minor_Salivary_Gland	COVAR	17399	0.0095464	0.017787	0.0093692	0.15413	Minor_Salivary_Gland
Muscle_Skeletal	COVAR	17399	-0.0029379	-0.0055594	0.0067948	0.66726	Muscle_Skeletal
Nerve_Tibial	COVAR	17399	-0.0083145	-0.016756	0.0099986	0.79717	Nerve_Tibial
Ovary	COVAR	17399	-0.012397	-0.025155	0.0095286	0.90337	Ovary
Pancreas	COVAR	17399	0.019188	0.032109	0.00829	0.010324	Pancreas
Pituitary	COVAR	17399	-0.0067414	-0.012585	0.008808	0.77797	Pituitary
Prostate	COVAR	17399	0.0029146	0.0055373	0.011822	0.40263	Prostate
Skin_Not_Sun_Exposed_Suprapu...	COVAR	17399	0.0032356	0.006247	0.0076492	0.33615	Skin_Not_Sun_Exposed_Suprapubic
Skin_Sun_Exposed_Lower_leg	COVAR	17399	0.0030017	0.0058316	0.0076604	0.34759	Skin_Sun_Exposed_Lower_leg
Small_Intestine_Terminal_Ile...	COVAR	17399	0.010951	0.020165	0.0092182	0.11743	Small_Intestine_Terminal_Ileum
Spleen	COVAR	17399	0.0041452	0.0082223	0.0071909	0.28216	Spleen
Stomach	COVAR	17399	0.025157	0.045752	0.011608	0.015116	Stomach
Testis	COVAR	17399	0.0013462	0.0023277	0.0055468	0.40412	Testis
Thyroid	COVAR	17399	0.0048803	0.0096762	0.0096495	0.30652	Thyroid
Uterus	COVAR	17399	-0.0034853	-0.007109	0.010761	0.62698	Uterus
Vagina	COVAR	17399	0.0065518	0.012545	0.0109	0.2739	Vagina
Whole_Blood	COVAR	17399	0.0034811	0.0062921	0.0055876	0.26665	Whole_Blood

Supplementary table S11. Enrichment test results of DEG sets

Category	GeneSet	N_genes	N_overlap	*p*	adj*P*	genes
DEG.up	Adipose_Subcutaneous	2127	118	0.00807960182844803	0.436298498736194	ENSG00000158292:ENSG00000171819:ENSG00000187942:ENSG00000066056:ENSG00000162367:ENSG00000171488:ENSG00000163131:ENSG00000143387:ENSG00000187800:ENSG00000143226:ENSG00000174175:ENSG00000143322:ENSG00000122176:ENSG00000163050:ENSG00000116962:ENSG00000134463:ENSG00000107738:ENSG00000119927:ENSG00000198873:ENSG00000173599:ENSG00000173621:ENSG00000175567:ENSG00000062282:ENSG00000137492:ENSG00000137672:ENSG00000166250:ENSG00000134531:ENSG00000134532:ENSG00000123407:ENSG00000123388:ENSG00000123360:ENSG00000127314:ENSG00000166130:ENSG00000074590:ENSG00000133110:ENSG00000136167:ENSG00000178695:ENSG00000100889:ENSG00000203546:ENSG00000136378:ENSG00000140511:ENSG00000085721:ENSG00000140682:ENSG00000196470:ENSG00000103064:ENSG00000172137:ENSG00000132514:ENSG00000184988:ENSG00000189050:ENSG00000154217:ENSG00000065989:ENSG00000269190:ENSG00000079435:ENSG00000105483:ENSG00000105383:ENSG00000115705:ENSG00000065911:ENSG00000115602:ENSG00000153208:ENSG00000115963:ENSG00000153250:ENSG00000018280:ENSG00000162804:ENSG00000155313:ENSG00000157617:ENSG00000100342:ENSG00000177096:ENSG00000075234:ENSG00000177989:ENSG00000251322:ENSG00000170293:ENSG00000144648:ENSG00000068001:ENSG00000184500:ENSG00000144843:ENSG00000113971:ENSG00000129048:ENSG00000158186:ENSG00000114698:ENSG00000163762:ENSG00000169282:ENSG00000174899:ENSG00000075420:ENSG00000145014:ENSG00000152953:ENSG00000109321:ENSG00000185477:ENSG00000205403:ENSG00000164128:ENSG00000164129:ENSG00000113361:ENSG00000171522:ENSG00000132357:ENSG00000086200:ENSG00000175471:ENSG00000120318:ENSG00000131446:ENSG00000078401:ENSG00000164530:ENSG00000118432:ENSG00000118503:ENSG00000120278:ENSG00000186340:ENSG00000253293:ENSG00000152926:ENSG00000213462:ENSG00000135218:ENSG00000127920:ENSG00000005249:ENSG00000106484:ENSG00000196329:ENSG00000120910:ENSG00000164949:ENSG00000120526:ENSG00000044459:ENSG00000136826:ENSG00000243444:ENSG00000160339
DEG.up	Adipose_Visceral_Omentum	1774	115	3.51450674541092e-05	0.0018978336425219	ENSG00000158292:ENSG00000066056:ENSG00000162367:ENSG00000171488:ENSG00000163131:ENSG00000187800:ENSG00000143226:ENSG00000173110:ENSG00000203747:ENSG00000174175:ENSG00000143322:ENSG00000122176:ENSG00000116962:ENSG00000134463:ENSG00000065328:ENSG00000165997:ENSG00000095794:ENSG00000107736:ENSG00000107738:ENSG00000026103:ENSG00000119927:ENSG00000198873:ENSG00000173599:ENSG00000175567:ENSG00000062282:ENSG00000166250:ENSG00000064309:ENSG00000013588:ENSG00000134531:ENSG00000123360:ENSG00000127314:ENSG00000166130:ENSG00000133110:ENSG00000136167:ENSG00000178695:ENSG00000102524:ENSG00000100889:ENSG00000140406:ENSG00000140511:ENSG00000140682:ENSG00000103064:ENSG00000172137:ENSG00000132514:ENSG00000065325:ENSG00000154217:ENSG00000181143:ENSG00000079435:ENSG00000079385:ENSG00000105483:ENSG00000105383:ENSG00000143867:ENSG00000065911:ENSG00000115602:ENSG00000115604:ENSG00000115607:ENSG00000115963:ENSG00000153250:ENSG00000018280:ENSG00000162804:ENSG00000196209:ENSG00000188559:ENSG00000125966:ENSG00000124107:ENSG00000157617:ENSG00000100342:ENSG00000177989:ENSG00000251322:ENSG00000170293:ENSG00000144648:ENSG00000068001:ENSG00000184500:ENSG00000144843:ENSG00000114698:ENSG00000163762:ENSG00000174899:ENSG00000075420:ENSG00000145014:ENSG00000163975:ENSG00000174145:ENSG00000169429:ENSG00000124875:ENSG00000163739:ENSG00000109321:ENSG00000205403:ENSG00000164128:ENSG00000164129:ENSG00000171522:ENSG00000132357:ENSG00000016082:ENSG00000175471:ENSG00000173926:ENSG00000120318:ENSG00000078401:ENSG00000244731:ENSG00000124743:ENSG00000118432:ENSG00000118503:ENSG00000120278:ENSG00000186340:ENSG00000241644:ENSG00000152926:ENSG00000213462:ENSG00000135218:ENSG00000127920:ENSG00000005249:ENSG00000106484:ENSG00000146966:ENSG00000196329:ENSG00000121039:ENSG00000164949:ENSG00000120526:ENSG00000080608:ENSG00000182752:ENSG00000148339:ENSG00000160339
DEG.up	Adrenal_Gland	1549	92	0.00316091281892288	0.170689292221836	ENSG00000198841:ENSG00000162616:ENSG00000197147:ENSG00000162694:ENSG00000198715:ENSG00000203747:ENSG00000057252:ENSG00000163050:ENSG00000134463:ENSG00000095794:ENSG00000138134:ENSG00000026103:ENSG00000119927:ENSG00000165832:ENSG00000149809:ENSG00000174276:ENSG00000173599:ENSG00000137714:ENSG00000023171:ENSG00000111684:ENSG00000135094:ENSG00000139410:ENSG00000139505:ENSG00000123179:ENSG00000118939:ENSG00000150527:ENSG00000170270:ENSG00000254656:ENSG00000172137:ENSG00000167515:ENSG00000205022:ENSG00000181350:ENSG00000142494:ENSG00000068079:ENSG00000168234:ENSG00000101638:ENSG00000081913:ENSG00000104833:ENSG00000263264:ENSG00000104883:ENSG00000237440:ENSG00000104835:ENSG00000079435:ENSG00000079385:ENSG00000163803:ENSG00000115602:ENSG00000153208:ENSG00000163040:ENSG00000157617:ENSG00000160284:ENSG00000184792:ENSG00000170293:ENSG00000114378:ENSG00000081307:ENSG00000158186:ENSG00000175110:ENSG00000114115:ENSG00000073803:ENSG00000114279:ENSG00000173085:ENSG00000205403:ENSG00000164128:ENSG00000164129:ENSG00000132357:ENSG00000113742:ENSG000000481

						40:ENSG00000124787:ENSG00000204344:ENSG00000244731:ENSG00000137168:ENSG00000244067:ENSG00000243955:ENSG00000198945:ENSG00000112357:ENSG00000106038:ENSG00000153814:ENSG00000241644:ENSG00000152926:ENSG00000213462:ENSG00000177679:ENSG00000085563:ENSG00000005249:ENSG00000106484:ENSG00000158623:ENSG00000147465:ENSG00000104499:ENSG00000160882:ENSG00000179142:ENSG00000177335:ENSG00000167081:ENSG00000148335:ENSG00000160339
DEG. up	Artery_Aorta	2768	141	0.0511949644354069	1	ENSG00000116237:ENSG00000171819:ENSG00000162458:ENSG00000065526:ENSG00000187942:ENSG00000157978:ENSG00000160094:ENSG00000127603:ENSG00000186564:ENSG00000162604:ENSG00000162616:ENSG00000153904:ENSG00000162694:ENSG00000168679:ENSG00000143387:ENSG00000160753:ENSG00000132718:ENSG00000116584:ENSG00000187800:ENSG00000143226:ENSG00000203747:ENSG00000122176:ENSG00000162885:ENSG00000165476:ENSG00000165338:ENSG00000059573:ENSG00000203867:ENSG00000198873:ENSG00000184545:ENSG00000268403:ENSG00000166483:ENSG00000168060:ENSG00000162300:ENSG00000166250:ENSG00000082805:ENSG00000010219:ENSG00000013588:ENSG00000134532:ENSG00000123094:ENSG00000087494:ENSG00000123360:ENSG00000139292:ENSG00000166130:ENSG00000111647:ENSG00000235162:ENSG00000074590:ENSG00000139505:ENSG00000133110:ENSG00000136167:ENSG00000102531:ENSG00000136153:ENSG00000134901:ENSG00000165355:ENSG00000179476:ENSG00000087301:ENSG00000165417:ENSG00000261794:ENSG00000186198:ENSG00000103710:ENSG00000234438:ENSG00000136378:ENSG00000157766:ENSG00000140511:ENSG00000008710:ENSG00000103512:ENSG00000140682:ENSG00000141750:ENSG00000184988:ENSG00000078687:ENSG00000141469:ENSG00000171105:ENSG00000065989:ENSG00000132000:ENSG00000123146:ENSG00000099308:ENSG00000269190:ENSG00000167766:ENSG00000143867:ENSG00000065911:ENSG00000153208:ENSG00000153214:ENSG00000153250:ENSG00000231672:ENSG00000018280:ENSG00000162804:ENSG00000125779:ENSG00000101290:ENSG00000125864:ENSG00000124201:ENSG00000157617:ENSG00000100342:ENSG00000198951:ENSG00000177096:ENSG00000144560:ENSG00000144749:ENSG00000114861:ENSG00000184500:ENSG00000144843:ENSG00000172780:ENSG00000240303:ENSG00000113971:ENSG00000129048:ENSG00000158163:ENSG00000175066:ENSG00000114698:ENSG00000169282:ENSG00000075420:ENSG00000013288:ENSG00000169019:ENSG00000145246:ENSG00000109171:ENSG00000145248:ENSG00000164128:ENSG00000164129:ENSG00000240184:ENSG00000197948:ENSG00000164385:ENSG00000079691:ENSG00000146112:ENSG00000137404:ENSG00000235863:ENSG00000164530:ENSG00000198945:ENSG00000112406:ENSG00000146416:ENSG00000131018:ENSG00000186340:ENSG00000153814:ENSG00000241644:ENSG00000075790:ENSG00000182158:ENSG00000147601:ENSG00000164949:ENSG00000178031:ENSG00000186354:ENSG00000213694:ENSG00000136936:ENSG00000243444:ENSG00000136840:ENSG00000148411:ENSG00000198113
DEG. up	Artery_Coronary	2052	99	0.216243884060226	1	ENSG00000171819:ENSG00000162458:ENSG00000187942:ENSG00000160094:ENSG00000127603:ENSG00000162604:ENSG00000162616:ENSG00000168679:ENSG00000163131:ENSG00000160753:ENSG00000132718:ENSG00000187800:ENSG00000143226:ENSG00000203747:ENSG00000174175:ENSG00000122176:ENSG00000162885:ENSG00000165476:ENSG00000026103:ENSG00000165338:ENSG00000188649:ENSG00000203867:ENSG00000119927:ENSG00000198873:ENSG00000168060:ENSG00000166130:ENSG00000235162:ENSG00000074590:ENSG00000133110:ENSG00000136167:ENSG00000134901:ENSG00000102524:ENSG00000179476:ENSG00000186198:ENSG00000103710:ENSG00000136378:ENSG00000103888:ENSG00000157766:ENSG00000140511:ENSG00000140682:ENSG00000132514:ENSG00000166484:ENSG00000128482:ENSG00000184988:ENSG00000078687:ENSG00000141469:ENSG00000132000:ENSG00000123146:ENSG00000079435:ENSG00000063127:ENSG00000143867:ENSG00000065911:ENSG00000153208:ENSG00000153250:ENSG00000231672:ENSG00000018280:ENSG00000101290:ENSG00000124201:ENSG00000157617:ENSG00000100342:ENSG00000198951:ENSG00000177096:ENSG00000144560:ENSG00000114861:ENSG00000184500:ENSG00000169379:ENSG00000114446:ENSG00000144843:ENSG00000158186:ENSG00000114698:ENSG00000169282:ENSG00000075420:ENSG00000013288:ENSG00000145246:ENSG00000109171:ENSG00000205403:ENSG00000113361:ENSG00000086189:ENSG00000253159:ENSG00000120318:ENSG00000164385:ENSG00000079691:ENSG00000146112:ENSG00000164530:ENSG00000198945:ENSG00000186340:ENSG00000241644:ENSG00000152926:ENSG00000135218:ENSG00000127920:ENSG00000075790:ENSG00000182158:ENSG00000120910:ENSG00000147601:ENSG00000164949:ENSG00000186354:ENSG00000213694:ENSG00000136840:ENSG00000148411
DEG. up	Artery_Tibial	2717	144	0.0162627370584273	0.878187801155073	ENSG00000116237:ENSG00000171819:ENSG00000162458:ENSG00000065526:ENSG00000187942:ENSG00000160094:ENSG00000127603:ENSG00000186564:ENSG00000162604:ENSG00000162616:ENSG00000117569:ENSG00000168679:ENSG00000163349:ENSG00000197323:ENSG00000092621:ENSG00000143437:ENSG00000160753:ENSG00000116539:ENSG00000132718:ENSG00000116584:ENSG00000187800:ENSG00000174175:ENSG00000122176:ENSG00000162885:ENSG00000116962:ENSG00000165476:ENSG00000165338:ENSG00000188649:ENSG00000198873:ENSG00000184545:ENSG00000166478:ENSG00000110025:ENSG00000168060:ENSG00000076053:ENSG00000082805:ENSG00000013588:ENSG00000134532:ENSG00000123094:ENSG00000123407:ENSG00000123388:ENSG00000123338:ENSG00000123360:ENSG00000127314:ENSG00000111647:ENSG00000235162:ENSG00000139505:ENSG00000133110:ENSG00000120688:ENSG00000102531:ENSG00000129493:ENSG00000179476:ENSG00000087301:ENSG00000165861:ENSG00000165417:ENSG00000140262:ENSG00000103707:ENSG00000186198:ENSG00000103710:ENSG00000234438:ENSG00000136378:ENSG00000157766:ENSG00000140511:ENSG00000008710:ENSG00000140682:ENSG00000127804:ENSG00000166484:ENSG00000067191:ENSG00000141750:ENSG00000078687:ENSG00000017797:ENSG00000141469:ENSG00000132000:ENSG00000123146:ENSG00000099308:ENSG00000269190:ENSG00000063127:ENSG00000143867:ENSG00000065911:ENSG00000153208:ENSG00000153214:ENSG00000115963:ENSG00000153250:ENSG

						00000118260:ENSG00000231672:ENSG00000132326:ENSG00000088876:ENSG00000101290:ENSG00000089048:ENSG00000125864:ENSG00000124201:ENSG00000157617:ENSG00000177096:ENSG00000186951:ENSG00000144560:ENSG00000144712:ENSG00000170011:ENSG00000177842:ENSG00000144749:ENSG00000114861:ENSG00000184500:ENSG00000169379:ENSG00000113971:ENSG00000158163:ENSG00000114098:ENSG00000114698:ENSG00000169282:ENSG00000075420:ENSG00000013288:ENSG00000145246:ENSG00000109171:ENSG00000164128:ENSG00000164129:ENSG00000113361:ENSG00000171522:ENSG00000067248:ENSG00000039123:ENSG00000086189:ENSG00000164385:ENSG00000079691:ENSG00000146112:ENSG00000118432:ENSG00000146416:ENSG00000131018:ENSG00000272047:ENSG00000186340:ENSG00000253293:ENSG00000005073:ENSG00000153814:ENSG00000241644:ENSG00000075790:ENSG00000182158:ENSG00000120910:ENSG00000147601:ENSG00000164949:ENSG00000120533:ENSG00000131773:ENSG00000196378:ENSG00000044459:ENSG00000186354:ENSG00000213694:ENSG00000136925:ENSG00000136936:ENSG00000243444:ENSG00000148411
DEG.up	Bladder	843	58	0.00077208697135536	0.0416926964531894	ENSG00000158286:ENSG00000162458:ENSG00000187942:ENSG00000116455:ENSG00000160767:ENSG00000122176:ENSG00000165338:ENSG00000166483:ENSG00000149150:ENSG00000166250:ENSG00000082805:ENSG00000013588:ENSG00000123338:ENSG00000165379:ENSG00000179476:ENSG00000139926:ENSG00000140262:ENSG00000103710:ENSG00000140682:ENSG00000132514:ENSG00000065325:ENSG00000182963:ENSG00000141401:ENSG00000123146:ENSG00000269190:ENSG00000105492:ENSG00000198464:ENSG00000143867:ENSG00000144218:ENSG00000197713:ENSG00000124107:ENSG00000100342:ENSG00000177096:ENSG00000138944:ENSG00000197385:ENSG00000182983:ENSG00000114861:ENSG00000158163:ENSG00000114115:ENSG00000109171:ENSG00000120318:ENSG00000135074:ENSG00000113272:ENSG00000155858:ENSG00000164530:ENSG00000105855:ENSG00000253293:ENSG00000005073:ENSG00000106031:ENSG00000169902:ENSG00000127920:ENSG00000005249:ENSG00000147601:ENSG00000121039:ENSG00000164949:ENSG00000213694:ENSG00000167081:ENSG00000196152
DEG.up	Brain_Amygdala	1825	91	0.142584773532593	1	ENSG00000097021:ENSG00000183888:ENSG00000121904:ENSG00000177181:ENSG00000153904:ENSG00000118733:ENSG00000092621:ENSG00000132718:ENSG00000160781:ENSG00000134376:ENSG00000148468:ENSG00000214688:ENSG00000148604:ENSG00000197430:ENSG00000184545:ENSG00000129151:ENSG00000019505:ENSG00000162174:ENSG00000183644:ENSG00000183715:ENSG00000135094:ENSG00000122966:ENSG00000187848:ENSG00000184226:ENSG00000168952:ENSG00000176165:ENSG00000186960:ENSG00000165379:ENSG00000180998:ENSG00000100433:ENSG00000170113:ENSG00000169918:ENSG00000197535:ENSG00000140600:ENSG00000183454:ENSG00000172137:ENSG00000233670:ENSG00000128482:ENSG00000108830:ENSG00000073670:ENSG00000154217:ENSG00000141540:ENSG00000196169:ENSG00000141576:ENSG00000141469:ENSG00000081913:ENSG00000197971:ENSG00000104833:ENSG00000180739:ENSG00000099308:ENSG00000079462:ENSG00000167619:ENSG00000185453:ENSG00000105464:ENSG00000152467:ENSG00000197872:ENSG00000114993:ENSG00000144283:ENSG00000153253:ENSG00000144485:ENSG00000100321:ENSG00000128285:ENSG00000138944:ENSG00000008735:ENSG00000163517:ENSG00000170011:ENSG00000154917:ENSG00000114279:ENSG00000080493:ENSG00000109466:ENSG00000215218:ENSG00000113100:ENSG00000145794:ENSG00000240184:ENSG00000240764:ENSG00000182132:ENSG00000074317:ENSG00000175766:ENSG00000249109:ENSG00000124772:ENSG00000172461:ENSG00000135525:ENSG00000105855:ENSG00000164600:ENSG00000166924:ENSG00000172209:ENSG00000154319:ENSG00000104267:ENSG00000104327:ENSG00000165023:ENSG00000148411
DEG.up	Brain_Anterior_cingulate_cortex_BA24	2376	128	0.0144113404553077	0.778212384586616	ENSG00000097021:ENSG00000183888:ENSG00000121904:ENSG00000177181:ENSG00000117155:ENSG00000153904:ENSG00000197147:ENSG00000069712:ENSG00000118733:ENSG00000132718:ENSG00000160781:ENSG00000134376:ENSG00000148468:ENSG00000214688:ENSG00000148604:ENSG00000197430:ENSG00000184545:ENSG00000006071:ENSG00000129151:ENSG00000019505:ENSG00000162174:ENSG00000173599:ENSG00000149313:ENSG00000023171:ENSG00000109832:ENSG00000183715:ENSG00000111262:ENSG00000087494:ENSG00000123360:ENSG00000135094:ENSG00000122966:ENSG00000184992:ENSG00000215475:ENSG00000184226:ENSG00000168952:ENSG00000176165:ENSG00000186960:ENSG00000165379:ENSG00000180998:ENSG00000006432:ENSG00000100433:ENSG00000170113:ENSG00000169918:ENSG00000197535:ENSG00000140600:ENSG00000183454:ENSG00000179965:ENSG00000172137:ENSG00000109047:ENSG00000233670:ENSG00000181350:ENSG00000128482:ENSG00000067191:ENSG00000141750:ENSG00000108830:ENSG00000073670:ENSG00000198933:ENSG00000154217:ENSG00000180616:ENSG00000141576:ENSG00000141469:ENSG00000101638:ENSG00000081913:ENSG00000197971:ENSG00000104833:ENSG00000065989:ENSG00000099308:ENSG00000124302:ENSG00000079462:ENSG00000167619:ENSG00000105429:ENSG00000185453:ENSG00000105464:ENSG00000152467:ENSG00000115756:ENSG00000197872:ENSG00000171094:ENSG00000119866:ENSG00000153253:ENSG00000144485:ENSG00000125966:ENSG00000124134:ENSG00000100321:ENSG00000128285:ENSG00000138944:ENSG00000205593:ENSG00000008735:ENSG00000251322:ENSG00000163517:ENSG00000170011:ENSG00000168032:ENSG00000240891:ENSG00000154917:ENSG00000158186:ENSG00000169282:ENSG00000114279:ENSG00000174145:ENSG00000145248:ENSG00000080493:ENSG00000109466:ENSG00000215218:ENSG00000113100:ENSG00000181751:ENSG00000145794:ENSG00000240184:ENSG00000242419:ENSG00000240764:ENSG00000164620:ENSG00000182132:ENSG00000074317:ENSG00000175766:ENSG00000249109:ENSG00000124772:ENSG00000118432:ENSG00000172461:ENSG00000164600:ENSG00000177679:ENSG00000166924:ENSG00000172209:ENSG00000154319:ENSG00000168546:ENSG00000165061:ENSG00000023287:ENSG00000104267:ENSG00000104327:ENSG00000131773:ENSG00000165023:ENSG00000148411

DEG.up	Brain_Caudate_basal_ganglia	2216	123	0.00681428429491685	0.36797135192551	ENSG00000097021:ENSG00000183888:ENSG00000158748:ENSG00000121904:ENSG00000177181:ENSG00000243710:ENSG00000117155:ENSG00000153904:ENSG00000069712:ENSG00000118733:ENSG00000092621:ENSG00000132718:ENSG00000160781:ENSG00000171786:ENSG00000134376:ENSG00000148468:ENSG00000214688:ENSG00000148604:ENSG00000197430:ENSG00000095587:ENSG00000184545:ENSG00000006071:ENSG00000129151:ENSG00000162174:ENSG00000173599:ENSG00000149313:ENSG00000183644:ENSG00000023171:ENSG00000109832:ENSG00000183715:ENSG00000111262:ENSG00000010626:ENSG00000123360:ENSG00000135094:ENSG00000122966:ENSG00000215475:ENSG00000184226:ENSG00000168952:ENSG00000176165:ENSG00000186960:ENSG00000100473:ENSG00000180998:ENSG00000100433:ENSG00000170113:ENSG00000169918:ENSG00000197535:ENSG00000140600:ENSG00000183454:ENSG00000103056:ENSG00000172137:ENSG00000007174:ENSG00000108641:ENSG00000128482:ENSG00000180638:ENSG00000067191:ENSG00000108830:ENSG00000154217:ENSG00000141540:ENSG00000196169:ENSG00000141576:ENSG00000141469:ENSG00000081913:ENSG00000197971:ENSG00000104833:ENSG00000180739:ENSG00000099308:ENSG00000079462:ENSG00000188368:ENSG00000167619:ENSG00000185453:ENSG00000198633:ENSG00000152467:ENSG00000197872:ENSG00000119866:ENSG00000144283:ENSG00000153253:ENSG00000123977:ENSG00000144485:ENSG00000125966:ENSG00000184792:ENSG00000100321:ENSG00000205593:ENSG00000008735:ENSG00000163517:ENSG00000168032:ENSG00000004838:ENSG00000007402:ENSG00000114670:ENSG00000154917:ENSG00000158186:ENSG00000169282:ENSG00000114279:ENSG00000145248:ENSG00000080493:ENSG00000164129:ENSG00000109466:ENSG00000113100:ENSG00000113361:ENSG00000175471:ENSG00000145794:ENSG00000253159:ENSG00000240184:ENSG00000240764:ENSG00000164620:ENSG00000182132:ENSG00000074317:ENSG00000249109:ENSG00000124772:ENSG00000172461:ENSG00000135525:ENSG00000164488:ENSG00000105855:ENSG00000177679:ENSG00000085563:ENSG00000166924:ENSG00000005249:ENSG00000184608:ENSG00000154319:ENSG00000165061:ENSG00000104267:ENSG00000104327:ENSG00000165023:ENSG00000148411
DEG.up	Brain_Cerebellar_Hemisphere	6335	283	0.493811453531706	1	ENSG00000158286:ENSG00000097021:ENSG00000198793:ENSG00000065526:ENSG00000183888:ENSG00000204178:ENSG00000157978:ENSG00000160050:ENSG00000160051:ENSG00000116525:ENSG00000160094:ENSG00000121904:ENSG00000177181:ENSG00000134709:ENSG00000117155:ENSG00000162642:ENSG00000153904:ENSG00000069712:ENSG00000117569:ENSG00000162694:ENSG00000118733:ENSG00000116396:ENSG00000085465:ENSG00000163349:ENSG00000197323:ENSG00000052723:ENSG00000143434:ENSG00000163155:ENSG00000160767:ENSG00000143630:ENSG00000143627:ENSG00000160753:ENSG00000116539:ENSG00000132718:ENSG00000116584:ENSG00000242252:ENSG00000160781:ENSG00000132694:ENSG00000171786:ENSG00000143322:ENSG00000177888:ENSG00000134376:ENSG00000143801:ENSG00000163050:ENSG00000198879:ENSG00000148468:ENSG00000241058:ENSG00000188234:ENSG00000107736:ENSG00000165338:ENSG00000197430:ENSG00000155256:ENSG00000184545:ENSG00000205869:ENSG00000185940:ENSG00000110169:ENSG00000268403:ENSG00000188211:ENSG00000187486:ENSG00000006071:ENSG00000019505:ENSG00000162174:ENSG00000110025:ENSG00000168061:ENSG00000173653:ENSG00000173599:ENSG00000173621:ENSG00000179240:ENSG00000149313:ENSG00000154127:ENSG00000109943:ENSG00000023171:ENSG00000109832:ENSG00000064309:ENSG00000183715:ENSG00000082805:ENSG00000139180:ENSG00000111262:ENSG00000010626:ENSG00000110925:ENSG00000074590:ENSG00000110887:ENSG00000122966:ENSG00000188735:ENSG00000184992:ENSG00000177084:ENSG00000215475:ENSG00000184226:ENSG00000152749:ENSG00000134897:ENSG00000092140:ENSG00000179454:ENSG00000198718:ENSG00000185246:ENSG00000087301:ENSG00000180998:ENSG00000006432:ENSG00000165417:ENSG00000100433:ENSG00000170113:ENSG00000128731:ENSG00000169918:ENSG00000174197:ENSG00000137802:ENSG00000243708:ENSG00000243789:ENSG00000197535:ENSG00000181827:ENSG00000103707:ENSG00000188779:ENSG00000140350:ENSG00000140406:ENSG00000140600:ENSG00000185418:ENSG00000008710:ENSG00000183454:ENSG00000103512:ENSG00000183426:ENSG00000085721:ENSG00000157106:ENSG00000155714:ENSG00000102879:ENSG00000132207:ENSG00000180035:ENSG00000179965:ENSG00000140691:ENSG00000197302:ENSG00000103066:ENSG00000132600:ENSG00000103056:ENSG00000172137:ENSG00000167515:ENSG00000205022:ENSG00000197912:ENSG00000196689:ENSG00000005100:ENSG00000109047:ENSG00000233670:ENSG00000141127:ENSG00000072134:ENSG00000128482:ENSG00000067191:ENSG00000141750:ENSG00000108830:ENSG00000073670:ENSG00000225190:ENSG00000198933:ENSG00000011258:ENSG00000011260:ENSG00000153933:ENSG00000154217:ENSG00000153822:ENSG00000180616:ENSG00000141576:ENSG00000078687:ENSG00000141577:ENSG00000185504:ENSG00000134265:ENSG00000101638:ENSG00000197971:ENSG00000104833:ENSG00000065989:ENSG00000132016:ENSG00000132017:ENSG00000132005:ENSG00000127527:ENSG00000124302:ENSG00000262484:ENSG00000269190:ENSG00000028277:ENSG00000160570:ENSG00000079462:ENSG00000188368:ENSG00000167619:ENSG00000105429:ENSG00000105464:ENSG00000142544:ENSG00000198633:ENSG00000167766:ENSG00000152467:ENSG00000176293:ENSG00000115756:ENSG00000084676:ENSG00000171103:ENSG00000162929:ENSG00000075292:ENSG00000144034:ENSG00000217702:ENSG00000188687:ENSG00000005448:ENSG00000183281:ENSG00000198885:ENSG00000144218:ENSG00000115762:ENSG00000144283:ENSG00000153253:ENSG00000186235:ENSG00000144485:ENSG00000125779:ENSG00000101290:ENSG00000101247:ENSG00000125966:ENSG00000203896:ENSG00000183628:ENSG00000184792:ENSG00000128335:ENSG00000100321:ENSG00000100324:ENSG00000100197:ENSG00000100416:ENSG00000205593:ENSG00000205560:ENSG00000008735:ENSG00000251322:ENSG00000170011:ENSG00000168032:ENSG00000177842:ENSG00000182606:ENSG00000182983:ENSG00000186792:ENSG00000114388:ENSG00000007402:ENSG00000180376:ENSG00000240891:ENSG00000082701:ENSG00000154917:ENSG00000114098:ENSG00000155903:ENSG00000175066:ENSG00000169282:ENSG00000073803:ENSG00000163904:ENSG00000114279:ENSG00000080493:ENSG00000185477:ENSG00000151247:ENSG00000123739:ENSG00000215218:ENSG00000113360:ENSG00000082213:ENSG00000164331:ENSG00000175471:ENSG00000181751:ENSG00000168916:ENSG00000145794:ENSG00000240184:ENSG0000024241

						9:ENSG00000240764:ENSG00000164620:ENSG00000155858:ENSG00000182132:ENSG00000113742:ENSG00000074317:ENSG0000175766:ENSG00000249109:ENSG00000124535:ENSG00000079691:ENSG00000118432:ENSG00000172461:ENSG00000135525:ENSG00000131018:ENSG00000213066:ENSG00000153814:ENSG00000164600:ENSG00000146757:ENSG00000177679:ENSG00000085514:ENSG00000078487:ENSG00000160813:ENSG00000166924:ENSG00000172209:ENSG00000158623:ENSG00000214106:ENSG00000023287:ENSG00000147601:ENSG00000104327:ENSG00000196378:ENSG00000230453:ENSG00000165023:ENSG00000136925:ENSG00000148356:ENSG00000170835:ENSG00000148411
DEG. up	Brain_Cerebellum	6198	271	0.651993575652755	1	ENSG00000158286:ENSG00000097021:ENSG00000198793:ENSG00000065526:ENSG00000183888:ENSG00000204178:ENSG0000157978:ENSG00000160050:ENSG00000160051:ENSG00000116525:ENSG00000160094:ENSG00000121904:ENSG00000177181:ENSG00000198198:ENSG00000134709:ENSG00000117155:ENSG00000153904:ENSG00000069712:ENSG00000118733:ENSG00000116396:ENSG00000085465:ENSG00000163349:ENSG00000143434:ENSG00000163155:ENSG00000160767:ENSG00000143630:ENSG00000143627:ENSG00000160753:ENSG00000116539:ENSG00000132718:ENSG00000116584:ENSG00000242252:ENSG00000160781:ENSG00000132694:ENSG00000171786:ENSG00000177888:ENSG00000134376:ENSG00000143801:ENSG00000163050:ENSG00000198879:ENSG00000148468:ENSG00000241058:ENSG00000188234:ENSG00000107736:ENSG00000197430:ENSG00000155256:ENSG00000184545:ENSG00000205869:ENSG00000185940:ENSG00000110169:ENSG00000268403:ENSG00000188211:ENSG00000187486:ENSG00000006071:ENSG00000019505:ENSG00000162174:ENSG00000110025:ENSG00000168061:ENSG00000173653:ENSG00000173599:ENSG00000173621:ENSG00000175564:ENSG00000179240:ENSG00000149313:ENSG00000154127:ENSG00000109943:ENSG00000023171:ENSG00000109832:ENSG00000064309:ENSG00000183715:ENSG00000082805:ENSG00000111262:ENSG00000010626:ENSG00000110925:ENSG00000074590:ENSG00000110887:ENSG00000122966:ENSG00000188735:ENSG00000184992:ENSG00000177084:ENSG00000215475:ENSG00000184226:ENSG00000152749:ENSG00000134897:ENSG00000198718:ENSG00000185246:ENSG00000087301:ENSG00000180998:ENSG00000006432:ENSG00000100433:ENSG00000170113:ENSG00000128731:ENSG00000169918:ENSG00000174197:ENSG00000137802:ENSG00000243708:ENSG00000243789:ENSG00000197535:ENSG00000181827:ENSG00000188779:ENSG00000140350:ENSG00000103888:ENSG00000140406:ENSG00000140600:ENSG00000185418:ENSG00000008710:ENSG00000183454:ENSG00000103512:ENSG00000183426:ENSG00000157106:ENSG00000155714:ENSG00000102879:ENSG00000132207:ENSG00000261052:ENSG00000198064:ENSG00000180035:ENSG00000179965:ENSG00000140691:ENSG00000197302:ENSG00000103066:ENSG00000132600:ENSG00000103056:ENSG00000172137:ENSG00000158717:ENSG00000167515:ENSG00000205022:ENSG00000197912:ENSG00000196689:ENSG00000005100:ENSG00000233670:ENSG00000141127:ENSG00000072134:ENSG00000128482:ENSG00000067191:ENSG00000141750:ENSG00000108830:ENSG00000073670:ENSG00000225190:ENSG00000198933:ENSG00000011258:ENSG00000153933:ENSG00000154217:ENSG00000153822:ENSG00000180616:ENSG00000141576:ENSG00000078687:ENSG00000141577:ENSG00000185504:ENSG00000134265:ENSG00000101638:ENSG00000081913:ENSG00000197971:ENSG00000104833:ENSG00000065989:ENSG00000132016:ENSG00000132017:ENSG00000132005:ENSG00000127527:ENSG00000124302:ENSG00000262484:ENSG00000269190:ENSG00000028277:ENSG00000079462:ENSG00000188368:ENSG00000167619:ENSG00000105429:ENSG00000105464:ENSG00000182324:ENSG00000142544:ENSG00000198633:ENSG00000167766:ENSG00000152467:ENSG00000176293:ENSG00000115756:ENSG00000162929:ENSG00000075292:ENSG00000217702:ENSG00000188687:ENSG00000005448:ENSG00000183281:ENSG00000198885:ENSG00000144218:ENSG00000115762:ENSG00000144283:ENSG00000153253:ENSG00000186235:ENSG00000144485:ENSG00000125779:ENSG00000101290:ENSG00000101247:ENSG00000125966:ENSG00000203896:ENSG00000160190:ENSG00000183628:ENSG00000184792:ENSG00000128335:ENSG00000100321:ENSG00000100324:ENSG00000100197:ENSG00000100416:ENSG00000205593:ENSG00000205560:ENSG00000008735:ENSG00000251322:ENSG00000170011:ENSG00000168032:ENSG00000177842:ENSG00000182606:ENSG00000182983:ENSG00000186792:ENSG00000243477:ENSG00000114388:ENSG00000007402:ENSG00000180376:ENSG00000240891:ENSG00000082701:ENSG00000154917:ENSG00000114098:ENSG00000155903:ENSG00000169282:ENSG00000114279:ENSG00000080493:ENSG00000185477:ENSG00000151247:ENSG00000215218:ENSG00000113360:ENSG00000175471:ENSG00000181751:ENSG00000168916:ENSG00000145794:ENSG00000240184:ENSG00000242419:ENSG00000240764:ENSG00000164620:ENSG00000155858:ENSG00000182132:ENSG00000113742:ENSG00000074317:ENSG00000175766:ENSG00000048140:ENSG00000249109:ENSG00000124535:ENSG00000079691:ENSG00000118432:ENSG00000172461:ENSG00000135525:ENSG00000131018:ENSG00000213066:ENSG00000153814:ENSG00000164600:ENSG00000146757:ENSG00000177679:ENSG00000085514:ENSG00000078487:ENSG00000160813:ENSG00000166924:ENSG00000172209:ENSG00000158623:ENSG00000214106:ENSG00000154319:ENSG00000147601:ENSG00000104327:ENSG00000169436:ENSG00000196378:ENSG00000230453:ENSG00000165023:ENSG00000127080:ENSG00000136925:ENSG00000148356:ENSG00000170835:ENSG00000148411
DEG. up	Brain_Cortex	2803	145	0.0321822418867992	1	ENSG00000097021:ENSG00000183888:ENSG00000158748:ENSG00000121904:ENSG00000177181:ENSG00000117155:ENSG00000153904:ENSG00000197147:ENSG00000069712:ENSG00000118733:ENSG00000116396:ENSG00000092621:ENSG00000160753:ENSG00000132718:ENSG00000160781:ENSG00000162746:ENSG00000134376:ENSG00000148468:ENSG00000214688:ENSG00000148604:ENSG00000197430:ENSG00000184545:ENSG00000187486:ENSG00000006071:ENSG00000129151:ENSG00000019505:ENSG00000162174:ENSG00000173599:ENSG00000173621:ENSG00000149256:ENSG00000023171:ENSG00000109832:ENSG00000183715:ENSG00000111262:ENSG00000123360:ENSG00000074590:ENSG00000135094:ENSG00000122966:ENSG00000139714:ENSG00000184992:ENSG00000215475:ENSG00000184226:ENSG00000168952:ENSG00000176165:ENSG00000186960:ENSG00000165379:ENSG00000180998:ENSG00000006432:ENSG00000100433:ENSG00000170113:ENSG00000169918:ENSG00000197535:ENSG00000140600:ENSG00000183454:ENSG00000102879:ENSG00000180035:ENSG00000179965:ENSG00000

						172137:ENSG00000109047:ENSG00000181350:ENSG00000128482:ENSG00000067191:ENSG00000141750:ENSG00000108830:ENSG00000073670:ENSG00000198933:ENSG00000154217:ENSG00000180616:ENSG00000141540:ENSG00000141576:ENSG00000141431:ENSG00000141469:ENSG00000101638:ENSG00000081913:ENSG00000197971:ENSG00000104833:ENSG00000065989:ENSG00000099308:ENSG00000124302:ENSG00000262484:ENSG00000079462:ENSG00000167619:ENSG00000105429:ENSG00000185453:ENSG00000105464:ENSG00000152467:ENSG00000115756:ENSG00000197872:ENSG00000171094:ENSG00000119866:ENSG00000153253:ENSG00000144485:ENSG00000125966:ENSG00000124134:ENSG00000183628:ENSG00000184792:ENSG00000100321:ENSG00000128285:ENSG00000138944:ENSG00000205593:ENSG00000008735:ENSG00000251322:ENSG00000163517:ENSG00000170011:ENSG00000168032:ENSG00000186792:ENSG00000240891:ENSG00000154917:ENSG00000158186:ENSG00000114279:ENSG00000174145:ENSG00000145248:ENSG00000080493:ENSG00000109466:ENSG00000215218:ENSG00000113100:ENSG00000152669:ENSG00000181751:ENSG00000145794:ENSG00000240184:ENSG00000242419:ENSG00000240764:ENSG00000164620:ENSG00000182132:ENSG00000074317:ENSG00000175766:ENSG00000249109:ENSG00000124772:ENSG00000172461:ENSG00000132429:ENSG00000135525:ENSG00000106113:ENSG00000164600:ENSG00000177679:ENSG00000166924:ENSG00000172209:ENSG00000154319:ENSG00000168546:ENSG00000165061:ENSG00000023287:ENSG00000104267:ENSG00000104327:ENSG00000131773:ENSG00000165023:ENSG00000148411
DEG. up	Brain_Frontal_Cortex_BA9	3142	160	0.0398863642033498	1	ENSG00000097021:ENSG00000183888:ENSG00000158748:ENSG00000121904:ENSG00000177181:ENSG00000117155:ENSG00000162642:ENSG00000153904:ENSG00000197147:ENSG00000069712:ENSG00000162694:ENSG00000118733:ENSG00000116396:ENSG00000132718:ENSG00000160781:ENSG00000162746:ENSG00000134376:ENSG00000148468:ENSG00000148604:ENSG00000138134:ENSG00000197430:ENSG00000095587:ENSG00000165832:ENSG00000184545:ENSG00000187486:ENSG00000006071:ENSG00000129151:ENSG00000019505:ENSG00000162174:ENSG00000173599:ENSG00000149256:ENSG00000149313:ENSG00000023171:ENSG00000109832:ENSG00000183715:ENSG00000111262:ENSG00000123360:ENSG00000074590:ENSG00000135094:ENSG00000122966:ENSG00000139714:ENSG00000184992:ENSG00000215475:ENSG00000184226:ENSG00000168952:ENSG00000176165:ENSG00000186960:ENSG00000165379:ENSG00000180998:ENSG00000006432:ENSG00000100433:ENSG00000170113:ENSG00000169918:ENSG00000197535:ENSG00000140600:ENSG00000183454:ENSG00000102879:ENSG00000179965:ENSG00000172137:ENSG00000109047:ENSG00000181350:ENSG00000128482:ENSG00000067191:ENSG00000141750:ENSG00000108830:ENSG00000073670:ENSG00000198933:ENSG00000154217:ENSG00000180616:ENSG00000141540:ENSG00000141576:ENSG00000134265:ENSG00000141431:ENSG00000141469:ENSG00000101638:ENSG00000081913:ENSG00000197971:ENSG00000104833:ENSG00000065989:ENSG00000099308:ENSG00000124302:ENSG00000262484:ENSG00000079462:ENSG00000167619:ENSG00000105429:ENSG00000185453:ENSG00000105464:ENSG00000152467:ENSG00000115756:ENSG00000197872:ENSG00000171094:ENSG00000119866:ENSG00000162929:ENSG00000163170:ENSG00000144218:ENSG00000144283:ENSG00000153253:ENSG00000144485:ENSG00000101290:ENSG00000125966:ENSG00000124134:ENSG00000183628:ENSG00000184792:ENSG00000100321:ENSG00000128285:ENSG00000138944:ENSG00000205593:ENSG00000008735:ENSG00000251322:ENSG00000163517:ENSG00000170011:ENSG00000168032:ENSG00000186792:ENSG00000240891:ENSG00000154917:ENSG00000158186:ENSG00000169282:ENSG00000073803:ENSG00000114279:ENSG00000174145:ENSG00000145248:ENSG00000080493:ENSG00000151247:ENSG00000109466:ENSG00000215218:ENSG00000113100:ENSG00000175471:ENSG00000181751:ENSG00000145794:ENSG00000240184:ENSG00000242419:ENSG00000240764:ENSG00000164620:ENSG00000155858:ENSG00000182132:ENSG00000074317:ENSG00000175766:ENSG00000249109:ENSG00000124772:ENSG00000118432:ENSG00000172461:ENSG00000132429:ENSG00000135525:ENSG00000106113:ENSG00000164600:ENSG00000177679:ENSG00000085563:ENSG00000166924:ENSG00000005249:ENSG00000172209:ENSG00000154319:ENSG00000168546:ENSG00000165061:ENSG00000023287:ENSG00000104267:ENSG00000104327:ENSG00000129295:ENSG00000131773:ENSG00000165023:ENSG00000148411
DEG. up	Brain_Hippocampus	1970	97	0.163138843994766	1	ENSG00000097021:ENSG00000183888:ENSG00000121904:ENSG00000177181:ENSG00000179178:ENSG00000153904:ENSG00000197147:ENSG00000118733:ENSG00000132718:ENSG00000160781:ENSG00000134376:ENSG00000148468:ENSG00000214688:ENSG00000197430:ENSG00000184545:ENSG00000006071:ENSG00000129151:ENSG00000019505:ENSG00000162174:ENSG00000183644:ENSG00000023171:ENSG00000183715:ENSG00000111262:ENSG00000135094:ENSG00000122966:ENSG00000187848:ENSG00000184226:ENSG00000176165:ENSG00000186960:ENSG00000165379:ENSG00000180998:ENSG00000100433:ENSG00000170113:ENSG00000169918:ENSG00000197535:ENSG00000156206:ENSG00000140600:ENSG00000183454:ENSG00000233670:ENSG00000007174:ENSG00000128482:ENSG00000180638:ENSG00000067191:ENSG00000108830:ENSG00000073670:ENSG00000180616:ENSG00000141540:ENSG00000196169:ENSG00000141576:ENSG00000141469:ENSG00000101638:ENSG00000081913:ENSG00000197971:ENSG00000104833:ENSG00000180739:ENSG00000099308:ENSG00000124302:ENSG00000167619:ENSG00000134323:ENSG00000197872:ENSG00000119866:ENSG00000114993:ENSG00000144283:ENSG00000153253:ENSG00000123977:ENSG00000144485:ENSG00000100321:ENSG00000128285:ENSG00000138944:ENSG00000008735:ENSG00000163517:ENSG00000170011:ENSG00000004838:ENSG00000154917:ENSG00000169282:ENSG00000114279:ENSG00000109466:ENSG00000215218:ENSG00000113100:ENSG00000145794:ENSG00000240764:ENSG00000182132:ENSG00000074317:ENSG00000249109:ENSG00000172461:ENSG00000135525:ENSG00000164600:ENSG00000085563:ENSG00000166924:ENSG00000172209:ENSG00000184608:ENSG00000154319:ENSG00000165061:ENSG00000104267:ENSG00000104327:ENSG00000131773:ENSG00000165023
DEG. up	Brain_Hypothalamus	2539	132	0.0350083730309899	1	ENSG00000097021:ENSG00000183888:ENSG00000158816:ENSG00000121904:ENSG00000177181:ENSG00000116745:ENSG00000153904:ENSG00000069712:ENSG00000118733:ENSG00000116396:ENSG00000143630:ENSG00000132718:ENSG00000160781:ENSG00000160838:ENSG00000171786:ENSG00000134376:ENSG00

						000148468:ENSG00000214688:ENSG00000148604:ENSG00000197430:ENSG00000095587:ENSG00000184545:ENSG00000006071:ENSG00000129151:ENSG00000019505:ENSG00000162174:ENSG00000149256:ENSG00000149313:ENSG00000183644:ENSG00000109832:ENSG00000183715:ENSG00000010626:ENSG00000135094:ENSG00000122966:ENSG00000184992:ENSG00000215475:ENSG00000184226:ENSG00000168952:ENSG00000165379:ENSG00000180998:ENSG00000184302:ENSG00000006432:ENSG00000100433:ENSG00000254656:ENSG00000170113:ENSG00000169918:ENSG00000197535:ENSG00000156206:ENSG00000140600:ENSG00000172137:ENSG00000233670:ENSG00000007174:ENSG00000128482:ENSG00000180638:ENSG00000067191:ENSG00000108830:ENSG00000073670:ENSG00000154217:ENSG00000153822:ENSG00000141540:ENSG00000171595:ENSG00000196169:ENSG00000141576:ENSG00000141469:ENSG00000081913:ENSG00000197971:ENSG00000104833:ENSG00000180739:ENSG00000124302:ENSG00000079462:ENSG00000167619:ENSG00000185453:ENSG00000105464:ENSG00000152467:ENSG00000115756:ENSG00000197872:ENSG00000171094:ENSG00000162929:ENSG00000163040:ENSG00000144283:ENSG00000153253:ENSG00000123977:ENSG00000144485:ENSG00000101290:ENSG00000125966:ENSG00000124089:ENSG00000100321:ENSG00000128285:ENSG00000138944:ENSG00000205593:ENSG00000008735:ENSG00000163517:ENSG00000163576:ENSG00000170011:ENSG00000168032:ENSG00000004838:ENSG00000007402:ENSG00000240891:ENSG00000114670:ENSG00000154917:ENSG00000158186:ENSG00000114279:ENSG00000152953:ENSG00000174145:ENSG00000145248:ENSG00000080493:ENSG00000109466:ENSG00000215218:ENSG00000113100:ENSG00000016082:ENSG00000181751:ENSG00000145794:ENSG00000242419:ENSG00000164620:ENSG00000182132:ENSG00000074317:ENSG00000175766:ENSG00000137252:ENSG00000172461:ENSG00000135525:ENSG00000106113:ENSG00000177679:ENSG00000085563:ENSG00000166924:ENSG00000184608:ENSG00000154319:ENSG00000168546:ENSG00000147465:ENSG00000165061:ENSG00000104267:ENSG00000104327:ENSG00000165023
DEG.up	Brain_Nucleus_accumbens_basal_ganglia	2460	119	0.182993692924749	1	ENSG00000097021:ENSG00000183888:ENSG00000158748:ENSG00000121904:ENSG00000177181:ENSG00000117155:ENSG00000153904:ENSG00000069712:ENSG00000118733:ENSG00000092621:ENSG00000132718:ENSG00000160781:ENSG00000171786:ENSG00000134376:ENSG00000148468:ENSG00000214688:ENSG00000148604:ENSG00000197430:ENSG00000095587:ENSG00000184545:ENSG00000006071:ENSG00000129151:ENSG00000162174:ENSG00000173599:ENSG00000149256:ENSG00000149313:ENSG00000023171:ENSG00000109832:ENSG00000183715:ENSG00000111262:ENSG00000123360:ENSG00000135094:ENSG00000122966:ENSG00000188735:ENSG00000184992:ENSG00000215475:ENSG00000184226:ENSG00000176165:ENSG00000186960:ENSG00000100473:ENSG00000180998:ENSG00000170113:ENSG00000169918:ENSG00000197535:ENSG00000140600:ENSG00000183454:ENSG00000179965:ENSG00000103056:ENSG00000233670:ENSG00000007174:ENSG00000108641:ENSG00000128482:ENSG00000067191:ENSG00000141750:ENSG00000108830:ENSG00000154217:ENSG00000180616:ENSG00000141540:ENSG00000141576:ENSG00000224877:ENSG00000081913:ENSG00000197971:ENSG00000104833:ENSG00000099308:ENSG00000079462:ENSG00000188368:ENSG00000167619:ENSG00000105429:ENSG00000185453:ENSG00000198633:ENSG00000152467:ENSG00000197872:ENSG00000119866:ENSG00000144283:ENSG00000153253:ENSG00000123977:ENSG00000125966:ENSG00000184792:ENSG00000100321:ENSG00000205593:ENSG00000008735:ENSG00000163517:ENSG00000168032:ENSG00000004838:ENSG00000007402:ENSG00000114670:ENSG00000154917:ENSG00000158186:ENSG00000169282:ENSG00000114279:ENSG00000145248:ENSG00000080493:ENSG00000164129:ENSG00000109466:ENSG00000113100:ENSG00000152669:ENSG00000175471:ENSG00000145794:ENSG00000253159:ENSG00000240184:ENSG00000240764:ENSG00000164620:ENSG00000182132:ENSG00000074317:ENSG00000249109:ENSG00000124772:ENSG00000172461:ENSG00000135525:ENSG00000164488:ENSG00000177679:ENSG00000166924:ENSG00000005249:ENSG00000184608:ENSG00000154319:ENSG00000165061:ENSG00000104267:ENSG00000104327:ENSG00000165023:ENSG00000148411
DEG.up	Brain_Putamen_basal_ganglia	1745	92	0.0532765752845281	1	ENSG00000183888:ENSG00000158748:ENSG00000121904:ENSG00000177181:ENSG00000153904:ENSG00000069712:ENSG00000118733:ENSG00000092621:ENSG00000132718:ENSG00000160781:ENSG00000171786:ENSG00000134376:ENSG00000148468:ENSG00000148604:ENSG00000197430:ENSG00000095587:ENSG00000184545:ENSG00000129151:ENSG00000162174:ENSG00000173599:ENSG00000109832:ENSG00000183715:ENSG00000111262:ENSG00000123360:ENSG00000135094:ENSG00000122966:ENSG00000215475:ENSG00000184226:ENSG00000168952:ENSG00000176165:ENSG00000186960:ENSG00000100473:ENSG00000180998:ENSG00000100433:ENSG00000170113:ENSG00000169918:ENSG00000140600:ENSG00000183454:ENSG00000103056:ENSG00000128482:ENSG00000108830:ENSG00000154217:ENSG00000141540:ENSG00000196169:ENSG00000141576:ENSG00000141469:ENSG00000081913:ENSG00000197971:ENSG00000104833:ENSG00000180739:ENSG00000099308:ENSG00000262484:ENSG00000167619:ENSG00000185453:ENSG00000182324:ENSG00000197872:ENSG00000119866:ENSG00000114993:ENSG00000144283:ENSG00000153253:ENSG00000184792:ENSG00000100321:ENSG00000008735:ENSG00000163517:ENSG00000168032:ENSG00000154917:ENSG00000114115:ENSG00000169282:ENSG00000145248:ENSG00000080493:ENSG00000109466:ENSG00000113100:ENSG00000175471:ENSG00000145794:ENSG00000240184:ENSG00000240764:ENSG00000182132:ENSG00000074317:ENSG00000249109:ENSG00000124772:ENSG00000172461:ENSG00000135525:ENSG00000164488:ENSG00000177679:ENSG00000085563:ENSG00000166924:ENSG00000154319:ENSG00000165061:ENSG00000104267:ENSG00000104327:ENSG00000165023:ENSG00000148411
DEG.up	Brain_Spinal_cord_cervical_c-1	2235	114	0.0718636043871518	1	ENSG00000097021:ENSG00000183888:ENSG00000188672:ENSG00000157978:ENSG00000116525:ENSG00000177181:ENSG00000179178:ENSG00000116205:ENSG00000116745:ENSG00000197147:ENSG00000069712:ENSG00000092621:ENSG00000143409:ENSG00000132718:ENSG00000160781:ENSG00000203747:ENSG00000134376:ENSG00000198879:ENSG00000148468:ENSG00000165476:ENSG00000214688:ENSG00000197430:ENSG00000095587:ENSG00000006071:ENSG000001

						29151:ENSG00000162174:ENSG00000173599:ENSG00000139292:ENSG00000110887:ENSG00000139410:ENSG00000122966:ENSG00000184992:ENSG00000184226:ENSG00000129535:ENSG00000168952:ENSG00000180998:ENSG00000165807:ENSG00000080815:ENSG00000100433:ENSG00000170113:ENSG00000169918:ENSG00000140600:ENSG00000185418:ENSG00000172137:ENSG00000167515:ENSG00000029725:ENSG00000233670:ENSG00000007174:ENSG00000141127:ENSG00000072134:ENSG00000180638:ENSG00000108830:ENSG00000123700:ENSG00000141540:ENSG00000196169:ENSG00000141576:ENSG00000134265:ENSG00000141469:ENSG00000081913:ENSG00000197971:ENSG00000104833:ENSG00000180739:ENSG00000124302:ENSG00000262484:ENSG00000167619:ENSG00000079435:ENSG00000134323:ENSG00000162929:ENSG00000217702:ENSG00000114993:ENSG00000144283:ENSG00000153832:ENSG00000186235:ENSG00000144485:ENSG00000089048:ENSG00000125864:ENSG00000154642:ENSG00000008735:ENSG00000163517:ENSG00000178700:ENSG00000154917:ENSG00000114115:ENSG00000123739:ENSG00000109466:ENSG00000068796:ENSG00000181751:ENSG00000168916:ENSG00000145794:ENSG00000064651:ENSG00000182132:ENSG00000074317:ENSG00000137266:ENSG00000164530:ENSG00000172461:ENSG00000132429:ENSG00000135525:ENSG00000146416:ENSG00000120278:ENSG00000026297:ENSG00000105855:ENSG00000146757:ENSG00000086730:ENSG00000085563:ENSG00000078487:ENSG00000185955:ENSG00000105865:ENSG00000146966:ENSG00000197993:ENSG00000104267:ENSG00000104327:ENSG00000131773:ENSG00000165023:ENSG00000167081:ENSG00000148411
DEG.up	Brain_Substantia_nigra	1650	85	0.0915634420399849	1	ENSG00000097021:ENSG00000183888:ENSG00000121904:ENSG00000177181:ENSG00000179178:ENSG00000116745:ENSG00000153904:ENSG00000118733:ENSG00000092621:ENSG00000132718:ENSG00000160781:ENSG00000203747:ENSG00000148468:ENSG00000214688:ENSG00000148604:ENSG00000197430:ENSG00000129151:ENSG00000019505:ENSG00000162174:ENSG00000173599:ENSG00000139292:ENSG00000110887:ENSG00000135094:ENSG00000139410:ENSG00000215475:ENSG00000184226:ENSG00000180998:ENSG00000100433:ENSG00000254656:ENSG00000170113:ENSG00000169918:ENSG00000197535:ENSG00000103710:ENSG00000140600:ENSG00000172137:ENSG00000233670:ENSG00000108830:ENSG00000154217:ENSG00000153822:ENSG00000141540:ENSG00000196169:ENSG00000141576:ENSG00000141469:ENSG00000081913:ENSG00000197971:ENSG00000104833:ENSG00000180739:ENSG00000124302:ENSG00000167619:ENSG00000079435:ENSG00000105464:ENSG00000134323:ENSG00000114993:ENSG00000144283:ENSG00000153253:ENSG00000144485:ENSG00000154642:ENSG00000100321:ENSG00000128285:ENSG00000008735:ENSG00000163517:ENSG00000240891:ENSG00000154917:ENSG00000158186:ENSG00000114115:ENSG00000114279:ENSG00000145248:ENSG00000182223:ENSG00000080493:ENSG00000109466:ENSG00000181751:ENSG00000145794:ENSG00000164620:ENSG00000182132:ENSG00000074317:ENSG00000172461:ENSG00000135525:ENSG00000105855:ENSG00000085563:ENSG00000185955:ENSG00000166924:ENSG00000146966:ENSG00000104267:ENSG00000165023:ENSG00000148411
DEG.up	Breast_Mammary_Tissue	1978	103	0.055640448700789	1	ENSG00000158292:ENSG00000183888:ENSG00000066056:ENSG00000162367:ENSG00000171488:ENSG00000134215:ENSG00000134240:ENSG00000143387:ENSG00000163354:ENSG00000187800:ENSG00000174175:ENSG00000122176:ENSG00000116962:ENSG00000198879:ENSG00000134463:ENSG00000119927:ENSG00000110169:ENSG00000135373:ENSG00000124939:ENSG00000124935:ENSG00000173599:ENSG00000175567:ENSG00000062282:ENSG00000166250:ENSG00000111684:ENSG00000213782:ENSG00000134531:ENSG00000087494:ENSG00000123360:ENSG00000074590:ENSG00000133110:ENSG00000178695:ENSG00000140511:ENSG00000140682:ENSG00000121270:ENSG00000196470:ENSG00000103064:ENSG00000172137:ENSG00000141750:ENSG00000123700:ENSG00000167775:ENSG00000079435:ENSG00000105483:ENSG00000115705:ENSG00000196208:ENSG00000143867:ENSG00000116039:ENSG00000065911:ENSG00000198885:ENSG00000144218:ENSG00000115602:ENSG00000163040:ENSG00000115963:ENSG00000162804:ENSG00000157617:ENSG00000100342:ENSG00000075234:ENSG00000075275:ENSG00000177989:ENSG00000251322:ENSG00000070950:ENSG00000170293:ENSG00000182983:ENSG00000068001:ENSG00000184500:ENSG00000144843:ENSG00000113971:ENSG00000114698:ENSG00000163762:ENSG00000174899:ENSG00000075420:ENSG00000145014:ENSG00000163975:ENSG00000152953:ENSG00000157765:ENSG00000174145:ENSG00000109321:ENSG00000205403:ENSG00000164128:ENSG00000171522:ENSG00000175471:ENSG00000120318:ENSG00000078401:ENSG00000118432:ENSG00000120278:ENSG00000091831:ENSG00000186340:ENSG00000122644:ENSG00000152926:ENSG00000213462:ENSG00000146757:ENSG00000165215:ENSG00000189143:ENSG00000135218:ENSG00000085563:ENSG00000127920:ENSG00000005249:ENSG00000106484:ENSG00000159763:ENSG00000196329:ENSG00000164949:ENSG00000120526:ENSG00000160339
DEG.up	Cells_Cultured_fibroblasts	4343	259	3.61137551757351e-07	1.95014277948969e-05	ENSG00000116237:ENSG00000097021:ENSG00000120942:ENSG00000134668:ENSG00000121904:ENSG00000117395:ENSG00000117399:ENSG00000123473:ENSG00000117862:ENSG00000198841:ENSG00000134717:ENSG00000162604:ENSG00000162616:ENSG00000142867:ENSG00000153904:ENSG00000171488:ENSG00000162694:ENSG00000117543:ENSG00000162775:ENSG00000168679:ENSG00000116455:ENSG00000213281:ENSG00000052723:ENSG00000092621:ENSG00000143457:ENSG00000143387:ENSG00000160767:ENSG00000198715:ENSG00000187800:ENSG00000162746:ENSG00000152332:ENSG00000143322:ENSG00000057252:ENSG00000066279:ENSG00000177888:ENSG00000162885:ENSG00000116962:ENSG00000065328:ENSG00000148468:ENSG00000108094:ENSG00000165476:ENSG00000138134:ENSG00000026103:ENSG00000059573:ENSG00000119927:ENSG00000165832:ENSG00000166483:ENSG00000198730:ENSG00000188211:ENSG00000168061:ENSG00000146670:ENSG00000173621:ENSG00000137492:ENSG00000149313:ENSG00000076053:ENSG00000154127:ENSG00000166250:ENSG00000082805:ENSG00000126749:ENSG00000134531:ENSG00000123094:ENSG00000135457:ENSG00000123364:ENSG00000123388:ENSG00000127314:ENSG00000166130:ENSG00000120868:ENSG00000111647:ENSG00000235162:ENSG00000174989:ENSG00000122966:ENSG00000150990:ENSG00000176915:ENSG00000139505:ENSG00000133110:ENSG00000102743:ENSG00000120688:ENSG00000102531:ENSG00000123179:ENSG00000102753:ENSG00000118939:ENSG00000136153:ENSG

						00000088451:ENSG00000152749:ENSG00000134901:ENSG00000134897:ENSG00000134899:ENSG00000100889:ENSG00000092140:ENSG00000092108:ENSG00000129493:ENSG00000129480:ENSG00000187790:ENSG00000139926:ENSG00000197930:ENSG00000126787:ENSG00000119599:ENSG00000133961:ENSG00000165417:ENSG00000165934:ENSG00000170270:ENSG00000140157:ENSG00000187951:ENSG00000021776:ENSG00000198146:ENSG00000197535:ENSG00000181827:ENSG00000140262:ENSG00000136378:ENSG00000103888:ENSG00000117899:ENSG00000103512:ENSG00000140682:ENSG00000103064:ENSG00000103061:ENSG00000225614:ENSG00000167515:ENSG00000167522:ENSG00000127804:ENSG00000005100:ENSG00000072849:ENSG00000012048:ENSG00000182963:ENSG00000011260:ENSG00000121060:ENSG00000198265:ENSG00000197170:ENSG00000224877:ENSG00000017797:ENSG00000154845:ENSG00000152223:ENSG00000071564:ENSG00000167670:ENSG00000132000:ENSG00000123146:ENSG00000128626:ENSG00000167625:ENSG00000105429:ENSG00000105464:ENSG00000105447:ENSG00000198464:ENSG00000115761:ENSG00000143867:ENSG00000171103:ENSG00000162928:ENSG00000082898:ENSG00000144048:ENSG00000163170:ENSG00000065911:ENSG00000005448:ENSG00000153107:ENSG00000153214:ENSG00000115963:ENSG00000153250:ENSG00000197713:ENSG00000168955:ENSG00000123977:ENSG00000198612:ENSG00000162804:ENSG00000125779:ENSG00000089048:ENSG00000124201:ENSG00000155313:ENSG00000179750:ENSG00000198951:ENSG00000177096:ENSG00000075218:ENSG00000130489:ENSG00000070950:ENSG00000134077:ENSG00000177873:ENSG00000114395:ENSG00000163946:ENSG00000144736:ENSG00000163320:ENSG00000175105:ENSG00000179021:ENSG00000184500:ENSG00000169379:ENSG00000178700:ENSG00000163607:ENSG00000163608:ENSG00000082701:ENSG00000240682:ENSG00000017260:ENSG00000129048:ENSG00000144867:ENSG00000158163:ENSG00000138231:ENSG00000175110:ENSG00000174899:ENSG00000075420:ENSG00000145014:ENSG00000169019:ENSG00000145246:ENSG00000169429:ENSG00000124875:ENSG00000109272:ENSG00000163739:ENSG00000173085:ENSG00000145331:ENSG00000113360:ENSG00000082213:ENSG00000132357:ENSG00000067248:ENSG00000039123:ENSG00000068796:ENSG00000086189:ENSG00000086200:ENSG00000164338:ENSG00000153140:ENSG00000181751:ENSG00000168916:ENSG00000253159:ENSG00000240184:ENSG00000113272:ENSG00000048140:ENSG00000131446:ENSG00000124787:ENSG00000197935:ENSG00000146112:ENSG00000137404:ENSG00000204220:ENSG00000137168:ENSG00000118432:ENSG00000132429:ENSG00000198945:ENSG00000272047:ENSG00000186340:ENSG00000130023:ENSG00000253293:ENSG00000005073:ENSG00000168303:ENSG00000126522:ENSG00000169902:ENSG00000049541:ENSG00000127920:ENSG00000105829:ENSG00000196937:ENSG00000106477:ENSG00000158623:ENSG00000131558:ENSG00000182158:ENSG00000146918:ENSG00000154319:ENSG00000035681:ENSG00000120526:ENSG00000080608:ENSG00000044459:ENSG00000178031:ENSG00000213694:ENSG00000243444:ENSG00000182752:ENSG00000119446:ENSG00000196152:ENSG00000136840:ENSG00000171169:ENSG00000148335:ENSG00000198113
DEG. up	Cells_EBV-transformed_lymphocytes	5125	269	0.00178400192168413	0.096336103770943	ENSG00000097021:ENSG00000160050:ENSG00000177181:ENSG00000117395:ENSG00000243710:ENSG00000117399:ENSG00000123473:ENSG00000198841:ENSG00000116205:ENSG00000117133:ENSG00000142867:ENSG00000117543:ENSG00000162775:ENSG00000116455:ENSG00000213281:ENSG00000052723:ENSG00000143457:ENSG00000163131:ENSG00000143379:ENSG00000163154:ENSG00000163156:ENSG00000143622:ENSG00000196189:ENSG00000253831:ENSG00000171786:ENSG00000132185:ENSG00000162746:ENSG00000152332:ENSG00000186283:ENSG00000057252:ENSG00000066279:ENSG00000107929:ENSG00000198879:ENSG00000065328:ENSG00000165997:ENSG00000108094:ENSG00000138134:ENSG00000026103:ENSG00000059573:ENSG00000165832:ENSG00000166483:ENSG00000198730:ENSG00000168061:ENSG00000146670:ENSG00000175567:ENSG00000149313:ENSG00000110777:ENSG00000139180:ENSG00000111679:ENSG00000126749:ENSG00000135457:ENSG00000123338:ENSG00000127314:ENSG00000205057:ENSG00000166130:ENSG00000120868:ENSG00000235162:ENSG00000122966:ENSG00000150990:ENSG00000184992:ENSG00000177084:ENSG00000176915:ENSG00000102743:ENSG00000136167:ENSG00000102445:ENSG00000102531:ENSG00000152213:ENSG00000102753:ENSG00000136104:ENSG00000118939:ENSG00000088451:ENSG00000152749:ENSG00000134899:ENSG00000102524:ENSG00000100889:ENSG00000092140:ENSG00000092108:ENSG00000100473:ENSG00000129480:ENSG00000165355:ENSG00000179454:ENSG00000187790:ENSG00000197930:ENSG00000126787:ENSG00000119599:ENSG00000165417:ENSG00000165934:ENSG00000170270:ENSG00000012963:ENSG00000140157:ENSG00000170113:ENSG00000187951:ENSG00000021776:ENSG00000198146:ENSG00000174197:ENSG00000197535:ENSG00000181827:ENSG00000103707:ENSG00000140350:ENSG00000140471:ENSG00000007038:ENSG00000085721:ENSG00000157106:ENSG00000102879:ENSG00000169627:ENSG00000180096:ENSG00000179958:ENSG00000197302:ENSG00000102970:ENSG00000072736:ENSG00000103064:ENSG00000103061:ENSG00000185669:ENSG00000127804:ENSG00000029725:ENSG00000005100:ENSG00000141127:ENSG00000161405:ENSG00000186075:ENSG00000068079:ENSG00000012048:ENSG00000184988:ENSG00000073861:ENSG00000011260:ENSG00000153933:ENSG00000121060:ENSG00000197170:ENSG00000180616:ENSG00000141576:ENSG00000224877:ENSG00000186765:ENSG00000168234:ENSG00000141384:ENSG00000130856:ENSG00000071564:ENSG00000167670:ENSG00000205744:ENSG00000263264:ENSG00000104880:ENSG00000132017:ENSG00000123146:ENSG00000096996:ENSG00000099308:ENSG00000237440:ENSG00000104835:ENSG00000128626:ENSG00000105369:ENSG00000028277:ENSG00000167625:ENSG00000079462:ENSG00000105483:ENSG00000105447:ENSG00000196214:ENSG00000198464:ENSG00000213020:ENSG00000196417:ENSG00000115761:ENSG00000171103:ENSG00000119866:ENSG00000082898:ENSG00000144034:ENSG00000144048:ENSG00000163170:ENSG00000065911:ENSG00000005448:ENSG00000198885:ENSG00000115604:ENSG00000153107:ENSG00000153214:ENSG00000163040:ENSG00000118260:ENSG00000144401:ENSG00000197713:ENSG00000135912:ENSG00000125779:ENSG00000089048:ENSG00000124201:ENSG00000124256:ENSG00000203896:ENSG00000154642:ENSG00000160190:ENSG00000100342:ENSG00000179750:ENSG00000198951:ENSG00000075218:ENSG00000100416:ENSG00000205593:ENSG00000130489:ENSG00000177989:ENSG

						00000130487:ENSG00000205560:ENSG00000070950:ENSG00000134077:ENSG00000177873:ENSG00000114395:ENSG00000163946:ENSG00000144736:ENSG00000163320:ENSG00000175105:ENSG00000179021:ENSG00000163606:ENSG00000163607:ENSG00000163608:ENSG00000144843:ENSG00000082701:ENSG00000240682:ENSG00000017260:ENSG00000081307:ENSG00000144867:ENSG00000138231:ENSG00000175110:ENSG00000114127:ENSG00000172578:ENSG00000163898:ENSG00000041802:ENSG00000163975:ENSG00000169019:ENSG00000145287:ENSG00000173085:ENSG00000151247:ENSG00000145331:ENSG00000113360:ENSG00000082213:ENSG00000164190:ENSG00000039123:ENSG00000068796:ENSG00000086189:ENSG00000086200:ENSG00000164338:ENSG00000164180:ENSG00000153140:ENSG00000175471:ENSG00000181751:ENSG00000164241:ENSG00000164620:ENSG00000113272:ENSG00000146112:ENSG00000137404:ENSG00000137337:ENSG00000204220:ENSG00000124772:ENSG00000137168:ENSG00000118503:ENSG00000026297:ENSG00000213066:ENSG00000168303:ENSG00000146757:ENSG00000086730:ENSG00000049541:ENSG00000105829:ENSG00000160813:ENSG00000105865:ENSG00000158623:ENSG00000182158:ENSG00000214106:ENSG00000146918:ENSG00000136573:ENSG00000120910:ENSG00000129691:ENSG00000254087:ENSG00000035681:ENSG00000120526:ENSG00000120533:ENSG00000080608:ENSG00000167081:ENSG00000196152:ENSG00000136840:ENSG00000171169:ENSG00000198113
DEG. up	Cervix_Ectocervix	195	15	0.0286806418793992	1	ENSG00000143387:ENSG00000160753:ENSG00000132680:ENSG00000122176:ENSG00000123179:ENSG00000139926:ENSG00000243708:ENSG00000072134:ENSG00000166484:ENSG00000091831:ENSG00000186340:ENSG00000105855:ENSG00000005073:ENSG00000106031:ENSG00000148339
DEG. up	Cervix_Endocervix	1849	118	5.40295748482942e-05	0.00291759704180789	ENSG00000162458:ENSG00000117569:ENSG00000162694:ENSG00000116396:ENSG00000143457:ENSG00000143387:ENSG00000143437:ENSG00000143379:ENSG00000143363:ENSG00000163155:ENSG00000160753:ENSG00000116580:ENSG00000132718:ENSG00000132680:ENSG00000187800:ENSG00000186283:ENSG00000122176:ENSG00000162714:ENSG00000148468:ENSG00000165338:ENSG00000168060:ENSG00000162300:ENSG00000137492:ENSG00000076053:ENSG00000166250:ENSG00000134532:ENSG00000123094:ENSG00000166130:ENSG00000074590:ENSG00000174989:ENSG00000188735:ENSG00000102531:ENSG00000123179:ENSG00000178695:ENSG00000088451:ENSG00000134901:ENSG00000134897:ENSG00000092108:ENSG00000165379:ENSG00000185246:ENSG00000100767:ENSG00000133961:ENSG00000174197:ENSG00000243708:ENSG00000140262:ENSG00000103707:ENSG00000117899:ENSG00000140682:ENSG00000132600:ENSG00000127804:ENSG00000072134:ENSG00000166484:ENSG00000142494:ENSG00000068079:ENSG00000184988:ENSG00000153933:ENSG00000185504:ENSG00000130856:ENSG00000071564:ENSG00000132017:ENSG00000132005:ENSG00000123146:ENSG00000269190:ENSG00000079462:ENSG00000196214:ENSG00000167766:ENSG00000213020:ENSG00000196208:ENSG00000084676:ENSG00000075292:ENSG00000144283:ENSG00000153250:ENSG00000118260:ENSG00000162804:ENSG00000088876:ENSG00000100321:ENSG00000100324:ENSG00000177096:ENSG00000138944:ENSG00000205643:ENSG00000075234:ENSG00000182983:ENSG00000180376:ENSG00000163946:ENSG00000144749:ENSG00000163320:ENSG00000114446:ENSG00000163608:ENSG00000113971:ENSG00000158163:ENSG00000013288:ENSG00000039123:ENSG00000164180:ENSG00000120318:ENSG00000196670:ENSG00000137404:ENSG00000137337:ENSG00000198945:ENSG00000091831:ENSG00000186340:ENSG00000253293:ENSG00000005073:ENSG00000106031:ENSG00000153814:ENSG00000169902:ENSG00000127920:ENSG00000160813:ENSG00000075790:ENSG00000182158:ENSG00000146966:ENSG00000035681:ENSG00000147601:ENSG00000164949:ENSG00000196378:ENSG00000044459:ENSG00000136925:ENSG00000182752:ENSG00000148339
DEG. up	Colon_Sigmoid	1644	75	0.433852098339653	1	ENSG00000171819:ENSG00000162458:ENSG00000187942:ENSG00000127603:ENSG00000117155:ENSG00000117569:ENSG00000160767:ENSG00000242252:ENSG00000122176:ENSG00000163050:ENSG00000116962:ENSG00000165476:ENSG00000075073:ENSG00000120057:ENSG00000203867:ENSG00000149150:ENSG00000110025:ENSG00000168060:ENSG00000166250:ENSG00000064309:ENSG00000123094:ENSG00000123338:ENSG00000123360:ENSG00000136104:ENSG00000179476:ENSG00000258987:ENSG00000128731:ENSG00000103710:ENSG00000117971:ENSG00000183426:ENSG00000180035:ENSG00000140682:ENSG00000132514:ENSG00000065325:ENSG00000233670:ENSG00000128482:ENSG00000068079:ENSG00000182963:ENSG00000263264:ENSG00000104880:ENSG00000143867:ENSG00000171094:ENSG00000119866:ENSG00000231672:ENSG00000127831:ENSG00000144485:ENSG00000100342:ENSG00000177096:ENSG00000138944:ENSG00000205643:ENSG00000144749:ENSG00000114861:ENSG00000175066:ENSG00000109171:ENSG00000171522:ENSG00000113722:ENSG00000135074:ENSG00000113272:ENSG00000155858:ENSG00000244731:ENSG00000118432:ENSG00000253293:ENSG00000005073:ENSG00000106031:ENSG00000153814:ENSG00000182158:ENSG00000121039:ENSG00000079112:ENSG00000164949:ENSG00000165071:ENSG00000044459:ENSG00000136936:ENSG00000167081:ENSG00000148356:ENSG00000148411
DEG. up	Colon_Transverse	1612	80	0.170976886790845	1	ENSG00000162460:ENSG00000162458:ENSG00000179178:ENSG00000253313:ENSG00000117399:ENSG00000117472:ENSG00000162366:ENSG00000186564:ENSG00000162390:ENSG00000162391:ENSG00000203943:ENSG00000142867:ENSG00000134215:ENSG00000134240:ENSG00000163131:ENSG00000075073:ENSG00000059573:ENSG00000120057:ENSG00000187908:ENSG00000135373:ENSG00000168060:ENSG00000166391:ENSG00000110777:ENSG00000166250:ENSG00000013588:ENSG00000111725:ENSG00000184992:ENSG00000102445:ENSG00000100889:ENSG00000186198:ENSG00000103067:ENSG00000103056:ENSG00000132514:ENSG00000065325:ENSG00000233670:ENSG00000181350:ENSG00000182963:ENSG00000263264:ENSG00000104880:ENSG00000129354:ENSG00000123146:ENSG00000105369:ENSG00000079385:ENSG00000142273:ENSG00000127831:ENSG00000168955:ENSG00000144485:ENSG00000124102:ENSG00000160190:ENSG00000179750:ENSG00000138944:ENSG00000075234:ENSG00000178750:ENSG00000144852:ENSG00000114248:ENSG00000163898:ENSG00000145014:ENSG00000

						080493:ENSG00000163739:ENSG00000145287:ENSG00000174358:ENSG00000171522:ENSG00000181751:ENSG00000064651:ENSG00000186314:ENSG00000113722:ENSG00000135074:ENSG00000137266:ENSG00000112812:ENSG00000253293:ENSG00000005073:ENSG00000106031:ENSG00000126522:ENSG00000165215:ENSG00000189143:ENSG00000085563:ENSG00000104267:ENSG00000079112:ENSG00000130958:ENSG00000198113
DEG.up	Esophagus_Gastroesophageal_Junction	1291	58	0.494654215724465	1	ENSG00000171819:ENSG00000162458:ENSG00000187942:ENSG00000162390:ENSG00000117114:ENSG00000117569:ENSG00000116962:ENSG00000075073:ENSG00000107736:ENSG00000165338:ENSG00000120057:ENSG00000198873:ENSG00000149150:ENSG00000168060:ENSG00000137672:ENSG00000166250:ENSG00000087494:ENSG00000187848:ENSG00000184226:ENSG00000178695:ENSG00000168952:ENSG00000179476:ENSG00000100767:ENSG00000258987:ENSG00000103710:ENSG00000251209:ENSG00000183426:ENSG00000140682:ENSG00000065325:ENSG00000182963:ENSG00000141401:ENSG00000105492:ENSG00000115705:ENSG00000143867:ENSG00000144218:ENSG00000153208:ENSG00000124107:ENSG00000177096:ENSG00000138944:ENSG00000144749:ENSG00000114861:ENSG00000016082:ENSG00000064651:ENSG00000135074:ENSG00000155858:ENSG00000255587:ENSG00000164530:ENSG00000153814:ENSG00000241644:ENSG00000005249:ENSG00000146966:ENSG00000121039:ENSG00000164949:ENSG00000178031:ENSG00000186354:ENSG00000213694:ENSG00000167081:ENSG00000148411
DEG.up	Esophagus_Mucosa	2244	109	0.183135190595557	1	ENSG00000158286:ENSG00000069812:ENSG00000253313:ENSG00000117399:ENSG00000162366:ENSG00000123473:ENSG00000162390:ENSG00000134709:ENSG00000142867:ENSG00000134215:ENSG00000116455:ENSG00000175984:ENSG00000213281:ENSG00000143457:ENSG00000143452:ENSG00000143412:ENSG00000143409:ENSG00000143631:ENSG00000203786:ENSG00000143622:ENSG00000196189:ENSG00000163472:ENSG00000066279:ENSG00000065328:ENSG00000075073:ENSG00000203867:ENSG00000129151:ENSG00000135373:ENSG00000165905:ENSG00000146670:ENSG00000149809:ENSG00000173653:ENSG00000134531:ENSG00000087494:ENSG00000184992:ENSG00000187848:ENSG00000123179:ENSG00000118939:ENSG00000136153:ENSG00000150527:ENSG00000139926:ENSG00000197930:ENSG00000126787:ENSG00000006432:ENSG00000187951:ENSG00000198146:ENSG00000137802:ENSG00000243708:ENSG00000243789:ENSG00000103888:ENSG00000102921:ENSG00000102970:ENSG00000103067:ENSG00000188522:ENSG00000166484:ENSG00000225190:ENSG00000108423:ENSG00000154845:ENSG00000141401:ENSG00000206073:ENSG00000167670:ENSG00000180739:ENSG00000129354:ENSG00000079462:ENSG00000105427:ENSG00000189377:ENSG00000079385:ENSG00000142273:ENSG00000119866:ENSG00000144048:ENSG00000180251:ENSG00000115963:ENSG00000175121:ENSG00000168703:ENSG00000124102:ENSG00000124107:ENSG00000160190:ENSG00000075218:ENSG00000075275:ENSG00000130489:ENSG00000197385:ENSG00000168032:ENSG00000178750:ENSG00000163606:ENSG00000144867:ENSG00000163898:ENSG00000169019:ENSG00000163739:ENSG00000109321:ENSG00000145287:ENSG00000132357:ENSG00000197822:ENSG00000137266:ENSG00000124787:ENSG00000112812:ENSG00000204544:ENSG00000235863:ENSG00000135525:ENSG00000122644:ENSG00000213462:ENSG00000146757:ENSG00000189143:ENSG00000080608:ENSG00000230453:ENSG00000127080:ENSG00000070159:ENSG00000167103:ENSG00000233198:ENSG00000198569
DEG.up	Esophagus_Muscularis	1316	66	0.174387329977592	1	ENSG00000171819:ENSG00000162458:ENSG00000187942:ENSG00000127603:ENSG00000162390:ENSG00000117114:ENSG00000117569:ENSG00000162694:ENSG00000160767:ENSG00000143801:ENSG00000163050:ENSG00000116962:ENSG00000198879:ENSG00000075073:ENSG00000107736:ENSG00000165338:ENSG00000120057:ENSG00000198873:ENSG00000168060:ENSG00000137672:ENSG00000166250:ENSG00000082805:ENSG00000187848:ENSG00000136104:ENSG00000184226:ENSG00000178695:ENSG00000168952:ENSG00000179476:ENSG00000258987:ENSG00000103710:ENSG00000251209:ENSG00000008710:ENSG00000140682:ENSG00000065325:ENSG00000182963:ENSG00000141401:ENSG00000105492:ENSG00000115705:ENSG00000143867:ENSG00000144218:ENSG00000144283:ENSG00000153250:ENSG00000101290:ENSG00000125864:ENSG00000177096:ENSG00000138944:ENSG00000144749:ENSG00000114861:ENSG00000114098:ENSG00000109171:ENSG00000064651:ENSG00000135074:ENSG00000155858:ENSG00000244731:ENSG00000164530:ENSG00000153814:ENSG00000241644:ENSG00000005249:ENSG00000146966:ENSG00000121039:ENSG00000164949:ENSG00000178031:ENSG00000186354:ENSG00000213694:ENSG00000167081:ENSG00000148411
DEG.up	Fallopian_Tube	490	27	0.151951761615191	1	ENSG00000162458:ENSG00000143387:ENSG00000143437:ENSG00000122176:ENSG00000133110:ENSG00000102531:ENSG00000136104:ENSG00000178695:ENSG00000133961:ENSG00000103710:ENSG00000008710:ENSG00000140682:ENSG00000123146:ENSG00000167766:ENSG00000196208:ENSG00000162804:ENSG00000124107:ENSG00000100342:ENSG00000251322:ENSG00000169379:ENSG00000114115:ENSG00000205403:ENSG00000171522:ENSG00000086189:ENSG00000091831:ENSG00000241644:ENSG00000127920
DEG.up	Heart_Atrial_Appendage	745	33	0.54027201964232	1	ENSG00000158286:ENSG00000122176:ENSG00000120057:ENSG00000203867:ENSG00000111254:ENSG00000127863:ENSG00000136153:ENSG00000139914:ENSG00000103710:ENSG00000136378:ENSG00000140986:ENSG00000073670:ENSG00000163170:ENSG00000128285:ENSG00000205560:ENSG00000144712:ENSG00000183873:ENSG00000179564:ENSG00000007402:ENSG00000184500:ENSG00000158186:ENSG00000114279:ENSG00000248713:ENSG00000135074:ENSG00000124772:ENSG00000137168:ENSG00000164530:ENSG00000124743:ENSG00000132429:ENSG00000241644:ENSG00000135218:ENSG00000172209:ENSG00000165071

DEG.up	Heart_Left_Ventricle	536	23	0.604439948227586	1	ENSG00000158286:ENSG00000162390:ENSG00000134240:ENSG00000095587:ENSG00000203867:ENSG00000139180:ENSG00000136153:ENSG00000139914:ENSG00000140986:ENSG00000123700:ENSG00000205560:ENSG00000144712:ENSG00000183873:ENSG00000179564:ENSG00000114378:ENSG00000114279:ENSG00000248713:ENSG00000124772:ENSG00000124743:ENSG00000132429:ENSG00000135218:ENSG00000172209:ENSG00000165071
DEG.up	Kidney_Cortex	1343	80	0.00528951112360471	0.285633600674654	ENSG00000158286:ENSG00000162460:ENSG00000158816:ENSG00000253313:ENSG00000117472:ENSG00000162366:ENSG00000162391:ENSG00000153904:ENSG00000134215:ENSG00000168679:ENSG00000134240:ENSG00000143412:ENSG00000143627:ENSG00000162746:ENSG00000134463:ENSG00000152779:ENSG00000148702:ENSG00000129151:ENSG00000165905:ENSG00000162174:ENSG00000235162:ENSG00000110887:ENSG00000139410:ENSG00000111725:ENSG00000100889:ENSG00000100767:ENSG00000186198:ENSG00000103066:ENSG00000154025:ENSG00000142494:ENSG00000180638:ENSG00000184988:ENSG00000153822:ENSG00000141401:ENSG00000129354:ENSG00000160951:ENSG00000250799:ENSG00000161270:ENSG00000269190:ENSG00000079385:ENSG00000116039:ENSG00000114993:ENSG00000115602:ENSG00000180251:ENSG00000127831:ENSG00000125966:ENSG00000203896:ENSG00000075234:ENSG00000075275:ENSG00000100253:ENSG00000130487:ENSG00000180432:ENSG00000114378:ENSG00000163762:ENSG00000205835:ENSG00000152953:ENSG00000157765:ENSG00000080493:ENSG00000079557:ENSG00000163739:ENSG00000205403:ENSG00000164128:ENSG00000174358:ENSG00000164363:ENSG00000113361:ENSG00000244731:ENSG00000244067:ENSG00000243955:ENSG00000269964:ENSG00000105855:ENSG00000126522:ENSG00000165215:ENSG00000189143:ENSG00000104267:ENSG00000104327:ENSG00000070159:ENSG00000160339:ENSG00000233198:ENSG00000198569:ENSG00000197768
DEG.up	Kidney_Medulla	95	4	0.616431068353487	1	ENSG00000117472:ENSG00000153904:ENSG00000111725:ENSG00000104267
DEG.up	Liver	1536	81	0.0660629675182697	1	ENSG00000162460:ENSG00000168389:ENSG00000162391:ENSG00000134709:ENSG00000153904:ENSG00000117528:ENSG00000134240:ENSG00000143412:ENSG00000143630:ENSG00000143627:ENSG00000132703:ENSG00000198734:ENSG00000120341:ENSG00000134389:ENSG00000143278:ENSG00000134463:ENSG00000241058:ENSG00000119927:ENSG00000148702:ENSG00000110169:ENSG00000129151:ENSG00000149150:ENSG00000149809:ENSG00000173599:ENSG00000166391:ENSG00000062282:ENSG00000137714:ENSG00000110887:ENSG00000135094:ENSG00000139410:ENSG00000102743:ENSG00000123179:ENSG00000088451:ENSG00000100889:ENSG00000139914:ENSG00000150527:ENSG00000121270:ENSG00000103067:ENSG00000196689:ENSG00000197417:ENSG00000161944:ENSG00000142494:ENSG00000168234:ENSG00000104883:ENSG00000250799:ENSG00000269190:ENSG00000079385:ENSG00000142273:ENSG00000114993:ENSG00000183281:ENSG00000127831:ENSG00000124107:ENSG00000203896:ENSG00000100342:ENSG00000100197:ENSG00000186951:ENSG00000075234:ENSG00000170293:ENSG00000144648:ENSG00000180432:ENSG00000114378:ENSG00000184500:ENSG00000144852:ENSG00000172780:ENSG00000240303:ENSG00000205835:ENSG00000079557:ENSG00000138823:ENSG00000205403:ENSG00000145826:ENSG00000244731:ENSG00000244067:ENSG00000243955:ENSG00000146416:ENSG00000126522:ENSG00000165215:ENSG00000085563:ENSG00000104267:ENSG00000070159:ENSG00000148339:ENSG00000160339
DEG.up	Lung	4262	225	0.00340286140340546	0.183754515783895	ENSG00000158292:ENSG00000162458:ENSG00000116809:ENSG00000134668:ENSG00000127603:ENSG00000168389:ENSG00000243710:ENSG00000179178:ENSG00000253313:ENSG00000066056:ENSG00000117472:ENSG00000162366:ENSG00000162367:ENSG00000117114:ENSG00000137976:ENSG00000171488:ENSG00000162775:ENSG00000168679:ENSG00000213281:ENSG00000163131:ENSG00000163154:ENSG00000143622:ENSG00000116584:ENSG00000196189:ENSG00000187800:ENSG00000160838:ENSG00000143226:ENSG00000173110:ENSG00000203747:ENSG00000152332:ENSG00000174175:ENSG00000057252:ENSG00000122176:ENSG00000116962:ENSG00000107929:ENSG00000198879:ENSG00000165997:ENSG00000107736:ENSG00000107738:ENSG00000026103:ENSG00000152779:ENSG00000198873:ENSG00000187908:ENSG00000006071:ENSG00000162174:ENSG00000187726:ENSG00000175567:ENSG00000137672:ENSG00000183644:ENSG00000154127:ENSG00000111671:ENSG00000010626:ENSG00000111679:ENSG00000111684:ENSG00000013588:ENSG00000134531:ENSG00000110911:ENSG00000123338:ENSG00000127314:ENSG00000166130:ENSG00000120868:ENSG00000135094:ENSG00000111725:ENSG00000187848:ENSG00000127863:ENSG00000136167:ENSG00000102445:ENSG00000102531:ENSG00000136153:ENSG00000178695:ENSG00000139826:ENSG00000102524:ENSG00000168952:ENSG00000133961:ENSG00000186198:ENSG00000103710:ENSG00000136378:ENSG00000103888:ENSG00000140406:ENSG00000156206:ENSG00000140511:ENSG00000007038:ENSG00000102879:ENSG00000180096:ENSG00000140682:ENSG00000102921:ENSG00000102970:ENSG00000103067:ENSG00000103066:ENSG00000185669:ENSG00000158717:ENSG00000132514:ENSG00000007174:ENSG00000161405:ENSG00000068079:ENSG00000184988:ENSG00000182963:ENSG00000073861:ENSG00000214226:ENSG00000153933:ENSG00000121060:ENSG00000198265:ENSG00000171595:ENSG00000154889:ENSG00000152223:ENSG00000205744:ENSG00000104880:ENSG00000065989:ENSG00000129354:ENSG00000123146:ENSG00000160951:ENSG00000096996:ENSG00000189377:ENSG00000105483:ENSG00000105383:ENSG00000105492:ENSG00000171049:ENSG00000115756:ENSG00000197872:ENSG00000143867:ENSG00000162929:ENSG00000065911:ENSG00000144218:ENSG00000115602:ENSG00000115604:ENSG00000115607:ENSG00000153214:ENSG00000115762:ENSG00000153250:ENSG00000018280:ENSG00000123977:ENSG00000196209:ENSG00000188559:ENSG00000125966:ENSG00000124102:ENSG00000124107:ENSG00000124201:ENSG00000124256:ENSG00000128335:ENSG00000100342:ENSG00000198951:ENSG00000177096:ENSG00000075275:ENSG00000130489:ENSG00000177989:ENSG00000251322:ENSG00000163576:ENSG00000170293:ENSG00000114378:ENSG00000068001:ENSG00000004838:ENSG00000114395:ENSG00000007402:ENSG00000114861:ENSG00000184500:ENSG00000169379:ENSG0

						0000178750:ENSG00000114446:ENSG00000144843:ENSG00000169704:ENSG00000172780:ENSG00000158163:ENSG00000114113:ENSG00000155903:ENSG00000114698:ENSG00000174899:ENSG00000075420:ENSG00000172578:ENSG00000163898:ENSG00000145014:ENSG00000157765:ENSG00000109689:ENSG00000169429:ENSG00000163739:ENSG00000109321:ENSG00000145287:ENSG00000185477:ENSG00000205403:ENSG00000113361:ENSG00000171522:ENSG00000132357:ENSG00000197822:ENSG00000168916:ENSG00000253159:ENSG00000240184:ENSG00000120318:ENSG00000135074:ENSG00000078401:ENSG00000079691:ENSG00000112812:ENSG00000146112:ENSG00000137404:ENSG00000255587:ENSG00000244067:ENSG00000243955:ENSG00000118503:ENSG00000112406:ENSG00000120278:ENSG00000131018:ENSG00000026297:ENSG00000241644:ENSG00000152926:ENSG00000126522:ENSG00000165215:ENSG00000189143:ENSG00000086730:ENSG00000135218:ENSG00000127920:ENSG00000085514:ENSG00000106484:ENSG00000146966:ENSG00000181444:ENSG00000196329:ENSG00000154319:ENSG00000254087:ENSG00000121039:ENSG00000104267:ENSG00000164949:ENSG00000165071:ENSG00000080608:ENSG00000130958:ENSG00000243444:ENSG00000136840:ENSG00000148339:ENSG00000198113
DEG.up	Minor_Salivary_Gland	1899	90	0.285004595160199	1	ENSG00000158286:ENSG00000183888:ENSG00000187942:ENSG00000188672:ENSG00000157978:ENSG00000179178:ENSG00000253313:ENSG00000117472:ENSG00000162366:ENSG00000134709:ENSG00000137976:ENSG00000116748:ENSG00000092621:ENSG00000134240:ENSG00000163157:ENSG00000196189:ENSG00000163472:ENSG00000198734:ENSG00000059573:ENSG00000187908:ENSG00000135373:ENSG00000165905:ENSG00000124939:ENSG00000124935:ENSG00000149809:ENSG00000175567:ENSG00000110777:ENSG00000013588:ENSG00000087494:ENSG00000110911:ENSG00000205057:ENSG00000127863:ENSG00000150527:ENSG00000197930:ENSG00000243789:ENSG00000007038:ENSG00000180209:ENSG00000102970:ENSG00000103067:ENSG00000141750:ENSG00000206073:ENSG00000181143:ENSG00000129354:ENSG00000105369:ENSG00000105427:ENSG00000189377:ENSG00000079385:ENSG00000142273:ENSG00000134323:ENSG00000143867:ENSG00000116039:ENSG00000153214:ENSG00000144283:ENSG00000132326:ENSG00000124102:ENSG00000124107:ENSG00000160190:ENSG00000075275:ENSG00000177989:ENSG00000197385:ENSG00000170293:ENSG00000168032:ENSG00000182606:ENSG00000114395:ENSG00000178750:ENSG00000175066:ENSG00000114248:ENSG00000163898:ENSG00000163975:ENSG00000157765:ENSG00000169429:ENSG00000124875:ENSG00000163739:ENSG00000164128:ENSG00000064651:ENSG00000137266:ENSG00000112812:ENSG00000204544:ENSG00000243955:ENSG00000135525:ENSG00000026297:ENSG00000164488:ENSG00000105855:ENSG00000165215:ENSG00000189143:ENSG00000106477:ENSG00000159763:ENSG00000254087:ENSG00000121039:ENSG00000104267
DEG.up	Muscle_Skeletal	1143	51	0.517763284582766	1	ENSG00000160050:ENSG00000175984:ENSG00000116748:ENSG00000143434:ENSG00000163157:ENSG00000163050:ENSG00000134463:ENSG00000203867:ENSG00000187486:ENSG00000175567:ENSG00000175564:ENSG00000139180:ENSG00000139292:ENSG00000102753:ENSG00000136153:ENSG00000139914:ENSG00000258987:ENSG00000234438:ENSG00000185418:ENSG00000140986:ENSG00000157106:ENSG00000180209:ENSG00000072736:ENSG00000103066:ENSG00000103064:ENSG00000185669:ENSG00000142494:ENSG00000067191:ENSG00000108878:ENSG00000197170:ENSG00000141576:ENSG00000182446:ENSG00000141401:ENSG00000127527:ENSG00000155313:ENSG00000183628:ENSG00000186951:ENSG00000205560:ENSG00000144712:ENSG00000179564:ENSG00000248713:ENSG00000215218:ENSG00000113742:ENSG00000124743:ENSG00000132429:ENSG00000253293:ENSG00000135218:ENSG00000120910:ENSG00000070159:ENSG00000148339:ENSG00000148335
DEG.up	Nerve_Tibial	5911	283	0.0919971321641335	1	ENSG00000158292:ENSG00000171819:ENSG00000120942:ENSG00000065526:ENSG00000187942:ENSG00000160094:ENSG00000168389:ENSG00000066056:ENSG00000198198:ENSG00000186790:ENSG00000186564:ENSG00000162604:ENSG00000162642:ENSG00000197147:ENSG00000171488:ENSG00000069712:ENSG00000117528:ENSG00000117569:ENSG00000168679:ENSG00000197323:ENSG00000143387:ENSG00000143437:ENSG00000143379:ENSG00000143412:ENSG00000143409:ENSG00000143363:ENSG00000143434:ENSG00000163155:ENSG00000163354:ENSG00000160753:ENSG00000116539:ENSG00000125459:ENSG00000116580:ENSG00000132718:ENSG00000132680:ENSG00000116584:ENSG00000160781:ENSG00000187800:ENSG00000143226:ENSG00000162746:ENSG00000174175:ENSG00000120341:ENSG00000186283:ENSG00000143322:ENSG00000177888:ENSG00000122176:ENSG00000116962:ENSG00000162714:ENSG00000198879:ENSG00000148468:ENSG00000241058:ENSG00000188234:ENSG00000107738:ENSG00000152779:ENSG00000165338:ENSG00000095587:ENSG00000155256:ENSG00000120057:ENSG00000198873:ENSG00000166313:ENSG00000173599:ENSG00000173621:ENSG00000137492:ENSG00000179240:ENSG00000166250:ENSG00000023171:ENSG00000082805:ENSG00000010626:ENSG00000134531:ENSG00000134532:ENSG00000123094:ENSG00000087494:ENSG00000135457:ENSG00000139292:ENSG00000074590:ENSG00000122966:ENSG00000188735:ENSG00000150990:ENSG00000127863:ENSG00000120688:ENSG00000102531:ENSG00000123179:ENSG00000184226:ENSG00000136153:ENSG00000178734:ENSG00000178695:ENSG00000088451:ENSG00000134899:ENSG00000168952:ENSG00000092140:ENSG00000092108:ENSG00000129493:ENSG00000203546:ENSG00000165379:ENSG00000179476:ENSG00000179454:ENSG00000198718:ENSG00000185246:ENSG00000139926:ENSG00000087301:ENSG00000165861:ENSG00000100767:ENSG00000133961:ENSG00000128731:ENSG00000261794:ENSG00000021776:ENSG00000137802:ENSG00000243708:ENSG00000197535:ENSG00000140262:ENSG00000103707:ENSG00000103710:ENSG00000136378:ENSG00000185418:ENSG00000183426:ENSG00000085721:ENSG00000140682:ENSG00000196470:ENSG00000072736:ENSG00000103064:ENSG00000103061:ENSG00000132600:ENSG00000127804:ENSG00000196689:ENSG00000197417:ENSG00000029725:ENSG00000005100:ENSG00000132514:ENSG00000141127:ENSG00000072134:ENSG00000166484:ENSG00000068079:ENSG00000184988:ENSG00000011258:ENSG00000214226:ENSG00000153933:ENSG00000198265:ENSG00000141540:ENSG00000196169:ENSG00000078687:ENSG00000141577:ENSG00000017797:ENSG00000141431:ENSG00000152223:ENSG00000264278:ENSG00000130856:ENSG00000197971:ENSG00000104833:EN

						SG00000171105:ENSG00000104883:ENSG00000198342:ENSG00000132016:ENSG00000132000:ENSG00000269190:ENSG00000105429:ENSG00000185453:ENSG00000167766:ENSG00000213020:ENSG00000196417:ENSG00000176293:ENSG00000196208:ENSG00000134323:ENSG00000084676:ENSG00000162929:ENSG00000082898:ENSG00000075292:ENSG00000217702:ENSG00000188687:ENSG00000005448:ENSG00000114993:ENSG00000153208:ENSG00000163040:ENSG00000153250:ENSG00000118260:ENSG00000018280:ENSG00000135912:ENSG00000186235:ENSG00000162804:ENSG00000088876:ENSG00000089048:ENSG00000124107:ENSG00000155313:ENSG00000157617:ENSG00000128335:ENSG00000100321:ENSG00000100324:ENSG00000198951:ENSG00000177096:ENSG00000205643:ENSG00000075234:ENSG00000100416:ENSG00000205593:ENSG00000251322:ENSG00000144560:ENSG00000163517:ENSG00000163576:ENSG00000144648:ENSG00000180432:ENSG00000182983:ENSG00000179564:ENSG00000243477:ENSG00000068001:ENSG00000180376:ENSG00000163946:ENSG00000175105:ENSG00000184500:ENSG00000169379:ENSG00000178700:ENSG00000163608:ENSG00000017260:ENSG00000034533:ENSG00000240303:ENSG00000113971:ENSG00000158163:ENSG00000158186:ENSG00000155903:ENSG00000175066:ENSG00000114127:ENSG00000114698:ENSG00000174899:ENSG00000163904:ENSG00000145014:ENSG00000041802:ENSG00000013288:ENSG00000109689:ENSG00000205403:ENSG00000113361:ENSG00000164190:ENSG00000039123:ENSG00000086189:ENSG00000086200:ENSG00000164331:ENSG00000164180:ENSG00000175471:ENSG00000181751:ENSG00000168916:ENSG00000064651:ENSG00000197948:ENSG00000196670:ENSG00000078401:ENSG00000197935:ENSG00000146112:ENSG00000137404:ENSG00000137337:ENSG00000244731:ENSG00000223501:ENSG00000164530:ENSG00000132429:ENSG00000198945:ENSG00000120278:ENSG00000026297:ENSG00000186340:ENSG00000184786:ENSG00000130023:ENSG00000105855:ENSG00000253293:ENSG00000005073:ENSG00000241644:ENSG00000152926:ENSG00000126522:ENSG00000085563:ENSG00000127920:ENSG00000085514:ENSG00000078487:ENSG00000185955:ENSG00000166924:ENSG00000106484:ENSG00000182158:ENSG00000146966:ENSG00000196329:ENSG00000120910:ENSG00000147601:ENSG00000121039:ENSG00000120526:ENSG00000131773:ENSG00000196378:ENSG00000044459:ENSG00000178031:ENSG00000130958:ENSG00000136925:ENSG00000136936:ENSG00000179058:ENSG00000148411
DEG. up	Ovary	5714	246	0.74143856916593	1	ENSG00000158292:ENSG00000120942:ENSG00000162458:ENSG00000065526:ENSG00000204178:ENSG00000160051:ENSG00000198198:ENSG00000117862:ENSG00000198841:ENSG00000134717:ENSG00000162390:ENSG00000162604:ENSG00000117114:ENSG00000162694:ENSG00000117543:ENSG00000168679:ENSG00000085465:ENSG00000197323:ENSG00000143457:ENSG00000143387:ENSG00000143437:ENSG00000143379:ENSG00000143434:ENSG00000163155:ENSG00000116539:ENSG00000125459:ENSG00000132680:ENSG00000242252:ENSG00000187800:ENSG00000122176:ENSG00000163050:ENSG00000116962:ENSG00000162714:ENSG00000198879:ENSG00000241058:ENSG00000165997:ENSG00000095794:ENSG00000188234:ENSG00000107736:ENSG00000138134:ENSG00000026103:ENSG00000165338:ENSG00000155256:ENSG00000165832:ENSG00000198873:ENSG00000268403:ENSG00000166478:ENSG00000198730:ENSG00000149150:ENSG00000168060:ENSG00000149809:ENSG00000173653:ENSG00000149256:ENSG00000149292:ENSG00000076053:ENSG00000023171:ENSG00000064309:ENSG00000010219:ENSG00000013588:ENSG00000123094:ENSG00000050426:ENSG00000123360:ENSG00000120868:ENSG00000174989:ENSG00000150990:ENSG00000102531:ENSG00000123179:ENSG00000088451:ENSG00000134901:ENSG00000134897:ENSG00000179476:ENSG00000198718:ENSG00000185246:ENSG00000187790:ENSG00000087301:ENSG00000119599:ENSG00000100767:ENSG00000254656:ENSG00000128731:ENSG00000186399:ENSG00000021776:ENSG00000174197:ENSG00000137802:ENSG00000243708:ENSG00000243789:ENSG00000140262:ENSG00000103707:ENSG00000136378:ENSG00000103888:ENSG00000117899:ENSG00000183426:ENSG00000085721:ENSG00000179965:ENSG00000140691:ENSG00000140682:ENSG00000102910:ENSG00000196470:ENSG00000132600:ENSG00000225614:ENSG00000167522:ENSG00000197912:ENSG00000127804:ENSG00000196689:ENSG00000197417:ENSG00000072849:ENSG00000141127:ENSG00000166484:ENSG00000142494:ENSG00000068079:ENSG00000184988:ENSG00000198933:ENSG00000011258:ENSG00000011260:ENSG00000214226:ENSG00000108423:ENSG00000108443:ENSG00000198265:ENSG00000154217:ENSG00000141540:ENSG00000141577:ENSG00000141384:ENSG00000141431:ENSG00000152223:ENSG00000130856:ENSG00000071564:ENSG00000171105:ENSG00000198342:ENSG00000132005:ENSG00000237440:ENSG00000269190:ENSG00000167625:ENSG00000079462:ENSG00000063127:ENSG00000142544:ENSG00000196214:ENSG00000167766:ENSG00000213020:ENSG00000196417:ENSG00000176293:ENSG00000196208:ENSG00000134323:ENSG00000084676:ENSG00000170820:ENSG00000075292:ENSG00000144034:ENSG00000144048:ENSG00000005448:ENSG00000144218:ENSG00000153107:ENSG00000163040:ENSG00000153250:ENSG00000231672:ENSG00000162804:ENSG00000088876:ENSG00000157617:ENSG00000160284:ENSG00000100342:ENSG00000100321:ENSG00000100324:ENSG00000128285:ENSG00000198951:ENSG00000186951:ENSG00000205643:ENSG00000075234:ENSG00000100416:ENSG00000008735:ENSG00000144560:ENSG00000182983:ENSG00000114388:ENSG00000180376:ENSG00000163946:ENSG00000114861:ENSG00000163320:ENSG00000184500:ENSG00000178700:ENSG00000114446:ENSG00000172780:ENSG00000034533:ENSG00000114670:ENSG00000240303:ENSG00000113971:ENSG00000158163:ENSG00000138231:ENSG00000158186:ENSG00000114115:ENSG00000155903:ENSG00000175066:ENSG00000114127:ENSG00000114698:ENSG00000075420:ENSG00000163904:ENSG00000013288:ENSG00000109689:ENSG00000138823:ENSG00000123739:ENSG00000205403:ENSG00000113360:ENSG00000164190:ENSG00000039123:ENSG00000164331:ENSG00000164180:ENSG00000153140:ENSG00000197948:ENSG00000113272:ENSG00000196670:ENSG00000197935:ENSG00000137404:ENSG00000137337:ENSG00000204344:ENSG00000244731:ENSG00000243955:ENSG00000146243:ENSG00000198945:ENSG00000118503:ENSG00000146416:ENSG00000091831:ENSG00000131018:ENSG00000213066:ENSG00000186340:ENSG00000184786:ENSG00000130023:ENSG00000217455:ENSG00000152926:ENSG00000127920:ENSG00000105829:ENSG00000078487:ENSG00000160813:ENSG00000005249:ENSG00000106484:ENSG00000158623:ENSG00

						000131558:ENSG00000182158:ENSG00000146966:ENSG00000214106:ENSG00000129691:ENSG00000147465:ENSG00000147601:ENSG00000196378:ENSG00000080608:ENSG00000044459:ENSG00000130958:ENSG00000136925:ENSG00000167081:ENSG00000136840:ENSG00000148339:ENSG00000170835
DEG. up	Pancreas	747	49	0.00480115782366874	0.259262522478112	ENSG00000179178:ENSG00000253313:ENSG00000162366:ENSG00000134709:ENSG00000143412:ENSG00000132703:ENSG00000143801:ENSG00000163050:ENSG00000152779:ENSG00000120057:ENSG00000203867:ENSG00000148702:ENSG00000006071:ENSG00000165905:ENSG00000149150:ENSG00000102743:ENSG00000100473:ENSG00000165807:ENSG00000103067:ENSG00000153822:ENSG00000186765:ENSG00000141401:ENSG00000171105:ENSG00000129354:ENSG00000250799:ENSG00000161270:ENSG00000179751:ENSG00000189377:ENSG00000142273:ENSG00000127831:ENSG00000170293:ENSG00000168032:ENSG00000178750:ENSG00000144867:ENSG00000118017:ENSG00000114115:ENSG00000080493:ENSG00000171522:ENSG00000152669:ENSG00000197822:ENSG00000112812:ENSG00000244067:ENSG00000243955:ENSG00000165215:ENSG00000189143:ENSG00000091704:ENSG00000184608:ENSG00000136840:ENSG00000170835
DEG. up	Pituitary	5188	225	0.688323643810621	1	ENSG00000158286:ENSG00000158292:ENSG00000097021:ENSG00000158816:ENSG00000188672:ENSG00000157978:ENSG00000160050:ENSG00000160051:ENSG00000116525:ENSG00000117395:ENSG00000243710:ENSG00000179178:ENSG00000253313:ENSG00000198198:ENSG00000117472:ENSG00000162390:ENSG00000134709:ENSG00000117155:ENSG00000069712:ENSG00000162694:ENSG00000116396:ENSG00000143630:ENSG00000160753:ENSG00000125459:ENSG00000132718:ENSG00000132680:ENSG00000242252:ENSG00000132694:ENSG00000198734:ENSG00000120341:ENSG00000143801:ENSG00000198879:ENSG00000241058:ENSG00000095794:ENSG00000188234:ENSG00000138134:ENSG00000152779:ENSG00000059573:ENSG00000095587:ENSG00000155256:ENSG00000184545:ENSG00000205869:ENSG00000166313:ENSG00000268403:ENSG00000188211:ENSG00000187486:ENSG00000006071:ENSG00000019505:ENSG00000121680:ENSG00000165905:ENSG00000124939:ENSG00000162174:ENSG00000149809:ENSG00000174276:ENSG00000173653:ENSG00000149256:ENSG00000149292:ENSG00000180425:ENSG00000023171:ENSG00000109832:ENSG00000010219:ENSG00000010626:ENSG00000111679:ENSG00000050426:ENSG00000110925:ENSG00000139410:ENSG00000174989:ENSG00000188735:ENSG00000127863:ENSG00000102531:ENSG00000088451:ENSG00000152749:ENSG00000129535:ENSG00000176165:ENSG00000092108:ENSG00000100473:ENSG00000165355:ENSG00000198718:ENSG00000184302:ENSG00000165807:ENSG00000006432:ENSG00000170270:ENSG00000156414:ENSG00000186399:ENSG00000243708:ENSG00000103888:ENSG00000117899:ENSG00000156206:ENSG00000008710:ENSG00000183426:ENSG00000140691:ENSG00000102910:ENSG00000132600:ENSG00000103056:ENSG00000197912:ENSG00000181350:ENSG00000141127:ENSG00000108641:ENSG00000142494:ENSG00000108830:ENSG00000153933:ENSG00000180616:ENSG00000171595:ENSG00000078687:ENSG00000141577:ENSG00000141431:ENSG00000130856:ENSG00000129354:ENSG00000132005:ENSG00000160951:ENSG00000099308:ENSG00000124302:ENSG00000262484:ENSG00000160570:ENSG00000079462:ENSG00000167619:ENSG00000167766:ENSG00000152467:ENSG00000176293:ENSG00000115756:ENSG00000196208:ENSG00000171094:ENSG00000162929:ENSG00000075292:ENSG00000005448:ENSG00000163040:ENSG00000153253:ENSG00000153832:ENSG00000144485:ENSG00000132326:ENSG00000101290:ENSG00000125966:ENSG00000124107:ENSG00000203896:ENSG00000160190:ENSG00000160284:ENSG00000184792:ENSG00000128335:ENSG00000128285:ENSG00000100197:ENSG00000100416:ENSG00000075275:ENSG00000205593:ENSG00000130487:ENSG00000205560:ENSG00000008735:ENSG00000144712:ENSG00000197385:ENSG00000177842:ENSG00000004838:ENSG00000114388:ENSG00000007402:ENSG00000180376:ENSG00000114446:ENSG00000240891:ENSG00000114670:ENSG00000240303:ENSG00000081307:ENSG00000144867:ENSG00000154917:ENSG00000114115:ENSG00000073803:ENSG00000109689:ENSG00000169019:ENSG00000145248:ENSG00000185477:ENSG00000123739:ENSG00000109466:ENSG00000113360:ENSG00000016082:ENSG00000152669:ENSG00000153140:ENSG00000164241:ENSG00000145794:ENSG00000164620:ENSG00000197948:ENSG00000074317:ENSG00000175766:ENSG00000048140:ENSG00000124535:ENSG00000112812:ENSG00000137337:ENSG00000244731:ENSG00000124772:ENSG00000137168:ENSG00000269964:ENSG00000146243:ENSG00000118432:ENSG00000132429:ENSG00000198945:ENSG00000135525:ENSG00000146416:ENSG00000091831:ENSG00000131018:ENSG00000213066:ENSG00000184786:ENSG00000130023:ENSG00000106113:ENSG00000169902:ENSG00000165215:ENSG00000177679:ENSG00000105829:ENSG00000214300:ENSG00000166924:ENSG00000106477:ENSG00000106484:ENSG00000182158:ENSG00000146966:ENSG00000181444:ENSG00000214106:ENSG00000154319:ENSG00000121039:ENSG00000104327:ENSG00000129295:ENSG00000169436:ENSG00000177335:ENSG00000186354:ENSG00000213694:ENSG00000167103:ENSG00000148339:ENSG00000170835:ENSG00000160339:ENSG00000233198:ENSG00000198569:ENSG00000197768
DEG. up	Prostate	3333	138	0.836992002648394	1	ENSG00000158286:ENSG00000162458:ENSG00000157978:ENSG00000160094:ENSG00000179178:ENSG00000253313:ENSG00000198198:ENSG00000117472:ENSG00000186564:ENSG00000116745:ENSG00000117114:ENSG00000137976:ENSG00000085465:ENSG00000092621:ENSG00000134240:ENSG00000163354:ENSG00000125459:ENSG00000196189:ENSG00000242252:ENSG00000163472:ENSG00000198715:ENSG00000174175:ENSG00000057252:ENSG00000122176:ENSG00000143801:ENSG00000198879:ENSG00000241058:ENSG00000188234:ENSG00000165338:ENSG00000110169:ENSG00000268403:ENSG00000135373:ENSG00000165905:ENSG00000124939:ENSG00000124935:ENSG00000168060:ENSG00000149809:ENSG00000173653:ENSG00000149292:ENSG00000111679:ENSG00000235162:ENSG00000187848:ENSG00000127863:ENSG00000136167:ENSG00000100473:ENSG00000165379:ENSG00000139926:ENSG00000087301:ENSG00000153485:ENSG00000243708:ENSG00000243789:ENSG00000103710:ENSG00000007038:ENSG00000140682:ENSG00000121270:ENSG00000103067:ENSG00000128482:ENSG00000068079:ENSG00000214226:ENSG00000108423:ENSG00000167302:ENSG00000141469:ENSG00000171105:ENSG00000104883:ENSG00000129354:ENSG00000104835:ENSG00000269190:ENSG00000189377:ENSG00000079385:ENSG00000142273:ENSG00000198464:ENSG00000167766:ENSG00000196417:ENSG00000176293:E

						NSG00000196208:ENSG00000144218:ENSG00000153214:ENSG00000163040:ENSG00000115963:ENSG00000132326:ENSG00000124233:ENSG00000124157:ENSG00000203896:ENSG00000157617:ENSG00000160190:ENSG00000160284:ENSG00000100342:ENSG00000100321:ENSG00000100197:ENSG00000205643:ENSG00000075234:ENSG00000100416:ENSG00000075275:ENSG00000177989:ENSG00000197385:ENSG00000182983:ENSG00000114395:ENSG00000114861:ENSG00000178750:ENSG00000114446:ENSG00000017260:ENSG00000240303:ENSG00000081307:ENSG00000175066:ENSG00000163898:ENSG00000080493:ENSG00000124875:ENSG00000163739:ENSG00000016082:ENSG00000152669:ENSG00000164331:ENSG00000164241:ENSG00000120318:ENSG00000155858:ENSG00000196670:ENSG00000137266:ENSG00000112812:ENSG00000197935:ENSG00000243955:ENSG00000135525:ENSG00000091831:ENSG00000026297:ENSG00000164488:ENSG00000184786:ENSG00000253293:ENSG00000005073:ENSG00000106031:ENSG00000106038:ENSG00000241644:ENSG00000165215:ENSG00000189143:ENSG00000121039:ENSG00000169436:ENSG00000213694:ENSG00000167081:ENSG00000167103:ENSG00000160339:ENSG00000233198
DEG.up	Skin_Not_Sun_Exposed_Suprapubic	2760	155	0.00157386781505119	0.084988862012764	ENSG00000069812:ENSG00000116525:ENSG00000168389:ENSG00000253313:ENSG00000162366:ENSG00000134709:ENSG00000142867:ENSG00000134215:ENSG00000085465:ENSG00000175984:ENSG00000213281:ENSG00000092621:ENSG00000134240:ENSG00000143452:ENSG00000143387:ENSG00000143412:ENSG00000143631:ENSG00000203786:ENSG00000240386:ENSG00000186226:ENSG00000172155:ENSG00000197084:ENSG00000196734:ENSG00000163354:ENSG00000163357:ENSG00000143630:ENSG00000143627:ENSG00000196189:ENSG00000163472:ENSG00000196224:ENSG00000241598:ENSG00000185940:ENSG00000166483:ENSG00000129151:ENSG00000135373:ENSG00000165905:ENSG00000124939:ENSG00000124935:ENSG00000149809:ENSG00000173653:ENSG00000166391:ENSG00000062282:ENSG00000064309:ENSG00000213782:ENSG00000134531:ENSG00000123364:ENSG00000123407:ENSG00000123388:ENSG00000139292:ENSG00000074590:ENSG00000139714:ENSG00000184992:ENSG00000177084:ENSG00000127863:ENSG00000133110:ENSG00000123179:ENSG00000118939:ENSG00000100473:ENSG00000139926:ENSG00000119599:ENSG00000080815:ENSG00000134160:ENSG00000198146:ENSG00000137802:ENSG00000243708:ENSG00000243789:ENSG00000181827:ENSG00000140471:ENSG00000157106:ENSG00000102921:ENSG00000102970:ENSG00000103067:ENSG00000103056:ENSG00000188522:ENSG00000072134:ENSG00000142494:ENSG00000180638:ENSG00000141750:ENSG00000011258:ENSG00000011260:ENSG00000017797:ENSG00000141401:ENSG00000168234:ENSG00000141384:ENSG00000081913:ENSG00000206073:ENSG00000167670:ENSG00000180739:ENSG00000129354:ENSG00000132016:ENSG00000132017:ENSG00000262484:ENSG00000160570:ENSG00000105427:ENSG00000142273:ENSG00000182324:ENSG00000119866:ENSG00000116039:ENSG00000144048:ENSG00000114993:ENSG00000115963:ENSG00000132326:ENSG00000175121:ENSG00000168703:ENSG00000124102:ENSG00000124107:ENSG00000154642:ENSG00000183640:ENSG00000184459:ENSG00000100321:ENSG00000075275:ENSG00000182606:ENSG00000182983:ENSG00000243477:ENSG00000114378:ENSG00000178750:ENSG00000129048:ENSG00000158163:ENSG00000163898:ENSG00000163975:ENSG00000178597:ENSG00000145246:ENSG00000109321:ENSG00000173085:ENSG00000169067:ENSG00000197822:ENSG00000164338:ENSG00000173926:ENSG00000064651:ENSG00000197948:ENSG00000137266:ENSG00000112812:ENSG00000204344:ENSG00000235863:ENSG00000135525:ENSG00000112357:ENSG00000118503:ENSG00000112406:ENSG00000164488:ENSG00000186340:ENSG00000122644:ENSG00000189143:ENSG00000049541:ENSG00000159763:ENSG00000154319:ENSG00000147465:ENSG00000104267:ENSG00000230453:ENSG00000127080:ENSG00000136826:ENSG00000070159:ENSG00000148356:ENSG00000167103:ENSG00000122136:ENSG00000122133
DEG.up	Skin_Sun_Exposed_Lower_leg	2825	159	0.00125026231804428	0.067514165174391	ENSG00000158292:ENSG00000069812:ENSG00000116525:ENSG00000127603:ENSG00000168389:ENSG00000179178:ENSG00000253313:ENSG00000162366:ENSG00000134709:ENSG00000134215:ENSG00000085465:ENSG00000175984:ENSG00000213281:ENSG00000092621:ENSG00000134240:ENSG00000143452:ENSG00000143387:ENSG00000143412:ENSG00000143631:ENSG00000203786:ENSG00000240386:ENSG00000186226:ENSG00000172155:ENSG00000197084:ENSG00000196734:ENSG00000163354:ENSG00000163357:ENSG00000143630:ENSG00000196189:ENSG00000163472:ENSG00000196224:ENSG00000241598:ENSG00000185940:ENSG00000166483:ENSG00000129151:ENSG00000135373:ENSG00000165905:ENSG00000124939:ENSG00000124935:ENSG00000149809:ENSG00000173653:ENSG00000062282:ENSG00000154127:ENSG00000064309:ENSG00000213782:ENSG00000134531:ENSG00000123364:ENSG00000123407:ENSG00000123388:ENSG00000139292:ENSG00000074590:ENSG00000139714:ENSG00000177084:ENSG00000127863:ENSG00000133110:ENSG00000123179:ENSG00000118939:ENSG00000139926:ENSG00000119599:ENSG00000080815:ENSG00000100767:ENSG00000134160:ENSG00000198146:ENSG00000137802:ENSG00000243708:ENSG00000243789:ENSG00000181827:ENSG00000140471:ENSG00000157106:ENSG00000102921:ENSG00000102970:ENSG00000103067:ENSG00000103056:ENSG00000197417:ENSG00000161944:ENSG00000188522:ENSG00000072134:ENSG00000166484:ENSG00000180638:ENSG00000141750:ENSG00000225190:ENSG00000011258:ENSG00000011260:ENSG00000017797:ENSG00000141401:ENSG00000168234:ENSG00000141384:ENSG00000081913:ENSG00000167670:ENSG00000180739:ENSG00000129354:ENSG00000132016:ENSG00000132000:ENSG00000132017:ENSG00000262484:ENSG00000160570:ENSG00000105427:ENSG00000142273:ENSG00000182324:ENSG00000119866:ENSG00000116039:ENSG00000144048:ENSG00000114993:ENSG00000115963:ENSG00000153832:ENSG00000132326:ENSG00000188559:ENSG00000175121:ENSG00000168703:ENSG00000124102:ENSG00000124107:ENSG00000154642:ENSG00000183640:ENSG00000184459:ENSG00000100321:ENSG00000075275:ENSG00000070950:ENSG00000182606:ENSG00000182983:ENSG00000114378:ENSG00000178750:ENSG00000129048:ENSG00000158163:ENSG00000163898:ENSG00000041802:ENSG00000163975:ENSG00000178597:ENSG00000145246:ENSG00000173085:ENSG00000197822:ENSG00000164338:ENSG00000173926:ENSG00000064651:ENSG00000197948:ENSG00000137266:ENSG00000112812:ENSG00000204344:ENSG00000235863:ENSG00000164530:ENSG00000135525:ENSG00000112357:ENSG00000118503:ENSG00000112406:ENSG00000164488:ENSG00000186340:ENSG00000122644:ENSG00000165215:ENSG00000189143:EN

						SG00000049541:ENSG00000158623:ENSG00000159763:ENSG00000154319:ENSG00000127080:ENSG00000136826:ENSG00000070159:ENSG00000148356:ENSG00000167103:ENSG00000122136:ENSG00000122133
DEG. up	Small_Intestine_Terminal_Ileum	2786	140	0.0719528165199412	1	ENSG00000158292:ENSG00000162460:ENSG00000162458:ENSG00000168389:ENSG00000179178:ENSG00000253313:ENSG00000117399:ENSG00000117472:ENSG00000162366:ENSG00000162390:ENSG00000162391:ENSG00000142867:ENSG00000134240:ENSG00000163131:ENSG00000163154:ENSG00000143630:ENSG00000143627:ENSG00000132185:ENSG00000120341:ENSG00000186283:ENSG00000075073:ENSG00000138134:ENSG00000059573:ENSG00000120057:ENSG00000187908:ENSG00000135373:ENSG00000168060:ENSG00000175567:ENSG00000166391:ENSG00000110777:ENSG00000111679:ENSG00000111684:ENSG00000123338:ENSG00000139292:ENSG00000111725:ENSG00000184992:ENSG00000187848:ENSG00000102743:ENSG00000136167:ENSG00000102445:ENSG00000102524:ENSG00000100889:ENSG00000150527:ENSG00000100767:ENSG00000186198:ENSG00000102879:ENSG00000132207:ENSG00000180096:ENSG00000102970:ENSG00000103056:ENSG00000185669:ENSG00000158717:ENSG00000132514:ENSG00000065325:ENSG00000233670:ENSG00000181350:ENSG00000154025:ENSG00000161405:ENSG00000068079:ENSG00000214226:ENSG00000167302:ENSG00000071564:ENSG00000205744:ENSG00000263264:ENSG00000104880:ENSG00000129354:ENSG00000123146:ENSG00000160951:ENSG00000096996:ENSG00000105369:ENSG00000028277:ENSG00000079385:ENSG00000142273:ENSG00000105483:ENSG00000105492:ENSG00000115756:ENSG00000163803:ENSG00000119866:ENSG00000144218:ENSG00000115604:ENSG00000115607:ENSG00000153214:ENSG00000127831:ENSG00000168955:ENSG00000144485:ENSG00000124102:ENSG00000124256:ENSG00000160190:ENSG00000100342:ENSG00000100197:ENSG00000186951:ENSG00000075234:ENSG00000130487:ENSG00000197385:ENSG00000114395:ENSG00000178750:ENSG00000240891:ENSG00000144852:ENSG00000034533:ENSG00000114113:ENSG00000114248:ENSG00000172578:ENSG00000163898:ENSG00000163975:ENSG00000109689:ENSG00000163739:ENSG00000145287:ENSG00000185477:ENSG00000138823:ENSG00000174358:ENSG00000171522:ENSG00000164241:ENSG00000064651:ENSG00000255833:ENSG00000197948:ENSG00000186314:ENSG00000113722:ENSG00000135074:ENSG00000079691:ENSG00000112812:ENSG00000244067:ENSG00000243955:ENSG00000118503:ENSG00000026297:ENSG00000122644:ENSG00000146757:ENSG00000126522:ENSG00000165215:ENSG00000189143:ENSG00000086730:ENSG00000085563:ENSG00000196329:ENSG00000136573:ENSG00000254087:ENSG00000121039:ENSG00000079112:ENSG00000165071:ENSG00000130958:ENSG00000198569:ENSG00000198113
DEG. up	Spleen	4308	187	0.667015360077045	1	ENSG00000116809:ENSG00000157978:ENSG00000066056:ENSG00000117399:ENSG00000162367:ENSG00000198841:ENSG00000157211:ENSG00000134215:ENSG00000162775:ENSG00000116455:ENSG00000163131:ENSG00000143379:ENSG00000163154:ENSG00000163156:ENSG00000143622:ENSG00000116584:ENSG00000196189:ENSG00000143226:ENSG00000173110:ENSG00000203747:ENSG00000132185:ENSG00000162746:ENSG00000198734:ENSG00000120341:ENSG00000186283:ENSG00000198879:ENSG00000188234:ENSG00000214688:ENSG00000107738:ENSG00000138134:ENSG00000198873:ENSG00000268403:ENSG00000168060:ENSG00000146670:ENSG00000175567:ENSG00000110777:ENSG00000154127:ENSG00000109943:ENSG00000111679:ENSG00000123338:ENSG00000111725:ENSG00000122966:ENSG00000187848:ENSG00000177084:ENSG00000136167:ENSG00000102445:ENSG00000152213:ENSG00000102524:ENSG00000100889:ENSG00000139914:ENSG00000126787:ENSG00000100767:ENSG00000156414:ENSG00000140350:ENSG00000140406:ENSG00000140471:ENSG00000102879:ENSG00000132207:ENSG00000180096:ENSG00000103066:ENSG00000185669:ENSG00000158717:ENSG00000205022:ENSG00000132514:ENSG00000161944:ENSG00000181350:ENSG00000154025:ENSG00000228157:ENSG00000161405:ENSG00000068079:ENSG00000184988:ENSG00000198933:ENSG00000073861:ENSG00000214226:ENSG00000153933:ENSG00000121060:ENSG00000123700:ENSG00000180616:ENSG00000196169:ENSG00000141577:ENSG00000167302:ENSG00000185504:ENSG00000071564:ENSG00000205744:ENSG00000171105:ENSG00000263264:ENSG00000104880:ENSG00000167775:ENSG00000180739:ENSG00000132017:ENSG00000123146:ENSG00000160951:ENSG00000096996:ENSG00000099308:ENSG00000105369:ENSG00000028277:ENSG00000160570:ENSG00000105483:ENSG00000105383:ENSG00000171049:ENSG00000115705:ENSG00000197872:ENSG00000163803:ENSG00000119866:ENSG00000115604:ENSG00000115607:ENSG00000153208:ENSG00000144401:ENSG00000018280:ENSG00000196209:ENSG00000124102:ENSG00000124256:ENSG00000203896:ENSG00000157617:ENSG00000128335:ENSG00000100342:ENSG00000179750:ENSG00000075218:ENSG00000130489:ENSG00000177989:ENSG00000130487:ENSG00000205560:ENSG00000251322:ENSG00000197385:ENSG00000114378:ENSG00000068001:ENSG00000114388:ENSG00000114395:ENSG00000163606:ENSG00000144843:ENSG00000169704:ENSG00000138231:ENSG00000155903:ENSG00000163762:ENSG00000172578:ENSG00000145014:ENSG00000109689:ENSG00000145246:ENSG00000124875:ENSG00000109272:ENSG00000163739:ENSG00000145287:ENSG00000173085:ENSG00000185477:ENSG00000205403:ENSG00000164128:ENSG00000164129:ENSG00000171522:ENSG00000132357:ENSG00000164620:ENSG00000197948:ENSG00000120318:ENSG00000135074:ENSG00000131446:ENSG00000146112:ENSG00000255587:ENSG00000124772:ENSG00000198945:ENSG00000118503:ENSG00000112406:ENSG00000120278:ENSG00000026297:ENSG00000184786:ENSG00000130023:ENSG00000146757:ENSG00000126522:ENSG00000086730:ENSG00000135218:ENSG00000127920:ENSG00000085514:ENSG00000160813:ENSG00000091704:ENSG00000106484:ENSG00000197993:ENSG00000221937:ENSG00000268170:ENSG00000181444:ENSG00000196329:ENSG00000136573:ENSG00000168546:ENSG00000254087:ENSG00000165071:ENSG00000177335:ENSG00000136840:ENSG00000171169:ENSG00000160339:ENSG00000198113
DEG. up	Stomach	1109	59	0.0918933685647684	1	ENSG00000179178:ENSG00000253313:ENSG00000117472:ENSG00000162366:ENSG00000203943:ENSG00000134240:ENSG00000120341:ENSG00000122176:ENSG00000163050:ENSG00000075073:ENSG00000120057:ENSG00000148702:ENSG00000187486:ENSG00000135373:ENSG00000165905:ENSG00000149150:ENSG00

						000229859:ENSG00000124939:ENSG00000110777:ENSG00000111684:ENSG00000013588:ENSG00000235162:ENSG00000111725:ENSG00000133110:ENSG00000150527:ENSG00000165807:ENSG00000103067:ENSG00000103056:ENSG00000065325:ENSG00000153822:ENSG00000263264:ENSG00000129354:ENSG00000160951:ENSG00000189377:ENSG00000142273:ENSG00000115602:ENSG00000180251:ENSG00000170293:ENSG00000178750:ENSG00000118017:ENSG00000114248:ENSG00000163898:ENSG00000178597:ENSG00000145287:ENSG00000016082:ENSG00000234602:ENSG00000152669:ENSG00000197822:ENSG00000164180:ENSG00000064651:ENSG00000186314:ENSG00000112812:ENSG00000244067:ENSG00000243955:ENSG00000172461:ENSG00000112357:ENSG00000121039:ENSG00000104267:ENSG00000198113
DEG. up	Testis	12379	414	0.999999999999999	1	ENSG00000097021:ENSG00000198793:ENSG00000120942:ENSG00000116809:ENSG00000158816:ENSG00000188672:ENSG00000204178:ENSG00000134668:ENSG00000160050:ENSG00000160051:ENSG00000184389:ENSG00000168389:ENSG00000117395:ENSG00000243710:ENSG00000117400:ENSG00000117399:ENSG00000198198:ENSG00000117472:ENSG00000123473:ENSG00000186790:ENSG00000198841:ENSG00000116205:ENSG00000134709:ENSG00000203943:ENSG00000117133:ENSG00000180869:ENSG00000117155:ENSG00000197147:ENSG00000069712:ENSG00000117569:ENSG00000162775:ENSG00000085465:ENSG00000163349:ENSG00000197323:ENSG00000175984:ENSG00000092621:ENSG00000134240:ENSG00000143457:ENSG00000143452:ENSG00000143379:ENSG00000143409:ENSG00000143363:ENSG00000143434:ENSG00000163206:ENSG00000163354:ENSG00000163357:ENSG00000143627:ENSG00000125459:ENSG00000160838:ENSG00000132694:ENSG00000066279:ENSG00000134376:ENSG00000143801:ENSG00000162714:ENSG00000065328:ENSG00000241058:ENSG00000204683:ENSG00000108094:ENSG00000095794:ENSG00000231122:ENSG00000188234:ENSG00000107736:ENSG00000214688:ENSG00000148602:ENSG00000148604:ENSG00000138134:ENSG00000188649:ENSG00000155256:ENSG00000165832:ENSG00000268403:ENSG00000166478:ENSG00000166483:ENSG00000198730:ENSG00000188211:ENSG00000121680:ENSG00000162174:ENSG00000168061:ENSG00000168060:ENSG00000146670:ENSG00000187066:ENSG00000174276:ENSG00000173653:ENSG00000173599:ENSG00000173621:ENSG00000187726:ENSG00000175564:ENSG00000062282:ENSG00000179240:ENSG00000137707:ENSG00000183644:ENSG00000149292:ENSG00000180425:ENSG00000023171:ENSG00000109832:ENSG00000010219:ENSG00000111254:ENSG00000111671:ENSG00000010626:ENSG00000134532:ENSG00000123094:ENSG00000214511:ENSG00000110925:ENSG00000197651:ENSG00000235162:ENSG00000139410:ENSG00000111725:ENSG00000139714:ENSG00000177084:ENSG00000176915:ENSG00000102743:ENSG00000139656:ENSG00000136167:ENSG00000102445:ENSG00000102753:ENSG00000176124:ENSG00000118939:ENSG00000129535:ENSG00000100889:ENSG00000176165:ENSG00000186960:ENSG00000092140:ENSG00000203546:ENSG00000150527:ENSG00000165355:ENSG00000179476:ENSG00000187790:ENSG00000139926:ENSG00000180998:ENSG00000126787:ENSG00000165807:ENSG00000006432:ENSG00000165417:ENSG00000165934:ENSG00000170270:ENSG00000012963:ENSG00000258987:ENSG00000156414:ENSG00000179938:ENSG00000186399:ENSG00000178115:ENSG00000261794:ENSG00000187951:ENSG00000134160:ENSG00000169918:ENSG00000184507:ENSG00000174197:ENSG00000137802:ENSG00000181827:ENSG00000186198:ENSG00000188779:ENSG00000117971:ENSG00000156206:ENSG00000140600:ENSG00000251209:ENSG00000140986:ENSG00000007038:ENSG00000183426:ENSG00000155714:ENSG00000180209:ENSG00000140691:ENSG00000121270:ENSG00000196470:ENSG00000005194:ENSG00000072736:ENSG00000103067:ENSG00000103056:ENSG00000167515:ENSG00000197912:ENSG00000072849:ENSG00000007174:ENSG00000181350:ENSG00000141127:ENSG00000108641:ENSG00000166484:ENSG00000142494:ENSG00000186075:ENSG00000108830:ENSG00000012048:ENSG00000184988:ENSG00000073670:ENSG00000184361:ENSG00000198933:ENSG00000011258:ENSG00000011260:ENSG00000214226:ENSG00000153933:ENSG00000189050:ENSG00000198265:ENSG00000197170:ENSG00000141540:ENSG00000171595:ENSG00000141576:ENSG00000141577:ENSG00000182446:ENSG00000168234:ENSG00000141384:ENSG00000141431:ENSG00000206181:ENSG00000215474:ENSG00000081913:ENSG00000130856:ENSG00000071564:ENSG00000167670:ENSG00000104833:ENSG00000104883:ENSG00000167775:ENSG00000181786:ENSG00000181143:ENSG00000065989:ENSG00000132016:ENSG00000132017:ENSG00000132005:ENSG00000171136:ENSG00000250799:ENSG00000161270:ENSG00000104825:ENSG00000262484:ENSG00000104835:ENSG00000179751:ENSG00000028277:ENSG00000160570:ENSG00000079462:ENSG00000188368:ENSG00000167619:ENSG00000079435:ENSG00000105464:ENSG00000063127:ENSG00000126467:ENSG00000196214:ENSG00000198633:ENSG00000196417:ENSG00000224730:ENSG00000152467:ENSG00000176293:ENSG00000115756:ENSG00000115761:ENSG00000196208:ENSG00000134323:ENSG00000163803:ENSG00000163806:ENSG00000171094:ENSG00000170820:ENSG00000162928:ENSG00000082898:ENSG00000075292:ENSG00000144034:ENSG00000188687:ENSG00000005448:ENSG00000198885:ENSG00000153208:ENSG00000115762:ENSG00000188219:ENSG00000163040:ENSG00000118260:ENSG00000135912:ENSG00000168955:ENSG00000123977:ENSG00000153832:ENSG00000186235:ENSG00000144485:ENSG00000132326:ENSG00000125779:ENSG00000089048:ENSG00000101247:ENSG00000125864:ENSG00000131050:ENSG00000155313:ENSG00000154642:ENSG00000160190:ENSG00000160284:ENSG00000183628:ENSG00000184792:ENSG00000183066:ENSG00000100197:ENSG00000130943:ENSG00000075218:ENSG00000205593:ENSG00000217442:ENSG00000205560:ENSG00000008735:ENSG00000134077:ENSG00000144712:ENSG00000163517:ENSG00000092345:ENSG00000163576:ENSG00000230891:ENSG00000197385:ENSG00000240747:ENSG00000179564:ENSG00000186792:ENSG00000243477:ENSG00000004838:ENSG00000007402:ENSG00000180376:ENSG00000163946:ENSG00000144736:ENSG00000179021:ENSG00000114446:ENSG00000240891:ENSG00000163607:ENSG00000082701:ENSG00000169704:ENSG00000034533:ENSG00000114670:ENSG00000144867:ENSG00000114115:ENSG00000163904:ENSG00000163915:ENSG00000205835:ENSG00000152953:ENSG00000178597:ENSG00000169019:ENSG00000109171:ENSG00000182223:ENSG00000109321:ENSG00000151247:ENSG00000138813:ENSG00000145331:ENSG00000138823:ENSG00000164129:ENSG00000176979:

						ENSG00000164362:ENSG00000016082:ENSG00000152669:ENSG00000039123:ENSG00000068796:ENSG00000086200:ENSG00000153140:ENSG00000173926:ENSG00000164241:ENSG00000255833:ENSG00000145826:ENSG00000164620:ENSG00000204928:ENSG00000113722:ENSG00000039600:ENSG00000187658:ENSG00000113272:ENSG00000155858:ENSG00000185056:ENSG00000048140:ENSG00000249109:ENSG00000124787:ENSG00000079691:ENSG00000197935:ENSG00000137404:ENSG00000137337:ENSG00000204344:ENSG00000235863:ENSG00000204220:ENSG00000124772:ENSG00000137168:ENSG00000244067:ENSG00000243955:ENSG00000269964:ENSG00000146243:ENSG00000132429:ENSG00000198945:ENSG00000135525:ENSG00000112357:ENSG00000213066:ENSG00000184786:ENSG00000130023:ENSG00000122644:ENSG00000106038:ENSG00000106113:ENSG00000152926:ENSG00000146757:ENSG00000169902:ENSG00000214300:ENSG00000078487:ENSG00000160813:ENSG00000185955:ENSG00000166924:ENSG00000005249:ENSG00000105865:ENSG00000075790:ENSG00000091704:ENSG00000106477:ENSG00000106484:ENSG00000158623:ENSG00000213265:ENSG00000183470:ENSG00000197993:ENSG00000146918:ENSG00000168546:ENSG00000120910:ENSG00000129691:ENSG00000147465:ENSG00000023287:ENSG00000104267:ENSG00000176571:ENSG00000120526:ENSG00000129295:ENSG00000131773:ENSG00000169436:ENSG00000104499:ENSG00000160882:ENSG00000168263:ENSG00000044459:ENSG00000205442:ENSG00000230453:ENSG00000127080:ENSG00000188152:ENSG00000196152:ENSG00000167103:ENSG00000171169:ENSG00000148335:ENSG00000179058:ENSG00000122136:ENSG00000122133:ENSG00000233198:ENSG00000188163:ENSG00000197768
DEG. up	Thyroid	5722	248	0.701916109648004	1	ENSG00000158286:ENSG00000065526:ENSG00000116809:ENSG00000157978:ENSG00000160050:ENSG00000160051:ENSG00000179178:ENSG00000253313:ENSG00000066056:ENSG00000198198:ENSG00000117472:ENSG00000162367:ENSG00000162390:ENSG00000134709:ENSG00000117114:ENSG00000203943:ENSG00000168679:ENSG00000143379:ENSG00000143412:ENSG00000143409:ENSG00000143363:ENSG00000125459:ENSG00000116580:ENSG00000196189:ENSG00000242252:ENSG00000187800:ENSG00000132694:ENSG00000120341:ENSG00000122176:ENSG00000162714:ENSG00000107929:ENSG00000134463:ENSG00000241058:ENSG00000188234:ENSG00000152779:ENSG00000059573:ENSG00000268403:ENSG00000006071:ENSG00000129151:ENSG00000165905:ENSG00000149150:ENSG00000174276:ENSG00000175567:ENSG00000149256:ENSG00000137672:ENSG00000137714:ENSG00000149292:ENSG00000180425:ENSG00000064309:ENSG00000111671:ENSG00000111679:ENSG00000013588:ENSG00000134531:ENSG00000110911:ENSG00000050426:ENSG00000139410:ENSG00000174989:ENSG00000111725:ENSG00000188735:ENSG00000102743:ENSG00000102531:ENSG00000136104:ENSG00000118939:ENSG00000136153:ENSG00000088451:ENSG00000129535:ENSG00000100889:ENSG00000168952:ENSG00000092108:ENSG00000150527:ENSG00000165355:ENSG00000198718:ENSG00000119599:ENSG00000165861:ENSG00000100767:ENSG00000165417:ENSG00000153485:ENSG00000156414:ENSG00000186399:ENSG00000178115:ENSG00000261794:ENSG00000137802:ENSG00000243708:ENSG00000243789:ENSG00000117899:ENSG00000140471:ENSG00000132207:ENSG00000261052:ENSG00000198064:ENSG00000140691:ENSG00000197302:ENSG00000102910:ENSG00000103067:ENSG00000103066:ENSG00000103064:ENSG00000197912:ENSG00000127804:ENSG00000072849:ENSG00000141127:ENSG00000108641:ENSG00000068079:ENSG00000108830:ENSG00000012048:ENSG00000184988:ENSG00000108423:ENSG00000198265:ENSG00000153822:ENSG00000167302:ENSG00000186765:ENSG00000185504:ENSG00000154889:ENSG00000141431:ENSG00000152223:ENSG00000071564:ENSG00000171105:ENSG00000104883:ENSG00000167775:ENSG00000065989:ENSG00000129354:ENSG00000132005:ENSG00000237440:ENSG00000104835:ENSG00000105483:ENSG00000182324:ENSG00000142544:ENSG00000198464:ENSG00000167766:ENSG00000213020:ENSG00000152467:ENSG00000176293:ENSG00000115705:ENSG00000134323:ENSG00000197872:ENSG00000162928:ENSG00000075292:ENSG00000188687:ENSG00000114993:ENSG00000144283:ENSG00000144401:ENSG00000231672:ENSG00000153832:ENSG00000162804:ENSG00000088876:ENSG00000125864:ENSG00000188559:ENSG00000203896:ENSG00000160190:ENSG00000100197:ENSG00000205643:ENSG00000100416:ENSG00000075275:ENSG00000205593:ENSG00000100253:ENSG00000177989:ENSG00000205560:ENSG00000251322:ENSG00000144560:ENSG00000170293:ENSG00000177842:ENSG00000182606:ENSG00000144648:ENSG00000182983:ENSG00000068001:ENSG00000114395:ENSG00000180376:ENSG00000163946:ENSG00000169379:ENSG00000114446:ENSG00000163608:ENSG00000172780:ENSG00000034533:ENSG00000114670:ENSG00000113971:ENSG00000081307:ENSG00000138231:ENSG00000114698:ENSG00000163762:ENSG00000169282:ENSG00000174899:ENSG00000075420:ENSG00000073803:ENSG00000041802:ENSG00000013288:ENSG00000157765:ENSG00000145246:ENSG00000080493:ENSG00000205403:ENSG00000113360:ENSG00000164190:ENSG00000132357:ENSG00000234602:ENSG00000152669:ENSG00000067248:ENSG00000197822:ENSG00000164331:ENSG00000164180:ENSG00000168916:ENSG00000164241:ENSG00000197948:ENSG00000120318:ENSG00000113742:ENSG00000131446:ENSG00000196670:ENSG00000124535:ENSG00000112812:ENSG00000197935:ENSG00000137337:ENSG00000244731:ENSG00000223501:ENSG00000137168:ENSG00000164530:ENSG00000269964:ENSG00000146243:ENSG00000198945:ENSG00000146416:ENSG00000131018:ENSG00000026297:ENSG00000213066:ENSG00000184786:ENSG00000130023:ENSG00000106113:ENSG00000241644:ENSG00000126522:ENSG00000165215:ENSG00000189143:ENSG00000135218:ENSG00000127920:ENSG00000078487:ENSG00000160813:ENSG00000075790:ENSG00000196937:ENSG00000182158:ENSG00000181444:ENSG00000196329:ENSG00000154319:ENSG00000168546:ENSG00000165061:ENSG00000254087:ENSG00000129295:ENSG00000177335:ENSG00000130958:ENSG00000136925:ENSG00000136936:ENSG00000070159:ENSG00000167081:ENSG00000160339:ENSG00000233198:ENSG00000197768
DEG. up	Uterus	5589	278	0.0221360520096186	1	ENSG00000158286:ENSG00000158292:ENSG00000120942:ENSG00000162458:ENSG00000065526:ENSG00000116809:ENSG00000187942:ENSG00000160051:ENSG00000160094:ENSG00000127603:ENSG00000066056:ENSG00000198198:ENSG00000162367:ENSG00000162604:ENSG00000117133:ENSG00000171488:ENSG00

						000117569:ENSG00000162694:ENSG00000117543:ENSG00000116396:ENSG00000085465:ENSG00000197323:ENSG00000143387:ENSG00000143437:ENSG00000143379:ENSG00000143409:ENSG00000143363:ENSG00000143434:ENSG00000163155:ENSG00000160767:ENSG00000160753:ENSG00000116539:ENSG00000125459:ENSG00000116580:ENSG00000132718:ENSG00000242252:ENSG00000187800:ENSG00000132694:ENSG00000174175:ENSG00000122176:ENSG00000143801:ENSG00000116962:ENSG00000162714:ENSG00000198879:ENSG00000148468:ENSG00000188234:ENSG00000075073:ENSG00000107736:ENSG00000026103:ENSG00000165338:ENSG00000188649:ENSG00000203867:ENSG00000165832:ENSG00000268403:ENSG00000166478:ENSG00000166483:ENSG00000198730:ENSG00000149150:ENSG00000110025:ENSG00000168060:ENSG00000173653:ENSG00000137492:ENSG00000076053:ENSG00000166250:ENSG00000064309:ENSG00000010219:ENSG00000123094:ENSG00000087494:ENSG00000110925:ENSG00000135457:ENSG00000235162:ENSG00000074590:ENSG00000174989:ENSG00000188735:ENSG00000150990:ENSG00000120688:ENSG00000102531:ENSG00000123179:ENSG00000136104:ENSG00000178695:ENSG00000134901:ENSG00000134897:ENSG00000139826:ENSG00000092140:ENSG00000179476:ENSG00000198718:ENSG00000185246:ENSG00000139926:ENSG00000087301:ENSG00000100767:ENSG00000133961:ENSG00000128731:ENSG00000021776:ENSG00000174197:ENSG00000140262:ENSG00000103707:ENSG00000103710:ENSG00000136378:ENSG00000140511:ENSG00000140471:ENSG00000008710:ENSG00000183426:ENSG00000180035:ENSG00000179965:ENSG00000140691:ENSG00000140682:ENSG00000102910:ENSG00000196470:ENSG00000132600:ENSG00000225614:ENSG00000167522:ENSG00000127804:ENSG00000005100:ENSG00000072134:ENSG00000108641:ENSG00000166484:ENSG00000142494:ENSG00000068079:ENSG00000184988:ENSG00000073670:ENSG00000182963:ENSG00000198933:ENSG00000011258:ENSG00000214226:ENSG00000153933:ENSG00000108423:ENSG00000108443:ENSG00000198265:ENSG00000078687:ENSG00000141577:ENSG00000167302:ENSG00000186765:ENSG00000185504:ENSG00000130856:ENSG00000071564:ENSG00000171105:ENSG00000263264:ENSG00000104880:ENSG00000167775:ENSG00000132005:ENSG00000123146:ENSG00000269190:ENSG00000167625:ENSG00000185453:ENSG00000105483:ENSG00000105447:ENSG00000063127:ENSG00000142544:ENSG00000198464:ENSG00000167766:ENSG00000213020:ENSG00000196417:ENSG00000176293:ENSG00000115756:ENSG00000196208:ENSG00000084676:ENSG00000171103:ENSG00000082898:ENSG00000075292:ENSG00000144034:ENSG00000144048:ENSG00000065911:ENSG00000005448:ENSG00000144218:ENSG00000153107:ENSG00000163040:ENSG00000153250:ENSG00000118260:ENSG00000197713:ENSG00000231672:ENSG00000186235:ENSG00000144485:ENSG00000162804:ENSG00000088876:ENSG00000125864:ENSG00000155313:ENSG00000128335:ENSG00000100342:ENSG00000100321:ENSG00000100324:ENSG00000128285:ENSG00000198951:ENSG00000177096:ENSG00000138944:ENSG00000205643:ENSG00000205593:ENSG00000251322:ENSG00000144560:ENSG00000144712:ENSG00000170011:ENSG00000182983:ENSG00000243477:ENSG00000068001:ENSG00000180376:ENSG00000163946:ENSG00000163320:ENSG00000169379:ENSG00000178700:ENSG00000114446:ENSG00000163608:ENSG00000240682:ENSG00000034533:ENSG00000240303:ENSG00000113971:ENSG00000129048:ENSG00000158163:ENSG00000138231:ENSG00000114115:ENSG00000155903:ENSG00000175066:ENSG00000114127:ENSG00000114698:ENSG00000163762:ENSG00000075420:ENSG00000145014:ENSG00000041802:ENSG00000163975:ENSG00000152953:ENSG00000013288:ENSG00000109689:ENSG00000145246:ENSG00000205403:ENSG00000113360:ENSG00000164190:ENSG00000067248:ENSG00000039123:ENSG00000164331:ENSG00000164180:ENSG00000164241:ENSG00000240184:ENSG00000197948:ENSG00000120318:ENSG00000113272:ENSG00000155858:ENSG00000196670:ENSG00000146112:ENSG00000137404:ENSG00000137337:ENSG00000124772:ENSG00000146243:ENSG00000198945:ENSG00000091831:ENSG00000131018:ENSG00000213066:ENSG00000186340:ENSG00000184786:ENSG00000130023:ENSG00000253293:ENSG00000005073:ENSG00000106031:ENSG00000153814:ENSG00000169902:ENSG00000177679:ENSG00000085563:ENSG00000127920:ENSG00000105829:ENSG00000166924:ENSG00000075790:ENSG00000196937:ENSG00000158623:ENSG00000182158:ENSG00000146966:ENSG00000181444:ENSG00000196329:ENSG00000214106:ENSG00000147601:ENSG00000164949:ENSG00000120526:ENSG00000169436:ENSG00000177335:ENSG00000196378:ENSG00000044459:ENSG00000178031:ENSG00000213694:ENSG00000136925:ENSG00000136936:ENSG00000182752:ENSG00000148339
DEG. up	Vagina	2864	130	0.425890463217184	1	ENSG00000158286:ENSG00000158292:ENSG00000069812:ENSG00000168389:ENSG00000253313:ENSG00000117399:ENSG00000162366:ENSG00000162390:ENSG00000134709:ENSG00000117114:ENSG00000142867:ENSG00000134215:ENSG00000116396:ENSG00000175984:ENSG00000092621:ENSG00000143387:ENSG00000143412:ENSG00000203786:ENSG00000163354:ENSG00000143630:ENSG00000160753:ENSG00000143622:ENSG00000196189:ENSG00000163472:ENSG00000174175:ENSG00000122176:ENSG00000075073:ENSG00000203867:ENSG00000129151:ENSG00000135373:ENSG00000165905:ENSG00000168060:ENSG00000173653:ENSG00000166250:ENSG00000013588:ENSG00000134531:ENSG00000087494:ENSG00000235162:ENSG00000188735:ENSG00000123179:ENSG00000136153:ENSG00000100473:ENSG00000150527:ENSG00000165379:ENSG00000139926:ENSG00000197930:ENSG00000006432:ENSG00000198146:ENSG00000243708:ENSG00000243789:ENSG00000103067:ENSG00000196689:ENSG00000197417:ENSG00000188522:ENSG00000072134:ENSG00000166484:ENSG00000142494:ENSG00000225190:ENSG00000214226:ENSG00000108423:ENSG00000108443:ENSG00000141401:ENSG00000206073:ENSG00000180739:ENSG00000129354:ENSG00000132016:ENSG00000132000:ENSG00000269190:ENSG00000105427:ENSG00000189377:ENSG00000079385:ENSG00000142273:ENSG00000196208:ENSG00000119866:ENSG00000144048:ENSG00000115963:ENSG00000175121:ENSG00000168703:ENSG00000124102:ENSG00000100342:ENSG00000100321:ENSG00000138944:ENSG00000075275:ENSG00000144560:ENSG00000197385:ENSG00000182983:ENSG00000175105:ENSG00000178750:ENSG00000113971:ENSG00000158163:ENSG00000155903:ENSG00000163898:ENSG00000163975:ENSG00000145246:ENSG00000124875:ENSG00000109321:ENSG00000016082:ENSG00000197822:ENSG00000164180:ENSG00000197948:ENSG00000120318:ENSG00000155858:ENSG00000137266:ENSG00000124787:ENSG00000137404:ENSG00000204544:ENSG00000164530:

						ENSG00000198945:ENSG00000091831:ENSG00000186340:ENSG00000217455:ENSG00000122644:ENSG00000105855:ENSG00000253293:ENSG00000005073:ENSG00000106031:ENSG00000106038:ENSG00000153814:ENSG00000152926:ENSG00000213462:ENSG00000169902:ENSG00000189143:ENSG00000172209:ENSG00000164949:ENSG00000080608:ENSG00000213694:ENSG00000127080:ENSG00000182752:ENSG00000167081:ENSG00000167103
DEG.up	Whole_Blood	2073	93	0.489704547545253	1	ENSG00000188672:ENSG00000162366:ENSG00000163131:ENSG00000163154:ENSG00000143622:ENSG00000196189:ENSG00000143226:ENSG00000173110:ENSG00000203747:ENSG00000132185:ENSG00000198734:ENSG00000214688:ENSG00000107738:ENSG00000175567:ENSG00000062282:ENSG00000110777:ENSG00000109943:ENSG00000111679:ENSG00000123338:ENSG00000120868:ENSG00000136167:ENSG00000102445:ENSG00000152213:ENSG00000102524:ENSG00000156414:ENSG00000102879:ENSG00000180096:ENSG00000102921:ENSG00000185669:ENSG00000158717:ENSG00000132514:ENSG00000161944:ENSG00000109047:ENSG00000161405:ENSG00000225190:ENSG00000198933:ENSG00000073861:ENSG00000121060:ENSG00000123700:ENSG00000154889:ENSG00000205744:ENSG00000263264:ENSG00000180739:ENSG00000171136:ENSG00000123146:ENSG00000096996:ENSG00000099308:ENSG00000105369:ENSG00000028277:ENSG00000160570:ENSG00000079385:ENSG00000105483:ENSG00000105383:ENSG00000171049:ENSG00000115756:ENSG00000197872:ENSG00000163803:ENSG00000115604:ENSG00000115607:ENSG00000018280:ENSG00000196209:ENSG00000124102:ENSG00000124256:ENSG00000203896:ENSG00000184792:ENSG00000179750:ENSG00000130487:ENSG00000169704:ENSG00000172780:ENSG00000172578:ENSG00000169429:ENSG00000109272:ENSG00000145287:ENSG00000171522:ENSG00000132357:ENSG00000120318:ENSG00000135074:ENSG00000146112:ENSG00000137404:ENSG00000235863:ENSG00000255587:ENSG00000026297:ENSG00000086730:ENSG00000085514:ENSG00000197993:ENSG00000221937:ENSG00000268170:ENSG00000181444:ENSG00000196329:ENSG00000136573:ENSG00000254087:ENSG00000165071:ENSG00000198113
DEG.down	Adipose_Subcutaneous	1541	99	0.000170692890953188	0.00921741611147217	ENSG00000158286:ENSG00000097021:ENSG00000160050:ENSG00000168389:ENSG00000117472:ENSG00000162366:ENSG00000117155:ENSG00000153904:ENSG00000134215:ENSG00000168679:ENSG00000175984:ENSG00000092621:ENSG00000143412:ENSG00000143630:ENSG00000132718:ENSG00000196189:ENSG00000242252:ENSG00000160781:ENSG00000163472:ENSG00000075073:ENSG00000120057:ENSG00000184545:ENSG00000268403:ENSG00000187486:ENSG00000124935:ENSG00000146670:ENSG00000175564:ENSG00000154127:ENSG00000010219:ENSG00000010626:ENSG00000139410:ENSG00000122966:ENSG00000184992:ENSG00000127863:ENSG00000102743:ENSG00000136153:ENSG00000139914:ENSG00000170113:ENSG00000186198:ENSG00000103056:ENSG00000185669:ENSG00000188522:ENSG00000108641:ENSG00000128482:ENSG00000142494:ENSG00000067191:ENSG00000141750:ENSG00000108830:ENSG00000073670:ENSG00000197971:ENSG00000132000:ENSG00000099308:ENSG00000262484:ENSG00000179751:ENSG00000105369:ENSG00000028277:ENSG00000079462:ENSG00000105427:ENSG00000079385:ENSG00000196208:ENSG00000163803:ENSG00000114993:ENSG00000163040:ENSG00000144485:ENSG00000124102:ENSG00000203896:ENSG00000184792:ENSG00000100321:ENSG00000100197:ENSG00000138944:ENSG00000075275:ENSG00000205593:ENSG00000008735:ENSG00000144712:ENSG00000163517:ENSG00000007402:ENSG00000114115:ENSG00000114279:ENSG00000080493:ENSG00000163739:ENSG00000215218:ENSG00000164620:ENSG00000135074:ENSG00000244731:ENSG00000124772:ENSG00000137168:ENSG00000135525:ENSG00000105855:ENSG00000189143:ENSG00000177679:ENSG00000085563:ENSG00000185955:ENSG00000166924:ENSG00000159763:ENSG00000104267:ENSG00000127080:ENSG00000070159:ENSG00000167106:ENSG00000170835
DEG.down	Adipose_Visceral_Omentum	1661	103	0.000448086016617826	0.0241966448973626	ENSG00000097021:ENSG00000160050:ENSG00000160051:ENSG00000116525:ENSG00000117399:ENSG00000117472:ENSG00000134709:ENSG00000117155:ENSG00000153904:ENSG00000134215:ENSG00000175984:ENSG00000143630:ENSG00000132718:ENSG00000196189:ENSG00000160781:ENSG00000163472:ENSG00000120057:ENSG00000203867:ENSG00000268403:ENSG00000187486:ENSG00000229859:ENSG00000146670:ENSG00000175564:ENSG00000149256:ENSG00000149292:ENSG00000180425:ENSG00000023171:ENSG00000010626:ENSG00000111674:ENSG00000139410:ENSG00000122966:ENSG00000184992:ENSG00000127863:ENSG00000102743:ENSG00000136153:ENSG00000139914:ENSG00000180998:ENSG00000170113:ENSG00000137802:ENSG00000186198:ENSG00000185418:ENSG00000180209:ENSG00000180035:ENSG00000103067:ENSG00000185669:ENSG00000181350:ENSG00000188522:ENSG00000108641:ENSG00000128482:ENSG00000142494:ENSG00000067191:ENSG00000141750:ENSG00000108830:ENSG00000012048:ENSG00000073670:ENSG00000141540:ENSG00000141401:ENSG00000081913:ENSG00000197971:ENSG00000167670:ENSG00000104880:ENSG00000180739:ENSG00000129354:ENSG00000132000:ENSG00000099308:ENSG00000028277:ENSG00000079462:ENSG00000105427:ENSG00000185453:ENSG00000196208:ENSG00000188687:ENSG00000144218:ENSG00000163040:ENSG00000144485:ENSG00000124102:ENSG00000160190:ENSG00000183628:ENSG00000184792:ENSG00000100321:ENSG00000100197:ENSG00000138944:ENSG00000075275:ENSG00000205593:ENSG00000008735:ENSG00000144712:ENSG00000163517:ENSG00000007402:ENSG00000114279:ENSG00000215218:ENSG00000064651:ENSG00000164620:ENSG00000155858:ENSG00000112812:ENSG00000135525:ENSG00000184786:ENSG00000253293:ENSG00000005073:ENSG00000185955:ENSG00000154319:ENSG00000147465:ENSG00000104267:ENSG00000070159:ENSG00000167106
DEG.down	Adrenal_Gland	3492	198	0.000206964906865988	0.0111761049707633	ENSG00000158292:ENSG00000097021:ENSG00000065526:ENSG00000158747:ENSG00000187942:ENSG00000127603:ENSG00000117399:ENSG00000162366:ENSG00000117155:ENSG00000117569:ENSG00000134215:ENSG00000116396:ENSG00000168679:ENSG00000085465:ENSG00000092621:ENSG00000143387:ENSG00000143412:ENSG00000143409:ENSG00000143434:ENSG00000163354:ENSG00000132718:ENSG00000116584:ENSG00000196189:ENSG00000160781:ENSG000001

						74175:ENSG00000143322:ENSG00000162714:ENSG00000148468:ENSG00000107736:ENSG00000107738:ENSG00000184545:ENSG00000110169:ENSG00000166478:ENSG00000166483:ENSG00000165905:ENSG00000162174:ENSG00000168060:ENSG00000146670:ENSG00000175564:ENSG00000062282:ENSG00000154127:ENSG00000166250:ENSG00000013588:ENSG00000134531:ENSG00000123360:ENSG00000120868:ENSG00000235162:ENSG00000074590:ENSG00000184992:ENSG00000177084:ENSG00000127863:ENSG00000133110:ENSG00000102743:ENSG00000102445:ENSG00000102753:ENSG00000136153:ENSG00000178695:ENSG00000092140:ENSG00000100473:ENSG00000203546:ENSG00000179476:ENSG00000139926:ENSG00000087301:ENSG00000197930:ENSG00000006432:ENSG00000100767:ENSG00000156414:ENSG00000170113:ENSG00000243708:ENSG00000243789:ENSG00000186198:ENSG00000103710:ENSG00000140350:ENSG00000103888:ENSG00000140511:ENSG00000185418:ENSG00000008710:ENSG00000157045:ENSG00000157106:ENSG00000180096:ENSG00000102921:ENSG00000072736:ENSG00000103056:ENSG00000188522:ENSG00000072134:ENSG00000128482:ENSG00000067191:ENSG00000008294:ENSG00000011258:ENSG00000153933:ENSG00000108423:ENSG00000154217:ENSG00000141540:ENSG00000141576:ENSG00000185262:ENSG00000078687:ENSG00000141577:ENSG00000017797:ENSG00000141401:ENSG00000197971:ENSG00000071564:ENSG00000205744:ENSG00000065989:ENSG00000132017:ENSG00000099308:ENSG00000269190:ENSG00000028277:ENSG00000167619:ENSG00000105427:ENSG00000185453:ENSG00000063127:ENSG00000176293:ENSG00000143867:ENSG00000162929:ENSG00000065911:ENSG00000114993:ENSG00000144218:ENSG00000115604:ENSG00000144283:ENSG00000153250:ENSG00000118260:ENSG00000186235:ENSG00000132326:ENSG00000101290:ENSG00000188559:ENSG00000125966:ENSG00000124107:ENSG00000203896:ENSG00000155313:ENSG00000183628:ENSG00000177096:ENSG00000100197:ENSG00000025770:ENSG00000177989:ENSG00000205560:ENSG00000251322:ENSG00000144712:ENSG00000163517:ENSG00000182606:ENSG00000182983:ENSG00000243477:ENSG00000068028:ENSG00000144749:ENSG00000114861:ENSG00000175105:ENSG00000184500:ENSG00000169379:ENSG00000129048:ENSG00000154917:ENSG00000158163:ENSG00000155903:ENSG00000169282:ENSG00000145014:ENSG00000109171:ENSG00000163739:ENSG00000109321:ENSG00000215218:ENSG00000152669:ENSG00000068796:ENSG00000181751:ENSG00000197948:ENSG00000135074:ENSG00000074317:ENSG00000196670:ENSG00000137266:ENSG00000078401:ENSG00000112812:ENSG00000137337:ENSG00000164530:ENSG00000112406:ENSG00000120278:ENSG00000131018:ENSG00000026297:ENSG00000186340:ENSG00000122644:ENSG00000105855:ENSG00000005073:ENSG00000049541:ENSG00000135218:ENSG00000078487:ENSG00000166924:ENSG00000075790:ENSG00000146966:ENSG00000159763:ENSG00000146918:ENSG00000168546:ENSG00000120910:ENSG00000121039:ENSG00000104267:ENSG00000164949:ENSG00000131773:ENSG00000180900:ENSG00000196378:ENSG00000130958:ENSG00000136826:ENSG00000070159:ENSG00000130560:ENSG00000198113
DEG. down	Artery_Aorta	1473	103	4.22731108538199e-06	0.000228274798610627	ENSG00000158292:ENSG00000160051:ENSG00000168389:ENSG00000117399:ENSG00000117472:ENSG00000162367:ENSG00000197147:ENSG00000134215:ENSG00000175984:ENSG00000143412:ENSG00000143434:ENSG00000163354:ENSG00000143630:ENSG00000196189:ENSG00000160781:ENSG00000163472:ENSG00000163050:ENSG00000198879:ENSG00000134463:ENSG00000148468:ENSG00000075073:ENSG00000107736:ENSG00000138134:ENSG00000120057:ENSG00000187486:ENSG00000162174:ENSG00000146670:ENSG00000149809:ENSG00000173599:ENSG00000175564:ENSG00000062282:ENSG00000154127:ENSG00000064309:ENSG00000127863:ENSG00000102743:ENSG00000100767:ENSG00000156414:ENSG00000140600:ENSG00000180209:ENSG00000103056:ENSG00000172137:ENSG00000185669:ENSG00000196689:ENSG00000108641:ENSG00000108830:ENSG00000012048:ENSG00000073670:ENSG00000154217:ENSG00000141401:ENSG00000168234:ENSG00000081913:ENSG00000197971:ENSG00000167670:ENSG00000205744:ENSG00000104883:ENSG00000132016:ENSG00000105369:ENSG00000028277:ENSG00000079462:ENSG00000105427:ENSG00000079435:ENSG00000116039:ENSG00000114993:ENSG00000188559:ENSG00000124102:ENSG00000160190:ENSG00000183628:ENSG00000100321:ENSG00000100197:ENSG00000138944:ENSG00000205593:ENSG00000205560:ENSG00000251322:ENSG00000170293:ENSG00000170011:ENSG00000179564:ENSG00000114378:ENSG00000163762:ENSG00000163898:ENSG00000163975:ENSG00000080493:ENSG00000163739:ENSG00000175471:ENSG00000181751:ENSG00000064651:ENSG00000164620:ENSG00000137266:ENSG00000124772:ENSG00000243955:ENSG00000135525:ENSG00000122644:ENSG00000105855:ENSG00000135218:ENSG00000085563:ENSG00000185955:ENSG00000005249:ENSG00000106484:ENSG00000146966:ENSG00000181444:ENSG00000147465:ENSG00000104267:ENSG00000129295:ENSG00000127080
DEG. down	Artery_Coronary	954	66	0.000299198836330637	0.0161567371618544	ENSG00000168389:ENSG00000117399:ENSG00000117472:ENSG00000134215:ENSG00000175984:ENSG00000143412:ENSG00000143630:ENSG00000196189:ENSG00000160781:ENSG00000163472:ENSG00000075073:ENSG00000187486:ENSG00000162174:ENSG00000146670:ENSG00000149809:ENSG00000175564:ENSG00000122966:ENSG00000184992:ENSG00000139914:ENSG00000168952:ENSG00000100473:ENSG00000103056:ENSG00000181350:ENSG00000108830:ENSG00000073670:ENSG00000123700:ENSG00000141576:ENSG00000141401:ENSG00000168234:ENSG00000081913:ENSG00000197971:ENSG00000167670:ENSG00000104833:ENSG00000132016:ENSG00000079462:ENSG00000105427:ENSG00000079385:ENSG00000186235:ENSG00000124102:ENSG00000183628:ENSG00000100321:ENSG00000100197:ENSG00000138944:ENSG00000075275:ENSG00000205593:ENSG00000205560:ENSG00000168032:ENSG00000007402:ENSG00000163898:ENSG00000114279:ENSG00000080493:ENSG00000215218:ENSG00000181751:ENSG00000064651:ENSG00000164620:ENSG00000137266:ENSG00000124772:ENSG00000132429:ENSG00000135525:ENSG00000177679:ENSG00000085563:ENSG00000185955:ENSG00000166924:ENSG00000168546:ENSG00000104267:ENSG00000127080

DEG. down	Artery_Tibial	2044	135	2.78043461556427e-06	0.000150143469240471	ENSG00000158286:ENSG00000158292:ENSG00000134668:ENSG00000160050:ENSG00000160051:ENSG00000117472:ENSG00000162366:ENSG00000162367:ENSG00000117155:ENSG00000197147:ENSG00000175984:ENSG00000143412:ENSG00000143434:ENSG00000163154:ENSG00000163157:ENSG00000163354:ENSG00000143630:ENSG00000196189:ENSG00000160781:ENSG00000163472:ENSG00000173110:ENSG00000198879:ENSG00000134463:ENSG00000148468:ENSG00000241058:ENSG00000107736:ENSG00000138134:ENSG00000152779:ENSG00000120057:ENSG00000203867:ENSG00000119927:ENSG00000187486:ENSG00000162174:ENSG00000149809:ENSG00000173599:ENSG00000175567:ENSG00000175564:ENSG00000062282:ENSG00000137672:ENSG00000154127:ENSG00000166250:ENSG00000064309:ENSG00000111679:ENSG00000135094:ENSG00000139410:ENSG00000188735:ENSG00000184992:ENSG00000127863:ENSG00000102743:ENSG00000102445:ENSG00000168952:ENSG00000100767:ENSG00000243789:ENSG00000102879:ENSG00000180096:ENSG00000103056:ENSG00000181350:ENSG00000108641:ENSG00000108830:ENSG00000012048:ENSG00000073670:ENSG00000121060:ENSG00000154217:ENSG00000180616:ENSG00000141540:ENSG00000141401:ENSG00000168234:ENSG00000197971:ENSG00000205744:ENSG00000104835:ENSG00000179751:ENSG00000028277:ENSG00000079462:ENSG00000105427:ENSG00000079435:ENSG00000115756:ENSG00000197872:ENSG00000116039:ENSG00000005448:ENSG00000114993:ENSG00000115602:ENSG00000115604:ENSG00000163040:ENSG00000135912:ENSG00000196209:ENSG00000188559:ENSG00000124102:ENSG00000203896:ENSG00000160190:ENSG00000100321:ENSG00000138944:ENSG00000205593:ENSG00000130489:ENSG00000177989:ENSG00000205560:ENSG00000170293:ENSG00000183873:ENSG00000114378:ENSG00000114395:ENSG00000007402:ENSG00000114115:ENSG00000163762:ENSG00000163975:ENSG00000163739:ENSG00000185477:ENSG00000181751:ENSG00000168916:ENSG00000064651:ENSG00000164620:ENSG00000048140:ENSG00000137266:ENSG00000124772:ENSG00000132429:ENSG00000135525:ENSG00000120278:ENSG00000026297:ENSG00000122644:ENSG00000105855:ENSG00000146757:ENSG00000086730:ENSG00000177679:ENSG00000085563:ENSG00000005249:ENSG00000106484:ENSG00000146966:ENSG00000181444:ENSG00000168546:ENSG00000254087:ENSG00000121039:ENSG00000104267:ENSG00000129295:ENSG00000165071:ENSG00000127080:ENSG00000170835:ENSG00000198113
DEG. down	Bladder	105	10	0.0188290417476964	1	ENSG00000173599:ENSG00000122966:ENSG00000139914:ENSG00000142494:ENSG00000141750:ENSG00000007402:ENSG00000154917:ENSG00000181751:ENSG00000166924:ENSG00000104267
DEG. down	Brain_Amygdala	7751	517	9.91894849568672e-26	5.35623218767083e-24	ENSG00000158286:ENSG00000116237:ENSG00000171824:ENSG00000198793:ENSG00000120942:ENSG00000162458:ENSG00000065526:ENSG00000116809:ENSG00000117616:ENSG00000204178:ENSG00000157978:ENSG00000084652:ENSG00000160051:ENSG00000160094:ENSG00000134686:ENSG00000066056:ENSG00000159479:ENSG00000198198:ENSG00000162367:ENSG00000117862:ENSG00000198841:ENSG00000162390:ENSG00000134709:ENSG00000162604:ENSG00000162616:ENSG00000117114:ENSG00000117133:ENSG00000142867:ENSG00000171488:ENSG00000117528:ENSG00000162694:ENSG00000117543:ENSG00000134215:ENSG00000162775:ENSG00000168679:ENSG00000085465:ENSG00000116455:ENSG00000163349:ENSG00000197323:ENSG00000213281:ENSG00000052723:ENSG00000143457:ENSG00000163131:ENSG00000143387:ENSG00000143437:ENSG00000143379:ENSG00000143418:ENSG00000143412:ENSG00000143409:ENSG00000143363:ENSG00000143434:ENSG00000163156:ENSG00000163155:ENSG00000143537:ENSG00000160767:ENSG00000116521:ENSG00000176444:ENSG00000143630:ENSG00000160752:ENSG00000160753:ENSG00000116539:ENSG00000125459:ENSG00000163374:ENSG00000132676:ENSG00000116580:ENSG00000143622:ENSG00000132680:ENSG00000116584:ENSG00000160789:ENSG00000160783:ENSG00000163472:ENSG00000198715:ENSG00000187800:ENSG00000132694:ENSG00000143226:ENSG00000120341:ENSG00000186283:ENSG00000143322:ENSG00000057252:ENSG00000182004:ENSG00000143801:ENSG00000163050:ENSG00000162885:ENSG00000116962:ENSG00000162714:ENSG00000107929:ENSG00000134463:ENSG00000165997:ENSG00000108094:ENSG00000188234:ENSG00000165476:ENSG00000107736:ENSG00000107738:ENSG00000138134:ENSG00000107796:ENSG00000026103:ENSG00000165338:ENSG00000059573:ENSG00000155256:ENSG00000198873:ENSG00000166311:ENSG00000205339:ENSG00000166478:ENSG00000166483:ENSG00000198730:ENSG00000149150:ENSG00000162300:ENSG00000149823:ENSG00000174276:ENSG00000162298:ENSG00000149792:ENSG00000173653:ENSG00000175567:ENSG00000062282:ENSG00000137492:ENSG00000137693:ENSG00000137714:ENSG00000149292:ENSG00000076053:ENSG00000076043:ENSG00000082805:ENSG00000010219:ENSG00000139180:ENSG00000111671:ENSG00000111679:ENSG00000126749:ENSG00000111684:ENSG00000182326:ENSG00000134531:ENSG00000123094:ENSG00000110911:ENSG00000050426:ENSG00000123338:ENSG00000127314:ENSG00000166130:ENSG00000235162:ENSG00000074590:ENSG00000174989:ENSG00000111725:ENSG00000188735:ENSG00000150990:ENSG00000177169:ENSG00000177084:ENSG00000176915:ENSG00000127863:ENSG00000139505:ENSG00000102743:ENSG00000136167:ENSG00000102445:ENSG00000102531:ENSG00000123179:ENSG00000102753:ENSG00000136104:ENSG00000188243:ENSG00000136153:ENSG00000178695:ENSG00000088451:ENSG00000065150:ENSG00000134901:ENSG00000134897:ENSG00000139826:ENSG00000102524:ENSG00000100889:ENSG00000092140:ENSG00000092108:ENSG00000100473:ENSG00000150527:ENSG00000165355:ENSG00000179476:ENSG00000179454:ENSG00000185246:ENSG00000139926:ENSG00000197930:ENSG00000119599:ENSG00000165861:ENSG00000119707:ENSG00000100767:ENSG00000133961:ENSG00000165417:ENSG00000165934:ENSG00000153485:ENSG00000140157:ENSG00000128731:ENSG00000021776:ENSG00000198146:ENSG00000174197:ENSG00000137802:ENSG00000243708:ENSG00000243789:ENSG00000181827:ENSG00000140262:ENSG00000103707:ENSG00000186198:ENSG00000136378:ENSG00000103888:ENSG00000117899:ENSG00000140406:ENSG00000140511:ENSG00000140471:ENSG00000008710:ENSG00000103512:ENSG00000179889:ENSG00000085721:ENSG00000157106:ENSG00000180096:ENSG00000179958:ENSG00000140691:ENSG00000140682:ENSG00000197302:ENSG00000102910:ENSG00000196470:ENSG00000102921:ENSG00000005194:ENSG00000102978:

						ENSG00000072736:ENSG00000103066:ENSG00000103064:ENSG00000103061:ENSG00000132600:ENSG00000158717:ENSG00000167522:ENSG00000197912:ENSG00000127804:ENSG00000196689:ENSG00000197417:ENSG00000005100:ENSG00000072849:ENSG00000166484:ENSG00000166482:ENSG00000142494:ENSG00000068079:ENSG00000108828:ENSG00000012048:ENSG00000184988:ENSG00000182963:ENSG00000225190:ENSG00000141279:ENSG00000108424:ENSG00000011258:ENSG00000011260:ENSG00000153933:ENSG00000121060:ENSG00000108423:ENSG00000108443:ENSG00000189050:ENSG00000108878:ENSG00000198265:ENSG00000197170:ENSG00000185262:ENSG00000141577:ENSG00000167302:ENSG00000185504:ENSG00000182446:ENSG00000101608:ENSG00000017797:ENSG00000154845:ENSG00000154889:ENSG00000141401:ENSG00000168234:ENSG00000141384:ENSG00000152223:ENSG00000130856:ENSG00000071564:ENSG00000141985:ENSG00000167670:ENSG00000205744:ENSG00000171105:ENSG00000104880:ENSG00000167775:ENSG00000079999:ENSG00000104957:ENSG00000132017:ENSG00000132005:ENSG00000123146:ENSG00000123136:ENSG00000127527:ENSG00000237440:ENSG00000104835:ENSG00000128626:ENSG00000269190:ENSG00000028277:ENSG00000160570:ENSG00000167625:ENSG00000105722:ENSG00000105427:ENSG00000187244:ENSG00000105483:ENSG00000105447:ENSG00000142544:ENSG00000105383:ENSG00000196214:ENSG00000198464:ENSG00000167766:ENSG00000213020:ENSG00000115761:ENSG00000055917:ENSG00000171103:ENSG00000162928:ENSG00000082898:ENSG00000075292:ENSG00000144034:ENSG00000144048:ENSG00000114978:ENSG00000065911:ENSG00000115602:ENSG00000115604:ENSG00000153107:ENSG00000153208:ENSG00000153214:ENSG00000115963:ENSG00000153250:ENSG00000118260:ENSG00000197713:ENSG00000115457:ENSG00000115461:ENSG00000018280:ENSG00000144579:ENSG00000135912:ENSG00000132326:ENSG00000162804:ENSG00000196209:ENSG00000088876:ENSG00000125779:ENSG00000132646:ENSG00000188559:ENSG00000198959:ENSG00000124107:ENSG00000124228:ENSG00000124201:ENSG00000125520:ENSG00000198276:ENSG00000155313:ENSG00000157617:ENSG00000160190:ENSG00000100220:ENSG00000100342:ENSG00000198951:ENSG00000177096:ENSG00000100197:ENSG00000242247:ENSG00000077942:ENSG00000186951:ENSG00000205643:ENSG00000075234:ENSG00000100416:ENSG00000196576:ENSG00000100258:ENSG00000025770:ENSG00000130489:ENSG00000025708:ENSG00000177989:ENSG00000205560:ENSG00000100288:ENSG00000100299:ENSG00000134077:ENSG00000144560:ENSG00000144712:ENSG00000163513:ENSG00000170293:ENSG00000177873:ENSG00000182606:ENSG00000182983:ENSG00000214706:ENSG00000243477:ENSG00000114378:ENSG00000068001:ENSG00000068028:ENSG00000114388:ENSG00000114395:ENSG00000126062:ENSG00000007402:ENSG00000180376:ENSG00000163946:ENSG00000114861:ENSG00000144736:ENSG00000163320:ENSG00000175105:ENSG00000179021:ENSG00000184500:ENSG00000169379:ENSG00000114446:ENSG00000163607:ENSG00000163608:ENSG00000144843:ENSG00000082701:ENSG00000172780:ENSG00000240682:ENSG00000034533:ENSG00000240303:ENSG00000113971:ENSG00000081307:ENSG00000144867:ENSG00000138231:ENSG00000114098:ENSG00000175110:ENSG00000184432:ENSG00000155903:ENSG00000175066:ENSG00000114127:ENSG00000075420:ENSG00000161202:ENSG00000161204:ENSG00000145014:ENSG00000041802:ENSG00000163975:ENSG00000013288:ENSG00000109689:ENSG00000169019:ENSG00000145246:ENSG00000109171:ENSG00000169429:ENSG00000163739:ENSG00000173085:ENSG00000145331:ENSG00000205403:ENSG00000164128:ENSG00000129187:ENSG00000113360:ENSG00000164190:ENSG00000171522:ENSG00000067248:ENSG00000039123:ENSG00000086200:ENSG00000164331:ENSG00000164338:ENSG00000164180:ENSG00000173926:ENSG00000131504:ENSG00000171720:ENSG00000197948:ENSG00000120318:ENSG00000113721:ENSG00000135074:ENSG00000113272:ENSG00000048140:ENSG00000131446:ENSG00000196670:ENSG00000146112:ENSG00000137404:ENSG00000137337:ENSG00000137331:ENSG00000204344:ENSG00000223501:ENSG00000235863:ENSG00000227057:ENSG00000237441:ENSG00000231925:ENSG00000124762:ENSG00000198663:ENSG00000164530:ENSG00000112245:ENSG00000198945:ENSG00000112357:ENSG00000118503:ENSG00000112406:ENSG00000120278:ENSG00000131018:ENSG00000112096:ENSG00000213066:ENSG00000186340:ENSG00000184786:ENSG00000130023:ENSG00000122644:ENSG00000153814:ENSG00000168303:ENSG00000152926:ENSG00000213462:ENSG00000126522:ENSG00000049541:ENSG00000135218:ENSG00000127920:ENSG00000105829:ENSG00000121716:ENSG00000085514:ENSG00000146834:ENSG00000160813:ENSG00000075790:ENSG00000091704:ENSG00000106484:ENSG00000158623:ENSG00000131558:ENSG00000122786:ENSG00000182158:ENSG00000181444:ENSG00000196329:ENSG00000214106:ENSG00000146918:ENSG00000117868:ENSG00000168546:ENSG00000120910:ENSG00000129691:ENSG00000254087:ENSG00000035681:ENSG00000147601:ENSG00000121039:ENSG00000164949:ENSG00000120526:ENSG00000120533:ENSG00000196378:ENSG00000080608:ENSG00000213694:ENSG00000127080:ENSG00000130958:ENSG00000136925:ENSG00000136936:ENSG00000136826:ENSG00000070159:ENSG00000167081:ENSG00000196152:ENSG00000148356:ENSG00000136840:ENSG00000171169:ENSG00000148339:ENSG00000148335:ENSG00000148308:ENSG00000170835:ENSG00000198113
DEG.down	Brain_Anterior_cingulate_cortex_BA24	6725	432	1.64447101798448e-17	8.88014349711619e-16	ENSG00000158286:ENSG00000116237:ENSG00000198793:ENSG00000120942:ENSG00000162458:ENSG00000065526:ENSG00000116809:ENSG00000117616:ENSG00000204178:ENSG00000157978:ENSG00000084652:ENSG00000134686:ENSG00000066056:ENSG00000066322:ENSG00000159479:ENSG00000198198:ENSG00000162367:ENSG00000117862:ENSG00000198841:ENSG00000162390:ENSG00000162616:ENSG00000117114:ENSG00000117133:ENSG00000142867:ENSG00000171488:ENSG00000117528:ENSG00000117543:ENSG00000134215:ENSG00000162775:ENSG00000168679:ENSG00000116455:ENSG00000163349:ENSG00000197323:ENSG00000052723:ENSG00000143457:ENSG00000163131:ENSG00000143387:ENSG00000143437:ENSG00000143379:ENSG00000143418:ENSG00000143412:ENSG00000143409:ENSG00000143363:ENSG00000143434:ENSG00000163154:ENSG00000163156:ENSG00000143537:ENSG00000116521:ENSG00000176444:ENSG00000125459:ENSG00000163374:ENSG00000132676:ENSG00000116580:ENSG00000143622:ENSG00000132680:ENSG00000160789:ENSG00000160783:ENSG00000163472:ENSG00000198715:ENSG00000187800:ENSG00000143226:ENSG00000120341:ENSG00000186283:ENSG00000143322:ENSG00000057252:ENSG000001820

	04:ENSG00000163050:ENSG00000162885:ENSG00000116962:ENSG00000162714:ENSG00000107929:ENSG00000134463:ENSG00000165997:ENSG00000165476:ENSG00000075073:ENSG00000107736:ENSG00000107738:ENSG00000107796:ENSG00000026103:ENSG00000059573:ENSG00000198873:ENSG00000166311:ENSG00000166478:ENSG00000198730:ENSG00000149150:ENSG00000162300:ENSG00000174276:ENSG00000162298:ENSG00000149792:ENSG00000173653:ENSG00000175567:ENSG00000062282:ENSG00000137492:ENSG00000137672:ENSG00000137693:ENSG00000137714:ENSG00000149292:ENSG00000076053:ENSG00000076043:ENSG00000166250:ENSG00000082805:ENSG00000010219:ENSG00000111671:ENSG00000111679:ENSG00000126749:ENSG00000111684:ENSG00000182326:ENSG00000134531:ENSG00000123094:ENSG00000110911:ENSG00000123338:ENSG00000127314:ENSG00000139292:ENSG00000166130:ENSG00000235162:ENSG00000174989:ENSG00000111725:ENSG00000188735:ENSG00000150990:ENSG00000177084:ENSG00000176915:ENSG00000127863:ENSG00000102743:ENSG00000136167:ENSG00000102445:ENSG00000102531:ENSG00000123179:ENSG00000188243:ENSG00000118939:ENSG00000136153:ENSG00000178695:ENSG00000088451:ENSG00000134901:ENSG00000139826:ENSG00000102524:ENSG00000100889:ENSG00000092140:ENSG00000092108:ENSG00000129493:ENSG00000150527:ENSG00000179476:ENSG00000179454:ENSG00000185246:ENSG00000139926:ENSG00000197930:ENSG00000119599:ENSG00000165861:ENSG00000119707:ENSG00000080815:ENSG00000100767:ENSG00000133961:ENSG00000165934:ENSG00000153485:ENSG00000140157:ENSG00000021776:ENSG00000198146:ENSG00000174197:ENSG00000137802:ENSG00000243708:ENSG00000243789:ENSG00000181827:ENSG00000140262:ENSG00000103707:ENSG00000103710:ENSG00000117899:ENSG00000140406:ENSG00000140511:ENSG00000140471:ENSG00000179889:ENSG00000085721:ENSG00000157106:ENSG00000180096:ENSG00000140682:ENSG00000102910:ENSG00000196470:ENSG00000102921:ENSG00000072736:ENSG00000103066:ENSG00000103064:ENSG00000103061:ENSG00000158717:ENSG00000167522:ENSG00000127804:ENSG00000196689:ENSG00000197417:ENSG00000005100:ENSG00000072849:ENSG00000166484:ENSG00000166482:ENSG00000142494:ENSG00000068079:ENSG00000108828:ENSG00000182963:ENSG00000225190:ENSG00000011258:ENSG00000011260:ENSG00000121060:ENSG00000108423:ENSG00000108443:ENSG00000189050:ENSG00000198265:ENSG00000123700:ENSG00000185262:ENSG00000141577:ENSG00000167302:ENSG00000185504:ENSG00000182446:ENSG00000101608:ENSG00000154845:ENSG00000154889:ENSG00000141401:ENSG00000168234:ENSG00000152223:ENSG00000071564:ENSG00000141985:ENSG00000167670:ENSG00000205744:ENSG00000171105:ENSG00000104880:ENSG00000104883:ENSG00000167775:ENSG00000104957:ENSG00000132017:ENSG00000132005:ENSG00000123146:ENSG00000123136:ENSG00000128626:ENSG00000269190:ENSG00000028277:ENSG00000167625:ENSG00000105722:ENSG00000105427:ENSG00000079435:ENSG00000187244:ENSG00000105483:ENSG00000105447:ENSG00000142544:ENSG00000196214:ENSG00000198464:ENSG00000167766:ENSG00000213020:ENSG00000115761:ENSG00000163803:ENSG00000171103:ENSG00000162928:ENSG00000082898:ENSG00000075292:ENSG00000144034:ENSG00000144048:ENSG00000114978:ENSG00000065911:ENSG00000115602:ENSG00000153107:ENSG00000153214:ENSG00000115963:ENSG00000153250:ENSG00000118260:ENSG00000115457:ENSG00000115461:ENSG00000018280:ENSG00000144579:ENSG00000135912:ENSG00000132326:ENSG00000162804:ENSG00000088876:ENSG00000132646:ENSG00000089048:ENSG00000188559:ENSG00000198959:ENSG00000124107:ENSG00000124228:ENSG00000124201:ENSG00000125520:ENSG00000198276:ENSG00000155313:ENSG00000154642:ENSG00000157617:ENSG00000160190:ENSG00000100342:ENSG00000198951:ENSG00000177096:ENSG00000100197:ENSG00000242247:ENSG00000077942:ENSG00000186951:ENSG00000205643:ENSG00000075234:ENSG00000100416:ENSG00000196576:ENSG00000100258:ENSG00000130489:ENSG00000025708:ENSG00000177989:ENSG00000205560:ENSG00000100288:ENSG00000100299:ENSG00000070950:ENSG00000134077:ENSG00000144560:ENSG00000163513:ENSG00000170293:ENSG00000177873:ENSG00000182983:ENSG00000214706:ENSG00000114378:ENSG00000068001:ENSG00000068028:ENSG00000114395:ENSG00000126062:ENSG00000180376:ENSG00000163946:ENSG00000114861:ENSG00000144736:ENSG00000163320:ENSG00000175105:ENSG00000179021:ENSG00000184500:ENSG00000169379:ENSG00000114446:ENSG00000163607:ENSG00000163608:ENSG00000144843:ENSG00000172780:ENSG00000240682:ENSG00000034533:ENSG00000240303:ENSG00000113971:ENSG00000138231:ENSG00000114098:ENSG00000184432:ENSG00000155903:ENSG00000175066:ENSG00000114127:ENSG00000075420:ENSG00000161202:ENSG00000041802:ENSG00000013288:ENSG00000169019:ENSG00000145246:ENSG00000109171:ENSG00000169429:ENSG00000163739:ENSG00000173085:ENSG00000185477:ENSG00000145331:ENSG00000205403:ENSG00000164190:ENSG00000067248:ENSG00000039123:ENSG00000086200:ENSG00000164331:ENSG00000164338:ENSG00000164180:ENSG00000173926:ENSG00000064651:ENSG00000131504:ENSG00000171720:ENSG00000197948:ENSG00000120318:ENSG00000113721:ENSG00000135074:ENSG00000113272:ENSG00000131446:ENSG00000196670:ENSG00000078401:ENSG00000112812:ENSG00000146112:ENSG00000137404:ENSG00000137337:ENSG00000137331:ENSG00000204344:ENSG00000244731:ENSG00000223501:ENSG00000235863:ENSG00000227057:ENSG00000237441:ENSG00000124762:ENSG00000198663:ENSG00000164530:ENSG00000112245:ENSG00000198945:ENSG00000118503:ENSG00000112406:ENSG00000120278:ENSG00000186340:ENSG00000184786:ENSG00000130023:ENSG00000122644:ENSG00000152926:ENSG00000213462:ENSG00000126522:ENSG00000086730:ENSG00000049541:ENSG00000127920:ENSG00000105829:ENSG00000085514:ENSG00000078487:ENSG00000146834:ENSG00000160813:ENSG00000091704:ENSG00000131558:ENSG00000122786:ENSG00000182158:ENSG00000181444:ENSG00000196329:ENSG00000214106:ENSG00000146918:ENSG00000117868:ENSG00000120910:ENSG00000254087:ENSG00000035681:ENSG00000121039:ENSG00000164949:ENSG00000120526:ENSG00000196378:ENSG00000080608:ENSG00000213694:ENSG00000127080:ENSG00000130958:ENSG00000136925:ENSG00000136936:ENSG00000136826:ENSG00000070159:ENSG00000167081:ENSG00000196152:ENSG00000136840:ENSG00000171169:ENSG00000148339:ENSG00000148335:ENSG00000148308:ENSG00000170835:ENSG00000198113

DEG. down	Brain_Caudate_basal_ganglia	6927	458	2.76219234608686e-21	1.4915838668869e-19	ENSG00000158286:ENSG00000158292:ENSG00000171824:ENSG00000120942:ENSG00000162458:ENSG00000065526:ENSG00000116809:ENSG00000204178:ENSG00000157978:ENSG00000084652:ENSG00000116525:ENSG00000160094:ENSG00000134686:ENSG00000066056:ENSG00000066322:ENSG00000159479:ENSG00000198198:ENSG00000162367:ENSG00000117862:ENSG00000198841:ENSG00000117114:ENSG00000142867:ENSG00000171488:ENSG00000117528:ENSG00000117543:ENSG00000116396:ENSG00000162775:ENSG00000168679:ENSG00000116455:ENSG00000163349:ENSG00000197323:ENSG00000213281:ENSG00000052723:ENSG00000143457:ENSG00000163131:ENSG00000143387:ENSG00000143437:ENSG00000143379:ENSG00000143412:ENSG00000143409:ENSG00000143363:ENSG00000143434:ENSG00000163154:ENSG00000163156:ENSG00000143537:ENSG00000160767:ENSG00000116521:ENSG00000176444:ENSG00000143630:ENSG00000160752:ENSG00000160753:ENSG00000116539:ENSG00000125459:ENSG00000163374:ENSG00000132676:ENSG00000116580:ENSG00000143622:ENSG00000132680:ENSG00000160789:ENSG00000160783:ENSG00000163472:ENSG00000198715:ENSG00000187800:ENSG00000132694:ENSG00000143226:ENSG00000120341:ENSG00000186283:ENSG00000057252:ENSG00000174307:ENSG00000122176:ENSG00000182004:ENSG00000143801:ENSG00000163050:ENSG00000162885:ENSG00000116962:ENSG00000162714:ENSG00000107929:ENSG00000134463:ENSG00000165997:ENSG00000165476:ENSG00000107736:ENSG00000138134:ENSG00000107796:ENSG00000026103:ENSG00000165338:ENSG00000059573:ENSG00000203867:ENSG00000198873:ENSG00000166311:ENSG00000205339:ENSG00000166478:ENSG00000166483:ENSG00000198730:ENSG00000149150:ENSG00000162300:ENSG00000174276:ENSG00000162298:ENSG00000149792:ENSG00000173653:ENSG00000175567:ENSG00000175564:ENSG00000137492:ENSG00000137693:ENSG00000137714:ENSG00000149292:ENSG00000076053:ENSG00000076043:ENSG00000154127:ENSG00000064309:ENSG00000010219:ENSG00000111671:ENSG00000111679:ENSG00000126749:ENSG00000111684:ENSG00000182326:ENSG00000134531:ENSG00000123094:ENSG00000110911:ENSG00000123338:ENSG00000127314:ENSG00000166130:ENSG00000235162:ENSG00000074590:ENSG00000174989:ENSG00000111725:ENSG00000150990:ENSG00000177084:ENSG00000176915:ENSG00000127863:ENSG00000102743:ENSG00000136167:ENSG00000102445:ENSG00000102531:ENSG00000123179:ENSG00000102753:ENSG00000188243:ENSG00000178695:ENSG00000088451:ENSG00000065150:ENSG00000134901:ENSG00000139826:ENSG00000102524:ENSG00000100889:ENSG00000092108:ENSG00000150527:ENSG00000179476:ENSG00000179454:ENSG00000185246:ENSG00000139926:ENSG00000197930:ENSG00000119599:ENSG00000165861:ENSG00000119707:ENSG00000133961:ENSG00000165934:ENSG00000153485:ENSG00000140157:ENSG00000021776:ENSG00000198146:ENSG00000174197:ENSG00000137802:ENSG00000243708:ENSG00000243789:ENSG00000181827:ENSG00000140262:ENSG00000103707:ENSG00000117899:ENSG00000140511:ENSG00000140471:ENSG00000183426:ENSG00000179889:ENSG00000085721:ENSG00000157106:ENSG00000169221:ENSG00000180096:ENSG00000179958:ENSG00000140691:ENSG00000140682:ENSG00000197302:ENSG00000102910:ENSG00000196470:ENSG00000102921:ENSG00000005194:ENSG00000072736:ENSG00000103066:ENSG00000103064:ENSG00000103061:ENSG00000158717:ENSG00000167522:ENSG00000197912:ENSG00000127804:ENSG00000197417:ENSG00000005100:ENSG00000072849:ENSG00000166484:ENSG00000166482:ENSG00000142494:ENSG00000068079:ENSG00000012048:ENSG00000182963:ENSG00000225190:ENSG00000141279:ENSG00000108424:ENSG00000198933:ENSG00000011258:ENSG00000011260:ENSG00000214226:ENSG00000121060:ENSG00000108423:ENSG00000108443:ENSG00000189050:ENSG00000198265:ENSG00000197170:ENSG00000185262:ENSG00000141577:ENSG00000167302:ENSG00000185504:ENSG00000182446:ENSG00000101608:ENSG00000017797:ENSG00000154845:ENSG00000154889:ENSG00000141401:ENSG00000168234:ENSG00000141384:ENSG00000152223:ENSG00000071564:ENSG00000141985:ENSG00000167670:ENSG00000205744:ENSG00000171105:ENSG00000104880:ENSG00000104883:ENSG00000167775:ENSG00000079999:ENSG00000104957:ENSG00000132016:ENSG00000132024:ENSG00000132000:ENSG00000132017:ENSG00000132005:ENSG00000123146:ENSG00000123136:ENSG00000127527:ENSG00000104835:ENSG00000128626:ENSG00000269190:ENSG00000028277:ENSG00000160570:ENSG00000167625:ENSG00000105722:ENSG00000187244:ENSG00000105483:ENSG00000105447:ENSG00000063127:ENSG00000196214:ENSG00000198464:ENSG00000115756:ENSG00000115761:ENSG00000055917:ENSG00000163803:ENSG00000171103:ENSG00000162928:ENSG00000082898:ENSG00000144034:ENSG00000144048:ENSG00000114978:ENSG00000065911:ENSG00000115602:ENSG00000115604:ENSG00000153107:ENSG00000153214:ENSG00000115963:ENSG00000153250:ENSG00000118260:ENSG00000115457:ENSG00000115461:ENSG00000018280:ENSG00000144579:ENSG00000132326:ENSG00000162804:ENSG00000088876:ENSG00000125779:ENSG00000132646:ENSG00000188559:ENSG00000198959:ENSG00000124107:ENSG00000124228:ENSG00000124201:ENSG00000125520:ENSG00000198276:ENSG00000155313:ENSG00000157617:ENSG00000160190:ENSG00000100220:ENSG00000128335:ENSG00000100342:ENSG00000198951:ENSG00000177096:ENSG00000242247:ENSG00000077942:ENSG00000205643:ENSG00000075234:ENSG00000100416:ENSG00000196576:ENSG00000100258:ENSG00000025770:ENSG00000130489:ENSG00000025708:ENSG00000177989:ENSG00000205560:ENSG00000100288:ENSG00000100299:ENSG00000070950:ENSG00000134077:ENSG00000144560:ENSG00000163513:ENSG00000170293:ENSG00000177873:ENSG00000182606:ENSG00000182983:ENSG00000214706:ENSG00000114378:ENSG00000068001:ENSG00000068028:ENSG00000114395:ENSG00000126062:ENSG00000180376:ENSG00000163946:ENSG00000144741:ENSG00000144736:ENSG00000163320:ENSG00000175105:ENSG00000179021:ENSG00000184500:ENSG00000114446:ENSG00000163607:ENSG00000163608:ENSG00000144843:ENSG00000172780:ENSG00000240682:ENSG00000034533:ENSG00000240303:ENSG00000113971:ENSG00000081307:ENSG00000144867:ENSG00000158163:ENSG00000138231:ENSG00000114098:ENSG00000175110:ENSG00000184432:ENSG00000155903:ENSG00000175066:ENSG00000114127:ENSG00000114698:ENSG00000075420:ENSG00000161202:ENSG00000161204:ENSG00000041802:ENSG00000163975:ENSG00000145246:ENSG00000109171:ENSG00000169429:ENSG00000163739:ENSG00000173085:ENSG00000205403:ENSG00000129187:ENSG00000164190:ENSG00000132357:ENSG00000067248:ENSG0

						0000039123:ENSG00000086200:ENSG00000164331:ENSG00000164338:ENSG00000164180:ENSG00000173926:ENSG00000131504:ENSG00000171720:ENSG00000197948:ENSG00000120318:ENSG00000113721:ENSG00000135074:ENSG00000113272:ENSG00000155858:ENSG00000048140:ENSG00000131446:ENSG00000196670:ENSG00000146112:ENSG00000137404:ENSG00000137337:ENSG00000137331:ENSG00000204344:ENSG00000244731:ENSG00000223501:ENSG00000235863:ENSG00000227057:ENSG00000124762:ENSG00000198663:ENSG00000164530:ENSG00000112245:ENSG00000198945:ENSG00000118503:ENSG00000112406:ENSG00000120278:ENSG00000112096:ENSG00000186340:ENSG00000184786:ENSG00000130023:ENSG00000122644:ENSG00000153814:ENSG00000168303:ENSG00000152926:ENSG00000213462:ENSG00000126522:ENSG00000086730:ENSG00000049541:ENSG00000135218:ENSG00000127920:ENSG00000105829:ENSG00000085514:ENSG00000146834:ENSG00000160813:ENSG00000196937:ENSG00000091704:ENSG00000158623:ENSG00000131558:ENSG00000122786:ENSG00000182158:ENSG00000196329:ENSG00000146918:ENSG00000117868:ENSG00000168546:ENSG00000120910:ENSG00000129691:ENSG00000254087:ENSG00000035681:ENSG00000121039:ENSG00000164949:ENSG00000120526:ENSG00000120533:ENSG00000180900:ENSG00000196378:ENSG00000080608:ENSG00000044459:ENSG00000213694:ENSG00000127080:ENSG00000130958:ENSG00000136925:ENSG00000136936:ENSG00000136826:ENSG00000196152:ENSG00000148356:ENSG00000136840:ENSG00000171169:ENSG00000148335:ENSG00000148308:ENSG00000170835:ENSG00000198113
DEG. down	Brain_Cerebellar_Hemisphere	2646	176	4.41771595188308e-08	2.38556661401687e-06	ENSG00000158292:ENSG00000179178:ENSG00000066056:ENSG00000066322:ENSG00000162616:ENSG00000117114:ENSG00000117133:ENSG00000142867:ENSG00000197147:ENSG00000117525:ENSG00000175984:ENSG00000163131:ENSG00000143387:ENSG00000163154:ENSG00000143622:ENSG00000160789:ENSG00000196189:ENSG00000198715:ENSG00000187800:ENSG00000143226:ENSG00000203747:ENSG00000186283:ENSG00000057252:ENSG00000174307:ENSG00000116962:ENSG00000134463:ENSG00000095794:ENSG00000075073:ENSG00000107738:ENSG00000107796:ENSG00000026103:ENSG00000166483:ENSG00000149150:ENSG00000168060:ENSG00000146670:ENSG00000175567:ENSG00000137693:ENSG00000137714:ENSG00000149292:ENSG00000076043:ENSG00000111679:ENSG00000182326:ENSG00000134531:ENSG00000127314:ENSG00000235162:ENSG00000139410:ENSG00000150990:ENSG00000127863:ENSG00000102743:ENSG00000136167:ENSG00000102445:ENSG00000123179:ENSG00000136153:ENSG00000102524:ENSG00000100889:ENSG00000168952:ENSG00000129493:ENSG00000150527:ENSG00000119599:ENSG00000100767:ENSG00000103710:ENSG00000157045:ENSG00000140682:ENSG00000181350:ENSG00000166484:ENSG00000166482:ENSG00000142494:ENSG00000068079:ENSG00000108828:ENSG00000196169:ENSG00000185262:ENSG00000101608:ENSG00000168234:ENSG00000141469:ENSG00000071564:ENSG00000205744:ENSG00000104883:ENSG00000180739:ENSG00000123146:ENSG00000099308:ENSG00000105722:ENSG00000105427:ENSG00000187244:ENSG00000105483:ENSG00000114978:ENSG00000065911:ENSG00000114993:ENSG00000115602:ENSG00000153208:ENSG00000153214:ENSG00000163040:ENSG00000115963:ENSG00000153250:ENSG00000115457:ENSG00000018280:ENSG00000144579:ENSG00000198959:ENSG00000124107:ENSG00000124201:ENSG00000155313:ENSG00000154642:ENSG00000157617:ENSG00000100342:ENSG00000198951:ENSG00000077942:ENSG00000186951:ENSG00000075234:ENSG00000025708:ENSG00000144560:ENSG00000163513:ENSG00000170293:ENSG00000214706:ENSG00000114378:ENSG00000068001:ENSG00000114395:ENSG00000114861:ENSG00000184500:ENSG00000114446:ENSG00000144843:ENSG00000114670:ENSG00000113971:ENSG00000144867:ENSG00000114698:ENSG00000163762:ENSG00000075420:ENSG00000145014:ENSG00000013288:ENSG00000157765:ENSG00000145246:ENSG00000109171:ENSG00000169429:ENSG00000163739:ENSG00000173085:ENSG00000205403:ENSG00000164128:ENSG00000129187:ENSG00000067113:ENSG00000173926:ENSG00000197948:ENSG00000113721:ENSG00000113272:ENSG00000131446:ENSG00000124787:ENSG00000078401:ENSG00000146112:ENSG00000137404:ENSG00000137331:ENSG00000244731:ENSG00000235863:ENSG00000124762:ENSG00000164530:ENSG00000198945:ENSG00000112406:ENSG00000120278:ENSG00000026297:ENSG00000186340:ENSG00000122644:ENSG00000105855:ENSG00000152926:ENSG00000126522:ENSG00000189143:ENSG00000127920:ENSG00000091704:ENSG00000106484:ENSG00000182158:ENSG00000181444:ENSG00000196329:ENSG00000168546:ENSG00000121039:ENSG00000164949:ENSG00000129295:ENSG00000080608:ENSG00000130958:ENSG00000136826:ENSG00000070159:ENSG00000167081
DEG. down	Brain_Cerebellum	2778	186	1.05849719594324e-08	5.71588485809348e-07	ENSG00000158292:ENSG00000162458:ENSG00000066056:ENSG00000162367:ENSG00000162616:ENSG00000117114:ENSG00000117133:ENSG00000142867:ENSG00000197147:ENSG00000117525:ENSG00000134215:ENSG00000175984:ENSG00000143457:ENSG00000163131:ENSG00000143387:ENSG00000163154:ENSG00000143622:ENSG00000160789:ENSG00000196189:ENSG00000198715:ENSG00000187800:ENSG00000143226:ENSG00000173110:ENSG00000203747:ENSG00000186283:ENSG00000057252:ENSG00000174307:ENSG00000122176:ENSG00000116962:ENSG00000134463:ENSG00000095794:ENSG00000075073:ENSG00000107738:ENSG00000107796:ENSG00000026103:ENSG00000166483:ENSG00000149150:ENSG00000168060:ENSG00000146670:ENSG00000175567:ENSG00000137693:ENSG00000137714:ENSG00000149292:ENSG00000076043:ENSG00000111679:ENSG00000182326:ENSG00000134531:ENSG00000134532:ENSG00000127314:ENSG00000235162:ENSG00000139410:ENSG00000150990:ENSG00000127863:ENSG00000102743:ENSG00000136167:ENSG00000123179:ENSG00000188243:ENSG00000118939:ENSG00000136153:ENSG00000100889:ENSG00000168952:ENSG00000100473:ENSG00000129493:ENSG00000150527:ENSG00000119599:ENSG00000100767:ENSG00000103710:ENSG00000136378:ENSG00000117899:ENSG00000140511:ENSG00000157045:ENSG00000180209:ENSG00000140682:ENSG00000103067:ENSG00000103018:ENSG00000181350:ENSG00000166482:ENSG00000142494:ENSG00000068079:ENSG00000108828:ENSG00000101608:ENSG00000168234:ENSG00000141469:ENSG00000205744:ENSG00000104883:ENSG00000180739:ENSG00000123146:ENSG00000099308:ENSG00000104825:ENSG00000105722:ENSG00000105427:ENSG00000187244:ENSG00000105483:ENSG00000114978:ENSG00000065911:ENSG00000115602:ENSG00000153208:ENSG00000153214:ENSG00000163

						040:ENSG00000115963:ENSG00000153250:ENSG00000115457:ENSG00000018280:ENSG00000144579:ENSG00000198959:ENSG00000124107:ENSG00000155313:ENSG00000154642:ENSG00000157617:ENSG00000100342:ENSG00000198951:ENSG00000177096:ENSG00000077942:ENSG00000186951:ENSG00000075234:ENSG00000025708:ENSG00000144560:ENSG00000163513:ENSG00000170293:ENSG00000114378:ENSG00000068001:ENSG00000114395:ENSG00000114861:ENSG00000184500:ENSG00000114446:ENSG00000144843:ENSG00000113971:ENSG00000144867:ENSG00000114698:ENSG00000163762:ENSG00000075420:ENSG00000145014:ENSG00000013288:ENSG00000157765:ENSG00000145246:ENSG00000109171:ENSG00000169429:ENSG00000163739:ENSG00000173085:ENSG00000205403:ENSG00000164128:ENSG00000129187:ENSG00000132357:ENSG00000067113:ENSG00000173926:ENSG00000197948:ENSG00000113721:ENSG00000113272:ENSG00000131446:ENSG00000124787:ENSG00000078401:ENSG00000146112:ENSG00000137404:ENSG00000137331:ENSG00000244731:ENSG00000235863:ENSG00000124762:ENSG00000164530:ENSG00000112245:ENSG00000198945:ENSG00000118503:ENSG00000112406:ENSG00000120278:ENSG00000112096:ENSG00000026297:ENSG00000186340:ENSG00000122644:ENSG00000126522:ENSG00000189143:ENSG00000086730:ENSG00000127920:ENSG00000106484:ENSG00000182158:ENSG00000159763:ENSG00000181444:ENSG00000196329:ENSG00000121039:ENSG00000164949:ENSG00000129295:ENSG00000080608:ENSG00000178031:ENSG00000213694:ENSG00000130958:ENSG00000136826:ENSG00000070159:ENSG00000167081
DEG. down	Brain_Cortex	5617	346	3.26584098247331e-11	1.76355413053559e-09	ENSG00000116237:ENSG00000120942:ENSG00000162458:ENSG00000065526:ENSG00000157978:ENSG00000084652:ENSG00000066056:ENSG00000066322:ENSG00000198198:ENSG00000162367:ENSG00000117862:ENSG00000162616:ENSG00000117133:ENSG00000142867:ENSG00000171488:ENSG00000117528:ENSG00000117543:ENSG00000134215:ENSG00000162775:ENSG00000168679:ENSG00000116455:ENSG00000163349:ENSG00000197323:ENSG00000213281:ENSG00000052723:ENSG00000143457:ENSG00000163131:ENSG00000143387:ENSG00000143437:ENSG00000143379:ENSG00000143418:ENSG00000143412:ENSG00000143409:ENSG00000143363:ENSG00000163154:ENSG00000176444:ENSG00000163374:ENSG00000132676:ENSG00000116580:ENSG00000143622:ENSG00000132680:ENSG00000160789:ENSG00000160783:ENSG00000163472:ENSG00000198715:ENSG00000187800:ENSG00000143226:ENSG00000173110:ENSG00000203747:ENSG00000186283:ENSG00000143322:ENSG00000057252:ENSG00000122176:ENSG00000182004:ENSG00000163050:ENSG00000162885:ENSG00000116962:ENSG00000107929:ENSG00000134463:ENSG00000165997:ENSG00000165476:ENSG00000075073:ENSG00000107736:ENSG00000107738:ENSG00000107796:ENSG00000026103:ENSG00000059573:ENSG00000120057:ENSG00000198873:ENSG00000205339:ENSG00000166478:ENSG00000198730:ENSG00000149150:ENSG00000162298:ENSG00000175567:ENSG00000175564:ENSG00000062282:ENSG00000137492:ENSG00000137693:ENSG00000137714:ENSG00000149292:ENSG00000076053:ENSG00000076043:ENSG00000166250:ENSG00000064309:ENSG00000082805:ENSG00000111671:ENSG00000111679:ENSG00000126749:ENSG00000111684:ENSG00000182326:ENSG00000134531:ENSG00000123094:ENSG00000123338:ENSG00000127314:ENSG00000235162:ENSG00000174989:ENSG00000111725:ENSG00000150990:ENSG00000177084:ENSG00000176915:ENSG00000102743:ENSG00000136167:ENSG00000102531:ENSG00000123179:ENSG00000188243:ENSG00000118939:ENSG00000136153:ENSG00000178695:ENSG00000088451:ENSG00000134901:ENSG00000139826:ENSG00000102524:ENSG00000100889:ENSG00000092140:ENSG00000100473:ENSG00000129493:ENSG00000150527:ENSG00000179476:ENSG00000179454:ENSG00000185246:ENSG00000139926:ENSG00000197930:ENSG00000119707:ENSG00000133961:ENSG00000165417:ENSG00000165934:ENSG00000153485:ENSG00000021776:ENSG00000198146:ENSG00000174197:ENSG00000243789:ENSG00000181827:ENSG00000140262:ENSG00000103710:ENSG00000117899:ENSG00000140406:ENSG00000140511:ENSG00000140471:ENSG00000179889:ENSG00000085721:ENSG00000157106:ENSG00000180096:ENSG00000140682:ENSG00000196470:ENSG00000102921:ENSG00000072736:ENSG00000103064:ENSG00000158717:ENSG00000127804:ENSG00000197417:ENSG00000072849:ENSG00000166484:ENSG00000166482:ENSG00000142494:ENSG00000068079:ENSG00000108828:ENSG00000182963:ENSG00000225190:ENSG00000011258:ENSG00000121060:ENSG00000108423:ENSG00000108443:ENSG00000189050:ENSG00000198265:ENSG00000185262:ENSG00000141577:ENSG00000167302:ENSG00000185504:ENSG00000182446:ENSG00000101608:ENSG00000154845:ENSG00000154889:ENSG00000141401:ENSG00000168234:ENSG00000152223:ENSG00000071564:ENSG00000205744:ENSG00000171105:ENSG00000104883:ENSG00000104957:ENSG00000132000:ENSG00000132017:ENSG00000132005:ENSG00000123146:ENSG00000123136:ENSG00000269190:ENSG00000167625:ENSG00000105427:ENSG00000187244:ENSG00000105483:ENSG00000105447:ENSG00000196214:ENSG00000198464:ENSG00000167766:ENSG00000213020:ENSG00000115761:ENSG00000143867:ENSG00000163803:ENSG00000171103:ENSG00000162928:ENSG00000144034:ENSG00000144048:ENSG00000114978:ENSG00000065911:ENSG00000115604:ENSG00000153214:ENSG00000115963:ENSG00000153250:ENSG00000118260:ENSG00000115457:ENSG00000115461:ENSG00000018280:ENSG00000144579:ENSG00000132326:ENSG00000162804:ENSG00000088876:ENSG00000132646:ENSG00000188559:ENSG00000124107:ENSG00000124228:ENSG00000124201:ENSG00000125520:ENSG00000198276:ENSG00000155313:ENSG00000154642:ENSG00000100342:ENSG00000198951:ENSG00000177096:ENSG00000242247:ENSG00000077942:ENSG00000186951:ENSG00000075234:ENSG00000130489:ENSG00000025708:ENSG00000177989:ENSG00000070950:ENSG00000144560:ENSG00000163513:ENSG00000170293:ENSG00000182983:ENSG00000214706:ENSG00000114378:ENSG00000068001:ENSG00000068028:ENSG00000004838:ENSG00000114395:ENSG00000126062:ENSG00000180376:ENSG00000163946:ENSG00000114861:ENSG00000144736:ENSG00000163320:ENSG00000175105:ENSG00000179021:ENSG00000184500:ENSG00000169379:ENSG00000163607:ENSG00000163608:ENSG00000144843:ENSG00000172780:ENSG00000240682:ENSG00000240303:ENSG00000113971:ENSG00000138231:ENSG00000114098:ENSG00000184432:ENSG00000155903:ENSG00000175066:ENSG00000114127:ENSG00000114698:ENSG00000163762:ENSG00000075420:ENSG00000041802:ENSG00000169019:ENSG00000145246:ENSG00000109171:ENSG00000169429:ENSG00000163739:ENSG000001730

						85:ENSG00000205403:ENSG00000164190:ENSG00000067248:ENSG00000039123:ENSG00000086200:ENSG00000164331:ENSG00000164338:ENSG00000164180:ENSG00000173926:ENSG00000131504:ENSG00000197948:ENSG00000120318:ENSG00000135074:ENSG00000113272:ENSG00000131446:ENSG00000146112:ENSG00000137404:ENSG00000137337:ENSG00000137331:ENSG00000244731:ENSG00000235863:ENSG00000124762:ENSG00000164530:ENSG00000112245:ENSG00000198945:ENSG00000118503:ENSG00000112406:ENSG00000120278:ENSG00000112096:ENSG00000026297:ENSG00000186340:ENSG00000184786:ENSG00000130023:ENSG00000168303:ENSG00000152926:ENSG00000213462:ENSG00000126522:ENSG00000086730:ENSG00000127920:ENSG00000105829:ENSG00000160813:ENSG00000091704:ENSG00000122786:ENSG00000182158:ENSG00000181444:ENSG00000196329:ENSG00000214106:ENSG00000146918:ENSG00000117868:ENSG00000120910:ENSG00000254087:ENSG00000035681:ENSG00000121039:ENSG00000164949:ENSG00000120526:ENSG00000120533:ENSG00000080608:ENSG00000213694:ENSG00000127080:ENSG00000130958:ENSG00000136925:ENSG00000136826:ENSG00000167081:ENSG00000196152:ENSG00000136840:ENSG00000198113
DEG. down	Brain_Frontal_Cortex_BA9	5080	325	1.70377897020096e-12	9.2004064390852e-11	ENSG00000158286:ENSG00000120942:ENSG00000162458:ENSG00000065526:ENSG00000157978:ENSG00000134668:ENSG00000084652:ENSG00000066056:ENSG00000066322:ENSG00000198198:ENSG00000162367:ENSG00000117133:ENSG00000142867:ENSG00000117528:ENSG00000117543:ENSG00000134215:ENSG00000162775:ENSG00000168679:ENSG00000116455:ENSG00000143457:ENSG00000163131:ENSG00000143387:ENSG00000143437:ENSG00000143379:ENSG00000143418:ENSG00000143412:ENSG00000143409:ENSG00000143363:ENSG00000163154:ENSG00000143537:ENSG00000176444:ENSG00000125459:ENSG00000163374:ENSG00000132676:ENSG00000116580:ENSG00000143622:ENSG00000132680:ENSG00000160789:ENSG00000160783:ENSG00000163472:ENSG00000198715:ENSG00000187800:ENSG00000143226:ENSG00000186283:ENSG00000057252:ENSG00000122176:ENSG00000163050:ENSG00000162885:ENSG00000116962:ENSG00000162714:ENSG00000107929:ENSG00000134463:ENSG00000165997:ENSG00000165476:ENSG00000075073:ENSG00000107736:ENSG00000107738:ENSG00000107796:ENSG00000026103:ENSG00000059573:ENSG00000120057:ENSG00000198873:ENSG00000166478:ENSG00000149150:ENSG00000162298:ENSG00000173653:ENSG00000175567:ENSG00000175564:ENSG00000062282:ENSG00000137693:ENSG00000149292:ENSG00000076053:ENSG00000076043:ENSG00000166250:ENSG00000064309:ENSG00000082805:ENSG00000111671:ENSG00000111679:ENSG00000126749:ENSG00000111684:ENSG00000182326:ENSG00000134531:ENSG00000123094:ENSG00000123338:ENSG00000127314:ENSG00000166130:ENSG00000235162:ENSG00000174989:ENSG00000111725:ENSG00000188735:ENSG00000150990:ENSG00000177084:ENSG00000176915:ENSG00000127863:ENSG00000102743:ENSG00000136167:ENSG00000102531:ENSG00000123179:ENSG00000188243:ENSG00000118939:ENSG00000178695:ENSG00000088451:ENSG00000134901:ENSG00000100889:ENSG00000100473:ENSG00000129493:ENSG00000150527:ENSG00000179476:ENSG00000185246:ENSG00000139926:ENSG00000119707:ENSG00000100767:ENSG00000133961:ENSG00000165934:ENSG00000021776:ENSG00000174197:ENSG00000243789:ENSG00000181827:ENSG00000140262:ENSG00000103710:ENSG00000117899:ENSG00000140511:ENSG00000140471:ENSG00000179889:ENSG00000085721:ENSG00000157106:ENSG00000180096:ENSG00000140682:ENSG00000102921:ENSG00000072736:ENSG00000103064:ENSG00000158717:ENSG00000127804:ENSG00000197417:ENSG00000072849:ENSG00000166484:ENSG00000166482:ENSG00000142494:ENSG00000068079:ENSG00000108828:ENSG00000182963:ENSG00000225190:ENSG00000011258:ENSG00000121060:ENSG00000108423:ENSG00000189050:ENSG00000198265:ENSG00000185262:ENSG00000141577:ENSG00000167302:ENSG00000185504:ENSG00000182446:ENSG00000101608:ENSG00000154845:ENSG00000154889:ENSG00000141401:ENSG00000168234:ENSG00000152223:ENSG00000071564:ENSG00000205744:ENSG00000171105:ENSG00000104883:ENSG00000104957:ENSG00000132017:ENSG00000132005:ENSG00000123146:ENSG00000123136:ENSG00000269190:ENSG00000167625:ENSG00000105722:ENSG00000105427:ENSG00000079435:ENSG00000187244:ENSG00000105483:ENSG00000105447:ENSG00000196214:ENSG00000198464:ENSG00000167766:ENSG00000115761:ENSG00000163803:ENSG00000162928:ENSG00000144034:ENSG00000144048:ENSG00000114978:ENSG00000065911:ENSG00000115604:ENSG00000153214:ENSG00000115963:ENSG00000153250:ENSG00000118260:ENSG00000115457:ENSG00000115461:ENSG00000018280:ENSG00000144579:ENSG00000135912:ENSG00000132326:ENSG00000162804:ENSG00000088876:ENSG00000188559:ENSG00000198959:ENSG00000124107:ENSG00000124228:ENSG00000124201:ENSG00000125520:ENSG00000198276:ENSG00000154642:ENSG00000100342:ENSG00000198951:ENSG00000177096:ENSG00000100197:ENSG00000242247:ENSG00000077942:ENSG00000186951:ENSG00000075234:ENSG00000196576:ENSG00000130489:ENSG00000025708:ENSG00000177989:ENSG00000205560:ENSG00000100299:ENSG00000070950:ENSG00000144560:ENSG00000163513:ENSG00000170293:ENSG00000177873:ENSG00000182983:ENSG00000214706:ENSG00000114378:ENSG00000068001:ENSG00000068028:ENSG00000114395:ENSG00000126062:ENSG00000180376:ENSG00000163946:ENSG00000114861:ENSG00000144736:ENSG00000175105:ENSG00000184500:ENSG00000169379:ENSG00000163607:ENSG00000144843:ENSG00000172780:ENSG00000240682:ENSG00000240303:ENSG00000113971:ENSG00000138231:ENSG00000184432:ENSG00000175066:ENSG00000114127:ENSG00000114698:ENSG00000075420:ENSG00000041802:ENSG00000145246:ENSG00000109171:ENSG00000169429:ENSG00000163739:ENSG00000173085:ENSG00000185477:ENSG00000205403:ENSG00000164190:ENSG00000067248:ENSG00000039123:ENSG00000164331:ENSG00000164338:ENSG00000164180:ENSG00000173926:ENSG00000131504:ENSG00000197948:ENSG00000120318:ENSG00000113721:ENSG00000135074:ENSG00000113272:ENSG00000131446:ENSG00000146112:ENSG00000137404:ENSG00000137337:ENSG00000137331:ENSG00000204344:ENSG00000244731:ENSG00000235863:ENSG00000227057:ENSG00000237441:ENSG00000124762:ENSG00000164530:ENSG00000198945:ENSG00000118503:ENSG00000112406:ENSG00000120278:ENSG00000186340:ENSG00000184786:ENSG00000130023:ENSG00000152926:ENSG00000213462:ENSG00000126522:ENSG00000086730:ENSG00000127920:ENSG

						00000105829:ENSG00000085514:ENSG00000160813:ENSG00000091704:ENSG00000122786:ENSG00000182158:ENSG00000181444:ENSG00000196329:ENSG00000214106:ENSG00000146918:ENSG00000117868:ENSG00000120910:ENSG00000254087:ENSG00000035681:ENSG00000121039:ENSG00000164949:ENSG00000120526:ENSG00000080608:ENSG00000213694:ENSG00000127080:ENSG00000130958:ENSG00000136925:ENSG00000136826:ENSG00000167081:ENSG00000196152:ENSG00000136840:ENSG00000148308:ENSG00000170835:ENSG00000198113
DEG. down	Brain_Hippocampus	7571	513	3.91057233076787e-27	2.11170905861465e-25	ENSG00000158286:ENSG00000116237:ENSG00000158292:ENSG00000171824:ENSG00000198793:ENSG00000120942:ENSG00000162458:ENSG00000065526:ENSG00000116809:ENSG00000204178:ENSG00000157978:ENSG00000134668:ENSG00000084652:ENSG00000160051:ENSG00000160094:ENSG00000127603:ENSG00000066056:ENSG00000117399:ENSG00000159479:ENSG00000198198:ENSG00000162367:ENSG00000117862:ENSG00000198841:ENSG00000134709:ENSG00000162604:ENSG00000162616:ENSG00000117114:ENSG00000117133:ENSG00000142867:ENSG00000171488:ENSG00000117528:ENSG00000162694:ENSG00000117543:ENSG00000162775:ENSG00000168679:ENSG00000085465:ENSG00000116455:ENSG00000163349:ENSG00000197323:ENSG00000213281:ENSG00000052723:ENSG00000143457:ENSG00000163131:ENSG00000143387:ENSG00000143437:ENSG00000143379:ENSG00000143412:ENSG00000143409:ENSG00000143363:ENSG00000143434:ENSG00000163154:ENSG00000163156:ENSG00000163155:ENSG00000143537:ENSG00000160767:ENSG00000116521:ENSG00000176444:ENSG00000143630:ENSG00000160752:ENSG00000160753:ENSG00000116539:ENSG00000125459:ENSG00000163374:ENSG00000132676:ENSG00000116580:ENSG00000143622:ENSG00000132680:ENSG00000160789:ENSG00000160783:ENSG00000163472:ENSG00000198715:ENSG00000187800:ENSG00000132694:ENSG00000143226:ENSG00000120341:ENSG00000186283:ENSG00000143322:ENSG00000057252:ENSG00000122176:ENSG00000182004:ENSG00000143801:ENSG00000163050:ENSG00000162885:ENSG00000116962:ENSG00000162714:ENSG00000107929:ENSG00000134463:ENSG00000165997:ENSG00000108094:ENSG00000188234:ENSG00000165476:ENSG00000107736:ENSG00000107738:ENSG00000138134:ENSG00000107796:ENSG00000026103:ENSG00000165338:ENSG00000059573:ENSG00000119927:ENSG00000198873:ENSG00000166311:ENSG00000205339:ENSG00000166478:ENSG00000166483:ENSG00000198730:ENSG00000149150:ENSG00000162300:ENSG00000149823:ENSG00000174276:ENSG00000162298:ENSG00000149792:ENSG00000173653:ENSG00000175567:ENSG00000062282:ENSG00000137492:ENSG00000137693:ENSG00000137714:ENSG00000149292:ENSG00000076053:ENSG00000076043:ENSG00000154127:ENSG00000166250:ENSG00000082805:ENSG00000139180:ENSG00000111671:ENSG00000111679:ENSG00000126749:ENSG00000111684:ENSG00000182326:ENSG00000134531:ENSG00000123094:ENSG00000050426:ENSG00000123338:ENSG00000127314:ENSG00000166130:ENSG00000235162:ENSG00000174989:ENSG00000111725:ENSG00000188735:ENSG00000150990:ENSG00000177169:ENSG00000177084:ENSG00000176915:ENSG00000127863:ENSG00000102743:ENSG00000136167:ENSG00000102445:ENSG00000102531:ENSG00000123179:ENSG00000102753:ENSG00000136104:ENSG00000188243:ENSG00000118939:ENSG00000136153:ENSG00000088451:ENSG00000065150:ENSG00000134901:ENSG00000139826:ENSG00000102524:ENSG00000100889:ENSG00000092140:ENSG00000092108:ENSG00000100473:ENSG00000150527:ENSG00000165355:ENSG00000179476:ENSG00000179454:ENSG00000185246:ENSG00000139926:ENSG00000197930:ENSG00000119599:ENSG00000165861:ENSG00000119707:ENSG00000100767:ENSG00000133961:ENSG00000165417:ENSG00000165934:ENSG00000153485:ENSG00000140157:ENSG00000128731:ENSG00000021776:ENSG00000198146:ENSG00000174197:ENSG00000137802:ENSG00000243708:ENSG00000243789:ENSG00000181827:ENSG00000103707:ENSG00000186198:ENSG00000136378:ENSG00000103888:ENSG00000117899:ENSG00000140406:ENSG00000140511:ENSG00000140471:ENSG00000179889:ENSG00000157045:ENSG00000085721:ENSG00000157106:ENSG00000180096:ENSG00000179958:ENSG00000140691:ENSG00000140682:ENSG00000197302:ENSG00000102910:ENSG00000196470:ENSG00000102921:ENSG00000005194:ENSG00000102978:ENSG00000072736:ENSG00000103066:ENSG00000103064:ENSG00000103061:ENSG00000132600:ENSG00000158717:ENSG00000167522:ENSG00000197912:ENSG00000127804:ENSG00000197417:ENSG00000005100:ENSG00000072849:ENSG00000166484:ENSG00000166482:ENSG00000068079:ENSG00000108828:ENSG00000012048:ENSG00000184988:ENSG00000182963:ENSG00000225190:ENSG00000141279:ENSG00000108424:ENSG00000011258:ENSG00000011260:ENSG00000121060:ENSG00000108423:ENSG00000108443:ENSG00000189050:ENSG00000198265:ENSG00000197170:ENSG00000185262:ENSG00000141577:ENSG00000167302:ENSG00000185504:ENSG00000182446:ENSG00000101608:ENSG00000017797:ENSG00000154845:ENSG00000154889:ENSG00000141401:ENSG00000168234:ENSG00000141384:ENSG00000152223:ENSG00000130856:ENSG00000071564:ENSG00000141985:ENSG00000167670:ENSG00000205744:ENSG00000171105:ENSG00000104880:ENSG00000167775:ENSG00000079999:ENSG00000104957:ENSG00000132017:ENSG00000132005:ENSG00000123146:ENSG00000123136:ENSG00000127527:ENSG00000237440:ENSG00000104835:ENSG00000128626:ENSG00000269190:ENSG00000028277:ENSG00000160570:ENSG00000167625:ENSG00000105722:ENSG00000079432:ENSG00000105427:ENSG00000187244:ENSG00000105483:ENSG00000105447:ENSG00000063127:ENSG00000142544:ENSG00000105383:ENSG00000196214:ENSG00000198464:ENSG00000167766:ENSG00000213020:ENSG00000115705:ENSG00000115761:ENSG00000143867:ENSG00000055917:ENSG00000171103:ENSG00000162928:ENSG00000082898:ENSG00000144034:ENSG00000144048:ENSG00000114978:ENSG00000065911:ENSG00000188687:ENSG00000115602:ENSG00000115604:ENSG00000153107:ENSG00000153208:ENSG00000153214:ENSG00000115963:ENSG00000153250:ENSG00000118260:ENSG00000197713:ENSG00000115457:ENSG00000018280:ENSG00000144579:ENSG00000135912:ENSG00000132326:ENSG00000162804:ENSG00000196209:ENSG00000088876:ENSG00000125779:ENSG00000132646:ENSG00000188559:ENSG00000118705:ENSG00000198959:ENSG00000124107:ENSG00000124228:ENSG00000124201:ENSG00000125520:ENSG00000198276:ENSG00000155313:ENSG00000157617:ENSG00000160190:ENSG00000100220:ENSG00000128335:ENSG00000100342:ENSG00000198951:ENSG00000177096:ENSG00000100197:ENSG00000242247:ENSG00000077942:ENSG00

						000186951:ENSG00000205643:ENSG00000075234:ENSG00000100416:ENSG00000196576:ENSG00000100258:ENSG00000025770:ENSG00000130489:ENSG00000025708:ENSG00000177989:ENSG00000205560:ENSG00000100288:ENSG00000100299:ENSG00000070950:ENSG00000134077:ENSG00000144560:ENSG00000144712:ENSG00000163513:ENSG00000170293:ENSG00000177873:ENSG00000182606:ENSG00000182983:ENSG00000214706:ENSG00000243477:ENSG00000114378:ENSG00000068001:ENSG00000068028:ENSG00000114388:ENSG00000114395:ENSG00000126062:ENSG00000007402:ENSG00000180376:ENSG00000163946:ENSG00000114861:ENSG00000144736:ENSG00000163320:ENSG00000175105:ENSG00000179021:ENSG00000184500:ENSG00000169379:ENSG00000114446:ENSG00000163607:ENSG00000163608:ENSG00000144843:ENSG00000172780:ENSG00000240682:ENSG00000034533:ENSG00000240303:ENSG00000113971:ENSG00000081307:ENSG00000144867:ENSG00000158163:ENSG00000138231:ENSG00000114098:ENSG00000175110:ENSG00000184432:ENSG00000155903:ENSG00000175066:ENSG00000114127:ENSG00000114698:ENSG00000075420:ENSG00000161202:ENSG00000161204:ENSG00000145014:ENSG00000041802:ENSG00000163975:ENSG00000013288:ENSG00000169019:ENSG00000145246:ENSG00000109171:ENSG00000169429:ENSG00000163739:ENSG00000173085:ENSG00000185477:ENSG00000205403:ENSG00000164128:ENSG00000129187:ENSG00000113361:ENSG00000113360:ENSG00000164190:ENSG00000132357:ENSG00000067248:ENSG00000039123:ENSG00000086200:ENSG00000164331:ENSG00000164338:ENSG00000164180:ENSG00000173926:ENSG00000131504:ENSG00000171720:ENSG00000197948:ENSG00000120318:ENSG00000113721:ENSG00000135074:ENSG00000113272:ENSG00000155858:ENSG00000048140:ENSG00000131446:ENSG00000196670:ENSG00000079691:ENSG00000146112:ENSG00000137404:ENSG00000137337:ENSG00000137331:ENSG00000204344:ENSG00000244731:ENSG00000223501:ENSG00000235863:ENSG00000227057:ENSG00000237441:ENSG00000231925:ENSG00000124762:ENSG00000198663:ENSG00000112245:ENSG00000198945:ENSG00000112357:ENSG00000118503:ENSG00000112406:ENSG00000120278:ENSG00000112096:ENSG00000213066:ENSG00000186340:ENSG00000184786:ENSG00000130023:ENSG00000122644:ENSG00000153814:ENSG00000168303:ENSG00000152926:ENSG00000213462:ENSG00000126522:ENSG00000165215:ENSG00000049541:ENSG00000135218:ENSG00000127920:ENSG00000105829:ENSG00000085514:ENSG00000146834:ENSG00000160813:ENSG00000091704:ENSG00000106484:ENSG00000158623:ENSG00000131558:ENSG00000122786:ENSG00000182158:ENSG00000181444:ENSG00000196329:ENSG00000214106:ENSG00000146918:ENSG00000117868:ENSG00000120910:ENSG00000129691:ENSG00000254087:ENSG00000035681:ENSG00000121039:ENSG00000164949:ENSG00000120526:ENSG00000120533:ENSG00000196378:ENSG00000080608:ENSG00000213694:ENSG00000127080:ENSG00000130958:ENSG00000136925:ENSG00000136936:ENSG00000136826:ENSG00000070159:ENSG00000167081:ENSG00000196152:ENSG00000148356:ENSG00000136840:ENSG00000171169:ENSG00000148339:ENSG00000148335:ENSG00000148308:ENSG00000170835:ENSG00000198113
DEG. down	Brain_Hypothalamus	5967	385	1.28704900258626e-15	6.95006461396579e-14	ENSG00000116237:ENSG00000158292:ENSG00000198793:ENSG00000120942:ENSG00000162458:ENSG00000065526:ENSG00000116809:ENSG00000187942:ENSG00000157978:ENSG00000084652:ENSG00000160094:ENSG00000066056:ENSG00000198198:ENSG00000162367:ENSG00000162390:ENSG00000117114:ENSG00000117133:ENSG00000142867:ENSG00000171488:ENSG00000117528:ENSG00000134215:ENSG00000162775:ENSG00000168679:ENSG00000116455:ENSG00000197323:ENSG00000163131:ENSG00000143387:ENSG00000143437:ENSG00000143379:ENSG00000143412:ENSG00000143409:ENSG00000143363:ENSG00000163156:ENSG00000163354:ENSG00000143537:ENSG00000176444:ENSG00000116539:ENSG00000125459:ENSG00000163374:ENSG00000116580:ENSG00000143622:ENSG00000132680:ENSG00000116584:ENSG00000160789:ENSG00000160783:ENSG00000163472:ENSG00000198715:ENSG00000187800:ENSG00000120341:ENSG00000186283:ENSG00000143322:ENSG00000057252:ENSG00000122176:ENSG00000163050:ENSG00000162885:ENSG00000116962:ENSG00000162714:ENSG00000107929:ENSG00000134463:ENSG00000165997:ENSG00000165476:ENSG00000107738:ENSG00000107796:ENSG00000026103:ENSG00000165338:ENSG00000059573:ENSG00000119927:ENSG00000198873:ENSG00000166311:ENSG00000166478:ENSG00000166483:ENSG00000198730:ENSG00000149150:ENSG00000124935:ENSG00000162300:ENSG00000149823:ENSG00000162298:ENSG00000173653:ENSG00000175567:ENSG00000175564:ENSG00000062282:ENSG00000137492:ENSG00000137672:ENSG00000137693:ENSG00000149292:ENSG00000076053:ENSG00000076043:ENSG00000109943:ENSG00000166250:ENSG00000064309:ENSG00000111671:ENSG00000111679:ENSG00000126749:ENSG00000111684:ENSG00000182326:ENSG00000134531:ENSG00000050426:ENSG00000123338:ENSG00000127314:ENSG00000166130:ENSG00000074590:ENSG00000174989:ENSG00000111725:ENSG00000188735:ENSG00000150990:ENSG00000177169:ENSG00000177084:ENSG00000176915:ENSG00000127863:ENSG00000102743:ENSG00000136167:ENSG00000123179:ENSG00000136104:ENSG00000118939:ENSG00000136153:ENSG00000088451:ENSG00000065150:ENSG00000134901:ENSG00000139826:ENSG00000102524:ENSG00000100889:ENSG00000092140:ENSG00000150527:ENSG00000179476:ENSG00000185246:ENSG00000139926:ENSG00000197930:ENSG00000119599:ENSG00000165861:ENSG00000119707:ENSG00000100767:ENSG00000133961:ENSG00000165934:ENSG00000021776:ENSG00000198146:ENSG00000174197:ENSG00000181827:ENSG00000140262:ENSG00000103707:ENSG00000136378:ENSG00000140406:ENSG00000140511:ENSG00000140471:ENSG00000179889:ENSG00000157045:ENSG00000085721:ENSG00000157106:ENSG00000180096:ENSG00000179958:ENSG00000140682:ENSG00000102910:ENSG00000196470:ENSG00000102921:ENSG00000072736:ENSG00000103066:ENSG00000103064:ENSG00000185669:ENSG00000158717:ENSG00000167522:ENSG00000127804:ENSG00000197417:ENSG00000005100:ENSG00000072849:ENSG00000188522:ENSG00000166484:ENSG00000166482:ENSG00000068079:ENSG00000012048:ENSG00000184988:ENSG00000182963:ENSG00000225190:ENSG00000011258:ENSG00000011260:ENSG00000121060:ENSG00000108423:ENSG00000108443:ENSG00000189050:ENSG00000198265:ENSG00000185262:ENSG00000141577:ENSG00000167302:ENSG00000185504:ENSG00000182446:ENSG00000101608:ENSG00000017797:ENSG00000154845:ENSG00000154889:ENSG00000141401:ENSG00000168234:ENSG

						00000141384:ENSG00000152223:ENSG00000071564:ENSG00000141985:ENSG00000167670:ENSG00000205744:ENSG00000171105:ENSG00000104880:ENSG00000104957:ENSG00000132017:ENSG00000132005:ENSG00000123146:ENSG00000123136:ENSG00000128626:ENSG00000269190:ENSG00000167625:ENSG00000105722:ENSG00000105427:ENSG00000187244:ENSG00000105483:ENSG00000105447:ENSG00000063127:ENSG00000105383:ENSG00000196214:ENSG00000198464:ENSG00000167766:ENSG00000115761:ENSG00000055917:ENSG00000163803:ENSG00000162928:ENSG00000144048:ENSG00000114978:ENSG00000065911:ENSG00000188687:ENSG00000115604:ENSG00000153107:ENSG00000153208:ENSG00000153214:ENSG00000115963:ENSG00000153250:ENSG00000118260:ENSG00000115457:ENSG00000115461:ENSG00000144579:ENSG00000132326:ENSG00000162804:ENSG00000196209:ENSG00000088876:ENSG00000132646:ENSG00000188559:ENSG00000198959:ENSG00000124107:ENSG00000124228:ENSG00000124201:ENSG00000125520:ENSG00000198276:ENSG00000155313:ENSG00000157617:ENSG00000100342:ENSG00000198951:ENSG00000177096:ENSG00000242247:ENSG00000077942:ENSG00000186951:ENSG00000205643:ENSG00000075234:ENSG00000196576:ENSG00000130489:ENSG00000025708:ENSG00000177989:ENSG00000205560:ENSG00000100299:ENSG00000070950:ENSG00000144560:ENSG00000163513:ENSG00000170293:ENSG00000182606:ENSG00000182983:ENSG00000214706:ENSG00000114378:ENSG00000068001:ENSG00000068028:ENSG00000114395:ENSG00000126062:ENSG00000180376:ENSG00000163946:ENSG00000114861:ENSG00000144736:ENSG00000163320:ENSG00000175105:ENSG00000184500:ENSG00000169379:ENSG00000163607:ENSG00000163608:ENSG00000144843:ENSG00000172780:ENSG00000240682:ENSG00000240303:ENSG00000113971:ENSG00000158163:ENSG00000138231:ENSG00000114098:ENSG00000175110:ENSG00000184432:ENSG00000155903:ENSG00000175066:ENSG00000114127:ENSG00000075420:ENSG00000161202:ENSG00000041802:ENSG00000163975:ENSG00000013288:ENSG00000145246:ENSG00000109171:ENSG00000169429:ENSG00000163739:ENSG00000173085:ENSG00000205403:ENSG00000164190:ENSG00000171522:ENSG00000132357:ENSG00000067248:ENSG00000039123:ENSG00000086200:ENSG00000164338:ENSG00000164180:ENSG00000131504:ENSG00000171720:ENSG00000197948:ENSG00000120318:ENSG00000113721:ENSG00000135074:ENSG00000113272:ENSG00000048140:ENSG00000131446:ENSG00000196670:ENSG00000078401:ENSG00000079691:ENSG00000146112:ENSG00000137404:ENSG00000137337:ENSG00000137331:ENSG00000204344:ENSG00000244731:ENSG00000223501:ENSG00000235863:ENSG00000227057:ENSG00000237441:ENSG00000231925:ENSG00000124762:ENSG00000198945:ENSG00000118503:ENSG00000112406:ENSG00000120278:ENSG00000091831:ENSG00000186340:ENSG00000122644:ENSG00000152926:ENSG00000213462:ENSG00000126522:ENSG00000049541:ENSG00000135218:ENSG00000127920:ENSG00000085514:ENSG00000146834:ENSG00000160813:ENSG00000091704:ENSG00000131558:ENSG00000122786:ENSG00000182158:ENSG00000181444:ENSG00000196329:ENSG00000214106:ENSG00000146918:ENSG00000117868:ENSG00000120910:ENSG00000254087:ENSG00000035681:ENSG00000121039:ENSG00000164949:ENSG00000120526:ENSG00000080608:ENSG00000044459:ENSG00000213694:ENSG00000127080:ENSG00000130958:ENSG00000136925:ENSG00000136826:ENSG00000070159:ENSG00000196152:ENSG00000136840:ENSG00000171169:ENSG00000148335:ENSG00000148308:ENSG00000170835:ENSG00000198113
DEG.down	Brain_Nucleus_accumbens_basal_ganglia	6479	417	6.13822690278487e-17	3.31464252750383e-15	ENSG00000158286:ENSG00000158292:ENSG00000171824:ENSG00000198793:ENSG00000120942:ENSG00000162458:ENSG00000065526:ENSG00000116809:ENSG00000204178:ENSG00000157978:ENSG00000134668:ENSG00000084652:ENSG00000116525:ENSG00000134686:ENSG00000066056:ENSG00000066322:ENSG00000159479:ENSG00000198198:ENSG00000162367:ENSG00000117862:ENSG00000198841:ENSG00000162390:ENSG00000117114:ENSG00000142867:ENSG00000171488:ENSG00000117528:ENSG00000134215:ENSG00000116396:ENSG00000162775:ENSG00000168679:ENSG00000116455:ENSG00000163349:ENSG00000197323:ENSG00000213281:ENSG00000052723:ENSG00000143457:ENSG00000163131:ENSG00000143387:ENSG00000143437:ENSG00000143379:ENSG00000143418:ENSG00000143412:ENSG00000143409:ENSG00000143363:ENSG00000143434:ENSG00000163154:ENSG00000163156:ENSG00000143537:ENSG00000116521:ENSG00000176444:ENSG00000160752:ENSG00000125459:ENSG00000163374:ENSG00000132676:ENSG00000116580:ENSG00000143622:ENSG00000132680:ENSG00000160789:ENSG00000160783:ENSG00000163472:ENSG00000198715:ENSG00000187800:ENSG00000132694:ENSG00000143226:ENSG00000186283:ENSG00000057252:ENSG00000122176:ENSG00000182004:ENSG00000143801:ENSG00000163050:ENSG00000162885:ENSG00000116962:ENSG00000162714:ENSG00000107929:ENSG00000134463:ENSG00000165997:ENSG00000165476:ENSG00000075073:ENSG00000107736:ENSG00000107796:ENSG00000026103:ENSG00000059573:ENSG00000120057:ENSG00000203867:ENSG00000198873:ENSG00000166311:ENSG00000110169:ENSG00000205339:ENSG00000166478:ENSG00000198730:ENSG00000149150:ENSG00000162300:ENSG00000162298:ENSG00000173653:ENSG00000175567:ENSG00000175564:ENSG00000137492:ENSG00000137693:ENSG00000137714:ENSG00000149292:ENSG00000076053:ENSG00000076043:ENSG00000064309:ENSG00000111671:ENSG00000111679:ENSG00000126749:ENSG00000111684:ENSG00000182326:ENSG00000134531:ENSG00000123094:ENSG00000110911:ENSG00000123338:ENSG00000127314:ENSG00000139292:ENSG00000166130:ENSG00000235162:ENSG00000074590:ENSG00000174989:ENSG00000111725:ENSG00000150990:ENSG00000177084:ENSG00000176915:ENSG00000127863:ENSG00000102743:ENSG00000136167:ENSG00000102445:ENSG00000123179:ENSG00000088451:ENSG00000065150:ENSG00000134901:ENSG00000139826:ENSG00000102524:ENSG00000100889:ENSG00000092108:ENSG00000129493:ENSG00000150527:ENSG00000179476:ENSG00000179454:ENSG00000185246:ENSG00000139926:ENSG00000197930:ENSG00000119599:ENSG00000165861:ENSG00000119707:ENSG00000080815:ENSG00000133961:ENSG00000165934:ENSG00000153485:ENSG00000140157:ENSG00000021776:ENSG00000174197:ENSG00000243708:ENSG00000243789:ENSG00000181827:ENSG00000140262:ENSG00000140511:ENSG00000140471:ENSG00000179889:ENSG00000085721:ENSG00000157106:ENSG00000180096:ENSG00000179958:ENSG00000140682:ENSG00000196470:ENSG00000102921:ENSG00000005194:ENSG00000072736:ENSG00000103066:ENSG00000103064:ENSG00000103061:ENSG00000158717:ENSG00000167522:ENSG000001979

						12:ENSG00000127804:ENSG00000196689:ENSG00000197417:ENSG00000005100:ENSG00000072849:ENSG00000166484:ENSG00000166482:ENSG00000142494:ENSG00000068079:ENSG00000182963:ENSG00000225190:ENSG00000198933:ENSG00000011258:ENSG00000011260:ENSG00000121060:ENSG00000108423:ENSG00000108443:ENSG00000189050:ENSG00000198265:ENSG00000197170:ENSG00000185262:ENSG00000141577:ENSG00000167302:ENSG00000185504:ENSG00000182446:ENSG00000101608:ENSG00000017797:ENSG00000154845:ENSG00000154889:ENSG00000141401:ENSG00000168234:ENSG00000152223:ENSG00000071564:ENSG00000141985:ENSG00000167670:ENSG00000205744:ENSG00000171105:ENSG00000104880:ENSG00000104883:ENSG00000167775:ENSG00000079999:ENSG00000104957:ENSG00000132016:ENSG00000132000:ENSG00000132017:ENSG00000132005:ENSG00000123146:ENSG00000123136:ENSG00000127527:ENSG00000104835:ENSG00000128626:ENSG00000269190:ENSG00000167625:ENSG00000105722:ENSG00000187244:ENSG00000105483:ENSG00000105447:ENSG00000196214:ENSG00000198464:ENSG00000115756:ENSG00000115761:ENSG00000055917:ENSG00000171103:ENSG00000162928:ENSG00000144034:ENSG00000144048:ENSG00000114978:ENSG00000065911:ENSG00000115602:ENSG00000153107:ENSG00000153214:ENSG00000115963:ENSG00000153250:ENSG00000118260:ENSG00000115457:ENSG00000115461:ENSG00000018280:ENSG00000144579:ENSG00000132326:ENSG00000162804:ENSG00000088876:ENSG00000132646:ENSG00000188559:ENSG00000198959:ENSG00000124107:ENSG00000124228:ENSG00000124201:ENSG00000125520:ENSG00000198276:ENSG00000154642:ENSG00000157617:ENSG00000128335:ENSG00000100342:ENSG00000198951:ENSG00000177096:ENSG00000242247:ENSG00000077942:ENSG00000186951:ENSG00000205643:ENSG00000075234:ENSG00000196576:ENSG00000100258:ENSG00000130489:ENSG00000025708:ENSG00000177989:ENSG00000100288:ENSG00000100299:ENSG00000070950:ENSG00000134077:ENSG00000144560:ENSG00000163513:ENSG00000170293:ENSG00000177873:ENSG00000182606:ENSG00000182983:ENSG00000214706:ENSG00000114378:ENSG00000068001:ENSG00000068028:ENSG00000114395:ENSG00000126062:ENSG00000180376:ENSG00000163946:ENSG00000144736:ENSG00000163320:ENSG00000175105:ENSG00000184500:ENSG00000169379:ENSG00000114446:ENSG00000163607:ENSG00000163608:ENSG00000144843:ENSG00000172780:ENSG00000240682:ENSG00000034533:ENSG00000240303:ENSG00000113971:ENSG00000081307:ENSG00000144867:ENSG00000138231:ENSG00000114098:ENSG00000175110:ENSG00000184432:ENSG00000155903:ENSG00000175066:ENSG00000114127:ENSG00000114698:ENSG00000163762:ENSG00000075420:ENSG00000161202:ENSG00000161204:ENSG00000041802:ENSG00000163975:ENSG00000013288:ENSG00000145246:ENSG00000109171:ENSG00000169429:ENSG00000163739:ENSG00000173085:ENSG00000205403:ENSG00000164190:ENSG00000067248:ENSG00000039123:ENSG00000086200:ENSG00000164331:ENSG00000164338:ENSG00000164180:ENSG00000173926:ENSG00000064651:ENSG00000131504:ENSG00000171720:ENSG00000197948:ENSG00000120318:ENSG00000113721:ENSG00000113272:ENSG00000155858:ENSG00000048140:ENSG00000131446:ENSG00000196670:ENSG00000078401:ENSG00000146112:ENSG00000137404:ENSG00000137337:ENSG00000137331:ENSG00000204344:ENSG00000244731:ENSG00000223501:ENSG00000235863:ENSG00000227057:ENSG00000124762:ENSG00000164530:ENSG00000112245:ENSG00000198945:ENSG00000118503:ENSG00000112406:ENSG00000120278:ENSG00000112096:ENSG00000186340:ENSG00000122644:ENSG00000168303:ENSG00000152926:ENSG00000213462:ENSG00000126522:ENSG00000086730:ENSG00000049541:ENSG00000127920:ENSG00000105829:ENSG00000085514:ENSG00000078487:ENSG00000146834:ENSG00000160813:ENSG00000075790:ENSG00000091704:ENSG00000131558:ENSG00000122786:ENSG00000182158:ENSG00000196329:ENSG00000214106:ENSG00000146918:ENSG00000117868:ENSG00000168546:ENSG00000120910:ENSG00000254087:ENSG00000035681:ENSG00000121039:ENSG00000164949:ENSG00000120526:ENSG00000120533:ENSG00000180900:ENSG00000196378:ENSG00000080608:ENSG00000213694:ENSG00000127080:ENSG00000130958:ENSG00000136925:ENSG00000136826:ENSG00000196152:ENSG00000148356:ENSG00000136840:ENSG00000171169:ENSG00000148335:ENSG00000148308:ENSG00000170835:ENSG00000198113
DEG. down	Brain_Putamen_basal_ganglia	7853	520	3.72256255441065e-25	2.01018377938175e-23	ENSG00000158286:ENSG00000158292:ENSG00000171824:ENSG00000198793:ENSG00000120942:ENSG00000162458:ENSG00000065526:ENSG00000116809:ENSG00000117616:ENSG00000204178:ENSG00000157978:ENSG00000084652:ENSG00000160050:ENSG00000160051:ENSG00000116525:ENSG00000160094:ENSG00000134686:ENSG00000127603:ENSG00000066056:ENSG00000159479:ENSG00000198198:ENSG00000162367:ENSG00000117862:ENSG00000198841:ENSG00000134717:ENSG00000162390:ENSG00000162604:ENSG00000162616:ENSG00000117114:ENSG00000117133:ENSG00000142867:ENSG00000171488:ENSG00000117528:ENSG00000117543:ENSG00000134215:ENSG00000162775:ENSG00000168679:ENSG00000116455:ENSG00000163349:ENSG00000197323:ENSG00000213281:ENSG00000052723:ENSG00000143457:ENSG00000163131:ENSG00000143387:ENSG00000143437:ENSG00000143379:ENSG00000143412:ENSG00000143409:ENSG00000143363:ENSG00000143434:ENSG00000163154:ENSG00000163156:ENSG00000163155:ENSG00000143537:ENSG00000160767:ENSG00000116521:ENSG00000176444:ENSG00000143630:ENSG00000160752:ENSG00000160753:ENSG00000116539:ENSG00000125459:ENSG00000163374:ENSG00000132676:ENSG00000116580:ENSG00000143622:ENSG00000132680:ENSG00000160789:ENSG00000160783:ENSG00000163472:ENSG00000198715:ENSG00000187800:ENSG00000132694:ENSG00000143226:ENSG00000152332:ENSG00000120341:ENSG00000186283:ENSG00000143322:ENSG00000057252:ENSG00000177888:ENSG00000122176:ENSG00000182004:ENSG00000143801:ENSG00000163050:ENSG00000116962:ENSG00000162714:ENSG00000107929:ENSG00000198879:ENSG00000134463:ENSG00000241058:ENSG00000165997:ENSG00000108094:ENSG00000188234:ENSG00000165476:ENSG00000107736:ENSG00000107738:ENSG00000138134:ENSG00000107796:ENSG00000026103:ENSG00000165338:ENSG00000059573:ENSG00000203867:ENSG00000198873:ENSG00000166311:ENSG00000205339:ENSG00000166478:ENSG00000198730:ENSG00000149150:ENSG00000162300:ENSG00000149823:ENSG00000174276:ENSG00000162298:ENSG00000149792:ENSG00000173653:ENSG00000175567:ENSG00000137492:ENSG00000137693:ENSG00000137714:ENSG00000149292:ENSG00000076053:ENSG00000076043:ENSG00000154127:ENSG00

	000082805:ENSG00000010219:ENSG00000139180:ENSG00000111671:ENSG00000111679:ENSG00000126749:ENSG00000111684:ENSG00000182326:ENSG000001<br>34531:ENSG00000123094:ENSG00000110911:ENSG00000050426:ENSG00000135457:ENSG00000123338:ENSG00000127314:ENSG00000166130:ENSG0000012086<br>8:ENSG00000235162:ENSG00000074590:ENSG00000174989:ENSG00000111725:ENSG00000150990:ENSG00000177084:ENSG00000176915:ENSG00000127863:EN<br>SG00000102743:ENSG00000136167:ENSG00000102445:ENSG00000102531:ENSG00000123179:ENSG00000102753:ENSG00000136104:ENSG00000188243:ENSG0<br>0000118939:ENSG00000136153:ENSG00000178695:ENSG00000088451:ENSG00000065150:ENSG00000134901:ENSG00000139826:ENSG00000102524:ENSG00000<br>100889:ENSG00000092108:ENSG00000129493:ENSG00000150527:ENSG00000179476:ENSG00000179454:ENSG00000185246:ENSG00000139926:ENSG000001979<br>30:ENSG00000119599:ENSG00000165861:ENSG00000119707:ENSG00000100767:ENSG00000133961:ENSG00000165417:ENSG00000165934:ENSG00000153485:E<br>NSG00000170270:ENSG00000140157:ENSG00000128731:ENSG00000021776:ENSG00000198146:ENSG00000174197:ENSG00000137802:ENSG00000243708:ENSG<br>00000243789:ENSG00000181827:ENSG00000140262:ENSG00000103707:ENSG00000186198:ENSG00000103710:ENSG00000117899:ENSG00000140511:ENSG0000<br>0140471:ENSG00000008710:ENSG00000183426:ENSG00000179889:ENSG00000085721:ENSG00000157106:ENSG00000169217:ENSG00000169221:ENSG00000180<br>096:ENSG00000179958:ENSG00000140691:ENSG00000140682:ENSG00000197302:ENSG00000102910:ENSG00000196470:ENSG00000102921:ENSG00000005194:<br>ENSG00000102978:ENSG00000072736:ENSG00000103066:ENSG00000103064:ENSG00000103061:ENSG00000185669:ENSG00000158717:ENSG00000167522:ENS<br>G00000197912:ENSG00000127804:ENSG00000196689:ENSG00000197417:ENSG00000005100:ENSG00000072849:ENSG00000166484:ENSG00000166482:ENSG00<br>000142494:ENSG00000068079:ENSG00000012048:ENSG00000182963:ENSG00000225190:ENSG00000141279:ENSG00000108424:ENSG00000198933:ENSG000000<br>11258:ENSG00000011260:ENSG00000214226:ENSG00000121060:ENSG00000108423:ENSG00000108443:ENSG00000189050:ENSG00000198265:ENSG0000019717<br>0:ENSG00000153822:ENSG00000185262:ENSG00000141577:ENSG00000167302:ENSG00000185504:ENSG00000182446:ENSG00000101608:ENSG00000017797:EN<br>SG00000154845:ENSG00000154889:ENSG00000141401:ENSG00000141384:ENSG00000152223:ENSG00000130856:ENSG00000071564:ENSG00000141985:ENSG0<br>0000167670:ENSG00000205744:ENSG00000171105:ENSG00000104880:ENSG00000167775:ENSG00000065989:ENSG00000079999:ENSG00000104957:ENSG00000<br>132024:ENSG00000132000:ENSG00000132017:ENSG00000132005:ENSG00000123146:ENSG00000123136:ENSG00000127527:ENSG00000104835:ENSG000001286<br>26:ENSG00000269190:ENSG00000028277:ENSG00000160570:ENSG00000167625:ENSG00000105722:ENSG00000079432:ENSG00000187244:ENSG00000105483:E<br>NSG00000105447:ENSG00000142544:ENSG00000196214:ENSG00000198464:ENSG00000167766:ENSG00000213020:ENSG00000115756:ENSG00000115761:ENSG<br>00000055917:ENSG00000163803:ENSG00000171103:ENSG00000162928:ENSG00000082898:ENSG00000075292:ENSG00000144034:ENSG00000144048:ENSG0000<br>0114978:ENSG00000065911:ENSG00000115602:ENSG00000115604:ENSG00000153107:ENSG00000153214:ENSG00000115963:ENSG00000153250:ENSG00000118<br>260:ENSG00000197713:ENSG00000115457:ENSG00000115461:ENSG00000018280:ENSG00000144579:ENSG00000132326:ENSG00000162804:ENSG00000088876:<br>ENSG00000125779:ENSG00000132646:ENSG00000188559:ENSG00000198959:ENSG00000124107:ENSG00000124228:ENSG00000124201:ENSG00000125520:ENS<br>G00000198276:ENSG00000155313:ENSG00000157617:ENSG00000160190:ENSG00000100220:ENSG00000128335:ENSG00000100342:ENSG00000100324:ENSG00<br>000198951:ENSG00000177096:ENSG00000242247:ENSG00000077942:ENSG00000186951:ENSG00000205643:ENSG00000075234:ENSG00000100416:ENSG000001<br>96576:ENSG00000100258:ENSG00000025770:ENSG00000130489:ENSG00000025708:ENSG00000177989:ENSG00000205560:ENSG00000100288:ENSG0000010029<br>9:ENSG00000134077:ENSG00000144560:ENSG00000144712:ENSG00000163513:ENSG00000170293:ENSG00000170011:ENSG00000177873:ENSG00000182606:EN<br>SG00000182983:ENSG00000214706:ENSG00000114378:ENSG00000068001:ENSG00000068028:ENSG00000114388:ENSG00000114395:ENSG00000126062:ENSG0<br>0000180376:ENSG00000163946:ENSG00000144741:ENSG00000114861:ENSG00000144736:ENSG00000163320:ENSG00000175105:ENSG00000179021:ENSG00000<br>184500:ENSG00000169379:ENSG00000114446:ENSG00000163607:ENSG00000163608:ENSG00000144843:ENSG00000082701:ENSG00000172780:ENSG000002406<br>82:ENSG00000034533:ENSG00000240303:ENSG00000113971:ENSG00000081307:ENSG00000144867:ENSG00000138231:ENSG00000114098:ENSG00000175110:E<br>NSG00000184432:ENSG00000155903:ENSG00000175066:ENSG00000114127:ENSG00000114698:ENSG00000075420:ENSG00000161202:ENSG00000161204:ENSG<br>00000041802:ENSG00000163975:ENSG00000013288:ENSG00000109689:ENSG00000169019:ENSG00000145246:ENSG00000109171:ENSG00000169429:ENSG0000<br>0163739:ENSG00000173085:ENSG00000205403:ENSG00000129187:ENSG00000113360:ENSG00000164190:ENSG00000067248:ENSG00000039123:ENSG00000086<br>189:ENSG00000086200:ENSG00000164331:ENSG00000164338:ENSG00000164180:ENSG00000168916:ENSG00000173926:ENSG00000131504:ENSG00000171720:<br>ENSG00000197948:ENSG00000120318:ENSG00000113721:ENSG00000135074:ENSG00000113272:ENSG00000155858:ENSG00000113742:ENSG00000048140:ENS<br>G00000131446:ENSG00000196670:ENSG00000124535:ENSG00000124787:ENSG00000146112:ENSG00000137404:ENSG00000137337:ENSG00000137331:ENSG00<br>000204344:ENSG00000244731:ENSG00000223501:ENSG00000235863:ENSG00000227057:ENSG00000237441:ENSG00000124762:ENSG00000198663:ENSG000001<br>64530:ENSG00000112245:ENSG00000198945:ENSG00000112357:ENSG00000118503:ENSG00000112406:ENSG00000120278:ENSG00000112096:ENSG0000002629<br>7:ENSG00000213066:ENSG00000186340:ENSG00000184786:ENSG00000130023:ENSG00000122644:ENSG00000153814:ENSG00000168303:ENSG00000152926:EN<br>SG00000213462:ENSG00000126522:ENSG00000086730:ENSG00000049541:ENSG00000127920:ENSG00000105829:ENSG00000121716:ENSG00000085514:ENSG0<br>0000078487:ENSG00000146834:ENSG00000160813:ENSG00000075790:ENSG00000196937:ENSG00000091704:ENSG00000106484:ENSG00000158623:ENSG00000<br>131558:ENSG00000122786:ENSG00000182158:ENSG00000196329:ENSG00000214106:ENSG00000146918:ENSG00000117868:ENSG00000120910:ENSG000001296

						91:ENSG00000254087:ENSG00000035681:ENSG00000147601:ENSG00000121039:ENSG00000164949:ENSG00000120526:ENSG00000120533:ENSG00000180900:ENSG00000196378:ENSG00000080608:ENSG00000213694:ENSG00000127080:ENSG00000130958:ENSG00000136925:ENSG00000136936:ENSG00000136826:ENSG00000119446:ENSG00000196152:ENSG00000148356:ENSG00000136840:ENSG00000171169:ENSG00000148339:ENSG00000148335:ENSG00000148308:ENSG00000170835:ENSG00000198113
DEG. down	Brain_Spinal_cord_cervical_c-1	5136	339	6.29702102785338e-15	3.40039135504083e-13	ENSG00000158286:ENSG00000116237:ENSG00000158292:ENSG00000198793:ENSG00000171819:ENSG00000162458:ENSG00000065526:ENSG00000158747:ENSG00000084652:ENSG00000160050:ENSG00000160051:ENSG00000066056:ENSG00000198198:ENSG00000198841:ENSG00000117114:ENSG00000117133:ENSG00000117155:ENSG00000142867:ENSG00000117525:ENSG00000116396:ENSG00000162775:ENSG00000085465:ENSG00000116455:ENSG00000197323:ENSG00000143457:ENSG00000143387:ENSG00000143437:ENSG00000143379:ENSG00000143363:ENSG00000143434:ENSG00000163354:ENSG00000160767:ENSG00000176444:ENSG00000143630:ENSG00000160753:ENSG00000116539:ENSG00000125459:ENSG00000163374:ENSG00000132676:ENSG00000116580:ENSG00000143622:ENSG00000132680:ENSG00000160789:ENSG00000196189:ENSG00000198715:ENSG00000187800:ENSG00000132694:ENSG00000120341:ENSG00000186283:ENSG00000057252:ENSG00000122176:ENSG00000143801:ENSG00000163050:ENSG00000162885:ENSG00000116962:ENSG00000162714:ENSG00000107929:ENSG00000134463:ENSG00000165997:ENSG00000107736:ENSG00000107796:ENSG00000026103:ENSG00000165338:ENSG00000059573:ENSG00000120057:ENSG00000119927:ENSG00000198873:ENSG00000166483:ENSG00000198730:ENSG00000149150:ENSG00000168060:ENSG00000174276:ENSG00000173653:ENSG00000173621:ENSG00000175564:ENSG00000062282:ENSG00000137492:ENSG00000137693:ENSG00000137714:ENSG00000149292:ENSG00000076053:ENSG00000076043:ENSG00000082805:ENSG00000111684:ENSG00000182326:ENSG00000013588:ENSG00000134531:ENSG00000134532:ENSG00000123360:ENSG00000127314:ENSG00000166130:ENSG00000235162:ENSG00000174989:ENSG00000111725:ENSG00000188735:ENSG00000150990:ENSG00000177169:ENSG00000177084:ENSG00000176915:ENSG00000127863:ENSG00000102743:ENSG00000123179:ENSG00000118939:ENSG00000136153:ENSG00000134901:ENSG00000139826:ENSG00000100889:ENSG00000092108:ENSG00000100473:ENSG00000150527:ENSG00000165355:ENSG00000139926:ENSG00000197930:ENSG00000006432:ENSG00000119599:ENSG00000165861:ENSG00000100767:ENSG00000133961:ENSG00000140157:ENSG00000021776:ENSG00000198146:ENSG00000137802:ENSG00000181827:ENSG00000103707:ENSG00000140511:ENSG00000140471:ENSG00000008710:ENSG00000179889:ENSG00000085721:ENSG00000157106:ENSG00000180209:ENSG00000180096:ENSG00000179958:ENSG00000140682:ENSG00000102910:ENSG00000102921:ENSG00000005194:ENSG00000072736:ENSG00000103066:ENSG00000103064:ENSG00000103056:ENSG00000167522:ENSG00000197912:ENSG00000197417:ENSG00000005100:ENSG00000181350:ENSG00000166484:ENSG00000166482:ENSG00000142494:ENSG00000067191:ENSG00000068079:ENSG00000182963:ENSG00000225190:ENSG00000141279:ENSG00000198933:ENSG00000011258:ENSG00000121060:ENSG00000189050:ENSG00000198265:ENSG00000167302:ENSG00000185504:ENSG00000182446:ENSG00000154845:ENSG00000154889:ENSG00000141401:ENSG00000168234:ENSG00000141384:ENSG00000152223:ENSG00000071564:ENSG00000141985:ENSG00000167670:ENSG00000171105:ENSG00000065989:ENSG00000132000:ENSG00000132017:ENSG00000132005:ENSG00000123146:ENSG00000127527:ENSG00000104835:ENSG00000128626:ENSG00000269190:ENSG00000160570:ENSG00000167625:ENSG00000079432:ENSG00000105427:ENSG00000187244:ENSG00000105447:ENSG00000196214:ENSG00000115761:ENSG00000163803:ENSG00000162928:ENSG00000144048:ENSG00000114978:ENSG00000065911:ENSG00000005448:ENSG00000115604:ENSG00000153107:ENSG00000153208:ENSG00000153214:ENSG00000115963:ENSG00000153250:ENSG00000115457:ENSG00000115461:ENSG00000132326:ENSG00000162804:ENSG00000088876:ENSG00000125779:ENSG00000188559:ENSG00000124107:ENSG00000124228:ENSG00000124201:ENSG00000125520:ENSG00000198276:ENSG00000155313:ENSG00000160190:ENSG00000100342:ENSG00000198951:ENSG00000177096:ENSG00000100197:ENSG00000242247:ENSG00000077942:ENSG00000075234:ENSG00000196576:ENSG00000130489:ENSG00000177989:ENSG00000070950:ENSG00000144560:ENSG00000144712:ENSG00000163513:ENSG00000170293:ENSG00000168032:ENSG00000182606:ENSG00000182983:ENSG00000214706:ENSG00000186792:ENSG00000114378:ENSG00000068001:ENSG00000126062:ENSG00000144749:ENSG00000114861:ENSG00000144736:ENSG00000184500:ENSG00000114446:ENSG00000144843:ENSG00000172780:ENSG00000240682:ENSG00000144867:ENSG00000158163:ENSG00000184432:ENSG00000155903:ENSG00000114127:ENSG00000114698:ENSG00000075420:ENSG00000145014:ENSG00000041802:ENSG00000163975:ENSG00000013288:ENSG00000145246:ENSG00000109171:ENSG00000173085:ENSG00000164128:ENSG00000113360:ENSG00000164190:ENSG00000132357:ENSG00000067248:ENSG00000197822:ENSG00000164338:ENSG00000164180:ENSG00000175471:ENSG00000131504:ENSG00000171720:ENSG00000197948:ENSG00000120318:ENSG00000113721:ENSG00000135074:ENSG00000113272:ENSG00000155858:ENSG00000048140:ENSG00000131446:ENSG00000079691:ENSG00000146112:ENSG00000137404:ENSG00000137337:ENSG00000204344:ENSG00000244731:ENSG00000235863:ENSG00000227057:ENSG00000237441:ENSG00000231925:ENSG00000124772:ENSG00000118432:ENSG00000198945:ENSG00000118503:ENSG00000112406:ENSG00000131018:ENSG00000213066:ENSG00000122644:ENSG00000153814:ENSG00000241644:ENSG00000152926:ENSG00000213462:ENSG00000126522:ENSG00000127920:ENSG00000146834:ENSG00000160813:ENSG00000005249:ENSG00000196937:ENSG00000091704:ENSG00000106484:ENSG00000182158:ENSG00000181444:ENSG00000196329:ENSG00000214106:ENSG00000146918:ENSG00000117868:ENSG00000168546:ENSG00000120910:ENSG00000147465:ENSG00000023287:ENSG00000035681:ENSG00000121039:ENSG00000164949:ENSG00000120526:ENSG00000120533:ENSG00000129295:ENSG00000165071:ENSG00000160932:ENSG00000080608:ENSG00000044459:ENS

						G00000213694:ENSG00000127080:ENSG00000136925:ENSG00000136826:ENSG00000196152:ENSG00000136840:ENSG00000171169:ENSG00000148339:ENSG00000170835:ENSG00000198113
DEG. down	Brain_Substantia_nigra	7223	466	1.44093864819655e-19	7.78106870026139e-18	ENSG00000158286:ENSG00000116237:ENSG00000158292:ENSG00000171824:ENSG00000198793:ENSG00000120942:ENSG00000162458:ENSG00000065526:ENSG00000116809:ENSG00000204178:ENSG00000084652:ENSG00000160051:ENSG00000160094:ENSG00000117395:ENSG00000066056:ENSG00000117399:ENSG00000159479:ENSG00000198198:ENSG00000117472:ENSG00000117862:ENSG00000198841:ENSG00000117114:ENSG00000117133:ENSG00000117155:ENSG00000142867:ENSG00000117543:ENSG00000162775:ENSG00000168679:ENSG00000085465:ENSG00000116455:ENSG00000163349:ENSG00000197323:ENSG00000143457:ENSG00000163131:ENSG00000143387:ENSG00000143437:ENSG00000143379:ENSG00000143412:ENSG00000143363:ENSG00000143434:ENSG00000163156:ENSG00000163155:ENSG00000160767:ENSG00000176444:ENSG00000160753:ENSG00000116539:ENSG00000125459:ENSG00000163374:ENSG00000132676:ENSG00000116580:ENSG00000143622:ENSG00000132680:ENSG00000160789:ENSG00000196189:ENSG00000160783:ENSG00000163472:ENSG00000198715:ENSG00000187800:ENSG00000132694:ENSG00000120341:ENSG00000186283:ENSG00000143322:ENSG00000057252:ENSG00000122176:ENSG00000182004:ENSG00000143801:ENSG00000163050:ENSG00000162885:ENSG00000116962:ENSG00000162714:ENSG00000107929:ENSG00000134463:ENSG00000165997:ENSG00000108094:ENSG00000188234:ENSG00000138134:ENSG00000107796:ENSG00000026103:ENSG00000165338:ENSG00000059573:ENSG00000198873:ENSG00000166478:ENSG00000166483:ENSG00000198730:ENSG00000149150:ENSG00000110025:ENSG00000162300:ENSG00000149823:ENSG00000174276:ENSG00000162298:ENSG00000149792:ENSG00000173653:ENSG00000173621:ENSG00000175567:ENSG00000062282:ENSG00000137492:ENSG00000137672:ENSG00000137693:ENSG00000149292:ENSG00000076053:ENSG00000076043:ENSG00000166250:ENSG00000082805:ENSG00000111671:ENSG00000111679:ENSG00000126749:ENSG00000111684:ENSG00000182326:ENSG00000013588:ENSG00000134531:ENSG00000050426:ENSG00000123338:ENSG00000123360:ENSG00000127314:ENSG00000166130:ENSG00000120868:ENSG00000235162:ENSG00000074590:ENSG00000174989:ENSG00000111725:ENSG00000188735:ENSG00000150990:ENSG00000177169:ENSG00000177084:ENSG00000176915:ENSG00000127863:ENSG00000102743:ENSG00000136167:ENSG00000102531:ENSG00000123179:ENSG00000102753:ENSG00000136104:ENSG00000118939:ENSG00000136153:ENSG00000088451:ENSG00000065150:ENSG00000134901:ENSG00000139826:ENSG00000102524:ENSG00000100889:ENSG00000100897:ENSG00000092140:ENSG00000092108:ENSG00000150527:ENSG00000165355:ENSG00000179476:ENSG00000185246:ENSG00000139926:ENSG00000197930:ENSG00000119599:ENSG00000165861:ENSG00000119707:ENSG00000100767:ENSG00000133961:ENSG00000165417:ENSG00000165934:ENSG00000153485:ENSG00000156414:ENSG00000140157:ENSG00000021776:ENSG00000198146:ENSG00000174197:ENSG00000137802:ENSG00000243708:ENSG00000243789:ENSG00000181827:ENSG00000103707:ENSG00000136378:ENSG00000140406:ENSG00000140511:ENSG00000140471:ENSG00000008710:ENSG00000183426:ENSG00000179889:ENSG00000157045:ENSG00000085721:ENSG00000157106:ENSG00000180035:ENSG00000180096:ENSG00000179958:ENSG00000140691:ENSG00000140682:ENSG00000102910:ENSG00000196470:ENSG00000102921:ENSG00000005194:ENSG00000102978:ENSG00000072736:ENSG00000103066:ENSG00000103064:ENSG00000132600:ENSG00000103056:ENSG00000158717:ENSG00000167522:ENSG00000197912:ENSG00000127804:ENSG00000197417:ENSG00000005100:ENSG00000072849:ENSG00000108641:ENSG00000166484:ENSG00000166482:ENSG00000142494:ENSG00000067191:ENSG00000068079:ENSG00000184988:ENSG00000182963:ENSG00000225190:ENSG00000141279:ENSG00000011258:ENSG00000011260:ENSG00000153933:ENSG00000121060:ENSG00000108423:ENSG00000108443:ENSG00000189050:ENSG00000198265:ENSG00000180616:ENSG00000141577:ENSG00000167302:ENSG00000185504:ENSG00000182446:ENSG00000017797:ENSG00000154845:ENSG00000154889:ENSG00000141401:ENSG00000168234:ENSG00000141384:ENSG00000152223:ENSG00000130856:ENSG00000071564:ENSG00000141985:ENSG00000167670:ENSG00000205744:ENSG00000171105:ENSG00000104880:ENSG00000104957:ENSG00000132017:ENSG00000132005:ENSG00000123146:ENSG00000123136:ENSG00000127527:ENSG00000237440:ENSG00000104835:ENSG00000128626:ENSG00000269190:ENSG00000160570:ENSG00000167625:ENSG00000105722:ENSG00000079432:ENSG00000105427:ENSG00000187244:ENSG00000105483:ENSG00000105447:ENSG00000142544:ENSG00000105383:ENSG00000196214:ENSG00000198464:ENSG00000167766:ENSG00000213020:ENSG00000115761:ENSG00000055917:ENSG00000084676:ENSG00000171103:ENSG00000119866:ENSG00000162928:ENSG00000082898:ENSG00000075292:ENSG00000144048:ENSG00000114978:ENSG00000065911:ENSG00000005448:ENSG00000115604:ENSG00000153107:ENSG00000153208:ENSG00000153214:ENSG00000115963:ENSG00000153250:ENSG00000118260:ENSG00000115457:ENSG00000115461:ENSG00000144579:ENSG00000132326:ENSG00000162804:ENSG00000088876:ENSG00000125779:ENSG00000132646:ENSG00000188559:ENSG00000124107:ENSG00000124228:ENSG00000124201:ENSG00000125520:ENSG00000198276:ENSG00000155313:ENSG00000157617:ENSG00000160190:ENSG00000160284:ENSG00000100342:ENSG00000198951:ENSG00000177096:ENSG00000100197:ENSG00000242247:ENSG00000077942:ENSG00000205643:ENSG00000075234:ENSG00000196576:ENSG00000100258:ENSG00000025770:ENSG00000130489:ENSG00000025708:ENSG00000177989:ENSG00000205560:ENSG00000100288:ENSG00000100299:ENSG00000251322:ENSG00000070950:ENSG00000134077:ENSG00000144560:ENSG00000144712:ENSG00000163513:ENSG00000170293:ENSG00000182606:ENSG00000182983:ENSG00000214706:ENSG00000243477:ENSG00000114378:ENSG00000068001:ENSG00000068028:ENSG00000114395:ENSG00000126062:ENSG00000180376:ENSG00000163946:ENSG00000114861:ENSG00000144736:ENSG00000163320:ENSG00000175105:ENSG00000179021:ENSG00000184500:ENSG00000169379:ENSG00000114446:ENSG00000163607:ENSG00000163608:ENSG00000144843:ENSG00000172780:ENSG00000240682:ENSG00000034533:ENSG00000113971:ENSG00000081307:ENSG00000138231:ENSG00000114098:ENSG00000175110:ENSG00000184432:ENSG00000155903:ENSG0000017506

						6:ENSG00000114127:ENSG00000114698:ENSG00000169282:ENSG00000075420:ENSG00000161202:ENSG00000145014:ENSG00000041802:ENSG00000163975:ENSG00000013288:ENSG00000109689:ENSG00000145246:ENSG00000109171:ENSG00000173085:ENSG00000205403:ENSG00000164128:ENSG00000129187:ENSG00000113360:ENSG00000164190:ENSG00000171522:ENSG00000132357:ENSG00000067248:ENSG00000039123:ENSG00000086200:ENSG00000164331:ENSG00000164338:ENSG00000164180:ENSG00000175471:ENSG00000253159:ENSG00000131504:ENSG00000171720:ENSG00000197948:ENSG00000120318:ENSG00000113721:ENSG00000135074:ENSG00000113272:ENSG00000155858:ENSG00000048140:ENSG00000131446:ENSG00000196670:ENSG00000124535:ENSG00000124787:ENSG00000079691:ENSG00000146112:ENSG00000137404:ENSG00000137337:ENSG00000137331:ENSG00000204344:ENSG00000244731:ENSG00000223501:ENSG00000235863:ENSG00000227057:ENSG00000237441:ENSG00000231925:ENSG00000124762:ENSG00000118432:ENSG00000198945:ENSG00000118503:ENSG00000112406:ENSG00000131018:ENSG00000213066:ENSG00000184786:ENSG00000130023:ENSG00000122644:ENSG00000153814:ENSG00000168303:ENSG00000152926:ENSG00000213462:ENSG00000126522:ENSG00000049541:ENSG00000127920:ENSG00000105829:ENSG00000121716:ENSG00000085514:ENSG00000146834:ENSG00000160813:ENSG00000196937:ENSG00000091704:ENSG00000106484:ENSG00000158623:ENSG00000131558:ENSG00000122786:ENSG00000182158:ENSG00000181444:ENSG00000196329:ENSG00000214106:ENSG00000146918:ENSG00000117868:ENSG00000120910:ENSG00000129691:ENSG00000023287:ENSG00000254087:ENSG00000035681:ENSG00000121039:ENSG00000164949:ENSG00000120526:ENSG00000120533:ENSG00000196378:ENSG00000080608:ENSG00000044459:ENSG00000213694:ENSG00000127080:ENSG00000136925:ENSG00000136826:ENSG00000196152:ENSG00000148356:ENSG00000136840:ENSG00000171169:ENSG00000148339:ENSG00000148335:ENSG00000148308:ENSG00000170835:ENSG00000198113
DEG. down	Breast_Mammary_Tissue	1007	67	0.000766663446611817	0.0413998261170381	ENSG00000097021:ENSG00000160050:ENSG00000168389:ENSG00000117399:ENSG00000117155:ENSG00000153904:ENSG00000175984:ENSG00000132718:ENSG00000160781:ENSG00000173110:ENSG00000075073:ENSG00000120057:ENSG00000203867:ENSG00000146670:ENSG00000175564:ENSG00000154127:ENSG00000122966:ENSG00000184992:ENSG00000127863:ENSG00000136153:ENSG00000139914:ENSG00000168952:ENSG00000180998:ENSG00000006432:ENSG00000156414:ENSG00000170113:ENSG00000186198:ENSG00000103888:ENSG00000180209:ENSG00000185669:ENSG00000188522:ENSG00000128482:ENSG00000142494:ENSG00000067191:ENSG00000108830:ENSG00000073670:ENSG00000153822:ENSG00000141540:ENSG00000141469:ENSG00000197971:ENSG00000205744:ENSG00000099308:ENSG00000028277:ENSG00000163803:ENSG00000119866:ENSG00000115607:ENSG00000144485:ENSG00000125966:ENSG00000184792:ENSG00000008735:ENSG00000144712:ENSG00000163517:ENSG00000004838:ENSG00000007402:ENSG00000154917:ENSG00000114279:ENSG00000080493:ENSG00000145287:ENSG00000215218:ENSG00000164620:ENSG00000135074:ENSG00000124772:ENSG00000086730:ENSG00000177679:ENSG00000168546:ENSG00000104267:ENSG00000148411
DEG. down	Cells_Cultured_fibroblasts	2173	123	0.00367041420253149	0.1982023669367	ENSG00000158286:ENSG00000187942:ENSG00000197147:ENSG00000069712:ENSG00000117569:ENSG00000085465:ENSG00000175984:ENSG00000163131:ENSG00000143412:ENSG00000143409:ENSG00000143363:ENSG00000143434:ENSG00000143630:ENSG00000196189:ENSG00000160781:ENSG00000163050:ENSG00000134463:ENSG00000095794:ENSG00000149809:ENSG00000175567:ENSG00000149292:ENSG00000023171:ENSG00000177169:ENSG00000136167:ENSG00000102445:ENSG00000102524:ENSG00000179476:ENSG00000100767:ENSG00000165943:ENSG00000243708:ENSG00000243789:ENSG00000103710:ENSG00000140511:ENSG00000185418:ENSG00000102879:ENSG00000180096:ENSG00000158717:ENSG00000196689:ENSG00000128482:ENSG00000142494:ENSG00000067191:ENSG00000153933:ENSG00000108423:ENSG00000141540:ENSG00000078687:ENSG00000167302:ENSG00000141401:ENSG00000081913:ENSG00000197971:ENSG00000171105:ENSG00000132024:ENSG00000099308:ENSG00000105427:ENSG00000079435:ENSG00000187244:ENSG00000105483:ENSG00000176293:ENSG00000197872:ENSG00000084676:ENSG00000116039:ENSG00000114993:ENSG00000115461:ENSG00000144485:ENSG00000132326:ENSG00000188559:ENSG00000124102:ENSG00000124107:ENSG00000203896:ENSG00000183628:ENSG00000184792:ENSG00000100342:ENSG00000100321:ENSG00000183172:ENSG00000205593:ENSG00000177989:ENSG00000205560:ENSG00000251322:ENSG00000163517:ENSG00000170293:ENSG00000182983:ENSG00000114378:ENSG00000144749:ENSG00000114861:ENSG00000144843:ENSG00000154917:ENSG00000080493:ENSG00000205403:ENSG00000164620:ENSG00000120318:ENSG00000113742:ENSG00000137266:ENSG00000078401:ENSG00000244731:ENSG00000235863:ENSG00000237441:ENSG00000164530:ENSG00000112406:ENSG00000120278:ENSG00000131018:ENSG00000184786:ENSG00000105855:ENSG00000241644:ENSG00000152926:ENSG00000189143:ENSG00000177679:ENSG00000135218:ENSG00000121716:ENSG00000085514:ENSG00000078487:ENSG00000166924:ENSG00000005249:ENSG00000106484:ENSG00000146966:ENSG00000181444:ENSG00000147465:ENSG00000254087:ENSG00000121039:ENSG00000196378:ENSG00000070159:ENSG00000148356:ENSG00000167106:ENSG00000148339:ENSG00000170835
DEG. down	Cells_EBV-transformed_lymphocytes	2398	146	6.99040222106805e-05	0.00377481719937675	ENSG00000158292:ENSG00000158747:ENSG00000187942:ENSG00000116525:ENSG00000160094:ENSG00000134686:ENSG00000066056:ENSG00000162616:ENSG00000117569:ENSG00000116396:ENSG00000168679:ENSG00000085465:ENSG00000143387:ENSG00000143412:ENSG00000143409:ENSG00000197084:ENSG00000143537:ENSG00000143630:ENSG00000160753:ENSG00000242252:ENSG00000160781:ENSG00000132694:ENSG00000143226:ENSG00000143322:ENSG00000174307:ENSG00000143801:ENSG00000163050:ENSG00000116962:ENSG00000015171:ENSG00000134463:ENSG00000148468:ENSG00000107738:ENSG00000107796:ENSG00000119927:ENSG00000184545:ENSG00000166311:ENSG00000166313:ENSG00000162174:ENSG00000110025:ENSG00000173599:ENSG00000076043:ENSG00000154127:ENSG00000082805:ENSG00000182326:ENSG00000185432:ENSG00000110911:ENSG00000110925:ENSG00000123360:ENSG00000139410:ENSG00000177169:ENSG00000183722:ENSG00000178695:ENSG00000129493:ENSG00000179476:ENSG00000139926:ENSG00000100767:ENSG00000243708:ENSG00000

						136378:ENSG00000140511:ENSG00000008710:ENSG00000183426:ENSG00000179965:ENSG00000140682:ENSG00000103066:ENSG00000103056:ENSG00000196689:ENSG00000072134:ENSG00000108641:ENSG00000166484:ENSG00000142494:ENSG00000067191:ENSG00000108828:ENSG00000225190:ENSG00000198933:ENSG00000189050:ENSG00000141540:ENSG00000078687:ENSG00000154845:ENSG00000141401:ENSG00000104883:ENSG00000123159:ENSG00000262484:ENSG00000269190:ENSG00000105429:ENSG00000079435:ENSG00000063127:ENSG00000167766:ENSG00000153208:ENSG00000115963:ENSG00000144283:ENSG00000153250:ENSG00000115457:ENSG00000153832:ENSG00000144485:ENSG00000162804:ENSG00000198959:ENSG00000124107:ENSG00000100321:ENSG00000177096:ENSG00000100197:ENSG00000163517:ENSG00000163513:ENSG00000068001:ENSG00000126062:ENSG00000144749:ENSG00000114446:ENSG00000113971:ENSG00000175066:ENSG00000075420:ENSG00000145014:ENSG00000013288:ENSG00000109321:ENSG00000123739:ENSG00000171522:ENSG00000132357:ENSG00000067113:ENSG00000253159:ENSG00000240184:ENSG00000131507:ENSG00000113721:ENSG00000048140:ENSG00000137266:ENSG00000204344:ENSG00000237441:ENSG00000198663:ENSG00000112406:ENSG00000120278:ENSG00000131018:ENSG00000112096:ENSG00000122644:ENSG00000152926:ENSG00000169902:ENSG00000085563:ENSG00000127920:ENSG00000075790:ENSG00000106484:ENSG00000122786:ENSG00000023287:ENSG00000121039:ENSG00000104267:ENSG00000164949:ENSG00000185189:ENSG00000196378:ENSG00000044459:ENSG00000170835:ENSG00000148411
DEG. down	Cervix_Ectocervix	2	0	1	1	
DEG. down	Cervix_Endocervix	5	3	0.000825744047233424	0.0445901785506049	ENSG00000172137:ENSG00000141576:ENSG00000135218
DEG. down	Colon_Sigmoid	1685	107	0.000145765025951683	0.00787131140139089	ENSG00000179178:ENSG00000253313:ENSG00000117399:ENSG00000162366:ENSG00000134709:ENSG00000197147:ENSG00000134215:ENSG00000175984:ENSG00000143412:ENSG00000163354:ENSG00000196189:ENSG00000163472:ENSG00000134463:ENSG00000107736:ENSG00000119927:ENSG00000006071:ENSG00000135373:ENSG00000019505:ENSG00000162174:ENSG00000146670:ENSG00000149809:ENSG00000173599:ENSG00000062282:ENSG00000110777:ENSG00000154127:ENSG00000111679:ENSG00000235162:ENSG00000074590:ENSG00000139410:ENSG00000184992:ENSG00000127863:ENSG00000102743:ENSG00000136167:ENSG00000102445:ENSG00000136153:ENSG00000102524:ENSG00000100889:ENSG00000156414:ENSG00000140511:ENSG00000180209:ENSG00000103067:ENSG00000185669:ENSG00000188522:ENSG00000142494:ENSG00000067191:ENSG00000141750:ENSG00000123700:ENSG00000196169:ENSG00000141469:ENSG00000197971:ENSG00000205744:ENSG00000104833:ENSG00000129354:ENSG00000099308:ENSG00000179751:ENSG00000105369:ENSG00000105427:ENSG00000079435:ENSG00000142273:ENSG00000163803:ENSG00000116039:ENSG00000115602:ENSG00000115604:ENSG00000115607:ENSG00000162804:ENSG00000188559:ENSG00000125966:ENSG00000124102:ENSG00000183628:ENSG00000184792:ENSG00000100197:ENSG00000205560:ENSG00000170293:ENSG00000179564:ENSG00000114378:ENSG00000114395:ENSG00000007402:ENSG00000169282:ENSG00000172578:ENSG00000163898:ENSG00000114279:ENSG00000080493:ENSG00000169429:ENSG00000163739:ENSG00000145287:ENSG00000185477:ENSG00000164128:ENSG00000109466:ENSG00000132357:ENSG00000181751:ENSG00000253159:ENSG00000131504:ENSG00000164620:ENSG00000079691:ENSG00000124772:ENSG00000243955:ENSG00000135525:ENSG00000189143:ENSG00000086730:ENSG00000177679:ENSG00000166924:ENSG00000106484:ENSG00000254087:ENSG00000104267:ENSG00000127080:ENSG00000070159:ENSG00000198113
DEG. down	Colon_Transverse	1588	107	1.37086360583181e-05	0.000740266347149175	ENSG00000160050:ENSG00000116525:ENSG00000197147:ENSG00000069712:ENSG00000175984:ENSG00000092621:ENSG00000143412:ENSG00000143434:ENSG00000196189:ENSG00000163472:ENSG00000187800:ENSG00000173110:ENSG00000203747:ENSG00000134463:ENSG00000107736:ENSG00000119927:ENSG00000187486:ENSG00000006071:ENSG00000165905:ENSG00000149809:ENSG00000173599:ENSG00000175564:ENSG00000062282:ENSG00000149313:ENSG00000154127:ENSG00000010626:ENSG00000134532:ENSG00000123360:ENSG00000074590:ENSG00000188735:ENSG00000127863:ENSG00000102524:ENSG00000139914:ENSG00000100473:ENSG00000006432:ENSG00000198146:ENSG00000137802:ENSG00000243789:ENSG00000197535:ENSG00000103888:ENSG00000140511:ENSG00000103066:ENSG00000108641:ENSG00000142494:ENSG00000067191:ENSG00000141750:ENSG00000108830:ENSG00000154217:ENSG00000180616:ENSG00000141540:ENSG00000078687:ENSG00000168234:ENSG00000141469:ENSG00000197971:ENSG00000132000:ENSG00000262484:ENSG00000105427:ENSG00000079435:ENSG00000189377:ENSG00000063127:ENSG00000163803:ENSG00000188687:ENSG00000005448:ENSG00000115607:ENSG00000163040:ENSG00000144283:ENSG00000018280:ENSG00000153832:ENSG00000162804:ENSG00000184792:ENSG00000100321:ENSG00000075275:ENSG00000205593:ENSG00000008735:ENSG00000144712:ENSG00000170293:ENSG00000007402:ENSG00000114670:ENSG00000129048:ENSG00000154917:ENSG00000158186:ENSG00000169282:ENSG00000174899:ENSG00000169429:ENSG00000164128:ENSG00000109466:ENSG00000113361:ENSG00000132357:ENSG00000152669:ENSG00000175471:ENSG00000253159:ENSG00000240184:ENSG00000164620:ENSG00000113742:ENSG00000124772:ENSG00000132429:ENSG00000131018:ENSG00000105855:ENSG00000152926:ENSG00000213462:ENSG00000177679:ENSG00000085514:ENSG00000078487:ENSG00000166924:ENSG00000131773:ENSG00000127080:ENSG00000170835
DEG. down	Esophagus_Gastroesophageal_Junction	1570	100	0.000215198631900152	0.0116207261226082	ENSG00000168389:ENSG00000117472:ENSG00000153904:ENSG00000197147:ENSG00000069712:ENSG00000134215:ENSG00000175984:ENSG00000092621:ENSG00000143412:ENSG00000203786:ENSG00000163354:ENSG00000196189:ENSG00000160781:ENSG00000163472:ENSG00000173110:ENSG00000203747:ENSG00000134463:ENSG00000119927:ENSG00000187486:ENSG00000162174:ENSG00000149809:ENSG00000173599:ENSG00000062282:ENSG00000111679:ENSG000002

						35162:ENSG00000139410:ENSG00000184992:ENSG00000102743:ENSG00000136167:ENSG00000102445:ENSG00000118939:ENSG00000136153:ENSG0000013991 4:ENSG00000006432:ENSG00000156414:ENSG00000102879:ENSG00000180096:ENSG00000103066:ENSG00000103056:ENSG00000172137:ENSG00000181350:EN SG00000188522:ENSG00000067191:ENSG00000108830:ENSG00000073670:ENSG00000180616:ENSG00000141576:ENSG00000197971:ENSG00000205744:ENSG0 0000104833:ENSG00000132016:ENSG00000099308:ENSG00000262484:ENSG00000179751:ENSG00000079462:ENSG00000079435:ENSG00000189377:ENSG00000 079385:ENSG00000115756:ENSG00000119866:ENSG00000116039:ENSG00000115607:ENSG00000125966:ENSG00000124102:ENSG00000160190:ENSG000001836 28:ENSG00000184792:ENSG00000100197:ENSG00000075275:ENSG00000008735:ENSG00000170293:ENSG00000168032:ENSG00000114395:ENSG00000007402:E NSG00000169282:ENSG00000080493:ENSG00000169429:ENSG00000163739:ENSG00000173085:ENSG00000185477:ENSG00000164620:ENSG00000137266:ENSG 00000124772:ENSG00000132429:ENSG00000135525:ENSG00000091831:ENSG00000026297:ENSG00000189143:ENSG00000086730:ENSG00000177679:ENSG0000 0185955:ENSG00000166924:ENSG00000106484:ENSG00000158623:ENSG00000254087:ENSG00000104267:ENSG00000129295:ENSG00000127080:ENSG00000070 159:ENSG00000170835
DEG. down	Esophagus_M ucosa	3605	219	9.72523772 442419e-07	5.25162837 118906e-05	ENSG00000171819:ENSG00000187942:ENSG00000160050:ENSG00000160051:ENSG00000127603:ENSG00000066056:ENSG00000162367:ENSG00000162616:ENS G00000153904:ENSG00000171488:ENSG00000069712:ENSG00000117569:ENSG00000162694:ENSG00000168679:ENSG00000143434:ENSG00000163154:ENSG00 000132718:ENSG00000116584:ENSG00000160781:ENSG00000187800:ENSG00000143226:ENSG00000173110:ENSG00000203747:ENSG00000120341:ENSG000001 43322:ENSG00000057252:ENSG00000163050:ENSG00000162714:ENSG00000198879:ENSG00000148468:ENSG00000241058:ENSG00000095794:ENSG0000010773 6:ENSG00000165338:ENSG00000120057:ENSG00000119927:ENSG00000198873:ENSG00000184545:ENSG00000166313:ENSG00000187486:ENSG00000149150:EN SG00000124935:ENSG00000162174:ENSG00000168060:ENSG00000173621:ENSG00000175567:ENSG00000137492:ENSG00000180425:ENSG00000154127:ENSG0 0000064309:ENSG00000010626:ENSG00000111674:ENSG00000123094:ENSG00000110925:ENSG00000123360:ENSG00000166130:ENSG00000111647:ENSG00000 074590:ENSG00000139410:ENSG00000122966:ENSG00000188735:ENSG00000139505:ENSG00000133110:ENSG00000136167:ENSG00000102531:ENSG000001786 95:ENSG00000134901:ENSG00000102524:ENSG00000139914:ENSG00000168952:ENSG00000087301:ENSG00000100767:ENSG00000165943:ENSG00000197535:E NSG00000136378:ENSG00000140600:ENSG00000185418:ENSG00000008710:ENSG00000180035:ENSG00000179965:ENSG00000103066:ENSG00000103064:ENSG 00000132600:ENSG00000103056:ENSG00000185669:ENSG00000158717:ENSG00000196689:ENSG00000029725:ENSG00000108641:ENSG00000128482:ENSG0000 0142494:ENSG00000067191:ENSG00000141750:ENSG00000068079:ENSG00000108830:ENSG00000184988:ENSG00000182963:ENSG00000008294:ENSG00000154 217:ENSG00000141540:ENSG00000078687:ENSG00000197971:ENSG00000171105:ENSG00000167775:ENSG00000065989:ENSG00000123146:ENSG00000123143: ENSG00000099308:ENSG00000237440:ENSG00000269190:ENSG00000028277:ENSG00000105429:ENSG00000079435:ENSG00000105483:ENSG00000063127:ENS G00000105383:ENSG00000176293:ENSG00000115705:ENSG00000115756:ENSG00000197872:ENSG00000143867:ENSG00000163803:ENSG00000005448:ENSG00 000144218:ENSG00000115602:ENSG00000115604:ENSG00000115607:ENSG00000153208:ENSG00000163040:ENSG00000018280:ENSG00000153832:ENSG000001 98612:ENSG00000144485:ENSG00000162804:ENSG00000196209:ENSG00000101290:ENSG00000124201:ENSG00000160284:ENSG00000183628:ENSG0000018479 2:ENSG00000138944:ENSG00000205593:ENSG00000008735:ENSG00000251322:ENSG00000144712:ENSG00000170293:ENSG00000170011:ENSG00000114378:EN SG00000180376:ENSG00000144749:ENSG00000169379:ENSG00000114446:ENSG00000144843:ENSG00000172780:ENSG00000114670:ENSG00000113971:ENSG0 0000154917:ENSG00000158163:ENSG00000158186:ENSG00000114698:ENSG00000163762:ENSG00000169282:ENSG00000075420:ENSG00000172578:ENSG00000 152953:ENSG00000013288:ENSG00000109689:ENSG00000169429:ENSG00000185477:ENSG00000164128:ENSG00000113361:ENSG00000171522:ENSG000001754 71:ENSG00000181751:ENSG00000168916:ENSG00000173926:ENSG00000064651:ENSG00000253159:ENSG00000120318:ENSG00000135074:ENSG00000078401:E NSG00000137337:ENSG00000244731:ENSG00000124772:ENSG00000164530:ENSG00000118432:ENSG00000198945:ENSG00000118503:ENSG00000131018:ENSG 00000153814:ENSG00000241644:ENSG00000165215:ENSG00000086730:ENSG00000135218:ENSG00000085563:ENSG00000127920:ENSG00000085514:ENSG0000 0078487:ENSG00000166924:ENSG00000005249:ENSG00000106477:ENSG00000106484:ENSG00000146966:ENSG00000159763:ENSG00000181444:ENSG00000196 329:ENSG00000154319:ENSG00000120910:ENSG00000147601:ENSG00000164949:ENSG00000129295:ENSG00000165071:ENSG00000131773:ENSG00000196378: ENSG00000044459:ENSG00000213694:ENSG00000167081:ENSG00000148335:ENSG00000148411
DEG. down	Esophagus_M uscularis	1775	98	0.01682719 2119108	0.90866837 4431834	ENSG00000168389:ENSG00000117472:ENSG00000197147:ENSG00000069712:ENSG00000117525:ENSG00000134215:ENSG00000175984:ENSG00000143412:ENS G00000163354:ENSG00000196189:ENSG00000160781:ENSG00000163472:ENSG00000173110:ENSG00000134463:ENSG00000119927:ENSG00000205869:ENSG00 000187486:ENSG00000162174:ENSG00000149809:ENSG00000173599:ENSG00000062282:ENSG00000111679:ENSG00000013588:ENSG00000134532:ENSG000002 35162:ENSG00000184992:ENSG00000102743:ENSG00000136167:ENSG00000102445:ENSG00000136153:ENSG00000102524:ENSG00000100889:ENSG0000013991 4:ENSG00000100473:ENSG00000006432:ENSG00000102879:ENSG00000180209:ENSG00000180096:ENSG00000103066:ENSG00000103056:ENSG00000172137:EN SG00000181350:ENSG00000108641:ENSG00000067191:ENSG00000073670:ENSG00000180616:ENSG00000141576:ENSG00000197971:ENSG00000205744:ENSG0 0000104833:ENSG00000132016:ENSG00000099308:ENSG00000262484:ENSG00000079462:ENSG00000079435:ENSG00000079385:ENSG00000115756:ENSG00000 119866:ENSG00000115607:ENSG00000153253:ENSG00000162804:ENSG00000124102:ENSG00000160190:ENSG00000183628:ENSG00000184792:ENSG000001001

						97:ENSG00000075275:ENSG00000205593:ENSG00000170293:ENSG00000168032:ENSG00000114395:ENSG00000007402:ENSG00000169282:ENSG00000080493:ENSG00000169429:ENSG00000163739:ENSG00000173085:ENSG00000185477:ENSG00000164128:ENSG00000164620:ENSG00000079691:ENSG00000124772:ENSG00000198945:ENSG00000135525:ENSG00000026297:ENSG00000189143:ENSG00000086730:ENSG00000177679:ENSG00000185955:ENSG00000166924:ENSG00000106484:ENSG00000158623:ENSG00000254087:ENSG00000104267:ENSG00000129295:ENSG00000127080:ENSG00000070159:ENSG00000198113
DEG. down	Fallopian_Tube	1	0	1	1	
DEG. down	Heart_Atrial_Appendage	8488	518	3.92898194803006e-17	2.12165025193623e-15	ENSG00000097021:ENSG00000171824:ENSG00000198793:ENSG00000120942:ENSG00000065526:ENSG00000116809:ENSG00000117616:ENSG00000204178:ENSG00000157978:ENSG00000084652:ENSG00000160051:ENSG00000116525:ENSG00000160094:ENSG00000168389:ENSG00000117395:ENSG00000066322:ENSG00000159479:ENSG00000198198:ENSG00000162367:ENSG00000117862:ENSG00000198841:ENSG00000134717:ENSG00000116205:ENSG00000134709:ENSG00000117155:ENSG00000162642:ENSG00000142867:ENSG00000153904:ENSG00000197147:ENSG00000171488:ENSG00000069712:ENSG00000117525:ENSG00000117569:ENSG00000162694:ENSG00000134215:ENSG00000116396:ENSG00000085465:ENSG00000163349:ENSG00000197323:ENSG00000175984:ENSG00000213281:ENSG00000052723:ENSG00000092621:ENSG00000143457:ENSG00000163131:ENSG00000143437:ENSG00000143379:ENSG00000143412:ENSG00000163154:ENSG00000163156:ENSG00000160767:ENSG00000176444:ENSG00000143630:ENSG00000160753:ENSG00000116539:ENSG00000125459:ENSG00000163374:ENSG00000116580:ENSG00000132718:ENSG00000143622:ENSG00000132680:ENSG00000116584:ENSG00000160803:ENSG00000196189:ENSG00000160781:ENSG00000163472:ENSG00000198715:ENSG00000132694:ENSG00000143226:ENSG00000152332:ENSG00000186283:ENSG00000143322:ENSG00000057252:ENSG00000177888:ENSG00000182004:ENSG00000143801:ENSG00000162885:ENSG00000162714:ENSG00000107929:ENSG00000198879:ENSG00000148468:ENSG00000241058:ENSG00000099246:ENSG00000108094:ENSG00000188234:ENSG00000165476:ENSG00000075073:ENSG00000107736:ENSG00000107738:ENSG00000026103:ENSG00000165338:ENSG00000059573:ENSG00000155256:ENSG00000119927:ENSG00000165832:ENSG00000268403:ENSG00000166478:ENSG00000198730:ENSG00000006071:ENSG00000110442:ENSG00000121680:ENSG00000149150:ENSG00000156587:ENSG00000124935:ENSG00000162174:ENSG00000110025:ENSG00000168061:ENSG00000168060:ENSG00000162300:ENSG00000174276:ENSG00000162298:ENSG00000149792:ENSG00000173653:ENSG00000173599:ENSG00000173621:ENSG00000175567:ENSG00000062282:ENSG00000137492:ENSG00000149313:ENSG00000149292:ENSG00000180425:ENSG00000076053:ENSG00000064309:ENSG00000010219:ENSG00000111667:ENSG00000111671:ENSG00000111679:ENSG00000126749:ENSG00000111684:ENSG00000110911:ENSG00000050426:ENSG00000110925:ENSG00000135457:ENSG00000123338:ENSG00000123360:ENSG00000127314:ENSG00000120868:ENSG00000111647:ENSG00000235162:ENSG00000135094:ENSG00000174989:ENSG00000111725:ENSG00000188735:ENSG00000150990:ENSG00000184992:ENSG00000177084:ENSG00000176915:ENSG00000139505:ENSG00000102743:ENSG00000136167:ENSG00000102531:ENSG00000123179:ENSG00000136104:ENSG00000118939:ENSG00000178695:ENSG00000088451:ENSG00000152749:ENSG00000139826:ENSG00000100889:ENSG00000100897:ENSG00000092140:ENSG00000092108:ENSG00000129480:ENSG00000150527:ENSG00000165355:ENSG00000179476:ENSG00000179454:ENSG00000198718:ENSG00000185246:ENSG00000139926:ENSG00000087301:ENSG00000180998:ENSG00000197930:ENSG00000119599:ENSG00000165861:ENSG00000119707:ENSG00000080815:ENSG00000100767:ENSG00000133961:ENSG00000165417:ENSG00000165934:ENSG00000140157:ENSG00000170113:ENSG00000128731:ENSG00000021776:ENSG00000198146:ENSG00000174197:ENSG00000137802:ENSG00000243708:ENSG00000243789:ENSG00000197535:ENSG00000128989:ENSG00000181827:ENSG00000140262:ENSG00000186198:ENSG00000103888:ENSG00000117899:ENSG00000140406:ENSG00000140511:ENSG00000140471:ENSG00000183426:ENSG00000157045:ENSG00000085721:ENSG00000157106:ENSG00000102879:ENSG00000169217:ENSG00000169221:ENSG00000180035:ENSG00000180096:ENSG00000179965:ENSG00000140691:ENSG00000102910:ENSG00000196470:ENSG00000102921:ENSG00000072736:ENSG00000103061:ENSG00000132600:ENSG00000103056:ENSG00000172137:ENSG00000185669:ENSG00000158717:ENSG00000167515:ENSG00000167522:ENSG00000197912:ENSG00000127804:ENSG00000197417:ENSG00000029725:ENSG00000005100:ENSG00000072849:ENSG00000132514:ENSG00000141127:ENSG00000108641:ENSG00000166484:ENSG00000128482:ENSG00000067191:ENSG00000141750:ENSG00000068079:ENSG00000108830:ENSG00000012048:ENSG00000184988:ENSG00000225190:ENSG00000141279:ENSG00000008294:ENSG00000011258:ENSG00000011260:ENSG00000153933:ENSG00000121060:ENSG00000108423:ENSG00000108443:ENSG00000189050:ENSG00000198265:ENSG00000197170:ENSG00000154217:ENSG00000123700:ENSG00000141576:ENSG00000185262:ENSG00000078687:ENSG00000141577:ENSG00000167302:ENSG00000185504:ENSG00000182446:ENSG00000017797:ENSG00000154845:ENSG00000134265:ENSG00000154889:ENSG00000141401:ENSG00000168234:ENSG00000141384:ENSG00000152223:ENSG00000081913:ENSG00000130856:ENSG00000197971:ENSG00000071564:ENSG00000141985:ENSG00000167670:ENSG00000130254:ENSG00000205744:ENSG00000104833:ENSG00000104880:ENSG00000104883:ENSG00000065989:ENSG00000079999:ENSG00000104957:ENSG00000132024:ENSG00000132000:ENSG00000132017:ENSG00000132005:ENSG00000123136:ENSG00000099308:ENSG00000237440:ENSG00000104825:ENSG00000104835:ENSG00000269190:ENSG00000160570:ENSG00000167625:ENSG00000105723:ENSG00000079432:ENSG00000079462:ENSG00000079435:ENSG00000079385:ENSG00000185453:ENSG00000105483:ENSG00000105447:ENSG00000063127:ENSG00000142544:ENSG00000105383:ENSG00000196214:ENSG00000167766:ENSG00000213020:ENSG00000176293:ENSG00000115756:ENSG00000115761:ENSG00000196208:ENSG00000197872:ENSG00000143867:ENSG00000055917:ENSG00000084676:ENSG00000162928:ENSG00000162929:ENSG00000082898:ENSG00000116039:

						ENSG00000075292:ENSG00000144048:ENSG00000114978:ENSG00000065911:ENSG00000005448:ENSG00000114993:ENSG00000144218:ENSG00000115604:ENSG00000115607:ENSG00000153107:ENSG00000153208:ENSG00000153214:ENSG00000115762:ENSG00000163040:ENSG00000144283:ENSG00000118260:ENSG00000197713:ENSG00000135912:ENSG00000153832:ENSG00000144485:ENSG00000132326:ENSG00000162804:ENSG00000196209:ENSG00000088876:ENSG00000125779:ENSG00000132646:ENSG00000089048:ENSG00000188559:ENSG00000124102:ENSG00000124107:ENSG00000124228:ENSG00000124201:ENSG00000203896:ENSG00000101152:ENSG00000155313:ENSG00000154642:ENSG00000157617:ENSG00000160190:ENSG00000160284:ENSG00000160285:ENSG00000184792:ENSG00000128335:ENSG00000100324:ENSG00000198951:ENSG00000177096:ENSG00000242247:ENSG00000138944:ENSG00000205643:ENSG00000205593:ENSG00000025770:ENSG00000130489:ENSG00000070950:ENSG00000134077:ENSG00000144560:ENSG00000170011:ENSG00000182983:ENSG00000186792:ENSG00000243477:ENSG00000114388:ENSG00000114395:ENSG00000180376:ENSG00000163946:ENSG00000114861:ENSG00000163320:ENSG00000175105:ENSG00000179021:ENSG00000169379:ENSG00000178700:ENSG00000114446:ENSG00000163608:ENSG00000144843:ENSG00000082701:ENSG00000172780:ENSG00000240682:ENSG00000017260:ENSG00000034533:ENSG00000114670:ENSG00000113971:ENSG00000158163:ENSG00000138231:ENSG00000114098:ENSG00000155903:ENSG00000175066:ENSG00000114127:ENSG00000163762:ENSG00000169282:ENSG00000172578:ENSG00000073803:ENSG00000163904:ENSG00000145014:ENSG00000041802:ENSG00000163975:ENSG00000109689:ENSG00000145246:ENSG00000151247:ENSG00000145331:ENSG00000164128:ENSG00000109466:ENSG00000129187:ENSG00000113360:ENSG00000082213:ENSG00000164190:ENSG00000171522:ENSG00000132357:ENSG00000067248:ENSG00000039123:ENSG00000068796:ENSG00000086189:ENSG00000164331:ENSG00000164338:ENSG00000164180:ENSG00000153140:ENSG00000181751:ENSG00000064651:ENSG00000240184:ENSG00000131504:ENSG00000171720:ENSG00000164620:ENSG00000197948:ENSG00000113272:ENSG00000155858:ENSG00000074317:ENSG00000048140:ENSG00000131446:ENSG00000196670:ENSG00000124535:ENSG00000137266:ENSG00000124787:ENSG00000078401:ENSG00000079691:ENSG00000146112:ENSG00000137404:ENSG00000137337:ENSG00000204344:ENSG00000223501:ENSG00000235863:ENSG00000227057:ENSG00000204220:ENSG00000112245:ENSG00000198945:ENSG00000112357:ENSG00000120278:ENSG00000131018:ENSG00000026297:ENSG00000213066:ENSG00000184786:ENSG00000130023:ENSG00000122644:ENSG00000105855:ENSG00000153814:ENSG00000168303:ENSG00000152926:ENSG00000213462:ENSG00000146757:ENSG00000126522:ENSG00000169902:ENSG00000086730:ENSG00000049541:ENSG00000105829:ENSG00000085514:ENSG00000078487:ENSG00000146834:ENSG00000160813:ENSG00000091704:ENSG00000106484:ENSG00000158623:ENSG00000131558:ENSG00000181444:ENSG00000214106:ENSG00000146918:ENSG00000168546:ENSG00000129691:ENSG00000023287:ENSG00000254087:ENSG00000035681:ENSG00000147601:ENSG00000121039:ENSG00000120526:ENSG00000120533:ENSG00000129295:ENSG00000131773:ENSG00000180900:ENSG00000196378:ENSG00000080608:ENSG00000044459:ENSG00000127080:ENSG00000130958:ENSG00000136936:ENSG00000070159:ENSG00000196152:ENSG00000148356:ENSG00000136908:ENSG00000167106:ENSG00000171169:ENSG00000148339:ENSG00000148308:ENSG00000148411
DEG. down	Heart_Left_Ventricle	9531	614	4.89657719644904e-28	2.64415168608248e-26	ENSG00000158292:ENSG00000097021:ENSG00000171603:ENSG00000120948:ENSG00000171824:ENSG00000198793:ENSG00000171819:ENSG00000120942:ENSG00000065526:ENSG00000116809:ENSG00000187942:ENSG00000117616:ENSG00000183726:ENSG00000204178:ENSG00000157978:ENSG00000084652:ENSG00000160050:ENSG00000160051:ENSG00000116525:ENSG00000160094:ENSG00000127603:ENSG00000117395:ENSG00000066322:ENSG00000159479:ENSG00000198198:ENSG00000162367:ENSG00000117862:ENSG00000198841:ENSG00000134717:ENSG00000162604:ENSG00000117114:ENSG00000117133:ENSG00000117155:ENSG00000162642:ENSG00000142867:ENSG00000153904:ENSG00000197147:ENSG00000171488:ENSG00000117525:ENSG00000117569:ENSG00000162694:ENSG00000116396:ENSG00000162775:ENSG00000168679:ENSG00000085465:ENSG00000163349:ENSG00000197323:ENSG00000116752:ENSG00000213281:ENSG00000052723:ENSG00000092621:ENSG00000143457:ENSG00000163131:ENSG00000143387:ENSG00000143437:ENSG00000143379:ENSG00000143418:ENSG00000143412:ENSG00000163154:ENSG00000163156:ENSG00000163155:ENSG00000160767:ENSG00000176444:ENSG00000160752:ENSG00000160753:ENSG00000116539:ENSG00000125459:ENSG00000163374:ENSG00000116580:ENSG00000132718:ENSG00000143622:ENSG00000132680:ENSG00000116584:ENSG00000160803:ENSG00000160781:ENSG00000163472:ENSG00000198715:ENSG00000187800:ENSG00000132694:ENSG00000143226:ENSG00000173110:ENSG00000203747:ENSG00000152332:ENSG00000174175:ENSG00000120341:ENSG00000186283:ENSG00000143322:ENSG00000057252:ENSG00000177888:ENSG00000174307:ENSG00000159176:ENSG00000122176:ENSG00000182004:ENSG00000143801:ENSG00000162885:ENSG00000162714:ENSG00000015171:ENSG00000107929:ENSG00000198879:ENSG00000148468:ENSG00000241058:ENSG00000165997:ENSG00000099246:ENSG00000108094:ENSG00000095794:ENSG00000188234:ENSG00000165476:ENSG00000107736:ENSG00000107738:ENSG00000026103:ENSG00000059573:ENSG00000155256:ENSG00000120057:ENSG00000119927:ENSG00000165832:ENSG00000184545:ENSG00000268403:ENSG00000166478:ENSG00000198730:ENSG00000006071:ENSG00000110442:ENSG00000121680:ENSG00000149150:ENSG00000156587:ENSG00000162174:ENSG00000110025:ENSG00000168061:ENSG00000168060:ENSG00000162300:ENSG00000149823:ENSG00000174276:ENSG00000162298:ENSG00000149792:ENSG00000173653:ENSG00000173599:ENSG00000173621:ENSG00000175567:ENSG00000062282:ENSG00000137492:ENSG00000137693:ENSG00000149313:ENSG00000149292:ENSG00000180425:ENSG00000076053:ENSG00000166250:ENSG00000064309:ENSG00000082805:ENSG00000010219:ENSG00000111667:ENSG00000111671:ENSG00000010626:ENSG00000111674:ENSG00000111679:ENSG00000126749:ENSG00000111684:ENSG00000013588:ENSG00000134531:ENSG00000123094:ENSG00000185432:ENSG00000110911:ENSG00000050426:ENSG00000110925:ENSG00000135457:ENSG00000123360:ENSG00000127314:ENSG00000166130:ENSG00000120868:ENSG00000111647:ENSG00000139410:ENSG00000174989:ENSG00000111725:ENSG00000188735:ENSG000001509

	90:ENSG00000184992:ENSG00000177169:ENSG00000177084:ENSG00000176915:ENSG00000139505:ENSG00000133110:ENSG00000102743:ENSG00000120688:ENSG00000136167:ENSG00000102531:ENSG00000123179:ENSG00000136104:ENSG00000188243:ENSG00000118939:ENSG00000178695:ENSG00000088451:ENSG00000152749:ENSG00000065150:ENSG00000134901:ENSG00000139826:ENSG00000100889:ENSG00000100897:ENSG00000092140:ENSG00000092108:ENSG00000129493:ENSG00000203546:ENSG00000129480:ENSG00000150527:ENSG00000165355:ENSG00000179476:ENSG00000179454:ENSG00000198718:ENSG00000185246:ENSG00000139926:ENSG00000087301:ENSG00000197930:ENSG00000119599:ENSG00000165861:ENSG00000119707:ENSG00000080815:ENSG00000100767:ENSG00000133961:ENSG00000165417:ENSG00000165934:ENSG00000153485:ENSG00000012963:ENSG00000140157:ENSG00000170113:ENSG00000128731:ENSG00000021776:ENSG00000198146:ENSG00000174197:ENSG00000137802:ENSG00000243708:ENSG00000243789:ENSG00000197535:ENSG00000128989:ENSG00000181827:ENSG00000140262:ENSG00000103710:ENSG00000140350:ENSG00000117899:ENSG00000140406:ENSG00000140511:ENSG00000140471:ENSG00000008710:ENSG00000103512:ENSG00000183426:ENSG00000179889:ENSG00000157045:ENSG00000085721:ENSG00000157106:ENSG00000102879:ENSG00000169217:ENSG00000169221:ENSG00000180035:ENSG00000179965:ENSG00000179958:ENSG00000140691:ENSG00000140682:ENSG00000197302:ENSG00000102910:ENSG00000196470:ENSG00000102921:ENSG00000102978:ENSG00000072736:ENSG00000103061:ENSG00000132600:ENSG00000103018:ENSG00000185669:ENSG00000158717:ENSG00000167515:ENSG00000167522:ENSG00000197912:ENSG00000127804:ENSG00000196689:ENSG00000197417:ENSG00000029725:ENSG00000005100:ENSG00000072849:ENSG00000132514:ENSG00000141127:ENSG00000188522:ENSG00000072134:ENSG00000108641:ENSG00000166484:ENSG00000128482:ENSG00000142494:ENSG00000067191:ENSG00000068079:ENSG00000188554:ENSG00000184988:ENSG00000225190:ENSG00000141279:ENSG00000108424:ENSG00000198933:ENSG00000008294:ENSG00000011258:ENSG00000011260:ENSG00000153933:ENSG00000121060:ENSG00000108423:ENSG00000108443:ENSG00000189050:ENSG00000198265:ENSG00000197170:ENSG00000154217:ENSG00000141576:ENSG00000185262:ENSG00000078687:ENSG00000141577:ENSG00000167302:ENSG00000185504:ENSG00000182446:ENSG00000017797:ENSG00000154845:ENSG00000134265:ENSG00000154889:ENSG00000141401:ENSG00000152223:ENSG00000101638:ENSG00000081913:ENSG00000130856:ENSG00000197971:ENSG00000071564:ENSG00000141985:ENSG00000167670:ENSG00000130254:ENSG00000160633:ENSG00000104880:ENSG00000104883:ENSG00000167775:ENSG00000065989:ENSG00000079999:ENSG00000104957:ENSG00000132024:ENSG00000132017:ENSG00000132005:ENSG00000123146:ENSG00000123136:ENSG00000123159:ENSG00000132002:ENSG00000099308:ENSG00000104825:ENSG00000104835:ENSG00000269190:ENSG00000160570:ENSG00000167625:ENSG00000105723:ENSG00000105722:ENSG00000079432:ENSG00000079462:ENSG00000079385:ENSG00000185453:ENSG00000105483:ENSG00000105447:ENSG00000142544:ENSG00000105383:ENSG00000196214:ENSG00000167766:ENSG00000213020:ENSG00000176293:ENSG00000115756:ENSG00000115761:ENSG00000196208:ENSG00000197872:ENSG00000143867:ENSG00000055917:ENSG00000084676:ENSG00000119866:ENSG00000162928:ENSG00000162929:ENSG00000082898:ENSG00000075292:ENSG00000144034:ENSG00000144048:ENSG00000114978:ENSG00000065911:ENSG00000005448:ENSG00000114993:ENSG00000115604:ENSG00000153107:ENSG00000153208:ENSG00000153214:ENSG00000152102:ENSG00000115762:ENSG00000163040:ENSG00000115963:ENSG00000144283:ENSG00000153250:ENSG00000118260:ENSG00000144401:ENSG00000197713:ENSG00000018280:ENSG00000135912:ENSG00000168958:ENSG00000144485:ENSG00000132326:ENSG00000162804:ENSG00000088876:ENSG00000125779:ENSG00000089063:ENSG00000132646:ENSG00000101290:ENSG00000089048:ENSG00000188559:ENSG00000242372:ENSG00000124107:ENSG00000124228:ENSG00000124201:ENSG00000203896:ENSG00000101152:ENSG00000155313:ENSG00000154642:ENSG00000157617:ENSG00000160190:ENSG00000160284:ENSG00000160285:ENSG00000184792:ENSG00000100220:ENSG00000128335:ENSG00000100342:ENSG00000100321:ENSG00000100324:ENSG00000198951:ENSG00000177096:ENSG00000242247:ENSG00000205643:ENSG00000075234:ENSG00000075275:ENSG00000196576:ENSG00000100258:ENSG00000025770:ENSG00000130489:ENSG00000025708:ENSG00000177989:ENSG00000100299:ENSG00000134077:ENSG00000144560:ENSG00000163517:ENSG00000170293:ENSG00000177873:ENSG00000182983:ENSG00000243477:ENSG00000114383:ENSG00000068028:ENSG00000114388:ENSG00000114395:ENSG00000126062:ENSG00000180376:ENSG00000163946:ENSG00000144741:ENSG00000114861:ENSG00000144736:ENSG00000163320:ENSG00000175105:ENSG00000179021:ENSG00000169379:ENSG00000178700:ENSG00000114446:ENSG00000163608:ENSG00000082701:ENSG00000240682:ENSG00000017260:ENSG00000034533:ENSG00000240303:ENSG00000113971:ENSG00000129048:ENSG00000081307:ENSG00000144867:ENSG00000158163:ENSG00000138231:ENSG00000114098:ENSG00000184432:ENSG00000114115:ENSG00000155903:ENSG00000175066:ENSG00000114127:ENSG00000114698:ENSG00000169282:ENSG00000075420:ENSG00000161202:ENSG00000073803:ENSG00000163904:ENSG00000145014:ENSG00000041802:ENSG00000163975:ENSG00000013288:ENSG00000109689:ENSG00000169019:ENSG00000145246:ENSG00000109171:ENSG00000080493:ENSG00000169429:ENSG00000163739:ENSG00000109321:ENSG00000151247:ENSG00000145331:ENSG00000205403:ENSG00000109466:ENSG00000129187:ENSG00000113360:ENSG00000082213:ENSG00000164190:ENSG00000171522:ENSG00000132357:ENSG00000067248:ENSG00000039123:ENSG00000068796:ENSG00000086189:ENSG00000086200:ENSG00000164331:ENSG00000164338:ENSG00000164180:ENSG00000153140:ENSG00000181751:ENSG00000168916:ENSG00000064651:ENSG00000253159:ENSG00000240184:ENSG00000131504:ENSG00000171720:ENSG00000197948:ENSG00000131507:ENSG00000113272:ENSG00000048140:ENSG00000131446:ENSG00000196670:ENSG00000124535:ENSG00000137266:ENSG00000124787:ENSG00000078401:ENSG00000079691:ENSG00000146112:ENSG00000137404:ENSG00000137337:ENSG00000204344:ENSG00000244731:ENSG00000223501:ENSG00000235863:ENSG00000227057:ENSG00000204220:ENSG00000237441:ENSG00000198663:ENSG00000156639:ENSG00000112245:ENSG00000118503:ENSG00000112406:ENSG00000120278:ENSG00000131018:ENS

						G00000272047:ENSG00000026297:ENSG00000213066:ENSG00000186340:ENSG00000184786:ENSG00000130023:ENSG00000122644:ENSG00000153814:ENSG00000122545:ENSG00000168303:ENSG00000152926:ENSG00000213462:ENSG00000146757:ENSG00000126522:ENSG00000169902:ENSG00000086730:ENSG00000049541:ENSG00000177679:ENSG00000085563:ENSG00000105829:ENSG00000121716:ENSG00000085514:ENSG00000078487:ENSG00000146834:ENSG00000160813:ENSG00000166925:ENSG00000005249:ENSG00000105865:ENSG00000196937:ENSG00000091704:ENSG00000106484:ENSG00000158623:ENSG00000131558:ENSG00000182158:ENSG00000146966:ENSG00000181444:ENSG00000117868:ENSG00000129691:ENSG00000023287:ENSG00000254087:ENSG00000035681:ENSG00000147601:ENSG00000121039:ENSG00000104267:ENSG00000164949:ENSG00000120526:ENSG00000120533:ENSG00000131773:ENSG00000160932:ENSG00000180900:ENSG00000179950:ENSG00000185189:ENSG00000196378:ENSG00000080608:ENSG00000086065:ENSG00000135018:ENSG00000213694:ENSG00000127080:ENSG00000130958:ENSG00000136925:ENSG00000136936:ENSG00000196152:ENSG00000160408:ENSG00000136908:ENSG00000167106:ENSG00000171169:ENSG00000148339:ENSG00000148308:ENSG00000170835:ENSG00000148411:ENSG00000188986
DEG. down	Kidney_Cortex	5567	355	1.51986541584094e-13	8.20727324554107e-12	ENSG00000158292:ENSG00000171819:ENSG00000120942:ENSG00000065526:ENSG00000187942:ENSG00000204178:ENSG00000160050:ENSG00000116525:ENSG00000127603:ENSG00000198841:ENSG00000134717:ENSG00000162604:ENSG00000162616:ENSG00000162642:ENSG00000197147:ENSG00000171488:ENSG00000069712:ENSG00000117525:ENSG00000117569:ENSG00000117543:ENSG00000162775:ENSG00000163349:ENSG00000197323:ENSG00000143457:ENSG00000163131:ENSG00000143387:ENSG00000143437:ENSG00000143379:ENSG00000163154:ENSG00000163354:ENSG00000143537:ENSG00000160767:ENSG00000116539:ENSG00000163374:ENSG00000116580:ENSG00000132718:ENSG00000116584:ENSG00000160803:ENSG00000163472:ENSG00000132694:ENSG00000143322:ENSG00000057252:ENSG00000177888:ENSG00000174307:ENSG00000163050:ENSG00000162885:ENSG00000107929:ENSG00000198879:ENSG00000165997:ENSG00000099246:ENSG00000108094:ENSG00000165476:ENSG00000107738:ENSG00000138134:ENSG00000165338:ENSG00000119927:ENSG00000198873:ENSG00000184545:ENSG00000205339:ENSG00000166478:ENSG00000166483:ENSG00000198730:ENSG00000187486:ENSG00000149150:ENSG00000168061:ENSG00000168060:ENSG00000162300:ENSG00000173621:ENSG00000175564:ENSG00000062282:ENSG00000137492:ENSG00000137672:ENSG00000076053:ENSG00000166250:ENSG00000082805:ENSG00000010219:ENSG00000111674:ENSG00000134531:ENSG00000110925:ENSG00000135457:ENSG00000123338:ENSG00000123360:ENSG00000166130:ENSG00000120868:ENSG00000111647:ENSG00000074590:ENSG00000174989:ENSG00000150990:ENSG00000177084:ENSG00000176915:ENSG00000139505:ENSG00000120688:ENSG00000136167:ENSG00000102531:ENSG00000123179:ENSG00000102753:ENSG00000136104:ENSG00000178695:ENSG00000152749:ENSG00000065150:ENSG00000139826:ENSG00000102524:ENSG00000139914:ENSG00000092140:ENSG00000092108:ENSG00000100473:ENSG00000129493:ENSG00000129480:ENSG00000165355:ENSG00000179476:ENSG00000179454:ENSG00000198718:ENSG00000185246:ENSG00000139926:ENSG00000087301:ENSG00000197930:ENSG00000119707:ENSG00000080815:ENSG00000165417:ENSG00000165934:ENSG00000012963:ENSG00000170113:ENSG00000128731:ENSG00000021776:ENSG00000198146:ENSG00000174197:ENSG00000137802:ENSG00000243789:ENSG00000197535:ENSG00000181827:ENSG00000140262:ENSG00000103710:ENSG00000136378:ENSG00000140600:ENSG00000140511:ENSG00000185418:ENSG00000008710:ENSG00000103512:ENSG00000183426:ENSG00000085721:ENSG00000157106:ENSG00000102879:ENSG00000169221:ENSG00000102910:ENSG00000196470:ENSG00000102921:ENSG00000072736:ENSG00000103064:ENSG00000132600:ENSG00000103056:ENSG00000185669:ENSG00000167522:ENSG00000127804:ENSG00000029725:ENSG00000005100:ENSG00000132514:ENSG00000072134:ENSG00000166484:ENSG00000128482:ENSG00000067191:ENSG00000182963:ENSG00000225190:ENSG00000141279:ENSG00000108424:ENSG00000008294:ENSG00000011258:ENSG00000011260:ENSG00000214226:ENSG00000153933:ENSG00000121060:ENSG00000108423:ENSG00000108443:ENSG00000189050:ENSG00000198265:ENSG00000197170:ENSG00000154217:ENSG00000141540:ENSG00000141576:ENSG00000078687:ENSG00000154845:ENSG00000134265:ENSG00000168234:ENSG00000141384:ENSG00000152223:ENSG00000081913:ENSG00000130856:ENSG00000197971:ENSG00000167670:ENSG00000104833:ENSG00000065989:ENSG00000079999:ENSG00000127527:ENSG00000099308:ENSG00000167625:ENSG00000079432:ENSG00000105429:ENSG00000105427:ENSG00000079435:ENSG00000185453:ENSG00000105483:ENSG00000105447:ENSG00000105383:ENSG00000196214:ENSG00000197872:ENSG00000143867:ENSG00000055917:ENSG00000084676:ENSG00000162929:ENSG00000082898:ENSG00000075292:ENSG00000144048:ENSG00000065911:ENSG00000144218:ENSG00000153107:ENSG00000153214:ENSG00000115963:ENSG00000118260:ENSG00000135912:ENSG00000198612:ENSG00000144485:ENSG00000132326:ENSG00000162804:ENSG00000196209:ENSG00000088876:ENSG00000125779:ENSG00000101290:ENSG00000089048:ENSG00000188559:ENSG00000124102:ENSG00000124228:ENSG00000124201:ENSG00000155313:ENSG00000154642:ENSG00000157617:ENSG00000160284:ENSG00000160285:ENSG00000183628:ENSG00000184792:ENSG00000100321:ENSG00000100324:ENSG00000177096:ENSG00000025770:ENSG00000008735:ENSG00000070950:ENSG00000134077:ENSG00000144560:ENSG00000144712:ENSG00000170011:ENSG00000182606:ENSG00000182983:ENSG00000186792:ENSG00000243477:ENSG00000068028:ENSG00000180376:ENSG00000163946:ENSG00000144749:ENSG00000114861:ENSG00000144736:ENSG00000175105:ENSG00000179021:ENSG00000169379:ENSG00000178700:ENSG00000144843:ENSG00000082701:ENSG00000240682:ENSG00000017260:ENSG00000034533:ENSG00000113971:ENSG00000154917:ENSG00000138231:ENSG00000114098:ENSG00000158186:ENSG00000155903:ENSG00000175066:ENSG00000114127:ENSG00000114698:ENSG00000163904:ENSG00000041802:ENSG00000013288:ENSG00000109171:ENSG00000109321:ENSG00000151247:ENSG00000109466:ENSG00000113360:ENSG00000082213:ENSG00000164190:ENSG00000132357:ENSG00000067248:ENSG00000039123:ENSG00000068796:ENSG00000086189:ENSG00000086200:ENSG00000164331:ENSG00000164338:ENSG00000181751:ENSG00000173926:ENSG

						00000064651:ENSG00000240184:ENSG00000197948:ENSG00000135074:ENSG00000113272:ENSG00000155858:ENSG00000131446:ENSG00000124535:ENSG00000137266:ENSG00000124787:ENSG00000146112:ENSG00000137404:ENSG00000137337:ENSG00000235863:ENSG00000227057:ENSG00000124772:ENSG00000198663:ENSG00000164530:ENSG00000118432:ENSG00000112406:ENSG00000026297:ENSG00000186340:ENSG00000122644:ENSG00000153814:ENSG00000122545:ENSG00000152926:ENSG00000213462:ENSG00000146757:ENSG00000086730:ENSG00000049541:ENSG00000135218:ENSG00000078487:ENSG00000166924:ENSG00000005249:ENSG00000075790:ENSG00000106477:ENSG00000158623:ENSG00000131558:ENSG00000146966:ENSG00000146918:ENSG00000117868:ENSG00000120910:ENSG00000129691:ENSG00000023287:ENSG00000254087:ENSG00000035681:ENSG00000120526:ENSG00000165071:ENSG00000196378:ENSG00000044459:ENSG00000127080:ENSG00000136925:ENSG00000136936:ENSG00000136826:ENSG00000167081:ENSG00000196152:ENSG00000148308:ENSG00000130560:ENSG00000148411
DEG. down	Kidney_Medulla	0	0	1	1	
DEG. down	Liver	8059	514	4.963075038315e-21	2.6800605206901e-19	ENSG00000116237:ENSG00000158292:ENSG00000097021:ENSG00000171603:ENSG00000171824:ENSG00000198793:ENSG00000162458:ENSG00000065526:ENSG00000116809:ENSG00000158747:ENSG00000183726:ENSG00000184007:ENSG00000160050:ENSG00000160051:ENSG00000116525:ENSG00000160094:ENSG00000127603:ENSG00000066056:ENSG00000117399:ENSG00000066322:ENSG00000198198:ENSG00000162366:ENSG00000162367:ENSG00000117862:ENSG00000198841:ENSG00000134717:ENSG00000162384:ENSG00000162604:ENSG00000162616:ENSG00000117133:ENSG00000117155:ENSG00000162642:ENSG00000142867:ENSG00000197147:ENSG00000117525:ENSG00000117569:ENSG00000162694:ENSG00000117543:ENSG00000168679:ENSG00000116455:ENSG00000163349:ENSG00000197323:ENSG00000116752:ENSG00000213281:ENSG00000143457:ENSG00000143387:ENSG00000143437:ENSG00000143379:ENSG00000143409:ENSG00000143363:ENSG00000143434:ENSG00000163154:ENSG00000163156:ENSG00000163155:ENSG00000163354:ENSG00000143537:ENSG00000160767:ENSG00000116521:ENSG00000160753:ENSG00000116539:ENSG00000163374:ENSG00000116580:ENSG00000143622:ENSG00000132680:ENSG00000116584:ENSG00000160803:ENSG00000196189:ENSG00000242252:ENSG00000160781:ENSG00000163472:ENSG00000187800:ENSG00000132694:ENSG00000143226:ENSG00000174175:ENSG00000186283:ENSG00000143322:ENSG00000057252:ENSG00000177888:ENSG00000174307:ENSG00000122176:ENSG00000182004:ENSG00000143801:ENSG00000162885:ENSG00000162714:ENSG00000015171:ENSG00000107929:ENSG00000148468:ENSG00000099246:ENSG00000108094:ENSG00000095794:ENSG00000188234:ENSG00000165476:ENSG00000107738:ENSG00000026103:ENSG00000165832:ENSG00000198873:ENSG00000184545:ENSG00000166313:ENSG00000205339:ENSG00000268403:ENSG00000166478:ENSG00000166483:ENSG00000110442:ENSG00000162174:ENSG00000213465:ENSG00000110025:ENSG00000162300:ENSG00000149823:ENSG00000173653:ENSG00000173621:ENSG00000175567:ENSG00000137492:ENSG00000137693:ENSG00000149313:ENSG00000149292:ENSG00000076043:ENSG00000082805:ENSG00000010219:ENSG00000111667:ENSG00000111671:ENSG00000010626:ENSG00000111674:ENSG00000126749:ENSG00000134531:ENSG00000123094:ENSG00000110911:ENSG00000050426:ENSG00000110925:ENSG00000135457:ENSG00000123338:ENSG00000127314:ENSG00000166130:ENSG00000120868:ENSG00000111647:ENSG00000235162:ENSG00000074590:ENSG00000174989:ENSG00000111725:ENSG00000188735:ENSG00000150990:ENSG00000176915:ENSG00000139505:ENSG00000122033:ENSG00000133110:ENSG00000183722:ENSG00000120688:ENSG00000102753:ENSG00000136104:ENSG00000188243:ENSG00000136153:ENSG00000178695:ENSG00000065150:ENSG00000102524:ENSG00000092140:ENSG00000092108:ENSG00000129493:ENSG00000203546:ENSG00000129480:ENSG00000165355:ENSG00000179476:ENSG00000179454:ENSG00000198718:ENSG00000185246:ENSG00000139926:ENSG00000087301:ENSG00000006432:ENSG00000119599:ENSG00000165861:ENSG00000119707:ENSG00000080815:ENSG00000100767:ENSG00000133961:ENSG00000165417:ENSG00000165934:ENSG00000165943:ENSG00000153485:ENSG00000012963:ENSG00000140157:ENSG00000170113:ENSG00000128731:ENSG00000021776:ENSG00000198146:ENSG00000174197:ENSG00000137802:ENSG00000243708:ENSG00000243789:ENSG00000197535:ENSG00000128989:ENSG00000181827:ENSG00000140262:ENSG00000103707:ENSG00000186198:ENSG00000140350:ENSG00000140406:ENSG00000140471:ENSG00000185418:ENSG00000008710:ENSG00000103512:ENSG00000183426:ENSG00000157045:ENSG00000157106:ENSG00000102879:ENSG00000169217:ENSG00000169221:ENSG00000180035:ENSG00000180096:ENSG00000179958:ENSG00000140682:ENSG00000197302:ENSG00000196470:ENSG00000102921:ENSG00000005194:ENSG00000102978:ENSG00000072736:ENSG00000103066:ENSG00000103064:ENSG00000103061:ENSG00000132600:ENSG00000103056:ENSG00000103018:ENSG00000158717:ENSG00000167515:ENSG00000167522:ENSG00000127804:ENSG00000029725:ENSG00000005100:ENSG00000141127:ENSG00000072134:ENSG00000108641:ENSG00000166484:ENSG00000166482:ENSG00000108828:ENSG00000108830:ENSG00000184988:ENSG00000225190:ENSG00000141279:ENSG00000108424:ENSG00000198933:ENSG00000008294:ENSG00000011258:ENSG00000011260:ENSG00000153933:ENSG00000108423:ENSG00000108443:ENSG00000198265:ENSG00000154217:ENSG00000141576:ENSG00000185262:ENSG00000078687:ENSG00000141577:ENSG00000167302:ENSG00000185504:ENSG00000182446:ENSG00000017797:ENSG00000154845:ENSG00000134265:ENSG00000154889:ENSG00000152223:ENSG00000130856:ENSG00000197971:ENSG00000071564:ENSG00000141985:ENSG00000167670:ENSG00000130254:ENSG00000160633:ENSG00000104880:ENSG00000167775:ENSG00000065989:ENSG00000079999:ENSG00000104957:ENSG00000132024:ENSG00000132017:ENSG00000132005:ENSG00000123146:ENSG00000123136:ENSG00000123143:ENSG00000123159:ENSG00000127527:ENSG00000099308:ENSG00000160570:ENSG00000167625:ENSG00000105723:ENSG00000079432:ENSG00000079462:ENSG00000105429:ENSG00000185453:ENSG00000105483:ENSG00000142544:E

						NSG00000105383:ENSG00000196214:ENSG00000198464:ENSG00000167766:ENSG00000115761:ENSG00000196208:ENSG00000197872:ENSG00000055917:ENSG00000084676:ENSG00000213639:ENSG00000171103:ENSG00000082898:ENSG00000075292:ENSG00000144034:ENSG00000144048:ENSG00000163170:ENSG00000114978:ENSG00000065911:ENSG00000204843:ENSG00000005448:ENSG00000153107:ENSG00000153208:ENSG00000153214:ENSG00000152102:ENSG00000115762:ENSG00000173272:ENSG00000144283:ENSG00000153250:ENSG00000118260:ENSG00000197713:ENSG00000115461:ENSG00000018280:ENSG00000135912:ENSG00000168958:ENSG00000198612:ENSG00000144485:ENSG00000132326:ENSG00000162804:ENSG00000088876:ENSG00000125779:ENSG00000132646:ENSG00000101290:ENSG00000089048:ENSG00000101247:ENSG00000124102:ENSG00000124228:ENSG00000124201:ENSG00000101152:ENSG00000198276:ENSG00000155313:ENSG00000154642:ENSG00000157617:ENSG00000160190:ENSG00000183628:ENSG00000100321:ENSG00000100324:ENSG00000177096:ENSG00000077942:ENSG00000205643:ENSG00000100416:ENSG00000075275:ENSG00000205593:ENSG00000025770:ENSG00000251322:ENSG00000070950:ENSG00000134077:ENSG00000144560:ENSG00000144712:ENSG00000163517:ENSG00000163513:ENSG00000177873:ENSG00000182606:ENSG00000182983:ENSG00000243477:ENSG00000068001:ENSG00000114383:ENSG00000068028:ENSG00000114388:ENSG00000126062:ENSG00000180376:ENSG00000163946:ENSG00000144741:ENSG00000144749:ENSG00000114861:ENSG00000144736:ENSG00000163320:ENSG00000175105:ENSG00000179021:ENSG00000169379:ENSG00000178700:ENSG00000114446:ENSG00000163607:ENSG00000163608:ENSG00000144843:ENSG00000082701:ENSG00000240682:ENSG00000017260:ENSG00000034533:ENSG00000113971:ENSG00000138231:ENSG00000114098:ENSG00000158186:ENSG00000155903:ENSG00000175066:ENSG00000114127:ENSG00000163762:ENSG00000161202:ENSG00000163904:ENSG00000145014:ENSG00000163975:ENSG00000013288:ENSG00000109689:ENSG00000169019:ENSG00000145246:ENSG00000109171:ENSG00000109321:ENSG00000151247:ENSG00000164128:ENSG00000113360:ENSG00000082213:ENSG00000164190:ENSG00000132357:ENSG00000067248:ENSG00000039123:ENSG00000068796:ENSG00000086189:ENSG00000086200:ENSG00000164331:ENSG00000164338:ENSG00000164180:ENSG00000153140:ENSG00000168916:ENSG00000064651:ENSG00000240184:ENSG00000164620:ENSG00000197948:ENSG00000120318:ENSG00000113721:ENSG00000135074:ENSG00000113272:ENSG00000155858:ENSG00000113312:ENSG00000113742:ENSG00000048140:ENSG00000196670:ENSG00000124535:ENSG00000078401:ENSG00000079691:ENSG00000146112:ENSG00000137404:ENSG00000137337:ENSG00000223501:ENSG00000227057:ENSG00000237441:ENSG00000198663:ENSG00000156639:ENSG00000198945:ENSG00000135525:ENSG00000112357:ENSG00000118503:ENSG00000112406:ENSG00000120278:ENSG00000131018:ENSG00000272047:ENSG00000026297:ENSG00000186340:ENSG00000184786:ENSG00000122644:ENSG00000153814:ENSG00000122545:ENSG00000152926:ENSG00000213462:ENSG00000146757:ENSG00000189143:ENSG00000086730:ENSG00000135218:ENSG00000127920:ENSG00000085514:ENSG00000078487:ENSG00000146834:ENSG00000160813:ENSG00000166925:ENSG00000005249:ENSG00000105865:ENSG00000075790:ENSG00000196937:ENSG00000158623:ENSG00000131558:ENSG00000182158:ENSG00000146966:ENSG00000181444:ENSG00000196329:ENSG00000214106:ENSG00000146918:ENSG00000117868:ENSG00000120910:ENSG00000129691:ENSG00000023287:ENSG00000254087:ENSG00000035681:ENSG00000147601:ENSG00000164949:ENSG00000120526:ENSG00000120533:ENSG00000131773:ENSG00000180900:ENSG00000196378:ENSG00000080608:ENSG00000086065:ENSG00000213694:ENSG00000127080:ENSG00000136925:ENSG00000136936:ENSG00000136826:ENSG00000119446:ENSG00000167081:ENSG00000196152:ENSG00000148356:ENSG00000136840:ENSG00000167106:ENSG00000148308:ENSG00000170835:ENSG00000130560:ENSG00000148411:ENSG00000188986:ENSG00000198113
DEG. down	Lung	911	58	0.00434668919457887	0.234721216507259	ENSG00000158286:ENSG00000069812:ENSG00000171819:ENSG00000162390:ENSG00000117155:ENSG00000069712:ENSG00000175984:ENSG00000092621:ENSG00000134240:ENSG00000143412:ENSG00000143434:ENSG00000143630:ENSG00000160781:ENSG00000163472:ENSG00000163050:ENSG00000075073:ENSG00000120057:ENSG00000203867:ENSG00000119927:ENSG00000165905:ENSG00000173599:ENSG00000023171:ENSG00000064309:ENSG00000139914:ENSG00000140600:ENSG00000180209:ENSG00000179965:ENSG00000172137:ENSG00000188522:ENSG00000141750:ENSG00000108830:ENSG00000141576:ENSG00000081913:ENSG00000262484:ENSG00000105427:ENSG00000079435:ENSG00000142273:ENSG00000196208:ENSG00000116039:ENSG00000184792:ENSG00000100321:ENSG00000008735:ENSG00000144712:ENSG00000163517:ENSG00000170011:ENSG00000168032:ENSG00000154917:ENSG00000169282:ENSG00000163975:ENSG00000164128:ENSG00000181751:ENSG00000124772:ENSG00000118432:ENSG00000135525:ENSG00000177679:ENSG00000168546:ENSG00000178031:ENSG00000170835
DEG. down	Minor_Salivary_Gland	1870	110	0.0017920113328029	0.0967686119713564	ENSG00000158292:ENSG00000097021:ENSG00000160050:ENSG00000168389:ENSG00000066056:ENSG00000162367:ENSG00000162616:ENSG00000171488:ENSG00000069712:ENSG00000162694:ENSG00000168679:ENSG00000132718:ENSG00000160781:ENSG00000187800:ENSG00000173110:ENSG00000095794:ENSG00000107736:ENSG00000138134:ENSG00000198873:ENSG00000166313:ENSG00000187486:ENSG00000019505:ENSG00000168060:ENSG00000154127:ENSG00000023171:ENSG00000064309:ENSG00000010626:ENSG00000111674:ENSG00000134532:ENSG00000123094:ENSG00000123360:ENSG00000235162:ENSG00000074590:ENSG00000122966:ENSG00000134901:ENSG00000170113:ENSG00000197535:ENSG00000103710:ENSG00000136378:ENSG00000140600:ENSG00000185418:ENSG00000008710:ENSG00000183426:ENSG00000179965:ENSG00000103064:ENSG00000128482:ENSG00000142494:ENSG00000161405:ENSG00000108830:ENSG00000182963:ENSG00000008294:ENSG00000141540:ENSG00000141576:ENSG00000197971:ENSG00000099308:ENSG00000269190:ENSG00000063127:ENSG00000115756:ENSG00000197872:ENSG00000005448:ENSG00000144218:ENSG00000115602:ENSG00000115607:ENSG00000153208:ENSG00000163040:ENSG00000144485:ENSG00000101290:ENSG00000125966:ENSG00000198959:ENSG00000160284:ENSG00000144712:ENSG00000007402:ENSG00000154917:ENSG00000158163:E

						NSG00000158186:ENSG00000169282:ENSG00000114279:ENSG00000080493:ENSG00000215218:ENSG00000175471:ENSG00000181751:ENSG00000253159:ENSG00000120318:ENSG00000135074:ENSG00000155858:ENSG00000137404:ENSG00000124772:ENSG00000118432:ENSG00000132429:ENSG00000131018:ENSG00000272047:ENSG00000153814:ENSG00000241644:ENSG00000169902:ENSG00000085563:ENSG00000127920:ENSG00000085514:ENSG00000078487:ENSG00000166924:ENSG00000075790:ENSG00000146966:ENSG00000154319:ENSG00000147465:ENSG00000129295:ENSG00000196378:ENSG00000178031:ENSG00000213694:ENSG00000167081:ENSG00000148411:ENSG00000198113
DEG. down	Muscle_Skeletal	6908	446	1.31995640360324e-18	7.12776457945749e-17	ENSG00000158286:ENSG00000158292:ENSG00000097021:ENSG00000171603:ENSG00000162458:ENSG00000116809:ENSG00000158747:ENSG00000117616:ENSG00000183726:ENSG00000157978:ENSG00000084652:ENSG00000160051:ENSG00000116525:ENSG00000160094:ENSG00000127603:ENSG00000066056:ENSG00000066322:ENSG00000159479:ENSG00000198198:ENSG00000117472:ENSG00000162367:ENSG00000117862:ENSG00000198841:ENSG00000162390:ENSG00000162604:ENSG00000117114:ENSG00000117155:ENSG00000162642:ENSG00000142867:ENSG00000153904:ENSG00000197147:ENSG00000171488:ENSG00000117528:ENSG00000117525:ENSG00000117569:ENSG00000162694:ENSG00000116396:ENSG00000168679:ENSG00000085465:ENSG00000197323:ENSG00000213281:ENSG00000052723:ENSG00000143457:ENSG00000163131:ENSG00000143387:ENSG00000143379:ENSG00000143418:ENSG00000143412:ENSG00000143409:ENSG00000143537:ENSG00000176444:ENSG00000160752:ENSG00000160753:ENSG00000125459:ENSG00000116580:ENSG00000132718:ENSG00000143622:ENSG00000132680:ENSG00000116584:ENSG00000196189:ENSG00000242252:ENSG00000160781:ENSG00000163472:ENSG00000198715:ENSG00000187800:ENSG00000132694:ENSG00000143226:ENSG00000173110:ENSG00000203747:ENSG00000174175:ENSG00000143322:ENSG00000057252:ENSG00000177888:ENSG00000159176:ENSG00000122176:ENSG00000182004:ENSG00000116962:ENSG00000198879:ENSG00000148468:ENSG00000241058:ENSG00000165997:ENSG00000099246:ENSG00000095794:ENSG00000188234:ENSG00000165476:ENSG00000075073:ENSG00000107738:ENSG00000026103:ENSG00000152779:ENSG00000165338:ENSG00000059573:ENSG00000155256:ENSG00000120057:ENSG00000119927:ENSG00000165832:ENSG00000198873:ENSG00000166483:ENSG00000006071:ENSG00000121680:ENSG00000156587:ENSG00000124935:ENSG00000162174:ENSG00000162300:ENSG00000149809:ENSG00000173653:ENSG00000173599:ENSG00000173621:ENSG00000062282:ENSG00000149292:ENSG00000076053:ENSG00000154127:ENSG00000166250:ENSG00000082805:ENSG00000010219:ENSG00000111671:ENSG00000111674:ENSG00000111679:ENSG00000134531:ENSG00000110911:ENSG00000050426:ENSG00000123360:ENSG00000127314:ENSG00000166130:ENSG00000120868:ENSG00000139410:ENSG00000174989:ENSG00000111725:ENSG00000177084:ENSG00000176915:ENSG00000127863:ENSG00000139505:ENSG00000133110:ENSG00000183722:ENSG00000136167:ENSG00000102531:ENSG00000123179:ENSG00000136104:ENSG00000188243:ENSG00000178695:ENSG00000088451:ENSG00000152749:ENSG00000139826:ENSG00000100889:ENSG00000092140:ENSG00000092108:ENSG00000129493:ENSG00000129480:ENSG00000165355:ENSG00000179476:ENSG00000185246:ENSG00000139926:ENSG00000087301:ENSG00000197930:ENSG00000006432:ENSG00000119707:ENSG00000080815:ENSG00000100767:ENSG00000133961:ENSG00000140157:ENSG00000170113:ENSG00000021776:ENSG00000174197:ENSG00000137802:ENSG00000243708:ENSG00000243789:ENSG00000197535:ENSG00000140262:ENSG00000103710:ENSG00000140350:ENSG00000136378:ENSG00000117899:ENSG00000140406:ENSG00000140511:ENSG00000140471:ENSG00000183426:ENSG00000102879:ENSG00000180035:ENSG00000140682:ENSG00000197302:ENSG00000196470:ENSG00000102921:ENSG00000103056:ENSG00000158717:ENSG00000167515:ENSG00000167522:ENSG00000127804:ENSG00000196689:ENSG00000197417:ENSG00000072849:ENSG00000132514:ENSG00000141127:ENSG00000188522:ENSG00000072134:ENSG00000108641:ENSG00000166484:ENSG00000166482:ENSG00000128482:ENSG00000068079:ENSG00000108828:ENSG00000182963:ENSG00000011258:ENSG00000121060:ENSG00000108423:ENSG00000154217:ENSG00000141540:ENSG00000078687:ENSG00000141577:ENSG00000167302:ENSG00000185504:ENSG00000017797:ENSG00000154845:ENSG00000134265:ENSG00000152223:ENSG00000081913:ENSG00000130856:ENSG00000071564:ENSG00000141985:ENSG00000104833:ENSG00000104880:ENSG00000104883:ENSG00000167775:ENSG00000104957:ENSG00000132017:ENSG00000132005:ENSG00000123146:ENSG00000123136:ENSG00000132002:ENSG00000099308:ENSG00000269190:ENSG00000160570:ENSG00000105722:ENSG00000079432:ENSG00000079462:ENSG00000105429:ENSG00000105427:ENSG00000079435:ENSG00000187244:ENSG00000185453:ENSG00000105483:ENSG00000142544:ENSG00000196214:ENSG00000167766:ENSG00000115756:ENSG00000196208:ENSG00000197872:ENSG00000143867:ENSG00000055917:ENSG00000163803:ENSG00000162929:ENSG00000082898:ENSG00000144048:ENSG00000114978:ENSG00000005448:ENSG00000114993:ENSG00000115602:ENSG00000153208:ENSG00000153214:ENSG00000115762:ENSG00000173272:ENSG00000115963:ENSG00000144283:ENSG00000153250:ENSG00000118260:ENSG00000197713:ENSG00000115457:ENSG00000018280:ENSG00000135912:ENSG00000132326:ENSG00000088876:ENSG00000125779:ENSG00000132646:ENSG00000101290:ENSG00000124228:ENSG00000124201:ENSG00000203896:ENSG00000101152:ENSG00000154642:ENSG00000157617:ENSG00000160190:ENSG00000160284:ENSG00000160285:ENSG00000184792:ENSG00000128335:ENSG00000100342:ENSG00000100321:ENSG00000100324:ENSG00000198951:ENSG00000177096:ENSG00000242247:ENSG00000077942:ENSG00000205643:ENSG00000075234:ENSG00000100416:ENSG00000196576:ENSG00000100258:ENSG00000025770:ENSG00000130489:ENSG00000025708:ENSG00000177989:ENSG00000100288:ENSG00000100299:ENSG00000251322:ENSG00000144560:ENSG00000163513:ENSG00000170293:ENSG00000170011:ENSG00000182983:ENSG00000114353:ENSG00000243477:ENSG00000114378:ENSG00000068001:ENSG00000068028:ENSG00000114395:ENSG00000126062:ENSG00000007402:ENSG00000180376:ENSG00000163946:ENSG00000114861:ENSG00000163320:ENSG00000175105:ENSG00000179021:ENSG00000184500:ENSG00000114446:ENSG00000017260:ENSG00000034533:ENSG00000240303:ENSG00000113971:ENSG00000081307:ENSG00000144867:ENS

						G00000154917:ENSG00000138231:ENSG00000184432:ENSG00000114115:ENSG00000155903:ENSG00000175066:ENSG00000114698:ENSG00000163762:ENSG00000169282:ENSG00000145014:ENSG00000013288:ENSG00000109689:ENSG00000169019:ENSG00000145246:ENSG00000163739:ENSG00000151247:ENSG00000205403:ENSG00000109466:ENSG00000129187:ENSG00000113360:ENSG00000164190:ENSG00000171522:ENSG00000132357:ENSG00000068796:ENSG00000086189:ENSG00000164331:ENSG00000164180:ENSG00000153140:ENSG00000168916:ENSG00000173926:ENSG00000064651:ENSG00000253159:ENSG00000240184:ENSG00000240764:ENSG00000197948:ENSG00000120318:ENSG00000131507:ENSG00000135074:ENSG00000113272:ENSG00000155858:ENSG00000131446:ENSG00000196670:ENSG00000137266:ENSG00000078401:ENSG00000146112:ENSG00000137404:ENSG00000137337:ENSG00000137331:ENSG00000204344:ENSG00000235863:ENSG00000231925:ENSG00000137168:ENSG00000164530:ENSG00000118432:ENSG00000198945:ENSG00000112357:ENSG00000118503:ENSG00000146416:ENSG00000120278:ENSG00000131018:ENSG00000026297:ENSG00000213066:ENSG00000186340:ENSG00000184786:ENSG00000130023:ENSG00000122644:ENSG00000153814:ENSG00000241644:ENSG00000122545:ENSG00000168303:ENSG00000152926:ENSG00000213462:ENSG00000146757:ENSG00000126522:ENSG00000169902:ENSG00000086730:ENSG00000127920:ENSG00000105829:ENSG00000085514:ENSG00000078487:ENSG00000160813:ENSG00000166925:ENSG00000005249:ENSG00000105865:ENSG00000075790:ENSG00000196937:ENSG00000091704:ENSG00000106484:ENSG00000122786:ENSG00000182158:ENSG00000146966:ENSG00000159763:ENSG00000196329:ENSG00000146918:ENSG00000035681:ENSG00000147601:ENSG00000121039:ENSG00000164949:ENSG00000120526:ENSG00000160932:ENSG00000185189:ENSG00000196378:ENSG00000080608:ENSG00000086065:ENSG00000213694:ENSG00000127080:ENSG00000130958:ENSG00000136936:ENSG00000136826:ENSG00000167081:ENSG00000148356:ENSG00000136840:ENSG00000136908:ENSG00000167106:ENSG00000171169:ENSG00000170835:ENSG00000148411
DEG. down	Nerve_Tibial	909	53	0.0288664014537024	1	ENSG00000179178:ENSG00000117472:ENSG00000117155:ENSG00000134215:ENSG00000163157:ENSG00000196189:ENSG00000075073:ENSG00000203867:ENSG00000166483:ENSG00000149809:ENSG00000154127:ENSG00000013588:ENSG00000139410:ENSG00000184992:ENSG00000139914:ENSG00000103888:ENSG00000157766:ENSG00000188522:ENSG00000142494:ENSG00000141750:ENSG00000141401:ENSG00000141469:ENSG00000205744:ENSG00000179751:ENSG00000028277:ENSG00000105427:ENSG00000197872:ENSG00000163803:ENSG00000116039:ENSG00000115607:ENSG00000160190:ENSG00000184792:ENSG00000138944:ENSG00000075275:ENSG00000008735:ENSG00000168032:ENSG00000186792:ENSG00000007402:ENSG00000154917:ENSG00000114115:ENSG00000080493:ENSG00000169429:ENSG00000163739:ENSG00000215218:ENSG00000240764:ENSG00000164620:ENSG00000124772:ENSG00000177679:ENSG00000197993:ENSG00000254087:ENSG00000104267:ENSG00000070159:ENSG00000198113
DEG. down	Ovary	1539	120	1.72818412120834e-09	9.33219425452503e-08	ENSG00000097021:ENSG00000171819:ENSG00000168389:ENSG00000179178:ENSG00000117399:ENSG00000117472:ENSG00000162367:ENSG00000134709:ENSG00000117155:ENSG00000153904:ENSG00000197147:ENSG00000163131:ENSG00000163154:ENSG00000163157:ENSG00000160781:ENSG00000203747:ENSG00000120057:ENSG00000184545:ENSG00000006071:ENSG00000124939:ENSG00000124935:ENSG00000162174:ENSG00000146670:ENSG00000175567:ENSG00000175564:ENSG00000062282:ENSG00000111679:ENSG00000134531:ENSG00000123338:ENSG00000235162:ENSG00000135094:ENSG00000184992:ENSG00000133110:ENSG00000136167:ENSG00000118939:ENSG00000102524:ENSG00000100889:ENSG00000139914:ENSG00000168952:ENSG00000176165:ENSG00000006432:ENSG00000170113:ENSG00000102879:ENSG00000180209:ENSG00000103064:ENSG00000185669:ENSG00000132514:ENSG00000188522:ENSG00000182963:ENSG00000123700:ENSG00000141576:ENSG00000185262:ENSG00000197971:ENSG00000205744:ENSG00000104833:ENSG00000065989:ENSG00000105369:ENSG00000105427:ENSG00000079435:ENSG00000079385:ENSG00000105383:ENSG00000115756:ENSG00000163803:ENSG00000116039:ENSG00000163170:ENSG00000115602:ENSG00000115604:ENSG00000115762:ENSG00000115963:ENSG00000144485:ENSG00000196209:ENSG00000125966:ENSG00000124102:ENSG00000124107:ENSG00000184792:ENSG00000075275:ENSG00000130489:ENSG00000025708:ENSG00000177989:ENSG00000205560:ENSG00000251322:ENSG00000170293:ENSG00000168032:ENSG00000182606:ENSG00000114378:ENSG00000144843:ENSG00000129048:ENSG00000154917:ENSG00000169282:ENSG00000163898:ENSG00000163975:ENSG00000157765:ENSG00000080493:ENSG00000163739:ENSG00000164128:ENSG00000109466:ENSG00000113361:ENSG00000171522:ENSG00000132357:ENSG00000175471:ENSG00000164620:ENSG00000113742:ENSG00000078401:ENSG00000235863:ENSG00000164530:ENSG00000118432:ENSG00000135525:ENSG00000122644:ENSG00000005073:ENSG00000165215:ENSG00000189143:ENSG00000086730:ENSG00000135218:ENSG00000159763:ENSG00000196329:ENSG00000254087:ENSG00000104267:ENSG00000131773:ENSG00000213694:ENSG00000070159
DEG. down	Pancreas	9668	625	3.2461053668572e-29	1.75289689810289e-27	ENSG00000116237:ENSG00000158292:ENSG00000097021:ENSG00000120948:ENSG00000171824:ENSG00000198793:ENSG00000120942:ENSG00000065526:ENSG00000116809:ENSG00000158747:ENSG00000070831:ENSG00000117616:ENSG00000183726:ENSG00000184007:ENSG00000084652:ENSG00000160050:ENSG00000160051:ENSG00000116525:ENSG00000160094:ENSG00000134686:ENSG00000127603:ENSG00000117395:ENSG00000066056:ENSG00000066322:ENSG00000159479:ENSG00000198198:ENSG00000117472:ENSG00000198841:ENSG00000134717:ENSG00000162384:ENSG00000162604:ENSG00000162616:ENSG00000117114:ENSG00000117133:ENSG00000117155:ENSG00000162642:ENSG00000142867:ENSG00000153904:ENSG00000197147:ENSG00000069712:ENSG00000117528:ENSG00000117569:ENSG00000162694:ENSG00000134215:ENSG00000162775:ENSG00000134248:ENSG00000085465:ENSG00000116455:ENSG00000163349:ENSG00000197323:ENSG00000116752:ENSG00000213281:ENSG00000052723:ENSG00000163131:ENSG00000143387:ENSG00000143437:ENSG00000143379:ENSG00000143363:ENSG00000143434:ENSG00000163154:ENSG00000163156:ENSG00000163155:ENSG00000143537:ENSG00000160767:ENSG00000116521:ENSG000001436

	30:ENSG00000160753:ENSG00000116539:ENSG00000125459:ENSG00000163374:ENSG00000132676:ENSG00000116580:ENSG00000132718:ENSG00000143622:ENSG00000132680:ENSG00000116584:ENSG00000160803:ENSG00000160789:ENSG00000196189:ENSG00000160783:ENSG00000242252:ENSG00000160781:ENSG00000198952:ENSG00000163472:ENSG00000198715:ENSG00000187800:ENSG00000132694:ENSG00000143226:ENSG00000173110:ENSG00000203747:ENSG00000152332:ENSG00000174175:ENSG00000186283:ENSG00000143322:ENSG00000177888:ENSG00000174307:ENSG00000122176:ENSG00000182004:ENSG00000069275:ENSG00000116962:ENSG00000015171:ENSG00000107929:ENSG00000241058:ENSG00000165997:ENSG00000099246:ENSG00000108094:ENSG00000095794:ENSG00000188234:ENSG00000165476:ENSG00000107738:ENSG00000138134:ENSG00000107796:ENSG00000026103:ENSG00000165338:ENSG00000155256:ENSG00000119927:ENSG00000165832:ENSG00000198873:ENSG00000166311:ENSG00000166313:ENSG00000205339:ENSG00000268403:ENSG00000166478:ENSG00000166483:ENSG00000198730:ENSG00000110442:ENSG00000019505:ENSG00000156587:ENSG00000162174:ENSG00000110025:ENSG00000168061:ENSG00000162300:ENSG00000174276:ENSG00000149792:ENSG00000173653:ENSG00000173599:ENSG00000173621:ENSG00000175567:ENSG00000062282:ENSG00000137693:ENSG00000149313:ENSG00000149292:ENSG00000180425:ENSG00000076053:ENSG00000076043:ENSG00000166250:ENSG00000082805:ENSG00000010219:ENSG00000139180:ENSG00000111667:ENSG00000111671:ENSG00000010626:ENSG00000111674:ENSG00000126749:ENSG00000013588:ENSG00000134531:ENSG00000123094:ENSG00000110925:ENSG00000135457:ENSG00000123360:ENSG00000127314:ENSG00000166130:ENSG00000120868:ENSG00000111647:ENSG00000074590:ENSG00000174989:ENSG00000111725:ENSG00000177084:ENSG00000176915:ENSG00000127863:ENSG00000139505:ENSG00000122033:ENSG00000133110:ENSG00000183722:ENSG00000120688:ENSG00000136167:ENSG00000102753:ENSG00000136104:ENSG00000188243:ENSG00000118939:ENSG00000136153:ENSG00000178695:ENSG00000134901:ENSG00000134897:ENSG00000139826:ENSG00000102524:ENSG00000092140:ENSG00000129493:ENSG00000129480:ENSG00000165355:ENSG00000179476:ENSG00000179454:ENSG00000198718:ENSG00000185246:ENSG00000100442:ENSG00000139926:ENSG00000087301:ENSG00000197930:ENSG00000165861:ENSG00000119707:ENSG00000080815:ENSG00000100767:ENSG00000133961:ENSG00000165417:ENSG00000165934:ENSG00000165943:ENSG00000153485:ENSG00000170270:ENSG00000012963:ENSG00000140157:ENSG00000170113:ENSG00000128731:ENSG00000021776:ENSG00000198146:ENSG00000174197:ENSG00000137802:ENSG00000243708:ENSG00000197535:ENSG00000128989:ENSG00000181827:ENSG00000140262:ENSG00000103707:ENSG00000103710:ENSG00000140350:ENSG00000117899:ENSG00000140511:ENSG00000140471:ENSG00000185418:ENSG00000008710:ENSG00000183426:ENSG00000157045:ENSG00000085721:ENSG00000157106:ENSG00000102879:ENSG00000169217:ENSG00000169221:ENSG00000180035:ENSG00000179965:ENSG00000179958:ENSG00000140691:ENSG00000140682:ENSG00000197302:ENSG00000102910:ENSG00000196470:ENSG00000102921:ENSG00000005194:ENSG00000088682:ENSG00000102978:ENSG00000072736:ENSG00000103066:ENSG00000103064:ENSG00000103061:ENSG00000132600:ENSG00000103056:ENSG00000103018:ENSG00000185669:ENSG00000158717:ENSG00000167515:ENSG00000167522:ENSG00000197912:ENSG00000127804:ENSG00000196689:ENSG00000029725:ENSG00000005100:ENSG00000181350:ENSG00000141127:ENSG00000072134:ENSG00000166484:ENSG00000166482:ENSG00000128482:ENSG00000067191:ENSG00000068079:ENSG00000188554:ENSG00000184988:ENSG00000225190:ENSG00000141279:ENSG00000108424:ENSG00000198933:ENSG00000008294:ENSG00000011258:ENSG00000011260:ENSG00000153933:ENSG00000121060:ENSG00000108423:ENSG00000108443:ENSG00000198265:ENSG00000197170:ENSG00000141540:ENSG00000141576:ENSG00000185262:ENSG00000078687:ENSG00000141577:ENSG00000167302:ENSG00000224877:ENSG00000182446:ENSG00000017797:ENSG00000154845:ENSG00000134265:ENSG00000154889:ENSG00000168234:ENSG00000141384:ENSG00000152223:ENSG00000081913:ENSG00000130856:ENSG00000197971:ENSG00000071564:ENSG00000141985:ENSG00000167670:ENSG00000130254:ENSG00000160633:ENSG00000104833:ENSG00000104883:ENSG00000167775:ENSG00000065989:ENSG00000079999:ENSG00000104957:ENSG00000132017:ENSG00000132005:ENSG00000123146:ENSG00000123136:ENSG00000123159:ENSG00000132002:ENSG00000127527:ENSG00000104825:ENSG00000128626:ENSG00000269190:ENSG00000160570:ENSG00000167625:ENSG00000105723:ENSG00000105722:ENSG00000079432:ENSG00000079462:ENSG00000167619:ENSG00000105429:ENSG00000105427:ENSG00000079435:ENSG00000079385:ENSG00000185453:ENSG00000105483:ENSG00000105447:ENSG00000142544:ENSG00000196214:ENSG00000198464:ENSG00000167766:ENSG00000213020:ENSG00000176293:ENSG00000115756:ENSG00000115761:ENSG00000197872:ENSG00000143867:ENSG00000055917:ENSG00000084676:ENSG00000213639:ENSG00000171103:ENSG00000119866:ENSG00000162928:ENSG00000162929:ENSG00000082898:ENSG00000116039:ENSG00000075292:ENSG00000144034:ENSG00000144048:ENSG00000163170:ENSG00000114978:ENSG00000204843:ENSG00000005448:ENSG00000115602:ENSG00000115604:ENSG00000153107:ENSG00000153208:ENSG00000153214:ENSG00000152102:ENSG00000115762:ENSG00000163040:ENSG00000115963:ENSG00000144283:ENSG00000153250:ENSG00000118260:ENSG00000144401:ENSG00000197713:ENSG00000115461:ENSG00000018280:ENSG00000135912:ENSG00000168958:ENSG00000198612:ENSG00000132326:ENSG00000162804:ENSG00000088876:ENSG00000125779:ENSG00000089063:ENSG00000132646:ENSG00000101290:ENSG00000089048:ENSG00000101247:ENSG00000198959:ENSG00000124107:ENSG00000124228:ENSG00000124201:ENSG00000203896:ENSG00000125520:ENSG00000101150:ENSG00000101152:ENSG00000198276:ENSG00000155313:ENSG00000154642:ENSG00000157617:ENSG00000160190:ENSG00000160284:ENSG00000160285:ENSG00000183628:ENSG00000184792:ENSG00000128335:ENSG00000100342:ENSG00000100321:ENSG00000100324:ENSG00000198951:ENSG00000184983:ENSG00000100197:ENSG00000077942:ENSG00000186951:ENSG00000205643:ENSG00000100416:ENSG00000205593:ENSG00000025770:ENSG00000130489:ENSG00000025708:ENSG00000177989:ENSG00000205560:ENSG00000100288:ENSG00000008735:ENSG00000251322:ENSG00000070950:ENSG00000134077:ENSG00000144560:ENSG00000144712:ENSG00000182606:ENS

						G00000114353:ENSG00000186792:ENSG00000243477:ENSG00000114378:ENSG00000068001:ENSG00000114383:ENSG00000068028:ENSG00000004838:ENSG00000126062:ENSG00000007402:ENSG00000180376:ENSG00000163946:ENSG00000144741:ENSG00000114861:ENSG00000144736:ENSG00000163320:ENSG00000175105:ENSG00000179021:ENSG00000184500:ENSG00000169379:ENSG00000178700:ENSG00000114446:ENSG00000163607:ENSG00000163608:ENSG00000144843:ENSG00000082701:ENSG00000065518:ENSG00000172780:ENSG00000240682:ENSG00000017260:ENSG00000034533:ENSG00000114670:ENSG00000240303:ENSG00000113971:ENSG00000154917:ENSG00000138231:ENSG00000114098:ENSG00000158186:ENSG00000175110:ENSG00000155903:ENSG00000175066:ENSG00000114127:ENSG00000114698:ENSG00000163762:ENSG00000169282:ENSG00000161202:ENSG00000161204:ENSG00000163904:ENSG00000114279:ENSG00000145014:ENSG00000041802:ENSG00000163975:ENSG00000013288:ENSG00000109689:ENSG00000169019:ENSG00000145246:ENSG00000109171:ENSG00000109321:ENSG00000173085:ENSG00000151247:ENSG00000109466:ENSG00000129187:ENSG00000113360:ENSG00000082213:ENSG00000164190:ENSG00000132357:ENSG00000067248:ENSG00000039123:ENSG00000067113:ENSG00000068796:ENSG00000086189:ENSG00000086200:ENSG00000164331:ENSG00000164338:ENSG00000164180:ENSG00000153140:ENSG00000181751:ENSG00000168916:ENSG00000173926:ENSG00000064651:ENSG00000240184:ENSG00000171720:ENSG00000164620:ENSG00000197948:ENSG00000120318:ENSG00000113721:ENSG00000113272:ENSG00000155858:ENSG00000113312:ENSG00000113742:ENSG00000131446:ENSG00000196670:ENSG00000124535:ENSG00000124787:ENSG00000078401:ENSG00000079691:ENSG00000146112:ENSG00000137404:ENSG00000137337:ENSG00000204344:ENSG00000223501:ENSG00000235863:ENSG00000227057:ENSG00000204220:ENSG00000237441:ENSG00000137168:ENSG00000198663:ENSG00000156639:ENSG00000198945:ENSG00000118503:ENSG00000112406:ENSG00000146416:ENSG00000120278:ENSG00000131018:ENSG00000272047:ENSG00000026297:ENSG00000213066:ENSG00000186340:ENSG00000184786:ENSG00000130023:ENSG00000122644:ENSG00000105855:ENSG00000153814:ENSG00000241644:ENSG00000122545:ENSG00000168303:ENSG00000152926:ENSG00000213462:ENSG00000146757:ENSG00000169902:ENSG00000086730:ENSG00000049541:ENSG00000170027:ENSG00000135218:ENSG00000127920:ENSG00000085514:ENSG00000078487:ENSG00000146834:ENSG00000160813:ENSG00000166925:ENSG00000005249:ENSG00000105865:ENSG00000075790:ENSG00000158623:ENSG00000131558:ENSG00000122786:ENSG00000182158:ENSG00000181444:ENSG00000196329:ENSG00000214106:ENSG00000146918:ENSG00000117868:ENSG00000154319:ENSG00000120910:ENSG00000129691:ENSG00000023287:ENSG00000254087:ENSG00000035681:ENSG00000147601:ENSG00000121039:ENSG00000164949:ENSG00000120526:ENSG00000120533:ENSG00000129295:ENSG00000131773:ENSG00000160932:ENSG00000180900:ENSG00000179950:ENSG00000185189:ENSG00000196378:ENSG00000080608:ENSG00000086065:ENSG00000135018:ENSG00000213694:ENSG00000127080:ENSG00000136925:ENSG00000136936:ENSG00000136826:ENSG00000119446:ENSG00000167081:ENSG00000196152:ENSG00000148356:ENSG00000171169:ENSG00000148335:ENSG00000148308:ENSG00000130560:ENSG00000148411:ENSG00000188986:ENSG00000198113
DEG. down	Pituitary	1937	110	0.00532359549477943	0.287474156718089	ENSG00000187942:ENSG00000162366:ENSG00000142867:ENSG00000134215:ENSG00000175984:ENSG00000213281:ENSG00000163131:ENSG00000143412:ENSG00000163154:ENSG00000163354:ENSG00000116584:ENSG00000160803:ENSG00000196189:ENSG00000163050:ENSG00000116962:ENSG00000148468:ENSG00000107736:ENSG00000107738:ENSG00000107796:ENSG00000026103:ENSG00000120057:ENSG00000119927:ENSG00000149150:ENSG00000062282:ENSG00000137693:ENSG00000166250:ENSG00000013588:ENSG00000134531:ENSG00000123338:ENSG00000123360:ENSG00000235162:ENSG00000074590:ENSG00000135094:ENSG00000177084:ENSG00000133110:ENSG00000102743:ENSG00000136167:ENSG00000102753:ENSG00000136153:ENSG00000178695:ENSG00000102524:ENSG00000100889:ENSG00000139914:ENSG00000139926:ENSG00000119599:ENSG00000080815:ENSG00000186198:ENSG00000103710:ENSG00000136378:ENSG00000140600:ENSG00000140511:ENSG00000157045:ENSG00000180096:ENSG00000179958:ENSG00000140682:ENSG00000072736:ENSG00000132514:ENSG00000141750:ENSG00000123700:ENSG00000017797:ENSG00000141384:ENSG00000141469:ENSG00000197971:ENSG00000104833:ENSG00000065989:ENSG00000123146:ENSG00000105427:ENSG00000079435:ENSG00000142273:ENSG00000063127:ENSG00000105383:ENSG00000143867:ENSG00000119866:ENSG00000114978:ENSG00000115604:ENSG00000115607:ENSG00000115963:ENSG00000196209:ENSG00000124102:ENSG00000155313:ENSG00000157617:ENSG00000170293:ENSG00000114378:ENSG00000184500:ENSG00000144843:ENSG00000169282:ENSG00000145014:ENSG00000109171:ENSG00000163739:ENSG00000171522:ENSG00000132357:ENSG00000135074:ENSG00000078401:ENSG00000079691:ENSG00000146112:ENSG00000137404:ENSG00000204544:ENSG00000118503:ENSG00000112406:ENSG00000122644:ENSG00000135218:ENSG00000085563:ENSG00000075790:ENSG00000159763:ENSG00000196329:ENSG00000104267:ENSG00000165071:ENSG00000136826:ENSG00000148411:ENSG00000198113
DEG. down	Prostate	744	45	0.0244944801430078	1	ENSG00000168389:ENSG00000117399:ENSG00000171488:ENSG00000152779:ENSG00000119927:ENSG00000146670:ENSG00000062282:ENSG00000154127:ENSG00000064309:ENSG00000123360:ENSG00000139292:ENSG00000122966:ENSG00000184992:ENSG00000197535:ENSG00000140986:ENSG00000172137:ENSG00000188522:ENSG00000141540:ENSG00000141576:ENSG00000081913:ENSG00000197971:ENSG00000167619:ENSG00000079435:ENSG00000144485:ENSG00000183628:ENSG00000184792:ENSG00000154917:ENSG00000158186:ENSG00000114279:ENSG00000164128:ENSG00000171522:ENSG00000132357:ENSG00000175471:ENSG00000173926:ENSG00000253159:ENSG00000113742:ENSG00000124743:ENSG00000118432:ENSG00000132429:ENSG00000120278:ENSG00000135218:ENSG00000166924:ENSG00000154319:ENSG00000104267:ENSG00000127080

DEG. down	Skin_Not_Sun_Exposed_Suprapubic	2708	156	0.000522643349852791	0.0282227408920507	ENSG00000158286:ENSG00000187942:ENSG00000160050:ENSG00000066056:ENSG00000162367:ENSG00000162616:ENSG00000117155:ENSG00000153904:ENSG00000069712:ENSG00000162694:ENSG00000168679:ENSG00000163131:ENSG00000163154:ENSG00000132718:ENSG00000116584:ENSG00000187800:ENSG00000143226:ENSG00000203747:ENSG00000174175:ENSG00000120341:ENSG00000162714:ENSG00000198879:ENSG00000148468:ENSG00000095794:ENSG00000075073:ENSG00000107736:ENSG00000165338:ENSG00000120057:ENSG00000203867:ENSG00000119927:ENSG00000198873:ENSG00000184545:ENSG00000166313:ENSG00000268403:ENSG00000187486:ENSG00000162174:ENSG00000168060:ENSG00000173599:ENSG00000175567:ENSG00000175564:ENSG00000010626:ENSG00000123094:ENSG00000123338:ENSG00000123360:ENSG00000166130:ENSG00000139410:ENSG00000122966:ENSG00000102531:ENSG00000152749:ENSG00000134901:ENSG00000102524:ENSG00000139914:ENSG00000129493:ENSG00000103710:ENSG00000136378:ENSG00000140406:ENSG00000185418:ENSG00000102879:ENSG00000180209:ENSG00000179965:ENSG00000158717:ENSG00000108641:ENSG00000128482:ENSG00000068079:ENSG00000108830:ENSG00000184988:ENSG00000073670:ENSG00000182963:ENSG00000198933:ENSG00000214226:ENSG00000153933:ENSG00000154217:ENSG00000141540:ENSG00000196169:ENSG00000078687:ENSG00000104833:ENSG00000167775:ENSG00000065989:ENSG00000123146:ENSG00000123143:ENSG00000028277:ENSG00000105429:ENSG00000105483:ENSG00000063127:ENSG00000105383:ENSG00000115705:ENSG00000115756:ENSG00000197872:ENSG00000163170:ENSG00000005448:ENSG00000153208:ENSG00000144401:ENSG00000018280:ENSG00000144485:ENSG00000196209:ENSG00000089063:ENSG00000101290:ENSG00000198959:ENSG00000128335:ENSG00000100342:ENSG00000138944:ENSG00000205593:ENSG00000008735:ENSG00000144712:ENSG00000170011:ENSG00000114383:ENSG00000144843:ENSG00000172780:ENSG00000154917:ENSG00000158186:ENSG00000114698:ENSG00000163762:ENSG00000169282:ENSG00000174899:ENSG00000075420:ENSG00000145014:ENSG00000163739:ENSG00000205403:ENSG00000109466:ENSG00000215218:ENSG00000132357:ENSG00000175471:ENSG00000181751:ENSG00000168916:ENSG00000120318:ENSG00000135074:ENSG00000124772:ENSG00000137168:ENSG00000124743:ENSG00000118432:ENSG00000091831:ENSG00000005073:ENSG00000153814:ENSG00000241644:ENSG00000086730:ENSG00000177679:ENSG00000085563:ENSG00000085514:ENSG00000166924:ENSG00000005249:ENSG00000106477:ENSG00000106484:ENSG00000182158:ENSG00000146966:ENSG00000196329:ENSG00000168546:ENSG00000254087:ENSG00000129295:ENSG00000165071:ENSG00000131773:ENSG00000044459:ENSG00000178031:ENSG00000213694:ENSG00000136840:ENSG00000148411:ENSG00000198113
DEG. down	Skin_Sun_Exposed_Lower_leg	2490	143	0.00101442501128077	0.0547789506091614	ENSG00000158286:ENSG00000187942:ENSG00000160050:ENSG00000117472:ENSG00000162367:ENSG00000162616:ENSG00000117155:ENSG00000153904:ENSG00000069712:ENSG00000162694:ENSG00000168679:ENSG00000163131:ENSG00000163154:ENSG00000132718:ENSG00000116584:ENSG00000242252:ENSG00000143226:ENSG00000173110:ENSG00000203747:ENSG00000174175:ENSG00000120341:ENSG00000057252:ENSG00000198879:ENSG00000148468:ENSG00000095794:ENSG00000075073:ENSG00000107736:ENSG00000152779:ENSG00000120057:ENSG00000203867:ENSG00000119927:ENSG00000184545:ENSG00000166313:ENSG00000268403:ENSG00000187486:ENSG00000162174:ENSG00000168060:ENSG00000173599:ENSG00000175567:ENSG00000175564:ENSG00000010626:ENSG00000123094:ENSG00000123338:ENSG00000123360:ENSG00000166130:ENSG00000139410:ENSG00000122966:ENSG00000102531:ENSG00000152749:ENSG00000102524:ENSG00000139914:ENSG00000170270:ENSG00000136378:ENSG00000140406:ENSG00000185418:ENSG00000102879:ENSG00000179965:ENSG00000158717:ENSG00000181350:ENSG00000108641:ENSG00000128482:ENSG00000184988:ENSG00000073670:ENSG00000182963:ENSG00000198933:ENSG00000214226:ENSG00000153933:ENSG00000154217:ENSG00000141540:ENSG00000141576:ENSG00000078687:ENSG00000104833:ENSG00000167775:ENSG00000065989:ENSG00000028277:ENSG00000105429:ENSG00000105483:ENSG00000105383:ENSG00000115705:ENSG00000115756:ENSG00000197872:ENSG00000163170:ENSG00000144218:ENSG00000153208:ENSG00000144401:ENSG00000018280:ENSG00000144485:ENSG00000196209:ENSG00000101290:ENSG00000198959:ENSG00000100342:ENSG00000138944:ENSG00000205593:ENSG00000144712:ENSG00000170011:ENSG00000114383:ENSG00000144843:ENSG00000154917:ENSG00000158186:ENSG00000114115:ENSG00000163762:ENSG00000169282:ENSG00000075420:ENSG00000163739:ENSG00000185477:ENSG00000205403:ENSG00000109466:ENSG00000215218:ENSG00000113361:ENSG00000132357:ENSG00000068796:ENSG00000181751:ENSG00000168916:ENSG00000120318:ENSG00000135074:ENSG00000124772:ENSG00000137168:ENSG00000118432:ENSG00000005073:ENSG00000153814:ENSG00000241644:ENSG00000152926:ENSG00000146757:ENSG00000086730:ENSG00000177679:ENSG00000085563:ENSG00000085514:ENSG00000166924:ENSG00000005249:ENSG00000106477:ENSG00000106484:ENSG00000182158:ENSG00000146966:ENSG00000168546:ENSG00000254087:ENSG00000129295:ENSG00000165071:ENSG00000131773:ENSG00000178031:ENSG00000213694:ENSG00000136840:ENSG00000148411:ENSG00000198113
DEG. down	Small_Intestine_Terminal_Ileum	1384	82	0.00547650585243667	0.29573131603158	ENSG00000069812:ENSG00000197147:ENSG00000069712:ENSG00000092621:ENSG00000143412:ENSG00000132718:ENSG00000196189:ENSG00000160781:ENSG00000173110:ENSG00000203747:ENSG00000174307:ENSG00000134463:ENSG00000095794:ENSG00000107736:ENSG00000203867:ENSG00000119927:ENSG00000187486:ENSG00000006071:ENSG00000149809:ENSG00000173621:ENSG00000062282:ENSG00000010626:ENSG00000134532:ENSG00000123094:ENSG00000074590:ENSG00000127863:ENSG00000203546:ENSG00000103888:ENSG00000103066:ENSG00000072134:ENSG00000108641:ENSG00000142494:ENSG00000067191:ENSG00000141750:ENSG00000108830:ENSG00000073670:ENSG00000123700:ENSG00000141540:ENSG00000081913:ENSG00000197971:ENSG00000104833:ENSG00000132000:ENSG00000269190:ENSG00000116039:ENSG00000188687:ENSG00000163040:ENSG00000153832:ENSG00000125966:ENSG00000183628:ENSG00000184792:ENSG00000100321:ENSG00000008735:ENSG00000144712:ENSG00000163517:ENSG00000168032:ENSG00000004838:ENSG00000007402:ENSG00000114670:ENSG00000154917:ENSG00000158163:ENSG00000158186:ENSG00000169282:ENSG00000164128:ENSG00000109466:ENSG00000215218:ENSG000001526

						69:ENSG00000253159:ENSG00000240184:ENSG00000113742:ENSG00000164530:ENSG00000131018:ENSG00000272047:ENSG00000152926:ENSG00000177679:ENSG00000185955:ENSG00000166924:ENSG00000075790:ENSG00000154319:ENSG00000104267:ENSG00000129295:ENSG00000131773:ENSG00000127080
DEG. down	Spleen	2075	104	0.113770595766144	1	ENSG00000158286:ENSG00000116237:ENSG00000158747:ENSG00000168389:ENSG00000117472:ENSG00000162366:ENSG00000134709:ENSG00000117155:ENSG00000153904:ENSG00000197147:ENSG00000069712:ENSG00000117528:ENSG00000117525:ENSG00000092621:ENSG00000134240:ENSG00000143412:ENSG00000143434:ENSG00000163354:ENSG00000143630:ENSG00000132718:ENSG00000174175:ENSG00000143322:ENSG00000174307:ENSG00000162885:ENSG00000165476:ENSG00000119927:ENSG00000184545:ENSG00000205339:ENSG00000187486:ENSG00000162174:ENSG00000149809:ENSG00000173599:ENSG00000137672:ENSG00000137693:ENSG00000023171:ENSG00000064309:ENSG00000082805:ENSG00000010626:ENSG00000134532:ENSG00000123094:ENSG00000235162:ENSG00000074590:ENSG00000102743:ENSG00000102753:ENSG00000136153:ENSG00000198146:ENSG00000103888:ENSG00000179965:ENSG00000188522:ENSG00000072134:ENSG00000108641:ENSG00000067191:ENSG00000008294:ENSG00000141576:ENSG00000081913:ENSG00000132016:ENSG00000269190:ENSG00000167619:ENSG00000188687:ENSG00000114993:ENSG00000115602:ENSG00000163040:ENSG00000144283:ENSG00000198612:ENSG00000132326:ENSG00000160285:ENSG00000183628:ENSG00000100321:ENSG00000077942:ENSG00000008735:ENSG00000144560:ENSG00000144712:ENSG00000163517:ENSG00000170293:ENSG00000007402:ENSG00000144749:ENSG00000114670:ENSG00000129048:ENSG00000158186:ENSG00000175066:ENSG00000169282:ENSG00000163975:ENSG00000109171:ENSG00000123739:ENSG00000168916:ENSG00000064651:ENSG00000253159:ENSG00000240184:ENSG00000079691:ENSG00000112812:ENSG00000164530:ENSG00000112245:ENSG00000118432:ENSG00000135525:ENSG00000112357:ENSG00000186340:ENSG00000166924:ENSG00000075790:ENSG00000117868:ENSG00000154319:ENSG00000023287:ENSG00000121039:ENSG00000131773:ENSG00000148411
DEG. down	Stomach	2622	160	2.72312241806767e-05	0.00147048610575654	ENSG00000158292:ENSG00000171819:ENSG00000160050:ENSG00000168389:ENSG00000066056:ENSG00000162367:ENSG00000117155:ENSG00000197147:ENSG00000171488:ENSG00000069712:ENSG00000085465:ENSG00000175984:ENSG00000143412:ENSG00000143434:ENSG00000163154:ENSG00000163354:ENSG00000143630:ENSG00000132718:ENSG00000196189:ENSG00000160781:ENSG00000163472:ENSG00000187800:ENSG00000143226:ENSG00000203747:ENSG00000095794:ENSG00000107736:ENSG00000026103:ENSG00000203867:ENSG00000119927:ENSG00000110169:ENSG00000173621:ENSG00000175564:ENSG00000062282:ENSG00000137672:ENSG00000149313:ENSG00000154127:ENSG00000010626:ENSG00000123338:ENSG00000139292:ENSG00000166130:ENSG00000120868:ENSG00000074590:ENSG00000135094:ENSG00000127863:ENSG00000102524:ENSG00000139914:ENSG00000139926:ENSG00000165417:ENSG00000156414:ENSG00000198146:ENSG00000197535:ENSG00000186198:ENSG00000103710:ENSG00000136378:ENSG00000140511:ENSG00000072736:ENSG00000103066:ENSG00000103064:ENSG00000172137:ENSG00000029725:ENSG00000072134:ENSG00000108641:ENSG00000128482:ENSG00000142494:ENSG00000067191:ENSG00000108830:ENSG00000073670:ENSG00000008294:ENSG00000123700:ENSG00000141540:ENSG00000196169:ENSG00000141576:ENSG00000078687:ENSG00000017797:ENSG00000141469:ENSG00000081913:ENSG00000197971:ENSG00000104833:ENSG00000132016:ENSG00000099308:ENSG00000028277:ENSG00000167619:ENSG00000105429:ENSG00000105427:ENSG00000079435:ENSG00000105383:ENSG00000176293:ENSG00000197872:ENSG00000005448:ENSG00000163040:ENSG00000144283:ENSG00000153832:ENSG00000162804:ENSG00000196209:ENSG00000154642:ENSG00000183628:ENSG00000184792:ENSG00000100321:ENSG00000100197:ENSG00000205593:ENSG00000177989:ENSG00000008735:ENSG00000251322:ENSG00000144712:ENSG00000163517:ENSG00000168032:ENSG00000184500:ENSG00000114670:ENSG00000154917:ENSG00000158186:ENSG00000155903:ENSG00000114698:ENSG00000163762:ENSG00000169282:ENSG00000163975:ENSG00000205403:ENSG00000164128:ENSG00000109466:ENSG00000215218:ENSG00000113361:ENSG00000068796:ENSG00000153140:ENSG00000175471:ENSG00000173926:ENSG00000253159:ENSG00000240184:ENSG00000164620:ENSG00000120318:ENSG00000137404:ENSG00000124772:ENSG00000118432:ENSG00000198945:ENSG00000146416:ENSG00000120278:ENSG00000186340:ENSG00000105855:ENSG00000153814:ENSG00000152926:ENSG00000213462:ENSG00000169902:ENSG00000086730:ENSG00000177679:ENSG00000085563:ENSG00000085514:ENSG00000078487:ENSG00000166924:ENSG00000075790:ENSG00000106477:ENSG00000106484:ENSG00000158623:ENSG00000146966:ENSG00000154319:ENSG00000168546:ENSG00000120910:ENSG00000129295:ENSG00000165071:ENSG00000131773:ENSG00000196378:ENSG00000127080:ENSG00000182752
DEG. down	Testis	2951	160	0.00521898910192598	0.281825411504003	ENSG00000158286:ENSG00000116237:ENSG00000158292:ENSG00000187942:ENSG00000183726:ENSG00000157978:ENSG00000066056:ENSG00000162366:ENSG00000162367:ENSG00000117114:ENSG00000163131:ENSG00000163154:ENSG00000143537:ENSG00000160753:ENSG00000196189:ENSG00000198715:ENSG00000143226:ENSG00000173110:ENSG00000203747:ENSG00000174175:ENSG00000186283:ENSG00000174307:ENSG00000122176:ENSG00000163050:ENSG00000116962:ENSG00000198879:ENSG00000148468:ENSG00000075073:ENSG00000107738:ENSG00000026103:ENSG00000120057:ENSG00000198873:ENSG00000184545:ENSG00000205339:ENSG00000187486:ENSG00000006071:ENSG00000019505:ENSG00000165905:ENSG00000149150:ENSG00000156587:ENSG00000110025:ENSG00000137693:ENSG00000166250:ENSG00000111674:ENSG00000111679:ENSG00000111684:ENSG00000013588:ENSG00000134531:ENSG00000185432:ENSG00000123338:ENSG00000123360:ENSG00000074590:ENSG00000127863:ENSG00000133110:ENSG00000136153:ENSG00000152749:ENSG00000134901:ENSG00000134897:ENSG00000168952:ENSG00000100473:ENSG00000197930:ENSG00000197535:ENSG00000140350:ENSG00000140406:ENSG00000140511:ENSG00000102879:ENSG00000180096:ENSG00000102910:ENSG00000188522:ENSG00000068079:ENSG00000225190:ENSG00000154217:ENSG00000017797:ENSG00000141401:ENSG00000197971:ENSG00000205744:ENSG00000180739:ENSG00000123146:ENSG00000099308:ENSG00000269190:ENSG00000105427:ENSG00000142273:ENSG

						00000105383:ENSG00000171049:ENSG00000197872:ENSG00000119866:ENSG00000114993:ENSG00000115602:ENSG00000115604:ENSG00000153214:ENSG00000115963:ENSG00000153250:ENSG00000018280:ENSG00000198959:ENSG00000124102:ENSG00000157617:ENSG00000128335:ENSG00000100342:ENSG00000177096:ENSG00000138944:ENSG00000077942:ENSG00000186951:ENSG00000075234:ENSG00000075275:ENSG00000025708:ENSG00000251322:ENSG00000163513:ENSG00000170293:ENSG00000170011:ENSG00000114378:ENSG00000114861:ENSG00000184500:ENSG00000144843:ENSG00000158186:ENSG00000163762:ENSG00000169282:ENSG00000075420:ENSG00000013288:ENSG00000145246:ENSG00000123739:ENSG00000205403:ENSG00000164128:ENSG00000109466:ENSG00000129187:ENSG00000215218:ENSG00000132357:ENSG00000175471:ENSG00000240184:ENSG00000120318:ENSG00000135074:ENSG00000074317:ENSG00000078401:ENSG00000146112:ENSG00000137331:ENSG00000244731:ENSG00000118432:ENSG00000112096:ENSG00000026297:ENSG00000186340:ENSG00000126522:ENSG00000165215:ENSG00000086730:ENSG00000135218:ENSG00000085563:ENSG00000127920:ENSG00000196937:ENSG00000182158:ENSG00000146966:ENSG00000159763:ENSG00000181444:ENSG00000196329:ENSG00000254087:ENSG00000035681:ENSG00000164949:ENSG00000165071:ENSG00000213694:ENSG00000130958:ENSG00000136840:ENSG00000148339:ENSG00000198113
DEG. down	Thyroid	1042	63	0.00917922292183655	0.495678037779173	ENSG00000158292:ENSG00000097021:ENSG00000171819:ENSG00000162366:ENSG00000175984:ENSG00000134240:ENSG00000143434:ENSG00000163154:ENSG00000132718:ENSG00000107736:ENSG00000138134:ENSG00000120057:ENSG00000184545:ENSG00000019505:ENSG00000168061:ENSG00000146670:ENSG00000166250:ENSG00000023171:ENSG00000111262:ENSG00000123338:ENSG00000235162:ENSG00000122966:ENSG00000127863:ENSG00000139914:ENSG00000100473:ENSG00000073670:ENSG00000123700:ENSG00000141576:ENSG00000168234:ENSG00000269190:ENSG00000179751:ENSG00000028277:ENSG00000105427:ENSG00000142273:ENSG00000105383:ENSG00000119866:ENSG00000198885:ENSG00000115607:ENSG00000115963:ENSG00000186235:ENSG00000144485:ENSG00000196209:ENSG00000124102:ENSG00000138944:ENSG00000008735:ENSG00000114115:ENSG00000114279:ENSG00000163739:ENSG00000109321:ENSG00000145287:ENSG00000215218:ENSG00000078401:ENSG00000124743:ENSG00000086730:ENSG00000177679:ENSG00000185955:ENSG00000166924:ENSG00000005249:ENSG00000146966:ENSG00000159763:ENSG00000131773:ENSG00000213694:ENSG00000148411
DEG. down	Uterus	846	57	0.00138031764277188	0.0745371527096817	ENSG00000171819:ENSG00000168389:ENSG00000117399:ENSG00000197147:ENSG00000175984:ENSG00000163131:ENSG00000160781:ENSG00000203747:ENSG00000120057:ENSG00000187486:ENSG00000165905:ENSG00000146670:ENSG00000149809:ENSG00000173599:ENSG00000175567:ENSG00000062282:ENSG00000154127:ENSG00000111679:ENSG00000123338:ENSG00000139292:ENSG00000122966:ENSG00000184992:ENSG00000136153:ENSG00000102524:ENSG00000100889:ENSG00000139914:ENSG00000172137:ENSG00000185669:ENSG00000123700:ENSG00000196169:ENSG00000197971:ENSG00000205744:ENSG00000129354:ENSG00000132000:ENSG00000028277:ENSG00000196209:ENSG00000124102:ENSG00000124107:ENSG00000168032:ENSG00000169429:ENSG00000163739:ENSG00000109321:ENSG00000215218:ENSG00000152669:ENSG00000181751:ENSG00000112812:ENSG00000132429:ENSG00000135525:ENSG00000189143:ENSG00000086730:ENSG00000135218:ENSG00000159763:ENSG00000254087:ENSG00000104267:ENSG00000165071:ENSG00000131773:ENSG00000070159
DEG. down	Vagina	649	39	0.0372680270670196	1	ENSG00000153904:ENSG00000203747:ENSG00000119927:ENSG00000184545:ENSG00000162174:ENSG00000173599:ENSG00000175567:ENSG00000175564:ENSG00000154127:ENSG00000139292:ENSG00000139410:ENSG00000139914:ENSG00000168952:ENSG00000185669:ENSG00000141576:ENSG00000141469:ENSG00000104833:ENSG00000028277:ENSG00000115705:ENSG00000115607:ENSG00000144485:ENSG00000183628:ENSG00000184792:ENSG00000008735:ENSG00000007402:ENSG00000129048:ENSG00000154917:ENSG00000158186:ENSG00000152669:ENSG00000181751:ENSG00000173926:ENSG00000064651:ENSG00000244731:ENSG00000132429:ENSG00000086730:ENSG00000166924:ENSG00000106484:ENSG00000159763:ENSG00000104267
DEG. down	Whole_Blood	6940	470	3.1800867716206e-24	1.71724685667512e-22	ENSG00000116237:ENSG00000158292:ENSG00000097021:ENSG00000171603:ENSG00000120948:ENSG00000171824:ENSG00000198793:ENSG00000120942:ENSG00000065526:ENSG00000158747:ENSG00000204178:ENSG00000084652:ENSG00000160050:ENSG00000160051:ENSG00000116525:ENSG00000160094:ENSG00000127603:ENSG00000168389:ENSG00000117395:ENSG00000159479:ENSG00000198198:ENSG00000198841:ENSG00000134717:ENSG00000162384:ENSG00000162604:ENSG00000162616:ENSG00000117133:ENSG00000117155:ENSG00000162642:ENSG00000117528:ENSG00000117569:ENSG00000117543:ENSG00000085465:ENSG00000116455:ENSG00000197323:ENSG00000116752:ENSG00000213281:ENSG00000052723:ENSG00000092621:ENSG00000143457:ENSG00000143387:ENSG00000143437:ENSG00000143379:ENSG00000143412:ENSG00000143363:ENSG00000143434:ENSG00000163155:ENSG00000163157:ENSG00000143537:ENSG00000160767:ENSG00000116521:ENSG00000176444:ENSG00000160753:ENSG00000116539:ENSG00000125459:ENSG00000132676:ENSG00000116580:ENSG00000132718:ENSG00000132680:ENSG00000160803:ENSG00000160789:ENSG00000242252:ENSG00000163472:ENSG00000187800:ENSG00000152332:ENSG00000186283:ENSG00000143322:ENSG00000159176:ENSG00000182004:ENSG00000069275:ENSG00000143801:ENSG00000163050:ENSG00000116962:ENSG00000162714:ENSG00000015171:ENSG00000107929:ENSG00000198879:ENSG00000241058:ENSG00000165997:ENSG00000099246:ENSG00000108094:ENSG00000188234:ENSG00000165476:ENSG00000138134:ENSG00000107796:ENSG00000059573:ENSG00000165832:ENSG00000198873:ENSG00000184545:ENSG00000166311:ENSG00000166313:ENSG00000205339:ENSG00000166483:ENSG00000198730:ENSG00000149150:ENSG00000162174:ENSG00000213465:ENSG00000110025:ENSG00000168061:ENSG00000168060:ENSG00000162300:ENSG00000149809:ENSG00000174276:ENSG00000162298:ENSG00000149792:ENSG00000173653:ENSG00000173599:ENSG00000173621:ENSG00000175564:ENSG00000137492:ENSG00000149313:ENSG00000137714:ENSG00000149292:ENSG00000180425:ENSG00000076053:ENS

	G00000076043:ENSG00000023171:ENSG00000082805:ENSG00000010219:ENSG00000139180:ENSG00000111667:ENSG00000010626:ENSG00000111674:ENSG00000111676:ENSG00000111678:ENSG00000126749:ENSG00000182326:ENSG00000134531:ENSG00000185432:ENSG00000110911:ENSG00000050426:ENSG00000110925:ENSG00000135457:ENSG00000123360:ENSG00000235162:ENSG00000139410:ENSG00000174989:ENSG00000111725:ENSG00000188735:ENSG00000150990:ENSG00000184992:ENSG00000177084:ENSG00000176915:ENSG00000122033:ENSG00000120688:ENSG00000102531:ENSG00000123179:ENSG00000102753:ENSG00000136104:ENSG00000188243:ENSG00000118939:ENSG00000178695:ENSG00000088451:ENSG00000065150:ENSG00000100889:ENSG00000139914:ENSG00000092140:ENSG00000092108:ENSG00000129493:ENSG00000129480:ENSG00000179454:ENSG00000185246:ENSG00000100442:ENSG00000087301:ENSG00000119599:ENSG00000165861:ENSG00000119707:ENSG00000100767:ENSG00000165417:ENSG00000165934:ENSG00000165943:ENSG00000153485:ENSG00000170270:ENSG00000012963:ENSG00000128731:ENSG00000021776:ENSG00000198146:ENSG00000174197:ENSG00000137802:ENSG00000243708:ENSG00000243789:ENSG00000197535:ENSG00000128989:ENSG00000181827:ENSG00000140262:ENSG00000103707:ENSG00000117899:ENSG00000140406:ENSG00000140511:ENSG00000185418:ENSG00000008710:ENSG00000103512:ENSG00000183426:ENSG00000179889:ENSG00000085721:ENSG00000157106:ENSG00000180035:ENSG00000179958:ENSG00000140691:ENSG00000140682:ENSG00000102910:ENSG00000196470:ENSG00000005194:ENSG00000088682:ENSG00000103066:ENSG00000103064:ENSG00000103061:ENSG00000132600:ENSG00000103056:ENSG00000103018:ENSG00000167515:ENSG00000167522:ENSG00000197912:ENSG00000127804:ENSG00000196689:ENSG00000197417:ENSG00000029725:ENSG00000005100:ENSG00000141127:ENSG00000188522:ENSG00000166484:ENSG00000166482:ENSG00000067191:ENSG00000108828:ENSG00000012048:ENSG00000141279:ENSG00000008294:ENSG00000011258:ENSG00000011260:ENSG00000214226:ENSG00000153933:ENSG00000108423:ENSG00000108443:ENSG00000189050:ENSG00000198265:ENSG00000197170:ENSG00000141540:ENSG00000141576:ENSG00000078687:ENSG00000141577:ENSG00000224877:ENSG00000185504:ENSG00000182446:ENSG00000134265:ENSG00000081913:ENSG00000071564:ENSG00000167670:ENSG00000130254:ENSG00000104833:ENSG00000171105:ENSG00000167775:ENSG00000079999:ENSG00000129354:ENSG00000132024:ENSG00000123159:ENSG00000104835:ENSG00000128626:ENSG00000167625:ENSG00000079462:ENSG00000105429:ENSG00000105427:ENSG00000079435:ENSG00000187244:ENSG00000185453:ENSG00000105447:ENSG00000063127:ENSG00000196214:ENSG00000198464:ENSG00000167766:ENSG00000213020:ENSG00000115761:ENSG00000055917:ENSG00000171103:ENSG00000119866:ENSG00000162928:ENSG00000082898:ENSG00000075292:ENSG00000144034:ENSG00000144048:ENSG00000163170:ENSG00000065911:ENSG00000204843:ENSG00000005448:ENSG00000115602:ENSG00000153107:ENSG00000153208:ENSG00000153214:ENSG00000152102:ENSG00000173272:ENSG00000144283:ENSG00000118260:ENSG00000197713:ENSG00000115457:ENSG00000115461:ENSG00000168958:ENSG00000198612:ENSG00000144485:ENSG00000132326:ENSG00000088876:ENSG00000089063:ENSG00000132646:ENSG00000125868:ENSG00000198959:ENSG00000124228:ENSG00000125520:ENSG00000198276:ENSG00000155313:ENSG00000154642:ENSG00000160190:ENSG00000160284:ENSG00000160285:ENSG00000100220:ENSG00000100342:ENSG00000100321:ENSG00000100324:ENSG00000177096:ENSG00000183172:ENSG00000184983:ENSG00000100197:ENSG00000205643:ENSG00000100416:ENSG00000196576:ENSG00000205593:ENSG00000025770:ENSG00000070950:ENSG00000134077:ENSG00000144560:ENSG00000163517:ENSG00000170293:ENSG00000182606:ENSG00000214706:ENSG00000243477:ENSG00000068001:ENSG00000114383:ENSG00000004838:ENSG00000126062:ENSG00000007402:ENSG00000180376:ENSG00000163946:ENSG00000144741:ENSG00000144749:ENSG00000114861:ENSG00000144736:ENSG00000163320:ENSG00000175105:ENSG00000179021:ENSG00000184500:ENSG00000114446:ENSG00000163607:ENSG00000163608:ENSG00000144843:ENSG00000065518:ENSG00000017260:ENSG00000034533:ENSG00000240303:ENSG00000113971:ENSG00000081307:ENSG00000144867:ENSG00000154917:ENSG00000138231:ENSG00000114098:ENSG00000175110:ENSG00000175066:ENSG00000161202:ENSG00000161204:ENSG00000073803:ENSG00000163904:ENSG00000041802:ENSG00000109689:ENSG00000169019:ENSG00000145246:ENSG00000109171:ENSG00000151247:ENSG00000123739:ENSG00000129187:ENSG00000113360:ENSG00000082213:ENSG00000164190:ENSG00000067248:ENSG00000039123:ENSG00000067113:ENSG00000068796:ENSG00000086189:ENSG00000086200:ENSG00000164331:ENSG00000168916:ENSG00000173926:ENSG00000131507:ENSG00000113721:ENSG00000113272:ENSG00000113312:ENSG00000113742:ENSG00000048140:ENSG00000196670:ENSG00000124535:ENSG00000137266:ENSG00000078401:ENSG00000137337:ENSG00000204344:ENSG00000223501:ENSG00000227057:ENSG00000204220:ENSG00000124762:ENSG00000137168:ENSG00000198663:ENSG00000164530:ENSG00000244067:ENSG00000243955:ENSG00000112245:ENSG00000112357:ENSG00000146416:ENSG00000120278:ENSG00000131018:ENSG00000272047:ENSG00000213066:ENSG00000130023:ENSG00000122644:ENSG00000122545:ENSG00000168303:ENSG00000152926:ENSG00000146757:ENSG00000170027:ENSG00000085563:ENSG00000127920:ENSG00000105829:ENSG00000078487:ENSG00000146834:ENSG00000005249:ENSG00000075790:ENSG00000196937:ENSG00000091704:ENSG00000158623:ENSG00000131558:ENSG00000122786:ENSG00000182158:ENSG00000146918:ENSG00000117868:ENSG00000120910:ENSG00000129691:ENSG00000023287:ENSG00000035681:ENSG00000147601:ENSG00000121039:ENSG00000120533:ENSG00000180900:ENSG00000179950:ENSG00000185189:ENSG00000196378:ENSG00000080608:ENSG00000086065:ENSG00000135018:ENSG00000213694:ENSG00000127080:ENSG00000130958:ENSG00000136925:ENSG00000136936:ENSG00000136826:ENSG00000119446:ENSG00000167081:ENSG00000196152:ENSG00000148356:ENSG00000160408:ENSG00000167106:ENSG00000148339:ENSG00000148335:ENSG00000148308:ENSG00000170835:ENSG00000130560:ENSG00000148411:ENSG00000188986

DEG.twoside	Adipose_Subcutaneous	3668	217	6.8397853657971e-06	0.000369348409753044	ENSG00000158286:ENSG00000158292:ENSG00000097021:ENSG00000171819:ENSG00000187942:ENSG00000160050:ENSG00000168389:ENSG00000066056:ENSG00000117472:ENSG00000162366:ENSG00000162367:ENSG00000117155:ENSG00000153904:ENSG00000171488:ENSG00000134215:ENSG00000168679:ENSG00000175984:ENSG00000092621:ENSG00000163131:ENSG00000143387:ENSG00000143412:ENSG00000143630:ENSG00000132718:ENSG00000196189:ENSG00000242252:ENSG00000160781:ENSG00000163472:ENSG00000187800:ENSG00000143226:ENSG00000174175:ENSG00000143322:ENSG00000122176:ENSG00000163050:ENSG00000116962:ENSG00000134463:ENSG00000075073:ENSG00000107738:ENSG00000120057:ENSG00000119927:ENSG00000198873:ENSG00000184545:ENSG00000268403:ENSG00000187486:ENSG00000124935:ENSG00000146670:ENSG00000173599:ENSG00000173621:ENSG00000175567:ENSG00000175564:ENSG00000062282:ENSG00000137492:ENSG00000137672:ENSG00000154127:ENSG00000166250:ENSG00000010219:ENSG00000010626:ENSG00000134531:ENSG00000134532:ENSG00000123407:ENSG00000123388:ENSG00000123360:ENSG00000127314:ENSG00000166130:ENSG00000074590:ENSG00000139410:ENSG00000122966:ENSG00000184992:ENSG00000127863:ENSG00000133110:ENSG00000102743:ENSG00000136167:ENSG00000136153:ENSG00000178695:ENSG00000100889:ENSG00000139914:ENSG00000203546:ENSG00000170113:ENSG00000186198:ENSG00000136378:ENSG00000140511:ENSG00000085721:ENSG00000140682:ENSG00000196470:ENSG00000103064:ENSG00000103056:ENSG00000172137:ENSG00000185669:ENSG00000132514:ENSG00000188522:ENSG00000108641:ENSG00000128482:ENSG00000142494:ENSG00000067191:ENSG00000141750:ENSG00000108830:ENSG00000184988:ENSG00000073670:ENSG00000189050:ENSG00000154217:ENSG00000197971:ENSG00000065989:ENSG00000132000:ENSG00000099308:ENSG00000262484:ENSG00000269190:ENSG00000179751:ENSG00000105369:ENSG00000028277:ENSG00000079462:ENSG00000105427:ENSG00000079435:ENSG00000079385:ENSG00000105483:ENSG00000105383:ENSG00000115705:ENSG00000196208:ENSG00000163803:ENSG00000065911:ENSG00000114993:ENSG00000115602:ENSG00000153208:ENSG00000163040:ENSG00000115963:ENSG00000153250:ENSG00000018280:ENSG00000144485:ENSG00000162804:ENSG00000124102:ENSG00000203896:ENSG00000155313:ENSG00000157617:ENSG00000184792:ENSG00000100342:ENSG00000100321:ENSG00000177096:ENSG00000100197:ENSG00000138944:ENSG00000075234:ENSG00000075275:ENSG00000205593:ENSG00000177989:ENSG00000008735:ENSG00000251322:ENSG00000144712:ENSG00000163517:ENSG00000170293:ENSG00000144648:ENSG00000068001:ENSG00000007402:ENSG00000184500:ENSG00000144843:ENSG00000113971:ENSG00000129048:ENSG00000158186:ENSG00000114115:ENSG00000114698:ENSG00000163762:ENSG00000169282:ENSG00000174899:ENSG00000075420:ENSG00000114279:ENSG00000145014:ENSG00000152953:ENSG00000080493:ENSG00000163739:ENSG00000109321:ENSG00000185477:ENSG00000205403:ENSG00000164128:ENSG00000164129:ENSG00000215218:ENSG00000113361:ENSG00000171522:ENSG00000132357:ENSG00000086200:ENSG00000175471:ENSG00000164620:ENSG00000120318:ENSG00000135074:ENSG00000131446:ENSG00000078401:ENSG00000244731:ENSG00000124772:ENSG00000137168:ENSG00000164530:ENSG00000118432:ENSG00000135525:ENSG00000118503:ENSG00000120278:ENSG00000186340:ENSG00000105855:ENSG00000253293:ENSG00000152926:ENSG00000213462:ENSG00000189143:ENSG00000177679:ENSG00000135218:ENSG00000085563:ENSG00000127920:ENSG00000185955:ENSG00000166924:ENSG00000005249:ENSG00000106484:ENSG00000159763:ENSG00000196329:ENSG00000120910:ENSG00000104267:ENSG00000164949:ENSG00000120526:ENSG00000044459:ENSG00000127080:ENSG00000136826:ENSG00000070159:ENSG00000243444:ENSG00000167106:ENSG00000170835:ENSG00000160339
DEG.twoside	Adipose_Visceral_Omentum	3435	218	3.83340667386445e-08	2.0700396038868e-06	ENSG00000158292:ENSG00000097021:ENSG00000160050:ENSG00000160051:ENSG00000116525:ENSG00000066056:ENSG00000117399:ENSG00000117472:ENSG00000162367:ENSG00000134709:ENSG00000117155:ENSG00000153904:ENSG00000171488:ENSG00000134215:ENSG00000175984:ENSG00000163131:ENSG00000143630:ENSG00000132718:ENSG00000196189:ENSG00000160781:ENSG00000163472:ENSG00000187800:ENSG00000143226:ENSG00000173110:ENSG00000203747:ENSG00000174175:ENSG00000143322:ENSG00000122176:ENSG00000116962:ENSG00000134463:ENSG00000065328:ENSG00000165997:ENSG00000095794:ENSG00000107736:ENSG00000107738:ENSG00000026103:ENSG00000120057:ENSG00000203867:ENSG00000119927:ENSG00000198873:ENSG00000268403:ENSG00000187486:ENSG00000229859:ENSG00000146670:ENSG00000173599:ENSG00000175567:ENSG00000175564:ENSG00000062282:ENSG00000149256:ENSG00000149292:ENSG00000180425:ENSG00000166250:ENSG00000023171:ENSG00000064309:ENSG00000010626:ENSG00000111674:ENSG00000013588:ENSG00000134531:ENSG00000123360:ENSG00000127314:ENSG00000166130:ENSG00000139410:ENSG00000122966:ENSG00000184992:ENSG00000127863:ENSG00000133110:ENSG00000102743:ENSG00000136167:ENSG00000136153:ENSG00000178695:ENSG00000102524:ENSG00000100889:ENSG00000139914:ENSG00000180998:ENSG00000170113:ENSG00000137802:ENSG00000186198:ENSG00000140406:ENSG00000140511:ENSG00000185418:ENSG00000180209:ENSG00000180035:ENSG00000140682:ENSG00000103067:ENSG00000103064:ENSG00000172137:ENSG00000185669:ENSG00000132514:ENSG00000065325:ENSG00000181350:ENSG00000188522:ENSG00000108641:ENSG00000128482:ENSG00000142494:ENSG00000067191:ENSG00000141750:ENSG00000108830:ENSG00000012048:ENSG00000073670:ENSG00000154217:ENSG00000141540:ENSG00000141401:ENSG00000081913:ENSG00000197971:ENSG00000167670:ENSG00000104880:ENSG00000181143:ENSG00000180739:ENSG00000129354:ENSG00000132000:ENSG00000099308:ENSG00000028277:ENSG00000079462:ENSG00000105427:ENSG00000079435:ENSG00000079385:ENSG00000185453:ENSG00000105483:ENSG00000105383:ENSG00000196208:ENSG00000143867:ENSG00000065911:ENSG00000188687:ENSG00000144218:ENSG00000115602:ENSG00000115604:ENSG00000115607:ENSG00000163040:ENSG00000115963:ENSG00000153250:ENSG00000018280:ENSG00000144485:ENSG00000162804:ENSG00000196209:ENSG00000188559:ENSG00000125966:ENSG00000124102:ENSG00000124107:ENSG00000157617:ENSG00000160190:ENSG00000183628:ENSG00000184792:ENSG00000100342:ENSG00000100321:ENSG00000100197:ENSG00000138944:ENSG00000075275:ENSG00000205593:EN

						SG00000177989:ENSG00000008735:ENSG00000251322:ENSG00000144712:ENSG00000163517:ENSG00000170293:ENSG00000144648:ENSG00000068001:ENSG00000007402:ENSG00000184500:ENSG00000144843:ENSG00000114698:ENSG00000163762:ENSG00000174899:ENSG00000075420:ENSG00000114279:ENSG00000145014:ENSG00000163975:ENSG00000174145:ENSG00000169429:ENSG00000124875:ENSG00000163739:ENSG00000109321:ENSG00000205403:ENSG00000164128:ENSG00000164129:ENSG00000215218:ENSG00000171522:ENSG00000132357:ENSG00000016082:ENSG00000175471:ENSG00000173926:ENSG00000064651:ENSG00000164620:ENSG00000120318:ENSG00000155858:ENSG00000078401:ENSG00000112812:ENSG00000244731:ENSG00000124743:ENSG00000118432:ENSG00000135525:ENSG00000118503:ENSG00000120278:ENSG00000186340:ENSG00000184786:ENSG00000253293:ENSG00000005073:ENSG00000241644:ENSG00000152926:ENSG00000213462:ENSG00000135218:ENSG00000127920:ENSG00000185955:ENSG00000005249:ENSG00000106484:ENSG00000146966:ENSG00000196329:ENSG00000154319:ENSG00000147465:ENSG00000121039:ENSG00000104267:ENSG00000164949:ENSG00000120526:ENSG00000080608:ENSG00000070159:ENSG00000182752:ENSG00000167106:ENSG00000148339:ENSG00000160339
DEG.twoside	Adrenal_Gland	5041	290	1.41786935155917e-06	7.65649449841949e-05	ENSG00000158292:ENSG00000097021:ENSG00000065526:ENSG00000158747:ENSG00000187942:ENSG00000127603:ENSG00000117399:ENSG00000162366:ENSG00000198841:ENSG00000162616:ENSG00000117155:ENSG00000197147:ENSG00000117569:ENSG00000162694:ENSG00000134215:ENSG00000116396:ENSG00000168679:ENSG00000085465:ENSG00000092621:ENSG00000143387:ENSG00000143412:ENSG00000143409:ENSG00000143434:ENSG00000163354:ENSG00000132718:ENSG00000116584:ENSG00000196189:ENSG00000160781:ENSG00000198715:ENSG00000203747:ENSG00000174175:ENSG00000143322:ENSG00000057252:ENSG00000163050:ENSG00000162714:ENSG00000134463:ENSG00000148468:ENSG00000095794:ENSG00000107736:ENSG00000107738:ENSG00000138134:ENSG00000026103:ENSG00000119927:ENSG00000165832:ENSG00000184545:ENSG00000110169:ENSG00000166478:ENSG00000166483:ENSG00000165905:ENSG00000162174:ENSG00000168060:ENSG00000146670:ENSG00000149809:ENSG00000174276:ENSG00000173599:ENSG00000175564:ENSG00000062282:ENSG00000137714:ENSG00000154127:ENSG00000166250:ENSG00000023171:ENSG00000111684:ENSG00000013588:ENSG00000134531:ENSG00000123360:ENSG00000120868:ENSG00000235162:ENSG00000074590:ENSG00000135094:ENSG00000139410:ENSG00000184992:ENSG00000177084:ENSG00000127863:ENSG00000139505:ENSG00000133110:ENSG00000102743:ENSG00000102445:ENSG00000123179:ENSG00000102753:ENSG00000118939:ENSG00000136153:ENSG00000178695:ENSG00000092140:ENSG00000100473:ENSG00000203546:ENSG00000150527:ENSG00000179476:ENSG00000139926:ENSG00000087301:ENSG00000197930:ENSG00000006432:ENSG00000100767:ENSG00000170270:ENSG00000254656:ENSG00000156414:ENSG00000170113:ENSG00000243708:ENSG00000243789:ENSG00000186198:ENSG00000103710:ENSG00000140350:ENSG00000103888:ENSG00000140511:ENSG00000185418:ENSG00000008710:ENSG00000157045:ENSG00000157106:ENSG00000180096:ENSG00000102921:ENSG00000072736:ENSG00000103056:ENSG00000172137:ENSG00000167515:ENSG00000205022:ENSG00000181350:ENSG00000188522:ENSG00000072134:ENSG00000128482:ENSG00000142494:ENSG00000067191:ENSG00000068079:ENSG00000008294:ENSG00000011258:ENSG00000153933:ENSG00000108423:ENSG00000154217:ENSG00000141540:ENSG00000141576:ENSG00000185262:ENSG00000078687:ENSG00000141577:ENSG00000017797:ENSG00000141401:ENSG00000168234:ENSG00000101638:ENSG00000081913:ENSG00000197971:ENSG00000071564:ENSG00000205744:ENSG00000104833:ENSG00000263264:ENSG00000104883:ENSG00000065989:ENSG00000132017:ENSG00000099308:ENSG00000237440:ENSG00000104835:ENSG00000269190:ENSG00000028277:ENSG00000167619:ENSG00000105427:ENSG00000079435:ENSG00000079385:ENSG00000185453:ENSG00000063127:ENSG00000176293:ENSG00000143867:ENSG00000163803:ENSG00000162929:ENSG00000065911:ENSG00000114993:ENSG00000144218:ENSG00000115602:ENSG00000115604:ENSG00000153208:ENSG00000163040:ENSG00000144283:ENSG00000153250:ENSG00000118260:ENSG00000186235:ENSG00000132326:ENSG00000101290:ENSG00000188559:ENSG00000125966:ENSG00000124107:ENSG00000203896:ENSG00000155313:ENSG00000157617:ENSG00000160284:ENSG00000183628:ENSG00000184792:ENSG00000177096:ENSG00000100197:ENSG00000025770:ENSG00000177989:ENSG00000205560:ENSG00000251322:ENSG00000144712:ENSG00000163517:ENSG00000170293:ENSG00000182606:ENSG00000182983:ENSG00000243477:ENSG00000114378:ENSG00000068028:ENSG00000144749:ENSG00000114861:ENSG00000175105:ENSG00000184500:ENSG00000169379:ENSG00000129048:ENSG00000081307:ENSG00000154917:ENSG00000158163:ENSG00000158186:ENSG00000175110:ENSG00000114115:ENSG00000155903:ENSG00000169282:ENSG00000073803:ENSG00000114279:ENSG00000145014:ENSG00000109171:ENSG00000163739:ENSG00000109321:ENSG00000173085:ENSG00000205403:ENSG00000164128:ENSG00000164129:ENSG00000215218:ENSG00000132357:ENSG00000152669:ENSG00000068796:ENSG00000181751:ENSG00000197948:ENSG00000135074:ENSG00000113742:ENSG00000074317:ENSG00000048140:ENSG00000196670:ENSG00000137266:ENSG00000124787:ENSG00000078401:ENSG00000112812:ENSG00000137337:ENSG00000204344:ENSG00000244731:ENSG00000137168:ENSG00000164530:ENSG00000244067:ENSG00000243955:ENSG00000198945:ENSG00000112357:ENSG00000112406:ENSG00000120278:ENSG00000131018:ENSG00000026297:ENSG00000186340:ENSG00000122644:ENSG00000105855:ENSG00000005073:ENSG00000106038:ENSG00000153814:ENSG00000241644:ENSG00000152926:ENSG00000213462:ENSG00000049541:ENSG00000177679:ENSG00000135218:ENSG00000085563:ENSG00000078487:ENSG00000166924:ENSG00000005249:ENSG00000075790:ENSG00000106484:ENSG00000158623:ENSG00000146966:ENSG00000159763:ENSG00000146918:ENSG00000168546:ENSG00000120910:ENSG00000147465:ENSG00000121039:ENSG00000104267:ENSG00000164949:ENSG00000131773:ENSG00000104499:ENSG00000160882:ENSG00000179142:ENSG00000177335:ENSG00000180900:ENSG00000196378:ENSG00000130958:ENSG00000136826:ENSG00000070159:ENSG00000167081:ENSG00000148335:ENSG00000160339:ENSG00000130560:ENSG00000198113

DEG.twoside	Artery_Aorta	4241	244	1.21600418730934e-05	0.000656642261147044	ENSG00000116237:ENSG00000158292:ENSG00000171819:ENSG00000162458:ENSG00000065526:ENSG00000187942:ENSG00000157978:ENSG00000160051:ENSG00000160094:ENSG00000127603:ENSG00000168389:ENSG00000117399:ENSG00000117472:ENSG00000162367:ENSG00000186564:ENSG00000162604:ENSG00000162616:ENSG00000153904:ENSG00000197147:ENSG00000162694:ENSG00000134215:ENSG00000168679:ENSG00000175984:ENSG00000143387:ENSG00000143412:ENSG00000143434:ENSG00000163354:ENSG00000143630:ENSG00000160753:ENSG00000132718:ENSG00000116584:ENSG00000196189:ENSG00000160781:ENSG00000163472:ENSG00000187800:ENSG00000143226:ENSG00000203747:ENSG00000122176:ENSG00000163050:ENSG00000162885:ENSG00000198879:ENSG00000134463:ENSG00000148468:ENSG00000165476:ENSG00000075073:ENSG00000107736:ENSG00000138134:ENSG00000165338:ENSG00000059573:ENSG00000120057:ENSG00000203867:ENSG00000198873:ENSG00000184545:ENSG00000268403:ENSG00000166483:ENSG00000187486:ENSG00000162174:ENSG00000168060:ENSG00000146670:ENSG00000162300:ENSG00000149809:ENSG00000173599:ENSG00000175564:ENSG00000062282:ENSG00000154127:ENSG00000166250:ENSG00000064309:ENSG00000082805:ENSG00000010219:ENSG00000013588:ENSG00000134532:ENSG00000123094:ENSG00000087494:ENSG00000123360:ENSG00000139292:ENSG00000166130:ENSG00000111647:ENSG00000235162:ENSG00000074590:ENSG00000127863:ENSG00000139505:ENSG00000133110:ENSG00000102743:ENSG00000136167:ENSG00000102531:ENSG00000136153:ENSG00000134901:ENSG00000165355:ENSG00000179476:ENSG00000087301:ENSG00000100767:ENSG00000165417:ENSG00000156414:ENSG00000261794:ENSG00000186198:ENSG00000103710:ENSG00000234438:ENSG00000136378:ENSG00000140600:ENSG00000157766:ENSG00000140511:ENSG00000008710:ENSG00000103512:ENSG00000180209:ENSG00000140682:ENSG00000103056:ENSG00000172137:ENSG00000185669:ENSG00000196689:ENSG00000108641:ENSG00000141750:ENSG00000108830:ENSG00000012048:ENSG00000184988:ENSG00000073670:ENSG00000154217:ENSG00000078687:ENSG00000141401:ENSG00000168234:ENSG00000141469:ENSG00000081913:ENSG00000197971:ENSG00000167670:ENSG00000205744:ENSG00000171105:ENSG00000104883:ENSG00000065989:ENSG00000132016:ENSG00000132000:ENSG00000123146:ENSG00000099308:ENSG00000269190:ENSG00000105369:ENSG00000028277:ENSG00000079462:ENSG00000105427:ENSG00000079435:ENSG00000167766:ENSG00000143867:ENSG00000116039:ENSG00000065911:ENSG00000114993:ENSG00000153208:ENSG00000153214:ENSG00000153250:ENSG00000231672:ENSG00000018280:ENSG00000162804:ENSG00000125779:ENSG00000101290:ENSG00000125864:ENSG00000188559:ENSG00000124102:ENSG00000124201:ENSG00000157617:ENSG00000160190:ENSG00000183628:ENSG00000100342:ENSG00000100321:ENSG00000198951:ENSG00000177096:ENSG00000100197:ENSG00000138944:ENSG00000205593:ENSG00000205560:ENSG00000251322:ENSG00000144560:ENSG00000170293:ENSG00000170011:ENSG00000179564:ENSG00000114378:ENSG00000144749:ENSG00000114861:ENSG00000184500:ENSG00000144843:ENSG00000172780:ENSG00000240303:ENSG00000113971:ENSG00000129048:ENSG00000158163:ENSG00000175066:ENSG00000114698:ENSG00000163762:ENSG00000169282:ENSG00000075420:ENSG00000163898:ENSG00000163975:ENSG00000013288:ENSG00000169019:ENSG00000145246:ENSG00000109171:ENSG00000145248:ENSG00000080493:ENSG00000163739:ENSG00000164128:ENSG00000164129:ENSG00000175471:ENSG00000181751:ENSG00000064651:ENSG00000240184:ENSG00000164620:ENSG00000197948:ENSG00000164385:ENSG00000137266:ENSG00000079691:ENSG00000146112:ENSG00000137404:ENSG00000235863:ENSG00000124772:ENSG00000164530:ENSG00000243955:ENSG00000198945:ENSG00000135525:ENSG00000112406:ENSG00000146416:ENSG00000131018:ENSG00000186340:ENSG00000122644:ENSG00000105855:ENSG00000153814:ENSG00000241644:ENSG00000135218:ENSG00000085563:ENSG00000185955:ENSG00000005249:ENSG00000075790:ENSG00000106484:ENSG00000182158:ENSG00000146966:ENSG00000181444:ENSG00000147465:ENSG00000147601:ENSG00000104267:ENSG00000164949:ENSG00000129295:ENSG00000178031:ENSG00000186354:ENSG00000213694:ENSG00000127080:ENSG00000136936:ENSG00000243444:ENSG00000136840:ENSG00000148411:ENSG00000198113
DEG.twoside	Artery_Coronary	3006	165	0.00284281481661144	0.153512000097018	ENSG00000171819:ENSG00000162458:ENSG00000187942:ENSG00000160094:ENSG00000127603:ENSG00000168389:ENSG00000117399:ENSG00000117472:ENSG00000162604:ENSG00000162616:ENSG00000134215:ENSG00000168679:ENSG00000175984:ENSG00000163131:ENSG00000143412:ENSG00000143630:ENSG00000160753:ENSG00000132718:ENSG00000196189:ENSG00000160781:ENSG00000163472:ENSG00000187800:ENSG00000143226:ENSG00000203747:ENSG00000174175:ENSG00000122176:ENSG00000162885:ENSG00000165476:ENSG00000075073:ENSG00000026103:ENSG00000165338:ENSG00000188649:ENSG00000203867:ENSG00000119927:ENSG00000198873:ENSG00000187486:ENSG00000162174:ENSG00000168060:ENSG00000146670:ENSG00000149809:ENSG00000175564:ENSG00000166130:ENSG00000235162:ENSG00000074590:ENSG00000122966:ENSG00000184992:ENSG00000133110:ENSG00000136167:ENSG00000134901:ENSG00000102524:ENSG00000139914:ENSG00000168952:ENSG00000100473:ENSG00000179476:ENSG00000186198:ENSG00000103710:ENSG00000136378:ENSG00000103888:ENSG00000157766:ENSG00000140511:ENSG00000140682:ENSG00000103056:ENSG00000132514:ENSG00000181350:ENSG00000166484:ENSG00000128482:ENSG00000108830:ENSG00000184988:ENSG00000073670:ENSG00000123700:ENSG00000141576:ENSG00000078687:ENSG00000141401:ENSG00000168234:ENSG00000141469:ENSG00000081913:ENSG00000197971:ENSG00000167670:ENSG00000104833:ENSG00000132016:ENSG00000132000:ENSG00000123146:ENSG00000079462:ENSG00000105427:ENSG00000079435:ENSG00000079385:ENSG00000063127:ENSG00000143867:ENSG00000065911:ENSG00000153208:ENSG00000153250:ENSG00000231672:ENSG00000018280:ENSG00000186235:ENSG00000101290:ENSG00000124102:ENSG00000124201:ENSG00000157617:ENSG00000183628:ENSG00000100342:ENSG00000100321:ENSG00000198951:ENSG00000177096:ENSG00000100197:ENSG00000138944:ENSG00000075275:ENSG00000205593:ENSG00000205560:ENSG00000144560:ENSG00000168032:ENSG00000007402:ENSG00000114861:ENSG00000184500:ENSG00000169379:ENSG00000114446:ENSG00000144843:ENSG00000158186:ENSG00000114698:ENSG00000169282:ENSG00000075420:ENSG00000163898:ENSG00000114279:ENSG00000013288:ENSG00

						000145246:ENSG00000109171:ENSG00000080493:ENSG00000205403:ENSG00000215218:ENSG00000113361:ENSG00000086189:ENSG00000181751:ENSG00000064651:ENSG00000253159:ENSG00000164620:ENSG00000120318:ENSG00000164385:ENSG00000137266:ENSG00000079691:ENSG00000146112:ENSG00000124772:ENSG00000164530:ENSG00000132429:ENSG00000198945:ENSG00000135525:ENSG00000186340:ENSG00000241644:ENSG00000152926:ENSG00000177679:ENSG00000135218:ENSG00000085563:ENSG00000127920:ENSG00000185955:ENSG00000166924:ENSG00000075790:ENSG00000182158:ENSG00000168546:ENSG00000120910:ENSG00000147601:ENSG00000104267:ENSG00000164949:ENSG00000186354:ENSG00000213694:ENSG00000127080:ENSG00000136840:ENSG00000148411
DEG.twoside	Artery_Tibial	4761	279	5.12711547545876e-07	2.76864235674773e-05	ENSG00000158286:ENSG00000116237:ENSG00000158292:ENSG00000171819:ENSG00000162458:ENSG00000065526:ENSG00000187942:ENSG00000134668:ENSG00000160050:ENSG00000160051:ENSG00000160094:ENSG00000127603:ENSG00000117472:ENSG00000162366:ENSG00000162367:ENSG00000186564:ENSG00000162604:ENSG00000162616:ENSG00000117155:ENSG00000197147:ENSG00000117569:ENSG00000168679:ENSG00000163349:ENSG00000197323:ENSG00000175984:ENSG00000092621:ENSG00000143437:ENSG00000143412:ENSG00000143434:ENSG00000163154:ENSG00000163157:ENSG00000163354:ENSG00000143630:ENSG00000160753:ENSG00000116539:ENSG00000132718:ENSG00000116584:ENSG00000196189:ENSG00000160781:ENSG00000163472:ENSG00000187800:ENSG00000173110:ENSG00000174175:ENSG00000122176:ENSG00000162885:ENSG00000116962:ENSG00000198879:ENSG00000134463:ENSG00000148468:ENSG00000241058:ENSG00000165476:ENSG00000107736:ENSG00000138134:ENSG00000152779:ENSG00000165338:ENSG00000188649:ENSG00000120057:ENSG00000203867:ENSG00000119927:ENSG00000198873:ENSG00000184545:ENSG00000166478:ENSG00000187486:ENSG00000162174:ENSG00000110025:ENSG00000168060:ENSG00000149809:ENSG00000173599:ENSG00000175567:ENSG00000175564:ENSG00000062282:ENSG00000137672:ENSG00000076053:ENSG00000154127:ENSG00000166250:ENSG00000064309:ENSG00000082805:ENSG00000111679:ENSG00000013588:ENSG00000134532:ENSG00000123094:ENSG00000123407:ENSG00000123388:ENSG00000123338:ENSG00000123360:ENSG00000127314:ENSG00000111647:ENSG00000235162:ENSG00000135094:ENSG00000139410:ENSG00000188735:ENSG00000184992:ENSG00000127863:ENSG00000139505:ENSG00000133110:ENSG00000102743:ENSG00000120688:ENSG00000102445:ENSG00000102531:ENSG00000168952:ENSG00000129493:ENSG00000179476:ENSG00000087301:ENSG00000165861:ENSG00000100767:ENSG00000165417:ENSG00000243789:ENSG00000140262:ENSG00000103707:ENSG00000186198:ENSG00000103710:ENSG00000234438:ENSG00000136378:ENSG00000157766:ENSG00000140511:ENSG00000008710:ENSG00000102879:ENSG00000180096:ENSG00000140682:ENSG00000103056:ENSG00000127804:ENSG00000181350:ENSG00000108641:ENSG00000166484:ENSG00000067191:ENSG00000141750:ENSG00000108830:ENSG00000012048:ENSG00000073670:ENSG00000121060:ENSG00000154217:ENSG00000180616:ENSG00000141540:ENSG00000078687:ENSG00000017797:ENSG00000141401:ENSG00000168234:ENSG00000141469:ENSG00000197971:ENSG00000205744:ENSG00000132000:ENSG00000123146:ENSG00000099308:ENSG00000104835:ENSG00000269190:ENSG00000179751:ENSG00000028277:ENSG00000079462:ENSG00000105427:ENSG00000079435:ENSG00000063127:ENSG00000115756:ENSG00000197872:ENSG00000143867:ENSG00000116039:ENSG00000065911:ENSG00000005448:ENSG00000114993:ENSG00000115602:ENSG00000115604:ENSG00000153208:ENSG00000153214:ENSG00000163040:ENSG00000115963:ENSG00000153250:ENSG00000118260:ENSG00000231672:ENSG00000135912:ENSG00000132326:ENSG00000196209:ENSG00000088876:ENSG00000101290:ENSG00000089048:ENSG00000125864:ENSG00000188559:ENSG00000124102:ENSG00000124201:ENSG00000203896:ENSG00000157617:ENSG00000160190:ENSG00000100321:ENSG00000177096:ENSG00000138944:ENSG00000186951:ENSG00000205593:ENSG00000130489:ENSG00000177989:ENSG00000205560:ENSG00000144560:ENSG00000144712:ENSG00000170293:ENSG00000183873:ENSG00000170011:ENSG00000177842:ENSG00000114378:ENSG00000114395:ENSG00000007402:ENSG00000144749:ENSG00000114861:ENSG00000184500:ENSG00000169379:ENSG00000113971:ENSG00000158163:ENSG00000114098:ENSG00000114115:ENSG00000114698:ENSG00000163762:ENSG00000169282:ENSG00000075420:ENSG00000163975:ENSG00000013288:ENSG00000145246:ENSG00000109171:ENSG00000163739:ENSG00000185477:ENSG00000164128:ENSG00000164129:ENSG00000113361:ENSG00000171522:ENSG00000067248:ENSG00000039123:ENSG00000086189:ENSG00000181751:ENSG00000168916:ENSG00000064651:ENSG00000164620:ENSG00000048140:ENSG00000164385:ENSG00000137266:ENSG00000079691:ENSG00000146112:ENSG00000124772:ENSG00000118432:ENSG00000132429:ENSG00000135525:ENSG00000146416:ENSG00000120278:ENSG00000131018:ENSG00000272047:ENSG00000026297:ENSG00000186340:ENSG00000122644:ENSG00000105855:ENSG00000253293:ENSG00000005073:ENSG00000153814:ENSG00000241644:ENSG00000146757:ENSG00000086730:ENSG00000177679:ENSG00000085563:ENSG00000005249:ENSG00000075790:ENSG00000106484:ENSG00000182158:ENSG00000146966:ENSG00000181444:ENSG00000168546:ENSG00000120910:ENSG00000254087:ENSG00000147601:ENSG00000121039:ENSG00000104267:ENSG00000164949:ENSG00000120533:ENSG00000129295:ENSG00000165071:ENSG00000131773:ENSG00000196378:ENSG00000044459:ENSG00000186354:ENSG00000213694:ENSG00000127080:ENSG00000136925:ENSG00000136936:ENSG00000243444:ENSG00000170835:ENSG00000148411:ENSG00000198113
DEG.twoside	Bladder	948	68	8.61559337449297e-05	0.00465242042222621	ENSG00000158286:ENSG00000162458:ENSG00000187942:ENSG00000116455:ENSG00000160767:ENSG00000122176:ENSG00000165338:ENSG00000166483:ENSG00000149150:ENSG00000173599:ENSG00000166250:ENSG00000082805:ENSG00000013588:ENSG00000123338:ENSG00000122966:ENSG00000139914:ENSG00000165379:ENSG00000179476:ENSG00000139926:ENSG00000140262:ENSG00000103710:ENSG00000140682:ENSG00000132514:ENSG00000065325:ENSG00000142494:ENSG00000141750:ENSG00000182963:ENSG00000141401:ENSG00000123146:ENSG00000269190:ENSG00000105492:ENSG00000198464:ENSG00000143867:ENSG00000144218:ENSG00000197713:ENSG00000124107:ENSG00000100342:ENSG00000177096:ENSG00000138944:ENSG00000197385:ENSG00000182983:EN

						SG00000007402:ENSG00000114861:ENSG00000154917:ENSG00000158163:ENSG00000114115:ENSG00000109171:ENSG00000181751:ENSG00000120318:ENSG00000135074:ENSG00000113272:ENSG00000155858:ENSG00000164530:ENSG00000105855:ENSG00000253293:ENSG00000005073:ENSG00000106031:ENSG00000169902:ENSG00000127920:ENSG00000166924:ENSG00000005249:ENSG00000147601:ENSG00000121039:ENSG00000104267:ENSG00000164949:ENSG00000213694:ENSG00000167081:ENSG00000196152
DEG.twoside	Brain_Amygdala	9576	608	7.18934404566483e-26	3.88224578465901e-24	ENSG00000158286:ENSG00000116237:ENSG00000097021:ENSG00000171824:ENSG00000198793:ENSG00000120942:ENSG00000162458:ENSG00000065526:ENSG00000116809:ENSG00000183888:ENSG00000117616:ENSG00000204178:ENSG00000157978:ENSG00000084652:ENSG00000160051:ENSG00000160094:ENSG00000134686:ENSG00000121904:ENSG00000177181:ENSG00000066056:ENSG00000159479:ENSG00000198198:ENSG00000162367:ENSG00000117862:ENSG00000198841:ENSG00000162390:ENSG00000134709:ENSG00000162604:ENSG00000162616:ENSG00000117114:ENSG00000117133:ENSG00000142867:ENSG00000153904:ENSG00000171488:ENSG00000117528:ENSG00000162694:ENSG00000117543:ENSG00000118733:ENSG00000134215:ENSG00000162775:ENSG00000168679:ENSG00000085465:ENSG00000116455:ENSG00000163349:ENSG00000197323:ENSG00000213281:ENSG00000052723:ENSG00000092621:ENSG00000143457:ENSG00000163131:ENSG00000143387:ENSG00000143437:ENSG00000143379:ENSG00000143418:ENSG00000143412:ENSG00000143409:ENSG00000143363:ENSG00000143434:ENSG00000163156:ENSG00000163155:ENSG00000143537:ENSG00000160767:ENSG00000116521:ENSG00000176444:ENSG00000143630:ENSG00000160752:ENSG00000160753:ENSG00000116539:ENSG00000125459:ENSG00000163374:ENSG00000132676:ENSG00000116580:ENSG00000132718:ENSG00000143622:ENSG00000132680:ENSG00000116584:ENSG00000160789:ENSG00000160783:ENSG00000160781:ENSG00000163472:ENSG00000198715:ENSG00000187800:ENSG00000132694:ENSG00000143226:ENSG00000120341:ENSG00000186283:ENSG00000143322:ENSG00000057252:ENSG00000134376:ENSG00000182004:ENSG00000143801:ENSG00000163050:ENSG00000162885:ENSG00000116962:ENSG00000162714:ENSG00000107929:ENSG00000134463:ENSG00000148468:ENSG00000165997:ENSG00000108094:ENSG00000188234:ENSG00000165476:ENSG00000107736:ENSG00000214688:ENSG00000107738:ENSG00000148604:ENSG00000138134:ENSG00000107796:ENSG00000026103:ENSG00000165338:ENSG00000059573:ENSG00000197430:ENSG00000155256:ENSG00000198873:ENSG00000184545:ENSG00000166311:ENSG00000205339:ENSG00000166478:ENSG00000166483:ENSG00000198730:ENSG00000129151:ENSG00000019505:ENSG00000149150:ENSG00000162174:ENSG00000162300:ENSG00000149823:ENSG00000174276:ENSG00000162298:ENSG00000149792:ENSG00000173653:ENSG00000175567:ENSG00000062282:ENSG00000137492:ENSG00000137693:ENSG00000137714:ENSG00000183644:ENSG00000149292:ENSG00000076053:ENSG00000076043:ENSG00000183715:ENSG00000082805:ENSG00000010219:ENSG00000139180:ENSG00000111671:ENSG00000111679:ENSG00000126749:ENSG00000111684:ENSG00000182326:ENSG00000134531:ENSG00000123094:ENSG00000110911:ENSG00000050426:ENSG00000123338:ENSG00000127314:ENSG00000166130:ENSG00000235162:ENSG00000074590:ENSG00000135094:ENSG00000174989:ENSG00000111725:ENSG00000122966:ENSG00000188735:ENSG00000150990:ENSG00000177169:ENSG00000187848:ENSG00000177084:ENSG00000176915:ENSG00000127863:ENSG00000139505:ENSG00000102743:ENSG00000136167:ENSG00000102445:ENSG00000102531:ENSG00000123179:ENSG00000102753:ENSG00000136104:ENSG00000184226:ENSG00000188243:ENSG00000136153:ENSG00000178695:ENSG00000088451:ENSG00000065150:ENSG00000134901:ENSG00000134897:ENSG00000139826:ENSG00000102524:ENSG00000100889:ENSG00000168952:ENSG00000176165:ENSG00000186960:ENSG00000092140:ENSG00000092108:ENSG00000100473:ENSG00000150527:ENSG00000165355:ENSG00000165379:ENSG00000179476:ENSG00000179454:ENSG00000185246:ENSG00000139926:ENSG00000180998:ENSG00000197930:ENSG00000119599:ENSG00000165861:ENSG00000119707:ENSG00000100767:ENSG00000133961:ENSG00000165417:ENSG00000100433:ENSG00000165934:ENSG00000153485:ENSG00000140157:ENSG00000170113:ENSG00000128731:ENSG00000169918:ENSG00000021776:ENSG00000198146:ENSG00000174197:ENSG00000137802:ENSG00000243708:ENSG00000243789:ENSG00000197535:ENSG00000181827:ENSG00000140262:ENSG00000103707:ENSG00000186198:ENSG00000136378:ENSG00000103888:ENSG00000117899:ENSG00000140406:ENSG00000140600:ENSG00000140511:ENSG00000140471:ENSG00000008710:ENSG00000183454:ENSG00000103512:ENSG00000179889:ENSG00000085721:ENSG00000157106:ENSG00000180096:ENSG00000179958:ENSG00000140691:ENSG00000140682:ENSG00000197302:ENSG00000102910:ENSG00000196470:ENSG00000102921:ENSG00000005194:ENSG00000102978:ENSG00000072736:ENSG00000103066:ENSG00000103064:ENSG00000103061:ENSG00000132600:ENSG00000172137:ENSG00000158717:ENSG00000167522:ENSG00000197912:ENSG00000127804:ENSG00000196689:ENSG00000197417:ENSG00000005100:ENSG00000072849:ENSG00000233670:ENSG00000166484:ENSG00000166482:ENSG00000128482:ENSG00000142494:ENSG00000068079:ENSG00000108828:ENSG00000108830:ENSG00000012048:ENSG00000184988:ENSG00000073670:ENSG00000182963:ENSG00000225190:ENSG00000141279:ENSG00000108424:ENSG00000011258:ENSG00000011260:ENSG00000153933:ENSG00000121060:ENSG00000108423:ENSG00000108443:ENSG00000189050:ENSG00000108878:ENSG00000198265:ENSG00000197170:ENSG00000154217:ENSG00000141540:ENSG00000196169:ENSG00000141576:ENSG00000185262:ENSG00000141577:ENSG00000167302:ENSG00000185504:ENSG00000182446:ENSG00000101608:ENSG00000017797:ENSG00000154845:ENSG00000154889:ENSG00000141401:ENSG00000168234:ENSG00000141384:ENSG00000141469:ENSG00000152223:ENSG00000081913:ENSG00000130856:ENSG00000197971:ENSG00000071564:ENSG00000141985:ENSG00000167670:ENSG00000205744:ENSG00000104833:ENSG00000171105:ENSG00000104880:ENSG00000167775:ENSG00000079999:ENSG00000180739:ENSG00000104957:ENSG00000132017:ENSG00000132005:ENSG00000123146:ENSG00000123136:ENSG00000127527:ENSG00000099308:ENSG00000237440:ENSG00000104835:ENSG00000128626:ENSG00000269190:ENSG00000028277:ENSG00000160570:ENSG00000167625:ENSG00000105722:ENSG00000079462:ENSG00000167619:ENSG00000105427:ENSG00

						000187244:ENSG00000185453:ENSG00000105483:ENSG00000105464:ENSG00000105447:ENSG00000142544:ENSG00000105383:ENSG00000196214:ENSG00000198464:ENSG00000167766:ENSG00000213020:ENSG00000152467:ENSG00000115761:ENSG00000197872:ENSG00000055917:ENSG00000171103:ENSG00000162928:ENSG00000082898:ENSG00000075292:ENSG00000144034:ENSG00000144048:ENSG00000114978:ENSG00000065911:ENSG00000114993:ENSG00000115602:ENSG00000115604:ENSG00000153107:ENSG00000153208:ENSG00000153214:ENSG00000115963:ENSG00000144283:ENSG00000153250:ENSG00000153253:ENSG00000118260:ENSG00000197713:ENSG00000115457:ENSG00000115461:ENSG00000018280:ENSG00000144579:ENSG00000135912:ENSG00000144485:ENSG00000132326:ENSG00000162804:ENSG00000196209:ENSG00000088876:ENSG00000125779:ENSG00000132646:ENSG00000188559:ENSG00000198959:ENSG00000124107:ENSG00000124228:ENSG00000124201:ENSG00000125520:ENSG00000198276:ENSG00000155313:ENSG00000157617:ENSG00000160190:ENSG00000100220:ENSG00000100342:ENSG00000100321:ENSG00000128285:ENSG00000198951:ENSG00000177096:ENSG00000100197:ENSG00000242247:ENSG00000138944:ENSG00000077942:ENSG00000186951:ENSG00000205643:ENSG00000075234:ENSG00000100416:ENSG00000196576:ENSG00000100258:ENSG00000025770:ENSG00000130489:ENSG00000025708:ENSG00000177989:ENSG00000205560:ENSG00000100288:ENSG00000008735:ENSG00000100299:ENSG00000134077:ENSG00000144560:ENSG00000144712:ENSG00000163517:ENSG00000163513:ENSG00000170293:ENSG00000170011:ENSG00000177873:ENSG00000182606:ENSG00000182983:ENSG00000214706:ENSG00000243477:ENSG00000114378:ENSG00000068001:ENSG00000068028:ENSG00000114388:ENSG00000114395:ENSG00000126062:ENSG00000007402:ENSG00000180376:ENSG00000163946:ENSG00000114861:ENSG00000144736:ENSG00000163320:ENSG00000175105:ENSG00000179021:ENSG00000184500:ENSG00000169379:ENSG00000114446:ENSG00000163607:ENSG00000163608:ENSG00000144843:ENSG00000082701:ENSG00000172780:ENSG00000240682:ENSG00000034533:ENSG00000240303:ENSG00000113971:ENSG00000081307:ENSG00000144867:ENSG00000154917:ENSG00000138231:ENSG00000114098:ENSG00000175110:ENSG00000184432:ENSG00000155903:ENSG00000175066:ENSG00000114127:ENSG00000075420:ENSG00000161202:ENSG00000161204:ENSG00000114279:ENSG00000145014:ENSG00000041802:ENSG00000163975:ENSG00000013288:ENSG00000109689:ENSG00000169019:ENSG00000145246:ENSG00000109171:ENSG00000080493:ENSG00000169429:ENSG00000163739:ENSG00000173085:ENSG00000145331:ENSG00000205403:ENSG00000164128:ENSG00000109466:ENSG00000129187:ENSG00000215218:ENSG00000113100:ENSG00000113360:ENSG00000164190:ENSG00000171522:ENSG00000067248:ENSG00000039123:ENSG00000086200:ENSG00000164331:ENSG00000164338:ENSG00000164180:ENSG00000173926:ENSG00000145794:ENSG00000240184:ENSG00000240764:ENSG00000131504:ENSG00000171720:ENSG00000197948:ENSG00000120318:ENSG00000113721:ENSG00000135074:ENSG00000113272:ENSG00000182132:ENSG00000074317:ENSG00000175766:ENSG00000048140:ENSG00000249109:ENSG00000131446:ENSG00000196670:ENSG00000146112:ENSG00000137404:ENSG00000137337:ENSG00000137331:ENSG00000204344:ENSG00000223501:ENSG00000235863:ENSG00000227057:ENSG00000237441:ENSG00000231925:ENSG00000124762:ENSG00000124772:ENSG00000198663:ENSG00000164530:ENSG00000112245:ENSG00000172461:ENSG00000198945:ENSG00000135525:ENSG00000112357:ENSG00000118503:ENSG00000112406:ENSG00000120278:ENSG00000131018:ENSG00000112096:ENSG00000213066:ENSG00000186340:ENSG00000184786:ENSG00000130023:ENSG00000122644:ENSG00000105855:ENSG00000153814:ENSG00000164600:ENSG00000168303:ENSG00000152926:ENSG00000213462:ENSG00000126522:ENSG00000049541:ENSG00000135218:ENSG00000127920:ENSG00000105829:ENSG00000121716:ENSG00000085514:ENSG00000146834:ENSG00000160813:ENSG00000166924:ENSG00000172209:ENSG00000075790:ENSG00000091704:ENSG00000106484:ENSG00000158623:ENSG00000131558:ENSG00000122786:ENSG00000182158:ENSG00000181444:ENSG00000196329:ENSG00000214106:ENSG00000146918:ENSG00000117868:ENSG00000154319:ENSG00000168546:ENSG00000120910:ENSG00000129691:ENSG00000254087:ENSG00000035681:ENSG00000147601:ENSG00000121039:ENSG00000104267:ENSG00000104327:ENSG00000164949:ENSG00000120526:ENSG00000120533:ENSG00000196378:ENSG00000080608:ENSG00000213694:ENSG00000165023:ENSG00000127080:ENSG00000130958:ENSG00000136925:ENSG00000136936:ENSG00000136826:ENSG00000070159:ENSG00000167081:ENSG00000196152:ENSG00000148356:ENSG00000136840:ENSG00000171169:ENSG00000148339:ENSG00000148335:ENSG00000148308:ENSG00000170835:ENSG00000148411:ENSG00000198113
DEG.twoside	Brain_Anterior_cingulate_cortex_BA24	9101	560	8.27398177188973e-20	4.46795015682045e-18	ENSG00000158286:ENSG00000116237:ENSG00000097021:ENSG00000198793:ENSG00000120942:ENSG00000162458:ENSG00000065526:ENSG00000116809:ENSG00000183888:ENSG00000117616:ENSG00000204178:ENSG00000157978:ENSG00000084652:ENSG00000134686:ENSG00000121904:ENSG00000177181:ENSG00000066056:ENSG00000066322:ENSG00000159479:ENSG00000198198:ENSG00000162367:ENSG00000117862:ENSG00000198841:ENSG00000162390:ENSG00000162616:ENSG00000117114:ENSG00000117133:ENSG00000117155:ENSG00000142867:ENSG00000153904:ENSG00000197147:ENSG00000171488:ENSG00000069712:ENSG00000117528:ENSG00000117543:ENSG00000118733:ENSG00000134215:ENSG00000162775:ENSG00000168679:ENSG00000116455:ENSG00000163349:ENSG00000197323:ENSG00000052723:ENSG00000143457:ENSG00000163131:ENSG00000143387:ENSG00000143437:ENSG00000143379:ENSG00000143418:ENSG00000143412:ENSG00000143409:ENSG00000143363:ENSG00000143434:ENSG00000163154:ENSG00000163156:ENSG00000143537:ENSG00000116521:ENSG00000176444:ENSG00000125459:ENSG00000163374:ENSG00000132676:ENSG00000116580:ENSG00000132718:ENSG00000143622:ENSG00000132680:ENSG00000160789:ENSG00000160783:ENSG00000160781:ENSG00000163472:ENSG00000198715:ENSG00000187800:ENSG00000143226:ENSG00000120341:ENSG00000186283:ENSG00000143322:ENSG00000057252:ENSG00000134376:ENSG00000182004:ENSG00000163050:ENSG00000162885:ENSG00000116962:ENSG00000162714:ENSG00000107929:ENSG00000134463:ENSG00000148468:ENSG00000165997:ENSG00000165476:ENSG00000075073:ENSG00000107736:ENSG00000214688:ENSG00000107738:ENSG00000148604:ENSG00000107796:ENSG00000026103:ENSG00000059573:ENSG00000197430:ENSG00000198873:ENSG00000184545:ENSG00000166

	311:ENSG00000166478:ENSG00000198730:ENSG00000006071:ENSG00000129151:ENSG00000019505:ENSG00000149150:ENSG00000162174:ENSG00000162300:ENSG00000174276:ENSG00000162298:ENSG00000149792:ENSG00000173653:ENSG00000173599:ENSG00000175567:ENSG00000062282:ENSG00000137492:ENSG00000137672:ENSG00000137693:ENSG00000149313:ENSG00000137714:ENSG00000149292:ENSG00000076053:ENSG00000076043:ENSG00000166250:ENSG00000023171:ENSG00000109832:ENSG00000183715:ENSG00000082805:ENSG00000010219:ENSG00000111262:ENSG00000111671:ENSG00000111679:ENSG00000126749:ENSG00000111684:ENSG00000182326:ENSG00000134531:ENSG00000123094:ENSG00000087494:ENSG00000110911:ENSG00000123338:ENSG00000123360:ENSG00000127314:ENSG00000139292:ENSG00000166130:ENSG00000235162:ENSG00000135094:ENSG00000174989:ENSG00000111725:ENSG00000122966:ENSG00000188735:ENSG00000150990:ENSG00000184992:ENSG00000177084:ENSG00000176915:ENSG00000127863:ENSG00000102743:ENSG00000215475:ENSG00000136167:ENSG00000102445:ENSG00000102531:ENSG00000123179:ENSG00000184226:ENSG00000188243:ENSG00000118939:ENSG00000136153:ENSG00000178695:ENSG00000088451:ENSG00000134901:ENSG00000139826:ENSG00000102524:ENSG00000100889:ENSG00000168952:ENSG00000176165:ENSG00000186960:ENSG00000092140:ENSG00000092108:ENSG00000129493:ENSG00000150527:ENSG00000165379:ENSG00000179476:ENSG00000179454:ENSG00000185246:ENSG00000139926:ENSG00000180998:ENSG00000197930:ENSG00000006432:ENSG00000119599:ENSG00000165861:ENSG00000119707:ENSG00000080815:ENSG00000100767:ENSG00000133961:ENSG00000100433:ENSG00000165934:ENSG00000153485:ENSG00000140157:ENSG00000170113:ENSG00000169918:ENSG00000021776:ENSG00000198146:ENSG00000174197:ENSG00000137802:ENSG00000243708:ENSG00000243789:ENSG00000197535:ENSG00000181827:ENSG00000140262:ENSG00000103707:ENSG00000103710:ENSG00000117899:ENSG00000140406:ENSG00000140600:ENSG00000140511:ENSG00000140471:ENSG00000183454:ENSG00000179889:ENSG00000085721:ENSG00000157106:ENSG00000180096:ENSG00000179965:ENSG00000140682:ENSG00000102910:ENSG00000196470:ENSG00000102921:ENSG00000072736:ENSG00000103066:ENSG00000103064:ENSG00000103061:ENSG00000172137:ENSG00000158717:ENSG00000167522:ENSG00000127804:ENSG00000196689:ENSG00000197417:ENSG00000005100:ENSG00000072849:ENSG00000109047:ENSG00000233670:ENSG00000181350:ENSG00000166484:ENSG00000166482:ENSG00000128482:ENSG00000142494:ENSG00000067191:ENSG00000141750:ENSG00000068079:ENSG00000108828:ENSG00000108830:ENSG00000073670:ENSG00000182963:ENSG00000225190:ENSG00000198933:ENSG00000011258:ENSG00000011260:ENSG00000121060:ENSG00000108423:ENSG00000108443:ENSG00000189050:ENSG00000198265:ENSG00000154217:ENSG00000123700:ENSG00000180616:ENSG00000141576:ENSG00000185262:ENSG00000141577:ENSG00000167302:ENSG00000185504:ENSG00000182446:ENSG00000101608:ENSG00000154845:ENSG00000154889:ENSG00000141401:ENSG00000168234:ENSG00000141469:ENSG00000152223:ENSG00000101638:ENSG00000081913:ENSG00000197971:ENSG00000071564:ENSG00000141985:ENSG00000167670:ENSG00000205744:ENSG00000104833:ENSG00000171105:ENSG00000104880:ENSG00000104883:ENSG00000167775:ENSG00000065989:ENSG00000104957:ENSG00000132017:ENSG00000132005:ENSG00000123146:ENSG00000123136:ENSG00000099308:ENSG00000124302:ENSG00000128626:ENSG00000269190:ENSG00000028277:ENSG00000167625:ENSG00000105722:ENSG00000079462:ENSG00000167619:ENSG00000105429:ENSG00000105427:ENSG00000079435:ENSG00000187244:ENSG00000185453:ENSG00000105483:ENSG00000105464:ENSG00000105447:ENSG00000142544:ENSG00000196214:ENSG00000198464:ENSG00000167766:ENSG00000213020:ENSG00000152467:ENSG00000115756:ENSG00000115761:ENSG00000197872:ENSG00000163803:ENSG00000171103:ENSG00000171094:ENSG00000119866:ENSG00000162928:ENSG00000082898:ENSG00000075292:ENSG00000144034:ENSG00000144048:ENSG00000114978:ENSG00000065911:ENSG00000115602:ENSG00000153107:ENSG00000153214:ENSG00000115963:ENSG00000153250:ENSG00000153253:ENSG00000118260:ENSG00000115457:ENSG00000115461:ENSG00000018280:ENSG00000144579:ENSG00000135912:ENSG00000144485:ENSG00000132326:ENSG00000162804:ENSG00000088876:ENSG00000132646:ENSG00000089048:ENSG00000188559:ENSG00000125966:ENSG00000198959:ENSG00000124134:ENSG00000124107:ENSG00000124228:ENSG00000124201:ENSG00000125520:ENSG00000198276:ENSG00000155313:ENSG00000154642:ENSG00000157617:ENSG00000160190:ENSG00000100342:ENSG00000100321:ENSG00000128285:ENSG00000198951:ENSG00000177096:ENSG00000100197:ENSG00000242247:ENSG00000138944:ENSG00000077942:ENSG00000186951:ENSG00000205643:ENSG00000075234:ENSG00000100416:ENSG00000196576:ENSG00000205593:ENSG00000100258:ENSG00000130489:ENSG00000025708:ENSG00000177989:ENSG00000205560:ENSG00000100288:ENSG00000008735:ENSG00000100299:ENSG00000251322:ENSG00000070950:ENSG00000134077:ENSG00000144560:ENSG00000163517:ENSG00000163513:ENSG00000170293:ENSG00000170011:ENSG00000168032:ENSG00000177873:ENSG00000182983:ENSG00000214706:ENSG00000114378:ENSG00000068001:ENSG00000068028:ENSG00000114395:ENSG00000126062:ENSG00000180376:ENSG00000163946:ENSG00000114861:ENSG00000144736:ENSG00000163320:ENSG00000175105:ENSG00000179021:ENSG00000184500:ENSG00000169379:ENSG00000114446:ENSG00000240891:ENSG00000163607:ENSG00000163608:ENSG00000144843:ENSG00000172780:ENSG00000240682:ENSG00000034533:ENSG00000240303:ENSG00000113971:ENSG00000154917:ENSG00000138231:ENSG00000114098:ENSG00000158186:ENSG00000184432:ENSG00000155903:ENSG00000175066:ENSG00000114127:ENSG00000169282:ENSG00000075420:ENSG00000161202:ENSG00000114279:ENSG00000041802:ENSG00000013288:ENSG00000174145:ENSG00000169019:ENSG00000145246:ENSG00000109171:ENSG00000145248:ENSG00000080493:ENSG00000169429:ENSG00000163739:ENSG00000173085:ENSG00000185477:ENSG00000145331:ENSG00000205403:ENSG00000109466:ENSG00000215218:ENSG00000113100:ENSG00000164190:ENSG00000067248:ENSG00000039123:ENSG00000086200:ENSG00000164331:ENSG00000164338:ENSG00000164180:ENSG00000181751:ENSG00000173926:ENSG00000145794:ENSG00000064651:ENSG00000240184:ENSG00000242419:ENSG00000240764:ENSG00000131504:ENSG00000171720:ENSG00000164620:ENSG00000197948:ENSG00000120318:ENSG00000113721:ENSG00000135074:EN

						SG00000113272:ENSG00000182132:ENSG00000074317:ENSG00000175766:ENSG00000249109:ENSG00000131446:ENSG00000196670:ENSG00000078401:ENSG00000112812:ENSG00000146112:ENSG00000137404:ENSG00000137337:ENSG00000137331:ENSG00000204344:ENSG00000244731:ENSG00000223501:ENSG00000235863:ENSG00000227057:ENSG00000237441:ENSG00000124762:ENSG00000124772:ENSG00000198663:ENSG00000164530:ENSG00000112245:ENSG00000118432:ENSG00000172461:ENSG00000198945:ENSG00000118503:ENSG00000112406:ENSG00000120278:ENSG00000186340:ENSG00000184786:ENSG00000130023:ENSG00000122644:ENSG00000164600:ENSG00000152926:ENSG00000213462:ENSG00000126522:ENSG00000086730:ENSG00000049541:ENSG00000177679:ENSG00000127920:ENSG00000105829:ENSG00000085514:ENSG00000078487:ENSG00000146834:ENSG00000160813:ENSG00000166924:ENSG00000172209:ENSG00000091704:ENSG00000131558:ENSG00000122786:ENSG00000182158:ENSG00000181444:ENSG00000196329:ENSG00000214106:ENSG00000146918:ENSG00000117868:ENSG00000154319:ENSG00000168546:ENSG00000120910:ENSG00000165061:ENSG00000023287:ENSG00000254087:ENSG00000035681:ENSG00000121039:ENSG00000104267:ENSG00000104327:ENSG00000164949:ENSG00000120526:ENSG00000131773:ENSG00000196378:ENSG00000080608:ENSG00000213694:ENSG00000165023:ENSG00000127080:ENSG00000130958:ENSG00000136925:ENSG00000136936:ENSG00000136826:ENSG00000070159:ENSG00000167081:ENSG00000196152:ENSG00000136840:ENSG00000171169:ENSG00000148339:ENSG00000148335:ENSG00000148308:ENSG00000170835:ENSG00000148411:ENSG00000198113
DEG.twoside	Brain_Caudate_basal_ganglia	9143	581	2.30369916126038e-24	1.2439975470806e-22	ENSG00000158286:ENSG00000158292:ENSG00000097021:ENSG00000171824:ENSG00000120942:ENSG00000162458:ENSG00000065526:ENSG00000116809:ENSG00000183888:ENSG00000158748:ENSG00000204178:ENSG00000157978:ENSG00000084652:ENSG00000116525:ENSG00000160094:ENSG00000134686:ENSG00000121904:ENSG00000177181:ENSG00000243710:ENSG00000066056:ENSG00000066322:ENSG00000159479:ENSG00000198198:ENSG00000162367:ENSG00000117862:ENSG00000198841:ENSG00000117114:ENSG00000117155:ENSG00000142867:ENSG00000153904:ENSG00000171488:ENSG00000069712:ENSG00000117528:ENSG00000117543:ENSG00000118733:ENSG00000116396:ENSG00000162775:ENSG00000168679:ENSG00000116455:ENSG00000163349:ENSG00000197323:ENSG00000213281:ENSG00000052723:ENSG00000092621:ENSG00000143457:ENSG00000163131:ENSG00000143387:ENSG00000143437:ENSG00000143379:ENSG00000143412:ENSG00000143409:ENSG00000143363:ENSG00000143434:ENSG00000163154:ENSG00000163156:ENSG00000143537:ENSG00000160767:ENSG00000116521:ENSG00000176444:ENSG00000143630:ENSG00000160752:ENSG00000160753:ENSG00000116539:ENSG00000125459:ENSG00000163374:ENSG00000132676:ENSG00000116580:ENSG00000132718:ENSG00000143622:ENSG00000132680:ENSG00000160789:ENSG00000160783:ENSG00000160781:ENSG00000163472:ENSG00000198715:ENSG00000187800:ENSG00000132694:ENSG00000171786:ENSG00000143226:ENSG00000120341:ENSG00000186283:ENSG00000057252:ENSG00000134376:ENSG00000174307:ENSG00000122176:ENSG00000182004:ENSG00000143801:ENSG00000163050:ENSG00000162885:ENSG00000116962:ENSG00000162714:ENSG00000107929:ENSG00000134463:ENSG00000148468:ENSG00000165997:ENSG00000165476:ENSG00000107736:ENSG00000214688:ENSG00000148604:ENSG00000138134:ENSG00000107796:ENSG00000026103:ENSG00000165338:ENSG00000059573:ENSG00000197430:ENSG00000095587:ENSG00000203867:ENSG00000198873:ENSG00000184545:ENSG00000166311:ENSG00000205339:ENSG00000166478:ENSG00000166483:ENSG00000198730:ENSG00000006071:ENSG00000129151:ENSG00000149150:ENSG00000162174:ENSG00000162300:ENSG00000174276:ENSG00000162298:ENSG00000149792:ENSG00000173653:ENSG00000173599:ENSG00000175567:ENSG00000175564:ENSG00000137492:ENSG00000137693:ENSG00000149313:ENSG00000137714:ENSG00000183644:ENSG00000149292:ENSG00000076053:ENSG00000076043:ENSG00000154127:ENSG00000023171:ENSG00000109832:ENSG00000064309:ENSG00000183715:ENSG00000010219:ENSG00000111262:ENSG00000111671:ENSG00000010626:ENSG00000111679:ENSG00000126749:ENSG00000111684:ENSG00000182326:ENSG00000134531:ENSG00000123094:ENSG00000110911:ENSG00000123338:ENSG00000123360:ENSG00000127314:ENSG00000166130:ENSG00000235162:ENSG00000074590:ENSG00000135094:ENSG00000174989:ENSG00000111725:ENSG00000122966:ENSG00000150990:ENSG00000177084:ENSG00000176915:ENSG00000127863:ENSG00000102743:ENSG00000215475:ENSG00000136167:ENSG00000102445:ENSG00000102531:ENSG00000123179:ENSG00000102753:ENSG00000184226:ENSG00000188243:ENSG00000178695:ENSG00000088451:ENSG00000065150:ENSG00000134901:ENSG00000139826:ENSG00000102524:ENSG00000100889:ENSG00000168952:ENSG00000176165:ENSG00000186960:ENSG00000092108:ENSG00000100473:ENSG00000150527:ENSG00000179476:ENSG00000179454:ENSG00000185246:ENSG00000139926:ENSG00000180998:ENSG00000197930:ENSG00000119599:ENSG00000165861:ENSG00000119707:ENSG00000133961:ENSG00000100433:ENSG00000165934:ENSG00000153485:ENSG00000140157:ENSG00000170113:ENSG00000169918:ENSG00000021776:ENSG00000198146:ENSG00000174197:ENSG00000137802:ENSG00000243708:ENSG00000243789:ENSG00000197535:ENSG00000181827:ENSG00000140262:ENSG00000103707:ENSG00000117899:ENSG00000140600:ENSG00000140511:ENSG00000140471:ENSG00000183454:ENSG00000183426:ENSG00000179889:ENSG00000085721:ENSG00000157106:ENSG00000169221:ENSG00000180096:ENSG00000179958:ENSG00000140691:ENSG00000140682:ENSG00000197302:ENSG00000102910:ENSG00000196470:ENSG00000102921:ENSG00000005194:ENSG00000072736:ENSG00000103066:ENSG00000103064:ENSG00000103061:ENSG00000103056:ENSG00000172137:ENSG00000158717:ENSG00000167522:ENSG00000197912:ENSG00000127804:ENSG00000197417:ENSG00000005100:ENSG00000072849:ENSG00000007174:ENSG00000108641:ENSG00000166484:ENSG00000166482:ENSG00000128482:ENSG00000142494:ENSG00000180638:ENSG00000067191:ENSG00000068079:ENSG00000108830:ENSG00000012048:ENSG00000182963:ENSG00000225190:ENSG00000141279:ENSG00000108424:ENSG00000198933:ENSG00000011258:ENSG00000011260:ENSG00000214226:ENSG00000121060:ENSG00000108423:ENSG00000108443:ENSG00000189050:ENSG00000198265:ENSG00000197170:ENSG00000154217:ENSG00000141540:ENSG00000

						196169:ENSG00000141576:ENSG00000185262:ENSG00000141577:ENSG00000167302:ENSG00000185504:ENSG00000182446:ENSG00000101608:ENSG00000177 97:ENSG00000154845:ENSG00000154889:ENSG00000141401:ENSG00000168234:ENSG00000141384:ENSG00000141469:ENSG00000152223:ENSG00000081913:E NSG00000197971:ENSG00000071564:ENSG00000141985:ENSG00000167670:ENSG00000205744:ENSG00000104833:ENSG00000171105:ENSG00000104880:ENSG 00000104883:ENSG00000167775:ENSG00000079999:ENSG00000180739:ENSG00000104957:ENSG00000132016:ENSG00000132024:ENSG00000132000:ENSG0000 0132017:ENSG00000132005:ENSG00000123146:ENSG00000123136:ENSG00000127527:ENSG00000099308:ENSG00000104835:ENSG00000128626:ENSG00000269 190:ENSG00000028277:ENSG00000160570:ENSG00000167625:ENSG00000105722:ENSG00000079462:ENSG00000188368:ENSG00000167619:ENSG00000187244: ENSG00000185453:ENSG00000105483:ENSG00000105447:ENSG00000063127:ENSG00000196214:ENSG00000198464:ENSG00000198633:ENSG00000152467:ENS G00000115756:ENSG00000115761:ENSG00000197872:ENSG00000055917:ENSG00000163803:ENSG00000171103:ENSG00000119866:ENSG00000162928:ENSG00 000082898:ENSG00000144034:ENSG00000144048:ENSG00000114978:ENSG00000065911:ENSG00000115602:ENSG00000115604:ENSG00000153107:ENSG000001 53214:ENSG00000115963:ENSG00000144283:ENSG00000153250:ENSG00000153253:ENSG00000118260:ENSG00000115457:ENSG00000115461:ENSG0000001828 0:ENSG00000144579:ENSG00000123977:ENSG00000144485:ENSG00000132326:ENSG00000162804:ENSG00000088876:ENSG00000125779:ENSG00000132646:EN SG00000188559:ENSG00000125966:ENSG00000198959:ENSG00000124107:ENSG00000124228:ENSG00000124201:ENSG00000125520:ENSG00000198276:ENSG0 0000155313:ENSG00000157617:ENSG00000160190:ENSG00000184792:ENSG00000100220:ENSG00000128335:ENSG00000100342:ENSG00000100321:ENSG00000 198951:ENSG00000177096:ENSG00000242247:ENSG00000077942:ENSG00000205643:ENSG00000075234:ENSG00000100416:ENSG00000196576:ENSG000002055 93:ENSG00000100258:ENSG00000025770:ENSG00000130489:ENSG00000025708:ENSG00000177989:ENSG00000205560:ENSG00000100288:ENSG00000008735:E NSG00000100299:ENSG00000070950:ENSG00000134077:ENSG00000144560:ENSG00000163517:ENSG00000163513:ENSG00000170293:ENSG00000168032:ENSG 00000177873:ENSG00000182606:ENSG00000182983:ENSG00000214706:ENSG00000114378:ENSG00000068001:ENSG00000068028:ENSG00000004838:ENSG0000 0114395:ENSG00000126062:ENSG00000007402:ENSG00000180376:ENSG00000163946:ENSG00000144741:ENSG00000144736:ENSG00000163320:ENSG00000175 105:ENSG00000179021:ENSG00000184500:ENSG00000114446:ENSG00000163607:ENSG00000163608:ENSG00000144843:ENSG00000172780:ENSG00000240682: ENSG00000034533:ENSG00000114670:ENSG00000240303:ENSG00000113971:ENSG00000081307:ENSG00000144867:ENSG00000154917:ENSG00000158163:ENS G00000138231:ENSG00000114098:ENSG00000158186:ENSG00000175110:ENSG00000184432:ENSG00000155903:ENSG00000175066:ENSG00000114127:ENSG00 000114698:ENSG00000169282:ENSG00000075420:ENSG00000161202:ENSG00000161204:ENSG00000114279:ENSG00000041802:ENSG00000163975:ENSG000001 45246:ENSG00000109171:ENSG00000145248:ENSG00000080493:ENSG00000169429:ENSG00000163739:ENSG00000173085:ENSG00000205403:ENSG0000016412 9:ENSG00000109466:ENSG00000129187:ENSG00000113100:ENSG00000113361:ENSG00000164190:ENSG00000132357:ENSG00000067248:ENSG00000039123:EN SG00000086200:ENSG00000164331:ENSG00000164338:ENSG00000164180:ENSG00000175471:ENSG00000173926:ENSG00000145794:ENSG00000253159:ENSG0 0000240184:ENSG00000240764:ENSG00000131504:ENSG00000171720:ENSG00000164620:ENSG00000197948:ENSG00000120318:ENSG00000113721:ENSG00000 135074:ENSG00000113272:ENSG00000155858:ENSG00000182132:ENSG00000074317:ENSG00000048140:ENSG00000249109:ENSG00000131446:ENSG000001966 70:ENSG00000146112:ENSG00000137404:ENSG00000137337:ENSG00000137331:ENSG00000204344:ENSG00000244731:ENSG00000223501:ENSG00000235863:E NSG00000227057:ENSG00000124762:ENSG00000124772:ENSG00000198663:ENSG00000164530:ENSG00000112245:ENSG00000172461:ENSG00000198945:ENSG 00000135525:ENSG00000118503:ENSG00000112406:ENSG00000120278:ENSG00000112096:ENSG00000164488:ENSG00000186340:ENSG00000184786:ENSG0000 0130023:ENSG00000122644:ENSG00000105855:ENSG00000153814:ENSG00000168303:ENSG00000152926:ENSG00000213462:ENSG00000126522:ENSG00000086 730:ENSG00000049541:ENSG00000177679:ENSG00000135218:ENSG00000085563:ENSG00000127920:ENSG00000105829:ENSG00000085514:ENSG00000146834: ENSG00000160813:ENSG00000166924:ENSG00000005249:ENSG00000196937:ENSG00000091704:ENSG00000158623:ENSG00000131558:ENSG00000122786:ENS G00000182158:ENSG00000196329:ENSG00000146918:ENSG00000117868:ENSG00000184608:ENSG00000154319:ENSG00000168546:ENSG00000120910:ENSG00 000129691:ENSG00000165061:ENSG00000254087:ENSG00000035681:ENSG00000121039:ENSG00000104267:ENSG00000104327:ENSG00000164949:ENSG000001 20526:ENSG00000120533:ENSG00000180900:ENSG00000196378:ENSG00000080608:ENSG00000044459:ENSG00000213694:ENSG00000165023:ENSG0000012708 0:ENSG00000130958:ENSG00000136925:ENSG00000136936:ENSG00000136826:ENSG00000196152:ENSG00000148356:ENSG00000136840:ENSG00000171169:EN SG00000148335:ENSG00000148308:ENSG00000170835:ENSG00000148411:ENSG00000198113
DEG.twoside	Brain_Cerebellar_Hemisphere	8981	459	0.000242284759614317	0.0130833770191731	ENSG00000158286:ENSG00000158292:ENSG00000097021:ENSG00000198793:ENSG00000065526:ENSG00000183888:ENSG00000204178:ENSG00000157978:ENS G00000160050:ENSG00000160051:ENSG00000116525:ENSG00000160094:ENSG00000121904:ENSG00000177181:ENSG00000179178:ENSG00000066056:ENSG00 000066322:ENSG00000134709:ENSG00000162616:ENSG00000117114:ENSG00000117133:ENSG00000117155:ENSG00000162642:ENSG00000142867:ENSG000001 53904:ENSG00000197147:ENSG00000069712:ENSG00000117525:ENSG00000117569:ENSG00000162694:ENSG00000118733:ENSG00000116396:ENSG0000008546 5:ENSG00000163349:ENSG00000197323:ENSG00000175984:ENSG00000052723:ENSG00000163131:ENSG00000143387:ENSG00000143434:ENSG00000163154:EN SG00000163155:ENSG00000160767:ENSG00000143630:ENSG00000143627:ENSG00000160753:ENSG00000116539:ENSG00000132718:ENSG00000143622:ENSG0 0000116584:ENSG00000160789:ENSG00000196189:ENSG00000242252:ENSG00000160781:ENSG00000198715:ENSG00000187800:ENSG00000132694:ENSG00000

171786:ENSG00000143226:ENSG00000203747:ENSG00000186283:ENSG00000143322:ENSG00000057252:ENSG00000177888:ENSG00000134376:ENSG000001743
07:ENSG00000143801:ENSG00000163050:ENSG00000116962:ENSG00000198879:ENSG00000134463:ENSG00000148468:ENSG00000241058:ENSG00000095794:E
NSG00000188234:ENSG00000075073:ENSG00000107736:ENSG00000107738:ENSG00000107796:ENSG00000026103:ENSG00000165338:ENSG00000197430:ENSG
00000155256:ENSG00000184545:ENSG00000205869:ENSG00000185940:ENSG00000110169:ENSG00000268403:ENSG00000166483:ENSG00000188211:ENSG0000
0187486:ENSG00000006071:ENSG00000019505:ENSG00000149150:ENSG00000162174:ENSG00000110025:ENSG00000168061:ENSG00000168060:ENSG00000146
670:ENSG00000173653:ENSG00000173599:ENSG00000173621:ENSG00000175567:ENSG00000179240:ENSG00000137693:ENSG00000149313:ENSG00000137714:
ENSG00000149292:ENSG00000076043:ENSG00000154127:ENSG00000109943:ENSG00000023171:ENSG00000109832:ENSG00000064309:ENSG00000183715:ENS
G00000082805:ENSG00000139180:ENSG00000111262:ENSG00000010626:ENSG00000111679:ENSG00000182326:ENSG00000134531:ENSG00000110925:ENSG00
000127314:ENSG00000235162:ENSG00000074590:ENSG00000110887:ENSG00000139410:ENSG00000122966:ENSG00000188735:ENSG00000150990:ENSG000001
84992:ENSG00000177084:ENSG00000127863:ENSG00000102743:ENSG00000215475:ENSG00000136167:ENSG00000102445:ENSG00000123179:ENSG0000018422
6:ENSG00000136153:ENSG00000152749:ENSG00000134897:ENSG00000102524:ENSG00000100889:ENSG00000168952:ENSG00000092140:ENSG00000129493:EN
SG00000150527:ENSG00000179454:ENSG00000198718:ENSG00000185246:ENSG00000087301:ENSG00000180998:ENSG00000006432:ENSG00000119599:ENSG0
0000100767:ENSG00000165417:ENSG00000100433:ENSG00000170113:ENSG00000128731:ENSG00000169918:ENSG00000174197:ENSG00000137802:ENSG00000
243708:ENSG00000243789:ENSG00000197535:ENSG00000181827:ENSG00000103707:ENSG00000103710:ENSG00000188779:ENSG00000140350:ENSG000001404
06:ENSG00000140600:ENSG00000185418:ENSG00000008710:ENSG00000183454:ENSG00000103512:ENSG00000183426:ENSG00000157045:ENSG00000085721:E
NSG00000157106:ENSG00000155714:ENSG00000102879:ENSG00000132207:ENSG00000180035:ENSG00000179965:ENSG00000140691:ENSG00000140682:ENSG
00000197302:ENSG00000103066:ENSG00000132600:ENSG00000103056:ENSG00000172137:ENSG00000167515:ENSG00000205022:ENSG00000197912:ENSG0000
0196689:ENSG00000005100:ENSG00000109047:ENSG00000233670:ENSG00000181350:ENSG00000141127:ENSG00000072134:ENSG00000166484:ENSG00000166
482:ENSG00000128482:ENSG00000142494:ENSG00000067191:ENSG00000141750:ENSG00000068079:ENSG00000108828:ENSG00000108830:ENSG00000073670:
ENSG00000225190:ENSG00000198933:ENSG00000011258:ENSG00000011260:ENSG00000153933:ENSG00000154217:ENSG00000153822:ENSG00000180616:ENS
G00000196169:ENSG00000141576:ENSG00000185262:ENSG00000078687:ENSG00000141577:ENSG00000185504:ENSG00000101608:ENSG00000134265:ENSG00
000168234:ENSG00000141469:ENSG00000101638:ENSG00000197971:ENSG00000071564:ENSG00000205744:ENSG00000104833:ENSG00000104883:ENSG000000
65989:ENSG00000180739:ENSG00000132016:ENSG00000132017:ENSG00000132005:ENSG00000123146:ENSG00000127527:ENSG00000099308:ENSG0000012430
2:ENSG00000262484:ENSG00000269190:ENSG00000028277:ENSG00000160570:ENSG00000105722:ENSG00000079462:ENSG00000188368:ENSG00000167619:EN
SG00000105429:ENSG00000105427:ENSG00000187244:ENSG00000105483:ENSG00000105464:ENSG00000142544:ENSG00000198633:ENSG00000167766:ENSG0
0000152467:ENSG00000176293:ENSG00000115756:ENSG00000084676:ENSG00000171103:ENSG00000162929:ENSG00000075292:ENSG00000144034:ENSG00000
217702:ENSG00000114978:ENSG00000065911:ENSG00000188687:ENSG00000005448:ENSG00000114993:ENSG00000183281:ENSG00000198885:ENSG000001442
18:ENSG00000115602:ENSG00000153208:ENSG00000153214:ENSG00000115762:ENSG00000163040:ENSG00000115963:ENSG00000144283:ENSG00000153250:E
NSG00000153253:ENSG00000115457:ENSG00000018280:ENSG00000144579:ENSG00000186235:ENSG00000144485:ENSG00000125779:ENSG00000101290:ENSG
00000101247:ENSG00000125966:ENSG00000198959:ENSG00000124107:ENSG00000124201:ENSG00000203896:ENSG00000155313:ENSG00000154642:ENSG0000
0157617:ENSG00000183628:ENSG00000184792:ENSG00000128335:ENSG00000100342:ENSG00000100321:ENSG00000100324:ENSG00000198951:ENSG00000100
197:ENSG00000077942:ENSG00000186951:ENSG00000075234:ENSG00000100416:ENSG00000205593:ENSG00000025708:ENSG00000205560:ENSG00000008735:
ENSG00000251322:ENSG00000144560:ENSG00000163513:ENSG00000170293:ENSG00000170011:ENSG00000168032:ENSG00000177842:ENSG00000182606:ENS
G00000182983:ENSG00000214706:ENSG00000186792:ENSG00000114378:ENSG00000068001:ENSG00000114388:ENSG00000114395:ENSG00000007402:ENSG00
000180376:ENSG00000114861:ENSG00000184500:ENSG00000114446:ENSG00000240891:ENSG00000144843:ENSG00000082701:ENSG00000114670:ENSG000001
13971:ENSG00000144867:ENSG00000154917:ENSG00000114098:ENSG00000155903:ENSG00000175066:ENSG00000114698:ENSG00000163762:ENSG0000016928
2:ENSG00000075420:ENSG00000073803:ENSG00000163904:ENSG00000114279:ENSG00000145014:ENSG00000013288:ENSG00000157765:ENSG00000145246:EN
SG00000109171:ENSG00000080493:ENSG00000169429:ENSG00000163739:ENSG00000173085:ENSG00000185477:ENSG00000151247:ENSG00000123739:ENSG0
0000205403:ENSG00000164128:ENSG00000129187:ENSG00000215218:ENSG00000113360:ENSG00000082213:ENSG00000067113:ENSG00000164331:ENSG00000
175471:ENSG00000181751:ENSG00000168916:ENSG00000173926:ENSG00000145794:ENSG00000240184:ENSG00000242419:ENSG00000240764:ENSG000001646
20:ENSG00000197948:ENSG00000113721:ENSG00000113272:ENSG00000155858:ENSG00000182132:ENSG00000113742:ENSG00000074317:ENSG00000175766:E
NSG00000249109:ENSG00000131446:ENSG00000124535:ENSG00000124787:ENSG00000078401:ENSG00000079691:ENSG00000146112:ENSG00000137404:ENSG
00000137331:ENSG00000244731:ENSG00000235863:ENSG00000124762:ENSG00000164530:ENSG00000118432:ENSG00000172461:ENSG00000198945:ENSG0000
0135525:ENSG00000112406:ENSG00000120278:ENSG00000131018:ENSG00000026297:ENSG00000213066:ENSG00000186340:ENSG00000122644:ENSG00000105
855:ENSG00000153814:ENSG00000164600:ENSG00000152926:ENSG00000146757:ENSG00000126522:ENSG00000189143:ENSG00000177679:ENSG00000127920:

						ENSG00000085514:ENSG00000078487:ENSG00000160813:ENSG00000166924:ENSG00000172209:ENSG00000091704:ENSG00000106484:ENSG00000158623:ENSG00000182158:ENSG00000181444:ENSG00000196329:ENSG00000214106:ENSG00000168546:ENSG00000023287:ENSG00000147601:ENSG00000121039:ENSG00000104327:ENSG00000164949:ENSG00000129295:ENSG00000196378:ENSG00000080608:ENSG00000230453:ENSG00000165023:ENSG00000130958:ENSG00000136925:ENSG00000136826:ENSG00000070159:ENSG00000167081:ENSG00000148356:ENSG00000170835:ENSG00000148411
DEG.twoside	Brain_Cerebellum	8976	457	0.00035660635208335	0.0192567430125009	ENSG00000158286:ENSG00000158292:ENSG00000097021:ENSG00000198793:ENSG00000162458:ENSG00000065526:ENSG00000183888:ENSG00000204178:ENSG00000157978:ENSG00000160050:ENSG00000160051:ENSG00000116525:ENSG00000160094:ENSG00000121904:ENSG00000177181:ENSG00000066056:ENSG00000198198:ENSG00000162367:ENSG00000134709:ENSG00000162616:ENSG00000117114:ENSG00000117133:ENSG00000117155:ENSG00000142867:ENSG00000153904:ENSG00000197147:ENSG00000069712:ENSG00000117525:ENSG00000118733:ENSG00000134215:ENSG00000116396:ENSG00000085465:ENSG00000163349:ENSG00000175984:ENSG00000143457:ENSG00000163131:ENSG00000143387:ENSG00000143434:ENSG00000163154:ENSG00000163155:ENSG00000160767:ENSG00000143630:ENSG00000143627:ENSG00000160753:ENSG00000116539:ENSG00000132718:ENSG00000143622:ENSG00000116584:ENSG00000160789:ENSG00000196189:ENSG00000242252:ENSG00000160781:ENSG00000198715:ENSG00000187800:ENSG00000132694:ENSG00000171786:ENSG00000143226:ENSG00000173110:ENSG00000203747:ENSG00000186283:ENSG00000057252:ENSG00000177888:ENSG00000134376:ENSG00000174307:ENSG00000122176:ENSG00000143801:ENSG00000163050:ENSG00000116962:ENSG00000198879:ENSG00000134463:ENSG00000148468:ENSG00000241058:ENSG00000095794:ENSG00000188234:ENSG00000075073:ENSG00000107736:ENSG00000107738:ENSG00000107796:ENSG00000026103:ENSG00000197430:ENSG00000155256:ENSG00000184545:ENSG00000205869:ENSG00000185940:ENSG00000110169:ENSG00000268403:ENSG00000166483:ENSG00000188211:ENSG00000187486:ENSG00000006071:ENSG00000019505:ENSG00000149150:ENSG00000162174:ENSG00000110025:ENSG00000168061:ENSG00000168060:ENSG00000146670:ENSG00000173653:ENSG00000173599:ENSG00000173621:ENSG00000175567:ENSG00000175564:ENSG00000179240:ENSG00000137693:ENSG00000149313:ENSG00000137714:ENSG00000149292:ENSG00000076043:ENSG00000154127:ENSG00000109943:ENSG00000023171:ENSG00000109832:ENSG00000064309:ENSG00000183715:ENSG00000082805:ENSG00000111262:ENSG00000010626:ENSG00000111679:ENSG00000182326:ENSG00000134531:ENSG00000134532:ENSG00000110925:ENSG00000127314:ENSG00000235162:ENSG00000074590:ENSG00000110887:ENSG00000139410:ENSG00000122966:ENSG00000188735:ENSG00000150990:ENSG00000184992:ENSG00000177084:ENSG00000127863:ENSG00000102743:ENSG00000215475:ENSG00000136167:ENSG00000123179:ENSG00000184226:ENSG00000188243:ENSG00000118939:ENSG00000136153:ENSG00000152749:ENSG00000134897:ENSG00000100889:ENSG00000168952:ENSG00000100473:ENSG00000129493:ENSG00000150527:ENSG00000198718:ENSG00000185246:ENSG00000087301:ENSG00000180998:ENSG00000006432:ENSG00000119599:ENSG00000100767:ENSG00000100433:ENSG00000170113:ENSG00000128731:ENSG00000169918:ENSG00000174197:ENSG00000137802:ENSG00000243708:ENSG00000243789:ENSG00000197535:ENSG00000181827:ENSG00000103710:ENSG00000188779:ENSG00000140350:ENSG00000136378:ENSG00000103888:ENSG00000117899:ENSG00000140406:ENSG00000140600:ENSG00000140511:ENSG00000185418:ENSG00000008710:ENSG00000183454:ENSG00000103512:ENSG00000183426:ENSG00000157045:ENSG00000157106:ENSG00000155714:ENSG00000102879:ENSG00000132207:ENSG00000261052:ENSG00000198064:ENSG00000180209:ENSG00000180035:ENSG00000179965:ENSG00000140691:ENSG00000140682:ENSG00000197302:ENSG00000103067:ENSG00000103066:ENSG00000132600:ENSG00000103056:ENSG00000103018:ENSG00000172137:ENSG00000158717:ENSG00000167515:ENSG00000205022:ENSG00000197912:ENSG00000196689:ENSG00000005100:ENSG00000233670:ENSG00000181350:ENSG00000141127:ENSG00000072134:ENSG00000166482:ENSG00000128482:ENSG00000142494:ENSG00000067191:ENSG00000141750:ENSG00000068079:ENSG00000108828:ENSG00000108830:ENSG00000073670:ENSG00000225190:ENSG00000198933:ENSG00000011258:ENSG00000153933:ENSG00000154217:ENSG00000153822:ENSG00000180616:ENSG00000141576:ENSG00000078687:ENSG00000141577:ENSG00000185504:ENSG00000101608:ENSG00000134265:ENSG00000168234:ENSG00000141469:ENSG00000101638:ENSG00000081913:ENSG00000197971:ENSG00000205744:ENSG00000104833:ENSG00000104883:ENSG00000065989:ENSG00000180739:ENSG00000132016:ENSG00000132017:ENSG00000132005:ENSG00000123146:ENSG00000127527:ENSG00000099308:ENSG00000124302:ENSG00000104825:ENSG00000262484:ENSG00000269190:ENSG00000028277:ENSG00000105722:ENSG00000079462:ENSG00000188368:ENSG00000167619:ENSG00000105429:ENSG00000105427:ENSG00000187244:ENSG00000105483:ENSG00000105464:ENSG00000182324:ENSG00000142544:ENSG00000198633:ENSG00000167766:ENSG00000152467:ENSG00000176293:ENSG00000115756:ENSG00000162929:ENSG00000075292:ENSG00000217702:ENSG00000114978:ENSG00000065911:ENSG00000188687:ENSG00000005448:ENSG00000183281:ENSG00000198885:ENSG00000144218:ENSG00000115602:ENSG00000153208:ENSG00000153214:ENSG00000115762:ENSG00000163040:ENSG00000115963:ENSG00000144283:ENSG00000153250:ENSG00000153253:ENSG00000115457:ENSG00000018280:ENSG00000144579:ENSG00000186235:ENSG00000144485:ENSG00000125779:ENSG00000101290:ENSG00000101247:ENSG00000125966:ENSG00000198959:ENSG00000124107:ENSG00000203896:ENSG00000155313:ENSG00000154642:ENSG00000157617:ENSG00000160190:ENSG00000183628:ENSG00000184792:ENSG00000128335:ENSG00000100342:ENSG00000100321:ENSG00000100324:ENSG00000198951:ENSG00000177096:ENSG00000100197:ENSG00000077942:ENSG00000186951:ENSG00000075234:ENSG00000100416:ENSG00000205593:ENSG00000025708:ENSG00000205560:ENSG00000008735:ENSG00000251322:ENSG00000144560:ENSG00000163513:ENSG00000170293:ENSG00000170011:ENSG00000168032:ENSG00000177842:ENSG00000182606:ENSG00000182983:ENSG00000186792:ENSG00000243477:ENSG00000114378:ENSG00000068001:ENSG00000114388:ENSG00000114395:ENSG00000007402:ENSG00000180376:ENSG00000114861:ENSG00

						000184500:ENSG00000114446:ENSG00000240891:ENSG00000144843:ENSG00000082701:ENSG00000113971:ENSG00000144867:ENSG00000154917:ENSG00000114098:ENSG00000155903:ENSG00000114698:ENSG00000163762:ENSG00000169282:ENSG00000075420:ENSG00000114279:ENSG00000145014:ENSG00000013288:ENSG00000157765:ENSG00000145246:ENSG00000109171:ENSG00000080493:ENSG00000169429:ENSG00000163739:ENSG00000173085:ENSG00000185477:ENSG00000151247:ENSG00000205403:ENSG00000164128:ENSG00000129187:ENSG00000215218:ENSG00000113360:ENSG00000132357:ENSG00000067113:ENSG00000175471:ENSG00000181751:ENSG00000168916:ENSG00000173926:ENSG00000145794:ENSG00000240184:ENSG00000242419:ENSG00000240764:ENSG00000164620:ENSG00000197948:ENSG00000113721:ENSG00000113272:ENSG00000155858:ENSG00000182132:ENSG00000113742:ENSG00000074317:ENSG00000175766:ENSG00000048140:ENSG00000249109:ENSG00000131446:ENSG00000124535:ENSG00000124787:ENSG00000078401:ENSG00000079691:ENSG00000146112:ENSG00000137404:ENSG00000137331:ENSG00000244731:ENSG00000235863:ENSG00000124762:ENSG00000164530:ENSG00000112245:ENSG00000118432:ENSG00000172461:ENSG00000198945:ENSG00000135525:ENSG00000118503:ENSG00000112406:ENSG00000120278:ENSG00000131018:ENSG00000112096:ENSG00000026297:ENSG00000213066:ENSG00000186340:ENSG00000122644:ENSG00000153814:ENSG00000164600:ENSG00000146757:ENSG00000126522:ENSG00000189143:ENSG00000086730:ENSG00000177679:ENSG00000127920:ENSG00000085514:ENSG00000078487:ENSG00000160813:ENSG00000166924:ENSG00000172209:ENSG00000106484:ENSG00000158623:ENSG00000182158:ENSG00000159763:ENSG00000181444:ENSG00000196329:ENSG00000214106:ENSG00000154319:ENSG00000147601:ENSG00000121039:ENSG00000104327:ENSG00000164949:ENSG00000129295:ENSG00000169436:ENSG00000196378:ENSG00000080608:ENSG00000178031:ENSG00000230453:ENSG00000213694:ENSG00000165023:ENSG00000127080:ENSG00000130958:ENSG00000136925:ENSG00000136826:ENSG00000070159:ENSG00000167081:ENSG00000148356:ENSG00000170835:ENSG00000148411
DEG.twoside	Brain_Cortex	8420	491	1.89614435949776e-12	1.02391795412879e-10	ENSG00000116237:ENSG00000097021:ENSG00000120942:ENSG00000162458:ENSG00000065526:ENSG00000183888:ENSG00000158748:ENSG00000157978:ENSG00000084652:ENSG00000121904:ENSG00000177181:ENSG00000066056:ENSG00000066322:ENSG00000198198:ENSG00000162367:ENSG00000117862:ENSG00000162616:ENSG00000117133:ENSG00000117155:ENSG00000142867:ENSG00000153904:ENSG00000197147:ENSG00000171488:ENSG00000069712:ENSG00000117528:ENSG00000117543:ENSG00000118733:ENSG00000134215:ENSG00000116396:ENSG00000162775:ENSG00000168679:ENSG00000116455:ENSG00000163349:ENSG00000197323:ENSG00000213281:ENSG00000052723:ENSG00000092621:ENSG00000143457:ENSG00000163131:ENSG00000143387:ENSG00000143437:ENSG00000143379:ENSG00000143418:ENSG00000143412:ENSG00000143409:ENSG00000143363:ENSG00000163154:ENSG00000176444:ENSG00000160753:ENSG00000163374:ENSG00000132676:ENSG00000116580:ENSG00000132718:ENSG00000143622:ENSG00000132680:ENSG00000160789:ENSG00000160783:ENSG00000160781:ENSG00000163472:ENSG00000198715:ENSG00000187800:ENSG00000143226:ENSG00000173110:ENSG00000203747:ENSG00000162746:ENSG00000186283:ENSG00000143322:ENSG00000057252:ENSG00000134376:ENSG00000122176:ENSG00000182004:ENSG00000163050:ENSG00000162885:ENSG00000116962:ENSG00000107929:ENSG00000134463:ENSG00000148468:ENSG00000165997:ENSG00000165476:ENSG00000075073:ENSG00000107736:ENSG00000214688:ENSG00000107738:ENSG00000148604:ENSG00000107796:ENSG00000026103:ENSG00000059573:ENSG00000197430:ENSG00000120057:ENSG00000198873:ENSG00000184545:ENSG00000205339:ENSG00000166478:ENSG00000198730:ENSG00000187486:ENSG00000006071:ENSG00000129151:ENSG00000019505:ENSG00000149150:ENSG00000162174:ENSG00000162298:ENSG00000173599:ENSG00000173621:ENSG00000175567:ENSG00000175564:ENSG00000062282:ENSG00000137492:ENSG00000149256:ENSG00000137693:ENSG00000137714:ENSG00000149292:ENSG00000076053:ENSG00000076043:ENSG00000166250:ENSG00000023171:ENSG00000109832:ENSG00000064309:ENSG00000183715:ENSG00000082805:ENSG00000111262:ENSG00000111671:ENSG00000111679:ENSG00000126749:ENSG00000111684:ENSG00000182326:ENSG00000134531:ENSG00000123094:ENSG00000123338:ENSG00000123360:ENSG00000127314:ENSG00000235162:ENSG00000074590:ENSG00000135094:ENSG00000174989:ENSG00000111725:ENSG00000122966:ENSG00000139714:ENSG00000150990:ENSG00000184992:ENSG00000177084:ENSG00000176915:ENSG00000102743:ENSG00000215475:ENSG00000136167:ENSG00000102531:ENSG00000123179:ENSG00000184226:ENSG00000188243:ENSG00000118939:ENSG00000136153:ENSG00000178695:ENSG00000088451:ENSG00000134901:ENSG00000139826:ENSG00000102524:ENSG00000100889:ENSG00000168952:ENSG00000176165:ENSG00000186960:ENSG00000092140:ENSG00000100473:ENSG00000129493:ENSG00000150527:ENSG00000165379:ENSG00000179476:ENSG00000179454:ENSG00000185246:ENSG00000139926:ENSG00000180998:ENSG00000197930:ENSG00000006432:ENSG00000119707:ENSG00000133961:ENSG00000165417:ENSG00000100433:ENSG00000165934:ENSG00000153485:ENSG00000170113:ENSG00000169918:ENSG00000021776:ENSG00000198146:ENSG00000174197:ENSG00000243789:ENSG00000197535:ENSG00000181827:ENSG00000140262:ENSG00000103710:ENSG00000117899:ENSG00000140406:ENSG00000140600:ENSG00000140511:ENSG00000140471:ENSG00000183454:ENSG00000179889:ENSG00000085721:ENSG00000157106:ENSG00000102879:ENSG00000180035:ENSG00000180096:ENSG00000179965:ENSG00000140682:ENSG00000196470:ENSG00000102921:ENSG00000072736:ENSG00000103064:ENSG00000172137:ENSG00000158717:ENSG00000127804:ENSG00000197417:ENSG00000072849:ENSG00000109047:ENSG00000181350:ENSG00000166484:ENSG00000166482:ENSG00000128482:ENSG00000142494:ENSG00000067191:ENSG00000141750:ENSG00000068079:ENSG00000108828:ENSG00000108830:ENSG00000073670:ENSG00000182963:ENSG00000225190:ENSG00000198933:ENSG00000011258:ENSG00000121060:ENSG00000108423:ENSG00000108443:ENSG00000189050:ENSG00000198265:ENSG00000154217:ENSG00000180616:ENSG00000141540:ENSG00000141576:ENSG00000185262:ENSG00000141577:ENSG00000167302:ENSG00000185504:ENSG00000182446:ENSG00000101608:ENSG00000154845:ENSG00000154889:ENSG00000141401:ENSG00000168234:ENSG00000141431:ENSG0000014146

						9:ENSG00000152223:ENSG00000101638:ENSG00000081913:ENSG00000197971:ENSG00000071564:ENSG00000205744:ENSG00000104833:ENSG00000171105:ENSG00000104883:ENSG00000065989:ENSG00000104957:ENSG00000132000:ENSG00000132017:ENSG00000132005:ENSG00000123146:ENSG00000123136:ENSG00000099308:ENSG00000124302:ENSG00000262484:ENSG00000269190:ENSG00000167625:ENSG00000079462:ENSG00000167619:ENSG00000105429:ENSG00000105427:ENSG00000187244:ENSG00000185453:ENSG00000105483:ENSG00000105464:ENSG00000105447:ENSG00000196214:ENSG00000198464:ENSG00000167766:ENSG00000213020:ENSG00000152467:ENSG00000115756:ENSG00000115761:ENSG00000197872:ENSG00000143867:ENSG00000163803:ENSG00000171103:ENSG00000171094:ENSG00000119866:ENSG00000162928:ENSG00000144034:ENSG00000144048:ENSG00000114978:ENSG00000065911:ENSG00000115604:ENSG00000153214:ENSG00000115963:ENSG00000153250:ENSG00000153253:ENSG00000118260:ENSG00000115457:ENSG00000115461:ENSG00000018280:ENSG00000144579:ENSG00000144485:ENSG00000132326:ENSG00000162804:ENSG00000088876:ENSG00000132646:ENSG00000188559:ENSG00000125966:ENSG00000124134:ENSG00000124107:ENSG00000124228:ENSG00000124201:ENSG00000125520:ENSG00000198276:ENSG00000155313:ENSG00000154642:ENSG00000183628:ENSG00000184792:ENSG00000100342:ENSG00000100321:ENSG00000128285:ENSG00000198951:ENSG00000177096:ENSG00000242247:ENSG00000138944:ENSG00000077942:ENSG00000186951:ENSG00000075234:ENSG00000205593:ENSG00000130489:ENSG00000025708:ENSG00000177989:ENSG00000008735:ENSG00000251322:ENSG00000070950:ENSG00000144560:ENSG00000163517:ENSG00000163513:ENSG00000170293:ENSG00000170011:ENSG00000168032:ENSG00000182983:ENSG00000214706:ENSG00000186792:ENSG00000114378:ENSG00000068001:ENSG00000068028:ENSG00000004838:ENSG00000114395:ENSG00000126062:ENSG00000180376:ENSG00000163946:ENSG00000114861:ENSG00000144736:ENSG00000163320:ENSG00000175105:ENSG00000179021:ENSG00000184500:ENSG00000169379:ENSG00000240891:ENSG00000163607:ENSG00000163608:ENSG00000144843:ENSG00000172780:ENSG00000240682:ENSG00000240303:ENSG00000113971:ENSG00000154917:ENSG00000138231:ENSG00000114098:ENSG00000158186:ENSG00000184432:ENSG00000155903:ENSG00000175066:ENSG00000114127:ENSG00000114698:ENSG00000163762:ENSG00000075420:ENSG00000114279:ENSG00000041802:ENSG00000174145:ENSG00000169019:ENSG00000145246:ENSG00000109171:ENSG00000145248:ENSG00000080493:ENSG00000169429:ENSG00000163739:ENSG00000173085:ENSG00000205403:ENSG00000109466:ENSG00000215218:ENSG00000113100:ENSG00000164190:ENSG00000152669:ENSG00000067248:ENSG00000039123:ENSG00000086200:ENSG00000164331:ENSG00000164338:ENSG00000164180:ENSG00000181751:ENSG00000173926:ENSG00000145794:ENSG00000240184:ENSG00000242419:ENSG00000240764:ENSG00000131504:ENSG00000164620:ENSG00000197948:ENSG00000120318:ENSG00000135074:ENSG00000113272:ENSG00000182132:ENSG00000074317:ENSG00000175766:ENSG00000249109:ENSG00000131446:ENSG00000146112:ENSG00000137404:ENSG00000137337:ENSG00000137331:ENSG00000244731:ENSG00000235863:ENSG00000124762:ENSG00000124772:ENSG00000164530:ENSG00000112245:ENSG00000172461:ENSG00000132429:ENSG00000198945:ENSG00000135525:ENSG00000118503:ENSG00000112406:ENSG00000120278:ENSG00000112096:ENSG00000026297:ENSG00000186340:ENSG00000184786:ENSG00000130023:ENSG00000106113:ENSG00000164600:ENSG00000168303:ENSG00000152926:ENSG00000213462:ENSG00000126522:ENSG00000086730:ENSG00000177679:ENSG00000127920:ENSG00000105829:ENSG00000160813:ENSG00000166924:ENSG00000172209:ENSG00000091704:ENSG00000122786:ENSG00000182158:ENSG00000181444:ENSG00000196329:ENSG00000214106:ENSG00000146918:ENSG00000117868:ENSG00000154319:ENSG00000168546:ENSG00000120910:ENSG00000165061:ENSG00000023287:ENSG00000254087:ENSG00000035681:ENSG00000121039:ENSG00000104267:ENSG00000104327:ENSG00000164949:ENSG00000120526:ENSG00000120533:ENSG00000131773:ENSG00000080608:ENSG00000213694:ENSG00000165023:ENSG00000127080:ENSG00000130958:ENSG00000136925:ENSG00000136826:ENSG00000167081:ENSG00000196152:ENSG00000136840:ENSG00000148411:ENSG00000198113
DEG.twoside	Brain_Frontal_Cortex_BA9	8222	485	4.13805742848741e-13	2.2345510113832e-11	ENSG00000158286:ENSG00000097021:ENSG00000120942:ENSG00000162458:ENSG00000065526:ENSG00000183888:ENSG00000158748:ENSG00000157978:ENSG00000134668:ENSG00000084652:ENSG00000121904:ENSG00000177181:ENSG00000066056:ENSG00000066322:ENSG00000198198:ENSG00000162367:ENSG00000117133:ENSG00000117155:ENSG00000162642:ENSG00000142867:ENSG00000153904:ENSG00000197147:ENSG00000069712:ENSG00000117528:ENSG00000162694:ENSG00000117543:ENSG00000118733:ENSG00000134215:ENSG00000116396:ENSG00000162775:ENSG00000168679:ENSG00000116455:ENSG00000143457:ENSG00000163131:ENSG00000143387:ENSG00000143437:ENSG00000143379:ENSG00000143418:ENSG00000143412:ENSG00000143409:ENSG00000143363:ENSG00000163154:ENSG00000143537:ENSG00000176444:ENSG00000125459:ENSG00000163374:ENSG00000132676:ENSG00000116580:ENSG00000132718:ENSG00000143622:ENSG00000132680:ENSG00000160789:ENSG00000160783:ENSG00000160781:ENSG00000163472:ENSG00000198715:ENSG00000187800:ENSG00000143226:ENSG00000162746:ENSG00000186283:ENSG00000057252:ENSG00000134376:ENSG00000122176:ENSG00000163050:ENSG00000162885:ENSG00000116962:ENSG00000162714:ENSG00000107929:ENSG00000134463:ENSG00000148468:ENSG00000165997:ENSG00000165476:ENSG00000075073:ENSG00000107736:ENSG00000107738:ENSG00000148604:ENSG00000138134:ENSG00000107796:ENSG00000026103:ENSG00000059573:ENSG00000197430:ENSG00000095587:ENSG00000120057:ENSG00000165832:ENSG00000198873:ENSG00000184545:ENSG00000166478:ENSG00000187486:ENSG00000006071:ENSG00000129151:ENSG00000019505:ENSG00000149150:ENSG00000162174:ENSG00000162298:ENSG00000173653:ENSG00000173599:ENSG00000175567:ENSG00000175564:ENSG00000062282:ENSG00000149256:ENSG00000137693:ENSG00000149313:ENSG00000149292:ENSG00000076053:ENSG00000076043:ENSG00000166250:ENSG00000023171:ENSG00000109832:ENSG00000064309:ENSG00000183715:ENSG00000082805:ENSG00000111262:ENSG00000111671:ENSG00000111679:ENSG00000126749:ENSG00000111684:ENSG00000182326:ENSG00000134531:ENSG00000123094:ENSG00000123338:ENSG00000123360:ENSG00000127314:ENSG00000166130:ENSG00

	000235162:ENSG00000074590:ENSG00000135094:ENSG00000174989:ENSG00000111725:ENSG00000122966:ENSG00000139714:ENSG00000188735:ENSG00000150990:ENSG00000184992:ENSG00000177084:ENSG00000176915:ENSG00000127863:ENSG00000102743:ENSG00000215475:ENSG00000136167:ENSG00000102531:ENSG00000123179:ENSG00000184226:ENSG00000188243:ENSG00000118939:ENSG00000178695:ENSG00000088451:ENSG00000134901:ENSG00000100889:ENSG00000168952:ENSG00000176165:ENSG00000186960:ENSG00000100473:ENSG00000129493:ENSG00000150527:ENSG00000165379:ENSG00000179476:ENSG00000185246:ENSG00000139926:ENSG00000180998:ENSG00000006432:ENSG00000119707:ENSG00000100767:ENSG00000133961:ENSG00000100433:ENSG00000165934:ENSG00000170113:ENSG00000169918:ENSG00000021776:ENSG00000174197:ENSG00000243789:ENSG00000197535:ENSG00000181827:ENSG00000140262:ENSG00000103710:ENSG00000117899:ENSG00000140600:ENSG00000140511:ENSG00000140471:ENSG00000183454:ENSG00000179889:ENSG00000085721:ENSG00000157106:ENSG00000102879:ENSG00000180096:ENSG00000179965:ENSG00000140682:ENSG00000102921:ENSG00000072736:ENSG00000103064:ENSG00000172137:ENSG00000158717:ENSG00000127804:ENSG00000197417:ENSG00000072849:ENSG00000109047:ENSG00000181350:ENSG00000166484:ENSG00000166482:ENSG00000128482:ENSG00000142494:ENSG00000067191:ENSG00000141750:ENSG00000068079:ENSG00000108828:ENSG00000108830:ENSG00000073670:ENSG00000182963:ENSG00000225190:ENSG00000198933:ENSG00000011258:ENSG00000121060:ENSG00000108423:ENSG00000189050:ENSG00000198265:ENSG00000154217:ENSG00000180616:ENSG00000141540:ENSG00000141576:ENSG00000185262:ENSG00000141577:ENSG00000167302:ENSG00000185504:ENSG00000182446:ENSG00000101608:ENSG00000154845:ENSG00000134265:ENSG00000154889:ENSG00000141401:ENSG00000168234:ENSG00000141431:ENSG00000141469:ENSG00000152223:ENSG00000101638:ENSG00000081913:ENSG00000197971:ENSG00000071564:ENSG00000205744:ENSG00000104833:ENSG00000171105:ENSG00000104883:ENSG00000065989:ENSG00000104957:ENSG00000132017:ENSG00000132005:ENSG00000123146:ENSG00000123136:ENSG00000099308:ENSG00000124302:ENSG00000262484:ENSG00000269190:ENSG00000167625:ENSG00000105722:ENSG00000079462:ENSG00000167619:ENSG00000105429:ENSG00000105427:ENSG00000079435:ENSG00000187244:ENSG00000185453:ENSG00000105483:ENSG00000105464:ENSG00000105447:ENSG00000196214:ENSG00000198464:ENSG00000167766:ENSG00000152467:ENSG00000115756:ENSG00000115761:ENSG00000197872:ENSG00000163803:ENSG00000171094:ENSG00000119866:ENSG00000162928:ENSG00000162929:ENSG00000144034:ENSG00000144048:ENSG00000163170:ENSG00000114978:ENSG00000065911:ENSG00000144218:ENSG00000115604:ENSG00000153214:ENSG00000115963:ENSG00000144283:ENSG00000153250:ENSG00000153253:ENSG00000118260:ENSG00000115457:ENSG00000115461:ENSG00000018280:ENSG00000144579:ENSG00000135912:ENSG00000144485:ENSG00000132326:ENSG00000162804:ENSG00000088876:ENSG00000101290:ENSG00000188559:ENSG00000125966:ENSG00000198959:ENSG00000124134:ENSG00000124107:ENSG00000124228:ENSG00000124201:ENSG00000125520:ENSG00000198276:ENSG00000154642:ENSG00000183628:ENSG00000184792:ENSG00000100342:ENSG00000100321:ENSG00000128285:ENSG00000198951:ENSG00000177096:ENSG00000100197:ENSG00000242247:ENSG00000138944:ENSG00000077942:ENSG00000186951:ENSG00000075234:ENSG00000196576:ENSG00000205593:ENSG00000130489:ENSG00000025708:ENSG00000177989:ENSG00000205560:ENSG00000008735:ENSG00000100299:ENSG00000251322:ENSG00000070950:ENSG00000144560:ENSG00000163517:ENSG00000163513:ENSG00000170293:ENSG00000170011:ENSG00000168032:ENSG00000177873:ENSG00000182983:ENSG00000214706:ENSG00000186792:ENSG00000114378:ENSG00000068001:ENSG00000068028:ENSG00000114395:ENSG00000126062:ENSG00000180376:ENSG00000163946:ENSG00000114861:ENSG00000144736:ENSG00000175105:ENSG00000184500:ENSG00000169379:ENSG00000240891:ENSG00000163607:ENSG00000144843:ENSG00000172780:ENSG00000240682:ENSG00000240303:ENSG00000113971:ENSG00000154917:ENSG00000138231:ENSG00000158186:ENSG00000184432:ENSG00000175066:ENSG00000114127:ENSG00000114698:ENSG00000169282:ENSG00000075420:ENSG00000073803:ENSG00000114279:ENSG00000041802:ENSG00000174145:ENSG00000145246:ENSG00000109171:ENSG00000145248:ENSG00000080493:ENSG00000169429:ENSG00000163739:ENSG00000173085:ENSG00000185477:ENSG00000151247:ENSG00000205403:ENSG00000109466:ENSG00000215218:ENSG00000113100:ENSG00000164190:ENSG00000067248:ENSG00000039123:ENSG00000164331:ENSG00000164338:ENSG00000164180:ENSG00000175471:ENSG00000181751:ENSG00000173926:ENSG00000145794:ENSG00000240184:ENSG00000242419:ENSG00000240764:ENSG00000131504:ENSG00000164620:ENSG00000197948:ENSG00000120318:ENSG00000113721:ENSG00000135074:ENSG00000113272:ENSG00000155858:ENSG00000182132:ENSG00000074317:ENSG00000175766:ENSG00000249109:ENSG00000131446:ENSG00000146112:ENSG00000137404:ENSG00000137337:ENSG00000137331:ENSG00000204344:ENSG00000244731:ENSG00000235863:ENSG00000227057:ENSG00000237441:ENSG00000124762:ENSG00000124772:ENSG00000164530:ENSG00000118432:ENSG00000172461:ENSG00000132429:ENSG00000198945:ENSG00000135525:ENSG00000118503:ENSG00000112406:ENSG00000120278:ENSG00000186340:ENSG00000184786:ENSG00000130023:ENSG00000106113:ENSG00000164600:ENSG00000152926:ENSG00000213462:ENSG00000126522:ENSG00000086730:ENSG00000177679:ENSG00000085563:ENSG00000127920:ENSG00000105829:ENSG00000085514:ENSG00000160813:ENSG00000166924:ENSG00000005249:ENSG00000172209:ENSG00000091704:ENSG00000122786:ENSG00000182158:ENSG00000181444:ENSG00000196329:ENSG00000214106:ENSG00000146918:ENSG00000117868:ENSG00000154319:ENSG00000168546:ENSG00000120910:ENSG00000165061:ENSG00000023287:ENSG00000254087:ENSG00000035681:ENSG00000121039:ENSG00000104267:ENSG00000104327:ENSG00000164949:ENSG00000120526:ENSG00000129295:ENSG00000131773:ENSG00000080608:ENSG00000213694:ENSG00000165023:ENSG00000127080:ENSG00000130958:ENSG00000136925:ENSG00000136826:ENSG00000167081:ENSG00000196152:ENSG00000136840:ENSG00000148308:ENSG00000170835:ENSG00000148411:ENSG00000198113

DEG.twoside	Brain_Hippocampus	9541	610	7.73257064321815e-27	4.1755881473378e-25	ENSG00000158286:ENSG00000116237:ENSG00000158292:ENSG00000097021:ENSG00000171824:ENSG00000198793:ENSG00000120942:ENSG00000162458:ENSG00000065526:ENSG00000116809:ENSG00000183888:ENSG00000204178:ENSG00000157978:ENSG00000134668:ENSG00000084652:ENSG00000160051:ENSG00000160094:ENSG00000121904:ENSG00000127603:ENSG00000177181:ENSG00000179178:ENSG00000066056:ENSG00000117399:ENSG00000159479:ENSG00000198198:ENSG00000162367:ENSG00000117862:ENSG00000198841:ENSG00000134709:ENSG00000162604:ENSG00000162616:ENSG00000117114:ENSG00000117133:ENSG00000142867:ENSG00000153904:ENSG00000197147:ENSG00000171488:ENSG00000117528:ENSG00000162694:ENSG00000117543:ENSG00000118733:ENSG00000162775:ENSG00000168679:ENSG00000085465:ENSG00000116455:ENSG00000163349:ENSG00000197323:ENSG00000213281:ENSG00000052723:ENSG00000143457:ENSG00000163131:ENSG00000143387:ENSG00000143437:ENSG00000143379:ENSG00000143412:ENSG00000143409:ENSG00000143363:ENSG00000143434:ENSG00000163154:ENSG00000163156:ENSG00000163155:ENSG00000143537:ENSG00000160767:ENSG00000116521:ENSG00000176444:ENSG00000143630:ENSG00000160752:ENSG00000160753:ENSG00000116539:ENSG00000125459:ENSG00000163374:ENSG00000132676:ENSG00000116580:ENSG00000132718:ENSG00000143622:ENSG00000132680:ENSG00000160789:ENSG00000160783:ENSG00000160781:ENSG00000163472:ENSG00000198715:ENSG00000187800:ENSG00000132694:ENSG00000143226:ENSG00000120341:ENSG00000186283:ENSG00000143322:ENSG00000057252:ENSG00000134376:ENSG00000122176:ENSG00000182004:ENSG00000143801:ENSG00000163050:ENSG00000162885:ENSG00000116962:ENSG00000162714:ENSG00000107929:ENSG00000134463:ENSG00000148468:ENSG00000165997:ENSG00000108094:ENSG00000188234:ENSG00000165476:ENSG00000107736:ENSG00000214688:ENSG00000107738:ENSG00000138134:ENSG00000107796:ENSG00000026103:ENSG00000165338:ENSG00000059573:ENSG00000197430:ENSG00000119927:ENSG00000198873:ENSG00000184545:ENSG00000166311:ENSG00000205339:ENSG00000166478:ENSG00000166483:ENSG00000198730:ENSG00000006071:ENSG00000129151:ENSG00000019505:ENSG00000149150:ENSG00000162174:ENSG00000162300:ENSG00000149823:ENSG00000174276:ENSG00000162298:ENSG00000149792:ENSG00000173653:ENSG00000175567:ENSG00000062282:ENSG00000137492:ENSG00000137693:ENSG00000137714:ENSG00000183644:ENSG00000149292:ENSG00000076053:ENSG00000076043:ENSG00000154127:ENSG00000166250:ENSG00000023171:ENSG00000183715:ENSG00000082805:ENSG00000139180:ENSG00000111262:ENSG00000111671:ENSG00000111679:ENSG00000126749:ENSG00000111684:ENSG00000182326:ENSG00000134531:ENSG00000123094:ENSG00000050426:ENSG00000123338:ENSG00000127314:ENSG00000166130:ENSG00000235162:ENSG00000135094:ENSG00000174989:ENSG00000111725:ENSG00000122966:ENSG00000188735:ENSG00000150990:ENSG00000177169:ENSG00000187848:ENSG00000177084:ENSG00000176915:ENSG00000127863:ENSG00000102743:ENSG00000136167:ENSG00000102445:ENSG00000102531:ENSG00000123179:ENSG00000102753:ENSG00000136104:ENSG00000184226:ENSG00000188243:ENSG00000118939:ENSG00000136153:ENSG00000088451:ENSG00000065150:ENSG00000134901:ENSG00000139826:ENSG00000102524:ENSG00000100889:ENSG00000176165:ENSG00000186960:ENSG00000092140:ENSG00000092108:ENSG00000100473:ENSG00000150527:ENSG00000165355:ENSG00000165379:ENSG00000179476:ENSG00000179454:ENSG00000185246:ENSG00000139926:ENSG00000180998:ENSG00000197930:ENSG00000119599:ENSG00000165861:ENSG00000119707:ENSG00000100767:ENSG00000133961:ENSG00000165417:ENSG00000100433:ENSG00000165934:ENSG00000153485:ENSG00000140157:ENSG00000170113:ENSG00000128731:ENSG00000169918:ENSG00000021776:ENSG00000198146:ENSG00000174197:ENSG00000137802:ENSG00000243708:ENSG00000243789:ENSG00000197535:ENSG00000181827:ENSG00000103707:ENSG00000186198:ENSG00000136378:ENSG00000103888:ENSG00000117899:ENSG00000140406:ENSG00000156206:ENSG00000140600:ENSG00000140511:ENSG00000140471:ENSG00000183454:ENSG00000179889:ENSG00000157045:ENSG00000085721:ENSG00000157106:ENSG00000180096:ENSG00000179958:ENSG00000140691:ENSG00000140682:ENSG00000197302:ENSG00000102910:ENSG00000196470:ENSG00000102921:ENSG00000005194:ENSG00000102978:ENSG00000072736:ENSG00000103066:ENSG00000103064:ENSG00000103061:ENSG00000132600:ENSG00000158717:ENSG00000167522:ENSG00000197912:ENSG00000127804:ENSG00000197417:ENSG00000005100:ENSG00000072849:ENSG00000233670:ENSG00000007174:ENSG00000166484:ENSG00000166482:ENSG00000128482:ENSG00000180638:ENSG00000067191:ENSG00000068079:ENSG00000108828:ENSG00000108830:ENSG00000012048:ENSG00000184988:ENSG00000073670:ENSG00000182963:ENSG00000225190:ENSG00000141279:ENSG00000108424:ENSG00000011258:ENSG00000011260:ENSG00000121060:ENSG00000108423:ENSG00000108443:ENSG00000189050:ENSG00000198265:ENSG00000197170:ENSG00000180616:ENSG00000141540:ENSG00000196169:ENSG00000141576:ENSG00000185262:ENSG00000141577:ENSG00000167302:ENSG00000185504:ENSG00000182446:ENSG00000101608:ENSG00000017797:ENSG00000154845:ENSG00000154889:ENSG00000141401:ENSG00000168234:ENSG00000141384:ENSG00000141469:ENSG00000152223:ENSG00000101638:ENSG00000081913:ENSG00000130856:ENSG00000197971:ENSG00000071564:ENSG00000141985:ENSG00000167670:ENSG00000205744:ENSG00000104833:ENSG00000171105:ENSG00000104880:ENSG00000167775:ENSG00000079999:ENSG00000180739:ENSG00000104957:ENSG00000132017:ENSG00000132005:ENSG00000123146:ENSG00000123136:ENSG00000127527:ENSG00000099308:ENSG00000237440:ENSG00000124302:ENSG00000104835:ENSG00000128626:ENSG00000269190:ENSG00000028277:ENSG00000160570:ENSG00000167625:ENSG00000105722:ENSG00000079432:ENSG00000167619:ENSG00000105427:ENSG00000187244:ENSG00000105483:ENSG00000105447:ENSG00000063127:ENSG00000142544:ENSG00000105383:ENSG00000196214:ENSG00000198464:ENSG00000167766:ENSG00000213020:ENSG00000115705:ENSG00000115761:ENSG00000134323:ENSG00000197872:ENSG00000143867:ENSG00000055917:ENSG00000171103:ENSG00000119866:ENSG00000162928:ENSG00000082898:ENSG00000144034:ENSG00000144048:ENSG00000114978:ENSG00000065911:ENSG00000188687:ENSG00000114993:ENSG00000115602:ENSG00000115604:ENSG00000153107:ENSG00000153208:ENSG00000153214:ENSG00000115963:ENSG00000144283:ENSG00000153250:ENSG00000153253:ENSG0

						0000118260:ENSG00000197713:ENSG00000115457:ENSG00000018280:ENSG00000144579:ENSG00000135912:ENSG00000123977:ENSG00000144485:ENSG00000132326:ENSG00000162804:ENSG00000196209:ENSG00000088876:ENSG00000125779:ENSG00000132646:ENSG00000188559:ENSG00000118705:ENSG00000198959:ENSG00000124107:ENSG00000124228:ENSG00000124201:ENSG00000125520:ENSG00000198276:ENSG00000155313:ENSG00000157617:ENSG00000160190:ENSG00000100220:ENSG00000128335:ENSG00000100342:ENSG00000100321:ENSG00000128285:ENSG00000198951:ENSG00000177096:ENSG00000100197:ENSG00000242247:ENSG00000138944:ENSG00000077942:ENSG00000186951:ENSG00000205643:ENSG00000075234:ENSG00000100416:ENSG00000196576:ENSG00000100258:ENSG00000025770:ENSG00000130489:ENSG00000025708:ENSG00000177989:ENSG00000205560:ENSG00000100288:ENSG00000008735:ENSG00000100299:ENSG00000070950:ENSG00000134077:ENSG00000144560:ENSG00000144712:ENSG00000163517:ENSG00000163513:ENSG00000170293:ENSG00000170011:ENSG00000177873:ENSG00000182606:ENSG00000182983:ENSG00000214706:ENSG00000243477:ENSG00000114378:ENSG00000068001:ENSG00000068028:ENSG00000004838:ENSG00000114388:ENSG00000114395:ENSG00000126062:ENSG00000007402:ENSG00000180376:ENSG00000163946:ENSG00000114861:ENSG00000144736:ENSG00000163320:ENSG00000175105:ENSG00000179021:ENSG00000184500:ENSG00000169379:ENSG00000114446:ENSG00000163607:ENSG00000163608:ENSG00000144843:ENSG00000172780:ENSG00000240682:ENSG00000034533:ENSG00000240303:ENSG00000113971:ENSG00000081307:ENSG00000144867:ENSG00000154917:ENSG00000158163:ENSG00000138231:ENSG00000114098:ENSG00000175110:ENSG00000184432:ENSG00000155903:ENSG00000175066:ENSG00000114127:ENSG00000114698:ENSG00000169282:ENSG00000075420:ENSG00000161202:ENSG00000161204:ENSG00000114279:ENSG00000145014:ENSG00000041802:ENSG00000163975:ENSG00000013288:ENSG00000169019:ENSG00000145246:ENSG00000109171:ENSG00000169429:ENSG00000163739:ENSG00000173085:ENSG00000185477:ENSG00000205403:ENSG00000164128:ENSG00000109466:ENSG00000129187:ENSG00000215218:ENSG00000113100:ENSG00000113361:ENSG00000113360:ENSG00000164190:ENSG00000132357:ENSG00000067248:ENSG00000039123:ENSG00000086200:ENSG00000164331:ENSG00000164338:ENSG00000164180:ENSG00000173926:ENSG00000145794:ENSG00000240764:ENSG00000131504:ENSG00000171720:ENSG00000197948:ENSG00000120318:ENSG00000113721:ENSG00000135074:ENSG00000113272:ENSG00000155858:ENSG00000182132:ENSG00000074317:ENSG00000048140:ENSG00000249109:ENSG00000131446:ENSG00000196670:ENSG00000079691:ENSG00000146112:ENSG00000137404:ENSG00000137337:ENSG00000137331:ENSG00000204344:ENSG00000244731:ENSG00000223501:ENSG00000235863:ENSG00000227057:ENSG00000237441:ENSG00000231925:ENSG00000124762:ENSG00000198663:ENSG00000112245:ENSG00000172461:ENSG00000198945:ENSG00000135525:ENSG00000112357:ENSG00000118503:ENSG00000112406:ENSG00000120278:ENSG00000112096:ENSG00000213066:ENSG00000186340:ENSG00000184786:ENSG00000130023:ENSG00000122644:ENSG00000153814:ENSG00000164600:ENSG00000168303:ENSG00000152926:ENSG00000213462:ENSG00000126522:ENSG00000165215:ENSG00000049541:ENSG00000135218:ENSG00000085563:ENSG00000127920:ENSG00000105829:ENSG00000085514:ENSG00000146834:ENSG00000160813:ENSG00000166924:ENSG00000172209:ENSG00000091704:ENSG00000106484:ENSG00000158623:ENSG00000131558:ENSG00000122786:ENSG00000182158:ENSG00000181444:ENSG00000196329:ENSG00000214106:ENSG00000146918:ENSG00000117868:ENSG00000184608:ENSG00000154319:ENSG00000120910:ENSG00000129691:ENSG00000165061:ENSG00000254087:ENSG00000035681:ENSG00000121039:ENSG00000104267:ENSG00000104327:ENSG00000164949:ENSG00000120526:ENSG00000120533:ENSG00000131773:ENSG00000196378:ENSG00000080608:ENSG00000213694:ENSG00000165023:ENSG00000127080:ENSG00000130958:ENSG00000136925:ENSG00000136936:ENSG00000136826:ENSG00000070159:ENSG00000167081:ENSG00000196152:ENSG00000148356:ENSG00000136840:ENSG00000171169:ENSG00000148339:ENSG00000148335:ENSG00000148308:ENSG00000170835:ENSG00000198113
DEG.twoside	Brain_Hypothalamus	8506	517	9.86352891296313e-17	5.32630561300009e-15	ENSG00000116237:ENSG00000158292:ENSG00000097021:ENSG00000198793:ENSG00000120942:ENSG00000162458:ENSG00000065526:ENSG00000116809:ENSG00000183888:ENSG00000158816:ENSG00000187942:ENSG00000157978:ENSG00000084652:ENSG00000160094:ENSG00000121904:ENSG00000177181:ENSG00000066056:ENSG00000198198:ENSG00000162367:ENSG00000162390:ENSG00000116745:ENSG00000117114:ENSG00000117133:ENSG00000142867:ENSG00000153904:ENSG00000171488:ENSG00000069712:ENSG00000117528:ENSG00000118733:ENSG00000134215:ENSG00000116396:ENSG00000162775:ENSG00000168679:ENSG00000116455:ENSG00000197323:ENSG00000163131:ENSG00000143387:ENSG00000143437:ENSG00000143379:ENSG00000143412:ENSG00000143409:ENSG00000143363:ENSG00000163156:ENSG00000163354:ENSG00000143537:ENSG00000176444:ENSG00000143630:ENSG00000116539:ENSG00000125459:ENSG00000163374:ENSG00000116580:ENSG00000132718:ENSG00000143622:ENSG00000132680:ENSG00000116584:ENSG00000160789:ENSG00000160783:ENSG00000160781:ENSG00000163472:ENSG00000198715:ENSG00000187800:ENSG00000160838:ENSG00000171786:ENSG00000120341:ENSG00000186283:ENSG00000143322:ENSG00000057252:ENSG00000134376:ENSG00000122176:ENSG00000163050:ENSG00000162885:ENSG00000116962:ENSG00000162714:ENSG00000107929:ENSG00000134463:ENSG00000148468:ENSG00000165997:ENSG00000165476:ENSG00000214688:ENSG00000107738:ENSG00000148604:ENSG00000107796:ENSG00000026103:ENSG00000165338:ENSG00000059573:ENSG00000197430:ENSG00000095587:ENSG00000119927:ENSG00000198873:ENSG00000184545:ENSG00000166311:ENSG00000166478:ENSG00000166483:ENSG00000198730:ENSG00000006071:ENSG00000129151:ENSG00000019505:ENSG00000149150:ENSG00000124935:ENSG00000162174:ENSG00000162300:ENSG00000149823:ENSG00000162298:ENSG00000173653:ENSG00000175567:ENSG00000175564:ENSG00000062282:ENSG00000137492:ENSG00000149256:ENSG00000137672:ENSG00000137693:ENSG00000149313:ENSG00000183644:ENSG00000149292:ENSG00000076053:ENSG00000076043:ENSG00000109943:ENSG00000166250:ENSG00000109832:ENSG00000064309:ENSG00000183715:ENSG00000111671:ENSG00000010626:ENSG00

000111679:ENSG00000126749:ENSG00000111684:ENSG00000182326:ENSG00000134531:ENSG00000050426:ENSG00000123338:ENSG00000127314:ENSG000001
66130:ENSG00000074590:ENSG00000135094:ENSG00000174989:ENSG00000111725:ENSG00000122966:ENSG00000188735:ENSG00000150990:ENSG0000018499
2:ENSG00000177169:ENSG00000177084:ENSG00000176915:ENSG00000127863:ENSG00000102743:ENSG00000215475:ENSG00000136167:ENSG00000123179:EN
SG00000136104:ENSG00000184226:ENSG00000118939:ENSG00000136153:ENSG00000088451:ENSG00000065150:ENSG00000134901:ENSG00000139826:ENSG0
0000102524:ENSG00000100889:ENSG00000168952:ENSG00000092140:ENSG00000150527:ENSG00000165379:ENSG00000179476:ENSG00000185246:ENSG00000
139926:ENSG00000180998:ENSG00000197930:ENSG00000184302:ENSG00000006432:ENSG00000119599:ENSG00000165861:ENSG00000119707:ENSG000001007
67:ENSG00000133961:ENSG00000100433:ENSG00000165934:ENSG00000254656:ENSG00000170113:ENSG00000169918:ENSG00000021776:ENSG00000198146:E
NSG00000174197:ENSG00000197535:ENSG00000181827:ENSG00000140262:ENSG00000103707:ENSG00000136378:ENSG00000140406:ENSG00000156206:ENSG
00000140600:ENSG00000140511:ENSG00000140471:ENSG00000179889:ENSG00000157045:ENSG00000085721:ENSG00000157106:ENSG00000180096:ENSG0000
0179958:ENSG00000140682:ENSG00000102910:ENSG00000196470:ENSG00000102921:ENSG00000072736:ENSG00000103066:ENSG00000103064:ENSG00000172
137:ENSG00000185669:ENSG00000158717:ENSG00000167522:ENSG00000127804:ENSG00000197417:ENSG00000005100:ENSG00000072849:ENSG00000233670:
ENSG00000007174:ENSG00000188522:ENSG00000166484:ENSG00000166482:ENSG00000128482:ENSG00000180638:ENSG00000067191:ENSG00000068079:ENS
G00000108830:ENSG00000012048:ENSG00000184988:ENSG00000073670:ENSG00000182963:ENSG00000225190:ENSG00000011258:ENSG00000011260:ENSG00
000121060:ENSG00000108423:ENSG00000108443:ENSG00000189050:ENSG00000198265:ENSG00000154217:ENSG00000153822:ENSG00000141540:ENSG000001
71595:ENSG00000196169:ENSG00000141576:ENSG00000185262:ENSG00000141577:ENSG00000167302:ENSG00000185504:ENSG00000182446:ENSG0000010160
8:ENSG00000017797:ENSG00000154845:ENSG00000154889:ENSG00000141401:ENSG00000168234:ENSG00000141384:ENSG00000141469:ENSG00000152223:EN
SG00000081913:ENSG00000197971:ENSG00000071564:ENSG00000141985:ENSG00000167670:ENSG00000205744:ENSG00000104833:ENSG00000171105:ENSG0
0000104880:ENSG00000180739:ENSG00000104957:ENSG00000132017:ENSG00000132005:ENSG00000123146:ENSG00000123136:ENSG00000124302:ENSG00000
128626:ENSG00000269190:ENSG00000167625:ENSG00000105722:ENSG00000079462:ENSG00000167619:ENSG00000105427:ENSG00000187244:ENSG000001854
53:ENSG00000105483:ENSG00000105464:ENSG00000105447:ENSG00000063127:ENSG00000105383:ENSG00000196214:ENSG00000198464:ENSG00000167766:E
NSG00000152467:ENSG00000115756:ENSG00000115761:ENSG00000197872:ENSG00000055917:ENSG00000163803:ENSG00000171094:ENSG00000162928:ENSG
00000162929:ENSG00000144048:ENSG00000114978:ENSG00000065911:ENSG00000188687:ENSG00000115604:ENSG00000153107:ENSG00000153208:ENSG0000
0153214:ENSG00000163040:ENSG00000115963:ENSG00000144283:ENSG00000153250:ENSG00000153253:ENSG00000118260:ENSG00000115457:ENSG00000115
461:ENSG00000144579:ENSG00000123977:ENSG00000144485:ENSG00000132326:ENSG00000162804:ENSG00000196209:ENSG00000088876:ENSG00000132646:
ENSG00000101290:ENSG00000188559:ENSG00000125966:ENSG00000198959:ENSG00000124107:ENSG00000124228:ENSG00000124201:ENSG00000124089:ENS
G00000125520:ENSG00000198276:ENSG00000155313:ENSG00000157617:ENSG00000100342:ENSG00000100321:ENSG00000128285:ENSG00000198951:ENSG00
000177096:ENSG00000242247:ENSG00000138944:ENSG00000077942:ENSG00000186951:ENSG00000205643:ENSG00000075234:ENSG00000196576:ENSG000002
05593:ENSG00000130489:ENSG00000025708:ENSG00000177989:ENSG00000205560:ENSG00000008735:ENSG00000100299:ENSG00000070950:ENSG0000014456
0:ENSG00000163517:ENSG00000163576:ENSG00000163513:ENSG00000170293:ENSG00000170011:ENSG00000168032:ENSG00000182606:ENSG00000182983:EN
SG00000214706:ENSG00000114378:ENSG00000068001:ENSG00000068028:ENSG00000004838:ENSG00000114395:ENSG00000126062:ENSG00000007402:ENSG0
0000180376:ENSG00000163946:ENSG00000114861:ENSG00000144736:ENSG00000163320:ENSG00000175105:ENSG00000184500:ENSG00000169379:ENSG00000
240891:ENSG00000163607:ENSG00000163608:ENSG00000144843:ENSG00000172780:ENSG00000240682:ENSG00000114670:ENSG00000240303:ENSG000001139
71:ENSG00000154917:ENSG00000158163:ENSG00000138231:ENSG00000114098:ENSG00000158186:ENSG00000175110:ENSG00000184432:ENSG00000155903:E
NSG00000175066:ENSG00000114127:ENSG00000075420:ENSG00000161202:ENSG00000114279:ENSG00000041802:ENSG00000163975:ENSG00000152953:ENSG
00000013288:ENSG00000174145:ENSG00000145246:ENSG00000109171:ENSG00000145248:ENSG00000080493:ENSG00000169429:ENSG00000163739:ENSG0000
0173085:ENSG00000205403:ENSG00000109466:ENSG00000215218:ENSG00000113100:ENSG00000164190:ENSG00000171522:ENSG00000132357:ENSG00000016
082:ENSG00000067248:ENSG00000039123:ENSG00000086200:ENSG00000164338:ENSG00000164180:ENSG00000181751:ENSG00000145794:ENSG00000242419:
ENSG00000131504:ENSG00000171720:ENSG00000164620:ENSG00000197948:ENSG00000120318:ENSG00000113721:ENSG00000135074:ENSG00000113272:ENS
G00000182132:ENSG00000074317:ENSG00000175766:ENSG00000048140:ENSG00000131446:ENSG00000196670:ENSG00000078401:ENSG00000079691:ENSG00
000146112:ENSG00000137404:ENSG00000137337:ENSG00000137331:ENSG00000204344:ENSG00000244731:ENSG00000223501:ENSG00000235863:ENSG000002
27057:ENSG00000237441:ENSG00000231925:ENSG00000124762:ENSG00000137252:ENSG00000172461:ENSG00000198945:ENSG00000135525:ENSG0000011850
3:ENSG00000112406:ENSG00000120278:ENSG00000091831:ENSG00000186340:ENSG00000122644:ENSG00000106113:ENSG00000152926:ENSG00000213462:EN
SG00000126522:ENSG00000049541:ENSG00000177679:ENSG00000135218:ENSG00000085563:ENSG00000127920:ENSG00000085514:ENSG00000146834:ENSG0
0000160813:ENSG00000166924:ENSG00000091704:ENSG00000131558:ENSG00000122786:ENSG00000182158:ENSG00000181444:ENSG00000196329:ENSG00000
214106:ENSG00000146918:ENSG00000117868:ENSG00000184608:ENSG00000154319:ENSG00000168546:ENSG00000120910:ENSG00000147465:ENSG000001650

						61:ENSG00000254087:ENSG00000035681:ENSG00000121039:ENSG00000104267:ENSG00000104327:ENSG00000164949:ENSG00000120526:ENSG00000080608:ENSG00000044459:ENSG00000213694:ENSG00000165023:ENSG00000127080:ENSG00000130958:ENSG00000136925:ENSG00000136826:ENSG00000070159:ENSG00000196152:ENSG00000136840:ENSG00000171169:ENSG00000148335:ENSG00000148308:ENSG00000170835:ENSG00000198113
DEG.twoside	Brain_Nucleus_accumbens_basal_ganglia	8939	536	2.6363293097519e-16	1.42361782726602e-14	ENSG00000158286:ENSG00000158292:ENSG00000097021:ENSG00000171824:ENSG00000198793:ENSG00000120942:ENSG00000162458:ENSG00000065526:ENSG00000116809:ENSG00000183888:ENSG00000158748:ENSG00000204178:ENSG00000157978:ENSG00000134668:ENSG00000084652:ENSG00000116525:ENSG00000134686:ENSG00000121904:ENSG00000177181:ENSG00000066056:ENSG00000066322:ENSG00000159479:ENSG00000198198:ENSG00000162367:ENSG00000117862:ENSG00000198841:ENSG00000162390:ENSG00000117114:ENSG00000117155:ENSG00000142867:ENSG00000153904:ENSG00000171488:ENSG00000069712:ENSG00000117528:ENSG00000118733:ENSG00000134215:ENSG00000116396:ENSG00000162775:ENSG00000168679:ENSG00000116455:ENSG00000163349:ENSG00000197323:ENSG00000213281:ENSG00000052723:ENSG00000092621:ENSG00000143457:ENSG00000163131:ENSG00000143387:ENSG00000143437:ENSG00000143379:ENSG00000143418:ENSG00000143412:ENSG00000143409:ENSG00000143363:ENSG00000143434:ENSG00000163154:ENSG00000163156:ENSG00000143537:ENSG00000116521:ENSG00000176444:ENSG00000160752:ENSG00000125459:ENSG00000163374:ENSG00000132676:ENSG00000116580:ENSG00000132718:ENSG00000143622:ENSG00000132680:ENSG00000160789:ENSG00000160783:ENSG00000160781:ENSG00000163472:ENSG00000198715:ENSG00000187800:ENSG00000132694:ENSG00000171786:ENSG00000143226:ENSG00000186283:ENSG00000057252:ENSG00000134376:ENSG00000122176:ENSG00000182004:ENSG00000143801:ENSG00000163050:ENSG00000162885:ENSG00000116962:ENSG00000162714:ENSG00000107929:ENSG00000134463:ENSG00000148468:ENSG00000165997:ENSG00000165476:ENSG00000075073:ENSG00000107736:ENSG00000214688:ENSG00000148604:ENSG00000107796:ENSG00000026103:ENSG00000059573:ENSG00000197430:ENSG00000095587:ENSG00000120057:ENSG00000203867:ENSG00000198873:ENSG00000184545:ENSG00000166311:ENSG00000110169:ENSG00000205339:ENSG00000166478:ENSG00000198730:ENSG00000006071:ENSG00000129151:ENSG00000149150:ENSG00000162174:ENSG00000162300:ENSG00000162298:ENSG00000173653:ENSG00000173599:ENSG00000175567:ENSG00000175564:ENSG00000137492:ENSG00000149256:ENSG00000137693:ENSG00000149313:ENSG00000137714:ENSG00000149292:ENSG00000076053:ENSG00000076043:ENSG00000023171:ENSG00000109832:ENSG00000064309:ENSG00000183715:ENSG00000111262:ENSG00000111671:ENSG00000111679:ENSG00000126749:ENSG00000111684:ENSG00000182326:ENSG00000134531:ENSG00000123094:ENSG00000110911:ENSG00000123338:ENSG00000123360:ENSG00000127314:ENSG00000139292:ENSG00000166130:ENSG00000235162:ENSG00000074590:ENSG00000135094:ENSG00000174989:ENSG00000111725:ENSG00000122966:ENSG00000188735:ENSG00000150990:ENSG00000184992:ENSG00000177084:ENSG00000176915:ENSG00000127863:ENSG00000102743:ENSG00000215475:ENSG00000136167:ENSG00000102445:ENSG00000123179:ENSG00000184226:ENSG00000088451:ENSG00000065150:ENSG00000134901:ENSG00000139826:ENSG00000102524:ENSG00000100889:ENSG00000176165:ENSG00000186960:ENSG00000092108:ENSG00000100473:ENSG00000129493:ENSG00000150527:ENSG00000179476:ENSG00000179454:ENSG00000185246:ENSG00000139926:ENSG00000180998:ENSG00000197930:ENSG00000119599:ENSG00000165861:ENSG00000119707:ENSG00000080815:ENSG00000133961:ENSG00000165934:ENSG00000153485:ENSG00000140157:ENSG00000170113:ENSG00000169918:ENSG00000021776:ENSG00000174197:ENSG00000243708:ENSG00000243789:ENSG00000197535:ENSG00000181827:ENSG00000140262:ENSG00000140600:ENSG00000140511:ENSG00000140471:ENSG00000183454:ENSG00000179889:ENSG00000085721:ENSG00000157106:ENSG00000180096:ENSG00000179965:ENSG00000179958:ENSG00000140682:ENSG00000196470:ENSG00000102921:ENSG00000005194:ENSG00000072736:ENSG00000103066:ENSG00000103064:ENSG00000103061:ENSG00000103056:ENSG00000158717:ENSG00000167522:ENSG00000197912:ENSG00000127804:ENSG00000196689:ENSG00000197417:ENSG00000005100:ENSG00000072849:ENSG00000233670:ENSG00000007174:ENSG00000108641:ENSG00000166484:ENSG00000166482:ENSG00000128482:ENSG00000142494:ENSG00000067191:ENSG00000141750:ENSG00000068079:ENSG00000108830:ENSG00000182963:ENSG00000225190:ENSG00000198933:ENSG00000011258:ENSG00000011260:ENSG00000121060:ENSG00000108423:ENSG00000108443:ENSG00000189050:ENSG00000198265:ENSG00000197170:ENSG00000154217:ENSG00000180616:ENSG00000141540:ENSG00000141576:ENSG00000185262:ENSG00000141577:ENSG00000167302:ENSG00000224877:ENSG00000185504:ENSG00000182446:ENSG00000101608:ENSG00000017797:ENSG00000154845:ENSG00000154889:ENSG00000141401:ENSG00000168234:ENSG00000152223:ENSG00000081913:ENSG00000197971:ENSG00000071564:ENSG00000141985:ENSG00000167670:ENSG00000205744:ENSG00000104833:ENSG00000171105:ENSG00000104880:ENSG00000104883:ENSG00000167775:ENSG00000079999:ENSG00000104957:ENSG00000132016:ENSG00000132000:ENSG00000132017:ENSG00000132005:ENSG00000123146:ENSG00000123136:ENSG00000127527:ENSG00000099308:ENSG00000104835:ENSG00000128626:ENSG00000269190:ENSG00000167625:ENSG00000105722:ENSG00000079462:ENSG00000188368:ENSG00000167619:ENSG00000105429:ENSG00000187244:ENSG00000185453:ENSG00000105483:ENSG00000105447:ENSG00000196214:ENSG00000198464:ENSG00000198633:ENSG00000152467:ENSG00000115756:ENSG00000115761:ENSG00000197872:ENSG00000055917:ENSG00000171103:ENSG00000119866:ENSG00000162928:ENSG00000144034:ENSG00000144048:ENSG00000114978:ENSG00000065911:ENSG00000115602:ENSG00000153107:ENSG00000153214:ENSG00000115963:ENSG00000144283:ENSG00000153250:ENSG00000153253:ENSG00000118260:ENSG00000115457:ENSG00000115461:ENSG00000018280:ENSG00000144579:ENSG00000123977:ENSG00000132326:ENSG00000162804:ENSG00000088876:ENSG00000132646:ENSG00000188559:ENSG00000125966:ENSG00000198959:ENSG00000124107:ENSG00000124228:ENSG00000124201:ENSG00000125520:ENSG00000198276:ENSG00000154642:ENSG00000157617:ENSG00000184792:ENSG00000128335:ENSG00000100342:ENSG00000100321:ENSG000001

						98951:ENSG00000177096:ENSG00000242247:ENSG00000077942:ENSG00000186951:ENSG00000205643:ENSG00000075234:ENSG00000196576:ENSG00000205593:ENSG00000100258:ENSG00000130489:ENSG00000025708:ENSG00000177989:ENSG00000100288:ENSG00000008735:ENSG00000100299:ENSG00000070950:ENSG00000134077:ENSG00000144560:ENSG00000163517:ENSG00000163513:ENSG00000170293:ENSG00000168032:ENSG00000177873:ENSG00000182606:ENSG00000182983:ENSG00000214706:ENSG00000114378:ENSG00000068001:ENSG00000068028:ENSG00000004838:ENSG00000114395:ENSG00000126062:ENSG00000007402:ENSG00000180376:ENSG00000163946:ENSG00000144736:ENSG00000163320:ENSG00000175105:ENSG00000184500:ENSG00000169379:ENSG00000114446:ENSG00000163607:ENSG00000163608:ENSG00000144843:ENSG00000172780:ENSG00000240682:ENSG00000034533:ENSG00000114670:ENSG00000240303:ENSG00000113971:ENSG00000081307:ENSG00000144867:ENSG00000154917:ENSG00000138231:ENSG00000114098:ENSG00000158186:ENSG00000175110:ENSG00000184432:ENSG00000155903:ENSG00000175066:ENSG00000114127:ENSG00000114698:ENSG00000163762:ENSG00000169282:ENSG00000075420:ENSG00000161202:ENSG00000161204:ENSG00000114279:ENSG00000041802:ENSG00000163975:ENSG00000013288:ENSG00000145246:ENSG00000109171:ENSG00000145248:ENSG00000080493:ENSG00000169429:ENSG00000163739:ENSG00000173085:ENSG00000205403:ENSG00000164129:ENSG00000109466:ENSG00000113100:ENSG00000164190:ENSG00000152669:ENSG00000067248:ENSG00000039123:ENSG00000086200:ENSG00000164331:ENSG00000164338:ENSG00000164180:ENSG00000175471:ENSG00000173926:ENSG00000145794:ENSG00000064651:ENSG00000253159:ENSG00000240184:ENSG00000240764:ENSG00000131504:ENSG00000171720:ENSG00000164620:ENSG00000197948:ENSG00000120318:ENSG00000113721:ENSG00000113272:ENSG00000155858:ENSG00000182132:ENSG00000074317:ENSG00000048140:ENSG00000249109:ENSG00000131446:ENSG00000196670:ENSG00000078401:ENSG00000146112:ENSG00000137404:ENSG00000137337:ENSG00000137331:ENSG00000204344:ENSG00000244731:ENSG00000223501:ENSG00000235863:ENSG00000227057:ENSG00000124762:ENSG00000124772:ENSG00000164530:ENSG00000112245:ENSG00000172461:ENSG00000198945:ENSG00000135525:ENSG00000118503:ENSG00000112406:ENSG00000120278:ENSG00000112096:ENSG00000164488:ENSG00000186340:ENSG00000122644:ENSG00000168303:ENSG00000152926:ENSG00000213462:ENSG00000126522:ENSG00000086730:ENSG00000049541:ENSG00000177679:ENSG00000127920:ENSG00000105829:ENSG00000085514:ENSG00000078487:ENSG00000146834:ENSG00000160813:ENSG00000166924:ENSG00000005249:ENSG00000075790:ENSG00000091704:ENSG00000131558:ENSG00000122786:ENSG00000182158:ENSG00000196329:ENSG00000214106:ENSG00000146918:ENSG00000117868:ENSG00000184608:ENSG00000154319:ENSG00000168546:ENSG00000120910:ENSG00000165061:ENSG00000254087:ENSG00000035681:ENSG00000121039:ENSG00000104267:ENSG00000104327:ENSG00000164949:ENSG00000120526:ENSG00000120533:ENSG00000180900:ENSG00000196378:ENSG00000080608:ENSG00000213694:ENSG00000165023:ENSG00000127080:ENSG00000130958:ENSG00000136925:ENSG00000136826:ENSG00000196152:ENSG00000148356:ENSG00000136840:ENSG00000171169:ENSG00000148335:ENSG00000148308:ENSG00000170835:ENSG00000148411:ENSG00000198113
DEG.twoside	Brain_Putamen_basal_ganglia	9598	612	1.23642395906783e-26	6.67668937896631e-25	ENSG00000158286:ENSG00000158292:ENSG00000171824:ENSG00000198793:ENSG00000120942:ENSG00000162458:ENSG00000065526:ENSG00000116809:ENSG00000183888:ENSG00000158748:ENSG00000117616:ENSG00000204178:ENSG00000157978:ENSG00000084652:ENSG00000160050:ENSG00000160051:ENSG00000116525:ENSG00000160094:ENSG00000134686:ENSG00000121904:ENSG00000127603:ENSG00000177181:ENSG00000066056:ENSG00000159479:ENSG00000198198:ENSG00000162367:ENSG00000117862:ENSG00000198841:ENSG00000134717:ENSG00000162390:ENSG00000162604:ENSG00000162616:ENSG00000117114:ENSG00000117133:ENSG00000142867:ENSG00000153904:ENSG00000171488:ENSG00000069712:ENSG00000117528:ENSG00000117543:ENSG00000118733:ENSG00000134215:ENSG00000162775:ENSG00000168679:ENSG00000116455:ENSG00000163349:ENSG00000197323:ENSG00000213281:ENSG00000052723:ENSG00000092621:ENSG00000143457:ENSG00000163131:ENSG00000143387:ENSG00000143437:ENSG00000143379:ENSG00000143412:ENSG00000143409:ENSG00000143363:ENSG00000143434:ENSG00000163154:ENSG00000163156:ENSG00000163155:ENSG00000143537:ENSG00000160767:ENSG00000116521:ENSG00000176444:ENSG00000143630:ENSG00000160752:ENSG00000160753:ENSG00000116539:ENSG00000125459:ENSG00000163374:ENSG00000132676:ENSG00000116580:ENSG00000132718:ENSG00000143622:ENSG00000132680:ENSG00000160789:ENSG00000160783:ENSG00000160781:ENSG00000163472:ENSG00000198715:ENSG00000187800:ENSG00000132694:ENSG00000171786:ENSG00000143226:ENSG00000152332:ENSG00000120341:ENSG00000186283:ENSG00000143322:ENSG00000057252:ENSG00000177888:ENSG00000134376:ENSG00000122176:ENSG00000182004:ENSG00000143801:ENSG00000163050:ENSG00000116962:ENSG00000162714:ENSG00000107929:ENSG00000198879:ENSG00000134463:ENSG00000148468:ENSG00000241058:ENSG00000165997:ENSG00000108094:ENSG00000188234:ENSG00000165476:ENSG00000107736:ENSG00000107738:ENSG00000148604:ENSG00000138134:ENSG00000107796:ENSG00000026103:ENSG00000165338:ENSG00000059573:ENSG00000197430:ENSG00000095587:ENSG00000203867:ENSG00000198873:ENSG00000184545:ENSG00000166311:ENSG00000205339:ENSG00000166478:ENSG00000198730:ENSG00000129151:ENSG00000149150:ENSG00000162174:ENSG00000162300:ENSG00000149823:ENSG00000174276:ENSG00000162298:ENSG00000149792:ENSG00000173653:ENSG00000173599:ENSG00000175567:ENSG00000137492:ENSG00000137693:ENSG00000137714:ENSG00000149292:ENSG00000076053:ENSG00000076043:ENSG00000154127:ENSG00000109832:ENSG00000183715:ENSG00000082805:ENSG00000010219:ENSG00000139180:ENSG00000111262:ENSG00000111671:ENSG00000111679:ENSG00000126749:ENSG00000111684:ENSG00000182326:ENSG00000134531:ENSG00000123094:ENSG00000110911:ENSG00000050426:ENSG00000135457:ENSG00000123338:ENSG00000123360:ENSG00000127314:ENSG00000166130:ENSG00000120868:ENSG00000235162:ENSG00000074590:ENSG00000135094:ENSG00000174989:ENSG00000111725:ENSG00000122966:ENSG00000150990:ENSG00000177084:ENSG000001769

	15:ENSG00000127863:ENSG00000102743:ENSG00000215475:ENSG00000136167:ENSG00000102445:ENSG00000102531:ENSG00000123179:ENSG00000102753:ENSG00000136104:ENSG00000184226:ENSG00000188243:ENSG00000118939:ENSG00000136153:ENSG00000178695:ENSG00000088451:ENSG00000065150:ENSG00000134901:ENSG00000139826:ENSG00000102524:ENSG00000100889:ENSG00000168952:ENSG00000176165:ENSG00000186960:ENSG00000092108:ENSG00000100473:ENSG00000129493:ENSG00000150527:ENSG00000179476:ENSG00000179454:ENSG00000185246:ENSG00000139926:ENSG00000180998:ENSG00000197930:ENSG00000119599:ENSG00000165861:ENSG00000119707:ENSG00000100767:ENSG00000133961:ENSG00000165417:ENSG00000100433:ENSG00000165934:ENSG00000153485:ENSG00000170270:ENSG00000140157:ENSG00000170113:ENSG00000128731:ENSG00000169918:ENSG00000021776:ENSG00000198146:ENSG00000174197:ENSG00000137802:ENSG00000243708:ENSG00000243789:ENSG00000181827:ENSG00000140262:ENSG00000103707:ENSG00000186198:ENSG00000103710:ENSG00000117899:ENSG00000140600:ENSG00000140511:ENSG00000140471:ENSG00000008710:ENSG00000183454:ENSG00000183426:ENSG00000179889:ENSG00000085721:ENSG00000157106:ENSG00000169217:ENSG00000169221:ENSG00000180096:ENSG00000179958:ENSG00000140691:ENSG00000140682:ENSG00000197302:ENSG00000102910:ENSG00000196470:ENSG00000102921:ENSG00000005194:ENSG00000102978:ENSG00000072736:ENSG00000103066:ENSG00000103064:ENSG00000103061:ENSG00000103056:ENSG00000185669:ENSG00000158717:ENSG00000167522:ENSG00000197912:ENSG00000127804:ENSG00000196689:ENSG00000197417:ENSG00000005100:ENSG00000072849:ENSG00000166484:ENSG00000166482:ENSG00000128482:ENSG00000142494:ENSG00000068079:ENSG00000108830:ENSG00000012048:ENSG00000182963:ENSG00000225190:ENSG00000141279:ENSG00000108424:ENSG00000198933:ENSG00000011258:ENSG00000011260:ENSG00000214226:ENSG00000121060:ENSG00000108423:ENSG00000108443:ENSG00000189050:ENSG00000198265:ENSG00000197170:ENSG00000154217:ENSG00000153822:ENSG00000141540:ENSG00000196169:ENSG00000141576:ENSG00000185262:ENSG00000141577:ENSG00000167302:ENSG00000185504:ENSG00000182446:ENSG00000101608:ENSG00000017797:ENSG00000154845:ENSG00000154889:ENSG00000141401:ENSG00000141384:ENSG00000141469:ENSG00000152223:ENSG00000081913:ENSG00000130856:ENSG00000197971:ENSG00000071564:ENSG00000141985:ENSG00000167670:ENSG00000205744:ENSG00000104833:ENSG00000171105:ENSG00000104880:ENSG00000167775:ENSG00000065989:ENSG00000079999:ENSG00000180739:ENSG00000104957:ENSG00000132024:ENSG00000132000:ENSG00000132017:ENSG00000132005:ENSG00000123146:ENSG00000123136:ENSG00000127527:ENSG00000099308:ENSG00000262484:ENSG00000104835:ENSG00000128626:ENSG00000269190:ENSG00000028277:ENSG00000160570:ENSG00000167625:ENSG00000105722:ENSG00000079432:ENSG00000167619:ENSG00000187244:ENSG00000185453:ENSG00000105483:ENSG00000105447:ENSG00000182324:ENSG00000142544:ENSG00000196214:ENSG00000198464:ENSG00000167766:ENSG00000213020:ENSG00000115756:ENSG00000115761:ENSG00000197872:ENSG00000055917:ENSG00000163803:ENSG00000171103:ENSG00000119866:ENSG00000162928:ENSG00000082898:ENSG00000075292:ENSG00000144034:ENSG00000144048:ENSG00000114978:ENSG00000065911:ENSG00000114993:ENSG00000115602:ENSG00000115604:ENSG00000153107:ENSG00000153214:ENSG00000115963:ENSG00000144283:ENSG00000153250:ENSG00000153253:ENSG00000118260:ENSG00000197713:ENSG00000115457:ENSG00000115461:ENSG00000018280:ENSG00000144579:ENSG00000132326:ENSG00000162804:ENSG00000088876:ENSG00000125779:ENSG00000132646:ENSG00000188559:ENSG00000198959:ENSG00000124107:ENSG00000124228:ENSG00000124201:ENSG00000125520:ENSG00000198276:ENSG00000155313:ENSG00000157617:ENSG00000160190:ENSG00000184792:ENSG00000100220:ENSG00000128335:ENSG00000100342:ENSG00000100321:ENSG00000100324:ENSG00000198951:ENSG00000177096:ENSG00000242247:ENSG00000077942:ENSG00000186951:ENSG00000205643:ENSG00000075234:ENSG00000100416:ENSG00000196576:ENSG00000100258:ENSG00000025770:ENSG00000130489:ENSG00000025708:ENSG00000177989:ENSG00000205560:ENSG00000100288:ENSG00000008735:ENSG00000100299:ENSG00000134077:ENSG00000144560:ENSG00000144712:ENSG00000163517:ENSG00000163513:ENSG00000170293:ENSG00000170011:ENSG00000168032:ENSG00000177873:ENSG00000182606:ENSG00000182983:ENSG00000214706:ENSG00000114378:ENSG00000068001:ENSG00000068028:ENSG00000114388:ENSG00000114395:ENSG00000126062:ENSG00000180376:ENSG00000163946:ENSG00000144741:ENSG00000114861:ENSG00000144736:ENSG00000163320:ENSG00000175105:ENSG00000179021:ENSG00000184500:ENSG00000169379:ENSG00000114446:ENSG00000163607:ENSG00000163608:ENSG00000144843:ENSG00000082701:ENSG00000172780:ENSG00000240682:ENSG00000034533:ENSG00000240303:ENSG00000113971:ENSG00000081307:ENSG00000144867:ENSG00000154917:ENSG00000138231:ENSG00000114098:ENSG00000175110:ENSG00000184432:ENSG00000114115:ENSG00000155903:ENSG00000175066:ENSG00000114127:ENSG00000114698:ENSG00000169282:ENSG00000075420:ENSG00000161202:ENSG00000161204:ENSG00000041802:ENSG00000163975:ENSG00000013288:ENSG00000109689:ENSG00000169019:ENSG00000145246:ENSG00000109171:ENSG00000145248:ENSG00000080493:ENSG00000169429:ENSG00000163739:ENSG00000173085:ENSG00000205403:ENSG00000109466:ENSG00000129187:ENSG00000113100:ENSG00000113360:ENSG00000164190:ENSG00000067248:ENSG00000039123:ENSG00000086189:ENSG00000086200:ENSG00000164331:ENSG00000164338:ENSG00000164180:ENSG00000175471:ENSG00000168916:ENSG00000173926:ENSG00000145794:ENSG00000240184:ENSG00000240764:ENSG00000131504:ENSG00000171720:ENSG00000197948:ENSG00000120318:ENSG00000113721:ENSG00000135074:ENSG00000113272:ENSG00000155858:ENSG00000182132:ENSG00000113742:ENSG00000074317:ENSG00000048140:ENSG00000249109:ENSG00000131446:ENSG00000196670:ENSG00000124535:ENSG00000124787:ENSG00000146112:ENSG00000137404:ENSG00000137337:ENSG00000137331:ENSG00000204344:ENSG00000244731:ENSG00000223501:ENSG00000235863:ENSG00000227057:ENSG00000237441:ENSG00000124762:ENSG00000124772:ENSG00000198663:ENSG00000164530:ENSG00000112245:ENSG00000172461:ENSG00000198945:ENSG00000135525:ENSG00000112357:ENSG00000118503:ENSG00000112406:ENSG00000120278:ENSG00000112096:ENSG00000026297:ENS

						G00000213066:ENSG00000164488:ENSG00000186340:ENSG00000184786:ENSG00000130023:ENSG00000122644:ENSG00000153814:ENSG00000168303:ENSG00000152926:ENSG00000213462:ENSG00000126522:ENSG00000086730:ENSG00000049541:ENSG00000177679:ENSG00000085563:ENSG00000127920:ENSG00000105829:ENSG00000121716:ENSG00000085514:ENSG00000078487:ENSG00000146834:ENSG00000160813:ENSG00000166924:ENSG00000075790:ENSG00000196937:ENSG00000091704:ENSG00000106484:ENSG00000158623:ENSG00000131558:ENSG00000122786:ENSG00000182158:ENSG00000196329:ENSG00000214106:ENSG00000146918:ENSG00000117868:ENSG00000154319:ENSG00000120910:ENSG00000129691:ENSG00000165061:ENSG00000254087:ENSG00000035681:ENSG00000147601:ENSG00000121039:ENSG00000104267:ENSG00000104327:ENSG00000164949:ENSG00000120526:ENSG00000120533:ENSG00000180900:ENSG00000196378:ENSG00000080608:ENSG00000213694:ENSG00000165023:ENSG00000127080:ENSG00000130958:ENSG00000136925:ENSG00000136936:ENSG00000136826:ENSG00000119446:ENSG00000196152:ENSG00000148356:ENSG00000136840:ENSG00000171169:ENSG00000148339:ENSG00000148335:ENSG00000148308:ENSG00000170835:ENSG00000148411:ENSG00000198113
DEG.twoside	Brain_Spinal_cord_cervical_c-1	7371	453	4.97567875502558e-15	2.68686652771382e-13	ENSG00000158286:ENSG00000116237:ENSG00000158292:ENSG00000097021:ENSG00000198793:ENSG00000171819:ENSG00000162458:ENSG00000065526:ENSG00000183888:ENSG00000158747:ENSG00000188672:ENSG00000157978:ENSG00000084652:ENSG00000160050:ENSG00000160051:ENSG00000116525:ENSG00000177181:ENSG00000179178:ENSG00000066056:ENSG00000198198:ENSG00000198841:ENSG00000116205:ENSG00000116745:ENSG00000117114:ENSG00000117133:ENSG00000117155:ENSG00000142867:ENSG00000197147:ENSG00000069712:ENSG00000117525:ENSG00000116396:ENSG00000162775:ENSG00000085465:ENSG00000116455:ENSG00000197323:ENSG00000092621:ENSG00000143457:ENSG00000143387:ENSG00000143437:ENSG00000143379:ENSG00000143409:ENSG00000143363:ENSG00000143434:ENSG00000163354:ENSG00000160767:ENSG00000176444:ENSG00000143630:ENSG00000160753:ENSG00000116539:ENSG00000125459:ENSG00000163374:ENSG00000132676:ENSG00000116580:ENSG00000132718:ENSG00000143622:ENSG00000132680:ENSG00000160789:ENSG00000196189:ENSG00000160781:ENSG00000198715:ENSG00000187800:ENSG00000132694:ENSG00000203747:ENSG00000120341:ENSG00000186283:ENSG00000057252:ENSG00000134376:ENSG00000122176:ENSG00000143801:ENSG00000163050:ENSG00000162885:ENSG00000116962:ENSG00000162714:ENSG00000107929:ENSG00000198879:ENSG00000134463:ENSG00000148468:ENSG00000165997:ENSG00000165476:ENSG00000107736:ENSG00000214688:ENSG00000107796:ENSG00000026103:ENSG00000165338:ENSG00000059573:ENSG00000197430:ENSG00000095587:ENSG00000120057:ENSG00000119927:ENSG00000198873:ENSG00000166483:ENSG00000198730:ENSG00000006071:ENSG00000129151:ENSG00000149150:ENSG00000162174:ENSG00000168060:ENSG00000174276:ENSG00000173653:ENSG00000173599:ENSG00000173621:ENSG00000175564:ENSG00000062282:ENSG00000137492:ENSG00000137693:ENSG00000137714:ENSG00000149292:ENSG00000076053:ENSG00000076043:ENSG00000082805:ENSG00000111684:ENSG00000182326:ENSG00000013588:ENSG00000134531:ENSG00000134532:ENSG00000123360:ENSG00000127314:ENSG00000139292:ENSG00000166130:ENSG00000235162:ENSG00000110887:ENSG00000139410:ENSG00000174989:ENSG00000111725:ENSG00000122966:ENSG00000188735:ENSG00000150990:ENSG00000184992:ENSG00000177169:ENSG00000177084:ENSG00000176915:ENSG00000127863:ENSG00000102743:ENSG00000123179:ENSG00000184226:ENSG00000118939:ENSG00000136153:ENSG00000134901:ENSG00000139826:ENSG00000129535:ENSG00000100889:ENSG00000168952:ENSG00000092108:ENSG00000100473:ENSG00000150527:ENSG00000165355:ENSG00000139926:ENSG00000180998:ENSG00000197930:ENSG00000165807:ENSG00000006432:ENSG00000119599:ENSG00000165861:ENSG00000080815:ENSG00000100767:ENSG00000133961:ENSG00000100433:ENSG00000140157:ENSG00000170113:ENSG00000169918:ENSG00000021776:ENSG00000198146:ENSG00000137802:ENSG00000181827:ENSG00000103707:ENSG00000140600:ENSG00000140511:ENSG00000140471:ENSG00000185418:ENSG00000008710:ENSG00000179889:ENSG00000085721:ENSG00000157106:ENSG00000180209:ENSG00000180096:ENSG00000179958:ENSG00000140682:ENSG00000102910:ENSG00000102921:ENSG00000005194:ENSG00000072736:ENSG00000103066:ENSG00000103064:ENSG00000103056:ENSG00000172137:ENSG00000167515:ENSG00000167522:ENSG00000197912:ENSG00000197417:ENSG00000029725:ENSG00000005100:ENSG00000233670:ENSG00000007174:ENSG00000181350:ENSG00000141127:ENSG00000072134:ENSG00000166484:ENSG00000166482:ENSG00000142494:ENSG00000180638:ENSG00000067191:ENSG00000068079:ENSG00000108830:ENSG00000182963:ENSG00000225190:ENSG00000141279:ENSG00000198933:ENSG00000011258:ENSG00000121060:ENSG00000189050:ENSG00000198265:ENSG00000123700:ENSG00000141540:ENSG00000196169:ENSG00000141576:ENSG00000167302:ENSG00000185504:ENSG00000182446:ENSG00000154845:ENSG00000134265:ENSG00000154889:ENSG00000141401:ENSG00000168234:ENSG00000141384:ENSG00000141469:ENSG00000152223:ENSG00000081913:ENSG00000197971:ENSG00000071564:ENSG00000141985:ENSG00000167670:ENSG00000104833:ENSG00000171105:ENSG00000065989:ENSG00000180739:ENSG00000132000:ENSG00000132017:ENSG00000132005:ENSG00000123146:ENSG00000127527:ENSG00000124302:ENSG00000262484:ENSG00000104835:ENSG00000128626:ENSG00000269190:ENSG00000160570:ENSG00000167625:ENSG00000079432:ENSG00000167619:ENSG00000105427:ENSG00000079435:ENSG00000187244:ENSG00000105447:ENSG00000196214:ENSG00000115761:ENSG00000134323:ENSG00000163803:ENSG00000162928:ENSG00000162929:ENSG00000144048:ENSG00000217702:ENSG00000114978:ENSG00000065911:ENSG00000005448:ENSG00000114993:ENSG00000115604:ENSG00000153107:ENSG00000153208:ENSG00000153214:ENSG00000115963:ENSG00000144283:ENSG00000153250:ENSG00000115457:ENSG00000115461:ENSG00000153832:ENSG00000186235:ENSG00000144485:ENSG00000132326:ENSG00000162804:ENSG00000088876:ENSG00000125779:ENSG00000089048:ENSG00000125864:ENSG00000188559:ENSG00000124107:ENSG00000124228:ENSG00000124201:ENSG00000125520:ENSG00000198276:ENSG00000155313:ENSG00000154642:ENSG00000160190:ENSG00000100342:ENSG00000198951:ENSG00000177096:ENSG00000100197:ENSG

						00000242247:ENSG00000077942:ENSG00000075234:ENSG00000196576:ENSG00000130489:ENSG00000177989:ENSG00000008735:ENSG00000070950:ENSG00000144560:ENSG00000144712:ENSG00000163517:ENSG00000163513:ENSG00000170293:ENSG00000168032:ENSG00000182606:ENSG00000182983:ENSG00000214706:ENSG00000186792:ENSG00000114378:ENSG00000068001:ENSG00000126062:ENSG00000144749:ENSG00000114861:ENSG00000144736:ENSG00000184500:ENSG00000178700:ENSG00000114446:ENSG00000144843:ENSG00000172780:ENSG00000240682:ENSG00000144867:ENSG00000154917:ENSG00000158163:ENSG00000184432:ENSG00000114115:ENSG00000155903:ENSG00000114127:ENSG00000114698:ENSG00000075420:ENSG00000145014:ENSG00000041802:ENSG00000163975:ENSG00000013288:ENSG00000145246:ENSG00000109171:ENSG00000173085:ENSG00000123739:ENSG00000164128:ENSG00000109466:ENSG00000113360:ENSG00000164190:ENSG00000132357:ENSG00000067248:ENSG00000068796:ENSG00000197822:ENSG00000164338:ENSG00000164180:ENSG00000175471:ENSG00000181751:ENSG00000168916:ENSG00000145794:ENSG00000064651:ENSG00000131504:ENSG00000171720:ENSG00000197948:ENSG00000120318:ENSG00000113721:ENSG00000135074:ENSG00000113272:ENSG00000155858:ENSG00000182132:ENSG00000074317:ENSG00000048140:ENSG00000131446:ENSG00000137266:ENSG00000079691:ENSG00000146112:ENSG00000137404:ENSG00000137337:ENSG00000204344:ENSG00000244731:ENSG00000235863:ENSG00000227057:ENSG00000237441:ENSG00000231925:ENSG00000124772:ENSG00000164530:ENSG00000118432:ENSG00000172461:ENSG00000132429:ENSG00000198945:ENSG00000135525:ENSG00000118503:ENSG00000112406:ENSG00000146416:ENSG00000120278:ENSG00000131018:ENSG00000026297:ENSG00000213066:ENSG00000122644:ENSG00000105855:ENSG00000153814:ENSG00000241644:ENSG00000152926:ENSG00000213462:ENSG00000146757:ENSG00000126522:ENSG00000086730:ENSG00000085563:ENSG00000127920:ENSG00000078487:ENSG00000146834:ENSG00000160813:ENSG00000185955:ENSG00000005249:ENSG00000105865:ENSG00000196937:ENSG00000091704:ENSG00000106484:ENSG00000182158:ENSG00000146966:ENSG00000197993:ENSG00000181444:ENSG00000196329:ENSG00000214106:ENSG00000146918:ENSG00000117868:ENSG00000168546:ENSG00000120910:ENSG00000147465:ENSG00000023287:ENSG00000035681:ENSG00000121039:ENSG00000104267:ENSG00000104327:ENSG00000164949:ENSG00000120526:ENSG00000120533:ENSG00000129295:ENSG00000165071:ENSG00000131773:ENSG00000160932:ENSG00000080608:ENSG00000044459:ENSG00000213694:ENSG00000165023:ENSG00000127080:ENSG00000136925:ENSG00000136826:ENSG00000167081:ENSG00000196152:ENSG00000136840:ENSG00000171169:ENSG00000148339:ENSG00000170835:ENSG00000148411:ENSG00000198113
DEG.twoside	Brain_Substantia_nigra	8873	551	2.7107864415358e-20	1.46382467842933e-18	ENSG00000158286:ENSG00000116237:ENSG00000158292:ENSG00000097021:ENSG00000171824:ENSG00000198793:ENSG00000120942:ENSG00000162458:ENSG00000065526:ENSG00000116809:ENSG00000183888:ENSG00000204178:ENSG00000084652:ENSG00000160051:ENSG00000160094:ENSG00000121904:ENSG00000177181:ENSG00000117395:ENSG00000179178:ENSG00000066056:ENSG00000117399:ENSG00000159479:ENSG00000198198:ENSG00000117472:ENSG00000117862:ENSG00000198841:ENSG00000116745:ENSG00000117114:ENSG00000117133:ENSG00000117155:ENSG00000142867:ENSG00000153904:ENSG00000117543:ENSG00000118733:ENSG00000162775:ENSG00000168679:ENSG00000085465:ENSG00000116455:ENSG00000163349:ENSG00000197323:ENSG00000092621:ENSG00000143457:ENSG00000163131:ENSG00000143387:ENSG00000143437:ENSG00000143379:ENSG00000143412:ENSG00000143363:ENSG00000143434:ENSG00000163156:ENSG00000163155:ENSG00000160767:ENSG00000176444:ENSG00000160753:ENSG00000116539:ENSG00000125459:ENSG00000163374:ENSG00000132676:ENSG00000116580:ENSG00000132718:ENSG00000143622:ENSG00000132680:ENSG00000160789:ENSG00000196189:ENSG00000160783:ENSG00000160781:ENSG00000163472:ENSG00000198715:ENSG00000187800:ENSG00000132694:ENSG00000203747:ENSG00000120341:ENSG00000186283:ENSG00000143322:ENSG00000057252:ENSG00000122176:ENSG00000182004:ENSG00000143801:ENSG00000163050:ENSG00000162885:ENSG00000116962:ENSG00000162714:ENSG00000107929:ENSG00000134463:ENSG00000148468:ENSG00000165997:ENSG00000108094:ENSG00000188234:ENSG00000214688:ENSG00000148604:ENSG00000138134:ENSG00000107796:ENSG00000026103:ENSG00000165338:ENSG00000059573:ENSG00000197430:ENSG00000198873:ENSG00000166478:ENSG00000166483:ENSG00000198730:ENSG00000129151:ENSG00000019505:ENSG00000149150:ENSG00000162174:ENSG00000110025:ENSG00000162300:ENSG00000149823:ENSG00000174276:ENSG00000162298:ENSG00000149792:ENSG00000173653:ENSG00000173599:ENSG00000173621:ENSG00000175567:ENSG00000062282:ENSG00000137492:ENSG00000137672:ENSG00000137693:ENSG00000149292:ENSG00000076053:ENSG00000076043:ENSG00000166250:ENSG00000082805:ENSG00000111671:ENSG00000111679:ENSG00000126749:ENSG00000111684:ENSG00000182326:ENSG00000013588:ENSG00000134531:ENSG00000050426:ENSG00000123338:ENSG00000123360:ENSG00000127314:ENSG00000139292:ENSG00000166130:ENSG00000120868:ENSG00000235162:ENSG00000074590:ENSG00000110887:ENSG00000135094:ENSG00000139410:ENSG00000174989:ENSG00000111725:ENSG00000188735:ENSG00000150990:ENSG00000177169:ENSG00000177084:ENSG00000176915:ENSG00000127863:ENSG00000102743:ENSG00000215475:ENSG00000136167:ENSG00000102531:ENSG00000123179:ENSG00000102753:ENSG00000136104:ENSG00000184226:ENSG00000118939:ENSG00000136153:ENSG00000088451:ENSG00000065150:ENSG00000134901:ENSG00000139826:ENSG00000102524:ENSG00000100889:ENSG00000100897:ENSG00000092140:ENSG00000092108:ENSG00000150527:ENSG00000165355:ENSG00000179476:ENSG00000185246:ENSG00000139926:ENSG00000180998:ENSG00000197930:ENSG00000119599:ENSG00000165861:ENSG00000119707:ENSG00000100767:ENSG00000133961:ENSG00000165417:ENSG00000100433:ENSG00000165934:ENSG00000153485:ENSG00000254656:ENSG00000156414:ENSG00000140157:ENSG00000170113:ENSG00000169918:ENSG00000021776:ENSG00000198146:ENSG00000174197:ENSG00000137802:ENSG00000243708:ENSG00000243789:ENSG00000197535:ENSG00000181827:ENSG00000103707:ENSG00000103710:ENSG00000136378:ENSG00000140406:ENSG00000140600:ENSG00000140511:ENSG00000140471:ENSG00000008

						710:ENSG00000183426:ENSG00000179889:ENSG00000157045:ENSG00000085721:ENSG00000157106:ENSG00000180035:ENSG00000180096:ENSG00000179958:ENSG00000140691:ENSG00000140682:ENSG00000102910:ENSG00000196470:ENSG00000102921:ENSG00000005194:ENSG00000102978:ENSG00000072736:ENSG00000103066:ENSG00000103064:ENSG00000132600:ENSG00000103056:ENSG00000172137:ENSG00000158717:ENSG00000167522:ENSG00000197912:ENSG00000127804:ENSG00000197417:ENSG00000005100:ENSG00000072849:ENSG00000233670:ENSG00000108641:ENSG00000166484:ENSG00000166482:ENSG00000142494:ENSG00000067191:ENSG00000068079:ENSG00000108830:ENSG00000184988:ENSG00000182963:ENSG00000225190:ENSG00000141279:ENSG00000011258:ENSG00000011260:ENSG00000153933:ENSG00000121060:ENSG00000108423:ENSG00000108443:ENSG00000189050:ENSG00000198265:ENSG00000154217:ENSG00000153822:ENSG00000180616:ENSG00000141540:ENSG00000196169:ENSG00000141576:ENSG00000141577:ENSG00000167302:ENSG00000185504:ENSG00000182446:ENSG00000017797:ENSG00000154845:ENSG00000154889:ENSG00000141401:ENSG00000168234:ENSG00000141384:ENSG00000141469:ENSG00000152223:ENSG00000081913:ENSG00000130856:ENSG00000197971:ENSG00000071564:ENSG00000141985:ENSG00000167670:ENSG00000205744:ENSG00000104833:ENSG00000171105:ENSG00000104880:ENSG00000180739:ENSG00000104957:ENSG00000132017:ENSG00000132005:ENSG00000123146:ENSG00000123136:ENSG00000127527:ENSG00000237440:ENSG00000124302:ENSG00000104835:ENSG00000128626:ENSG00000269190:ENSG00000160570:ENSG00000167625:ENSG00000105722:ENSG00000079432:ENSG00000167619:ENSG00000105427:ENSG00000079435:ENSG00000187244:ENSG00000105483:ENSG00000105464:ENSG00000105447:ENSG00000142544:ENSG00000105383:ENSG00000196214:ENSG00000198464:ENSG00000167766:ENSG00000213020:ENSG00000115761:ENSG00000134323:ENSG00000055917:ENSG00000084676:ENSG00000171103:ENSG00000119866:ENSG00000162928:ENSG00000082898:ENSG00000075292:ENSG00000144048:ENSG00000114978:ENSG00000065911:ENSG00000005448:ENSG00000114993:ENSG00000115604:ENSG00000153107:ENSG00000153208:ENSG00000153214:ENSG00000115963:ENSG00000144283:ENSG00000153250:ENSG00000153253:ENSG00000118260:ENSG00000115457:ENSG00000115461:ENSG00000144579:ENSG00000144485:ENSG00000132326:ENSG00000162804:ENSG00000088876:ENSG00000125779:ENSG00000132646:ENSG00000188559:ENSG00000124107:ENSG00000124228:ENSG00000124201:ENSG00000125520:ENSG00000198276:ENSG00000155313:ENSG00000154642:ENSG00000157617:ENSG00000160190:ENSG00000160284:ENSG00000100342:ENSG00000100321:ENSG00000128285:ENSG00000198951:ENSG00000177096:ENSG00000100197:ENSG00000242247:ENSG00000077942:ENSG00000205643:ENSG00000075234:ENSG00000196576:ENSG00000100258:ENSG00000025770:ENSG00000130489:ENSG00000025708:ENSG00000177989:ENSG00000205560:ENSG00000100288:ENSG00000008735:ENSG00000100299:ENSG00000251322:ENSG00000070950:ENSG00000134077:ENSG00000144560:ENSG00000144712:ENSG00000163517:ENSG00000163513:ENSG00000170293:ENSG00000182606:ENSG00000182983:ENSG00000214706:ENSG00000243477:ENSG00000114378:ENSG00000068001:ENSG00000068028:ENSG00000114395:ENSG00000126062:ENSG00000180376:ENSG00000163946:ENSG00000114861:ENSG00000144736:ENSG00000163320:ENSG00000175105:ENSG00000179021:ENSG00000184500:ENSG00000169379:ENSG00000114446:ENSG00000240891:ENSG00000163607:ENSG00000163608:ENSG00000144843:ENSG00000172780:ENSG00000240682:ENSG00000034533:ENSG00000113971:ENSG00000081307:ENSG00000154917:ENSG00000138231:ENSG00000114098:ENSG00000158186:ENSG00000175110:ENSG00000184432:ENSG00000114115:ENSG00000155903:ENSG00000175066:ENSG00000114127:ENSG00000114698:ENSG00000169282:ENSG00000075420:ENSG00000161202:ENSG00000114279:ENSG00000145014:ENSG00000041802:ENSG00000163975:ENSG00000013288:ENSG00000109689:ENSG00000145246:ENSG00000109171:ENSG00000145248:ENSG00000182223:ENSG00000080493:ENSG00000173085:ENSG00000205403:ENSG00000164128:ENSG00000109466:ENSG00000129187:ENSG00000113360:ENSG00000164190:ENSG00000171522:ENSG00000132357:ENSG00000067248:ENSG00000039123:ENSG00000086200:ENSG00000164331:ENSG00000164338:ENSG00000164180:ENSG00000175471:ENSG00000181751:ENSG00000145794:ENSG00000253159:ENSG00000131504:ENSG00000171720:ENSG00000164620:ENSG00000197948:ENSG00000120318:ENSG00000113721:ENSG00000135074:ENSG00000113272:ENSG00000155858:ENSG00000182132:ENSG00000074317:ENSG00000048140:ENSG00000131446:ENSG00000196670:ENSG00000124535:ENSG00000124787:ENSG00000079691:ENSG00000146112:ENSG00000137404:ENSG00000137337:ENSG00000137331:ENSG00000204344:ENSG00000244731:ENSG00000223501:ENSG00000235863:ENSG00000227057:ENSG00000237441:ENSG00000231925:ENSG00000124762:ENSG00000118432:ENSG00000172461:ENSG00000198945:ENSG00000135525:ENSG00000118503:ENSG00000112406:ENSG00000131018:ENSG00000213066:ENSG00000184786:ENSG00000130023:ENSG00000122644:ENSG00000105855:ENSG00000153814:ENSG00000168303:ENSG00000152926:ENSG00000213462:ENSG00000126522:ENSG00000049541:ENSG00000085563:ENSG00000127920:ENSG00000105829:ENSG00000121716:ENSG00000085514:ENSG00000146834:ENSG00000160813:ENSG00000185955:ENSG00000166924:ENSG00000196937:ENSG00000091704:ENSG00000106484:ENSG00000158623:ENSG00000131558:ENSG00000122786:ENSG00000182158:ENSG00000146966:ENSG00000181444:ENSG00000196329:ENSG00000214106:ENSG00000146918:ENSG00000117868:ENSG00000120910:ENSG00000129691:ENSG00000023287:ENSG00000254087:ENSG00000035681:ENSG00000121039:ENSG00000104267:ENSG00000164949:ENSG00000120526:ENSG00000120533:ENSG00000196378:ENSG00000080608:ENSG00000044459:ENSG00000213694:ENSG00000165023:ENSG00000127080:ENSG00000136925:ENSG00000136826:ENSG00000196152:ENSG00000148356:ENSG00000136840:ENSG00000171169:ENSG00000148339:ENSG00000148335:ENSG00000148308:ENSG00000170835:ENSG00000148411:ENSG00000198113
DEG.twoside	Breast_Mammary_Tissue	2985	170	0.000495483985342512	0.0267561352084956	ENSG00000158292:ENSG00000097021:ENSG00000183888:ENSG00000160050:ENSG00000168389:ENSG00000066056:ENSG00000117399:ENSG00000162367:ENSG00000117155:ENSG00000153904:ENSG00000171488:ENSG00000134215:ENSG00000175984:ENSG00000134240:ENSG00000143387:ENSG00000163354:ENSG00000132718:ENSG00000160781:ENSG00000187800:ENSG00000173110:ENSG00000174175:ENSG00000122176:ENSG00000116962:ENSG00000198879:ENSG000001

						34463:ENSG00000075073:ENSG00000120057:ENSG00000203867:ENSG00000119927:ENSG00000110169:ENSG00000135373:ENSG00000124939:ENSG00000124935:ENSG00000146670:ENSG00000173599:ENSG00000175567:ENSG00000175564:ENSG00000062282:ENSG00000154127:ENSG00000166250:ENSG00000111684:ENSG00000213782:ENSG00000134531:ENSG00000087494:ENSG00000123360:ENSG00000074590:ENSG00000122966:ENSG00000184992:ENSG00000127863:ENSG00000133110:ENSG00000136153:ENSG00000178695:ENSG00000139914:ENSG00000168952:ENSG00000180998:ENSG00000006432:ENSG00000156414:ENSG00000170113:ENSG00000186198:ENSG00000103888:ENSG00000140511:ENSG00000180209:ENSG00000140682:ENSG00000121270:ENSG00000196470:ENSG00000103064:ENSG00000172137:ENSG00000185669:ENSG00000188522:ENSG00000128482:ENSG00000142494:ENSG00000067191:ENSG00000141750:ENSG00000108830:ENSG00000073670:ENSG00000153822:ENSG00000123700:ENSG00000141540:ENSG00000141469:ENSG00000197971:ENSG00000205744:ENSG00000167775:ENSG00000099308:ENSG00000028277:ENSG00000079435:ENSG00000105483:ENSG00000115705:ENSG00000196208:ENSG00000143867:ENSG00000163803:ENSG00000119866:ENSG00000116039:ENSG00000065911:ENSG00000198885:ENSG00000144218:ENSG00000115602:ENSG00000115607:ENSG00000163040:ENSG00000115963:ENSG00000144485:ENSG00000162804:ENSG00000125966:ENSG00000157617:ENSG00000184792:ENSG00000100342:ENSG00000075234:ENSG00000075275:ENSG00000177989:ENSG00000008735:ENSG00000251322:ENSG00000070950:ENSG00000144712:ENSG00000163517:ENSG00000170293:ENSG00000182983:ENSG00000068001:ENSG00000004838:ENSG00000007402:ENSG00000184500:ENSG00000144843:ENSG00000113971:ENSG00000154917:ENSG00000114698:ENSG00000163762:ENSG00000174899:ENSG00000075420:ENSG00000114279:ENSG00000145014:ENSG00000163975:ENSG00000152953:ENSG00000157765:ENSG00000174145:ENSG00000080493:ENSG00000109321:ENSG00000145287:ENSG00000205403:ENSG00000164128:ENSG00000215218:ENSG00000171522:ENSG00000175471:ENSG00000164620:ENSG00000120318:ENSG00000135074:ENSG00000078401:ENSG00000124772:ENSG00000118432:ENSG00000120278:ENSG00000091831:ENSG00000186340:ENSG00000122644:ENSG00000152926:ENSG00000213462:ENSG00000146757:ENSG00000165215:ENSG00000189143:ENSG00000086730:ENSG00000177679:ENSG00000135218:ENSG00000085563:ENSG00000127920:ENSG00000005249:ENSG00000106484:ENSG00000159763:ENSG00000196329:ENSG00000168546:ENSG00000104267:ENSG00000164949:ENSG00000120526:ENSG00000160339:ENSG00000148411
DEG.twoside	Cells_Cultured_fibroblasts	6516	382	1.25742817544916e-09	6.79011214742544e-08	ENSG00000158286:ENSG00000116237:ENSG00000097021:ENSG00000120942:ENSG00000187942:ENSG00000134668:ENSG00000121904:ENSG00000117395:ENSG00000117399:ENSG00000123473:ENSG00000117862:ENSG00000198841:ENSG00000134717:ENSG00000162604:ENSG00000162616:ENSG00000142867:ENSG00000153904:ENSG00000197147:ENSG00000171488:ENSG00000069712:ENSG00000117569:ENSG00000162694:ENSG00000117543:ENSG00000162775:ENSG00000168679:ENSG00000085465:ENSG00000116455:ENSG00000175984:ENSG00000213281:ENSG00000052723:ENSG00000092621:ENSG00000143457:ENSG00000163131:ENSG00000143387:ENSG00000143412:ENSG00000143409:ENSG00000143363:ENSG00000143434:ENSG00000160767:ENSG00000143630:ENSG00000196189:ENSG00000160781:ENSG00000198715:ENSG00000187800:ENSG00000162746:ENSG00000152332:ENSG00000143322:ENSG00000057252:ENSG00000066279:ENSG00000177888:ENSG00000163050:ENSG00000162885:ENSG00000116962:ENSG00000134463:ENSG00000065328:ENSG00000148468:ENSG00000108094:ENSG00000095794:ENSG00000165476:ENSG00000138134:ENSG00000026103:ENSG00000059573:ENSG00000119927:ENSG00000165832:ENSG00000166483:ENSG00000198730:ENSG00000188211:ENSG00000168061:ENSG00000146670:ENSG00000149809:ENSG00000173621:ENSG00000175567:ENSG00000137492:ENSG00000149313:ENSG00000149292:ENSG00000076053:ENSG00000154127:ENSG00000166250:ENSG00000023171:ENSG00000082805:ENSG00000126749:ENSG00000134531:ENSG00000123094:ENSG00000135457:ENSG00000123364:ENSG00000123388:ENSG00000127314:ENSG00000166130:ENSG00000120868:ENSG00000111647:ENSG00000235162:ENSG00000174989:ENSG00000122966:ENSG00000150990:ENSG00000177169:ENSG00000176915:ENSG00000139505:ENSG00000133110:ENSG00000102743:ENSG00000120688:ENSG00000136167:ENSG00000102445:ENSG00000102531:ENSG00000123179:ENSG00000102753:ENSG00000118939:ENSG00000136153:ENSG00000088451:ENSG00000152749:ENSG00000134901:ENSG00000134897:ENSG00000134899:ENSG00000102524:ENSG00000100889:ENSG00000092140:ENSG00000092108:ENSG00000129493:ENSG00000129480:ENSG00000179476:ENSG00000187790:ENSG00000139926:ENSG00000197930:ENSG00000126787:ENSG00000119599:ENSG00000100767:ENSG00000133961:ENSG00000165417:ENSG00000165934:ENSG00000165943:ENSG00000170270:ENSG00000140157:ENSG00000187951:ENSG00000021776:ENSG00000198146:ENSG00000243708:ENSG00000243789:ENSG00000197535:ENSG00000181827:ENSG00000140262:ENSG00000103710:ENSG00000136378:ENSG00000103888:ENSG00000117899:ENSG00000140511:ENSG00000185418:ENSG00000103512:ENSG00000102879:ENSG00000180096:ENSG00000140682:ENSG00000103064:ENSG00000103061:ENSG00000225614:ENSG00000158717:ENSG00000167515:ENSG00000167522:ENSG00000127804:ENSG00000196689:ENSG00000005100:ENSG00000072849:ENSG00000128482:ENSG00000142494:ENSG00000067191:ENSG00000012048:ENSG00000182963:ENSG00000011260:ENSG00000153933:ENSG00000121060:ENSG00000108423:ENSG00000198265:ENSG00000197170:ENSG00000141540:ENSG00000078687:ENSG00000167302:ENSG00000224877:ENSG00000017797:ENSG00000154845:ENSG00000141401:ENSG00000152223:ENSG00000081913:ENSG00000197971:ENSG00000071564:ENSG00000167670:ENSG00000171105:ENSG00000132024:ENSG00000132000:ENSG00000123146:ENSG00000099308:ENSG00000128626:ENSG00000167625:ENSG00000105429:ENSG00000105427:ENSG00000079435:ENSG00000187244:ENSG00000105483:ENSG00000105464:ENSG00000105447:ENSG00000198464:ENSG00000176293:ENSG00000115761:ENSG00000197872:ENSG00000143867:ENSG00000084676:ENSG00000171103:ENSG00000162928:ENSG00000082898:ENSG00000116039:ENSG00000144048:ENSG00000163170:ENSG00000065911:ENSG00000005448:ENSG00000114993:ENSG00000153107:ENSG00000153214:ENSG00000115963:ENSG00000153250:ENSG00000197713:ENSG00000115461:ENSG00000168955:ENSG00000123977:ENSG00000198612:ENSG00000144485:ENSG00000132326:ENS

						G00000162804:ENSG00000125779:ENSG00000089048:ENSG00000188559:ENSG00000124102:ENSG00000124107:ENSG00000124201:ENSG00000203896:ENSG00000155313:ENSG00000183628:ENSG00000184792:ENSG00000100342:ENSG00000179750:ENSG00000100321:ENSG00000198951:ENSG00000177096:ENSG00000183172:ENSG00000075218:ENSG00000205593:ENSG00000130489:ENSG00000177989:ENSG00000205560:ENSG00000251322:ENSG00000070950:ENSG00000134077:ENSG00000163517:ENSG00000170293:ENSG00000177873:ENSG00000182983:ENSG00000114378:ENSG00000114395:ENSG00000163946:ENSG00000144749:ENSG00000114861:ENSG00000144736:ENSG00000163320:ENSG00000175105:ENSG00000179021:ENSG00000184500:ENSG00000169379:ENSG00000178700:ENSG00000163607:ENSG00000163608:ENSG00000144843:ENSG00000082701:ENSG00000240682:ENSG00000017260:ENSG00000129048:ENSG00000144867:ENSG00000154917:ENSG00000158163:ENSG00000138231:ENSG00000175110:ENSG00000174899:ENSG00000075420:ENSG00000145014:ENSG00000169019:ENSG00000145246:ENSG00000080493:ENSG00000169429:ENSG00000124875:ENSG00000109272:ENSG00000163739:ENSG00000173085:ENSG00000145331:ENSG00000205403:ENSG00000113360:ENSG00000082213:ENSG00000132357:ENSG00000067248:ENSG00000039123:ENSG00000068796:ENSG00000086189:ENSG00000086200:ENSG00000164338:ENSG00000153140:ENSG00000181751:ENSG00000168916:ENSG00000253159:ENSG00000240184:ENSG00000164620:ENSG00000120318:ENSG00000113272:ENSG00000113742:ENSG00000048140:ENSG00000131446:ENSG00000137266:ENSG00000124787:ENSG00000078401:ENSG00000197935:ENSG00000146112:ENSG00000137404:ENSG00000244731:ENSG00000235863:ENSG00000204220:ENSG00000237441:ENSG00000137168:ENSG00000164530:ENSG00000118432:ENSG00000132429:ENSG00000198945:ENSG00000112406:ENSG00000120278:ENSG00000131018:ENSG00000272047:ENSG00000186340:ENSG00000184786:ENSG00000130023:ENSG00000105855:ENSG00000253293:ENSG00000005073:ENSG00000241644:ENSG00000168303:ENSG00000152926:ENSG00000126522:ENSG00000169902:ENSG00000189143:ENSG00000049541:ENSG00000177679:ENSG00000135218:ENSG00000127920:ENSG00000105829:ENSG00000121716:ENSG00000085514:ENSG00000078487:ENSG00000166924:ENSG00000005249:ENSG00000196937:ENSG00000106477:ENSG00000106484:ENSG00000158623:ENSG00000131558:ENSG00000182158:ENSG00000146966:ENSG00000181444:ENSG00000146918:ENSG00000154319:ENSG00000147465:ENSG00000254087:ENSG00000035681:ENSG00000121039:ENSG00000120526:ENSG00000196378:ENSG00000080608:ENSG00000044459:ENSG00000178031:ENSG00000213694:ENSG00000070159:ENSG00000243444:ENSG00000182752:ENSG00000119446:ENSG00000196152:ENSG00000148356:ENSG00000136840:ENSG00000167106:ENSG00000171169:ENSG00000148339:ENSG00000148335:ENSG00000170835:ENSG00000198113
DEG.twoside	Cells_EBV-transformed_lymphocytes	7523	415	3.32038632421478e-07	1.79300861507598e-05	ENSG00000158292:ENSG00000097021:ENSG00000158747:ENSG00000187942:ENSG00000160050:ENSG00000116525:ENSG00000160094:ENSG00000134686:ENSG00000177181:ENSG00000117395:ENSG00000243710:ENSG00000066056:ENSG00000117399:ENSG00000123473:ENSG00000198841:ENSG00000116205:ENSG00000162616:ENSG00000117133:ENSG00000142867:ENSG00000117569:ENSG00000117543:ENSG00000116396:ENSG00000162775:ENSG00000168679:ENSG00000085465:ENSG00000116455:ENSG00000213281:ENSG00000052723:ENSG00000143457:ENSG00000163131:ENSG00000143387:ENSG00000143379:ENSG00000143412:ENSG00000143409:ENSG00000163154:ENSG00000163156:ENSG00000197084:ENSG00000143537:ENSG00000143630:ENSG00000160753:ENSG00000143622:ENSG00000196189:ENSG00000242252:ENSG00000160781:ENSG00000132694:ENSG00000253831:ENSG00000171786:ENSG00000143226:ENSG00000132185:ENSG00000162746:ENSG00000152332:ENSG00000186283:ENSG00000143322:ENSG00000057252:ENSG00000066279:ENSG00000174307:ENSG00000143801:ENSG00000163050:ENSG00000116962:ENSG00000015171:ENSG00000107929:ENSG00000198879:ENSG00000134463:ENSG00000065328:ENSG00000148468:ENSG00000165997:ENSG00000108094:ENSG00000107738:ENSG00000138134:ENSG00000107796:ENSG00000026103:ENSG00000059573:ENSG00000119927:ENSG00000165832:ENSG00000184545:ENSG00000166311:ENSG00000166313:ENSG00000166483:ENSG00000198730:ENSG00000162174:ENSG00000110025:ENSG00000168061:ENSG00000146670:ENSG00000173599:ENSG00000175567:ENSG00000149313:ENSG00000110777:ENSG00000076043:ENSG00000154127:ENSG00000082805:ENSG00000139180:ENSG00000111679:ENSG00000126749:ENSG00000182326:ENSG00000185432:ENSG00000110911:ENSG00000110925:ENSG00000135457:ENSG00000123338:ENSG00000123360:ENSG00000127314:ENSG00000205057:ENSG00000166130:ENSG00000120868:ENSG00000235162:ENSG00000139410:ENSG00000122966:ENSG00000150990:ENSG00000184992:ENSG00000177169:ENSG00000177084:ENSG00000176915:ENSG00000183722:ENSG00000102743:ENSG00000136167:ENSG00000102445:ENSG00000102531:ENSG00000152213:ENSG00000102753:ENSG00000136104:ENSG00000118939:ENSG00000178695:ENSG00000088451:ENSG00000152749:ENSG00000134899:ENSG00000102524:ENSG00000100889:ENSG00000092140:ENSG00000092108:ENSG00000100473:ENSG00000129493:ENSG00000129480:ENSG00000165355:ENSG00000179476:ENSG00000179454:ENSG00000187790:ENSG00000139926:ENSG00000197930:ENSG00000126787:ENSG00000119599:ENSG00000100767:ENSG00000165417:ENSG00000165934:ENSG00000170270:ENSG00000012963:ENSG00000140157:ENSG00000170113:ENSG00000187951:ENSG00000021776:ENSG00000198146:ENSG00000174197:ENSG00000243708:ENSG00000197535:ENSG00000181827:ENSG00000103707:ENSG00000140350:ENSG00000136378:ENSG00000140511:ENSG00000140471:ENSG00000008710:ENSG00000007038:ENSG00000183426:ENSG00000085721:ENSG00000157106:ENSG00000102879:ENSG00000169627:ENSG00000180096:ENSG00000179965:ENSG00000179958:ENSG00000140682:ENSG00000197302:ENSG00000102970:ENSG00000072736:ENSG00000103066:ENSG00000103064:ENSG00000103061:ENSG00000103056:ENSG00000185669:ENSG00000127804:ENSG00000196689:ENSG00000029725:ENSG00000005100:ENSG00000141127:ENSG00000072134:ENSG00000108641:ENSG00000166484:ENSG00000142494:ENSG00000067191:ENSG00000161405:ENSG00000186075:ENSG00000068079:ENSG00000108828:ENSG00000012048:ENSG00000184988:ENSG00000225190:ENSG00000198933:ENSG00000073861:ENSG00000011260:ENSG00000153933:ENSG00000121060:ENSG00000189050:ENSG00000197170:ENSG00000180616:ENSG00000141540:ENSG00000141576:ENSG00000078

						687:ENSG00000224877:ENSG00000186765:ENSG00000154845:ENSG00000141401:ENSG00000168234:ENSG00000141384:ENSG00000130856:ENSG00000071564:ENSG00000167670:ENSG00000205744:ENSG00000263264:ENSG00000104880:ENSG00000104883:ENSG00000132017:ENSG00000123146:ENSG00000123159:ENSG00000096996:ENSG00000099308:ENSG00000237440:ENSG00000262484:ENSG00000104835:ENSG00000128626:ENSG00000269190:ENSG00000105369:ENSG00000028277:ENSG00000167625:ENSG00000079462:ENSG00000105429:ENSG00000079435:ENSG00000105483:ENSG00000105447:ENSG00000063127:ENSG00000196214:ENSG00000198464:ENSG00000167766:ENSG00000213020:ENSG00000196417:ENSG00000115761:ENSG00000171103:ENSG00000119866:ENSG00000082898:ENSG00000144034:ENSG00000144048:ENSG00000163170:ENSG00000065911:ENSG00000005448:ENSG00000198885:ENSG00000115604:ENSG00000153107:ENSG00000153208:ENSG00000153214:ENSG00000163040:ENSG00000115963:ENSG00000144283:ENSG00000153250:ENSG00000118260:ENSG00000144401:ENSG00000197713:ENSG00000115457:ENSG00000135912:ENSG00000153832:ENSG00000144485:ENSG00000162804:ENSG00000125779:ENSG00000089048:ENSG00000198959:ENSG00000124107:ENSG00000124201:ENSG00000124256:ENSG00000203896:ENSG00000154642:ENSG00000160190:ENSG00000100342:ENSG00000179750:ENSG00000100321:ENSG00000198951:ENSG00000177096:ENSG00000100197:ENSG00000075218:ENSG00000100416:ENSG00000205593:ENSG00000130489:ENSG00000177989:ENSG00000130487:ENSG00000205560:ENSG00000070950:ENSG00000134077:ENSG00000163517:ENSG00000163513:ENSG00000177873:ENSG00000068001:ENSG00000114395:ENSG00000126062:ENSG00000163946:ENSG00000144749:ENSG00000144736:ENSG00000163320:ENSG00000175105:ENSG00000179021:ENSG00000114446:ENSG00000163606:ENSG00000163607:ENSG00000163608:ENSG00000144843:ENSG00000082701:ENSG00000240682:ENSG00000017260:ENSG00000113971:ENSG00000081307:ENSG00000144867:ENSG00000138231:ENSG00000175110:ENSG00000175066:ENSG00000114127:ENSG00000075420:ENSG00000172578:ENSG00000163898:ENSG00000145014:ENSG00000041802:ENSG00000163975:ENSG00000013288:ENSG00000169019:ENSG00000109321:ENSG00000145287:ENSG00000173085:ENSG00000151247:ENSG00000145331:ENSG00000123739:ENSG00000113360:ENSG00000082213:ENSG00000164190:ENSG00000171522:ENSG00000132357:ENSG00000039123:ENSG00000067113:ENSG00000068796:ENSG00000086189:ENSG00000086200:ENSG00000164338:ENSG00000164180:ENSG00000153140:ENSG00000175471:ENSG00000181751:ENSG00000164241:ENSG00000253159:ENSG00000240184:ENSG00000164620:ENSG00000131507:ENSG00000113721:ENSG00000113272:ENSG00000048140:ENSG00000137266:ENSG00000146112:ENSG00000137404:ENSG00000137337:ENSG00000204344:ENSG00000204220:ENSG00000237441:ENSG00000124772:ENSG00000137168:ENSG00000198663:ENSG00000118503:ENSG00000112406:ENSG00000120278:ENSG00000131018:ENSG00000112096:ENSG00000026297:ENSG00000213066:ENSG00000122644:ENSG00000168303:ENSG00000152926:ENSG00000146757:ENSG00000169902:ENSG00000086730:ENSG00000049541:ENSG00000085563:ENSG00000127920:ENSG00000105829:ENSG00000160813:ENSG00000105865:ENSG00000075790:ENSG00000106484:ENSG00000158623:ENSG00000122786:ENSG00000182158:ENSG00000214106:ENSG00000146918:ENSG00000136573:ENSG00000120910:ENSG00000129691:ENSG00000023287:ENSG00000254087:ENSG00000035681:ENSG00000121039:ENSG00000104267:ENSG00000164949:ENSG00000120526:ENSG00000120533:ENSG00000185189:ENSG00000196378:ENSG00000080608:ENSG00000044459:ENSG00000167081:ENSG00000196152:ENSG00000136840:ENSG00000171169:ENSG00000170835:ENSG00000148411:ENSG00000198113
DEG.twoside	Cervix_Ectocervix	197	15	0.0310111520528648	1	ENSG00000143387:ENSG00000160753:ENSG00000132680:ENSG00000122176:ENSG00000123179:ENSG00000139926:ENSG00000243708:ENSG00000072134:ENSG00000166484:ENSG00000091831:ENSG00000186340:ENSG00000105855:ENSG00000005073:ENSG00000106031:ENSG00000148339
DEG.twoside	Cervix_Endocervix	1854	121	1.61642179783885e-05	0.000872867770832978	ENSG00000162458:ENSG00000117569:ENSG00000162694:ENSG00000116396:ENSG00000143457:ENSG00000143387:ENSG00000143437:ENSG00000143379:ENSG00000143363:ENSG00000163155:ENSG00000160753:ENSG00000116580:ENSG00000132718:ENSG00000132680:ENSG00000187800:ENSG00000186283:ENSG00000122176:ENSG00000162714:ENSG00000148468:ENSG00000165338:ENSG00000168060:ENSG00000162300:ENSG00000137492:ENSG00000076053:ENSG00000166250:ENSG00000134532:ENSG00000123094:ENSG00000166130:ENSG00000074590:ENSG00000174989:ENSG00000188735:ENSG00000102531:ENSG00000123179:ENSG00000178695:ENSG00000088451:ENSG00000134901:ENSG00000134897:ENSG00000092108:ENSG00000165379:ENSG00000185246:ENSG00000100767:ENSG00000133961:ENSG00000174197:ENSG00000243708:ENSG00000140262:ENSG00000103707:ENSG00000117899:ENSG00000140682:ENSG00000132600:ENSG00000172137:ENSG00000127804:ENSG00000072134:ENSG00000166484:ENSG00000142494:ENSG00000068079:ENSG00000184988:ENSG00000153933:ENSG00000141576:ENSG00000185504:ENSG00000130856:ENSG00000071564:ENSG00000132017:ENSG00000132005:ENSG00000123146:ENSG00000269190:ENSG00000079462:ENSG00000196214:ENSG00000167766:ENSG00000213020:ENSG00000196208:ENSG00000084676:ENSG00000075292:ENSG00000144283:ENSG00000153250:ENSG00000118260:ENSG00000162804:ENSG00000088876:ENSG00000100321:ENSG00000100324:ENSG00000177096:ENSG00000138944:ENSG00000205643:ENSG00000075234:ENSG00000182983:ENSG00000180376:ENSG00000163946:ENSG00000144749:ENSG00000163320:ENSG00000114446:ENSG00000163608:ENSG00000113971:ENSG00000158163:ENSG00000013288:ENSG00000039123:ENSG00000164180:ENSG00000120318:ENSG00000196670:ENSG00000137404:ENSG00000137337:ENSG00000198945:ENSG00000091831:ENSG00000186340:ENSG00000253293:ENSG00000005073:ENSG00000106031:ENSG00000153814:ENSG00000169902:ENSG00000135218:ENSG00000127920:ENSG00000160813:ENSG00000075790:ENSG00000182158:ENSG00000146966:ENSG00000035681:ENSG00000147601:ENSG00000164949:ENSG00000196378:ENSG00000044459:ENSG00000136925:ENSG00000182752:ENSG00000148339

DEG.twoside	Colon_Sigmoid	3329	182	0.00205075908269947	0.110740990465771	ENSG00000171819:ENSG00000162458:ENSG00000187942:ENSG00000127603:ENSG00000179178:ENSG00000253313:ENSG00000117399:ENSG00000162366:ENSG00000134709:ENSG00000117155:ENSG00000197147:ENSG00000117569:ENSG00000134215:ENSG00000175984:ENSG00000143412:ENSG00000163354:ENSG00000160767:ENSG00000196189:ENSG00000242252:ENSG00000163472:ENSG00000122176:ENSG00000163050:ENSG00000116962:ENSG00000134463:ENSG00000165476:ENSG00000075073:ENSG00000107736:ENSG00000120057:ENSG00000203867:ENSG00000119927:ENSG00000006071:ENSG00000135373:ENSG00000019505:ENSG00000149150:ENSG00000162174:ENSG00000110025:ENSG00000168060:ENSG00000146670:ENSG00000149809:ENSG00000173599:ENSG00000062282:ENSG00000110777:ENSG00000154127:ENSG00000166250:ENSG00000064309:ENSG00000111679:ENSG00000123094:ENSG00000123338:ENSG00000123360:ENSG00000235162:ENSG00000074590:ENSG00000139410:ENSG00000184992:ENSG00000127863:ENSG00000102743:ENSG00000136167:ENSG00000102445:ENSG00000136104:ENSG00000136153:ENSG00000102524:ENSG00000100889:ENSG00000179476:ENSG00000258987:ENSG00000156414:ENSG00000128731:ENSG00000103710:ENSG00000117971:ENSG00000140511:ENSG00000183426:ENSG00000180209:ENSG00000180035:ENSG00000140682:ENSG00000103067:ENSG00000185669:ENSG00000132514:ENSG00000065325:ENSG00000233670:ENSG00000188522:ENSG00000128482:ENSG00000142494:ENSG00000067191:ENSG00000141750:ENSG00000068079:ENSG00000182963:ENSG00000123700:ENSG00000196169:ENSG00000141469:ENSG00000197971:ENSG00000205744:ENSG00000104833:ENSG00000263264:ENSG00000104880:ENSG00000129354:ENSG00000099308:ENSG00000179751:ENSG00000105369:ENSG00000105427:ENSG00000079435:ENSG00000142273:ENSG00000143867:ENSG00000163803:ENSG00000171094:ENSG00000119866:ENSG00000116039:ENSG00000115602:ENSG00000115604:ENSG00000115607:ENSG00000231672:ENSG00000127831:ENSG00000144485:ENSG00000162804:ENSG00000188559:ENSG00000125966:ENSG00000124102:ENSG00000183628:ENSG00000184792:ENSG00000100342:ENSG00000177096:ENSG00000100197:ENSG00000138944:ENSG00000205643:ENSG00000205560:ENSG00000170293:ENSG00000179564:ENSG00000114378:ENSG00000114395:ENSG00000007402:ENSG00000144749:ENSG00000114861:ENSG00000175066:ENSG00000169282:ENSG00000172578:ENSG00000163898:ENSG00000114279:ENSG00000109171:ENSG00000080493:ENSG00000169429:ENSG00000163739:ENSG00000145287:ENSG00000185477:ENSG00000164128:ENSG00000109466:ENSG00000171522:ENSG00000132357:ENSG00000181751:ENSG00000253159:ENSG00000131504:ENSG00000164620:ENSG00000113722:ENSG00000135074:ENSG00000113272:ENSG00000155858:ENSG00000079691:ENSG00000244731:ENSG00000124772:ENSG00000243955:ENSG00000118432:ENSG00000135525:ENSG00000253293:ENSG00000005073:ENSG00000106031:ENSG00000153814:ENSG00000189143:ENSG00000086730:ENSG00000177679:ENSG00000166924:ENSG00000106484:ENSG00000182158:ENSG00000254087:ENSG00000121039:ENSG00000104267:ENSG00000079112:ENSG00000164949:ENSG00000165071:ENSG00000044459:ENSG00000127080:ENSG00000136936:ENSG00000070159:ENSG00000167081:ENSG00000148356:ENSG00000148411:ENSG00000198113
DEG.twoside	Colon_Transverse	3200	187	6.49857791436746e-05	0.00350923207375843	ENSG00000162460:ENSG00000162458:ENSG00000160050:ENSG00000116525:ENSG00000179178:ENSG00000253313:ENSG00000117399:ENSG00000117472:ENSG00000162366:ENSG00000186564:ENSG00000162390:ENSG00000162391:ENSG00000203943:ENSG00000142867:ENSG00000197147:ENSG00000069712:ENSG00000134215:ENSG00000175984:ENSG00000092621:ENSG00000134240:ENSG00000163131:ENSG00000143412:ENSG00000143434:ENSG00000196189:ENSG00000163472:ENSG00000187800:ENSG00000173110:ENSG00000203747:ENSG00000134463:ENSG00000075073:ENSG00000107736:ENSG00000059573:ENSG00000120057:ENSG00000119927:ENSG00000187908:ENSG00000187486:ENSG00000006071:ENSG00000135373:ENSG00000165905:ENSG00000168060:ENSG00000149809:ENSG00000173599:ENSG00000175564:ENSG00000166391:ENSG00000062282:ENSG00000149313:ENSG00000110777:ENSG00000154127:ENSG00000166250:ENSG00000010626:ENSG00000013588:ENSG00000134532:ENSG00000123360:ENSG00000074590:ENSG00000111725:ENSG00000188735:ENSG00000184992:ENSG00000127863:ENSG00000102445:ENSG00000102524:ENSG00000100889:ENSG00000139914:ENSG00000100473:ENSG00000006432:ENSG00000198146:ENSG00000137802:ENSG00000243789:ENSG00000197535:ENSG00000186198:ENSG00000103888:ENSG00000140511:ENSG00000103067:ENSG00000103066:ENSG00000103056:ENSG00000132514:ENSG00000065325:ENSG00000233670:ENSG00000181350:ENSG00000108641:ENSG00000142494:ENSG00000067191:ENSG00000141750:ENSG00000108830:ENSG00000182963:ENSG00000154217:ENSG00000180616:ENSG00000141540:ENSG00000078687:ENSG00000168234:ENSG00000141469:ENSG00000197971:ENSG00000263264:ENSG00000104880:ENSG00000129354:ENSG00000132000:ENSG00000123146:ENSG00000262484:ENSG00000105369:ENSG00000105427:ENSG00000079435:ENSG00000189377:ENSG00000079385:ENSG00000142273:ENSG00000063127:ENSG00000163803:ENSG00000188687:ENSG00000005448:ENSG00000115607:ENSG00000163040:ENSG00000144283:ENSG00000018280:ENSG00000127831:ENSG00000168955:ENSG00000153832:ENSG00000144485:ENSG00000162804:ENSG00000124102:ENSG00000160190:ENSG00000184792:ENSG00000179750:ENSG00000100321:ENSG00000138944:ENSG00000075234:ENSG00000075275:ENSG00000205593:ENSG00000008735:ENSG00000144712:ENSG00000170293:ENSG00000007402:ENSG00000178750:ENSG00000144852:ENSG00000114670:ENSG00000129048:ENSG00000154917:ENSG00000158186:ENSG00000169282:ENSG00000174899:ENSG00000114248:ENSG00000163898:ENSG00000145014:ENSG00000080493:ENSG00000169429:ENSG00000163739:ENSG00000145287:ENSG00000164128:ENSG00000109466:ENSG00000174358:ENSG00000113361:ENSG00000171522:ENSG00000132357:ENSG00000152669:ENSG00000175471:ENSG00000181751:ENSG00000064651:ENSG00000253159:ENSG00000240184:ENSG00000164620:ENSG00000186314:ENSG00000113722:ENSG00000135074:ENSG00000113742:ENSG00000137266:ENSG00000112812:ENSG00000124772:ENSG00000132429:ENSG00000131018:ENSG00000105855:ENSG00000253293:ENSG00000005073:ENSG00000106031:ENSG00000152926:ENSG00000213462:ENSG000001265

						22:ENSG00000165215:ENSG00000189143:ENSG00000177679:ENSG00000085563:ENSG00000085514:ENSG00000078487:ENSG00000166924:ENSG00000104267:ENSG00000079112:ENSG00000131773:ENSG00000127080:ENSG00000130958:ENSG00000170835:ENSG00000198113
DEG.twoside	Esophagus_Gastroesophageal_Junction	2861	158	0.00276168273847204	0.14913086787749	ENSG00000171819:ENSG00000162458:ENSG00000187942:ENSG00000168389:ENSG00000117472:ENSG00000162390:ENSG00000117114:ENSG00000153904:ENSG00000197147:ENSG00000069712:ENSG00000117569:ENSG00000134215:ENSG00000175984:ENSG00000092621:ENSG00000143412:ENSG00000203786:ENSG00000163354:ENSG00000196189:ENSG00000160781:ENSG00000163472:ENSG00000173110:ENSG00000203747:ENSG00000116962:ENSG00000134463:ENSG00000075073:ENSG00000107736:ENSG00000165338:ENSG00000120057:ENSG00000119927:ENSG00000198873:ENSG00000187486:ENSG00000149150:ENSG00000162174:ENSG00000168060:ENSG00000149809:ENSG00000173599:ENSG00000062282:ENSG00000137672:ENSG00000166250:ENSG00000111679:ENSG00000087494:ENSG00000235162:ENSG00000139410:ENSG00000184992:ENSG00000187848:ENSG00000102743:ENSG00000136167:ENSG00000102445:ENSG00000184226:ENSG00000118939:ENSG00000136153:ENSG00000178695:ENSG00000139914:ENSG00000168952:ENSG00000179476:ENSG00000006432:ENSG00000100767:ENSG00000258987:ENSG00000156414:ENSG00000103710:ENSG00000251209:ENSG00000183426:ENSG00000102879:ENSG00000180096:ENSG00000140682:ENSG00000103066:ENSG00000103056:ENSG00000172137:ENSG00000065325:ENSG00000181350:ENSG00000188522:ENSG00000067191:ENSG00000108830:ENSG00000073670:ENSG00000182963:ENSG00000180616:ENSG00000141576:ENSG00000141401:ENSG00000197971:ENSG00000205744:ENSG00000104833:ENSG00000132016:ENSG00000099308:ENSG00000262484:ENSG00000179751:ENSG00000079462:ENSG00000079435:ENSG00000189377:ENSG00000079385:ENSG00000105492:ENSG00000115705:ENSG00000115756:ENSG00000143867:ENSG00000119866:ENSG00000116039:ENSG00000144218:ENSG00000115607:ENSG00000153208:ENSG00000125966:ENSG00000124102:ENSG00000124107:ENSG00000160190:ENSG00000183628:ENSG00000184792:ENSG00000177096:ENSG00000100197:ENSG00000138944:ENSG00000075275:ENSG00000008735:ENSG00000170293:ENSG00000168032:ENSG00000114395:ENSG00000007402:ENSG00000144749:ENSG00000114861:ENSG00000169282:ENSG00000080493:ENSG00000169429:ENSG00000163739:ENSG00000173085:ENSG00000185477:ENSG00000016082:ENSG00000064651:ENSG00000164620:ENSG00000135074:ENSG00000155858:ENSG00000137266:ENSG00000255587:ENSG00000124772:ENSG00000164530:ENSG00000132429:ENSG00000135525:ENSG00000091831:ENSG00000026297:ENSG00000153814:ENSG00000241644:ENSG00000189143:ENSG00000086730:ENSG00000177679:ENSG00000185955:ENSG00000166924:ENSG00000005249:ENSG00000106484:ENSG00000158623:ENSG00000146966:ENSG00000254087:ENSG00000121039:ENSG00000104267:ENSG00000164949:ENSG00000129295:ENSG00000178031:ENSG00000186354:ENSG00000213694:ENSG00000127080:ENSG00000070159:ENSG00000167081:ENSG00000170835:ENSG00000148411
DEG.twoside	Esophagus_Mucosa	5849	328	2.40207677353124e-06	0.000129712145770687	ENSG00000158286:ENSG00000069812:ENSG00000171819:ENSG00000187942:ENSG00000160050:ENSG00000160051:ENSG00000127603:ENSG00000253313:ENSG00000066056:ENSG00000117399:ENSG00000162366:ENSG00000162367:ENSG00000123473:ENSG00000162390:ENSG00000134709:ENSG00000162616:ENSG00000142867:ENSG00000153904:ENSG00000171488:ENSG00000069712:ENSG00000117569:ENSG00000162694:ENSG00000134215:ENSG00000168679:ENSG00000116455:ENSG00000175984:ENSG00000213281:ENSG00000143457:ENSG00000143452:ENSG00000143412:ENSG00000143409:ENSG00000143434:ENSG00000163154:ENSG00000143631:ENSG00000203786:ENSG00000132718:ENSG00000143622:ENSG00000116584:ENSG00000196189:ENSG00000160781:ENSG00000163472:ENSG00000187800:ENSG00000143226:ENSG00000173110:ENSG00000203747:ENSG00000120341:ENSG00000143322:ENSG00000057252:ENSG00000066279:ENSG00000163050:ENSG00000162714:ENSG00000198879:ENSG00000065328:ENSG00000148468:ENSG00000241058:ENSG00000095794:ENSG00000075073:ENSG00000107736:ENSG00000165338:ENSG00000120057:ENSG00000203867:ENSG00000119927:ENSG00000198873:ENSG00000184545:ENSG00000166313:ENSG00000187486:ENSG00000129151:ENSG00000135373:ENSG00000165905:ENSG00000149150:ENSG00000124935:ENSG00000162174:ENSG00000168060:ENSG00000146670:ENSG00000149809:ENSG00000173653:ENSG00000173621:ENSG00000175567:ENSG00000137492:ENSG00000180425:ENSG00000154127:ENSG00000064309:ENSG00000010626:ENSG00000111674:ENSG00000134531:ENSG00000123094:ENSG00000087494:ENSG00000110925:ENSG00000123360:ENSG00000166130:ENSG00000111647:ENSG00000074590:ENSG00000139410:ENSG00000122966:ENSG00000188735:ENSG00000184992:ENSG00000187848:ENSG00000139505:ENSG00000133110:ENSG00000136167:ENSG00000102531:ENSG00000123179:ENSG00000118939:ENSG00000136153:ENSG00000178695:ENSG00000134901:ENSG00000102524:ENSG00000139914:ENSG00000168952:ENSG00000150527:ENSG00000139926:ENSG00000087301:ENSG00000197930:ENSG00000126787:ENSG00000006432:ENSG00000100767:ENSG00000165943:ENSG00000187951:ENSG00000198146:ENSG00000137802:ENSG00000243708:ENSG00000243789:ENSG00000197535:ENSG00000136378:ENSG00000103888:ENSG00000140600:ENSG00000185418:ENSG00000008710:ENSG00000180035:ENSG00000179965:ENSG00000102921:ENSG00000102970:ENSG00000103067:ENSG00000103066:ENSG00000103064:ENSG00000132600:ENSG00000103056:ENSG00000185669:ENSG00000158717:ENSG00000196689:ENSG00000029725:ENSG00000188522:ENSG00000108641:ENSG00000166484:ENSG00000128482:ENSG00000142494:ENSG00000067191:ENSG00000141750:ENSG00000068079:ENSG00000108830:ENSG00000184988:ENSG00000182963:ENSG00000225190:ENSG00000008294:ENSG00000108423:ENSG00000154217:ENSG00000141540:ENSG00000078687:ENSG00000154845:ENSG00000141401:ENSG00000206073:ENSG00000197971:ENSG00000167670:ENSG00000171105:ENSG00000167775:ENSG00000065989:ENSG00000180739:ENSG00000129354:ENSG00000123146:ENSG00000123143:ENSG00000099308:ENSG00000237440:ENSG00000269190:ENSG00000028277:ENSG00000079462:ENSG00000105429:ENSG00000105427:ENSG00000079435:ENSG00000189377:ENSG00000079385:ENSG00000142273:ENSG00000105483:ENSG00000063127:ENSG00000105383:ENSG00000176293:ENSG00000115705:ENSG00000115756:ENSG00000197872:ENSG00000143867:ENSG

						00000163803:ENSG00000119866:ENSG00000144048:ENSG00000005448:ENSG00000144218:ENSG00000115602:ENSG00000115604:ENSG00000115607:ENSG00000180251:ENSG00000153208:ENSG00000163040:ENSG00000115963:ENSG00000018280:ENSG00000153832:ENSG00000198612:ENSG00000144485:ENSG00000162804:ENSG00000196209:ENSG00000101290:ENSG00000175121:ENSG00000168703:ENSG00000124102:ENSG00000124107:ENSG00000124201:ENSG00000160190:ENSG00000160284:ENSG00000183628:ENSG00000184792:ENSG00000138944:ENSG00000075218:ENSG00000075275:ENSG00000205593:ENSG00000130489:ENSG00000008735:ENSG00000251322:ENSG00000144712:ENSG00000197385:ENSG00000170293:ENSG00000170011:ENSG00000168032:ENSG00000114378:ENSG00000180376:ENSG00000144749:ENSG00000169379:ENSG00000178750:ENSG00000114446:ENSG00000163606:ENSG00000144843:ENSG00000172780:ENSG00000114670:ENSG00000113971:ENSG00000144867:ENSG00000154917:ENSG00000158163:ENSG00000158186:ENSG00000114698:ENSG00000163762:ENSG00000169282:ENSG00000075420:ENSG00000172578:ENSG00000163898:ENSG00000152953:ENSG00000013288:ENSG00000109689:ENSG00000169019:ENSG00000169429:ENSG00000163739:ENSG00000109321:ENSG00000145287:ENSG00000185477:ENSG00000164128:ENSG00000113361:ENSG00000171522:ENSG00000132357:ENSG00000197822:ENSG00000175471:ENSG00000181751:ENSG00000168916:ENSG00000173926:ENSG00000064651:ENSG00000253159:ENSG00000120318:ENSG00000135074:ENSG00000137266:ENSG00000124787:ENSG00000078401:ENSG00000112812:ENSG00000137337:ENSG00000204544:ENSG00000244731:ENSG00000235863:ENSG00000124772:ENSG00000164530:ENSG00000118432:ENSG00000198945:ENSG00000135525:ENSG00000118503:ENSG00000131018:ENSG00000122644:ENSG00000153814:ENSG00000241644:ENSG00000213462:ENSG00000146757:ENSG00000165215:ENSG00000189143:ENSG00000086730:ENSG00000135218:ENSG00000085563:ENSG00000127920:ENSG00000085514:ENSG00000078487:ENSG00000166924:ENSG00000005249:ENSG00000106477:ENSG00000106484:ENSG00000146966:ENSG00000159763:ENSG00000181444:ENSG00000196329:ENSG00000154319:ENSG00000120910:ENSG00000147601:ENSG00000164949:ENSG00000129295:ENSG00000165071:ENSG00000131773:ENSG00000196378:ENSG00000080608:ENSG00000044459:ENSG00000230453:ENSG00000213694:ENSG00000127080:ENSG00000070159:ENSG00000167081:ENSG00000167103:ENSG00000148335:ENSG00000148411:ENSG00000233198:ENSG00000198569
DEG.twoside	Esophagus_Muscularis	3091	164	0.0102733493321526	0.55476086393624	ENSG00000171819:ENSG00000162458:ENSG00000187942:ENSG00000127603:ENSG00000168389:ENSG00000117472:ENSG00000162390:ENSG00000117114:ENSG00000197147:ENSG00000069712:ENSG00000117525:ENSG00000117569:ENSG00000162694:ENSG00000134215:ENSG00000175984:ENSG00000143412:ENSG00000163354:ENSG00000160767:ENSG00000196189:ENSG00000160781:ENSG00000163472:ENSG00000173110:ENSG00000143801:ENSG00000163050:ENSG00000116962:ENSG00000198879:ENSG00000134463:ENSG00000075073:ENSG00000107736:ENSG00000165338:ENSG00000120057:ENSG00000119927:ENSG00000198873:ENSG00000205869:ENSG00000187486:ENSG00000162174:ENSG00000168060:ENSG00000149809:ENSG00000173599:ENSG00000062282:ENSG00000137672:ENSG00000166250:ENSG00000082805:ENSG00000111679:ENSG00000013588:ENSG00000134532:ENSG00000235162:ENSG00000184992:ENSG00000187848:ENSG00000102743:ENSG00000136167:ENSG00000102445:ENSG00000136104:ENSG00000184226:ENSG00000136153:ENSG00000178695:ENSG00000102524:ENSG00000100889:ENSG00000139914:ENSG00000168952:ENSG00000100473:ENSG00000179476:ENSG00000006432:ENSG00000258987:ENSG00000103710:ENSG00000251209:ENSG00000008710:ENSG00000102879:ENSG00000180209:ENSG00000180096:ENSG00000140682:ENSG00000103066:ENSG00000103056:ENSG00000172137:ENSG00000065325:ENSG00000181350:ENSG00000108641:ENSG00000067191:ENSG00000073670:ENSG00000182963:ENSG00000180616:ENSG00000141576:ENSG00000141401:ENSG00000197971:ENSG00000205744:ENSG00000104833:ENSG00000132016:ENSG00000099308:ENSG00000262484:ENSG00000079462:ENSG00000079435:ENSG00000079385:ENSG00000105492:ENSG00000115705:ENSG00000115756:ENSG00000143867:ENSG00000119866:ENSG00000144218:ENSG00000115607:ENSG00000144283:ENSG00000153250:ENSG00000153253:ENSG00000162804:ENSG00000101290:ENSG00000125864:ENSG00000124102:ENSG00000160190:ENSG00000183628:ENSG00000184792:ENSG00000177096:ENSG00000100197:ENSG00000138944:ENSG00000075275:ENSG00000205593:ENSG00000170293:ENSG00000168032:ENSG00000114395:ENSG00000007402:ENSG00000144749:ENSG00000114861:ENSG00000114098:ENSG00000169282:ENSG00000109171:ENSG00000080493:ENSG00000169429:ENSG00000163739:ENSG00000173085:ENSG00000185477:ENSG00000164128:ENSG00000064651:ENSG00000164620:ENSG00000135074:ENSG00000155858:ENSG00000079691:ENSG00000244731:ENSG00000124772:ENSG00000164530:ENSG00000198945:ENSG00000135525:ENSG00000026297:ENSG00000153814:ENSG00000241644:ENSG00000189143:ENSG00000086730:ENSG00000177679:ENSG00000185955:ENSG00000166924:ENSG00000005249:ENSG00000106484:ENSG00000158623:ENSG00000146966:ENSG00000254087:ENSG00000121039:ENSG00000104267:ENSG00000164949:ENSG00000129295:ENSG00000178031:ENSG00000186354:ENSG00000213694:ENSG00000127080:ENSG00000070159:ENSG00000167081:ENSG00000148411:ENSG00000198113
DEG.twoside	Fallopian_Tube	491	27	0.154365489187016	1	ENSG00000162458:ENSG00000143387:ENSG00000143437:ENSG00000122176:ENSG00000133110:ENSG00000102531:ENSG00000136104:ENSG00000178695:ENSG00000133961:ENSG00000103710:ENSG00000008710:ENSG00000140682:ENSG00000123146:ENSG00000167766:ENSG00000196208:ENSG00000162804:ENSG00000124107:ENSG00000100342:ENSG00000251322:ENSG00000169379:ENSG00000114115:ENSG00000205403:ENSG00000171522:ENSG00000086189:ENSG00000091831:ENSG00000241644:ENSG00000127920
DEG.twoside	Heart_Atrial_Appendage	9233	551	1.79903657284265e-16	9.7147974933503e-15	ENSG00000158286:ENSG00000097021:ENSG00000171824:ENSG00000198793:ENSG00000120942:ENSG00000065526:ENSG00000116809:ENSG00000117616:ENSG00000204178:ENSG00000157978:ENSG00000084652:ENSG00000160051:ENSG00000116525:ENSG00000160094:ENSG00000168389:ENSG00000117395:ENSG00000066322:ENSG00000159479:ENSG00000198198:ENSG00000162367:ENSG00000117862:ENSG00000198841:ENSG00000134717:ENSG00000116205:ENSG00000134709:ENSG00000117155:ENSG00000162642:ENSG00000142867:ENSG00000153904:ENSG00000197147:ENSG00000171488:ENSG00000069712:ENSG0000011752

	5:ENSG00000117569:ENSG00000162694:ENSG00000134215:ENSG00000116396:ENSG00000085465:ENSG00000163349:ENSG00000197323:ENSG00000175984:ENSG00000213281:ENSG00000052723:ENSG00000092621:ENSG00000143457:ENSG00000163131:ENSG00000143437:ENSG00000143379:ENSG00000143412:ENSG00000163154:ENSG00000163156:ENSG00000160767:ENSG00000176444:ENSG00000143630:ENSG00000160753:ENSG00000116539:ENSG00000125459:ENSG00000163374:ENSG00000116580:ENSG00000132718:ENSG00000143622:ENSG00000132680:ENSG00000116584:ENSG00000160803:ENSG00000196189:ENSG00000160781:ENSG00000163472:ENSG00000198715:ENSG00000132694:ENSG00000143226:ENSG00000152332:ENSG00000186283:ENSG00000143322:ENSG00000057252:ENSG00000177888:ENSG00000122176:ENSG00000182004:ENSG00000143801:ENSG00000162885:ENSG00000162714:ENSG00000107929:ENSG00000198879:ENSG00000148468:ENSG00000241058:ENSG00000099246:ENSG00000108094:ENSG00000188234:ENSG00000165476:ENSG00000075073:ENSG00000107736:ENSG00000107738:ENSG00000026103:ENSG00000165338:ENSG00000059573:ENSG00000155256:ENSG00000120057:ENSG00000203867:ENSG00000119927:ENSG00000165832:ENSG00000268403:ENSG00000166478:ENSG00000198730:ENSG00000006071:ENSG00000110442:ENSG00000121680:ENSG00000149150:ENSG00000156587:ENSG00000124935:ENSG00000162174:ENSG00000110025:ENSG00000168061:ENSG00000168060:ENSG00000162300:ENSG00000174276:ENSG00000162298:ENSG00000149792:ENSG00000173653:ENSG00000173599:ENSG00000173621:ENSG00000175567:ENSG00000062282:ENSG00000137492:ENSG00000149313:ENSG00000149292:ENSG00000180425:ENSG00000076053:ENSG00000064309:ENSG00000010219:ENSG00000111254:ENSG00000111667:ENSG00000111671:ENSG00000111679:ENSG00000126749:ENSG00000111684:ENSG00000110911:ENSG00000050426:ENSG00000110925:ENSG00000135457:ENSG00000123338:ENSG00000123360:ENSG00000127314:ENSG00000120868:ENSG00000111647:ENSG00000235162:ENSG00000135094:ENSG00000174989:ENSG00000111725:ENSG00000188735:ENSG00000150990:ENSG00000184992:ENSG00000177084:ENSG00000176915:ENSG00000127863:ENSG00000139505:ENSG00000102743:ENSG00000136167:ENSG00000102531:ENSG00000123179:ENSG00000136104:ENSG00000118939:ENSG00000136153:ENSG00000178695:ENSG00000088451:ENSG00000152749:ENSG00000139826:ENSG00000100889:ENSG00000100897:ENSG00000139914:ENSG00000092140:ENSG00000092108:ENSG00000129480:ENSG00000150527:ENSG00000165355:ENSG00000179476:ENSG00000179454:ENSG00000198718:ENSG00000185246:ENSG00000139926:ENSG00000087301:ENSG00000180998:ENSG00000197930:ENSG00000119599:ENSG00000165861:ENSG00000119707:ENSG00000080815:ENSG00000100767:ENSG00000133961:ENSG00000165417:ENSG00000165934:ENSG00000140157:ENSG00000170113:ENSG00000128731:ENSG00000021776:ENSG00000198146:ENSG00000174197:ENSG00000137802:ENSG00000243708:ENSG00000243789:ENSG00000197535:ENSG00000128989:ENSG00000181827:ENSG00000140262:ENSG00000186198:ENSG00000103710:ENSG00000136378:ENSG00000103888:ENSG00000117899:ENSG00000140406:ENSG00000140511:ENSG00000140471:ENSG00000140986:ENSG00000183426:ENSG00000157045:ENSG00000085721:ENSG00000157106:ENSG00000102879:ENSG00000169217:ENSG00000169221:ENSG00000180035:ENSG00000180096:ENSG00000179965:ENSG00000140691:ENSG00000102910:ENSG00000196470:ENSG00000102921:ENSG00000072736:ENSG00000103061:ENSG00000132600:ENSG00000103056:ENSG00000172137:ENSG00000185669:ENSG00000158717:ENSG00000167515:ENSG00000167522:ENSG00000197912:ENSG00000127804:ENSG00000197417:ENSG00000029725:ENSG00000005100:ENSG00000072849:ENSG00000132514:ENSG00000141127:ENSG00000108641:ENSG00000166484:ENSG00000128482:ENSG00000067191:ENSG00000141750:ENSG00000068079:ENSG00000108830:ENSG00000012048:ENSG00000184988:ENSG00000073670:ENSG00000225190:ENSG00000141279:ENSG00000008294:ENSG00000011258:ENSG00000011260:ENSG00000153933:ENSG00000121060:ENSG00000108423:ENSG00000108443:ENSG00000189050:ENSG00000198265:ENSG00000197170:ENSG00000154217:ENSG00000123700:ENSG00000141576:ENSG00000185262:ENSG00000078687:ENSG00000141577:ENSG00000167302:ENSG00000185504:ENSG00000182446:ENSG00000017797:ENSG00000154845:ENSG00000134265:ENSG00000154889:ENSG00000141401:ENSG00000168234:ENSG00000141384:ENSG00000152223:ENSG00000081913:ENSG00000130856:ENSG00000197971:ENSG00000071564:ENSG00000141985:ENSG00000167670:ENSG00000130254:ENSG00000205744:ENSG00000104833:ENSG00000104880:ENSG00000104883:ENSG00000065989:ENSG00000079999:ENSG00000104957:ENSG00000132024:ENSG00000132000:ENSG00000132017:ENSG00000132005:ENSG00000123136:ENSG00000099308:ENSG00000237440:ENSG00000104825:ENSG00000104835:ENSG00000269190:ENSG00000160570:ENSG00000167625:ENSG00000105723:ENSG00000079432:ENSG00000079462:ENSG00000079435:ENSG00000079385:ENSG00000185453:ENSG00000105483:ENSG00000105447:ENSG00000063127:ENSG00000142544:ENSG00000105383:ENSG00000196214:ENSG00000167766:ENSG00000213020:ENSG00000176293:ENSG00000115756:ENSG00000115761:ENSG00000196208:ENSG00000197872:ENSG00000143867:ENSG00000055917:ENSG00000084676:ENSG00000162928:ENSG00000162929:ENSG00000082898:ENSG00000116039:ENSG00000075292:ENSG00000144048:ENSG00000163170:ENSG00000114978:ENSG00000065911:ENSG00000005448:ENSG00000114993:ENSG00000144218:ENSG00000115604:ENSG00000115607:ENSG00000153107:ENSG00000153208:ENSG00000153214:ENSG00000115762:ENSG00000163040:ENSG00000144283:ENSG00000118260:ENSG00000197713:ENSG00000135912:ENSG00000153832:ENSG00000144485:ENSG00000132326:ENSG00000162804:ENSG00000196209:ENSG00000088876:ENSG00000125779:ENSG00000132646:ENSG00000089048:ENSG00000188559:ENSG00000124102:ENSG00000124107:ENSG00000124228:ENSG00000124201:ENSG00000203896:ENSG00000101152:ENSG00000155313:ENSG00000154642:ENSG00000157617:ENSG00000160190:ENSG00000160284:ENSG00000160285:ENSG00000184792:ENSG00000128335:ENSG00000100324:ENSG00000128285:ENSG00000198951:ENSG00000177096:ENSG00000242247:ENSG00000138944:ENSG00000205643:ENSG00000205593:ENSG00000025770:ENSG00000130489:ENSG00000205560:ENSG00000070950:ENSG00000134077:ENSG00000144560:ENSG00000144712:ENSG00000183873:ENSG00000170011:ENSG00000182983:ENSG00000179564:ENSG00000186792:ENSG00000243477:ENSG00000114388:ENSG00000114395:ENSG00000007402:ENSG00000180376:ENSG00000163946:ENSG00000114861:ENSG

						00000163320:ENSG00000175105:ENSG00000179021:ENSG00000184500:ENSG00000169379:ENSG00000178700:ENSG00000114446:ENSG00000163608:ENSG00000144843:ENSG00000082701:ENSG00000172780:ENSG00000240682:ENSG00000017260:ENSG00000034533:ENSG00000114670:ENSG00000113971:ENSG00000158163:ENSG00000138231:ENSG00000114098:ENSG00000158186:ENSG00000155903:ENSG00000175066:ENSG00000114127:ENSG00000163762:ENSG00000169282:ENSG00000172578:ENSG00000073803:ENSG00000163904:ENSG00000114279:ENSG00000145014:ENSG00000041802:ENSG00000163975:ENSG00000109689:ENSG00000145246:ENSG00000151247:ENSG00000145331:ENSG00000248713:ENSG00000164128:ENSG00000109466:ENSG00000129187:ENSG00000113360:ENSG00000082213:ENSG00000164190:ENSG00000171522:ENSG00000132357:ENSG00000067248:ENSG00000039123:ENSG00000068796:ENSG00000086189:ENSG00000164331:ENSG00000164338:ENSG00000164180:ENSG00000153140:ENSG00000181751:ENSG00000064651:ENSG00000240184:ENSG00000131504:ENSG00000171720:ENSG00000164620:ENSG00000197948:ENSG00000135074:ENSG00000113272:ENSG00000155858:ENSG00000074317:ENSG00000048140:ENSG00000131446:ENSG00000196670:ENSG00000124535:ENSG00000137266:ENSG00000124787:ENSG00000078401:ENSG00000079691:ENSG00000146112:ENSG00000137404:ENSG00000137337:ENSG00000204344:ENSG00000223501:ENSG00000235863:ENSG00000227057:ENSG00000204220:ENSG00000124772:ENSG00000137168:ENSG00000164530:ENSG00000124743:ENSG00000112245:ENSG00000132429:ENSG00000198945:ENSG00000112357:ENSG00000120278:ENSG00000131018:ENSG00000026297:ENSG00000213066:ENSG00000184786:ENSG00000130023:ENSG00000122644:ENSG00000105855:ENSG00000153814:ENSG00000241644:ENSG00000168303:ENSG00000152926:ENSG00000213462:ENSG00000146757:ENSG00000126522:ENSG00000169902:ENSG00000086730:ENSG00000049541:ENSG00000135218:ENSG00000105829:ENSG00000085514:ENSG00000078487:ENSG00000146834:ENSG00000160813:ENSG00000172209:ENSG00000091704:ENSG00000106484:ENSG00000158623:ENSG00000131558:ENSG00000181444:ENSG00000214106:ENSG00000146918:ENSG00000168546:ENSG00000129691:ENSG00000023287:ENSG00000254087:ENSG00000035681:ENSG00000147601:ENSG00000121039:ENSG00000120526:ENSG00000120533:ENSG00000129295:ENSG00000165071:ENSG00000131773:ENSG00000180900:ENSG00000196378:ENSG00000080608:ENSG00000044459:ENSG00000127080:ENSG00000130958:ENSG00000136936:ENSG00000070159:ENSG00000196152:ENSG00000148356:ENSG00000136908:ENSG00000167106:ENSG00000171169:ENSG00000148339:ENSG00000148308:ENSG00000148411
DEG.twoside	Heart_Left_Ventricle	10067	637	3.07079404673465e-27	1.65822878523671e-25	ENSG00000158286:ENSG00000158292:ENSG00000097021:ENSG00000171603:ENSG00000120948:ENSG00000171824:ENSG00000198793:ENSG00000171819:ENSG00000120942:ENSG00000065526:ENSG00000116809:ENSG00000187942:ENSG00000117616:ENSG00000183726:ENSG00000204178:ENSG00000157978:ENSG00000084652:ENSG00000160050:ENSG00000160051:ENSG00000116525:ENSG00000160094:ENSG00000127603:ENSG00000117395:ENSG00000066322:ENSG00000159479:ENSG00000198198:ENSG00000162367:ENSG00000117862:ENSG00000198841:ENSG00000134717:ENSG00000162390:ENSG00000162604:ENSG00000117114:ENSG00000117133:ENSG00000117155:ENSG00000162642:ENSG00000142867:ENSG00000153904:ENSG00000197147:ENSG00000171488:ENSG00000117525:ENSG00000117569:ENSG00000162694:ENSG00000116396:ENSG00000162775:ENSG00000168679:ENSG00000085465:ENSG00000163349:ENSG00000197323:ENSG00000116752:ENSG00000213281:ENSG00000052723:ENSG00000092621:ENSG00000134240:ENSG00000143457:ENSG00000163131:ENSG00000143387:ENSG00000143437:ENSG00000143379:ENSG00000143418:ENSG00000143412:ENSG00000163154:ENSG00000163156:ENSG00000163155:ENSG00000160767:ENSG00000176444:ENSG00000160752:ENSG00000160753:ENSG00000116539:ENSG00000125459:ENSG00000163374:ENSG00000116580:ENSG00000132718:ENSG00000143622:ENSG00000132680:ENSG00000116584:ENSG00000160803:ENSG00000160781:ENSG00000163472:ENSG00000198715:ENSG00000187800:ENSG00000132694:ENSG00000143226:ENSG00000173110:ENSG00000203747:ENSG00000152332:ENSG00000174175:ENSG00000120341:ENSG00000186283:ENSG00000143322:ENSG00000057252:ENSG00000177888:ENSG00000174307:ENSG00000159176:ENSG00000122176:ENSG00000182004:ENSG00000143801:ENSG00000162885:ENSG00000162714:ENSG00000015171:ENSG00000107929:ENSG00000198879:ENSG00000148468:ENSG00000241058:ENSG00000165997:ENSG00000099246:ENSG00000108094:ENSG00000095794:ENSG00000188234:ENSG00000165476:ENSG00000107736:ENSG00000107738:ENSG00000026103:ENSG00000059573:ENSG00000095587:ENSG00000155256:ENSG00000120057:ENSG00000203867:ENSG00000119927:ENSG00000165832:ENSG00000184545:ENSG00000268403:ENSG00000166478:ENSG00000198730:ENSG00000006071:ENSG00000110442:ENSG00000121680:ENSG00000149150:ENSG00000156587:ENSG00000162174:ENSG00000110025:ENSG00000168061:ENSG00000168060:ENSG00000162300:ENSG00000149823:ENSG00000174276:ENSG00000162298:ENSG00000149792:ENSG00000173653:ENSG00000173599:ENSG00000173621:ENSG00000175567:ENSG00000062282:ENSG00000137492:ENSG00000137693:ENSG00000149313:ENSG00000149292:ENSG00000180425:ENSG00000076053:ENSG00000166250:ENSG00000064309:ENSG00000082805:ENSG00000010219:ENSG00000139180:ENSG00000111667:ENSG00000111671:ENSG00000010626:ENSG00000111674:ENSG00000111679:ENSG00000126749:ENSG00000111684:ENSG00000013588:ENSG00000134531:ENSG00000123094:ENSG00000185432:ENSG00000110911:ENSG00000050426:ENSG00000110925:ENSG00000135457:ENSG00000123360:ENSG00000127314:ENSG00000166130:ENSG00000120868:ENSG00000111647:ENSG00000139410:ENSG00000174989:ENSG00000111725:ENSG00000188735:ENSG00000150990:ENSG00000184992:ENSG00000177169:ENSG00000177084:ENSG00000176915:ENSG00000139505:ENSG00000133110:ENSG00000102743:ENSG00000120688:ENSG00000136167:ENSG00000102531:ENSG00000123179:ENSG00000136104:ENSG00000188243:ENSG00000118939:ENSG00000136153:ENSG00000178695:ENSG00000088451:ENSG00000152749:ENSG00000065150:ENSG00000134901:ENSG00000139826:ENSG00000100889:ENSG00000100897:ENSG00000139914:ENSG00000092140:ENSG00000092108:ENSG00000129493:ENSG00000203546:ENSG00000129480:ENSG00000150527:ENSG00000165355:ENSG00000179476:ENSG00000179454:ENSG00000198718:ENSG00000185246:ENSG00000139926:ENSG00000087301:ENSG00000197930:ENSG00000119599:ENSG00000165861:ENSG00000119707:ENSG00000080815:ENSG00000100767:ENS

	G00000133961:ENSG00000165417:ENSG00000165934:ENSG00000153485:ENSG00000012963:ENSG00000140157:ENSG00000170113:ENSG00000128731:ENSG00000021776:ENSG00000198146:ENSG00000174197:ENSG00000137802:ENSG00000243708:ENSG00000243789:ENSG00000197535:ENSG00000128989:ENSG00000181827:ENSG00000140262:ENSG00000103710:ENSG00000140350:ENSG00000117899:ENSG00000140406:ENSG00000140511:ENSG00000140471:ENSG00000140986:ENSG00000008710:ENSG00000103512:ENSG00000183426:ENSG00000179889:ENSG00000157045:ENSG00000085721:ENSG00000157106:ENSG00000102879:ENSG00000169217:ENSG00000169221:ENSG00000180035:ENSG00000179965:ENSG00000179958:ENSG00000140691:ENSG00000140682:ENSG00000197302:ENSG00000102910:ENSG00000196470:ENSG00000102921:ENSG00000102978:ENSG00000072736:ENSG00000103061:ENSG00000132600:ENSG00000103018:ENSG00000185669:ENSG00000158717:ENSG00000167515:ENSG00000167522:ENSG00000197912:ENSG00000127804:ENSG00000196689:ENSG00000197417:ENSG00000029725:ENSG00000005100:ENSG00000072849:ENSG00000132514:ENSG00000141127:ENSG00000188522:ENSG00000072134:ENSG00000108641:ENSG00000166484:ENSG00000128482:ENSG00000142494:ENSG00000067191:ENSG00000068079:ENSG00000188554:ENSG00000184988:ENSG00000225190:ENSG00000141279:ENSG00000108424:ENSG00000198933:ENSG00000008294:ENSG00000011258:ENSG00000011260:ENSG00000153933:ENSG00000121060:ENSG00000108423:ENSG00000108443:ENSG00000189050:ENSG00000198265:ENSG00000197170:ENSG00000154217:ENSG00000123700:ENSG00000141576:ENSG00000185262:ENSG00000078687:ENSG00000141577:ENSG00000167302:ENSG00000185504:ENSG00000182446:ENSG00000017797:ENSG00000154845:ENSG00000134265:ENSG00000154889:ENSG00000141401:ENSG00000152223:ENSG00000101638:ENSG00000081913:ENSG00000130856:ENSG00000197971:ENSG00000071564:ENSG00000141985:ENSG00000167670:ENSG00000130254:ENSG00000160633:ENSG00000104880:ENSG00000104883:ENSG00000167775:ENSG00000065989:ENSG00000079999:ENSG00000104957:ENSG00000132024:ENSG00000132017:ENSG00000132005:ENSG00000123146:ENSG00000123136:ENSG00000123159:ENSG00000132002:ENSG00000099308:ENSG00000104825:ENSG00000104835:ENSG00000269190:ENSG00000160570:ENSG00000167625:ENSG00000105723:ENSG00000105722:ENSG00000079432:ENSG00000079462:ENSG00000079385:ENSG00000185453:ENSG00000105483:ENSG00000105447:ENSG00000142544:ENSG00000105383:ENSG00000196214:ENSG00000167766:ENSG00000213020:ENSG00000176293:ENSG00000115756:ENSG00000115761:ENSG00000196208:ENSG00000197872:ENSG00000143867:ENSG00000055917:ENSG00000084676:ENSG00000119866:ENSG00000162928:ENSG00000162929:ENSG00000082898:ENSG00000075292:ENSG00000144034:ENSG00000144048:ENSG00000114978:ENSG00000065911:ENSG00000005448:ENSG00000114993:ENSG00000115604:ENSG00000153107:ENSG00000153208:ENSG00000153214:ENSG00000152102:ENSG00000115762:ENSG00000163040:ENSG00000115963:ENSG00000144283:ENSG00000153250:ENSG00000118260:ENSG00000144401:ENSG00000197713:ENSG00000018280:ENSG00000135912:ENSG00000168958:ENSG00000144485:ENSG00000132326:ENSG00000162804:ENSG00000088876:ENSG00000125779:ENSG00000089063:ENSG00000132646:ENSG00000101290:ENSG00000089048:ENSG00000188559:ENSG00000242372:ENSG00000124107:ENSG00000124228:ENSG00000124201:ENSG00000203896:ENSG00000101152:ENSG00000155313:ENSG00000154642:ENSG00000157617:ENSG00000160190:ENSG00000160284:ENSG00000160285:ENSG00000184792:ENSG00000100220:ENSG00000128335:ENSG00000100342:ENSG00000100321:ENSG00000100324:ENSG00000198951:ENSG00000177096:ENSG00000242247:ENSG00000205643:ENSG00000075234:ENSG00000075275:ENSG00000196576:ENSG00000100258:ENSG00000025770:ENSG00000130489:ENSG00000025708:ENSG00000177989:ENSG00000205560:ENSG00000100299:ENSG00000134077:ENSG00000144560:ENSG00000144712:ENSG00000163517:ENSG00000170293:ENSG00000183873:ENSG00000177873:ENSG00000182983:ENSG00000179564:ENSG00000243477:ENSG00000114378:ENSG00000114383:ENSG00000068028:ENSG00000114388:ENSG00000114395:ENSG00000126062:ENSG00000180376:ENSG00000163946:ENSG00000144741:ENSG00000114861:ENSG00000144736:ENSG00000163320:ENSG00000175105:ENSG00000179021:ENSG00000169379:ENSG00000178700:ENSG00000114446:ENSG00000163608:ENSG00000082701:ENSG00000240682:ENSG00000017260:ENSG00000034533:ENSG00000240303:ENSG00000113971:ENSG00000129048:ENSG00000081307:ENSG00000144867:ENSG00000158163:ENSG00000138231:ENSG00000114098:ENSG00000184432:ENSG00000114115:ENSG00000155903:ENSG00000175066:ENSG00000114127:ENSG00000114698:ENSG00000169282:ENSG00000075420:ENSG00000161202:ENSG00000073803:ENSG00000163904:ENSG00000114279:ENSG00000145014:ENSG00000041802:ENSG00000163975:ENSG00000013288:ENSG00000109689:ENSG00000169019:ENSG00000145246:ENSG00000109171:ENSG00000080493:ENSG00000169429:ENSG00000163739:ENSG00000109321:ENSG00000151247:ENSG00000145331:ENSG00000248713:ENSG00000205403:ENSG00000109466:ENSG00000129187:ENSG00000113360:ENSG00000082213:ENSG00000164190:ENSG00000171522:ENSG00000132357:ENSG00000067248:ENSG00000039123:ENSG00000068796:ENSG00000086189:ENSG00000086200:ENSG00000164331:ENSG00000164338:ENSG00000164180:ENSG00000153140:ENSG00000181751:ENSG00000168916:ENSG00000064651:ENSG00000253159:ENSG00000240184:ENSG00000131504:ENSG00000171720:ENSG00000197948:ENSG00000131507:ENSG00000113272:ENSG00000048140:ENSG00000131446:ENSG00000196670:ENSG00000124535:ENSG00000137266:ENSG00000124787:ENSG00000078401:ENSG00000079691:ENSG00000146112:ENSG00000137404:ENSG00000137337:ENSG00000204344:ENSG00000244731:ENSG00000223501:ENSG00000235863:ENSG00000227057:ENSG00000204220:ENSG00000237441:ENSG00000124772:ENSG00000198663:ENSG00000156639:ENSG00000124743:ENSG00000112245:ENSG00000132429:ENSG00000118503:ENSG00000112406:ENSG00000120278:ENSG00000131018:ENSG00000272047:ENSG00000026297:ENSG00000213066:ENSG00000186340:ENSG00000184786:ENSG00000130023:ENSG00000122644:ENSG00000153814:ENSG00000122545:ENSG00000168303:ENSG00000152926:ENSG00000213462:ENSG00000146757:ENSG00000126522:ENSG00000169902:ENSG00000086730:ENSG00000049541:ENSG00000177679:ENSG00000135218:ENSG00000085563:ENSG00000105829:ENSG00000121716:ENSG00000085514:ENSG00000078487:ENSG00000146834:ENSG00000160813:ENSG00000166925:ENSG00000005249:ENSG00000172209:ENSG00000

						105865:ENSG00000196937:ENSG00000091704:ENSG00000106484:ENSG00000158623:ENSG00000131558:ENSG00000182158:ENSG00000146966:ENSG00000181444:ENSG00000117868:ENSG00000129691:ENSG00000023287:ENSG00000254087:ENSG00000035681:ENSG00000147601:ENSG00000121039:ENSG00000104267:ENSG00000164949:ENSG00000120526:ENSG00000120533:ENSG00000165071:ENSG00000131773:ENSG00000160932:ENSG00000180900:ENSG00000179950:ENSG00000185189:ENSG00000196378:ENSG00000080608:ENSG00000086065:ENSG00000135018:ENSG00000213694:ENSG00000127080:ENSG00000130958:ENSG00000136925:ENSG00000136936:ENSG00000196152:ENSG00000160408:ENSG00000136908:ENSG00000167106:ENSG00000171169:ENSG00000148339:ENSG00000148308:ENSG00000170835:ENSG00000148411:ENSG00000188986
DEG.twoside	Kidney_Cortex	6910	435	4.29122543376104e-16	2.31726173423096e-14	ENSG00000158286:ENSG00000158292:ENSG00000171819:ENSG00000120942:ENSG00000162460:ENSG00000065526:ENSG00000158816:ENSG00000187942:ENSG00000204178:ENSG00000160050:ENSG00000116525:ENSG00000127603:ENSG00000253313:ENSG00000117472:ENSG00000162366:ENSG00000198841:ENSG00000134717:ENSG00000162391:ENSG00000162604:ENSG00000162616:ENSG00000162642:ENSG00000153904:ENSG00000197147:ENSG00000171488:ENSG00000069712:ENSG00000117525:ENSG00000117569:ENSG00000117543:ENSG00000134215:ENSG00000162775:ENSG00000168679:ENSG00000163349:ENSG00000197323:ENSG00000134240:ENSG00000143457:ENSG00000163131:ENSG00000143387:ENSG00000143437:ENSG00000143379:ENSG00000143412:ENSG00000163154:ENSG00000163354:ENSG00000143537:ENSG00000160767:ENSG00000143627:ENSG00000116539:ENSG00000163374:ENSG00000116580:ENSG00000132718:ENSG00000116584:ENSG00000160803:ENSG00000163472:ENSG00000132694:ENSG00000162746:ENSG00000143322:ENSG00000057252:ENSG00000177888:ENSG00000174307:ENSG00000163050:ENSG00000162885:ENSG00000107929:ENSG00000198879:ENSG00000134463:ENSG00000165997:ENSG00000099246:ENSG00000108094:ENSG00000165476:ENSG00000107738:ENSG00000138134:ENSG00000152779:ENSG00000165338:ENSG00000119927:ENSG00000148702:ENSG00000198873:ENSG00000184545:ENSG00000205339:ENSG00000166478:ENSG00000166483:ENSG00000198730:ENSG00000187486:ENSG00000129151:ENSG00000165905:ENSG00000149150:ENSG00000162174:ENSG00000168061:ENSG00000168060:ENSG00000162300:ENSG00000173621:ENSG00000175564:ENSG00000062282:ENSG00000137492:ENSG00000137672:ENSG00000076053:ENSG00000166250:ENSG00000082805:ENSG00000010219:ENSG00000111674:ENSG00000134531:ENSG00000110925:ENSG00000135457:ENSG00000123338:ENSG00000123360:ENSG00000166130:ENSG00000120868:ENSG00000111647:ENSG00000235162:ENSG00000074590:ENSG00000110887:ENSG00000139410:ENSG00000174989:ENSG00000111725:ENSG00000150990:ENSG00000177084:ENSG00000176915:ENSG00000139505:ENSG00000120688:ENSG00000136167:ENSG00000102531:ENSG00000123179:ENSG00000102753:ENSG00000136104:ENSG00000178695:ENSG00000152749:ENSG00000065150:ENSG00000139826:ENSG00000102524:ENSG00000100889:ENSG00000139914:ENSG00000092140:ENSG00000092108:ENSG00000100473:ENSG00000129493:ENSG00000129480:ENSG00000165355:ENSG00000179476:ENSG00000179454:ENSG00000198718:ENSG00000185246:ENSG00000139926:ENSG00000087301:ENSG00000197930:ENSG00000119707:ENSG00000080815:ENSG00000100767:ENSG00000165417:ENSG00000165934:ENSG00000012963:ENSG00000170113:ENSG00000128731:ENSG00000021776:ENSG00000198146:ENSG00000174197:ENSG00000137802:ENSG00000243789:ENSG00000197535:ENSG00000181827:ENSG00000140262:ENSG00000186198:ENSG00000103710:ENSG00000136378:ENSG00000140600:ENSG00000140511:ENSG00000185418:ENSG00000008710:ENSG00000103512:ENSG00000183426:ENSG00000085721:ENSG00000157106:ENSG00000102879:ENSG00000169221:ENSG00000102910:ENSG00000196470:ENSG00000102921:ENSG00000072736:ENSG00000103066:ENSG00000103064:ENSG00000132600:ENSG00000103056:ENSG00000185669:ENSG00000167522:ENSG00000127804:ENSG00000029725:ENSG00000005100:ENSG00000132514:ENSG00000154025:ENSG00000072134:ENSG00000166484:ENSG00000128482:ENSG00000142494:ENSG00000180638:ENSG00000067191:ENSG00000184988:ENSG00000182963:ENSG00000225190:ENSG00000141279:ENSG00000108424:ENSG00000008294:ENSG00000011258:ENSG00000011260:ENSG00000214226:ENSG00000153933:ENSG00000121060:ENSG00000108423:ENSG00000108443:ENSG00000189050:ENSG00000198265:ENSG00000197170:ENSG00000154217:ENSG00000153822:ENSG00000141540:ENSG00000141576:ENSG00000078687:ENSG00000154845:ENSG00000134265:ENSG00000141401:ENSG00000168234:ENSG00000141384:ENSG00000152223:ENSG00000081913:ENSG00000130856:ENSG00000197971:ENSG00000167670:ENSG00000104833:ENSG00000065989:ENSG00000079999:ENSG00000129354:ENSG00000160951:ENSG00000127527:ENSG00000099308:ENSG00000250799:ENSG00000161270:ENSG00000269190:ENSG00000167625:ENSG00000079432:ENSG00000105429:ENSG00000105427:ENSG00000079435:ENSG00000079385:ENSG00000185453:ENSG00000105483:ENSG00000105447:ENSG00000105383:ENSG00000196214:ENSG00000197872:ENSG00000143867:ENSG00000055917:ENSG00000084676:ENSG00000162929:ENSG00000082898:ENSG00000116039:ENSG00000075292:ENSG00000144048:ENSG00000065911:ENSG00000114993:ENSG00000144218:ENSG00000115602:ENSG00000180251:ENSG00000153107:ENSG00000153214:ENSG00000115963:ENSG00000118260:ENSG00000127831:ENSG00000135912:ENSG00000198612:ENSG00000144485:ENSG00000132326:ENSG00000162804:ENSG00000196209:ENSG00000088876:ENSG00000125779:ENSG00000101290:ENSG00000089048:ENSG00000188559:ENSG00000125966:ENSG00000124102:ENSG00000124228:ENSG00000124201:ENSG00000203896:ENSG00000155313:ENSG00000154642:ENSG00000157617:ENSG00000160284:ENSG00000160285:ENSG00000183628:ENSG00000184792:ENSG00000100321:ENSG00000100324:ENSG00000177096:ENSG00000075234:ENSG00000075275:ENSG00000100253:ENSG00000025770:ENSG00000130487:ENSG00000008735:ENSG00000070950:ENSG00000134077:ENSG00000144560:ENSG00000144712:ENSG00000170011:ENSG00000182606:ENSG00000180432:ENSG00000182983:ENSG00000186792:ENSG00000243477:ENSG00000114378:ENSG00000068028:ENSG00000180376:ENSG00000163946:ENSG00000144749:ENSG00000114861:ENSG00000144736:ENSG00000175105:ENSG00000179021:ENSG00000169379:ENSG00000178700:ENSG00000144843:ENSG00000082701:ENSG00000240682:ENSG00000017260:ENSG00000034533:ENSG00000113971:

						ENSG00000154917:ENSG00000138231:ENSG00000114098:ENSG00000158186:ENSG00000155903:ENSG00000175066:ENSG00000114127:ENSG00000114698:ENSG00000163762:ENSG00000163904:ENSG00000205835:ENSG00000041802:ENSG00000152953:ENSG00000013288:ENSG00000157765:ENSG00000109171:ENSG00000080493:ENSG00000079557:ENSG00000163739:ENSG00000109321:ENSG00000151247:ENSG00000205403:ENSG00000164128:ENSG00000109466:ENSG00000174358:ENSG00000164363:ENSG00000113361:ENSG00000113360:ENSG00000082213:ENSG00000164190:ENSG00000132357:ENSG00000067248:ENSG00000039123:ENSG00000068796:ENSG00000086189:ENSG00000086200:ENSG00000164331:ENSG00000164338:ENSG00000181751:ENSG00000173926:ENSG00000064651:ENSG00000240184:ENSG00000197948:ENSG00000135074:ENSG00000113272:ENSG00000155858:ENSG00000131446:ENSG00000124535:ENSG00000137266:ENSG00000124787:ENSG00000146112:ENSG00000137404:ENSG00000137337:ENSG00000244731:ENSG00000235863:ENSG00000227057:ENSG00000124772:ENSG00000198663:ENSG00000164530:ENSG00000244067:ENSG00000243955:ENSG00000269964:ENSG00000118432:ENSG00000112406:ENSG00000026297:ENSG00000186340:ENSG00000122644:ENSG00000105855:ENSG00000153814:ENSG00000122545:ENSG00000152926:ENSG00000213462:ENSG00000146757:ENSG00000126522:ENSG00000165215:ENSG00000189143:ENSG00000086730:ENSG00000049541:ENSG00000135218:ENSG00000078487:ENSG00000166924:ENSG00000005249:ENSG00000075790:ENSG00000106477:ENSG00000158623:ENSG00000131558:ENSG00000146966:ENSG00000146918:ENSG00000117868:ENSG00000120910:ENSG00000129691:ENSG00000023287:ENSG00000254087:ENSG00000035681:ENSG00000104267:ENSG00000104327:ENSG00000120526:ENSG00000165071:ENSG00000196378:ENSG00000044459:ENSG00000127080:ENSG00000136925:ENSG00000136936:ENSG00000136826:ENSG00000070159:ENSG00000167081:ENSG00000196152:ENSG00000148308:ENSG00000160339:ENSG00000130560:ENSG00000148411:ENSG00000233198:ENSG00000198569:ENSG00000197768
DEG.twoside	Kidney_Medulla	95	4	0.616431068353487	1	ENSG00000117472:ENSG00000153904:ENSG00000111725:ENSG00000104267
DEG.twoside	Liver	9595	595	2.14990209201339e-22	1.16094712968723e-20	ENSG00000116237:ENSG00000158292:ENSG00000097021:ENSG00000171603:ENSG00000171824:ENSG00000198793:ENSG00000162460:ENSG00000162458:ENSG00000065526:ENSG00000116809:ENSG00000158747:ENSG00000183726:ENSG00000184007:ENSG00000160050:ENSG00000160051:ENSG00000116525:ENSG00000160094:ENSG00000127603:ENSG00000168389:ENSG00000066056:ENSG00000117399:ENSG00000066322:ENSG00000198198:ENSG00000162366:ENSG00000162367:ENSG00000117862:ENSG00000198841:ENSG00000134717:ENSG00000162384:ENSG00000162391:ENSG00000134709:ENSG00000162604:ENSG00000162616:ENSG00000117133:ENSG00000117155:ENSG00000162642:ENSG00000142867:ENSG00000153904:ENSG00000197147:ENSG00000117528:ENSG00000117525:ENSG00000117569:ENSG00000162694:ENSG00000117543:ENSG00000168679:ENSG00000116455:ENSG00000163349:ENSG00000197323:ENSG00000116752:ENSG00000213281:ENSG00000134240:ENSG00000143457:ENSG00000143387:ENSG00000143437:ENSG00000143379:ENSG00000143412:ENSG00000143409:ENSG00000143363:ENSG00000143434:ENSG00000163154:ENSG00000163156:ENSG00000163155:ENSG00000163354:ENSG00000143537:ENSG00000160767:ENSG00000116521:ENSG00000143630:ENSG00000143627:ENSG00000160753:ENSG00000116539:ENSG00000163374:ENSG00000116580:ENSG00000143622:ENSG00000132680:ENSG00000116584:ENSG00000160803:ENSG00000196189:ENSG00000242252:ENSG00000160781:ENSG00000163472:ENSG00000187800:ENSG00000132694:ENSG00000132703:ENSG00000143226:ENSG00000198734:ENSG00000174175:ENSG00000120341:ENSG00000186283:ENSG00000143322:ENSG00000057252:ENSG00000134389:ENSG00000143278:ENSG00000177888:ENSG00000174307:ENSG00000122176:ENSG00000182004:ENSG00000143801:ENSG00000162885:ENSG00000162714:ENSG00000015171:ENSG00000107929:ENSG00000134463:ENSG00000148468:ENSG00000241058:ENSG00000099246:ENSG00000108094:ENSG00000095794:ENSG00000188234:ENSG00000165476:ENSG00000107738:ENSG00000026103:ENSG00000119927:ENSG00000148702:ENSG00000165832:ENSG00000198873:ENSG00000184545:ENSG00000166313:ENSG00000110169:ENSG00000205339:ENSG00000268403:ENSG00000166478:ENSG00000166483:ENSG00000129151:ENSG00000110442:ENSG00000149150:ENSG00000162174:ENSG00000213465:ENSG00000110025:ENSG00000162300:ENSG00000149823:ENSG00000149809:ENSG00000173653:ENSG00000173599:ENSG00000173621:ENSG00000175567:ENSG00000166391:ENSG00000062282:ENSG00000137492:ENSG00000137693:ENSG00000149313:ENSG00000137714:ENSG00000149292:ENSG00000076043:ENSG00000082805:ENSG00000010219:ENSG00000111667:ENSG00000111671:ENSG00000010626:ENSG00000111674:ENSG00000126749:ENSG00000134531:ENSG00000123094:ENSG00000110911:ENSG00000050426:ENSG00000110925:ENSG00000135457:ENSG00000123338:ENSG00000127314:ENSG00000166130:ENSG00000120868:ENSG00000111647:ENSG00000235162:ENSG00000074590:ENSG00000110887:ENSG00000135094:ENSG00000139410:ENSG00000174989:ENSG00000111725:ENSG00000188735:ENSG00000150990:ENSG00000176915:ENSG00000139505:ENSG00000122033:ENSG00000133110:ENSG00000183722:ENSG00000102743:ENSG00000120688:ENSG00000123179:ENSG00000102753:ENSG00000136104:ENSG00000188243:ENSG00000136153:ENSG00000178695:ENSG00000088451:ENSG00000065150:ENSG00000102524:ENSG00000100889:ENSG00000139914:ENSG00000092140:ENSG00000092108:ENSG00000129493:ENSG00000203546:ENSG00000129480:ENSG00000150527:ENSG00000165355:ENSG00000179476:ENSG00000179454:ENSG00000198718:ENSG00000185246:ENSG00000139926:ENSG00000087301:ENSG00000006432:ENSG00000119599:ENSG00000165861:ENSG00000119707:ENSG00000080815:ENSG00000100767:ENSG00000133961:ENSG00000165417:ENSG00000165934:ENSG00000165943:ENSG00000153485:ENSG00000012963:ENSG00000140157:ENSG00000170113:ENSG00000128731:ENSG00000021776:ENSG00000198146:ENSG00000174197:ENSG00000137802:ENSG00000243708:ENSG00000243789:ENSG00000197535:ENSG00000128989:ENSG00000181827:ENSG00000140262:ENSG00000103707:ENSG00000186198:ENSG00000140350:ENSG00000140406:ENSG00

	000140471:ENSG00000185418:ENSG00000008710:ENSG00000103512:ENSG00000183426:ENSG00000157045:ENSG00000157106:ENSG00000102879:ENSG00000169217:ENSG00000169221:ENSG00000180035:ENSG00000180096:ENSG00000179958:ENSG00000140682:ENSG00000197302:ENSG00000121270:ENSG00000196470:ENSG00000102921:ENSG00000005194:ENSG00000102978:ENSG00000072736:ENSG00000103067:ENSG00000103066:ENSG00000103064:ENSG00000103061:ENSG00000132600:ENSG00000103056:ENSG00000103018:ENSG00000158717:ENSG00000167515:ENSG00000167522:ENSG00000127804:ENSG00000196689:ENSG00000197417:ENSG00000029725:ENSG00000005100:ENSG00000161944:ENSG00000141127:ENSG00000072134:ENSG00000108641:ENSG00000166484:ENSG00000166482:ENSG00000142494:ENSG00000108828:ENSG00000108830:ENSG00000184988:ENSG00000225190:ENSG00000141279:ENSG00000108424:ENSG00000198933:ENSG00000008294:ENSG00000011258:ENSG00000011260:ENSG00000153933:ENSG00000108423:ENSG00000108443:ENSG00000198265:ENSG00000154217:ENSG00000141576:ENSG00000185262:ENSG00000078687:ENSG00000141577:ENSG00000167302:ENSG00000185504:ENSG00000182446:ENSG00000017797:ENSG00000154845:ENSG00000134265:ENSG00000154889:ENSG00000168234:ENSG00000152223:ENSG00000130856:ENSG00000197971:ENSG00000071564:ENSG00000141985:ENSG00000167670:ENSG00000130254:ENSG00000160633:ENSG00000104880:ENSG00000104883:ENSG00000167775:ENSG00000065989:ENSG00000079999:ENSG00000104957:ENSG00000132024:ENSG00000132017:ENSG00000132005:ENSG00000123146:ENSG00000123136:ENSG00000123143:ENSG00000123159:ENSG00000127527:ENSG00000099308:ENSG00000250799:ENSG00000269190:ENSG00000160570:ENSG00000167625:ENSG00000105723:ENSG00000079432:ENSG00000079462:ENSG00000105429:ENSG00000079385:ENSG00000142273:ENSG00000185453:ENSG00000105483:ENSG00000142544:ENSG00000105383:ENSG00000196214:ENSG00000198464:ENSG00000167766:ENSG00000115761:ENSG00000196208:ENSG00000197872:ENSG00000055917:ENSG00000084676:ENSG00000213639:ENSG00000171103:ENSG00000082898:ENSG00000075292:ENSG00000144034:ENSG00000144048:ENSG00000163170:ENSG00000114978:ENSG00000065911:ENSG00000204843:ENSG00000005448:ENSG00000114993:ENSG00000183281:ENSG00000153107:ENSG00000153208:ENSG00000153214:ENSG00000152102:ENSG00000115762:ENSG00000173272:ENSG00000144283:ENSG00000153250:ENSG00000118260:ENSG00000197713:ENSG00000115461:ENSG00000018280:ENSG00000127831:ENSG00000135912:ENSG00000168958:ENSG00000198612:ENSG00000144485:ENSG00000132326:ENSG00000162804:ENSG00000088876:ENSG00000125779:ENSG00000132646:ENSG00000101290:ENSG00000089048:ENSG00000101247:ENSG00000124102:ENSG00000124107:ENSG00000124228:ENSG00000124201:ENSG00000203896:ENSG00000101152:ENSG00000198276:ENSG00000155313:ENSG00000154642:ENSG00000157617:ENSG00000160190:ENSG00000183628:ENSG00000100342:ENSG00000100321:ENSG00000100324:ENSG00000177096:ENSG00000100197:ENSG00000077942:ENSG00000186951:ENSG00000205643:ENSG00000075234:ENSG00000100416:ENSG00000075275:ENSG00000205593:ENSG00000025770:ENSG00000251322:ENSG00000070950:ENSG00000134077:ENSG00000144560:ENSG00000144712:ENSG00000163517:ENSG00000163513:ENSG00000170293:ENSG00000177873:ENSG00000182606:ENSG00000144648:ENSG00000180432:ENSG00000182983:ENSG00000243477:ENSG00000114378:ENSG00000068001:ENSG00000114383:ENSG00000068028:ENSG00000114388:ENSG00000126062:ENSG00000180376:ENSG00000163946:ENSG00000144741:ENSG00000144749:ENSG00000114861:ENSG00000144736:ENSG00000163320:ENSG00000175105:ENSG00000179021:ENSG00000184500:ENSG00000169379:ENSG00000178700:ENSG00000114446:ENSG00000163607:ENSG00000163608:ENSG00000144843:ENSG00000144852:ENSG00000082701:ENSG00000172780:ENSG00000240682:ENSG00000017260:ENSG00000034533:ENSG00000240303:ENSG00000113971:ENSG00000138231:ENSG00000114098:ENSG00000158186:ENSG00000155903:ENSG00000175066:ENSG00000114127:ENSG00000163762:ENSG00000161202:ENSG00000163904:ENSG00000205835:ENSG00000145014:ENSG00000163975:ENSG00000013288:ENSG00000109689:ENSG00000169019:ENSG00000145246:ENSG00000109171:ENSG00000079557:ENSG00000109321:ENSG00000151247:ENSG00000138823:ENSG00000205403:ENSG00000164128:ENSG00000113360:ENSG00000082213:ENSG00000164190:ENSG00000132357:ENSG00000067248:ENSG00000039123:ENSG00000068796:ENSG00000086189:ENSG00000086200:ENSG00000164331:ENSG00000164338:ENSG00000164180:ENSG00000153140:ENSG00000168916:ENSG00000064651:ENSG00000145826:ENSG00000240184:ENSG00000164620:ENSG00000197948:ENSG00000120318:ENSG00000113721:ENSG00000135074:ENSG00000113272:ENSG00000155858:ENSG00000113312:ENSG00000113742:ENSG00000048140:ENSG00000196670:ENSG00000124535:ENSG00000078401:ENSG00000079691:ENSG00000146112:ENSG00000137404:ENSG00000137337:ENSG00000244731:ENSG00000223501:ENSG00000227057:ENSG00000237441:ENSG00000198663:ENSG00000156639:ENSG00000244067:ENSG00000243955:ENSG00000198945:ENSG00000135525:ENSG00000112357:ENSG00000118503:ENSG00000112406:ENSG00000146416:ENSG00000120278:ENSG00000131018:ENSG00000272047:ENSG00000026297:ENSG00000186340:ENSG00000184786:ENSG00000122644:ENSG00000153814:ENSG00000122545:ENSG00000152926:ENSG00000213462:ENSG00000146757:ENSG00000126522:ENSG00000165215:ENSG00000189143:ENSG00000086730:ENSG00000135218:ENSG00000085563:ENSG00000127920:ENSG00000085514:ENSG00000078487:ENSG00000146834:ENSG00000160813:ENSG00000166925:ENSG00000005249:ENSG00000105865:ENSG00000075790:ENSG00000196937:ENSG00000158623:ENSG00000131558:ENSG00000182158:ENSG00000146966:ENSG00000181444:ENSG00000196329:ENSG00000214106:ENSG00000146918:ENSG00000117868:ENSG00000120910:ENSG00000129691:ENSG00000023287:ENSG00000254087:ENSG00000035681:ENSG00000147601:ENSG00000104267:ENSG00000164949:ENSG00000120526:ENSG00000120533:ENSG00000131773:ENSG00000180900:ENSG00000196378:ENSG00000080608:ENSG00000086065:ENSG00000213694:ENSG00000127080:ENSG00000136925:ENSG00000136936:ENSG00000136826:ENSG00000070159:ENSG00000119446:ENSG00000167081:ENSG00000196152:ENSG00000148356:ENSG00000136840:ENSG00000167106:ENSG00000148339:ENSG00000148308:ENSG00000170835:ENSG00000160339:ENSG00000130560:ENSG00000148411:ENSG00000188986:ENSG00000198113

DEG.twoside	Lung	5173	283	9.19814971381202e-05	0.00496700084545849	ENSG00000158286:ENSG00000158292:ENSG00000069812:ENSG00000171819:ENSG00000162458:ENSG00000116809:ENSG00000134668:ENSG00000127603:ENSG00000168389:ENSG00000243710:ENSG00000179178:ENSG00000253313:ENSG00000066056:ENSG00000117472:ENSG00000162366:ENSG00000162367:ENSG00000162390:ENSG00000117114:ENSG00000137976:ENSG00000117155:ENSG00000171488:ENSG00000069712:ENSG00000162775:ENSG00000168679:ENSG00000175984:ENSG00000213281:ENSG00000092621:ENSG00000134240:ENSG00000163131:ENSG00000143412:ENSG00000143434:ENSG00000163154:ENSG00000143630:ENSG00000143622:ENSG00000116584:ENSG00000196189:ENSG00000160781:ENSG00000163472:ENSG00000187800:ENSG00000160838:ENSG00000143226:ENSG00000173110:ENSG00000203747:ENSG00000152332:ENSG00000174175:ENSG00000057252:ENSG00000122176:ENSG00000163050:ENSG00000116962:ENSG00000107929:ENSG00000198879:ENSG00000165997:ENSG00000075073:ENSG00000107736:ENSG00000107738:ENSG00000026103:ENSG00000152779:ENSG00000120057:ENSG00000203867:ENSG00000119927:ENSG00000198873:ENSG00000187908:ENSG00000006071:ENSG00000165905:ENSG00000162174:ENSG00000173599:ENSG00000187726:ENSG00000175567:ENSG00000137672:ENSG00000183644:ENSG00000154127:ENSG00000023171:ENSG00000064309:ENSG00000111671:ENSG00000010626:ENSG00000111679:ENSG00000111684:ENSG00000013588:ENSG00000134531:ENSG00000110911:ENSG00000123338:ENSG00000127314:ENSG00000166130:ENSG00000120868:ENSG00000135094:ENSG00000111725:ENSG00000187848:ENSG00000127863:ENSG00000136167:ENSG00000102445:ENSG00000102531:ENSG00000136153:ENSG00000178695:ENSG00000139826:ENSG00000102524:ENSG00000139914:ENSG00000168952:ENSG00000133961:ENSG00000186198:ENSG00000103710:ENSG00000136378:ENSG00000103888:ENSG00000140406:ENSG00000156206:ENSG00000140600:ENSG00000140511:ENSG00000007038:ENSG00000102879:ENSG00000180209:ENSG00000180096:ENSG00000179965:ENSG00000140682:ENSG00000102921:ENSG00000102970:ENSG00000103067:ENSG00000103066:ENSG00000172137:ENSG00000185669:ENSG00000158717:ENSG00000132514:ENSG00000007174:ENSG00000188522:ENSG00000141750:ENSG00000161405:ENSG00000068079:ENSG00000108830:ENSG00000184988:ENSG00000182963:ENSG00000073861:ENSG00000214226:ENSG00000153933:ENSG00000121060:ENSG00000198265:ENSG00000171595:ENSG00000141576:ENSG00000154889:ENSG00000152223:ENSG00000081913:ENSG00000205744:ENSG00000104880:ENSG00000065989:ENSG00000129354:ENSG00000123146:ENSG00000160951:ENSG00000096996:ENSG00000262484:ENSG00000105427:ENSG00000079435:ENSG00000189377:ENSG00000142273:ENSG00000105483:ENSG00000105383:ENSG00000105492:ENSG00000171049:ENSG00000115756:ENSG00000196208:ENSG00000197872:ENSG00000143867:ENSG00000162929:ENSG00000116039:ENSG00000065911:ENSG00000144218:ENSG00000115602:ENSG00000115604:ENSG00000115607:ENSG00000153214:ENSG00000115762:ENSG00000153250:ENSG00000018280:ENSG00000123977:ENSG00000196209:ENSG00000188559:ENSG00000125966:ENSG00000124102:ENSG00000124107:ENSG00000124201:ENSG00000124256:ENSG00000184792:ENSG00000128335:ENSG00000100342:ENSG00000100321:ENSG00000198951:ENSG00000177096:ENSG00000075275:ENSG00000130489:ENSG00000177989:ENSG00000008735:ENSG00000251322:ENSG00000144712:ENSG00000163517:ENSG00000163576:ENSG00000170293:ENSG00000170011:ENSG00000168032:ENSG00000114378:ENSG00000068001:ENSG00000004838:ENSG00000114395:ENSG00000007402:ENSG00000114861:ENSG00000184500:ENSG00000169379:ENSG00000178750:ENSG00000114446:ENSG00000144843:ENSG00000169704:ENSG00000172780:ENSG00000154917:ENSG00000158163:ENSG00000114113:ENSG00000155903:ENSG00000114698:ENSG00000169282:ENSG00000174899:ENSG00000075420:ENSG00000172578:ENSG00000163898:ENSG00000145014:ENSG00000163975:ENSG00000157765:ENSG00000109689:ENSG00000169429:ENSG00000163739:ENSG00000109321:ENSG00000145287:ENSG00000185477:ENSG00000205403:ENSG00000164128:ENSG00000113361:ENSG00000171522:ENSG00000132357:ENSG00000197822:ENSG00000181751:ENSG00000168916:ENSG00000253159:ENSG00000240184:ENSG00000120318:ENSG00000135074:ENSG00000078401:ENSG00000079691:ENSG00000112812:ENSG00000146112:ENSG00000137404:ENSG00000255587:ENSG00000124772:ENSG00000244067:ENSG00000243955:ENSG00000118432:ENSG00000135525:ENSG00000118503:ENSG00000112406:ENSG00000120278:ENSG00000131018:ENSG00000026297:ENSG00000241644:ENSG00000152926:ENSG00000126522:ENSG00000165215:ENSG00000189143:ENSG00000086730:ENSG00000177679:ENSG00000135218:ENSG00000127920:ENSG00000085514:ENSG00000106484:ENSG00000146966:ENSG00000181444:ENSG00000196329:ENSG00000154319:ENSG00000168546:ENSG00000254087:ENSG00000121039:ENSG00000104267:ENSG00000164949:ENSG00000165071:ENSG00000080608:ENSG00000178031:ENSG00000130958:ENSG00000243444:ENSG00000136840:ENSG00000148339:ENSG00000170835:ENSG00000198113
DEG.twoside	Minor_Salivary_Gland	3769	200	0.00466670607947308	0.252002128291546	ENSG00000158286:ENSG00000158292:ENSG00000097021:ENSG00000183888:ENSG00000187942:ENSG00000188672:ENSG00000157978:ENSG00000160050:ENSG00000168389:ENSG00000179178:ENSG00000253313:ENSG00000066056:ENSG00000117472:ENSG00000162366:ENSG00000162367:ENSG00000134709:ENSG00000162616:ENSG00000137976:ENSG00000171488:ENSG00000069712:ENSG00000162694:ENSG00000168679:ENSG00000116748:ENSG00000092621:ENSG00000134240:ENSG00000163157:ENSG00000132718:ENSG00000196189:ENSG00000160781:ENSG00000163472:ENSG00000187800:ENSG00000173110:ENSG00000198734:ENSG00000095794:ENSG00000107736:ENSG00000138134:ENSG00000059573:ENSG00000198873:ENSG00000187908:ENSG00000166313:ENSG00000187486:ENSG00000135373:ENSG00000019505:ENSG00000165905:ENSG00000124939:ENSG00000124935:ENSG00000168060:ENSG00000149809:ENSG00000175567:ENSG00000110777:ENSG00000154127:ENSG00000023171:ENSG00000064309:ENSG00000010626:ENSG00000111674:ENSG00000013588:ENSG00000134532:ENSG00000123094:ENSG00000087494:ENSG00000110911:ENSG00000123360:ENSG00000205057:ENSG00000235162:ENSG00000074590:ENSG00000122966:ENSG00000127863:ENSG00000134901:ENSG00000150527:ENSG00000197930:ENSG00000170113:ENSG00000243789:ENSG00000197535:ENSG00000103710:ENSG00000136378:ENSG00000140600:ENSG00000185418:ENSG00000008710:ENSG00000007038:ENSG00000183426:ENSG00000180209:ENSG00000179965:ENSG00000102970:ENSG

						00000103067:ENSG00000103064:ENSG00000128482:ENSG00000142494:ENSG00000141750:ENSG00000161405:ENSG00000108830:ENSG00000182963:ENSG00000008294:ENSG00000141540:ENSG00000141576:ENSG00000206073:ENSG00000197971:ENSG00000181143:ENSG00000129354:ENSG00000099308:ENSG00000269190:ENSG00000105369:ENSG00000105427:ENSG00000189377:ENSG00000079385:ENSG00000142273:ENSG00000063127:ENSG00000115756:ENSG00000134323:ENSG00000197872:ENSG00000143867:ENSG00000116039:ENSG00000005448:ENSG00000144218:ENSG00000115602:ENSG00000115607:ENSG00000153208:ENSG00000153214:ENSG00000163040:ENSG00000144283:ENSG00000144485:ENSG00000132326:ENSG00000101290:ENSG00000125966:ENSG00000198959:ENSG00000124102:ENSG00000124107:ENSG00000160190:ENSG00000160284:ENSG00000075275:ENSG00000177989:ENSG00000144712:ENSG00000197385:ENSG00000170293:ENSG00000168032:ENSG00000182606:ENSG00000114395:ENSG00000007402:ENSG00000178750:ENSG00000154917:ENSG00000158163:ENSG00000158186:ENSG00000175066:ENSG00000169282:ENSG00000114248:ENSG00000163898:ENSG00000114279:ENSG00000163975:ENSG00000157765:ENSG00000080493:ENSG00000169429:ENSG00000124875:ENSG00000163739:ENSG00000164128:ENSG00000215218:ENSG00000175471:ENSG00000181751:ENSG00000064651:ENSG00000253159:ENSG00000120318:ENSG00000135074:ENSG00000155858:ENSG00000137266:ENSG00000112812:ENSG00000137404:ENSG00000204544:ENSG00000124772:ENSG00000243955:ENSG00000118432:ENSG00000132429:ENSG00000135525:ENSG00000131018:ENSG00000272047:ENSG00000026297:ENSG00000164488:ENSG00000105855:ENSG00000153814:ENSG00000241644:ENSG00000169902:ENSG00000165215:ENSG00000189143:ENSG00000085563:ENSG00000127920:ENSG00000085514:ENSG00000078487:ENSG00000166924:ENSG00000075790:ENSG00000106477:ENSG00000146966:ENSG00000159763:ENSG00000154319:ENSG00000147465:ENSG00000254087:ENSG00000121039:ENSG00000104267:ENSG00000129295:ENSG00000196378:ENSG00000178031:ENSG00000213694:ENSG00000167081:ENSG00000148411:ENSG00000198113
DEG.twoside	Muscle_Skeletal	8051	497	3.14494359037935e-17	1.69826953880485e-15	ENSG00000158286:ENSG00000158292:ENSG00000097021:ENSG00000171603:ENSG00000162458:ENSG00000116809:ENSG00000158747:ENSG00000117616:ENSG00000183726:ENSG00000157978:ENSG00000084652:ENSG00000160050:ENSG00000160051:ENSG00000116525:ENSG00000160094:ENSG00000127603:ENSG00000066056:ENSG00000066322:ENSG00000159479:ENSG00000198198:ENSG00000117472:ENSG00000162367:ENSG00000117862:ENSG00000198841:ENSG00000162390:ENSG00000162604:ENSG00000117114:ENSG00000117155:ENSG00000162642:ENSG00000142867:ENSG00000153904:ENSG00000197147:ENSG00000171488:ENSG00000117528:ENSG00000117525:ENSG00000117569:ENSG00000162694:ENSG00000116396:ENSG00000168679:ENSG00000085465:ENSG00000197323:ENSG00000175984:ENSG00000116748:ENSG00000213281:ENSG00000052723:ENSG00000143457:ENSG00000163131:ENSG00000143387:ENSG00000143379:ENSG00000143418:ENSG00000143412:ENSG00000143409:ENSG00000143434:ENSG00000163157:ENSG00000143537:ENSG00000176444:ENSG00000160752:ENSG00000160753:ENSG00000125459:ENSG00000116580:ENSG00000132718:ENSG00000143622:ENSG00000132680:ENSG00000116584:ENSG00000196189:ENSG00000242252:ENSG00000160781:ENSG00000163472:ENSG00000198715:ENSG00000187800:ENSG00000132694:ENSG00000143226:ENSG00000173110:ENSG00000203747:ENSG00000174175:ENSG00000143322:ENSG00000057252:ENSG00000177888:ENSG00000159176:ENSG00000122176:ENSG00000182004:ENSG00000163050:ENSG00000116962:ENSG00000198879:ENSG00000134463:ENSG00000148468:ENSG00000241058:ENSG00000165997:ENSG00000099246:ENSG00000095794:ENSG00000188234:ENSG00000165476:ENSG00000075073:ENSG00000107738:ENSG00000026103:ENSG00000152779:ENSG00000165338:ENSG00000059573:ENSG00000155256:ENSG00000120057:ENSG00000203867:ENSG00000119927:ENSG00000165832:ENSG00000198873:ENSG00000166483:ENSG00000187486:ENSG00000006071:ENSG00000121680:ENSG00000156587:ENSG00000124935:ENSG00000162174:ENSG00000162300:ENSG00000149809:ENSG00000173653:ENSG00000173599:ENSG00000173621:ENSG00000175567:ENSG00000175564:ENSG00000062282:ENSG00000149292:ENSG00000076053:ENSG00000154127:ENSG00000166250:ENSG00000082805:ENSG00000010219:ENSG00000139180:ENSG00000111671:ENSG00000111674:ENSG00000111679:ENSG00000134531:ENSG00000110911:ENSG00000050426:ENSG00000123360:ENSG00000127314:ENSG00000139292:ENSG00000166130:ENSG00000120868:ENSG00000139410:ENSG00000174989:ENSG00000111725:ENSG00000177084:ENSG00000176915:ENSG00000127863:ENSG00000139505:ENSG00000133110:ENSG00000183722:ENSG00000136167:ENSG00000102531:ENSG00000123179:ENSG00000102753:ENSG00000136104:ENSG00000188243:ENSG00000136153:ENSG00000178695:ENSG00000088451:ENSG00000152749:ENSG00000139826:ENSG00000100889:ENSG00000139914:ENSG00000092140:ENSG00000092108:ENSG00000129493:ENSG00000129480:ENSG00000165355:ENSG00000179476:ENSG00000185246:ENSG00000139926:ENSG00000087301:ENSG00000197930:ENSG00000006432:ENSG00000119707:ENSG00000080815:ENSG00000100767:ENSG00000133961:ENSG00000258987:ENSG00000140157:ENSG00000170113:ENSG00000021776:ENSG00000174197:ENSG00000137802:ENSG00000243708:ENSG00000243789:ENSG00000197535:ENSG00000140262:ENSG00000103710:ENSG00000234438:ENSG00000140350:ENSG00000136378:ENSG00000117899:ENSG00000140406:ENSG00000140511:ENSG00000140471:ENSG00000185418:ENSG00000140986:ENSG00000183426:ENSG00000157106:ENSG00000102879:ENSG00000180209:ENSG00000180035:ENSG00000140682:ENSG00000197302:ENSG00000196470:ENSG00000102921:ENSG00000072736:ENSG00000103066:ENSG00000103064:ENSG00000103056:ENSG00000185669:ENSG00000158717:ENSG00000167515:ENSG00000167522:ENSG00000127804:ENSG00000196689:ENSG00000197417:ENSG00000072849:ENSG00000132514:ENSG00000141127:ENSG00000188522:ENSG00000072134:ENSG00000108641:ENSG00000166484:ENSG00000166482:ENSG00000128482:ENSG00000142494:ENSG00000067191:ENSG00000068079:ENSG00000108828:ENSG00000182963:ENSG00000011258:ENSG00000121060:ENSG00000108423:ENSG00000108878:ENSG00000197170:ENSG00000154217:ENSG00000141540:ENSG00000141576:ENSG00000078687:ENSG00000141577:ENSG00000167302:ENSG00000185504:ENSG00000182446:ENSG00000017797:ENSG00000154845:ENSG00000134265:ENSG00000141401:ENSG00000152223:ENSG0000008191

						3:ENSG00000130856:ENSG00000071564:ENSG00000141985:ENSG00000104833:ENSG00000104880:ENSG00000104883:ENSG00000167775:ENSG00000104957:ENSG00000132017:ENSG00000132005:ENSG00000123146:ENSG00000123136:ENSG00000132002:ENSG00000127527:ENSG00000099308:ENSG00000269190:ENSG00000160570:ENSG00000105722:ENSG00000079432:ENSG00000079462:ENSG00000105429:ENSG00000105427:ENSG00000079435:ENSG00000187244:ENSG00000185453:ENSG00000105483:ENSG00000142544:ENSG00000196214:ENSG00000167766:ENSG00000115756:ENSG00000196208:ENSG00000197872:ENSG00000143867:ENSG00000055917:ENSG00000163803:ENSG00000162929:ENSG00000082898:ENSG00000144048:ENSG00000114978:ENSG00000005448:ENSG00000114993:ENSG00000115602:ENSG00000153208:ENSG00000153214:ENSG00000115762:ENSG00000173272:ENSG00000115963:ENSG00000144283:ENSG00000153250:ENSG00000118260:ENSG00000197713:ENSG00000115457:ENSG00000018280:ENSG00000135912:ENSG00000132326:ENSG00000088876:ENSG00000125779:ENSG00000132646:ENSG00000101290:ENSG00000124228:ENSG00000124201:ENSG00000203896:ENSG00000101152:ENSG00000155313:ENSG00000154642:ENSG00000157617:ENSG00000160190:ENSG00000160284:ENSG00000160285:ENSG00000183628:ENSG00000184792:ENSG00000128335:ENSG00000100342:ENSG00000100321:ENSG00000100324:ENSG00000198951:ENSG00000177096:ENSG00000242247:ENSG00000077942:ENSG00000186951:ENSG00000205643:ENSG00000075234:ENSG00000100416:ENSG00000196576:ENSG00000100258:ENSG00000025770:ENSG00000130489:ENSG00000025708:ENSG00000177989:ENSG00000205560:ENSG00000100288:ENSG00000100299:ENSG00000251322:ENSG00000144560:ENSG00000144712:ENSG00000163513:ENSG00000170293:ENSG00000170011:ENSG00000182983:ENSG00000114353:ENSG00000179564:ENSG00000243477:ENSG00000114378:ENSG00000068001:ENSG00000068028:ENSG00000114395:ENSG00000126062:ENSG00000007402:ENSG00000180376:ENSG00000163946:ENSG00000114861:ENSG00000163320:ENSG00000175105:ENSG00000179021:ENSG00000184500:ENSG00000114446:ENSG00000017260:ENSG00000034533:ENSG00000240303:ENSG00000113971:ENSG00000081307:ENSG00000144867:ENSG00000154917:ENSG00000138231:ENSG00000184432:ENSG00000114115:ENSG00000155903:ENSG00000175066:ENSG00000114698:ENSG00000163762:ENSG00000169282:ENSG00000145014:ENSG00000013288:ENSG00000109689:ENSG00000169019:ENSG00000145246:ENSG00000163739:ENSG00000151247:ENSG00000248713:ENSG00000205403:ENSG00000109466:ENSG00000129187:ENSG00000215218:ENSG00000113360:ENSG00000164190:ENSG00000171522:ENSG00000132357:ENSG00000068796:ENSG00000086189:ENSG00000164331:ENSG00000164180:ENSG00000153140:ENSG00000168916:ENSG00000173926:ENSG00000064651:ENSG00000253159:ENSG00000240184:ENSG00000240764:ENSG00000197948:ENSG00000120318:ENSG00000131507:ENSG00000135074:ENSG00000113272:ENSG00000155858:ENSG00000113742:ENSG00000131446:ENSG00000196670:ENSG00000137266:ENSG00000078401:ENSG00000146112:ENSG00000137404:ENSG00000137337:ENSG00000137331:ENSG00000204344:ENSG00000235863:ENSG00000231925:ENSG00000137168:ENSG00000164530:ENSG00000124743:ENSG00000118432:ENSG00000132429:ENSG00000198945:ENSG00000112357:ENSG00000118503:ENSG00000146416:ENSG00000120278:ENSG00000131018:ENSG00000026297:ENSG00000213066:ENSG00000186340:ENSG00000184786:ENSG00000130023:ENSG00000122644:ENSG00000253293:ENSG00000153814:ENSG00000241644:ENSG00000122545:ENSG00000168303:ENSG00000152926:ENSG00000213462:ENSG00000146757:ENSG00000126522:ENSG00000169902:ENSG00000086730:ENSG00000135218:ENSG00000127920:ENSG00000105829:ENSG00000085514:ENSG00000078487:ENSG00000160813:ENSG00000166925:ENSG00000005249:ENSG00000105865:ENSG00000075790:ENSG00000196937:ENSG00000091704:ENSG00000106484:ENSG00000122786:ENSG00000182158:ENSG00000146966:ENSG00000159763:ENSG00000196329:ENSG00000146918:ENSG00000120910:ENSG00000035681:ENSG00000147601:ENSG00000121039:ENSG00000164949:ENSG00000120526:ENSG00000160932:ENSG00000185189:ENSG00000196378:ENSG00000080608:ENSG00000086065:ENSG00000213694:ENSG00000127080:ENSG00000130958:ENSG00000136936:ENSG00000136826:ENSG00000070159:ENSG00000167081:ENSG00000148356:ENSG00000136840:ENSG00000136908:ENSG00000167106:ENSG00000171169:ENSG00000148339:ENSG00000148335:ENSG00000170835:ENSG00000148411
DEG.twoside	Nerve_Tibial	6820	336	0.0189645848582002	1	ENSG00000158292:ENSG00000171819:ENSG00000120942:ENSG00000065526:ENSG00000187942:ENSG00000160094:ENSG00000168389:ENSG00000179178:ENSG00000066056:ENSG00000198198:ENSG00000117472:ENSG00000186790:ENSG00000186564:ENSG00000162604:ENSG00000117155:ENSG00000162642:ENSG00000197147:ENSG00000171488:ENSG00000069712:ENSG00000117528:ENSG00000117569:ENSG00000134215:ENSG00000168679:ENSG00000197323:ENSG00000143387:ENSG00000143437:ENSG00000143379:ENSG00000143412:ENSG00000143409:ENSG00000143363:ENSG00000143434:ENSG00000163155:ENSG00000163157:ENSG00000163354:ENSG00000160753:ENSG00000116539:ENSG00000125459:ENSG00000116580:ENSG00000132718:ENSG00000132680:ENSG00000116584:ENSG00000196189:ENSG00000160781:ENSG00000187800:ENSG00000143226:ENSG00000162746:ENSG00000174175:ENSG00000120341:ENSG00000186283:ENSG00000143322:ENSG00000177888:ENSG00000122176:ENSG00000116962:ENSG00000162714:ENSG00000198879:ENSG00000148468:ENSG00000241058:ENSG00000188234:ENSG00000075073:ENSG00000107738:ENSG00000152779:ENSG00000165338:ENSG00000095587:ENSG00000155256:ENSG00000120057:ENSG00000203867:ENSG00000198873:ENSG00000166313:ENSG00000166483:ENSG00000149809:ENSG00000173599:ENSG00000173621:ENSG00000137492:ENSG00000179240:ENSG00000154127:ENSG00000166250:ENSG00000023171:ENSG00000082805:ENSG00000010626:ENSG00000013588:ENSG00000134531:ENSG00000134532:ENSG00000123094:ENSG00000087494:ENSG00000135457:ENSG00000139292:ENSG00000074590:ENSG00000139410:ENSG00000122966:ENSG00000188735:ENSG00000150990:ENSG00000184992:ENSG00000127863:ENSG00000120688:ENSG00000102531:ENSG00000123179:ENSG00000184226:ENSG00000136153:ENSG00000178734:ENSG00000178695:ENSG00000088451:ENSG00000134899:ENSG00000139914:ENSG00000168952:ENSG00000092140:ENSG00000092108:ENSG00000129493:ENSG00000203546:ENSG00000165379:ENSG00000179476:ENSG00000179454:ENSG00000198718:ENSG00000185246:ENSG00000139926:ENSG00000087301:ENS

						G00000165861:ENSG00000100767:ENSG00000133961:ENSG00000128731:ENSG00000261794:ENSG00000021776:ENSG00000137802:ENSG00000243708:ENSG00000197535:ENSG00000140262:ENSG00000103707:ENSG00000103710:ENSG00000136378:ENSG00000103888:ENSG00000157766:ENSG00000185418:ENSG00000183426:ENSG00000085721:ENSG00000140682:ENSG00000196470:ENSG00000072736:ENSG00000103064:ENSG00000103061:ENSG00000132600:ENSG00000127804:ENSG00000196689:ENSG00000197417:ENSG00000029725:ENSG00000005100:ENSG00000132514:ENSG00000141127:ENSG00000188522:ENSG00000072134:ENSG00000166484:ENSG00000142494:ENSG00000141750:ENSG00000068079:ENSG00000184988:ENSG00000011258:ENSG00000214226:ENSG00000153933:ENSG00000198265:ENSG00000141540:ENSG00000196169:ENSG00000078687:ENSG00000141577:ENSG00000017797:ENSG00000141401:ENSG00000141431:ENSG00000141469:ENSG00000152223:ENSG00000264278:ENSG00000130856:ENSG00000197971:ENSG00000205744:ENSG00000104833:ENSG00000171105:ENSG00000104883:ENSG00000198342:ENSG00000132016:ENSG00000132000:ENSG00000269190:ENSG00000179751:ENSG00000028277:ENSG00000105429:ENSG00000105427:ENSG00000185453:ENSG00000167766:ENSG00000213020:ENSG00000196417:ENSG00000176293:ENSG00000196208:ENSG00000134323:ENSG00000197872:ENSG00000084676:ENSG00000163803:ENSG00000162929:ENSG00000082898:ENSG00000116039:ENSG00000075292:ENSG00000217702:ENSG00000188687:ENSG00000005448:ENSG00000114993:ENSG00000115607:ENSG00000153208:ENSG00000163040:ENSG00000153250:ENSG00000118260:ENSG00000018280:ENSG00000135912:ENSG00000186235:ENSG00000162804:ENSG00000088876:ENSG00000089048:ENSG00000124107:ENSG00000155313:ENSG00000157617:ENSG00000160190:ENSG00000184792:ENSG00000128335:ENSG00000100321:ENSG00000100324:ENSG00000198951:ENSG00000177096:ENSG00000138944:ENSG00000205643:ENSG00000075234:ENSG00000100416:ENSG00000075275:ENSG00000205593:ENSG00000008735:ENSG00000251322:ENSG00000144560:ENSG00000163517:ENSG00000163576:ENSG00000168032:ENSG00000144648:ENSG00000180432:ENSG00000182983:ENSG00000179564:ENSG00000186792:ENSG00000243477:ENSG00000068001:ENSG00000007402:ENSG00000180376:ENSG00000163946:ENSG00000175105:ENSG00000184500:ENSG00000169379:ENSG00000178700:ENSG00000163608:ENSG00000017260:ENSG00000034533:ENSG00000240303:ENSG00000113971:ENSG00000154917:ENSG00000158163:ENSG00000158186:ENSG00000114115:ENSG00000155903:ENSG00000175066:ENSG00000114127:ENSG00000114698:ENSG00000174899:ENSG00000163904:ENSG00000145014:ENSG00000041802:ENSG00000013288:ENSG00000109689:ENSG00000080493:ENSG00000169429:ENSG00000163739:ENSG00000205403:ENSG00000215218:ENSG00000113361:ENSG00000164190:ENSG00000039123:ENSG00000086189:ENSG00000086200:ENSG00000164331:ENSG00000164180:ENSG00000175471:ENSG00000181751:ENSG00000168916:ENSG00000064651:ENSG00000240764:ENSG00000164620:ENSG00000197948:ENSG00000196670:ENSG00000078401:ENSG00000197935:ENSG00000146112:ENSG00000137404:ENSG00000137337:ENSG00000244731:ENSG00000223501:ENSG00000124772:ENSG00000164530:ENSG00000132429:ENSG00000198945:ENSG00000120278:ENSG00000026297:ENSG00000186340:ENSG00000184786:ENSG00000130023:ENSG00000105855:ENSG00000253293:ENSG00000005073:ENSG00000241644:ENSG00000152926:ENSG00000126522:ENSG00000177679:ENSG00000085563:ENSG00000127920:ENSG00000085514:ENSG00000078487:ENSG00000185955:ENSG00000166924:ENSG00000106484:ENSG00000182158:ENSG00000146966:ENSG00000197993:ENSG00000196329:ENSG00000120910:ENSG00000254087:ENSG00000147601:ENSG00000121039:ENSG00000104267:ENSG00000120526:ENSG00000131773:ENSG00000196378:ENSG00000044459:ENSG00000178031:ENSG00000130958:ENSG00000136925:ENSG00000136936:ENSG00000070159:ENSG00000179058:ENSG00000148411:ENSG00000198113
DEG.twoside	Ovary	7253	366	0.0033435006547922	0.180549035358779	ENSG00000158292:ENSG00000097021:ENSG00000171819:ENSG00000120942:ENSG00000162458:ENSG00000065526:ENSG00000204178:ENSG00000160051:ENSG00000168389:ENSG00000179178:ENSG00000117399:ENSG00000198198:ENSG00000117472:ENSG00000162367:ENSG00000117862:ENSG00000198841:ENSG00000134717:ENSG00000162390:ENSG00000134709:ENSG00000162604:ENSG00000117114:ENSG00000117155:ENSG00000153904:ENSG00000197147:ENSG00000162694:ENSG00000117543:ENSG00000168679:ENSG00000085465:ENSG00000197323:ENSG00000143457:ENSG00000163131:ENSG00000143387:ENSG00000143437:ENSG00000143379:ENSG00000143434:ENSG00000163154:ENSG00000163155:ENSG00000163157:ENSG00000116539:ENSG00000125459:ENSG00000132680:ENSG00000242252:ENSG00000160781:ENSG00000187800:ENSG00000203747:ENSG00000122176:ENSG00000163050:ENSG00000116962:ENSG00000162714:ENSG00000198879:ENSG00000241058:ENSG00000165997:ENSG00000095794:ENSG00000188234:ENSG00000107736:ENSG00000138134:ENSG00000026103:ENSG00000165338:ENSG00000155256:ENSG00000120057:ENSG00000165832:ENSG00000198873:ENSG00000184545:ENSG00000268403:ENSG00000166478:ENSG00000198730:ENSG00000006071:ENSG00000149150:ENSG00000124939:ENSG00000124935:ENSG00000162174:ENSG00000168060:ENSG00000146670:ENSG00000149809:ENSG00000173653:ENSG00000175567:ENSG00000175564:ENSG00000062282:ENSG00000149256:ENSG00000149292:ENSG00000076053:ENSG00000023171:ENSG00000064309:ENSG00000010219:ENSG00000111679:ENSG00000013588:ENSG00000134531:ENSG00000123094:ENSG00000050426:ENSG00000123338:ENSG00000123360:ENSG00000120868:ENSG00000235162:ENSG00000135094:ENSG00000174989:ENSG00000150990:ENSG00000184992:ENSG00000133110:ENSG00000136167:ENSG00000102531:ENSG00000123179:ENSG00000118939:ENSG00000088451:ENSG00000134901:ENSG00000134897:ENSG00000102524:ENSG00000100889:ENSG00000139914:ENSG00000168952:ENSG00000176165:ENSG00000179476:ENSG00000198718:ENSG00000185246:ENSG00000187790:ENSG00000087301:ENSG00000006432:ENSG00000119599:ENSG00000100767:ENSG00000254656:ENSG00000170113:ENSG00000128731:ENSG00000186399:ENSG00000021776:ENSG00000174197:ENSG00000137802:ENSG00000243708:ENSG00000243789:ENSG00000140262:ENSG00000103707:ENSG00000136378:ENSG00000103888:ENSG00000117899:ENSG00000183426:ENSG00000085721:ENSG00000102879:ENSG00000180209:ENSG00000179965:ENSG00000140691:ENSG00000140682:ENSG00000102910:ENSG00000196470:ENSG00000103064:ENSG00000132600:ENSG00000225614:ENSG00000185669:ENSG00000167522:ENSG00000197912:ENSG00000127804:EN

					SG00000196689:ENSG00000197417:ENSG00000072849:ENSG00000132514:ENSG00000141127:ENSG00000188522:ENSG00000166484:ENSG00000142494:ENSG00000068079:ENSG00000184988:ENSG00000182963:ENSG00000198933:ENSG00000011258:ENSG00000011260:ENSG00000214226:ENSG00000108423:ENSG00000108443:ENSG00000198265:ENSG00000154217:ENSG00000123700:ENSG00000141540:ENSG00000141576:ENSG00000185262:ENSG00000141577:ENSG00000141384:ENSG00000141431:ENSG00000152223:ENSG00000130856:ENSG00000197971:ENSG00000071564:ENSG00000205744:ENSG00000104833:ENSG00000171105:ENSG00000065989:ENSG00000198342:ENSG00000132005:ENSG00000237440:ENSG00000269190:ENSG00000105369:ENSG00000167625:ENSG00000079462:ENSG00000105427:ENSG00000079435:ENSG00000079385:ENSG00000063127:ENSG00000142544:ENSG00000105383:ENSG00000196214:ENSG00000167766:ENSG00000213020:ENSG00000196417:ENSG00000176293:ENSG00000115756:ENSG00000196208:ENSG00000134323:ENSG00000084676:ENSG00000163803:ENSG00000170820:ENSG00000116039:ENSG00000075292:ENSG00000144034:ENSG00000144048:ENSG00000163170:ENSG00000005448:ENSG00000144218:ENSG00000115602:ENSG00000115604:ENSG00000153107:ENSG00000115762:ENSG00000163040:ENSG00000115963:ENSG00000153250:ENSG00000231672:ENSG00000144485:ENSG00000162804:ENSG00000196209:ENSG00000088876:ENSG00000125966:ENSG00000124102:ENSG00000124107:ENSG00000157617:ENSG00000160284:ENSG00000184792:ENSG00000100342:ENSG00000100321:ENSG00000100324:ENSG00000128285:ENSG00000198951:ENSG00000186951:ENSG00000205643:ENSG00000075234:ENSG00000100416:ENSG00000075275:ENSG00000130489:ENSG00000025708:ENSG00000177989:ENSG00000205560:ENSG00000008735:ENSG00000251322:ENSG00000144560:ENSG00000170293:ENSG00000168032:ENSG00000182606:ENSG00000182983:ENSG00000114378:ENSG00000114388:ENSG00000180376:ENSG00000163946:ENSG00000114861:ENSG00000163320:ENSG00000184500:ENSG00000178700:ENSG00000114446:ENSG00000144843:ENSG00000172780:ENSG00000034533:ENSG00000114670:ENSG00000240303:ENSG00000113971:ENSG00000129048:ENSG00000154917:ENSG00000158163:ENSG00000138231:ENSG00000158186:ENSG00000114115:ENSG00000155903:ENSG00000175066:ENSG00000114127:ENSG00000114698:ENSG00000169282:ENSG00000075420:ENSG00000163898:ENSG00000163904:ENSG00000163975:ENSG00000013288:ENSG00000157765:ENSG00000109689:ENSG00000080493:ENSG00000163739:ENSG00000138823:ENSG00000123739:ENSG00000205403:ENSG00000164128:ENSG00000109466:ENSG00000113361:ENSG00000113360:ENSG00000164190:ENSG00000171522:ENSG00000132357:ENSG00000039123:ENSG00000164331:ENSG00000164180:ENSG00000153140:ENSG00000175471:ENSG00000164620:ENSG00000197948:ENSG00000113272:ENSG00000113742:ENSG00000196670:ENSG00000078401:ENSG00000197935:ENSG00000137404:ENSG00000137337:ENSG00000204344:ENSG00000244731:ENSG00000235863:ENSG00000164530:ENSG00000243955:ENSG00000146243:ENSG00000118432:ENSG00000198945:ENSG00000135525:ENSG00000118503:ENSG00000146416:ENSG00000091831:ENSG00000131018:ENSG00000213066:ENSG00000186340:ENSG00000184786:ENSG00000130023:ENSG00000217455:ENSG00000122644:ENSG00000005073:ENSG00000152926:ENSG00000165215:ENSG00000189143:ENSG00000086730:ENSG00000135218:ENSG00000127920:ENSG00000105829:ENSG00000078487:ENSG00000160813:ENSG00000005249:ENSG00000106484:ENSG00000158623:ENSG00000131558:ENSG00000182158:ENSG00000146966:ENSG00000159763:ENSG00000196329:ENSG00000214106:ENSG00000129691:ENSG00000147465:ENSG00000254087:ENSG00000147601:ENSG00000104267:ENSG00000131773:ENSG00000196378:ENSG00000080608:ENSG00000044459:ENSG00000213694:ENSG00000130958:ENSG00000136925:ENSG00000070159:ENSG00000167081:ENSG00000136840:ENSG00000148339:ENSG00000170835
DEG.twoside	Pancreas	10415	674	7.61289986662469e-33	4.11096592797733e-31	ENSG00000116237:ENSG00000158292:ENSG00000097021:ENSG00000120948:ENSG00000171824:ENSG00000198793:ENSG00000120942:ENSG00000065526:ENSG00000116809:ENSG00000158747:ENSG00000070831:ENSG00000117616:ENSG00000183726:ENSG00000184007:ENSG00000084652:ENSG00000160050:ENSG00000160051:ENSG00000116525:ENSG00000160094:ENSG00000134686:ENSG00000127603:ENSG00000117395:ENSG00000179178:ENSG00000253313:ENSG00000066056:ENSG00000066322:ENSG00000159479:ENSG00000198198:ENSG00000117472:ENSG00000162366:ENSG00000198841:ENSG00000134717:ENSG00000162384:ENSG00000134709:ENSG00000162604:ENSG00000162616:ENSG00000117114:ENSG00000117133:ENSG00000117155:ENSG00000162642:ENSG00000142867:ENSG00000153904:ENSG00000197147:ENSG00000069712:ENSG00000117528:ENSG00000117569:ENSG00000162694:ENSG00000134215:ENSG00000162775:ENSG00000134248:ENSG00000085465:ENSG00000116455:ENSG00000163349:ENSG00000197323:ENSG00000116752:ENSG00000213281:ENSG00000052723:ENSG00000163131:ENSG00000143387:ENSG00000143437:ENSG00000143379:ENSG00000143412:ENSG00000143363:ENSG00000143434:ENSG00000163154:ENSG00000163156:ENSG00000163155:ENSG00000143537:ENSG00000160767:ENSG00000116521:ENSG00000143630:ENSG00000160753:ENSG00000116539:ENSG00000125459:ENSG00000163374:ENSG00000132676:ENSG00000116580:ENSG00000132718:ENSG00000143622:ENSG00000132680:ENSG00000116584:ENSG00000160803:ENSG00000160789:ENSG00000196189:ENSG00000160783:ENSG00000242252:ENSG00000160781:ENSG00000198952:ENSG00000163472:ENSG00000198715:ENSG00000187800:ENSG00000132694:ENSG00000132703:ENSG00000143226:ENSG00000173110:ENSG00000203747:ENSG00000152332:ENSG00000174175:ENSG00000186283:ENSG00000143322:ENSG00000177888:ENSG00000174307:ENSG00000122176:ENSG00000182004:ENSG00000069275:ENSG00000143801:ENSG00000163050:ENSG00000116962:ENSG00000015171:ENSG00000107929:ENSG00000241058:ENSG00000165997:ENSG00000099246:ENSG00000108094:ENSG00000095794:ENSG00000188234:ENSG00000165476:ENSG00000107738:ENSG00000138134:ENSG00000107796:ENSG00000026103:ENSG00000152779:ENSG00000165338:ENSG00000155256:ENSG00000120057:ENSG00000203867:ENSG00000119927:ENSG00000148702:ENSG00000165832:ENSG00000198873:ENSG00000166311:ENSG00000166313:ENSG00000205339:ENSG00000268403:ENSG00000166478:ENSG00000166483:ENSG00000198730:ENSG00000006071:ENSG00000110442:ENSG00000019505:ENSG00000165905:ENSG00000149150:ENSG00000156587:ENSG00000162174:ENSG00000110025:ENSG00000168061:ENSG00000162300:ENSG00000174276:EN

[truncated: 83,239 more chars]
